# lncRNA Sequencing Reveals Neurodegeneration-Associated FUS Mutations Alter Transcriptional Landscape of iPS Cells That Persists in Motor Neurons

**DOI:** 10.3390/cells12202461

**Published:** 2023-10-16

**Authors:** Vincent E. Provasek, Manohar Kodavati, Wenting Guo, Haibo Wang, Istvan Boldogh, Ludo Van Den Bosch, Gavin Britz, Muralidhar L. Hegde

**Affiliations:** 1 Division of DNA Repair Research within the Center for Neuroregeneration, Department of Neurosurgery, Houston Methodist Research Institute, Houston, TX 77030, USA; vprovasek@houstonmethodist.org (V.E.P.); mkodavati@houstonmethodist.org (M.K.); whb.bio@gmail.com (H.W.); 2 School of Medicine, Texas A&M University, College Station, TX 77843, USA; 3 INSERM, UMR-S1118, Mécanismes Centraux et Périphériques de la Neurodégénérescence, Université de Strasbourg, CRBS, 67000 Strasbourg, France; wenting.guo@inserm.fr; 4 VIB, Center for Brain & Disease Research, 3000 Leuven, Belgium; 5 Leuven Brain Institute (LBI), 3000 Leuven, Belgium; 6 Stem Cell Institute, Department of Development and Regeneration, KU Leuven, 3000 Leuven, Belgium; ludo.vandenbosch@kuleuven.be; 7 Department of Microbiology and Immunology, University of Texas Medical Branch, Galveston, TX 77555, USA; sboldogh@utmb.edu; 8 Department of Neurosurgery, Houston Methodist Research Institute, Houston, TX 77030, USA; gbritz@houstonmethodist.org; 9 Department of Neurosurgery, Weill Cornell Medical College, New York, NY 10065, USA

**Keywords:** fused-in sarcoma (FUS), neurodegenerative disorders, induced pluripotent stem cells (iPSCs), long non-coding RNAs (lncRNAs), RNA sequencing

## Abstract

Fused-in sarcoma (FUS) gene mutations have been implicated in amyotrophic lateral sclerosis (ALS). This study aimed to investigate the impact of FUS mutations (R521H and P525L) on the transcriptome of induced pluripotent stem cells (iPSCs) and iPSC-derived motor neurons (iMNs). Using RNA sequencing (RNA Seq), we characterized differentially expressed genes (DEGs) and differentially expressed lncRNAs (DELs) and subsequently predicted lncRNA–mRNA target pairs (TAR pairs). Our results show that FUS mutations significantly altered the expression profiles of mRNAs and lncRNAs in iPSCs. Using this large dataset, we identified and verified six key differentially regulated TAR pairs in iPSCs that were also altered in iMNs. These target transcripts included: GPR149, NR4A, LMO3, SLC15A4, ZNF404, and CRACD. These findings indicated that selected mutant FUS-induced transcriptional alterations persist from iPSCs into differentiated iMNs. Functional enrichment analyses of DEGs indicated pathways associated with neuronal development and carcinogenesis as likely altered by these FUS mutations. Furthermore, ingenuity pathway analysis (IPA) and GO network analysis of lncRNA-targeted mRNAs indicated associations between RNA metabolism, lncRNA regulation, and DNA damage repair. Our findings provide insights into potential molecular mechanisms underlying the pathophysiology of ALS-associated FUS mutations and suggest potential therapeutic targets for the treatment of ALS.

## 1. Introduction

Neurodegenerative disorders, such as amyotrophic lateral sclerosis (ALS), pose significant challenges to our understanding of the molecular mechanisms that drive neuronal dysfunction and degeneration. While 90% of ALS is sporadic (sALS), the remaining 10% of patients suffer from the familial variant of the disease (fALS). One of the mutated genes responsible for fALS is the fused-in sarcoma (FUS) gene, which has been identified as an important contributor to the pathogenesis of fALS [1,2]. FUS is an RNA/DNA-binding protein that plays a crucial role in various aspects of RNA metabolism, including transcription, splicing, and transport, in addition to its role in DNA repair [3,4]. The dysregulation of FUS due to mutations has been implicated in the pathogenesis of ALS, although the precise molecular events leading to neurodegeneration remain unclear.

FUS mutations typically disrupt the nuclear localization signal (NLS) of the protein and can affect its RNA-binding capacity. Key mutations in the FUS gene, such as R521H and P525L, are located within the NLS domain and have been shown to promote the mislocalization of FUS from the nucleus to the cytoplasm of motor neurons and glial cells in ALS patients, leading to its aggregation [5]. This aberrant FUS localization results in both the loss of its normal function in the nucleus and the gain of toxic properties in the cytoplasm, suggesting that FUS mislocalization and aggregation plays a central role in disease pathogenesis [6].

FUS-associated neurodegeneration is thought to be driven by a combination of loss of nuclear function and cytoplasmic toxicity [7,8]. The loss of FUS function in the nucleus may lead to impaired RNA processing and transcriptional regulation, as well as defective DNA repair, thereby affecting neuronal survival and function [3,9,10,11,12,13]. On the other hand, the accumulation of cytoplasmic FUS aggregates may disrupt cellular homeostasis by impairing the function of other RNA-binding proteins and sequestering essential cellular components [8,14]. This dual mechanism, involving both the loss of nuclear function and the gain of cytoplasmic toxicity, may contribute to the complex pathophysiology of ALS. Previous studies have implicated alterations in RNA metabolism, stress granule dynamics, and DNA damage repair pathways in FUS-associated neurodegeneration [15,16,17]. However, a comprehensive understanding of the transcriptional landscape and the affected pathways in the context of FUS mutations is still lacking.

LncRNAs play a significant role in regulating gene expression, protein activity, and chromatin structure. They can interact with DNA, RNA, and proteins and have been shown to regulate various biological processes, including development, differentiation, and diseases [18,19]. Altered RNA metabolism and regulation has been identified as a critical factor in the pathogenesis of ALS. Many familial mutations associated with ALS occur in DNA/RNA-binding proteins, such as FUS, TDP-43, and others [20]. In particular, FUS has been shown to interact with a wide variety of RNAs, including mRNAs, miRNAs, and lncRNAs, and to play a multi-faceted role in lncRNA regulation [21]. FUS has also been shown to be important for the localization of lncRNAs to specific subcellular compartments, such as the nucleus or cytoplasm, with significant consequences for their function. The lncRNA nuclear paraspeckle assembly transcript 1 (NEAT1) is one such example, where FUS has been shown to bind to a specific region of NEAT1 and assist in localizing it to nuclear paraspeckles, which are subnuclear bodies involved in regulating gene expression [6,22]. Furthermore, FUS is necessary for the formation of these paraspeckles in some cells, suggesting that FUS-NEAT1 interactions play a role in the regulation of this subnuclear structure. Interestingly, proteins enriched in the pool of proteins affected by ALS-causative mutations are also found in paraspeckles.

Here, we employed RNA-Seq to examine the effects of neurodegeneration-linked FUS mutations (R521H and P525L) on the transcriptional profiles of iPSCs and assessed whether any of these changes persisted into the differentiated motor neuron state. We first analyzed the expression profiles of both mRNAs and long non-coding RNAs (lncRNAs) in control and FUS-mutant iPSCs and used software tools to predict the interaction between lncRNAs and their putative target mRNA transcripts. Using these datasets, we identified the top differentially regulated lncRNAs and mRNAs that were predicted to have a functional relationship and confirmed their expression trends using RT-PCR in iPSCs. We also tested these targets in induced motor neurons (iMNs) using RT-PCR and discovered a set of three TAR pairs for each FUS mutant that reliably exhibited differential expression with important links to neuropathology. Furthermore, these data allowed us to identify significant biological processes involving RNA metabolism, lncRNA regulation, and DNA damage repair using ingenuity pathway analysis (IPA) and GO network analysis on lncRNA-targeted mRNAs.

This study helps shed light on potential lncRNA-mediated molecular mechanisms underlying mutant FUS-induced pathology in neurodegenerative disease and may help improve understanding for new therapeutic targets for ALS.

## 2. Methods

### 2.1. Cell Culture

The control human induced pluripotent stem cells (iPSCs) used in this study were obtained from ATCC (#KYOU-DXR0109B). The FUS mutant patient-derived iPSCs and their isogenic controls were generously provided by VIB-KU Leuven [3,23]. All iPSCs were cultured on Geltrex LDEV-free hESC-qualified basement membrane matrix, supplemented with 1X Essential 8 supplement. Colonies were regularly passaged using 0.5 mM EDTA (15575-020, Invitrogen, Waltham, MA, USA) in Dulbecco’s phosphate-buffered saline (DPBS). The cultures were routinely monitored for mycoplasma contamination by PCR. Motor neurons were generated from the iPSC lines following a previously published protocol [24]. Briefly, iPSC clones were suspended using collagenase and transferred to a T-25 flask with neuronal basic medium. Cells were then cultured for 48 h in the presence of a 5 μM ROCK inhibitor (Y-27632, RI, Millipore, Burlington, MA, USA), a 40 μM TGF- β inhibitor (SB 431524, SB, Tocris Bioscience, Bristol, UK), a 0.2 μM bone morphogenetic protein inhibitor (LDN-193189, LDN, Stemgent, Beltsville, MD, USA), and a 3 μM GSK-3 inhibitor (CHIR99021, CHIR, Tocris Bioscience). The cells were then suspended and incubated with a neuronal basic medium containing 0.1 μM retinoic acid (RA, from Sigma, St. Louis, MO, USA) and 500 nM Smoothened Agonist (SAG, from Merck Millipore) over 4 days. The media was then changed, and the cells were incubated for 48 h in neuronal basic medium containing RA, SAG, 10 ng/mL brain-derived neurotrophic factor (BDNF, Peprotech, Rocky Hill, NJ, USA), and 10 ng/mL glial-cell-derived neurotrophic factor (GDNF, Peprotech). Cell spheres were then dissociated in neuronal basic medium containing trypsin (0.025%) for 20 min at 37 °C, before being separated into single cells with trypsin-inhibitor-containing medium (1.2 mg/mL). The cells were quantified and distributed onto laminin (Life technologies, Carlsbad, CA, USA)-coated dishes according to manufacturer’s instruction and incubated for 5 days in neuronal basic medium with RA, SAG, BDNF, GDNF, and 10 μM DAPT. The media was then changed to neuronal basic medium with BDNF, GDNF, and a 20 μM γ-secretase inhibitor (DAPT, Tocris Bioscience) for 48 h. These were then used for motor neuron differentiation by maintaining the cells for 7 days in medium containing BDNF, GDNF, and 10 ng/mL ciliary neurotrophic factor (CNTF, Peprotech).

### 2.2. RNA Isolation, Library Construction, and Sequencing

Approximately 2 × 10^6^ cells were collected and centrifuged at 4 °C at 2000 rpm for 3 min for the extraction of RNA. Total RNA was isolated using Trizol (Thermo Scientific, Waltham, MA, USA) according to the manufacturer’s instruction. RNA purity and quantification was determined using a NanoDrop 2000 spectrophotometer (Thermo Fisher Scientific, Waltham, MA, USA). The extracted RNA was sent to BGI (Shenzhen, China) for transcriptome library construction and data analysis. During this process, RNA quality was measured using an Agilent 2100 Bioanalyzer (Agilent Technologies, Santa Clara, CS, USA). The RNA samples were further purified using a Ribo-Zero rRNA Removal Kit before fragmentation occurred. Random primers and a TruSeq reverse transcriptase kit were used for first-strand cDNA synthesis, followed by DNA polymerase I and RNaseH for double-stranded cDNA synthesis. The library was then sequenced using BGISEQ-500. Low-quality reads, adapter contamination, and unknown N bases were filtered. The remaining clean reads were mapped to the reference genome (GRCh37) using HISAT (v2.0.4) with default parameters. The resulting transcripts were assembled using StringTie (v1.0.4). lncRNA transcripts were further annotated using the NONCODE [25] database.

### 2.3. Coding Ability Prediction

Novel transcripts were assessed for their coding ability to distinguish mRNA from lncRNA. The software tools CPC (Coding Potential Calculator) [26], txCdsPredict [27], and CNCI (Coding Non Coding Index) [2] were used to score the coding capacity of novel transcripts, requiring CPC and CNCI scores of <0 and txCdsPredict scores of <500. These results were also queried against the database pfam [28] to identify known protein-coding domains. Novel transcripts were designated as lncRNA or mRNA when at least three of the prediction methods were in agreement.

### 2.4. Identification of Differentially Expressed lncRNA and mRNA

lncRNA and mRNA expression levels were calculated using the fragment per kilobase of transcript per million mapped reads (FPKM). To calculate the differential expression analysis of genes and transcripts, Bowtie2 was used to align clean reads to the reference sequence, and then RSEM was used to calculate gene and transcript expression levels. Subsequently, the PossionDis tool (PossionDisFoldChange ≥ 2.00 and FDR ≤ 0.001) was used to analyze the significance of differentially expressed lncRNAs (DELs) and differentially expressed mRNAs (DEGs) between the SA (control) samples and the SB (FUS-P525L) and SC (FUS-R521H) samples. Initial screening identified DELs and DEGs as those transcripts with *p*-values of <0.05 and an absolute log_2_ fold change of ≥1.5.

### 2.5. lncRNA Target Gene Prediction

To better understand the potential functional roles of DELs, mRNA target gene prediction was used. The analysis methods used in this study included calculating Spearman and Pearson correlation coefficients of the expression values of DELs and mRNA. Genes for which Spearman_cor ≥ 0.6 and Pearson_cor ≥ 0.6 were selected as interaction pairs. These pairs were then classified as cis or trans. Any lncRNA in the 10 kB upstream or 20 kB downstream of the putative mRNA was designated as cis. Targets beyond this range were identified by binding the energy of lncRNA and mRNA using RNAplex.

### 2.6. Functional Enrichment of DEGs and Target Genes of DELs

The datasets of DEGs, DELs, and DEL targets were cross examined to identify TAR pairs containing DELs predicted to target DEGs; these pairs were further refined such that each DEL and DEG pair exhibited a 5-fold or greater increase/decrease relative to the control. These pairs were then selected for further study. Furthermore, the datasets of DEGs and DEL target genes were used for functional enrichment analysis using gene ontology (GO) analysis, Kyoto Encyclopedia of Genes and Genomes (KEGG) pathway analysis, and ingenuity pathway analysis (IPA—QIAGEN). GO, KEGG, and IPA terms for which *p* < 0.05 were accepted as significant.

### 2.7. qRT-PCR Validation of Differentially Expressed lncRNAs and mRNAs

The validity of the results was determined by RT-PCR using primers selected for certain highly differentially expressed DELs and their DEG targets. Using iPSC cell lines separate from those used for RNA-Seq, the total RNA of each cell line was extracted using Trizol reagent (Invitrogen, Carlsbad, CA, USA), according to the manufacturer’s instructions. The total RNA was reverse transcribed into cDNA using the Super Script Vilo Kit (Thermo Scientific, Waltham, MA, USA). qRT-PCR amplification was performed in triplicate using the ABI 7500 (Applied Biosystems, Foster City, CA, USA) and the PowerUp SYBER Green Master Mix (Thermo Scientific, Waltham, MA, USA). Multiple housekeeping genes were utilized as an internal control and included the HPRT and GAPDH genes. All RT-PCR reactions were conducted in triplicate. Primers for the lncRNAs and mRNAs were purchased from Sigma and are shown in Supplementary Table S1. The relative expression of each validated gene was determined using the 2^−ΔΔCt^ method. Student’s *t*-test was performed and results for which *p* < 0.05 were accepted as significant.

## 3. Results

### 3.1. Results of Sequencing and Characteristics of Transcripts

The overall design of this study is depicted in Supplementary Figure S1. Human derived iPSCs were grown under standard conditions before total RNA isolation. After verifying the quality of the extracted RNA, sequencing libraries were prepared (BGI). Short-read sequencing was used to analyze the transcriptomes of iPSCs derived from ALS patients carrying the FUS P525L or FUS R521H mutations and compared to control iPSCs. A total of 106,975,508 reads were collected from control samples, and 108,244,484 and 92,527,778 reads were collected from the P525L and R521H mutants, respectively (Table 1). These data were then filtered to remove low-quality reads and adaptor sequences, which yielded a total of 99,081,102 clean reads for the control and 99,842,418 and 85,427,768 clean reads for the R521H and P525L mutants, respectively. We observed mapping rates of these clean reads to the reference transcriptome (GRh37) of 92.25% for control and 93.48% and 94.21% for R521H and P525L, respectively. Over 80% of all reads mapped to a single location in the reference transcriptome, indicating that the data were acceptable for accurate differential gene expression analysis. Additional general characteristics of the mapped reads are summarized in Supplementary Figures S2 and S3.

**Figure 1:**
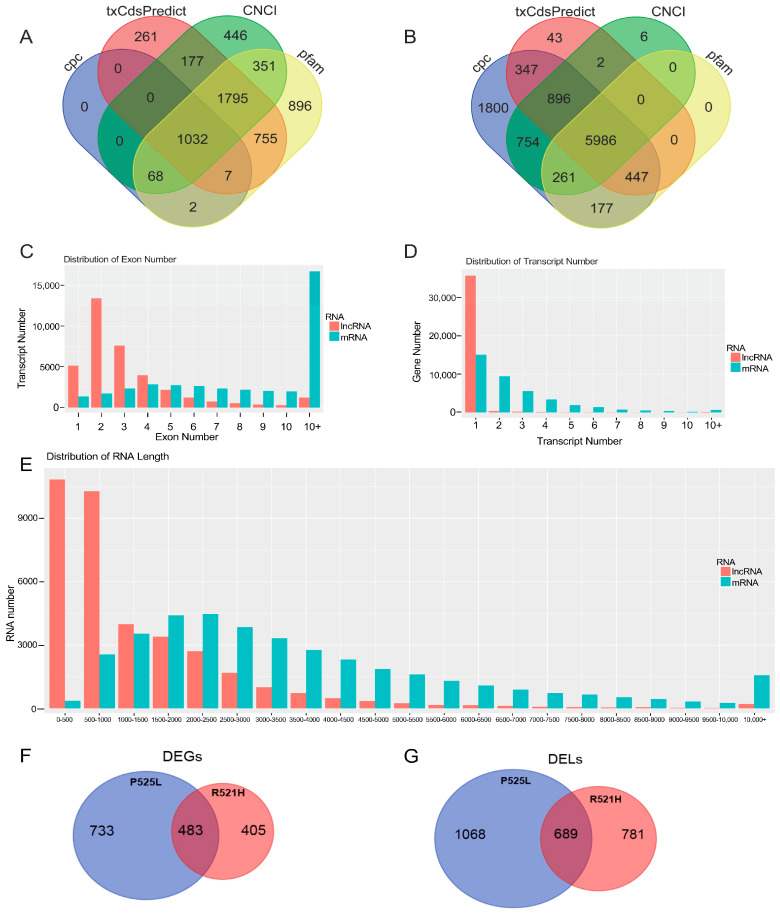
Specific characteristics of human iPS-cell-derived mRNAs and lncRNAs. A Venn diagram of the analysis of merged transcripts from all samples, revealing 1795 novel lncRNAs (**A**) and 5986 novel mRNAs (**B**), which were identified using four prediction software tools, indicated by color. (**C**) Exon number analysis in transcripts, including mRNA and lncRNA; X-axis: number of exons, Y-axis: number of transcripts, color: RNA classification. (**D**) Distribution of the transcript number for genes, including mRNA and lncRNA; X-axis: number of transcripts, Y-axis: number of genes, color: RNA classification. (**E**) A statistical figure showing RNA length, including mRNA and lncRNA; X-axis: RNA length, Y-axis: number of transcripts, color: RNA classification. (**F**) A Venn diagram of the significant differentially expressed mRNA genes (DEGs) in FUS mutants P525L and R521H over WT. (**G**) A Venn diagram of the significant differentially expressed lncRNAs (DELs) in FUS mutants P525L and R521H over WT.

Following transcriptome assembly across all samples, transcripts identified as novel were merged and simultaneously assessed for protein coding capacity using four predictive software tools. This analysis step identified 1795 novel lncRNAs (1703 in the control, 1716 in R521H, and 1697 in P525L), and 5986 novel mRNAs (4035 in the control, 4033 in R521H, and 4034 in P525L) across all samples combined (Figure 1A,B). A summary of the quantitative analysis is given in Table 2. Only known mRNA transcripts mapping to the reference sequence and lncRNAs identified in the NONCODE database were used for the downstream analysis of differential expression. 

Additional characteristics of the mapped transcripts are shown in Figure 1. Expectedly, analysis of the distribution of exon number across the transcripts revealed that lncRNAs tended to have two exons, while mRNAs contained more than ten (Figure 1C,D). Similarly, transcripts with a length of 2–2.5 kb made up the majority of mRNAs, while predicted lncRNAs were comprised mostly of transcripts between 0 and 500 bp (Figure 1E). Additionally, when we analyzed the number of DEGs and DELs between each comparison group, we discovered that FUS-P525L mutant cells contained 58% more DEGs and 31% more DELs than FUS-R521H mutant cells, relative to the controls (Figure 1F,G).

### 3.2. Differential Expression Analysis

**Figure 2:**
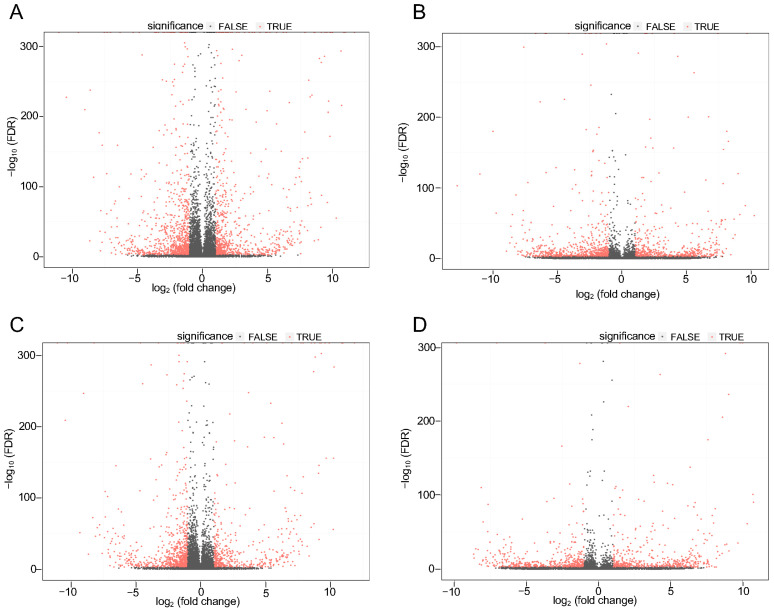
Expression profiles of distinct RNAs in human iPS cells carrying neurodegeneration-associated FUS mutations. The volcano plots depict the log fold change of uniquely mapped mRNA and lncRNAs from each sample of control and FUS mutant human iPS cells. (**A**) Control vs. P252L, mRNA. (**B**) Control vs. P525L, lncRNA. (**C**) Control vs. R521H, mRNA. (**D**) Control vs. R521H, lncRNA.

LncRNA and mRNA expression levels were calculated using the fragment per kilobase of transcript per million mapped reads (FPKM). To perform a differential expression analysis of the genes and transcripts, Bowtie2 [29] was used to align clean reads to the reference sequence, and then RSEM [30] was used to calculate gene and transcript expression levels. Subsequently, the PossionDis [31] tool (PossionDisFoldChange ≥ 2.00 and FDR ≤ 0.001) was used to analyze the significance of DELs and DEGs between the control, P525L, and R521H. The analyses of DEGs and DELs are depicted in Figure 2A–D. In total, 1734 significantly differentially expressed mRNAs and 1239 lncRNAs were identified in the control as compared to the P525L samples, while 1317 mRNAs and 1041 lncRNAs were detected in the control as compared to the R521H samples. Interestingly, there was significant overlap between the most upregulated genes in the comparisons between the control and each mutant. Of the top ten most upregulated genes, comparisons between the control and each mutant shared six commonly identified targets. These included RPS4y1, DDX3Y, MXLOC_037825, EIF1AY, RPL17-C18orf32, and n379185. Evaluation of the most downregulated genes revealed slightly less concordance, with each comparison between control and mutant sharing only three of the top ten most downregulated genes. These included MXLOC_016157, MAGEA12, and LXLOC_037100. With respect to the total number of differentially expressed genes, the control vs. P525L and control vs. R521H comparisons yielded 711 and 593 upregulated targets, respectively. The same analysis of downregulated genes revealed 839 and 637 targets for the control vs. P525L and control vs. R521H comparisons, respectively. Supplementary Tables S2 and S3 contain a list of all of the differentially regulated mRNA transcripts and predicted lncRNAs for the control vs. P525L and control vs. R521H comparisons, respectively. Overall, these findings suggest that while each of the two different mutations in the same FUS protein exerted similar effects on the transcriptional landscape of iPSCs, there are important differences to consider in future work.

### 3.3. Identification of Differentially Regulated lncRNA–mRNA Target Pairs

**Figure 3:**
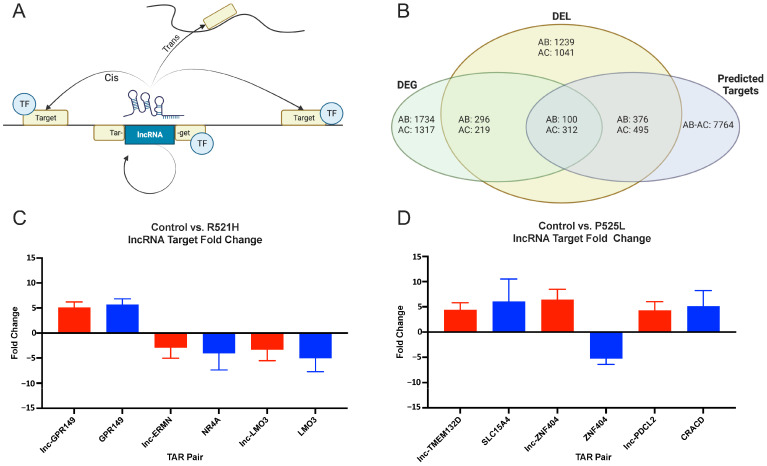
RNA-Seq reveals key differentially regulated lncRNA–mRNA target pairs in FUS mutant human iPS cells. (**A**) Schematic illustrating how lncRNAs may target mRNAs in a cis manner (i.e., mRNAs within 20 kB of the lncRNA) or a trans manner (i.e., predicted based on calculated binding energy between the TAR pair). (**B**) Venn diagram outlining the results of the manual annotation of raw data sets. A total of 1734 and 1317 well-annotated DEGs and 1239 and 1042 DELs were identified and cross-referenced against a total of 7764 predicted TAR pairs. The data sets were further refined to reveal 100 and 312 TAR pairs wherein each lncRNA and mRNA target were differentially regulated by twofold or greater. (**C**,**D**) Three differentially regulated TAR pairs were selected and the log2 fold expression was visualized. The lncRNA fold expression vs. the control is shown in red. The target mRNA fold expression vs. the control is shown in blue. The TAR pairs are juxtaposed along the X-axis. AB: control vs. P525L; AC: control vs. R521H; DEG: differentially expressed genes referencing mRNAs; DEL: differentially expressed lncRNAs; TF: transcription factor.

One of the mechanisms by which lncRNAs can alter gene expression is through cis- or trans-acting effects with target mRNAs (Figure 3A). To this end, we questioned whether any relationship existed between the DEGs and DELs identified between the control and FUS mutant iPSCs. To accomplish this, we cross-referenced datasets containing lists of DEGs and DELs against a list of software-generated predictions of lncRNA–mRNA target pairs (TarPairs) classified as cis/trans-acting (Supplementary Table S4) and overlapping (Supplementary Table S5). The results of this analysis are summarized in Figure 3B. We refined the list of 7764 TarPairs by filtering those pairs whose members were differentially regulated by a factor of two or greater in either direction. This filtering step resulted in the identification of 100 significantly regulated TarPairs between the control and the P525L mutant, and 312 between the control and the R521H mutant. Figure 3C,D show selected TarPairs exhibiting at least a fivefold expression difference between the control vs. P525L and control vs. R521H comparisons. The selected TarPairs for the control vs. R521H comparison included lnc-GPR149/GPR149, lnc-ERMN/NR4A, and lnc-LMO3/LMO3. The selected TarPairs for the control vs. P525L comparison included lnc-TMEM132D/SLC15A4, lnc-ZNF404/ZNF404, and lnc-PDCL2/CRACD. In many cases, the direction of mRNA target expression change was congruent with its putative interacting lncRNA. In other cases, such as the ZNF404 TarPair, the expression change direction was inversed.

### 3.4. RT-PCR Confirmation of TarPairs in iPSC and Motor Neuron Cell Cultures

**Figure 4:**
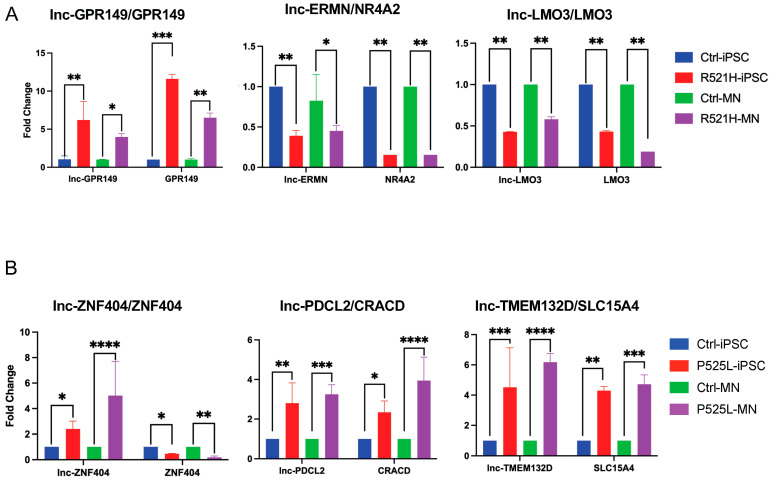
RT-PCR validation of RNA-Seq results. Selected differentially regulated TAR pairs identified in iPSC lines by RNA-Seq are confirmed by RT-PCR. The expression of the same TAR pairs was also tested by RT-PCR using RNA isolated motor neurons induced from separate cultures of the same iPSC lines. (**A**) Validation experiments for the three key TAR pairs identified in the FUS R521H cell line. (**B**) Validation experiments for three key TAR pairs identified in the FUS P525L cell line. RNA expression of the control iPSC and mutant iPSC are shown in blue and red, respectively. The RNA expression of the control and mutant induced motor neurons are shown in green and purple, respectively. iPSC: induced pluripotent stem cell; MN: motor neuron. *p*-values 0.0332 (*), 0.0021 (**), 0.0002 (***), <0.0001 (****).

To validate our sequencing results, six TarPairs were selected for RT-PCR analysis. New iPSC cell cultures were grown and total RNA isolates from each new cell culture were used to conduct validation experiments. As shown in Figure 4, the expression patterns of the DELs and their predicted target DEGs in iPSCs remained consistent with our sequencing data. Given the disease relevance of FUS P525L and R521H mutations for ALS pathogenesis, we questioned whether these effects persisted in motor neurons. To answer this question, we removed a subset of iPSCs and differentiated them into terminally differentiated motor neurons using the methods previously described [24]. RNA samples from these motor neurons were then evaluated by RT-PCR, and the results are shown in Figure 4. Interestingly, each of the selected TarPairs maintained the differential expression pattern observed in their iPSC precursor. In a final step, we further confirmed these expression differences by performing RT-PCR using RNA isolated from isogenic controls for each FUS mutant cell line, R521R vs. control, and P525P vs. control (Supplementary Figure S4). In each case, the isolated correction of the FUS mutant abrogated the observed expression differences. Taken together, these data suggest that at least some of the FUS mutant associated alterations in lncRNA and mRNA expression persist across cell development. 

### 3.5. Functional Enrichment Analysis of mRNAs Co-Expressed with lncRNAs

**Figure 5:**
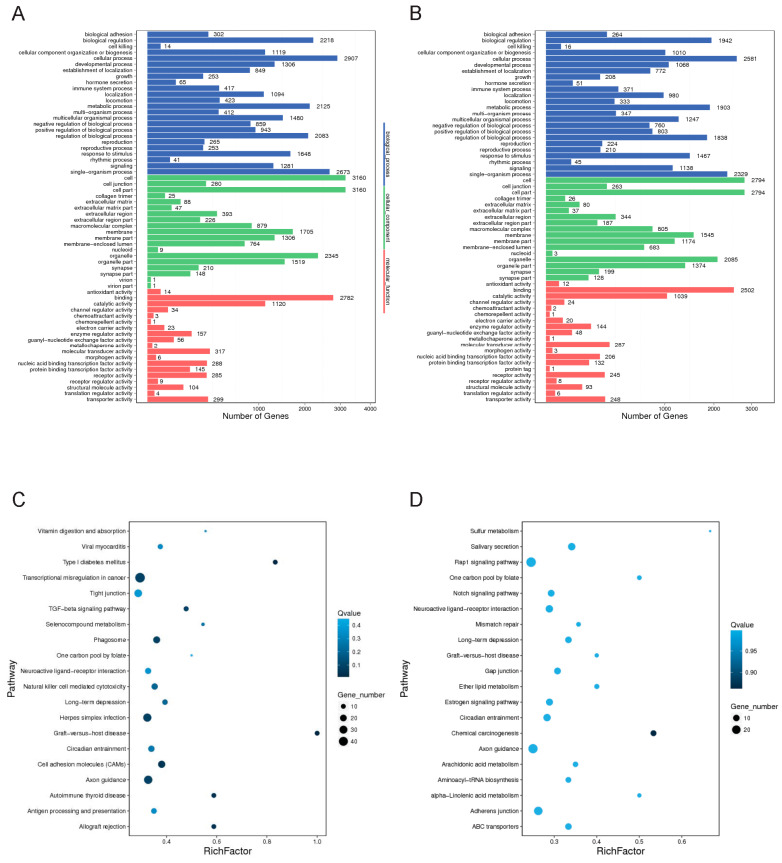
Enrichment analysis of differentially expressed genes. (**A**,**B**) Gene ontology (GO) enrichment analysis of differentially expressed genes of the control vs. R521H and control vs. P525L comparisons, respectively. X-axis: number of genes, Y-axis: GO entry, color: GO classification. (**C**,**D**) KEGG enrichment analysis of differentially expressed genes of the control vs. R521H and control vs. P525L comparisons, respectively. Multiple pathway analyses indicate that pathways associated with neuronal development and carcinogenesis are likely altered by FUS mutations. X-axis: enrichment factor, Y-axis: pathway, color: *p*-value, size: number of genes.

**Figure 6:**
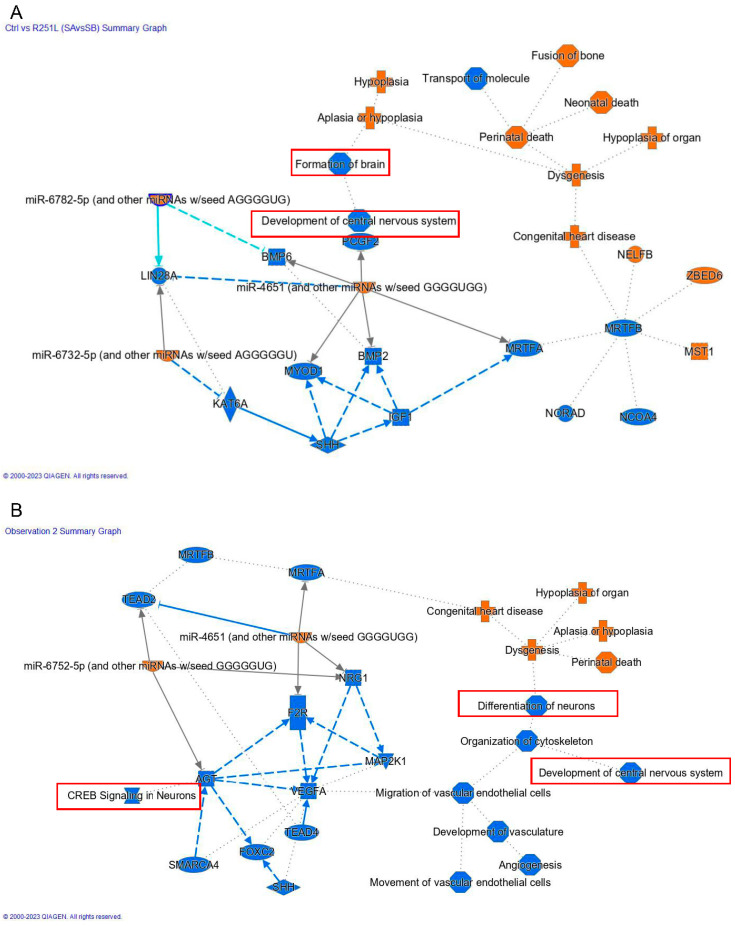
IPA network analysis. Ingenuity pathway analysis (QIAGEN) of DEGs revealed both inferred and direct effects of network connections involved in the development of the CNS and neural signaling (outlined in red). IPA analysis performed on DEGs identified from comparisons of (**A**) control vs. P525L and (**B**) control vs. R521H. Key terms related to neuronal development or function are outlined in red.

**Figure 7:**
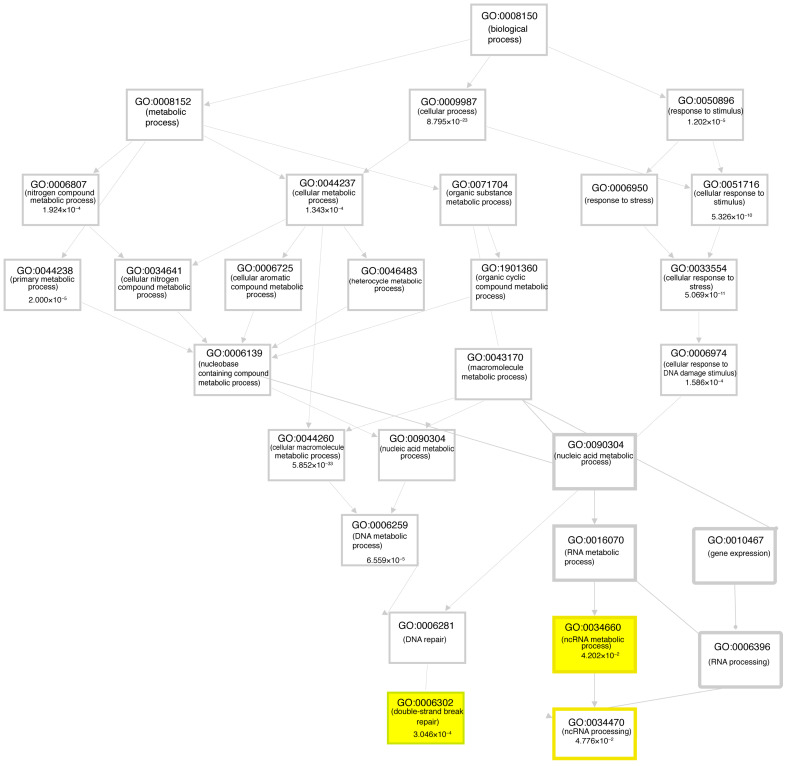
GO network analysis of lncRNA-targeted mRNAs. GO network analysis of lncRNA-targeted mRNAs indicated significant biological processes associated with RNA metabolism, lncRNA regulation, and DNA damage repair (highlighted in yellow).

Multiple network and functional pathway analysis tools were used to infer the functional consequences of the differentially expressed genes observed in FUS mutant iPSCs compared to the control. The potential function of the FUS P525L and R521H mutations were studied using gene ontology (GO) annotation and enrichment analysis. For the GO enrichment analysis of differentially expressed genes between samples, targets were classified into three general categories of biological processes, molecular function, and cellular component (Figure 5A,B). Within the biological process class, terms with the greatest number of associated genes found in both mutants included response to stimulus (GO:0050896), regulation of biological process (GO:0048519), cellular process (GO:0009987), and biological regulation (GO:0065007). We also analyzed functional enrichment analysis using the Kyoto Encyclopedia of Genes and Genomes (KEGG) tool (Figure 5C,D). Interestingly, we observed that functional enrichment included multiple nervous-system-related terms, including neuroactive ligand–receptor interactions, axon guidance, and cell adhesion molecules. Additionally, we observed functional enrichment around transcriptional misregulation in cancer, a function that might be expected given the strong association of FUS with neoplastic pathologies. We observed similar functional enrichment in the control vs. R521H comparison. Finally, we complemented these two approaches with ingenuity pathway analysis (QIAGEN) performed on DEGs identified between each mutant comparison (Figure 6A,B). Despite these analyses being conducted in iPSCs, we again observed the network effects associated with the nervous system, including CNS development in the control vs. P525L comparison and neuronal differentiation and signaling in the control vs. R521H comparison. Finally, we conducted GO analysis using the predicted mRNA targets of the identified lncRNAs (Figure 7). Given the general function of FUS as a regulator of RNA function, we observed significant GO terms indicating noncoding RNA processing (GO:0006396) and metabolic processes (GO:0034660). Additionally, we observed the significant terms of DNA damage signaling and repair (GO:0006302), and lncRNA metabolic process (GO:0034660).

## 4. Discussion

In this study, we identified differentially expressed genes, lncRNAs, and predicted lncRNA–mRNA target pairs (TAR pairs) associated with R521H and P525L FUS mutations in patient-derived iPSCs and iMNs. Our study demonstrates the impact of the neurodegeneration-associated FUS mutations R521H and P525L on the transcriptional landscape in iPSC cells and indicates that these changes may persist into the terminally differentiated state. By conducting RNA-seq analysis, we characterized the expression profiles of both mRNAs and lncRNAs in control and FUS mutant iPSCs. Our findings revealed significant changes in the expression profiles of distinct lncRNAs in FUS mutant iPSCs. Notably, these differentially expressed lncRNAs were correlated with a similar change in the expression of their putative target mRNAs. Unlike conventional RNA-Seq studies that primarily focus on changes in the landscape of mRNA or non-coding RNAs in isolation, our study is unique in that we have identified and validated the co-expression of several predicted lncRNA–mRNA TAR pairs in FUS mutant iPSCs that carried over into the iMN state. Moreover, we verified the direct impact of mutant FUS by analyzing the expression of select lncRNAs in mutation-corrected isogenic iPSC lines. While our study was limited to six TAR pairs, we believe these confirmed interactions positively contribute to the field’s understanding of how FUS mutations affect the development and function of neural cells. Here, we will review what is known about each differentially expressed mRNA target and discuss how their dysfunction may contribute to the development of FUS-linked neurodegenerative disease. 

Our study identified three differentially expressed targets associated with transcriptional regulation: LMO3, ZNF404, and NR4A2/Nurr1. LMO3 (LIM domain only 3) is a neuronal basic helix-loop-helix transcriptional regulator involved in cell fate determination and differentiation during embryonic development [32]. LMO3 plays a role in the neuronal differentiation of dopaminergic neurons of the substantia nigra, and neurons of the globus pallidus externus and has been utilized as a marker of interneuron development [33,34,35,36]. LMO3 is specifically involved in the development of dopaminergic neurons by acting as a transcriptional co-activator of Pitx3, ALDH1A1, and genes required for retinoic acid and GABA synthesis [33]. LMO3 is likewise preferentially expressed in the substantia nigra medial dopaminergic neurons [34], where its downregulation has been associated with Parkinsons’s disease (PD) neurodegeneration [35]. LMO3 expression is also maintained in motor neurons and has been shown to be downregulated and alternatively spliced in SHSY cell lines expressing ALS-associated G93A-SOD1 mutations and in SHSY cell lines treated with neurotoxic pesticide Paraquat [37]. Loss of LMO3 has also been shown to induce the adoption of depressive and anxiety-like behavioral phenotypes in LMO3 knockout mouse models and to alter animal response to ethanol administration [38]. Finally, LMO3 has been implicated in neuroblastoma progression, where the overexpression of LMO3 caused rapid and aggressive tumor growth and was subsequently associated with decreased patient survival [39]. These reports suggest a critical role for LMO3 in neurodevelopment and subsequent cellular behavior, with upregulation underpinning cancer cell growth and downregulation promoting neurodegeneration. Our results indicate that FUS mutants induce the downregulation of lncLMO3 and its putative target LMO3. This finding correlates well with previous reports that identified the downregulation of LMO3 in SOD1 ALS cell lines. The reports of LMO3 downregulation in PD are also of interest. While FUS mutations have not been strongly linked to PD, both PD and FUS ALS-affected neurons share similar histopathological characteristics, including cytosolic protein inclusions and increased genomic instability [40]. This further supports the notion that LMO3 downregulation contributes to a neurodegenerative phenotype. Based on our data, further investigation into the full impact of LMO3 on neural cell differentiation and subsequent motor neuron health seems warranted.

ZNF404 is another differentially expressed gene associated with mutant FUS. ZNF404 is a nuclear zinc finger protein that is predicted to be involved in the negative regulation of RNA polymerase II transcriptional activity, in addition to other cellular processes, such as DNA binding and protein–protein interactions [41]. Mutations in ZNF404 have also been recognized in transcriptional analyses of breast cancer and as a regulating factor in gingival progenitor cells [42,43]. The role of ZNF404 in neural development and neurodegeneration remains unknown. However, given the central role of altered transcriptional regulation and protein–protein interaction in the pathogenesis of ALS, it seems plausible that ZNF404 may contribute to disease progression, possibly through the nonspecific dysregulation of whole gene networks. Our data indicate that FUS may indirectly regulate LMO3 and ZNF404 functionality via lncRNAs. These observations expand what is known regarding the mechanisms of how FUS regulates transcriptional activity. Within the context of ALS, the FUS-mutant-induced loss of LMO3 likely plays a role in the degeneration of motor neurons, while increases in lnc-ZNF404, with the subsequent downregulation of ZNF404 mRNA, exacerbate wide-scale perturbations in diseased motor neuron transcriptomes. It is important to note, however, that the mechanisms underlying these FUS-induced alterations persist from iPSCs to motor neurons. How this specificity is achieved remains unknown and should be the subject of further studies.

NR4A2/Nurr1 is a nuclear receptor and transcription factor that has been associated with dopaminergic neuron differentiation, dopaminergic signaling, and the modulation of microglial- and astrocyte-mediated inflammation [44]. NR4A2 has been best studied in the context of PD, where its expression levels have been found to be diminished in both post-mortem tissues and living PD patients [45,46]. NR4A2 has been found to inhibit the expression of proinflammatory mediators and has been linked to a protective role against inflammatory neuron cell death [47]. In fact, the knockdown of NR4A2 exaggerated the inflammation responses of microglia and astrocytes, which in turn contributed to the demise of dopaminergic neurons [48]. While the role of NR4A2 in PD has been best described, more recent reports have demonstrated its role in Alzheimer’s disease, multiple sclerosis, stroke, depression, and intellectual disability [44,49,50,51]. For example, in rat models of ischemic stroke, NR4A2 was found to be regulated by miR-145-5p, and anti-miR-145-5p treatment enhanced neurological recovery following reperfusion [52]. Interestingly, NR4A2 levels have also been found to be decreased in the post-mortem tissues of aged brains, suggesting that NR4A2 may contribute to the aging brain [53]. Another recent report connected NR4A2 to cognitive ability. Specifically, it was found that long-lasting changes in synaptic plasticity within the hippocampus are regulated by NR4A2 [54]. Based on these documented roles, gene- and cell-based therapies targeting NR4A2 have become promising candidates for the treatment of neurodegenerative disease [55]. Here, we show that mutant FUS may also contribute to the regulation of NR4A2, possibly via the downregulation of lnc-ERMN. Whether the resulting downregulation of NR4A2 is a compensatory response to cell stress or is working in new ways to advance the disease process remains unknown. Taken together, our data add to the growing body of evidence linking NR4A2 to neurodegenerative disease and highlight two novel means by which disease progression may be targeted via FUS and lnc-ERMN.

CRACD (capping protein inhibiting regulator of actin dynamics) is involved in the negative regulation of actin filament capping within the cytosol. This process occurs through direct interactions with actin-capping proteins, resulting in a decreased affinity for actin. This negative regulation prevents the addition of a protective cap onto the barbed end of actin filaments, thereby enabling filament elongation or degradation [56]. It is known that actin filament regulation is critical for neurons as it regulates growth, axon stability, and synaptic function [57,58,59]. CRACD downregulation has been strongly associated with various types of cancer and metastasis, including small-cell lung carcinoma [60] and colorectal cancer stem cells [61]. Haplotypes of CRACD have been linked to opioid use in patients, as indicated by a recent genome-wide association study (GWAS) [62]. During development, CRACD is expressed during early timepoints and is thought to play a role in tissue differentiation, particularly in the heart and both the peripheral and central nervous systems. However, its expression is typically lost in most terminally differentiated adult tissues [63]. Interestingly, our findings show the upregulation of CRACD in both iPSCs and terminally differentiated motor neurons. While the exact role of CRACD in neurodegeneration remains unexplored, cytoskeletal dyshomeostasis is a known factor in ALS pathogenesis. For example, mutations in profilin 1 (pfn1), another regulator of actin polymerization, are a known cause of familial ALS [64]. Specifically, it has been shown that motor neurons with pfn1 mutations contained decreased levels of pfn1-bound actin with subsequently smaller growth cones and a reduced F-/G-actin ratio, indicating significant cytoskeletal perturbations [64]. Another example is the ALS2 protein, which regulates actin-based neurite outgrowth via the Rab5 GTPase signaling [59,65,66]. These proteins act to regulate actin polymerization and have been associated with early endosome dynamics. Mutations in ALS2 are also a rare cause of juvenile ALS, where it is believed that it causes the unlinking of ALS2 and Rab5, leading to altered actin-based cargo movement and excitatory synaptic signaling [59,67]. Importantly, reports have shown that neurons lacking ALS2 demonstrate greater numbers of glutamate receptors and sensitivity to oxidative stress—both factors which are partially regulated by actin filament dynamics [59,68,69,70,71,72,73]. Based on these reports, it appears that the regulation of actin lies at the crossroads of several different disease-contributing pathways in ALS-affected motor neurons. The fact that our data show the upregulation of CRACD suggests that mutant FUS may exert currently unknown and potentially significant effects on cytoskeletal homeostasis. Therefore, CRACD involvement in neurodegeneration may prove to be a fruitful avenue for additional investigation.

GPR149, an orphan G-protein-coupled receptor (GPCR), has a limited documented functional role [74]. Many orphan GPCRs, including GPR149, have been investigated as potential drug targets due to their unknown ligands and functions [75]. Some studies have reported that several orphan GPCRs are highly expressed in the prefrontal cortex of the mouse brain, which is involved in learning and memory [76]. GPR149 is known to regulate myelination and remyelination [77] and is enriched in oligodendrocyte precursor cells (OPCs), where it negatively regulates OPC to oligodendrocyte differentiation as well as myelination and remyelination. GPR149 deficiency promotes OPC-to-oligodendrocyte differentiation and earlier myelin development. Blocking GPR149 may even promote myelin repair in demyelinating diseases. GPR149 has also been implicated in neuroendocrine signaling and was detected in the ventromedial hypothalamus transcriptome of mice, where it is highly expressed in inhibitory interneurons. Gene variants of GPR149 have also been reported in studies investigating migraine disorder susceptibility [78,79]. Overall, our data show that cells carrying FUS mutants show an increase in lnc-GPR149 and in its predicted mRNA target, GPR149. These increases in GPR149 are of particular interest given its contribution to inhibitor interneurons and myelination, as excitotoxicity and myelination defects have been noted in some cases of ALS [80,81]. Overall, our findings present a compelling case for further exploration of the role of GPR149 in neurodegeneration.

GO and KEGG enrichment analyses of DEGs indicated that FUS mutations likely affect pathways related to neuronal development and carcinogenesis. These findings suggest that FUS mutations might have broader implications in cellular processes beyond neurodegeneration, especially in light of the fact that FUS itself is an oncogene. Furthermore, IPA and GO network analysis of lncRNA-targeted mRNAs revealed significant biological processes involving RNA metabolism, lncRNA regulation, and DNA damage repair. These results support the idea that FUS mutations contribute to the pathophysiology of neurodegenerative diseases through multiple mechanisms, including the dysregulation of RNA metabolism and impaired DNA repair.

It is important to note that while this study has identified some novel pathways involving lncRNAs in FUS-associated neurodegeneration, additional research will be required to further elucidate how these interactions contribute to neurodegenerative disease. Taken together, our data highlight the extensive and varied aberrations caused by mutant FUS. While our data highlight several important aspects of neurodegenerative pathophysiology, this study does have three main limitations. First, the iPSCs utilized in our research may not completely represent the neuronal and glial environment in vivo, so future studies using in vivo models could offer more comprehensive insights into the molecular repercussions of FUS mutations. Second, our focus was primarily on the impact of FUS mutations on the transcriptional landscape of iPSCs and the subsequent correlation with differentiated iMNs; future work will include a full exploration of FUS-induced changes in the transcriptomic landscape of these iMNs. Lastly, determining the functional implications of the discovered TAR pairs and their potential contribution to neuronal dysfunction and degeneration will be an important area of future inquiry. 

## 5. Conclusions

In conclusion, our study provides additional insight into the molecular mechanisms underlying the pathophysiology of FUS-associated neurodegeneration. By investigating the impact of FUS mutations on the transcriptional landscape of iPSCs and their persistence in differentiated motor neurons, our findings contribute to the growing body of knowledge needed to develop effective therapies for these devastating disorders. Importantly, this study highlights the promise of the transcriptional profiling of FUS mutant iMNs, as our data indicate that at least six genes critical to neuronal function and disease are dysregulated at both the iPSC and motor neuron levels. Taken together, these data emphasize the importance of understanding how lncRNAs act to modulate gene expression in each cell differentiation state and how this intricate network of lncRNAs and mRNAs help explain the complexity of neurodegenerative pathophysiology. 

## Figures and Tables

**Table 1:** Summary of reads after quality control. Raw and filtered reads obtained from BGISEQ-500 platform.

Samples	Total Raw Reads	Total Clean Reads	Total Clean Reads Ratio	Total Mapping Reads	Uniquely Mapping Reads
Control	106,975,508	99,081,102	92.620%	92.25%	80.16%
FUS R521H	108,244,484	99,842,418	92.238%	93.48%	80.24%
FUS P525L	92,527,778	85,427,768	92.327%	94.21%	80.90%

**Table 2:** Summary of identified mRNA and lncRNA transcripts in human iPS cells. Quantitative analysis of all transcripts separated by sample.

Sample	Known lncRNA	Known mRNA	Novel lncRNA	Novel mRNA
Control	11,399	15,274	1703	4035
FUS R521H	11,516	15,177	1716	4033
FUS P525L	11,098	15,163	1697	4034

## Data Availability

These sequence data have been submitted to the BioProject database (hosted by the NCBI) (https://www.ncbi.nlm.nih.gov/bioproject/ accessed on 4 July 2023) under accession number PRJNA990965.

## Supplementary Materials

The following supporting information can be downloaded at: https://www.mdpi.com/article/10.3390/cells12202461/s1, Figure S1: Overview of experimental approach; Figure S2: Expression density at transcript level; Figure S3: Statistics of different genes between samples; Figure S4: RT-PCR validation using isogenic controls; Table S1: Selected primers used for RT-PCR validation; Table S2: List of differentially expressed genes (DEGs) and lncRNAs (DELs) for control vs. P525L; Table S3: List of differentially expressed genes (DEGs) and lncRNAs (DELs) for control vs. R521H; Table S4: Cis-trans TAR pair prediction; Table S5: Overlap TAR pair prediction.
