# Supplementary material for: lncRNA Sequencing Reveals Neurodegeneration-Associated FUS Mutations Alter Transcriptional Landscape of iPS Cells That Persists in Motor Neurons
Source: Cells. 2023 Oct 16;12(20):2461. doi: 10.3390/cells12202461 (PMC10604943; doi:10.3390/cells12202461)
Supplement: Supplementary file 1 [file cells-12-02461-s001.zip › cells-2645572-supplementary.pdf]

Supplementalmaterial

lncRNA Sequencing Reveals Neurodegeneration-associated FUS Mutations Alter Transcriptional Landscape of iPS Cells That Persists In Motor Neurons

*Vincent E. Provasek[1,2], Manohar Kodavati[1], Wenting Guo[3,45], Haibo Wang[1] Istvan Boldogh[5], Ludo Van Den Bosch[4], Gavin Britz[6,7] and Muralidhar Hegde[1,2,7]**

[1]Division of DNA Repair Research within the Center for Neuroregeneration, Department of Neurosurgery, Houston Methodist Research Institute, Houston, TX 77030, USA.

[2]School of Medicine, Texas A&M University, College Station, TX 77843, USA.
[3]KU Leuven-Department of Neurosciences, Experimental Neurology and Leuven Brain Institute (LBI), Leuven, 3000, Belgium.

[4]Stem Cell Institute, Department of Development and Regeneration, KU Leuven, Leuven, Belgium

[5]Department of Microbiology and Immunology, University of Texas Medical Branch, Galveston, TX 77555, USA.

[6]Department of Neurosurgery, Houston Methodist Research Institute, Houston, TX 77030, USA.
[7]Weill Cornell Medical College, New York, NY 10065, USA.

***Corresponding Author: M.L.H. Email:** mlhegde@houstonmethodist.org

Supplemental material associated with this article includes 5 Tables and 4 Figures.

Supplementary Figures

Figure S1. Overview of the study's experimental approach. A) Cultured patient-derived induced pluripotent stem cells (iPSCs) containing Fused-in Sarcoma (FUS) gene mutations R521H and P525L were utilized. Total RNA was extracted and sequenced using the BGISEQ-500 platform. B) The bioinformatics analysis workflow employed in the study.

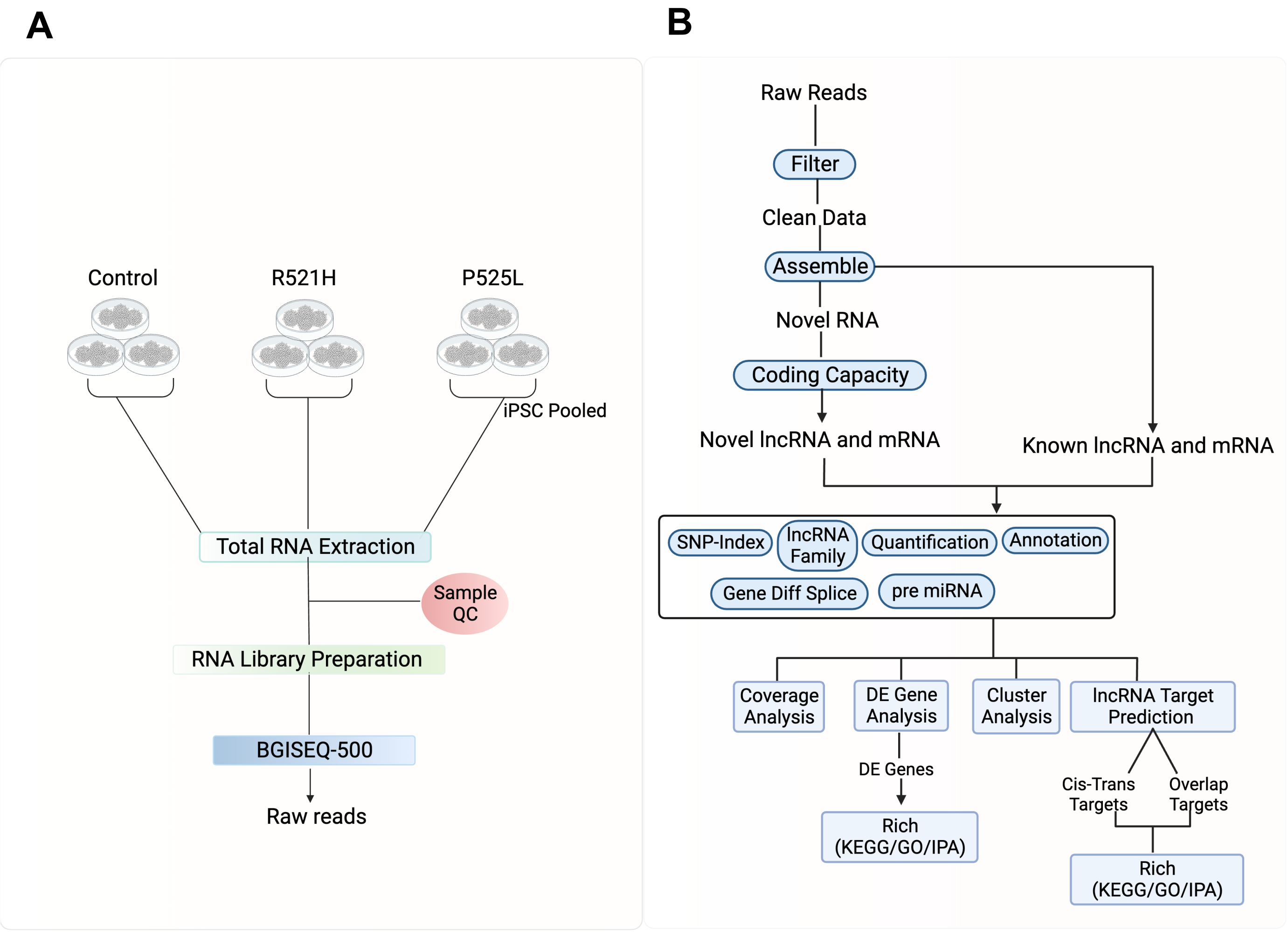

Figure S2. Expression density at transcript level.

Panels A-C show violin maps of expression levels of lncRNAs and mRNAs in control, P525L, and R521H, respectively. The y-axis represents the average log 2 (FPKM) values. T-test p-values <0.05 are considered significant.

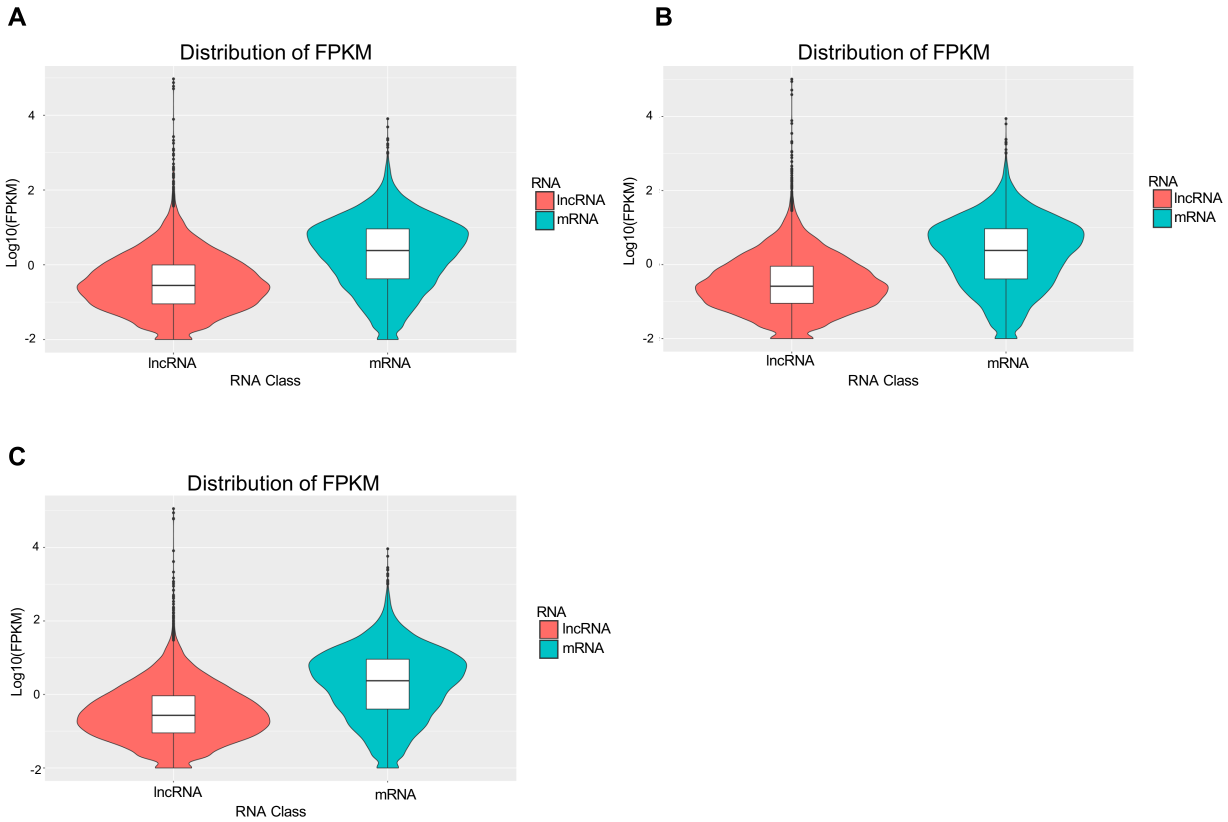

Figure S3. Statistics of different genes between samples.

The histogram shows changes in number of different RNAs identified between sample comparisons.

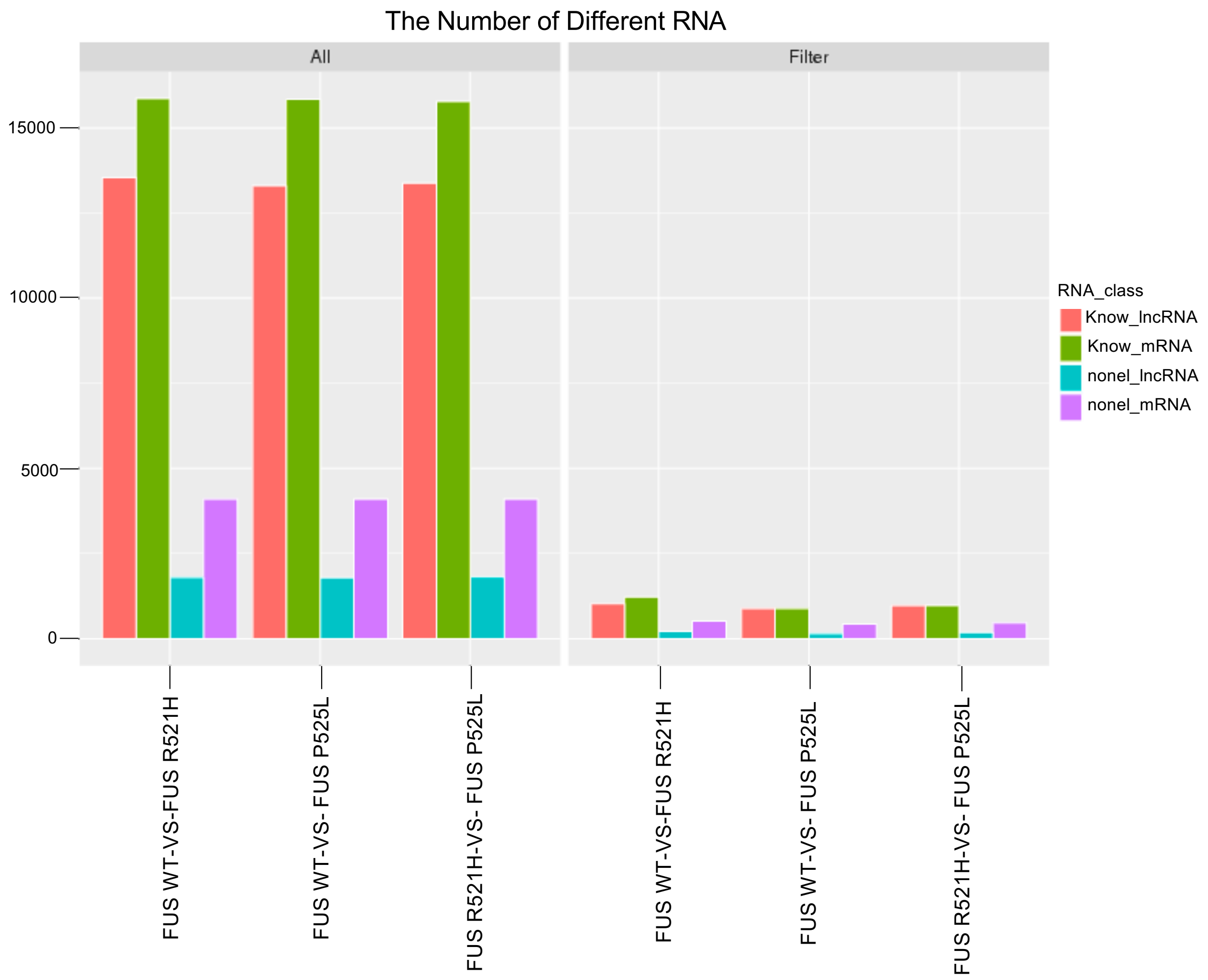

Figure S4. RT-PCR validation using isogenic controls.

The histograms show relative fold change of lncRNA and mRNA target gene expression in iPSC samples from control patients (blue) a nd from isogenic controls P525P (red) a nd R521R (green).

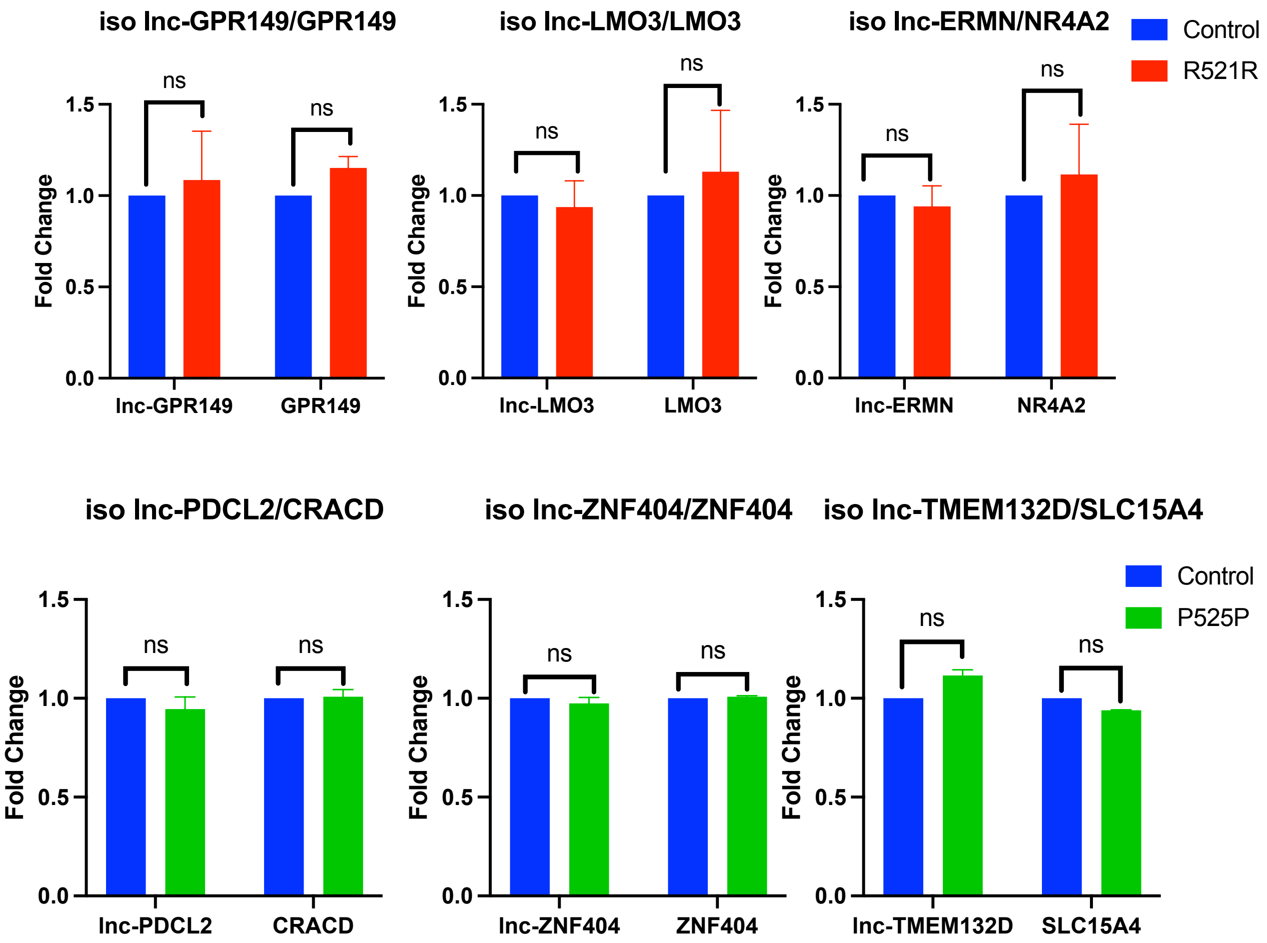

Table S1: Selected Primers used for RT-PCR Validation

lncRNA Primers	Forward		Reverse
lnc-GPR149-Fwd	CAT CTG GTG GTG ACT CCT ATA AAC	lnc-GPR149-Rev	CTG TCA GTC ACA ACT CTC AGA AG
lnc-ERMN-Fwd	TCT TTG TAG CAC CAG CAC TC	lnc-ERMN-Rev	GAC ACA CTC ACC GCC TAT TC
lnc-LMO3-Fwd	ACC AAG CCG AAA GGT TGT	lnc-LMO3-Rev	GGC AGT CTT CAT GCC AGT AT
lnc-TMEM132D-Fwd	AGG ACA TCG AGG AGA CAG AA	lnc-TMEM132D-Rev	GAG ACA GGT ACT GAC AGC ATA AG
lnc-PDCL2-Fwd	CTT AGG GAA CTG GCT CTG ATT T	lnc-PDCL2-Rev	CAC TGC CGT CCT GAA GTA AA
lnc-ZNF404-Fwd	CCA GGA ACC TGC TGT GAA TTA G	lnc-ZNF404-Rev	CCA GAT GGG AGT AGG AGA GAA A
**mRNA Primers**	**Forward**		**Reverse**
mRNA-GPR149-Fwd	CCT CTC TAT CGT GTA CGC TTT G	mRNA-GPR149-Rev	TGG TAG TTG GAG TGG AGT CT
mRNA-NR4A-Fwd	GTTCAGGCGCAGTATGGGTC	mRNA-NR4A-Rev	CTCCCGAAGAGTGGTAACTGT
mRNA-LMO3-Fwd	GCTCCACCCTGTACACTAAAG	mRNA-LMO3-Rev	GTAAGCTCATCCCTGCCTTT
mRNA-SLC15A4-Fwd	GCACTGAGAACAGGCAAGA	mRNA-SLC15A4-Rev	CTTTCCCTCTATCCAAAGGAGC
mRNA-CRACD-Fwd	CAGTGATTCCCAGGCTTTCT	mRNA-CRACD-Rev	GCCTCCCTTCTTCTTGTCTATC
mRNA-ZNF404-Fwd	CCA GGA ACC TGC TGT GAA TTA G	mRNA-ZNF404-Rev	CCA GAT GGG AGT AGG AGA GAA A

Table S2 Control vs P525L List of Differentially Expressed Genes and lncRNAs

geneID	geneLength	SA-Expression	SB-Expression	SA-FPKM	SB-FPKM	log2 Ratio(SB/SA)	Regulatio	P-value	FDR
6192	897	1	3677.99	0.04	129.97	11.66589103	Up	0	0
8653	4488	0	2938	0.01	19.76	10.94836723	Up	0	0
MXLOC_037825	9712	0	5984.27	0.01	18.47	10.85096815	Up	0	0
9086	1399	0	737	0.01	16.33	10.67330908	Up	5.88E-219	9.70E-217
100526842	1941	0	1000.58	0.01	15.81	10.62662165	Up	7.78E-297	1.88E-294
MXLOC_031435	519	0	192.86	0.01	12.34	10.26912668	Up	1.42E-57	5.75E-56
n379185	566	0	211.2	0.01	12.28	10.26209485	Up	3.35E-63	1.49E-61
MXLOC_023488	8271.06	0	2771.23	0.01	10.06	9.97441459	Up	0	0
51142	831	6	5974	0.23	229.01	9.95956102	Up	0	0
10752	7650	0	2310.77	0.01	9.07	9.824958741	Up	0	0
MXLOC_031523	2048	0	588.63	0.01	8.8	9.781359714	Up	7.76E-175	1.02E-172
8284	5489.13	1	1598	0.01	8.77	9.776433032	Up	0	0
n371595	5241	3	2870.55	0.02	16.5	9.688250309	Up	0	0
MXLOC_012296	2814	0	757.5	0.01	8.18	9.675957033	Up	7.03E-225	1.20E-222
23641	1381	1	712	0.02	15.99	9.642954223	Up	5.25E-209	8.28E-207
9284	1102	0	269.86	0.01	7.67	9.583082768	Up	2.24E-80	1.28E-78
n377198	1076	0	259.94	0.01	7.58	9.566054038	Up	2.05E-77	1.11E-75
83259	5594.55	11.45	5598.6	0.04	30.13	9.556984958	Up	0	0
MXLOC_025908	5024	4.94	3390.46	0.03	20.34	9.405141463	Up	0	0
85453	4463	0	973	0.01	6.58	9.361943774	Up	7.70E-289	1.74E-286
n370241	1205	0	216.94	0.01	5.62	9.13442632	Up	1.11E-64	5.05E-63
9087	1669	0	304.57	0.01	5.62	9.13442632	Up	9.69E-91	6.35E-89
7544	5218.51	2	962.59	0.01	5.56	9.118941073	Up	1.59E-280	3.48E-278
85460	6936	0	1281.68	0.01	5.55	9.116343961	Up	0	0
246126	865	0	147.27	0.01	5.41	9.079484784	Up	2.99E-44	9.39E-43
n341836	2466	0	414.47	0.01	5.12	9	Up	2.59E-123	2.43E-121
121256	5777	0	963	0.01	5.02	8.971543554	Up	7.04E-286	1.56E-283
3040	605	0	87.84	0.01	4.74	8.888743249	Up	1.75E-26	3.39E-25
n371628	882	0	120.99	0.01	4.35	8.764871591	Up	2.96E-36	7.66E-35
n339540	1456	0	194.46	0.01	4.13	8.689997971	Up	3.62E-58	1.49E-56
57209	4883	6	2538.32	0.04	15.67	8.613789464	Up	0	0
n381224	837	0	102.69	0.01	3.91	8.611024797	Up	6.33E-31	1.42E-29
MXLOC_036697	695	0	82.74	0.01	3.84	8.584962501	Up	5.29E-25	9.74E-24
MXLOC_037837	16045	0	2033.86	0.01	3.79	8.566054038	Up	0	0
n374606	249	0	22.78	0.01	3.45	8.430452552	Up	3.09E-07	1.95E-06
57482	7013	0	787.5	0.01	3.37	8.396604781	Up	9.19E-234	1.65E-231
MXLOC_009124	2498	1	267.1	0.01	3.26	8.348728154	Up	1.17E-77	6.36E-76
MXLOC_014045	2810	0	290.69	0.01	3.14	8.294620749	Up	1.36E-86	8.50E-85
22829	2023.96	4.32	809.02	0.04	12.24	8.257387843	Up	3.06E-231	5.46E-229
n408177	5631	0	568.73	0.01	3.04	8.247927513	Up	6.49E-169	8.30E-167
83742	3237	0	317.99	0.01	2.98	8.21916852	Up	1.37E-94	9.43E-93
MXLOC_031930	9068	0	859.36	0.01	2.84	8.14974712	Up	4.40E-255	8.72E-253
n386623	6660	0	616.72	0.01	2.78	8.118941073	Up	3.97E-183	5.46E-181
n371627	630	0	52.69	0.01	2.72	8.087462841	Up	4.05E-16	5.11E-15
90120	2845	0	254.22	0.01	2.72	8.087462841	Up	6.20E-76	3.28E-74
MXLOC_018160	5385	0	484.52	0.01	2.71	8.082149041	Up	4.85E-144	5.22E-142
23475	1555	0	133.9	0.01	2.66	8.055282436	Up	4.19E-40	1.19E-38
n407042	1769	0	148.64	0.01	2.58	8.011227255	Up	1.51E-44	4.78E-43
n405982	2303	0	192.85	0.01	2.56	8	Up	1.42E-57	5.75E-56
n384866	442	0	30.63	0.01	2.35	7.876516947	Up	1.32E-09	1.05E-08
n342404	3500	1.81	537.03	0.02	4.65	7.861086906	Up	2.62E-157	3.10E-155
MXLOC_009135	5466.46	9	2110.35	0.05	11.62	7.860466259	Up	0	0
7404	5686.57	4	1314	0.03	6.96	7.857980995	Up	0	0
339263	7912	0	609.64	0.01	2.31	7.851749041	Up	4.70E-181	6.35E-179
n408154	4822	0	366.23	0.01	2.29	7.839203788	Up	4.24E-109	3.43E-107
MXLOC_031465	2017	0	149.64	0.01	2.27	7.826548487	Up	7.66E-45	2.42E-43
n385888	482	0	32	0.01	2.22	7.794415866	Up	3.38E-10	2.84E-09
LXLOC_031442	236	0	13.72	0.01	2.22	7.794415866	Up	0.000143034	0.000622357

n379527	607	0	40.91	0.01	2.2	7.781359714	Up	1.45E-12	1.45E-11
n342576	2623	0	188.33	0.01	2.19	7.77478706	Up	2.17E-56	8.69E-55
n377827	2763	0	191.13	0.01	2.1	7.714245518	Up	2.80E-57	1.13E-55
n410162	2708	0	184.37	0.01	2.07	7.693486957	Up	3.31E-55	1.30E-53
n346355	274	0	15.32	0.01	2.06	7.686500527	Up	3.66E-05	0.000175528
n408895	2542	0	172.02	0.01	2.06	7.686500527	Up	1.18E-51	4.33E-50
MXLOC_004812	7129.57	0	481.68	0.01	2.03	7.665335917	Up	3.75E-143	4.01E-141
121260	2789	0	179.9	0.01	1.96	7.614709844	Up	1.00E-53	3.86E-52
7691	3031	2	385	0.02	3.86	7.592457037	Up	1.85E-110	1.51E-108
MXLOC_037797	1264	0	77.01	0.01	1.9	7.569855608	Up	1.60E-23	2.79E-22
n408118	2573	0	158.51	0.01	1.88	7.554588852	Up	1.66E-47	5.64E-46
340578	2599	0	158	0.01	1.85	7.531381461	Up	1.66E-47	5.64E-46
MXLOC_031433	371	0	19.72	0.01	1.85	7.531381461	Up	2.39E-06	1.37E-05
n378194	823	0	47.87	0.01	1.85	7.531381461	Up	1.22E-14	1.40E-13
n378584	1233	0	72.78	0.01	1.84	7.523561956	Up	4.84E-22	8.04E-21
MXLOC_001705	7788	0	465.68	0.01	1.8	7.491853096	Up	2.05E-138	2.12E-136
144423	2721	0	160.06	0.01	1.79	7.483815777	Up	4.23E-48	1.45E-46
83444	1266	0	72.33	0.01	1.78	7.475733431	Up	4.84E-22	8.04E-21
728927	2098	0	120.64	0.01	1.76	7.459431619	Up	2.96E-36	7.65E-35
114815	6797.87	0	393	0.01	1.74	7.442943496	Up	4.29E-117	3.70E-115
55780	2078	0	117.87	0.01	1.74	7.442943496	Up	2.29E-35	5.81E-34
LXLOC_009135	9488	5	1081.38	0.02	3.42	7.417852515	Up	0	0
MXLOC_003309	7623	0	434.83	0.01	1.71	7.417852515	Up	3.10E-129	3.05E-127
MXLOC_026024	2354	0	131.45	0.01	1.7	7.409390936	Up	1.64E-39	4.60E-38
n381340	941	0	49.09	0.01	1.65	7.366322214	Up	3.13E-15	3.73E-14
n367152	605	0	30.59	0.01	1.65	7.366322214	Up	1.32E-09	1.05E-08
LXLOC_031665	782	0	40	0.01	1.64	7.357552005	Up	1.45E-12	1.45E-11
n408324	1420	0	74.68	0.01	1.63	7.348728154	Up	1.24E-22	2.09E-21
n369385	394	0	18.59	0.01	1.62	7.339850003	Up	4.73E-06	2.58E-05
LXLOC_032867	1762	0	92.49	0.01	1.61	7.330916878	Up	5.79E-28	1.18E-26
n410075	2077	0	108.42	0.01	1.6	7.321928095	Up	1.06E-32	2.50E-31
n381958	1547	0	79	0.01	1.58	7.303780748	Up	4.09E-24	7.28E-23
n410542	3330	0	173.74	0.01	1.58	7.303780748	Up	5.99E-52	2.21E-50
MXLOC_037155	1784	0	90.92	0.01	1.57	7.294620749	Up	2.26E-27	4.53E-26
n367153	690	0	33.03	0.01	1.55	7.276124405	Up	1.71E-10	1.47E-09
55893	4802	0	245.06	0.01	1.54	7.266786541	Up	2.86E-73	1.47E-71
n340530	1485	0	71.12	0.01	1.48	7.209453366	Up	9.57E-22	1.57E-20
n377331	962	0	45.13	0.01	1.48	7.209453366	Up	4.78E-14	5.29E-13
n377275	957	0	44.43	0.01	1.47	7.199672345	Up	9.46E-14	1.03E-12
n385240	698	0	31.63	0.01	1.46	7.189824559	Up	6.69E-10	5.46E-09
MXLOC_026382	8163.72	0	390.98	0.01	1.44	7.169925001	Up	3.32E-116	2.83E-114
n4229	574	0	23.77	0.01	1.36	7.087462841	Up	1.56E-07	1.02E-06
n385891	513	0	21	0.01	1.36	7.087462841	Up	6.12E-07	3.74E-06
MXLOC_007731	7863	0	357.05	0.01	1.36	7.087462841	Up	1.96E-106	1.53E-104
n366112	562	0	22.9	0.01	1.34	7.06608919	Up	3.09E-07	1.95E-06
MXLOC_030800	1697	0	74	0.01	1.34	7.06608919	Up	1.24E-22	2.09E-21
LXLOC_035390	1865	0	81	0.01	1.33	7.055282436	Up	1.05E-24	1.90E-23
n376366	3319	0	143.45	0.01	1.31	7.033423002	Up	4.58E-43	1.39E-41
n335677	490	0	18.99	0.01	1.3	7.022367813	Up	4.73E-06	2.59E-05
84631	4050.31	0	174	0.01	1.3	7.022367813	Up	3.03E-52	1.13E-50
LXLOC_015176	3136	0	130.69	0.01	1.26	6.977279923	Up	3.24E-39	9.06E-38
n387126	605	0	22.85	0.01	1.23	6.942514505	Up	3.09E-07	1.95E-06
202559	2300	0	91.88	0.01	1.22	6.930737338	Up	1.14E-27	2.31E-26
n380876	450	0	16.23	0.01	1.22	6.930737338	Up	1.85E-05	9.30E-05
LXLOC_030897	4855	1	194	0.01	1.2	6.906890596	Up	3.53E-56	1.41E-54
LXLOC_037890	1524.46	0	59	0.01	1.2	6.906890596	Up	3.42E-18	4.80E-17
158931	1532	0	59.48	0.01	1.2	6.906890596	Up	3.42E-18	4.79E-17
n409637	1759	0	68.26	0.01	1.19	6.894817763	Up	7.40E-21	1.17E-19
n379132	719	0	26.39	0.01	1.18	6.882643049	Up	2.02E-08	1.44E-07
LXLOC_023467	401	0	13.68	0.01	1.17	6.87036472	Up	0.000143034	0.000622818

n406213	1491	0	56	0.01	1.16	6.857980995	Up		2.65E-17	3.55E-16
n373069	422	0	14.27	0.01	1.15	6.845490051	Up		7.23E-05	0.000331169
9413	2170.12	0.43	81.57	0.01	1.15	6.845490051	Up		1.05E-24	1.90E-23
79746	1654	0	61	0.01	1.14	6.832890014	Up		8.75E-19	1.26E-17
n383700	1412	0.79	102.92	0.02	2.26	6.820178962	Up		6.33E-31	1.42E-29
84445	2848	0	106.26	0.01	1.13	6.820178962	Up		4.14E-32	9.55E-31
n367818	676	0	23.34	0.01	1.12	6.807354922	Up		1.56E-07	1.02E-06
MXLOC_019220	3625	1.72	133	0.01	1.11	6.794415866	Up		2.81E-38	7.66E-37
n386391	1178	0	41.73	0.01	1.11	6.794415866	Up		7.32E-13	7.52E-12
100506164	1955	0	70	0.01	1.1	6.781359714	Up		1.89E-21	3.07E-20
n386118	2448	0	87.03	0.01	1.08	6.754887502	Up		1.75E-26	3.38E-25
23504	6322	0	227.27	0.01	1.08	6.754887502	Up		6.13E-68	2.93E-66
MXLOC_037798	6779	0	240.41	0.01	1.07	6.741466986	Up		8.66E-72	4.36E-70
n324702	426	0	13.1	0.01	1.05	6.714245518	Up		0.000143034	0.000622511
9177	1837	0	62.9	0.01	1.05	6.714245518	Up		4.42E-19	6.52E-18
1429	2274.53	1	156	0.02	2.09	6.707359132	Up		5.09E-45	1.62E-43
LXLOC_036454	621	7	733.65	0.37	38.52	6.701938622	Up		1.55E-203	2.39E-201
n410005	4278	0	147.17	0.01	1.04	6.700439718	Up		2.99E-44	9.38E-43
55139	2579	0	86.01	0.01	1.02	6.672425342	Up		3.46E-26	6.63E-25
n408124	7217	2.84	245.97	0.01	1.02	6.672425342	Up		2.16E-69	1.06E-67
MXLOC_037830	11834	7.94	799.58	0.02	2.02	6.658211483	Up		8.06E-223	1.36E-220
987	10099.31	0	331.86	0.01	0.99	6.62935662	Up		9.80E-99	7.15E-97
n371605	1494	0	47.4	0.01	0.98	6.614709844	Up		1.22E-14	1.41E-13
2560	2226	0	71	0.01	0.97	6.599912842	Up		9.57E-22	1.57E-20
n409314	824	0	25	0.01	0.97	6.599912842	Up		4.00E-08	2.77E-07
MXLOC_033062	2939	0	94.31	0.01	0.97	6.599912842	Up		1.48E-28	3.10E-27
n380296	872	0	26.65	0.01	0.97	6.599912842	Up		2.02E-08	1.44E-07
10742	2376.05	0	74.07	0.01	0.95	6.569855608	Up		1.24E-22	2.10E-21
MXLOC_000220	7837	0	244.51	0.01	0.94	6.554588852	Up		5.66E-73	2.89E-71
LXLOC_037827	534	0	15	0.01	0.93	6.539158811	Up		3.66E-05	0.000175456
10256	2536	0	77	0.01	0.92	6.523561956	Up		1.60E-23	2.79E-22
n379627	643	0	17.97	0.01	0.91	6.50779464	Up		9.35E-06	4.92E-05
LXLOC_037867	6834	5.32	414.45	0.02	1.82	6.50779464	Up		8.24E-114	6.86E-112
25780	4694	2	141	0.01	0.91	6.50779464	Up		4.57E-39	1.27E-37
n410986	1684	0	49.02	0.01	0.9	6.491853096	Up		3.13E-15	3.73E-14
MXLOC_022238	2867	0	84.89	0.01	0.9	6.491853096	Up		1.35E-25	2.54E-24
3294	1434	0	41	0.01	0.89	6.475733431	Up		7.32E-13	7.52E-12
8287	10048	0	295.5	0.01	0.88	6.459431619	Up		4.48E-88	2.84E-86
n410111	2819	0	81.61	0.01	0.88	6.459431619	Up		1.05E-24	1.90E-23
n407161	578	0	15.33	0.01	0.87	6.442943496	Up		3.66E-05	0.000175719
10586	2769	84	7368	0.94	80.9	6.427335136	Up		0	0
n326481	767	0	20.39	0.01	0.85	6.409390936	Up		1.21E-06	7.15E-06
n340297	2010	0	54.58	0.01	0.83	6.375039431	Up		1.03E-16	1.35E-15
653268	2420	1.85	131.53	0.02	1.66	6.375039431	Up		1.08E-37	2.91E-36
MXLOC_010648	1550	0	41.57	0.01	0.83	6.375039431	Up		7.32E-13	7.51E-12
LXLOC_016230	605	0	15.25	0.01	0.82	6.357552005	Up		3.66E-05	0.00017548
n385807	2040	0	53.84	0.01	0.81	6.339850003	Up		2.05E-16	2.62E-15
n378102	1899	0	50.34	0.01	0.81	6.339850003	Up		1.58E-15	1.93E-14
80778	1943	0	50.45	0.01	0.8	6.321928095	Up		1.58E-15	1.93E-14
651302	1640	0	42	0.01	0.79	6.303780748	Up		3.70E-13	3.88E-12
n336793	649	0	15.1	0.01	0.76	6.247927513	Up		3.66E-05	0.000175695
343171	945	0	22.78	0.01	0.76	6.247927513	Up		3.09E-07	1.95E-06
MXLOC_016469	5233	0	129.59	0.01	0.75	6.22881869	Up		6.40E-39	1.77E-37
113451	2182	0	53.09	0.01	0.74	6.209453366	Up		2.05E-16	2.63E-15
n409347	5945	0	146.99	0.01	0.74	6.209453366	Up		5.92E-44	1.84E-42
n340193	827	0	19.3	0.01	0.74	6.209453366	Up		2.39E-06	1.37E-05
152756	2174	5	367	0.07	5.16	6.203872333	Up		3.76E-100	2.79E-98
MXLOC_030586	2160	16.84	1243.6	0.24	17.6	6.196397213	Up		0	0
n408303	1772	0	42.02	0.01	0.73	6.189824559	Up		3.70E-13	3.88E-12
n378964	1322	0	30.94	0.01	0.73	6.189824559	Up		1.32E-09	1.05E-08

55582	4653	0	110.87	0.01	0.72	6.169925001	Up	2.71E-33	6.50E-32
n368017	607	0	13.26	0.01	0.71	6.14974712	Up	0.000143034	0.000622588
n385093	1719	0	39.35	0.01	0.7	6.129283017	Up	2.86E-12	2.80E-11
n341221	1198	0	26.53	0.01	0.69	6.108524457	Up	2.02E-08	1.44E-07
MXLOC_023493	6484.88	8.71	568.85	0.04	2.64	6.044394119	Up	6.81E-154	7.83E-152
n407987	1591	0	34.15	0.01	0.66	6.044394119	Up	8.65E-11	7.62E-10
127253	3433	2	147	0.02	1.3	6.022367813	Up	8.29E-41	2.40E-39
n377818	2906	0	61.78	0.01	0.65	6.022367813	Up	8.75E-19	1.26E-17
n337722	2281	0	47.44	0.01	0.64	6	Up	1.22E-14	1.40E-13
n383703	3607	0	75.46	0.01	0.63	5.977279923	Up	6.26E-23	1.07E-21
n339566	5298	0	111.34	0.01	0.63	5.977279923	Up	1.37E-33	3.31E-32
n364173	2186	0	44.05	0.01	0.62	5.95419631	Up	9.46E-14	1.03E-12
n338575	1549	0	30.88	0.01	0.62	5.95419631	Up	1.32E-09	1.05E-08
n339470	2888	0	59.2	0.01	0.62	5.95419631	Up	3.42E-18	4.79E-17
n385702	1308	0	25.95	0.01	0.62	5.95419631	Up	4.00E-08	2.77E-07
2555	2541.92	0	51	0.01	0.61	5.930737338	Up	8.00E-16	9.93E-15
57636	5906	0	120.03	0.01	0.61	5.930737338	Up	2.96E-36	7.66E-35
n386068	2486	0	48.98	0.01	0.6	5.906890596	Up	6.19E-15	7.22E-14
22986	5757	0	114	0.01	0.6	5.906890596	Up	1.77E-34	4.38E-33
n384454	3518	0	70.28	0.01	0.6	5.906890596	Up	1.89E-21	3.07E-20
n384541	1994	0	39.25	0.01	0.6	5.906890596	Up	2.86E-12	2.80E-11
5032	1977	0	38.71	0.01	0.6	5.906890596	Up	5.66E-12	5.43E-11
497190	1943	0	37.07	0.01	0.59	5.882643049	Up	1.12E-11	1.06E-10
n368072	1493	0	28.63	0.01	0.59	5.882643049	Up	5.17E-09	3.89E-08
80714	1730	0	33.41	0.01	0.59	5.882643049	Up	1.71E-10	1.47E-09
401720	4694	0	91.14	0.01	0.59	5.882643049	Up	1.14E-27	2.31E-26
MXLOC_009711	3065	0	59.39	0.01	0.59	5.882643049	Up	3.42E-18	4.79E-17
55626	5023	0	95.87	0.01	0.58	5.857980995	Up	7.48E-29	1.58E-27
n345807	1578	0	29.56	0.01	0.58	5.857980995	Up	2.62E-09	2.02E-08
n378557	2440	0	46.45	0.01	0.58	5.857980995	Up	2.42E-14	2.73E-13
7051	2777	0	52.86	0.01	0.58	5.857980995	Up	4.05E-16	5.11E-15
LXLOC_025065	3356	3.19	191.09	0.03	1.73	5.849665727	Up	4.18E-52	1.55E-50
n386119	2377	0	43.95	0.01	0.56	5.807354922	Up	1.87E-13	2.00E-12
n337711	1017	0	18.22	0.01	0.56	5.807354922	Up	4.73E-06	2.59E-05
27255	3530	0	65	0.01	0.56	5.807354922	Up	5.72E-20	8.71E-19
LXLOC_034376	993	0	17.34	0.01	0.55	5.781359714	Up	9.35E-06	4.91E-05
MXLOC_011575	2150	0	38.38	0.01	0.55	5.781359714	Up	5.66E-12	5.43E-11
n339699	2865	0	48.67	0.01	0.52	5.700439718	Up	6.19E-15	7.22E-14
100534012	1948	0	33.35	0.01	0.52	5.700439718	Up	1.71E-10	1.47E-09
n410625	1485	0	25.04	0.01	0.52	5.700439718	Up	4.00E-08	2.77E-07
9909	5688	0	97.99	0.01	0.52	5.700439718	Up	1.91E-29	4.11E-28
6575	3656	0	63.43	0.01	0.52	5.700439718	Up	2.24E-19	3.33E-18
2917	4151.46	45	2394	0.34	17.42	5.679066067	Up	0	0
5121	556	14	733	0.85	43.45	5.675749526	Up	8.30E-193	1.22E-190
MXLOC_029278	5169	0	87.24	0.01	0.51	5.672425342	Up	1.75E-26	3.39E-25
n371345	1167	0	18.99	0.01	0.51	5.672425342	Up	4.73E-06	2.59E-05
n376420	1118	0	18.12	0.01	0.51	5.672425342	Up	4.73E-06	2.59E-05
79258	2902	3	146	0.03	1.53	5.672425342	Up	4.02E-39	1.12E-37
n377479	3176	0	52.03	0.01	0.5	5.64385619	Up	4.05E-16	5.11E-15
3248	2665.42	0	44	0.01	0.5	5.64385619	Up	9.46E-14	1.03E-12
n335522	843	0	13.35	0.01	0.5	5.64385619	Up	0.000143034	0.000623433
n406922	2147	0	34.78	0.01	0.5	5.64385619	Up	8.65E-11	7.62E-10
n340814	2442	1	40	0.01	0.5	5.64385619	Up	3.08E-11	2.81E-10
85411	1527	0	24.76	0.01	0.5	5.64385619	Up	7.91E-08	5.32E-07
n378773	1045	0	16.36	0.01	0.49	5.614709844	Up	1.85E-05	9.30E-05
3231	1975	0	31.5	0.01	0.49	5.614709844	Up	6.69E-10	5.46E-09
n410774	951	0	14.85	0.01	0.49	5.614709844	Up	7.23E-05	0.000330998
MXLOC_026600	1353	0	21.36	0.01	0.49	5.614709844	Up	6.12E-07	3.73E-06
LXLOC_025669	13228.65	31.87	1067.48	0.05	2.42	5.596935142	Up	5.61E-266	1.15E-263
n367091	2114	0	33.39	0.01	0.48	5.584962501	Up	1.71E-10	1.47E-09

n338458	1873	0	29.53	0.01	0.48	5.584962501	Up	2.62E-09	2.02E-08
n384276	1289	0	19.99	0.01	0.48	5.584962501	Up	2.39E-06	1.37E-05
n405865	1401	0	21.82	0.01	0.48	5.584962501	Up	6.12E-07	3.73E-06
80864	1292	0	19.59	0.01	0.47	5.554588852	Up	2.39E-06	1.37E-05
MXLOC_000387	4391	0	68.8	0.01	0.47	5.554588852	Up	7.40E-21	1.17E-19
440695	1977	0	30.21	0.01	0.47	5.554588852	Up	1.32E-09	1.05E-08
n346481	1114	0	16.54	0.01	0.47	5.554588852	Up	1.85E-05	9.30E-05
n338984	1744	0	26.7	0.01	0.47	5.554588852	Up	2.02E-08	1.44E-07
n366291	1390	0	20.95	0.01	0.47	5.554588852	Up	1.21E-06	7.14E-06
MXLOC_001310	3293	0	50.51	0.01	0.46	5.523561956	Up	1.58E-15	1.93E-14
n339431	2666	0	40	0.01	0.46	5.523561956	Up	1.45E-12	1.45E-11
n366453	984	0	14.5	0.01	0.46	5.523561956	Up	7.23E-05	0.000331255
n370154	924	0	13.41	0.01	0.46	5.523561956	Up	0.000143034	0.000623125
n339976	1216	0	17.78	0.01	0.46	5.523561956	Up	9.35E-06	4.91E-05
1826	7109	0	110	0.01	0.46	5.523561956	Up	2.71E-33	6.50E-32
n407017	2356	0	34.87	0.01	0.45	5.491853096	Up	8.65E-11	7.62E-10
n410672	11598	0	175.7	0.01	0.45	5.491853096	Up	1.53E-52	5.74E-51
n369799	2028	0	29	0.01	0.44	5.459431619	Up	2.62E-09	2.02E-08
LXLOC_027755	10144	4.22	148.21	0.01	0.44	5.459431619	Up	2.04E-38	5.57E-37
n409306	3879	0	56.58	0.01	0.44	5.459431619	Up	2.65E-17	3.55E-16
n379927	3108	0	44	0.01	0.43	5.426264755	Up	9.46E-14	1.03E-12
55857	2200	0	30.13	0.01	0.42	5.392317423	Up	1.32E-09	1.05E-08
80728	5143	0	71.43	0.01	0.42	5.392317423	Up	9.57E-22	1.57E-20
n341917	1594	0	21.28	0.01	0.41	5.357552005	Up	6.12E-07	3.74E-06
n342628	2432	0	32.98	0.01	0.41	5.357552005	Up	3.38E-10	2.84E-09
9142	2447	0	33	0.01	0.41	5.357552005	Up	1.71E-10	1.47E-09
n340532	3711	0	50.23	0.01	0.41	5.357552005	Up	1.58E-15	1.93E-14
54531	2746	0	36.91	0.01	0.41	5.357552005	Up	2.21E-11	2.04E-10
n406467	6053	0	81.99	0.01	0.41	5.357552005	Up	1.05E-24	1.90E-23
4081	2907	3	117	0.03	1.22	5.345774837	Up	8.17E-31	1.83E-29
n410503	1524	0	19.75	0.01	0.4	5.321928095	Up	2.39E-06	1.37E-05
n410591	1072	0	13.66	0.01	0.4	5.321928095	Up	0.000143034	0.000623356
n340647	2122	0	28.06	0.01	0.4	5.321928095	Up	5.17E-09	3.89E-08
113675	1397	0	17.92	0.01	0.4	5.321928095	Up	9.35E-06	4.91E-05
MXLOC_008235	3496	10.13	411.69	0.09	3.57	5.309855263	Up	7.94E-106	6.15E-104
n338063	1682	0	21.56	0.01	0.39	5.285402219	Up	6.12E-07	3.74E-06
n341430	1799	0	23	0.01	0.39	5.285402219	Up	1.56E-07	1.02E-06
n342518	2109	0	27	0.01	0.39	5.285402219	Up	1.02E-08	7.51E-08
MXLOC_012008	1395	0	17.52	0.01	0.39	5.285402219	Up	9.35E-06	4.91E-05
n376075	1791	0	23	0.01	0.39	5.285402219	Up	1.56E-07	1.02E-06
n385949	1195	0	14.39	0.01	0.38	5.247927513	Up	7.23E-05	0.000331384
n408210	3139	0	38.89	0.01	0.38	5.247927513	Up	5.66E-12	5.43E-11
MXLOC_020648	5346	0	67.13	0.01	0.38	5.247927513	Up	1.46E-20	2.28E-19
n410267	1333	0	16	0.01	0.37	5.209453366	Up	1.85E-05	9.29E-05
n381067	1748	0	20.22	0.01	0.36	5.169925001	Up	1.21E-06	7.15E-06
n341741	1254	0	14.55	0.01	0.36	5.169925001	Up	7.23E-05	0.00033104
139065	8704.26	62	2295	0.22	7.91	5.168102266	Up	0	0
66004	4712.54	25.41	947.63	0.17	6.06	5.155711142	Up	2.11E-239	3.90E-237
n339647	2239	22	806	0.31	11	5.149091498	Up	4.50E-203	6.90E-201
147645	3674	0	42.54	0.01	0.35	5.129283017	Up	3.70E-13	3.88E-12
10749	7917	0	91.43	0.01	0.35	5.129283017	Up	1.14E-27	2.30E-26
222584	3167	0	37	0.01	0.35	5.129283017	Up	1.12E-11	1.05E-10
n379820	2209	0	25	0.01	0.35	5.129283017	Up	4.00E-08	2.77E-07
84033	20402	0	229.87	0.01	0.34	5.087462841	Up	1.57E-68	7.58E-67
n339549	1466	0	16	0.01	0.34	5.087462841	Up	1.85E-05	9.30E-05
n410691	4753	0	53.07	0.01	0.34	5.087462841	Up	2.05E-16	2.62E-15
388323	1222	0	13.28	0.01	0.34	5.087462841	Up	0.000143034	0.000623279
n384720	2704	0	30.59	0.01	0.34	5.087462841	Up	1.32E-09	1.05E-08
n384727	2977	0	33.29	0.01	0.34	5.087462841	Up	1.71E-10	1.47E-09
169026	5394.42	14	483	0.08	2.7	5.076815597	Up	2.83E-121	2.61E-119

56034	3008	0	33	0.01	0.33	5.044394119	Up	1.71E-10	1.47E-09
n378896	1531	0	16.43	0.01	0.33	5.044394119	Up	1.85E-05	9.30E-05
100528021	2282	0	24.39	0.01	0.33	5.044394119	Up	7.91E-08	5.32E-07
n386152	3367	0	36.46	0.01	0.33	5.044394119	Up	2.21E-11	2.04E-10
n407687	2660	6	199.62	0.07	2.28	5.025535092	Up	1.78E-50	6.35E-49
54925	3027	0	32.01	0.01	0.32	5	Up	3.38E-10	2.84E-09
n409114	1474	0	15.19	0.01	0.32	5	Up	3.66E-05	0.000175671
MXLOC_003080	1302	0	13.55	0.01	0.32	5	Up	0.000143034	0.000622281
n407776	3596	0	37.66	0.01	0.32	5	Up	1.12E-11	1.05E-10
126068	4448	15	466.41	0.1	3.16	4.981852653	Up	2.98E-115	2.52E-113
10590	1482	4	136	0.09	2.84	4.979822118	Up	5.25E-35	1.32E-33
n410886	1072	0.86	32.12	0.03	0.94	4.969626351	Up	3.38E-10	2.84E-09
3107	1525	27.23	857.97	0.56	17.38	4.955857445	Up	2.21E-211	3.51E-209
n410951	2201	0	22.06	0.01	0.31	4.95419631	Up	3.09E-07	1.95E-06
n342584	2565	0	26.09	0.01	0.31	4.95419631	Up	2.02E-08	1.44E-07
9122	2516.12	0	25.55	0.01	0.31	4.95419631	Up	4.00E-08	2.77E-07
MXLOC_017395	2060	1.29	41.88	0.02	0.62	4.95419631	Up	1.59E-11	1.49E-10
n385560	1438	0	14.45	0.01	0.31	4.95419631	Up	7.23E-05	0.000331212
n374209	1808	0	18.31	0.01	0.31	4.95419631	Up	4.73E-06	2.59E-05
n375019	1840	0	18.72	0.01	0.31	4.95419631	Up	4.73E-06	2.58E-05
51309	2141	122	3826	1.78	54.65	4.94027235	Up	0	0
n407872	3835	0	37.76	0.01	0.3	4.906890596	Up	1.12E-11	1.06E-10
n339251	1543	0	14.74	0.01	0.3	4.906890596	Up	7.23E-05	0.000331341
n384111	2427	0	23.84	0.01	0.3	4.906890596	Up	1.56E-07	1.02E-06
n374913	1485	0	14.38	0.01	0.3	4.906890596	Up	7.23E-05	0.000331298
MXLOC_031502	423	1.41	43.9	0.12	3.54	4.882643049	Up	4.26E-12	4.12E-11
344758	2323	53	1573	0.71	20.67	4.863575554	Up	0	0
LXLOC_034677	4523	13.59	392.37	0.09	2.62	4.863498	Up	9.94E-97	7.06E-95
n407227	4063	0	39.47	0.01	0.29	4.857980995	Up	2.86E-12	2.81E-11
n410878	1633	0	15.61	0.01	0.29	4.857980995	Up	3.66E-05	0.000175791
387522	2918	0	27.46	0.01	0.29	4.857980995	Up	1.02E-08	7.51E-08
n386256	2366	0	22.43	0.01	0.29	4.857980995	Up	3.09E-07	1.95E-06
MXLOC_031415	1590	21.57	639.93	0.43	12.42	4.852184704	Up	5.67E-157	6.67E-155
3039	576	2	60.16	0.12	3.43	4.837102265	Up	8.53E-16	1.06E-14
n340930	1965	0	18.23	0.01	0.28	4.807354922	Up	4.73E-06	2.59E-05
n411669	2314	0	21.28	0.01	0.28	4.807354922	Up	6.12E-07	3.74E-06
n339982	1940	0	18	0.01	0.28	4.807354922	Up	4.73E-06	2.59E-05
86614	1427	0	13	0.01	0.28	4.807354922	Up	0.000143034	0.000623049
n385789	6662	0	61.83	0.01	0.28	4.807354922	Up	8.75E-19	1.26E-17
92999	5507	0	50.44	0.01	0.28	4.807354922	Up	1.58E-15	1.93E-14
n407962	7111	0	66.48	0.01	0.28	4.807354922	Up	2.89E-20	4.47E-19
n406328	1708	0	15	0.01	0.27	4.754887502	Up	3.66E-05	0.000175743
9957	1965	0	17	0.01	0.27	4.754887502	Up	9.35E-06	4.92E-05
3437	2542	1.01	22.87	0.01	0.27	4.754887502	Up	3.83E-06	2.12E-05
n365084	2359	0	21.03	0.01	0.27	4.754887502	Up	6.12E-07	3.73E-06
n410561	1853	0	15.91	0.01	0.26	4.700439718	Up	3.66E-05	0.000175886
n407919	3108	0	26.57	0.01	0.26	4.700439718	Up	2.02E-08	1.44E-07
n408257	6837	0	58.74	0.01	0.26	4.700439718	Up	6.77E-18	9.34E-17
n371653	1519	0	13	0.01	0.26	4.700439718	Up	0.000143034	0.00062351
MXLOC_005870	5911	0	51	0.01	0.26	4.700439718	Up	8.00E-16	9.92E-15
n386084	2235	0	19.02	0.01	0.26	4.700439718	Up	2.39E-06	1.37E-05
n375857	2455	2.3	61.84	0.03	0.77	4.68182404	Up	4.45E-16	5.60E-15
MXLOC_035597	1703	0	13.93	0.01	0.25	4.64385619	Up	0.000143034	0.000623587
n377716	2927	0	24.36	0.01	0.25	4.64385619	Up	7.91E-08	5.32E-07
60676	4370	0	36.63	0.01	0.25	4.64385619	Up	2.21E-11	2.04E-10
375611	2600.67	0	21	0.01	0.25	4.64385619	Up	6.12E-07	3.73E-06
3043	626	7	177	0.37	9.21	4.63760398	Up	3.86E-43	1.18E-41
n379148	2287	0	17.69	0.01	0.24	4.584962501	Up	9.35E-06	4.92E-05
9459	5272	0	41.67	0.01	0.24	4.584962501	Up	7.32E-13	7.52E-12
n344747	2730	0	21.75	0.01	0.24	4.584962501	Up	6.12E-07	3.74E-06

LXLOC_010147	2026	0	15.67	0.01	0.24	4.584962501	Up	3.66E-05	0.000175576
n381616	3256	0	25.97	0.01	0.24	4.584962501	Up	4.00E-08	2.77E-07
n387383	2171	0	17.06	0.01	0.24	4.584962501	Up	9.35E-06	4.91E-05
3083	2038	0	16.12	0.01	0.24	4.584962501	Up	1.85E-05	9.29E-05
55604	5413	0	41.13	0.01	0.23	4.523561956	Up	7.32E-13	7.52E-12
n410952	2451	0	18.52	0.01	0.23	4.523561956	Up	4.73E-06	2.58E-05
200186	2678	0	20.47	0.01	0.23	4.523561956	Up	1.21E-06	7.14E-06
MXLOC_037635	2369	0	17.91	0.01	0.23	4.523561956	Up	9.35E-06	4.91E-05
5747	4542	5.07	102.31	0.03	0.68	4.502500341	Up	2.19E-24	3.93E-23
MXLOC_006078	4638	25.46	591.79	0.17	3.85	4.501251794	Up	5.29E-139	5.47E-137
4319	1759	1	26	0.02	0.45	4.491853096	Up	2.90E-07	1.84E-06
LXLOC_026991	4748.09	0	34.43	0.01	0.22	4.459431619	Up	8.65E-11	7.62E-10
6262	16365	0	118.99	0.01	0.22	4.459431619	Up	1.16E-35	2.96E-34
152330	5113.05	72.09	1553.8	0.43	9.16	4.412939033	Up	0	0
401207	2082.9	5	43	0.03	0.63	4.392317423	Up	1.18E-08	8.62E-08
n337733	2768	0	18.77	0.01	0.21	4.392317423	Up	4.73E-06	2.59E-05
n387462	3143	0	21.29	0.01	0.21	4.392317423	Up	6.12E-07	3.74E-06
n339574	1987	0	13.58	0.01	0.21	4.392317423	Up	0.000143034	0.000623202
n345534	4859	0	33.58	0.01	0.21	4.392317423	Up	1.71E-10	1.47E-09
55630	2168	0	15	0.01	0.21	4.392317423	Up	3.66E-05	0.000175504
n342381	1994	0	13.43	0.01	0.21	4.392317423	Up	0.000143034	0.000621974
MXLOC_031497	3645	13.38	277.16	0.11	2.3	4.386058432	Up	1.39E-64	6.33E-63
n410638	3335	9.96	203.19	0.09	1.85	4.361456459	Up	3.03E-48	1.04E-46
LXLOC_037828	7653	63.13	1279.4	0.25	5.02	4.327687364	Up	1.41E-289	3.22E-287
n409156	2041	0	13	0.01	0.2	4.321928095	Up	0.000143034	0.000623818
MXLOC_018066	2432	0	15.94	0.01	0.2	4.321928095	Up	3.66E-05	0.000175647
n377191	4690	0	31.17	0.01	0.2	4.321928095	Up	6.69E-10	5.46E-09
MXLOC_024755	6123	0	39.74	0.01	0.2	4.321928095	Up	2.86E-12	2.80E-11
n341291	3170	0	20.65	0.01	0.2	4.321928095	Up	1.21E-06	7.14E-06
MXLOC_004820	8645	26	519.21	0.09	1.8	4.321928095	Up	4.87E-118	4.29E-116
79937	5229	0	34.72	0.01	0.2	4.321928095	Up	8.65E-11	7.61E-10
n326183	2860	1	18	0.01	0.19	4.247927513	Up	4.92E-05	0.000231294
n408111	3630	0	23.1	0.01	0.19	4.247927513	Up	1.56E-07	1.02E-06
9663	6229	0	39.3	0.01	0.19	4.247927513	Up	2.86E-12	2.80E-11
282973	6488	0	40.16	0.01	0.19	4.247927513	Up	1.45E-12	1.45E-11
57562	7043	0	45.58	0.01	0.19	4.247927513	Up	4.78E-14	5.30E-13
3434	1861	2	34	0.03	0.56	4.222392421	Up	1.49E-08	1.08E-07
n410438	2507	0	15.03	0.01	0.18	4.169925001	Up	3.66E-05	0.000175552
n340792	2569	0	14.94	0.01	0.18	4.169925001	Up	7.23E-05	0.000331083
126433	2338	0	13.55	0.01	0.18	4.169925001	Up	0.000143034	0.000622051
LXLOC_008834	3204	11.97	227	0.12	2.15	4.163230349	Up	7.99E-53	3.01E-51
n379258	424	0.81	15.19	0.07	1.22	4.123382416	Up	3.66E-05	0.000175839
MXLOC_026623	5906.66	49.91	884.7	0.26	4.51	4.116543905	Up	3.01E-195	4.46E-193
n339236	2683	0	15	0.01	0.17	4.087462841	Up	3.66E-05	0.000175862
LXLOC_034111	2899	0	15.89	0.01	0.17	4.087462841	Up	3.66E-05	0.000175815
6653	10971	0	61.26	0.01	0.17	4.087462841	Up	8.75E-19	1.26E-17
n410518	3045	0	17.31	0.01	0.17	4.087462841	Up	9.35E-06	4.91E-05
n338617	1541	1	16.74	0.02	0.34	4.087462841	Up	0.00017393	0.000745853
LXLOC_027781	775	2.56	44.74	0.11	1.85	4.071949842	Up	2.61E-11	2.40E-10
3773	3970.45	5	88	0.04	0.67	4.06608919	Up	1.52E-20	2.37E-19
n342663	4505	512.77	8716.81	3.51	58.39	4.056178375	Up	0	0
n346538	291	1	17.01	0.13	2.13	4.034269902	Up	9.26E-05	0.000416634
n342407	796	44.78	734.72	1.83	29.49	4.010310269	Up	3.06E-159	3.70E-157
n409724	2671	0	14.29	0.01	0.16	4	Up	7.23E-05	0.000331427
55182	3010	0	15.5	0.01	0.16	4	Up	3.66E-05	0.000175599
n410537	2837	0	14.94	0.01	0.16	4	Up	7.23E-05	0.000331126
64799	2616.94	0	13.45	0.01	0.16	4	Up	0.000143034	0.000622434
5649	11570.1	191	3124	0.51	8.09	3.98757055	Up	0	0
10468	1869.74	8	135	0.14	2.22	3.987060944	Up	1.53E-30	3.38E-29
n374596	2827	0	13.84	0.01	0.15	3.906890596	Up	0.000143034	0.000623664

n339737	3971	0	19.87	0.01	0.15	3.906890596	Up	2.39E-06	1.37E-05
4899	3530	0	17.94	0.01	0.15	3.906890596	Up	9.35E-06	4.91E-05
n406646	2703	0	13.03	0.01	0.15	3.906890596	Up	0.000143034	0.000622741
9630	2491	18	282	0.23	3.45	3.906890596	Up	4.02E-61	1.73E-59
340419	3149	29.3	448.55	0.29	4.32	3.896906507	Up	7.62E-96	5.31E-94
MXLOC_003020	1854.45	16.56	258.9	0.29	4.29	3.886852842	Up	1.80E-56	7.23E-55
MXLOC_016265	901	3.65	53.52	0.13	1.88	3.854149134	Up	7.65E-13	7.84E-12
LXLOC_011374	241	1.11	16.44	0.18	2.6	3.852442812	Up	0.00017393	0.000745943
n386555	1148	3.27	47.53	0.09	1.29	3.841302254	Up	3.28E-11	2.98E-10
7803	5082	116	1673	0.7	9.92	3.824913293	Up	0	0
147381	2894	478	6855	5.13	71.96	3.810164456	Up	0	0
n338421	2868	0.5	13.11	0.01	0.14	3.807354922	Up	0.000143034	0.000623741
n407171	3416	0	15.87	0.01	0.14	3.807354922	Up	3.66E-05	0.000175623
182	5986	0	28.72	0.01	0.14	3.807354922	Up	5.17E-09	3.89E-08
MXLOC_004314	12284.15	3.63	55.41	0.01	0.14	3.807354922	Up	2.17E-13	2.31E-12
n375625	2797	0	13.33	0.01	0.14	3.807354922	Up	0.000143034	0.000622895
n341715	4386	0	20.66	0.01	0.14	3.807354922	Up	1.21E-06	7.14E-06
n378856	3238	0	14.45	0.01	0.14	3.807354922	Up	7.23E-05	0.000330955
n377463	3044	0	13.91	0.01	0.14	3.807354922	Up	0.000143034	0.000622127
n337962	4245	24.97	350.48	0.18	2.49	3.790076931	Up	5.13E-74	2.65E-72
n341980	2509	3.62	45	0.04	0.55	3.781359714	Up	1.14E-10	9.93E-10
MXLOC_014012	2002	10.68	149.03	0.17	2.28	3.745427173	Up	1.63E-32	3.81E-31
n346235	8096	3	34.98	0.01	0.13	3.700439718	Up	9.61E-08	6.39E-07
64919	7665.77	7	100	0.03	0.39	3.700439718	Up	5.35E-22	8.87E-21
n410543	3636	0	16.01	0.01	0.13	3.700439718	Up	1.85E-05	9.30E-05
n380786	3299	0	13.82	0.01	0.13	3.700439718	Up	0.000143034	0.000622972
LXLOC_034225	4062	0	17.38	0.01	0.13	3.700439718	Up	9.35E-06	4.91E-05
MXLOC_037006	3066	56.12	743.51	0.57	7.36	3.690671942	Up	5.70E-151	6.43E-149
5593	3328	34.09	452.3	0.32	4.12	3.686500527	Up	1.53E-92	1.03E-90
9877	4776	39.47	504.85	0.25	3.19	3.673556424	Up	4.34E-102	3.28E-100
LXLOC_011311	962	2	27	0.07	0.89	3.668378509	Up	1.17E-06	6.91E-06
n407986	1725	11.14	135.5	0.2	2.42	3.596935142	Up	5.93E-28	1.21E-26
84842	1803	2	21	0.03	0.36	3.584962501	Up	4.51E-05	0.000213359
646658	2607	3	41	0.04	0.48	3.584962501	Up	1.35E-09	1.06E-08
85450	4364	0	17.33	0.01	0.12	3.584962501	Up	9.35E-06	4.92E-05
29940	4005	0	16.48	0.01	0.12	3.584962501	Up	1.85E-05	9.29E-05
LXLOC_004206	3952	0	15.25	0.01	0.12	3.584962501	Up	3.66E-05	0.000175432
326624	3045	19	239	0.2	2.38	3.572889668	Up	8.51E-49	2.96E-47
6863	1179.65	3543	42967.98	95.88	1137.48	3.56846746	Up	0	0
474	1065	4	46	0.12	1.36	3.502500341	Up	3.94E-10	3.29E-09
9248	2020	8	89	0.12	1.35	3.491853096	Up	2.75E-18	3.88E-17
MXLOC_008193	6453	20.3	237.83	0.1	1.11	3.472487771	Up	1.83E-47	6.22E-46
n1849	6087	0	22.83	0.01	0.11	3.459431619	Up	3.09E-07	1.95E-06
150221	5802	0	21.47	0.01	0.11	3.459431619	Up	6.12E-07	3.74E-06
221806	5583	2	21	0.01	0.11	3.459431619	Up	4.51E-05	0.000213273
57565	4261	6	62	0.04	0.44	3.459431619	Up	8.54E-13	8.73E-12
22837	4923	0	18	0.01	0.11	3.459431619	Up	4.73E-06	2.58E-05
n406648	5682	0	20.6	0.01	0.11	3.459431619	Up	1.21E-06	7.14E-06
n342427	1694	6.67	72.19	0.12	1.31	3.448460501	Up	2.14E-15	2.58E-14
6323	8090.87	17.99	202.73	0.07	0.75	3.421463768	Up	1.05E-40	3.03E-39
51294	5182	12	127	0.07	0.74	3.402098444	Up	4.29E-25	7.92E-24
LXLOC_024547	965	3	32	0.1	1.05	3.392317423	Up	3.20E-07	2.01E-06
n338589	2005	2.62	27.36	0.04	0.42	3.392317423	Up	1.17E-06	6.91E-06
LXLOC_036453	248	11	115	1.7	17.51	3.364572432	Up	9.47E-23	1.61E-21
n407543	2874	15.71	165.52	0.17	1.75	3.363748271	Up	1.22E-32	2.88E-31
794	1465.46	11	121	0.25	2.55	3.350497247	Up	2.67E-24	4.79E-23
n384198	673	1.67	16.94	0.08	0.81	3.339850003	Up	0.00017393	0.000746034
n376203	557	3.09	32.42	0.19	1.92	3.337034987	Up	3.20E-07	2.01E-06
116154	2462.5	105	1132	1.39	14.01	3.333300168	Up	2.80E-213	4.49E-211
LXLOC_010084	1245	1.72	16.06	0.04	0.4	3.321928095	Up	0.00017393	0.000746125

7984	5488	0	19.07	0.01	0.1	3.321928095	Up	2.39E-06	1.37E-05
441631	5562	0	17.72	0.01	0.1	3.321928095	Up	9.35E-06	4.92E-05
n372080	8793	0	30.24	0.01	0.1	3.321928095	Up	1.32E-09	1.05E-08
9759	8976	0	30.88	0.01	0.1	3.321928095	Up	1.32E-09	1.05E-08
n410141	5053	0	16	0.01	0.1	3.321928095	Up	1.85E-05	9.29E-05
643641	6897	0	23.29	0.01	0.1	3.321928095	Up	1.56E-07	1.02E-06
170961	4026	0	13.35	0.01	0.1	3.321928095	Up	0.000143034	0.000622664
LXLOC_036423	832	3.29	33.62	0.13	1.29	3.310787537	Up	1.75E-07	1.14E-06
201164	2577	32	325	0.39	3.84	3.299560282	Up	5.08E-61	2.18E-59
n341766	2277	3	29	0.04	0.39	3.285402219	Up	1.91E-06	1.10E-05
MXLOC_026420	2419	6.03	62.02	0.08	0.78	3.285402219	Up	8.54E-13	8.72E-12
64849	4084	4.52	39.66	0.03	0.29	3.273018494	Up	2.57E-08	1.81E-07
63982	5936	11.86	115.04	0.06	0.58	3.273018494	Up	9.47E-23	1.61E-21
n409080	1486	9.47	92.41	0.2	1.92	3.263034406	Up	2.59E-18	3.65E-17
n377986	1638	14.81	141.65	0.28	2.67	3.25334101	Up	3.29E-27	6.53E-26
MXLOC_011599	3906	52.62	508.57	0.42	3.94	3.229734397	Up	4.95E-93	3.36E-91
3652	3224.77	15.82	198.63	0.2	1.87	3.224966365	Up	2.76E-41	8.04E-40
n386501	1009	8.38	80.79	0.27	2.52	3.222392421	Up	5.78E-16	7.23E-15
2115	6486.79	205.5	1909.54	0.95	8.85	3.219678037	Up	0	0
n368913	554	3.13	29.73	0.19	1.77	3.219678037	Up	1.91E-06	1.10E-05
n378429	776	5.65	53.97	0.24	2.23	3.215937399	Up	3.33E-11	3.03E-10
6941	3106.52	26	235.84	0.25	2.3	3.201633861	Up	1.69E-42	5.03E-41
4907	3918	6	59	0.05	0.46	3.201633861	Up	5.03E-12	4.85E-11
n340942	1460	3	26.15	0.06	0.55	3.196397213	Up	1.11E-05	5.76E-05
n377746	1737	9.85	92.94	0.18	1.65	3.196397213	Up	2.59E-18	3.65E-17
n346101	396	2	19	0.18	1.65	3.196397213	Up	0.000148775	0.000645191
n387139	793	3.12	29.56	0.13	1.19	3.194378045	Up	1.91E-06	1.10E-05
n344610	4926	0	14.67	0.01	0.09	3.169925001	Up	7.23E-05	0.00033147
n410190	5251	0	15.44	0.01	0.09	3.169925001	Up	3.66E-05	0.000175767
7498	5717	12	103	0.06	0.54	3.169925001	Up	5.30E-19	7.76E-18
26281	1016	3	26	0.09	0.81	3.169925001	Up	1.11E-05	5.76E-05
LXLOC_036869	1436.85	53.94	414.97	1	8.94	3.160274831	Up	6.59E-69	3.20E-67
n338277	2524	2.33	21.25	0.03	0.26	3.115477217	Up	4.51E-05	0.00021333
n386276	5110	4.25	44.06	0.03	0.26	3.115477217	Up	1.31E-09	1.04E-08
n342849	4011	30	263.83	0.23	1.99	3.113062664	Up	9.16E-47	3.05E-45
MXLOC_011954	5293.6	52.74	480.3	0.32	2.73	3.092757141	Up	5.96E-86	3.70E-84
LXLOC_031802	3563.63	52	449	0.45	3.81	3.081794091	Up	3.42E-78	1.88E-76
MXLOC_011005	2739	9.04	76	0.1	0.84	3.070389328	Up	2.90E-14	3.26E-13
n407773	2608	6.92	56.37	0.08	0.66	3.044394119	Up	2.93E-11	2.68E-10
n384485	754	2.64	21.24	0.11	0.9	3.032421478	Up	4.51E-05	0.000213244
n373943	1083	5	42	0.15	1.22	3.023846742	Up	2.11E-08	1.50E-07
3062	1816	212	1738	3.66	29.4	3.005900601	Up	1.72E-290	3.99E-288
n383025	6196	0	16.39	0.01	0.08	3	Up	1.85E-05	9.29E-05
10320	5980.33	0	16	0.01	0.08	3	Up	1.85E-05	9.29E-05
n342880	1822	5.56	46.83	0.1	0.79	2.981852653	Up	2.07E-09	1.61E-08
MXLOC_013142	3435	20.56	169.29	0.19	1.49	2.971241007	Up	4.32E-30	9.46E-29
n339966	1572	6.47	51.64	0.13	1.01	2.957771765	Up	5.33E-10	4.39E-09
n346541	1210	3	24	0.08	0.62	2.95419631	Up	3.53E-05	0.000170036
58511	1009	4	32	0.13	1	2.943416472	Up	1.51E-06	8.80E-06
3305	2537	28.76	224.02	0.35	2.69	2.942179346	Up	1.97E-38	5.40E-37
n384122	1381	20.21	157.19	0.46	3.53	2.939962417	Up	3.94E-27	7.80E-26
63906	2129	4.19	31.97	0.06	0.46	2.938599455	Up	2.67E-06	1.52E-05
2669	2187	4	33	0.06	0.46	2.938599455	Up	8.50E-07	5.11E-06
7975	3350	18.42	143.68	0.17	1.3	2.934904972	Up	5.63E-25	1.03E-23
3759	5397	65	504	0.37	2.81	2.924972955	Up	3.18E-83	1.88E-81
MXLOC_018325	6195	28.27	218.41	0.14	1.06	2.920565533	Up	5.82E-37	1.53E-35
n406457	3267	34.69	268.12	0.33	2.49	2.915607813	Up	2.32E-45	7.44E-44
n367728	469	5.34	40.7	0.39	2.92	2.90442234	Up	6.64E-08	4.50E-07
MXLOC_020459	1770	13.38	103.05	0.24	1.79	2.898853277	Up	2.38E-18	3.37E-17
56999	7309	11	89	0.05	0.37	2.887525271	Up	3.49E-16	4.43E-15

MXLOC_032582	2520	14.41	109.68	0.18	1.33	2.885357434	Up	3.47E-19	5.14E-18
n406889	3499	13.95	102.05	0.12	0.88	2.874469118	Up	4.19E-18	5.84E-17
n381430	8670	16.4	126.19	0.06	0.44	2.874469118	Up	4.40E-22	7.34E-21
LXLOC_016125	5832.37	94.53	747.38	0.53	3.86	2.864536583	Up	5.05E-124	4.79E-122
8324	3851	47	352	0.38	2.76	2.860596943	Up	1.31E-57	5.31E-56
n408068	2494	7.27	53.05	0.09	0.65	2.852442812	Up	7.37E-10	6.00E-09
3433	3495	6	42	0.05	0.36	2.847996907	Up	8.73E-08	5.84E-07
n381333	2594	26.44	195.4	0.32	2.29	2.839203788	Up	1.28E-32	3.01E-31
LXLOC_027098	549	10.14	73.53	0.62	4.42	2.833706249	Up	6.80E-13	7.01E-12
MXLOC_034544	3146	29.7	213.45	0.29	2.06	2.828519532	Up	4.01E-35	1.01E-33
n340261	1124	4	28	0.11	0.78	2.8259706	Up	1.45E-05	7.43E-05
MXLOC_012908	8796	40.03	290.01	0.14	0.99	2.822001698	Up	5.91E-47	1.98E-45
n384750	9591	0	23.25	0.01	0.07	2.807354922	Up	1.56E-07	1.02E-06
n408096	7183	0	16.19	0.01	0.07	2.807354922	Up	1.85E-05	9.30E-05
11113	8708	0	19.95	0.01	0.07	2.807354922	Up	2.39E-06	1.37E-05
MXLOC_001299	7894	0	17.77	0.01	0.07	2.807354922	Up	9.35E-06	4.91E-05
n411582	5960	0	13.43	0.01	0.07	2.807354922	Up	0.000143034	0.000622204
1395	2995.03	4	28	0.04	0.28	2.807354922	Up	1.45E-05	7.43E-05
MXLOC_000774	5888	5.58	41.79	0.03	0.21	2.807354922	Up	3.74E-08	2.60E-07
2118	2300.42	261.01	1833.78	3.49	24.33	2.801437379	Up	8.21E-283	1.81E-280
2913	4260	638	4533	4.62	32.13	2.797956224	Up	0	0
n332618	1240	8	58	0.21	1.46	2.797507136	Up	1.82E-10	1.56E-09
590	2447	42	295	0.53	3.67	2.791715798	Up	6.01E-47	2.02E-45
440279	8140	67.38	465.43	0.25	1.72	2.782408565	Up	1.34E-72	6.79E-71
n383098	2405	21.52	151.64	0.28	1.92	2.777607579	Up	4.70E-25	8.67E-24
LXLOC_023125	351	5.72	39.65	0.58	3.96	2.771375625	Up	1.17E-07	7.74E-07
164633	2982	11	74	0.11	0.75	2.769387072	Up	1.53E-12	1.53E-11
84765	3069.8	49.59	358.11	0.52	3.54	2.767165832	Up	7.95E-58	3.25E-56
n373563	2469	4.15	27.74	0.05	0.34	2.765534746	Up	2.54E-05	0.000125273
n381793	2363	3.79	26.3	0.05	0.34	2.765534746	Up	1.11E-05	5.76E-05
MXLOC_023106	1884	4.42	28.82	0.07	0.47	2.74723393	Up	1.45E-05	7.43E-05
65999	1957.14	31	208	0.49	3.26	2.73401831	Up	9.77E-33	2.31E-31
n386086	4100	21.46	144.26	0.16	1.06	2.727920455	Up	2.23E-23	3.85E-22
11249	2560	4	28	0.05	0.33	2.722466024	Up	1.45E-05	7.43E-05
53405	5711	10	62	0.05	0.33	2.722466024	Up	2.84E-10	2.40E-09
n405918	1755	4	26	0.07	0.46	2.716207034	Up	4.42E-05	0.000209489
3038	3500.61	12.27	68.19	0.09	0.59	2.712718048	Up	1.34E-10	1.17E-09
n335583	694	3.21	21.16	0.15	0.98	2.707819249	Up	0.000194608	0.000827888
23150	5261	6.78	45.24	0.04	0.26	2.700439718	Up	1.63E-08	1.17E-07
MXLOC_019340	1653	15.53	103.29	0.3	1.93	2.685566442	Up	3.98E-17	5.28E-16
LXLOC_000820	1767	8	52	0.14	0.9	2.684498174	Up	4.98E-09	3.75E-08
n383750	2791	66.25	435.77	0.74	4.75	2.682330338	Up	4.37E-66	2.04E-64
1278	5411	18	115	0.1	0.64	2.678071905	Up	3.02E-18	4.24E-17
n337663	3411	8.77	57.26	0.08	0.51	2.672425342	Up	3.17E-10	2.67E-09
7018	2791	33	216	0.37	2.35	2.667063581	Up	1.79E-33	4.32E-32
MXLOC_023367	4813	4.35	30.63	0.03	0.19	2.662965013	Up	4.72E-06	2.58E-05
n409761	5122	13.87	84.62	0.08	0.5	2.64385619	Up	9.02E-14	9.83E-13
MXLOC_025909	5806	520.91	3292.54	2.76	17.07	2.628722886	Up	0	0
285220	3677	52	329	0.44	2.71	2.622717423	Up	3.37E-49	1.18E-47
MXLOC_019494	3026	6.69	42.65	0.07	0.43	2.618909833	Up	8.73E-08	5.84E-07
LXLOC_018015	464	47.31	294.38	3.5	21.35	2.608809243	Up	7.74E-44	2.40E-42
n380153	638	3.68	22.55	0.19	1.15	2.597562538	Up	0.000110615	0.00049135
n408902	6136	16	97.04	0.08	0.48	2.584962501	Up	3.75E-15	4.44E-14
n383760	5089	2.61	21	0.02	0.12	2.584962501	Up	4.51E-05	0.000213302
9919	9042	21.05	127.31	0.07	0.42	2.584962501	Up	2.17E-19	3.23E-18
79365	3780	15	90	0.12	0.72	2.584962501	Up	4.51E-14	5.01E-13
29119	3174	7	44	0.07	0.42	2.584962501	Up	1.08E-07	7.12E-07
n410632	5037	75.17	459.98	0.46	2.75	2.579725852	Up	1.21E-66	5.72E-65
MXLOC_023609	8371	275.14	1676.35	1.01	6.01	2.573009698	Up	5.24E-238	9.54E-236
730	4012	111.17	673	0.86	5.07	2.559577182	Up	2.34E-96	1.65E-94

2122	5251.39	45.35	287.48	0.28	1.65	2.558967292	Up	2.54E-43	7.78E-42
n410967	1329	12.03	72.63	0.29	1.7	2.551409941	Up	1.62E-11	1.51E-10
MXLOC_015152	3284	126.56	749.02	1.19	6.91	2.537724137	Up	1.04E-105	8.02E-104
n407077	2693	8.49	50.98	0.1	0.58	2.5360529	Up	1.48E-08	1.07E-07
56479	6556	11	63	0.05	0.29	2.5360529	Up	5.68E-10	4.68E-09
4311	5650.57	14	86	0.08	0.46	2.523561956	Up	1.10E-13	1.19E-12
55066	7999	61.78	367.79	0.24	1.38	2.523561956	Up	4.71E-53	1.79E-51
n335640	318	7.72	44.96	0.88	5.05	2.520707959	Up	1.08E-07	7.12E-07
83449	3455.19	82.19	477.06	0.73	4.18	2.517534573	Up	5.17E-67	2.46E-65
LXLOC_012364	4538	56.79	332.98	0.39	2.21	2.502500341	Up	1.04E-47	3.57E-46
n382030	3218	26.47	147.06	0.25	1.39	2.475084883	Up	2.51E-21	4.05E-20
n385211	972	13.41	74.8	0.44	2.43	2.465380885	Up	1.89E-11	1.76E-10
51337	2264	135	759	1.86	10.24	2.460841189	Up	2.95E-103	2.24E-101
n384107	1499	43.46	243.91	0.92	5.03	2.450852634	Up	2.79E-34	6.86E-33
n334776	505	16.96	94.04	1.14	6.2	2.443234391	Up	1.84E-14	2.10E-13
58157	1876	69	382	1.15	6.25	2.442222329	Up	2.86E-52	1.07E-50
n385566	1510	26.7	146.95	0.56	3.01	2.426264755	Up	4.22E-21	6.73E-20
113763	2873	16	86	0.17	0.91	2.420331799	Up	1.21E-12	1.22E-11
MXLOC_035820	887	4.73	25.03	0.17	0.9	2.404390255	Up	7.65E-05	0.000348197
MXLOC_024396	3584	8	44	0.07	0.37	2.402098444	Up	3.61E-07	2.26E-06
n375398	2063	4.7	24.69	0.07	0.37	2.402098444	Up	0.000131978	0.000579177
MXLOC_007016	2556.15	235.53	1067.75	2.41	12.72	2.399993619	Up	5.85E-123	5.47E-121
LXLOC_034997	6138.51	243.93	1252.57	1.17	6.14	2.391730126	Up	5.21E-159	6.24E-157
n379020	920	5.9	31.95	0.21	1.1	2.389042291	Up	1.01E-05	5.28E-05
LXLOC_025419	945	6	31	0.2	1.04	2.378511623	Up	3.28E-05	0.000158895
388336	7358	16.32	87.97	0.07	0.36	2.362570079	Up	7.20E-13	7.42E-12
n409342	5170	13.43	70.57	0.08	0.41	2.357552005	Up	1.52E-10	1.32E-09
LXLOC_037803	10535	511.47	2667.03	1.49	7.59	2.348787555	Up	0	0
163081	2689	31.37	159.82	0.36	1.81	2.329920886	Up	1.62E-21	2.64E-20
MXLOC_017688	812	11	56	0.44	2.2	2.321928095	Up	2.15E-08	1.53E-07
23334	10217	0	18.54	0.01	0.05	2.321928095	Up	4.73E-06	2.59E-05
353376	3074	11.93	60.32	0.12	0.6	2.321928095	Up	2.74E-09	2.10E-08
n378226	766	4.84	24.97	0.21	1.04	2.308122295	Up	0.000131978	0.000579249
10788	5769	40	199	0.21	1.04	2.308122295	Up	5.46E-26	1.04E-24
1773	3098	13.05	65.66	0.13	0.64	2.299560282	Up	1.97E-09	1.54E-08
n387663	8619	65.1	325.37	0.23	1.13	2.296617006	Up	1.27E-41	3.71E-40
MXLOC_034168	10266.65	480.35	2414.68	1.44	7.05	2.291554446	Up	2.73E-299	6.68E-297
n410461	2237	20.13	99.83	0.28	1.36	2.280107919	Up	1.37E-13	1.47E-12
1807	2123	28	138	0.41	1.99	2.279072616	Up	2.41E-18	3.41E-17
MXLOC_018997	1499	12.59	63.29	0.27	1.31	2.278535499	Up	1.81E-09	1.42E-08
MXLOC_037651	5240	132	657.33	0.78	3.78	2.276840205	Up	3.65E-82	2.13E-80
6383	3485	542	2676	4.81	23.25	2.273121917	Up	0	0
n384691	2730	10.37	51.62	0.12	0.58	2.273018494	Up	9.23E-08	6.15E-07
10253	2111	333	1630	4.93	23.62	2.260349413	Up	1.46E-198	2.19E-196
LXLOC_018054	1040	6	30	0.19	0.91	2.259867127	Up	5.50E-05	0.000256059
5317	5384	7	34	0.04	0.19	2.247927513	Up	2.08E-05	0.000103795
LXLOC_032728	1902	19	91	0.31	1.47	2.245476034	Up	2.58E-12	2.54E-11
LXLOC_029720	10695	187	913	0.54	2.56	2.245112498	Up	8.06E-112	6.65E-110
n339473	2253	16.86	80.75	0.23	1.09	2.244622369	Up	2.59E-11	2.38E-10
n406199	770	6.01	29.51	0.26	1.23	2.242074787	Up	9.17E-05	0.000413016
LXLOC_028296	6716.57	271.02	1379	1.31	6.17	2.235703678	Up	2.46E-173	3.18E-171
6041	4241	57	271	0.41	1.93	2.234905033	Up	1.13E-33	2.74E-32
3760	2890	156	749	1.68	7.87	2.227902403	Up	7.29E-91	4.78E-89
MXLOC_029223	9004.85	270.88	1219.73	0.87	4.06	2.222392421	Up	1.52E-139	1.59E-137
MXLOC_006989	5011	148.46	701.99	0.91	4.22	2.213304549	Up	3.03E-84	1.83E-82
MXLOC_007163	2991.75	10.24	73.29	0.16	0.74	2.209453366	Up	6.80E-13	7.01E-12
n375647	8111	22.07	99.33	0.08	0.37	2.209453366	Up	1.09E-12	1.11E-11
84466	7689	19	94	0.08	0.37	2.209453366	Up	5.74E-13	5.95E-12
10911	570	62.78	295.13	3.69	17.02	2.205538316	Up	1.54E-36	4.02E-35
23527	7170	11.11	55.5	0.05	0.23	2.201633861	Up	3.58E-08	2.49E-07

LXLOC_012496	4264	24.01	110.54	0.17	0.78	2.197939378	Up	3.85E-14	4.29E-13
n386463	1084	18.53	87.15	0.55	2.52	2.19592021	Up	6.58E-12	6.30E-11
MXLOC_017801	3492	34.98	163.49	0.31	1.42	2.195550809	Up	5.57E-21	8.85E-20
7068	7415.64	22	101	0.09	0.41	2.187627003	Up	4.12E-13	4.30E-12
388407	1530	70	324	1.44	6.54	2.183221824	Up	4.06E-39	1.13E-37
MXLOC_032109	4743	20.22	93.35	0.13	0.59	2.182203331	Up	2.71E-12	2.66E-11
n338697	2190	11.97	55.14	0.17	0.77	2.179323699	Up	3.58E-08	2.49E-07
3110	2128.03	69	317	1.01	4.56	2.174678531	Up	4.43E-38	1.20E-36
n375859	1710	17.47	79.87	0.32	1.44	2.169925001	Up	1.23E-10	1.07E-09
n407964	2542	10.19	45.42	0.12	0.54	2.169925001	Up	1.88E-06	1.09E-05
n387668	1914	67.56	310.9	1.11	4.98	2.165586066	Up	1.74E-37	4.63E-36
157506	3531	183.31	840.2	1.61	7.2	2.160936218	Up	4.54E-98	3.29E-96
MXLOC_001485	4434	120.17	550.47	0.84	3.75	2.158429363	Up	9.76E-65	4.46E-63
51473	4667.87	38	172.02	0.25	1.11	2.150559677	Up	4.14E-21	6.60E-20
n342897	1344	386.68	1750.37	9.13	40.44	2.147096232	Up	3.36E-200	5.07E-198
57666	4548	17.64	80.29	0.12	0.53	2.142957954	Up	7.50E-11	6.66E-10
3303	2429	1136.31	5110.34	14.58	64.14	2.137233634	Up	0	0
n407670	3959	27.54	120.75	0.21	0.92	2.131244533	Up	6.05E-15	7.07E-14
n409766	2015	22.11	97.51	0.34	1.48	2.121990524	Up	2.88E-12	2.82E-11
n363978	1304	21.33	94.38	0.52	2.25	2.113341473	Up	4.57E-12	4.42E-11
9758	8465	60.82	267.81	0.22	0.95	2.11042399	Up	1.69E-31	3.83E-30
n379950	5246	16.22	75.18	0.1	0.43	2.10433666	Up	3.17E-10	2.67E-09
64843	1798	8	35	0.14	0.6	2.099535674	Up	3.51E-05	0.000169277
55258	2112	12.97	55.61	0.19	0.81	2.091922489	Up	1.03E-07	6.81E-07
LXLOC_033375	1091	7	31	0.21	0.89	2.083416008	Up	9.37E-05	0.000421304
n380110	1079	7.02	30.65	0.21	0.89	2.083416008	Up	0.000153181	0.000663078
56142	4320.14	229.28	1398.31	2.31	9.77	2.080465711	Up	4.66E-199	7.01E-197
283373	8656	49.68	218.03	0.18	0.76	2.078002512	Up	5.39E-26	1.03E-24
55809	7557	21.77	96.73	0.09	0.38	2.078002512	Up	1.72E-12	1.72E-11
MXLOC_023334	5818	69.62	300.68	0.37	1.56	2.075948853	Up	1.60E-34	3.96E-33
n372916	1645	7.19	31.64	0.14	0.59	2.075288127	Up	9.37E-05	0.000421358
84976	4924	45.02	193.32	0.28	1.18	2.075288127	Up	1.69E-22	2.86E-21
n339405	1501	12	51	0.25	1.05	2.070389328	Up	7.22E-07	4.37E-06
27343	2749.85	48.54	205.08	0.55	2.27	2.045188774	Up	1.02E-23	1.79E-22
122786	4828	50	212	0.32	1.32	2.044394119	Up	2.50E-24	4.48E-23
n338713	906	36.88	155.63	1.32	5.44	2.043068722	Up	2.05E-18	2.92E-17
n387627	994	7.95	33.76	0.26	1.07	2.041027268	Up	3.45E-05	0.000166621
123036	4900.09	29	120	0.18	0.74	2.039528364	Up	4.15E-14	4.62E-13
n410021	2951	9.55	40.29	0.1	0.41	2.03562391	Up	8.10E-06	4.29E-05
n380454	2525	51	214	0.63	2.58	2.033947332	Up	2.51E-24	4.50E-23
LXLOC_024975	2344	30	125	0.4	1.63	2.026800059	Up	1.01E-14	1.16E-13
80036	5245.99	54.53	191	0.27	1.1	2.026472211	Up	1.21E-18	1.73E-17
n410215	3510	56.37	235.05	0.5	2.03	2.021479727	Up	1.44E-26	2.81E-25
n381013	1577	67.25	277.79	1.34	5.44	2.021373651	Up	1.23E-30	2.75E-29
n377704	855	10.87	44.73	0.41	1.66	2.017487427	Up	3.07E-06	1.72E-05
LXLOC_028968	613	10	41	0.54	2.18	2.013296823	Up	1.31E-05	6.74E-05
339896	3655	38	156	0.32	1.29	2.011227255	Up	8.51E-18	1.17E-16
n333471	568	97.55	399.71	5.76	23.14	2.006248148	Up	3.15E-43	9.65E-42
LXLOC_018802	1763	35.56	145.1	0.63	2.53	2.005713651	Up	9.00E-17	1.18E-15
n372036	676	19.44	79.59	0.95	3.81	2.003791579	Up	8.80E-10	7.11E-09
n407843	1729	31.57	128.15	0.57	2.28	2	Up	6.28E-15	7.32E-14
2569	3161	16	67	0.16	0.64	2	Up	1.55E-08	1.11E-07
LXLOC_020892	878	10	41	0.37	1.48	2	Up	1.31E-05	6.74E-05
56141	3829.89	92.34	549.58	1.09	4.34	1.993366908	Up	3.88E-78	2.13E-76
LXLOC_036460	2030	73.55	297.81	1.13	4.49	1.990392672	Up	2.88E-32	6.70E-31
n383674	1165	15.1	60.53	0.41	1.62	1.982297998	Up	1.67E-07	1.08E-06
n364178	1030	12.07	49	0.38	1.5	1.980891177	Up	1.88E-06	1.08E-05
n407119	3830	18.02	75.02	0.15	0.59	1.975752454	Up	2.29E-09	1.77E-08
2321	6430.63	17.94	126.86	0.15	0.59	1.975752454	Up	1.86E-21	3.02E-20
MXLOC_018655	4580	43.03	173.39	0.29	1.14	1.974909019	Up	3.10E-19	4.61E-18

4922	1239	1528	6133	39.25	154.23	1.974318859	Up	0	0
11166	2514	10	39.15	0.12	0.47	1.969626351	Up	3.37E-05	0.000162984
MXLOC_036521	8562	182.18	725.19	0.65	2.54	1.966316874	Up	7.02E-75	3.67E-73
n377428	6764	17.74	70.37	0.08	0.31	1.95419631	Up	9.54E-09	7.02E-08
LXLOC_033404	9624	99.59	395.14	0.32	1.23	1.942514505	Up	1.25E-41	3.69E-40
7881	4518	446	1746	3.04	11.66	1.93942456	Up	9.52E-175	1.24E-172
57646	4632	43.39	169.91	0.29	1.11	1.936434871	Up	1.96E-18	2.79E-17
266722	7800	1250	4892	4.92	18.83	1.936302779	Up	0	0
MXLOC_027170	3323	38.37	150.07	0.36	1.37	1.928107082	Up	1.38E-16	1.78E-15
MXLOC_012365	3139	35.51	137.45	0.35	1.33	1.925999419	Up	3.70E-15	4.39E-14
25791	2812	131	508	1.45	5.49	1.920753249	Up	1.23E-51	4.49E-50
126823	1338	11.99	45.71	0.28	1.06	1.920565533	Up	4.95E-06	2.70E-05
n346207	1097	31711.39	122311.04	925.21	3494.22	1.91711768	Up	0	0
1804	4504.42	1563	6071	10.77	40.67	1.916946741	Up	0	0
n409323	5198	22.68	84.62	0.13	0.49	1.914270126	Up	1.31E-09	1.04E-08
LXLOC_019186	1980	90	346	1.42	5.35	1.913647962	Up	2.14E-35	5.43E-34
6692	2472.46	13	52	0.17	0.64	1.912537159	Up	1.14E-06	6.79E-06
n346198	347	1002.61	3813.96	103.36	386.28	1.901969008	Up	0	0
222696	3161	26.33	101.39	0.26	0.97	1.899473124	Up	1.85E-11	1.72E-10
55568	5956	307	1174	1.59	5.93	1.899005339	Up	2.08E-115	1.76E-113
1008	3436	175	668	1.58	5.89	1.898343076	Up	2.55E-66	1.20E-64
MXLOC_015981	4305.92	39.27	154.2	0.29	1.08	1.896906507	Up	5.37E-17	7.08E-16
MXLOC_007628	1743	33.98	128.87	0.61	2.27	1.89581115	Up	3.87E-14	4.31E-13
148229	5090	11	44	0.07	0.26	1.893084796	Up	7.94E-06	4.22E-05
LXLOC_031120	6627	14.98	57.84	0.07	0.26	1.893084796	Up	2.72E-07	1.73E-06
n335664	595	17.37	65.46	0.97	3.6	1.891940254	Up	9.81E-08	6.52E-07
23140	11395	228.12	855.22	0.61	2.25	1.883043854	Up	6.84E-83	4.04E-81
n410994	1516	46.89	176.89	0.98	3.61	1.881145183	Up	1.14E-18	1.64E-17
23506	6515	84.22	318.75	0.4	1.47	1.87774425	Up	3.55E-32	8.21E-31
51676	2756	11	40	0.12	0.44	1.874469118	Up	5.07E-05	0.000238117
116984	7362	29	109	0.12	0.44	1.874469118	Up	6.65E-12	6.35E-11
n408333	1883	9.93	37.75	0.17	0.62	1.866733469	Up	3.48E-05	0.000167673
n377200	583	31071.39	115192.92	1783.06	6480.73	1.861801079	Up	0	0
9924	5296.39	59.87	214.94	0.34	1.22	1.843274496	Up	3.02E-21	4.85E-20
n345587	1138	19	69	0.53	1.9	1.841935154	Up	8.88E-08	5.94E-07
n346166	469	9	33	0.66	2.36	1.83824893	Up	0.000227174	0.000953672
MXLOC_035443	7974	168.59	615.74	0.65	2.32	1.835613182	Up	1.20E-58	4.97E-57
57495	7477	317	1154	1.3	4.64	1.835613182	Up	7.50E-108	5.96E-106
MXLOC_018946	3515	49.75	182.65	0.44	1.57	1.83518913	Up	1.01E-18	1.46E-17
n409622	1286	15.71	57.31	0.39	1.39	1.833538854	Up	6.70E-07	4.07E-06
n338479	2357	90.14	326.79	1.19	4.23	1.82969609	Up	1.61E-31	3.67E-30
79595	4130.65	65.08	238.19	0.49	1.74	1.828233652	Up	1.10E-23	1.94E-22
MXLOC_026877	4956	63.54	233.6	0.4	1.42	1.827819025	Up	1.89E-23	3.28E-22
n341331	6237	478.19	1735.63	2.36	8.37	1.826440763	Up	1.08E-160	1.31E-158
3655	5747.57	106.65	378.06	0.56	1.98	1.822001698	Up	1.05E-35	2.69E-34
345557	7739	2608	9409	10.34	36.5	1.819660278	Up	0	0
n410445	773	31.85	114.34	1.35	4.74	1.811927652	Up	3.86E-12	3.75E-11
MXLOC_017734	7432	112.79	407.75	0.47	1.65	1.811733363	Up	4.42E-39	1.23E-37
LXLOC_036427	492	10.85	38.7	0.75	2.63	1.810100299	Up	5.37E-05	0.000250522
MXLOC_020489	9787.09	190.93	686.51	0.6	2.1	1.807354922	Up	2.80E-64	1.27E-62
166929	5955	27	96	0.14	0.49	1.807354922	Up	4.12E-10	3.43E-09
LXLOC_027158	3635	116.26	413.55	0.99	3.44	1.796908135	Up	8.55E-39	2.36E-37
MXLOC_018456	4057.92	293.63	1039.45	2.23	7.74	1.795289856	Up	1.39E-94	9.57E-93
201456	1733.24	26	92	0.47	1.63	1.794139303	Up	1.06E-09	8.48E-09
1129	2724.3	243	857	2.77	9.57	1.788632948	Up	7.25E-78	3.95E-76
51088	7682.45	2523.8	8968.53	10.16	35.05	1.786514042	Up	0	0
MXLOC_034851	1836	19.94	69.77	0.34	1.17	1.782901878	Up	8.88E-08	5.94E-07
n344473	977	23	81	0.76	2.61	1.779978483	Up	1.17E-08	8.51E-08
MXLOC_016173	8098.74	514.06	1784.22	1.93	6.61	1.776049424	Up	2.97E-157	3.51E-155
MXLOC_034554	13150.62	284.43	991.82	0.66	2.26	1.775784843	Up	1.03E-88	6.61E-87

599	3560	59.69	208.8	0.52	1.78	1.775293713	Up	4.20E-20	6.43E-19
n384662	1357	10.29	35.91	0.24	0.82	1.772589504	Up	0.00021129	0.000891869
n385115	3370	197.01	687.71	1.81	6.18	1.771617141	Up	5.30E-62	2.30E-60
n379862	2066	11	39	0.17	0.58	1.770518154	Up	7.97E-05	0.000361667
LXLOC_022329	1068	1575.61	5488.14	47.28	161.27	1.770176148	Up	0	0
n373932	910	30.96	107.82	1.1	3.75	1.769387072	Up	3.77E-11	3.41E-10
57509	4082.69	315	1066	2.32	7.89	1.765900495	Up	4.82E-92	3.21E-90
MXLOC_000242	3477	62	214.43	0.55	1.87	1.765534746	Up	3.45E-20	5.29E-19
MXLOC_030078	6436.3	162.11	551.9	0.76	2.58	1.763299742	Up	1.07E-48	3.70E-47
n372904	1536	13.63	47.24	0.28	0.95	1.762500686	Up	1.15E-05	5.94E-05
MXLOC_032598	7006	145.5	505.46	0.64	2.17	1.761551232	Up	5.81E-46	1.90E-44
MXLOC_008799	7475	1409.42	4886.12	5.79	19.63	1.761424919	Up	0	0
MXLOC_004098	1150	13	45	0.36	1.22	1.760812336	Up	2.80E-05	0.000137147
n378539	854	15.12	51.88	0.57	1.93	1.759567023	Up	9.88E-06	5.17E-05
n407266	1727	35.52	123.48	0.65	2.2	1.7589919	Up	1.97E-12	1.96E-11
MXLOC_030481	5449	189.12	655.37	1.07	3.62	1.758378901	Up	8.67E-59	3.60E-57
MXLOC_006554	2745	82.42	283.26	0.93	3.14	1.755461938	Up	3.31E-26	6.36E-25
56062	3309.84	68	228	0.62	2.09	1.753162822	Up	9.42E-21	1.48E-19
n406571	1580	58.92	203.08	1.18	3.97	1.750352148	Up	1.65E-19	2.46E-18
100528030	5496	19.52	67.26	0.11	0.37	1.750021747	Up	2.16E-07	1.39E-06
n410019	6295	281.82	968.43	1.38	4.63	1.746343926	Up	1.85E-85	1.14E-83
7772	4964	322.21	1104.25	2	6.71	1.746312766	Up	1.09E-96	7.71E-95
n408157	2410	26.39	90.48	0.34	1.14	1.745427173	Up	2.55E-09	1.96E-08
n410727	3560	22.52	78.34	0.2	0.67	1.744161096	Up	1.94E-08	1.39E-07
MXLOC_019206	6073.09	29	101.91	0.15	0.5	1.736965594	Up	2.33E-10	1.98E-09
219790	6641	20	67	0.09	0.3	1.736965594	Up	4.80E-07	2.96E-06
57626	4150	152	517	1.13	3.76	1.734409889	Up	8.48E-46	2.75E-44
6332	7186	615.64	2092.82	2.63	8.75	1.734220217	Up	7.00E-180	9.36E-178
1075	2701.12	83	227.26	0.77	2.56	1.733213459	Up	4.49E-16	5.64E-15
MXLOC_024622	2660	60.12	202.76	0.7	2.32	1.728697978	Up	1.23E-18	1.76E-17
MXLOC_000667	4024	253.44	857.86	1.95	6.44	1.723586564	Up	1.97E-74	1.03E-72
n385357	3092	69.57	235.5	0.7	2.31	1.722466024	Up	1.04E-21	1.71E-20
MXLOC_010997	5445	114.89	387.64	0.65	2.14	1.719099173	Up	1.28E-34	3.17E-33
MXLOC_023196	4508	225.18	754.48	1.54	5.05	1.713353037	Up	6.82E-65	3.13E-63
MXLOC_006090	6279	31.56	103.03	0.15	0.49	1.707819249	Up	4.72E-10	3.91E-09
n346385	2058	9.9	33.25	0.15	0.49	1.707819249	Up	0.000227174	0.000953785
n381354	1385	11.84	39.52	0.27	0.88	1.704544116	Up	7.97E-05	0.000361713
22895	4594	77	257	0.52	1.69	1.700439718	Up	4.40E-23	7.56E-22
MXLOC_013226	3382	12.75	43.79	0.12	0.39	1.700439718	Up	3.02E-05	0.000146878
n408350	2091	63.91	213.14	0.96	3.12	1.700439718	Up	1.16E-19	1.74E-18
n378917	788	36.07	119.29	1.49	4.84	1.699694717	Up	2.42E-11	2.23E-10
283078	3639	507	1681	4.31	13.98	1.697604586	Up	1.20E-140	1.26E-138
MXLOC_025213	5767	48.65	161.52	0.26	0.84	1.691877705	Up	4.33E-15	5.12E-14
MXLOC_013870	844	19.8	65.05	0.76	2.45	1.688710426	Up	5.21E-07	3.20E-06
MXLOC_025184	4074.48	65.51	217.58	0.5	1.61	1.687060688	Up	9.93E-20	1.50E-18
MXLOC_030477	7066	31.15	105.14	0.14	0.45	1.684498174	Up	2.01E-10	1.72E-09
n380253	921	49.33	161.68	1.73	5.56	1.684312845	Up	9.31E-15	1.07E-13
5138	4295.34	362.54	1183.01	2.59	8.32	1.68363143	Up	7.73E-98	5.56E-96
n346349	4859	45.08	149.94	0.29	0.93	1.681177816	Up	7.05E-14	7.73E-13
MXLOC_017682	1528	97.69	320.14	2.02	6.47	1.679410419	Up	5.45E-28	1.11E-26
29109	3854	37.29	121.91	0.3	0.96	1.678071905	Up	2.22E-11	2.05E-10
n385577	2904	73	238	0.78	2.49	1.674599713	Up	6.24E-21	9.87E-20
LXLOC_034066	3676	435.7	1423.15	3.67	11.71	1.673889108	Up	1.88E-117	1.64E-115
n339143	3069	16.15	51.33	0.16	0.51	1.672425342	Up	2.13E-05	0.00010612
7224	5840	52	167	0.27	0.86	1.671377253	Up	7.13E-15	8.29E-14
n346276	639	13.22	42.97	0.69	2.19	1.666262603	Up	0.000103719	0.000463283
n338774	1440	13	43	0.29	0.92	1.665580961	Up	6.74E-05	0.000310082
MXLOC_006159	8908	313.91	1016.75	1.08	3.42	1.662965013	Up	1.32E-83	7.89E-82
58473	2023.28	28	90	0.43	1.36	1.661198087	Up	1.19E-08	8.66E-08
79057	1287	27.59	89	0.68	2.15	1.660730008	Up	8.55E-09	6.31E-08

84441	5428	578	1870	3.28	10.37	1.660648174	Up	1.08E-151	1.23E-149
25829	3620.14	15.17	49.36	0.13	0.41	1.657112286	Up	2.35E-05	0.000116475
MXLOC_003584	2739	116.58	374.27	1.32	4.16	1.656045599	Up	1.36E-31	3.11E-30
9435	3043	228	733	2.32	7.31	1.655746601	Up	4.12E-60	1.75E-58
n338234	3484	23	72	0.2	0.63	1.655351829	Up	5.52E-07	3.39E-06
MXLOC_021728	2079	29.33	93.51	0.44	1.38	1.649092838	Up	7.12E-09	5.29E-08
168090	1839	13.11	41.2	0.22	0.69	1.649092838	Up	0.000158919	0.000685981
1871	5009	61.42	197.82	0.38	1.19	1.64689025	Up	2.15E-17	2.90E-16
89857	6320	16.59	52.03	0.08	0.25	1.64385619	Up	1.39E-05	7.14E-05
n407638	4953	196.34	625.51	1.22	3.81	1.64290985	Up	5.31E-51	1.92E-49
n381725	1277	63.96	203.56	1.59	4.96	1.641313355	Up	7.80E-18	1.07E-16
728492	1174.38	1061.63	3067.1	26.19	81.57	1.639022592	Up	2.32E-213	3.73E-211
23046	9895	231.62	738.38	0.72	2.24	1.637429921	Up	4.75E-60	2.01E-58
n337964	1941	16.65	53	0.27	0.84	1.637429921	Up	9.06E-06	4.77E-05
n387667	1071	55.46	176.19	1.66	5.16	1.636187824	Up	1.56E-15	1.90E-14
7556	4405	111.33	352.77	0.78	2.42	1.633461018	Up	3.02E-29	6.45E-28
n345577	6030	38.28	123.66	0.2	0.62	1.632268215	Up	2.03E-11	1.88E-10
5026	2285.52	255.28	817.09	3.53	10.92	1.629232768	Up	1.60E-66	7.55E-65
51555	3577	86.19	274.51	0.75	2.32	1.629162305	Up	3.08E-23	5.30E-22
140767	2408	58	183	0.75	2.32	1.629162305	Up	7.76E-16	9.63E-15
79827	2643	56	177	0.66	2.04	1.628031223	Up	2.14E-15	2.58E-14
MXLOC_011652	2525	36.01	112.32	0.44	1.36	1.628031223	Up	4.49E-10	3.72E-09
5732	2480	39	123	0.49	1.51	1.623694895	Up	4.24E-11	3.82E-10
MXLOC_004099	7103	508.87	1597.71	2.2	6.76	1.619519723	Up	2.81E-125	2.69E-123
56670	1650	1544	4843	29.46	90.44	1.618203562	Up	0	0
n368062	3235	15.19	48.56	0.15	0.46	1.61667136	Up	3.61E-05	0.000173529
LXLOC_015654	1232	23.16	72.75	0.6	1.84	1.61667136	Up	5.52E-07	3.39E-06
401612	6224	32.17	100.52	0.16	0.49	1.614709844	Up	3.50E-09	2.67E-08
741	1296	34	106	0.83	2.54	1.613645255	Up	1.25E-09	9.95E-09
MXLOC_023122	2648	72.21	226.44	0.85	2.6	1.612976877	Up	4.27E-19	6.30E-18
n406623	2706	15.68	48.58	0.18	0.55	1.611434712	Up	3.61E-05	0.000173505
283316	4583	27	84	0.18	0.55	1.611434712	Up	6.95E-08	4.70E-07
MXLOC_004729	1034	58.95	183.11	1.83	5.57	1.605833679	Up	7.76E-16	9.64E-15
147660	6741	256.67	797.6	1.17	3.56	1.605368711	Up	1.15E-62	5.07E-61
MXLOC_011281	2784	227.5	704.59	2.54	7.69	1.598155101	Up	2.75E-55	1.08E-53
n342494	4338	400.86	1232.99	2.85	8.58	1.590015728	Up	1.42E-94	9.73E-93
84159	7941	1618.13	4973.29	6.25	18.8	1.588804567	Up	0	0
253650	3738	26.97	80.99	0.22	0.66	1.584962501	Up	1.76E-07	1.14E-06
n406672	2988	22.81	70.94	0.24	0.72	1.584962501	Up	6.10E-07	3.73E-06
LXLOC_020219	2306	24.1	74.44	0.33	0.99	1.584962501	Up	4.96E-07	3.06E-06
2043	6348	643	1969	3.11	9.33	1.584962501	Up	6.90E-149	7.61E-147
n405572	4396	19.95	61.17	0.14	0.42	1.584962501	Up	2.90E-06	1.64E-05
338811	4056	933	2856	7.1	21.28	1.583607221	Up	5.82E-215	9.43E-213
283989	1941	61.65	189.24	1	2.99	1.580145484	Up	5.77E-16	7.21E-15
n375764	2517	49.69	150.76	0.61	1.82	1.577057303	Up	8.74E-13	8.93E-12
MXLOC_029787	13026	452.1	1376.48	1.06	3.16	1.575860294	Up	8.05E-104	6.12E-102
29015	2597	39	119	0.47	1.4	1.574694165	Up	2.18E-10	1.86E-09
5950	929	34	103	1.18	3.51	1.572684171	Up	4.26E-09	3.22E-08
MXLOC_001792	9309	401.65	1215.48	1.32	3.92	1.570315725	Up	2.53E-91	1.67E-89
MXLOC_027858	5539	469.39	1426.41	2.61	7.75	1.570146504	Up	2.37E-107	1.87E-105
MXLOC_034580	5145.8	622.41	1815.99	3.58	10.63	1.570110104	Up	9.46E-129	9.23E-127
MXLOC_015469	3814	39.06	119.38	0.32	0.95	1.569855608	Up	2.18E-10	1.86E-09
MXLOC_007676	1679.58	99.79	306.1	1.89	5.61	1.569614536	Up	7.54E-25	1.37E-23
n384200	1447	67.06	203.87	1.47	4.36	1.56851198	Up	1.38E-16	1.78E-15
132204	2607	22	66	0.26	0.77	1.566346823	Up	3.19E-06	1.78E-05
MXLOC_013656	4369	33.71	103.09	0.24	0.71	1.564784619	Up	2.09E-09	1.63E-08
n379122	2556	39.23	118.98	0.48	1.42	1.564784619	Up	3.26E-10	2.74E-09
54477	4278.04	294.4	811.41	1.94	5.73	1.562478487	Up	4.63E-54	1.79E-52
n384327	2260	12.88	39	0.18	0.53	1.557995453	Up	0.000176343	0.00075519
5764	1549	359	1079	7.31	21.51	1.557064213	Up	1.91E-80	1.09E-78

MXLOC_004762	3879	41.66	123.99	0.33	0.97	1.555518723	Up	1.75E-10	1.50E-09
n407074	3364	85.54	257.12	0.79	2.32	1.554200247	Up	1.49E-20	2.33E-19
n386504	984	47.71	142.9	1.56	4.58	1.553801569	Up	5.44E-12	5.23E-11
MXLOC_018546	1276	43.13	128.7	1.07	3.14	1.553153762	Up	9.37E-11	8.23E-10
MXLOC_007378	4012.63	133.7	397.62	1.02	2.99	1.551576332	Up	2.14E-30	4.72E-29
89884	1879	82	245	1.37	4	1.545824107	Up	2.25E-19	3.35E-18
4886	2958	1190	3551	12.49	36.45	1.545145338	Up	7.92E-258	1.61E-255
80333	2231.83	416.97	1222.98	5.74	16.74	1.544176886	Up	4.23E-88	2.69E-86
MXLOC_020166	3817	27.72	80.51	0.22	0.64	1.540568381	Up	3.54E-07	2.22E-06
137868	2178	22	66	0.32	0.93	1.539158811	Up	3.19E-06	1.78E-05
LXLOC_014925	2384.7	494.3	1528.31	6.73	19.55	1.538490198	Up	1.72E-117	1.50E-115
1740	7827.58	223.03	679.23	0.9	2.61	1.5360529	Up	3.78E-52	1.40E-50
10888	4282	297.35	878.01	2.14	6.19	1.532328613	Up	2.34E-64	1.06E-62
MXLOC_011992	4960	315.55	931.35	1.96	5.66	1.529948399	Up	4.10E-68	1.97E-66
441024	2361	63.92	187.45	0.84	2.42	1.526545814	Up	5.17E-15	6.08E-14
26002	3039	5103	15017	52.09	149.98	1.525691786	Up	0	0
n410133	1995	133.62	393.57	2.1	6.04	1.524159222	Up	1.03E-29	2.24E-28
338645	5257	128.47	375.8	0.75	2.15	1.519374159	Up	4.17E-28	8.55E-27
n409147	4505	41.83	123.99	0.29	0.83	1.517058436	Up	1.75E-10	1.50E-09
n341186	3053	20.76	60.65	0.21	0.6	1.514573173	Up	9.07E-06	4.78E-05
114781	8796.02	430.27	1254.96	1.5	4.28	1.512648296	Up	1.84E-89	1.19E-87
26230	5814.31	311.89	893.1	1.62	4.62	1.511899039	Up	7.30E-63	3.22E-61
MXLOC_009884	3712	30.65	90.88	0.26	0.74	1.509013647	Up	5.01E-08	3.43E-07
LXLOC_013241	4747	28.56	84.35	0.19	0.54	1.506959989	Up	1.41E-07	9.23E-07
n341956	2029	16	47	0.25	0.71	1.50589093	Up	0.000111989	0.000497079
MXLOC_011953	2835	96.56	280.68	1.06	3.01	1.505699222	Up	2.95E-21	4.75E-20
n371762	1714	17	49	0.31	0.88	1.505235308	Up	9.87E-05	0.000442529
n378828	2680	26.9	77.69	0.31	0.88	1.505235308	Up	5.95E-07	3.64E-06
284521	1884	44	127	0.73	2.07	1.503662399	Up	2.73E-10	2.31E-09
MXLOC_022707	3572.06	562.51	1286.25	3.85	10.9	1.501397784	Up	9.48E-62	4.11E-60
55130	3572	116.48	336.68	1.01	2.85	1.496606626	Up	5.72E-25	1.05E-23
23493	2672	24.36	69	0.28	0.79	1.496425826	Up	3.74E-06	2.07E-05
26974	2686	269	771.58	3.11	8.74	1.490718699	Up	2.24E-54	8.68E-53
25834	1934	998.91	2867.2	16.18	45.46	1.490386076	Up	7.92E-198	1.18E-195
n381294	2352	27	78	0.36	1.01	1.488286481	Up	7.86E-07	4.74E-06
2668	3772.49	223.25	637.38	1.83	5.11	1.481479643	Up	5.18E-45	1.65E-43
n342987	9848	386.1	1100.55	1.2	3.35	1.48112669	Up	3.34E-76	1.78E-74
n384946	2538	49.69	141.49	0.61	1.7	1.478653599	Up	3.13E-11	2.85E-10
200958	6338.87	28.89	82	0.14	0.39	1.478047297	Up	3.14E-07	1.98E-06
MXLOC_005083	6286	213.16	603.18	1.04	2.89	1.474485964	Up	3.62E-42	1.08E-40
n411745	2130	33.67	94.85	0.49	1.36	1.472752997	Up	7.74E-08	5.22E-07
LXLOC_028925	2781	28	79	0.31	0.86	1.472068444	Up	1.03E-06	6.11E-06
MXLOC_022117	3148	39.24	111.98	0.39	1.08	1.469485283	Up	5.22E-09	3.93E-08
LXLOC_028295	8405	35.85	101.27	0.13	0.36	1.469485283	Up	1.87E-08	1.34E-07
MXLOC_025199	4952	76	213	0.47	1.3	1.467778961	Up	1.10E-15	1.35E-14
LXLOC_034827	1426	29	81.46	0.64	1.77	1.46760555	Up	9.01E-07	5.41E-06
29951	3325.66	930	2692	8.87	24.53	1.467541224	Up	6.97E-188	9.81E-186
5774	6741.24	120.13	308.9	0.5	1.38	1.464668267	Up	2.27E-19	3.37E-18
1806	2692.89	28.57	70.41	0.29	0.8	1.4639471	Up	3.00E-05	0.000146111
26240	1618	106	298	2.06	5.68	1.463246592	Up	1.93E-21	3.13E-20
222183	3615	173.12	485.86	1.48	4.07	1.459431619	Up	8.96E-34	2.18E-32
55638	2891.2	658.61	1837.48	7.04	19.31	1.45570083	Up	2.59E-122	2.40E-120
n386957	1216	16.99	48	0.45	1.23	1.450661409	Up	7.45E-05	0.00033997
MXLOC_014432	4225.18	147.24	435.77	1.14	3.11	1.447880756	Up	8.88E-33	2.11E-31
MXLOC_035023	6643.02	419.54	1163.26	1.93	5.26	1.446461952	Up	1.85E-77	9.98E-76
MXLOC_026648	7607	340.56	946.05	1.37	3.73	1.444999737	Up	1.45E-63	6.46E-62
5069	10970	3802	10585	10.62	28.91	1.444784843	Up	0	0
348013	1039	22	61	0.68	1.85	1.443918619	Up	2.32E-05	0.000115338
MXLOC_017092	6035.25	42.86	136.98	0.25	0.68	1.443606651	Up	1.74E-12	1.74E-11
23116	6242	64.88	180.51	0.32	0.87	1.442943496	Up	1.53E-13	1.64E-12

57057	1823.65	2478	6882	42.64	115.92	1.442850157	Up	0	0
n408278	2226	17.29	47.22	0.24	0.65	1.437405312	Up	0.000217966	0.000918731
55013	1274	27	74	0.67	1.81	1.433756697	Up	3.73E-06	2.07E-05
n405708	1356	160.35	441.3	3.75	10.1	1.429392792	Up	4.02E-30	8.81E-29
n346460	478	42.26	115.91	3.02	8.13	1.428706803	Up	7.65E-09	5.67E-08
115908	1220	57	157	1.49	4.01	1.428289906	Up	1.15E-11	1.08E-10
n371302	4593	47.35	130.53	0.32	0.86	1.426264755	Up	5.96E-10	4.89E-09
n407558	1610	17.78	48.99	0.35	0.94	1.425305835	Up	0.000147027	0.00063824
MXLOC_006229	6005	173.96	477.52	0.89	2.39	1.425133377	Up	1.82E-32	4.26E-31
LXLOC_030592	694	146.1	399.92	6.94	18.61	1.423070487	Up	4.51E-27	8.91E-26
n338192	6065	50	135	0.25	0.67	1.422233001	Up	5.84E-10	4.80E-09
23196	4957	221.31	605.02	1.38	3.68	1.415037499	Up	2.60E-40	7.42E-39
387890	1573	60	163	1.2	3.2	1.415037499	Up	7.73E-12	7.36E-11
n372967	5934	16.69	46.92	0.09	0.24	1.415037499	Up	0.000167655	0.000721398
140699	2191.04	26.78	63.28	0.33	0.88	1.415037499	Up	0.000118982	0.000526398
55532	2854	57.06	155.31	0.62	1.65	1.412125904	Up	2.43E-11	2.24E-10
MXLOC_011396	4485	54.8	150.31	0.38	1.01	1.410283969	Up	2.46E-11	2.27E-10
MXLOC_009471	4158	50.87	138.78	0.38	1.01	1.410283969	Up	1.89E-10	1.62E-09
n408060	2399	42.42	114.68	0.55	1.46	1.408464845	Up	1.12E-08	8.14E-08
1620	3196	1412	3826	13.69	36.3	1.406847102	Up	1.88E-241	3.51E-239
8641	4578	1631	4421.4	10.99	29.14	1.406809491	Up	7.84E-279	1.71E-276
MXLOC_002865	5522	111.88	301.57	0.62	1.64	1.403355694	Up	1.42E-20	2.21E-19
27303	5676.92	1923.86	4523.65	9.07	23.99	1.403258702	Up	5.97E-224	1.01E-221
n410559	2849	74.63	200.83	0.81	2.14	1.401616984	Up	4.21E-14	4.67E-13
LXLOC_031743	2811.75	38.79	114.99	0.47	1.24	1.399607459	Up	8.15E-10	6.60E-09
1111	2100.53	165.22	434.6	2.4	6.33	1.399171094	Up	1.09E-27	2.20E-26
3756	8060.96	475	1282	1.81	4.77	1.397999569	Up	1.08E-81	6.27E-80
1848	2815.5	1248	3352	13.75	36.19	1.396159489	Up	6.29E-209	9.87E-207
n385116	1569	149.72	402.43	3.01	7.92	1.395736943	Up	9.27E-27	1.81E-25
100529144	3658	99.53	267.13	0.84	2.21	1.395585137	Up	2.80E-18	3.94E-17
6196	5812.78	3057	8228	16.2	42.6	1.394859617	Up	0	0
MXLOC_024593	2804	48.58	130.92	0.54	1.42	1.394859617	Up	1.11E-09	8.88E-09
55063	2337	20	54	0.27	0.71	1.394859617	Up	9.74E-05	0.000437008
MXLOC_025252	9095	383.26	1028.32	1.29	3.39	1.393914208	Up	2.58E-65	1.20E-63
1308	5610	107	289	0.59	1.55	1.393481356	Up	1.05E-19	1.59E-18
144165	4311.91	745.69	2002	5.34	14.02	1.392574702	Up	1.12E-125	1.08E-123
342184	12355	95.26	258.2	0.24	0.63	1.392317423	Up	6.87E-18	9.47E-17
2324	5349.86	27	74.11	0.16	0.42	1.392317423	Up	3.73E-06	2.07E-05
MXLOC_016624	9966.63	344.06	1188.28	1.36	3.57	1.392317423	Up	9.24E-105	7.09E-103
MXLOC_036611	6151.5	365.53	979.5	1.83	4.79	1.388182007	Up	3.22E-62	1.41E-60
4343	3521	21	55	0.18	0.47	1.38466385	Up	0.000123266	0.000543511
MXLOC_021460	3579.4	79.4	213	0.69	1.8	1.38332864	Up	7.11E-15	8.27E-14
n407929	2424	17.56	47.83	0.23	0.6	1.38332864	Up	0.000217966	0.000918621
n387299	2391	25.57	67.73	0.33	0.86	1.381870635	Up	1.54E-05	7.83E-05
n339738	1372	25.95	69.06	0.6	1.56	1.378511623	Up	7.17E-06	3.83E-05
MXLOC_023261	1651	38.8	102.9	0.74	1.92	1.375509135	Up	8.62E-08	5.77E-07
285	5267.99	46.99	123	0.27	0.7	1.374395515	Up	4.47E-09	3.37E-08
197335	5541	78.58	210.34	0.44	1.14	1.373458396	Up	1.18E-14	1.35E-13
n407569	1648	32.6	85.32	0.62	1.6	1.367731785	Up	1.28E-06	7.55E-06
MXLOC_002660	4768	40.3	106.62	0.26	0.67	1.365649472	Up	6.54E-08	4.43E-07
MXLOC_010915	5282	35.91	94.56	0.21	0.54	1.362570079	Up	2.75E-07	1.75E-06
MXLOC_020522	9436	20.74	57.55	0.07	0.18	1.362570079	Up	3.03E-05	0.000147273
2741	1815.56	21	56	0.37	0.95	1.360402243	Up	8.45E-05	0.000381841
n378169	2381	75.65	198.06	0.99	2.54	1.359328067	Up	1.61E-13	1.72E-12
n341941	524	22.4	58.84	1.45	3.72	1.359249721	Up	7.32E-05	0.000334799
80243	5132	95	248	0.57	1.46	1.356934545	Up	2.69E-16	3.44E-15
n344787	2082	30.04	78.04	0.45	1.15	1.353636955	Up	5.19E-06	2.83E-05
54798	5943.32	546.08	1357.74	2.69	6.87	1.352703926	Up	1.37E-75	7.25E-74
MXLOC_010585	3052	39.61	102.98	0.4	1.02	1.350497247	Up	1.57E-07	1.02E-06
n411614	3411	262.57	682.67	2.38	6.06	1.34835622	Up	6.90E-42	2.03E-40

5592	3819.48	578	1502	4.67	11.89	1.34825426	Up		6.92E-90	4.51E-88
1958	3136	33	87	0.33	0.84	1.347923303	Up		1.11E-06	6.62E-06
10040	2340	41.19	107.07	0.55	1.4	1.347923303	Up		8.22E-08	5.51E-07
n410170	1167	24.92	64.67	0.68	1.73	1.347165386	Up		2.59E-05	0.000127135
166752	6624	232	604	1.08	2.74	1.343144581	Up		2.68E-37	7.11E-36
414919	1932	175.38	453.7	2.84	7.2	1.342105977	Up		3.95E-28	8.12E-27
7762	3658	44.44	115.77	0.38	0.96	1.337034987	Up		2.58E-08	1.82E-07
LXLOC_034054	6764.86	281	727	1.28	3.23	1.335390355	Up		4.13E-44	1.29E-42
6330	4521.52	165.78	426.89	1.13	2.85	1.334639147	Up		1.93E-26	3.73E-25
LXLOC_036250	3102	22.84	59.7	0.23	0.58	1.334419039	Up		5.01E-05	0.00023574
94104	3664.44	174.11	516.17	1.69	4.26	1.333830184	Up		1.10E-38	3.02E-37
MXLOC_020509	8258	261.43	672.47	0.97	2.44	1.330824495	Up		1.37E-40	3.96E-39
23218	8827	111.64	287.64	0.39	0.98	1.329307625	Up		2.34E-18	3.32E-17
MXLOC_033987	4330.86	67.46	172.72	0.48	1.2	1.321928095	Up		1.84E-11	1.71E-10
286	7567.74	59.47	164.85	0.26	0.65	1.321928095	Up		2.84E-12	2.78E-11
LXLOC_014395	1823	28	70.95	0.48	1.2	1.321928095	Up		3.00E-05	0.000146091
4267	1231.97	85	216	2.19	5.46	1.317970081	Up		8.12E-14	8.86E-13
MXLOC_026893	15999	763.92	1945.26	1.46	3.64	1.317970081	Up		3.01E-112	2.49E-110
MXLOC_016431	3220	52.99	135.14	0.51	1.27	1.316259345	Up		1.94E-09	1.51E-08
84517	2005	29	73	0.45	1.12	1.315501826	Up		1.80E-05	9.07E-05
MXLOC_011847	10505.58	1450.26	3557.55	4.08	10.15	1.31483867	Up		1.25E-190	1.79E-188
MXLOC_007377	2747.11	156.88	365.51	1.63	4.05	1.313049944	Up		1.89E-19	2.82E-18
MXLOC_022917	1859	54.88	139.93	0.93	2.31	1.31259023	Up		1.46E-09	1.15E-08
MXLOC_012127	1828	138.3	349.15	2.37	5.87	1.308473444	Up		3.01E-21	4.84E-20
3356	3564	94	239	0.82	2.03	1.307783913	Up		3.80E-15	4.51E-14
9928	7277	40	101	0.17	0.42	1.304854582	Up		4.05E-07	2.52E-06
LXLOC_035876	8122	215.2	540.95	0.81	2	1.304006187	Up		1.18E-31	2.70E-30
10611	3292.45	152.34	322.03	1.2	2.96	1.30256277	Up		2.16E-14	2.45E-13
MXLOC_026689	3777	86.23	218.59	0.71	1.75	1.301463992	Up		7.03E-14	7.71E-13
54437	4707	250	631	1.64	4.04	1.300659478	Up		5.24E-37	1.38E-35
MXLOC_014431	4674	154.68	388.35	1.02	2.51	1.299118212	Up		2.62E-23	4.53E-22
9079	2546.52	130	327	1.59	3.91	1.298141842	Up		7.79E-20	1.18E-18
94122	4711.94	988.47	2482.5	6.47	15.89	1.296281507	Up		3.12E-139	3.25E-137
28227	2426	100	250	1.28	3.14	1.294620749	Up		2.28E-15	2.74E-14
n325081	1542	64.24	160.15	1.31	3.21	1.293006486	Up		2.40E-10	2.04E-09
78996	1572	43.18	108.31	0.87	2.13	1.291766124	Up		1.82E-07	1.18E-06
LXLOC_025430	5770	141.98	356.08	0.76	1.86	1.291231298	Up		1.38E-21	2.26E-20
26301	1949	37.77	94.95	0.61	1.49	1.288431183	Up		9.02E-07	5.41E-06
342865	1243	44	110	1.13	2.76	1.288345494	Up		1.57E-07	1.02E-06
23432	2676	91.92	229.55	1.07	2.61	1.28643901	Up		2.40E-14	2.72E-13
n407268	539	28.07	69.94	1.76	4.29	1.285402219	Up		4.29E-05	0.000203744
n409082	3121	172.53	428.4	1.71	4.16	1.282587203	Up		4.87E-25	8.97E-24
n409659	3155	57.18	141.88	0.56	1.36	1.280107919	Up		3.90E-09	2.96E-08
256987	6015.94	421	1058	2.18	5.29	1.278939587	Up		1.89E-60	8.04E-59
80757	1519	78	193	1.62	3.93	1.278535499	Up		5.31E-12	5.11E-11
11138	4147	136	337	1.01	2.45	1.278426456	Up		6.56E-20	9.97E-19
n338756	365	91.33	225.38	8.88	21.51	1.276375943	Up		9.73E-14	1.06E-12
n376186	1137	22.88	56.47	0.64	1.55	1.276124405	Up		0.000153994	0.000666023
MXLOC_020488	9862	3486.14	8634.88	10.84	26.25	1.275952666	Up		0	0
283464	7446.16	445	1103	1.84	4.45	1.27409957	Up		1.71E-61	7.37E-60
n386306	2249	100.7	248.57	1.4	3.38	1.271596419	Up		4.58E-15	5.39E-14
LXLOC_002858	2715	25	62.14	0.29	0.7	1.271302022	Up		9.74E-05	0.000437092
MXLOC_028619	3740	41	101.09	0.34	0.82	1.270089163	Up		7.09E-07	4.30E-06
n382021	3681	101.62	249.39	0.85	2.05	1.270089163	Up		5.59E-15	6.54E-14
LXLOC_005394	1793	25	62	0.44	1.06	1.268488836	Up		9.74E-05	0.000437036
151313	1265	54.05	132.34	1.36	3.26	1.261265313	Up		1.74E-08	1.25E-07
n376985	7164	2251.55	5515.59	9.65	23.13	1.261164418	Up		9.13E-294	2.18E-291
83943	1519	120	293	2.49	5.96	1.259166588	Up		4.29E-17	5.68E-16
399694	4556	1052.7	2576.85	7.13	17.06	1.258643665	Up		3.67E-138	3.78E-136
7789	5204	124.8	305.98	0.74	1.77	1.258152184	Up		6.01E-18	8.32E-17

3739	4179	187	457	1.38	3.3	1.257797757	Up		7.68E-26	1.45E-24
LXLOC_036561	2792	32	79	0.36	0.86	1.256339753	Up		1.14E-05	5.92E-05
55512	5269	1688	4118	9.87	23.54	1.253992331	Up		3.24E-218	5.32E-216
n377202	617	36	87.34	1.94	4.62	1.251836199	Up		6.17E-06	3.33E-05
11167	3840	606	1476	4.88	11.62	1.251657016	Up		3.16E-79	1.77E-77
130574	4093	355	865	2.68	6.38	1.251323423	Up		3.38E-47	1.14E-45
MXLOC_001834	3224	103.57	252.75	1	2.38	1.250961574	Up		5.87E-15	6.86E-14
85378	6019	325.93	795.11	1.67	3.97	1.249290905	Up		8.23E-44	2.54E-42
53947	2092	41	99	0.61	1.45	1.249171752	Up		1.42E-06	8.34E-06
n386373	1337	29.08	70.59	0.69	1.64	1.249027548	Up		5.23E-05	0.000244349
MXLOC_007087	4423	84.02	205.55	0.59	1.4	1.246639968	Up		2.18E-12	2.16E-11
2173	1005	112	271	3.58	8.49	1.245804966	Up		1.09E-15	1.35E-14
n410507	6909	79.64	191.18	0.35	0.83	1.245756414	Up		1.82E-11	1.69E-10
55509	981	78	189	2.56	6.07	1.245552706	Up		2.11E-11	1.96E-10
8225	1180	289	699	7.81	18.5	1.244130817	Up		5.62E-38	1.52E-36
5638	4124.15	119.69	338.38	1.05	2.48	1.239950793	Up		1.87E-24	3.37E-23
n338763	3092	36	87	0.36	0.85	1.239465935	Up		6.17E-06	3.33E-05
MXLOC_032068	2433	75.54	183.01	0.97	2.29	1.239290946	Up		3.31E-11	3.02E-10
n407950	2743	680.56	1639.36	7.71	18.17	1.236755654	Up		3.75E-86	2.34E-84
MXLOC_000880	7111	216.31	518.36	0.93	2.19	1.235628248	Up		3.77E-28	7.76E-27
4298	4505	90.28	217.84	0.62	1.46	1.235628248	Up		8.87E-13	9.05E-12
MXLOC_031526	2667.65	126.44	303.78	1.47	3.46	1.234955883	Up		3.46E-17	4.61E-16
4986	4959	4264.72	10243.86	26.5	62.27	1.23254492	Up		0	0
3983	6985.77	662.91	1563.44	2.86	6.72	1.232446086	Up		3.27E-79	1.83E-77
53942	4138.66	1791	4293	13.34	31.33	1.231786103	Up		2.83E-220	4.71E-218
MXLOC_031237	3080	65.89	157.56	0.66	1.55	1.231730286	Up		1.17E-09	9.34E-09
MXLOC_006298	10137.88	1923.69	4630.86	5.83	13.69	1.231554658	Up		2.40E-239	4.42E-237
n363604	2257	118.96	284.7	1.64	3.85	1.231162631	Up		3.17E-16	4.03E-15
6048	1137	74.02	177.49	2.08	4.88	1.230297619	Up		1.51E-10	1.31E-09
26353	2002	128	306	2	4.68	1.22650853	Up		3.59E-17	4.78E-16
n375460	2257	65.08	155.41	0.9	2.1	1.222392421	Up		2.32E-09	1.80E-08
n337678	3352	39	93	0.36	0.84	1.222392421	Up		3.87E-06	2.14E-05
56139	4036.13	100.36	243.29	0.78	1.82	1.222392421	Up		2.56E-14	2.89E-13
LXLOC_020489	16710	232.44	559.67	0.43	1	1.217591435	Up		1.69E-30	3.73E-29
7694	2314.29	100.72	164.06	0.93	2.16	1.215728691	Up		0.000166417	0.000716333
6672	3676.11	67.13	148.85	0.53	1.23	1.214594051	Up		6.24E-08	4.24E-07
114788	13126	788.57	1888.18	1.86	4.31	1.212385248	Up		6.78E-98	4.90E-96
2787	806	35	83	1.42	3.29	1.212196654	Up		1.44E-05	7.36E-05
221421	965.64	65	153	2.16	5	1.210896782	Up		4.55E-09	3.43E-08
404734	8349	146.01	347.66	0.54	1.25	1.210896782	Up		4.45E-19	6.55E-18
MXLOC_033798	1645	35.26	82.62	0.67	1.55	1.210035215	Up		2.03E-05	0.000101196
1636	4837.08	51	119	0.32	0.74	1.209453366	Up		3.00E-07	1.90E-06
8447	1073	537	1266	16.04	37.02	1.206630753	Up		2.49E-64	1.13E-62
5338	3471	43.62	103.36	0.39	0.9	1.206450877	Up		1.05E-06	6.23E-06
MXLOC_017046	4063	125.26	294.22	0.95	2.19	1.204931451	Up		4.19E-16	5.29E-15
MXLOC_000580	2135	60.79	143.08	0.89	2.05	1.203746669	Up		9.83E-09	7.22E-08
8738	1189	191	449	5.12	11.79	1.203348003	Up		9.08E-24	1.60E-22
n339810	3161	322.59	757.07	3.16	7.26	1.20004499	Up		5.79E-39	1.61E-37
5137	2913.91	692	1626	7.38	16.95	1.199592552	Up		1.34E-81	7.79E-80
n341449	2069	36	84	0.54	1.24	1.199308808	Up		1.73E-05	8.73E-05
MXLOC_000638	5153.15	2172.29	4931.97	12.58	28.84	1.196939242	Up		8.51E-228	1.49E-225
n410489	716	247.89	579.62	11.38	26.07	1.195890025	Up		4.93E-30	1.08E-28
8543	5405	1142	2668	6.5	14.87	1.193893024	Up		3.01E-131	3.02E-129
374879	2094	33.11	76.63	0.49	1.12	1.192645078	Up		5.49E-05	0.000255566
207063	2577	96.49	225	1.17	2.66	1.184917716	Up		1.36E-12	1.37E-11
79703	2323	65.19	152.61	0.88	2	1.184424571	Up		6.35E-09	4.74E-08
n340024	1981	44	103	0.7	1.59	1.183599938	Up		1.77E-06	1.02E-05
80346	1694	31.87	73.87	0.59	1.34	1.183446141	Up		5.39E-05	0.000251202
n407188	1616	53.26	123.84	1.04	2.36	1.182203331	Up		2.19E-07	1.41E-06
57205	6550	373	863	1.75	3.96	1.178145508	Up		6.77E-43	2.04E-41

n382312	1445	83.81	193.27	1.83	4.14	1.177787119	Up	7.40E-11	6.57E-10
LXLOC_022664	1356	36	83	0.84	1.9	1.177538186	Up	2.42E-05	0.000119619
n410676	4304	31.8	74.39	0.23	0.52	1.176877762	Up	3.82E-05	0.000182671
n338960	2039	35	81	0.54	1.22	1.175849835	Up	2.84E-05	0.000138902
MXLOC_035816	5143.08	96.95	224.14	0.58	1.31	1.175442006	Up	1.90E-12	1.89E-11
n406590	3401	34.01	79.1	0.31	0.7	1.175086707	Up	3.33E-05	0.000161437
1010	4345	427.3	984.74	3.03	6.84	1.174678531	Up	2.64E-48	9.12E-47
1602	5239	568	1309	3.34	7.53	1.172801762	Up	1.10E-63	4.93E-62
MXLOC_004813	4736.31	302.99	703.85	1.99	4.48	1.170730302	Up	1.18E-35	3.02E-34
MXLOC_036029	1695	43.18	99.32	0.8	1.8	1.169925001	Up	4.05E-06	2.23E-05
n406352	6635	95.08	217.87	0.44	0.99	1.169925001	Up	1.14E-11	1.07E-10
118429	5614.5	119	272	0.65	1.46	1.167456746	Up	2.80E-14	3.14E-13
80179	3323.27	88.69	152.83	0.62	1.39	1.164744762	Up	7.18E-05	0.000329328
55220	2500	469	1072	5.84	13.07	1.162218867	Up	1.17E-51	4.28E-50
MXLOC_033192	14528	2705.26	6189.23	5.7	12.75	1.161463423	Up	8.25E-290	1.89E-287
147948	2297	40.61	92.49	0.55	1.23	1.161154792	Up	9.12E-06	4.80E-05
153090	4334.93	183.76	408.23	1.27	2.84	1.161062433	Up	9.90E-20	1.49E-18
MXLOC_018335	5804	1006.56	2302.97	5.34	11.94	1.16089119	Up	4.44E-109	3.58E-107
1021	11612	98	226	0.26	0.58	1.157541277	Up	2.69E-12	2.64E-11
7840	12922	53.49	124.22	0.13	0.29	1.157541277	Up	1.57E-07	1.02E-06
MXLOC_018962	6202	911.73	2078.84	4.52	10.08	1.157100961	Up	4.85E-98	3.51E-96
6558	6860	79.04	179.07	0.35	0.78	1.156119202	Up	9.82E-10	7.89E-09
MXLOC_015187	2747.24	152.99	382.48	1.9	4.23	1.154658245	Up	7.03E-23	1.20E-21
n376604	801	31.61	72	1.29	2.87	1.153679671	Up	7.58E-05	0.000345208
MXLOC_035392	1236	60.69	137.6	1.56	3.47	1.153389634	Up	7.26E-08	4.91E-07
54883	2330	76.86	174.88	1.03	2.29	1.152703261	Up	1.14E-09	9.08E-09
11123	1700	125.9	285.92	2.33	5.18	1.152622143	Up	7.96E-15	9.21E-14
MXLOC_001804	3978	161.64	368.84	1.26	2.8	1.152003093	Up	8.56E-19	1.24E-17
n345954	1811	62.14	141.18	1.08	2.4	1.152003093	Up	5.29E-08	3.61E-07
54941	5888	617.12	1399.54	3.22	7.15	1.150882554	Up	7.75E-66	3.61E-64
8895	4864	1951	4423	12.36	27.42	1.149549828	Up	5.78E-204	8.94E-202
84236	5160	38.44	87.92	0.23	0.51	1.148863386	Up	1.75E-05	8.86E-05
6606	1625	495.22	1125.55	9.63	21.35	1.148628367	Up	1.56E-53	5.98E-52
219749	3719	782.01	1770.15	6.5	14.4	1.147557188	Up	1.59E-82	9.38E-81
MXLOC_016475	2853	85.32	193.56	0.93	2.06	1.147341716	Up	2.00E-10	1.71E-09
n337752	6778	581.96	1314.47	2.64	5.83	1.142957954	Up	1.23E-61	5.32E-60
9369	4772.8	84.05	184.5	0.53	1.17	1.142444265	Up	2.17E-09	1.69E-08
MXLOC_028056	6497	358.81	809.62	1.7	3.75	1.141355849	Up	1.49E-38	4.10E-37
MXLOC_036733	1819	239.39	538.6	4.13	9.1	1.139724764	Up	5.38E-26	1.02E-24
201780	2118	273	614	4.03	8.87	1.138154266	Up	2.17E-29	4.65E-28
25893	5158	377.23	848.13	2.25	4.95	1.137503524	Up	5.72E-40	1.62E-38
MXLOC_017605	2723	40.39	90.63	0.46	1.01	1.134649527	Up	1.76E-05	8.91E-05
MXLOC_000475	3451	68.68	154.5	0.62	1.36	1.133266531	Up	1.48E-08	1.07E-07
MXLOC_014548	3529	231.82	518.81	2.03	4.45	1.132325609	Up	6.63E-25	1.21E-23
MXLOC_004143	6938	190.06	424.32	0.84	1.84	1.131244533	Up	1.76E-20	2.73E-19
4214	7512	1162	2602	4.75	10.4	1.13058411	Up	7.27E-118	6.37E-116
2675	3314.53	760	1700	7.1	15.54	1.130095574	Up	1.71E-77	9.30E-76
MXLOC_014325	2205	60.93	135.84	0.86	1.88	1.128324097	Up	1.39E-07	9.12E-07
n345856	5222	91.55	204.34	0.54	1.18	1.127755547	Up	1.07E-10	9.36E-10
MXLOC_004945	4435.44	335.96	748.39	2.33	5.09	1.127335701	Up	6.00E-35	1.50E-33
n335717	358	38.27	84.89	3.8	8.29	1.125372683	Up	4.69E-05	0.000221253
n407441	2862	45.79	102.52	0.5	1.09	1.124328135	Up	4.09E-06	2.25E-05
n409184	4046	155.09	344.45	1.18	2.57	1.1229815	Up	9.93E-17	1.29E-15
LXLOC_030798	4351	189.98	424.15	1.35	2.94	1.122856748	Up	1.10E-20	1.72E-19
MXLOC_005196	3718	53.66	120.11	0.45	0.98	1.122856748	Up	5.89E-07	3.61E-06
MXLOC_007836	10282	286.39	633.66	0.85	1.85	1.121990524	Up	2.56E-29	5.48E-28
MXLOC_002480	2091	61.13	135.01	0.91	1.98	1.12156198	Up	2.25E-07	1.44E-06
1030	3861	79	175	0.63	1.37	1.120752159	Up	3.53E-09	2.69E-08
7681	2714	75.2	166.69	0.86	1.87	1.120629705	Up	9.18E-09	6.76E-08
286257	812	69.95	155.58	2.81	6.11	1.12060225	Up	1.74E-08	1.25E-07

LXLOC_011392	667	56.09	123.86	2.78	6.02	1.114678604	Up	9.53E-07	5.70E-06
n379810	2223	86.15	190.66	1.21	2.62	1.114559764	Up	8.41E-10	6.80E-09
MXLOC_036702	3501	144.48	319.99	1.28	2.77	1.113742166	Up	1.44E-15	1.76E-14
MXLOC_016625	5262	93.78	207.52	0.55	1.19	1.11345805	Up	1.07E-10	9.36E-10
MXLOC_009665	8821	90.07	197.61	0.31	0.67	1.11189288	Up	6.07E-10	4.98E-09
LXLOC_017611	2753	138.56	305.22	1.56	3.37	1.111202562	Up	6.95E-15	8.09E-14
23007	6169.76	50.68	110.04	0.25	0.54	1.111031312	Up	3.47E-06	1.93E-05
30008	2068	46	102	0.7	1.51	1.109121722	Up	6.65E-06	3.57E-05
MXLOC_006854	5224.98	87.11	190.09	0.51	1.1	1.108934372	Up	1.34E-09	1.06E-08
MXLOC_001518	11105	716	1581.21	1.98	4.27	1.10873564	Up	2.48E-70	1.23E-68
774	9790	469.96	1035.68	1.47	3.17	1.108666685	Up	1.34E-46	4.44E-45
n345453	7742	113.01	249.55	0.45	0.97	1.108059746	Up	2.38E-12	2.35E-11
2845	2966	62	137	0.65	1.4	1.106915204	Up	1.92E-07	1.23E-06
n407957	3814	73.22	160.6	0.59	1.27	1.106041637	Up	2.38E-08	1.68E-07
7581	6119.8	1915.13	4211.86	9.62	20.7	1.105521969	Up	1.44E-182	1.96E-180
MXLOC_025759	7355	79.02	174.52	0.33	0.71	1.105353	Up	4.84E-09	3.65E-08
n377400	780	56.14	122.57	2.35	5.03	1.097897643	Up	1.31E-06	7.68E-06
54753	3578	61.06	134.11	0.53	1.13	1.092258508	Up	3.09E-07	1.95E-06
54981	1172.01	77.76	172.43	2.16	4.6	1.090602549	Up	3.53E-09	2.69E-08
n386099	1559	42.75	92.52	0.86	1.83	1.089435084	Up	2.43E-05	0.000120024
3291	1884	33	72	0.55	1.17	1.089005006	Up	0.000204194	0.000865642
MXLOC_023693	5380	221.62	482.85	1.27	2.7	1.08813091	Up	4.65E-22	7.73E-21
154810	8999	1424.09	3093.25	4.85	10.31	1.08798768	Up	3.46E-131	3.46E-129
3489	966	48	104	1.6	3.4	1.087462841	Up	9.06E-06	4.77E-05
MXLOC_035253	7166.43	113.52	243.97	0.48	1.02	1.087462841	Up	1.52E-11	1.43E-10
MXLOC_011635	11934	3680.25	7988.21	9.44	20.05	1.086743472	Up	0	0
MXLOC_037605	13485	935.03	2029.12	2.12	4.5	1.085860737	Up	1.51E-86	9.45E-85
225	5341	242	527	1.4	2.97	1.085036104	Up	6.72E-24	1.19E-22
8707	3548	1816	3935	15.84	33.58	1.084029895	Up	1.64E-165	2.07E-163
LXLOC_022759	4857.48	771.78	1680.76	4.92	10.43	1.084008937	Up	1.33E-72	6.79E-71
LXLOC_011968	4691.68	514.62	1161.86	3.53	7.47	1.08144006	Up	1.57E-54	6.09E-53
n339968	1683	36.94	79.97	0.69	1.46	1.081300102	Up	9.00E-05	0.00040545
LXLOC_024484	2849.35	531.96	946.52	4.78	10.09	1.077843651	Up	1.76E-25	3.30E-24
5493	6238	277	599	1.37	2.89	1.0768936	Up	1.45E-26	2.81E-25
n337723	1036	65.04	140.4	2.02	4.26	1.076498137	Up	3.02E-07	1.91E-06
200150	3169.73	96	203	0.92	1.94	1.076350886	Up	1.45E-09	1.15E-08
LXLOC_028924	6411	96	207	0.46	0.97	1.076350886	Up	4.31E-10	3.58E-09
56934	3119.77	3315	7157	33.02	69.6	1.075747186	Up	5.31E-297	1.29E-294
7386	1224	1295	2786	33.69	70.96	1.074685581	Up	5.80E-116	4.94E-114
4137	5548.95	13156.69	28286.49	72.91	153.48	1.073862063	Up	0	0
n408889	2386	51.35	110.27	0.67	1.41	1.073462162	Up	5.53E-06	3.00E-05
2911	6903.3	65	139	0.29	0.61	1.072756342	Up	4.11E-07	2.55E-06
LXLOC_000242	2174.25	150.02	294.73	1.97	4.14	1.071435138	Up	2.95E-11	2.70E-10
1387	10176.81	466.88	1005.43	1.41	2.96	1.069902013	Up	3.30E-43	1.01E-41
n378919	1080	46.01	98.09	1.36	2.85	1.067355268	Up	2.35E-05	0.000116479
728661	6003	1616.23	3459.54	8.28	17.34	1.066401226	Up	6.20E-142	6.57E-140
127833	7604	323	688.17	1.3	2.72	1.065095028	Up	2.44E-29	5.24E-28
81789	3493	123.25	263.27	1.09	2.28	1.064705689	Up	3.06E-12	2.99E-11
132158	3335.45	81.59	152.45	0.66	1.38	1.064130337	Up	6.68E-06	3.58E-05
1767	15573	58	122	0.11	0.23	1.064130337	Up	3.24E-06	1.81E-05
MXLOC_000224	6501	144.98	311.37	0.69	1.44	1.061400545	Up	1.71E-14	1.95E-13
286075	2114	40	85	0.59	1.23	1.059871456	Up	8.70E-05	0.000392587
MXLOC_029753	2177	41.39	87.68	0.59	1.23	1.059871456	Up	7.36E-05	0.000336574
64282	8097.6	157.55	336.13	0.6	1.25	1.058893689	Up	2.91E-15	3.48E-14
MXLOC_016151	7051	164.63	351.6	0.72	1.5	1.058893689	Up	7.04E-16	8.76E-15
81576	1619	132.02	280.96	2.57	5.35	1.057770532	Up	9.75E-13	9.90E-12
10235	2254.44	277.43	590.03	3.84	7.99	1.057089192	Up	2.18E-25	4.07E-24
1847	2528	83	176	1.02	2.12	1.055495113	Up	1.62E-08	1.16E-07
8322	7394	248	525.67	1.03	2.14	1.054966459	Up	1.78E-22	3.00E-21
n378268	566	138.17	292.71	8.19	17.01	1.054447784	Up	3.72E-13	3.90E-12

30812	3049	78.44	166.8	0.8	1.66	1.053111336	Up	3.65E-08	2.53E-07
57559	1997	581	1228	9.11	18.84	1.048276006	Up	3.40E-50	1.21E-48
3735	2079.14	1358.7	2723.52	19.4	40.09	1.047185765	Up	1.48E-96	1.04E-94
LXLOC_001068	3849.68	79.64	296.45	1.13	2.33	1.044007182	Up	9.33E-30	2.02E-28
MXLOC_035858	5721	487.16	1026.4	2.62	5.4	1.043392596	Up	6.28E-42	1.85E-40
LXLOC_002653	19021	1036.33	2191.39	1.67	3.44	1.042560462	Up	2.93E-88	1.86E-86
22924	1870	60.59	128.23	1.02	2.1	1.041820176	Up	1.27E-06	7.46E-06
LXLOC_028248	7040	130	274.62	0.57	1.17	1.037474705	Up	2.45E-12	2.41E-11
4645	9520	194	402	0.62	1.27	1.034488376	Up	8.33E-17	1.09E-15
23359	4707	1057.45	2213.65	6.93	14.18	1.032930275	Up	3.75E-87	2.36E-85
151354	6355	607	1269	2.94	6	1.029146346	Up	1.39E-50	4.98E-49
n411756	2271	249.49	520.38	3.43	7	1.029146346	Up	1.20E-21	1.96E-20
MXLOC_011611	5237	131.12	272.82	0.77	1.57	1.027834208	Up	6.81E-12	6.51E-11
MXLOC_010768	4527.5	157.48	318.02	1.04	2.12	1.027480736	Up	5.78E-13	5.99E-12
MXLOC_007578	5748.69	539.86	1129.63	2.9	5.91	1.02710523	Up	2.19E-45	7.05E-44
26031	6637.9	59	122	0.27	0.55	1.026472211	Up	5.01E-06	2.73E-05
n345482	2837	100.72	209.44	1.1	2.24	1.025995209	Up	1.38E-09	1.09E-08
n377781	1955	227.25	472.77	3.64	7.41	1.025535092	Up	1.31E-19	1.96E-18
84328	1407	228.83	475.81	5.15	10.48	1.024994379	Up	8.30E-20	1.26E-18
LXLOC_028057	5765.61	495.35	958.44	2.46	5	1.023269779	Up	3.16E-32	7.32E-31
54453	4244	128.92	268.82	0.94	1.91	1.022839976	Up	6.13E-12	5.87E-11
23371	4907.17	51	105	0.32	0.65	1.022367813	Up	2.55E-05	0.000125499
2172	663.45	444	919	22.1	44.89	1.022347727	Up	2.70E-36	7.00E-35
n373790	4146	91.82	189.53	0.68	1.38	1.021061616	Up	1.08E-08	7.91E-08
79627	1740	251	520	4.53	9.19	1.020553811	Up	2.80E-21	4.51E-20
n407407	4513	105.62	218.75	0.72	1.46	1.019899557	Up	8.26E-10	6.68E-09
7706	5744	280	580	1.5	3.04	1.019108823	Up	1.51E-23	2.64E-22
56121	2656	362.26	749.53	4.24	8.59	1.018593867	Up	7.30E-30	1.59E-28
2195	14764	2316.73	4794.42	4.8	9.72	1.017921908	Up	9.93E-182	1.35E-179
374654	4558	126.13	259.7	0.85	1.72	1.016873819	Up	3.68E-11	3.33E-10
n406148	946	116.76	241.08	3.98	8.05	1.016220352	Up	1.03E-10	9.06E-10
22844	4916	71	149	0.45	0.91	1.015941544	Up	2.83E-07	1.80E-06
n410533	1837	512.03	1057.93	8.75	17.69	1.015579126	Up	3.00E-41	8.73E-40
n341955	2491	124.72	257.15	1.56	3.15	1.0138058	Up	2.83E-11	2.59E-10
MXLOC_037803	11051.28	2361.76	4527.21	6.08	12.27	1.01299202	Up	3.95E-142	4.20E-140
23464	1516.39	246.55	512.03	5.18	10.44	1.011097709	Up	3.48E-21	5.58E-20
MXLOC_006612	1339	56.87	117.29	1.35	2.72	1.010647244	Up	6.10E-06	3.29E-05
9189	4548.78	904.52	1860.94	6.14	12.34	1.007031834	Up	1.06E-70	5.29E-69
MXLOC_009588	5472	296.95	608.5	1.67	3.35	1.004312993	Up	3.72E-24	6.63E-23
153572	2980.6	742	1539	7.82	15.68	1.003685047	Up	6.79E-60	2.86E-58
6567	4372	1222	2503	8.62	17.28	1.003343443	Up	1.62E-93	1.10E-91
n375748	970	83.33	170.53	2.77	5.55	1.002601795	Up	9.66E-08	6.42E-07
93663	3499	1216	2490	10.76	21.55	1.002009791	Up	5.59E-93	3.78E-91
23237	2943	56	114	0.59	1.18	1	Up	1.49E-05	7.61E-05
MXLOC_030771	6470	107.47	220.3	0.51	1.02	1	Up	1.07E-09	8.59E-09
LXLOC_028415	958	47	96	1.58	3.16	1	Up	6.70E-05	0.000308734
8671	7686.12	106	215	0.42	0.84	1	Up	3.01E-09	2.31E-08
50940	9278	81	166.83	0.27	0.54	1	Up	1.34E-07	8.80E-07
LXLOC_034753	201	343.73	0	69.48	0.01	-12.76238204	Down	1.09E-105	8.38E-104
MXLOC_016157	2049	1378.9	0	21.05	0.01	-11.03960452	Down	0	0
LXLOC_031451	651	399.73	0	20.34	0.01	-10.99010396	Down	7.94E-123	7.40E-121
4111	1675.56	751	0	14.1	0.01	-10.46147945	Down	1.54E-230	2.72E-228
n408906	1830	596.86	0	10.24	0.01	-10	Down	4.14E-183	5.67E-181
LXLOC_031383	820	216.7	0	8.6	0.01	-9.74819285	Down	7.92E-67	3.74E-65
6873	5020	1214.59	0	7.45	0.01	-9.541096615	Down	0	0
MXLOC_032044	4070	693.11	0	5.26	0.01	-9.038918989	Down	8.61E-213	1.38E-210
n387356	296	39.86	0	4.96	0.01	-8.95419631	Down	1.15E-12	1.16E-11
LXLOC_037100	629.43	78.14	0	4.12	0.01	-8.686500527	Down	1.34E-24	2.42E-23
158511	659	80.8	0	4.06	0.01	-8.665335917	Down	3.27E-25	6.07E-24
MXLOC_032948	6098	784.25	0	3.96	0.01	-8.62935662	Down	1.23E-240	2.28E-238

n376385	1272	156.34	0	3.91	0.01	-8.611024797	Down	1.81E-48	6.27E-47
LXLOC_031385	911	104.64	0	3.71	0.01	-8.535275377	Down	1.48E-32	3.48E-31
n340847	1818	209.25	0	3.61	0.01	-8.495855027	Down	1.10E-64	5.01E-63
MXLOC_031386	3539	378.68	0	3.31	0.01	-8.370687407	Down	2.12E-116	1.82E-114
n378731	621	59.42	0	3.18	0.01	-8.312882955	Down	8.73E-19	1.26E-17
n385168	503	308	1	20.8	0.07	-8.215012891	Down	8.77E-93	5.90E-91
n381594	839	75.31	0	2.92	0.01	-8.189824559	Down	1.11E-23	1.94E-22
n332617	324	23.9	0	2.67	0.01	-8.060695932	Down	9.06E-08	6.05E-07
n377667	504	39.47	0	2.66	0.01	-8.055282436	Down	1.15E-12	1.16E-11
MXLOC_008449	7282.08	586.06	0	2.47	0.01	-7.948367232	Down	4.76E-180	6.39E-178
n379732	528	37	0	2.37	0.01	-7.888743249	Down	4.71E-12	4.54E-11
441531	1678	125.61	0	2.36	0.01	-7.882643049	Down	5.56E-39	1.54E-37
8354	477	32	0	2.29	0.01	-7.839203788	Down	1.60E-10	1.38E-09
LXLOC_013376	497	33	0	2.26	0.01	-7.820178962	Down	7.89E-11	6.98E-10
55760	3070	222.52	0	2.25	0.01	-7.813781191	Down	1.15E-68	5.59E-67
n373235	335	19.92	0	2.14	0.01	-7.741466986	Down	1.52E-06	8.84E-06
n335630	662	42.6	0	2.13	0.01	-7.73470962	Down	1.39E-13	1.50E-12
n386307	2359	160.77	0	2.12	0.01	-7.727920455	Down	1.08E-49	3.82E-48
2953	1119	73.67	0	2.11	0.01	-7.721099189	Down	4.54E-23	7.79E-22
MXLOC_028550	7795	529.68	0	2.09	0.01	-7.707359132	Down	1.32E-162	1.63E-160
n346542	328	18.21	0	2.01	0.01	-7.651051691	Down	3.07E-06	1.72E-05
n407038	1496	95.14	0	2.01	0.01	-7.651051691	Down	8.41E-30	1.83E-28
n384644	2841	183.98	0	2.01	0.01	-7.651051691	Down	9.92E-57	3.99E-55
n408109	3550	227.83	0	1.99	0.01	-7.636624621	Down	3.41E-70	1.68E-68
LXLOC_017539	16600.77	1036.19	7.87	1.95	0.01	-7.607330314	Down	2.12E-302	5.31E-300
n377180	304	15.66	0	1.89	0.01	-7.562242424	Down	2.54E-05	0.000125114
55885	3460.52	7087.72	37.61	61.73	0.33	-7.547361957	Down	0	0
n410136	2164	125.51	0	1.81	0.01	-7.499845887	Down	5.56E-39	1.54E-37
MXLOC_028414	2107	120.76	0	1.79	0.01	-7.483815777	Down	1.88E-37	5.00E-36
LXLOC_031455	309	14.3	0	1.69	0.01	-7.400879436	Down	5.14E-05	0.000240816
63934	3860	206.62	0	1.65	0.01	-7.366322214	Down	9.09E-64	4.07E-62
9764	7361	395.14	0	1.65	0.01	-7.366322214	Down	1.33E-121	1.23E-119
n406475	706	35.3	0	1.65	0.01	-7.366322214	Down	1.93E-11	1.79E-10
n370183	464	22	0	1.63	0.01	-7.348728154	Down	1.83E-07	1.18E-06
n385673	1685	87.24	0	1.63	0.01	-7.348728154	Down	2.36E-27	4.71E-26
n380553	1765	90.33	0	1.61	0.01	-7.330916878	Down	2.85E-28	5.89E-27
4826	1266	5090	33	130.3	0.81	-7.329699461	Down	0	0
n385241	650	30.89	0	1.57	0.01	-7.294620749	Down	6.53E-10	5.33E-09
n376378	2483	372.74	2.44	4.68	0.03	-7.285402219	Down	2.62E-110	2.14E-108
LXLOC_000422	634	29.65	0	1.55	0.01	-7.276124405	Down	1.32E-09	1.05E-08
n339111	3011	139.13	0	1.43	0.01	-7.159871337	Down	2.89E-43	8.85E-42
n376605	582	24.39	0	1.4	0.01	-7.129283017	Down	4.48E-08	3.08E-07
100130086	1966	87	0	1.39	0.01	-7.118941073	Down	2.36E-27	4.71E-26
n386246	494	20.11	0	1.39	0.01	-7.118941073	Down	7.50E-07	4.53E-06
n373032	566	23	0	1.36	0.01	-7.087462841	Down	9.06E-08	6.04E-07
22993	5406	1180.61	9.59	6.72	0.05	-7.070389328	Down	0	0
MXLOC_023099	5253.52	224.13	0	1.31	0.01	-7.033423002	Down	2.82E-69	1.38E-67
n380672	704	28	0	1.31	0.01	-7.033423002	Down	2.67E-09	2.06E-08
n407956	4044	172.05	0	1.31	0.01	-7.033423002	Down	2.30E-53	8.81E-52
n338422	765	30.35	0	1.3	0.01	-7.022367813	Down	6.53E-10	5.34E-09
51334	1764	71.7	0	1.28	0.01	-7	Down	1.86E-22	3.13E-21
55184	3500	145.05	0	1.28	0.01	-7	Down	4.21E-45	1.34E-43
11272	564	21.48	0	1.28	0.01	-7	Down	3.71E-07	2.31E-06
n346398	2178	86.4	0	1.24	0.01	-6.95419631	Down	4.77E-27	9.41E-26
n406454	2516	98	0	1.21	0.01	-6.918863237	Down	1.02E-30	2.27E-29
n376380	1416	54.33	0	1.21	0.01	-6.918863237	Down	2.96E-17	3.95E-16
n340286	2454	93.06	0	1.18	0.01	-6.882643049	Down	3.44E-29	7.33E-28
n381248	476	16.37	0	1.18	0.01	-6.882643049	Down	1.26E-05	6.49E-05
n335608	532	18.56	0	1.18	0.01	-6.882643049	Down	3.07E-06	1.72E-05
n378078	439	14.88	0	1.17	0.01	-6.87036472	Down	5.14E-05	0.000240752

n376618	480	16.38	0	1.17	0.01	-6.87036472	Down	1.26E-05	6.48E-05
n366606	441	14.86	0	1.16	0.01	-6.857980995	Down	5.14E-05	0.00024104
n378652	737	51.05	0.52	2.27	0.02	-6.826548487	Down	2.45E-16	3.13E-15
n376384	813	28.27	0	1.13	0.01	-6.820178962	Down	2.67E-09	2.06E-08
n365843	1066	36.9	0	1.11	0.01	-6.794415866	Down	9.53E-12	9.02E-11
n365633	1157	40.03	0	1.1	0.01	-6.781359714	Down	5.69E-13	5.90E-12
MXLOC_016550	1358	47.09	0	1.1	0.01	-6.781359714	Down	4.10E-15	4.85E-14
MXLOC_022915	7133	253.5	0	1.09	0.01	-6.768184325	Down	3.77E-78	2.07E-76
n335618	487	14.93	0	1.05	0.01	-6.714245518	Down	5.14E-05	0.000240912
n384865	592	18.37	0	1.04	0.01	-6.700439718	Down	3.07E-06	1.73E-05
57713	7922	267.81	0	1.04	0.01	-6.700439718	Down	1.96E-82	1.15E-80
n373317	449	13.5	0	1.04	0.01	-6.700439718	Down	0.00010399	0.000463734
LXLOC_031406	618	1167.01	11.56	62.83	0.61	-6.686500527	Down	0	0
MXLOC_031381	487	14.71	0	1.03	0.01	-6.686500527	Down	5.14E-05	0.00024072
MXLOC_031398	1152	36.94	0	1.02	0.01	-6.672425342	Down	9.53E-12	9.03E-11
MXLOC_005007	2905	94.61	0	1.01	0.01	-6.658211483	Down	1.70E-29	3.66E-28
n339176	2099	1084.53	10.7	16.15	0.16	-6.65731845	Down	0	0
n381061	1160	36.17	0	1	0.01	-6.64385619	Down	9.53E-12	9.02E-11
n346139	632	18.85	0	0.99	0.01	-6.62935662	Down	3.07E-06	1.72E-05
n367780	576	16.67	0	0.97	0.01	-6.599912842	Down	1.26E-05	6.48E-05
n410437	1950	59.59	0	0.96	0.01	-6.584962501	Down	8.73E-19	1.26E-17
n409085	1250	37.43	0	0.95	0.01	-6.569855608	Down	4.71E-12	4.55E-11
3358	4751	147	0	0.95	0.01	-6.569855608	Down	1.03E-45	3.33E-44
MXLOC_014004	6083	187.11	0	0.95	0.01	-6.569855608	Down	5.92E-58	2.43E-56
n332847	540	15.28	0	0.95	0.01	-6.569855608	Down	2.54E-05	0.000125183
85445	4866	567	6	3.71	0.04	-6.535275377	Down	2.54E-162	3.12E-160
n409331	1678	48.79	0	0.91	0.01	-6.50779464	Down	2.03E-15	2.45E-14
n378714	1329	38	0	0.91	0.01	-6.50779464	Down	2.33E-12	2.30E-11
MXLOC_003928	3259	95.71	0	0.91	0.01	-6.50779464	Down	8.41E-30	1.83E-28
n334875	570	15.28	0	0.9	0.01	-6.491853096	Down	2.54E-05	0.000125079
MXLOC_028719	2375	66.89	0	0.88	0.01	-6.459431619	Down	6.29E-21	9.95E-20
n377656	2260	63.29	0	0.87	0.01	-6.442943496	Down	5.21E-20	7.95E-19
n382375	615	15.73	0	0.85	0.01	-6.409390936	Down	2.54E-05	0.000125253
MXLOC_021340	12190	333.18	0	0.84	0.01	-6.392317423	Down	1.25E-102	9.45E-101
n376189	2050	55.12	1	0.84	0.01	-6.392317423	Down	4.29E-16	5.39E-15
MXLOC_000169	2715	73.09	0	0.84	0.01	-6.392317423	Down	4.54E-23	7.78E-22
n339352	2100	56.62	0	0.84	0.01	-6.392317423	Down	7.23E-18	9.96E-17
n375785	808	20.66	0	0.83	0.01	-6.375039431	Down	7.50E-07	4.54E-06
MXLOC_004901	3074	1201	15	12.12	0.15	-6.336283388	Down	0	0
n376493	4357	791.44	9.41	5.61	0.07	-6.324502039	Down	6.92E-225	1.19E-222
n409157	2084	53	0	0.8	0.01	-6.321928095	Down	5.98E-17	7.88E-16
LXLOC_017108	1077	26.86	0	0.8	0.01	-6.321928095	Down	1.09E-08	7.99E-08
n375477	4056	104.23	0	0.79	0.01	-6.303780748	Down	1.48E-32	3.47E-31
283927	1093	26.86	0	0.79	0.01	-6.303780748	Down	1.09E-08	7.99E-08
n342355	2479	62.73	0	0.79	0.01	-6.303780748	Down	1.05E-19	1.59E-18
n410083	1427	35.35	0	0.78	0.01	-6.285402219	Down	1.93E-11	1.79E-10
n376382	1431	34.23	0	0.76	0.01	-6.247927513	Down	3.90E-11	3.53E-10
n367668	2757	67.56	0	0.76	0.01	-6.247927513	Down	3.11E-21	5.00E-20
n379895	590	13.47	0	0.76	0.01	-6.247927513	Down	0.00010399	0.000463617
MXLOC_034248	2320	55.67	0	0.75	0.01	-6.22881869	Down	1.46E-17	1.99E-16
50651	2401	57.7	0	0.75	0.01	-6.22881869	Down	3.57E-18	4.99E-17
n384519	748	16.98	0	0.74	0.01	-6.209453366	Down	1.26E-05	6.49E-05
100316904	837	18.81	0	0.73	0.01	-6.189824559	Down	3.07E-06	1.72E-05
n381244	559	12.13	0	0.73	0.01	-6.189824559	Down	0.000210376	0.000889287
n376381	2792	65.7	0	0.73	0.01	-6.189824559	Down	1.27E-20	1.99E-19
n378627	1628	37	0	0.72	0.01	-6.169925001	Down	4.71E-12	4.55E-11
n377913	1315	29.77	0	0.72	0.01	-6.169925001	Down	1.32E-09	1.05E-08
n335548	740	15.82	0	0.7	0.01	-6.129283017	Down	2.54E-05	0.000125061
n345222	4088	91.27	0	0.69	0.01	-6.108524457	Down	1.41E-28	2.95E-27
n375738	883	18.62	0	0.68	0.01	-6.087462841	Down	3.07E-06	1.72E-05

n410474	1687	35.84	0	0.67	0.01	-6.06608919	Down	1.93E-11	1.79E-10
n408888	1071	22.48	0	0.67	0.01	-6.06608919	Down	1.83E-07	1.18E-06
284459	2929	61.92	0	0.66	0.01	-6.044394119	Down	2.13E-19	3.18E-18
n411738	635	12.03	0	0.63	0.01	-5.977279923	Down	0.000210376	0.000890246
9464	2368	48	1	0.63	0.01	-5.977279923	Down	5.23E-14	5.77E-13
n377476	3129	63.09	0	0.63	0.01	-5.977279923	Down	5.21E-20	7.94E-19
n407294	758	14.36	0	0.62	0.01	-5.95419631	Down	5.14E-05	0.000240656
n338076	1435	2391.15	39.81	52.72	0.86	-5.9378699	Down	0	0
n381052	951	17.95	0	0.61	0.01	-5.930737338	Down	6.21E-06	3.34E-05
n409144	1644	31.41	0	0.6	0.01	-5.906890596	Down	3.23E-10	2.72E-09
n379029	2014	38.37	0	0.6	0.01	-5.906890596	Down	2.33E-12	2.30E-11
n383764	1863	35.61	0	0.6	0.01	-5.906890596	Down	1.93E-11	1.79E-10
n346311	1352	25.2	0	0.59	0.01	-5.882643049	Down	2.21E-08	1.57E-07
389903	787.78	14.22	0	0.59	0.01	-5.882643049	Down	5.14E-05	0.000240561
n383560	1523	451.38	8	9.36	0.16	-5.87036472	Down	1.98E-124	1.89E-122
n339978	2437	2551.65	44.91	32.62	0.56	-5.864186145	Down	0	0
MXLOC_028495	4303	80.79	0	0.58	0.01	-5.857980995	Down	3.27E-25	6.07E-24
n378806	3519	65.94	0	0.58	0.01	-5.857980995	Down	1.27E-20	1.99E-19
n411728	3767	1620.22	28.39	13.3	0.23	-5.853648574	Down	0	0
n378514	756	13.18	0	0.57	0.01	-5.832890014	Down	0.00010399	0.00046432
n378472	808	14.06	0	0.57	0.01	-5.832890014	Down	5.14E-05	0.000240976
n325303	900	15.54	0	0.56	0.01	-5.807354922	Down	2.54E-05	0.000125288
LXLOC_017788	1849	32.89	0	0.56	0.01	-5.807354922	Down	1.60E-10	1.38E-09
n379095	884	14.9	0	0.55	0.01	-5.781359714	Down	5.14E-05	0.000241104
2990	2300	40.5	0	0.55	0.01	-5.781359714	Down	5.69E-13	5.90E-12
n339381	1531	26.82	0	0.55	0.01	-5.781359714	Down	1.09E-08	7.99E-08
4101	1979	34	0	0.54	0.01	-5.754887502	Down	3.90E-11	3.52E-10
n384728	1102	18.42	0	0.53	0.01	-5.727920455	Down	3.07E-06	1.72E-05
n381508	862	14.21	0	0.53	0.01	-5.727920455	Down	5.14E-05	0.000240688
3481	4839	83	0	0.53	0.01	-5.727920455	Down	3.95E-26	7.55E-25
n341208	1605	26.99	0	0.53	0.01	-5.727920455	Down	1.09E-08	7.99E-08
n383901	805	12.73	0	0.52	0.01	-5.700439718	Down	0.000210376	0.00088982
57186	9426	160.88	0	0.52	0.01	-5.700439718	Down	1.08E-49	3.82E-48
n405444	2036	33.55	0	0.52	0.01	-5.700439718	Down	7.89E-11	6.98E-10
n410049	1128	18.24	0	0.52	0.01	-5.700439718	Down	3.07E-06	1.72E-05
6939	1227	20	0	0.52	0.01	-5.700439718	Down	7.50E-07	4.53E-06
100137049	2735	44.68	0	0.51	0.01	-5.672425342	Down	3.40E-14	3.80E-13
n340430	1410	22.75	0	0.51	0.01	-5.672425342	Down	1.83E-07	1.18E-06
256302	4786	312.27	6.42	2.01	0.04	-5.651051691	Down	7.92E-86	4.90E-84
n369997	1608	25.56	0	0.5	0.01	-5.64385619	Down	2.21E-08	1.57E-07
n376398	3102	50.44	0	0.5	0.01	-5.64385619	Down	4.95E-16	6.21E-15
259289	1027	16.01	0	0.5	0.01	-5.64385619	Down	1.26E-05	6.48E-05
n340702	893	13.55	0	0.49	0.01	-5.614709844	Down	0.00010399	0.000464203
n406921	3448	54.92	0	0.49	0.01	-5.614709844	Down	2.96E-17	3.96E-16
n407091	2167	34.08	0	0.49	0.01	-5.614709844	Down	3.90E-11	3.52E-10
n364385	1627	25.18	0	0.49	0.01	-5.614709844	Down	2.21E-08	1.57E-07
78989	1304	20	0	0.49	0.01	-5.614709844	Down	7.50E-07	4.53E-06
8776	2732	41.99	0	0.48	0.01	-5.584962501	Down	2.81E-13	2.98E-12
1373	5725	89.11	0	0.48	0.01	-5.584962501	Down	5.76E-28	1.18E-26
144347	2150	33.31	0	0.48	0.01	-5.584962501	Down	7.89E-11	6.98E-10
n407908	6925	108.95	0	0.48	0.01	-5.584962501	Down	8.85E-34	2.15E-32
n363906	1847	27.74	0	0.47	0.01	-5.554588852	Down	5.41E-09	4.06E-08
LXLOC_018030	433	47	1	3.76	0.08	-5.554588852	Down	1.04E-13	1.12E-12
n384395	1325	19.19	0	0.46	0.01	-5.523561956	Down	1.52E-06	8.84E-06
728378	4337	449	10.17	3.19	0.07	-5.510057692	Down	4.71E-121	4.32E-119
n376619	359	45.62	1	4.52	0.1	-5.498250868	Down	4.07E-13	4.25E-12
n376457	2730	39.66	0	0.45	0.01	-5.491853096	Down	1.15E-12	1.16E-11
n376379	1480	21.06	0	0.45	0.01	-5.491853096	Down	3.71E-07	2.31E-06
n344852	2837	41.39	0	0.45	0.01	-5.491853096	Down	2.81E-13	2.97E-12
MXLOC_013575	5210	76.88	0	0.45	0.01	-5.491853096	Down	5.48E-24	9.72E-23

n407156	3081	44.76	0	0.45	0.01	-5.491853096	Down	3.40E-14	3.80E-13
n410887	900	12.62	0	0.45	0.01	-5.491853096	Down	0.000210376	0.000888755
n378673	741	29.95	0.77	1.33	0.03	-5.470319935	Down	1.32E-09	1.05E-08
n369738	2509	35.82	0	0.44	0.01	-5.459431619	Down	1.93E-11	1.79E-10
MXLOC_008078	7269	104.52	0	0.44	0.01	-5.459431619	Down	1.48E-32	3.47E-31
9788	5010	70.8	0	0.44	0.01	-5.459431619	Down	3.76E-22	6.29E-21
n376348	1895	26.32	0	0.44	0.01	-5.459431619	Down	1.09E-08	7.99E-08
552891	1499	20.67	0	0.44	0.01	-5.459431619	Down	7.50E-07	4.53E-06
MXLOC_022421	3384	48.61	0	0.44	0.01	-5.459431619	Down	2.03E-15	2.45E-14
n379822	1360	18.66	0	0.43	0.01	-5.426264755	Down	3.07E-06	1.73E-05
n410720	915	12.28	0	0.43	0.01	-5.426264755	Down	0.000210376	0.00089014
n407800	2757	38	0	0.43	0.01	-5.426264755	Down	2.33E-12	2.30E-11
n408896	4462	183.2	4.43	1.27	0.03	-5.403722186	Down	3.34E-50	1.19E-48
85002	2438	33.25	0	0.42	0.01	-5.392317423	Down	7.89E-11	6.99E-10
n379972	2179	29.43	0	0.42	0.01	-5.392317423	Down	1.32E-09	1.05E-08
MXLOC_014171	4715	63.58	0	0.42	0.01	-5.392317423	Down	5.21E-20	7.95E-19
n410678	6285	86.55	0	0.42	0.01	-5.392317423	Down	4.77E-27	9.41E-26
n370715	3182	43.15	0	0.42	0.01	-5.392317423	Down	6.87E-14	7.53E-13
4929	3531	95	2	0.83	0.02	-5.375039431	Down	1.04E-26	2.03E-25
n346124	2419	32.18	0	0.41	0.01	-5.357552005	Down	1.60E-10	1.38E-09
n337683	3099	41.44	0	0.41	0.01	-5.357552005	Down	2.81E-13	2.97E-12
n375810	1117	14.19	0	0.41	0.01	-5.357552005	Down	5.14E-05	0.000241008
745	5745	77	0	0.41	0.01	-5.357552005	Down	2.71E-24	4.85E-23
MXLOC_022794	2356	30.88	0	0.41	0.01	-5.357552005	Down	6.53E-10	5.34E-09
389941	2493	1358.35	34.09	16.97	0.42	-5.336453427	Down	0	0
n373937	1181	14.93	0	0.4	0.01	-5.321928095	Down	5.14E-05	0.000241136
n411736	1065	13.36	0	0.4	0.01	-5.321928095	Down	0.00010399	0.000464027
n338985	5467	71.39	0	0.4	0.01	-5.321928095	Down	1.86E-22	3.13E-21
n376392	1067	13	0	0.39	0.01	-5.285402219	Down	0.00010399	0.000464144
n405913	19250	247.69	0	0.39	0.01	-5.285402219	Down	2.58E-76	1.38E-74
n365749	338	21.58	0.54	2.29	0.06	-5.254241287	Down	3.71E-07	2.31E-06
n383110	1068	12.58	0	0.38	0.01	-5.247927513	Down	0.000210376	0.000889926
119016	2431	29.92	0	0.38	0.01	-5.247927513	Down	1.32E-09	1.05E-08
n410532	3890	48.18	0	0.38	0.01	-5.247927513	Down	2.03E-15	2.45E-14
n337667	3179	38.54	0	0.38	0.01	-5.247927513	Down	2.33E-12	2.30E-11
n385522	1106	12.93	0	0.37	0.01	-5.209453366	Down	0.000210376	0.000890033
728343	3959	47.95	0	0.37	0.01	-5.209453366	Down	4.10E-15	4.85E-14
MXLOC_027857	8247	99.3	0	0.37	0.01	-5.209453366	Down	5.02E-31	1.13E-29
n338882	1596	18.39	0	0.36	0.01	-5.169925001	Down	3.07E-06	1.73E-05
MXLOC_019207	4626	54.4	0	0.36	0.01	-5.169925001	Down	2.96E-17	3.96E-16
n410259	1360	15.62	0	0.36	0.01	-5.169925001	Down	2.54E-05	0.000125201
n409072	4704	54.41	0	0.36	0.01	-5.169925001	Down	2.96E-17	3.96E-16
56994	1567	17.29	0	0.35	0.01	-5.129283017	Down	6.21E-06	3.35E-05
n326734	1210	13.47	0	0.35	0.01	-5.129283017	Down	0.00010399	0.000463792
LXLOC_014078	261	508.72	15.2	73.81	2.17	-5.088049342	Down	5.04E-132	5.10E-130
n406605	1828	19.81	0	0.34	0.01	-5.087462841	Down	1.52E-06	8.85E-06
n381852	2084	22.9	0	0.34	0.01	-5.087462841	Down	1.83E-07	1.18E-06
n410166	3069	34	0	0.34	0.01	-5.087462841	Down	3.90E-11	3.52E-10
MXLOC_005500	2963	32.51	0	0.34	0.01	-5.087462841	Down	1.60E-10	1.38E-09
n383548	2047	22.5	0	0.34	0.01	-5.087462841	Down	1.83E-07	1.18E-06
n411619	1208	25.53	0.68	0.67	0.02	-5.06608919	Down	2.21E-08	1.57E-07
n411681	2202	23.15	0	0.33	0.01	-5.044394119	Down	9.06E-08	6.05E-07
n378153	1941	20.18	0	0.33	0.01	-5.044394119	Down	7.50E-07	4.54E-06
n342254	2919	30.77	0	0.33	0.01	-5.044394119	Down	6.53E-10	5.34E-09
n410609	1316	13.56	0	0.33	0.01	-5.044394119	Down	0.00010399	0.00046391
3219	2701	28	0	0.32	0.01	-5	Down	2.67E-09	2.06E-08
LXLOC_016916	3202.94	32.65	0	0.32	0.01	-5	Down	1.60E-10	1.38E-09
n376393	1829	18.6	0	0.32	0.01	-5	Down	3.07E-06	1.72E-05
n385111	1558	15.58	0	0.32	0.01	-5	Down	2.54E-05	0.000125166
MXLOC_036252	2749.43	28.33	0	0.32	0.01	-5	Down	2.67E-09	2.06E-08

1280	4880	53	2	0.32	0.01	-5	Down	2.44E-14	2.75E-13
LXLOC_014380	2044	144.68	5	2.21	0.07	-4.980547637	Down	1.73E-37	4.59E-36
27124	2235	22.5	0	0.31	0.01	-4.95419631	Down	1.83E-07	1.18E-06
MXLOC_030857	3126	31.02	0	0.31	0.01	-4.95419631	Down	3.23E-10	2.71E-09
642968	1131	33	1	0.93	0.03	-4.95419631	Down	1.44E-09	1.13E-08
n378507	2244	21.98	0	0.31	0.01	-4.95419631	Down	3.71E-07	2.31E-06
n376844	1301	12.76	0	0.31	0.01	-4.95419631	Down	0.000210376	0.000888542
81	3954	39.97	0	0.31	0.01	-4.95419631	Down	1.15E-12	1.16E-11
MXLOC_015044	3254	31.42	0	0.3	0.01	-4.906890596	Down	3.23E-10	2.72E-09
257068	2063	19.51	0	0.3	0.01	-4.906890596	Down	1.52E-06	8.84E-06
n373838	1521	14.58	0	0.3	0.01	-4.906890596	Down	5.14E-05	0.000240624
MXLOC_011163	4145	1678.23	59.69	12.5	0.43	-4.861447625	Down	0	0
MXLOC_012189	10734	102.97	0	0.29	0.01	-4.857980995	Down	6.06E-32	1.39E-30
342125	3303	31.31	0.7	0.29	0.01	-4.857980995	Down	3.23E-10	2.72E-09
373863	1603	14.53	0	0.29	0.01	-4.857980995	Down	5.14E-05	0.000240944
n345501	1707	16	0	0.29	0.01	-4.857980995	Down	1.26E-05	6.48E-05
n409399	1799	16.56	0	0.29	0.01	-4.857980995	Down	1.26E-05	6.48E-05
n337979	2188	20.06	0	0.29	0.01	-4.857980995	Down	7.50E-07	4.53E-06
n369920	1545	14.13	0	0.29	0.01	-4.857980995	Down	5.14E-05	0.000240529
100529257	1914.32	17.49	0	0.29	0.01	-4.857980995	Down	6.21E-06	3.34E-05
MXLOC_031390	2170	180.45	6.3	2.6	0.09	-4.852442812	Down	7.77E-47	2.60E-45
MXLOC_017878	3681	32.91	0	0.28	0.01	-4.807354922	Down	1.60E-10	1.38E-09
LXLOC_024477	2973	27.19	0	0.28	0.01	-4.807354922	Down	5.41E-09	4.06E-08
n342805	1591	14	0	0.28	0.01	-4.807354922	Down	5.14E-05	0.000240848
n407954	1597	14.24	0	0.28	0.01	-4.807354922	Down	5.14E-05	0.000240784
n409303	2026	17.82	0	0.28	0.01	-4.807354922	Down	6.21E-06	3.35E-05
n369674	2566	23.43	0	0.28	0.01	-4.807354922	Down	9.06E-08	6.05E-07
n339340	4114	36.88	0	0.28	0.01	-4.807354922	Down	9.53E-12	9.02E-11
314	2584	22.94	0	0.28	0.01	-4.807354922	Down	1.83E-07	1.18E-06
n410470	3629	32.36	0	0.28	0.01	-4.807354922	Down	1.60E-10	1.38E-09
MXLOC_012695	639	37.51	1.34	1.95	0.07	-4.799975392	Down	9.52E-11	8.35E-10
MXLOC_035473	428	27	1	2.19	0.08	-4.77478706	Down	8.20E-08	5.50E-07
n410562	1691	14.32	0	0.27	0.01	-4.754887502	Down	5.14E-05	0.000241168
n337985	5918	52.13	2.2	0.27	0.01	-4.754887502	Down	4.77E-14	5.28E-13
n407885	5534	48.81	0	0.27	0.01	-4.754887502	Down	2.03E-15	2.45E-14
n408156	2127	18.44	0	0.27	0.01	-4.754887502	Down	3.07E-06	1.72E-05
n340974	1557	13.09	0	0.27	0.01	-4.754887502	Down	0.00010399	0.000463968
n379141	2737	23.54	0	0.27	0.01	-4.754887502	Down	9.06E-08	6.04E-07
n410101	1547	13.35	0	0.27	0.01	-4.754887502	Down	0.00010399	0.000463851
MXLOC_023119	3119.71	442.4	14.23	3.68	0.14	-4.716207034	Down	6.62E-114	5.54E-112
154796	6514	352.14	13.47	1.57	0.06	-4.709658248	Down	7.69E-89	4.93E-87
n338959	3593	30.08	0	0.26	0.01	-4.700439718	Down	6.53E-10	5.34E-09
n386038	2130	17.56	0	0.26	0.01	-4.700439718	Down	6.21E-06	3.35E-05
7026	3501	38	1	0.26	0.01	-4.700439718	Down	4.82E-11	4.33E-10
n341514	3282	27.36	0	0.26	0.01	-4.700439718	Down	5.41E-09	4.06E-08
MXLOC_023008	5615	511.17	20.82	2.86	0.11	-4.700439718	Down	4.98E-127	4.83E-125
n411670	2117	17.5	0	0.26	0.01	-4.700439718	Down	6.21E-06	3.35E-05
10267	910	221	9	7.85	0.31	-4.662352533	Down	2.32E-55	9.16E-54
MXLOC_023529	4838	38.81	0	0.25	0.01	-4.64385619	Down	2.33E-12	2.30E-11
MXLOC_017334	5411	1186.91	48.42	6.75	0.27	-4.64385619	Down	6.08E-291	1.42E-288
n378978	1648	12.91	0	0.25	0.01	-4.64385619	Down	0.000210376	0.0008895
n338401	2655	21.63	0	0.25	0.01	-4.64385619	Down	3.71E-07	2.31E-06
728689	3053	23.78	0	0.24	0.01	-4.584962501	Down	9.06E-08	6.05E-07
100529241	4279	33.11	0	0.24	0.01	-4.584962501	Down	7.89E-11	6.99E-10
5627	3569	27.35	0	0.24	0.01	-4.584962501	Down	5.41E-09	4.06E-08
n411677	5021	38.36	0	0.24	0.01	-4.584962501	Down	2.33E-12	2.30E-11
374786	3745	29.41	0	0.24	0.01	-4.584962501	Down	1.32E-09	1.05E-08
105	8426	263.94	11.48	0.96	0.04	-4.584962501	Down	2.63E-65	1.22E-63
MXLOC_015359	1597	12.03	0	0.24	0.01	-4.584962501	Down	0.000210376	0.000888861
MXLOC_008586	1964	14.68	0	0.23	0.01	-4.523561956	Down	5.14E-05	0.000241072

n407892	4920	37.11	0	0.23	0.01	-4.523561956	Down	4.71E-12	4.55E-11
4188	1617	12	0	0.23	0.01	-4.523561956	Down	0.000210376	0.000889607
n408132	4427	33.16	0	0.23	0.01	-4.523561956	Down	7.89E-11	6.98E-10
8614	5343	280	13	1.61	0.07	-4.523561956	Down	4.59E-68	2.21E-66
n406583	5511	41.93	0	0.23	0.01	-4.523561956	Down	2.81E-13	2.97E-12
n410512	2365	17.32	0	0.23	0.01	-4.523561956	Down	6.21E-06	3.34E-05
n337737	1264	63.8	2.99	1.61	0.07	-4.523561956	Down	2.95E-17	3.95E-16
MXLOC_015657	2179	16.24	0	0.23	0.01	-4.523561956	Down	1.26E-05	6.48E-05
3216	1676	485.5	22.24	9.12	0.41	-4.47533801	Down	2.33E-117	2.02E-115
n406602	2632	954.26	44.49	11.28	0.51	-4.46712601	Down	1.58E-228	2.77E-226
2329	2139	15	1	0.22	0.01	-4.459431619	Down	0.000230996	0.000968335
n374518	2729	19	0.78	0.22	0.01	-4.459431619	Down	1.52E-06	8.84E-06
3490	1110	23	1	0.66	0.03	-4.459431619	Down	1.19E-06	7.04E-06
3693	3372	24	0	0.22	0.01	-4.459431619	Down	4.48E-08	3.08E-07
n384663	756	20	1	0.87	0.04	-4.442943496	Down	8.71E-06	4.60E-05
2697	3130	109	5	1.08	0.05	-4.432959407	Down	2.32E-27	4.64E-26
84929	3119	218.54	10.02	2.16	0.1	-4.432959407	Down	1.98E-53	7.59E-52
n335998	299	62.22	3.01	7.65	0.36	-4.409390936	Down	6.53E-16	8.15E-15
n375042	2211	15	0	0.21	0.01	-4.392317423	Down	2.54E-05	0.000125306
n339851	2061	13.61	0	0.21	0.01	-4.392317423	Down	0.00010399	0.000464262
202	7553	51.82	0	0.21	0.01	-4.392317423	Down	2.45E-16	3.13E-15
57084	3925	512	26	4.03	0.2	-4.332707934	Down	6.23E-121	5.70E-119
n342387	4185	26.65	0	0.2	0.01	-4.321928095	Down	1.09E-08	8.00E-08
n409414	2784	18.22	0	0.2	0.01	-4.321928095	Down	3.07E-06	1.73E-05
4772	3140	104	4	0.8	0.04	-4.321928095	Down	5.59E-27	1.10E-25
n406497	2158	13.92	0	0.2	0.01	-4.321928095	Down	0.00010399	0.000464086
79611	3033	20	0	0.2	0.01	-4.321928095	Down	7.50E-07	4.54E-06
n405311	1115	21	1.11	0.6	0.03	-4.321928095	Down	4.49E-06	2.47E-05
n339066	1011	55	3	1.75	0.09	-4.28128611	Down	6.47E-14	7.11E-13
3371	7616	190	9	0.77	0.04	-4.266786541	Down	1.91E-46	6.30E-45
n381924	3384	20.7	0	0.19	0.01	-4.247927513	Down	7.50E-07	4.54E-06
n370710	2497	15.25	0	0.19	0.01	-4.247927513	Down	2.54E-05	0.000125131
n409289	2408	14.3	0	0.19	0.01	-4.247927513	Down	5.14E-05	0.000240593
121227	3661	23	0	0.19	0.01	-4.247927513	Down	9.06E-08	6.04E-07
LXLOC_031131	2906	18.01	0	0.19	0.01	-4.247927513	Down	3.07E-06	1.72E-05
n411634	2970	18.15	0	0.19	0.01	-4.247927513	Down	3.07E-06	1.72E-05
1901	3050	56	3	0.57	0.03	-4.247927513	Down	3.36E-14	3.77E-13
MXLOC_015267	4035	121.5	6.76	0.93	0.05	-4.217230716	Down	8.86E-30	1.92E-28
n341290	2506	74.56	4.09	0.93	0.05	-4.217230716	Down	2.32E-18	3.29E-17
LXLOC_029572	2986	196.31	10.78	2.04	0.11	-4.212993723	Down	3.83E-47	1.29E-45
MXLOC_032738	4145	270.3	14.7	2.01	0.11	-4.191620073	Down	3.45E-64	1.55E-62
n380885	1075	18.15	1.01	0.54	0.03	-4.169925001	Down	3.26E-05	0.000157858
n346107	3073	18	0	0.18	0.01	-4.169925001	Down	3.07E-06	1.72E-05
n408044	5913	35.02	0	0.18	0.01	-4.169925001	Down	1.93E-11	1.79E-10
1520	4107	23.3	0	0.18	0.01	-4.169925001	Down	9.06E-08	6.05E-07
n410596	1939	1934.24	111.35	31.25	1.76	-4.150208856	Down	0	0
8368	364	18	1	1.76	0.1	-4.137503524	Down	3.26E-05	0.000157879
LXLOC_020928	3742	104	6	0.86	0.05	-4.10433666	Down	5.94E-25	1.09E-23
120376	1414	15	1	0.34	0.02	-4.087462841	Down	0.000230996	0.00096845
143098	5207	28.68	0	0.17	0.01	-4.087462841	Down	2.67E-09	2.06E-08
5358	3327	18.21	0	0.17	0.01	-4.087462841	Down	3.07E-06	1.73E-05
368	2835.21	15.26	0	0.17	0.01	-4.087462841	Down	2.54E-05	0.000125271
n340548	2955	16	0	0.17	0.01	-4.087462841	Down	1.26E-05	6.48E-05
MXLOC_013399	2954	16.35	0	0.17	0.01	-4.087462841	Down	1.26E-05	6.48E-05
6507	4170	23	0	0.17	0.01	-4.087462841	Down	9.06E-08	6.04E-07
3125	1158	72.66	4.37	2	0.12	-4.058893689	Down	8.57E-18	1.18E-16
n340290	3357	196.99	12.6	1.82	0.11	-4.048363022	Down	3.26E-45	1.04E-43
7757	9088	97.11	5.62	0.33	0.02	-4.044394119	Down	6.25E-24	1.11E-22
n378002	1959	20.61	1.06	0.33	0.02	-4.044394119	Down	8.71E-06	4.60E-05
152573	1971	31	2	0.49	0.03	-4.029747343	Down	4.91E-08	3.37E-07

n377342	590	88.64	5.57	5.02	0.31	-4.017347244	Down	2.24E-21	3.62E-20
n386056	2432	12.56	0	0.16	0.01	-4	Down	0.000210376	0.000890353
84077	2826	16	1	0.16	0.01	-4	Down	0.000120534	0.000532932
LXLOC_025990	4361	21.99	0	0.16	0.01	-4	Down	3.71E-07	2.31E-06
445582	3440	17.3	0	0.16	0.01	-4	Down	6.21E-06	3.35E-05
n341171	3201	16.23	0	0.16	0.01	-4	Down	1.26E-05	6.48E-05
n344540	6515	33.37	0	0.16	0.01	-4	Down	7.89E-11	6.98E-10
6571	3872	257.17	16.81	2.05	0.13	-3.979040381	Down	1.35E-58	5.60E-57
LXLOC_020930	6166	315	21	1.57	0.1	-3.972692654	Down	3.24E-70	1.60E-68
196051	1521	150	10	3.11	0.2	-3.958842675	Down	3.52E-34	8.65E-33
n384608	1807	18	1	0.31	0.02	-3.95419631	Down	3.26E-05	0.000157814
3399	1288	74	5	1.83	0.12	-3.930737338	Down	1.90E-17	2.58E-16
LXLOC_011369	2892	85	6	0.91	0.06	-3.922832139	Down	1.23E-19	1.85E-18
n374668	3814	18	0	0.15	0.01	-3.906890596	Down	3.07E-06	1.73E-05
n407062	5924	28.27	0	0.15	0.01	-3.906890596	Down	2.67E-09	2.06E-08
n370721	5024	24.02	0	0.15	0.01	-3.906890596	Down	4.48E-08	3.08E-07
n409187	2499	12.17	0	0.15	0.01	-3.906890596	Down	0.000210376	0.000888436
386617	2593	185	13	2.22	0.15	-3.887525271	Down	3.04E-41	8.83E-40
10194	4967	190.09	13.32	1.18	0.08	-3.882643049	Down	1.25E-42	3.74E-41
n365842	970	79.91	5.65	2.65	0.18	-3.879923548	Down	7.64E-19	1.11E-17
9	1799	26	2	0.44	0.03	-3.874469118	Down	1.22E-06	7.19E-06
2662	2650	25	2	0.29	0.02	-3.857980995	Down	2.30E-06	1.32E-05
LXLOC_019970	2945	465.14	33.12	4.9	0.34	-3.849175098	Down	3.91E-101	2.93E-99
3592	1450	39	3	0.85	0.06	-3.824428435	Down	1.97E-09	1.54E-08
n410807	1707	46	3.06	0.85	0.06	-3.824428435	Down	2.24E-11	2.06E-10
MXLOC_015020	4097	18.33	0	0.14	0.01	-3.807354922	Down	3.07E-06	1.73E-05
n409254	4084	18.08	0	0.14	0.01	-3.807354922	Down	3.07E-06	1.73E-05
n408008	3436	15.98	0	0.14	0.01	-3.807354922	Down	2.54E-05	0.000125236
MXLOC_028713	3782.74	17.25	0	0.14	0.01	-3.807354922	Down	6.21E-06	3.35E-05
148398	2554	742.72	54.83	9.05	0.65	-3.799406169	Down	3.17E-159	3.81E-157
MXLOC_035472	650	40	3	2.04	0.15	-3.765534746	Down	1.05E-09	8.38E-09
LXLOC_027556	3708	97.33	7.83	0.81	0.06	-3.754887502	Down	4.25E-22	7.08E-21
26280	2985	378.97	29.22	3.94	0.3	-3.715161224	Down	1.24E-80	7.10E-79
MXLOC_012350	4800	326.69	25.3	2.1	0.16	-3.714245518	Down	9.18E-70	4.51E-68
60680	5008	20.32	0	0.13	0.01	-3.700439718	Down	7.50E-07	4.54E-06
n407275	4184	18.08	0	0.13	0.01	-3.700439718	Down	3.07E-06	1.73E-05
2571	3476	15	0	0.13	0.01	-3.700439718	Down	2.54E-05	0.000125218
79659	13684.02	57.74	0	0.13	0.01	-3.700439718	Down	3.57E-18	4.99E-17
7784	1300	16.95	1.19	0.39	0.03	-3.700439718	Down	0.000120534	0.000532865
5027	3680	15	0	0.13	0.01	-3.700439718	Down	2.54E-05	0.000125149
MXLOC_024955	7119	31.16	0	0.13	0.01	-3.700439718	Down	3.23E-10	2.72E-09
LXLOC_020926	6128	181	15	0.91	0.07	-3.700439718	Down	1.85E-38	5.05E-37
153	2862	23.57	1.52	0.26	0.02	-3.700439718	Down	1.19E-06	7.04E-06
LXLOC_020922	1104	76	6	2.2	0.17	-3.693896872	Down	3.74E-17	4.97E-16
LXLOC_020923	10487	613	48	1.79	0.14	-3.676460855	Down	6.10E-129	5.98E-127
MXLOC_036897	1160	1932.5	154.43	53.18	4.16	-3.676228344	Down	0	0
9249	1806	66	5	1.15	0.09	-3.67556505	Down	3.13E-15	3.74E-14
MXLOC_002398	3003	122.14	10.23	1.26	0.1	-3.655351829	Down	1.84E-26	3.56E-25
n410475	3343	148.49	12.42	1.38	0.11	-3.649092838	Down	6.42E-32	1.47E-30
4600	2961	23.77	2.07	0.25	0.02	-3.64385619	Down	8.14E-06	4.31E-05
3198	2549	534	44	6.6	0.53	-3.63840176	Down	1.00E-110	8.25E-109
5655	2968	83.57	7	0.87	0.07	-3.635588574	Down	2.94E-18	4.13E-17
n1850	1116	38.76	3.03	1.11	0.09	-3.624490865	Down	3.72E-09	2.83E-08
n342655	3444	41.2	3.95	0.37	0.03	-3.624490865	Down	5.53E-10	4.55E-09
MXLOC_036284	5675	270.46	21.98	1.47	0.12	-3.614709844	Down	8.60E-58	3.52E-56
LXLOC_020927	1486	23	2	0.49	0.04	-3.614709844	Down	8.14E-06	4.31E-05
MXLOC_015655	5523	11911.31	1005.08	66.38	5.48	-3.598500831	Down	0	0
143872	4752	37	3	0.24	0.02	-3.584962501	Down	7.00E-09	5.20E-08
3662	5327	20	0	0.12	0.01	-3.584962501	Down	7.50E-07	4.54E-06
9705	6330	222.34	18.96	1.08	0.09	-3.584962501	Down	3.59E-47	1.21E-45

n384089	1589	102.67	8.92	2.04	0.17	-3.584962501	Down	1.24E-22	2.10E-21
n372461	921	24	2	0.84	0.07	-3.584962501	Down	4.33E-06	2.38E-05
n383232	829	522.24	44.45	20.49	1.71	-3.582851754	Down	1.82E-107	1.44E-105
MXLOC_021970	2773	95.89	8.13	1.07	0.09	-3.571541985	Down	1.01E-20	1.58E-19
55553	8865	242	21	0.83	0.07	-3.567684509	Down	3.57E-50	1.27E-48
MXLOC_035471	1674	126	11	2.37	0.2	-3.566815154	Down	9.53E-27	1.86E-25
n377728	1266	23.44	2	0.59	0.05	-3.560714954	Down	8.14E-06	4.31E-05
n384826	3669	96.87	7.99	0.82	0.07	-3.550197083	Down	8.02E-22	1.32E-20
MXLOC_031484	3100	279.41	24.89	2.8	0.24	-3.544320516	Down	8.76E-58	3.58E-56
53353	16531	1826	162	3.38	0.29	-3.542898441	Down	0	0
3491	2295	43	4	0.58	0.05	-3.5360529	Down	9.55E-10	7.68E-09
204962	3215.07	327.67	35.75	3.85	0.34	-3.501251794	Down	2.90E-62	1.27E-60
84189	4181	61	5	0.45	0.04	-3.491853096	Down	7.37E-14	8.07E-13
392617	3742	431.42	39.46	3.56	0.32	-3.475733431	Down	6.84E-87	4.30E-85
LXLOC_003663	516	22	2	1.44	0.13	-3.469485283	Down	1.52E-05	7.77E-05
374860	4617	17	0	0.11	0.01	-3.459431619	Down	6.21E-06	3.35E-05
LXLOC_029508	8615	92.95	9.27	0.33	0.03	-3.459431619	Down	3.80E-19	5.62E-18
n324090	931	76	7	2.64	0.24	-3.459431619	Down	2.30E-16	2.95E-15
27145	4622	17.05	0	0.11	0.01	-3.459431619	Down	6.21E-06	3.34E-05
LXLOC_034284	3342	12.37	0	0.11	0.01	-3.459431619	Down	0.000210376	0.000889074
22915	4969	18	1	0.11	0.01	-3.459431619	Down	3.26E-05	0.000157836
n345101	4258	30.89	2.53	0.22	0.02	-3.459431619	Down	9.37E-08	6.24E-07
222546	3507	13	0	0.11	0.01	-3.459431619	Down	0.00010399	0.000463675
MXLOC_010996	1673	137.28	13.26	2.58	0.24	-3.426264755	Down	3.68E-28	7.58E-27
n369035	5849	61.79	6.73	0.32	0.03	-3.415037499	Down	4.30E-13	4.48E-12
n379717	2868	59.12	5.29	0.64	0.06	-3.415037499	Down	2.59E-13	2.74E-12
n341471	752	22	2	0.96	0.09	-3.415037499	Down	1.52E-05	7.76E-05
n342022	2086	21.52	1.99	0.32	0.03	-3.415037499	Down	4.49E-06	2.47E-05
n339808	1616	16.46	1.79	0.32	0.03	-3.415037499	Down	0.000120534	0.000532665
n379712	307	62.73	6.05	7.47	0.71	-3.395217313	Down	2.32E-13	2.47E-12
2261	3951	29	3	0.21	0.02	-3.392317423	Down	1.03E-06	6.11E-06
2042	5756.91	8104	805	43.95	4.21	-3.383971027	Down	0	0
n407967	1615	58.63	5.92	1.14	0.11	-3.373458396	Down	4.84E-13	5.04E-12
143903	2066	115.85	11.45	1.75	0.17	-3.363748271	Down	8.63E-24	1.52E-22
n385571	1971	32	3.2	0.51	0.05	-3.350497247	Down	1.61E-07	1.04E-06
1277	5927	99	9	0.51	0.05	-3.350497247	Down	5.07E-21	8.05E-20
3108	1122	60	6	1.71	0.17	-3.330389674	Down	7.94E-13	8.13E-12
5100	3886.67	964	99	7.74	0.77	-3.329403215	Down	2.47E-183	3.40E-181
MXLOC_030983	3597	292.38	29.22	2.51	0.25	-3.327687364	Down	2.07E-57	8.36E-56
25909	8633	707.38	70.87	2.51	0.25	-3.327687364	Down	7.37E-137	7.54E-135
4703	26202	82.26	0	0.1	0.01	-3.321928095	Down	7.99E-26	1.51E-24
MXLOC_035895	6258	19.47	0	0.1	0.01	-3.321928095	Down	1.52E-06	8.84E-06
3269	4278	55	6	0.4	0.04	-3.321928095	Down	1.67E-11	1.56E-10
n410811	5007	16.29	0	0.1	0.01	-3.321928095	Down	1.26E-05	6.48E-05
n387524	3615	12.24	0	0.1	0.01	-3.321928095	Down	0.000210376	0.000889393
23418	5006	17	0	0.1	0.01	-3.321928095	Down	6.21E-06	3.34E-05
n406527	3951	12.75	0	0.1	0.01	-3.321928095	Down	0.000210376	0.000888649
n338544	2515	16.25	1.27	0.2	0.02	-3.321928095	Down	0.000120534	0.000532798
n382104	6322	20.51	0	0.1	0.01	-3.321928095	Down	7.50E-07	4.53E-06
n409199	5631	18.6	0	0.1	0.01	-3.321928095	Down	3.07E-06	1.72E-05
n339726	2570	41	4	0.5	0.05	-3.321928095	Down	3.29E-09	2.52E-08
285440	4704	198.77	20.19	1.3	0.13	-3.321928095	Down	3.66E-39	1.02E-37
51454	3451	34	3	0.3	0.03	-3.321928095	Down	4.62E-08	3.17E-07
MXLOC_004318	3953	126.85	12.82	0.99	0.1	-3.307428525	Down	5.63E-26	1.07E-24
n386682	17357	506.49	54.48	0.89	0.09	-3.30580843	Down	1.42E-95	9.84E-94
5176	1533	19	2	0.39	0.04	-3.285402219	Down	9.83E-05	0.000440861
23180	3002	519	54	5.36	0.55	-3.284729477	Down	5.44E-99	3.99E-97
3354	2048	81.99	9	1.25	0.13	-3.265344567	Down	3.06E-16	3.90E-15
LXLOC_006404	3275	121.48	12.51	1.15	0.12	-3.26052755	Down	1.21E-24	2.19E-23
3910	5065.4	32	8	0.47	0.05	-3.232660757	Down	8.37E-05	0.000378212

n340046	3619	88	9	0.75	0.08	-3.22881869	Down	4.39E-18	6.12E-17
n381838	7863	72.46	7.2	0.28	0.03	-3.222392421	Down	2.71E-15	3.25E-14
MXLOC_015653	2068	48.97	5.21	0.74	0.08	-3.209453366	Down	2.34E-10	2.00E-09
1289	8439	102	12	0.37	0.04	-3.209453366	Down	1.18E-19	1.77E-18
n337645	1711	20	2	0.37	0.04	-3.209453366	Down	5.30E-05	0.00024745
130399	8761	108	12	0.37	0.04	-3.209453366	Down	3.24E-21	5.19E-20
n406902	2602	100	11	1.2	0.13	-3.206450877	Down	8.04E-20	1.22E-18
MXLOC_006990	7193	212.25	24.4	0.91	0.1	-3.185866545	Down	5.54E-40	1.57E-38
MXLOC_035257	526	16.93	1.97	1.09	0.12	-3.183221824	Down	0.000120534	0.000532732
n344861	1762	15.02	1.47	0.27	0.03	-3.169925001	Down	0.000230996	0.00096822
n406634	924	53.95	6.01	1.89	0.21	-3.169925001	Down	5.59E-11	5.00E-10
n338827	1892	27	3	0.45	0.05	-3.169925001	Down	3.48E-06	1.94E-05
n408234	3294	67.29	7.59	0.63	0.07	-3.169925001	Down	5.79E-14	6.37E-13
n407999	7491	21.36	0	0.09	0.01	-3.169925001	Down	3.71E-07	2.31E-06
n338846	4408	12.38	0	0.09	0.01	-3.169925001	Down	0.000210376	0.000888968
MXLOC_011459	15557	44.1	0	0.09	0.01	-3.169925001	Down	3.40E-14	3.79E-13
64283	6291	18	0	0.09	0.01	-3.169925001	Down	3.07E-06	1.72E-05
n338096	2601	247	28	2.95	0.33	-3.160177025	Down	2.61E-46	8.62E-45
56243	6205.74	238	26	1.16	0.13	-3.157541277	Down	2.31E-45	7.42E-44
MXLOC_037356	2264	44.65	4.96	0.62	0.07	-3.146841388	Down	5.13E-10	4.24E-09
n379069	3839	77.57	9.04	0.62	0.07	-3.146841388	Down	3.38E-15	4.02E-14
n410473	2003	38.86	4.9	0.61	0.07	-3.123382416	Down	2.08E-08	1.48E-07
MXLOC_011854	3267	256.13	30.32	2.43	0.28	-3.117457582	Down	2.87E-47	9.71E-46
n379749	410	182.37	21.65	15.51	1.81	-3.099137084	Down	2.93E-34	7.20E-33
LXLOC_020925	3166	96	11	0.94	0.11	-3.095157233	Down	8.90E-19	1.28E-17
n341049	1988	21.29	2.77	0.34	0.04	-3.087462841	Down	2.85E-05	0.000139221
7104	1607	1443	174	28.29	3.34	-3.082372169	Down	2.12E-255	4.22E-253
LXLOC_008663	6568	1656.62	200.36	7.75	0.92	-3.074490544	Down	1.32E-292	3.14E-290
MXLOC_014295	1297	51.34	6.29	1.26	0.15	-3.070389328	Down	1.86E-10	1.59E-09
n342302	4415	95.87	12	0.67	0.08	-3.06608919	Down	7.45E-18	1.03E-16
MXLOC_031421	774	220.11	26.8	9.29	1.11	-3.06511892	Down	1.10E-40	3.16E-39
n365255	10397	480.95	60	1.42	0.17	-3.062284278	Down	9.97E-85	6.09E-83
n345091	1182	55.71	6.93	1.5	0.18	-3.058893689	Down	1.67E-11	1.56E-10
n383733	4525	146.3	18.64	1	0.12	-3.058893689	Down	5.42E-27	1.06E-25
6530	3274.25	4059	511	38.76	4.73	-3.034656482	Down	0	0
29899	3039	143.57	18.49	1.47	0.18	-3.029747343	Down	3.17E-26	6.09E-25
3199	1778	110.62	13.76	1.95	0.24	-3.022367813	Down	4.72E-21	7.52E-20
LXLOC_020929	3305	78	10	0.73	0.09	-3.019899557	Down	8.47E-15	9.79E-14
n406645	2558	32.96	4.12	0.4	0.05	-3	Down	7.75E-07	4.68E-06
9902	5983	47	6	0.24	0.03	-3	Down	2.01E-09	1.56E-08
2053	2196	39.5	4.93	0.56	0.07	-3	Down	1.13E-08	8.21E-08
n411009	3918	41.05	4.59	0.32	0.04	-3	Down	3.29E-09	2.52E-08
n377684	3049	31.53	4.42	0.32	0.04	-3	Down	1.40E-06	8.23E-06
5924	4078	334	43.77	2.53	0.32	-2.982993575	Down	1.22E-58	5.07E-57
MXLOC_015649	5565	1028.08	132.22	5.69	0.72	-2.982359841	Down	3.48E-177	4.58E-175
n386308	1975	34.89	4.52	0.55	0.07	-2.974004791	Down	2.34E-07	1.50E-06
n346404	2627	185.83	24.44	2.2	0.28	-2.974004791	Down	4.82E-33	1.15E-31
n334773	678	24.06	3.05	1.17	0.15	-2.963474124	Down	2.13E-05	0.000105935
11144	2269	28.44	3.86	0.39	0.05	-2.963474124	Down	1.89E-06	1.09E-05
100532736	2210	99.13	13.25	1.4	0.18	-2.959358016	Down	3.11E-18	4.37E-17
80709	7387	149.88	20.72	0.62	0.08	-2.95419631	Down	1.80E-26	3.48E-25
n381254	5053	139.25	18.19	0.85	0.11	-2.949959318	Down	3.31E-25	6.15E-24
MXLOC_018771	2573	265.93	35.7	3.22	0.42	-2.938599455	Down	2.77E-46	9.11E-45
n371974	780	22	3	0.92	0.12	-2.938599455	Down	6.97E-05	0.000320432
n410557	1394	26.78	3.79	0.61	0.08	-2.930737338	Down	6.39E-06	3.43E-05
153339	4587	10053.23	1358.19	67.59	8.93	-2.920077734	Down	0	0
n376692	1290	27.56	3.53	0.68	0.09	-2.91753784	Down	3.48E-06	1.94E-05
100131187	578.46	263	38	16.31	2.16	-2.916653565	Down	6.24E-44	1.94E-42
MXLOC_031445	597	240.16	32.39	13.43	1.78	-2.915510158	Down	7.15E-42	2.11E-40
n378839	542	55.56	7.56	3.45	0.46	-2.906890596	Down	7.75E-11	6.87E-10

4921	3172	77	10	0.75	0.1	-2.906890596	Down	1.52E-14	1.74E-13
n406432	1184	96.87	13.11	2.61	0.35	-2.89862298	Down	1.79E-17	2.42E-16
n345975	1261	26.64	3.44	0.67	0.09	-2.896164189	Down	6.39E-06	3.43E-05
54504	1691	36	5	0.67	0.09	-2.896164189	Down	3.06E-07	1.93E-06
55790	4221.86	91.25	12	0.67	0.09	-2.896164189	Down	7.78E-17	1.02E-15
MXLOC_021275	1628	61.39	8.4	1.19	0.16	-2.894817763	Down	9.80E-12	9.27E-11
150297	805	22	3	0.89	0.12	-2.89077093	Down	6.97E-05	0.000320515
n342938	1702	20	3	0.37	0.05	-2.887525271	Down	0.00022474	0.000944015
1012	4021	1157	161	8.95	1.21	-2.886880635	Down	2.48E-191	3.57E-189
n381071	1111	28	4	0.81	0.11	-2.880418384	Down	8.18E-06	4.33E-05
64405	3657	217.75	30.22	1.84	0.25	-2.879705766	Down	2.44E-37	6.46E-36
n406840	2732	19.21	2.27	0.22	0.03	-2.874469118	Down	9.83E-05	0.000440917
LXLOC_036913	2645	62.03	8.78	0.73	0.1	-2.867896464	Down	5.45E-12	5.24E-11
7576	4594	237.77	33.02	1.6	0.22	-2.862496476	Down	1.70E-40	4.88E-39
n341787	2516	164	23	2.03	0.28	-2.857980995	Down	2.22E-28	4.62E-27
222537	2744	293.89	41.79	3.33	0.46	-2.855816411	Down	1.42E-49	5.00E-48
MXLOC_019114	1975	223	31.5	3.54	0.49	-2.852895706	Down	3.17E-38	8.61E-37
n365544	843	35.6	4.91	1.37	0.19	-2.85010457	Down	1.28E-07	8.44E-07
n341227	527	28	4	1.8	0.25	-2.847996907	Down	8.18E-06	4.33E-05
LXLOC_010989	293.91	98940.31	14003.83	12417.01	1728.97	-2.844333075	Down	0	0
MXLOC_018806	5066	165.2	23.18	1	0.14	-2.836501268	Down	1.25E-28	2.63E-27
n386184	1855	84.19	11.81	1.42	0.2	-2.827819025	Down	1.09E-15	1.34E-14
7082	7165	272.71	41.65	1.2	0.17	-2.819427754	Down	2.26E-44	7.09E-43
LXLOC_012659	380	57.03	8.28	5.29	0.75	-2.818305222	Down	1.01E-10	8.84E-10
1287	6427.05	14.43	0	0.07	0.01	-2.807354922	Down	5.14E-05	0.00024088
n408230	7673	17.49	0	0.07	0.01	-2.807354922	Down	6.21E-06	3.35E-05
LXLOC_012243	5954	27.69	4.31	0.14	0.02	-2.807354922	Down	1.46E-05	7.46E-05
9098	7971	200.98	27.97	0.77	0.11	-2.807354922	Down	6.18E-35	1.55E-33
26509	6860	15	0	0.07	0.01	-2.807354922	Down	2.54E-05	0.000125096
LXLOC_011162	3262	15.02	1.64	0.14	0.02	-2.807354922	Down	0.000230996	0.000968105
MXLOC_028351	4722	459.11	67.48	3	0.43	-2.802553936	Down	4.59E-75	2.41E-73
MXLOC_001632	7875	391.82	57.1	1.53	0.22	-2.797956224	Down	3.11E-64	1.41E-62
MXLOC_031512	921	496.49	73.12	17.42	2.51	-2.794985355	Down	1.27E-80	7.28E-79
MXLOC_019319	1551	64.49	9.52	1.31	0.19	-2.785495488	Down	7.08E-12	6.76E-11
11341	903	1591	236	57	8.28	-2.783259246	Down	1.47E-253	2.88E-251
84239	4274	219	32	1.58	0.23	-2.780218792	Down	1.27E-36	3.31E-35
LXLOC_001883	9182	1169	176	3.9	0.57	-2.7744403	Down	2.79E-185	3.90E-183
n337805	568	45	6.7	2.66	0.39	-2.769880217	Down	6.51E-09	4.85E-08
3776	3298.32	675	101	6.33	0.93	-2.766902878	Down	4.07E-108	3.25E-106
n407040	1344	26.02	3.9	0.61	0.09	-2.760812336	Down	6.39E-06	3.43E-05
n375515	611	65.78	9.94	3.59	0.53	-2.759919579	Down	3.97E-12	3.85E-11
79924	4246	74	11.72	0.54	0.08	-2.754887502	Down	3.53E-13	3.71E-12
2719	2319	264	40	3.55	0.53	-2.74375476	Down	5.41E-43	1.64E-41
238	6265	846	129	4.15	0.62	-2.742771216	Down	1.21E-133	1.23E-131
604	3567	148	22	1.27	0.19	-2.740757173	Down	5.26E-25	9.68E-24
56103	2436	30.02	2.21	0.2	0.03	-2.736965594	Down	9.37E-08	6.24E-07
3096	8874	1172.12	179.27	4.05	0.61	-2.73104076	Down	2.91E-184	4.03E-182
5923	6354.17	293	46	1.46	0.22	-2.73039294	Down	1.28E-46	4.26E-45
57596	2749.98	216.5	34.41	2.52	0.38	-2.72935241	Down	1.01E-34	2.51E-33
6133	750	8963.53	1382.46	391.3	59.15	-2.725825037	Down	0	0
n409501	2010	21	3	0.33	0.05	-2.722466024	Down	0.000125491	0.000552563
n372244	1817	38	6	0.66	0.1	-2.722466024	Down	3.70E-07	2.31E-06
MXLOC_017353	3824	40.6	6.25	0.33	0.05	-2.722466024	Down	1.18E-07	7.79E-07
LXLOC_017105	7052	151.33	22.98	0.66	0.1	-2.722466024	Down	9.52E-26	1.80E-24
LXLOC_008170	1294	27	4.31	0.66	0.1	-2.722466024	Down	1.46E-05	7.46E-05
2302	2641	28	4	0.33	0.05	-2.722466024	Down	8.18E-06	4.33E-05
n406661	2418	77.21	12.21	0.99	0.15	-2.722466024	Down	2.43E-13	2.58E-12
n338650	935	65	10	2.24	0.34	-2.719892081	Down	1.55E-11	1.45E-10
n340059	2246	32.74	5.48	0.46	0.07	-2.716207034	Down	3.08E-06	1.72E-05
MXLOC_000836	2812	22.71	3.51	0.26	0.04	-2.700439718	Down	6.97E-05	0.000320474

3123	1166	47.1	7.62	1.29	0.2	-2.689299161	Down	8.15E-09	6.02E-08
MXLOC_021837	4819.46	385.78	58.84	2.37	0.37	-2.679289883	Down	3.66E-62	1.60E-60
n380977	656	29.11	4.65	1.47	0.23	-2.676110389	Down	4.56E-06	2.50E-05
n410323	3753	147.4	24.16	1.21	0.19	-2.670935724	Down	1.28E-23	2.25E-22
LXLOC_021123	3225	72.74	11.78	0.7	0.11	-2.669851398	Down	1.10E-12	1.11E-11
LXLOC_011368	597	25	4	1.4	0.22	-2.669851398	Down	4.60E-05	0.000217591
256380	4536	290.24	46.86	1.97	0.31	-2.667855509	Down	6.88E-46	2.24E-44
114823	3970	628.47	100.62	4.89	0.77	-2.666904114	Down	3.14E-97	2.25E-95
10777	2950.3	124	19	1.27	0.2	-2.666756592	Down	7.54E-21	1.19E-19
LXLOC_007737	1971	24	4	0.38	0.06	-2.662965013	Down	8.12E-05	0.000368039
MXLOC_003438	7551	202.64	33.77	0.82	0.13	-2.657112286	Down	6.80E-32	1.56E-30
117154	2425	53.29	8.76	0.69	0.11	-2.649092838	Down	1.00E-09	8.07E-09
2201	10724	88	14	0.25	0.04	-2.64385619	Down	6.75E-15	7.87E-14
158219	3185.35	71.82	11.48	0.68	0.11	-2.628031223	Down	1.94E-12	1.93E-11
6696	1594.01	72	12	1.42	0.23	-2.626185163	Down	4.00E-12	3.88E-11
n341517	2336	328.03	54.15	4.38	0.71	-2.62503994	Down	1.37E-50	4.91E-49
MXLOC_008995	6325	75.25	12.44	0.37	0.06	-2.624490865	Down	7.49E-13	7.69E-12
9858	4932	186.85	31.45	1.17	0.19	-2.622437206	Down	3.79E-29	8.07E-28
n341786	651	132	22	6.72	1.1	-2.610957709	Down	4.17E-21	6.65E-20
130940	1665.23	38	6	0.67	0.11	-2.606657572	Down	3.70E-07	2.31E-06
283450	15336	728.43	125.12	1.45	0.24	-2.594946589	Down	1.99E-107	1.57E-105
n338183	2975	207.98	35.67	2.17	0.36	-2.591626231	Down	5.37E-32	1.24E-30
n342100	2920	90	15	0.96	0.16	-2.584962501	Down	7.93E-15	9.18E-14
MXLOC_000022	3240	593.28	101.17	5.67	0.95	-2.577349317	Down	2.84E-88	1.81E-86
56884	4828.93	6631	1139	42.4	7.11	-2.5761428	Down	0	0
LXLOC_016651	8587	416.83	70.65	1.49	0.25	-2.575312331	Down	6.79E-63	3.00E-61
3222	1613	741	127	14.47	2.43	-2.574036703	Down	1.94E-109	1.58E-107
LXLOC_037782	6677	204	35	0.94	0.16	-2.554588852	Down	2.79E-31	6.30E-30
n341156	1852	28	5	0.47	0.08	-2.554588852	Down	2.92E-05	0.00014229
LXLOC_012531	336	23	4	2.46	0.42	-2.550197083	Down	0.00014253	0.000621929
55187	16329	562.67	100.92	1.05	0.18	-2.544320516	Down	1.80E-81	1.05E-79
LXLOC_004604	4576	2298	405	15.49	2.67	-2.536425497	Down	0	0
n367640	2160	59.95	10.81	0.87	0.15	-2.5360529	Down	4.49E-10	3.73E-09
4883	6813.03	442	83	2.07	0.37	-2.484033592	Down	3.37E-62	1.47E-60
MXLOC_031397	1139	221.29	40.29	6.21	1.11	-2.484033592	Down	1.16E-32	2.73E-31
n408887	1447	96.69	17.54	2.11	0.38	-2.473171675	Down	3.43E-15	4.08E-14
388662	6403	358	65	1.72	0.31	-2.472068444	Down	7.90E-52	2.90E-50
285267	3090.42	200.32	34	1.83	0.33	-2.471305719	Down	7.25E-31	1.63E-29
MXLOC_022237	3491	69.23	12.93	0.61	0.11	-2.471305719	Down	2.10E-11	1.95E-10
n342503	570	61.2	11.21	3.6	0.65	-2.469485283	Down	5.04E-10	4.17E-09
11174	7268	52.12	8.81	0.22	0.04	-2.459431619	Down	1.78E-09	1.39E-08
n384518	2839	80.43	15.12	0.88	0.16	-2.459431619	Down	1.88E-12	1.87E-11
n338097	1328	32.41	5.85	0.77	0.14	-2.459431619	Down	3.08E-06	1.72E-05
90161	4484.47	3209	596	22.05	4.01	-2.459104514	Down	0	0
2491	2563	274.1	51.73	3.33	0.61	-2.44864103	Down	2.11E-39	5.93E-38
283232	1590	30.23	5.57	0.6	0.11	-2.447458977	Down	9.55E-06	5.01E-05
3397	983	114	21	3.65	0.67	-2.445663463	Down	2.32E-17	3.12E-16
n339026	4065	149.72	28.18	1.14	0.21	-2.440572591	Down	5.61E-22	9.30E-21
8654	6772	167.71	31.24	0.76	0.14	-2.440572591	Down	1.19E-24	2.16E-23
114822	3715	325.24	60.95	2.71	0.5	-2.438292852	Down	1.08E-46	3.61E-45
445329	1397	85.52	16.44	1.94	0.36	-2.429987841	Down	4.07E-13	4.25E-12
MXLOC_007388	5659	683.09	129.58	3.71	0.69	-2.42675092	Down	8.45E-95	5.83E-93
LXLOC_005462	1238.26	52.19	9.93	1.34	0.25	-2.422233001	Down	6.31E-09	4.71E-08
54510	5906	62	11	0.32	0.06	-2.415037499	Down	2.92E-10	2.46E-09
LXLOC_015603	810	37	7	1.49	0.28	-2.411813598	Down	2.18E-06	1.25E-05
7422	3525.79	318.47	62.29	2.82	0.53	-2.411630898	Down	4.52E-44	1.41E-42
55137	4535	195	38	1.33	0.25	-2.411426246	Down	1.24E-27	2.50E-26
MXLOC_015269	2306	102.02	19.57	1.38	0.26	-2.408084739	Down	1.43E-15	1.75E-14
4916	3067.15	455	89	4.67	0.88	-2.407847121	Down	3.30E-62	1.44E-60
LXLOC_026846	3697.65	1720.55	300.87	13.04	2.46	-2.406213649	Down	8.89E-249	1.72E-246

56136	3937.98	753.05	139.44	5.67	1.07	-2.405737939	Down	5.76E-106	4.47E-104
5090	2873.98	8142.14	1573.86	87.96	16.64	-2.402192171	Down	0	0
4599	3098.16	121	23.11	1.21	0.23	-2.395301281	Down	5.54E-18	7.67E-17
n371399	2445	32.76	6.53	0.42	0.08	-2.392317423	Down	1.04E-05	5.45E-05
196740	5405.35	371.66	64.34	1.89	0.36	-2.392317423	Down	2.03E-55	8.02E-54
LXLOC_031747	4288	5025.18	1000.56	36.98	7.05	-2.391050062	Down	0	0
n379246	763	286.2	55.6	12.27	2.34	-2.390554814	Down	3.58E-40	1.02E-38
n338129	1711	48.37	9.3	0.89	0.17	-2.38827059	Down	5.63E-08	3.84E-07
n386202	1627	75.47	15.03	1.46	0.28	-2.382469637	Down	2.70E-11	2.48E-10
LXLOC_004870	1636	165.42	32.17	3.18	0.61	-2.382145618	Down	1.10E-23	1.93E-22
84889	2255.19	128	25	1.77	0.34	-2.380142709	Down	1.31E-18	1.87E-17
MXLOC_020356	10720	1346.95	266.23	3.85	0.74	-2.37926127	Down	3.22E-179	4.27E-177
4914	2655	44	9	0.52	0.1	-2.378511623	Down	4.80E-07	2.96E-06
MXLOC_006809	3676	30.65	5.88	0.26	0.05	-2.378511623	Down	9.55E-06	5.00E-05
10678	2749	201.43	39.46	2.28	0.44	-2.373458396	Down	1.61E-28	3.36E-27
1890	1588.58	31.45	6.36	0.62	0.12	-2.36923381	Down	1.80E-05	9.07E-05
LXLOC_035352	8732	896.02	177.69	3.15	0.61	-2.368470681	Down	5.83E-120	5.22E-118
445	1786	76	15	1.34	0.26	-2.365649472	Down	1.59E-11	1.49E-10
MXLOC_015703	5728	1775	354	9.53	1.86	-2.357173593	Down	3.69E-234	6.64E-232
23213	5604.7	465	94	2.55	0.5	-2.350497247	Down	4.58E-62	1.99E-60
2561	7315.16	642	128	2.7	0.53	-2.348895142	Down	6.84E-86	4.25E-84
LXLOC_015663	1186.35	913	183	24.53	4.82	-2.347442182	Down	6.35E-121	5.79E-119
n342341	2930	163.17	32.52	1.73	0.34	-2.347165386	Down	3.16E-23	5.45E-22
n381552	2743	278	56	3.15	0.62	-2.345011708	Down	7.44E-38	2.01E-36
n325768	435	24.29	4.9	1.93	0.38	-2.344529524	Down	8.12E-05	0.000367992
n339255	1998	48.7	9.94	0.76	0.15	-2.341036918	Down	5.63E-08	3.84E-07
n410905	775	955.59	192.62	40.29	7.96	-2.33958147	Down	4.02E-126	3.87E-124
n408280	2116	125.97	25.35	1.86	0.37	-2.329705445	Down	6.36E-18	8.79E-17
n409627	274	255.27	51.71	34.89	6.95	-2.327728715	Down	4.98E-35	1.25E-33
LXLOC_025545	925	42.83	8.85	1.5	0.3	-2.321928095	Down	4.57E-07	2.83E-06
10125	5025	294	60	1.8	0.36	-2.321928095	Down	1.43E-39	4.02E-38
MXLOC_000098	6308	287.48	57.77	1.4	0.28	-2.321928095	Down	2.02E-39	5.68E-38
6678	3141	132	27	1.3	0.26	-2.321928095	Down	1.47E-18	2.09E-17
170691	6331	31	7.04	0.15	0.03	-2.321928095	Down	5.24E-05	0.000245014
n407810	2451	27.64	5.31	0.35	0.07	-2.321928095	Down	5.06E-05	0.00023779
n409416	4187	67.9	14.4	0.5	0.1	-2.321928095	Down	6.07E-10	4.98E-09
n384104	1842	383.64	78.3	6.53	1.31	-2.31751618	Down	3.87E-51	1.40E-49
n344768	2625	147.08	30.3	1.74	0.35	-2.313660479	Down	1.54E-20	2.39E-19
n407934	1737	82.4	16.74	1.49	0.3	-2.312277925	Down	2.02E-12	2.01E-11
MXLOC_007177	2712	341.66	70.26	3.92	0.79	-2.310929096	Down	1.90E-45	6.12E-44
MXLOC_030828	3756	143.95	30	1.19	0.24	-2.309855263	Down	1.23E-19	1.85E-18
n378209	892	62.69	13.04	2.28	0.46	-2.309328058	Down	2.83E-09	2.17E-08
MXLOC_013720	1944	58.12	12.28	0.94	0.19	-2.306661338	Down	7.81E-09	5.78E-08
5915	3020.56	1958	402	19.97	4.04	-2.305407135	Down	1.34E-252	2.61E-250
MXLOC_035398	4118	947.6	196.65	7.11	1.44	-2.303780748	Down	2.03E-122	1.89E-120
1789	3959.27	70.89	14.34	0.54	0.11	-2.295455884	Down	1.25E-10	1.09E-09
7265	1386	240.12	50.11	5.49	1.12	-2.293307417	Down	4.13E-32	9.54E-31
MXLOC_025006	2909.66	46.47	9.93	0.49	0.1	-2.292781749	Down	1.65E-07	1.07E-06
283726	3131	138	29	1.37	0.28	-2.290677161	Down	5.62E-19	8.21E-18
3005	2330	3983	837	53.31	10.96	-2.282158384	Down	0	0
n377923	3075	34.07	7.41	0.34	0.07	-2.280107919	Down	1.09E-05	5.66E-05
n386710	904	211.38	44.64	7.56	1.56	-2.276840205	Down	2.14E-28	4.46E-27
64427	2929	104.57	22.36	1.11	0.23	-2.27085391	Down	1.40E-14	1.61E-13
n409669	4390	363.46	77.47	2.55	0.53	-2.266432982	Down	4.08E-47	1.37E-45
79412	2005.41	116.89	24.04	1.78	0.37	-2.266280065	Down	2.36E-16	3.03E-15
9317	3738	29	6	0.24	0.05	-2.263034406	Down	5.26E-05	0.00024558
132884	4410	35	7	0.24	0.05	-2.263034406	Down	6.39E-06	3.44E-05
n406201	1379	245.89	52.63	5.65	1.18	-2.259464008	Down	2.65E-32	6.18E-31
n405570	5115	658.7	140.65	3.97	0.83	-2.257955766	Down	7.78E-84	4.67E-82
3119	1224	33	7	0.86	0.18	-2.256339753	Down	1.85E-05	9.29E-05

n374593	800	25.68	5.56	1.05	0.22	-2.254813899	Down	0.000150331	0.00065154
n365663	1593	41	9	0.81	0.17	-2.252387162	Down	2.31E-06	1.32E-05
LXLOC_025711	6078.49	1244.53	270.08	6.37	1.34	-2.249060372	Down	1.90E-154	2.20E-152
145773	3468	128	27	1.14	0.24	-2.247927513	Down	1.17E-17	1.60E-16
815	4871	120	26	0.76	0.16	-2.247927513	Down	2.51E-16	3.21E-15
100526835	2782	52.41	11	0.57	0.12	-2.247927513	Down	6.18E-08	4.19E-07
MXLOC_033567	4636	155.05	34.34	1.04	0.22	-2.2410081	Down	1.74E-20	2.70E-19
n381378	2031	119.18	25.8	1.84	0.39	-2.238159737	Down	1.46E-16	1.88E-15
9586	8343.42	652	143	2.4	0.51	-2.234465254	Down	3.83E-81	2.21E-79
4839	2661	188.8	41.18	2.21	0.47	-2.233313708	Down	1.22E-24	2.20E-23
429	2490	49	11	0.61	0.13	-2.230297619	Down	2.92E-07	1.85E-06
10082	7103	2924	638	12.64	2.7	-2.226965151	Down	0	0
119391	827	74.71	15.26	2.76	0.59	-2.225881407	Down	4.58E-11	4.12E-10
389692	2373	2017	443	26.5	5.69	-2.219491802	Down	7.46E-247	1.43E-244
LXLOC_024794	3023	54.21	13.99	0.65	0.14	-2.215012891	Down	1.68E-07	1.09E-06
LXLOC_015662	893	795	175	28.82	6.21	-2.214405162	Down	2.89E-98	2.10E-96
57493	9156	151	33	0.51	0.11	-2.212993723	Down	4.76E-20	7.27E-19
n407850	2285	1269.35	279.96	17.33	3.74	-2.212161479	Down	6.66E-156	7.81E-154
MXLOC_013382	1333	291.9	64.51	6.95	1.5	-2.212050477	Down	4.68E-37	1.23E-35
5179	1291.5	460	98	10.92	2.36	-2.210114092	Down	4.56E-59	1.90E-57
374395	1043	50.94	11.38	1.57	0.34	-2.207157908	Down	1.74E-07	1.13E-06
23105	5382	757	168	4.33	0.94	-2.203634363	Down	4.80E-93	3.26E-91
467	2229.53	288.09	64.22	4.05	0.88	-2.202346479	Down	2.14E-36	5.55E-35
8801	2354	35	8	0.46	0.1	-2.201633861	Down	1.83E-05	9.24E-05
n342899	3492	51.85	11	0.46	0.1	-2.201633861	Down	1.04E-07	6.89E-07
MXLOC_012154	8389	62.44	12.61	0.23	0.05	-2.201633861	Down	9.39E-10	7.56E-09
n382405	4150	92.21	20.22	0.69	0.15	-2.201633861	Down	8.30E-13	8.49E-12
n409454	752	26.45	5.78	1.15	0.25	-2.201633861	Down	8.75E-05	0.000394521
MXLOC_029818	8508	345.18	77	1.24	0.27	-2.199308808	Down	3.91E-43	1.19E-41
55959	3957.4	3829.22	853.16	29.92	6.52	-2.198166306	Down	0	0
222235	2923.26	140.87	33.05	1.56	0.34	-2.197939378	Down	1.21E-17	1.66E-16
MXLOC_026479	11259	1648.71	366.8	4.49	0.98	-2.195861791	Down	3.00E-200	4.54E-198
MXLOC_002714	2505	30.99	7.31	0.41	0.09	-2.187627003	Down	8.77E-05	0.000395381
MXLOC_007381	13528	222.79	47.53	0.5	0.11	-2.184424571	Down	1.81E-29	3.89E-28
10251	9038	174.22	39.03	0.59	0.13	-2.182203331	Down	1.91E-22	3.21E-21
400745	3834	353	81	2.9	0.64	-2.17990909	Down	3.97E-43	1.21E-41
3218	1823	325.98	73.43	5.61	1.24	-2.17766065	Down	1.60E-40	4.59E-39
n377857	430	196.96	44.53	15.86	3.52	-2.171745437	Down	4.59E-25	8.46E-24
n407652	6007	34.99	8.86	0.18	0.04	-2.169925001	Down	3.06E-05	0.00014867
MXLOC_004536	7816.49	903.5	206.77	3.55	0.79	-2.167894466	Down	3.53E-108	2.83E-106
57507	5645	2155.28	490.87	11.75	2.62	-2.16502204	Down	3.18E-256	6.38E-254
n378149	893	31	7	1.12	0.25	-2.163498732	Down	5.24E-05	0.000244982
MXLOC_030023	5294	229.72	51.89	1.34	0.3	-2.159198595	Down	3.28E-29	7.00E-28
389206	8808.33	2352.29	538.2	8.17	1.83	-2.15849243	Down	4.57E-278	9.91E-276
n376102	2245	141.28	31.99	1.96	0.44	-2.155278225	Down	9.84E-19	1.42E-17
LXLOC_003974	2454	77.14	17.81	0.98	0.22	-2.155278225	Down	8.10E-11	7.15E-10
4664	4469	199.46	45.35	1.38	0.31	-2.154328146	Down	2.78E-25	5.18E-24
116159	3079	141	32	1.42	0.32	-2.14974712	Down	2.73E-18	3.85E-17
n337810	1764	35	8	0.62	0.14	-2.146841388	Down	1.83E-05	9.24E-05
n405876	4202	41.59	9.85	0.31	0.07	-2.146841388	Down	2.31E-06	1.32E-05
MXLOC_024971	6707	306.9	71.76	1.41	0.32	-2.139551352	Down	2.92E-37	7.71E-36
390	2683	4505	1045	52.21	11.85	-2.1394391	Down	0	0
284	4338	62	15	0.44	0.1	-2.137503524	Down	2.15E-08	1.53E-07
n410123	2094	182.26	42.16	2.72	0.62	-2.133266531	Down	6.98E-23	1.19E-21
MXLOC_017534	6491	509.69	117.75	2.41	0.55	-2.131529623	Down	2.31E-61	9.96E-60
MXLOC_031479	1454.17	836.74	194.41	18.09	4.14	-2.127489735	Down	7.23E-99	5.28E-97
9232	712	30	7	1.39	0.32	-2.118941073	Down	8.77E-05	0.000395431
n381174	1201	39.06	9.13	1.04	0.24	-2.115477217	Down	6.45E-06	3.47E-05
22943	1805	77	18	1.34	0.31	-2.11189288	Down	2.22E-10	1.89E-09
n409327	3495	122.34	28.99	1.08	0.25	-2.111031312	Down	7.01E-16	8.72E-15

100131827	3068	93.98	21.87	0.95	0.22	-2.11042399	Down	1.39E-12	1.40E-11
n381272	4462	125	29	0.86	0.2	-2.10433666	Down	4.21E-16	5.31E-15
613212	1683	46	11	0.86	0.2	-2.10433666	Down	1.34E-06	7.84E-06
4257	923	38	9	1.33	0.31	-2.101086125	Down	1.07E-05	5.58E-05
n383807	3969	38.85	8.67	0.3	0.07	-2.099535674	Down	3.86E-06	2.14E-05
n385841	3182	79.55	19.2	0.77	0.18	-2.096861539	Down	2.17E-10	1.85E-09
10659	8395.35	671.45	162.71	2.48	0.58	-2.096215315	Down	4.73E-77	2.55E-75
50487	2575	39	9	0.47	0.11	-2.095157233	Down	6.45E-06	3.47E-05
8553	3035	33	8	0.34	0.08	-2.087462841	Down	5.07E-05	0.000238146
LXLOC_017500	1294	76.14	18.45	1.87	0.44	-2.087462841	Down	3.66E-10	3.06E-09
2264	3026	117	28	1.19	0.28	-2.087462841	Down	8.48E-15	9.81E-14
MXLOC_021116	2059	167.88	40.43	2.55	0.6	-2.087462841	Down	1.70E-20	2.63E-19
n409708	4651	102.3	24.39	0.68	0.16	-2.087462841	Down	2.99E-13	3.16E-12
84966	1944	1930	467	31.1	7.37	-2.077178056	Down	1.04E-217	1.69E-215
n363648	969	40.71	9.96	1.35	0.32	-2.076815597	Down	3.87E-06	2.14E-05
83857	8753.19	1309.04	317.41	4.59	1.09	-2.074166019	Down	3.55E-148	3.90E-146
MXLOC_008260	5158	134.3	32.14	0.8	0.19	-2.074000581	Down	9.00E-17	1.18E-15
387914	2889	59	14	0.63	0.15	-2.070389328	Down	3.64E-08	2.53E-07
n381446	2876	38.51	9.55	0.42	0.1	-2.070389328	Down	1.07E-05	5.58E-05
MXLOC_016890	1647	36.9	9.02	0.71	0.17	-2.062284278	Down	2.92E-05	0.000142563
n378128	2150	34.2	8.78	0.5	0.12	-2.058893689	Down	3.06E-05	0.000148691
MXLOC_023107	2200	34.91	8.93	0.5	0.12	-2.058893689	Down	3.06E-05	0.00014865
5139	4124	72	18	0.54	0.13	-2.054447784	Down	2.64E-09	2.04E-08
353189	5334	144	36	0.83	0.2	-2.053111336	Down	2.97E-17	3.98E-16
91319	2606.66	192	46	2.24	0.54	-2.05246742	Down	2.44E-23	4.22E-22
4636	661	39.78	9.84	1.99	0.48	-2.05166212	Down	6.45E-06	3.46E-05
165082	4224.24	33	9.81	0.29	0.07	-2.050626073	Down	0.000126764	0.000557889
58158	3956	79	19	0.62	0.15	-2.047305715	Down	2.17E-10	1.85E-09
LXLOC_030526	2910	146.71	36.35	1.57	0.38	-2.046693235	Down	1.12E-17	1.54E-16
9671	4636	440	109	2.93	0.71	-2.045009735	Down	5.61E-50	1.99E-48
n339302	2159	396	98	5.73	1.39	-2.043450256	Down	3.47E-45	1.11E-43
246213	3983	175	44	1.36	0.33	-2.043068722	Down	1.49E-20	2.32E-19
MXLOC_021553	6062	1682	416.28	8.53	2.07	-2.042914974	Down	2.73E-186	3.82E-184
57451	9645	1446	361	4.6	1.12	-2.038135129	Down	4.64E-159	5.57E-157
5874	1336	107.1	26.5	2.54	0.62	-2.034488376	Down	1.73E-13	1.86E-12
MXLOC_016472	4291	130.23	32.52	0.94	0.23	-2.031026896	Down	6.44E-16	8.04E-15
MXLOC_016229	7898	409.31	101.45	1.59	0.39	-2.027480736	Down	1.05E-46	3.51E-45
n341308	2109	71.56	17.84	1.06	0.26	-2.027480736	Down	1.68E-09	1.31E-08
3232	2299	1685.59	422.65	22.87	5.61	-2.02738369	Down	1.72E-184	2.38E-182
n339208	3148	70	18	0.69	0.17	-2.021061616	Down	7.00E-09	5.20E-08
n1845	2095	67.75	17.02	1.01	0.25	-2.014355293	Down	1.20E-08	8.74E-08
MXLOC_035968	7614.01	278.87	70.74	1.13	0.28	-2.01282404	Down	1.03E-31	2.35E-30
n373958	6096	31.32	8.55	0.16	0.04	-2	Down	0.000137365	0.000600801
LXLOC_028963	3618	126	32	1.08	0.27	-2	Down	4.50E-15	5.31E-14
1293	8760	12	0	0.04	0.01	-2	Down	0.000210376	0.000889713
7087	3002	147	38	1.52	0.38	-2	Down	4.38E-17	5.80E-16
79026	18815	51	15	0.08	0.02	-2	Down	4.19E-06	2.30E-05
n424067	22743	2935.87	757.08	3.94	0.99	-1.992695199	Down	0	0
LXLOC_003672	661	39	10	1.95	0.49	-1.99262047	Down	1.68E-05	8.50E-05
n409298	2873	117.58	30.57	1.27	0.32	-1.988684687	Down	5.54E-14	6.11E-13
256714	3900	153.42	38.16	1.19	0.3	-1.987927168	Down	2.42E-18	3.42E-17
n409089	2196	75.34	19.7	1.07	0.27	-1.986579484	Down	1.55E-09	1.22E-08
MXLOC_009844	2657	283.3	73.42	3.32	0.84	-1.982722009	Down	1.45E-31	3.29E-30
n406943	1983	50	13	0.79	0.2	-1.981852653	Down	1.21E-06	7.13E-06
79750	1812	269.25	69.54	4.66	1.18	-1.981543095	Down	3.10E-30	6.80E-29
LXLOC_027049	3982	81	21	0.63	0.16	-1.977279923	Down	5.27E-10	4.35E-09
MXLOC_035470	1051	62	16	1.89	0.48	-1.977279923	Down	5.46E-08	3.73E-07
n407869	1148	65.24	16.73	1.81	0.46	-1.976283931	Down	1.26E-08	9.14E-08
138046	2623.23	959.74	277.38	12.66	3.22	-1.975144811	Down	6.41E-92	4.26E-90
n340549	595	262	68	14.7	3.74	-1.97470598	Down	3.55E-29	7.55E-28

n386398	3165	252.02	65.35	2.47	0.63	-1.971087308	Down		2.79E-28	5.78E-27
LXLOC_020924	10087	438.72	116.06	1.33	0.34	-1.967819594	Down		8.48E-47	2.83E-45
10389	4198	111.54	29.51	0.82	0.21	-1.965234582	Down		3.99E-13	4.18E-12
MXLOC_030899	9461.22	494.52	130.91	1.6	0.41	-1.96437609	Down		6.92E-53	2.61E-51
n385795	2703	91.45	24	1.05	0.27	-1.959358016	Down		6.44E-11	5.74E-10
MXLOC_010637	9774	447.92	117.94	1.4	0.36	-1.959358016	Down		2.94E-48	1.01E-46
MXLOC_015962	9616	1195.79	314.36	3.81	0.98	-1.958937343	Down		1.34E-125	1.28E-123
10226	2340	128	34	1.71	0.44	-1.958420896	Down		1.05E-14	1.21E-13
n345506	3597	36	9	0.31	0.08	-1.95419631	Down		2.92E-05	0.000142543
8787	2238.04	123.48	37.9	2.01	0.52	-1.950611973	Down		1.29E-12	1.30E-11
7372	6738	110.1	29.45	0.5	0.13	-1.943416472	Down		6.43E-13	6.65E-12
54754	2561	40.94	10.92	0.5	0.13	-1.943416472	Down		1.03E-05	5.35E-05
n381766	4375	1731	461	12.21	3.18	-1.94096453	Down		1.03E-178	1.36E-176
n385632	869	75	20	2.8	0.73	-1.939458458	Down		3.82E-09	2.90E-08
2908	6278.25	397.75	105	1.91	0.5	-1.933572638	Down		1.26E-42	3.77E-41
8602	2918	39.48	10.54	0.42	0.11	-1.932885804	Down		1.68E-05	8.50E-05
91851	4066.49	76	20	0.57	0.15	-1.925999419	Down		2.37E-09	1.83E-08
261734	5004	240.36	64.47	1.48	0.39	-1.924051147	Down		3.39E-26	6.50E-25
1490	2344	83	22	1.1	0.29	-1.923378718	Down		4.95E-10	4.10E-09
n342280	1903	47.8	13.28	0.79	0.21	-1.911463325	Down		5.09E-06	2.77E-05
n382921	3552	90.91	24.84	0.79	0.21	-1.911463325	Down		1.04E-10	9.08E-10
MXLOC_015370	5894	266.78	72.68	1.39	0.37	-1.909487707	Down		1.84E-28	3.83E-27
26115	11747	503.86	137.53	1.31	0.35	-1.904139985	Down		4.64E-52	1.72E-50
8366	357	404	110	40.29	10.78	-1.902064627	Down		3.72E-42	1.11E-40
3790	2330	53	14.19	0.71	0.19	-1.901819606	Down		6.96E-07	4.22E-06
10579	4074.01	52.12	14.44	0.41	0.11	-1.898120386	Down		1.12E-06	6.67E-06
MXLOC_013406	3233	128.21	34.92	1.23	0.33	-1.898120386	Down		1.05E-14	1.21E-13
65975	2707	309.58	85.74	3.56	0.96	-1.89077093	Down		2.67E-32	6.21E-31
25837	1624	84	23	1.63	0.44	-1.889296536	Down		7.39E-10	6.01E-09
MXLOC_031384	3400.07	241.09	64.31	2.11	0.57	-1.888209175	Down		2.12E-26	4.08E-25
6925	8320	99.94	27.78	0.37	0.1	-1.887525271	Down		2.03E-11	1.88E-10
1305	2921	35	10	0.37	0.1	-1.887525271	Down		0.000115131	0.00051019
n407051	853	41.87	11.62	1.59	0.43	-1.886618201	Down		1.56E-05	7.94E-05
2027	1535.42	127	35.63	2.66	0.72	-1.885357434	Down		4.00E-14	4.45E-13
253738	4361	1090.36	301.89	7.72	2.09	-1.885097905	Down		3.18E-109	2.58E-107
51085	3278	51	14	0.48	0.13	-1.884522783	Down		1.81E-06	1.05E-05
MXLOC_008179	2683	92.47	25.2	1.07	0.29	-1.883485991	Down		9.66E-11	8.47E-10
3233	1298	1070.18	296.58	26.19	7.1	-1.88312513	Down		4.63E-107	3.63E-105
n374482	2987	67	19	0.7	0.19	-1.881355504	Down		6.97E-08	4.72E-07
8091	3347.1	124	28	0.92	0.25	-1.879705766	Down		2.56E-16	3.27E-15
7277	1478	43	12	0.92	0.25	-1.879705766	Down		1.43E-05	7.34E-05
122481	2648	116	32	1.36	0.37	-1.878009476	Down		5.21E-13	5.41E-12
n406279	388	47	13	4.26	1.16	-1.876728625	Down		5.09E-06	2.77E-05
54796	12926	93	26	0.22	0.06	-1.874469118	Down		1.42E-10	1.24E-09
53904	5784	41	12	0.22	0.06	-1.874469118	Down		3.66E-05	0.000175531
5784	13443	48.78	13	0.11	0.03	-1.874469118	Down		3.16E-06	1.77E-05
54221	1888	33	9	0.55	0.15	-1.874469118	Down		0.000126764	0.000557959
84171	3657	39	11	0.33	0.09	-1.874469118	Down		4.06E-05	0.000193327
LXLOC_021764	3185	79.48	24.66	0.84	0.23	-1.868755467	Down		1.68E-08	1.21E-07
n339334	1624	52.54	14.53	1.02	0.28	-1.86507042	Down		1.12E-06	6.67E-06
342667	3277	150.07	41.76	1.42	0.39	-1.864344901	Down		1.50E-16	1.93E-15
n387192	5480	70.41	20.08	0.4	0.11	-1.862496476	Down		4.00E-08	2.77E-07
n332789	410	37.66	10.55	3.2	0.88	-1.862496476	Down		4.44E-05	0.000210424
7056	4032	38	11	0.29	0.08	-1.857980995	Down		6.49E-05	0.000299563
LXLOC_008865	4531	196.48	56	1.34	0.37	-1.856635825	Down		2.73E-20	4.22E-19
n342403	2711	41	12	0.47	0.13	-1.854149134	Down		3.66E-05	0.000175508
55515	2901	768	218	8.22	2.28	-1.85010457	Down		2.45E-75	1.29E-73
11005	3639	92	26	0.79	0.22	-1.84434913	Down		2.27E-10	1.93E-09
797	1031	144	41	4.48	1.25	-1.841570637	Down		2.41E-15	2.90E-14
MXLOC_011727	4335	871.1	248.31	6.2	1.73	-1.841496178	Down		6.30E-85	3.87E-83

n409326	3263	90.29	26.25	0.86	0.24	-1.841302254	Down	5.70E-10	4.69E-09
n380486	1038	127.48	36.41	3.94	1.1	-1.840692106	Down	9.31E-14	1.01E-12
n406552	1016	134.78	38.53	4.26	1.19	-1.839891857	Down	1.97E-14	2.23E-13
n364575	1156	161.95	46.23	4.47	1.25	-1.838346737	Down	6.28E-17	8.26E-16
MXLOC_023320	3566.26	86.3	25.16	0.75	0.21	-1.836501268	Down	1.57E-09	1.23E-08
800	4291	37.04	10.08	0.25	0.07	-1.836501268	Down	4.44E-05	0.000210453
642938	4407	616.4	176.38	4.32	1.21	-1.836024265	Down	2.30E-60	9.78E-59
n385823	1662	531.65	152.79	10.07	2.83	-1.831189725	Down	3.61E-52	1.34E-50
n411702	1942	79.19	22.71	1.28	0.36	-1.830074999	Down	3.27E-09	2.50E-08
n384808	1570	51.11	15	1.03	0.29	-1.828519532	Down	4.19E-06	2.30E-05
9322	2011	91	26	1.42	0.4	-1.827819025	Down	3.60E-10	3.01E-09
9120	3860.01	273	79	2.2	0.62	-1.827163403	Down	2.63E-27	5.24E-26
9675	3888	146.59	41.84	1.17	0.33	-1.8259706	Down	9.60E-16	1.19E-14
79953	2437	108.12	31.57	1.38	0.39	-1.823122238	Down	9.11E-12	8.64E-11
1793	6751	302.64	86.68	1.38	0.39	-1.823122238	Down	1.55E-30	3.43E-29
n342184	2069	100.7	29.06	1.52	0.43	-1.821662759	Down	6.86E-11	6.10E-10
n384703	3380	57.35	16.3	0.53	0.15	-1.821029859	Down	5.92E-07	3.63E-06
n410583	4060	69.21	20.07	0.53	0.15	-1.821029859	Down	6.34E-08	4.30E-07
LXLOC_001386	4472	540.3	156.37	3.73	1.06	-1.815111366	Down	1.61E-52	6.04E-51
4208	6282.24	59	17.65	0.28	0.08	-1.807354922	Down	5.39E-07	3.31E-06
MXLOC_029572	2575	115.46	33.93	1.4	0.4	-1.807354922	Down	1.92E-12	1.91E-11
n382707	3416	54.54	15.79	0.49	0.14	-1.807354922	Down	1.04E-06	6.17E-06
MXLOC_020690	9477	635.73	186.67	2.06	0.59	-1.803857478	Down	1.48E-60	6.30E-59
3398	1364	458	134	10.64	3.05	-1.802617003	Down	3.32E-44	1.04E-42
57540	5214	507.99	149	3	0.86	-1.802553936	Down	1.60E-48	5.53E-47
5458	3141	254	75	2.51	0.72	-1.801618553	Down	5.47E-25	1.01E-23
256130	3364	798.82	234.48	7.35	2.11	-1.800501251	Down	1.47E-75	7.74E-74
MXLOC_002719	10079	949.18	278.07	2.89	0.83	-1.799886251	Down	1.30E-89	8.43E-88
n367641	701	34	10.01	1.6	0.46	-1.798366139	Down	0.000183917	0.000784392
8613	3308	163	48	1.53	0.44	-1.797956224	Down	1.29E-16	1.67E-15
51231	1950	36.79	11.1	0.59	0.17	-1.795180208	Down	0.000163445	0.000704313
727866	1701	91.78	27.26	1.7	0.49	-1.794681092	Down	8.13E-10	6.58E-09
3428	2734	40	12	0.45	0.13	-1.791413378	Down	5.81E-05	0.000269612
8350	469	379	112	27.68	8.03	-1.78537205	Down	1.78E-36	4.63E-35
9729	8124	81.86	23.55	0.31	0.09	-1.784271309	Down	3.00E-09	2.30E-08
MXLOC_005630	10928	109.78	34.44	0.31	0.09	-1.784271309	Down	6.28E-11	5.60E-10
54212	1889	145	43	2.41	0.7	-1.783606319	Down	7.80E-15	9.04E-14
n380073	2455	267.76	79.54	3.4	0.99	-1.780034316	Down	3.93E-26	7.51E-25
n340674	2982	448.45	133.15	4.67	1.36	-1.779815898	Down	1.32E-42	3.94E-41
124540	1640.94	764	232	14.97	4.36	-1.779674181	Down	1.10E-69	5.41E-68
n340993	2973	2062.33	616.82	21.53	6.3	-1.772924586	Down	2.34E-188	3.31E-186
79828	8135	370.03	111.77	1.4	0.41	-1.771731012	Down	4.46E-35	1.12E-33
n406963	3117	274.6	82.47	2.73	0.8	-1.770829046	Down	1.86E-26	3.60E-25
n367346	555	1548	463	93.77	27.5	-1.769694814	Down	1.05E-141	1.11E-139
9388	4141	1915	574	14.28	4.19	-1.76897383	Down	2.04E-174	2.65E-172
n410038	3414	138.6	41.28	1.26	0.37	-1.767826558	Down	3.68E-14	4.11E-13
3762	2912	63.61	19.52	0.68	0.2	-1.765534746	Down	4.37E-07	2.70E-06
56905	4427	171	52	1.19	0.35	-1.765534746	Down	8.55E-17	1.12E-15
3738	3346	103.11	30.6	0.95	0.28	-1.762500686	Down	3.94E-11	3.56E-10
5173	2574	191	57	2.27	0.67	-1.760459297	Down	5.89E-19	8.59E-18
LXLOC_005101	1404	66	20	1.49	0.44	-1.759736902	Down	2.49E-07	1.59E-06
MXLOC_010603	4780	341.26	103.11	2.2	0.65	-1.7589919	Down	3.24E-32	7.51E-31
n386214	1112	53	16	1.52	0.45	-1.756074417	Down	3.75E-06	2.08E-05
n383773	1782	92	28	1.62	0.48	-1.754887502	Down	1.14E-09	9.13E-09
LXLOC_031326	375	43	13	4.05	1.2	-1.754887502	Down	3.27E-05	0.000158464
n338668	1955	261	79	4.18	1.24	-1.753162822	Down	5.70E-25	1.05E-23
n407681	1032	149.43	45.24	4.65	1.38	-1.752562449	Down	6.34E-15	7.39E-14
57580	6633	80	25	0.37	0.11	-1.750021747	Down	2.33E-08	1.65E-07
3363	3258.55	989	301	9.41	2.8	-1.748767896	Down	1.90E-89	1.23E-87
161835	2813	213.2	64.97	2.35	0.7	-1.74723393	Down	8.08E-21	1.27E-19

9796	3267.81	303	94	2.92	0.87	-1.746881063	Down	5.68E-28	1.16E-26
347731	5609	917	279	5.03	1.5	-1.745595899	Down	4.25E-83	2.52E-81
2622	3185	186.48	57.02	1.81	0.54	-1.744958385	Down	5.43E-18	7.53E-17
MXLOC_001673	4922	320.98	98.68	2.01	0.6	-1.744161096	Down	7.07E-30	1.54E-28
94274	718	46	14	2.11	0.63	-1.743819265	Down	1.84E-05	9.28E-05
2045	6644	10865.48	3325.09	50.26	15.04	-1.740606104	Down	0	0
57569	6189	120	37	0.6	0.18	-1.736965594	Down	4.89E-12	4.72E-11
284805	4793	46.19	13.63	0.3	0.09	-1.736965594	Down	8.15E-06	4.32E-05
6261	15378.97	253	75	0.5	0.15	-1.736965594	Down	8.57E-25	1.56E-23
MXLOC_012278	2941	57.16	17.75	0.6	0.18	-1.736965594	Down	1.35E-06	7.92E-06
MXLOC_034348	4196	136.52	41.8	1	0.3	-1.736965594	Down	9.02E-14	9.82E-13
57619	11020	37	11	0.1	0.03	-1.736965594	Down	0.000103247	0.000461291
MXLOC_024552	14555	1597.75	491.46	3.36	1.01	-1.73410594	Down	1.26E-141	1.33E-139
54549	10722	432	134	1.23	0.37	-1.73306114	Down	3.34E-39	9.34E-38
MXLOC_023901	4142	164.85	50.56	1.23	0.37	-1.73306114	Down	4.01E-16	5.08E-15
n339965	1366	377.78	116.25	8.77	2.64	-1.732038913	Down	9.88E-35	2.46E-33
n381982	2100	62.74	19.38	0.93	0.28	-1.731803889	Down	6.85E-07	4.16E-06
25946	2336	62	19	0.83	0.25	-1.731183242	Down	6.85E-07	4.16E-06
n384210	1834	228.25	70.54	3.91	1.18	-1.728381748	Down	1.13E-21	1.85E-20
80139	2174	304	94	4.37	1.32	-1.72709535	Down	3.67E-28	7.56E-27
n342706	1625	387.57	119.49	7.51	2.27	-1.72612061	Down	1.23E-35	3.15E-34
51268	2412	61.07	19.2	0.79	0.24	-1.718818247	Down	1.07E-06	6.37E-06
83875	2912	117	36	1.25	0.38	-1.717856771	Down	8.53E-12	8.11E-11
LXLOC_035968	6441.42	46.06	14.73	0.23	0.07	-1.716207034	Down	1.84E-05	9.28E-05
51196	7765.35	59	19	0.23	0.07	-1.716207034	Down	2.59E-06	1.47E-05
MXLOC_031565	1578	152.62	47.75	3.05	0.93	-1.713506621	Down	7.92E-15	9.18E-14
n345694	1057	77.68	24.09	2.36	0.72	-1.712718048	Down	4.09E-08	2.82E-07
55683	5285	286.17	88.73	1.67	0.51	-1.711278951	Down	9.88E-27	1.93E-25
26034	6815.33	79	24.36	0.36	0.11	-1.710493383	Down	1.68E-08	1.20E-07
23554	2564	194	61	2.35	0.72	-1.706591945	Down	3.45E-18	4.82E-17
23657	9648	193.58	61.07	0.62	0.19	-1.706268797	Down	5.33E-18	7.39E-17
729440	1825	43.37	13.79	0.75	0.23	-1.705256734	Down	3.27E-05	0.000158442
n342406	1247	89.2	28.04	2.28	0.7	-1.703606997	Down	4.33E-09	3.27E-08
MXLOC_031900	3811.25	2670.09	854.14	22.06	6.78	-1.702075612	Down	1.17E-224	2.00E-222
25769	10851	46	15	0.13	0.04	-1.700439718	Down	3.96E-05	0.000189061
9935	3362	1399	441	12.89	3.97	-1.699041351	Down	9.91E-121	8.95E-119
n344649	1005	160.78	50.78	5.14	1.59	-1.692741594	Down	2.34E-15	2.81E-14
MXLOC_026370	2872	77.91	24.75	0.84	0.26	-1.691877705	Down	4.09E-08	2.82E-07
27164	4775	135.21	42.64	0.87	0.27	-1.688055994	Down	3.05E-13	3.22E-12
LXLOC_034204	2230	136	43	1.9	0.59	-1.687212559	Down	4.20E-13	4.38E-12
LXLOC_016259	1786.17	100.99	32.18	1.77	0.55	-1.686245837	Down	7.13E-10	5.80E-09
3215	1830	563	179	9.65	3	-1.685566442	Down	3.70E-49	1.29E-47
n375624	2591	39.67	13.11	0.48	0.15	-1.678071905	Down	0.000194975	0.000829049
n383969	1402	45.19	14.31	1.02	0.32	-1.672425342	Down	2.90E-05	0.000141469
100506658	5660.35	139.94	41.61	0.7	0.22	-1.669851398	Down	2.35E-14	2.65E-13
10085	2974	17815	5742	185.9	58.62	-1.665061897	Down	0	0
n379841	1369	7129	2302	165.05	52.17	-1.6616108	Down	0	0
n410468	3175	941.85	304.6	9.2	2.91	-1.660614708	Down	1.90E-79	1.06E-77
58513	2774	127.03	40.86	1.42	0.45	-1.657894023	Down	2.22E-12	2.20E-11
27443	5636	150.51	48.85	0.82	0.26	-1.657112286	Down	4.03E-14	4.48E-13
57496	8634	178.13	58	0.63	0.2	-1.655351829	Down	3.71E-16	4.71E-15
79838	4600.39	69.18	21.05	0.44	0.14	-1.652076697	Down	1.42E-07	9.26E-07
55203	6339	506	165	2.45	0.78	-1.65123572	Down	4.85E-43	1.47E-41
29970	2136.83	2181.4	770.32	34.61	11.02	-1.651064718	Down	6.41E-162	7.83E-160
4094	5962.95	558	198	3.14	1	-1.650764559	Down	1.78E-42	5.30E-41
56963	3113.03	995	319	9.76	3.11	-1.649966567	Down	1.11E-84	6.76E-83
108	6553	1390	453	6.52	2.08	-1.648288436	Down	3.12E-115	2.63E-113
80765	1316	38.91	12.73	0.94	0.3	-1.647698256	Down	0.000143904	0.000625529
9771	6622	52.94	17.84	0.25	0.08	-1.64385619	Down	1.26E-05	6.48E-05
MXLOC_019112	3302.85	1122.23	359.78	10.31	3.3	-1.643506403	Down	1.75E-95	1.21E-93

n410795	2510	102.85	33.65	1.28	0.41	-1.642447995	Down	6.28E-10	5.14E-09
n375269	1023	139.16	45.56	4.37	1.4	-1.642206453	Down	5.05E-13	5.26E-12
7025	3210	966	317	9.33	2.99	-1.641731597	Down	5.78E-80	3.27E-78
MXLOC_032130	2080	165.8	54.5	2.49	0.8	-1.638073837	Down	5.23E-15	6.14E-14
n364053	1185	52	17	1.4	0.45	-1.637429921	Down	1.26E-05	6.48E-05
81035	3134	145	48	1.43	0.46	-1.636309381	Down	3.43E-13	3.61E-12
23285	4556	1550.76	510.29	10.5	3.38	-1.635294176	Down	9.22E-127	8.92E-125
MXLOC_021832	7186	129.44	45.29	0.59	0.19	-1.634715536	Down	3.41E-11	3.10E-10
28316	2855	276.9	91.45	3.01	0.97	-1.633706835	Down	6.81E-24	1.21E-22
LXLOC_012379	4502	185.39	61.49	1.27	0.41	-1.631132682	Down	1.65E-16	2.12E-15
11037	5534	117	39	0.65	0.21	-1.63005039	Down	7.91E-11	6.99E-10
122953	3865.59	136	46	1.11	0.36	-1.624490865	Down	3.70E-12	3.60E-11
MXLOC_033191	6269	458.66	152.47	2.25	0.73	-1.623956632	Down	2.73E-38	7.44E-37
LXLOC_015650	6311	790.14	261.79	3.85	1.25	-1.622930351	Down	3.55E-65	1.64E-63
170689	5676	74	24	0.4	0.13	-1.621488377	Down	1.52E-07	9.95E-07
5167	7442	96.09	32	0.4	0.13	-1.621488377	Down	3.98E-09	3.02E-08
LXLOC_033416	1948	78.65	25.9	1.26	0.41	-1.619727919	Down	5.61E-08	3.83E-07
84680	2173	90	30	1.29	0.42	-1.618909833	Down	1.22E-08	8.89E-08
57214	7080	100	34	0.43	0.14	-1.618909833	Down	3.02E-09	2.31E-08
55970	4409	66	22	0.46	0.15	-1.61667136	Down	1.12E-06	6.65E-06
n375266	2340	177	59	2.36	0.77	-1.615856509	Down	1.16E-15	1.43E-14
646851	10322	329.37	109.49	0.98	0.32	-1.614709844	Down	5.10E-28	1.04E-26
MXLOC_034223	4834	393.98	130.94	2.51	0.82	-1.613991549	Down	3.20E-33	7.68E-32
222008	1286	562.56	188.02	13.9	4.55	-1.611146433	Down	3.77E-46	1.24E-44
MXLOC_010841	3224	60.11	20.07	0.58	0.19	-1.610053482	Down	3.49E-06	1.95E-05
114795	7578	144	47	0.58	0.19	-1.610053482	Down	2.55E-13	2.71E-12
n385532	1253	101.88	34.38	2.59	0.85	-1.607417352	Down	1.98E-09	1.54E-08
30011	4766	1001.02	336.64	6.49	2.13	-1.607365048	Down	1.75E-80	1.00E-78
MXLOC_007802	3548	76.4	26.29	0.67	0.22	-1.606657572	Down	2.72E-07	1.73E-06
2217	1629	41	14	0.79	0.26	-1.60334103	Down	0.000168196	0.000723374
n342053	1521	86.35	29.23	1.79	0.59	-1.601172728	Down	3.28E-08	2.30E-07
MXLOC_021823	4060	119.25	39.78	0.91	0.3	-1.600904045	Down	3.37E-11	3.07E-10
57178	7555	5601.82	1889.99	22.76	7.51	-1.599615745	Down	0	0
6256	5522	195.09	65.39	1.09	0.36	-1.598259323	Down	4.19E-17	5.55E-16
n382397	1934	150.33	50.9	2.44	0.81	-1.590887335	Down	1.72E-13	1.84E-12
25816	1969	165	56	2.62	0.87	-1.590479506	Down	2.17E-14	2.45E-13
83698	9471	676.39	229.76	2.19	0.73	-1.584962501	Down	2.70E-54	1.05E-52
n406573	6269	37.61	12.09	0.18	0.06	-1.584962501	Down	0.000224752	0.000943953
n410471	3352	262.63	89.47	2.43	0.81	-1.584962501	Down	5.87E-22	9.71E-21
n407026	3365	120.98	41.47	1.11	0.37	-1.584962501	Down	9.14E-11	8.03E-10
60484	1771	39	13	0.69	0.23	-1.584962501	Down	0.000194975	0.000829249
n408149	1477	39.14	13.37	0.84	0.28	-1.584962501	Down	0.000194975	0.000829149
63876	3730	439	150	3.64	1.22	-1.577057303	Down	1.83E-35	4.67E-34
MXLOC_031504	840	87.91	29.99	3.4	1.14	-1.576500922	Down	2.15E-08	1.53E-07
2977	2954	1289	442	13.54	4.54	-1.576463536	Down	2.64E-100	1.97E-98
n378431	1117	111.07	38.34	3.18	1.07	-1.571415969	Down	4.89E-10	4.05E-09
n372953	2869	85.27	29.74	0.92	0.31	-1.569365646	Down	5.01E-08	3.43E-07
3166	1896	145	50	2.4	0.81	-1.567040593	Down	1.39E-12	1.40E-11
339855	5704	417	144.42	2.25	0.76	-1.565853678	Down	2.57E-33	6.18E-32
90594	2550	235.47	80.91	2.87	0.97	-1.564994084	Down	8.44E-20	1.28E-18
9123	2049.81	163	56	2.48	0.84	-1.561878888	Down	4.98E-14	5.50E-13
54361	3905	380	132	3.01	1.02	-1.561194335	Down	2.83E-30	6.23E-29
n374607	7481	1247.07	433.93	5.12	1.74	-1.557056504	Down	1.67E-95	1.16E-93
MXLOC_017500	4696	218.63	76.07	1.44	0.49	-1.555215157	Down	6.05E-18	8.37E-17
n325579	2766	41.97	14.17	0.47	0.16	-1.554588852	Down	0.000168196	0.000723285
149076	3106	468.05	162.55	4.67	1.59	-1.554395784	Down	4.47E-37	1.18E-35
n339259	3721	161.98	56.35	1.35	0.46	-1.553253641	Down	1.14E-13	1.23E-12
9734	4279.74	2125.69	739.05	15.29	5.21	-1.553233129	Down	4.54E-161	5.53E-159
n365080	3048	43.69	15.41	0.44	0.15	-1.552541023	Down	0.00014442	0.000627537
1644	1955	110	38	1.76	0.6	-1.552541023	Down	7.41E-10	6.02E-09

MXLOC_028565	6856	593.73	207.26	2.66	0.91	-1.547487795	Down	5.03E-46	1.65E-44
n410553	6773	83.41	30.12	0.38	0.13	-1.547487795	Down	2.26E-07	1.45E-06
6238	3715.22	317.27	110.88	2.63	0.9	-1.547065893	Down	1.49E-25	2.80E-24
2170	1097	3561	1246	103.9	35.6	-1.545246508	Down	2.50E-266	5.16E-264
2690	4365	49.93	17	0.35	0.12	-1.544320516	Down	4.56E-05	0.000215654
MXLOC_011894	11941	945	331.56	2.42	0.83	-1.543823806	Down	5.57E-72	2.81E-70
92211	5349.18	1305.4	450.93	7.4	2.54	-1.542696774	Down	8.80E-101	6.60E-99
LXLOC_000994	5273	115.37	39.91	0.67	0.23	-1.542527234	Down	1.84E-10	1.58E-09
MXLOC_025999	7459	145.34	54.98	0.64	0.22	-1.540568381	Down	1.94E-11	1.80E-10
362	1821	74	26	1.28	0.44	-1.540568381	Down	6.28E-07	3.82E-06
152185	5448	113.24	39.53	0.64	0.22	-1.540568381	Down	4.24E-10	3.53E-09
85407	2586	80	28	0.96	0.33	-1.540568381	Down	2.03E-07	1.31E-06
MXLOC_005319	3949	536.9	193.56	4.3	1.48	-1.538739484	Down	4.53E-40	1.28E-38
27012	2929	85	30	0.9	0.31	-1.537656786	Down	9.95E-08	6.61E-07
MXLOC_001012	8780.41	1307.26	483.38	4.79	1.65	-1.537559631	Down	9.39E-92	6.22E-90
n341727	1435	67	23.56	1.48	0.51	-1.537028024	Down	1.49E-06	8.68E-06
n410212	2808	160.35	56.07	1.77	0.61	-1.536868213	Down	1.71E-13	1.84E-12
MXLOC_002989	2924	598.2	210.99	6.35	2.19	-1.535825722	Down	4.93E-46	1.61E-44
79727	4014	721.91	254.42	5.56	1.92	-1.533978572	Down	6.48E-55	2.53E-53
LXLOC_024456	1886.71	141.25	56.61	2.66	0.92	-1.531720479	Down	3.07E-10	2.59E-09
388677	4962	297.59	105.62	1.85	0.64	-1.531381461	Down	1.79E-23	3.11E-22
MXLOC_005332	9347	1011.6	357.64	3.32	1.15	-1.52954938	Down	4.76E-76	2.53E-74
n408057	4012	523.84	185.66	4.03	1.4	-1.525353012	Down	4.38E-40	1.24E-38
n408144	3831	528.42	187.75	4.26	1.48	-1.525256255	Down	2.19E-40	6.28E-39
148	2301.84	135.19	48.95	1.87	0.65	-1.524526647	Down	2.19E-11	2.03E-10
LXLOC_020355	9033	728.31	258.63	2.47	0.86	-1.522102477	Down	5.45E-55	2.13E-53
22874	7399	157.95	56.86	0.66	0.23	-1.520832163	Down	5.82E-13	6.03E-12
n385849	1176	88.99	31.77	2.41	0.84	-1.520571913	Down	5.67E-08	3.87E-07
n372317	589	42	15	2.38	0.83	-1.519778332	Down	0.000220038	0.000925917
n384792	1558	63.66	22.49	1.29	0.45	-1.519374159	Down	3.99E-06	2.21E-05
160335	4852	6389	2278	40.58	14.16	-1.5189476	Down	0	0
84529	2477	65.7	23.4	0.83	0.29	-1.517058436	Down	3.43E-06	1.91E-05
n406964	3257	216.92	77.31	2.06	0.72	-1.516575526	Down	2.66E-17	3.57E-16
753	8719.26	171	62	0.6	0.21	-1.514573173	Down	1.05E-13	1.14E-12
50865	1209	53	19	1.4	0.49	-1.514573173	Down	3.35E-05	0.00016195
n411133	1313	66.01	23.45	1.6	0.56	-1.514573173	Down	2.26E-06	1.30E-05
n383439	3202	97.21	35.09	0.94	0.33	-1.510194732	Down	2.05E-08	1.46E-07
140609	4134	3081.83	1114.53	23.02	8.14	-1.499787134	Down	1.86E-220	3.11E-218
22901	2770	57.5	20.92	0.65	0.23	-1.498805857	Down	1.25E-05	6.44E-05
655	4031	2692.12	974.2	20.63	7.3	-1.498775452	Down	1.07E-192	1.57E-190
n382102	1799	63	23	1.1	0.39	-1.495957495	Down	7.80E-06	4.15E-05
MXLOC_009619	4812	554.32	200.41	3.55	1.26	-1.494395291	Down	3.25E-41	9.46E-40
7004	1795	76	28	1.35	0.48	-1.491853096	Down	1.04E-06	6.20E-06
285973	4576	87.58	32.11	0.59	0.21	-1.490325627	Down	1.65E-07	1.07E-06
92126	9281	3891.1	1418.11	12.86	4.58	-1.489471139	Down	9.03E-275	1.91E-272
LXLOC_007989	6052.49	1366.56	552.18	7.69	2.74	-1.488807705	Down	1.37E-83	8.15E-82
n409597	3805	158.85	57.36	1.29	0.46	-1.487665299	Down	7.50E-13	7.69E-12
n409752	5499	99.62	37.24	0.56	0.2	-1.485426827	Down	3.34E-08	2.33E-07
LXLOC_011732	1617	79.03	28.67	1.54	0.55	-1.485426827	Down	3.07E-07	1.94E-06
MXLOC_015652	2105.91	256.03	63.87	2.6	0.93	-1.483209002	Down	6.39E-30	1.40E-28
n406564	3042	164.2	59.97	1.67	0.6	-1.476813697	Down	2.48E-13	2.63E-12
MXLOC_020047	4452	610.39	224.53	4.23	1.52	-1.47658634	Down	3.72E-44	1.16E-42
n342764	2691	185	68	2.14	0.77	-1.474680446	Down	1.89E-14	2.15E-13
n345742	2592	228.78	84.25	2.75	0.99	-1.473931188	Down	2.05E-17	2.78E-16
56106	4373.83	1012.58	357.67	6.85	2.47	-1.471592946	Down	3.18E-76	1.70E-74
n380060	3942	124	45	0.97	0.35	-1.470629825	Down	2.61E-10	2.22E-09
n378704	897	58.34	21.42	2.1	0.76	-1.466318004	Down	1.61E-05	8.19E-05
285513	6352	577	214	2.79	1.01	-1.465909829	Down	2.81E-41	8.19E-40
56937	4914	92	34.7	0.58	0.21	-1.465663572	Down	8.04E-08	5.40E-07
57538	10917	208	77	0.58	0.21	-1.465663572	Down	6.48E-16	8.09E-15

55619	7431	167	61	0.69	0.25	-1.464668267	Down	2.73E-13	2.89E-12
3856	1796	694.45	257.02	12.14	4.4	-1.464192993	Down	2.07E-49	7.26E-48
11067	2060	243	90	3.69	1.34	-1.461387816	Down	2.48E-18	3.51E-17
8913	7265.08	362	132	1.51	0.55	-1.457045026	Down	3.97E-27	7.87E-26
9884	5177	304.04	112.64	1.81	0.66	-1.455451768	Down	1.03E-22	1.75E-21
n405879	3277	802.57	299.8	7.59	2.77	-1.454213909	Down	2.77E-56	1.11E-54
MXLOC_007697	6558.44	133.86	49.48	0.63	0.23	-1.453717967	Down	9.41E-11	8.26E-10
26227	2015	1448	541	22.49	8.22	-1.452073362	Down	7.50E-100	5.54E-98
10018	5129.18	187.79	76.82	1.23	0.45	-1.450661409	Down	1.08E-12	1.10E-11
8969	498	126	47	8.61	3.15	-1.450661409	Down	4.24E-10	3.53E-09
8675	4904	64.49	24.21	0.41	0.15	-1.450661409	Down	9.93E-06	5.19E-05
n381184	2481	169.64	63.23	2.13	0.78	-1.449307401	Down	4.41E-13	4.60E-12
51162	1529	77.79	29.09	1.61	0.59	-1.448273829	Down	1.32E-06	7.76E-06
n411761	1529	77.76	29.04	1.61	0.59	-1.448273829	Down	1.32E-06	7.76E-06
8720	4347	1402.36	525.38	9.96	3.65	-1.448249278	Down	2.03E-96	1.43E-94
91133	3587	70	26	0.6	0.22	-1.447458977	Down	3.21E-06	1.80E-05
MXLOC_020018	4756	231.82	86.86	1.5	0.55	-1.447458977	Down	2.23E-17	3.01E-16
400224	1812	69	26	1.2	0.44	-1.447458977	Down	4.79E-06	2.62E-05
n364204	1038	366.63	137.53	11.34	4.16	-1.446765207	Down	1.94E-26	3.75E-25
n407859	2395	110.81	41.78	1.44	0.53	-1.442004547	Down	5.36E-09	4.03E-08
2104	5281.18	718.09	270	4.18	1.54	-1.440572591	Down	6.01E-50	2.13E-48
n340626	1856	45	17	0.76	0.28	-1.440572591	Down	0.000238332	0.000996956
MXLOC_035169	4828	619.72	234.33	3.96	1.46	-1.439532061	Down	5.74E-43	1.74E-41
5357	3706	55	21	0.46	0.17	-1.436099115	Down	5.42E-05	0.000252788
4745	3114	1103.67	418.18	10.99	4.07	-1.433090687	Down	6.37E-75	3.33E-73
LXLOC_003285	8853	167.87	60.48	0.54	0.2	-1.432959407	Down	1.43E-13	1.53E-12
169611	6542	57.85	21.78	0.27	0.1	-1.432959407	Down	2.43E-05	0.000120037
MXLOC_016299	2134	121.6	46.36	1.78	0.66	-1.431339312	Down	1.62E-09	1.27E-08
22882	4359	87	33	0.62	0.23	-1.430634354	Down	3.11E-07	1.96E-06
LXLOC_033737	1588	49.11	18.43	0.97	0.36	-1.429987841	Down	8.95E-05	0.000403446
30010	2921	157	60	1.67	0.62	-1.429507982	Down	7.42E-12	7.07E-11
LXLOC_015700	5377	151	57	0.86	0.32	-1.426264755	Down	1.20E-11	1.13E-10
MXLOC_031377	834	52.36	19.82	2.04	0.76	-1.424497829	Down	5.05E-05	0.000237348
n380158	560	97.58	37.11	5.85	2.18	-1.42410849	Down	7.34E-08	4.95E-07
LXLOC_017411	1513	81	31	1.69	0.63	-1.423599513	Down	9.49E-07	5.68E-06
n383732	3793	165.01	63.16	1.34	0.5	-1.422233001	Down	2.10E-12	2.08E-11
124925	4227.09	212.03	80.56	1.55	0.58	-1.41814341	Down	8.85E-16	1.09E-14
1026	2159	218	83	3.15	1.18	-1.416564969	Down	5.48E-16	6.85E-15
567	987	685	262	22.34	8.37	-1.416329658	Down	1.47E-46	4.85E-45
n371665	2189	55.84	21.47	0.8	0.3	-1.415037499	Down	5.42E-05	0.000252854
4651	11430	60.99	21.04	0.16	0.06	-1.415037499	Down	7.05E-06	3.77E-05
158326	10086	236	90	0.72	0.27	-1.415037499	Down	3.80E-17	5.05E-16
23309	5129	106.71	40.01	0.64	0.24	-1.415037499	Down	1.39E-08	1.00E-07
MXLOC_029992	7799	769.9	295.34	3.03	1.14	-1.410283969	Down	7.88E-52	2.90E-50
3765	3029	392	151	4.01	1.51	-1.409053687	Down	4.54E-27	8.97E-26
LXLOC_018055	2695	200.1	77	2.31	0.87	-1.408805546	Down	1.46E-14	1.67E-13
3761	2019.7	91	34	1.38	0.52	-1.408084739	Down	1.20E-07	7.89E-07
n339406	2157	73	28	1.06	0.4	-1.40599236	Down	3.43E-06	1.91E-05
57348	1896.17	55	21	0.9	0.34	-1.404390255	Down	5.42E-05	0.000252821
9779	6851.84	364.82	149.03	1.72	0.65	-1.403896942	Down	4.89E-23	8.38E-22
MXLOC_010306	1386	48.4	18.61	1.11	0.42	-1.402098444	Down	0.0001344	0.000588926
1063	10296	123	48	0.37	0.14	-1.402098444	Down	2.52E-09	1.95E-08
MXLOC_028588	5974	1025.72	396.13	5.28	2	-1.40053793	Down	9.07E-68	4.34E-66
10312	2620.76	54	21.25	0.66	0.25	-1.40053793	Down	8.05E-05	0.000365051
MXLOC_037235	4637	285.98	111.36	1.9	0.72	-1.399930607	Down	8.66E-20	1.31E-18
n386903	1018	60.15	23.33	1.9	0.72	-1.399930607	Down	2.60E-05	0.000127747
n337872	3744	70.3	27.71	0.58	0.22	-1.398549376	Down	6.01E-06	3.25E-05
MXLOC_012349	2726	102.09	39.27	1.16	0.44	-1.398549376	Down	3.57E-08	2.48E-07
57504	1857	260.25	101.17	4.4	1.67	-1.397655421	Down	2.97E-18	4.17E-17
LXLOC_016641	11738	785.02	306.54	2.05	0.78	-1.394077881	Down	1.30E-51	4.75E-50

LXLOC_030567	7153.6	186.16	75.86	0.84	0.32	-1.392317423	Down		8.88E-13	9.06E-12
MXLOC_020978	5429.16	965.21	475.7	6.93	2.64	-1.392317423	Down		2.50E-41	7.32E-40
6490	2176.86	412	161	5.93	2.26	-1.391709332	Down		8.95E-28	1.81E-26
n344528	1703	52.7	20.36	0.97	0.37	-1.390459477	Down		9.57E-05	0.000429756
656	3773	67.09	26.2	0.55	0.21	-1.389042291	Down		1.05E-05	5.49E-05
MXLOC_009970	2577	186.24	72.85	2.25	0.86	-1.387516437	Down		1.53E-13	1.64E-12
n385355	2638	115.18	45.24	1.36	0.52	-1.387023123	Down		8.95E-09	6.60E-08
n384480	2216	263	103	3.71	1.42	-1.385528257	Down		3.12E-18	4.38E-17
n383794	2004	112.3	43.58	1.75	0.67	-1.385121921	Down		8.49E-09	6.27E-08
n410552	2049	207.2	81.03	3.16	1.21	-1.384917511	Down		1.11E-14	1.27E-13
51168	11863	182	72	0.47	0.18	-1.38466385	Down		6.98E-13	7.19E-12
MXLOC_003333	8469.72	4334.05	1685.14	15.57	5.97	-1.382966108	Down		1.25E-277	2.69E-275
3105	1549	4873.22	1909.14	99.26	38.06	-1.382936893	Down		0.00E+00	5.15E-306
n365510	1588	50.01	19.33	0.99	0.38	-1.381429107	Down		0.0001135	0.000503532
MXLOC_023404	3918	564.72	221.95	4.45	1.71	-1.379809011	Down		2.89E-37	7.64E-36
54432	1852	215.01	84.59	3.64	1.4	-1.378511623	Down		3.16E-15	3.76E-14
n340631	6463	55.5	22	0.26	0.1	-1.378511623	Down		9.97E-05	0.000446783
1006	4952	313.15	123.22	1.95	0.75	-1.378511623	Down		2.66E-21	4.29E-20
23338	6463	55.5	22	0.26	0.1	-1.378511623	Down		9.97E-05	0.000446726
9899	11313.96	712.15	278.79	1.92	0.74	-1.375509135	Down		7.43E-47	2.48E-45
n341249	3029	179.35	70.84	1.84	0.71	-1.373814837	Down		6.67E-13	6.88E-12
MXLOC_031115	10202.66	855.17	339.16	2.59	1	-1.372952098	Down		1.32E-54	5.11E-53
63895	9713	319.46	125	1.01	0.39	-1.372809264	Down		9.00E-22	1.48E-20
LXLOC_027701	4058	99.11	38.49	0.75	0.29	-1.370837695	Down		6.22E-08	4.23E-07
n341537	1711	57.62	22.71	1.06	0.41	-1.37036845	Down		4.57E-05	0.00021622
154043	3228	110	44	1.06	0.41	-1.37036845	Down		3.32E-08	2.32E-07
MXLOC_004228	9327	635.6	253.07	2.09	0.81	-1.367509129	Down		1.09E-40	3.14E-39
8359	372	342	135	32.52	12.61	-1.366758982	Down		5.60E-23	9.59E-22
MXLOC_037568	6770	1543.97	613.59	7.01	2.72	-1.365807793	Down		8.67E-97	6.18E-95
10231	3318	2401.04	952.59	22.42	8.7	-1.365698972	Down		5.23E-150	5.85E-148
3213	3618	1579	627	13.5	5.24	-1.36532069	Down		4.44E-99	3.26E-97
5922	2592	71.21	28.48	0.85	0.33	-1.364996817	Down		7.48E-06	3.98E-05
3488	6239	1897	752	9.35	3.63	-1.364996817	Down		5.72E-119	5.09E-117
11069	4258.47	118.56	47.08	0.85	0.33	-1.364996817	Down		9.33E-09	6.87E-08
2903	12374.8	1352	553	3.45	1.34	-1.364363361	Down		3.93E-81	2.27E-79
n339179	1125	93.37	37.07	2.65	1.03	-1.363348022	Down		3.43E-07	2.15E-06
n387135	2181	66.4	26.63	0.95	0.37	-1.360402243	Down		1.55E-05	7.90E-05
4212	3259.89	7223	2881	68.73	26.79	-1.359245378	Down		0	0
282996	7233	279	111	1.18	0.46	-1.359081093	Down		8.28E-19	1.20E-17
n364518	1147	94.83	37.84	2.64	1.03	-1.357893592	Down		2.35E-07	1.50E-06
LXLOC_037624	3821.15	370.15	133.12	2.69	1.05	-1.357216845	Down		3.14E-28	6.49E-27
23768	7179	1120.08	447.06	4.79	1.87	-1.356987386	Down		3.01E-70	1.49E-68
7122	1702	72	29	1.33	0.52	-1.354842717	Down		9.21E-06	4.85E-05
3696	8777	1353.73	540.64	4.73	1.85	-1.354314913	Down		1.77E-84	1.07E-82
9728	7175	1547.46	619.45	6.62	2.59	-1.353879119	Down		6.19E-96	4.32E-94
MXLOC_022311	4133.84	603.05	211.65	3.96	1.55	-1.353232215	Down		1.27E-46	4.21E-45
64579	3351	221	88	2.04	0.8	-1.350497247	Down		3.45E-15	4.10E-14
6299	5143	131.12	53	0.79	0.31	-1.349584438	Down		2.29E-09	1.78E-08
MXLOC_028545	5394	7631.41	3063.5	43.56	17.1	-1.349007629	Down		0	0
MXLOC_022941	5945	811.02	326.86	4.2	1.65	-1.347923303	Down		8.87E-51	3.19E-49
85360	3254	59	24	0.56	0.22	-1.347923303	Down		6.98E-05	0.000320676
23683	5903	53	22	0.28	0.11	-1.347923303	Down		0.00021349	0.000900725
MXLOC_037313	3711	238.82	98.99	2.06	0.81	-1.346650524	Down		1.90E-15	2.31E-14
25805	1732	116.48	47	2.11	0.83	-1.346059757	Down		1.98E-08	1.41E-07
22999	5724.97	426.95	165.71	2.21	0.87	-1.344959064	Down		4.82E-29	1.02E-27
9021	2734	446	180	5.07	2	-1.341985747	Down		1.70E-28	3.54E-27
MXLOC_016641	5781.25	358.79	134.43	1.77	0.7	-1.338322533	Down		6.77E-26	1.28E-24
n341821	2187	124	50	1.77	0.7	-1.338322533	Down		5.58E-09	4.18E-08
LXLOC_015514	2020	89.78	35.98	1.39	0.55	-1.337581359	Down		4.84E-07	2.98E-06
134429	2264	1008.59	407.5	13.9	5.5	-1.337581359	Down		3.07E-62	1.35E-60

6744	5175	280.14	111.11	1.64	0.65	-1.335184192	Down	5.70E-19	8.32E-18
9516	2471	130	53	1.64	0.65	-1.335184192	Down	3.32E-09	2.54E-08
MXLOC_037281	6295	934.01	377.93	4.56	1.81	-1.333044127	Down	7.87E-58	3.23E-56
n377928	3108	67.75	27.67	0.68	0.27	-1.332575339	Down	1.92E-05	9.59E-05
n371891	3005	80.31	33.06	0.83	0.33	-1.330645312	Down	4.54E-06	2.49E-05
10797	2188	5082.65	2066.04	72.55	28.86	-1.329904314	Down	1.22E-302	3.09E-300
LXLOC_015513	3870	246	100	1.96	0.78	-1.329307625	Down	3.12E-16	3.97E-15
LXLOC_033558	1714	125.88	51.07	2.31	0.92	-1.328187085	Down	6.81E-09	5.07E-08
4299	9285	374.09	150.2	1.23	0.49	-1.327804661	Down	2.19E-24	3.93E-23
3221	1673.07	999	408	18.84	7.51	-1.326914152	Down	1.44E-60	6.13E-59
5116	10560	525.78	214.93	1.53	0.61	-1.326650505	Down	9.72E-33	2.30E-31
6695	4827	2768	1130	17.67	7.06	-1.323561951	Down	1.25E-164	1.56E-162
8492	4573	118	49	0.8	0.32	-1.321928095	Down	2.92E-08	2.05E-07
4646	8662	83.9	33.31	0.3	0.12	-1.321928095	Down	1.47E-06	8.57E-06
LXLOC_032625	4612.91	2319.18	948.52	15.47	6.2	-1.319133076	Down	6.23E-138	6.39E-136
MXLOC_026212	4586	233.21	95.89	1.57	0.63	-1.317340825	Down	2.21E-15	2.66E-14
MXLOC_011298	4809	632.99	260.65	4.06	1.63	-1.316607763	Down	1.70E-38	4.66E-37
92949	6809.59	267	111	1.22	0.49	-1.316027493	Down	6.79E-17	8.91E-16
n385327	3993	151.8	62.31	1.17	0.47	-1.315775868	Down	2.31E-10	1.97E-09
57232	2529.8	177.11	73.26	2.19	0.88	-1.315355441	Down	7.90E-12	7.52E-11
55966	2858.92	367	152	4.03	1.62	-1.314786026	Down	8.69E-23	1.48E-21
MXLOC_000368	2281	67.2	27.33	0.92	0.37	-1.31410859	Down	1.92E-05	9.60E-05
n339481	2533	275.01	113.45	3.38	1.36	-1.313416595	Down	1.13E-17	1.54E-16
57224	7290.97	986.79	406.32	4.15	1.67	-1.313263234	Down	5.24E-59	2.18E-57
n340509	2904	77	32	0.82	0.33	-1.313157885	Down	7.86E-06	4.18E-05
n338629	4217	1037.74	426.61	7.6	3.06	-1.312467766	Down	3.31E-62	1.44E-60
n376212	1845	190.83	78.98	3.25	1.31	-1.310872906	Down	1.11E-12	1.12E-11
161253	1859	325	134	5.48	2.21	-1.310129524	Down	1.68E-20	2.61E-19
10650	1720.49	96.25	39.6	1.76	0.71	-1.309684499	Down	3.54E-07	2.22E-06
3597	4006	148	61	1.14	0.46	-1.309328058	Down	3.96E-10	3.31E-09
285175	13500.07	1288.54	534.02	2.92	1.18	-1.30718151	Down	1.17E-75	6.18E-74
MXLOC_001098	3899	184.5	76.24	1.46	0.59	-1.30718151	Down	3.26E-12	3.18E-11
n344514	922	68.53	28.2	2.4	0.97	-1.306977753	Down	2.34E-05	0.000115904
LXLOC_015515	5960	244	101	1.26	0.51	-1.304854582	Down	1.13E-15	1.40E-14
553158	1857	71.44	29.48	1.21	0.49	-1.304153393	Down	1.34E-05	6.91E-05
8294	370	240	99	22.96	9.3	-1.303820021	Down	1.60E-15	1.95E-14
10507	4628	522.07	216.54	3.48	1.41	-1.303392143	Down	8.75E-32	2.00E-30
n341048	800	116	48	4.73	1.92	-1.300733873	Down	3.48E-08	2.43E-07
n324570	543	80.53	33.3	5	2.03	-1.300448367	Down	4.54E-06	2.49E-05
84168	4835.98	176	86	1.33	0.54	-1.300394933	Down	7.15E-09	5.30E-08
25850	3113	96.31	39.93	0.96	0.39	-1.299560282	Down	3.54E-07	2.22E-06
23397	4495	78	33	0.54	0.22	-1.295455884	Down	9.48E-06	4.97E-05
LXLOC_018576	10205	612.9	255.55	1.84	0.75	-1.294743266	Down	1.46E-36	3.81E-35
MXLOC_029084	1970	112.44	46.86	1.79	0.73	-1.293991218	Down	4.95E-08	3.39E-07
n407952	2376	171.64	72	2.25	0.92	-1.290219235	Down	4.01E-11	3.62E-10
3200	3327.32	531.14	224.89	5.01	2.05	-1.289186694	Down	2.69E-31	6.09E-30
n409064	3822	184.18	76.93	1.49	0.61	-1.288431183	Down	3.26E-12	3.18E-11
51195	3727	226.16	95.22	1.88	0.77	-1.287802311	Down	2.80E-14	3.15E-13
n406622	2568	229.08	96.11	2.78	1.14	-1.286051059	Down	1.64E-14	1.87E-13
MXLOC_025046	4761	118.55	50.43	0.78	0.32	-1.285402219	Down	5.06E-08	3.46E-07
55345	6740	257.6	108.39	1.17	0.48	-1.285402219	Down	4.82E-16	6.05E-15
53834	3088	57	23	0.56	0.23	-1.283792966	Down	8.35E-05	0.000377433
442213	3103.77	407.5	179.45	4.26	1.75	-1.283498508	Down	1.05E-22	1.78E-21
n387224	926	53	22.11	1.85	0.76	-1.283453947	Down	0.00021349	0.000900832
92285	6210	260.71	110.32	1.29	0.53	-1.283306801	Down	4.86E-16	6.09E-15
MXLOC_034692	5041	287.03	120.94	1.75	0.72	-1.28128611	Down	6.94E-18	9.58E-17
n363690	1992	266.25	111.8	4.18	1.72	-1.281094377	Down	9.74E-17	1.27E-15
2138	3973	113	46	0.85	0.35	-1.280107919	Down	3.41E-08	2.38E-07
11259	3274	120	51	1.14	0.47	-1.278301162	Down	4.23E-08	2.91E-07
285704	4580	8025	3386	54.04	22.3	-1.276983965	Down	0	0

84465	5914	1760.27	743.37	9.16	3.78	-1.276961364	Down	1.39E-99	1.03E-97
LXLOC_034205	1938	78	33	1.26	0.52	-1.276840205	Down	9.48E-06	4.97E-05
MXLOC_004857	5052.95	1533.85	667.13	9.64	3.98	-1.276264716	Down	1.78E-82	1.05E-80
23556	4876	118.79	49.69	0.75	0.31	-1.27462238	Down	2.92E-08	2.05E-07
n341415	3351	188	80	1.74	0.72	-1.273018494	Down	6.83E-12	6.52E-11
63916	3579	631.18	267.7	5.46	2.26	-1.272578179	Down	1.09E-36	2.85E-35
MXLOC_032424	3913.17	84.63	37.25	0.7	0.29	-1.271302022	Down	9.33E-06	4.91E-05
n384212	1987	804.6	341.03	12.68	5.26	-1.269420041	Down	4.23E-46	1.39E-44
n385799	3250	98.52	41.53	0.94	0.39	-1.269186633	Down	5.21E-07	3.21E-06
MXLOC_014330	2103	98.61	42.22	1.47	0.61	-1.268935007	Down	8.99E-07	5.40E-06
n383738	2070	747.57	317.03	11.3	4.69	-1.268662945	Down	6.52E-43	1.97E-41
n383499	1898	71.71	30.5	1.18	0.49	-1.267933205	Down	2.36E-05	0.000116797
MXLOC_017820	1256	63.71	26.95	1.61	0.67	-1.264827688	Down	4.87E-05	0.0002295
23090	4818	664.95	283.54	4.25	1.77	-1.263713481	Down	4.74E-38	1.28E-36
MXLOC_005344	5049	1166.64	497.11	7.12	2.97	-1.26141431	Down	1.69E-65	7.86E-64
MXLOC_037713	3300	122	52	1.15	0.48	-1.26052755	Down	3.54E-08	2.46E-07
LXLOC_015594	1875	66	27.89	1.1	0.46	-1.257797757	Down	2.80E-05	0.000136862
LXLOC_012504	4805	290.21	124.67	1.86	0.78	-1.253756592	Down	2.04E-17	2.77E-16
n406996	1425	83.56	35.77	1.86	0.78	-1.253756592	Down	4.58E-06	2.51E-05
MXLOC_004287	6152	361.94	156.14	1.81	0.76	-1.251918374	Down	6.22E-21	9.85E-20
n406883	10263	398.91	170.65	1.19	0.5	-1.250961574	Down	2.26E-23	3.92E-22
LXLOC_029710	2047	103	44	1.57	0.66	-1.25022663	Down	4.37E-07	2.71E-06
MXLOC_005750	1863	63.75	27.61	1.07	0.45	-1.24961389	Down	8.50E-05	0.000383922
MXLOC_011583	5695.02	1085.58	467.76	5.87	2.47	-1.248849462	Down	4.83E-60	2.04E-58
27245	6496	81.17	34.03	0.38	0.16	-1.247927513	Down	5.49E-06	2.98E-05
23216	5688	69.54	30.44	0.38	0.16	-1.247927513	Down	4.87E-05	0.000229511
n338347	4523	56.14	24.47	0.38	0.16	-1.247927513	Down	0.000213764	0.000901773
7060	3223	59	25	0.57	0.24	-1.247927513	Down	0.000122992	0.000542507
MXLOC_001865	5257	97.15	42.01	0.57	0.24	-1.247927513	Down	1.29E-06	7.57E-06
n408153	3102	1421.69	612.59	14.21	5.99	-1.246278646	Down	5.45E-78	2.98E-76
3556	3204.94	190	82	1.85	0.78	-1.245979242	Down	9.72E-12	9.19E-11
LXLOC_013856	2168	101.83	44.02	1.47	0.62	-1.245476034	Down	8.92E-07	5.36E-06
MXLOC_008002	4343	126.04	54.78	0.9	0.38	-1.243925583	Down	2.47E-08	1.75E-07
n406833	1599	58.94	25.37	1.16	0.49	-1.243271151	Down	0.000177396	0.000759148
6813	1890	70	30	1.16	0.49	-1.243271151	Down	3.40E-05	0.000164202
n384090	2167	331.53	143.06	4.78	2.02	-1.242655325	Down	2.50E-19	3.71E-18
MXLOC_014639	6179.81	1470.57	619.86	7.14	3.02	-1.241375525	Down	7.56E-84	4.55E-82
MXLOC_026616	8935	675.64	295.85	2.34	0.99	-1.2410081	Down	5.28E-37	1.39E-35
n410697	3287	90.39	39.46	0.85	0.36	-1.239465935	Down	3.18E-06	1.78E-05
n387725	1681	94.34	40.98	1.77	0.75	-1.23878686	Down	1.29E-06	7.60E-06
100533183	2413	91.31	39.91	1.18	0.5	-1.23878686	Down	2.22E-06	1.27E-05
MXLOC_025833	2404	207.15	89.99	2.69	1.14	-1.238572348	Down	9.72E-13	9.88E-12
n379031	1522	75.98	33.21	1.58	0.67	-1.237691558	Down	2.79E-05	0.000136601
79784	6789	73	32	0.33	0.14	-1.237039197	Down	3.36E-05	0.00016261
MXLOC_016498	4899	105.09	46.23	0.66	0.28	-1.237039197	Down	6.19E-07	3.77E-06
7474	5838	63	28	0.33	0.14	-1.237039197	Down	0.000144599	0.00062824
MXLOC_019886	7012.86	246.01	104.12	1.06	0.45	-1.236067358	Down	2.83E-15	3.39E-14
n407587	3769	89.25	38.15	0.73	0.31	-1.235628248	Down	2.66E-06	1.51E-05
26011	13548	1321.41	575.17	2.99	1.27	-1.235316987	Down	2.86E-71	1.43E-69
85363	2826.33	82.85	31.89	0.8	0.34	-1.234465254	Down	6.40E-07	3.89E-06
n381323	2321	64.63	28.07	0.87	0.37	-1.23349013	Down	0.000101271	0.000453151
n407060	4373	123.13	53.8	0.87	0.37	-1.23349013	Down	4.22E-08	2.91E-07
84435	4283	329	143	2.37	1.01	-1.230531766	Down	5.01E-19	7.35E-18
51171	1237	66.29	29.05	1.71	0.73	-1.228027956	Down	8.40E-05	0.000379517
644815	2930	84	37	0.89	0.38	-1.227805918	Down	9.33E-06	4.91E-05
MXLOC_033082	2226	63.17	27.6	0.89	0.38	-1.227805918	Down	8.50E-05	0.000383873
627	4046.5	649.14	281.9	4.94	2.11	-1.227268043	Down	3.12E-36	8.07E-35
LXLOC_000220	8176	587.5	255.4	2.2	0.94	-1.226770862	Down	9.70E-33	2.30E-31
MXLOC_036955	3639	137.79	60	1.17	0.5	-1.22650853	Down	1.19E-08	8.66E-08
MXLOC_006710	3505	296.47	129.18	2.62	1.12	-1.226068079	Down	3.42E-17	4.55E-16

54976	1376	440.86	192.35	10.15	4.34	-1.22571278	Down	9.40E-25	1.71E-23
6494	3501	164	72	1.45	0.62	-1.22571278	Down	4.77E-10	3.95E-09
MXLOC_008603	3142.28	1252.58	547.6	12.37	5.29	-1.225505873	Down	3.43E-67	1.63E-65
341	461	211.03	92	15.71	6.72	-1.225150043	Down	1.15E-12	1.16E-11
8821	4040.25	2043.71	892.99	15.61	6.68	-1.22455053	Down	1.34E-108	1.07E-106
MXLOC_002722	4324	215.8	93.95	1.54	0.66	-1.222392421	Down	4.77E-13	4.97E-12
LXLOC_035562	2842	57.45	24.88	0.63	0.27	-1.222392421	Down	0.000147877	0.00064161
MXLOC_011935	8362	94.96	41.19	0.35	0.15	-1.222392421	Down	2.20E-06	1.26E-05
n340271	2329	105.72	46.29	1.42	0.61	-1.219009782	Down	6.19E-07	3.77E-06
23224	11979.84	1843.54	713.95	4.14	1.78	-1.217753527	Down	4.03E-120	3.62E-118
MXLOC_027659	3384.14	414.3	144	3	1.29	-1.217591435	Down	8.77E-33	2.09E-31
79602	3965	619.3	272.38	4.83	2.08	-1.215439661	Down	9.56E-34	2.32E-32
n409087	1836	151.92	66.72	2.6	1.12	-1.215012891	Down	2.00E-09	1.56E-08
MXLOC_001779	2426.43	101.22	44.81	1.3	0.56	-1.215012891	Down	8.92E-07	5.36E-06
n345390	2401	95	42	1.23	0.53	-1.214594051	Down	2.60E-06	1.48E-05
4864	4814	1406	619	9	3.88	-1.213868349	Down	3.42E-74	1.77E-72
6391	2833.2	179.53	78.82	1.97	0.85	-1.212660883	Down	5.69E-11	5.09E-10
387496	1543	93	41	1.9	0.82	-1.212303604	Down	3.13E-06	1.75E-05
n363356	2280	272.46	120.46	3.73	1.61	-1.212114942	Down	1.35E-15	1.66E-14
MXLOC_004691	5636.01	1353.85	672.71	8.31	3.59	-1.210864633	Down	3.93E-56	1.57E-54
MXLOC_014976	4739	182.15	80.18	1.18	0.51	-1.210217707	Down	5.62E-11	5.03E-10
MXLOC_032678	9355	361.23	159.35	1.18	0.51	-1.210217707	Down	2.91E-20	4.49E-19
2120	5989	203	90	1.04	0.45	-1.208586622	Down	6.55E-12	6.27E-11
253832	5340	324.7	143.89	1.87	0.81	-1.207044457	Down	2.81E-18	3.96E-17
n337650	2959	264	117	2.77	1.2	-1.20685157	Down	4.60E-15	5.41E-14
221016	2061.59	60.54	26.42	0.9	0.39	-1.206450877	Down	0.000147185	0.000638765
n364205	667	173.6	76.71	8.6	3.73	-1.205161029	Down	1.64E-10	1.41E-09
MXLOC_014257	2511	67.15	29.98	0.83	0.36	-1.20511443	Down	5.87E-05	0.000272247
n337653	4475	351.31	155.62	2.42	1.05	-1.20461772	Down	1.18E-19	1.77E-18
112399	2722	517	229	5.9	2.56	-1.204571144	Down	4.69E-28	9.60E-27
MXLOC_020975	3821.61	1309	610	11.13	4.83	-1.204358499	Down	4.21E-62	1.83E-60
25960	6034	1058	469	5.39	2.34	-1.203776743	Down	1.42E-55	5.64E-54
LXLOC_015647	4040	129	58	0.99	0.43	-1.203091865	Down	6.85E-08	4.63E-07
2674	9323.67	1572	694	5.13	2.23	-1.201915116	Down	2.57E-82	1.51E-80
54768	5167.56	75	35	0.46	0.2	-1.201633861	Down	7.63E-05	0.000347408
114784	13108	295.16	129.22	0.69	0.3	-1.201633861	Down	4.83E-17	6.38E-16
3815	5176	104	46	0.62	0.27	-1.199308808	Down	8.79E-07	5.28E-06
MXLOC_000782	7675	798.24	354.71	3.19	1.39	-1.198471541	Down	2.75E-42	8.18E-41
MXLOC_008192	2645	113.45	50.74	1.33	0.58	-1.19730144	Down	2.97E-07	1.88E-06
LXLOC_026079	2121	69.74	31.19	1.03	0.45	-1.194647431	Down	8.21E-05	0.000371751
MXLOC_009740	1339	100.4	44.85	2.38	1.04	-1.194378045	Down	1.27E-06	7.48E-06
LXLOC_009047	1241	969	433	24.85	10.87	-1.192893911	Down	2.67E-50	9.50E-49
MXLOC_020866	5463	1111.05	496.77	6.26	2.74	-1.191986764	Down	1.64E-57	6.65E-56
n387718	738	6123.06	2740.73	272.07	119.32	-1.189142003	Down	4.00E-307	1.02E-304
MXLOC_026246	2165	118.43	53.17	1.71	0.75	-1.189033824	Down	2.42E-07	1.55E-06
147166	1982	139.86	62.64	2.21	0.97	-1.187989717	Down	1.65E-08	1.19E-07
375449	10240.51	133	63	0.41	0.18	-1.187627003	Down	2.01E-07	1.29E-06
197370	1062	466	209	14.07	6.18	-1.186943585	Down	6.93E-25	1.27E-23
MXLOC_028470	2440	135.22	60.79	1.73	0.76	-1.186700714	Down	2.38E-08	1.69E-07
23175	5363	2640	1186.48	15.16	6.66	-1.186675671	Down	1.57E-132	1.60E-130
1261	3794	81	36	0.66	0.29	-1.186413124	Down	1.60E-05	8.10E-05
339488	2189	64	29	0.91	0.4	-1.185866545	Down	0.000169955	0.000730135
5796	6087	218	98	1.09	0.48	-1.183221824	Down	2.17E-12	2.15E-11
MXLOC_003844	8040	154.55	70.29	0.59	0.26	-1.182203331	Down	5.37E-09	4.03E-08
MXLOC_008511	3083	626.29	282	6.3	2.78	-1.180266946	Down	1.31E-32	3.08E-31
MXLOC_006186	5545.57	1598.98	722.7	8.88	3.92	-1.179706022	Down	4.24E-80	2.41E-78
n385102	5783	225.87	101.63	1.2	0.53	-1.178970141	Down	8.95E-13	9.13E-12
128553	12161.68	890.78	404	2.24	0.99	-1.177998302	Down	5.31E-45	1.68E-43
1268	5840.35	6158	2799.07	32.59	14.42	-1.176358187	Down	1.49E-299	3.69E-297
LXLOC_035461	4056.67	435.57	192.75	3.25	1.44	-1.174370906	Down	5.24E-24	9.29E-23

7571	3242	101	45.56	0.97	0.43	-1.173648087	Down	1.50E-06	8.74E-06
n410704	3636	574.64	260.45	4.89	2.17	-1.172139423	Down	9.81E-30	2.12E-28
27346	2568	6422	2914	77.83	34.55	-1.171640647	Down	0	0
90627	5833	103	46	0.54	0.24	-1.169925001	Down	1.24E-06	7.34E-06
158471	12585	3070.95	1393.25	7.47	3.32	-1.169925001	Down	7.13E-151	8.02E-149
11214	13302	308.74	159.45	0.81	0.36	-1.169925001	Down	6.43E-13	6.64E-12
MXLOC_030763	2990	164.59	74.8	1.71	0.76	-1.169925001	Down	1.31E-09	1.04E-08
90113	4020	82	37	0.63	0.28	-1.169925001	Down	1.88E-05	9.41E-05
30820	1939.19	1635	782	27.76	12.36	-1.167328825	Down	3.91E-73	2.00E-71
56981	2229	72	33	1.01	0.45	-1.166358386	Down	7.95E-05	0.000360734
90293	3384.51	8297.03	3717.41	74.68	33.28	-1.166066494	Down	0	0
MXLOC_017266	7921.19	1811.23	1122.64	9.53	4.25	-1.165013373	Down	6.61E-41	1.91E-39
11245	2816	3973	1813	43.83	19.57	-1.163274923	Down	1.64E-192	2.39E-190
339500	8554	575.34	261.93	2.06	0.92	-1.162938571	Down	1.14E-29	2.47E-28
MXLOC_036987	5028	322.55	147.07	1.97	0.88	-1.162620201	Down	4.00E-17	5.30E-16
90427	4624.72	208	96	1.41	0.63	-1.162271429	Down	2.33E-11	2.15E-10
341359	3286	130	60	1.23	0.55	-1.161154792	Down	1.31E-07	8.57E-07
6769	2963	137	62	1.43	0.64	-1.159871337	Down	3.28E-08	2.30E-07
n339291	2963	137	62	1.43	0.64	-1.159871337	Down	3.28E-08	2.30E-07
n341195	1562	85	39	1.72	0.77	-1.159478214	Down	1.82E-05	9.15E-05
352954	2740	143.75	65.75	1.63	0.73	-1.158903595	Down	1.89E-08	1.35E-07
5947	903.9	8137.05	3730	291.79	130.74	-1.158229839	Down	0	0
4128	4073	509	233	3.86	1.73	-1.15782881	Down	4.88E-26	9.30E-25
MXLOC_034230	5533	8193.62	3755.27	45.58	20.43	-1.157711721	Down	0	0
n406544	6814	64.14	29.18	0.29	0.13	-1.157541277	Down	0.000169955	0.000730224
n408094	4618	246.85	112.62	1.65	0.74	-1.156868849	Down	1.70E-13	1.82E-12
5366	1936	102	47	1.65	0.74	-1.156868849	Down	2.88E-06	1.63E-05
706	866	290	134	10.97	4.92	-1.156833305	Down	3.09E-15	3.69E-14
59307	1639	157.89	71.67	3.01	1.35	-1.15680408	Down	3.19E-09	2.44E-08
n409240	2330	95.01	43.41	1.27	0.57	-1.155794673	Down	4.33E-06	2.38E-05
n406247	2272	136.45	62.77	1.87	0.84	-1.154577037	Down	4.60E-08	3.16E-07
6764	4400.04	205	112	1.71	0.77	-1.151065974	Down	4.93E-08	3.38E-07
158763	3087	70.88	32.49	0.71	0.32	-1.14974712	Down	9.61E-05	0.000431521
n382778	229	65.57	30	11.2	5.05	-1.149143439	Down	0.00019835	0.000842181
5362	11444	2749	1268	7.36	3.32	-1.148522525	Down	5.07E-131	5.04E-129
n383551	2996	541.83	250.76	5.61	2.54	-1.143172274	Down	4.36E-27	8.62E-26
8544	1544.34	185.6	86.71	3.82	1.73	-1.1428006	Down	3.94E-10	3.29E-09
n340992	1970	73.66	33.91	1.17	0.53	-1.142444265	Down	5.63E-05	0.000261694
n338495	1802	104	48	1.81	0.82	-1.142293882	Down	2.39E-06	1.37E-05
n385408	3520	2477	1147.22	21.78	9.87	-1.141881964	Down	2.44E-117	2.12E-115
116985	4969.92	715.75	328.6	4.39	1.99	-1.141452509	Down	9.07E-36	2.32E-34
84626	4019	111.73	52.28	0.86	0.39	-1.140862536	Down	1.58E-06	9.17E-06
n410711	1198	101.15	46.84	2.69	1.22	-1.140725025	Down	2.47E-06	1.41E-05
MXLOC_017951	1904	235.58	109.21	3.88	1.76	-1.140481224	Down	1.54E-12	1.55E-11
MXLOC_031447	587	944.95	437.34	53.82	24.42	-1.140079191	Down	7.22E-46	2.35E-44
n386176	4204	162.29	75.07	1.19	0.54	-1.139930261	Down	4.17E-09	3.16E-08
25925	4971	1057.85	492.1	6.56	2.98	-1.138383484	Down	1.33E-50	4.76E-49
90313	1453	172	80	3.74	1.7	-1.137503524	Down	1.66E-09	1.30E-08
MXLOC_005716	1561	92.65	42.81	1.87	0.85	-1.137503524	Down	7.35E-06	3.92E-05
55008	3889	111	51	0.88	0.4	-1.137503524	Down	9.74E-07	5.82E-06
196	6247	357.21	166.04	1.76	0.8	-1.137503524	Down	3.39E-18	4.76E-17
MXLOC_016288	5895	83.91	39.17	0.44	0.2	-1.137503524	Down	3.56E-05	0.000171638
100526832	4206	275.5	127.43	2.02	0.92	-1.134649527	Down	1.54E-14	1.76E-13
n339110	1695	164.22	76.41	3.05	1.39	-1.13372436	Down	3.47E-09	2.65E-08
MXLOC_018491	4124	1494.87	696.03	11.19	5.1	-1.133640884	Down	1.15E-70	5.70E-69
MXLOC_019736	1964	143.18	66.51	2.28	1.04	-1.132450296	Down	3.07E-08	2.15E-07
LXLOC_026354	8131	152.05	70.37	0.57	0.26	-1.132450296	Down	1.05E-08	7.70E-08
n363687	727	85.85	39.93	3.88	1.77	-1.132307292	Down	1.82E-05	9.15E-05
7430	3119.45	585	273	5.8	2.65	-1.130060541	Down	1.29E-28	2.71E-27
9252	3883	343	171	2.91	1.33	-1.129592907	Down	2.63E-15	3.15E-14

91373	3365	113.93	53.02	1.05	0.48	-1.129283017	Down	1.31E-06	7.68E-06
n407050	968	1084.37	506.05	36.09	16.5	-1.129133119	Down	1.46E-51	5.33E-50
8605	2530.58	408	191	5.03	2.3	-1.128924539	Down	2.58E-20	3.99E-19
LXLOC_035614	3403.55	360.83	168.04	3.28	1.5	-1.128733314	Down	3.27E-18	4.58E-17
n410180	1694	101.33	47.04	1.88	0.86	-1.128324097	Down	4.03E-06	2.23E-05
MXLOC_006856	1874.37	6494.99	3087.05	110.5	50.55	-1.128263372	Down	2.16E-288	4.86E-286
8646	3521	338	158	2.97	1.36	-1.12685628	Down	3.88E-17	5.15E-16
148113	4199	98	46	0.72	0.33	-1.125530882	Down	6.79E-06	3.64E-05
n408344	2097	80.59	37.72	1.2	0.55	-1.125530882	Down	3.72E-05	0.000178254
MXLOC_023634	2979	127.26	59.42	1.33	0.61	-1.124545098	Down	2.19E-07	1.41E-06
MXLOC_027827	17380	3782.02	1775.08	6.65	3.05	-1.124545098	Down	2.47E-173	3.18E-171
MXLOC_037636	2214	137.32	64.46	1.94	0.89	-1.124179411	Down	8.65E-08	5.79E-07
n374520	3624	229.29	107.18	1.96	0.9	-1.122856748	Down	4.29E-12	4.16E-11
23215	10384	1377.72	648.66	4.07	1.87	-1.121990524	Down	5.73E-64	2.57E-62
LXLOC_015661	663	255.77	120.1	12.76	5.87	-1.120195921	Down	3.94E-13	4.13E-12
6770	2695	510.44	240.14	5.89	2.71	-1.119974782	Down	9.48E-25	1.72E-23
64927	3691.67	191.35	91.78	1.63	0.75	-1.119909464	Down	5.75E-10	4.73E-09
5525	3128	228	107	2.26	1.04	-1.119739244	Down	5.95E-12	5.71E-11
MXLOC_024706	3225	368.19	173.44	3.54	1.63	-1.118877396	Down	2.51E-18	3.54E-17
57419	3889	1494	704	11.87	5.47	-1.117707197	Down	4.62E-69	2.25E-67
MXLOC_036551	8166	305.94	143.8	1.15	0.53	-1.117569596	Down	1.59E-15	1.94E-14
MXLOC_005312	7182.48	594.81	280.95	2.56	1.18	-1.117356951	Down	1.82E-28	3.79E-27
n407891	5191	175.25	83.28	1.04	0.48	-1.115477217	Down	2.54E-09	1.96E-08
MXLOC_031093	16307	69.21	33.68	0.13	0.06	-1.115477217	Down	0.000218478	0.000920669
1001	4276	72	34	0.52	0.24	-1.115477217	Down	0.000129248	0.000568188
22981	4973	570.39	269.58	3.53	1.63	-1.114796219	Down	2.55E-27	5.08E-26
112885	3517.42	1759.28	830.05	15.48	7.15	-1.114390324	Down	9.21E-81	5.29E-79
MXLOC_021073	5065.39	438.29	216.95	2.79	1.29	-1.112894056	Down	1.45E-19	2.17E-18
9717	6456	168	80	0.8	0.37	-1.112474729	Down	6.11E-09	4.57E-08
80000	6052	760.59	359.56	3.87	1.79	-1.112373979	Down	8.20E-36	2.10E-34
n345653	2539	185.49	87.73	2.27	1.05	-1.11230297	Down	6.31E-10	5.16E-09
MXLOC_034309	6674.29	1394.66	660.04	6.42	2.97	-1.112110366	Down	6.09E-64	2.73E-62
MXLOC_029837	3299	71.42	33.27	0.67	0.31	-1.11189288	Down	0.000111768	0.000496163
n370153	1103	1411.28	667.41	40.94	18.96	-1.110552138	Down	6.60E-65	3.04E-63
n378391	1752	68.52	32.74	1.23	0.57	-1.109624491	Down	0.00018974	0.000807957
n377427	10803	342.11	162.89	0.97	0.45	-1.108059746	Down	6.79E-17	8.91E-16
n409149	3160	85.72	40.28	0.84	0.39	-1.106915204	Down	2.95E-05	0.000143739
MXLOC_013312	10269	1270.12	601.57	3.79	1.76	-1.10662242	Down	1.80E-58	7.41E-57
2904	5941	191	90	0.99	0.46	-1.105794664	Down	3.62E-10	3.03E-09
220213	3113	432	205	4.3	2	-1.10433666	Down	7.26E-21	1.15E-19
9509	3725.85	78	25	0.43	0.2	-1.10433666	Down	5.61E-08	3.83E-07
619279	14386	2497.79	1186.83	5.31	2.47	-1.104200819	Down	2.54E-112	2.11E-110
n380562	2744	88.8	42.27	1.01	0.47	-1.103622631	Down	2.80E-05	0.000137184
8911	9897	187	90	0.58	0.27	-1.103093493	Down	1.31E-09	1.04E-08
642475	3251	137.26	65.81	1.31	0.61	-1.102685664	Down	1.38E-07	9.07E-07
MXLOC_035524	5561	1738.91	827.61	9.62	4.48	-1.102538162	Down	1.85E-78	1.03E-76
MXLOC_004940	4231	241.06	114.72	1.76	0.82	-1.101879614	Down	2.27E-12	2.25E-11
575	5528	396.25	189.51	2.21	1.03	-1.101402032	Down	4.84E-19	7.11E-18
LXLOC_001005	553.18	358.52	201.6	25.75	12.01	-1.100336281	Down	3.76E-12	3.65E-11
MXLOC_015588	2765	331.48	158.27	3.73	1.74	-1.100088324	Down	3.68E-16	4.67E-15
51332	11723	115	54	0.3	0.14	-1.099535674	Down	1.08E-06	6.44E-06
3781	2515	159	77	1.99	0.93	-1.097465809	Down	2.80E-08	1.97E-07
8900	1917.26	614	290	9.92	4.64	-1.096215315	Down	2.95E-29	6.30E-28
n378606	3523	70.69	33.21	0.62	0.29	-1.096215315	Down	0.000156584	0.000676561
4781	8276.25	1738.31	832.4	6.45	3.02	-1.094750611	Down	1.75E-77	9.49E-76
79094	1560	681	326	13.77	6.45	-1.094157494	Down	2.06E-31	4.66E-30
n371672	987	77.83	37.2	2.54	1.19	-1.093866923	Down	0.000101211	0.000453001
165	4081	127	61	0.96	0.45	-1.093109404	Down	5.59E-07	3.43E-06
8727	2445	164.82	79.78	2.1	0.99	-1.084888898	Down	1.40E-08	1.01E-07
MXLOC_024851	9009	360.39	174.87	1.23	0.58	-1.08453351	Down	5.04E-17	6.66E-16

83642	2315	157.14	75.92	2.12	1	-1.084064265	Down	2.14E-08	1.52E-07
MXLOC_010160	4409	993	478.7	6.95	3.28	-1.083317163	Down	1.60E-44	5.05E-43
n381274	1864	74	36	1.25	0.59	-1.083141235	Down	0.000170031	0.000730375
6217	590	1744	840	98.78	46.64	-1.08265118	Down	9.40E-77	5.06E-75
MXLOC_013695	5116	362.07	175.36	2.18	1.03	-1.081683798	Down	4.20E-17	5.56E-16
MXLOC_008097	1447	117	56.35	2.56	1.21	-1.081136763	Down	1.43E-06	8.38E-06
5243	4716	2790.98	1348.96	18.25	8.63	-1.080463999	Down	7.54E-121	6.83E-119
3164	2606.23	141.54	68.71	1.69	0.8	-1.078951341	Down	1.50E-07	9.83E-07
n410669	7542	1077.19	522.17	4.39	2.08	-1.077637411	Down	1.86E-47	6.30E-46
MXLOC_031608	3234	301.58	146.32	2.89	1.37	-1.0768936	Down	2.23E-14	2.52E-13
n337201	989	186	89.93	6.05	2.87	-1.075884405	Down	1.15E-09	9.16E-09
28232	4283.12	186	92	1.37	0.65	-1.07566427	Down	4.36E-09	3.29E-08
2895	3282	230	112	2.17	1.03	-1.075050705	Down	3.04E-11	2.78E-10
n380842	1047	753.6	365.25	23.09	10.96	-1.075020375	Down	1.10E-33	2.67E-32
3600	2323	117.8	56.95	1.58	0.75	-1.074962058	Down	1.43E-06	8.38E-06
n382029	3508	90.88	44.35	0.8	0.38	-1.074000581	Down	3.66E-05	0.000175623
2564	3152	81	40	0.8	0.38	-1.074000581	Down	0.00010857	0.000482755
116113	5942.27	78.12	37.56	0.4	0.19	-1.074000581	Down	7.28E-05	0.000332937
6901	1870.67	142.4	69.48	2.4	1.14	-1.074000581	Down	1.72E-07	1.11E-06
n407055	4012	314.42	152.96	2.42	1.15	-1.073373186	Down	5.39E-15	6.32E-14
n378108	643	186.82	90.66	9.63	4.58	-1.0721882	Down	1.81E-09	1.41E-08
MXLOC_037560	5072	794.77	387.79	4.83	2.3	-1.070389328	Down	5.04E-35	1.27E-33
11217	6896.01	1868.09	919.52	8.39	4.01	-1.065068574	Down	1.97E-78	1.09E-76
3148	1527	314	154	6.52	3.12	-1.063325935	Down	1.30E-14	1.49E-13
LXLOC_028493	3765	257.49	125.27	2.11	1.01	-1.062887706	Down	2.01E-12	2.00E-11
692312	3270	281.54	137.85	2.67	1.28	-1.060695932	Down	2.19E-13	2.33E-12
401190	3821	1057	518	8.55	4.1	-1.06030051	Down	1.57E-45	5.06E-44
MXLOC_019703	2984	170.37	83.1	1.77	0.85	-1.058214614	Down	1.25E-08	9.08E-08
3638	2984.23	5440	2666	56.37	27.12	-1.055570389	Down	1.31E-226	2.27E-224
n341757	2765	93.9	46.73	1.06	0.51	-1.055495113	Down	3.44E-05	0.000165971
50863	3269.09	376.49	193.85	3.74	1.8	-1.055041364	Down	1.23E-15	1.51E-14
23286	6728.17	295.78	146	1.35	0.65	-1.054447784	Down	1.43E-13	1.54E-12
LXLOC_004687	1631.59	91.02	49.51	1.95	0.94	-1.052741462	Down	0.000218722	0.000921257
MXLOC_012551	2323	208.72	102.97	2.8	1.35	-1.05246742	Down	3.64E-10	3.05E-09
64506	3154.68	1369.49	676.55	13.47	6.5	-1.051238228	Down	1.68E-57	6.78E-56
MXLOC_016069	6065	236.15	116.9	1.2	0.58	-1.0489096	Down	2.71E-11	2.48E-10
8825	1230	1202	594	31.11	15.05	-1.047614908	Down	1.28E-50	4.61E-49
84695	3121	326.61	161.47	3.24	1.57	-1.045229254	Down	6.63E-15	7.73E-14
57526	9613.49	105	51	0.33	0.16	-1.044394119	Down	7.02E-06	3.75E-05
54815	5672	183	90.55	0.99	0.48	-1.044394119	Down	4.64E-09	3.50E-08
10472	4036.06	345.36	173.16	2.68	1.3	-1.043721377	Down	3.33E-15	3.97E-14
n378353	998	125.43	62.25	4.04	1.96	-1.043501639	Down	1.65E-06	9.57E-06
MXLOC_001935	9097.09	2833.33	1394.16	9.47	4.6	-1.041730565	Down	6.34E-118	5.57E-116
442117	2850	2066	1026	22.51	10.94	-1.040953319	Down	8.92E-85	5.46E-83
389333	3251	114	57	1.09	0.53	-1.04026387	Down	5.80E-06	3.14E-05
283431	2341	82.96	41.22	1.11	0.54	-1.039528364	Down	0.000122955	0.000542411
n341617	6253	150.59	75.33	0.74	0.36	-1.039528364	Down	1.95E-07	1.25E-06
6506	11974.89	2808	1399.4	7.19	3.5	-1.038636849	Down	1.03E-113	8.53E-112
n384306	2096	104.69	52.06	1.56	0.76	-1.037474705	Down	1.50E-05	7.64E-05
n339894	3586	554.28	276.08	4.78	2.33	-1.036680663	Down	8.16E-24	1.44E-22
n384177	1602	82.35	40.95	1.62	0.79	-1.036069255	Down	7.88E-05	0.000357835
MXLOC_036883	5899.09	487.2	288.52	3.01	1.47	-1.033947332	Down	6.45E-14	7.09E-13
MXLOC_028705	2401	165.77	83.01	2.15	1.05	-1.033947332	Down	5.90E-08	4.02E-07
MXLOC_006323	3056	257.79	128.8	2.62	1.28	-1.033423002	Down	7.39E-12	7.04E-11
29993	5325.29	81	41	0.47	0.23	-1.031026896	Down	0.000168144	0.000723235
9037	11808	182.96	89.8	0.47	0.23	-1.031026896	Down	4.09E-09	3.10E-08
78986	1649	1518.62	759.13	28.99	14.19	-1.030680744	Down	7.75E-62	3.36E-60
2847	2423	300	150	3.86	1.89	-1.030214613	Down	1.71E-13	1.84E-12
3214	2033	1657	829	25.5	12.49	-1.02972377	Down	3.04E-67	1.45E-65
n326057	503	170.45	85.21	11.51	5.64	-1.029120766	Down	2.99E-08	2.10E-07

MXLOC_036319	5657	378.29	189.23	2.06	1.01	-1.028289044	Down	1.29E-16	1.67E-15
83931	3846	135.01	67.53	1.08	0.53	-1.026967048	Down	6.42E-07	3.91E-06
57110	1062	110	55	3.32	1.63	-1.026311277	Down	8.47E-06	4.48E-05
283212	1938	171.47	86.25	2.77	1.36	-1.026279325	Down	3.36E-08	2.35E-07
7447	2005	257	129	4.01	1.97	-1.025406607	Down	1.13E-11	1.06E-10
MXLOC_017260	3159	118.02	59.65	1.16	0.57	-1.025090981	Down	3.97E-06	2.19E-05
MXLOC_024879	6051	939.5	473.05	4.78	2.35	-1.024349861	Down	2.77E-38	7.54E-37
LXLOC_013857	926	85.71	43.07	2.99	1.47	-1.024329329	Down	0.000114404	0.000507161
MXLOC_036182	9223	568.78	286.41	1.89	0.93	-1.023083613	Down	7.95E-24	1.40E-22
n385533	2968	124.34	62.88	1.3	0.64	-1.022367813	Down	2.25E-06	1.29E-05
n337830	4939	2419	1217	15.09	7.43	-1.02215869	Down	4.32E-96	3.03E-94
LXLOC_031733	1988	127.68	63.98	2.01	0.99	-1.021695071	Down	1.37E-06	8.00E-06
8293	1913	570.32	436.66	14.2	7	-1.020464103	Down	4.44E-06	2.44E-05
81786	2174.17	98	51	1.46	0.72	-1.019899557	Down	6.16E-05	0.000285307
n375994	3504	248	125.38	2.19	1.08	-1.019899557	Down	3.21E-11	2.92E-10
n378039	1002	89.27	45.14	2.87	1.42	-1.015159807	Down	7.79E-05	0.000354112
n406106	2172	147.48	74.82	2.12	1.05	-1.013674937	Down	3.19E-07	2.01E-06
80746	2249.84	243.41	116.37	3.19	1.58	-1.013631866	Down	2.98E-12	2.92E-11
7541	3498	266.79	135.25	2.36	1.17	-1.01227833	Down	9.18E-12	8.70E-11
3161	3093	140	70	1.39	0.69	-1.010416616	Down	4.98E-07	3.07E-06
n385517	1716	929	471	17.02	8.45	-1.010207791	Down	2.37E-37	6.29E-36
6745	9874	603.6	306.97	1.87	0.93	-1.007735649	Down	9.04E-25	1.65E-23
n338142	1401	248	126	5.61	2.79	-1.007735649	Down	4.86E-11	4.36E-10
MXLOC_034022	4480.02	834.76	415.05	5.63	2.8	-1.007708095	Down	4.05E-35	1.02E-33
6470	2507.73	162.84	84.09	2.05	1.02	-1.007054758	Down	2.20E-07	1.41E-06
11145	1383.87	364	193	8.7	4.33	-1.006648376	Down	3.92E-14	4.37E-13
MXLOC_012283	6117	589.9	301.29	2.97	1.48	-1.004865755	Down	7.37E-24	1.30E-22
MXLOC_016650	4274.2	898.76	462.03	6.53	3.26	-1.002211027	Down	5.57E-35	1.40E-33
n384753	9070	83.98	42.26	0.28	0.14	-1	Down	0.000138678	0.000606168
LXLOC_028947	4775	244.68	124.69	1.58	0.79	-1	Down	7.05E-11	6.26E-10
n340418	4383	111	57	0.78	0.39	-1	Down	1.45E-05	7.43E-05
57572	6387	225.4	113.75	1.08	0.54	-1	Down	2.20E-10	1.87E-09
n344637	15820	12.62	0	0.02	0.01	-1	Down	0.000210376	0.00088918
MXLOC_007878	7402	259.35	133.46	1.08	0.54	-1	Down	3.25E-11	2.96E-10
MXLOC_025613	8056	88.14	45.19	0.34	0.17	-1	Down	0.000106069	0.000472227

Table S3 Control vs R521H List of Differentially Expressed Genes and lncRNAs

geneID	geneLength	SA-Expression	SC-Expression	SA-FPKM	SC-FPKM	log2 Ratio(SC/SA)	Up-Down-Regulation(SC/SA)	P-value	FDR
6192	897	1	3803.33	0.04	150.65	11.87891305	Up	0	0
100526842	1941	0	1140.92	0.01	20.21	10.98085361	Up	0	0
8653	4488	0	2595	0.01	19.57	10.93442804	Up	0	0
MXLOC_037825	9712	0	5434.43	0.01	18.82	10.87805091	Up	0	0
n367152	605	0	288.15	0.01	17.44	10.76818432	Up	2.15E-93	3.07E-91
n367153	690	0	321.83	0.01	16.88	10.72109919	Up	5.21E-104	8.29E-102
n379185	566	0	196.61	0.01	12.8	10.32192809	Up	8.47E-64	7.44E-62
MXLOC_018066	2432	0	892.61	0.01	12.55	10.29347165	Up	1.07E-287	5.79E-285
9086	1399	0	492	0.01	12.22	10.25502857	Up	5.10E-159	1.37E-156
MXLOC_031534	556	0	180.79	0.01	12	10.22881869	Up	1.19E-58	9.36E-57
LXLOC_025669	11497.71	31.87	17504.39	0.05	51.17	9.999154423	Up	0	0
85453	4463	0	1278	0.01	9.69	9.920352855	Up	0	0
51142	831	6	5169	0.23	222.09	9.915294858	Up	0	0
n371595	5241	3	2810.49	0.02	18.12	9.82336724	Up	0	0
MXLOC_031523	2048	0	492.05	0.01	8.25	9.688250309	Up	5.10E-159	1.36E-156
n370886	527	0	116	0.01	8.17	9.674192268	Up	4.60E-38	2.13E-36
8284	5484.85	1	1254	0.01	7.72	9.592457037	Up	0	0
7544	5173.47	2	966.25	0.01	6.31	9.301496195	Up	2.00E-306	1.23E-303
22986	5757	0	1073	0.01	6.29	9.296916207	Up	0	0
85460	6936	0	1199.57	0.01	5.83	9.187352073	Up	0	0
246126	865	0	135.93	0.01	5.6	9.129283017	Up	3.56E-44	1.94E-42
MXLOC_012296	2814	0	461.61	0.01	5.59	9.126704473	Up	4.78E-149	1.18E-146
23641	1381	1	433	0.02	10.9	9.09011242	Up	1.11E-137	2.54E-135
n344758	575	0	82.86	0.01	5.3	9.049848549	Up	3.99E-27	1.21E-25
LXLOC_030897	4855	1	749	0.01	5.22	9.027905997	Up	4.24E-239	1.75E-236
n381224	837	0	111.32	0.01	4.75	8.891783703	Up	1.87E-36	8.18E-35
114815	6914.13	0	935	0.01	4.56	8.832890014	Up	1.58E-301	9.38E-299
n342404	3500	1.81	924.34	0.02	8.97	8.808964175	Up	2.65E-295	1.50E-292
83259	5935.58	11.45	3128.36	0.04	17.79	8.796850795	Up	0	0
n371628	882	0	108.45	0.01	4.37	8.77148947	Up	1.72E-35	7.37E-34
9087	1669	0	206.15	0.01	4.27	8.73809226	Up	5.14E-67	4.88E-65
57482	7013	0	871.4	0.01	4.19	8.710806434	Up	6.12E-281	3.15E-278
n408177	5631	0	647.55	0.01	3.88	8.599912842	Up	7.00E-209	2.45E-206
n367818	676	0	71	0.01	3.81	8.573647187	Up	1.38E-23	3.57E-22
MXLOC_037837	16045	0	1767.1	0.01	3.7	8.531381461	Up	0	0
MXLOC_037155	1784	0	189.45	0.01	3.66	8.515699838	Up	1.51E-61	1.27E-59
n385770	692	0	64.43	0.01	3.37	8.396604781	Up	2.46E-21	5.73E-20
MXLOC_036697	695	0	63.11	0.01	3.28	8.357552005	Up	5.16E-21	1.17E-19
n340661	911	0	79	0.01	3.08	8.266786541	Up	3.68E-26	1.08E-24
n370241	1205	0	102.04	0.01	2.96	8.209453366	Up	1.47E-33	5.85E-32
MXLOC_037797	1264	0	106.67	0.01	2.95	8.204571144	Up	7.59E-35	3.16E-33
n376387	618	0	49.03	0.01	2.9	8.17990909	Up	1.65E-16	2.85E-15
LXLOC_010423	201	0	12.83	0.01	2.86	8.159871337	Up	0.000131537	0.000655161
n367646	3239	0	261.14	0.01	2.74	8.098032083	Up	1.04E-84	1.29E-82
n367151	202	0	12	0.01	2.65	8.049848549	Up	0.000131537	0.000655814
n410162	2708	0	199.91	0.01	2.52	7.977279923	Up	9.18E-65	8.29E-63
121256	5777	0	410	0.01	2.4	7.906890596	Up	1.22E-132	2.59E-130
7404	5742.88	4	1219	0.03	7.17	7.900866808	Up	0	0
n382689	3356	0	232.86	0.01	2.36	7.882643049	Up	2.22E-75	2.37E-73
n387383	2171	0	146.82	0.01	2.32	7.857980995	Up	1.03E-47	6.10E-46
LXLOC_031486	303	0	17.27	0.01	2.3	7.845490051	Up	3.24E-06	2.15E-05
MXLOC_030800	1697	0	111.98	0.01	2.28	7.832890014	Up	1.87E-36	8.17E-35
22829	1408.31	4.32	362.8	0.04	8.95	7.805743872	Up	1.90E-109	3.12E-107
MXLOC_024347	1955	0	126.54	0.01	2.23	7.8008999	Up	2.79E-41	1.43E-39
57209	4883	6	1223.57	0.04	8.47	7.726218159	Up	0	0
n408895	2542	0	155.02	0.01	2.08	7.700439718	Up	1.31E-50	8.50E-49

MXLOC_017395	2060	1.29	248.21	0.02	4.14	7.693486957	Up	2.08E-78	2.33E-76
202559	2300	0	138.02	0.01	2.05	7.6794801	Up	3.85E-45	2.16E-43
n376099	277	0	13.72	0.01	2.04	7.672425342	Up	6.27E-05	0.000334323
n380876	450	0	23.98	0.01	2.02	7.658211483	Up	3.81E-08	3.24E-07
83444	1266	0	70.55	0.01	1.94	7.599912842	Up	2.89E-23	7.43E-22
n379711	700	0	37.45	0.01	1.93	7.592457037	Up	1.19E-12	1.59E-11
n366112	562	0	29.25	0.01	1.92	7.584962501	Up	4.47E-10	4.71E-09
LXLOC_027755	10144	4.22	578.3	0.01	1.92	7.584962501	Up	3.96E-178	1.19E-175
158931	1532	0	83.69	0.01	1.89	7.562242424	Up	1.90E-27	5.88E-26
n380097	624	0	32.32	0.01	1.89	7.562242424	Up	4.84E-11	5.62E-10
n379132	719	0	37.53	0.01	1.88	7.554588852	Up	1.19E-12	1.60E-11
n385953	852	0	44.59	0.01	1.87	7.54689446	Up	6.68E-15	1.04E-13
MXLOC_009124	2498	1	135.19	0.01	1.85	7.531381461	Up	2.57E-42	1.36E-40
n386119	2377	0	127.75	0.01	1.84	7.523561956	Up	1.33E-41	6.91E-40
n375805	287	0	12.76	0.01	1.81	7.499845887	Up	0.000131537	0.000653489
LXLOC_009481	535	0	26	0.01	1.8	7.491853096	Up	4.12E-09	3.91E-08
MXLOC_005248	4414	0	222.43	0.01	1.71	7.417852515	Up	3.66E-72	3.75E-70
n342576	2623	0	129.28	0.01	1.68	7.392317423	Up	3.03E-42	1.60E-40
83742	3237	0	159.29	0.01	1.67	7.383704292	Up	6.77E-52	4.54E-50
n379627	643	0	28.64	0.01	1.62	7.339850003	Up	9.38E-10	9.58E-09
n378557	2440	0	114.31	0.01	1.6	7.321928095	Up	2.03E-37	9.14E-36
n326481	767	0	33.84	0.01	1.58	7.303780748	Up	2.31E-11	2.75E-10
n410542	3330	0	154.92	0.01	1.58	7.303780748	Up	2.75E-50	1.75E-48
n371627	630	0	26.61	0.01	1.54	7.266786541	Up	4.12E-09	3.91E-08
MXLOC_031416	446	0	18.05	0.01	1.53	7.257387843	Up	1.54E-06	1.08E-05
MXLOC_018160	5385	0	242.3	0.01	1.52	7.247927513	Up	1.35E-78	1.52E-76
MXLOC_000220	7837	0	350.88	0.01	1.51	7.238404739	Up	2.44E-113	4.25E-111
MXLOC_031465	2017	0	85.53	0.01	1.46	7.189824559	Up	4.32E-28	1.37E-26
MXLOC_000788	4357	0	184	0.01	1.43	7.159871337	Up	6.14E-60	4.93E-58
340578	2599	0	106	0.01	1.39	7.118941073	Up	7.59E-35	3.17E-33
n339566	5298	0	216.43	0.01	1.38	7.108524457	Up	3.12E-70	3.10E-68
n406213	1491	0	59	0.01	1.37	7.098032083	Up	9.99E-20	2.10E-18
MXLOC_020364	971	0	37.26	0.01	1.36	7.087462841	Up	1.19E-12	1.59E-11
n377331	962	0	36.05	0.01	1.33	7.055282436	Up	2.50E-12	3.26E-11
MXLOC_009135	5327.96	9	1020.85	0.05	6.47	7.015693807	Up	0	0
7691	3031	2	229	0.02	2.57	7.005624549	Up	1.52E-70	1.51E-68
LXLOC_037890	1498	0	55	0.01	1.27	6.988684687	Up	1.93E-18	3.78E-17
LXLOC_032867	1762	0	64.29	0.01	1.26	6.977279923	Up	2.46E-21	5.74E-20
n337837	1634	0	59.17	0.01	1.25	6.965784285	Up	9.99E-20	2.10E-18
139067	594	0	20	0.01	1.23	6.942514505	Up	3.51E-07	2.66E-06
MXLOC_011575	2150	0	77.36	0.01	1.23	6.942514505	Up	1.62E-25	4.63E-24
MXLOC_009923	4736	0	169.13	0.01	1.21	6.918863237	Up	4.11E-55	3.00E-53
n383449	827	0	27.99	0.01	1.21	6.918863237	Up	1.97E-09	1.94E-08
9743	10093.87	0	363.45	0.01	1.21	6.918863237	Up	1.61E-117	2.96E-115
n408154	4822	0	169.01	0.01	1.19	6.894817763	Up	4.11E-55	3.01E-53
LXLOC_031665	782	0	26	0.01	1.19	6.894817763	Up	4.12E-09	3.91E-08
n407477	1073	0	36.44	0.01	1.19	6.894817763	Up	2.50E-12	3.26E-11
LXLOC_037827	534	0	17	0.01	1.18	6.882643049	Up	3.24E-06	2.15E-05
n335158	413	0	12.58	0.01	1.17	6.87036472	Up	0.000131537	0.000653952
n384552	2161	0	73.93	0.01	1.17	6.87036472	Up	3.13E-24	8.33E-23
55582	4653	0	159.72	0.01	1.16	6.857980995	Up	6.77E-52	4.53E-50
n408124	7217	2.84	245.73	0.01	1.15	6.845490051	Up	1.24E-75	1.33E-73
55747	4326	0	140.84	0.01	1.1	6.781359714	Up	8.76E-46	5.03E-44
n340530	1485	0	46.97	0.01	1.1	6.781359714	Up	1.52E-15	2.47E-14
n409330	3314	0	105.36	0.01	1.08	6.754887502	Up	1.59E-34	6.54E-33
n371652	500	0	14.43	0.01	1.08	6.754887502	Up	2.99E-05	0.000169043
MXLOC_037798	6779	0	217.75	0.01	1.08	6.754887502	Up	1.49E-70	1.49E-68
n340193	827	0	25	0.01	1.08	6.754887502	Up	8.65E-09	7.90E-08
n378802	1135	0	34.65	0.01	1.07	6.741466986	Up	1.10E-11	1.35E-10
MXLOC_004812	7180.07	0	227.43	0.01	1.07	6.741466986	Up	9.03E-74	9.38E-72

113451	2182	0	67.24	0.01	1.06	6.727920455	Up	2.67E-22	6.52E-21
n384866	442	0	12.35	0.01	1.06	6.727920455	Up	0.000131537	0.000654231
n341221	1198	0	36.03	0.01	1.05	6.714245518	Up	2.50E-12	3.26E-11
54923	1199	0	36.07	0.01	1.05	6.714245518	Up	2.50E-12	3.26E-11
n382011	1723	0	52.23	0.01	1.05	6.714245518	Up	1.78E-17	3.31E-16
n341754	1032	0	30.32	0.01	1.04	6.700439718	Up	2.13E-10	2.33E-09
LXLOC_025065	3356	3.19	305.87	0.03	3.1	6.691161905	Up	5.14E-93	7.32E-91
100506164	1955	0	58	0.01	1.02	6.672425342	Up	2.10E-19	4.31E-18
n376420	1118	0	32.23	0.01	1.01	6.658211483	Up	4.84E-11	5.62E-10
n410075	2077	0	61.16	0.01	1.01	6.658211483	Up	2.27E-20	4.95E-19
152756	2174	5	445	0.07	7.02	6.647972298	Up	4.04E-134	8.85E-132
64172	2316	0	67.48	0.01	1	6.64385619	Up	2.67E-22	6.52E-21
80778	1943	0	55.92	0.01	0.99	6.62935662	Up	1.93E-18	3.78E-17
n378285	614	0	16.65	0.01	0.99	6.62935662	Up	6.80E-06	4.28E-05
23504	6322	0	183.47	0.01	0.98	6.614709844	Up	1.29E-59	1.03E-57
LXLOC_036330	9841	3	286.87	0.01	0.98	6.614709844	Up	5.50E-87	6.96E-85
n335677	490	0	12.68	0.01	0.97	6.599912842	Up	0.000131537	0.000655627
n381340	941	0	25.84	0.01	0.97	6.599912842	Up	8.65E-09	7.91E-08
MXLOC_031930	9068	0	259.74	0.01	0.96	6.584962501	Up	4.59E-84	5.62E-82
728927	2098	0	57.26	0.01	0.94	6.554588852	Up	4.40E-19	8.93E-18
n339540	1456	0	39.36	0.01	0.94	6.554588852	Up	2.71E-13	3.81E-12
n410691	4753	0	130.43	0.01	0.93	6.539158811	Up	1.44E-42	7.69E-41
LXLOC_015176	3136	0	85.44	0.01	0.93	6.539158811	Up	4.32E-28	1.37E-26
8287	10048	0	274.02	0.01	0.92	6.523561956	Up	6.86E-89	9.13E-87
n332598	497	0	12.11	0.01	0.91	6.50779464	Up	0.000131537	0.000654417
n341836	2466	0	64.73	0.01	0.9	6.491853096	Up	2.46E-21	5.74E-20
51305	1303	0	33	0.01	0.88	6.459431619	Up	2.31E-11	2.75E-10
n374869	837	0	20.49	0.01	0.87	6.442943496	Up	3.51E-07	2.66E-06
n339251	1543	0	38.78	0.01	0.87	6.442943496	Up	5.69E-13	7.80E-12
n385891	513	0	12	0.01	0.87	6.442943496	Up	0.000131537	0.000655068
113675	1397	0	34.78	0.01	0.87	6.442943496	Up	1.10E-11	1.35E-10
n339699	2865	0	72.21	0.01	0.86	6.426264755	Up	6.57E-24	1.73E-22
54925	3027	0	76.27	0.01	0.86	6.426264755	Up	3.39E-25	9.51E-24
n411024	3692	0	93.82	0.01	0.86	6.426264755	Up	1.15E-30	4.07E-29
MXLOC_037830	11834	7.94	597.87	0.02	1.7	6.409390936	Up	5.25E-179	1.59E-176
n369482	646	0	15	0.01	0.85	6.409390936	Up	1.43E-05	8.49E-05
n342917	788	0	18.44	0.01	0.84	6.392317423	Up	1.54E-06	1.08E-05
10256	2536	0	62	0.01	0.84	6.392317423	Up	1.08E-20	2.42E-19
n380296	872	0	20.41	0.01	0.83	6.375039431	Up	3.51E-07	2.65E-06
LXLOC_009135	9488	5	465.68	0.02	1.65	6.366322214	Up	1.85E-140	4.29E-138
n377827	2763	0	66.49	0.01	0.82	6.357552005	Up	5.59E-22	1.35E-20
113402	691	0	15.73	0.01	0.82	6.357552005	Up	1.43E-05	8.50E-05
139065	8742.28	62	4651	0.22	17.91	6.347118003	Up	0	0
n407017	2356	0	55.63	0.01	0.81	6.339850003	Up	1.93E-18	3.78E-17
n338122	1310	0	30.43	0.01	0.81	6.339850003	Up	2.13E-10	2.33E-09
MXLOC_016469	5233	0	125.93	0.01	0.81	6.339850003	Up	5.86E-41	2.97E-39
MXLOC_028216	2572	0	60.69	0.01	0.81	6.339850003	Up	4.76E-20	1.02E-18
54531	2746	0	64.7	0.01	0.8	6.321928095	Up	2.46E-21	5.73E-20
MXLOC_022238	2867	0	67.72	0.01	0.8	6.321928095	Up	2.67E-22	6.51E-21
n384454	3518	0	81.97	0.01	0.79	6.303780748	Up	8.36E-27	2.51E-25
728936	3831	0	88.41	0.01	0.78	6.285402219	Up	4.68E-29	1.56E-27
n384275	1450	0	32.72	0.01	0.78	6.285402219	Up	4.84E-11	5.62E-10
84445	2848	0	64.82	0.01	0.78	6.285402219	Up	2.46E-21	5.72E-20
147741	2802	10	704	0.11	8.57	6.283719775	Up	4.54E-208	1.58E-205
n381062	2173	0	49.1	0.01	0.77	6.266786541	Up	1.65E-16	2.85E-15
n368112	580	0	12.18	0.01	0.77	6.266786541	Up	0.000131537	0.000654882
51059	6962	0	159	0.01	0.77	6.266786541	Up	6.77E-52	4.52E-50
MXLOC_026382	8034.24	0	184.15	0.01	0.77	6.266786541	Up	6.14E-60	4.95E-58
5746	2713	0	61	0.01	0.77	6.266786541	Up	2.27E-20	4.95E-19
n364173	2186	0	48.18	0.01	0.76	6.247927513	Up	3.45E-16	5.86E-15

1826	7109	0	160	0.01	0.76	6.247927513	Up	3.23E-52	2.19E-50
n342118	2272	0	49.99	0.01	0.75	6.22881869	Up	1.65E-16	2.85E-15
LXLOC_037867	6834	5.32	300.26	0.02	1.48	6.209453366	Up	2.55E-88	3.34E-86
n411604	604	0	12.13	0.01	0.74	6.209453366	Up	0.000131537	0.000653767
n383255	2185	0	45.86	0.01	0.72	6.169925001	Up	3.19E-15	5.08E-14
n407042	1769	0	37.05	0.01	0.72	6.169925001	Up	1.19E-12	1.60E-11
n341917	1594	0	33.21	0.01	0.72	6.169925001	Up	2.31E-11	2.75E-10
n385240	698	0	13.51	0.01	0.7	6.129283017	Up	6.27E-05	0.000334527
MXLOC_026024	2354	0	48	0.01	0.7	6.129283017	Up	3.45E-16	5.85E-15
n410549	807	0	15.75	0.01	0.7	6.129283017	Up	1.43E-05	8.50E-05
MXLOC_036737	2881	0	58.92	0.01	0.7	6.129283017	Up	2.10E-19	4.32E-18
n342541	2161	0	43.79	0.01	0.69	6.108524457	Up	1.40E-14	2.14E-13
n386598	687	0	13.11	0.01	0.69	6.108524457	Up	6.27E-05	0.000334731
MXLOC_031029	2306	0	46.72	0.01	0.69	6.108524457	Up	1.52E-15	2.47E-14
n407987	1591	0	31.65	0.01	0.69	6.108524457	Up	1.02E-10	1.14E-09
n408345	2176	0	43.72	0.01	0.69	6.108524457	Up	1.40E-14	2.13E-13
n337733	2768	0	54.74	0.01	0.67	6.06608919	Up	4.06E-18	7.76E-17
n383897	1186	0	22.73	0.01	0.67	6.06608919	Up	7.98E-08	6.54E-07
n335522	843	0	15.69	0.01	0.66	6.044394119	Up	1.43E-05	8.51E-05
n365470	838	0	15.48	0.01	0.66	6.044394119	Up	1.43E-05	8.50E-05
n335658	740	0	13.66	0.01	0.66	6.044394119	Up	6.27E-05	0.000334374
n409314	824	0	15	0.01	0.65	6.022367813	Up	1.43E-05	8.51E-05
MXLOC_025881	9390	0	180.09	0.01	0.65	6.022367813	Up	1.19E-58	9.33E-57
n385378	715	0	12.97	0.01	0.65	6.022367813	Up	0.000131537	0.000654975
n370154	924	0	16.84	0.01	0.65	6.022367813	Up	6.80E-06	4.28E-05
MXLOC_000891	1290	0	23.88	0.01	0.65	6.022367813	Up	3.81E-08	3.23E-07
n365807	821	0	14.81	0.01	0.64	6	Up	2.99E-05	0.000168961
n342518	2109	0	39	0.01	0.63	5.977279923	Up	2.71E-13	3.81E-12
MXLOC_007731	7863	0	147.34	0.01	0.63	5.977279923	Up	4.91E-48	2.94E-46
10742	2226	0	40.14	0.01	0.62	5.95419631	Up	1.29E-13	1.85E-12
n375011	921	0	16.04	0.01	0.62	5.95419631	Up	6.80E-06	4.27E-05
85450	4364	0	78.44	0.01	0.61	5.930737338	Up	7.72E-26	2.23E-24
n379528	2240	0	39.02	0.01	0.6	5.906890596	Up	2.71E-13	3.81E-12
n386118	2448	0	43.31	0.01	0.6	5.906890596	Up	1.40E-14	2.14E-13
n375076	901	0	15.21	0.01	0.6	5.906890596	Up	1.43E-05	8.49E-05
n410986	1684	0	28.54	0.01	0.59	5.882643049	Up	9.38E-10	9.58E-09
255919	2097	0	36.02	0.01	0.59	5.882643049	Up	2.50E-12	3.26E-11
55139	2578.99	0	44.11	0.01	0.58	5.857980995	Up	6.68E-15	1.05E-13
55893	4802	0	81.75	0.01	0.58	5.857980995	Up	8.36E-27	2.51E-25
80256	3028	0	50.98	0.01	0.57	5.832890014	Up	7.85E-17	1.39E-15
n342584	2565	0	42.44	0.01	0.57	5.832890014	Up	2.94E-14	4.39E-13
n383700	1412	0.79	46.38	0.02	1.14	5.832890014	Up	1.52E-15	2.47E-14
n382637	1318	0	21.69	0.01	0.57	5.832890014	Up	1.67E-07	1.32E-06
n409255	2210	0	36.29	0.01	0.56	5.807354922	Up	2.50E-12	3.27E-11
n381958	1547	0	25	0.01	0.56	5.807354922	Up	8.65E-09	7.90E-08
n407274	3674	0	59.36	0.01	0.55	5.781359714	Up	9.99E-20	2.10E-18
n377414	1107	0	16.75	0.01	0.53	5.727920455	Up	6.80E-06	4.28E-05
n339574	1987	0	29.98	0.01	0.52	5.700439718	Up	4.47E-10	4.71E-09
n338063	1682	0	25.01	0.01	0.51	5.672425342	Up	8.65E-09	7.90E-08
MXLOC_023493	6462.9	8.71	390.52	0.04	2.04	5.672425342	Up	2.81E-112	4.78E-110
126068	4448	15	664.07	0.1	5.05	5.658211483	Up	2.95E-188	9.30E-186
n410878	1633	0	23.75	0.01	0.5	5.64385619	Up	3.81E-08	3.24E-07
n341430	1799	0	26.21	0.01	0.5	5.64385619	Up	4.12E-09	3.91E-08
n382503	860	0	12.13	0.01	0.5	5.64385619	Up	0.000131537	0.000654138
79746	1654	0	24	0.01	0.5	5.64385619	Up	1.81E-08	1.60E-07
n339648	1302	0	18.65	0.01	0.5	5.64385619	Up	1.54E-06	1.07E-05
n368072	1493	0	21.3	0.01	0.49	5.614709844	Up	1.67E-07	1.32E-06
n339431	2666	0	38	0.01	0.49	5.614709844	Up	5.69E-13	7.80E-12
MXLOC_006511	1383	0	19.68	0.01	0.49	5.614709844	Up	7.37E-07	5.33E-06
n372429	1559	0	21.85	0.01	0.49	5.614709844	Up	1.67E-07	1.32E-06

169044	6346	0	90	0.01	0.48	5.584962501	Up	1.06E-29	3.65E-28
n338984	1744	0	23.79	0.01	0.47	5.554588852	Up	3.81E-08	3.24E-07
n339862	2005	0	27.25	0.01	0.47	5.554588852	Up	1.97E-09	1.94E-08
n408054	1594	0	21.17	0.01	0.46	5.523561956	Up	1.67E-07	1.32E-06
n346481	1114	0	14.49	0.01	0.46	5.523561956	Up	2.99E-05	0.00016907
n408903	1709	0	22.98	0.01	0.46	5.523561956	Up	7.98E-08	6.55E-07
n383564	1562	0	20.56	0.01	0.46	5.523561956	Up	3.51E-07	2.65E-06
n387313	1647	0	22	0.01	0.46	5.523561956	Up	7.98E-08	6.54E-07
n343066	2308	0	31	0.01	0.46	5.523561956	Up	1.02E-10	1.14E-09
n407171	3416	0	44.93	0.01	0.45	5.491853096	Up	6.68E-15	1.05E-13
n407832	3274	0	43.22	0.01	0.45	5.491853096	Up	1.40E-14	2.14E-13
n381078	1100	0	14.15	0.01	0.45	5.491853096	Up	2.99E-05	0.000168988
n340244	1129	0	14	0.01	0.44	5.459431619	Up	2.99E-05	0.000169125
147645	3674	0	47.26	0.01	0.44	5.459431619	Up	7.24E-16	1.20E-14
n410111	2819	0	36.72	0.01	0.44	5.459431619	Up	2.50E-12	3.26E-11
n341491	1982	0	24.81	0.01	0.43	5.426264755	Up	1.81E-08	1.60E-07
121260	2789	0	34.81	0.01	0.43	5.426264755	Up	1.10E-11	1.35E-10
n410267	1333	0	16.33	0.01	0.43	5.426264755	Up	6.80E-06	4.28E-05
MXLOC_037635	3355	0	42.35	0.01	0.43	5.426264755	Up	2.94E-14	4.38E-13
MXLOC_026600	1353	0	16.61	0.01	0.43	5.426264755	Up	6.80E-06	4.27E-05
MXLOC_031415	1590	21.57	843.74	0.43	18.36	5.416085589	Up	5.74E-236	2.34E-233
497190	1943	0	23.95	0.01	0.42	5.392317423	Up	3.81E-08	3.24E-07
n340730	2954	0	36.73	0.01	0.42	5.392317423	Up	2.50E-12	3.26E-11
144423	2721	0	32.74	0.01	0.41	5.357552005	Up	4.84E-11	5.63E-10
n384541	1994	0	24.08	0.01	0.41	5.357552005	Up	1.81E-08	1.60E-07
n408118	2573	0	30.63	0.01	0.41	5.357552005	Up	2.13E-10	2.33E-09
n410518	3045	0	37.03	0.01	0.41	5.357552005	Up	1.19E-12	1.60E-11
MXLOC_033012	2592	0	31.35	0.01	0.41	5.357552005	Up	1.02E-10	1.14E-09
MXLOC_006847	3916	0	45.95	0.01	0.4	5.321928095	Up	3.19E-15	5.08E-14
MXLOC_014045	2810	0	32.98	0.01	0.4	5.321928095	Up	4.84E-11	5.62E-10
MXLOC_001705	7788	0	93.18	0.01	0.4	5.321928095	Up	1.15E-30	4.07E-29
n338277	2524	2.33	86.21	0.03	1.17	5.285402219	Up	2.26E-25	6.37E-24
n340532	3711	0	42.3	0.01	0.39	5.285402219	Up	2.94E-14	4.38E-13
LXLOC_027648	3174.76	7.12	255.04	0.07	2.73	5.285402219	Up	1.56E-71	1.58E-69
n407996	3353	0	37.15	0.01	0.38	5.247927513	Up	1.19E-12	1.60E-11
n407409	2963	1	33	0.01	0.38	5.247927513	Up	4.34E-10	4.59E-09
n378204	1706	0	18.91	0.01	0.38	5.247927513	Up	1.54E-06	1.07E-05
n408111	3630	0	40.54	0.01	0.38	5.247927513	Up	1.29E-13	1.85E-12
11122	12653.44	0	145	0.01	0.38	5.247927513	Up	2.16E-47	1.28E-45
n340931	1668	0	18.51	0.01	0.38	5.247927513	Up	1.54E-06	1.07E-05
n409151	1660	0	17.63	0.01	0.37	5.209453366	Up	3.24E-06	2.15E-05
n386068	2486	0	27.21	0.01	0.37	5.209453366	Up	1.97E-09	1.94E-08
n411124	1577	0	16.69	0.01	0.37	5.209453366	Up	6.80E-06	4.28E-05
n378521	1169	0	12.42	0.01	0.37	5.209453366	Up	0.000131537	0.000654789
MXLOC_003080	1302	0	13.91	0.01	0.37	5.209453366	Up	6.27E-05	0.000334272
n338834	1882	0	20.29	0.01	0.37	5.209453366	Up	3.51E-07	2.65E-06
n340297	2010	0	21.12	0.01	0.36	5.169925001	Up	1.67E-07	1.32E-06
LXLOC_034677	4523	13.59	426.45	0.09	3.19	5.147487612	Up	6.12E-117	1.11E-114
n377679	1512	0	15.36	0.01	0.35	5.129283017	Up	1.43E-05	8.51E-05
LXLOC_026962	1574	0	15.8	0.01	0.35	5.129283017	Up	1.43E-05	8.50E-05
51309	2141	122	3823	1.78	61.24	5.104525137	Up	0	0
n342890	4661	0	47.33	0.01	0.34	5.087462841	Up	7.24E-16	1.20E-14
n381833	1282	0	12.43	0.01	0.34	5.087462841	Up	0.000131537	0.000654045
80728	5143	0	51.77	0.01	0.34	5.087462841	Up	3.74E-17	6.76E-16
LXLOC_036359	1528	0	15.13	0.01	0.34	5.087462841	Up	1.43E-05	8.50E-05
182	5986	0	58.61	0.01	0.33	5.044394119	Up	2.10E-19	4.32E-18
9909	5688	0	55.69	0.01	0.33	5.044394119	Up	1.93E-18	3.77E-17
n410503	1524	0	14.06	0.01	0.32	5	Up	2.99E-05	0.000169098
MXLOC_033062	2939	0	27.62	0.01	0.32	5	Up	1.97E-09	1.94E-08
MXLOC_009177	1454	0	13.26	0.01	0.32	5	Up	6.27E-05	0.000334476

643641	6897	0	65.08	0.01	0.32	5	Up	1.17E-21	2.78E-20
57562	7043	0	67.85	0.01	0.32	5	Up	2.67E-22	6.52E-21
n385203	1902	0	17.66	0.01	0.32	5	Up	3.24E-06	2.15E-05
n340930	1965	0	17.46	0.01	0.31	4.95419631	Up	3.24E-06	2.15E-05
n386566	1403	0	12.71	0.01	0.31	4.95419631	Up	0.000131537	0.00065451
n363336	2598	0	23.47	0.01	0.31	4.95419631	Up	3.81E-08	3.24E-07
27255	3530	0	32	0.01	0.31	4.95419631	Up	4.84E-11	5.62E-10
MXLOC_027263	1761	3.64	93.52	0.06	1.83	4.930737338	Up	2.51E-26	7.40E-25
66004	4653.58	25.41	706.55	0.17	5.14	4.918161708	Up	1.23E-188	3.91E-186
n408015	5051	0	44.69	0.01	0.3	4.906890596	Up	6.68E-15	1.05E-13
84033	22457.45	0	200.22	0.01	0.3	4.906890596	Up	4.38E-65	3.98E-63
n406636	2562	0	22.19	0.01	0.3	4.906890596	Up	7.98E-08	6.54E-07
n386362	3134	0	28.08	0.01	0.3	4.906890596	Up	9.38E-10	9.58E-09
n410951	2201	0	18.7	0.01	0.29	4.857980995	Up	1.54E-06	1.08E-05
3248	2721.83	0	23	0.01	0.29	4.857980995	Up	3.81E-08	3.24E-07
n409076	1947	0	16.38	0.01	0.29	4.857980995	Up	6.80E-06	4.28E-05
MXLOC_008022	3242	0	27.91	0.01	0.29	4.857980995	Up	1.97E-09	1.94E-08
n407158	4177	0	35.9	0.01	0.29	4.857980995	Up	5.25E-12	6.64E-11
n376419	2547	8	213	0.1	2.86	4.837943242	Up	2.16E-57	1.65E-55
n325598	2491	1.82	40.5	0.02	0.56	4.807354922	Up	2.90E-12	3.76E-11
n407262	6477	0	54.31	0.01	0.28	4.807354922	Up	4.06E-18	7.75E-17
n341347	4007	0	33.53	0.01	0.28	4.807354922	Up	2.31E-11	2.75E-10
LXLOC_027263	6859	17.38	449.72	0.08	2.21	4.787902559	Up	2.97E-119	5.62E-117
90485	2774	6.32	157	0.07	1.93	4.785102115	Up	1.56E-42	8.28E-41
2555	2421	0	19.31	0.01	0.27	4.754887502	Up	7.37E-07	5.33E-06
90665	2312	0	18	0.01	0.27	4.754887502	Up	1.54E-06	1.07E-05
9196	1608	0	12.34	0.01	0.27	4.754887502	Up	0.000131537	0.000655254
n384437	2400	0	19	0.01	0.27	4.754887502	Up	7.37E-07	5.33E-06
n410886	1072	0.86	24.76	0.03	0.81	4.754887502	Up	1.81E-08	1.60E-07
60676	4370	0	35.25	0.01	0.27	4.754887502	Up	5.25E-12	6.64E-11
MXLOC_005870	5911	0	47	0.01	0.27	4.754887502	Up	7.24E-16	1.20E-14
6650	4744	0	36.61	0.01	0.26	4.700439718	Up	2.50E-12	3.27E-11
LXLOC_000662	2621	0	20.19	0.01	0.26	4.700439718	Up	3.51E-07	2.66E-06
n377818	2906	0	22.07	0.01	0.26	4.700439718	Up	7.98E-08	6.54E-07
n379552	2773	0	21.19	0.01	0.26	4.700439718	Up	1.67E-07	1.32E-06
987	10109.28	0	78.55	0.01	0.26	4.700439718	Up	7.72E-26	2.23E-24
126433	2338	0	17.43	0.01	0.26	4.700439718	Up	3.24E-06	2.15E-05
n378819	3891	0	29	0.01	0.25	4.64385619	Up	4.47E-10	4.71E-09
LXLOC_010147	2026	0	15	0.01	0.25	4.64385619	Up	1.43E-05	8.50E-05
n342880	1822	5.56	129.75	0.1	2.45	4.614709844	Up	4.26E-35	1.80E-33
1088	2303	0	16.21	0.01	0.24	4.584962501	Up	6.80E-06	4.29E-05
n338306	2297	0	16.05	0.01	0.24	4.584962501	Up	6.80E-06	4.28E-05
n408257	6837	0	48.57	0.01	0.24	4.584962501	Up	3.45E-16	5.85E-15
MXLOC_033737	4439.41	0	31.98	0.01	0.24	4.584962501	Up	1.02E-10	1.14E-09
n368039	1445	1	20	0.02	0.48	4.584962501	Up	4.21E-06	2.75E-05
n376318	2368	0.91	16.85	0.01	0.24	4.584962501	Up	6.80E-06	4.28E-05
282973	6488	0	45.55	0.01	0.24	4.584962501	Up	3.19E-15	5.08E-14
60680	2140.79	20.32	190.05	0.13	3.04	4.547487795	Up	9.35E-40	4.59E-38
n410190	5251	0	35.02	0.01	0.23	4.523561956	Up	5.25E-12	6.64E-11
4899	3530	0	23.85	0.01	0.23	4.523561956	Up	3.81E-08	3.24E-07
79772	3810.94	0	25.74	0.01	0.23	4.523561956	Up	8.65E-09	7.90E-08
n410543	3636	0	24.45	0.01	0.23	4.523561956	Up	1.81E-08	1.60E-07
MXLOC_014012	2002	10.68	222.66	0.17	3.82	4.489965987	Up	6.27E-58	4.86E-56
n368044	4043	11.52	239.9	0.09	2.01	4.48112669	Up	5.22E-62	4.43E-60
n409697	1974	0	12.48	0.01	0.22	4.459431619	Up	0.000131537	0.000655907
n339236	2683	0	17	0.01	0.22	4.459431619	Up	3.24E-06	2.15E-05
n410158	7753	0	49.5	0.01	0.22	4.459431619	Up	1.65E-16	2.85E-15
n410672	11598	0	77.24	0.01	0.22	4.459431619	Up	1.62E-25	4.62E-24
LXLOC_026224	1872.36	23.69	343.94	0.29	6.32	4.445799753	Up	2.92E-81	3.42E-79
n366113	827	1.33	24.87	0.05	1.07	4.419538892	Up	2.55E-07	1.97E-06

n344939	3263	0	20.16	0.01	0.21	4.392317423	Up	3.51E-07	2.66E-06
MXLOC_019220	3625	1.72	22.09	0.01	0.21	4.392317423	Up	1.04E-06	7.41E-06
3682	3861	0	23.51	0.01	0.21	4.392317423	Up	3.81E-08	3.24E-07
MXLOC_009711	3065	0	19.32	0.01	0.21	4.392317423	Up	7.37E-07	5.33E-06
MXLOC_032968	2933	0	18.41	0.01	0.21	4.392317423	Up	1.54E-06	1.07E-05
MXLOC_026420	2419	6.03	115.02	0.08	1.63	4.348728154	Up	8.40E-30	2.88E-28
n379410	2589	0	15	0.01	0.2	4.321928095	Up	1.43E-05	8.50E-05
LXLOC_037828	7653	63.13	1098.72	0.25	4.84	4.275007047	Up	1.35E-266	6.32E-264
MXLOC_031485	1466	3.75	64.93	0.08	1.54	4.266786541	Up	1.84E-17	3.41E-16
n377716	2927	0	16.69	0.01	0.19	4.247927513	Up	6.80E-06	4.28E-05
n409347	5945	0	33.9	0.01	0.19	4.247927513	Up	2.31E-11	2.75E-10
n379927	3108	0	16.98	0.01	0.19	4.247927513	Up	6.80E-06	4.28E-05
n342249	3063	0	16.69	0.01	0.19	4.247927513	Up	6.80E-06	4.27E-05
n383561	661	8.41	143.27	0.42	7.87	4.227902403	Up	3.29E-36	1.43E-34
n383256	2031	5	80.76	0.08	1.37	4.098032083	Up	2.53E-20	5.52E-19
n407227	4063	0	20.37	0.01	0.17	4.087462841	Up	3.51E-07	2.66E-06
55626	5023	0	24.58	0.01	0.17	4.087462841	Up	1.81E-08	1.60E-07
n337755	3857	0	19.58	0.01	0.17	4.087462841	Up	7.37E-07	5.33E-06
n339330	6368	0	31.3	0.01	0.17	4.087462841	Up	1.02E-10	1.14E-09
n406460	2918	0	14.28	0.01	0.17	4.087462841	Up	2.99E-05	0.000168934
280	2181	11.46	170.81	0.16	2.68	4.06608919	Up	2.23E-41	1.15E-39
MXLOC_008235	3496	10.13	151.36	0.09	1.47	4.029747343	Up	1.07E-36	4.71E-35
7803	5082	116	1717	0.7	11.42	4.028063918	Up	0	0
MXLOC_028755	3327	0	16.05	0.01	0.16	4	Up	6.80E-06	4.28E-05
n409066	4177	0	20.02	0.01	0.16	4	Up	3.51E-07	2.66E-06
n338470	2709	0	13.09	0.01	0.16	4	Up	6.27E-05	0.000334425
n344596	539	2.16	32.28	0.14	2.22	3.987060944	Up	8.33E-09	7.62E-08
64849	4084	4.52	54.22	0.03	0.45	3.906890596	Up	1.48E-13	2.11E-12
441631	5562	0	24.07	0.01	0.15	3.906890596	Up	1.81E-08	1.60E-07
n410718	3623	0	15.53	0.01	0.15	3.906890596	Up	1.43E-05	8.51E-05
n381430	8670	16.4	232.36	0.06	0.9	3.906890596	Up	4.77E-55	3.48E-53
n384427	3081	0	13.36	0.01	0.15	3.906890596	Up	6.27E-05	0.000334629
53335	4362.14	2	38	0.02	0.3	3.906890596	Up	1.34E-10	1.49E-09
LXLOC_018922	226	0.93	12.29	0.16	2.35	3.876516947	Up	0.000131537	0.000655441
130367	1200	1	15	0.03	0.44	3.874469118	Up	0.000133596	0.000663059
LXLOC_026450	6461	40.64	529.44	0.19	2.76	3.860596943	Up	1.99E-120	3.78E-118
n342407	796	44.78	574.61	1.83	25.85	3.820248727	Up	5.16E-130	1.08E-127
n379947	1610	4.97	65.87	0.1	1.41	3.817623258	Up	8.52E-17	1.50E-15
56034	3008	0	12	0.01	0.14	3.807354922	Up	0.000131537	0.000655534
7226	5876	0	24.84	0.01	0.14	3.807354922	Up	1.81E-08	1.60E-07
n339737	3971	0	16.22	0.01	0.14	3.807354922	Up	6.80E-06	4.28E-05
2998	3132	0	13	0.01	0.14	3.807354922	Up	6.27E-05	0.00033468
92293	4947	0	20	0.01	0.14	3.807354922	Up	3.51E-07	2.65E-06
n384784	2818	23	285.18	0.25	3.45	3.786596362	Up	2.25E-64	2.00E-62
MXLOC_026921	2591.01	32	396.98	0.38	5.23	3.782739623	Up	8.40E-89	1.11E-86
n407964	2542	10.19	119.43	0.12	1.61	3.745954377	Up	2.21E-27	6.83E-26
n378429	776	5.65	69.14	0.24	3.2	3.736965594	Up	4.38E-17	7.87E-16
n338234	3484	23	272	0.2	2.65	3.727920455	Up	1.23E-60	1.00E-58
n407543	2874	15.71	187.69	0.17	2.23	3.713437059	Up	7.97E-43	4.27E-41
MXLOC_031497	3645	13.38	154.49	0.11	1.44	3.710493383	Up	5.55E-35	2.32E-33
n365082	3493	0	13.79	0.01	0.13	3.700439718	Up	6.27E-05	0.000334782
n375857	2455	2.3	28.35	0.03	0.39	3.700439718	Up	1.27E-07	1.01E-06
n338436	3178	0	12.05	0.01	0.13	3.700439718	Up	0.000131537	0.000654696
n384279	3222	0	12.34	0.01	0.13	3.700439718	Up	0.000131537	0.000654603
n407948	4508	0	17.91	0.01	0.13	3.700439718	Up	3.24E-06	2.15E-05
n385789	6662	0	26.1	0.01	0.13	3.700439718	Up	4.12E-09	3.91E-08
n410169	3209	0	12.22	0.01	0.13	3.700439718	Up	0.000131537	0.000654324
4791	3049	0	12	0.01	0.13	3.700439718	Up	0.000131537	0.00065386
n384662	1357	10.29	121.17	0.24	3.11	3.695808269	Up	5.88E-28	1.85E-26
7757	9088	97.11	1150.18	0.33	4.26	3.690315501	Up	2.94E-251	1.26E-248

n365174	2398	7	81.47	0.09	1.16	3.688055994	Up	6.69E-19	1.35E-17
n339966	1572	6.47	75.02	0.13	1.65	3.665882496	Up	5.50E-18	1.04E-16
n386086	4100	21.46	241.11	0.16	1.99	3.636624621	Up	2.16E-53	1.51E-51
152330	5190.56	72.09	812.17	0.43	5.29	3.620859157	Up	4.49E-175	1.31E-172
MXLOC_033676	1534	4.68	53.09	0.1	1.2	3.584962501	Up	2.89E-13	4.06E-12
29940	4039	0	14.05	0.01	0.12	3.584962501	Up	2.99E-05	0.000169016
n411582	5960	0	20.31	0.01	0.12	3.584962501	Up	3.51E-07	2.65E-06
n410638	3335	9.96	102.76	0.09	1.05	3.544320516	Up	2.57E-23	6.63E-22
n379098	3475	9.29	92.92	0.08	0.91	3.50779464	Up	1.78E-20	3.92E-19
386618	2119	3	28	0.04	0.45	3.491853096	Up	7.30E-07	5.29E-06
LXLOC_020539	3964	0	13.14	0.01	0.11	3.459431619	Up	6.27E-05	0.000334578
n345534	4859	0	16.32	0.01	0.11	3.459431619	Up	6.80E-06	4.28E-05
n376096	4116	0	13.45	0.01	0.11	3.459431619	Up	6.27E-05	0.000334221
51473	4700.63	38	380	0.25	2.74	3.454175893	Up	1.83E-79	2.09E-77
LXLOC_013847	373	2	20	0.19	2.08	3.452512205	Up	2.64E-05	0.000150525
LXLOC_018116	2057	19	183	0.29	3.05	3.394684437	Up	1.54E-38	7.28E-37
63906	2129	4.19	38.97	0.06	0.63	3.392317423	Up	5.78E-09	5.38E-08
LXLOC_014761	465	4.09	38.73	0.3	3.14	3.387730153	Up	5.78E-09	5.38E-08
LXLOC_024547	965	3	28.32	0.1	1.04	3.378511623	Up	7.30E-07	5.29E-06
56142	4351.09	229.28	3034.22	2.31	23.62	3.354044208	Up	0	0
n406672	2988	22.81	215.05	0.24	2.45	3.351675438	Up	2.94E-45	1.65E-43
n342022	2086	21.52	197.95	0.32	3.26	3.348728154	Up	5.67E-41	2.88E-39
5747	4558.27	5.07	40.18	0.03	0.3	3.321928095	Up	7.77E-09	7.14E-08
170692	5913	0	18	0.01	0.1	3.321928095	Up	1.54E-06	1.07E-05
170961	4026	0	12.05	0.01	0.1	3.321928095	Up	0.000131537	0.000653674
n409339	4215	0	12.53	0.01	0.1	3.321928095	Up	0.000131537	0.000653581
5121	556	14	127	0.85	8.43	3.309997885	Up	1.37E-26	4.07E-25
n384750	9591	0	27.08	0.01	0.09	3.169925001	Up	1.97E-09	1.94E-08
n339785	7972	0	21.73	0.01	0.09	3.169925001	Up	1.67E-07	1.32E-06
MXLOC_004314	12236	3.63	31.36	0.01	0.09	3.169925001	Up	1.04E-07	8.36E-07
3043	626	7	57	0.37	3.32	3.165586066	Up	3.68E-12	4.70E-11
n369035	5849	61.79	495.77	0.32	2.86	3.159871337	Up	8.79E-95	1.27E-92
n379405	2381	7.99	61.58	0.1	0.88	3.137503524	Up	2.92E-13	4.10E-12
n368913	554	3.13	25.12	0.19	1.67	3.135776779	Up	5.02E-06	3.23E-05
n339647	2239	22	178	0.31	2.72	3.133266531	Up	4.81E-35	2.03E-33
n345973	415	1.85	15.21	0.16	1.4	3.129283017	Up	0.000133596	0.000662965
MXLOC_030586	2160	16.84	128.27	0.24	2.04	3.087462841	Up	1.74E-25	4.96E-24
n380153	638	3.68	27.38	0.19	1.56	3.037474705	Up	1.39E-06	9.75E-06
2917	4147	45	341	0.34	2.79	3.036658471	Up	7.80E-64	6.86E-62
MXLOC_030638	7456	0	17.51	0.01	0.08	3	Up	3.24E-06	2.15E-05
n335716	1115	4.79	35.18	0.14	1.11	2.987060944	Up	3.98E-08	3.37E-07
MXLOC_003020	1813.3	16.56	120.48	0.29	2.29	2.981222793	Up	2.52E-23	6.50E-22
n386555	1148	3.27	23.3	0.09	0.71	2.979822118	Up	1.78E-05	0.000104207
346562	1065	7	50	0.21	1.65	2.974004791	Up	2.88E-10	3.09E-09
n341082	554	9.3	65.01	0.56	4.33	2.950868293	Up	4.92E-13	6.79E-12
n384485	754	2.64	17.93	0.11	0.85	2.949959318	Up	0.000185455	0.00089867
6323	8100	17.99	129.71	0.07	0.54	2.94753258	Up	4.25E-25	1.19E-23
n337962	4245	24.97	171.1	0.18	1.37	2.928107082	Up	7.26E-32	2.71E-30
n384122	1381	20.21	138.87	0.46	3.5	2.927649156	Up	1.32E-25	3.79E-24
n339357	4455	50.05	341.65	0.35	2.6	2.893084796	Up	1.10E-60	9.02E-59
n337663	3411	8.77	58.9	0.08	0.59	2.882643049	Up	8.72E-12	1.08E-10
n385566	1510	26.7	179.19	0.56	4.11	2.875639662	Up	9.61E-33	3.71E-31
n344793	7281	9.16	62.21	0.04	0.29	2.857980995	Up	3.10E-12	4.00E-11
n337845	1100	4.58	29.29	0.13	0.94	2.854149134	Up	1.75E-06	1.21E-05
5552	1254	6	38	0.15	1.06	2.821029859	Up	1.10E-07	8.83E-07
n363314	1529	8	53	0.17	1.2	2.819427754	Up	1.86E-10	2.04E-09
3652	3191.47	15.82	132.57	0.2	1.41	2.817623258	Up	2.89E-27	8.89E-26
MXLOC_013226	2828.5	12.75	69.61	0.12	0.84	2.807354922	Up	2.49E-12	3.26E-11
n407986	1725	11.14	70.16	0.2	1.4	2.807354922	Up	3.77E-13	5.26E-12
n375398	2063	4.7	29.54	0.07	0.49	2.807354922	Up	1.75E-06	1.21E-05

113835	3522	12	80	0.11	0.77	2.807354922	Up		3.54E-15	5.63E-14
n405969	950	3.92	24.22	0.13	0.9	2.791413378	Up		9.47E-06	5.82E-05
n371502	822	2.7	17.55	0.11	0.76	2.788495895	Up		0.000185455	0.000898795
LXLOC_029508	8615	92.95	577.95	0.33	2.26	2.775784843	Up		4.07E-97	6.01E-95
MXLOC_016265	901	3.65	22.55	0.13	0.89	2.775293713	Up		3.34E-05	0.000186652
n380550	809	6.25	38.34	0.25	1.7	2.765534746	Up		1.10E-07	8.82E-07
128414	1430	5.13	30.34	0.11	0.74	2.750021747	Up		3.65E-06	2.41E-05
590	2447	42	255	0.53	3.56	2.747812976	Up		4.52E-43	2.43E-41
5055	1908	4	22	0.06	0.4	2.736965594	Up		0.000122815	0.000616364
n385523	1022	2.94	17.49	0.09	0.6	2.736965594	Up		0.000185455	0.000898546
n381793	2363	3.79	22.72	0.05	0.33	2.722466024	Up		3.34E-05	0.000186622
n386501	1009	8.38	50.96	0.27	1.78	2.720845929	Up		1.13E-09	1.15E-08
n382030	3218	26.47	155.61	0.25	1.64	2.713695815	Up		1.64E-26	4.88E-25
MXLOC_037006	3066	56.12	330	0.57	3.66	2.682809824	Up		2.42E-54	1.73E-52
344758	2323	53	308.35	0.71	4.54	2.676801368	Up		2.01E-50	1.29E-48
n369870	1527	5.73	33.38	0.12	0.76	2.662965013	Up		5.97E-07	4.38E-06
55809	7557	21.77	127.8	0.09	0.57	2.662965013	Up		3.74E-22	9.10E-21
126823	1328.08	11.99	67.64	0.28	1.77	2.660250628	Up		2.26E-12	2.96E-11
51337	2264	135	777	1.86	11.75	2.65928623	Up		6.70E-124	1.32E-121
n407773	2608	6.92	38.02	0.08	0.5	2.64385619	Up		1.10E-07	8.83E-07
n408250	3016	8.86	49.41	0.09	0.56	2.637429921	Up		2.06E-09	2.02E-08
7498	5717	12	62	0.06	0.37	2.624490865	Up		1.43E-10	1.59E-09
LXLOC_017108	1077	26.86	151.07	0.8	4.93	2.623515741	Up		1.71E-25	4.88E-24
n406199	770	6.01	34.38	0.26	1.6	2.621488377	Up		1.19E-06	8.42E-06
388336	7415	16.32	93.79	0.07	0.43	2.618909833	Up		3.00E-16	5.11E-15
401024	21054	214	1185	0.31	1.89	2.608046114	Up		1.35E-183	4.23E-181
79572	7328	103	566.04	0.43	2.6	2.596103058	Up		3.18E-88	4.14E-86
n406889	3499	13.95	74.34	0.12	0.72	2.584962501	Up		4.67E-13	6.45E-12
n406538	6814	0	12.82	0.01	0.06	2.584962501	Up		0.000131537	0.000655347
n383731	2213	5	27	0.07	0.42	2.584962501	Up		2.16E-05	0.000124505
n372975	1785	67.81	367.23	1.19	7.09	2.574824054	Up		1.09E-57	8.42E-56
LXLOC_009431	684.44	62.7	348.39	3.14	18.43	2.553219607	Up		1.32E-55	9.77E-54
n387357	660	52.42	279.96	2.63	15.41	2.550732157	Up		1.10E-43	5.95E-42
LXLOC_012354	1084	4.29	23	0.13	0.75	2.528378972	Up		6.79E-05	0.000358792
n384580	3699	11.62	62.26	0.1	0.57	2.510961919	Up		4.30E-11	5.01E-10
MXLOC_018931	3376	34.24	173.03	0.31	1.74	2.488747185	Up		8.92E-27	2.67E-25
n409766	2015	22.11	110.17	0.34	1.88	2.46712601	Up		2.31E-17	4.26E-16
LXLOC_023125	351	5.72	28.49	0.58	3.19	2.459431619	Up		1.20E-05	7.24E-05
MXLOC_019340	1653	15.53	78.66	0.3	1.64	2.450661409	Up		5.20E-13	7.15E-12
n363978	1304	21.33	106.1	0.52	2.84	2.449307401	Up		7.00E-17	1.24E-15
9877	4776	39.47	191.69	0.25	1.36	2.443606651	Up		1.11E-28	3.64E-27
154214	1784	6.37	30.48	0.11	0.59	2.423211431	Up		1.22E-05	7.35E-05
7620	1660	6.01	28.5	0.11	0.59	2.423211431	Up		3.79E-05	0.000209429
n341630	5684	5	27	0.03	0.16	2.415037499	Up		2.16E-05	0.000124525
n380110	1079	7.02	34.38	0.21	1.12	2.415037499	Up		3.85E-06	2.53E-05
137209	3276	34	164	0.32	1.7	2.409390936	Up		1.37E-24	3.72E-23
100528030	5496	19.52	94.22	0.11	0.58	2.398549376	Up		6.12E-15	9.60E-14
n408902	6136	16	76.42	0.08	0.42	2.392317423	Up		5.00E-12	6.34E-11
3773	4014	5	25	0.04	0.21	2.392317423	Up		6.88E-05	0.000363043
25942	6666.03	27.09	134.85	0.13	0.68	2.387023123	Up		9.69E-21	2.18E-19
MXLOC_032578	5027	98.9	473.12	0.61	3.18	2.382145618	Up		5.74E-68	5.51E-66
580	2607	28.52	132.55	0.34	1.74	2.355480655	Up		9.00E-20	1.90E-18
MXLOC_011005	2739	9.04	40.66	0.1	0.51	2.350497247	Up		1.18E-06	8.31E-06
3987	4447.99	16.98	80.75	0.12	0.61	2.345774837	Up		5.32E-13	7.31E-12
LXLOC_036869	1446.5	53.94	211.32	1	5.07	2.341985747	Up		4.80E-27	1.45E-25
79595	4173	65.08	305.26	0.49	2.48	2.339486466	Up		1.49E-43	8.08E-42
n378122	723	34.17	156.36	1.55	7.8	2.331205908	Up		1.13E-22	2.82E-21
n376379	1480	21.06	96.36	0.45	2.26	2.328325866	Up		1.83E-14	2.76E-13
n409080	1486	9.47	42.55	0.2	0.99	2.307428525	Up		3.88E-07	2.91E-06
MXLOC_006684	1116	23.28	105.34	0.67	3.31	2.304598216	Up		1.12E-15	1.84E-14

10586	2769	84	377	0.94	4.64	2.303392143	Up	9.66E-52	6.44E-50
64843	1798	8	36	0.14	0.69	2.301169535	Up	3.72E-06	2.45E-05
163081	2689	31.37	139.55	0.36	1.77	2.297680549	Up	4.89E-20	1.05E-18
79703	2323	65.19	292.73	0.88	4.31	2.29211244	Up	1.79E-40	8.90E-39
57646	4632	43.39	193.71	0.29	1.42	2.291766124	Up	2.90E-27	8.91E-26
n377692	1478	17.94	79.2	0.38	1.86	2.291231298	Up	2.83E-12	3.67E-11
MXLOC_022117	3148	39.24	173.85	0.39	1.88	2.269186633	Up	2.19E-24	5.85E-23
23527	7170	11.11	50.71	0.05	0.24	2.263034406	Up	3.95E-08	3.35E-07
n409761	5122	13.87	57.06	0.08	0.38	2.247927513	Up	7.00E-09	6.47E-08
26167	2905	394.73	1703.22	4.22	19.98	2.243241679	Up	7.14E-221	2.72E-218
794	1469	11	50	0.25	1.18	2.23878686	Up	3.95E-08	3.35E-07
1621	2760	6	27	0.07	0.33	2.237039197	Up	6.63E-05	0.000350777
200958	6356.07	28.89	124.02	0.14	0.66	2.237039197	Up	7.14E-18	1.35E-16
LXLOC_011347	291	12	51	1.52	7.12	2.227805918	Up	6.48E-08	5.36E-07
11166	2514	10	41.3	0.12	0.56	2.222392421	Up	1.89E-06	1.30E-05
MXLOC_007016	2508.7	235.53	824.95	2.41	11.24	2.221536984	Up	8.86E-90	1.20E-87
n378806	3519	65.94	280.26	0.58	2.7	2.218834602	Up	1.14E-37	5.19E-36
2122	5255.35	45.35	201.96	0.28	1.3	2.215012891	Up	3.13E-28	1.00E-26
n376384	813	28.27	117.1	1.13	5.15	2.18824966	Up	3.04E-16	5.19E-15
n378914	3505	9.65	42.63	0.09	0.41	2.187627003	Up	3.88E-07	2.91E-06
n373836	1775	14.93	60.83	0.26	1.18	2.182203331	Up	3.85E-09	3.67E-08
9924	5330.82	59.87	242.5	0.34	1.54	2.179323699	Up	1.52E-31	5.58E-30
MXLOC_013142	3435	20.56	87.09	0.19	0.86	2.178337241	Up	8.54E-13	1.15E-11
n344473	977	23	95	0.76	3.44	2.178337241	Up	2.45E-13	3.46E-12
57666	4548	17.64	71.89	0.12	0.54	2.169925001	Up	2.18E-10	2.37E-09
n411738	635	12.03	49.16	0.63	2.82	2.162271429	Up	1.89E-07	1.48E-06
n408279	1909	10.04	39.59	0.16	0.71	2.14974712	Up	5.46E-06	3.50E-05
MXLOC_012908	8796	40.03	161.07	0.14	0.62	2.146841388	Up	3.53E-21	8.10E-20
LXLOC_031802	3610.64	52	211	0.45	1.98	2.137503524	Up	1.82E-27	5.65E-26
n384112	1302	8.31	32.9	0.2	0.88	2.137503524	Up	3.28E-05	0.00018383
n380885	1075	18.15	71.78	0.54	2.35	2.121629444	Up	5.79E-10	6.03E-09
n410512	2365	17.32	68.8	0.23	1	2.120294234	Up	1.07E-09	1.08E-08
n410215	3510	56.37	223.87	0.5	2.17	2.117695043	Up	1.68E-28	5.44E-27
n386880	558	14.15	55.59	0.85	3.68	2.11417102	Up	5.51E-08	4.59E-07
MXLOC_007676	1680.16	99.79	395.83	1.89	8.14	2.10664256	Up	2.51E-49	1.56E-47
MXLOC_002274	1719	24.92	97.73	0.46	1.96	2.091147888	Up	2.28E-13	3.22E-12
n406571	1580	58.92	226.36	1.18	4.96	2.071553261	Up	2.38E-28	7.69E-27
MXLOC_011396	4485	54.8	210.66	0.38	1.59	2.064955442	Up	2.08E-26	6.17E-25
n338313	1739	48.71	185.01	0.88	3.67	2.060204634	Up	2.85E-23	7.34E-22
LXLOC_037803	10535	511.47	1932.29	1.49	6.17	2.049958159	Up	2.35E-223	9.16E-221
n378539	854	15.12	56.45	0.57	2.36	2.049753035	Up	8.34E-08	6.82E-07
84765	3034.21	49.59	191.71	0.52	2.15	2.047753131	Up	3.33E-24	8.81E-23
n407957	3814	73.22	273.46	0.59	2.43	2.042169454	Up	9.77E-33	3.77E-31
n377986	1638	14.81	54.29	0.28	1.15	2.038135129	Up	9.29E-08	7.54E-07
MXLOC_008193	6453	20.3	78.89	0.1	0.41	2.03562391	Up	1.01E-10	1.14E-09
n384690	2239	7.83	29.08	0.11	0.45	2.032421478	Up	6.16E-05	0.000329328
7840	12922	53.49	205.25	0.13	0.53	2.027480736	Up	1.06E-25	3.06E-24
1773	3098	13.05	48.26	0.13	0.53	2.027480736	Up	8.21E-07	5.90E-06
84976	4924	45.02	166.3	0.28	1.14	2.025535092	Up	2.89E-20	6.25E-19
n377474	1499	8	30	0.17	0.69	2.021061616	Up	9.43E-05	0.000484145
MXLOC_032582	2520	14.41	53.8	0.18	0.73	2.019899557	Up	1.56E-07	1.23E-06
n410795	2510	102.85	380.39	1.28	5.18	2.016808288	Up	8.88E-45	4.91E-43
n410632	5037	75.17	277.19	0.46	1.86	2.015596855	Up	7.93E-33	3.08E-31
3110	2133.7	69	254	1.01	4.08	2.014213859	Up	3.82E-30	1.32E-28
9369	4813.96	84.05	301.59	0.53	2.12	2	Up	1.44E-34	5.94E-33
n335640	318	7.72	27.99	0.88	3.52	2	Up	0.000179825	0.000873808
n384691	2730	10.37	38.48	0.12	0.48	2	Up	9.22E-06	5.68E-05
n379950	5246	16.22	61.83	0.1	0.4	2	Up	1.59E-08	1.41E-07
MXLOC_023334	5818	69.62	252.5	0.37	1.47	1.990218979	Up	1.04E-29	3.58E-28
MXLOC_022707	3379.73	562.51	1517.14	3.85	15.26	1.986824611	Up	2.52E-121	4.88E-119

n341107	1948	12.08	42.47	0.19	0.75	1.980891177	Up		7.15E-06	4.48E-05
n341941	524	22.4	80.67	1.45	5.72	1.979962247	Up		2.27E-10	2.47E-09
n410558	1426	7.8	27.08	0.17	0.66	1.956931278	Up		0.000179825	0.000873687
n338697	2190	11.97	42.04	0.17	0.66	1.956931278	Up		2.91E-06	1.94E-05
n408350	2091	63.91	226.75	0.96	3.72	1.95419631	Up		2.25E-26	6.67E-25
MXLOC_017801	3492	34.98	123.2	0.31	1.2	1.952694285	Up		3.88E-15	6.14E-14
56105	4706.88	77.19	294.57	0.55	2.12	1.946560741	Up		8.92E-36	3.85E-34
n406457	3267	34.69	122.18	0.33	1.27	1.944290567	Up		6.40E-15	1.00E-13
MXLOC_018997	1499	12.59	44.66	0.27	1.03	1.931613025	Up		2.59E-06	1.74E-05
80333	2242.63	416.97	1426.98	5.74	21.8	1.925205493	Up		1.45E-150	3.69E-148
MXLOC_010447	3394	118.87	409.33	1.08	4.1	1.924592597	Up		6.19E-45	3.44E-43
n387299	2391	25.57	87.36	0.33	1.25	1.921390165	Up		9.44E-11	1.07E-09
n410126	2269	14.12	47.33	0.19	0.71	1.901819606	Up		3.25E-06	2.16E-05
n408157	2410	26.39	89.58	0.34	1.27	1.901221846	Up		8.25E-11	9.37E-10
n377704	855	10.87	36.53	0.41	1.52	1.890375509	Up		2.59E-05	0.000147976
346007	3665.84	84	302	0.76	2.8	1.881355504	Up		8.77E-35	3.64E-33
LXLOC_012029	1483	22	74	0.47	1.73	1.880039376	Up		4.50E-09	4.24E-08
MXLOC_031565	1578	152.62	510.79	3.05	11.2	1.876617584	Up		7.61E-54	5.39E-52
3038	4219.3	12.27	41.64	0.09	0.33	1.874469118	Up		1.18E-05	7.14E-05
6579	7682	6.75	26.13	0.03	0.11	1.874469118	Up		0.000115295	0.000583977
MXLOC_019035	2134	61.76	204.34	0.9	3.28	1.865698908	Up		1.99E-22	4.89E-21
340267	3515	17.65	59.53	0.16	0.58	1.857980995	Up		1.08E-07	8.67E-07
147660	6741	256.67	848.51	1.17	4.24	1.857555735	Up		3.36E-87	4.27E-85
LXLOC_020892	878	10	33	0.37	1.34	1.856635825	Up		0.000117534	0.000594116
1278	5411	18	57	0.1	0.36	1.847996907	Up		6.52E-07	4.76E-06
8863	6203	62.52	204.49	0.31	1.11	1.840219556	Up		4.50E-22	1.09E-20
100129792	3491	86	277	0.76	2.7	1.828888084	Up		8.75E-29	2.87E-27
MXLOC_025384	737	10	32	0.44	1.56	1.8259706	Up		0.000192273	0.000929396
n409297	2912	24.23	78.96	0.26	0.92	1.823122238	Up		3.33E-09	3.20E-08
n335664	595	17.37	55.68	0.97	3.43	1.822151924	Up		7.67E-07	5.54E-06
n409269	6684	65.64	209.35	0.3	1.06	1.821029859	Up		4.64E-22	1.12E-20
n384135	2810	25.8	84.43	0.29	1.02	1.814444347	Up		4.13E-10	4.38E-09
n383218	11191	238.03	759.3	0.65	2.28	1.810522201	Up		3.72E-75	3.93E-73
MXLOC_008507	5309	52.51	164.95	0.3	1.05	1.807354922	Up		2.72E-17	4.97E-16
LXLOC_024308	1350	13	42	0.31	1.08	1.800691192	Up		1.66E-05	9.75E-05
7975	3350	18.42	56.92	0.17	0.58	1.770518154	Up		1.05E-06	7.47E-06
n368661	1413	35	108	0.78	2.66	1.769880217	Up		1.28E-11	1.57E-10
n385197	1765	12.2	38.16	0.22	0.75	1.769387072	Up		5.16E-05	0.000279695
n342356	3666	15.3	48	0.13	0.44	1.7589919	Up		4.52E-06	2.94E-05
MXLOC_023122	2648	72.21	221.81	0.85	2.86	1.750480401	Up		3.89E-22	9.46E-21
2487	2770	23	71	0.26	0.87	1.742503778	Up		4.24E-08	3.57E-07
n378672	1482	19.27	58.68	0.41	1.37	1.740480078	Up		8.85E-07	6.34E-06
MXLOC_017382	1709	19.81	59.61	0.36	1.2	1.736965594	Up		5.52E-07	4.07E-06
MXLOC_020459	1770	13.38	41.16	0.24	0.8	1.736965594	Up		2.69E-05	0.00015282
n406506	1074	19.68	59.83	0.59	1.96	1.732066795	Up		5.52E-07	4.06E-06
n333471	568	97.55	292.92	5.76	19	1.721858702	Up		3.88E-28	1.23E-26
LXLOC_031743	2263.51	38.79	102.28	0.47	1.55	1.721535554	Up		1.63E-09	1.63E-08
MXLOC_018325	6195	28.27	83.63	0.14	0.46	1.716207034	Up		7.00E-09	6.46E-08
LXLOC_028925	2781	28	82	0.31	1.01	1.704015172	Up		1.11E-08	9.97E-08
64282	8091.49	157.55	468.01	0.6	1.95	1.700439718	Up		1.27E-43	6.88E-42
286077	5587	36.8	107.44	0.2	0.65	1.700439718	Up		4.32E-11	5.03E-10
374879	2094	33.11	96.79	0.49	1.59	1.698173111	Up		7.22E-10	7.46E-09
4298	4505	90.28	267.29	0.62	2.01	1.696855381	Up		1.91E-25	5.42E-24
3305	2537	28.76	83.82	0.35	1.13	1.690895945	Up		7.00E-09	6.47E-08
LXLOC_002619	3076.62	6.85	52.44	0.18	0.58	1.688055994	Up		1.83E-11	2.21E-10
283316	4583	27	79	0.18	0.58	1.688055994	Up		2.09E-08	1.83E-07
2173	1005	112	328	3.58	11.52	1.686109224	Up		1.93E-30	6.75E-29
MXLOC_012365	3139	35.51	103.58	0.35	1.12	1.678071905	Up		1.29E-10	1.44E-09
9806	5246.98	2708.65	8197.81	16.54	52.8	1.674578695	Up		0	0
MXLOC_021728	2079	29.33	84.84	0.44	1.4	1.669851398	Up		9.21E-09	8.38E-08

LXLOC_034997	5400.26	243.93	594.72	1.17	3.72	1.668794092	Up		2.29E-42	1.21E-40
LXLOC_034066	3676	435.7	1255.8	3.67	11.6	1.660272837	Up		1.31E-109	2.17E-107
n380023	1972	192.34	552.31	3.05	9.63	1.658726555	Up		5.23E-49	3.21E-47
MXLOC_014171	4715	63.58	183.66	0.42	1.32	1.652076697	Up		1.67E-17	3.10E-16
n345975	1261	26.64	76	0.67	2.1	1.648156327	Up		3.97E-08	3.36E-07
n342849	4011	30	85.35	0.23	0.72	1.646363045	Up		1.20E-08	1.08E-07
n337723	1036	65.04	185.76	2.02	6.32	1.645569265	Up		2.84E-17	5.17E-16
MXLOC_016625	5262	93.78	268.26	0.55	1.72	1.644905041	Up		1.07E-24	2.91E-23
n380253	921	49.33	140.15	1.73	5.4	1.642187369	Up		1.75E-13	2.49E-12
n387668	1914	67.56	192.79	1.11	3.46	1.640212361	Up		5.19E-18	9.85E-17
LXLOC_016769	5719.02	37.48	95.61	0.18	0.56	1.637429921	Up		1.69E-08	1.50E-07
MXLOC_017682	1528	97.69	277.09	2.02	6.28	1.636409266	Up		3.32E-25	9.30E-24
LXLOC_032728	1902	19	53	0.31	0.96	1.63076619	Up		8.81E-06	5.46E-05
730	4012	111.17	315	0.86	2.66	1.629017681	Up		3.10E-28	9.92E-27
n406834	1455	61.05	171.72	1.33	4.1	1.624197664	Up		8.47E-16	1.40E-14
n409323	5198	22.68	61.2	0.13	0.4	1.621488377	Up		2.00E-06	1.37E-05
1871	5009	61.42	171.15	0.38	1.16	1.610053482	Up		8.47E-16	1.40E-14
n410727	3560	22.52	63.94	0.2	0.61	1.608809243	Up		8.12E-07	5.84E-06
n377334	579	21.67	60.04	1.25	3.81	1.607862903	Up		1.55E-06	1.07E-05
286	7757.89	59.47	182.06	0.26	0.79	1.60334103	Up		1.36E-18	2.68E-17
MXLOC_007628	1743	33.98	93.5	0.61	1.85	1.600644123	Up		2.78E-09	2.69E-08
8641	4578	1631	4505.78	10.99	33.31	1.599763968	Up		0	0
MXLOC_000667	4024	253.44	701.59	1.95	5.91	1.599684006	Up		5.33E-59	4.20E-57
26093	2078	24.72	68.1	0.37	1.12	1.597901556	Up		3.53E-07	2.67E-06
221120	1569	112.35	309.98	2.26	6.84	1.597673552	Up		8.55E-27	2.57E-25
n338114	1109	15	41	0.43	1.3	1.596103058	Up		0.000118249	0.00059747
MXLOC_007039	3442	69.81	192.94	0.63	1.9	1.592575685	Up		2.10E-17	3.86E-16
MXLOC_010997	5445	114.89	315.86	0.65	1.96	1.592342031	Up		2.46E-27	7.58E-26
LXLOC_018437	747	19	52	0.83	2.5	1.590744853	Up		1.38E-05	8.25E-05
LXLOC_016703	13912	0	12.48	0.01	0.03	1.584962501	Up		0.000131537	0.000655721
n410677	946	14.73	40.05	0.5	1.5	1.584962501	Up		9.18E-05	0.00047279
n340736	3341	26.59	74	0.25	0.75	1.584962501	Up		9.83E-08	7.95E-07
1770	13750	0	13	0.01	0.03	1.584962501	Up		6.27E-05	0.00033417
151790	2594	62.95	172.13	0.76	2.27	1.578620974	Up		1.09E-15	1.79E-14
n338222	1845	35.05	95.64	0.6	1.79	1.576925182	Up		4.57E-09	4.31E-08
342865	1243	44	120	1.13	3.37	1.576425819	Up		3.72E-11	4.36E-10
2317	9395	296.54	806.98	0.97	2.89	1.57501284	Up		4.47E-66	4.16E-64
LXLOC_009214	1186	34	91	0.91	2.69	1.563667722	Up		1.33E-08	1.19E-07
MXLOC_006159	8908	313.91	845.28	1.08	3.19	1.562525112	Up		2.05E-68	1.99E-66
MXLOC_033987	4354.46	67.46	181.1	0.48	1.41	1.554588852	Up		6.44E-16	1.08E-14
56104	4590	24.07	64.02	0.16	0.47	1.554588852	Up		2.08E-06	1.42E-05
MXLOC_019270	4263	64.89	173.39	0.47	1.38	1.553935605	Up		2.73E-15	4.38E-14
n405666	2472	106	284	1.34	3.93	1.552296312	Up		7.29E-24	1.91E-22
n363958	532	17	45	1.08	3.14	1.539733247	Up		7.82E-05	0.000408417
n411679	2015	20.72	54.58	0.32	0.93	1.539158811	Up		1.13E-05	6.84E-05
n405260	3164	31.1	80.62	0.3	0.87	1.5360529	Up		2.09E-07	1.62E-06
256949	2787	23	61	0.26	0.75	1.528378972	Up		3.94E-06	2.59E-05
LXLOC_029712	1992.89	73.06	154.83	0.93	2.67	1.521537121	Up		9.51E-10	9.70E-09
27109	1790.79	15.51	43.28	0.29	0.83	1.517058436	Up		4.77E-05	0.000259694
n342987	9848	386.1	1003.43	1.2	3.43	1.515174171	Up		8.52E-77	9.39E-75
MXLOC_010585	3052	39.61	102.52	0.4	1.14	1.510961919	Up		3.15E-09	3.04E-08
MXLOC_006554	2745	82.42	212.83	0.93	2.64	1.505235308	Up		2.15E-17	3.96E-16
85363	3387.14	82.85	226.56	0.8	2.27	1.504620392	Up		5.47E-20	1.17E-18
441024	2361	63.92	164.05	0.84	2.38	1.502500341	Up		6.58E-14	9.59E-13
n340568	2698	98.31	251.13	1.13	3.18	1.492703993	Up		4.58E-20	9.81E-19
n384835	1495	20.44	52.28	0.43	1.21	1.492598483	Up		2.70E-05	0.000153282
202	7553	51.82	131.2	0.21	0.59	1.490325627	Up		3.30E-11	3.88E-10
50488	4935.85	201.43	508.99	1.25	3.49	1.481298942	Up		2.90E-38	1.36E-36
LXLOC_029908	4703	532.72	1352.63	3.49	9.73	1.479212769	Up		1.07E-99	1.61E-97
MXLOC_017513	5010	162.85	411.75	1	2.78	1.475084883	Up		1.94E-31	7.09E-30

MXLOC_003584	2739	116.58	293.86	1.32	3.66	1.471305719	Up	9.86E-23	2.48E-21
n405970	3146	6268.61	15829.94	61.78	171.2	1.470470926	Up	0	0
4793	1289.16	38.11	89.13	0.87	2.41	1.46994584	Up	3.61E-07	2.71E-06
283989	1941	61.65	154.63	1	2.74	1.454175893	Up	1.21E-12	1.61E-11
n407569	1648	32.6	80.7	0.62	1.69	1.446683126	Up	3.92E-07	2.93E-06
MXLOC_034702	8924	81.93	201.64	0.28	0.76	1.440572591	Up	1.08E-15	1.78E-14
56130	3030	26.02	64.89	0.27	0.73	1.434937057	Up	7.46E-06	4.67E-05
MXLOC_017496	807.36	45.16	102.49	1.68	4.54	1.434231065	Up	1.13E-07	9.10E-07
n410445	773	31.85	78.29	1.35	3.63	1.427010141	Up	4.84E-07	3.59E-06
MXLOC_024112	2856	10.52	35.95	0.16	0.43	1.426264755	Up	4.32E-05	0.000236493
n385357	3092	69.57	170.64	0.7	1.88	1.425305835	Up	2.28E-13	3.23E-12
LXLOC_025421	1395	19	46	0.43	1.15	1.419225296	Up	0.000182147	0.000883864
n407929	2424	17.56	43.41	0.23	0.61	1.407175382	Up	0.000185865	0.000900282
n381079	1036	63.47	153.02	1.97	5.2	1.400315994	Up	6.11E-12	7.68E-11
n384107	1499	43.46	104.56	0.92	2.42	1.395301281	Up	1.64E-08	1.46E-07
83481	7516	79.07	188.36	0.32	0.84	1.392317423	Up	6.11E-14	8.93E-13
55066	7999	61.78	150.47	0.24	0.63	1.392317423	Up	6.23E-12	7.82E-11
8403	2043	24	58	0.37	0.97	1.390459477	Up	2.64E-05	0.000150174
7018	2791	33	79	0.37	0.97	1.390459477	Up	1.08E-06	7.67E-06
MXLOC_000717	5836.41	635.98	1364.83	3.01	7.89	1.390261813	Up	8.44E-77	9.33E-75
154313	2215	35.61	84.89	0.5	1.31	1.389566812	Up	4.66E-07	3.47E-06
797	1031	144	343	4.48	11.73	1.388632376	Up	3.19E-24	8.44E-23
MXLOC_025213	5767	48.65	116.04	0.26	0.68	1.387023123	Up	2.51E-09	2.44E-08
4808	2509.54	218	518	2.7	7.05	1.38466385	Up	1.27E-35	5.43E-34
n385823	1662	531.65	1260.42	10.07	26.2	1.379503128	Up	5.67E-84	6.92E-82
84517	2005	29	68	0.45	1.17	1.378511623	Up	8.71E-06	5.40E-05
n381595	1241	51.91	122.67	1.33	3.45	1.375170116	Up	1.32E-09	1.32E-08
n385954	624	23.54	55.38	1.25	3.24	1.374065718	Up	4.95E-05	0.000268952
MXLOC_010627	6483	138.95	328.41	0.66	1.71	1.373458396	Up	3.70E-23	9.46E-22
MXLOC_006078	4638	25.46	60.75	0.17	0.44	1.371968777	Up	2.11E-05	0.000122209
7772	4964	322.21	757.5	2	5.16	1.367371066	Up	1.96E-50	1.26E-48
n386373	1337	29.08	68.37	0.69	1.78	1.367208974	Up	8.71E-06	5.40E-05
MXLOC_024593	2804	48.58	114.37	0.54	1.39	1.364053571	Up	5.58E-09	5.21E-08
8681	3306.8	47.61	109.86	0.44	1.13	1.360747344	Up	2.27E-08	1.98E-07
n376385	1272	156.34	366.1	3.91	10.04	1.360518757	Up	3.67E-25	1.02E-23
n344646	3137	30.22	71.4	0.3	0.77	1.359895945	Up	4.67E-06	3.03E-05
LXLOC_001859	2735	34.59	79.92	0.39	1	1.358453971	Up	1.93E-06	1.32E-05
LXLOC_034753	201	343.73	798.15	69.48	177.64	1.354286817	Up	3.32E-52	2.25E-50
LXLOC_037082	6321	22.8	51.84	0.11	0.28	1.347923303	Up	0.000139303	0.000688751
LXLOC_020219	2306	24.1	56.52	0.33	0.84	1.347923303	Up	5.94E-05	0.000318386
n408896	4462	183.2	426.03	1.27	3.23	1.346705668	Up	1.12E-28	3.66E-27
3709	12568	106	246	0.26	0.66	1.343954401	Up	3.87E-17	6.99E-16
MXLOC_014325	2205	60.93	140.46	0.86	2.18	1.34191957	Up	1.88E-10	2.06E-09
MXLOC_019206	6078	29	68.47	0.15	0.38	1.341036918	Up	8.71E-06	5.40E-05
n407062	5924	28.27	66.79	0.15	0.38	1.341036918	Up	1.09E-05	6.62E-05
MXLOC_004813	4769.26	302.99	709.1	1.99	5.03	1.337789969	Up	3.04E-47	1.79E-45
MXLOC_017110	7555	669.67	1526.33	2.72	6.81	1.324048147	Up	4.62E-95	6.74E-93
n387356	296	39.86	90.48	4.96	12.38	1.319599289	Up	4.29E-07	3.20E-06
57509	4064.8	315	693	2.32	5.78	1.316944687	Up	1.43E-41	7.38E-40
n365072	987	46.91	106.33	1.53	3.81	1.316259345	Up	4.18E-08	3.53E-07
MXLOC_008195	4014.25	348.56	843.08	2.86	7.12	1.315862094	Up	1.34E-58	1.05E-56
5138	4312	362.54	817.42	2.59	6.42	1.309621199	Up	7.67E-51	5.01E-49
n411132	1338	72.21	162.69	1.71	4.23	1.306661338	Up	2.93E-11	3.46E-10
n381294	2352	27	61	0.36	0.89	1.30580843	Up	4.47E-05	0.000244507
n341717	4630	189.3	426.06	1.26	3.11	1.303490847	Up	2.98E-27	9.13E-26
MXLOC_000224	6501	144.98	326.9	0.69	1.7	1.300866479	Up	2.25E-21	5.26E-20
23218	8827	111.64	253.04	0.39	0.96	1.299560282	Up	4.15E-17	7.48E-16
n375746	1225	22.67	50.98	0.59	1.45	1.297266041	Up	0.00020748	0.000996849
83943	1519	120	268	2.49	6.11	1.295026638	Up	1.81E-17	3.36E-16
293	1484	1008	2250	21.46	52.59	1.293138421	Up	3.02E-134	6.70E-132

6490	2173.77	412	921	5.93	14.53	1.292930693	Up	3.48E-56	2.60E-54
LXLOC_018802	1763	35.56	78.52	0.63	1.54	1.289506617	Up	4.96E-06	3.20E-05
LXLOC_017625	11008	81.23	182.42	0.23	0.56	1.283792966	Up	1.90E-12	2.50E-11
MXLOC_013722	4803	65.64	144.86	0.42	1.02	1.280107919	Up	6.24E-10	6.47E-09
MXLOC_025208	2950.3	190.05	418.87	2	4.84	1.275007047	Up	9.93E-26	2.86E-24
n407691	1336	169.14	372.61	4.02	9.71	1.272275794	Up	4.30E-23	1.09E-21
n375748	970	83.33	183.39	2.77	6.69	1.272120235	Up	3.77E-12	4.82E-11
28227	2426	100	219	1.28	3.09	1.271463028	Up	4.20E-14	6.20E-13
n376381	2792	65.7	144.41	0.73	1.76	1.26960706	Up	6.24E-10	6.47E-09
n410507	6909	79.64	172.55	0.35	0.84	1.263034406	Up	2.79E-11	3.29E-10
286204	5641	292	636	1.59	3.81	1.260764232	Up	1.21E-37	5.52E-36
n341210	1707	47	103	0.87	2.08	1.257496222	Up	2.27E-07	1.76E-06
MXLOC_031376	2447	112.24	244.18	1.43	3.41	1.253756592	Up	2.05E-15	3.32E-14
LXLOC_028959	2385	32	70	0.42	1	1.251538767	Up	2.10E-05	0.000121538
8731	6179	1113.77	2408.76	5.54	13.15	1.247104918	Up	7.04E-136	1.59E-133
MXLOC_030078	6435.59	162.11	343.71	0.76	1.8	1.243925583	Up	4.68E-20	1.00E-18
89884	1879	82	177	1.37	3.24	1.24181792	Up	2.07E-11	2.48E-10
92949	6746.46	267	577	1.22	2.88	1.239187664	Up	7.61E-34	3.06E-32
n384200	1447	67.06	144.6	1.47	3.47	1.239119508	Up	1.76E-09	1.75E-08
MXLOC_024663	5893.04	417.5	891.45	2.17	5.11	1.235628248	Up	2.59E-50	1.66E-48
26240	1618	106	227	2.06	4.85	1.23534041	Up	4.81E-14	7.08E-13
n407101	1613	59.72	128.31	1.17	2.75	1.232923089	Up	1.06E-08	9.57E-08
n387663	8619	65.1	137.03	0.23	0.54	1.231325546	Up	8.23E-09	7.54E-08
MXLOC_008063	6159	105.19	225.81	0.53	1.24	1.226275856	Up	6.01E-14	8.78E-13
MXLOC_011281	2784	227.5	485	2.54	5.94	1.225634434	Up	3.77E-28	1.20E-26
MXLOC_002525	4502	438.09	931.16	3	7	1.222392421	Up	5.29E-52	3.57E-50
10788	5769	40	83	0.21	0.49	1.222392421	Up	1.02E-05	6.23E-05
374654	4558	126.13	266.36	0.85	1.98	1.219965684	Up	8.14E-16	1.35E-14
MXLOC_022160	2860.96	34.6	71.96	0.37	0.86	1.216811389	Up	4.05E-05	0.000223209
151313	1265	54.05	114.71	1.36	3.16	1.216317907	Up	1.41E-07	1.12E-06
LXLOC_019967	11474.36	996	2110.18	2.66	6.18	1.216180592	Up	7.98E-115	1.41E-112
9016	1592	32.66	69.38	0.65	1.51	1.216036926	Up	3.07E-05	0.000172979
MXLOC_021993	5363	364.56	767.94	2.09	4.84	1.211504105	Up	1.78E-42	9.46E-41
MXLOC_009471	4158	50.87	107.43	0.38	0.88	1.211504105	Up	2.43E-07	1.87E-06
114781	8819.42	430.27	909.79	1.5	3.47	1.209973162	Up	2.59E-50	1.66E-48
1536	4318	54	115	0.39	0.9	1.206450877	Up	9.83E-08	7.95E-07
2115	6607.35	205.5	429.7	0.95	2.19	1.204931451	Up	3.47E-24	9.17E-23
n410527	2699	69.83	145.66	0.8	1.84	1.201633861	Up	3.35E-09	3.21E-08
7789	5204	124.8	262.25	0.74	1.7	1.199937571	Up	1.27E-15	2.08E-14
MXLOC_009095	8866	69.3	145.21	0.24	0.55	1.196397213	Up	3.35E-09	3.21E-08
MXLOC_009809	7654	308.75	644.48	1.24	2.84	1.195550809	Up	1.96E-35	8.33E-34
338917	2995	30.39	62.48	0.31	0.71	1.195550809	Up	0.00014771	0.000727438
5570	1872.19	1671.15	3480.15	27.96	63.99	1.194482106	Up	3.88E-182	1.20E-179
n338713	906	36.88	77.18	1.32	3.02	1.19401062	Up	1.23E-05	7.39E-05
83449	3461.36	82.19	170.39	0.73	1.67	1.193879734	Up	2.58E-10	2.79E-09
207063	2577	96.49	201.33	1.17	2.67	1.190331212	Up	3.86E-12	4.93E-11
404734	8349	146.01	304.43	0.54	1.23	1.187627003	Up	1.95E-17	3.59E-16
n383882	1680	36.88	76.22	0.69	1.57	1.186096292	Up	1.77E-05	0.000103755
MXLOC_033085	6169	96.95	198.36	0.48	1.09	1.183221824	Up	1.12E-11	1.37E-10
n381061	1160	36.17	75	1	2.27	1.182692298	Up	2.56E-05	0.000145938
3780	2657	168.03	347.71	1.97	4.47	1.182079202	Up	2.09E-19	4.31E-18
MXLOC_012901	7168	69.06	145.05	0.3	0.68	1.180572246	Up	3.35E-09	3.21E-08
11252	3193.8	210.55	426.82	2.01	4.55	1.178671044	Up	1.06E-22	2.65E-21
n337667	3179	38.54	80.15	0.38	0.86	1.178337241	Up	1.12E-05	6.81E-05
MXLOC_031942	2010	56.34	116.21	0.88	1.99	1.177193002	Up	1.85E-07	1.45E-06
4267	1235	85	175	2.19	4.95	1.176497655	Up	1.87E-10	2.05E-09
5625	2163.8	71.28	148.45	1.04	2.35	1.176077228	Up	3.05E-09	2.94E-08
388407	1530	70	143	1.44	3.24	1.169925001	Up	1.11E-08	9.97E-08
MXLOC_007163	5223.25	10.24	55.17	0.16	0.36	1.169925001	Up	7.44E-10	7.68E-09
n341713	2847	29	60	0.32	0.72	1.169925001	Up	0.000186517	0.00090319

219749	3719	782.01	1599.9	6.5	14.6	1.167456746	Up	8.03E-82	9.47E-80
56139	4014.27	100.36	207.81	0.78	1.75	1.165808893	Up	3.21E-12	4.13E-11
5649	11571	191	394	0.51	1.14	1.160464672	Up	8.98E-22	2.14E-20
2668	3747.05	223.25	451.49	1.83	4.09	1.160257195	Up	8.34E-24	2.18E-22
n376189	2050	55.12	111.69	0.84	1.87	1.154577037	Up	6.69E-07	4.88E-06
n384586	1204	37.14	74.95	0.98	2.18	1.153474481	Up	5.93E-05	0.000318051
26974	2686	269	544.19	3.11	6.91	1.15177113	Up	2.22E-28	7.17E-27
57556	6860	859.27	1740.02	3.85	8.55	1.151065974	Up	2.98E-87	3.79E-85
MXLOC_035519	3436	91.41	184.02	0.82	1.82	1.150242636	Up	1.38E-10	1.54E-09
23328	7709	150.11	303.32	0.6	1.33	1.14839184	Up	1.83E-16	3.15E-15
LXLOC_017539	16716.53	1036.19	2149.75	1.95	4.32	1.147557188	Up	2.22E-112	3.79E-110
MXLOC_021978	1346	215.87	435.66	5.09	11.27	1.146749954	Up	4.91E-23	1.25E-21
n407183	2827	34.47	69.96	0.38	0.84	1.144389909	Up	8.35E-05	0.000433583
55532	2854	57.06	114.39	0.62	1.37	1.143835773	Up	6.01E-07	4.40E-06
MXLOC_017726	2831	123.89	249.38	1.36	3	1.141355849	Up	7.55E-14	1.09E-12
MXLOC_028197	14958	358.29	715.72	0.73	1.61	1.141092319	Up	6.72E-36	2.90E-34
148156	3014	194.42	390.79	2	4.41	1.140778656	Up	1.45E-20	3.20E-19
LXLOC_024484	2407.61	531.96	740.23	4.78	10.52	1.138052181	Up	5.13E-14	7.54E-13
MXLOC_022741	6774	205.1	408.97	0.93	2.04	1.133266531	Up	4.87E-21	1.11E-19
LXLOC_028960	1302	34	68	0.83	1.82	1.132755209	Up	0.00011904	0.000601033
MXLOC_018039	1055	174.27	348.26	5.3	11.62	1.132545804	Up	2.43E-18	4.71E-17
MXLOC_005083	6286	213.16	424.01	1.04	2.28	1.132450296	Up	8.24E-22	1.97E-20
n375462	2365	76.87	152.77	1.01	2.21	1.129691077	Up	8.08E-09	7.41E-08
n409237	3863	39.63	79.48	0.32	0.7	1.129283017	Up	2.62E-05	0.000149491
n371302	4593	47.35	95.46	0.32	0.7	1.129283017	Up	4.16E-06	2.72E-05
4842	12183	108	214	0.27	0.59	1.127755547	Up	1.19E-11	1.46E-10
153090	4924.89	183.76	403.99	1.27	2.77	1.125057479	Up	6.64E-25	1.83E-23
MXLOC_011992	4960	315.55	625.99	1.96	4.27	1.123382416	Up	3.46E-31	1.25E-29
n409520	3342	48.66	96.78	0.45	0.98	1.122856748	Up	4.70E-06	3.05E-05
LXLOC_014925	2277.71	494.3	974.29	6.73	14.65	1.122222255	Up	4.69E-47	2.75E-45
n377792	878	109.08	216.34	4.03	8.77	1.121797004	Up	9.54E-12	1.18E-10
LXLOC_005493	2357.88	258.74	463.06	3.09	6.72	1.120854395	Up	4.52E-19	9.17E-18
MXLOC_021574	4578	388.64	769.76	2.62	5.69	1.118861841	Up	6.64E-38	3.05E-36
741	1296	34	67	0.83	1.8	1.116813665	Up	0.000168996	0.000824274
MXLOC_009665	8821	90.07	174.71	0.31	0.67	1.11189288	Up	2.60E-09	2.52E-08
LXLOC_017611	2753	138.56	272.04	1.56	3.37	1.111202562	Up	2.85E-14	4.26E-13
89958	3833	281.91	553.84	2.27	4.9	1.110089452	Up	2.44E-27	7.51E-26
10656	1957	690.54	1356.9	11.05	23.84	1.109337866	Up	2.22E-64	1.98E-62
8225	1180	289	566	7.81	16.79	1.104207777	Up	1.12E-27	3.50E-26
n341449	2069	36	70	0.54	1.16	1.103093493	Up	0.000149819	0.000736582
n377400	780	56.14	109.63	2.35	5.04	1.100762977	Up	2.10E-06	1.44E-05
MXLOC_031378	846	351.26	685.89	13.49	28.91	1.099678261	Up	6.49E-33	2.52E-31
n409307	3250	293.41	573.67	2.8	6	1.099535674	Up	6.44E-28	2.03E-26
79955	2801.16	451.07	769.18	4.37	9.36	1.09887525	Up	4.79E-27	1.45E-25
MXLOC_002774	7329	493.58	963.2	2.07	4.43	1.097675931	Up	1.14E-45	6.50E-44
7372	6738	110.1	214.7	0.5	1.07	1.097610797	Up	2.91E-11	3.44E-10
MXLOC_013588	5350.6	323.03	467.19	1.38	2.95	1.096046687	Up	1.20E-10	1.35E-09
n379194	889	59.88	116.58	2.18	4.66	1.09600182	Up	7.56E-07	5.47E-06
3303	2429	1136.31	2213.44	14.58	31.16	1.095704514	Up	5.19E-102	8.12E-100
56099	3887.05	471.97	1081.57	4.42	9.44	1.09474049	Up	1.44E-68	1.41E-66
MXLOC_017673	6345	77.35	147.78	0.37	0.79	1.094327383	Up	6.78E-08	5.59E-07
LXLOC_011401	862.17	45.86	86.62	1.68	3.58	1.091498354	Up	3.67E-05	0.000203682
10626	2876	363.02	704.31	3.92	8.35	1.090922543	Up	2.36E-33	9.37E-32
n409082	3121	172.53	334.1	1.71	3.64	1.089942125	Up	1.07E-16	1.88E-15
54477	4287.99	294.4	521.43	1.94	4.12	1.086587685	Up	1.17E-20	2.60E-19
n345879	10462	193.88	377.91	0.57	1.21	1.085973223	Up	7.25E-19	1.46E-17
64714	1726	173.74	335.15	3.16	6.7	1.084236537	Up	1.19E-16	2.09E-15
MXLOC_000092	2329	44.72	86.38	0.6	1.27	1.081794091	Up	2.35E-05	0.000134917
LXLOC_018508	5597.6	141.85	274.28	0.78	1.65	1.080919995	Up	5.53E-14	8.11E-13
MXLOC_013713	16108	553.86	1065.03	1.05	2.22	1.080170349	Up	7.36E-49	4.50E-47

MXLOC_032064	13892	503.81	968.32	1.11	2.34	1.075948853	Up		1.68E-44	9.27E-43
9189	4555.1	904.52	1741.67	6.14	12.94	1.075527057	Up		9.88E-79	1.11E-76
MXLOC_017734	7432	112.79	218.5	0.47	0.99	1.074767768	Up		1.86E-11	2.24E-10
LXLOC_031327	1655	883	1694	16.8	35.37	1.074064986	Up		5.01E-76	5.40E-74
157638	5481	102.03	194	0.57	1.2	1.074000581	Up		6.52E-10	6.75E-09
LXLOC_020885	649	238	456	12.15	25.56	1.072931522	Up		1.23E-21	2.90E-20
7386	1224	1295	2480	33.69	70.82	1.071836415	Up		5.28E-110	8.85E-108
LXLOC_037624	4314.42	370.15	719.42	2.69	5.65	1.070644695	Up		3.63E-34	1.47E-32
n376378	2483	372.74	713.17	4.68	9.82	1.069214495	Up		6.03E-33	2.35E-31
286319	2470	318	608	4.01	8.41	1.068503564	Up		3.42E-28	1.09E-26
7694	2631.24	100.72	149.99	0.93	1.95	1.068171503	Up		0.00012206	0.000613101
MXLOC_026891	1530	180.6	344.91	3.73	7.81	1.066146918	Up		1.29E-16	2.25E-15
56121	2656	362.26	690.51	4.24	8.87	1.06486984	Up		1.44E-31	5.28E-30
286075	2114	40	76	0.59	1.23	1.059871456	Up		0.000116505	0.000589594
n411756	2271	249.49	473.8	3.43	7.15	1.059734665	Up		5.40E-22	1.30E-20
MXLOC_018546	1276	43.13	81.38	1.07	2.23	1.059432914	Up		8.12E-05	0.000422822
11013	666	454.21	862.08	22.55	46.99	1.059226334	Up		1.07E-38	5.07E-37
83606	3607	85.41	161.99	0.73	1.52	1.058102955	Up		2.15E-08	1.87E-07
23095	9256.74	811.89	1724.61	3.02	6.27	1.053916893	Up		9.15E-95	1.32E-92
n382021	3681	101.62	190.7	0.85	1.76	1.050040682	Up		1.56E-09	1.56E-08
n333485	866	53.46	100.72	2	4.14	1.049630768	Up		1.14E-05	6.89E-05
MXLOC_018456	4047.64	293.63	550.55	2.23	4.61	1.047723041	Up		1.09E-24	2.97E-23
n408151	3124	45.74	85.82	0.45	0.93	1.047305715	Up		5.12E-05	0.000277334
23196	4957	221.31	418.37	1.38	2.85	1.046293652	Up		1.91E-19	3.96E-18
LXLOC_033404	9624	99.59	187.71	0.32	0.66	1.044394119	Up		1.77E-09	1.75E-08
84002	4123	116.8	217.79	0.87	1.79	1.040872281	Up		1.43E-10	1.58E-09
n411760	2318	70.28	131.78	0.95	1.95	1.037474705	Up		6.33E-07	4.63E-06
n409709	2195	197.47	368.55	2.81	5.76	1.035498681	Up		7.29E-17	1.29E-15
LXLOC_012519	3619	54	100	0.46	0.94	1.031026896	Up		1.73E-05	0.000101488
11060	4057.72	119.81	236.67	0.97	1.98	1.029443778	Up		1.02E-12	1.36E-11
57495	7477	317	587	1.3	2.65	1.027480736	Up		1.76E-25	4.99E-24
n376493	4357	791.44	1470.72	5.61	11.43	1.026752728	Up		9.17E-62	7.74E-60
285	5267.99	46.99	85.85	0.27	0.55	1.026472211	Up		7.83E-05	0.000409102
MXLOC_001250	1461.52	90.11	170.32	1.99	4.04	1.021586862	Up		9.64E-09	8.75E-08
MXLOC_029837	3299	71.42	132.05	0.67	1.36	1.021373651	Up		6.96E-07	5.06E-06
n377254	557	205.09	378.55	12.37	25.08	1.019691848	Up		8.18E-17	1.44E-15
23359	4707	1057.45	1954.01	6.93	14.05	1.019642873	Up		1.59E-80	1.83E-78
7556	4405	111.33	205.11	0.78	1.58	1.018378529	Up		8.19E-10	8.43E-09
MXLOC_026877	4956	63.54	118.4	0.4	0.81	1.017921908	Up		2.24E-06	1.52E-05
51095	2276	370	682	5.07	10.26	1.016973079	Up		4.78E-29	1.59E-27
27098	1869.65	51.68	95.49	0.87	1.76	1.016488123	Up		2.49E-05	0.000142291
MXLOC_006989	5011	148.46	272.68	0.91	1.84	1.015767316	Up		1.98E-12	2.61E-11
9014	2306	75.5	138.92	1.02	2.06	1.014075185	Up		5.34E-07	3.94E-06
727851	7299	609.85	1124.29	2.57	5.19	1.013966179	Up		6.48E-47	3.79E-45
2118	2327.24	261.01	477.65	3.49	7.03	1.010297653	Up		2.05E-20	4.50E-19
MXLOC_015714	11509	262.46	481.5	0.7	1.41	1.010268335	Up		8.91E-21	2.00E-19
1786	5356.94	953.09	1745.37	5.47	11.01	1.009201731	Up		1.51E-70	1.51E-68
LXLOC_030567	6713.71	186.16	336.67	0.84	1.69	1.008562014	Up		1.84E-14	2.77E-13
n344768	2625	147.08	269.03	1.74	3.5	1.008267616	Up		3.38E-12	4.35E-11
147686	3694	114.69	209.86	0.96	1.93	1.007494537	Up		7.87E-10	8.10E-09
22924	1870	60.59	111.1	1.02	2.05	1.007054758	Up		6.10E-06	3.88E-05
MXLOC_020175	15012	68.49	123.29	0.14	0.28	1	Up		3.54E-06	2.34E-05
n375218	4040	47.49	85.35	0.36	0.72	1	Up		0.000118137	0.00059699
11005	3678.06	92	171	0.79	1.58	1	Up		1.61E-08	1.43E-07
MXLOC_016157	2049	1378.9	0	21.05	0.01	-11.03960452	Down		0	0
4111	1675.56	751	0	14.1	0.01	-10.46147945	Down		5.72E-212	2.13E-209
MXLOC_011163	4145	1678.23	0	12.5	0.01	-10.28771238	Down		0	0
n405658	6075	1835.8	0	9.29	0.01	-9.859534786	Down		0	0
2812	958	190.54	0	6.41	0.01	-9.324180547	Down		3.73E-54	2.66E-52
83694	5195	888.57	0	5.27	0.01	-9.041659152	Down		1.65E-250	6.98E-248

LXLOC_037100	629.43	78.14	0	4.12	0.01	-8.686500527	Down	1.20E-22	2.98E-21
158511	659	80.8	0	4.06	0.01	-8.665335917	Down	3.27E-23	8.40E-22
n332608	498	59.06	0	4.03	0.01	-8.654636029	Down	2.65E-17	4.83E-16
n384745	914	105	0	3.71	0.01	-8.535275377	Down	3.04E-30	1.06E-28
n385950	982	101.17	0	3.32	0.01	-8.375039431	Down	4.05E-29	1.36E-27
MXLOC_018771	2573	265.93	0	3.22	0.01	-8.330916878	Down	2.97E-75	3.16E-73
n335722	339	29.3	0	3.1	0.01	-8.276124405	Down	7.27E-09	6.71E-08
26011	13548	1321.41	4.05	2.99	0.01	-8.224001674	Down	0	0
n342494	4338	400.86	0	2.85	0.01	-8.154818109	Down	3.14E-113	5.44E-111
n374556	312	24.19	0	2.83	0.01	-8.144658243	Down	1.85E-07	1.45E-06
283212	1938	171.47	0	2.77	0.01	-8.113742166	Down	8.24E-49	5.03E-47
n337805	568	45	0	2.66	0.01	-8.055282436	Down	2.30E-13	3.24E-12
n409585	610	47.77	0	2.61	0.01	-8.027905997	Down	6.28E-14	9.18E-13
n385839	631	49.5	0	2.61	0.01	-8.027905997	Down	1.72E-14	2.60E-13
n407963	2807	234.86	0	2.6	0.01	-8.022367813	Down	1.56E-66	1.47E-64
57596	2676	216.5	0	2.52	0.01	-7.977279923	Down	1.81E-61	1.51E-59
n378209	892	62.69	0	2.28	0.01	-7.832890014	Down	3.79E-18	7.26E-17
n378652	737	51.05	0	2.27	0.01	-7.826548487	Down	4.71E-15	7.44E-14
LXLOC_013376	497	33	0	2.26	0.01	-7.820178962	Down	5.45E-10	5.71E-09
9951	3203	230	0	2.23	0.01	-7.8008999	Down	2.08E-65	1.92E-63
386617	2593	185	1	2.22	0.01	-7.794415866	Down	8.52E-51	5.56E-49
n373235	335	19.92	0	2.14	0.01	-7.741466986	Down	4.73E-06	3.06E-05
n335630	662	42.6	0	2.13	0.01	-7.73470962	Down	1.60E-12	2.12E-11
n408887	1447	96.69	0	2.11	0.01	-7.721099189	Down	1.03E-27	3.23E-26
n410030	2798	187.25	0	2.08	0.01	-7.700439718	Down	2.60E-53	1.81E-51
n377761	4784	318.17	0	2.05	0.01	-7.6794801	Down	3.66E-90	5.01E-88
n346542	328	18.21	0	2.01	0.01	-7.651051691	Down	9.04E-06	5.59E-05
445329	1397	85.52	0	1.94	0.01	-7.599912842	Down	1.28E-24	3.49E-23
n409402	587	33.33	0	1.9	0.01	-7.569855608	Down	5.45E-10	5.70E-09
n406247	2272	136.45	0	1.87	0.01	-7.54689446	Down	5.78E-39	2.78E-37
n382312	1445	83.81	0	1.83	0.01	-7.515699838	Down	4.69E-24	1.24E-22
MXLOC_030763	2990	164.59	0	1.71	0.01	-7.417852515	Down	7.68E-47	4.48E-45
9764	7361	395.14	0	1.65	0.01	-7.366322214	Down	8.00E-112	1.35E-109
n406475	706	35.3	0	1.65	0.01	-7.366322214	Down	1.49E-10	1.65E-09
MXLOC_020583	5074	270.13	0	1.64	0.01	-7.357552005	Down	1.17E-76	1.28E-74
n337737	1264	63.8	0	1.61	0.01	-7.330916878	Down	1.98E-18	3.86E-17
n411133	1313	66.01	0	1.6	0.01	-7.321928095	Down	2.84E-19	5.81E-18
n409626	468	21.58	0	1.58	0.01	-7.303780748	Down	1.29E-06	9.11E-06
n380213	547	25.01	0	1.54	0.01	-7.266786541	Down	9.71E-08	7.86E-07
n372579	416	17.88	0	1.5	0.01	-7.22881869	Down	1.73E-05	0.00010128
LXLOC_021913	1117	50.94	0	1.46	0.01	-7.189824559	Down	9.00E-15	1.39E-13
n386202	1627	75.47	0	1.46	0.01	-7.189824559	Down	8.35E-22	1.99E-20
MXLOC_023635	8330	392.49	3.42	1.45	0.01	-7.17990909	Down	6.27E-105	1.00E-102
9897	4166	195.94	0	1.45	0.01	-7.17990909	Down	1.46E-55	1.08E-53
116154	2356.6	105	0	1.39	0.01	-7.118941073	Down	3.04E-30	1.06E-28
100130086	1966	87	0	1.39	0.01	-7.118941073	Down	3.52E-25	9.83E-24
n386246	494	20.11	0	1.39	0.01	-7.118941073	Down	2.47E-06	1.67E-05
n363648	969	40.71	0	1.35	0.01	-7.076815597	Down	5.85E-12	7.38E-11
n340064	5304	5574.01	37.96	32.36	0.24	-7.075033392	Down	0	0
n407956	4044	172.05	0	1.31	0.01	-7.033423002	Down	4.31E-49	2.66E-47
n338422	765	30.35	0	1.3	0.01	-7.022367813	Down	3.81E-09	3.63E-08
51334	1764	71.7	0	1.28	0.01	-7	Down	1.11E-20	2.48E-19
11272	564	21.48	0	1.28	0.01	-7	Down	1.29E-06	9.11E-06
83592	1610	64.23	0	1.26	0.01	-6.977279923	Down	1.04E-18	2.06E-17
MXLOC_029409	3402	136.3	0	1.24	0.01	-6.95419631	Down	5.78E-39	2.77E-37
n406454	2516	98	0	1.21	0.01	-6.918863237	Down	2.83E-28	9.08E-27
n340286	2454	93.06	0	1.18	0.01	-6.882643049	Down	7.21E-27	2.18E-25
9675	3888	146.59	0	1.17	0.01	-6.87036472	Down	8.89E-42	4.65E-40
n342779	970	35.37	0	1.17	0.01	-6.87036472	Down	1.49E-10	1.65E-09
n378078	439	14.88	0	1.17	0.01	-6.87036472	Down	0.000120619	0.000607344

n376618	480	16.38	0	1.17	0.01	-6.87036472	Down	3.30E-05	0.000184933
n376364	2406	88.9	0	1.15	0.01	-6.845490051	Down	1.84E-25	5.23E-24
n339042	2205	79.82	0	1.13	0.01	-6.820178962	Down	6.26E-23	1.58E-21
84865	4339	159.38	0	1.13	0.01	-6.820178962	Down	1.96E-45	1.11E-43
22998	6038.06	211.93	0	1.08	0.01	-6.754887502	Down	4.61E-60	3.72E-58
n367629	490	15.33	0.14	1.07	0.01	-6.741466986	Down	6.31E-05	0.000335224
n335618	487	14.93	0	1.05	0.01	-6.714245518	Down	0.000120619	0.000607868
n384865	592	18.37	0	1.04	0.01	-6.700439718	Down	9.04E-06	5.59E-05
n381174	1201	39.06	0	1.04	0.01	-6.700439718	Down	1.12E-11	1.37E-10
n384808	1570	51.11	0	1.03	0.01	-6.686500527	Down	4.71E-15	7.43E-14
MXLOC_031381	487	14.71	0	1.03	0.01	-6.686500527	Down	0.000120619	0.000607169
n380164	759	23.5	0	1.01	0.01	-6.658211483	Down	3.55E-07	2.67E-06
n380540	1071	33.63	0	1.01	0.01	-6.658211483	Down	5.45E-10	5.70E-09
MXLOC_005007	2905	94.61	0	1.01	0.01	-6.658211483	Down	3.77E-27	1.15E-25
n411537	2480	79.33	0	1	0.01	-6.64385619	Down	6.26E-23	1.58E-21
n346139	632	18.85	0	0.99	0.01	-6.62935662	Down	9.04E-06	5.58E-05
n405884	3508	110.32	0	0.97	0.01	-6.599912842	Down	1.19E-31	4.41E-30
MXLOC_014004	6083	187.11	0	0.95	0.01	-6.569855608	Down	2.60E-53	1.82E-51
n406463	4862	149.08	0	0.95	0.01	-6.569855608	Down	1.27E-42	6.79E-41
n387135	2181	66.4	0	0.95	0.01	-6.569855608	Down	2.84E-19	5.82E-18
343099	4314	131.6	0	0.94	0.01	-6.554588852	Down	1.47E-37	6.69E-36
80765	1316	38.91	0	0.94	0.01	-6.554588852	Down	2.14E-11	2.56E-10
n341290	2506	74.56	0	0.93	0.01	-6.539158811	Down	1.60E-21	3.75E-20
85445	4866	567	6	3.71	0.04	-6.535275377	Down	1.90E-148	4.65E-146
LXLOC_026003	1820	52.8	0	0.91	0.01	-6.50779464	Down	2.46E-15	3.96E-14
n410502	2731	80.08	0	0.91	0.01	-6.50779464	Down	3.27E-23	8.39E-22
n376141	645	17.73	0	0.91	0.01	-6.50779464	Down	1.73E-05	0.000101246
n334875	570	15.28	0	0.9	0.01	-6.491853096	Down	6.31E-05	0.000335376
n407060	4373	123.13	0	0.87	0.01	-6.442943496	Down	2.62E-35	1.12E-33
n382375	615	15.73	0	0.85	0.01	-6.409390936	Down	6.31E-05	0.000336039
n410807	1707	46	0	0.85	0.01	-6.409390936	Down	1.20E-13	1.72E-12
n408149	1477	39.14	0	0.84	0.01	-6.392317423	Down	1.12E-11	1.37E-10
n411115	1787	47.87	0	0.84	0.01	-6.392317423	Down	6.28E-14	9.17E-13
MXLOC_000169	2715	73.09	0	0.84	0.01	-6.392317423	Down	3.05E-21	7.05E-20
57568	6489	172.45	0	0.82	0.01	-6.357552005	Down	4.31E-49	2.66E-47
11214	11702.53	308.74	0	0.81	0.01	-6.339850003	Down	2.38E-87	3.05E-85
n409157	2084	53	0	0.8	0.01	-6.321928095	Down	1.29E-15	2.11E-14
n407074	3364	85.54	0	0.79	0.01	-6.303780748	Down	1.28E-24	3.49E-23
283927	1093	26.86	0	0.79	0.01	-6.303780748	Down	5.08E-08	4.24E-07
LXLOC_029793	800	18.92	0	0.77	0.01	-6.266786541	Down	9.04E-06	5.58E-05
9730	5842	144.52	1.17	0.76	0.01	-6.247927513	Down	2.28E-39	1.10E-37
MXLOC_017851	2882	70.67	0	0.76	0.01	-6.247927513	Down	2.13E-20	4.66E-19
MXLOC_023320	3582.37	86.3	0	0.75	0.01	-6.22881869	Down	6.72E-25	1.85E-23
MXLOC_034248	2320	55.67	0	0.75	0.01	-6.22881869	Down	3.53E-16	5.98E-15
n368606	1719	41.23	0	0.75	0.01	-6.22881869	Down	3.06E-12	3.95E-11
n384519	748	16.98	0	0.74	0.01	-6.209453366	Down	3.30E-05	0.000185021
n371304	2886	68.85	0	0.74	0.01	-6.209453366	Down	7.78E-20	1.65E-18
100316904	837	18.81	0	0.73	0.01	-6.189824559	Down	9.04E-06	5.59E-05
n379536	883	20	0	0.73	0.01	-6.189824559	Down	2.47E-06	1.67E-05
n341481	726	16.2	0	0.73	0.01	-6.189824559	Down	3.30E-05	0.000184814
113540	1062	23.7	0	0.72	0.01	-6.169925001	Down	3.55E-07	2.67E-06
n408125	6892	160.98	0	0.72	0.01	-6.169925001	Down	1.02E-45	5.84E-44
158763	3087	70.88	0	0.71	0.01	-6.14974712	Down	2.13E-20	4.66E-19
n335548	740	15.82	0	0.7	0.01	-6.129283017	Down	6.31E-05	0.000335274
n345222	4088	91.27	0	0.69	0.01	-6.108524457	Down	2.63E-26	7.76E-25
114784	13108	295.16	0	0.69	0.01	-6.108524457	Down	1.08E-83	1.31E-81
n372309	1190	25.32	0	0.68	0.01	-6.087462841	Down	9.71E-08	7.87E-07
n409708	4651	102.3	0	0.68	0.01	-6.087462841	Down	2.12E-29	7.15E-28
n340184	5092	113.22	0	0.68	0.01	-6.087462841	Down	1.71E-32	6.50E-31
n340990	1676	35.15	0	0.66	0.01	-6.044394119	Down	1.49E-10	1.65E-09

3490	1110	23	0	0.66	0.01	-6.044394119	Down	3.55E-07	2.67E-06
n382317	3358	141.96	1.62	1.31	0.02	-6.033423002	Down	1.56E-38	7.35E-37
n408056	1556	32.26	0	0.65	0.01	-6.022367813	Down	1.04E-09	1.06E-08
MXLOC_035443	7974	168.59	0	0.65	0.01	-6.022367813	Down	5.76E-48	3.43E-46
n407294	758	14.36	0	0.62	0.01	-5.95419631	Down	0.000120619	0.000607082
23140	11395	228.12	0	0.61	0.01	-5.930737338	Down	7.61E-65	6.91E-63
n410557	1394	26.78	0	0.61	0.01	-5.930737338	Down	5.08E-08	4.25E-07
n339451	1426	27.38	0	0.61	0.01	-5.930737338	Down	2.66E-08	2.30E-07
n407040	1344	26.02	0	0.61	0.01	-5.930737338	Down	5.08E-08	4.24E-07
n377812	809	15.26	0	0.61	0.01	-5.930737338	Down	6.31E-05	0.000335427
MXLOC_036474	964	178.17	3	6.64	0.11	-5.915607813	Down	9.65E-46	5.52E-44
n409144	1644	31.41	0	0.6	0.01	-5.906890596	Down	1.99E-09	1.96E-08
n339738	1372	25.95	0	0.6	0.01	-5.906890596	Down	9.71E-08	7.86E-07
n383764	1863	35.61	0	0.6	0.01	-5.906890596	Down	1.49E-10	1.65E-09
10251	9038	174.22	0	0.59	0.01	-5.882643049	Down	1.18E-49	7.44E-48
n372654	1398	26.08	0	0.59	0.01	-5.882643049	Down	5.08E-08	4.24E-07
389903	787.78	14.22	0	0.59	0.01	-5.882643049	Down	0.000120619	0.000606646
n378504	926	16.7	0	0.58	0.01	-5.857980995	Down	3.30E-05	0.000185051
MXLOC_028495	4303	80.79	0	0.58	0.01	-5.857980995	Down	3.27E-23	8.40E-22
n409319	4369	81.84	0	0.58	0.01	-5.857980995	Down	1.71E-23	4.43E-22
1901	3050	56	1	0.57	0.01	-5.832890014	Down	5.20E-15	8.19E-14
n374557	1278	23.07	0	0.57	0.01	-5.832890014	Down	3.55E-07	2.67E-06
n325303	900	15.54	0	0.56	0.01	-5.807354922	Down	6.31E-05	0.000336243
n383972	3278	59.43	0	0.56	0.01	-5.807354922	Down	2.65E-17	4.83E-16
n381394	1012	17.38	0	0.55	0.01	-5.781359714	Down	1.73E-05	0.000101365
n340634	1431	24.68	0	0.55	0.01	-5.781359714	Down	1.85E-07	1.45E-06
n376178	1124	19.11	0	0.54	0.01	-5.754887502	Down	4.73E-06	3.07E-05
4101	1979	34	0	0.54	0.01	-5.754887502	Down	2.85E-10	3.07E-09
MXLOC_024935	5331	93.77	0	0.54	0.01	-5.754887502	Down	7.21E-27	2.17E-25
n384728	1102	18.42	0	0.53	0.01	-5.727920455	Down	9.04E-06	5.59E-05
n372676	1540	25.72	0	0.53	0.01	-5.727920455	Down	9.71E-08	7.86E-07
599	3560	59.69	0	0.52	0.01	-5.700439718	Down	2.65E-17	4.84E-16
n409438	1609	26.72	0	0.52	0.01	-5.700439718	Down	5.08E-08	4.25E-07
n410049	1128	18.24	0	0.52	0.01	-5.700439718	Down	9.04E-06	5.58E-05
n377195	1225	20	0	0.52	0.01	-5.700439718	Down	2.47E-06	1.67E-05
100137049	2735	44.68	0	0.51	0.01	-5.672425342	Down	4.39E-13	6.08E-12
n383500	2791	45.6	0	0.51	0.01	-5.672425342	Down	2.30E-13	3.24E-12
n341245	1948	31.72	0	0.51	0.01	-5.672425342	Down	1.99E-09	1.96E-08
MXLOC_012048	2856	45.8	0	0.5	0.01	-5.64385619	Down	2.30E-13	3.24E-12
n375216	4042	63.33	0	0.48	0.01	-5.584962501	Down	1.98E-18	3.86E-17
n384351	1010	15.22	0	0.48	0.01	-5.584962501	Down	6.31E-05	0.000335784
144347	2150	33.31	0	0.48	0.01	-5.584962501	Down	5.45E-10	5.70E-09
n386564	1586	24.18	0	0.48	0.01	-5.584962501	Down	1.85E-07	1.45E-06
MXLOC_013720	1944	58.12	1.03	0.94	0.02	-5.554588852	Down	1.47E-15	2.40E-14
3910	7266	32	3	0.47	0.01	-5.554588852	Down	8.89E-07	6.37E-06
80731	5844	87.27	0	0.46	0.01	-5.523561956	Down	3.52E-25	9.84E-24
n340059	2246	32.74	0	0.46	0.01	-5.523561956	Down	1.04E-09	1.06E-08
n341426	2123	31	0	0.46	0.01	-5.523561956	Down	1.99E-09	1.96E-08
n384395	1325	19.19	0	0.46	0.01	-5.523561956	Down	4.73E-06	3.07E-05
161176	3275	48.78	0	0.46	0.01	-5.523561956	Down	3.29E-14	4.89E-13
n378282	1396	19.78	0	0.45	0.01	-5.491853096	Down	4.73E-06	3.07E-05
n376457	2730	39.66	0	0.45	0.01	-5.491853096	Down	1.12E-11	1.37E-10
6608	3738	54.92	0	0.45	0.01	-5.491853096	Down	6.75E-16	1.12E-14
MXLOC_013575	5210	76.88	0	0.45	0.01	-5.491853096	Down	4.37E-22	1.06E-20
n339715	3870	55.89	0	0.45	0.01	-5.491853096	Down	3.53E-16	5.97E-15
n342869	2160	30.47	0	0.44	0.01	-5.459431619	Down	3.81E-09	3.63E-08
n365080	3048	43.69	0	0.44	0.01	-5.459431619	Down	8.38E-13	1.13E-11
MXLOC_021957	3743	53.65	0	0.44	0.01	-5.459431619	Down	1.29E-15	2.11E-14
n407800	2757	38	0	0.43	0.01	-5.426264755	Down	2.14E-11	2.56E-10
3592	1450	39	1	0.85	0.02	-5.409390936	Down	2.25E-10	2.44E-09

491	8962	122.54	0	0.42	0.01	-5.392317423	Down	5.01E-35	2.10E-33
MXLOC_025278	3142	42.04	0	0.41	0.01	-5.357552005	Down	1.60E-12	2.12E-11
745	5745	77	0	0.41	0.01	-5.357552005	Down	2.29E-22	5.60E-21
n338776	2442	32.03	0	0.41	0.01	-5.357552005	Down	1.04E-09	1.06E-08
MXLOC_022794	2356	30.88	0	0.41	0.01	-5.357552005	Down	3.81E-09	3.63E-08
8675	4885.13	64.49	0	0.41	0.01	-5.357552005	Down	1.04E-18	2.06E-17
n406645	2558	32.96	0	0.4	0.01	-5.321928095	Down	1.04E-09	1.06E-08
n373937	1181	14.93	0	0.4	0.01	-5.321928095	Down	0.000120619	0.000608043
10252	2348	30	0	0.4	0.01	-5.321928095	Down	3.81E-09	3.63E-08
23506	6515	84.22	0	0.4	0.01	-5.321928095	Down	2.45E-24	6.55E-23
219699	4998	65.56	0	0.4	0.01	-5.321928095	Down	5.43E-19	1.10E-17
n377826	1273	16.01	0	0.4	0.01	-5.321928095	Down	3.30E-05	0.000184903
n368111	1141	14.17	0	0.4	0.01	-5.321928095	Down	0.000120619	0.000606908
342908	1843	23.14	0	0.39	0.01	-5.285402219	Down	3.55E-07	2.67E-06
n406513	4423	55.9	0	0.39	0.01	-5.285402219	Down	3.53E-16	5.98E-15
n405913	19250	247.69	0	0.39	0.01	-5.285402219	Down	3.44E-70	3.41E-68
11144	2269	28.44	0	0.39	0.01	-5.285402219	Down	1.39E-08	1.24E-07
n409622	1286	15.71	0	0.39	0.01	-5.285402219	Down	6.31E-05	0.000335733
119016	2431	29.92	0	0.38	0.01	-5.247927513	Down	7.27E-09	6.70E-08
MXLOC_027857	8247	99.3	0	0.37	0.01	-5.209453366	Down	1.48E-28	4.81E-27
n408025	1357	15.77	0	0.37	0.01	-5.209453366	Down	6.31E-05	0.000335529
n335998	299	62.22	1.56	7.65	0.21	-5.186998515	Down	1.18E-16	2.06E-15
MXLOC_019207	4626	54.4	0	0.36	0.01	-5.169925001	Down	6.75E-16	1.13E-14
n410259	1360	15.62	0	0.36	0.01	-5.169925001	Down	6.31E-05	0.000335937
LXLOC_029935	1254	14	0	0.36	0.01	-5.169925001	Down	0.000120619	0.000607693
MXLOC_006947	1560	17.25	0	0.35	0.01	-5.129283017	Down	1.73E-05	0.000101382
n407810	2451	27.64	0	0.35	0.01	-5.129283017	Down	2.66E-08	2.30E-07
MXLOC_004225	3764	42.12	0	0.35	0.01	-5.129283017	Down	1.60E-12	2.12E-11
n342391	2286	25.82	0	0.35	0.01	-5.129283017	Down	9.71E-08	7.86E-07
n385322	2735	30.34	1.14	0.34	0.01	-5.087462841	Down	6.01E-08	4.98E-07
n381852	2084	22.9	0.69	0.34	0.01	-5.087462841	Down	6.78E-07	4.93E-06
n410166	3069	34	0	0.34	0.01	-5.087462841	Down	2.85E-10	3.07E-09
n383548	2047	22.5	0	0.34	0.01	-5.087462841	Down	6.78E-07	4.93E-06
n378844	2593	28.5	0	0.34	0.01	-5.087462841	Down	1.39E-08	1.24E-07
n385286	3228	35.58	0	0.34	0.01	-5.087462841	Down	1.49E-10	1.65E-09
n410692	4146	90.47	2.01	0.67	0.02	-5.06608919	Down	5.01E-23	1.27E-21
n411681	2202	23.15	0	0.33	0.01	-5.044394119	Down	3.55E-07	2.68E-06
79784	6874.91	73	0	0.33	0.01	-5.044394119	Down	3.05E-21	7.05E-20
n342254	2919	30.77	0	0.33	0.01	-5.044394119	Down	3.81E-09	3.63E-08
n408019	1876	19.39	0	0.32	0.01	-5	Down	4.73E-06	3.07E-05
n375859	1710	17.47	0	0.32	0.01	-5	Down	1.73E-05	0.000101348
23116	6242	64.88	0	0.32	0.01	-5	Down	1.04E-18	2.06E-17
MXLOC_036252	2749.43	28.33	0	0.32	0.01	-5	Down	1.39E-08	1.24E-07
79815	2255	23.05	0	0.32	0.01	-5	Down	3.55E-07	2.67E-06
7011	10680	108.65	2.08	0.31	0.01	-4.95419631	Down	6.16E-28	1.94E-26
n409073	9046	90.35	0	0.31	0.01	-4.95419631	Down	5.04E-26	1.47E-24
n406915	3798	38.19	0	0.31	0.01	-4.95419631	Down	2.14E-11	2.56E-10
n373207	1470	14.29	0	0.31	0.01	-4.95419631	Down	0.000120619	0.000607256
n369918	2750	27.62	0	0.31	0.01	-4.95419631	Down	2.66E-08	2.30E-07
MXLOC_033304	1202	163.2	4.71	4.33	0.14	-4.950868293	Down	2.49E-40	1.24E-38
LXLOC_020941	1546	30	1	0.61	0.02	-4.930737338	Down	6.01E-08	4.98E-07
257068	2063	19.51	0	0.3	0.01	-4.906890596	Down	4.73E-06	3.07E-05
n373838	1521	14.58	0	0.3	0.01	-4.906890596	Down	0.000120619	0.00060682
MXLOC_010561	2324	22.22	0	0.3	0.01	-4.906890596	Down	6.78E-07	4.93E-06
n340062	2705	25.38	0	0.29	0.01	-4.857980995	Down	9.71E-08	7.87E-07
MXLOC_012189	10734	102.97	0	0.29	0.01	-4.857980995	Down	2.12E-29	7.16E-28
n371957	3825	36.43	1.12	0.29	0.01	-4.857980995	Down	1.46E-09	1.46E-08
MXLOC_034544	3146	29.7	0	0.29	0.01	-4.857980995	Down	7.27E-09	6.70E-08
n409399	1799	16.56	0	0.29	0.01	-4.857980995	Down	3.30E-05	0.000184874
n337979	2188	20.06	0	0.29	0.01	-4.857980995	Down	2.47E-06	1.67E-05

MXLOC_017878	3681	32.91	0	0.28	0.01	-4.807354922	Down	1.04E-09	1.06E-08
LXLOC_024477	2973	27.19	0	0.28	0.01	-4.807354922	Down	2.66E-08	2.30E-07
n381559	2195	19.73	0	0.28	0.01	-4.807354922	Down	4.73E-06	3.07E-05
n342805	1591	14	0	0.28	0.01	-4.807354922	Down	0.000120619	0.000607606
n407932	2588	23.67	0	0.28	0.01	-4.807354922	Down	3.55E-07	2.67E-06
n407954	1597	14.24	0	0.28	0.01	-4.807354922	Down	0.000120619	0.000607518
8905	2283	20.13	0	0.28	0.01	-4.807354922	Down	2.47E-06	1.67E-05
n339340	4114	36.88	0	0.28	0.01	-4.807354922	Down	7.81E-11	8.89E-10
n410470	3629	32.36	0	0.28	0.01	-4.807354922	Down	1.04E-09	1.06E-08
n410562	1691	14.32	0	0.27	0.01	-4.754887502	Down	0.000120619	0.00060813
n408238	3152	27.23	0	0.27	0.01	-4.754887502	Down	2.66E-08	2.30E-07
MXLOC_013359	1101	28	1	0.81	0.03	-4.754887502	Down	2.06E-07	1.60E-06
64208	1848	15.74	0	0.27	0.01	-4.754887502	Down	6.31E-05	0.000335682
n379141	2737	23.54	0	0.27	0.01	-4.754887502	Down	3.55E-07	2.67E-06
MXLOC_025216	2442	21.17	0	0.27	0.01	-4.754887502	Down	1.29E-06	9.10E-06
n337964	1941	16.65	0	0.27	0.01	-4.754887502	Down	3.30E-05	0.000184844
29103	2776	490	17	5.48	0.21	-4.70571466	Down	1.14E-112	1.97E-110
n385816	1874	15.8	0	0.26	0.01	-4.700439718	Down	6.31E-05	0.000336141
n341514	3282	27.36	0	0.26	0.01	-4.700439718	Down	2.66E-08	2.30E-07
n411670	2117	17.5	0	0.26	0.01	-4.700439718	Down	1.73E-05	0.000101263
MXLOC_004882	1914	47.55	1.45	0.78	0.03	-4.700439718	Down	1.50E-12	1.99E-11
n338992	1853	15.42	0	0.26	0.01	-4.700439718	Down	6.31E-05	0.000335325
MXLOC_011971	8785.03	2830.02	108.04	10.39	0.41	-4.663427934	Down	0	0
MXLOC_023529	4838	38.81	0	0.25	0.01	-4.64385619	Down	2.14E-11	2.56E-10
728689	3053	23.78	0	0.24	0.01	-4.584962501	Down	3.55E-07	2.68E-06
5627	3569	27.35	0	0.24	0.01	-4.584962501	Down	2.66E-08	2.30E-07
143872	4752	37	2	0.24	0.01	-4.584962501	Down	7.66E-09	7.05E-08
n409415	5224	41.1	0	0.24	0.01	-4.584962501	Down	3.06E-12	3.96E-11
n411677	5021	38.36	0	0.24	0.01	-4.584962501	Down	2.14E-11	2.56E-10
3358	4751	147	5	0.95	0.04	-4.569855608	Down	7.78E-35	3.24E-33
LXLOC_023112	5165	39.22	0	0.23	0.01	-4.523561956	Down	1.12E-11	1.37E-10
MXLOC_017334	5411	1186.91	47.63	6.75	0.3	-4.491853096	Down	6.06E-264	2.76E-261
n338901	1693	24	1	0.45	0.02	-4.491853096	Down	2.40E-06	1.62E-05
2329	2139	15	0	0.22	0.01	-4.459431619	Down	6.31E-05	0.00033609
n410877	2995	20.87	0	0.22	0.01	-4.459431619	Down	2.47E-06	1.67E-05
n379334	2014	14.1	0	0.22	0.01	-4.459431619	Down	0.000120619	0.000607431
n339956	2181	15.19	0	0.22	0.01	-4.459431619	Down	6.31E-05	0.000335631
n380672	704	28	1.18	1.31	0.06	-4.448460501	Down	2.06E-07	1.60E-06
n406225	835	167	7	6.5	0.3	-4.437405312	Down	5.70E-38	2.63E-36
n326479	704	46.03	1.99	2.15	0.1	-4.426264755	Down	2.81E-12	3.65E-11
9464	2368	48	2	0.63	0.03	-4.392317423	Down	9.96E-12	1.23E-10
n340342	2261	14.96	0	0.21	0.01	-4.392317423	Down	0.000120619	0.000607955
n407670	3959	27.54	0	0.21	0.01	-4.392317423	Down	2.66E-08	2.30E-07
n384392	2732	18.63	0	0.21	0.01	-4.392317423	Down	9.04E-06	5.58E-05
80183	3978	78	3.87	0.61	0.03	-4.345774837	Down	1.20E-18	2.37E-17
n342387	4185	26.65	0	0.2	0.01	-4.321928095	Down	5.08E-08	4.25E-07
n375022	2177	14	0	0.2	0.01	-4.321928095	Down	0.000120619	0.000607781
140733	4863	31.96	0	0.2	0.01	-4.321928095	Down	1.99E-09	1.96E-08
n376605	582	24.39	1.1	1.4	0.07	-4.321928095	Down	2.40E-06	1.62E-05
n365510	1588	50.01	2.19	0.99	0.05	-4.307428525	Down	2.94E-12	3.80E-11
5176	1533	19	1	0.39	0.02	-4.285402219	Down	4.98E-05	0.000270567
n384702	2562	15.89	0	0.19	0.01	-4.247927513	Down	6.31E-05	0.000336192
n407953	3074	19.15	0	0.19	0.01	-4.247927513	Down	4.73E-06	3.07E-05
n381924	3384	20.7	0	0.19	0.01	-4.247927513	Down	2.47E-06	1.67E-05
n409289	2408	14.3	0	0.19	0.01	-4.247927513	Down	0.000120619	0.000606733
54777	3164	19	0	0.19	0.01	-4.247927513	Down	4.73E-06	3.06E-05
n411634	2970	18.15	0	0.19	0.01	-4.247927513	Down	9.04E-06	5.58E-05
n406148	946	116.76	5.67	3.98	0.21	-4.244307198	Down	1.31E-26	3.90E-25
n365224	1753	52.57	2.59	0.94	0.05	-4.232660757	Down	8.66E-13	1.17E-11
55113	2158	38	2	0.55	0.03	-4.196397213	Down	4.21E-09	3.99E-08

n406573	6269	37.61	0	0.18	0.01	-4.169925001	Down	4.09E-11	4.78E-10
283373	8656	49.68	0	0.18	0.01	-4.169925001	Down	1.72E-14	2.60E-13
n408044	5913	35.02	0	0.18	0.01	-4.169925001	Down	1.49E-10	1.65E-09
1520	4107	23.3	0	0.18	0.01	-4.169925001	Down	3.55E-07	2.67E-06
1404	4678	27.5	2	0.18	0.01	-4.169925001	Down	2.83E-06	1.90E-05
143098	5207	28.68	0	0.17	0.01	-4.087462841	Down	1.39E-08	1.24E-07
5358	3327	18.21	0	0.17	0.01	-4.087462841	Down	9.04E-06	5.59E-05
n378581	3139	16.78	0	0.17	0.01	-4.087462841	Down	3.30E-05	0.000184962
432	1504	47.53	2.68	1.02	0.06	-4.087462841	Down	1.83E-11	2.21E-10
LXLOC_010238	556	19	1	1.15	0.07	-4.038135129	Down	4.98E-05	0.000270609
n341106	3409	18	0	0.16	0.01	-4	Down	9.04E-06	5.59E-05
n373958	6096	31.32	0	0.16	0.01	-4	Down	1.99E-09	1.96E-08
LXLOC_025990	4361	21.99	0	0.16	0.01	-4	Down	1.29E-06	9.11E-06
n407094	3565	18.5	0	0.16	0.01	-4	Down	9.04E-06	5.58E-05
401612	6224	32.17	0	0.16	0.01	-4	Down	1.04E-09	1.06E-08
n344540	6515	33.37	0	0.16	0.01	-4	Down	5.45E-10	5.70E-09
MXLOC_013221	2977	91.41	5.26	0.95	0.06	-3.984893108	Down	4.44E-20	9.53E-19
LXLOC_022829	4861	33.11	2.86	0.31	0.02	-3.95419631	Down	8.31E-08	6.79E-07
n370721	5024	24.02	0	0.15	0.01	-3.906890596	Down	1.85E-07	1.45E-06
5797	5600	54.8	4.1	0.3	0.02	-3.906890596	Down	1.72E-11	2.08E-10
n368062	3235	15.19	0	0.15	0.01	-3.906890596	Down	6.31E-05	0.00033558
n407119	3830	18.02	0	0.15	0.01	-3.906890596	Down	9.04E-06	5.58E-05
10125	5025	294	18	1.8	0.12	-3.906890596	Down	2.83E-60	2.30E-58
MXLOC_032948	6098	784.25	48.55	3.96	0.27	-3.874469118	Down	3.64E-158	9.51E-156
LXLOC_031770	1061	23.73	1.56	0.72	0.05	-3.847996907	Down	4.41E-06	2.88E-05
n324772	674	23.54	1.52	1.15	0.08	-3.845490051	Down	4.41E-06	2.88E-05
64478	14317	1467	94	3.14	0.22	-3.83518913	Down	1.16E-290	6.34E-288
n381011	5008	22.38	0	0.14	0.01	-3.807354922	Down	6.78E-07	4.93E-06
n373865	1288	17	1	0.42	0.03	-3.807354922	Down	0.000165556	0.000808393
9658	8626	38.82	0	0.14	0.01	-3.807354922	Down	2.14E-11	2.56E-10
n408008	3436	15.98	0	0.14	0.01	-3.807354922	Down	6.31E-05	0.000335988
LXLOC_012243	5954	27.69	0	0.14	0.01	-3.807354922	Down	2.66E-08	2.30E-07
283431	2341	82.96	5.45	1.11	0.08	-3.794415866	Down	9.25E-18	1.74E-16
150771	1814	17	1	0.27	0.02	-3.754887502	Down	0.000165556	0.000808281
n411728	3767	1620.22	113.65	13.3	1.02	-3.704785188	Down	0	0
n407275	4184	18.08	0	0.13	0.01	-3.700439718	Down	9.04E-06	5.59E-05
MXLOC_024955	7119	31.16	0	0.13	0.01	-3.700439718	Down	1.99E-09	1.96E-08
9674	4721	20	0	0.13	0.01	-3.700439718	Down	2.47E-06	1.67E-05
n332617	324	23.9	1.67	2.67	0.21	-3.668378509	Down	4.41E-06	2.88E-05
23180	3002	519	38	5.36	0.43	-3.639824436	Down	8.45E-100	1.28E-97
MXLOC_005649	5740	322.14	24	1.73	0.14	-3.627273306	Down	3.77E-62	3.21E-60
65999	1793	31	2	0.49	0.04	-3.614709844	Down	2.71E-07	2.08E-06
LXLOC_011968	4977	514.62	43.33	3.53	0.29	-3.605543378	Down	1.73E-94	2.49E-92
MXLOC_009062	4837	19.47	0	0.12	0.01	-3.584962501	Down	4.73E-06	3.07E-05
MXLOC_012722	6712	79.67	5.91	0.36	0.03	-3.584962501	Down	5.42E-17	9.66E-16
n345954	1811	62.14	4.61	1.08	0.09	-3.584962501	Down	1.61E-13	2.29E-12
n341043	771	273.32	21.07	11.59	0.98	-3.563955007	Down	2.22E-52	1.52E-50
80757	1519	78	6	1.62	0.14	-3.532495081	Down	6.69E-16	1.12E-14
84189	4181	61	5	0.45	0.04	-3.491853096	Down	1.88E-12	2.48E-11
n367458	1049	21.67	1.86	0.66	0.06	-3.459431619	Down	1.49E-05	8.84E-05
374860	4617	17	0	0.11	0.01	-3.459431619	Down	1.73E-05	0.000101314
54507	4202	15	0	0.11	0.01	-3.459431619	Down	6.31E-05	0.000335835
92689	4138	14.61	0	0.11	0.01	-3.459431619	Down	0.000120619	0.000606995
MXLOC_022421	3384	48.61	4.18	0.44	0.04	-3.459431619	Down	5.44E-10	5.70E-09
3693	3372	24	2	0.22	0.02	-3.459431619	Down	1.61E-05	9.50E-05
25909	8633	707.38	60.09	2.51	0.23	-3.447981598	Down	1.12E-128	2.31E-126
80323	4029	117	10	0.87	0.08	-3.442943496	Down	1.94E-22	4.77E-21
2953	1119	73.67	6.43	2.11	0.2	-3.399171094	Down	1.18E-14	1.81E-13
n382338	4429	60.83	5.3	0.42	0.04	-3.392317423	Down	3.34E-12	4.30E-11
n370715	3182	43.15	3.33	0.42	0.04	-3.392317423	Down	1.59E-09	1.58E-08

MXLOC_008449	7285	586.06	52.6	2.47	0.24	-3.363404731	Down	3.49E-105	5.61E-103
n386384	2089	212.15	18.96	3.18	0.31	-3.358686645	Down	1.19E-39	5.82E-38
n341368	1324	205.41	18.33	4.92	0.48	-3.357552005	Down	6.28E-38	2.89E-36
653333	1268	20.36	1.82	0.51	0.05	-3.350497247	Down	2.73E-05	0.000154914
n378816	2443	39.92	3.67	0.51	0.05	-3.350497247	Down	1.62E-08	1.44E-07
MXLOC_035895	6258	19.47	0	0.1	0.01	-3.321928095	Down	4.73E-06	3.07E-05
n410811	5007	16.29	0	0.1	0.01	-3.321928095	Down	3.30E-05	0.000184992
3673	7869	24.73	3.08	0.1	0.01	-3.321928095	Down	7.49E-05	0.00039232
219623	5155	17	1	0.1	0.01	-3.321928095	Down	0.000165556	0.000808168
LXLOC_014695	11665	37.27	0	0.1	0.01	-3.321928095	Down	4.09E-11	4.78E-10
n378128	2150	34.2	2.98	0.5	0.05	-3.321928095	Down	4.59E-08	3.85E-07
LXLOC_020912	1063	33	3	1	0.1	-3.321928095	Down	5.05E-07	3.74E-06
n375105	575	22	2	1.28	0.13	-3.299560282	Down	5.06E-05	0.00027439
8110	2271	35.75	3.26	0.49	0.05	-3.292781749	Down	1.62E-07	1.28E-06
n338650	935	65	6	2.24	0.23	-3.283792966	Down	1.10E-12	1.48E-11
MXLOC_009881	2426	150.93	14.34	1.94	0.2	-3.277984747	Down	1.67E-27	5.20E-26
7056	4032	38	4	0.29	0.03	-3.273018494	Down	1.53E-07	1.21E-06
285267	3987.37	200.32	22.02	1.83	0.19	-3.267772325	Down	7.63E-34	3.06E-32
55885	3512.21	7087.72	664.75	61.73	6.43	-3.263081149	Down	0	0
n378555	1106	62.98	5.98	1.82	0.19	-3.259867127	Down	1.06E-12	1.42E-11
548644	1680	153.13	14.58	2.87	0.3	-3.258016331	Down	3.11E-28	9.95E-27
n340046	3619	88	8	0.75	0.08	-3.22881869	Down	7.85E-17	1.39E-15
n337645	1711	20	2	0.37	0.04	-3.209453366	Down	0.000157212	0.000770554
1756	12214.38	1289.13	254.87	6.44	0.7	-3.201633861	Down	1.07E-146	2.59E-144
n335608	532	18.56	1.89	1.18	0.13	-3.182203331	Down	9.09E-05	0.000468995
n338827	1892	27	3	0.45	0.05	-3.169925001	Down	1.45E-05	8.64E-05
LXLOC_015203	5264	15.62	0	0.09	0.01	-3.169925001	Down	6.31E-05	0.000335886
n407999	7491	21.36	0	0.09	0.01	-3.169925001	Down	1.29E-06	9.10E-06
MXLOC_011459	15557	44.1	0	0.09	0.01	-3.169925001	Down	4.39E-13	6.08E-12
23217	4757	161.25	40	2.51	0.28	-3.164188632	Down	3.16E-16	5.38E-15
LXLOC_006404	3275	121.48	12.04	1.15	0.13	-3.145050333	Down	6.23E-22	1.49E-20
3481	4839	83	8	0.53	0.06	-3.142957954	Down	1.30E-15	2.12E-14
79838	4743	69.18	7.01	0.44	0.05	-3.137503524	Down	6.13E-13	8.36E-12
n378391	1752	68.52	7.27	1.23	0.14	-3.135159583	Down	1.07E-12	1.43E-11
MXLOC_000098	6308	287.48	30.51	1.4	0.16	-3.129283017	Down	5.27E-49	3.23E-47
81620	2675	22.33	2.01	0.26	0.03	-3.115477217	Down	5.06E-05	0.000274475
n324416	511	230.57	24.25	15.3	1.77	-3.111710387	Down	1.35E-39	6.56E-38
n345933	1659	586.31	61.85	11.12	1.29	-3.107713817	Down	1.37E-98	2.04E-96
n339334	1624	52.54	5.85	1.02	0.12	-3.087462841	Down	3.09E-10	3.31E-09
n410880	1598	63.76	6.81	1.26	0.15	-3.070389328	Down	3.39E-12	4.36E-11
n408888	1071	22.48	2.39	0.67	0.08	-3.06608919	Down	5.06E-05	0.000274432
n408346	2215	65.26	7.34	0.92	0.11	-3.064130337	Down	5.58E-12	7.05E-11
MXLOC_001834	3224	103.57	11.03	1	0.12	-3.058893689	Down	2.58E-18	4.98E-17
25893	5158	377.23	40.67	2.25	0.27	-3.058893689	Down	1.78E-63	1.55E-61
4600	2961	23.77	2.44	0.25	0.03	-3.058893689	Down	2.86E-05	0.000162068
n337683	3099	41.44	4.5	0.41	0.05	-3.03562391	Down	2.87E-08	2.47E-07
n381066	596	27.52	3.1	1.54	0.19	-3.018859027	Down	1.45E-05	8.64E-05
56136	2427	753.05	49.33	5.67	0.7	-3.017921908	Down	2.30E-149	5.72E-147
LXLOC_033737	1588	49.11	5.73	0.97	0.12	-3.014950341	Down	1.65E-09	1.64E-08
LXLOC_036460	2030	73.55	8.01	1.13	0.14	-3.01282404	Down	3.28E-13	4.59E-12
MXLOC_032413	14605.16	36.09	0	0.08	0.01	-3	Down	7.81E-11	8.89E-10
MXLOC_022836	9225	22.98	0	0.08	0.01	-3	Down	6.78E-07	4.93E-06
MXLOC_022915	7133	253.5	29.95	1.09	0.14	-2.960829403	Down	1.22E-41	6.37E-40
6256	5522	195.09	22.41	1.09	0.14	-2.960829403	Down	1.16E-32	4.45E-31
6840	8297	159.4	21.31	0.7	0.09	-2.959358016	Down	6.05E-25	1.67E-23
MXLOC_037356	2264	44.65	4.97	0.62	0.08	-2.95419631	Down	5.29E-09	4.95E-08
7093	6771	68	9	0.31	0.04	-2.95419631	Down	2.10E-11	2.51E-10
LXLOC_020379	3135	184.97	22.18	1.83	0.24	-2.930737338	Down	4.37E-30	1.51E-28
n374210	2769	33.88	3.98	0.38	0.05	-2.925999419	Down	5.05E-07	3.74E-06
51454	3451	34	4	0.3	0.04	-2.906890596	Down	1.37E-06	9.63E-06

n374593	800	25.68	3.18	1.05	0.14	-2.906890596	Down		4.35E-05	0.000238298
MXLOC_021275	1628	61.39	7.49	1.19	0.16	-2.894817763	Down		4.97E-11	5.75E-10
166752	6624	232	30	1.08	0.15	-2.847996907	Down		3.97E-36	1.73E-34
147495	2562	30	4	0.36	0.05	-2.847996907	Down		1.19E-05	7.17E-05
n346329	1248	47.93	6.1	1.22	0.17	-2.843274496	Down		2.19E-08	1.91E-07
n409597	3805	158.85	20.03	1.29	0.18	-2.841302254	Down		2.48E-25	7.00E-24
22943	1805	77	10	1.34	0.19	-2.818161677	Down		7.25E-13	9.85E-12
n407257	7259	17.17	0	0.07	0.01	-2.807354922	Down		1.73E-05	0.000101331
n408230	7673	17.49	0	0.07	0.01	-2.807354922	Down		1.73E-05	0.000101297
26509	6860	15	0	0.07	0.01	-2.807354922	Down		6.31E-05	0.000335478
2261	3951	29	3	0.21	0.03	-2.807354922	Down		4.79E-06	3.10E-05
LXLOC_031371	531	23	3	1.46	0.21	-2.797507136	Down		0.000128484	0.000642326
n384226	1757	26.74	3.45	0.48	0.07	-2.777607579	Down		2.52E-05	0.000143836
6678	3141	132	18	1.3	0.19	-2.7744403	Down		1.33E-20	2.94E-19
360	1824	24	3	0.41	0.06	-2.772589504	Down		7.49E-05	0.000392379
n375951	1040	210	28	6.48	0.95	-2.769994395	Down		2.52E-32	9.52E-31
n407523	1165	31.59	4.3	0.87	0.13	-2.742503778	Down		6.95E-06	4.37E-05
n335513	642	59.4	8.04	3.07	0.46	-2.738532889	Down		6.08E-10	6.32E-09
283232	1590	30.23	3.95	0.6	0.09	-2.736965594	Down		2.74E-06	1.84E-05
MXLOC_027998	2456	78.37	10.43	0.99	0.15	-2.722466024	Down		4.27E-13	5.92E-12
n338096	2601	247	34	2.95	0.45	-2.712718048	Down		4.05E-37	1.82E-35
n385794	1861	38.85	5.31	0.65	0.1	-2.700439718	Down		6.60E-07	4.81E-06
4299	9285	374.09	51.76	1.23	0.19	-2.694586992	Down		1.07E-55	7.95E-54
n382102	1799	63	8.79	1.1	0.17	-2.693896872	Down		7.30E-11	8.32E-10
LXLOC_021764	3746	79.48	14.04	0.84	0.13	-2.691877705	Down		4.20E-11	4.90E-10
MXLOC_032042	2596	48.34	6.63	0.58	0.09	-2.688055994	Down		1.28E-08	1.15E-07
123591	2399	59.32	8.26	0.77	0.12	-2.68182404	Down		6.08E-10	6.31E-09
n341222	4319	349.27	49.86	2.5	0.39	-2.680382066	Down		3.02E-51	1.99E-49
165	4081	127	18	0.96	0.15	-2.678071905	Down		1.79E-19	3.70E-18
n369736	3042	31.06	4.8	0.32	0.05	-2.678071905	Down		6.95E-06	4.36E-05
1012	4021	1157	166	8.95	1.4	-2.676460855	Down		6.42E-164	1.76E-161
n371500	504	29.19	4.16	1.97	0.31	-2.667855509	Down		2.02E-05	0.000116986
9771	6622	52.94	7.59	0.25	0.04	-2.64385619	Down		6.19E-09	5.74E-08
246213	3833	175	25	1.36	0.22	-2.628031223	Down		3.42E-26	1.00E-24
MXLOC_015866	2291.04	539.9	79.74	7.35	1.19	-2.626782676	Down		2.45E-76	2.67E-74
LXLOC_011732	1390	79.03	9.8	1.54	0.25	-2.622930351	Down		5.75E-14	8.42E-13
2564	3152	81	12	0.8	0.13	-2.621488377	Down		1.34E-12	1.78E-11
MXLOC_004901	3074	1201	178	12.12	1.97	-2.621122164	Down		8.02E-167	2.21E-164
n409326	3263	90.29	13.07	0.86	0.14	-2.618909833	Down		4.78E-14	7.04E-13
4160	1438	443	66	9.75	1.59	-2.616375453	Down		1.99E-62	1.70E-60
55968	3520.12	197.21	28.71	1.71	0.28	-2.610497593	Down		2.19E-29	7.38E-28
55790	3872	91.25	12.4	0.67	0.11	-2.606657572	Down		7.30E-15	1.14E-13
n342280	1903	47.8	6.92	0.79	0.13	-2.60334103	Down		2.19E-08	1.91E-07
389692	2373	2017	303	26.5	4.37	-2.600287175	Down		1.39E-276	6.95E-274
n375266	2340	177	27	2.36	0.39	-2.59724083	Down		1.67E-25	4.77E-24
2622	3185	186.48	27.94	1.81	0.3	-2.592955291	Down		1.68E-27	5.22E-26
n338643	1637	97	14.73	1.87	0.31	-2.59269815	Down		4.81E-15	7.58E-14
MXLOC_025046	4602	118.55	17.26	0.78	0.13	-2.584962501	Down		4.96E-18	9.43E-17
3736	7983	46.62	6.64	0.18	0.03	-2.584962501	Down		3.74E-08	3.19E-07
3747	3073.55	104	15	1.02	0.17	-2.584962501	Down		4.85E-16	8.13E-15
3371	7616	190	30	0.77	0.13	-2.566346823	Down		1.03E-26	3.09E-25
928	1314	27	4	0.65	0.11	-2.562936194	Down		5.77E-05	0.000310282
MXLOC_004364	3185	66.59	10.28	0.65	0.11	-2.562936194	Down		2.25E-10	2.44E-09
n374607	7481	1247.07	192.16	5.12	0.87	-2.557056504	Down		4.05E-169	1.14E-166
54620	3796	604.32	94.6	4.92	0.85	-2.533123569	Down		2.82E-82	3.36E-80
57582	4768	399	64	2.58	0.45	-2.519374159	Down		7.04E-54	5.00E-52
283726	3131	138	22	1.37	0.24	-2.513069582	Down		1.12E-19	2.35E-18
n341727	1435	67	10.73	1.48	0.26	-2.509013647	Down		1.34E-10	1.50E-09
n383714	1583	28.14	4.43	0.56	0.1	-2.485426827	Down		3.42E-05	0.000190722
n372815	788	41.83	6.75	1.73	0.31	-2.480431917	Down		5.18E-07	3.83E-06

MXLOC_034348	4196	136.52	22.14	1	0.18	-2.473931188	Down	3.06E-19	6.25E-18
2892	5181	83.45	14.38	0.5	0.09	-2.473931188	Down	5.83E-12	7.36E-11
3776	3317.08	675	111	6.33	1.14	-2.473171675	Down	1.53E-88	2.01E-86
93233	3220	46	8	0.44	0.08	-2.459431619	Down	4.75E-07	3.53E-06
MXLOC_008078	7269	104.52	17.43	0.44	0.08	-2.459431619	Down	6.13E-15	9.61E-14
MXLOC_003438	7551	202.64	33.76	0.82	0.15	-2.450661409	Down	1.03E-27	3.21E-26
MXLOC_008097	1447	117	19.6	2.56	0.47	-2.445411148	Down	1.05E-16	1.85E-15
MXLOC_008460	3784	166.56	27.67	1.36	0.25	-2.443606651	Down	4.26E-23	1.09E-21
5179	1286.39	460	74	10.92	2.01	-2.44170545	Down	9.44E-62	7.95E-60
23216	5688	69.54	12.26	0.38	0.07	-2.440572591	Down	5.68E-10	5.93E-09
MXLOC_037235	4637	285.98	47.78	1.9	0.35	-2.440572591	Down	2.98E-38	1.39E-36
2697	3130	109	18	1.08	0.2	-2.432959407	Down	1.70E-15	2.75E-14
MXLOC_021837	5008.46	385.78	65.07	2.37	0.44	-2.42931163	Down	2.48E-50	1.59E-48
MXLOC_032424	4351	84.63	17.25	0.7	0.13	-2.428843299	Down	1.02E-10	1.14E-09
1292	3153	66	10	0.59	0.11	-2.423211431	Down	2.25E-10	2.44E-09
MXLOC_006990	7193	212.25	36.85	0.91	0.17	-2.420331799	Down	2.67E-28	8.60E-27
MXLOC_008260	5158	134.3	23.35	0.8	0.15	-2.415037499	Down	2.75E-18	5.31E-17
5874	1336	107.1	18.26	2.54	0.48	-2.403722186	Down	4.59E-15	7.25E-14
84960	1716	45.83	7.98	0.84	0.16	-2.392317423	Down	2.36E-07	1.82E-06
MXLOC_001388	4431	187.78	32.32	1.31	0.25	-2.389566812	Down	5.22E-25	1.44E-23
347731	5609	917	159	5.03	0.96	-2.389452089	Down	2.10E-115	3.75E-113
55966	2500.93	367	56	4.03	0.77	-2.387849488	Down	2.97E-51	1.96E-49
3762	2912	63.61	11.07	0.68	0.13	-2.387023123	Down	3.43E-09	3.29E-08
n341156	1852	28	5	0.47	0.09	-2.38466385	Down	0.000115473	0.000584709
LXLOC_036334	5225	44.95	8.08	0.26	0.05	-2.378511623	Down	1.27E-06	8.97E-06
LXLOC_016267	1609	40	7	0.78	0.15	-2.378511623	Down	2.93E-06	1.96E-05
n369806	1717	45.19	7.9	0.83	0.16	-2.375039431	Down	2.36E-07	1.82E-06
MXLOC_026246	2165	118.43	20.71	1.71	0.33	-2.373458396	Down	2.13E-16	3.66E-15
LXLOC_003974	2454	77.14	13.56	0.98	0.19	-2.366782331	Down	3.50E-11	4.11E-10
MXLOC_001673	4922	320.98	56.7	2.01	0.39	-2.365649472	Down	4.30E-41	2.19E-39
n341227	527	28	5	1.8	0.35	-2.362570079	Down	0.000115473	0.000584794
55970	4409	66	12	0.46	0.09	-2.353636955	Down	2.48E-09	2.41E-08
n379160	875	221.93	39.55	8.22	1.61	-2.352077706	Down	1.09E-28	3.56E-27
n337267	613	30.03	5.36	1.63	0.32	-2.348728154	Down	4.23E-05	0.000232214
79589	2840	51	9	0.56	0.11	-2.347923303	Down	1.27E-07	1.01E-06
MXLOC_016890	1647	36.9	6.44	0.71	0.14	-2.342392197	Down	6.71E-06	4.24E-05
5737	5360	141	25	0.81	0.16	-2.339850003	Down	9.13E-19	1.83E-17
25850	3113	96.31	17.1	0.96	0.19	-2.337034987	Down	3.21E-13	4.50E-12
MXLOC_008002	4343	126.04	23.55	0.9	0.18	-2.321928095	Down	1.37E-16	2.38E-15
56171	12394	40	9	0.1	0.02	-2.321928095	Down	2.41E-05	0.000138288
8492	4573	118	21	0.8	0.16	-2.321928095	Down	6.85E-16	1.14E-14
3269	4278	55	10	0.4	0.08	-2.321928095	Down	5.56E-08	4.63E-07
1490	2344	83	15	1.1	0.22	-2.321928095	Down	1.86E-11	2.25E-10
n345886	16204	24.97	0	0.05	0.01	-2.321928095	Down	1.85E-07	1.45E-06
80167	2200	77.71	14.38	1.1	0.22	-2.321928095	Down	1.12E-10	1.25E-09
23007	6128	50.68	9.28	0.25	0.05	-2.321928095	Down	2.08E-07	1.62E-06
25959	5208	41	8	0.25	0.05	-2.321928095	Down	5.47E-06	3.50E-05
n342764	2691	185	34	2.14	0.43	-2.315202232	Down	1.38E-23	3.58E-22
n382921	3552	90.91	17	0.79	0.16	-2.303780748	Down	5.89E-12	7.42E-11
n383193	1714	32.13	5.83	0.59	0.12	-2.297680549	Down	1.53E-05	9.04E-05
n409467	2737	34.09	6.78	0.39	0.08	-2.285402219	Down	1.83E-05	0.00010675
n377923	3075	34.07	6.23	0.34	0.07	-2.280107919	Down	1.83E-05	0.000106732
n344649	1005	160.78	30.07	5.14	1.06	-2.277704095	Down	2.63E-20	5.71E-19
MXLOC_023008	3778.31	511.17	66.08	2.86	0.59	-2.277228287	Down	9.57E-78	1.06E-75
3491	2295	43	8	0.58	0.12	-2.273018494	Down	2.08E-06	1.42E-05
MXLOC_015898	4327	447.1	84.1	3.19	0.66	-2.273018494	Down	6.33E-54	4.51E-52
55556	5388	83.53	17.01	0.53	0.11	-2.268488836	Down	1.62E-10	1.79E-09
n345694	1057	77.68	14.78	2.36	0.49	-2.267933205	Down	1.12E-10	1.25E-09
MXLOC_021347	1659	266.38	50.22	5.05	1.05	-2.26589406	Down	1.01E-32	3.88E-31
n410096	6348	99.17	17.97	0.48	0.1	-2.263034406	Down	7.35E-14	1.07E-12

132884	4410	35	7	0.24	0.05	-2.263034406	Down	3.36E-05	0.000187958
4645	9520	194	37	0.62	0.13	-2.253756592	Down	5.13E-24	1.35E-22
4916	3097.28	455	89	4.67	0.98	-2.252568896	Down	3.28E-53	2.28E-51
10178	12896	298.39	55.7	0.71	0.15	-2.242856524	Down	5.78E-37	2.59E-35
MXLOC_027107	6150	65.31	13.18	0.33	0.07	-2.237039197	Down	1.19E-08	1.07E-07
n378532	588	43.17	8.32	2.45	0.52	-2.236198221	Down	2.08E-06	1.42E-05
n342775	2002	38.92	7.86	0.61	0.13	-2.230297619	Down	7.87E-06	4.91E-05
n410038	3414	138.6	27.05	1.26	0.27	-2.222392421	Down	3.55E-17	6.42E-16
2567	2004	152	30	2.37	0.51	-2.216317907	Down	1.18E-18	2.33E-17
343450	5909	419.45	82.5	2.18	0.47	-2.213595473	Down	4.12E-49	2.55E-47
LXLOC_009767	3437	117.35	23.54	1.06	0.23	-2.204358499	Down	1.01E-14	1.56E-13
8361	368	131	26	12.61	2.75	-2.197064752	Down	3.32E-16	5.65E-15
n379281	829	30.4	6.14	1.19	0.26	-2.194378045	Down	0.000129374	0.000646042
LXLOC_016916	3464	32.65	7.11	0.32	0.07	-2.192645078	Down	0.000138786	0.000686483
n410556	3708	48.94	9.71	0.41	0.09	-2.187627003	Down	5.51E-07	4.06E-06
22996	2715	35.86	7.32	0.41	0.09	-2.187627003	Down	3.36E-05	0.000187928
604	3608.73	148	30.08	1.27	0.28	-2.181329765	Down	7.67E-18	1.45E-16
25791	2812	131	26	1.45	0.32	-2.17990909	Down	3.32E-16	5.65E-15
9098	7971	200.98	40.59	0.77	0.17	-2.179323699	Down	7.51E-24	1.96E-22
n341479	1630	51.39	10.55	0.99	0.22	-2.169925001	Down	3.85E-07	2.89E-06
28316	2855	276.9	55.9	3.01	0.67	-2.167530486	Down	1.96E-32	7.41E-31
n337650	2959	264	54	2.77	0.62	-2.159545856	Down	1.85E-30	6.47E-29
n363309	4033	168.33	34.31	1.29	0.29	-2.15324626	Down	4.38E-20	9.40E-19
MXLOC_015267	4035	121.5	25.32	0.93	0.21	-2.146841388	Down	1.26E-14	1.93E-13
n379617	1809	30.67	6.38	0.53	0.12	-2.142957954	Down	0.000129374	0.000646134
MXLOC_028351	4722	459.11	94.32	3	0.68	-2.141355849	Down	9.69E-52	6.45E-50
284	4338	62	13	0.44	0.1	-2.137503524	Down	4.87E-08	4.08E-07
6560	4635	66	14	0.44	0.1	-2.137503524	Down	2.08E-08	1.82E-07
n345101	4258	30.89	5.84	0.22	0.05	-2.137503524	Down	4.23E-05	0.000232177
LXLOC_003229	1046	40.07	8.32	1.23	0.28	-2.135159583	Down	8.83E-06	5.46E-05
n377839	2577	47.45	9.94	0.57	0.13	-2.132450296	Down	8.95E-07	6.41E-06
MXLOC_004154	3071	64.03	13.11	0.65	0.15	-2.115477217	Down	1.91E-08	1.67E-07
3363	3251.69	989	208	9.41	2.18	-2.109866588	Down	4.43E-107	7.22E-105
n340534	1537	33.69	7.07	0.69	0.16	-2.108524457	Down	8.69E-05	0.000450017
n340271	2329	105.72	22.77	1.42	0.33	-2.105353	Down	9.78E-13	1.32E-11
MXLOC_015044	3254	31.42	6.36	0.3	0.07	-2.099535674	Down	7.98E-05	0.000416233
51168	11863	182	39	0.47	0.11	-2.095157233	Down	1.11E-20	2.48E-19
n385799	3250	98.52	20.59	0.94	0.22	-2.095157233	Down	3.26E-12	4.20E-11
23309	5129	106.71	22.72	0.64	0.15	-2.093109404	Down	6.16E-13	8.39E-12
2138	4140.29	113	24	0.85	0.2	-2.087462841	Down	1.84E-13	2.61E-12
6850	5010	83.08	18.41	0.51	0.12	-2.087462841	Down	4.46E-10	4.70E-09
22999	5356.44	426.95	82.61	2.21	0.52	-2.087462841	Down	1.52E-50	9.82E-49
57602	5234	121.82	25.93	0.72	0.17	-2.08246216	Down	1.26E-14	1.92E-13
n346404	2627	185.83	39.93	2.2	0.52	-2.080919995	Down	2.82E-21	6.52E-20
117584	4121	50.2	10.97	0.38	0.09	-2.078002512	Down	6.20E-07	4.53E-06
LXLOC_034204	2230	136	29	1.9	0.45	-2.078002512	Down	7.13E-16	1.19E-14
MXLOC_015863	8072	942.41	202.93	3.58	0.85	-2.074424841	Down	2.18E-100	3.36E-98
MXLOC_036284	5675	270.46	59.33	1.47	0.35	-2.070389328	Down	1.80E-29	6.11E-28
10267	910	221	48	7.85	1.87	-2.069654384	Down	1.61E-24	4.33E-23
MXLOC_035717	3341	212.83	46.24	1.97	0.47	-2.067462968	Down	1.33E-23	3.46E-22
MXLOC_007802	3548	76.4	17.21	0.67	0.16	-2.06608919	Down	4.08E-09	3.88E-08
n409416	4187	67.9	15.32	0.5	0.12	-2.058893689	Down	3.52E-08	3.02E-07
n382924	2252	92.95	20.41	1.29	0.31	-2.057030945	Down	5.22E-11	6.03E-10
7223	3436	56	13	0.54	0.13	-2.054447784	Down	7.58E-07	5.48E-06
LXLOC_000220	8176	587.5	127.55	2.2	0.53	-2.053439259	Down	9.94E-63	8.55E-61
26280	2985	378.97	82.99	3.94	0.95	-2.052196211	Down	6.90E-41	3.48E-39
145270	3641	34	8	0.29	0.07	-2.050626073	Down	0.000144089	0.00071181
n406902	2602	100	22	1.2	0.29	-2.0489096	Down	9.69E-12	1.20E-10
MXLOC_004318	3953	126.85	28.5	0.99	0.24	-2.044394119	Down	2.52E-14	3.78E-13
n407187	1844	41.33	9.33	0.7	0.17	-2.041820176	Down	1.52E-05	9.04E-05

222537	2744	293.89	65.04	3.33	0.81	-2.039528364	Down	1.94E-31	7.10E-30
n378277	1317	158.34	35.29	3.82	0.93	-2.038270017	Down	1.18E-17	2.20E-16
388722	683	56.88	12.56	2.75	0.67	-2.037198618	Down	2.88E-07	2.20E-06
11213	8344	110.61	24.52	0.41	0.1	-2.03562391	Down	7.26E-13	9.85E-12
1310	8725	129	29.47	0.45	0.11	-2.032421478	Down	1.71E-14	2.59E-13
n410583	4060	69.21	15.45	0.53	0.13	-2.027480736	Down	1.41E-08	1.26E-07
MXLOC_014295	1297	51.34	11.71	1.26	0.31	-2.023083613	Down	1.08E-06	7.65E-06
MXLOC_018475	982	118.86	26.74	3.9	0.96	-2.022367813	Down	1.35E-13	1.93E-12
LXLOC_036913	2645	62.03	14.01	0.73	0.18	-2.019899557	Down	1.30E-07	1.04E-06
MXLOC_030983	3597	292.38	65.75	2.51	0.62	-2.017347244	Down	3.05E-31	1.11E-29
7169	1327	184	42	4.44	1.1	-2.013056153	Down	8.28E-20	1.75E-18
5118	1620	70.2	16.05	1.37	0.34	-2.010569242	Down	2.36E-08	2.05E-07
LXLOC_001883	9182	1169	265	3.9	0.97	-2.007417472	Down	4.22E-118	7.86E-116
6571	3872	257.17	57.75	2.05	0.51	-2.007054758	Down	9.06E-28	2.83E-26
815	4904	120	27	0.76	0.19	-2	Down	1.44E-13	2.06E-12
MXLOC_006717	3767	141.64	32.24	1.16	0.29	-2	Down	1.38E-15	2.25E-14
n406154	9405	209	47	0.68	0.17	-2	Down	1.36E-22	3.38E-21
n363537	750	34.79	7.93	1.52	0.38	-2	Down	5.42E-05	0.000292475
339896	3655	38	9	0.32	0.08	-2	Down	5.98E-05	0.000320843
100533105	4563	34.9	8.76	0.24	0.06	-2	Down	0.000144089	0.000711509
n375399	1807	50.48	11.62	0.88	0.22	-2	Down	1.71E-06	1.18E-05
5924	4078	334	76.7	2.53	0.64	-1.982993575	Down	7.93E-35	3.29E-33
n385916	1189	51.39	11.8	1.38	0.35	-1.97924144	Down	1.08E-06	7.65E-06
LXLOC_005462	1243	52.19	12.12	1.34	0.34	-1.978626349	Down	1.79E-06	1.24E-05
n381552	2743	278	64	3.15	0.8	-1.977279923	Down	6.04E-29	2.01E-27
666	2601	1020.51	236.67	12.21	3.11	-1.973076715	Down	2.48E-101	3.85E-99
3397	1026.76	114	27	3.65	0.93	-1.972593843	Down	2.08E-12	2.73E-11
79363	1560	32.75	7.65	0.66	0.17	-1.956931278	Down	0.000138786	0.000686386
n377909	4271	37	9	0.27	0.07	-1.94753258	Down	9.36E-05	0.000481382
159195	6163	301.45	72.05	1.5	0.39	-1.943416472	Down	3.95E-30	1.37E-28
2264	2983.86	117	27	1.19	0.31	-1.940621453	Down	5.51E-13	7.57E-12
5366	1936	102	24	1.65	0.43	-1.94005746	Down	2.63E-11	3.12E-10
n341249	3029	179.35	42.41	1.84	0.48	-1.938599455	Down	7.58E-19	1.52E-17
6744	5175	280.14	65.81	1.64	0.43	-1.93128725	Down	6.35E-29	2.11E-27
7058	5809	35	8	0.19	0.05	-1.925999419	Down	9.14E-05	0.000471298
26873	3984	93	22	0.72	0.19	-1.921997488	Down	2.23E-10	2.43E-09
n382014	7541	82.63	20.97	0.34	0.09	-1.91753784	Down	4.58E-09	4.31E-08
4438	3203	34.65	8.04	0.34	0.09	-1.91753784	Down	0.000144089	0.000711609
81035	3134	145	35	1.43	0.38	-1.911943823	Down	3.78E-15	5.98E-14
LXLOC_037782	6677	204	50	0.94	0.25	-1.910732662	Down	1.95E-20	4.27E-19
374986	5292	1122.13	272.9	6.53	1.74	-1.907995686	Down	2.29E-106	3.69E-104
63895	9713	319.46	77.81	1.01	0.27	-1.903323981	Down	1.45E-31	5.32E-30
LXLOC_007319	3048	183.39	44.86	1.87	0.5	-1.90303827	Down	8.16E-19	1.63E-17
3399	1288	74	18	1.83	0.49	-1.900989994	Down	2.52E-08	2.19E-07
2053	2196	39.5	9.91	0.56	0.15	-1.900464326	Down	3.81E-05	0.00021059
7026	4506.18	38	9	0.26	0.07	-1.893084796	Down	5.98E-05	0.000320893
n340631	6463	55.5	13	0.26	0.07	-1.893084796	Down	1.19E-06	8.38E-06
n410604	2305	326.65	79.98	4.42	1.19	-1.893084796	Down	4.20E-32	1.58E-30
11025	2822	47.34	11.54	0.52	0.14	-1.893084796	Down	6.68E-06	4.22E-05
23338	6463	55.5	13	0.26	0.07	-1.893084796	Down	1.19E-06	8.38E-06
1289	8439	102	24	0.37	0.1	-1.887525271	Down	2.63E-11	3.12E-10
MXLOC_007177	2712	341.66	84.33	3.92	1.06	-1.88678939	Down	5.56E-33	2.17E-31
246175	8794	783.36	192.13	2.73	0.74	-1.883303775	Down	6.29E-74	6.58E-72
53904	5784	41	10	0.22	0.06	-1.874469118	Down	3.90E-05	0.000215133
MXLOC_016550	1358	47.09	11.6	1.1	0.3	-1.874469118	Down	6.68E-06	4.22E-05
79778	3099	43.85	10.89	0.44	0.12	-1.874469118	Down	1.59E-05	9.41E-05
n340025	1964	48	12	0.77	0.21	-1.874469118	Down	1.06E-05	6.47E-05
108	6553	1390	346	6.52	1.78	-1.872994723	Down	7.22E-128	1.48E-125
n379304	1445	43.58	10.8	0.95	0.26	-1.86941589	Down	1.59E-05	9.41E-05
n333290	527	40.96	10.17	2.63	0.72	-1.868993988	Down	6.06E-05	0.000324682

n365511	1853	36.57	9.17	0.62	0.17	-1.866733469	Down		0.000145855	0.000719012
6529	4494	74	18	0.51	0.14	-1.86507042	Down		2.52E-08	2.19E-07
n406602	2632	954.26	238.82	11.28	3.1	-1.863426947	Down		3.91E-88	5.08E-86
3061	2001	44	11	0.69	0.19	-1.860596943	Down		2.53E-05	0.000144555
n378484	2989	422.54	106.27	4.39	1.21	-1.859213892	Down		1.17E-39	5.70E-38
8001	3592	34	8	0.29	0.08	-1.857980995	Down		0.000144089	0.00071171
4839	2661	188.8	47.3	2.21	0.61	-1.857165222	Down		1.35E-18	2.66E-17
n341151	2257	55	14	0.76	0.21	-1.855610091	Down		2.90E-06	1.94E-05
29015	2597	39	10	0.47	0.13	-1.854149134	Down		9.39E-05	0.000482468
n345653	2539	185.49	47.09	2.27	0.63	-1.849268564	Down		4.85E-18	9.23E-17
10082	7103	2924	740	12.64	3.51	-1.848453528	Down		2.90E-262	1.29E-259
MXLOC_002398	3003	122.14	30.68	1.26	0.35	-1.847996907	Down		9.45E-13	1.27E-11
84929	3119	218.54	54.67	2.16	0.6	-1.847996907	Down		1.61E-21	3.78E-20
MXLOC_023254	4495	104.45	27.07	0.72	0.2	-1.847996907	Down		1.57E-10	1.73E-09
7049	6464.18	76	19	0.36	0.1	-1.847996907	Down		2.57E-08	2.22E-07
54212	1889	145	37	2.41	0.67	-1.846800146	Down		2.19E-14	3.30E-13
n385632	869	75	19	2.8	0.78	-1.843880798	Down		3.95E-08	3.35E-07
MXLOC_036611	6037.82	365.53	91.92	1.83	0.51	-1.843274496	Down		8.91E-35	3.69E-33
119391	863.6	74.71	18.7	2.76	0.77	-1.841737916	Down		2.52E-08	2.19E-07
6277	683	413	105	19.95	5.57	-1.840639514	Down		2.24E-38	1.05E-36
n380553	1765	90.33	23.1	1.61	0.45	-1.839063782	Down		2.00E-09	1.97E-08
n383551	2996	541.83	138.03	5.61	1.57	-1.837236212	Down		1.37E-49	8.59E-48
85352	6741	642.97	164.78	2.93	0.82	-1.83720485	Down		1.91E-58	1.49E-56
6261	15376	253	64	0.5	0.14	-1.836501268	Down		3.12E-24	8.30E-23
LXLOC_001981	1753	59.96	15.35	1.07	0.3	-1.834576391	Down		1.22E-06	8.62E-06
121601	4120	218.36	56.29	1.64	0.46	-1.833990049	Down		9.06E-21	2.04E-19
3597	4006	148	38	1.14	0.32	-1.832890014	Down		1.44E-14	2.19E-13
n411009	3918	41.05	10.5	0.32	0.09	-1.830074999	Down		3.90E-05	0.000215167
2719	2290.8	264	67	3.55	1	-1.827819025	Down		3.87E-25	1.08E-23
375790	7319	92.27	24.97	0.39	0.11	-1.8259706	Down		2.00E-09	1.97E-08
MXLOC_034022	4644.22	834.76	217.65	5.63	1.59	-1.824108157	Down		5.90E-74	6.19E-72
n346332	1130	36.08	9.38	1.02	0.29	-1.814444347	Down		0.000145855	0.000718911
768211	3618	396.62	111.5	3.69	1.05	-1.813231488	Down		3.40E-33	1.34E-31
84162	15575	1752.19	454.39	3.44	0.98	-1.811554911	Down		5.10E-154	1.31E-151
n409659	3155	57.18	15.29	0.56	0.16	-1.807354922	Down		2.88E-06	1.93E-05
10085	2974	17815	4645	185.9	53.19	-1.805299828	Down		0	0
MXLOC_035471	1674	126	33	2.37	0.68	-1.801280408	Down		2.28E-12	2.98E-11
MXLOC_000022	3240	593.28	155.27	5.67	1.63	-1.798476771	Down		7.95E-53	5.46E-51
124359	2236	600	157	8.38	2.41	-1.797917097	Down		2.28E-53	1.60E-51
MXLOC_029753	2177	41.39	11.11	0.59	0.17	-1.795180208	Down		9.25E-05	0.000476439
MXLOC_036496	3139	239.14	63.12	2.36	0.68	-1.795180208	Down		4.85E-22	1.17E-20
MXLOC_030198	1931	382.13	100.41	6.2	1.79	-1.792308628	Down		1.47E-34	6.07E-33
4599	3392	121	35.28	1.21	0.35	-1.78958022	Down		8.76E-11	9.91E-10
23160	3501	1548.19	407.66	13.69	3.96	-1.789550111	Down		3.50E-134	7.72E-132
148398	2554	742.72	195.73	9.05	2.62	-1.78835098	Down		2.74E-65	2.51E-63
n408075	1128	144.7	38.22	4.1	1.19	-1.784662336	Down		7.76E-14	1.12E-12
MXLOC_024525	13532	1030.9	275.08	2.34	0.68	-1.782901878	Down		2.12E-88	2.78E-86
MXLOC_026188	4979	355.84	94.12	2.2	0.64	-1.781359714	Down		7.66E-32	2.85E-30
n383036	1781	95.2	25.56	1.68	0.49	-1.777607579	Down		1.31E-09	1.31E-08
9902	5983	47	12	0.24	0.07	-1.777607579	Down		1.64E-05	9.65E-05
MXLOC_025847	1377	62.58	16.8	1.44	0.42	-1.777607579	Down		7.95E-07	5.73E-06
n375624	2591	39.67	10.88	0.48	0.14	-1.777607579	Down		9.39E-05	0.000482398
n339304	2046	521	139	7.97	2.33	-1.774249769	Down		1.33E-45	7.54E-44
MXLOC_009844	2657	283.3	76.67	3.32	0.98	-1.760329587	Down		2.55E-25	7.19E-24
3354	2048	81.99	22	1.25	0.37	-1.756330919	Down		3.86E-08	3.28E-07
339488	2189	64	17	0.91	0.27	-1.752907138	Down		7.88E-07	5.68E-06
252983	6046	449.26	121.24	2.29	0.68	-1.751740947	Down		4.23E-39	2.04E-37
n1850	1116	38.76	10.39	1.11	0.33	-1.750021747	Down		0.000144715	0.000714097
n409359	1144	52.91	14.45	1.48	0.44	-1.750021747	Down		1.04E-05	6.38E-05
23303	8766	488.77	133.09	1.71	0.51	-1.745427173	Down		7.83E-42	4.11E-40

55959	3947.98	3829.22	1040.93	29.92	8.94	-1.742763439	Down	0	0
84071	3413	95.58	25.95	0.87	0.26	-1.742503778	Down	1.31E-09	1.31E-08
5655	2968	83.57	23	0.87	0.26	-1.742503778	Down	3.78E-08	3.22E-07
4703	26202	82.26	20.78	0.1	0.03	-1.736965594	Down	4.58E-09	4.31E-08
MXLOC_016844	2908	84.71	23.15	0.9	0.27	-1.736965594	Down	2.50E-08	2.17E-07
MXLOC_011854	3267	256.13	70.31	2.43	0.73	-1.734987945	Down	1.31E-22	3.25E-21
n384095	2095	135.81	37.07	2.03	0.61	-1.73459858	Down	1.43E-12	1.90E-11
n408908	1375	67.63	18.4	1.56	0.47	-1.730813367	Down	5.10E-07	3.77E-06
n410630	1887	44	12	0.73	0.22	-1.73039294	Down	5.91E-05	0.000317244
6876	1311.51	105	31	2.72	0.82	-1.729910837	Down	2.50E-09	2.43E-08
23780	2545	238.01	65.45	2.91	0.88	-1.725443724	Down	3.75E-21	8.59E-20
MXLOC_017353	3824	40.6	11.77	0.33	0.1	-1.722466024	Down	0.000141288	0.000698266
51196	7992	59	17	0.23	0.07	-1.716207034	Down	6.27E-06	3.98E-05
n407142	2943	65.86	18.42	0.69	0.21	-1.716207034	Down	1.17E-06	8.30E-06
MXLOC_006207	2226	49.15	13.49	0.69	0.21	-1.716207034	Down	1.62E-05	9.57E-05
n406583	5511	41.93	11.81	0.23	0.07	-1.716207034	Down	9.25E-05	0.000476369
57572	6387	225.4	63.23	1.08	0.33	-1.710493383	Down	1.52E-19	3.16E-18
n385784	2212	171.59	47.49	2.42	0.74	-1.709409872	Down	1.67E-15	2.71E-14
LXLOC_035461	3917	435.57	115.42	3.25	1	-1.700439718	Down	1.01E-38	4.80E-37
147111	2217	83	23	1.17	0.36	-1.700439718	Down	3.78E-08	3.22E-07
LXLOC_027556	3708	97.33	26.93	0.81	0.25	-1.695993813	Down	1.29E-09	1.30E-08
n407934	1737	82.4	22.98	1.49	0.46	-1.695606564	Down	2.55E-08	2.21E-07
3216	1676	485.5	136.68	9.12	2.82	-1.693338662	Down	2.83E-40	1.40E-38
n410736	1673	135.38	38.3	2.55	0.79	-1.690572689	Down	3.16E-12	4.08E-11
LXLOC_012659	380	57.03	16.09	5.29	1.64	-1.689571907	Down	6.51E-06	4.12E-05
MXLOC_031900	3782.42	2670.09	762.93	22.06	6.84	-1.689364561	Down	2.82E-209	1.01E-206
57538	10917	208	57	0.58	0.18	-1.688055994	Down	1.32E-18	2.61E-17
n345777	4112	60.52	17.31	0.45	0.14	-1.684498174	Down	4.17E-06	2.72E-05
n409329	4346	248.39	70.79	1.76	0.55	-1.678071905	Down	3.35E-21	7.71E-20
n411702	1942	79.19	22.74	1.28	0.4	-1.678071905	Down	8.84E-08	7.19E-07
n379717	2868	59.12	16.57	0.64	0.2	-1.678071905	Down	2.84E-06	1.90E-05
92126	9281	3891.1	1111.14	12.86	4.03	-1.674038899	Down	6.00E-304	3.62E-301
1277	5927	99	28	0.51	0.16	-1.672425342	Down	2.81E-09	2.72E-08
MXLOC_027827	17380	3782.02	1080.95	6.65	2.09	-1.669851398	Down	2.25E-295	1.29E-292
90627	5909.26	103	30	0.54	0.17	-1.667424661	Down	2.61E-09	2.53E-08
n407915	1594	184.3	52.9	3.64	1.15	-1.662304589	Down	4.37E-16	7.35E-15
116159	3079	141	41	1.42	0.45	-1.657894023	Down	2.85E-12	3.70E-11
342667	3277	150.07	43.04	1.42	0.45	-1.657894023	Down	3.60E-13	5.02E-12
MXLOC_035470	1051	62	18	1.89	0.6	-1.655351829	Down	4.01E-06	2.62E-05
205	6881	514	149	2.29	0.73	-1.649379229	Down	5.80E-41	2.94E-39
n384703	3380	57.35	16.5	0.53	0.17	-1.640457613	Down	6.51E-06	4.12E-05
5796	6118.44	218	63	1.09	0.35	-1.638901308	Down	2.51E-18	4.87E-17
n410695	4048	142.49	41.5	1.09	0.35	-1.638901308	Down	1.92E-12	2.52E-11
6331	8343	78	22	0.28	0.09	-1.637429921	Down	1.33E-07	1.06E-06
MXLOC_021340	12190	333.18	97.81	0.84	0.27	-1.637429921	Down	6.01E-27	1.82E-25
222008	1286	562.56	164.66	13.9	4.47	-1.636738146	Down	2.80E-44	1.54E-42
51171	1237	66.29	19.42	1.71	0.55	-1.636492801	Down	1.71E-06	1.18E-05
23367	6593	1824.83	536.34	8.51	2.74	-1.634983239	Down	1.56E-138	3.58E-136
22901	2770	57.5	16.93	0.65	0.21	-1.63005039	Down	6.51E-06	4.12E-05
n342655	3444	41.2	12.42	0.37	0.12	-1.624490865	Down	0.000205228	0.000987112
MXLOC_020340	4065	492.92	145.74	3.75	1.22	-1.620009448	Down	1.66E-38	7.80E-37
MXLOC_012023	2947.68	208	63	2.24	0.73	-1.617530363	Down	1.27E-16	2.22E-15
MXLOC_031479	1465.61	836.74	249.31	18.09	5.9	-1.616405548	Down	2.05E-63	1.78E-61
1001	4276	72	21	0.52	0.17	-1.612976877	Down	7.00E-07	5.08E-06
n340777	1319	92.8	27.7	2.23	0.73	-1.611075341	Down	2.18E-08	1.91E-07
n380486	1038	127.48	37.92	3.94	1.29	-1.610824564	Down	3.64E-11	4.27E-10
256714	4035	153.42	46.16	1.19	0.39	-1.609415544	Down	1.04E-12	1.39E-11
116985	4849.23	715.75	207.15	4.39	1.44	-1.608152128	Down	2.07E-56	1.56E-54
n406634	924	53.95	16.17	1.89	0.62	-1.608046114	Down	3.30E-05	0.000184815
n379692	1417	57.34	17.09	1.28	0.42	-1.607682577	Down	1.41E-05	8.40E-05

MXLOC_032725	6648	144.13	44.34	0.67	0.22	-1.606657572	Down	8.04E-12	1.00E-10
n408121	2087	44.78	13.12	0.67	0.22	-1.606657572	Down	0.000129745	0.000647801
n408162	1221	508.31	152.3	13.26	4.36	-1.604680735	Down	5.62E-39	2.70E-37
MXLOC_002124	11502.58	834.07	258.9	2.31	0.76	-1.603821528	Down	2.83E-60	2.30E-58
MXLOC_001028	546	87.97	26.47	5.43	1.79	-1.600992611	Down	7.53E-08	6.19E-07
10242	2528.13	317	95	3.88	1.28	-1.599912842	Down	7.17E-25	1.97E-23
n409550	4451	605.87	183.1	4.2	1.39	-1.595304445	Down	1.76E-45	9.98E-44
1522	1500	156	47	3.28	1.09	-1.58936768	Down	6.70E-13	9.11E-12
57084	3925	512	155	4.03	1.34	-1.588546838	Down	1.06E-38	5.03E-37
4883	6665.64	442	137	2.07	0.69	-1.584962501	Down	1.20E-32	4.61E-31
n378833	874	42.03	12.89	1.56	0.52	-1.584962501	Down	0.000136135	0.000674608
112609	2214	217	66	3.06	1.02	-1.584962501	Down	3.42E-17	6.19E-16
n410666	4459	52.63	15.56	0.36	0.12	-1.584962501	Down	2.32E-05	0.000133298
n381184	2481	169.64	51.29	2.13	0.71	-1.584962501	Down	7.83E-14	1.13E-12
29899	3039	143.57	43.77	1.47	0.49	-1.584962501	Down	5.74E-12	7.25E-11
5787	10393	62.93	19.71	0.18	0.06	-1.584962501	Down	8.44E-06	5.25E-05
n372282	518	43.1	13.07	2.82	0.94	-1.584962501	Down	0.000194116	0.000937787
n381829	2167	50.18	14.99	0.72	0.24	-1.584962501	Down	2.41E-05	0.000138111
284805	4793	46.19	14.18	0.3	0.1	-1.584962501	Down	0.00012253	0.000615283
4664	4469	199.46	61.34	1.38	0.46	-1.584962501	Down	9.72E-16	1.60E-14
57477	9568	38.95	10.71	0.12	0.04	-1.584962501	Down	0.000144715	0.000713996
105	8426	263.94	80	0.96	0.32	-1.584962501	Down	1.61E-20	3.54E-19
MXLOC_032738	4145	270.3	82.54	2.01	0.67	-1.584962501	Down	4.56E-21	1.04E-19
8671	7693	106	31	0.42	0.14	-1.584962501	Down	1.68E-09	1.67E-08
29970	2120.33	2181.4	713.73	34.61	11.55	-1.58329609	Down	2.45E-143	5.76E-141
27253	8009	1509	460	5.78	1.93	-1.582468645	Down	1.06E-109	1.77E-107
MXLOC_021235	5301	484.16	146.87	2.81	0.94	-1.579837469	Down	7.70E-37	3.42E-35
MXLOC_028550	7795	529.68	162.75	2.09	0.7	-1.578076115	Down	2.34E-39	1.13E-37
345778	7864	397.49	121.14	1.55	0.52	-1.575684687	Down	4.06E-30	1.40E-28
n364053	1185	52	16	1.4	0.47	-1.574694165	Down	4.90E-05	0.000266161
2182	5195.68	1107.56	339.33	6.55	2.21	-1.567448537	Down	1.74E-80	2.00E-78
256364	3255	83.59	26.27	0.8	0.27	-1.567040593	Down	3.59E-07	2.70E-06
154796	6812.2	352.14	106.5	1.57	0.53	-1.566700294	Down	2.77E-27	8.51E-26
84221	1444.33	105	37	2.63	0.89	-1.563185558	Down	1.48E-07	1.17E-06
128272	3029	63	20	0.65	0.22	-1.562936194	Down	1.17E-05	7.06E-05
153768	2125.15	132.26	40.63	1.95	0.66	-1.562936194	Down	4.70E-11	5.47E-10
285973	4576	87.58	27.22	0.59	0.2	-1.560714954	Down	1.56E-07	1.23E-06
MXLOC_021293	7981	152.88	46.63	0.59	0.2	-1.560714954	Down	1.53E-12	2.03E-11
23554	2564	194	60	2.35	0.8	-1.554588852	Down	3.25E-15	5.17E-14
9842	5299	246.39	77.55	1.43	0.49	-1.545161493	Down	1.27E-18	2.51E-17
MXLOC_017669	5243	119.82	37.4	0.7	0.24	-1.544320516	Down	8.33E-10	8.56E-09
84898	2594	352	110	4.22	1.45	-1.541190099	Down	4.77E-26	1.39E-24
54510	5906	62	19	0.32	0.11	-1.540568381	Down	8.44E-06	5.25E-05
MXLOC_000922	6213	187.68	58.28	0.93	0.32	-1.539158811	Down	1.15E-14	1.76E-13
79858	2644.22	224.48	64.05	2.4	0.83	-1.531851164	Down	4.93E-19	9.99E-18
114798	5186	759	239	4.51	1.56	-1.531581404	Down	9.72E-54	6.88E-52
n410168	1567	79.25	24.94	1.59	0.55	-1.531523242	Down	4.05E-07	3.03E-06
100526832	4206	275.5	86.65	2.02	0.7	-1.528928466	Down	1.15E-20	2.56E-19
MXLOC_032436	9387.93	504.23	170.55	1.76	0.61	-1.528694281	Down	5.52E-33	2.16E-31
n382161	2301	54.98	17.37	0.75	0.26	-1.528378972	Down	4.59E-05	0.000250216
LXLOC_034023	853.26	140	43	5.19	1.8	-1.527737632	Down	1.84E-11	2.22E-10
LXLOC_028893	4585	141.54	44.64	0.95	0.33	-1.525461489	Down	2.55E-11	3.02E-10
23085	9211	424.88	134.06	1.41	0.49	-1.524841508	Down	1.33E-30	4.67E-29
55619	7431	167	54	0.69	0.24	-1.523561956	Down	1.37E-12	1.83E-11
n407465	3788	133.29	42.39	1.09	0.38	-1.520256811	Down	1.32E-10	1.48E-09
1630	10210	4720	1501	14.17	4.94	-1.520256811	Down	0	0
n406106	2172	147.48	46.77	2.12	0.74	-1.518467089	Down	1.05E-11	1.30E-10
MXLOC_004536	7950.8	903.5	293.53	3.55	1.24	-1.517478904	Down	4.70E-61	3.87E-59
6263	15559	1268.9	405	2.49	0.87	-1.517058436	Down	6.85E-87	8.63E-85
n364044	1859	59.41	18.74	1	0.35	-1.514573173	Down	1.33E-05	7.97E-05

MXLOC_001865	5257	97.15	31.72	0.57	0.2	-1.510961919	Down	5.57E-08	4.64E-07
n410468	3175	941.85	301	9.2	3.23	-1.510099696	Down	8.16E-65	7.39E-63
27143	4569	155	50	1.05	0.37	-1.504792152	Down	8.09E-12	1.01E-10
n385795	2703	91.45	29.33	1.05	0.37	-1.504792152	Down	1.37E-07	1.09E-06
n382225	3008	49.15	15.85	0.51	0.18	-1.502500341	Down	7.74E-05	0.000404926
1847	2528	83	27	1.02	0.36	-1.502500341	Down	7.16E-07	5.19E-06
122481	2648	116	37	1.36	0.48	-1.502500341	Down	2.61E-09	2.54E-08
5168	3194	100	33	0.99	0.35	-1.500073603	Down	7.00E-08	5.77E-07
84239	4274	219	71	1.58	0.56	-1.496425826	Down	5.20E-16	8.71E-15
10186	2158	1342	435	19.43	6.91	-1.491528285	Down	5.39E-90	7.35E-88
n382397	1934	150.33	48.76	2.44	0.87	-1.487793842	Down	1.35E-11	1.65E-10
23213	5611.25	465	151	2.55	0.91	-1.486558797	Down	3.24E-32	1.22E-30
n384753	9070	83.98	26.47	0.28	0.1	-1.485426827	Down	3.59E-07	2.70E-06
n382508	6032	55.81	17.5	0.28	0.1	-1.485426827	Down	3.10E-05	0.000174828
91319	3200	192	75	2.24	0.8	-1.485426827	Down	7.07E-11	8.07E-10
8913	7481.61	362	119	1.51	0.54	-1.483517237	Down	5.17E-25	1.43E-23
MXLOC_030023	5294	229.72	75.46	1.34	0.48	-1.48112669	Down	1.87E-16	3.21E-15
n410662	1916	62.99	20.85	1.03	0.37	-1.477047162	Down	1.71E-05	0.00010042
MXLOC_027849	5770	192.6	63.17	1.03	0.37	-1.477047162	Down	5.39E-14	7.91E-13
8871	7332	59	20	0.25	0.09	-1.473931188	Down	5.28E-05	0.000285726
n345768	1778	84.8	28.04	1.5	0.54	-1.473931188	Down	9.62E-07	6.88E-06
23556	4876	118.79	38.66	0.75	0.27	-1.473931188	Down	2.44E-09	2.37E-08
n385355	2638	115.18	37.72	1.36	0.49	-1.472752997	Down	3.81E-09	3.63E-08
64332	3934	110	36	0.86	0.31	-1.472068444	Down	1.26E-08	1.13E-07
MXLOC_005964	4829	94.92	30.84	0.61	0.22	-1.471305719	Down	8.72E-08	7.11E-07
n410552	2049	207.2	67.97	3.16	1.14	-1.470890734	Down	3.03E-15	4.85E-14
MXLOC_023634	2979	127.26	41.55	1.33	0.48	-1.470319935	Down	6.41E-10	6.64E-09
9628	5365.9	360	134	2.32	0.84	-1.465663572	Down	1.02E-20	2.29E-19
MXLOC_007883	3412	63.82	20.66	0.58	0.21	-1.465663572	Down	1.17E-05	7.06E-05
MXLOC_013971	2838	275.11	90.74	3.01	1.09	-1.465435352	Down	1.75E-19	3.63E-18
400954	7853	623	206	2.43	0.88	-1.465380885	Down	1.51E-41	7.82E-40
10777	3147.65	124	43	1.27	0.46	-1.465122731	Down	7.09E-09	6.55E-08
MXLOC_015930	7654	172.31	56.38	0.69	0.25	-1.464668267	Down	8.23E-13	1.11E-11
1793	6751	302.64	99.46	1.38	0.5	-1.464668267	Down	3.36E-21	7.71E-20
9750	3372.08	134.88	29.19	0.8	0.29	-1.4639471	Down	1.78E-15	2.88E-14
n408905	2223	178.44	58.8	2.51	0.91	-1.463748914	Down	3.40E-13	4.74E-12
91409	1471	68.13	22.65	1.46	0.53	-1.461904104	Down	6.88E-06	4.33E-05
n384644	2841	183.98	60.48	2.01	0.73	-1.461227132	Down	2.03E-13	2.87E-12
n385841	3182	79.55	26.43	0.77	0.28	-1.459431619	Down	1.64E-06	1.13E-05
57134	3201	114	38	1.1	0.4	-1.459431619	Down	1.08E-08	9.75E-08
55075	6857	66.82	22	0.3	0.11	-1.447458977	Down	1.46E-05	8.69E-05
n340928	1867	129.84	43.36	2.18	0.8	-1.44625623	Down	1.16E-09	1.17E-08
84940	2397	61	20	0.79	0.29	-1.445799753	Down	2.50E-05	0.000142923
n382707	3416	54.54	17.67	0.49	0.18	-1.444784843	Down	4.59E-05	0.000250177
25849	5054	387	130	2.36	0.87	-1.439699554	Down	7.60E-26	2.20E-24
3199	1778	110.62	36.9	1.95	0.72	-1.437405312	Down	1.26E-08	1.13E-07
MXLOC_004940	4231	241.06	80.66	1.76	0.65	-1.437063806	Down	6.19E-17	1.10E-15
4628	7618	960.43	323.69	3.87	1.43	-1.436318419	Down	1.56E-61	1.31E-59
MXLOC_002216	1311	49.08	16.54	1.19	0.44	-1.435386145	Down	0.000156907	0.000769594
MXLOC_018806	5066	165.2	54.86	1	0.37	-1.434402824	Down	2.89E-12	3.74E-11
n410584	4061	71.5	24.44	0.54	0.2	-1.432959407	Down	8.52E-06	5.29E-05
5028	3110	54	18	0.54	0.2	-1.432959407	Down	9.11E-05	0.000469929
6769	2963	137	46	1.43	0.53	-1.431950882	Down	4.39E-10	4.64E-09
n339291	2963	137	46	1.43	0.53	-1.431950882	Down	4.39E-10	4.63E-09
n385915	3517	349.07	118.41	3.07	1.14	-1.429204831	Down	3.01E-23	7.74E-22
100532736	2210	99.13	33.46	1.4	0.52	-1.428843299	Down	1.01E-07	8.19E-07
3763	2485	56	19	0.7	0.26	-1.428843299	Down	8.36E-05	0.00043401
MXLOC_035787	5231	177.98	59.74	1.05	0.39	-1.428843299	Down	9.50E-13	1.28E-11
n381580	1431	79.33	26.82	1.75	0.65	-1.428843299	Down	1.64E-06	1.13E-05
n344462	4442	49.81	16.42	0.35	0.13	-1.428843299	Down	0.000156907	0.000769486

9671	4636	440	149	2.93	1.09	-1.42657253	Down		7.75E-29	2.56E-27
57214	7080	100	33	0.43	0.16	-1.426264755	Down		7.00E-08	5.77E-07
MXLOC_007087	4423	84.02	29.37	0.59	0.22	-1.423211431	Down		1.84E-06	1.26E-05
LXLOC_015515	5960	244	83	1.26	0.47	-1.422691072	Down		1.45E-16	2.51E-15
n369739	2689	57.79	19.99	0.67	0.25	-1.422233001	Down		5.76E-05	0.000309573
90161	4502.4	3209	1095	22.05	8.23	-1.42181432	Down		4.12E-197	1.38E-194
3600	2323	117.8	39.73	1.58	0.59	-1.421137699	Down		6.90E-09	6.39E-08
1739	4953.9	253.2	87.14	1.58	0.59	-1.421137699	Down		7.26E-17	1.29E-15
57153	3375	109.84	36.47	0.99	0.37	-1.419903254	Down		1.83E-08	1.61E-07
5173	2683.54	191	67	2.27	0.85	-1.417157551	Down		9.95E-13	1.34E-11
4651	11430	60.99	22	0.16	0.06	-1.415037499	Down		0.000128467	0.000642428
LXLOC_025374	7842	61.52	21.28	0.24	0.09	-1.415037499	Down		4.83E-05	0.000262737
n341339	5643	87.61	29.74	0.48	0.18	-1.415037499	Down		6.13E-07	4.49E-06
MXLOC_008008	1535.09	413.71	135.08	8.12	3.05	-1.412670485	Down		1.55E-28	5.04E-27
3696	8777	1353.73	464	4.73	1.78	-1.409962942	Down		1.40E-83	1.70E-81
n410697	3287	90.39	30.46	0.85	0.32	-1.409390936	Down		3.91E-07	2.93E-06
157922	7631	2336.17	801.75	9.4	3.54	-1.408911396	Down		5.76E-143	1.34E-140
n339208	3148	70	24	0.69	0.26	-1.408084739	Down		1.23E-05	7.42E-05
145773	3468	128	44	1.14	0.43	-1.406625259	Down		3.17E-09	3.05E-08
79781	3188	54	19	0.53	0.2	-1.40599236	Down		0.000174107	0.000848019
4093	5544.88	324.03	111	1.8	0.68	-1.404390255	Down		2.72E-21	6.32E-20
n384826	3669	96.87	33.4	0.82	0.31	-1.403355694	Down		3.03E-07	2.31E-06
n337716	1987	75.37	25.86	1.19	0.45	-1.402964667	Down		3.74E-06	2.46E-05
MXLOC_030828	3756	143.95	49.5	1.19	0.45	-1.402964667	Down		3.42E-10	3.66E-09
n410472	2992	150.52	51.48	1.56	0.59	-1.40275917	Down		9.82E-11	1.11E-09
n386306	2249	100.7	35	1.4	0.53	-1.401362562	Down		2.54E-07	1.95E-06
3738	3346	103.11	34.99	0.95	0.36	-1.399930607	Down		4.47E-08	3.76E-07
n381323	2321	64.63	22.57	0.87	0.33	-1.398549376	Down		3.06E-05	0.000172703
n410203	2379	120.59	41.41	1.58	0.6	-1.396890153	Down		8.43E-09	7.72E-08
79633	16110	526.27	180.96	1	0.38	-1.395928676	Down		1.21E-33	4.84E-32
MXLOC_012085	7718	408.96	142.75	1.63	0.62	-1.394531844	Down		8.25E-26	2.38E-24
MXLOC_000368	2281	67.2	23.2	0.92	0.35	-1.394278939	Down		1.94E-05	0.000112811
63916	3579	631.18	218.73	5.46	2.08	-1.392317423	Down		1.80E-39	8.77E-38
n406113	3869	305.07	106.28	2.44	0.93	-1.391578526	Down		1.01E-19	2.12E-18
642475	3251	137.26	48.22	1.31	0.5	-1.389566812	Down		1.56E-09	1.56E-08
10388	5480	60	21	0.34	0.13	-1.387023123	Down		6.96E-05	0.00036687
n342331	2224	97	34	1.36	0.52	-1.387023123	Down		3.97E-07	2.97E-06
161357	5527.18	1821	636	10.17	3.89	-1.386477619	Down		9.31E-109	1.52E-106
n374520	3624	229.29	79.9	1.96	0.75	-1.385891154	Down		2.46E-15	3.96E-14
n406823	2511	86.68	30.01	1.07	0.41	-1.383914982	Down		1.68E-06	1.16E-05
MXLOC_031887	3159	61.52	21.77	0.6	0.23	-1.38332864	Down		4.83E-05	0.000262778
441250	3157	224.96	79.91	2.24	0.86	-1.381090167	Down		1.44E-14	2.19E-13
100131187	537.44	263	91	16.31	6.27	-1.379219434	Down		2.54E-17	4.66E-16
595	4289	361	126	2.6	1	-1.378511623	Down		6.40E-23	1.62E-21
64285	2992	88	31	0.91	0.35	-1.378511623	Down		1.53E-06	1.07E-05
n340474	1646	91.15	31.8	1.74	0.67	-1.376854305	Down		5.19E-07	3.84E-06
22809	2111.34	188.8	67.01	2.83	1.09	-1.376473918	Down		2.85E-12	3.69E-11
LXLOC_035766	2622	139.59	49.29	1.66	0.64	-1.375039431	Down		1.42E-09	1.43E-08
n341142	1931	51.13	17.7	0.83	0.32	-1.375039431	Down		0.000144466	0.000713067
n381026	683	79.91	28.03	3.86	1.49	-1.373288517	Down		5.92E-06	3.78E-05
MXLOC_005197	4219	102.28	35.72	0.75	0.29	-1.370837695	Down		1.24E-07	9.88E-07
n406670	3379	115.45	40.7	1.06	0.41	-1.37036845	Down		2.71E-08	2.34E-07
MXLOC_010773	5709	115.55	40.34	0.62	0.24	-1.36923381	Down		2.71E-08	2.33E-07
LXLOC_017910	7496.83	422.77	148.6	1.73	0.67	-1.368539037	Down		2.43E-26	7.17E-25
LXLOC_001005	524.96	358.52	141.1	25.75	9.98	-1.367460712	Down		9.10E-19	1.82E-17
9881	10484	168	59	0.49	0.19	-1.366782331	Down		2.40E-11	2.86E-10
51657	1416	59.89	21.08	1.34	0.52	-1.365649472	Down		9.98E-05	0.000510415
51195	3727	226.16	80.25	1.88	0.73	-1.364764293	Down		1.31E-14	2.01E-13
553158	1857	71.44	25.58	1.21	0.47	-1.364274386	Down		1.61E-05	9.51E-05
124989	3595.41	124	44	1.08	0.42	-1.362570079	Down		1.32E-08	1.18E-07

222256	6500	189.45	68.06	0.9	0.35	-1.362570079	Down		3.66E-12	4.69E-11
MXLOC_002838	1380	82.56	29.39	1.9	0.74	-1.360402243	Down		3.77E-06	2.48E-05
204962	2889.91	327.67	127.46	3.85	1.5	-1.359895945	Down		1.10E-17	2.05E-16
8605	2524	408	145	5.03	1.96	-1.359704746	Down		5.13E-25	1.42E-23
148	2290.28	135.19	48.89	1.87	0.73	-1.357069901	Down		3.16E-09	3.04E-08
22807	9448.63	410.27	146.45	1.33	0.52	-1.354842717	Down		4.68E-25	1.30E-23
390	2683	4505	1606	52.21	20.43	-1.353636955	Down		1.30E-257	5.64E-255
MXLOC_021460	3613	79.4	29.17	0.69	0.27	-1.353636955	Down		1.08E-05	6.60E-05
9641	3474	108	39	0.97	0.38	-1.351985329	Down		1.74E-07	1.36E-06
83933	2707	293.57	105.48	3.39	1.33	-1.349859028	Down		3.61E-18	6.93E-17
94122	4718.53	988.47	354.08	6.47	2.54	-1.348937215	Down		1.69E-57	1.30E-55
25837	1624	84	30	1.63	0.64	-1.348728154	Down		3.43E-06	2.27E-05
80852	7976	1178.1	421.92	4.53	1.78	-1.347633809	Down		1.71E-68	1.66E-66
201176	3323.26	181	68	1.78	0.7	-1.346450414	Down		5.57E-11	6.42E-10
MXLOC_000219	1234	174.34	62.56	4.5	1.77	-1.346175641	Down		1.83E-11	2.20E-10
n363908	1346	67.86	24.54	1.6	0.63	-1.344648171	Down		3.63E-05	0.000201572
MXLOC_007100	5236	937.63	336.36	5.51	2.17	-1.344357276	Down		1.50E-54	1.08E-52
1261	3794	81	29	0.66	0.26	-1.343954401	Down		5.38E-06	3.45E-05
MXLOC_002698	1960	85.98	30.55	1.37	0.54	-1.343144581	Down		2.40E-06	1.62E-05
57713	8024	267.81	98.56	1.04	0.41	-1.342887714	Down		4.27E-16	7.19E-15
10371	5659	5898	2119	32.07	12.65	-1.342086969	Down		0	0
n367668	2757	67.56	24	0.76	0.3	-1.341036918	Down		3.63E-05	0.000201604
MXLOC_029818	8508	345.18	123.8	1.24	0.49	-1.339486466	Down		2.73E-21	6.33E-20
n407908	6925	108.95	39.44	0.48	0.19	-1.337034987	Down		1.74E-07	1.36E-06
n339467	1976	233.66	84.71	3.7	1.47	-1.331709116	Down		1.28E-14	1.96E-13
n408304	1637	116.49	42.34	2.24	0.89	-1.331621491	Down		6.45E-08	5.34E-07
n410459	1497	110.91	39.97	2.34	0.93	-1.331205908	Down		8.61E-08	7.02E-07
n385532	1253	101.88	36.83	2.59	1.03	-1.330307761	Down		3.29E-07	2.50E-06
54469	1674	335.89	121.8	6.31	2.51	-1.329952641	Down		2.59E-20	5.63E-19
n410164	1903	138.54	50.49	2.28	0.91	-1.325095374	Down		3.69E-09	3.52E-08
9249	1806	66	24	1.15	0.46	-1.321928095	Down		5.17E-05	0.000279908
MXLOC_034635	6301	584.23	212.81	2.85	1.14	-1.321928095	Down		5.67E-34	2.29E-32
4853	9889.26	73.41	28.23	0.25	0.1	-1.321928095	Down		4.74E-05	0.000258163
n407054	2923	89.3	32.63	0.95	0.38	-1.321928095	Down		1.98E-06	1.36E-05
576	5415	87.12	32.73	0.5	0.2	-1.321928095	Down		3.98E-06	2.61E-05
n410117	2693	60.34	22.42	0.7	0.28	-1.321928095	Down		0.000128467	0.000642336
3105	1549	4873.22	1779.72	99.26	39.79	-1.318806556	Down		8.01E-268	3.79E-265
n409298	2873	117.58	43.15	1.27	0.51	-1.316259345	Down		8.25E-08	6.74E-07
9843	4438	138.25	51.31	0.97	0.39	-1.314510623	Down		6.64E-09	6.15E-08
MXLOC_034640	1358	61.56	22.78	1.44	0.58	-1.311944006	Down		9.03E-05	0.0004659
4052	5134	94.73	32.37	0.52	0.21	-1.308122295	Down		3.31E-07	2.51E-06
27340	9010	1194	439	4.06	1.64	-1.307783913	Down		2.78E-66	2.61E-64
337876	3492	341	125	3.02	1.22	-1.307667402	Down		3.49E-20	7.52E-19
n374555	972	75.43	27.89	2.5	1.01	-1.307572802	Down		1.33E-05	7.96E-05
256130	3364	798.82	294.29	7.35	2.97	-1.307281319	Down		1.00E-44	5.52E-43
2890	5730	3208	1183	17.23	6.97	-1.30569214	Down		4.72E-174	1.35E-171
n410678	6285	86.55	32.47	0.42	0.17	-1.304854582	Down		5.61E-06	3.59E-05
LXLOC_031068	2126	243.19	90.08	3.58	1.45	-1.303906687	Down		1.41E-14	2.15E-13
83642	2315	157.14	58.03	2.12	0.86	-1.3016557	Down		5.97E-10	6.21E-09
n339210	2330	75.53	28.11	1.01	0.41	-1.300659478	Down		2.40E-05	0.000137783
2826	1244	64.42	23.72	1.65	0.67	-1.300233024	Down		5.72E-05	0.000307735
LXLOC_033058	4919	254.77	94.03	1.6	0.65	-1.299560282	Down		3.44E-15	5.46E-14
147372	6260	65.36	24.69	0.32	0.13	-1.299560282	Down		7.34E-05	0.000385269
LXLOC_033413	12614	5259.58	1950.5	12.77	5.19	-1.298952081	Down		2.18E-281	1.14E-278
340529	2175	108.25	40.02	1.55	0.63	-1.298844482	Down		3.13E-07	2.38E-06
n339302	2159	396	147	5.73	2.33	-1.298205184	Down		9.84E-23	2.47E-21
n387634	3407	64.73	24.47	0.59	0.24	-1.297680549	Down		0.000103727	0.000529287
MXLOC_007878	7402	259.35	96.18	1.08	0.44	-1.295455884	Down		2.01E-15	3.25E-14
169611	6542	57.85	21.84	0.27	0.11	-1.295455884	Down		0.000202798	0.000976497
400	3215	1440	535	13.88	5.66	-1.29413361	Down		3.43E-78	3.82E-76

658	5560	89	33	0.49	0.2	-1.292781749	Down		3.59E-06	2.37E-05
LXLOC_001937	1864	87.15	32.51	1.47	0.6	-1.292781749	Down		3.98E-06	2.61E-05
81491	2098.8	128	48	1.91	0.78	-1.292026609	Down		3.51E-08	3.00E-07
MXLOC_034690	3947	119.28	43.8	0.93	0.38	-1.291231298	Down		4.13E-08	3.49E-07
MXLOC_035538	2784	2736.27	1019.75	30.54	12.49	-1.289926585	Down		2.96E-146	7.10E-144
LXLOC_030657	10349	221.62	84.1	0.66	0.27	-1.289506617	Down		7.34E-13	9.96E-12
79158	5630	5936	2216	32.45	13.29	-1.287877374	Down		0	0
1298	2831	76	28	0.83	0.34	-1.28757659	Down		1.70E-05	9.99E-05
3218	1823	325.98	121.94	5.61	2.3	-1.28636691	Down		7.65E-19	1.54E-17
n410471	3352	262.63	98.6	2.43	1	-1.280956314	Down		2.30E-15	3.70E-14
8932	4345.27	388.83	146.2	2.77	1.14	-1.280852152	Down		8.02E-22	1.92E-20
11069	4291.1	118.56	44.22	0.85	0.35	-1.280107919	Down		1.05E-07	8.45E-07
7148	13125	70.82	27	0.17	0.07	-1.280107919	Down		7.43E-05	0.000389781
LXLOC_006411	1493	104.27	39.08	2.21	0.91	-1.280107919	Down		6.87E-07	4.99E-06
57507	5645	2155.28	809.23	11.75	4.84	-1.279581804	Down		4.74E-114	8.31E-112
57528	5183	1033.53	388.64	6.14	2.53	-1.279101271	Down		1.45E-55	1.07E-53
n377342	590	88.64	33.24	5.02	2.07	-1.278056596	Down		5.05E-06	3.25E-05
MXLOC_014356	5402.76	412.08	212.12	3.22	1.33	-1.275634443	Down		4.55E-12	5.80E-11
4671	6026.74	528.89	203.39	2.76	1.14	-1.275634443	Down		4.28E-28	1.36E-26
n409655	4722	440.61	165.57	2.88	1.19	-1.275107238	Down		1.12E-24	3.05E-23
MXLOC_022831	4203	101.72	38.19	0.75	0.31	-1.27462238	Down		1.07E-06	7.61E-06
7122	1702	72	27	1.33	0.55	-1.273922722	Down		3.77E-05	0.000208632
n386137	933	67.68	25.64	2.34	0.97	-1.270451877	Down		6.59E-05	0.000349408
26147	3052.94	97	46	1.23	0.51	-1.270089163	Down		0.000182547	0.000885559
375449	9917	133	49	0.41	0.17	-1.270089163	Down		1.14E-08	1.03E-07
54566	5117.93	233.15	84.62	1.35	0.56	-1.269460675	Down		1.28E-14	1.96E-13
n381725	1277	63.96	24.08	1.59	0.66	-1.268488836	Down		0.000146116	0.000720097
MXLOC_015875	7622	351.78	132.95	1.42	0.59	-1.26710407	Down		6.62E-20	1.41E-18
4978	6396.82	5414.07	2056.95	26.1	10.85	-1.266354764	Down		7.48E-278	3.80E-275
MXLOC_016299	2134	121.6	46.33	1.78	0.74	-1.266280065	Down		1.19E-07	9.52E-07
MXLOC_016930	8611.6	609.96	234.49	2.21	0.92	-1.264340603	Down		3.43E-32	1.29E-30
118491	3714	87	33	0.72	0.3	-1.263034406	Down		7.09E-06	4.45E-05
n384700	2501	57.93	21.67	0.72	0.3	-1.263034406	Down		0.000202798	0.000976631
5794	3928	1338.09	508.11	10.53	4.39	-1.262212591	Down		3.88E-70	3.82E-68
n383794	2004	112.3	42.77	1.75	0.73	-1.261386553	Down		2.54E-07	1.96E-06
MXLOC_013382	1333	291.9	111.01	6.95	2.9	-1.260960078	Down		2.22E-16	3.80E-15
5137	2949.44	692	267	7.38	3.08	-1.260690465	Down		4.29E-36	1.86E-34
LXLOC_029086	3261.16	83.76	31.42	0.79	0.33	-1.259386629	Down		8.79E-06	5.44E-05
84465	5914	1760.27	671.69	9.16	3.83	-1.258003206	Down		5.09E-91	7.01E-89
656	3773	67.09	25.55	0.55	0.23	-1.257797757	Down		6.59E-05	0.000349355
388662	6403	358	136	1.72	0.72	-1.256339753	Down		6.10E-20	1.30E-18
84680	2173	90	34	1.29	0.54	-1.256339753	Down		4.54E-06	2.95E-05
MXLOC_006173	7725	294.99	112.15	1.17	0.49	-1.255654875	Down		1.44E-16	2.50E-15
MXLOC_037281	6295	934.01	356.59	4.56	1.91	-1.255461186	Down		3.68E-49	2.28E-47
2645	2587.58	84	33	1.05	0.44	-1.254813899	Down		1.93E-05	0.000112205
MXLOC_017996	2171	94.76	36.13	1.36	0.57	-1.254572827	Down		3.65E-06	2.40E-05
10892	5029	444.39	168.99	2.72	1.14	-1.254572827	Down		1.60E-24	4.31E-23
MXLOC_015889	9175	521.57	200.05	1.74	0.73	-1.253118937	Down		8.12E-28	2.54E-26
MXLOC_006186	5544.58	1598.98	612.08	8.88	3.73	-1.251384046	Down		3.71E-82	4.40E-80
n380118	2922	214.28	82.42	2.28	0.96	-1.247927513	Down		2.44E-12	3.19E-11
4854	8071	99	39	0.38	0.16	-1.247927513	Down		3.63E-06	2.39E-05
MXLOC_037858	1999	777.14	298.45	12.17	5.13	-1.246298437	Down		9.57E-41	4.82E-39
n405566	4009	83.57	32.22	0.64	0.27	-1.245112498	Down		1.55E-05	9.18E-05
MXLOC_016913	3244	113.63	44.11	1.09	0.46	-1.244622369	Down		5.57E-07	4.10E-06
1740	7605.78	223.03	86.78	0.9	0.38	-1.243925583	Down		1.15E-12	1.53E-11
51299	2057	353	136	5.37	2.27	-1.242229791	Down		3.13E-19	6.38E-18
MXLOC_000031	3939.03	304.13	120.27	2.46	1.04	-1.242074787	Down		4.23E-16	7.12E-15
n409064	3822	184.18	70.53	1.49	0.63	-1.241888597	Down		6.31E-11	7.23E-10
64427	2929	104.57	39.98	1.11	0.47	-1.239827015	Down		6.87E-07	4.99E-06
MXLOC_025947	5418	149.31	58.44	0.85	0.36	-1.239465935	Down		8.58E-09	7.85E-08

50859	2954.89	3136	1205	32.77	13.89	-1.238329074	Down		1.84E-158	4.84E-156
MXLOC_033529	5044.11	1138.45	434.18	6.86	2.91	-1.237189423	Down		1.85E-59	1.47E-57
9717	6456	168	66	0.8	0.34	-1.234465254	Down		1.33E-09	1.34E-08
9985	2262.56	975	377	13.42	5.71	-1.232822021	Down		5.64E-50	3.57E-48
MXLOC_034061	8363.94	129.38	48.67	0.47	0.2	-1.232660757	Down		2.50E-08	2.17E-07
51760	1912	627.78	243.24	10.29	4.38	-1.232240207	Down		1.32E-32	5.06E-31
n384480	2216	263	102	3.71	1.58	-1.231494628	Down		1.51E-14	2.29E-13
MXLOC_014714	5822.95	103.81	40.22	0.54	0.23	-1.231325546	Down		1.68E-06	1.16E-05
MXLOC_000290	4509	415.45	161.38	2.84	1.21	-1.230883882	Down		4.44E-22	1.07E-20
729288	5328	317.08	123.37	1.83	0.78	-1.230297619	Down		3.22E-17	5.84E-16
56241	3183	63	24	0.61	0.26	-1.230297619	Down		0.000146116	0.000719996
84439	3462	76	30	0.68	0.29	-1.229481846	Down		5.27E-05	0.000284887
LXLOC_029572	2986	196.31	76.49	2.04	0.87	-1.229481846	Down		3.31E-11	3.89E-10
4921	3252	77	31	0.75	0.32	-1.22881869	Down		6.49E-05	0.000343973
81832	4557.11	1010.69	385.53	6.7	2.86	-1.228145949	Down		5.18E-53	3.59E-51
283219	3378	462.26	180.17	4.24	1.81	-1.228074567	Down		3.17E-24	8.41E-23
n346356	4276	122.85	48.33	0.89	0.38	-1.227805918	Down		2.56E-07	1.97E-06
n410469	2330	66.58	25.64	0.89	0.38	-1.227805918	Down		9.30E-05	0.000478309
85407	2586	80	31	0.96	0.41	-1.227410496	Down		2.42E-05	0.000138713
n346398	2178	86.4	33.97	1.24	0.53	-1.226275856	Down		9.93E-06	6.08E-05
n410019	6295	281.82	110.45	1.38	0.59	-1.225881407	Down		3.32E-15	5.28E-14
n405523	1680	192.34	74.93	3.6	1.54	-1.225066556	Down		4.11E-11	4.80E-10
153478	11515	104.38	39.78	0.28	0.12	-1.222392421	Down		6.87E-07	4.99E-06
53373	5350	83	33	0.49	0.21	-1.222392421	Down		2.68E-05	0.000152268
151835	2048	297.88	116.05	4.55	1.95	-1.222392421	Down		4.76E-16	7.99E-15
LXLOC_025631	3221	96.78	37.45	0.93	0.4	-1.217230716	Down		3.27E-06	2.17E-05
MXLOC_029084	1970	112.44	44.26	1.79	0.77	-1.217029237	Down		7.72E-07	5.57E-06
MXLOC_033155	4100	173.02	67.3	1.3	0.56	-1.215012891	Down		4.51E-10	4.75E-09
n407869	1148	65.24	25.52	1.81	0.78	-1.214443668	Down		0.000130561	0.000651505
9001	3885.42	453	176	3.57	1.54	-1.212993723	Down		6.73E-24	1.77E-22
5122	5146.43	835	331	5.03	2.17	-1.212863357	Down		3.56E-41	1.82E-39
51116	1505	341	134	7.16	3.09	-1.212352749	Down		5.11E-18	9.72E-17
5098	4012.26	134.4	48.79	0.95	0.41	-1.212303604	Down		4.48E-09	4.22E-08
114800	3983	208.37	82.45	1.62	0.7	-1.210566986	Down		1.71E-11	2.07E-10
3781	2515	159	63	1.99	0.86	-1.210359866	Down		4.91E-09	4.61E-08
6925	8320	99.94	39.93	0.37	0.16	-1.209453366	Down		3.63E-06	2.39E-05
MXLOC_024721	5698	123.2	49.17	0.67	0.29	-1.208108195	Down		3.16E-07	2.40E-06
MXLOC_027852	4378.45	476.06	187.47	3.35	1.45	-1.208108195	Down		1.48E-24	4.01E-23
MXLOC_001261	3286	102.47	40.64	0.97	0.42	-1.207595419	Down		2.33E-06	1.58E-05
91133	3587	70	27	0.6	0.26	-1.206450877	Down		7.43E-05	0.000389723
55553	8887.44	242	96	0.83	0.36	-1.20511443	Down		5.27E-13	7.24E-12
59342	1952	260	103	4.17	1.81	-1.204057686	Down		6.75E-14	9.82E-13
MXLOC_011876	1703	82.56	32.63	1.52	0.66	-1.203533394	Down		2.16E-05	0.000124793
MXLOC_035169	4828	619.72	245.57	3.96	1.72	-1.203091865	Down		4.90E-31	1.76E-29
MXLOC_025833	2404	207.15	82.1	2.69	1.17	-1.201097643	Down		2.36E-11	2.80E-10
n341821	2187	124	49	1.77	0.77	-1.200819009	Down		2.29E-07	1.77E-06
79727	4014	721.91	286.51	5.56	2.42	-1.200077835	Down		9.73E-36	4.18E-34
23492	3964	109	43	0.85	0.37	-1.199937571	Down		1.20E-06	8.46E-06
4720	2067	220.34	87.41	3.33	1.45	-1.199469277	Down		5.16E-12	6.54E-11
339855	5704	417	166.02	2.25	0.98	-1.199071347	Down		3.30E-21	7.59E-20
MXLOC_036243	1747	112.4	44.64	2.02	0.88	-1.198779864	Down		7.72E-07	5.57E-06
LXLOC_034205	1938	78	31	1.26	0.55	-1.19592021	Down		4.69E-05	0.000255173
n340674	2982	448.45	178.41	4.67	2.04	-1.194853398	Down		9.54E-23	2.40E-21
MXLOC_020690	9477	635.73	252.55	2.06	0.9	-1.194647431	Down		1.20E-31	4.45E-30
MXLOC_000138	4673	772.29	308.48	5.1	2.23	-1.193453537	Down		9.47E-38	4.33E-36
n342100	2920	90	36	0.96	0.42	-1.192645078	Down		1.36E-05	8.15E-05
n406847	1317	143.01	57	3.45	1.51	-1.192047812	Down		3.50E-08	3.00E-07
3556	3092.3	190	74	1.85	0.81	-1.191531458	Down		7.89E-11	8.98E-10
54682	2297	161.42	64.19	2.19	0.96	-1.189824559	Down		4.38E-09	4.14E-08
59307	1639	157.89	62.56	3.01	1.32	-1.189225557	Down		5.49E-09	5.13E-08

n383232	829	522.24	208.66	20.49	8.99	-1.188526963	Down		4.14E-26	1.21E-24
341359	3286	130	52	1.23	0.54	-1.187627003	Down		1.62E-07	1.28E-06
261734	5004	240.36	95.82	1.48	0.65	-1.187085553	Down		5.90E-13	8.06E-12
MXLOC_033135	5970	335.87	134.73	1.73	0.76	-1.186700714	Down		3.43E-17	6.21E-16
4058	2944	85	35	0.91	0.4	-1.185866545	Down		4.00E-05	0.000220643
91526	1638	145.06	41.66	2	0.88	-1.184424571	Down		5.74E-13	7.86E-12
MXLOC_011534	15949	653.14	261.28	1.25	0.55	-1.184424571	Down		4.44E-32	1.67E-30
n371999	643	66.53	26.75	3.43	1.51	-1.183660027	Down		0.000162578	0.00079474
23768	7179	1120.08	449.15	4.79	2.11	-1.182782657	Down		1.62E-53	1.14E-51
196051	1521	150	60	3.11	1.37	-1.182738687	Down		1.80E-08	1.59E-07
MXLOC_003853	5361	323.85	129.96	1.86	0.82	-1.181606806	Down		1.13E-16	1.98E-15
56143	2451	146	59	1.86	0.82	-1.181606806	Down		3.82E-08	3.25E-07
MXLOC_007329	5888.22	560.8	228.49	2.97	1.31	-1.180896119	Down		8.10E-27	2.44E-25
n373790	4146	91.82	37.06	0.68	0.3	-1.180572246	Down		1.67E-05	9.80E-05
55337	2124	363	146	5.34	2.36	-1.178052882	Down		2.53E-18	4.89E-17
MXLOC_020640	1068	221.57	89.15	6.65	2.94	-1.177538186	Down		1.07E-11	1.31E-10
55466	3020.45	154	61	1.56	0.69	-1.176877762	Down		8.48E-09	7.76E-08
MXLOC_033567	4636	155.05	63.06	1.04	0.46	-1.176877762	Down		1.75E-08	1.54E-07
225689	1888	84	34	1.4	0.62	-1.175086707	Down		3.29E-05	0.00018424
LXLOC_014803	4229	300.43	120.69	2.19	0.97	-1.174874217	Down		1.50E-15	2.45E-14
51326	4971.11	1148.45	487.27	7.47	3.31	-1.174277026	Down		4.31E-49	2.66E-47
55683	5285	286.17	115.4	1.67	0.74	-1.174250927	Down		9.30E-15	1.44E-13
10659	8471.91	671.45	276.18	2.48	1.1	-1.172836597	Down		3.31E-31	1.20E-29
MXLOC_024359	3977	229.26	92.24	1.78	0.79	-1.171952683	Down		4.04E-12	5.15E-11
n338905	1957	128	52	2.05	0.91	-1.171685459	Down		3.06E-07	2.33E-06
23335	7230.1	2686.22	1088.05	11.42	5.07	-1.171504998	Down		2.55E-123	4.99E-121
90113	4020	82	33	0.63	0.28	-1.169925001	Down		3.70E-05	0.000205183
MXLOC_030728	7479	109.96	44.09	0.45	0.2	-1.169925001	Down		2.03E-06	1.39E-05
4036	15735	94	38	0.18	0.08	-1.169925001	Down		1.08E-05	6.55E-05
7025	3210	966	392	9.33	4.15	-1.168765745	Down		2.38E-45	1.34E-43
9516	2516.44	130	54	1.64	0.73	-1.167727446	Down		4.51E-07	3.36E-06
n379693	5967	231.51	94.18	1.19	0.53	-1.166897309	Down		6.00E-12	7.55E-11
n386098	1946	74.1	29.77	1.19	0.53	-1.166897309	Down		5.91E-05	0.00031739
7297	4248	228.97	93.29	1.66	0.74	-1.165586066	Down		9.20E-12	1.14E-10
4018	6489	155.79	64	0.74	0.33	-1.165059246	Down		2.90E-08	2.50E-07
n411113	1592	98.36	40.02	1.95	0.87	-1.164386818	Down		8.47E-06	5.26E-05
n410555	2697	105.3	42.62	1.21	0.54	-1.163975735	Down		2.57E-06	1.73E-05
MXLOC_012232	10095.69	495.38	200.13	1.5	0.67	-1.1627295	Down		3.17E-24	8.41E-23
MXLOC_027659	4159.79	414.3	164	3	1.34	-1.1627295	Down		3.00E-21	6.95E-20
3998	4835	3307	1346	21.08	9.42	-1.162075902	Down		5.67E-150	1.43E-147
5066	5316.54	697	285.72	4.07	1.82	-1.161090344	Down		1.02E-32	3.93E-31
27245	6496	81.17	33.54	0.38	0.17	-1.160464672	Down		5.10E-05	0.000276484
MXLOC_028498	3688.81	118	47.26	0.96	0.43	-1.158697746	Down		5.48E-07	4.04E-06
84267	2474	404.03	165	5.09	2.28	-1.158631832	Down		1.14E-19	2.39E-18
114569	2818	219	89	2.41	1.08	-1.158001834	Down		2.00E-11	2.40E-10
114795	7578	144	58	0.58	0.26	-1.157541277	Down		4.29E-08	3.62E-07
n411678	1978	80.29	33.04	1.27	0.57	-1.155794673	Down		7.01E-05	0.000369381
55114	3401.61	109.07	44.54	0.98	0.44	-1.155278225	Down		2.03E-06	1.39E-05
2903	13217.38	1352	611	3.45	1.55	-1.154328146	Down		3.44E-50	2.18E-48
158056	3660	244.43	99.93	2.07	0.93	-1.154328146	Down		1.32E-12	1.76E-11
n339894	3586	554.28	226.6	4.78	2.15	-1.152673958	Down		1.88E-26	5.60E-25
n345503	10381	69.41	28.6	0.2	0.09	-1.152003093	Down		0.0001776	0.000864073
n409328	3598	106.26	43.03	0.91	0.41	-1.150242636	Down		3.14E-06	2.09E-05
441430	3511	80.91	33.32	0.71	0.32	-1.14974712	Down		7.01E-05	0.000369325
26037	6007	237.78	98	1.22	0.55	-1.149377624	Down		6.98E-12	8.75E-11
MXLOC_013312	10269	1270.12	522.61	3.79	1.71	-1.148201523	Down		1.23E-57	9.47E-56
LXLOC_001250	1540	140.37	57.65	2.88	1.3	-1.147557188	Down		9.06E-08	7.36E-07
n339176	2099	1084.53	445.83	16.15	7.29	-1.147543445	Down		1.43E-49	8.99E-48
64778	7014.98	425	174	1.86	0.84	-1.146841388	Down		1.61E-20	3.54E-19
11247	1804	303.94	125.17	5.29	2.39	-1.146257104	Down		7.45E-15	1.16E-13

29953	5552	151	62	0.84	0.38	-1.144389909	Down	3.68E-08	3.14E-07
55205	6464	3249.92	1340.19	15.45	6.99	-1.144242477	Down	1.37E-143	3.25E-141
n381591	4686	80	33	0.53	0.24	-1.142957954	Down	7.01E-05	0.000369436
3745	3756	155.24	63.96	1.28	0.58	-1.142019005	Down	1.75E-08	1.55E-07
285440	4704	198.77	82.51	1.3	0.59	-1.139724764	Down	3.96E-10	4.20E-09
n407424	2017	98.27	40.67	1.52	0.69	-1.139403057	Down	8.47E-06	5.26E-05
57526	9614	105	44	0.33	0.15	-1.137503524	Down	7.13E-06	4.47E-05
MXLOC_002901	4155	459.37	189.54	3.41	1.55	-1.137503524	Down	8.35E-22	1.99E-20
n383699	1725	72.69	30.04	1.32	0.6	-1.137503524	Down	0.000191019	0.000923842
3700	3193	161	66	1.54	0.7	-1.137503524	Down	1.23E-08	1.10E-07
6695	4827	2768	1149	17.67	8.05	-1.134241352	Down	4.72E-121	9.03E-119
MXLOC_001525	1165	125	52	3.42	1.56	-1.132450296	Down	7.82E-07	5.64E-06
84790	1553	84.36	35.16	1.71	0.78	-1.132450296	Down	5.48E-05	0.000295707
n385849	1176	88.99	36.95	2.41	1.1	-1.131529623	Down	2.58E-05	0.000147506
LXLOC_023476	3584	106	44	0.92	0.42	-1.131244533	Down	5.23E-06	3.36E-05
57540	5214	507.99	211	3	1.37	-1.130786608	Down	1.91E-23	4.93E-22
57497	3144	443	185	4.37	2	-1.12763328	Down	1.51E-20	3.33E-19
LXLOC_031609	5927	203.47	86.67	1.07	0.49	-1.126757142	Down	6.10E-10	6.34E-09
5067	4997	173	72	1.07	0.49	-1.126757142	Down	6.03E-09	5.61E-08
LXLOC_013842	4059	140.99	59.01	1.07	0.49	-1.126757142	Down	2.46E-07	1.90E-06
220972	2589	221.93	99.18	2.86	1.31	-1.126448335	Down	1.26E-09	1.27E-08
MXLOC_021036	5171	2715.98	1135.33	16.18	7.42	-1.124720516	Down	4.32E-117	7.89E-115
LXLOC_015513	3870	246	103	1.96	0.9	-1.122856748	Down	5.24E-12	6.63E-11
n410616	2728	171.62	72.34	1.96	0.9	-1.122856748	Down	1.11E-08	1.00E-07
n344533	1369	122.12	51.23	2.83	1.3	-1.12229043	Down	1.20E-06	8.49E-06
n385078	1410	88	37	1.98	0.91	-1.12156198	Down	4.28E-05	0.000234815
9123	2043	163	68	2.48	1.14	-1.121306296	Down	1.80E-08	1.59E-07
6833	4978	263	110	1.63	0.75	-1.119909464	Down	9.18E-13	1.24E-11
MXLOC_004497	5831	382.68	160.26	2.02	0.93	-1.119052672	Down	7.92E-18	1.49E-16
221477	6751	2036.61	854.45	9.27	4.27	-1.118333269	Down	1.10E-87	1.42E-85
LXLOC_037760	5326	199	84	1.15	0.53	-1.117569596	Down	7.77E-10	8.01E-09
n341102	6645	1439.51	604.4	6.66	3.07	-1.117283522	Down	2.02E-62	1.72E-60
MXLOC_019265	18799.93	2368.46	995.22	3.86	1.78	-1.116723606	Down	2.58E-101	3.98E-99
n406964	3257	216.92	90.85	2.06	0.95	-1.116644919	Down	8.31E-11	9.43E-10
n346240	1058	148.78	62.57	4.51	2.08	-1.116543905	Down	9.28E-08	7.54E-07
79723	3117.32	271.22	114.61	2.71	1.25	-1.116364757	Down	5.70E-13	7.81E-12
n406516	1944	161.4	68.1	2.6	1.2	-1.115477217	Down	3.31E-08	2.84E-07
n340418	4383	111	47	0.78	0.36	-1.115477217	Down	4.93E-06	3.18E-05
85315	4738	1118	471	7.27	3.36	-1.113494131	Down	1.58E-48	9.57E-47
50863	3290.98	376.49	167.34	3.74	1.73	-1.112266232	Down	1.18E-15	1.93E-14
445	1786	76	32	1.34	0.62	-1.11189288	Down	0.00014875	0.000731741
3672	4796	188	80	1.21	0.56	-1.111508315	Down	3.11E-09	3.00E-08
83931	3846	135.01	56.62	1.08	0.5	-1.111031312	Down	2.60E-07	2.00E-06
341	461	211.03	89	15.71	7.28	-1.109672825	Down	2.33E-10	2.53E-09
MXLOC_017004	5698	76.38	31.35	0.41	0.19	-1.109624491	Down	8.95E-05	0.000462245
n406622	2568	229.08	96.95	2.78	1.29	-1.107713817	Down	3.00E-11	3.53E-10
63876	3730	439	186	3.64	1.69	-1.106915204	Down	8.09E-20	1.71E-18
LXLOC_032625	4627.17	2319.18	983.71	15.47	7.19	-1.105409521	Down	1.74E-97	2.58E-95
442213	3006.51	407.5	174.5	4.26	1.98	-1.105353	Down	3.64E-18	7.00E-17
50807	6042	1532.54	649.33	7.8	3.63	-1.103504576	Down	4.16E-65	3.79E-63
MXLOC_012349	2726	102.09	43	1.16	0.54	-1.103093493	Down	1.10E-05	6.68E-05
MXLOC_001012	8252.84	1307.26	547.39	4.79	2.23	-1.102981946	Down	3.43E-57	2.62E-55
MXLOC_013797	3893	110.59	46.71	0.88	0.41	-1.101879614	Down	4.10E-06	2.68E-05
MXLOC_033191	6269	458.66	195.06	2.25	1.05	-1.099535674	Down	2.05E-20	4.50E-19
8091	4140	124	52	0.92	0.43	-1.097297201	Down	1.06E-06	7.57E-06
n407059	2889	314.64	133.94	3.38	1.58	-1.097098688	Down	1.31E-14	2.00E-13
n383602	1966	126.36	54.01	2.01	0.94	-1.09646284	Down	1.53E-06	1.07E-05
29993	5327	81	35	0.47	0.22	-1.095157233	Down	0.000138269	0.000684411
83643	2737	152	65	1.73	0.81	-1.094778225	Down	1.18E-07	9.46E-07
n345685	3327	119.16	51.35	1.11	0.52	-1.093976148	Down	3.00E-06	2.00E-05

6594	4053.24	572.84	245.03	4.37	2.05	-1.09200937	Down		8.27E-25	2.27E-23
n409087	1836	151.92	65.05	2.6	1.22	-1.091630475	Down		1.59E-07	1.26E-06
MXLOC_022930	5922	93.82	40.22	0.49	0.23	-1.091147888	Down		3.99E-05	0.000220053
n342042	1435	178.59	76.34	3.94	1.85	-1.090670359	Down		9.23E-09	8.40E-08
64759	7618	164	71	0.66	0.31	-1.090197809	Down		5.62E-08	4.67E-07
MXLOC_031617	2959.38	126.08	53.74	1.32	0.62	-1.090197809	Down		9.43E-07	6.74E-06
56853	4006.54	1018.66	434.5	7.83	3.68	-1.089306541	Down		2.74E-43	1.48E-41
9858	4932	186.85	79.91	1.17	0.55	-1.089005006	Down		3.51E-09	3.36E-08
214	4741	3930.64	1686.05	25.56	12.03	-1.087251194	Down		8.79E-160	2.39E-157
n410506	2090	265.15	114.09	3.97	1.87	-1.086100737	Down		3.41E-12	4.38E-11
MXLOC_020294	8051	824.42	352.89	3.14	1.48	-1.085167383	Down		3.24E-35	1.37E-33
MXLOC_008004	4259	193.64	82.71	1.4	0.66	-1.084888898	Down		1.81E-09	1.79E-08
23285	4556	1550.76	668.28	10.5	4.96	-1.081977302	Down		1.51E-63	1.32E-61
117	6497.66	1559.94	670.93	7.36	3.48	-1.08061846	Down		2.79E-64	2.47E-62
LXLOC_018925	3510.79	2259.13	976.1	19.96	9.44	-1.080252956	Down		2.74E-91	3.79E-89
n339369	2027	85	36.62	1.31	0.62	-1.079226691	Down		6.61E-05	0.000349958
5243	4716	2790.98	1204	18.25	8.64	-1.078793246	Down		1.39E-112	2.38E-110
55163	3465	1106.34	477.53	9.88	4.68	-1.078002512	Down		8.93E-46	5.12E-44
6873	5020	1214.59	523.52	7.45	3.53	-1.077572242	Down		3.42E-50	2.17E-48
MXLOC_017951	1904	235.58	101.58	3.88	1.84	-1.076350886	Down		5.42E-11	6.25E-10
n384306	2096	104.69	45.47	1.56	0.74	-1.075948853	Down		1.57E-05	9.29E-05
10194	4967	190.09	82.07	1.18	0.56	-1.075288127	Down		4.42E-09	4.17E-08
n379945	2785	105.65	45.39	1.18	0.56	-1.075288127	Down		1.16E-05	7.04E-05
MXLOC_017858	3035	369.1	159.83	3.77	1.79	-1.074604936	Down		2.35E-16	4.02E-15
n375994	3504	248	107	2.19	1.04	-1.074347341	Down		1.94E-11	2.33E-10
5167	7442	96.09	41	0.4	0.19	-1.074000581	Down		2.60E-05	0.000148201
57628	4845.63	874	378	5.55	2.64	-1.071949842	Down		2.56E-36	1.12E-34
55603	5612	188	81	1.03	0.49	-1.071790683	Down		5.00E-09	4.69E-08
56100	4599	6049.15	2623.6	40.56	19.31	-1.070709488	Down		2.88E-239	1.20E-236
1730	4572.69	90	40	0.63	0.3	-1.070389328	Down		9.78E-05	0.000500856
196740	5911.98	371.66	158.07	1.89	0.9	-1.070389328	Down		8.14E-17	1.44E-15
MXLOC_010160	4409	993	431.32	6.95	3.31	-1.070181761	Down		9.95E-41	4.99E-39
MXLOC_017859	6784.29	756.6	326.42	3.4	1.62	-1.069540933	Down		7.87E-32	2.93E-30
1954	10966	383.56	166.78	1.07	0.51	-1.069041644	Down		1.00E-16	1.76E-15
30011	4753.62	1001.02	436.22	6.49	3.1	-1.065950263	Down		9.67E-41	4.86E-39
57167	3486	101	44	0.9	0.43	-1.065588342	Down		2.40E-05	0.000137848
MXLOC_028696	6241.44	729.64	324.75	3.68	1.76	-1.064130337	Down		7.30E-29	2.41E-27
MXLOC_037713	3300	122	53	1.15	0.55	-1.064130337	Down		3.15E-06	2.10E-05
MXLOC_033058	6679.39	903.01	393.93	4.16	1.99	-1.063815098	Down		5.58E-37	2.50E-35
n410180	1694	101.33	44.36	1.88	0.9	-1.062735755	Down		2.40E-05	0.00013787
MXLOC_026479	11259	1648.71	719.96	4.49	2.15	-1.062378785	Down		1.03E-65	9.52E-64
7111	3315.9	410	178	3.8	1.82	-1.062060968	Down		9.70E-18	1.82E-16
135152	1925	146	64	2.38	1.14	-1.061927749	Down		4.37E-07	3.26E-06
642938	4407	616.4	269.15	4.32	2.07	-1.061400545	Down		1.45E-25	4.15E-24
LXLOC_005568	1451	123.62	53.8	2.69	1.29	-1.060235107	Down		2.34E-06	1.58E-05
n381069	2413	173.85	76.12	2.25	1.08	-1.058893689	Down		4.04E-08	3.42E-07
3875	1421	139	59.73	3.04	1.46	-1.058102955	Down		3.33E-07	2.53E-06
255374	1502	110.81	48.32	2.33	1.12	-1.056831223	Down		1.07E-05	6.54E-05
54976	1376	440.86	192.91	10.15	4.88	-1.056526675	Down		9.85E-19	1.96E-17
MXLOC_007597	8995	1755.58	769.27	5.99	2.88	-1.056487191	Down		3.26E-69	3.19E-67
MXLOC_018271	3782	193.76	84.27	1.58	0.76	-1.055853235	Down		4.63E-09	4.36E-08
MXLOC_000939	3760	95.66	41.85	0.79	0.38	-1.055853235	Down		3.52E-05	0.000195801
MXLOC_025521	7028	243.55	106.83	1.06	0.51	-1.055495113	Down		5.28E-11	6.09E-10
MXLOC_015571	870.9	99.96	43.73	3.72	1.79	-1.055343034	Down		2.73E-05	0.000155119
1956	5157.78	232	97	1.33	0.64	-1.055282436	Down		1.96E-11	2.36E-10
MXLOC_013614	4142	108.18	47.3	0.81	0.39	-1.054447784	Down		1.22E-05	7.35E-05
LXLOC_028963	3618	126	55	1.08	0.52	-1.054447784	Down		2.45E-06	1.65E-05
MXLOC_017714	3180	83.51	36.1	0.81	0.39	-1.054447784	Down		0.000121648	0.000611206
968	1856	97.03	42.69	1.64	0.79	-1.053771256	Down		3.10E-05	0.000174648
57546	7032	503.04	221.06	2.2	1.06	-1.053439259	Down		6.72E-21	1.51E-19

MXLOC_029566	3237	114.99	50.9	1.1	0.53	-1.053439259	Down	8.33E-06	5.18E-05
389206	8830.93	2352.29	1034.04	8.17	3.94	-1.052140449	Down	1.81E-91	2.51E-89
LXLOC_029408	999	198.26	87.21	6.38	3.08	-1.050626073	Down	4.27E-09	4.04E-08
n340993	2973	2062.33	907.81	21.53	10.4	-1.049764791	Down	2.52E-80	2.88E-78
10636	2406	92.63	40.57	1.2	0.58	-1.0489096	Down	5.40E-05	0.000291332
9586	8299.76	652	285	2.4	1.16	-1.0489096	Down	6.53E-27	1.97E-25
n373066	1095	164.27	72.45	4.8	2.32	-1.0489096	Down	8.92E-08	7.26E-07
MXLOC_002989	2924	598.2	263.42	6.35	3.07	-1.048517936	Down	1.68E-24	4.52E-23
2037	3914.18	116	51	0.91	0.44	-1.048363022	Down	7.33E-06	4.59E-05
3215	1830	563	248	9.65	4.67	-1.047106392	Down	4.41E-23	1.12E-21
414189	2772	171.63	75.97	1.92	0.93	-1.04580369	Down	4.58E-08	3.84E-07
775	13480	1002.21	441.76	2.27	1.1	-1.045188774	Down	6.90E-40	3.39E-38
MXLOC_015749	6260.16	2906.23	1283.24	14.28	6.92	-1.045152036	Down	1.63E-111	2.74E-109
n339967	1415	175.99	77.6	3.94	1.91	-1.044622991	Down	3.56E-08	3.05E-07
389941	2493	1358.35	600.64	16.97	8.23	-1.044022229	Down	5.33E-53	3.69E-51
150786	3667	198.14	87.63	1.67	0.81	-1.04385429	Down	4.27E-09	4.04E-08
n377707	2029	86.92	38.37	1.34	0.65	-1.043721377	Down	0.000126745	0.000634811
8294	370	240	106	22.96	11.14	-1.043373409	Down	1.26E-10	1.40E-09
5244	3826	86.02	37	0.68	0.33	-1.043068722	Down	7.91E-05	0.000412797
MXLOC_030075	5093.14	1278.64	574.44	7.85	3.81	-1.042901656	Down	3.34E-48	2.02E-46
23118	4395	448.98	198.17	3.15	1.53	-1.041820176	Down	1.48E-18	2.92E-17
n384212	1987	804.6	356.41	12.68	6.16	-1.041552489	Down	6.40E-32	2.39E-30
MXLOC_016706	2685	186.37	82.8	2.16	1.05	-1.040641984	Down	1.42E-08	1.27E-07
LXLOC_012574	10431	2120.26	940.61	6.23	3.03	-1.03991437	Down	4.32E-81	5.03E-79
n377338	567	85	37.68	5.03	2.45	-1.037776651	Down	0.000107003	0.000544655
LXLOC_015674	10734.1	827.79	366.38	2.36	1.15	-1.037152998	Down	8.02E-33	3.11E-31
728404	2420	122.88	54.65	1.58	0.77	-1.036994207	Down	5.00E-06	3.23E-05
4734	5921.96	307	137	1.6	0.78	-1.036525876	Down	6.31E-13	8.60E-12
8558	1729.86	256.09	112.5	4.59	2.24	-1.034995421	Down	1.88E-11	2.26E-10
MXLOC_003145	3029.94	163.79	73.15	1.68	0.82	-1.034765418	Down	1.87E-07	1.46E-06
n381272	4462	125	56	0.86	0.42	-1.033947332	Down	5.17E-06	3.33E-05
2561	7316.39	642	287	2.7	1.32	-1.032421478	Down	2.72E-25	7.65E-24
57282	5606.01	997.42	445.38	5.48	2.68	-1.031942893	Down	1.66E-38	7.80E-37
n372953	2869	85.27	37.97	0.92	0.45	-1.03170886	Down	0.000107003	0.000544576
159090	2966.6	153.98	59.65	1.39	0.68	-1.031478231	Down	4.00E-09	3.80E-08
MXLOC_013935	4785.4	2309.57	1064.01	15.37	7.52	-1.031312598	Down	1.07E-80	1.23E-78
2042	5717.44	8104	3642	43.95	21.51	-1.030855641	Down	3.09E-295	1.72E-292
MXLOC_037239	7871	1011.63	452.08	3.94	1.93	-1.029594782	Down	6.99E-39	3.35E-37
10203	5006	181.87	81	1.12	0.55	-1.025995209	Down	3.83E-08	3.25E-07
n342406	1247	89.2	39.86	2.28	1.12	-1.025535092	Down	8.27E-05	0.000430377
84952	7218	147.04	66.7	0.63	0.31	-1.023083613	Down	8.09E-07	5.82E-06
MXLOC_000782	7675	798.24	358.72	3.19	1.57	-1.022791865	Down	8.29E-31	2.95E-29
55711	2095	1629.64	730.68	24.32	11.97	-1.022720077	Down	2.87E-61	2.38E-59
3198	2520.05	534	240	6.6	3.25	-1.022026306	Down	4.64E-21	1.06E-19
MXLOC_037560	5072	794.77	356.75	4.83	2.38	-1.021061616	Down	1.06E-30	3.76E-29
3761	2063	91	41	1.38	0.68	-1.021061616	Down	0.000114873	0.000582093
3547	3713.08	308	165	3.06	1.51	-1.018983103	Down	1.97E-08	1.72E-07
8407	1360	986	443	22.98	11.34	-1.018958158	Down	1.50E-37	6.78E-36
MXLOC_021818	2058	102.33	46.27	1.56	0.77	-1.018615678	Down	4.48E-05	0.000244734
MXLOC_001789	16670.66	2008	902	3.68	1.82	-1.015767316	Down	1.07E-74	1.13E-72
n379585	1114	101.51	45.58	2.91	1.44	-1.014950341	Down	3.81E-05	0.000210887
2235	7260.51	1400	631	5.92	2.93	-1.014696511	Down	3.17E-52	2.16E-50
54361	3905	380	171	3.01	1.49	-1.014451156	Down	2.21E-15	3.56E-14
MXLOC_037327	3330	333.83	150.67	3.11	1.54	-1.013984229	Down	1.24E-13	1.78E-12
LXLOC_007708	2710	183	83	2.1	1.04	-1.0138058	Down	5.23E-08	4.36E-07
MXLOC_016179	7140	1787.93	807.75	7.69	3.81	-1.0131926	Down	7.07E-66	6.57E-64
n408115	2865	556.1	251.46	6.03	2.99	-1.012012518	Down	1.18E-21	2.79E-20
MXLOC_013406	3233	128.21	58.14	1.23	0.61	-1.011777168	Down	5.32E-06	3.42E-05
56106	4617	1012.58	464.14	6.85	3.4	-1.010569242	Down	8.94E-37	3.96E-35
MXLOC_008682	3331.83	826.25	345.17	7.09	3.52	-1.010210199	Down	6.41E-37	2.86E-35

124540	1612.7	764	347	14.97	7.44	-1.008699695	Down	9.03E-29	2.96E-27
MXLOC_028163	6768	376.34	171.6	1.71	0.85	-1.008461579	Down	6.72E-15	1.05E-13
619279	14386	2497.79	1132.73	5.31	2.64	-1.008173931	Down	1.64E-90	2.25E-88
n339262	5570	345	155.94	1.91	0.95	-1.00757322	Down	3.79E-14	5.62E-13
253559	9487	714	323.66	2.31	1.15	-1.00625899	Down	3.12E-27	9.55E-26
8507	5517	1234	561	6.88	3.43	-1.004199989	Down	2.40E-45	1.35E-43
MXLOC_035544	4997.27	1575.72	704.06	9.54	4.76	-1.003027693	Down	1.23E-59	9.85E-58
n411676	3566	101.38	46.34	0.88	0.44	-1	Down	5.96E-05	0.000319882
54796	12926	93	43	0.22	0.11	-1	Down	0.000156275	0.000766709
285175	13512.98	1288.54	588.8	2.92	1.46	-1	Down	8.35E-47	4.87E-45
MXLOC_008504	6891	264.88	120	1.18	0.59	-1	Down	6.70E-11	7.65E-10
51332	11723	115	52	0.3	0.15	-1	Down	1.54E-05	9.12E-05
MXLOC_019293	3085	85.48	38.95	0.86	0.43	-1	Down	0.000170164	0.000829623
n346506	8804	142.94	64.26	0.5	0.25	-1	Down	1.39E-06	9.73E-06
MXLOC_014976	4739	182.15	82.03	1.18	0.59	-1	Down	4.48E-08	3.77E-07
n406891	6579	111.48	50.6	0.52	0.26	-1	Down	2.00E-05	0.000115813
7087	3002	147	67	1.52	0.76	-1	Down	1.25E-06	8.84E-06
344558	5608	98	45	0.54	0.27	-1	Down	9.04E-05	0.00046644
MXLOC_023816	2622	185.49	84.64	2.2	1.1	-1	Down	4.60E-08	3.86E-07
2134	4015	132	60	1.02	0.51	-1	Down	4.11E-06	2.69E-05
2298	2184	166.51	75.9	2.38	1.19	-1	Down	1.93E-07	1.50E-06
25894	4539.61	124	55	0.82	0.41	-1	Down	4.40E-06	2.87E-05

Table S4 Cis-Trans TAR Pair Prediction

lncRNA_class	distance	Overlap_class	lncRNA	mRNA	lncRNA_chr	lncRNA_start	lncRNA_end	lncRNA_length	lncRNA_chain	mRNA_chr	mRNA_start	mRNA_end	mRNA_length	mRNA_chain	minimum_free_energy_of_RNAplex	Pearson_correlation	Spearman_correlation	SC-FPKM_lncRNA	SA-FPKM_lncRNA	SB-FPKM_lncRNA	SC-FPKM_mRNA	SA-FPKM_mRNA	SB-FPKM_mRNA
cis_mRNA_up10k	1705	NA	n345940	MTCONS_00053260	chr4	110351838	110353262	315	-	chr4	110354966	110466257	9290	+	*	0.8603	0.866	0	0.12	0.12	0.75	1.26	1
tran	NA	NA	n378469	MTCONS_00051716	chr17	66097708	66111659	707	+	chr3	155538816	155572410	9728	-	-628.41	0.9855	1	0.15	0.73	0.47	2.4	2.77	2.66
tran	19485789	NA	n344565	MTCONS_00003518	chr1	247353145	247372435	2205	+	chr1	227751220	227867357	4453	+	-1239.05	0.9885	1	0.03	0.01	0	2.1	1.78	1.5
cis_mRNA_overlap	0	Lnc-AntiOverlap-mRNA	n344565	MTCONS_00007544	chr1	247353145	247372435	2205	+	chr1	247354222	247374158	484	-	*	-0.9773	-1	0.03	0.01	0	2.32	3.03	3.8
cis_mRNA_overlap	0	Lnc-CompleteIn-mRNAExon	n335187	MTCONS_00055976	chr5	61661136	61669597	308	+	chr5	61601975	61684340	5447	+	-158.93	0.9542	0.866	0	0.12	0.12	19.33	20.83	20.37
cis_mRNA_overlap	0	mRNA	n409697	MTCONS_00036490	chr19	46112658	46142675	1974	-	chr19	46112658	46148864	2975	-	-1574.2	-1	-1	0.22	0	0.13	3.34	6.29	4.54
cis_mRNA_dw20k	7950	NA	n364267	MTCONS_00003351	chr1	212802453	212811835	7580	-	chr1	212781994	212794504	2777	+	*	-0.8709	-0.866	0.06	0.06	0.07	0.8	0.51	0.21
cis_mRNA_up10k	3068	NA	LTCONS_00015742	MTCONS_00017658	chr12	52245628	52249139	3512	+	chr12	52211670	52242561	4777	-	*	0.992	1	1.59	2.53	1.71	10.12	11.51	10.47
cis_mRNA_overlap	0	mRNA	n379479	MTCONS_00052934	chr4	68567079	68946670	3352	+	chr4	68566991	68603076	6309	+	-1522.17	-0.9598	-1	0.39	0.54	0.67	2.82	2.4	2.29
cis_mRNA_dw20k	18615	NA	n367085	MTCONS_00053797	chr4	184653378	184658726	1925	-	chr4	184580446	184634764	5493	+	*	0.8842	1	0.59	0.83	0.48	2.96	3.18	2.32
cis_mRNA_up10k	1520	NA	LTCONS_00027257	MTCONS_00025799	chr16	2787077	2800811	1837	-	chr16	2802330	2809913	1556	+	*	-0.9959	-0.866	0.15	0.16	0.25	1.03	1.03	0.61
tran	NA	NA	n387042	MTCONS_00000290	chr17	894694	946816	838	+	chr1	10002829	10045765	4157	+	-806.9	-0.6605	-0.866	0.04	0.04	0.15	1.15	0.73	0.62
cis_mRNA_up10k	178	NA	n372578	MTCONS_00014359	chr11	82783124	82786230	2168	+	chr11	82683907	82782947	10135	-	*	0.9965	1	3.59	3.96	3.79	7.95	8.89	8.39
cis_mRNA_overlap	0	Lnc-AntiOverlap-mRNA	n406901	MTCONS_00044979	chr20	61885330	61892967	7638	+	chr20	61872135	61885892	1346	-	*	0.9309	1	0.1	0.08	0.09	11.27	5.09	6.08
tran	NA	NA	n367049	MTCONS_00045666	chr4	148530703	148538396	568	-	chr21	48055527	48093443	20399	+	-569.2	0.8095	1	0.13	0.41	0.23	6.01	6.61	6.57
tran	NA	NA	n380358	MTCONS_00021981	chr11	82783146	82803380	340	+	chr14	20833826	20881616	12237	-	-357.69	0.9639	1	0	0.4	0.91	1.5	1.64	2.22
tran	NA	NA	n380358	MTCONS_00040646	chr11	82783146	82803380	340	+	chr2	38783672	38831077	10590	-	-232.14	-0.8915	-1	0	0.4	0.91	1.57	1.44	1.42
tran	NA	NA	n380358	MTCONS_00043639	chr11	82783146	82803380	340	+	chr20	47835832	47860620	3762	+	-220.43	0.9748	1	0	0.4	0.91	4.33	6.3	7.43
cis_mRNA_up10k	126	NA	n340523	MTCONS_00056187	chr5	77654625	77656214	1362	-	chr5	77656339	77776580	6161	+	*	0.9336	1	1.96	1.28	1.3	1.62	0.92	1.19
tran	NA	NA	n324400	MTCONS_00054690	chr6	126540032	126565078	583	+	chr4	103938346	103998328	7376	-	-327.87	0.7303	1	0.7	0.95	0.74	4.23	4.55	4.5
tran	NA	NA	n325233	MTCONS_00024692	chr2	97333063	97335485	555	+	chr15	43650298	43663198	5882	-	-390.95	0.9813	1	4.98	6.74	6.07	6.1	6.84	6.69
cis_mRNA_overlap	0	Lnc-AntiOverlap-mRNA	n342020	MTCONS_00036937	chr19	55701076	55704619	1027	+	chr19	55692533	55720874	4031	-	*	0.9964	1	0	0.06	0.03	1.97	2.51	2.2
cis_mRNA_up10k	4328	NA	n381086	MTCONS_00000650	chr1	26551811	26556330	1077	+	chr1	26560657	26608744	6727	+	*	-0.719	-1	0.16	0.09	0.44	1.2	1.57	1.16
cis_mRNA_overlap	0	mRNA	n339248	MTCONS_00048274	chr3	10857917	10980146	1991	+	chr3	10857737	10966834	2565	+	-1173.49	-0.7053	-1	1.21	1.73	1.25	1.44	1.24	1.28
cis_mRNA_dw20k	5429	NA	n386393	MTCONS_00056157	chr5	74907301	74966977	1121	+	chr5	74829990	74901873	11456	+	*	-0.9969	-1	0.28	0.23	0.22	1.21	1.64	1.68
cis_mRNA_dw20k	3018	NA	n386393	MTCONS_00058041	chr5	74907301	74966977	1121	+	chr5	74969994	75013313	2107	-	*	0.903	1	0.28	0.23	0.22	3.16	1.79	0
tran	NA	NA	n324241	MTCONS_00039148	chr7	13860505	13865175	359	-	chr2	168148819	168730621	6608	+	-457.94	-0.6069	-1	3.33	3.24	2.38	10.67	15.23	15.46
tran	NA	NA	n324241	MTCONS_00030957	chr7	13860505	13865175	359	-	chr17	37408059	37559422	11920	-	-470.14	-0.9963	-0.866	3.33	3.24	2.38	0.62	0.62	0.64
cis_mRNA_overlap	0	mRNA	n326561	MTCONS_00001476	chr1	76249597	76257629	369	+	chr1	76251886	76378923	4608	+	*	0.8636	1	0.42	0.48	0.38	0.63	0.66	0.41
tran	NA	NA	n375018	MTCONS_00037620	chr16	72910	75121	1052	+	chr2	32288518	32383018	5380	+	-602.35	0.7043	1	0.61	1	0.7	2.96	3.95	3.91
cis_mRNA_up10k	5513	NA	n375018	MTCONS_00027113	chr16	72910	75121	1052	+	chr16	64484	67398	2407	-	*	0.699	1	0.61	1	0.7	2.83	4.39	4.34
tran	NA	NA	LTCONS_00074732	MTCONS_00035915	chrX	102025998	102047742	1022	+	chr19	22165269	22193783	5010	-	-476.2	-0.7649	-1	0.93	0.94	0.97	2.78	1	0.77
tran	NA	NA	n372715	MTCONS_00041200	chr11	58002206	58006146	280	-	chr2	85829929	85839179	8793	-	-455.86	-0.9984	-1	2.34	2.1	2.01	3.03	3.42	3.53
tran	NA	NA	n368666	MTCONS_00004585	chr6	35307829	35308188	320	-	chr1	31648621	31712835	6994	-	-304.47	0.8809	1	3.64	4.54	3.7	5.43	6.82	6.16
tran	NA	NA	n326200	MTCONS_00045666	chr1	223954929	224006568	1530	+	chr21	48055527	48093443	20399	+	-1160.79	0.9751	1	4.81	6.34	5.9	6.01	6.61	6.57
cis_mRNA_up10k	3203	NA	n346447	MTCONS_00032515	chr18	56320675	56335416	1479	+	chr18	56338618	56421473	9175	+	*	0.9003	1	0.02	0.13	0.06	0.73	1.74	1.5
tran	NA	NA	n325587	MTCONS_00040816	chr16	31623102	31624840	582	+	chr2	53889562	54014214	9905	-	-667.98	0.9013	1	1.01	1.57	0.95	5.36	6.05	4.64
cis_mRNA_overlap	0	Lnc-AntiOverlap-mRNA	LTCONS_00040284	MTCONS_00037258	chr2	9545753	9563753	2714	-	chr2	9346894	9547571	7485	+	*	-0.9747	-1	3.22	2.56	2.48	0.85	1.12	1.25
cis_mRNA_up10k	2150	NA	n377860	MTCONS_00002789	chr1	173833039	173835344	413	-	chr1	173837493	173872659	19182	+	*	-0.9637	-1	11.48	10.21	6.6	3.47	3.75	4.03
cis_mRNA_dw20k	7044	NA	n340558	MTCONS_00030636	chr17	19238462	19239440	967	-	chr17	19246483	19266046	1256	-	*	0.8792	1	0.11	0.17	0.03	0.13	0.5	0.08
tran	NA	NA	n326767	MTCONS_00042413	chr1	129653	130202	323	-	chr2	208470330	208490126	5962	-	-421.16	0.9895	0.866	1.11	1.76	0.99	1.77	1.78	1.77
tran	NA	NA	n326767	MTCONS_00014101	chr1	129653	130202	323	-	chr11	65540198	65549058	8762	-	-419.31	0.9896	1	1.11	1.76	0.99	3.26	3.79	3.04
cis_mRNA_dw20k	11448	NA	n346161	MTCONS_00048495	chr3	37420195	37425133	1089	+	chr3	37284682	37408748	8330	+	*	-0.9935	-1	0.45	0.26	0.23	0.01	1.45	1.47
tran	NA	NA	n345134	MTCONS_00030957	chr11	12093738	12094453	713	+	chr17	37408059	37559422	11920	-	-650.47	0.7559	0.866	0.15	0.29	0.36	0.62	0.62	0.64
tran	NA	NA	n387365	MTCONS_00049362	chr5	81436374	81437039	666	+	chr3	124449213	124470145	8967	+	-1378.6	0.8769	1	4.6	5.69	5.51	3.28	3.93	3.53
tran	NA	NA	n387365	MTCONS_00039494	chr5	81436374	81437039	666	+	chr2	201980877	202038063	11977	+	-1298.8	0.9275	1	4.6	5.69	5.51	0.42	0.83	0.62
tran	NA	NA	n387365	MTCONS_00004585	chr5	81436374	81437039	666	+	chr1	31648621	31712835	6994	-	-1288.45	0.9428	1	4.6	5.69	5.51	5.43	6.82	6.16
tran	NA	NA	n370462	MTCONS_00052157	chr8	38721756	38725271	1173	-	chr3	197299019	197354752	9196	-	-1197.56	-0.912	-0.866	0.39	0.33	0.33	1.79	2.76	2.36
cis_mRNA_dw20k	13475	NA	LTCONS_00030940	MTCONS_00030944	chr17	36890063	36908470	7190	-	chr17	36921944	36981589	9349	-	*	0.9946	1	0.6	0.85	0.87	3.12	3.8	3.95
cis_mRNA_up10k	119	NA	n377187	MTCONS_00046243	chr21	46707965	46717721	5121	+	chr21	46682942	46707847	3837	-	*	-0.9062	-1	0.57	0.32	0.46	0.95	1.13	1.1
cis_mRNA_up10k	80	NA	LTCONS_00056202	MTCONS_00058074	chr5	79287167	79315924	1109	+	chr5	79272432	79287088	8041	-	*	0.982	1	1.57	1.07	0.87	3.49	3.31	3.16
tran	NA	NA	n370931	MTCONS_00052157	chr9	38949737	38968330	810	+	chr3	197299019	197354752	9196	-	-446.18	0.922	1	0.04	0.21	0.08	1.79	2.76	2.36
tran	NA	NA	n324498	MTCONS_00045666	chr6	53355094	53355491	356	-	chr21	48055527	48093443	20399	+	-274.6	0.8764	1	0.34	0.49	0.41	6.01	6.61	6.57
cis_mRNA_up10k	204	NA	n409369	MTCONS_00052327	chr4	666225	668127	310	-	chr4	668330	675908	924	+	*	0.9456	1	12.64	10.73	17.61	2.19	1.41	2.78
tran	NA	NA	n338382	MTCONS_00036975	chr1	178449742	178450340	599	+	chr19	56891224	56904973	6027	-	-384.94	0.8168	1	0.37	0.45	0.66	1.17	1.41	1.46
tran	NA	NA	n338382	MTCONS_00035019	chr1	178449742	178450340	599	+	chr19	56879504	56894634	6776	+	-486.37	0.9746	1	0.37	0.45	0.66	1.61	1.63	2.01
cis_mRNA_dw20k	7622	NA	LTCONS_00048648	MTCONS_00052193	chr3	44903385	44907182	921	+	chr3	44914803	44915539	737	.	*	-0.7126	-1	0.57	1.26	0.64	1.56	0.44	0.65
tran	3809354	NA	n386103	MTCONS_00024535	chr15	28986247	28996820	967	+	chr15	32806173	32825942	1395	-	-401.06	0.9999	1	0.13	0	0.26	0.2	0	0.39
tran	NA	NA	LTCONS_00067909	MTCONS_00014101	chr7	128114894	128115838	945	.	chr11	65540198	65549058	8762	-	-749.5	0.8827	1	0.75	0.92	0.3	3.26	3.79	3.04
tran	NA	NA	n326161	MTCONS_00058457	chr1	237499648	237527860	448	-	chr5	132028323	132073287	6288	-	-418.41	-0.8907	-1	0.17	0.6	0.08	40.31	38.91	42.38
cis_mRNA_dw20k	2766	NA	n410475	MTCONS_00024037	chr15	76196200	76227608	3343	+	chr15	76135622	76193435	3045	+	*	-0.954	-1	1.25	1.38	0.11	6.41	5.36	8.05
cis_mRNA_up10k	58	NA	LTCONS_00015167	MTCONS_00017049	chr12	6982578	6996784	4417	+	chr12	6980061	6982521	1543	-	*	0.7893	1	1.6	2.5	2.35	1.5	1.92	1.6
cis_mRNA_up10k	5511	NA	n340463	MTCONS_00030249	chr17	3710047	3712148	2102	-	chr17	3617919	3704537	3765	-	*	0.9946	1	6.86	7.56	7.29	1.88	2.09	1.99

cis_mRNA_dw20k	18950	NA	LTCONS_00028403	MTCONS_00025960	chr16	14379754	14380310	557	.	chr16	14165194	14360805	8961	+	*		-0.9585	-1	1.66	1.45	0.65	1.23	1.38	1.55
cis_mRNA_overlap	0	Lnc-CompleteIn-mRNAExon	n384393	MTCONS_00052118	chr3	195961240	195964785	3546	-	chr3	195961085	196016887	7990	-		-2011.48	0.9188	1	2.86	3.2	3.61	1.68	1.71	2.1
cis_mRNA_dw20k	14791	NA	n375802	MTCONS_00029004	chr17	27894167	27895678	646	+	chr17	27717356	27879377	12678	+	*		-0.9276	-1	0	0.08	0.05	10.01	8	9.43
cis_mRNA_up10k	103	NA	LTCONS_00018891	MTCONS_00016601	chr12	120425702	120427510	1809	.	chr12	120427612	120532875	3958	+	*		0.6094	1	2.65	1.74	2.68	14.3	14.07	16.1
tran	NA	NA	n381240	MTCONS_00045666	chr1	145514196	145515899	1011	-	chr21	48055527	48093443	20399	+		-1242.15	0.9748	1	1.09	1.99	1.73	6.01	6.61	6.57
cis_mRNA_up10k	2135	NA	n377360	MTCONS_00047665	chr22	31367353	31374831	4892	+	chr22	31318633	31365219	8600	-	*		-0.9866	-1	1.17	0.92	0.75	4.38	4.67	5.02
cis_mRNA_up10k	343	NA	LTCONS_00030149	MTCONS_00028436	chr17	632317	635476	3160	-	chr17	635818	647537	3899	+	*		0.9988	1	1.18	1.26	1.34	0.18	0.31	0.42
tran	NA	NA	n366973	MTCONS_00012979	chr4	41878498	41884628	632	-	chr11	126152982	126169765	7744	+		-533.3	-0.9972	-1	1.5	1.37	0.57	0.93	0.96	1.07
tran	NA	NA	n366973	MTCONS_00008384	chr4	41878498	41884628	632	-	chr10	70480968	70553348	6024	+		-520.11	-0.9492	-1	1.5	1.37	0.57	2.2	2.64	3.21
cis_mRNA_overlap	0	Lnc-CompleteIn-mRNAIntron	n339293	MTCONS_00048495	chr3	37297354	37297961	608	+	chr3	37284682	37408748	8330	+	*		0.945	1	0.06	0.27	0.38	0.01	1.45	1.47
tran	NA	NA	n346273	MTCONS_00035069	chr22	22008050	22009208	901	+	chr19	57946666	57966538	12176	+		-803.23	0.7996	1	0.79	1.51	1.68	3.35	3.41	3.61
tran	NA	NA	n346273	MTCONS_00036975	chr22	22008050	22009208	901	+	chr19	56891224	56904973	6027	-		-646.3	0.9998	1	0.79	1.51	1.68	1.17	1.41	1.46
tran	NA	NA	n346273	MTCONS_00035019	chr22	22008050	22009208	901	+	chr19	56879504	56894634	6776	+		-721.21	0.6808	1	0.79	1.51	1.68	1.61	1.63	2.01
tran	270018	NA	n405981	MTCONS_00050144	chr3	195686792	195717150	2491	-	chr3	195384910	195416775	3199	+		-1613.11	-0.8952	-1	0.62	0.87	0.91	4.53	3.56	2.34
cis_mRNA_overlap	0	Lnc-CompleteIn-mRNAIntron	n384643	MTCONS_00057827	chr5	54824670	54830370	5701	-	chr5	54717354	54830905	4831	-	*		0.8062	1	0.45	0.67	0.63	1.02	1.22	1.07
cis_mRNA_overlap	0	mRNA	n335706	MTCONS_00035266	chr19	2431552	2456947	1821	-	chr19	2425527	2456958	4189	-		-1598.21	0.9078	1	0	0.07	0.02	0.48	0.84	0.72
tran	660966	NA	n386310	MTCONS_00047425	chr22	20387890	20400957	3875	-	chr22	21061922	21213091	8306	-		-2798.44	0.6574	1	3.23	5.74	3.59	1.07	1.66	1.62
cis_mRNA_overlap	0	Lnc-AntiOverlap-mRNA	LTCONS_00004223	MTCONS_00000441	chr1	16786595	16825744	25713	-	chr1	16767167	16788102	3716	+		-3159.7	-0.6186	-0.866	2.34	2.42	2.42	0.59	0.46	0.57
tran	NA	NA	n323887	MTCONS_00000634	chrX	41019936	41020975	835	+	chr1	26127068	26144764	4509	+		-429.13	-0.9364	-0.866	0.3	0.19	0.19	1.21	2.84	2.26
tran	111670	NA	n407587	MTCONS_00033690	chr19	12203078	12225494	3769	+	chr19	12075841	12091409	2853	+		-1320.98	-1	-1	0.57	0.73	0.31	1.37	0.93	2.06
tran	56253	NA	n407587	MTCONS_00035585	chr19	12203078	12225494	3769	+	chr19	12124713	12146826	3432	-		-1601.82	-0.964	-1	0.57	0.73	0.31	0.49	0.41	0.54
cis_mRNA_overlap	0	Lnc-AntiOverlap-mRNA	n379822	MTCONS_00053990	chr4	2936667	2942231	1360	+	chr4	2939568	2965343	3861	-	*		0.9902	1	0.07	0.43	0	5.4	6.12	5.39
cis_mRNA_overlap	0	Lnc-AntiOverlap-mRNA	n338539	MTCONS_00007247	chr1	224407315	224415745	1323	+	chr1	224414158	224517872	3913	-	*		-0.6306	-0.866	0.08	0.02	0.02	0.75	1.4	0.86
cis_mRNA_up10k	9297	NA	n381106	MTCONS_00004613	chr1	32697262	32707311	2713	-	chr1	32681087	32687966	1311	-	*		0.9717	1	0.06	0.18	0.25	0.44	1.19	2.21
tran	NA	NA	n367710	MTCONS_00039148	chr5	10137250	10138477	943	+	chr2	168148819	168730621	6608	+		-846.04	0.9891	1	1.09	1.41	1.49	10.67	15.23	15.46
tran	NA	NA	n380928	MTCONS_00027368	chr12	43373057	43390289	552	-	chr16	4845114	4853102	3193	-		-555.57	-0.6176	-0.866	0.16	0.06	0.06	2.18	3.36	2.36
tran	NA	NA	n380928	MTCONS_00041200	chr12	43373057	43390289	552	-	chr2	85829929	85839179	8793	-		-364.13	-0.9778	-0.866	0.16	0.06	0.06	3.03	3.42	3.53
cis_mRNA_up10k	40	NA	LTCONS_00020483	MTCONS_00019531	chr13	88236873	88323528	1245	-	chr13	88323567	88332209	5472	+	*		0.8828	1	0.26	0.04	0.4	1.96	1.67	3.35
tran	NA	NA	n372462	MTCONS_00033841	chr11	116645826	116646592	598	-	chr19	17325517	17330638	3216	+		-679.86	-0.9969	-1	11.9	9.24	10.71	2.25	2.56	2.41
cis_mRNA_dw20k	12682	NA	n383427	MTCONS_00035875	chr19	19867183	19887222	2534	-	chr19	19899903	19932573	3778	-	*		0.9974	1	1.41	0.92	0.58	4.89	3.95	3.11
tran	NA	NA	n368783	MTCONS_00008481	chr6	147480061	147502128	453	+	chr10	75503992	75540350	11297	+		-522.78	0.9717	1	1.89	3.14	2.73	7.32	8.55	7.89
tran	NA	NA	n368783	MTCONS_00006983	chr6	147480061	147502128	453	+	chr1	204391758	204464604	8332	-		-549.48	-0.9376	-1	1.89	3.14	2.73	3.69	3.33	3.56
tran	19252440	NA	n405705	MTCONS_00031605	chr17	43583249	43597889	7238	-	chr17	62850328	62915584	3402	-		-1833.34	0.6361	1	2.28	2.09	4.15	1.24	0.84	1.28
cis_mRNA_dw20k	10176	NA	n408117	MTCONS_00042754	chr2	242026509	242041747	2758	-	chr2	242051922	242089877	6960	-	*		0.8624	1	3.83	4.05	2.91	1.22	1.54	1.06
cis_mRNA_overlap	0	Lnc-CompleteIn-mRNAExon	n408117	MTCONS_00042748	chr2	242026509	242041747	2758	-	chr2	242026347	242041785	5463	-		-1676.69	0.9624	1	3.83	4.05	2.91	4.7	6.26	2.74
tran	NA	NA	n407042	MTCONS_00024396	chr1	14362	29370	1769	-	chr15	102500790	102516854	2835	+		-1329.98	0.996	1	0.72	0	2.58	1.44	1.06	3.01
tran	NA	NA	n407042	MTCONS_00014973	chr1	14362	29370	1769	-	chr12	73456	91263	2647	+		-1320.84	-0.7557	-1	0.72	0	2.58	6.23	9.05	6.03
cis_mRNA_dw20k	10	NA	n409272	MTCONS_00029271	chr17	39981334	39992488	1490	-	chr17	39968942	39981325	4759	+	*		0.9999	0.866	16.04	15.97	10.2	0.02	0.02	0
cis_mRNA_up10k	85	NA	n376048	MTCONS_00032405	chr18	42257061	42259599	2428	-	chr18	42259683	42648637	10615	+	*		-0.9781	-1	0.2	0.36	0.28	1.23	0.85	0.97
cis_mRNA_dw20k	692	NA	n342436	MTCONS_00036790	chr19	52561743	52566956	675	-	chr19	52567647	52599018	5551	-	*		0.995	1	0.97	0.78	0.86	2.97	2.34	2.66
cis_mRNA_overlap	0	Lnc-CompleteIn-mRNAExon	n338129	MTCONS_00005628	chr1	111197952	111199662	1711	-	chr1	111196209	111218054	10079	-		-712.93	0.9964	1	0.33	0.89	0.17	1.45	2.89	0.83
cis_mRNA_up10k	9086	NA	LTCONS_00036214	MTCONS_00036211	chr19	38219769	38225978	6210	-	chr19	38183957	38210684	7661	-	*		-0.7803	-0.866	3.96	4.45	4.14	0.73	0.71	0.71
cis_mRNA_overlap	0	mRNA-AntiCompleteIn-LncIntron	n386214	MTCONS_00036372	chr19	42901326	43156171	1112	+	chr19	42905569	42927913	3086	-	*		0.9999	1	0.85	1.52	0.45	1.91	2.37	1.62
cis_mRNA_dw20k	10614	NA	n339252	MTCONS_00026714	chr16	67595314	67596212	899	-	chr16	67562710	67584701	17635	+	*		0.9609	1	0.95	1.19	1.45	1.9	2.73	3.04
cis_mRNA_overlap	0	Lnc-AntiOverlap-mRNA	LTCONS_00050258	MTCONS_00048194	chr3	9365129	9404711	2981	-	chr3	9404225	9435671	11861	+	*		-0.9197	-1	2.37	2.6	1.96	0.35	0.3	0.37
tran	3725402	NA	n364378	MTCONS_00002104	chr1	148556023	148557204	733	+	chr1	144593657	144830622	7756	+		-495.98	0.8748	1	3.15	4.54	3.19	7.98	8.75	8.37
tran	NA	NA	n339200	MTCONS_00043639	chr16	47563101	47563827	494	+	chr20	47835832	47860620	3762	+		-289.45	0.9329	0.866	0.08	0.14	0.14	4.33	6.3	7.43
cis_mRNA_dw20k	733	NA	n409330	MTCONS_00031929	chr17	79860777	79869077	3314	-	chr17	79869809	79876058	4407	-	*		-0.9998	-1	1.08	0	0.05	1.37	1.94	1.9
cis_mRNA_up10k	1884	NA	n337797	MTCONS_00000663	chr1	27018623	27020622	2000	-	chr1	27022505	27108601	8599	+	*		-0.9757	-1	0.31	0.2	0.28	3.57	5.98	4.7
cis_mRNA_dw20k	15243	NA	n345778	MTCONS_00055289	chr4	185286377	185293634	2002	-	chr4	185308876	185395726	2121	-	*		0.7946	0.866	0.02	0.02	0	0.68	0.38	0.19
cis_mRNA_overlap	0	AntiCompleteIn-mRNAIntron	n364284	MTCONS_00003463	chr1	224795999	224803149	3540	-	chr1	224622592	224928257	1899	+	*		-0.7748	-1	0.15	0.21	0.19	1.29	0.69	1.27
tran	183327	NA	n408179	MTCONS_00003844	chr1	323892	328581	4370	+	chr1	131337	140566	5257	-		-3159	0.9853	1	2.74	3.61	2.98	0.2	0.57	0.24
tran	NA	NA	n408179	MTCONS_00027110	chr1	323892	328581	4370	+	chr16	90229013	90235598	2977	+		-2152.45	0.8211	1	2.74	3.61	2.98	0.06	0.95	0.79
cis_mRNA_overlap	0	mRNA	n410023	MTCONS_00028172	chr16	75632247	75657221	3603	-	chr16	75626950	75657267	9661	-		-2091.67	0.9838	1	0.66	0.89	0.88	3.3	3.52	3.47
tran	NA	NA	n377642	MTCONS_00030211	chr16	31711911	31718758	1812	+	chr17	2592315	2615083	5748	-		-1401	-0.6842	-0.866	2.12	1.15	1.15	1.98	2.13	2.61
tran	NA	NA	n367738	MTCONS_00016715	chr5	45035301	45098749	820	+	chr12	124196865	124246302	6453	+		-448.95	-0.9173	-1	0.05	0.15	0	0.41	0.1	1.11
tran	NA	NA	n324484	MTCONS_00012979	chr6	71161311	71165841	397	+	chr11	126152982	126169765	7744	+		-358.82	-0.9791	-0.866	0.43	0.43	0.41	0.93	0.96	1.07
tran	NA	NA	n324484	MTCONS_00050666	chr6	71161311	71165841	397	+	chr3	48331780	48342946	5019	-		-368.56	-0.9545	-0.866	0.43	0.43	0.41	5.86	5.64	6.36
tran	NA	NA	n325608	MTCONS_00049876	chr14	100485068	100488842	610	+	chr3	179040534	179059936	10093	+		-518.97	0.9844	1	0.3	0.11	0.21	2.68	2.25	2.41
cis_mRNA_overlap	0	Lnc-CompleteIn-mRNAIntron	n339893	MTCONS_00054182	chr4	24897087	24899819	2723	-	chr4	24805140	24914602	6595	-	*		0.9234	1	0.04	0.02	0.03	3.04	2.54	2.61

tran	NA	NA	n376479	MTCONS_00041449	chr19	13781870	13789989	773	-	chr2	101887052	101925178	6597	-		-548.18	0.8705	1	0.34	0.99	0.46	1.64	2.09	1.93
tran	NA	NA	n376479	MTCONS_00024784	chr19	13781870	13789989	773	-	chr15	50849351	50979012	10401	-		-662.94	-0.8834	-1	0.34	0.99	0.46	2.04	1.46	1.68
cis_mRNA_overlap	0	mRNA	n410153	MTCONS_00029371	chr17	42785976	42827917	3398	+	chr17	42785974	42836289	2103	+		-1513.64	0.984	1	1.1	1.65	0.84	0.74	1.47	0.61
cis_mRNA_up10k	8547	NA	n410153	MTCONS_00029376	chr17	42785976	42827917	3398	+	chr17	42836463	42859216	5008	+	*		-0.767	-1	1.1	1.65	0.84	10.07	9.88	15.31
cis_mRNA_up10k	168	NA	n365451	MTCONS_00037937	chr2	61367900	61372076	3350	-	chr2	61372243	61391964	982	+	*		-0.8242	-0.866	1.22	1.03	1.22	0.96	3.9	2.63
cis_mRNA_overlap	0	AntiCompleteIn-mRNAIntron	LTCONS_00025605	MTCONS_00024190	chr15	89154721	89155056	336	.	chr15	89148302	89175546	3267	+	*		0.9842	1	0.47	2.46	0.42	0.73	2.43	0.28
cis_mRNA_dw20k	5010	NA	n342331	MTCONS_00021439	chr14	74047398	74049622	2224	+	chr14	74035684	74042389	1398	+	*		0.9833	1	0.52	1.36	1.06	1.99	3.48	2.71
cis_mRNA_up10k	667	NA	n378397	MTCONS_00002789	chr1	173834997	173836827	841	-	chr1	173837493	173872659	19182	+	*		0.9594	1	1.09	1.5	2.76	3.47	3.75	4.03
cis_mRNA_dw20k	6372	NA	n364831	MTCONS_00040131	chr2	1624282	1629191	1641	-	chr2	1635562	1748334	7575	-	*		-0.982	-1	0.81	1.01	1.04	13.16	11.64	10.95
cis_mRNA_dw20k	2616	NA	n408346	MTCONS_00015943	chr12	57623356	57628718	2215	+	chr12	57612293	57620741	3548	+	*		0.9482	1	0.11	0.92	0.45	0.16	0.67	0.22
cis_mRNA_dw20k	3033	NA	n378669	MTCONS_00047425	chr22	21056965	21058890	737	+	chr22	21061922	21213091	8306	-	*		0.9709	1	1.82	2.18	2.07	1.07	1.66	1.62
tran	NA	NA	LTCONS_00055968	MTCONS_00039605	chr5	60452705	60453538	834	+	chr2	209130833	209223538	9831	+		-660.59	0.9896	1	2.23	1.83	1.61	0.78	0.71	0.69
tran	NA	NA	LTCONS_00055968	MTCONS_00018358	chr5	60452705	60453538	834	+	chr12	109886460	109915155	3973	-		-435.97	0.8737	1	2.23	1.83	1.61	2.27	2.13	1.54
cis_mRNA_overlap	0	mRNA	LTCONS_00055934	MTCONS_00055937	chr5	56204939	56213386	1723	+	chr5	56205844	56213386	1837	+		-703.94	0.7469	1	0.15	0.51	0.61	1.72	1.81	2.46
cis_mRNA_up10k	2623	NA	n338245	MTCONS_00002358	chr1	153591284	153603836	2184	-	chr1	153606458	153618837	2083	+	*		-0.9999	-0.866	0.09	0.09	0.1	3.68	3.67	2.94
cis_mRNA_overlap	0	AntiCompleteIn-mRNAIntron	n379814	MTCONS_00010658	chr10	103596053	103599032	2272	+	chr10	103585731	103603677	3718	-	*		0.8409	1	0.04	0.11	0.16	0.42	0.45	0.98
cis_mRNA_dw20k	6325	NA	n379814	MTCONS_00010665	chr10	103596053	103599032	2272	+	chr10	103605356	103815932	4219	-	*		0.9536	1	0.04	0.11	0.16	0.29	0.75	0.82
cis_mRNA_overlap	0	mRNA	n345152	MTCONS_00014574	chr11	110069279	110070929	858	-	chr11	110066283	110167437	2764	-	*		0.9081	1	0.08	0.15	0.04	10.54	10.81	9.75
cis_mRNA_dw20k	2791	NA	n383633	MTCONS_00037837	chr2	47713160	47715691	2000	-	chr2	47630263	47710370	5062	+	*		-0.6392	-0.866	0.07	0.02	0.02	12.55	13.66	12.75
tran	NA	NA	n326382	MTCONS_00014300	chr1	147843215	147843920	561	-	chr11	75297963	75382024	11247	-		-352.74	-0.9965	-1	0.33	0	0.06	14.6	18.81	17.71
cis_mRNA_overlap	0	mRNA	n345373	MTCONS_00033951	chr19	20967253	21017607	5212	+	chr19	20959100	20977400	1059	+		-488.19	-0.9549	-1	0.41	0.38	0.53	0.75	1.09	0.37
cis_mRNA_dw20k	17612	NA	n410461	MTCONS_00002794	chr1	174982094	174992591	2237	-	chr1	174768881	174964483	7048	+	*		-0.7402	-0.866	0.28	0.28	1.36	22.06	24.84	20.8
cis_mRNA_dw20k	7622	NA	LTCONS_00048647	MTCONS_00052193	chr3	44902441	44907182	2136	+	chr3	44914803	44915539	737	.	*		-0.7055	-1	3.53	3.86	3.56	1.56	0.44	0.65
tran	NA	NA	n369231	MTCONS_00023976	chr6	41349175	41350213	937	-	chr15	74420550	74429213	4335	+		-672.23	-0.7041	-0.866	0.17	0.13	0.17	5.02	6.2	1.73
cis_mRNA_overlap	0	mRNA	n409147	MTCONS_00031971	chr17	80347086	80376513	4505	-	chr17	80345790	80376632	5721	-		-3395.36	0.9996	1	0.41	0.29	0.83	0.44	0.38	0.69
cis_mRNA_dw20k	14231	NA	n383778	MTCONS_00039296	chr2	179278390	179298394	1247	+	chr2	179059208	179264160	7259	+	*		-0.9282	-1	0.88	0.77	0.67	0.25	0.46	0.49
cis_mRNA_overlap	0	Lnc-AntiOverlap-mRNA	n379906	MTCONS_00053730	chr4	174087220	174090078	593	-	chr4	174089904	174245640	4829	+	*		0.9177	0.866	0.06	0.06	0	6.52	5.42	3.77
cis_mRNA_up10k	230	NA	n410503	MTCONS_00013821	chr11	57479995	57508445	1524	+	chr11	57468662	57479766	3698	-	*		0.9912	1	0.32	0	0.4	2.67	2.19	2.9
cis_mRNA_dw20k	12621	NA	n342415	MTCONS_00037937	chr2	61404584	61416060	3084	+	chr2	61372243	61391964	982	+	*		-0.956	-1	4.66	4.09	4.18	0.96	3.9	2.63
cis_mRNA_dw20k	6159	NA	n378950	MTCONS_00040539	chr2	27580007	27581057	536	+	chr2	27587215	27593343	1964	-	*		-0.6814	-1	0.1	0.09	0.17	1.19	2.28	1.04
cis_mRNA_overlap	0	Lnc-AntiOverlap-mRNA	n379282	MTCONS_00048919	chr3	62249717	62355005	502	-	chr3	61547184	62284638	10685	+	*		-0.9887	-1	0.23	0	0.16	7.69	13.71	8.66
cis_mRNA_overlap	0	mRNA-AntiCompleteIn-LncIntron	n379282	MTCONS_00048921	chr3	62249717	62355005	502	-	chr3	62304641	62321852	3389	+	*		0.9329	1	0.23	0	0.16	8.28	7.77	7.96
tran	807033	NA	n334486	MTCONS_00057977	chr5	68936124	69521459	623	-	chr5	70328491	70585523	15856	-		-433.18	0.6147	1	0.08	0.09	0	3.97	4.43	3.95
tran	249321	NA	n334486	MTCONS_00057969	chr5	68936124	69521459	623	-	chr5	69770779	69881984	8211	-		-462.93	0.8281	1	0.08	0.09	0	3.85	4.56	3.47
cis_mRNA_dw20k	15366	NA	n337634	MTCONS_00003895	chr1	995114	1001833	974	+	chr1	1017198	1028234	2996	-	*		-0.9998	-1	1.85	2.08	1.43	1.3	1.11	1.62
tran	NA	NA	n345409	MTCONS_00052740	chr22	24268570	24274142	840	-	chr4	39063852	39137228	8789	+		-681	0.8066	0.866	0.09	0.31	0.46	0.63	0.63	0.81
cis_mRNA_up10k	466	NA	n342529	MTCONS_00045348	chr21	37504065	37528605	1499	-	chr21	37529070	37666829	8055	+	*		0.6586	0.866	0.02	0.06	0.06	1.92	2.16	3.09
tran	NA	NA	n363595	MTCONS_00047652	chr1	25579798	25594376	777	-	chr22	30771653	30783401	3679	-		-821.12	0.9999	1	0.18	0.49	0.51	4.58	5.95	6.02
tran	NA	NA	n324502	MTCONS_00040816	chr6	50589707	50596776	285	+	chr2	53889562	54014214	9905	-		-286.85	0.9996	1	0.63	0.76	0.48	5.36	6.05	4.64
cis_mRNA_overlap	0	Lnc-CompleteIn-mRNAExon	n342544	MTCONS_00042960	chr20	3898237	3899061	825	+	chr20	3869742	3907953	11798	+		-397.34	-0.9151	-1	4.44	3.59	4.21	1.78	2.66	2.34
tran	NA	NA	n332870	MTCONS_00048261	chr15	28839838	28840249	412	+	chr3	10183319	10195354	5394	+		-373.36	0.9934	1	0.84	1.32	2.08	6.11	6.49	6.89
cis_mRNA_dw20k	18205	NA	n372666	MTCONS_00013380	chr11	9137687	9142145	397	-	chr11	9160349	9286956	5001	-	*		-0.999	-1	0.1	0	0.09	12.18	15.82	12.71
cis_mRNA_dw20k	409	NA	n381294	MTCONS_00002810	chr1	176812380	176814731	2352	+	chr1	176524311	176811972	6906	+	*		0.9596	1	0.89	0.36	1.01	1.08	0.83	1.28
tran	NA	NA	LTCONS_00073839	MTCONS_00009847	chr9	69852020	69852511	492	.	chr10	42825274	42863493	8645	-		-221.14	0.9688	1	1.27	0.75	2.63	0.13	0.09	1.8
cis_mRNA_up10k	155	NA	n364340	MTCONS_00004891	chr1	45672404	45683543	320	+	chr1	45482071	45672250	6590	-	*		0.9656	1	0	0.34	0.22	3.55	5.37	4.31
tran	NA	NA	LTCONS_00070634	MTCONS_00026455	chr8	83204192	83204537	346	.	chr16	51679680	51681009	1330	+		-664.9	-0.9962	-1	0.45	1.03	0.3	1.68	1.16	1.75
tran	702760	NA	n382701	MTCONS_00025322	chr15	85747263	85747561	299	-	chr15	84941992	85044504	18018	-		-432.97	-0.9707	-1	1.59	1.8	3.77	10.55	9.49	7.34
cis_mRNA_up10k	99	NA	n363671	MTCONS_00001904	chr1	110751456	110752609	748	-	chr1	110752707	110776763	11197	+	*		-0.9718	-1	0.24	0.13	0	0.81	1.07	1.2
tran	NA	NA	n377888	MTCONS_00040619	chr1	9739858	9747632	983	-	chr2	37323735	37384351	12737	-		-805.85	-0.8486	-1	0.39	0.3	0.84	4.96	5.38	4.75
tran	NA	NA	n365591	MTCONS_00034476	chr2	214140522	214148921	607	-	chr19	44455373	44485875	16403	+		-345.53	0.9374	1	0.62	0.13	0.25	3.03	2.78	2.92
tran	NA	NA	n339167	MTCONS_00000290	chr2	231916755	231917352	598	+	chr1	10002829	10045765	4157	+		-631.66	-0.8699	-1	1.47	1.51	1.59	1.15	0.73	0.62
tran	NA	NA	n405708	MTCONS_00026455	chr12	54674533	54678663	1356	+	chr16	51679680	51681009	1330	+		-652	0.7095	1	4.81	3.75	10.1	1.68	1.16	1.75
cis_mRNA_overlap	0	Lnc-CompleteIn-mRNAExon	n407161	MTCONS_00057977	chr5	70370030	70388897	578	-	chr5	70328491	70585523	15856	-		-312.35	-0.9485	-1	0.56	0	0.87	3.97	4.43	3.95
cis_mRNA_dw20k	93	NA	n408196	MTCONS_00033248	chr18	77905807	77936315	734	+	chr18	77936407	78005397	901	-	*		0.9763	1	0.14	0.09	0.3	0.89	0.13	1.88
tran	374264	NA	LTCONS_00002098	MTCONS_00002104	chr1	144166564	144219394	638	+	chr1	144593657	144830622	7756	+		-391.57	0.9757	1	6.91	14.4	9.25	7.98	8.75	8.37
tran	NA	NA	n366150	MTCONS_00041449	chr3	184264502	184274706	571	-	chr2	101887052	101925178	6597	-		-426.33	0.8654	1	1.05	1.91	1.2	1.64	2.09	1.93
tran	NA	NA	n385921	MTCONS_00024396	chr1	15796	18061	1101	-	chr15	102500790	102516854	2835	+		-921.42	0.937	1	3.29	2.31	4.21	1.44	1.06	3.01
tran	NA	NA	n385921	MTCONS_00014973	chr1	15796	18061	1101	-	chr12	73456	91263	2647	+		-919.43	-0.9021	-1	3.29	2.31	4.21	6.23	9.05	6.03
tran	NA	NA	n366377	MTCONS_00057977	chr3	189846778	189847483	497	+	chr5	70328491	70585523	15856	-		-589.78	-0.9616	-1	4.25	3.43	4.63	3.97	4.43	3.95
cis_mRNA_dw20k	17300	NA	n384217	MTCONS_00050318	chr3	11577723	11580151	2429	+	chr3	11597450	11760988	3564	-	*		0.8347	0.866	0.03	0.05	0.03	7.52	7.91	7.04
cis_mRNA_up10k	1292	NA	n371934	MTCONS_00010975	chr10	134757471	134778793	2619	-	chr10	134742646	134756180	2179	-	*		-0.9316	-1	0.18	0.21	0.06	0.64	0.56	0.71
cis_mRNA_overlap	0	Lnc-AntiOverlap-mRNA	LTCONS_00053351	MTCONS_00054822	chr4	120375938	120473366	1820	+	chr4	120420772	120549981	7405	-	*		-0.9941	-1	0	0.91	1.51	0.11	0.04	0.01
cis_mRNA_overlap	0	mRNA	n339112	MTCONS_00027511	chr16	18849783	18861683	2016	-	chr16	18816175	18937751	16148	-		-1047.69	-0.9869	-1	1.86	1.83	0.96	2.94	3.24	4.43
tran	NA	NA	n383082	MTCONS_00024692	chr17	20319422	20320583	1162	+	chr15	43650298	43663198	5882	-		-1151.9	0.9458	1	1.3	1.64	1.47	6.1	6.84	6.69

cis_mRNA_dw20k	3163	NA	n338130	MTCONS_00024233	chr15	90788645	90789043	399	+	chr15	90777535	90785483	1703	+	*		0.9474	1	0.19	0.7	0.43	0.66	1.52	0.81
cis_mRNA_overlap	0	Lnc-AntiOverlap-mRNA	LTCONS_00054749	MTCONS_00053260	chr4	110268631	110354973	1227	-	chr4	110354966	110466257	9290	+	*		0.9176	1	0	0.22	0.19	0.75	1.26	1
cis_mRNA_dw20k	13527	NA	n338608	MTCONS_00003742	chr1	246845410	246848377	2968	+	chr1	246729562	246831884	5454	+	*		0.7351	1	0.22	0.24	0.38	0.64	1.21	1.35
tran	NA	NA	n332846	MTCONS_00044477	chr17	2515704	2516336	633	+	chr20	33432052	33460664	8075	-		-533.43	0.9458	1	0.23	0.89	0.62	6.4	11.12	7.82
tran	NA	NA	n332911	MTCONS_00051716	chr2	62230727	62231282	556	+	chr3	155538816	155572410	9728	-		-546.31	0.8855	1	0.2	0.49	0.28	2.4	2.77	2.66
tran	NA	NA	n380975	MTCONS_00042413	chr12	60486645	60489632	769	+	chr2	208470330	208490126	5962	-		-668.8	0.8854	0.866	0.61	1.04	0.81	1.77	1.78	1.77
tran	NA	NA	n372028	MTCONS_00041200	chr10	69609270	69610353	871	+	chr2	85829929	85839179	8793	-		-678.82	0.7775	1	0.55	0.56	0.61	3.03	3.42	3.53
tran	545895	NA	LTCONS_00037111	MTCONS_00036690	chr19	50578656	50579340	685	.	chr19	51125234	51143020	3035	-		-529.76	-0.9902	-1	0.23	0	0.07	1.79	3.77	3.43
cis_mRNA_dw20k	16649	NA	n381059	MTCONS_00000441	chr1	16804750	16817579	1122	-	chr1	16767167	16788102	3716	+	*		0.6186	0.866	1.13	0.96	0.96	0.59	0.46	0.57
cis_mRNA_overlap	0	Lnc-AntiOverlap-mRNA	LTCONS_00052405	MTCONS_00053990	chr4	2936626	2940906	4151	+	chr4	2939568	2965343	3861	-	*		-0.7319	-1	2.28	2.08	2.77	5.4	6.12	5.39
tran	NA	NA	n326238	MTCONS_00030957	chr1	212205860	212226119	476	+	chr17	37408059	37559422	11920	-		-478.5	0.7107	0.866	0.65	0.33	0.77	0.62	0.62	0.64
cis_mRNA_dw20k	11692	NA	n345714	MTCONS_00014181	chr11	67382251	67383718	918	-	chr11	67395409	67397579	916	-	*		-0.9914	-1	0.04	0	0.23	1.49	1.66	1.09
tran	NA	NA	n337636	MTCONS_00009539	chr1	1185157	1186714	1558	-	chr10	11502054	11537045	5168	-		-953.68	0.9802	1	0.16	0.2	0.19	2.68	3.09	3.07
cis_mRNA_dw20k	8567	NA	n364548	MTCONS_00039100	chr2	162101250	162105241	2013	+	chr2	162016844	162092684	2134	+	*		0.838	1	0.03	0	0.02	3.28	0.9	1.25
tran	NA	NA	n386435	MTCONS_00020107	chr7	55713312	55714642	1331	+	chr13	41958260	41959291	1032	-		-566.7	-0.9778	-1	0	0.12	0.05	0.24	0.09	0.15
tran	NA	NA	LTCONS_00065618	MTCONS_00003844	chr7	128166278	128303761	19895	+	chr1	131337	140566	5257	-		-4374.68	0.9695	1	0.71	1	0.81	0.2	0.57	0.24
tran	NA	NA	LTCONS_00065618	MTCONS_00027110	chr7	128166278	128303761	19895	+	chr16	90229013	90235598	2977	+		-2657.44	0.8621	1	0.71	1	0.81	0.06	0.95	0.79
tran	NA	NA	LTCONS_00052236	MTCONS_00024692	chr3	94224676	94225741	1066	.	chr15	43650298	43663198	5882	-		-558.68	0.9994	1	1.02	1.53	1.41	6.1	6.84	6.69
cis_mRNA_overlap	0	mRNA	n364757	MTCONS_00039148	chr2	168149681	168414843	462	+	chr2	168148819	168730621	6608	+		-339.43	0.9104	1	0	0.29	0.53	10.67	15.23	15.46
cis_mRNA_up10k	40	NA	n373932	MTCONS_00019531	chr13	88267539	88323528	910	-	chr13	88323567	88332209	5472	+	*		0.987	1	1.96	1.1	3.75	1.96	1.67	3.35
tran	NA	NA	n366886	MTCONS_00039148	chr4	124411423	124443261	539	+	chr2	168148819	168730621	6608	+		-376.04	0.6561	1	0	0.04	0.25	10.67	15.23	15.46
tran	NA	NA	n410152	MTCONS_00058478	chr12	17141681	17143562	1882	+	chr5	133483921	133512724	10174	-		-1131.29	0.9997	1	0.05	0.06	0.13	2.48	2.51	2.68
cis_mRNA_up10k	4975	NA	n406126	MTCONS_00009706	chr10	27534782	27541235	6454	-	chr10	27484143	27529808	4018	-	*		-0.9496	-1	0.05	0.06	0.17	20.92	19.31	16.76
cis_mRNA_overlap	0	Lnc-AntiOverlap-mRNA	n341028	MTCONS_00013723	chr11	46698079	46698679	601	+	chr11	46698581	46722200	3475	-	*		-0.9968	-1	0.12	0.06	0	3.38	4.64	6.31
cis_mRNA_dw20k	14431	NA	n383745	MTCONS_00038904	chr2	132036863	132057392	1739	+	chr2	131968620	132022433	3376	+	*		0.8816	1	0.18	0.06	0.14	1.74	0.31	0.64
cis_mRNA_overlap	0	AntiCompleteIn-mRNAIntron	n338625	MTCONS_00040134	chr2	1828184	1832439	3403	+	chr2	1792885	2335141	6493	-	*		-0.87	-0.866	0.01	0.01	0	4.37	5.08	5.81
tran	1462315	NA	n409500	MTCONS_00041776	chr2	132349268	132384999	2933	+	chr2	130824979	130886954	10720	-		-1603.67	-0.9862	-1	0	0.01	0.09	5.02	3.85	0.74
tran	NA	NA	LTCONS_00073835	MTCONS_00009847	chr9	66668650	66669481	832	.	chr10	42825274	42863493	8645	-		-495.43	0.9504	1	0.52	0.13	1.29	0.13	0.09	1.8
cis_mRNA_overlap	0	Lnc-CompleteIn-mRNAExon	n410698	MTCONS_00013938	chr11	62457734	62477091	2096	-	chr11	62457734	62494980	9309	-		-1404.9	-0.9146	-1	0.36	0.19	0.04	0.35	0.37	0.48
cis_mRNA_dw20k	29	NA	n405884	MTCONS_00036975	chr19	56879468	56891196	3508	+	chr19	56891224	56904973	6027	-	*		0.9999	1	0	0.97	1.15	1.17	1.41	1.46
cis_mRNA_overlap	0	mRNA	n405884	MTCONS_00035019	chr19	56879468	56891196	3508	+	chr19	56879504	56894634	6776	+		-1882.12	0.6549	1	0	0.97	1.15	1.61	1.63	2.01
tran	NA	NA	LTCONS_00031292	MTCONS_00046106	chr17	45968591	45973191	539	-	chr21	43369737	43374053	4099	-		-484.05	-0.7994	-1	6.26	6.45	7.16	1.66	1.21	1.09
cis_mRNA_overlap	0	Lnc-CompleteIn-mRNAExon	n339682	MTCONS_00049829	chr3	170112405	170115015	2226	+	chr3	170075203	170115015	7464	+		-819.6	0.9831	1	5.23	6.31	4.9	5.37	6.43	4.66
cis_mRNA_overlap	0	mRNA	n343019	MTCONS_00056688	chr5	138609441	138615317	1100	+	chr5	138609328	138667366	6070	+		-701.09	-0.9996	-1	0.73	1.54	1.68	0.88	0.5	0.42
cis_mRNA_up10k	671	NA	LTCONS_00055440	MTCONS_00057360	chr5	693912	696427	2516	+	chr5	659951	693242	6497	-	*		0.9976	1	3.18	2.94	4.9	1.8	1.7	3.75
tran	NA	NA	n345537	MTCONS_00000634	chr2	91690666	91693026	788	+	chr1	26127068	26144764	4509	+		-733.02	-0.9439	-1	0.38	0.18	0.31	1.21	2.84	2.26
tran	NA	NA	LTCONS_00072889	MTCONS_00020890	chr9	86534511	86536350	390	-	chr14	23775971	23782791	5725	+		-511.51	0.8633	1	1.44	1.12	0.87	2.59	2.55	2.04
cis_mRNA_overlap	0	Lnc-CompleteIn-mRNAExon	n411618	MTCONS_00045997	chr21	37406840	37432816	2893	-	chr21	37406839	37432835	3302	-		-1623.41	0.9968	1	0.68	0.84	1.23	1.21	1.32	1.72
cis_mRNA_overlap	0	mRNA	LTCONS_00022657	MTCONS_00022658	chr14	75548353	75553708	3262	-	chr14	75548353	75593778	6513	-		-1870.31	0.789	1	0.03	0.14	0.02	1.01	1.06	0.9
tran	NA	NA	n411614	MTCONS_00035989	chr11	3379157	3400452	3411	-	chr19	23921800	23941713	6491	-		-1295.47	-0.9719	-1	2.79	2.38	6.06	1.78	2.41	0.55
tran	NA	NA	n411614	MTCONS_00033981	chr11	3379157	3400452	3411	-	chr19	21711767	21723420	4770	+		-1246.75	0.999	1	2.79	2.38	6.06	1.93	1.78	2.74
tran	NA	NA	n374898	MTCONS_00040468	chr15	28936497	28944750	375	+	chr2	24461838	24583769	7406	-		-479	-0.9766	-0.866	0.62	0.19	0.19	1.89	2.37	2.51
tran	NA	NA	n324919	MTCONS_00000731	chr20	60735569	60737659	461	+	chr1	28995240	29043757	4306	+		-619.96	-0.9995	-0.866	0.82	0.82	0.58	2.63	2.67	3.76
tran	NA	NA	n345775	MTCONS_00003844	chr11	131476	134795	368	-	chr1	131337	140566	5257	-		-264.55	-0.8646	-1	1.82	0.97	1.32	0.2	0.57	0.24
tran	NA	NA	n345775	MTCONS_00027110	chr11	131476	134795	368	-	chr16	90229013	90235598	2977	+		-237.2	-0.9683	-1	1.82	0.97	1.32	0.06	0.95	0.79
cis_mRNA_up10k	61	NA	LTCONS_00025608	MTCONS_00024244	chr15	91072785	91073101	317	.	chr15	91073161	91188577	5205	+	*		0.9508	1	1.01	0.92	0.79	1.84	0.97	0.57
cis_mRNA_overlap	0	Lnc-CompleteIn-mRNAIntron	n381958	MTCONS_00018756	chr12	129297749	129299295	1547	-	chr12	129277737	129308417	2498	-	*		0.9707	1	0.56	0	1.58	1.85	0.01	3.26
cis_mRNA_up10k	1630	NA	n376177	MTCONS_00032649	chr18	76736573	76738579	1352	-	chr18	76740208	76763184	9616	+	*		-0.6616	-1	0.4	0.23	0.42	3.49	3.81	0.98
tran	NA	NA	n381258	MTCONS_00030115	chr1	155596547	155618335	1287	-	chr17	80693452	80703834	5094	+		-1906.1	-0.9409	-1	2.92	3.48	2.79	2.63	2.55	2.71
tran	NA	NA	n381258	MTCONS_00034837	chr1	155596547	155618335	1287	-	chr19	52872319	52925184	17190	+		-1657	0.8561	1	2.92	3.48	2.79	2.75	2.91	2.42
cis_mRNA_dw20k	16016	NA	n407946	MTCONS_00024135	chr15	83676702	83680393	1009	-	chr15	83654975	83660687	2430	+	*		0.7631	0.866	0.3	0.3	0.6	18.03	19.58	20.39
cis_mRNA_dw20k	1335	NA	n338988	MTCONS_00039092	chr2	160626484	160629028	2545	+	chr2	160568636	160625150	3963	+	*		0.9918	1	1.4	1.24	1.44	2.85	1.87	3.31
cis_mRNA_up10k	671	NA	n365202	MTCONS_00041409	chr2	99348118	99368056	315	+	chr2	99235515	99347448	8431	-	*		-0.7713	-1	0.38	0.12	0.11	1.94	3.16	5.69
cis_mRNA_overlap	0	mRNA	n407062	MTCONS_00008032	chr10	28821422	28912041	5924	+	chr10	28821293	28912041	6050	+		-2191.57	-0.8931	-1	0.38	0.15	0	3.62	3.78	4.47
cis_mRNA_up10k	3455	NA	n378452	MTCONS_00002789	chr1	173833118	173834039	575	-	chr1	173837493	173872659	19182	+	*		0.963	1	0.15	0.32	0.81	3.47	3.75	4.03
cis_mRNA_up10k	165	NA	n338175	MTCONS_00025543	chr15	101835624	101845942	2590	+	chr15	101821622	101835460	1072	-	*		0.7118	1	0.6	0.62	0.51	1.63	2.05	1.58
tran	NA	NA	n345394	MTCONS_00050894	chr21	11123088	11139671	1103	+	chr3	53121316	53164470	6197	-		-568.62	0.893	1	0.57	0.68	0.73	0.56	0.83	1.54
cis_mRNA_overlap	0	mRNA	n407391	MTCONS_00041244	chr2	87257798	87303536	2201	-	chr2	87257798	87303821	2442	-		-1232.4	0.7835	1	2.58	2.37	3.88	1.97	1.24	2.22
cis_mRNA_dw20k	9915	NA	LTCONS_00005701	MTCONS_00001981	chr1	114466622	114472114	1829	-	chr1	114447777	114456708	3760	+	*		-0.9253	-1	0.09	0.08	0	0.38	0.79	1.25
tran	NA	NA	n343078	MTCONS_00048261	chr11	117162062	117162865	804	+	chr3	10183319	10195354	5394	+		-718.31	-0.8984	-1	2.98	2.96	2.65	6.11	6.49	6.89
tran	NA	NA	n345463	MTCONS_00023705	chr1	150517408	150518106	699	-	chr15	52121825	52226551	12441	+		-621.89	-0.9646	-0.866	0	0.11	0.03	1.79	1.75	1.79
cis_mRNA_overlap	0	mRNA	n408180	MTCONS_00040526	chr2	27422455	27435175	3337	-	chr2	27422443	27434847	3474	-		-2113.8	-0.8986	-1	0.73	0.57	0.93	4.19	5.44	3.98
tran	NA	NA	n368360	MTCONS_00011286	chr5	149719783	149720699	800	-	chr11	6411644	6416228	3760	+		-509.03	0.9538	0.866	0.04	0.04	0.12	1.24	1.16	1.42
tran	NA	NA	n363319	MTCONS_00034879	chr1	53793905	53802889	1275	+	chr19	54057778	54083570	4063	+		-660.75	1	1	1.81	1.42	2.18	1.59	0.95	2.19
tran	NA	NA	n369003	MTCONS_00030115	chr6	25992927	26000717	1859	+	chr17	80693452	80703834	5094	+		-2047.4	-0.882	-1	4.68	4.72	3.65	2.63	2.55	2.71

tran	NA	NA	n369003	MTCONS_00034837	chr6	25992927	26000717	1859	+	chr19	52872319	52925184	17190	+	-1916.36	0.9574	1	4.68	4.72	3.65	2.75	2.91	2.42
cis_mRNA_dw20k	5528	NA	n411666	MTCONS_00025884	chr16	4746511	4784378	2667	-	chr16	4674813	4740984	3934	+	*	-0.8562	-1	2.1	2.05	3.07	3.39	4.56	2.45
tran	NA	NA	n346303	MTCONS_00028172	chr7	6748310	6751093	445	+	chr16	75626950	75657267	9661	-	-456.7	0.9967	1	0.68	1.01	0.91	3.3	3.52	3.47
cis_mRNA_up10k	162	NA	n378595	MTCONS_00022839	chr14	95624015	95646262	810	+	chr14	95552505	95623854	10599	-	*	0.7112	1	0.9	1.14	1.22	3.37	3.41	4.77
tran	NA	NA	n372245	MTCONS_00018846	chr10	130803350	130804436	892	-	chr12	133494970	133532981	9230	-	-867.97	0.9972	1	0.62	1.14	0.94	1.87	2.09	2.02
cis_mRNA_dw20k	17015	NA	n410717	MTCONS_00016452	chr12	109991521	110011358	4225	-	chr12	109915199	109974507	6159	+	*	-0.8543	-1	0.77	0.99	0.97	1.24	0.53	0.95
tran	NA	NA	n325293	MTCONS_00006983	chr2	64166988	64168884	563	-	chr1	204391758	204464604	8332	-	-502.39	-0.9343	-0.866	0.58	0.84	0.58	3.69	3.33	3.56
tran	NA	NA	n325293	MTCONS_00022937	chr2	64166988	64168884	563	-	chr14	102809051	102829253	2658	-	-300.23	0.9608	0.866	0.58	0.84	0.58	4.48	4.98	4.28
cis_mRNA_dw20k	2105	NA	n368174	MTCONS_00057033	chr5	154258474	154263999	1464	+	chr5	154236338	154256370	4200	+	*	-0.9892	-0.866	0.13	0.18	0.13	2.87	2.23	2.99
cis_mRNA_overlap	0	other	n368174	MTCONS_00058786	chr5	154258474	154263999	1464	+	chr5	154263999	154317905	8510	-	*	0.9707	0.866	0.13	0.18	0.13	0.99	1.47	1.11
cis_mRNA_overlap	0	Lnc-AntiOverlap-mRNA	n338010	MTCONS_00023976	chr15	74418256	74421179	2909	-	chr15	74420550	74429213	4335	+	*	0.7706	1	0.82	1.15	0.78	5.02	6.2	1.73
tran	NA	NA	n342452	MTCONS_00000290	chr2	166624403	166624724	316	+	chr1	10002829	10045765	4157	+	-413.32	-0.9898	-1	0.66	1.27	1.31	1.15	0.73	0.62
tran	NA	NA	n342452	MTCONS_00005711	chr2	166624403	166624724	316	+	chr1	115106620	115124265	4862	-	-410.6	0.9675	1	0.66	1.27	1.31	1.97	2.19	2.29
tran	NA	NA	n374771	MTCONS_00022023	chr15	64165701	64168589	496	-	chr14	21665762	21737638	14746	-	-588.71	-0.8184	-1	0.39	0.38	0.36	19.39	20.27	20.38
cis_mRNA_dw20k	2051	NA	n408198	MTCONS_00036858	chr19	53611132	53636173	2659	-	chr19	53638223	53662322	8294	-	-1322.15	0.9972	1	0.52	0.85	1.18	1.64	1.84	2.1
cis_mRNA_up10k	106	NA	n367847	MTCONS_00055431	chr5	269973	271631	522	-	chr5	271736	438406	6150	+	*	-0.9449	-0.866	0.21	0.13	0.13	0.07	0.33	0.46
cis_mRNA_dw20k	5094	NA	LTCONS_00028856	MTCONS_00030611	chr17	18170620	18172142	1499	+	chr17	18177235	18218321	4573	-	*	0.6026	1	0.49	0.86	0.85	1.15	2.16	1.26
cis_mRNA_dw20k	12169	NA	n382064	MTCONS_00019362	chr13	53063128	53161224	2612	+	chr13	53029820	53050960	4158	+	*	0.9807	0.866	0.09	0.02	0.09	0.88	0.38	1.01
tran	2822810	NA	n346088	MTCONS_00037080	chr19	12754021	12754504	323	-	chr19	9930431	9931212	782	.	-196.62	-0.9103	-0.866	0.11	0	0.11	2.75	3.34	2.33
tran	NA	NA	n346088	MTCONS_00042845	chr19	12754021	12754504	323	-	chr2	215711882	215712228	347	.	-180.89	-0.9744	-0.866	0.11	0	0.11	0.04	0.17	0
cis_mRNA_overlap	0	Lnc-CompleteIn-mRNAIntron	n338650	MTCONS_00019109	chr13	35935612	35936546	935	+	chr13	35516347	36246897	11212	+	*	-0.9367	-1	0.23	2.24	0.34	8.87	6.91	8.09
cis_mRNA_overlap	0	Lnc-AntiOverlap-mRNA	n406964	MTCONS_00029308	chr17	40905947	40913275	3257	-	chr17	40912939	40915644	1297	+	*	0.9996	1	0.95	2.06	0.72	0.31	1.26	0.15
cis_mRNA_overlap	0	Lnc-AntiOverlap-mRNA	n411661	MTCONS_00016427	chr12	106890188	107168609	569	-	chr12	107168077	107283094	5431	+	*	0.6449	0.866	0	0.06	0.06	1.97	2.29	2.03
cis_mRNA_up10k	46	NA	LTCONS_00018585	MTCONS_00016645	chr12	122231680	122241988	4133	-	chr12	122242033	122270592	8469	+	*	-0.9637	-1	0.59	0.34	0.37	1.66	2.14	1.96
cis_mRNA_overlap	0	mRNA	n386202	MTCONS_00033700	chr19	12305853	12349425	1627	+	chr19	12305830	12319194	422	+	*	0.9981	1	0	1.46	0.28	0.09	0.25	0.13
cis_mRNA_overlap	0	mRNA	n409240	MTCONS_00010635	chr10	103338639	103348027	2330	-	chr10	103338628	103348027	3374	-	-1760.43	0.9915	1	0.81	1.27	0.57	2.04	2.49	1.66
cis_mRNA_up10k	559	NA	n338078	MTCONS_00025285	chr15	83379225	83382778	2569	-	chr15	83328033	83378667	3641	-	*	0.9731	1	0.14	0.2	0.07	4.63	4.95	3.69
cis_mRNA_up10k	678	NA	n377418	MTCONS_00047421	chr22	20850911	20856009	1046	+	chr22	20795806	20850234	2648	-	*	-0.9998	-1	1.25	1.62	1.29	2.86	0.85	2.6
cis_mRNA_up10k	557	NA	n379593	MTCONS_00056566	chr5	127357220	127418766	2977	-	chr5	127419322	127525380	6973	+	*	0.8363	1	0.59	0.57	0.51	9.74	8.85	8.58
cis_mRNA_dw20k	1173	NA	n334529	MTCONS_00017810	chr12	56733916	56734193	278	+	chr12	56735365	56754037	4979	-	*	-0.9747	-1	0.14	0	0.03	4.98	8.37	6.95
tran	NA	NA	n364863	MTCONS_00000290	chr2	6968645	6973662	799	-	chr1	10002829	10045765	4157	+	-736.3	-0.9179	-1	0.09	0.12	0.16	1.15	0.73	0.62
cis_mRNA_up10k	5110	NA	n379581	MTCONS_00008960	chr10	104209595	104216051	1287	+	chr10	104221160	104238979	4915	+	*	0.9809	1	14.23	12.46	11.43	0.33	0.27	0.2
cis_mRNA_overlap	0	Lnc-CompleteIn-mRNAIntron	n381403	MTCONS_00007865	chr10	11295754	11298627	2874	+	chr10	11059825	11378674	8040	+	*	0.8713	1	0.02	0.08	0.01	0.46	0.59	0.26
cis_mRNA_dw20k	16090	NA	n372697	MTCONS_00013654	chr11	34876859	34880602	1535	-	chr11	34896691	34937942	1116	-	*	-0.9589	-0.866	0	0.08	0.08	3.31	0.67	1.44
tran	NA	NA	n381265	MTCONS_00019045	chr1	158169171	158173667	540	-	chr13	28712637	28847529	3366	+	-332.6	-0.9327	-1	0.07	0	0.06	2.72	3.24	2.97
tran	NA	NA	n381265	MTCONS_00024784	chr1	158169171	158173667	540	-	chr15	50849351	50979012	10401	-	-627.33	0.863	1	0.07	0	0.06	2.04	1.46	1.68
tran	NA	NA	n409314	MTCONS_00048561	chrY	14517915	14533389	824	-	chr3	40547530	40564083	6968	+	-649.01	-0.9962	-1	0.65	0	0.97	6.01	6.61	5.81
tran	14034582	NA	LTCONS_00027651	MTCONS_00027468	chr16	29502549	29506946	682	-	chr16	15457511	15467968	1333	-	-316.75	0.9884	1	3.15	4.32	3.01	2.9	6.95	1.5
cis_mRNA_up10k	350	NA	n373546	MTCONS_00019531	chr13	88096242	88323218	1675	-	chr13	88323567	88332209	5472	+	*	0.9926	1	0.1	0.09	0.31	1.96	1.67	3.35
cis_mRNA_up10k	5309	NA	LTCONS_00011013	MTCONS_00009790	chr10	33629141	33630933	1793	.	chr10	33466409	33623833	5869	-	*	0.7859	1	0.52	0.44	1.06	91.54	63.43	101.82
cis_mRNA_up10k	315	NA	LTCONS_00025570	MTCONS_00024501	chr15	29863241	29867504	4264	.	chr15	29412430	29862927	4960	-	*	0.8714	1	0.27	0.17	0.78	4.27	1.96	5.66
cis_mRNA_overlap	0	Lnc-CompleteIn-mRNAIntron	n338780	MTCONS_00040655	chr2	39242995	39244034	1040	-	chr2	39208236	39349079	10202	-	*	-0.7137	-1	0.34	0.15	0.12	2.31	2.45	3.21
cis_mRNA_overlap	0	Lnc-CompleteIn-mRNAExon	n384454	MTCONS_00054336	chr4	56294074	56297591	3518	-	chr4	56293990	56412695	10469	-	-1292.31	-0.9847	-1	0.79	0	0.6	5.26	7.13	5.38
cis_mRNA_overlap	0	Lnc-CompleteIn-mRNAIntron	n340754	MTCONS_00031402	chr17	53034121	53035787	1667	-	chr17	53029251	53046064	2725	-	*	0.8005	1	7.19	8.25	8.02	4.9	6.45	5.22
cis_mRNA_dw20k	9866	NA	n338226	MTCONS_00006049	chr1	151252500	151254405	622	-	chr1	151264270	151300191	4155	-	*	0.8255	1	0.18	0.59	0.21	1.55	3.41	2.71
cis_mRNA_up10k	1668	NA	n338226	MTCONS_00002297	chr1	151252500	151254405	622	-	chr1	151256072	151264381	6906	+	*	0.9954	1	0.18	0.59	0.21	0.68	0.97	0.73
tran	21760943	NA	n337279	MTCONS_00015507	chr12	9436255	9465837	353	+	chr12	31226779	31257730	4327	+	-286.98	0.9797	1	0	0.3	0.69	1.63	1.95	2.89
tran	NA	NA	n325191	MTCONS_00026659	chr2	107345207	107390217	616	+	chr16	66914383	66925004	7056	+	-514.74	-0.9286	-1	0.11	0.25	0.53	1.68	1.04	0.72
tran	NA	NA	n342056	MTCONS_00033964	chr12	125514067	125515683	1617	+	chr19	21324829	21380525	8338	+	-717.27	-0.98	-1	4.26	4.48	4.2	1.5	1.09	1.77
tran	NA	NA	n385118	MTCONS_00027624	chr7	32768504	32794385	1766	+	chr16	28385134	28415186	8839	-	-2937.44	0.9834	1	2.39	2.44	2.35	3.95	4.2	3.57
tran	NA	NA	n383432	MTCONS_00018846	chr19	21676922	21682695	1907	+	chr12	133494970	133532981	9230	-	-1109.8	0.9787	1	0.03	0.05	0.04	1.87	2.09	2.02
tran	4346285	NA	n383432	MTCONS_00033841	chr19	21676922	21682695	1907	+	chr19	17325517	17330638	3216	+	-1200.2	0.9998	1	0.03	0.05	0.04	2.25	2.56	2.41
tran	NA	NA	n383432	MTCONS_00023488	chr19	21676922	21682695	1907	+	chr15	40697680	40713512	6472	+	-1390.67	0.9382	1	0.03	0.05	0.04	1.46	2.18	2.05
cis_mRNA_dw20k	4859	NA	n411654	MTCONS_00031262	chr17	44668035	44834828	3945	+	chr17	44839686	44849065	3595	-	*	-0.9785	-1	0.09	0.29	0.15	3.23	2.31	3.14
cis_mRNA_overlap	0	mRNA	n346146	MTCONS_00008144	chr10	38645331	38666968	1629	+	chr10	38645308	38667433	1968	+	-801.02	-0.7869	-1	0.11	0.14	0.27	0.14	0.03	0
tran	NA	NA	n344976	MTCONS_00024811	chr7	156785276	156789232	430	-	chr15	52599480	52639005	8389	-	-446.59	0.9522	1	2.69	3.81	2.66	0.11	0.23	0.05
tran	NA	NA	n372371	MTCONS_00056157	chr10	72762837	72765495	856	-	chr5	74829990	74901873	11456	+	-500.62	-0.9999	-1	0.17	0.04	0.03	1.21	1.64	1.68
cis_mRNA_dw20k	3659	NA	n342358	MTCONS_00034137	chr19	35439942	35442109	2167	-	chr19	35417806	35436284	2685	+	*	-0.926	-1	2.29	2.22	1.83	1.05	2.16	3.25
cis_mRNA_overlap	0	Lnc-AntiOverlap-mRNA	n340206	MTCONS_00054822	chr4	120548945	120551908	2960	+	chr4	120420772	120549981	7405	-	*	0.8987	1	0.1	0.07	0	0.11	0.04	0.01
tran	NA	NA	n365315	MTCONS_00033841	chr2	203175137	203177250	1187	+	chr19	17325517	17330638	3216	+	-1185.25	-0.9658	-1	0.41	0.21	0.26	2.25	2.56	2.41
tran	NA	NA	n365315	MTCONS_00047425	chr2	203175137	203177250	1187	+	chr22	21061922	21213091	8306	-	-1158.66	-0.9835	-1	0.41	0.21	0.26	1.07	1.66	1.62
cis_mRNA_dw20k	2850	NA	n406833	MTCONS_00050314	chr3	10342615	10362858	1599	-	chr3	10365707	10547366	9194	-	*	0.9275	1	0.64	1.16	0.49	1.38	1.65	1.03
cis_mRNA_overlap	0	Lnc-CompleteIn-mRNAExon	n341917	MTCONS_00015737	chr12	52080207	52139732	1594	+	chr12	51984938	52203485	8422	+	-964.31	-0.9544	-1	0.72	0	0.41	3.01	4.13	3.79

cis_mRNA_overlap	0	mRNA	n379781	MTCONS_00052934	chr4	68567051	68588220	2233	+	chr4	68566991	68603076	6309	+	*		-0.9994	-1	5.24	6.85	7.19	2.82	2.4	2.29
cis_mRNA_up10k	75	NA	n346376	MTCONS_00006940	chr1	202830879	202843851	1726	+	chr1	202819405	202830805	3455	-	*		0.8577	1	1.1	0.76	0.53	4.25	4.14	3.19
tran	NA	NA	n346376	MTCONS_00041776	chr1	202830879	202843851	1726	+	chr2	130824979	130886954	10720	-		-1097.42	0.9334	1	1.1	0.76	0.53	5.02	3.85	0.74
tran	NA	NA	n411020	MTCONS_00040468	chr15	23599895	23613471	5454	+	chr2	24461838	24583769	7406	-		-2299.67	0.9766	0.866	0.24	0.3	0.3	1.89	2.37	2.51
cis_mRNA_overlap	0	AntiCompleteIn-mRNAIntron	n381541	MTCONS_00010568	chr10	99378562	99382516	2157	+	chr10	99374299	99393344	2336	-	*		-0.9994	-1	1.47	1.61	1.99	3.56	3.12	2.1
tran	NA	NA	n380665	MTCONS_00030115	chr22	18927009	18935205	1156	+	chr17	80693452	80703834	5094	+		-1733.53	-0.9837	-1	5.09	5.77	3.78	2.63	2.55	2.71
tran	NA	NA	n380665	MTCONS_00034837	chr22	18927009	18935205	1156	+	chr19	52872319	52925184	17190	+		-1507.5	0.9999	1	5.09	5.77	3.78	2.75	2.91	2.42
tran	NA	NA	LTCONS_00059129	MTCONS_00026455	chr5	79654447	79654906	460	.	chr16	51679680	51681009	1330	+		-587.2	0.6145	1	0.09	0.08	0.38	1.68	1.16	1.75
cis_mRNA_up10k	268	NA	n410108	MTCONS_00011331	chr11	9481103	9482245	1143	-	chr11	9482512	9550540	2952	+	*		-0.9961	-1	2.76	3.1	2.9	3.29	2.39	2.99
cis_mRNA_dw20k	17231	NA	n378187	MTCONS_00046485	chr22	21823036	21824224	788	+	chr22	21771672	21805806	6774	+	*		-0.9853	-1	0.83	1.27	1.11	2.04	0.93	1.5
cis_mRNA_up10k	369	NA	n378068	MTCONS_00002789	chr1	173834956	173837125	1060	-	chr1	173837493	173872659	19182	+	*		-0.9245	-1	6.27	6.18	5.64	3.47	3.75	4.03
cis_mRNA_up10k	1303	NA	n409392	MTCONS_00025322	chr15	85045806	85050249	4444	+	chr15	84941992	85044504	18018	-	*		0.9514	1	1.1	1.09	0.58	10.55	9.49	7.34
cis_mRNA_dw20k	17744	NA	n377897	MTCONS_00006742	chr1	180918835	180924023	1440	-	chr1	180941766	180992135	7627	-	*		-0.9608	-1	0.48	0.64	0.32	1.03	0.94	1.06
tran	NA	NA	n370934	MTCONS_00005235	chr9	47192550	47210048	487	+	chr1	78161672	78225557	4502	-		-238.66	-0.996	-1	0.08	0.6	0.27	7	3	5.86
cis_mRNA_overlap	0	Lnc-AntiOverlap-mRNA	LTCONS_00030525	MTCONS_00028755	chr17	13928951	13972797	8992	-	chr17	13972719	14116062	5746	+	*		0.9021	1	2.12	2.31	1.89	0.36	0.48	0.34
cis_mRNA_overlap	0	Lnc-CompleteIn-mRNAIntron	n341996	MTCONS_00018238	chr12	99597100	99598534	1434	-	chr12	99137752	100378495	6028	-	*		0.9999	1	0.16	0.12	0.21	1.64	1.42	1.9
tran	NA	NA	n366183	MTCONS_00008032	chr3	20387157	20389501	415	+	chr10	28821293	28912041	6050	+		-272.54	0.9879	1	0.09	0.17	0.33	3.62	3.78	4.47
tran	NA	NA	n366183	MTCONS_00047652	chr3	20387157	20389501	415	+	chr22	30771653	30783401	3679	-		-310.74	0.7834	1	0.09	0.17	0.33	4.58	5.95	6.02
cis_mRNA_dw20k	16274	NA	n339251	MTCONS_00026680	chr16	67198716	67203846	1543	+	chr16	67143828	67182443	2800	+	*		-0.9932	-1	0.87	0	0.3	2.07	2.87	2.68
cis_mRNA_dw20k	72	NA	n409777	MTCONS_00009593	chr10	14920782	14946304	2986	+	chr10	14946375	15058981	5512	-	*		0.9395	1	5.64	5.14	4.41	0.92	0.9	0.65
tran	NA	NA	n380069	MTCONS_00042413	chr5	158527491	158542331	691	+	chr2	208470330	208490126	5962	-		-619.67	-0.9963	-0.866	1.15	0.48	1.22	1.77	1.78	1.77
tran	9600967	NA	n386085	MTCONS_00024535	chr15	23194713	23205207	963	-	chr15	32806173	32825942	1395	-		-413.94	0.999	1	0.25	0.07	0.45	0.2	0	0.39
cis_mRNA_dw20k	279	NA	LTCONS_00037271	MTCONS_00037269	chr2	10074954	10076193	1240	+	chr2	9983554	10074676	2447	+	*		0.7322	1	2.62	2.75	3.54	2.14	3.67	4
cis_mRNA_up10k	3235	NA	n382698	MTCONS_00025322	chr15	85047738	85060078	7309	-	chr15	84941992	85044504	18018	-	*		-0.923	-1	1.46	1.9	2.11	10.55	9.49	7.34
cis_mRNA_overlap	0	AntiCompleteIn-mRNAIntron	n345153	MTCONS_00014574	chr11	110087285	110091622	1548	+	chr11	110066283	110167437	2764	-	*		0.8717	1	0.29	0.46	0.22	10.54	10.81	9.75
tran	NA	NA	n385360	MTCONS_00025580	chr8	12426979	12428991	2013	+	chr15	43407850	43408497	648	.		-293.29	0.9912	0.866	0.17	0.17	0.33	0	0.05	0.35
cis_mRNA_overlap	0	Lnc-CompleteIn-mRNAExon	n332617	MTCONS_00046983	chr22	38471400	38471723	324	+	chr22	38453262	38471756	2738	+		-310.81	-0.6048	-1	0.21	2.67	0	4.48	4.37	6.15
tran	NA	NA	n374202	MTCONS_00042389	chr14	90959135	90959933	532	+	chr2	206976735	207024301	14541	-		-595.31	-0.8942	-1	0.21	0.06	0.11	14.77	15.9	15.06
tran	NA	NA	n374202	MTCONS_00047616	chr14	90959135	90959933	532	+	chr22	29655844	29663960	3044	-		-825.1	0.9058	1	0.21	0.06	0.11	11.05	8.81	10.42
tran	NA	NA	n346070	MTCONS_00027110	chr1	664669	674281	640	-	chr16	90229013	90235598	2977	+		-364.48	0.9964	1	0.17	0.63	0.51	0.06	0.95	0.79
cis_mRNA_overlap	0	mRNA	LTCONS_00035889	MTCONS_00035890	chr19	20451086	20607846	1294	-	chr19	20505375	20519981	4696	-	*		0.9468	1	3.18	1.87	0.44	1.65	1.44	0.49
tran	NA	NA	n372755	MTCONS_00007912	chr11	94820008	94821880	942	-	chr10	15139117	15147770	4236	+		-737.16	0.9477	1	0.15	0.4	0.5	0.57	0.6	0.64
tran	NA	NA	n345809	MTCONS_00031911	chr4	119771465	119772685	785	+	chr17	79641591	79650954	7840	-		-601.03	0.9676	1	4.05	4.63	3.3	3.65	3.94	3.5
cis_mRNA_overlap	0	Lnc-CompleteIn-mRNAExon	n339701	MTCONS_00049873	chr3	178953578	178955289	1712	+	chr3	178866116	178957540	8947	+		-646.75	0.8811	1	3.47	2.01	2.57	6.34	5.94	6.27
cis_mRNA_dw20k	13138	NA	n411118	MTCONS_00041196	chr2	85764590	85766009	1420	-	chr2	85779146	85788657	2249	-	*		-0.9996	-1	3.69	3.52	2.75	1.99	2.08	2.42
tran	NA	NA	n325609	MTCONS_00007266	chr14	98694506	98695759	561	+	chr1	225674534	225840945	13893	-		-643.4	1	1	0.91	0.66	1.19	18.07	17.29	18.93
tran	NA	NA	n334599	MTCONS_00039883	chr6	2928327	2929772	424	-	chr2	232063206	232241727	8428	+		-640.44	0.8044	1	2.34	2.05	2.49	3.05	3	3.65
cis_mRNA_overlap	0	Lnc-AntiOverlap-mRNA	n338261	MTCONS_00033329	chr19	1372237	1374363	2127	-	chr19	1354953	1378430	4239	+	*		0.9937	1	0.1	0.15	0.19	4.78	7.75	9.32
tran	NA	NA	n345283	MTCONS_00051716	chr14	100701760	100704777	473	-	chr3	155538816	155572410	9728	-		-485.14	0.8246	1	0	0.43	0.07	2.4	2.77	2.66
cis_mRNA_up10k	9769	NA	n345643	MTCONS_00053031	chr4	77155456	77162957	1051	-	chr4	77172725	77204932	1377	+	*		-0.9822	-1	0.17	0.06	0.09	0.42	1.44	0.99
cis_mRNA_overlap	0	Lnc-CompleteIn-mRNAExon	n340647	MTCONS_00056713	chr5	139496843	139498964	2122	+	chr5	139487296	139505501	12385	+		-850.11	-0.9998	-1	0.17	0	0.4	1.54	1.72	1.28
cis_mRNA_up10k	74	NA	LTCONS_00025661	MTCONS_00025663	chr16	691840	699262	3125	+	chr16	699335	717829	8992	+	*		1	1	0.63	0.75	0.71	0.75	1.31	1.12
tran	NA	NA	n371628	MTCONS_00052157	chrY	14774468	14800184	882	+	chr3	197299019	197354752	9196	-		-484.04	-0.8136	-1	4.37	0	4.35	1.79	2.76	2.36
cis_mRNA_overlap	0	Lnc-CompleteIn-mRNAIntron	n342481	MTCONS_00027788	chr16	49551557	49552496	929	-	chr16	49523602	49890016	5794	-	*		0.8885	0.866	0	0.03	0.03	0.53	0.73	0.9
cis_mRNA_up10k	829	NA	n375723	MTCONS_00029468	chr17	46124254	46124834	343	-	chr17	46125662	46142854	8755	+	*		0.9449	1	0.92	1.05	0.41	4.5	5.37	3.64
tran	1666869	NA	LTCONS_00027481	MTCONS_00025969	chr16	16485934	16487821	1762	-	chr16	14813685	14819066	639	+		-577.61	-0.9995	-1	0.16	0.21	0.45	2.26	1.95	0.07
tran	57937321	NA	LTCONS_00027481	MTCONS_00028418	chr16	16485934	16487821	1762	-	chr16	74425141	74425984	844	.		-886.93	0.9999	1	0.16	0.21	0.45	0.44	0.76	2.45
cis_mRNA_up10k	9568	NA	n410054	MTCONS_00006433	chr1	160246599	160254941	3559	-	chr1	160236583	160237032	450	-	*		0.9736	1	0.44	0.87	1.26	0	0.1	0.31
tran	NA	NA	n371418	MTCONS_00033841	chrX	390760	398431	771	+	chr19	17325517	17330638	3216	+		-501.32	-0.9134	-1	0.23	0.08	0.21	2.25	2.56	2.41
tran	NA	NA	n371418	MTCONS_00042413	chrX	390760	398431	771	+	chr2	208470330	208490126	5962	-		-719.08	-0.9924	-0.866	0.23	0.08	0.21	1.77	1.78	1.77
tran	NA	NA	n371418	MTCONS_00015928	chrX	390760	398431	771	+	chr12	56914542	56989980	8281	+		-767.88	-0.7359	-1	0.23	0.08	0.21	0.72	1.14	1.06
cis_mRNA_dw20k	2325	NA	n379265	MTCONS_00028789	chr17	16342641	16345093	863	+	chr17	16318851	16340317	2810	+	*		-0.739	-1	6.72	9.35	6.06	0.4	0	3.14
cis_mRNA_overlap	0	Lnc-CompleteIn-mRNAIntron	n338025	MTCONS_00005268	chr1	84444801	84449983	2921	-	chr1	84330698	84464833	10521	-	*		0.9697	1	0.36	0.39	0.5	7.04	8.16	9.6
tran	NA	NA	n409638	MTCONS_00040816	chr7	7115401	7136417	21017	-	chr2	53889562	54014214	9905	-		-9166.28	0.9947	1	0.68	0.7	0.65	5.36	6.05	4.64
cis_mRNA_overlap	0	Lnc-AntiOverlap-mRNA	n379619	MTCONS_00012887	chr11	121318040	121323722	2908	-	chr11	121322912	121504517	11019	+	*		0.9822	0.866	0.05	0.1	0.05	6.26	9.16	5.54
tran	NA	NA	n365203	MTCONS_00015928	chr2	100107781	100125484	473	+	chr12	56914542	56989980	8281	+		-310.45	0.9527	1	2.17	3.34	2.79	0.72	1.14	1.06
cis_mRNA_overlap	0	Lnc-CompleteIn-mRNAIntron	n383634	MTCONS_00040816	chr2	53990098	53992017	1920	-	chr2	53889562	54014214	9905	-	*		0.9512	1	0.32	0.42	0.29	5.36	6.05	4.64
cis_mRNA_overlap	0	mRNA	n382486	MTCONS_00024535	chr15	32814894	32825942	1673	-	chr15	32806173	32825942	1395	-		-550.04	0.932	1	1.15	1.07	1.52	0.2	0	0.39
tran	NA	NA	n326362	MTCONS_00041449	chr1	156368458	156377053	834	-	chr2	101887052	101925178	6597	-		-570.68	-0.9548	-1	0.34	0.13	0.26	1.64	2.09	1.93
tran	NA	NA	LTCONS_00054817	MTCONS_00051716	chr4	120355920	120362086	583	-	chr3	155538816	155572410	9728	-		-573.69	-0.9666	-1	1.29	0.52	0.55	2.4	2.77	2.66
tran	NA	NA	LTCONS_00054817	MTCONS_00015928	chr4	120355920	120362086	583	-	chr12	56914542	56989980	8281	+		-339.12	-0.9894	-1	1.29	0.52	0.55	0.72	1.14	1.06

cis_mRNA_dw20k	17736	NA	n407731	MTCONS_00018284	chr12	104237527	104323989	4516	-	chr12	104341724	104351050	4327	-	*		0.8386	0.866	0.12	0.06	0.17	0.88	0.88	0.92
tran	NA	NA	n379064	MTCONS_00009726	chr16	30546394	30548465	1124	+	chr10	28808846	28821468	4854	-		-1487.92	0.9859	1	1.88	1.74	2.28	5.2	5.04	6.76
tran	NA	NA	n342526	MTCONS_00039494	chr21	19933583	20132130	662	-	chr2	201980877	202038063	11977	+		-702.17	-0.9494	-1	0.05	0	0.04	0.42	0.83	0.62
tran	28833274	NA	n380046	MTCONS_00057778	chr5	14651755	14653492	1676	+	chr5	43486765	43496143	2426	-		-2417.5	-0.9273	-1	0.45	0.32	0.37	0.05	0.23	0.1
tran	NA	NA	LTCONS_00054666	MTCONS_00039148	chr4	103340075	103352403	256	-	chr2	168148819	168730621	6608	+		-201.62	-0.9717	-1	1.25	0.75	0.55	10.67	15.23	15.46
cis_mRNA_up10k	3919	NA	n338180	MTCONS_00001981	chr1	114399257	114443859	1733	+	chr1	114447777	114456708	3760	+	*		0.9167	1	0.63	0.9	0.95	0.38	0.79	1.25
cis_mRNA_overlap	0	Lnc-AntiOverlap-mRNA	n377528	MTCONS_00047421	chr22	20850204	20853121	939	+	chr22	20795806	20850234	2648	-	*		-0.9426	-1	0.15	0.6	0.35	2.86	0.85	2.6
cis_mRNA_overlap	0	AntiCompleteIn-mRNAIntron	n378490	MTCONS_00043708	chr20	52014199	52017772	2929	-	chr20	51588893	52111946	12190	+	*		-0.9492	-0.866	0.21	0.13	0.21	0.27	0.84	0
cis_mRNA_up10k	1254	NA	LTCONS_00011149	MTCONS_00013163	chr11	1594477	1623308	1236	+	chr11	1575281	1593224	4547	-	*		0.9396	1	0	0.96	0.17	7.28	9.61	8.43
cis_mRNA_up10k	4410	NA	n338479	MTCONS_00035939	chr19	23437860	23457032	2357	-	chr19	23400420	23433451	7034	-	*		0.9333	1	1.25	1.19	4.23	1.84	1.59	2.25
cis_mRNA_up10k	367	NA	n378366	MTCONS_00002789	chr1	173833039	173837127	1698	-	chr1	173837493	173872659	19182	+	*		0.9999	1	6.65	7.5	8.31	3.47	3.75	4.03
cis_mRNA_up10k	553	NA	n378267	MTCONS_00001986	chr1	114466623	114471854	1321	-	chr1	114472406	114520493	8150	+	*		-0.8946	-1	0.15	0.09	0.16	7.87	8.01	7.69
tran	NA	NA	n405504	MTCONS_00007544	chr4	419224	467998	1895	-	chr1	247354222	247374158	484	-		-300.06	0.9995	1	3.49	3.68	3.91	2.32	3.03	3.8
cis_mRNA_up10k	115	NA	LTCONS_00059110	MTCONS_00055634	chr5	14142134	14143715	1582	.	chr5	14143829	14510312	10551	+	*		-0.9916	-1	0.63	0.52	0.76	19.82	20.84	17.88
cis_mRNA_overlap	0	Lnc-CompleteIn-mRNAIntron	n345424	MTCONS_00003463	chr1	224623283	224647271	4355	+	chr1	224622592	224928257	1899	+	*		-0.751	-1	0.19	0.39	0.33	1.29	0.69	1.27
tran	NA	NA	n366365	MTCONS_00028547	chr3	184433380	184477461	637	+	chr17	4901243	4931775	8021	+		-749.75	0.6341	1	0.64	0.72	0.71	3.02	4.68	3.11
tran	NA	NA	n366365	MTCONS_00056187	chr3	184433380	184477461	637	+	chr5	77656339	77776580	6161	+		-691.04	-0.9618	-1	0.64	0.72	0.71	1.62	0.92	1.19
cis_mRNA_overlap	0	mRNA	n409406	MTCONS_00009726	chr10	28808846	28821283	5376	-	chr10	28808846	28821468	4854	-		-2557.88	-0.7284	-1	0.58	0.94	0.48	5.2	5.04	6.76
tran	NA	NA	n368195	MTCONS_00030957	chr5	172700826	172708051	382	+	chr17	37408059	37559422	11920	-		-362.3	0.7472	0.866	0.72	0.91	1	0.62	0.62	0.64
cis_mRNA_dw20k	8330	NA	n387059	MTCONS_00033319	chr19	1267471	1270259	1355	-	chr19	1250692	1259142	3911	+	*		0.9585	1	0.05	0.02	0	1.91	1.44	1.37
cis_mRNA_dw20k	1001	NA	n346082	MTCONS_00036975	chr19	56879648	56890224	2597	+	chr19	56891224	56904973	6027	-	*		-0.7597	-1	2.95	2.77	1.99	1.17	1.41	1.46
cis_mRNA_overlap	0	mRNA	n346082	MTCONS_00035019	chr19	56879648	56890224	2597	+	chr19	56879504	56894634	6776	+		-1325.41	-0.9912	-1	2.95	2.77	1.99	1.61	1.63	2.01
cis_mRNA_overlap	0	Lnc-AntiOverlap-mRNA	LTCONS_00029039	MTCONS_00030754	chr17	29035714	29119437	3516	+	chr17	29115838	29151778	8052	-		-4512.74	-0.932	-1	2.01	1.93	1.43	0.35	0.46	0.58
cis_mRNA_up10k	356	NA	n374513	MTCONS_00024135	chr15	83626882	83654620	1261	-	chr15	83654975	83660687	2430	+	*		0.798	1	0.14	0.15	0.3	18.03	19.58	20.39
cis_mRNA_up10k	4619	NA	n326680	MTCONS_00000547	chr1	21785552	21790267	821	+	chr1	21794885	21811393	3477	+	*		0.9982	1	0.09	0.04	0.28	0.9	0.55	1.87
cis_mRNA_overlap	0	Lnc-CompleteIn-mRNAIntron	n342571	MTCONS_00032816	chr18	18635361	18637153	1793	-	chr18	18623614	18691812	3170	-	*		0.9449	0.866	0.23	0.19	0.19	1.26	0.9	1.02
cis_mRNA_overlap	0	Lnc-CompleteIn-mRNAExon	n338440	MTCONS_00006866	chr1	197473881	197476148	2263	-	chr1	197473872	197744760	8461	-		-884.04	-1	-1	1.36	1.55	1.63	4.49	4.25	4.15
cis_mRNA_overlap	0	Lnc-CompleteIn-mRNAExon	n335660	MTCONS_00047449	chr22	21982379	21982768	390	-	chr22	21982370	21985086	2108	-		-326.11	0.8933	1	3.33	1.18	2.52	2.14	1.42	1.57
tran	41434219	NA	n406993	MTCONS_00034660	chr19	7537723	7538247	525	-	chr19	48972465	48985574	9032	+		-478.14	-0.6583	-1	0.21	0.19	0.06	3.37	5.1	5.25
cis_mRNA_overlap	0	AntiCompleteIn-mRNAIntron	n379472	MTCONS_00051872	chr3	176908453	176912093	2170	+	chr3	176737169	176914870	8526	-	*		0.9962	1	0.05	0.02	0	7.08	6.42	5.82
cis_mRNA_overlap	0	mRNA	LTCONS_00055701	MTCONS_00055699	chr5	23951457	24206653	6859	+	chr5	23951457	23981154	1761	+		-721	1	0.866	2.21	0.08	0.09	1.83	0.06	0.06
cis_mRNA_dw20k	19953	NA	n375570	MTCONS_00028960	chr17	25664177	25666026	1075	+	chr17	25621074	25644225	7800	+	*		-0.8537	-1	0.39	0.27	0.23	32.65	34.47	39.56
tran	NA	NA	n370172	MTCONS_00056157	chr8	67104346	67109547	1499	+	chr5	74829990	74901873	11456	+		-725	-0.8039	-1	0.09	0.08	0.06	1.21	1.64	1.68
cis_mRNA_up10k	3074	NA	n377525	MTCONS_00047304	chr22	18260361	18261241	325	+	chr22	18216813	18257288	2040	-	*		-0.9922	-0.866	0	0.11	0.11	2.23	1.14	0.97
cis_mRNA_overlap	0	Lnc-AntiOverlap-mRNA	n410901	MTCONS_00056966	chr5	149372686	149380171	3988	-	chr5	149380169	149432839	5539	+	*		-0.9979	-1	0.49	0.45	0.29	1.8	2.61	7.75
cis_mRNA_up10k	1866	NA	LTCONS_00033210	MTCONS_00032643	chr18	74516930	74534251	4811	-	chr18	74536116	74607633	2274	+	*		-0.9978	-1	0.72	1.08	1.15	2.32	1.71	1.53
cis_mRNA_up10k	322	NA	LTCONS_00055404	MTCONS_00054925	chr4	140098656	140099104	449	.	chr4	139978779	140098335	3570	-	*		-0.9935	-1	0	0.08	0.23	10.17	9.18	7.96
tran	582303	NA	LTCONS_00056073	MTCONS_00057977	chr5	69734407	69746189	5552	+	chr5	70328491	70585523	15856	-		-3131.51	-0.7427	-1	2.13	1.93	2.64	3.97	4.43	3.95
tran	NA	NA	n324855	MTCONS_00039148	chr21	25180861	25196941	540	-	chr2	168148819	168730621	6608	+		-631.72	0.716	1	0	0.06	0.25	10.67	15.23	15.46
tran	NA	NA	n324855	MTCONS_00043639	chr21	25180861	25196941	540	-	chr20	47835832	47860620	3762	+		-666.66	0.9018	1	0	0.06	0.25	4.33	6.3	7.43
cis_mRNA_dw20k	9628	NA	n366667	MTCONS_00049981	chr3	184096018	184097565	1434	+	chr3	184079472	184086391	1845	+	*		-0.9598	-1	0.07	0.02	0	3.62	4.32	5.13
cis_mRNA_overlap	0	mRNA	n342695	MTCONS_00056688	chr5	138609820	138643213	630	+	chr5	138609328	138667366	6070	+		-368.93	0.9631	1	4.27	2.68	1.51	0.88	0.5	0.42
cis_mRNA_up10k	8587	NA	n366563	MTCONS_00052157	chr3	197363338	197374168	9659	-	chr3	197299019	197354752	9196	-	*		-0.9982	-1	0.61	0.33	0.46	1.79	2.76	2.36
tran	NA	NA	n325428	MTCONS_00031887	chr2	12371894	12618842	578	+	chr17	79217277	79269170	5912	-		-577.72	-0.9359	-0.866	0.06	0	0.06	2.21	2.49	2.31
cis_mRNA_overlap	0	Lnc-CompleteIn-mRNAExon	n337730	MTCONS_00024692	chr15	43650376	43652236	1860	-	chr15	43650298	43663198	5882	-		-933.61	-0.8968	-1	0.67	0.49	0.6	6.1	6.84	6.69
cis_mRNA_overlap	0	mRNA	LTCONS_00033278	MTCONS_00033277	chr19	680761	683419	2117	+	chr19	676593	683419	5895	+		-1928.6	-0.8611	-1	3.89	2.82	4.07	0.35	0.44	0.2
cis_mRNA_dw20k	1985	NA	n411736	MTCONS_00028789	chr17	16342301	16345340	1065	+	chr17	16318851	16340317	2810	+	*		-0.9656	-1	0.25	0.4	0	0.4	0	3.14
cis_mRNA_dw20k	1846	NA	n379594	MTCONS_00012152	chr11	66037303	66045996	2792	-	chr11	66023425	66035458	4829	+	*		0.9925	1	0.88	1.16	1.22	0.22	0.61	0.63
cis_mRNA_dw20k	10176	NA	n408118	MTCONS_00042754	chr2	242026509	242041747	2573	-	chr2	242051922	242089877	6960	-	*		-0.8753	-1	0.41	0	1.88	1.22	1.54	1.06
cis_mRNA_overlap	0	Lnc-CompleteIn-mRNAExon	n408118	MTCONS_00042748	chr2	242026509	242041747	2573	-	chr2	242026347	242041785	5463	-		-1602.02	-0.9691	-1	0.41	0	1.88	4.7	6.26	2.74
cis_mRNA_overlap	0	Lnc-CompleteIn-mRNAExon	n382021	MTCONS_00019166	chr13	42813346	42817026	3681	+	chr13	42614826	42817094	17567	+		-1520.54	0.7444	1	1.76	0.85	2.05	2.73	2.69	3.13
cis_mRNA_dw20k	19862	NA	n338259	MTCONS_00033273	chr19	637105	637537	433	-	chr19	612829	617244	4053	+	*		-0.913	-0.866	0.09	0.08	0.08	0.62	1.01	0.85
cis_mRNA_overlap	0	Lnc-AntiOverlap-mRNA	n377743	MTCONS_00045348	chr21	37502670	37648524	1304	-	chr21	37529070	37666829	8055	+	*		-0.8201	-1	0.13	0.07	0.05	1.92	2.16	3.09
cis_mRNA_overlap	0	mRNA	n367170	MTCONS_00052857	chr4	56251873	56253958	1487	+	chr4	56212388	56254858	7355	+		-775.34	-0.9097	-1	0.01	0.06	0	0.5	0.33	0.71
tran	NA	NA	n375602	MTCONS_00034476	chr17	40346647	40348097	415	+	chr19	44455373	44485875	16403	+		-549.06	-0.9907	-1	1.18	3.06	1.78	3.03	2.78	2.92
tran	NA	NA	n379686	MTCONS_00027624	chr5	43578439	43603039	503	-	chr16	28385134	28415186	8839	-		-664.37	-0.9527	-1	0.32	0.24	0.36	3.95	4.2	3.57
cis_mRNA_up10k	6286	NA	n364428	MTCONS_00001046	chr1	44404330	44406129	364	-	chr1	44412414	44433873	4217	+	*		0.9706	1	1.18	0.59	0.77	2.5	1.44	1.51
cis_mRNA_overlap	0	Lnc-AntiOverlap-mRNA	LTCONS_00058409	MTCONS_00056566	chr5	127396147	127419483	1348	-	chr5	127419322	127525380	6973	+	*		0.7748	1	0.9	0.83	0.43	9.74	8.85	8.58
tran	NA	NA	n324418	MTCONS_00046106	chr6	113784898	113795839	507	-	chr21	43369737	43374053	4099	-		-245.35	0.7444	1	0.28	0.27	0.2	1.66	1.21	1.09

tran	NA	NA	n364366	MTCONS_00041200	chr1	95148857	95153875	747	+	chr2	85829929	85839179	8793	-		-586.03	-0.7592	-1	0.61	0.57	0.32	3.03	3.42	3.53
cis_mRNA_overlap	0	Lnc-AntiOverlap-mRNA	n379126	MTCONS_00051372	chr3	123304403	123348669	568	+	chr3	123331143	123554784	7750	-	*		0.8483	1	0.15	0.1	0.08	0.46	0.41	0.23
cis_mRNA_overlap	0	Lnc-CompleteIn-mRNAExon	n335717	MTCONS_00021744	chr14	100610216	100610573	358	+	chr14	100485716	100610632	2112	+		-238.71	-0.7958	-1	5.26	3.8	8.29	21.28	34.22	20.15
tran	NA	NA	n383885	MTCONS_00050496	chr20	25990435	26002424	1548	+	chr3	37092469	37218318	5068	-		-1278.98	-0.6934	-0.866	0.24	0.26	0.24	2.13	2.1	2.22
tran	NA	NA	n383885	MTCONS_00015928	chr20	25990435	26002424	1548	+	chr12	56914542	56989980	8281	+		-1554.22	0.6472	0.866	0.24	0.26	0.24	0.72	1.14	1.06
tran	NA	NA	n366884	MTCONS_00035888	chr4	121560245	121571943	544	+	chr19	20334788	20349276	4685	-		-256.01	0.9036	1	0.08	0.31	0.06	5.26	5.92	4.62
tran	NA	NA	n364876	MTCONS_00027624	chr2	15808808	15820608	568	-	chr16	28385134	28415186	8839	-		-674.75	0.8781	1	0.04	0.15	0.02	3.95	4.2	3.57
cis_mRNA_dw20k	169	NA	n339579	MTCONS_00044168	chr20	10382257	10384178	1922	-	chr20	10384346	10414872	4027	-	*		-0.8862	-1	0.43	0.31	0.35	0.36	0.61	0.42
cis_mRNA_dw20k	11816	NA	n376129	MTCONS_00032583	chr18	68016206	68019682	613	+	chr18	67956126	68004391	7654	+	*		0.8963	0.866	0	0.1	0.18	0.25	0.69	0.69
cis_mRNA_dw20k	7423	NA	n374525	MTCONS_00024133	chr15	83511246	83514392	2618	+	chr15	83490056	83503824	3232	+	*		-0.9966	-0.866	0.04	0.01	0.01	0.18	0.38	0.4
tran	NA	NA	n367896	MTCONS_00045141	chr5	54317127	54319997	550	-	chr21	22370623	22863909	9470	+		-519.46	-0.9499	-1	2.35	4.43	4.07	16.17	8.24	11.91
tran	NA	NA	n367896	MTCONS_00041449	chr5	54317127	54319997	550	-	chr2	101887052	101925178	6597	-		-514.94	0.9809	1	2.35	4.43	4.07	1.64	2.09	1.93
cis_mRNA_up10k	451	NA	n379862	MTCONS_00057560	chr5	16180347	16185694	2066	+	chr5	16067278	16179897	1531	-	*		0.9969	1	0.08	0.17	0.58	0.66	0.99	1.95
cis_mRNA_dw20k	70	NA	n410210	MTCONS_00050279	chr3	9851644	9878040	4160	+	chr3	9878109	9885702	4133	-	*		0.9799	0.866	0.13	0.13	0.14	3.88	4	4.45
tran	2143670	NA	n382664	MTCONS_00025322	chr15	82764079	82798323	3634	-	chr15	84941992	85044504	18018	-		-6167.25	0.807	1	5.36	4.11	3.98	10.55	9.49	7.34
tran	NA	NA	LTCONS_00042936	MTCONS_00033841	chr20	3388848	3390736	1889	+	chr19	17325517	17330638	3216	+		-1472.52	0.8804	1	1	1.37	1.02	2.25	2.56	2.41
tran	NA	NA	LTCONS_00042936	MTCONS_00051716	chr20	3388848	3390736	1889	+	chr3	155538816	155572410	9728	-		-1343.6	0.7613	1	1	1.37	1.02	2.4	2.77	2.66
tran	NA	NA	LTCONS_00042936	MTCONS_00017810	chr20	3388848	3390736	1889	+	chr12	56735365	56754037	4979	-		-1081.9	0.8425	1	1	1.37	1.02	4.98	8.37	6.95
cis_mRNA_overlap	0	mRNA	LTCONS_00015414	MTCONS_00015413	chr12	21654696	21671598	6994	+	chr12	21654658	21671598	3607	+		-1373.57	0.999	1	4.44	5.12	4.78	3.38	4.01	3.72
cis_mRNA_up10k	94	NA	LTCONS_00015414	MTCONS_00017271	chr12	21654696	21671598	6994	+	chr12	21621749	21654603	3784	-	*		0.8738	1	4.44	5.12	4.78	0.25	1.36	1.34
tran	NA	NA	n385792	MTCONS_00035582	chrX	100141957	100143628	1672	-	chr19	11776297	11778218	1922	-		-769.81	-0.8462	-0.866	0.09	0.04	0.04	0.15	0.22	0.3
tran	NA	NA	n376368	MTCONS_00042702	chr19	23452692	23457021	541	-	chr2	239152679	239197362	6202	-		-362.11	0.9501	1	0	0.23	0.15	1.58	1.98	1.73
tran	NA	NA	n376368	MTCONS_00015928	chr19	23452692	23457021	541	-	chr12	56914542	56989980	8281	+		-350.69	0.9857	1	0	0.23	0.15	0.72	1.14	1.06
cis_mRNA_overlap	0	Lnc-AntiOverlap-mRNA	n380034	MTCONS_00023976	chr15	74418714	74421619	2566	-	chr15	74420550	74429213	4335	+	*		-0.989	-1	2.83	2.5	3.33	5.02	6.2	1.73
tran	NA	NA	n379661	MTCONS_00005711	chr17	48840212	48844918	940	-	chr1	115106620	115124265	4862	-		-664.67	0.8858	1	0.19	0.25	0.42	1.97	2.19	2.29
cis_mRNA_dw20k	3394	NA	LTCONS_00012826	MTCONS_00012823	chr11	118877069	118888762	9015	+	chr11	118868826	118873676	4322	+	*		0.9881	1	3.04	3.59	3.76	3.03	3.94	4.49
cis_mRNA_overlap	0	Lnc-AntiOverlap-mRNA	LTCONS_00012826	MTCONS_00014729	chr11	118877069	118888762	9015	+	chr11	118886370	118889169	656	-		-600.81	-0.9175	-1	3.04	3.59	3.76	191.66	176.54	154
tran	NA	NA	n365438	MTCONS_00057969	chr2	45401699	45402931	706	-	chr5	69770779	69881984	8211	-		-601.1	-0.9851	-1	0.05	0	0.1	3.85	4.56	3.47
tran	243079047	NA	n381375	MTCONS_00003844	chr1	243219612	243265046	3838	-	chr1	131337	140566	5257	-		-2312.4	0.9951	0.866	0.59	0.7	0.59	0.2	0.57	0.24
cis_mRNA_up10k	2512	NA	n376476	MTCONS_00033319	chr19	1246146	1248181	928	-	chr19	1250692	1259142	3911	+	*		-0.9444	-1	0.37	0.51	0.62	1.91	1.44	1.37
tran	16645463	NA	n405702	MTCONS_00025950	chr16	29313608	29376380	1585	+	chr16	12070585	12668146	8181	+		-1056.04	-0.9936	-1	0.04	0.02	0	3.03	3.73	4.2
tran	NA	NA	n381237	MTCONS_00035067	chr1	145004781	145005286	506	+	chr19	57922314	57936438	6065	+		-458.16	-0.9768	-1	7.66	4.03	6.05	1.57	1.89	1.65
tran	NA	NA	n381237	MTCONS_00023705	chr1	145004781	145005286	506	+	chr15	52121825	52226551	12441	+		-504.71	0.8967	0.866	7.66	4.03	6.05	1.79	1.75	1.79
cis_mRNA_overlap	0	Lnc-CompleteIn-mRNAIntron	n326317	MTCONS_00002829	chr1	178794735	178802277	1489	+	chr1	178694300	178892717	9235	+	*		0.8178	0.866	0.14	0.15	0.15	3.62	4.19	4.97
cis_mRNA_up10k	3451	NA	n378727	MTCONS_00002789	chr1	173833092	173834043	424	-	chr1	173837493	173872659	19182	+	*		-0.9904	-1	1.54	0.82	0.38	3.47	3.75	4.03
tran	NA	NA	LTCONS_00070636	MTCONS_00026455	chr8	115041783	115042384	602	.	chr16	51679680	51681009	1330	+		-456.1	-0.7675	-1	0.2	0.22	0.14	1.68	1.16	1.75
tran	3725859	NA	n363948	MTCONS_00002104	chr1	148556480	148558168	336	+	chr1	144593657	144830622	7756	+		-279.1	0.9203	1	2.52	3.81	2.7	7.98	8.75	8.37
cis_mRNA_dw20k	5394	NA	n345830	MTCONS_00055289	chr4	185299501	185303483	902	-	chr4	185308876	185395726	2121	-	*		0.9917	1	0.08	0.04	0	0.68	0.38	0.19
cis_mRNA_up10k	657	NA	n379586	MTCONS_00056566	chr5	127301580	127418666	1601	-	chr5	127419322	127525380	6973	+	*		0.7438	1	0.65	0.64	0.55	9.74	8.85	8.58
tran	NA	NA	n365012	MTCONS_00005969	chr2	177494893	177502290	838	-	chr1	149394058	149400543	5522	-		-957.06	-0.7403	-1	1.02	0.76	0.72	0.37	0.62	1.64
tran	NA	NA	LTCONS_00025610	MTCONS_00015928	chr15	93363768	93364554	787	.	chr12	56914542	56989980	8281	+		-485.15	0.6472	0.866	1.05	1.45	1.05	0.72	1.14	1.06
cis_mRNA_dw20k	9831	NA	n342278	MTCONS_00047830	chr22	38914954	38964295	2128	-	chr22	38974125	39052634	10291	-	*		0.9149	1	0.43	0.28	0.46	0.95	0.84	1.08
cis_mRNA_dw20k	1985	NA	n411739	MTCONS_00028789	chr17	16342301	16373962	547	+	chr17	16318851	16340317	2810	+	*		-0.878	-1	0.13	0.31	0	0.4	0	3.14
tran	NA	NA	n324408	MTCONS_00040816	chr6	114951308	114963732	402	-	chr2	53889562	54014214	9905	-		-391	0.868	1	2.42	3.48	2.4	5.36	6.05	4.64
cis_mRNA_overlap	0	mRNA	LTCONS_00051824	MTCONS_00051825	chr3	169805368	169817286	10407	-	chr3	169805368	169899537	15563	-		-4173.61	-0.6156	-0.866	0.12	0.17	0.17	1.16	0.96	1.13
cis_mRNA_up10k	1445	NA	n380562	MTCONS_00047665	chr22	31366663	31372049	2744	+	chr22	31318633	31365219	8600	-	*		-0.9968	-1	1.6	1.01	0.47	4.38	4.67	5.02
cis_mRNA_overlap	0	Lnc-AntiOverlap-mRNA	n339356	MTCONS_00050751	chr3	49760258	49762050	1793	+	chr3	49758837	49823990	6484	-		-3131.61	0.9973	1	0.1	0.07	0.15	2.61	1.84	3.59
cis_mRNA_dw20k	7942	NA	n383550	MTCONS_00034844	chr19	53099937	53103404	2483	+	chr19	53072884	53091996	3121	+	*		-0.9696	-1	0.51	0.75	0.65	1.3	1.09	1.13
tran	NA	NA	n405889	MTCONS_00037620	chr6	3493960	3496295	1064	+	chr6	32288518	32383018	5380	+		-821.89	0.8833	1	0.03	0.05	0.04	2.96	3.95	3.91
cis_mRNA_dw20k	4577	NA	n345476	MTCONS_00006742	chr1	180931250	180937190	990	-	chr1	180941766	180992135	7627	-	*		-0.891	-1	0.14	0.16	0.1	1.03	0.94	1.06
cis_mRNA_dw20k	10914	NA	n340017	MTCONS_00008183	chr10	44124265	44170147	1928	+	chr10	44103525	44113352	2374	+	*		0.8051	1	0	0.1	0.02	0.28	0.54	0.48
cis_mRNA_dw20k	123	NA	LTCONS_00023124	MTCONS_00021554	chr14	86096672	86097268	597	.	chr14	85999632	86096550	9774	+	*		0.9718	1	0.55	1.4	0.22	0.87	1.4	0.36
cis_mRNA_dw20k	3273	NA	n342394	MTCONS_00036450	chr19	44971329	44972014	683	+	chr19	44975286	45004575	7623	-	*		-0.996	-1	0.16	0.1	0.38	1.25	1.27	1.11
cis_mRNA_dw20k	1985	NA	n407689	MTCONS_00028789	chr17	16342301	16345340	890	+	chr17	16318851	16340317	2810	+	*		-0.8833	-1	67.56	82.64	56.22	0.4	0	3.14
tran	NA	NA	LTCONS_00018885	MTCONS_00008828	chr12	94033687	94035392	1706	.	chr10	99185873	99193194	1870	+		-3728	0.8561	1	0.19	0.34	0.24	149.34	167.42	164.15
tran	105963247	NA	n378518	MTCONS_00039172	chr2	64455567	64475672	547	+	chr2	170438918	170499115	9908	+		-524.26	-0.8977	-0.866	1.91	1.57	2.34	2.11	2.11	1.81
cis_mRNA_overlap	0	Lnc-AntiOverlap-mRNA	n384212	MTCONS_00048194	chr3	9431591	9439174	1987	-	chr3	9404225	9435671	11861	+		-3654.5	-0.9856	-1	6.16	12.68	5.26	0.35	0.3	0.37
cis_mRNA_up10k	1157	NA	n374292	MTCONS_00021218	chr14	56027865	56045769	463	-	chr14	56046925	56151413	4760	+	*		0.917	0.866	0.08	0.08	0.15	19.13	20.29	22.02
tran	40586	NA	n381076	MTCONS_00000547	chr1	21749601	21754300	2057	-	chr1	21794885	21811393	3477	+		-1055.46	-0.9229	-1	1.02	1.29	0.85	0.9	0.55	1.87
tran	NA	NA	n380263	MTCONS_00003844	chr11	129531	139099	2301	-	chr1	131337	140566	5257	-		-1701.26	-0.713	-1	0.88	0.66	0.7	0.2	0.57	0.24
tran	NA	NA	n380263	MTCONS_00027110	chr11	129531	139099	2301	-	chr16	90229013	90235598	2977	+		-1337.08	-1	-1	0.88	0.66	0.7	0.06	0.95	0.79
cis_mRNA_dw20k	6588	NA	n407468	MTCONS_00042698	chr2	239133754	239140318	846	-	chr2	239146905	239148681	1608	-	*		0.9897	1	0.63	0.47	0.44	18.12	13.21	13.19
cis_mRNA_up10k	181	NA	n374300	MTCONS_00021317	chr14	65170511	65170923	286	-	chr14	65171103	65212995	6956	+	*		-0.6007	-0.866	0	0.13	0.13	2.43	1.59	2.32
tran	NA	NA	n367819	MTCONS_00039148	chr5	135802366	135855987	427	+	chr2	168148819	168730621	6608	+		-287.93	-0.9955	-1	1.12	0.43	0.31	10.67	15.23	15.46
cis_mRNA_dw20k	2071	NA	n332918	MTCONS_00000607	chr1	24865207	24865735	529	+	chr1	24829355	24863137	2408	+	*		0.9697	1	22.17	21.97	25.37	20.42	18.77	24.23

cis_mRNA_overlap	0	Lnc-CompleteIn-mRNAExon	n406658	MTCONS_00040640	chr2	38522029	38604432	2644	-	chr2	38520753	38604432	4657	-		-1220.07	-0.9317	-1	0.18	0.39	0.6	8.07	7.76	6.16
tran	NA	NA	LTCONS_00014307	MTCONS_00047652	chr11	76152071	76155713	1005	-	chr22	30771653	30783401	3679	-		-903.22	-0.7863	-1	4.16	3.89	3.36	4.58	5.95	6.02
tran	NA	NA	n324056	MTCONS_00054182	chr9	84258080	84259960	599	+	chr4	24805140	24914602	6595	-		-620.55	-0.762	-1	0.21	0.3	0.23	3.04	2.54	2.61
tran	NA	NA	n324056	MTCONS_00043451	chr9	84258080	84259960	599	+	chr20	39969434	39989223	5301	+		-666.13	0.9979	1	0.21	0.3	0.23	0.94	2.81	1.47
tran	NA	NA	n343058	MTCONS_00039148	chr5	76008230	76008661	432	+	chr2	168148819	168730621	6608	+		-629.32	-0.9968	-1	0.76	0.37	0.31	10.67	15.23	15.46
cis_mRNA_up10k	3437	NA	LTCONS_00035978	MTCONS_00035963	chr19	23581791	23598903	3263	-	chr19	23486602	23578355	10046	-	*		-0.7713	-0.866	0	0.08	0.08	5.86	5.36	4.45
tran	NA	NA	n338641	MTCONS_00042413	chr13	33084429	33085024	596	-	chr2	208470330	208490126	5962	-		-596.6	0.7759	0.866	1.05	1.18	0.82	1.77	1.78	1.77
tran	NA	NA	n338641	MTCONS_00013720	chr13	33084429	33085024	596	-	chr11	46618045	46638777	8333	-		-607.24	-0.9498	-1	1.05	1.18	0.82	2.7	2.47	2.83
cis_mRNA_overlap	0	mRNA	n338855	MTCONS_00037937	chr2	61372266	61390298	565	+	chr2	61372243	61391964	982	+		-304.75	-0.9866	-1	0.66	0.17	0.31	0.96	3.9	2.63
tran	NA	NA	n363832	MTCONS_00015928	chr1	36916215	36917200	640	+	chr12	56914542	56989980	8281	+		-410.35	0.8846	1	9.26	12.05	10.31	0.72	1.14	1.06
tran	57308	NA	n345742	MTCONS_00035890	chr19	20577288	20607787	2592	-	chr19	20505375	20519981	4696	-		-1169.44	0.9989	1	3.02	2.75	0.99	1.65	1.44	0.49
cis_mRNA_up10k	107	NA	n377879	MTCONS_00038988	chr2	139191945	139259244	867	-	chr2	139259350	139331359	6217	+	*		0.6875	1	0.12	0.11	0.07	0.89	0.7	0.69
tran	NA	NA	n341586	MTCONS_00039172	chr8	110975869	110977604	1735	-	chr2	170438918	170499115	9908	+		-774.99	0.9979	0.866	0.27	0.26	0.13	2.11	2.11	1.81
tran	NA	NA	n373939	MTCONS_00025988	chr13	98714196	98716549	643	-	chr16	15489513	15507131	6113	+		-363.2	0.8949	1	0.11	0.15	0.1	3.06	3.56	2.27
tran	742043	NA	LTCONS_00057961	MTCONS_00057977	chr5	69416727	69586449	4517	-	chr5	70328491	70585523	15856	-		-2307.07	0.8376	1	2.97	4.06	1.47	3.97	4.43	3.95
tran	184331	NA	LTCONS_00057961	MTCONS_00057969	chr5	69416727	69586449	4517	-	chr5	69770779	69881984	8211	-		-6101.55	0.9653	1	2.97	4.06	1.47	3.85	4.56	3.47
tran	29129687	NA	n367641	MTCONS_00057977	chr5	99715209	99723958	701	-	chr5	70328491	70585523	15856	-		-461.86	0.9441	1	0.88	1.6	0.46	3.97	4.43	3.95
tran	29833226	NA	n367641	MTCONS_00057969	chr5	99715209	99723958	701	-	chr5	69770779	69881984	8211	-		-501.73	0.9998	1	0.88	1.6	0.46	3.85	4.56	3.47
tran	NA	NA	n326481	MTCONS_00042413	chr1	113164315	113193960	767	-	chr2	208470330	208490126	5962	-		-651.74	-0.8871	-0.866	1.58	0	0.85	1.77	1.78	1.77
cis_mRNA_dw20k	7019	NA	n338279	MTCONS_00035337	chr19	4523904	4535208	1081	-	chr19	4542226	4581714	3971	-	*		0.9063	1	0.2	0.13	0	7.29	6.32	5.97
cis_mRNA_up10k	130	NA	n379487	MTCONS_00012487	chr11	82903179	82904704	876	-	chr11	82904833	82926321	4093	+	*		0.9078	1	0.17	0.47	0.27	1.11	1.6	1.46
cis_mRNA_overlap	0	Lnc-CompleteIn-mRNAIntron	n382077	MTCONS_00020393	chr13	74611060	74613076	2017	-	chr13	74260001	74708538	11296	-	*		0.8064	1	0.66	0.93	0.85	1.52	2.05	1.59
cis_mRNA_overlap	0	Lnc-CompleteIn-mRNAIntron	n338243	MTCONS_00022214	chr14	30166405	30170138	3734	-	chr14	30045685	30398478	5282	-	*		0.9726	1	0.05	0.07	0.11	0.17	0.21	0.54
cis_mRNA_dw20k	18376	NA	n372216	MTCONS_00008908	chr10	102819376	102820683	855	-	chr10	102790904	102801001	3185	+	*		-0.8772	-1	0.33	0.3	0.26	0.11	0.65	0.72
cis_mRNA_dw20k	2895	NA	LTCONS_00045058	MTCONS_00043982	chr20	62877030	62878115	1086	.	chr20	62795667	62874136	6306	+	*		-0.9449	-1	0.74	0.8	0.9	4.36	4.26	4.21
cis_mRNA_overlap	0	Lnc-AntiOverlap-mRNA	n338778	MTCONS_00034660	chr19	48968868	48972950	630	-	chr19	48972465	48985574	9032	+	*		0.8997	1	0	0.05	0.1	3.37	5.1	5.25
cis_mRNA_dw20k	74	NA	n365789	MTCONS_00040493	chr2	25956629	25959457	265	-	chr2	25959530	26101420	10002	-	*		0.7559	0.866	0	0.14	0.14	1.56	1.95	1.69
cis_mRNA_up10k	116	NA	n374062	MTCONS_00021218	chr14	56042876	56046810	1379	-	chr14	56046925	56151413	4760	+	*		0.987	1	0	0.26	0.48	19.13	20.29	22.02
cis_mRNA_up10k	909	NA	n363561	MTCONS_00003844	chr1	141474	149707	4860	-	chr1	131337	140566	5257	-	*		0.8514	1	1.59	2.9	2.39	0.2	0.57	0.24
cis_mRNA_up10k	189	NA	n367261	MTCONS_00055123	chr4	169931656	169932431	680	+	chr4	169815819	169931468	861	-	*		0.9724	1	0	0.05	0.14	1.79	2.33	2.75
tran	NA	NA	n345943	MTCONS_00024396	chr1	16997	29348	830	-	chr15	102500790	102516854	2835	+		-699.52	0.989	1	0.04	0	0.12	1.44	1.06	3.01
tran	NA	NA	n345943	MTCONS_00014973	chr1	16997	29348	830	-	chr12	73456	91263	2647	+		-701.58	-0.7934	-1	0.04	0	0.12	6.23	9.05	6.03
tran	NA	NA	n370234	MTCONS_00006054	chr8	9012477	9014121	643	+	chr1	151311823	151319342	4933	-		-470.18	-0.9972	-1	0.23	0	0.1	1.34	1.6	1.47
cis_mRNA_up10k	151	NA	n379327	MTCONS_00026077	chr16	21601019	21610706	1333	-	chr16	21610856	21668793	3005	+	*		-0.9318	-1	0.05	0.14	0.09	11.26	8.68	11.01
cis_mRNA_overlap	0	Lnc-AntiOverlap-mRNA	LTCONS_00040285	MTCONS_00037258	chr2	9545753	9563753	5203	-	chr2	9346894	9547571	7485	+	*		0.9978	1	1.66	2.47	2.76	0.85	1.12	1.25
cis_mRNA_overlap	0	Lnc-CompleteIn-mRNAIntron	n325542	MTCONS_00029745	chr17	65469443	65491209	628	+	chr17	65373335	65693382	6313	+	*		-0.9811	-1	0	0.21	0.19	12.82	10.72	11.32
tran	NA	NA	n339125	MTCONS_00002740	chr16	23579994	23580903	910	+	chr1	169337194	169362826	8046	+		-487.59	-0.9658	-1	0.51	0.46	0.55	1.94	2.4	1.82
tran	NA	NA	n339125	MTCONS_00058457	chr16	23579994	23580903	910	+	chr5	132028323	132073287	6288	-		-760.85	0.9847	1	0.51	0.46	0.55	40.31	38.91	42.38
cis_mRNA_overlap	0	AntiCompleteIn-mRNAIntron	n339282	MTCONS_00028113	chr16	72823142	72825522	2381	+	chr16	72816771	73093083	16108	-	*		-0.8034	-1	0.14	0.29	0.27	2.22	1.05	1.88
cis_mRNA_overlap	0	mRNA	n384498	MTCONS_00053346	chr4	120299287	120326770	1821	+	chr4	120255514	120299693	793	+	*		-0.866	-0.866	0.15	0.05	0.05	0.39	0.55	0.47
cis_mRNA_overlap	0	Lnc-CompleteIn-mRNAExon	n338076	MTCONS_00047824	chr22	38879446	38902316	1435	-	chr22	38879360	38902374	8842	-		-727.21	0.6297	1	49.31	52.72	0.86	29.56	43.82	27.83
tran	NA	NA	n340598	MTCONS_00034660	chr5	118463897	118464783	885	+	chr19	48972465	48985574	9032	+		-712.64	0.9974	0.866	0	0.18	0.18	3.37	5.1	5.25
tran	NA	NA	n340598	MTCONS_00000290	chr5	118463897	118464783	885	+	chr1	10002829	10045765	4157	+		-707.49	-0.9805	-0.866	0	0.18	0.18	1.15	0.73	0.62
tran	NA	NA	n378867	MTCONS_00028289	chr2	179278695	179296068	793	-	chr16	86562917	86576302	3893	-		-501.43	0.9933	1	0.55	1.13	0.69	0.41	0.88	0.47
tran	67658	NA	n383439	MTCONS_00035939	chr19	23299777	23332763	3202	+	chr19	23400420	23433451	7034	-		-1190.87	-0.9113	-1	0.48	0.94	0.33	1.84	1.59	2.25
tran	NA	NA	LTCONS_00032059	MTCONS_00031605	chr17_ctg5_hap1	448083	493840	1232	-	chr17	62850328	62915584	3402	-		-696.19	0.9599	1	1.39	0.6	1.84	1.24	0.84	1.28
cis_mRNA_dw20k	8806	NA	LTCONS_00042798	MTCONS_00037517	chr2	27273371	27274421	1051	.	chr2	27255774	27264566	3782	+	*		-0.9201	-1	1.61	1.01	0.54	0.76	1.58	1.63
cis_mRNA_dw20k	18120	NA	n339292	MTCONS_00028172	chr16	75607138	75608831	1594	+	chr16	75626950	75657267	9661	-	*		0.8472	1	0.8	1.42	0.97	3.3	3.52	3.47
cis_mRNA_overlap	0	Lnc-CompleteIn-mRNAExon	n337251	MTCONS_00028521	chr17	4692842	4693628	705	+	chr17	4692254	4693884	1221	+		-779.01	0.9912	1	0.22	0.14	0.03	0.33	0.23	0
cis_mRNA_up10k	217	NA	n377190	MTCONS_00046243	chr21	46708063	46709414	542	+	chr21	46682942	46707847	3837	-	*		0.9649	1	6.58	10.2	8.71	0.95	1.13	1.1
cis_mRNA_overlap	0	Lnc-CompleteIn-mRNAIntron	n342348	MTCONS_00035911	chr19	22022399	22024449	2051	-	chr19	21987482	22034892	5837	-	*		0.666	1	1.98	1.97	2.55	0.95	0.71	1.01
cis_mRNA_up10k	178	NA	n372579	MTCONS_00014359	chr11	82783124	82802318	416	+	chr11	82683907	82782947	10135	-	*		0.9654	1	0	1.5	1.05	7.95	8.89	8.39
tran	NA	NA	n376762	MTCONS_00019209	chr20	58895895	58900316	869	+	chr13	45563687	45606963	4716	+		-690.8	-0.9338	-1	0.12	0.04	0.15	8.49	8.81	8.02
tran	NA	NA	n376762	MTCONS_00042413	chr20	58895895	58900316	869	+	chr2	208470330	208490126	5962	-		-714.25	-0.9646	-0.866	0.12	0.04	0.15	1.77	1.78	1.77
tran	NA	NA	LTCONS_00073836	MTCONS_00024692	chr9	67311158	67312019	862	.	chr15	43650298	43663198	5882	-		-751.23	0.9881	1	0	0.23	0.22	6.1	6.84	6.69
tran	19188027	NA	n344662	MTCONS_00031605	chr17	43622980	43662302	428	-	chr17	62850328	62915584	3402	-		-371.36	0.9996	1	0.71	0.35	0.76	1.24	0.84	1.28
tran	NA	NA	n366903	MTCONS_00042089	chr4	139694704	139722793	866	+	chr2	175294309	175351811	9436	-		-731.81	0.9853	0.866	0.23	0.23	0.4	0.09	0.07	0.18
tran	NA	NA	n366903	MTCONS_00014454	chr4	139694704	139722793	866	+	chr11	94150004	94227093	5763	-		-531.56	-0.6049	-0.866	0.23	0.23	0.4	2.44	1.87	1.78
tran	NA	NA	n363768	MTCONS_00055313	chr1	231658263	231664302	458	-	chr4	186079719	186125182	6092	-		-240.42	0.9347	1	1.04	1.22	1.15	1.34	1.88	1.86
cis_mRNA_overlap	0	mRNA	n408031	MTCONS_00051257	chr3	112721292	112738555	4705	-	chr3	112720221	112738587	5804	-		-2025.22	0.76	1	1.82	1.25	1.77	0.95	0.8	0.84
cis_mRNA_dw20k	3409	NA	n376473	MTCONS_00035057	chr19	57808920	57811732	1541	+	chr19	57790614	57805512	4452	+	*		-0.9491	-1	8.02	6.96	7.07	2.04	3.15	2.7
tran	NA	NA	n381447	MTCONS_00035582	chr10	47133297	47133836	540	-	chr19	11776297	11778218	1922	-		-321.58	-0.9936	-1	0.23	0.1	0	0.15	0.22	0.3

cis_mRNA_dw20k	14567	NA	n378503	MTCONS_00039296	chr2	179278726	179298716	918	+	chr2	179059208	179264160	7259	+	*	-0.8164	-1	4.19	3.98	3.53	0.25	0.46	0.49
cis_mRNA_dw20k	244	NA	n340492	MTCONS_00028625	chr17	7323422	7347400	1975	+	chr17	7307982	7323179	5761	+	*	-0.8737	-1	0.65	0.6	0.8	0.89	0.96	0.86
cis_mRNA_up10k	5185	NA	n410203	MTCONS_00031467	chr17	56597611	56606778	2379	-	chr17	56566881	56592427	6432	-	*	-0.6798	-1	0.6	1.58	1.36	5.58	4.53	5.57
cis_mRNA_overlap	0	Lnc-CompleteIn-mRNAExon	n339281	MTCONS_00026826	chr16	72127834	72146802	4226	+	chr16	72127523	72150955	11560	+	-2920.01	0.9946	1	5.75	5.1	10.16	0.36	0.33	0.46
cis_mRNA_up10k	67	NA	n339281	MTCONS_00028098	chr16	72127834	72146802	4226	+	chr16	72118683	72127768	1216	-	*	0.8465	1	5.75	5.1	10.16	0.76	0.48	0.92
tran	NA	NA	LTCONS_00003817	MTCONS_00027113	chr1	14050	29381	2735	-	chr16	64484	67398	2407	-	-1642.72	-0.95	-1	1	0.39	0.6	2.83	4.39	4.34
cis_mRNA_overlap	0	Lnc-AntiOverlap-mRNA	n406963	MTCONS_00050920	chr3	56591184	56655848	3117	+	chr3	56654148	56717279	8329	-	*	-0.9946	-1	2.42	2.73	0.8	2.45	2.42	3
tran	NA	NA	n368045	MTCONS_00034981	chr5	27494420	27496274	242	+	chr19	56152203	56164527	6653	+	-213.12	0.9919	1	0	0.29	0.44	0.83	1.18	1.47
tran	NA	NA	n368045	MTCONS_00003110	chr5	27494420	27496274	242	+	chr1	202317830	202557697	11105	+	-412	0.8884	1	0	0.29	0.44	1.27	1.98	4.27
cis_mRNA_overlap	0	Lnc-AntiOverlap-mRNA	n342682	MTCONS_00058289	chr5	110847924	111075423	5523	+	chr5	110831882	110848252	5394	-	*	0.9993	1	0.01	0.02	0	31.18	43.56	17.1
tran	NA	NA	LTCONS_00024395	MTCONS_00027113	chr15	102500501	102516854	3942	+	chr16	64484	67398	2407	-	-1804.41	-0.7741	-1	0.17	0.14	0.16	2.83	4.39	4.34
cis_mRNA_dw20k	9628	NA	n366740	MTCONS_00049981	chr3	184096018	184097565	335	-	chr3	184079472	184086391	1845	+	*	-0.9666	-1	0.24	0.21	0.11	3.62	4.32	5.13
tran	NA	NA	n373453	MTCONS_00012931	chr12	127367122	127373237	356	+	chr11	124492742	124505921	5215	+	-347.87	-0.9844	-1	0.11	0	0.18	5.9	6.85	5.62
tran	NA	NA	n326650	MTCONS_00033841	chr1	32587382	32588851	687	-	chr19	17325517	17330638	3216	+	-896.82	-0.9458	-1	1.42	0.74	1.27	2.25	2.56	2.41
tran	NA	NA	n326650	MTCONS_00036211	chr1	32587382	32588851	687	-	chr19	38183957	38210684	7661	-	-649.55	0.6707	0.866	1.42	0.74	1.27	0.73	0.71	0.71
tran	NA	NA	n326650	MTCONS_00023488	chr1	32587382	32588851	687	-	chr15	40697680	40713512	6472	+	-870.81	-0.7866	-1	1.42	0.74	1.27	1.46	2.18	2.05
cis_mRNA_dw20k	18888	NA	n326650	MTCONS_00000795	chr1	32587382	32588851	687	-	chr1	32541432	32568495	2281	+	*	-0.9837	-1	1.42	0.74	1.27	0.35	0.92	0.37
cis_mRNA_dw20k	4197	NA	n376420	MTCONS_00036435	chr19	44387971	44405994	1118	-	chr19	44410190	44439669	10949	-	*	0.9146	1	1.01	0	0.51	0.61	0.52	0.6
cis_mRNA_overlap	0	mRNA	n376420	MTCONS_00036428	chr19	44387971	44405994	1118	-	chr19	44376515	44405994	2831	-	-537.03	0.9851	1	1.01	0	0.51	3	1.36	1.94
cis_mRNA_overlap	0	mRNA	n338589	MTCONS_00003698	chr1	239549865	240049896	2005	+	chr1	239549802	240078814	9309	+	-1187.31	0.9528	1	0.2	0.04	0.42	1.67	1.32	3.92
tran	NA	NA	n387402	MTCONS_00039534	chr6	33367540	33368371	832	+	chr2	203879600	204056027	8171	+	-1159.3	-0.9661	-1	0.17	0.27	0.34	0.89	0.6	0.54
cis_mRNA_up10k	537	NA	n333477	MTCONS_00025685	chr16	1030303	1031272	970	-	chr16	1031808	1037079	4532	+	*	-0.8922	-1	2.71	1.58	1.78	1.34	2.01	1.61
cis_mRNA_up10k	90	NA	n364986	MTCONS_00038840	chr2	124774435	124782750	542	-	chr2	124782839	125678798	11246	+	*	0.9888	1	3.07	2.55	2.19	7.65	6.71	5.61
cis_mRNA_overlap	0	Lnc-CompleteIn-mRNAIntron	n381568	MTCONS_00009186	chr10	122666807	122667788	982	+	chr10	122610608	122669039	5831	+	*	0.9962	1	0.29	0.69	0.39	0.93	2.02	1.29
cis_mRNA_overlap	0	Lnc-CompleteIn-mRNAExon	n346494	MTCONS_00040072	chr2	243030887	243101644	670	+	chr2	243030817	243102469	1310	+	-310.63	-0.997	-1	0.08	0.24	0	1.04	0.59	1.2
cis_mRNA_up10k	1046	NA	LTCONS_00006205	MTCONS_00002432	chr1	155141806	155146258	1407	-	chr1	155147303	155157450	3800	+	*	-0.7708	-1	4.39	3.99	3.21	3.67	5.73	5.77
tran	NA	NA	n326366	MTCONS_00024133	chr1	155421802	155423819	471	+	chr15	83490056	83503824	3232	+	-733.36	-0.9559	-1	0.9	0.8	0.74	0.18	0.38	0.4
cis_mRNA_up10k	2974	NA	n382487	MTCONS_00024535	chr15	32828915	32872810	1279	+	chr15	32806173	32825942	1395	-	*	0.9153	1	0.46	0.2	0.49	0.2	0	0.39
tran	NA	NA	n366295	MTCONS_00052857	chr3	119817722	119824125	608	+	chr4	56212388	56254858	7355	+	-471.07	-0.9898	-1	0.21	0.33	0.12	0.5	0.33	0.71
cis_mRNA_up10k	3437	NA	LTCONS_00035975	MTCONS_00035963	chr19	23581791	23598903	2997	-	chr19	23486602	23578355	10046	-	*	0.9832	1	1	0.83	0.67	5.86	5.36	4.45
tran	5793399	NA	LTCONS_00024238	MTCONS_00025322	chr15	90837902	90838450	217	+	chr15	84941992	85044504	18018	-	-367.38	0.7663	1	0.81	0.37	0.36	10.55	9.49	7.34
cis_mRNA_overlap	0	Lnc-CompleteIn-mRNAExon	n365224	MTCONS_00038754	chr2	118594189	118596698	1753	+	chr2	118572251	118597355	10414	+	-1151.49	0.8937	0.866	0.05	0.94	0.54	0.63	0.76	0.76
cis_mRNA_dw20k	8802	NA	n406246	MTCONS_00034856	chr19	53868968	53889841	6094	+	chr19	53836947	53860167	6513	+	*	-0.9544	-1	2.08	1.79	2.07	1.92	2.43	2.09
cis_mRNA_dw20k	1740	NA	n345300	MTCONS_00024478	chr15	25570911	25580648	2022	+	chr15	25582387	25684237	5098	-	*	-0.9445	-1	0.07	0.15	0.09	27.71	24.16	25.77
cis_mRNA_overlap	0	mRNA-AntiCompleteIn-LncIntron	n385994	MTCONS_00008660	chr10	88965545	89102315	1420	-	chr10	88983430	88997403	8066	+	*	0.9982	1	0.25	0.21	0.44	0.92	0.76	1.45
cis_mRNA_dw20k	18472	NA	n325099	MTCONS_00042698	chr2	239084070	239128434	1975	-	chr2	239146905	239148681	1608	-	*	-0.949	-1	0.74	1.01	1.14	18.12	13.21	13.19
cis_mRNA_overlap	0	Lnc-AntiOverlap-mRNA	LTCONS_00032149	MTCONS_00032736	chr18	5895724	5901100	5377	+	chr18	5890093	5896850	3886	-	-2605.5	0.993	1	0.46	0.86	0.32	1.77	2.78	1.6
cis_mRNA_overlap	0	Lnc-CompleteIn-mRNAIntron	n345550	MTCONS_00039148	chr2	168351651	168394068	1044	+	chr2	168148819	168730621	6608	+	*	0.9749	1	1.02	1.42	1.57	10.67	15.23	15.46
cis_mRNA_up10k	2	NA	n337980	MTCONS_00005135	chr1	63154153	63176365	1228	+	chr1	62919212	63154152	8486	-	*	0.8561	1	0.77	0.62	1.24	8.2	5.94	9.06
tran	NA	NA	n387169	MTCONS_00049362	chr2	170183043	170183659	617	+	chr3	124449213	124470145	8967	+	-1340.21	0.9547	1	6.22	8.04	7.4	3.28	3.93	3.53
tran	NA	NA	n387169	MTCONS_00004585	chr2	170183043	170183659	617	+	chr1	31648621	31712835	6994	-	-1346.51	0.9901	1	6.22	8.04	7.4	5.43	6.82	6.16
cis_mRNA_overlap	0	Lnc-AntiOverlap-mRNA	n345121	MTCONS_00010273	chr10	75361281	75386426	2236	+	chr10	75257255	75385737	6930	-	*	0.7764	1	0.35	0.21	0.25	2.54	2.11	2.5
tran	NA	NA	n325153	MTCONS_00019045	chr2	122264506	122269086	650	+	chr13	28712637	28847529	3366	+	-552.39	0.991	1	0	0.11	0.04	2.72	3.24	2.97
cis_mRNA_overlap	0	AntiCompleteIn-mRNAIntron	n4886	MTCONS_00023166	chr15	23812616	23814443	1827	-	chr15	23809947	23875212	4485	+	*	0.9282	1	0.19	0.02	0.05	1.59	0.38	1.01
tran	NA	NA	n325607	MTCONS_00041449	chr14	100485068	100492267	739	+	chr2	101887052	101925178	6597	-	-480.37	0.9365	0.866	0	0.09	0.09	1.64	2.09	1.93
tran	NA	NA	n325607	MTCONS_00004052	chr14	100485068	100492267	739	+	chr1	6281253	6296045	4923	-	-699.12	0.9754	0.866	0	0.09	0.09	6.45	7.45	7.75
cis_mRNA_overlap	0	mRNA	LTCONS_00052625	MTCONS_00052624	chr4	20702416	20730233	3221	+	chr4	20702036	20730233	2167	+	-918.11	0.985	1	0.4	0.93	0.5	0.74	0.94	0.81
cis_mRNA_overlap	0	Lnc-AntiOverlap-mRNA	LTCONS_00052625	MTCONS_00054158	chr4	20702416	20730233	3221	+	chr4	20729979	20884333	2419	-	*	-0.9207	-1	0.4	0.93	0.5	1.63	0.08	0.78
cis_mRNA_up10k	9	NA	n340943	MTCONS_00011189	chr11	2419371	2421840	2470	-	chr11	2421848	2425110	1608	+	*	0.9895	1	0.36	0.2	0.43	11.21	6.76	12.1
tran	NA	NA	n332940	MTCONS_00010273	chr15	51034900	51035473	574	-	chr10	75257255	75385737	6930	-	-629.86	0.9999	1	0.38	0	0.34	2.54	2.11	2.5
cis_mRNA_overlap	0	mRNA	LTCONS_00000505	MTCONS_00000501	chr1	19638740	19660931	8176	+	chr1	19638709	19660931	7837	+	-5659.38	-0.9895	-1	0.53	2.2	0.94	1.51	0	0.94
tran	170900857	NA	n332848	MTCONS_00037932	chr2	232189116	232189629	514	+	chr2	61244808	61288260	10877	+	-524.03	-0.9983	-1	0	0.26	0.37	2.22	1.94	1.85
cis_mRNA_dw20k	16664	NA	n339168	MTCONS_00039883	chr2	232258390	232259702	1313	+	chr2	232063206	232241727	8428	+	*	-0.8478	-1	0.16	0.29	0.07	3.05	3	3.65
cis_mRNA_overlap	0	mRNA	n410892	MTCONS_00021809	chr14	102606189	102676867	2225	+	chr14	102606180	102686953	12342	+	-1359.11	-0.8592	-1	1.19	1.05	0.6	0.67	0.82	0.88
tran	NA	NA	n371860	MTCONS_00037620	chr10	38064299	38073059	712	-	chr2	32288518	32383018	5380	+	-639.72	0.9999	1	0.72	1.07	1.06	2.96	3.95	3.91
tran	NA	NA	n385194	MTCONS_00028965	chr7	65112466	65235797	1419	-	chr17	25783087	25953505	10574	+	-1583.94	0.9678	1	0	0.04	0.02	2.82	3.73	3.48
cis_mRNA_overlap	0	Lnc-AntiOverlap-mRNA	n340123	MTCONS_00010599	chr10	101686966	101718755	2651	+	chr10	101634837	101769744	6569	-	*	0.9832	1	0	0.01	0.02	0.26	0.46	0.85
tran	NA	NA	n367704	MTCONS_00046974	chr5	7373230	7379903	577	+	chr22	38301875	38339049	6269	+	-659.34	0.6719	0.866	0.1	0.18	0.1	6.51	6.96	6.86

cis_mRNA_overlap	0	Lnc-AntiOverlap-mRNA	n342279	MTCONS_00025285	chr15	83316521	83361572	2138	+	chr15	83328033	83378667	3641	-	*		-0.9935	-1	0.11	0.1	0.17	4.63	4.95	3.69
tran	2743586	NA	LTCONS_00005930	MTCONS_00002104	chr1	147574207	147578137	2587	-	chr1	144593657	144830622	7756	+		-1374.14	0.9964	1	1.28	1.78	1.57	7.98	8.75	8.37
tran	NA	NA	n345697	MTCONS_00057778	chr3	138544653	138545200	429	+	chr5	43486765	43496143	2426	-		-258.46	0.7146	0.866	0	0.08	0.08	0.05	0.23	0.1
cis_mRNA_overlap	0	Lnc-AntiOverlap-mRNA	n341094	MTCONS_00012532	chr11	89927674	89931288	3614	-	chr11	89867687	89931113	8593	+		-6380.2	0.7651	0.866	4.48	7.11	5.39	0.08	0.12	0.12
cis_mRNA_overlap	0	Lnc-AntiOverlap-mRNA	n324651	MTCONS_00047956	chr22	45316056	45322757	2949	+	chr22	45276967	45405879	3918	-	*		0.8472	1	0.16	0.22	0.11	4.4	4.45	1.71
cis_mRNA_overlap	0	Lnc-AntiOverlap-mRNA	LTCONS_00040287	MTCONS_00037258	chr2	9545753	9563753	2825	-	chr2	9346894	9547571	7485	+	*		0.9585	1	0.66	1.12	1.75	0.85	1.12	1.25
tran	NA	NA	n338331	MTCONS_00041449	chr1	161929297	161929914	615	+	chr2	101887052	101925178	6597	-		-354.13	-0.872	-1	4.02	3.86	3.99	1.64	2.09	1.93
cis_mRNA_up10k	772	NA	n345110	MTCONS_00008032	chr10	28813108	28820522	1471	-	chr10	28821293	28912041	6050	+	*		-0.9711	-1	1.59	1.32	0.93	3.62	3.78	4.47
tran	NA	NA	n344961	MTCONS_00041449	chr7	95306193	95308564	831	+	chr2	101887052	101925178	6597	-		-808.65	0.9933	1	0.9	1.34	1.23	1.64	2.09	1.93
cis_mRNA_up10k	5124	NA	n346051	MTCONS_00007297	chr1	228302157	228309028	1372	-	chr1	228294290	228297034	1386	-	*		0.9506	1	0.06	0.14	0	4.8	5.1	4.08
cis_mRNA_dw20k	1985	NA	n407704	MTCONS_00028789	chr17	16342301	16373962	898	+	chr17	16318851	16340317	2810	+	*		-0.9937	-1	0.34	0.39	0.18	0.4	0	3.14
cis_mRNA_up10k	8475	NA	LTCONS_00052171	MTCONS_00052157	chr3	197363226	197374168	7842	-	chr3	197299019	197354752	9196	-	*		0.9072	1	0.09	0.24	0.12	1.79	2.76	2.36
tran	NA	NA	n325722	MTCONS_00007912	chr13	53587925	53605002	1058	-	chr10	15139117	15147770	4236	+		-784.24	-1	-1	0.1	0.07	0.03	0.57	0.6	0.64
tran	NA	NA	n325722	MTCONS_00014454	chr13	53587925	53605002	1058	-	chr11	94150004	94227093	5763	-		-501.85	0.8871	1	0.1	0.07	0.03	2.44	1.87	1.78
cis_mRNA_dw20k	1973	NA	n379246	MTCONS_00028789	chr17	16342289	16345052	763	+	chr17	16318851	16340317	2810	+	*		-0.9926	-1	9.82	12.27	2.34	0.4	0	3.14
tran	NA	NA	LTCONS_00047464	MTCONS_00041449	chr22	22674152	22675668	1517	-	chr2	101887052	101925178	6597	-		-973.72	0.9546	1	1.28	1.99	1.55	1.64	2.09	1.93
tran	1461062	NA	LTCONS_00047464	MTCONS_00047425	chr22	22674152	22675668	1517	-	chr22	21061922	21213091	8306	-		-996.65	0.8252	1	1.28	1.99	1.55	1.07	1.66	1.62
cis_mRNA_overlap	0	mRNA	LTCONS_00047625	MTCONS_00047626	chr22	29832933	29869240	6132	-	chr22	29832933	29876337	5283	-		-3281.26	-0.7805	-1	0.55	0.56	0.47	2.32	2.11	2.4
cis_mRNA_dw20k	830	NA	n410894	MTCONS_00022224	chr14	31345385	31359014	1991	-	chr14	31359843	31495629	7265	-	*		0.9887	1	0.07	0.11	0.2	1.29	1.44	1.63
cis_mRNA_overlap	0	Lnc-AntiOverlap-mRNA	n344473	MTCONS_00013654	chr11	34736325	34899030	977	+	chr11	34896691	34937942	1116	-	*		0.8994	1	3.44	0.76	2.61	3.31	0.67	1.44
cis_mRNA_overlap	0	mRNA	n336935	MTCONS_00019280	chr13	49822070	49841950	855	+	chr13	49822047	49868035	3589	+		-421.87	-0.9912	-0.866	0.08	0	0.08	0.82	1.12	0.77
tran	2879559	NA	n405311	MTCONS_00047626	chr22	32755895	32766972	1115	-	chr22	29832933	29876337	5283	-		-632.18	-0.9925	-1	0.25	0.6	0.03	2.32	2.11	2.4
tran	NA	NA	n344625	MTCONS_00041449	chr5	25433945	25445978	426	-	chr2	101887052	101925178	6597	-		-344.53	0.9705	1	0	0.08	0.07	1.64	2.09	1.93
tran	NA	NA	n368759	MTCONS_00010917	chr6	111599135	111599945	557	+	chr10	127700851	128078892	9896	-		-525.81	0.8633	1	1.98	1.61	1.3	2	1.98	1.61
cis_mRNA_dw20k	3032	NA	n377298	MTCONS_00047425	chr22	21055402	21058891	1958	+	chr22	21061922	21213091	8306	-	*		-0.9884	-1	0.5	0.41	0.43	1.07	1.66	1.62
cis_mRNA_overlap	0	mRNA	n375968	MTCONS_00032583	chr18	68002593	68019686	888	+	chr18	67956126	68004391	7654	+		-467.46	-0.9734	-0.866	0.46	0	0.11	0.25	0.69	0.69
cis_mRNA_overlap	0	Lnc-CompleteIn-mRNAIntron	n326691	MTCONS_00000480	chr1	17916769	17949060	610	+	chr1	17866327	18024370	4482	+	*		-0.9152	-1	0.06	0.05	0.27	21.65	23.5	19.3
tran	NA	NA	n384848	MTCONS_00049362	chr6	27793690	27794715	588	+	chr3	124449213	124470145	8967	+		-504.07	0.9826	1	1	2.17	1.64	3.28	3.93	3.53
tran	NA	NA	n384848	MTCONS_00039494	chr6	27793690	27794715	588	+	chr2	201980877	202038063	11977	+		-472.1	0.9977	1	1	2.17	1.64	0.42	0.83	0.62
tran	NA	NA	n384848	MTCONS_00004585	chr6	27793690	27794715	588	+	chr1	31648621	31712835	6994	-		-512.94	0.9997	1	1	2.17	1.64	5.43	6.82	6.16
tran	NA	NA	n375912	MTCONS_00024784	chr18	7272467	7277412	786	+	chr15	50849351	50979012	10401	-		-753.52	-0.997	-1	0.14	0.43	0.34	2.04	1.46	1.68
tran	NA	NA	n386067	MTCONS_00008814	chr14	56232963	56234434	1472	+	chr10	98510030	98510686	657	+		-427.58	0.9972	1	0.09	0.04	0.06	4.13	2.15	2.81
cis_mRNA_overlap	0	Lnc-AntiOverlap-mRNA	n377414	MTCONS_00047421	chr22	20850199	20856009	1107	+	chr22	20795806	20850234	2648	-	*		0.9317	1	0.53	0	0.28	2.86	0.85	2.6
tran	2133186	NA	n406511	MTCONS_00034269	chr19	35417807	35436076	2664	+	chr19	37569261	37621663	4191	+		-1227.76	-0.9547	-0.866	3.05	2.64	3.23	3.92	4.31	3.92
cis_mRNA_up10k	1337	NA	n341041	MTCONS_00011964	chr11	61721451	61725149	639	-	chr11	61726485	61732030	3335	+	*		-0.8358	-0.866	0.4	0.31	0.2	0.09	0.19	0.19
tran	NA	NA	n378985	MTCONS_00039494	chr6	132223103	132241705	2305	+	chr2	201980877	202038063	11977	+		-1293.6	0.9999	1	0.03	0.05	0.04	0.42	0.83	0.62
cis_mRNA_dw20k	615	NA	n384189	MTCONS_00047174	chr22	46691041	46692557	1517	-	chr22	46663861	46690427	3132	+	*		0.9986	1	0.69	0.96	0.73	0.13	0.38	0.18
tran	NA	NA	LTCONS_00018856	MTCONS_00026659	chr12	8116219	8117218	1000	.	chr16	66914383	66925004	7056	+		-710.89	-0.9449	-0.866	0	0.03	0.03	1.68	1.04	0.72
tran	48797325	NA	LTCONS_00018856	MTCONS_00015928	chr12	8116219	8117218	1000	.	chr12	56914542	56989980	8281	+		-833.11	0.9838	0.866	0	0.03	0.03	0.72	1.14	1.06
cis_mRNA_overlap	0	Lnc-CompleteIn-mRNAExon	n410104	MTCONS_00056688	chr5	138609441	138667366	4515	+	chr5	138609328	138667366	6070	+		-1887.88	0.9437	1	0.73	0.6	0.48	0.88	0.5	0.42
tran	1937245	NA	LTCONS_00024120	MTCONS_00025322	chr15	83004404	83004748	345	+	chr15	84941992	85044504	18018	-		-311.11	-0.9374	-1	2.91	3.68	4.11	10.55	9.49	7.34
tran	NA	NA	n324054	MTCONS_00012837	chr9	88436882	88501231	530	+	chr11	119019750	119034182	8465	+		-526.77	0.9707	1	0.15	0.19	0.17	1.29	1.64	1.54
cis_mRNA_dw20k	6662	NA	n381552	MTCONS_00010704	chr10	106061962	106064704	2743	+	chr10	106071365	106088828	4664	-	*		-0.6959	-1	0.8	3.15	0.62	0.78	0.73	0.99
cis_mRNA_overlap	0	Lnc-AntiOverlap-mRNA	n339017	MTCONS_00039237	chr2	173587914	173600934	4625	-	chr2	173600525	173917621	4295	+	*		0.8573	0.866	0.01	0.03	0.03	2.36	4.03	3.17
cis_mRNA_up10k	9106	NA	n411615	MTCONS_00045997	chr21	37441940	37498938	1221	-	chr21	37406839	37432835	3302	-	*		-0.9501	-1	0.06	0.03	0	1.21	1.32	1.72
cis_mRNA_dw20k	8403	NA	LTCONS_00046817	MTCONS_00047652	chr22	30752108	30763251	2441	+	chr22	30771653	30783401	3679	-	*		0.679	1	0.94	1.09	1.73	4.58	5.95	6.02
cis_mRNA_overlap	0	mRNA	n337412	MTCONS_00038329	chr2	88038283	88038784	502	+	chr2	88019354	88043893	6481	+		-261.16	0.9629	1	0.28	1.48	0.73	1.18	1.95	1.27
cis_mRNA_overlap	0	Lnc-AntiOverlap-mRNA	LTCONS_00017400	MTCONS_00015507	chr12	31203850	31226781	1236	-	chr12	31226779	31257730	4327	+	*		0.9895	1	0.56	0.57	0.65	1.63	1.95	2.89
cis_mRNA_dw20k	1196	NA	n339886	MTCONS_00052624	chr4	20731428	20734177	2687	+	chr4	20702036	20730233	2167	+	*		1	1	1.02	1.6	1.22	0.74	0.94	0.81
cis_mRNA_overlap	0	Lnc-AntiOverlap-mRNA	n339886	MTCONS_00054158	chr4	20731428	20734177	2687	+	chr4	20729979	20884333	2419	-	*		-0.973	-1	1.02	1.6	1.22	1.63	0.08	0.78
tran	NA	NA	n339024	MTCONS_00018846	chr2	176047421	176048342	922	+	chr12	133494970	133532981	9230	-		-504.37	-0.998	-1	0.08	0	0.03	1.87	2.09	2.02
tran	NA	NA	n339024	MTCONS_00047616	chr2	176047421	176048342	922	+	chr22	29655844	29663960	3044	-		-580.72	0.9246	1	0.08	0	0.03	11.05	8.81	10.42
tran	NA	NA	n324674	MTCONS_00034660	chr22	31053158	31069092	361	-	chr19	48972465	48985574	9032	+		-511.37	0.6541	1	0.42	0.48	0.89	3.37	5.1	5.25
tran	NA	NA	n324119	MTCONS_00010680	chr9	36322947	36324969	388	-	chr10	104838242	104954942	14991	-		-356.79	0.9074	1	0	0.45	0.18	0.45	0.59	0.56
cis_mRNA_up10k	725	NA	n337282	MTCONS_00014277	chr11	73882788	73883602	815	+	chr11	73722714	73882064	9005	-	*		-0.8985	-0.866	0.35	0.28	0.35	1.98	2.12	1.87
cis_mRNA_overlap	0	mRNA	n325558	MTCONS_00029016	chr17	28256793	28257715	512	+	chr17	28256793	28435470	5092	+	*		-0.8846	-1	0.29	0.07	0.06	0.02	0.06	0.1
cis_mRNA_dw20k	4979	NA	n337729	MTCONS_00000422	chr1	16273333	16274279	947	+	chr1	16174350	16268355	14900	+	*		-0.9532	-1	0.15	0.2	0.23	1.82	1.48	1.45
cis_mRNA_overlap	0	Lnc-AntiOverlap-mRNA	n383549	MTCONS_00034837	chr19	52897580	52901010	1855	-	chr19	52872319	52925184	17190	+	*		-0.8078	-1	0.63	0.27	0.67	2.75	2.91	2.42
tran	7383427	NA	n372036	MTCONS_00008660	chr10	81598817	81600004	676	+	chr10	88983430	88997403	8066	+		-457.02	0.9997	1	1.55	0.95	3.81	0.92	0.76	1.45
cis_mRNA_overlap	0	Lnc-CompleteIn-mRNAIntron	n382275	MTCONS_00021800	chr14	102262244	102263248	1005	+	chr14	102228135	102379075	1744	+	*		-0.9062	-1	0.04	0	0.13	4.57	6.39	3.76

cis_mRNA_overlap	0	mRNA	n345594	MTCONS_00051281	chr3	114033687	114052767	4776	-	chr3	114036241	114173624	24126	-		-2090.99	0.9203	1	0.91	1.22	1.14	1.8	2.42	2.04
tran	NA	NA	n324708	MTCONS_00040619	chr22	21998803	22000680	346	+	chr2	37323735	37384351	12737	-		-431.85	0.9997	1	0.11	0.31	0	4.96	5.38	4.75
tran	NA	NA	n324708	MTCONS_00042413	chr22	21998803	22000680	346	+	chr2	208470330	208490126	5962	-		-428.42	0.9368	0.866	0.11	0.31	0	1.77	1.78	1.77
tran	NA	NA	n406201	MTCONS_00006433	chr19	23945816	24010919	1379	+	chr1	160236583	160237032	450	-		-281.15	-0.9902	-1	9.25	5.65	1.18	0	0.1	0.31
tran	NA	NA	n406201	MTCONS_00020697	chr19	23945816	24010919	1379	+	chr13	21535411	21535878	468	.		-293.75	0.9811	1	9.25	5.65	1.18	3.35	1.51	0.36
cis_mRNA_overlap	0	Lnc-AntiOverlap-mRNA	n407915	MTCONS_00017049	chr12	6977278	6980110	1594	+	chr12	6980061	6982521	1543	-	*		0.8852	1	1.15	3.64	2.81	1.5	1.92	1.6
tran	NA	NA	n385983	MTCONS_00047425	chr10	24736419	24736989	571	-	chr22	21061922	21213091	8306	-		-605.92	0.8675	1	0.94	1.14	1.03	1.07	1.66	1.62
cis_mRNA_overlap	0	Lnc-CompleteIn-mRNAExon	n341038	MTCONS_00011929	chr11	61114590	61116612	1639	+	chr11	61100636	61116684	6002	+		-1161.37	-0.6719	-0.866	0.02	0.02	0	1.2	1.76	1.92
cis_mRNA_overlap	0	Lnc-AntiOverlap-mRNA	n384341	MTCONS_00049655	chr3	150283627	150321015	1552	-	chr3	150264441	150304140	4349	+	*		-0.9796	-1	5.29	5.28	3.89	11.61	12.25	14.55
tran	NA	NA	n369953	MTCONS_00034476	chr7	50292780	50296511	946	-	chr19	44455373	44485875	16403	+		-723.14	-0.9325	-1	0.4	1.09	0.93	3.03	2.78	2.92
cis_mRNA_overlap	0	Lnc-AntiOverlap-mRNA	n374648	MTCONS_00025087	chr15	74044453	74062811	1286	+	chr15	74027689	74044549	6117	-	*		0.6668	1	0.2	0.22	0	1.68	2.97	1.48
tran	NA	NA	n406569	MTCONS_00024396	chr2	114341230	114356613	1958	+	chr15	102500790	102516854	2835	+		-1450.67	0.8263	1	12.65	7.35	14.61	1.44	1.06	3.01
tran	NA	NA	n406569	MTCONS_00014973	chr2	114341230	114356613	1958	+	chr12	73456	91263	2647	+		-1428.26	-0.9791	-1	12.65	7.35	14.61	6.23	9.05	6.03
tran	1173487	NA	n409660	MTCONS_00035875	chr19	21106059	21126783	1554	+	chr19	19899903	19932573	3778	-		-839.14	0.9975	1	6.2	3.82	2.16	4.89	3.95	3.11
cis_mRNA_up10k	4755	NA	LTCONS_00027143	MTCONS_00025637	chr16	433523	447104	1219	-	chr16	451858	462487	2323	+	*		0.7569	1	1	1.67	0.87	2.52	2.8	1.35
cis_mRNA_up10k	1574	NA	LTCONS_00027143	MTCONS_00027141	chr16	433523	447104	1219	-	chr16	420716	431950	3956	-	*		-0.6708	-1	1	1.67	0.87	1.28	1.22	2.12
cis_mRNA_overlap	0	mRNA	n379691	MTCONS_00045997	chr21	37426162	37481988	2124	-	chr21	37406839	37432835	3302	-	*		0.6669	0.866	0.05	0.06	0.06	1.21	1.32	1.72
tran	NA	NA	n382635	MTCONS_00052193	chr15	74646797	74656960	3336	+	chr3	44914803	44915539	737	.		-395.76	0.6449	0.866	0.07	0	0.07	1.56	0.44	0.65
cis_mRNA_up10k	23	NA	n376192	MTCONS_00035496	chr19	9649409	9650402	857	+	chr19	9636407	9649387	4891	-	*		-0.771	-1	2.6	1.36	1.6	5.91	6.4	6
cis_mRNA_dw20k	19763	NA	n385926	MTCONS_00003895	chr1	995083	997436	673	+	chr1	1017198	1028234	2996	-	*		-0.9661	-1	0.11	0.2	0.05	1.3	1.11	1.62
tran	NA	NA	n325771	MTCONS_00039148	chr13	29891656	29894374	448	-	chr2	168148819	168730621	6608	+		-580.32	0.7811	1	0.59	0.97	1.74	10.67	15.23	15.46
tran	NA	NA	n365163	MTCONS_00034660	chr2	59246798	59288575	754	+	chr19	48972465	48985574	9032	+		-661.4	-0.993	-1	0.06	0.02	0.01	3.37	5.1	5.25
cis_mRNA_overlap	0	mRNA	n341001	MTCONS_00013563	chr11	27717859	27899195	358	-	chr11	27676440	27722035	4359	-	*		-0.6054	-1	0.11	0.1	0	0.13	0.32	0.33
cis_mRNA_overlap	0	Lnc-AntiOverlap-mRNA	n373180	MTCONS_00018851	chr12	133692099	133698651	4867	+	chr12	133697491	133707110	1592	-		-2168.08	0.8932	1	0.03	0	0.02	3.26	2.12	2.41
cis_mRNA_dw20k	6796	NA	n339932	MTCONS_00033248	chr18	77905932	77929612	3205	+	chr18	77936407	78005397	901	-	*		0.9507	1	0.01	0	0.07	0.89	0.13	1.88
tran	NA	NA	n407672	MTCONS_00009164	chrX	129628915	129631421	2507	+	chr10	120863585	120900424	5429	+		-1281.34	-0.9449	-0.866	0.07	0.04	0.07	0.26	0.32	0.28
cis_mRNA_dw20k	546	NA	n341053	MTCONS_00014181	chr11	67387475	67394864	1859	+	chr11	67395409	67397579	916	-	*		-0.988	-1	0.03	0	0.2	1.49	1.66	1.09
cis_mRNA_overlap	0	Lnc-CompleteIn-mRNAIntron	n326140	MTCONS_00007455	chr1	241325916	241327512	530	-	chr1	240938755	241520769	2739	-	*		0.8734	1	0.07	0	0.19	3.66	1.32	4.16
cis_mRNA_overlap	0	mRNA	LTCONS_00033963	MTCONS_00033964	chr19	21324825	21380525	8116	+	chr19	21324829	21380525	8338	+		-3752.38	-0.955	-1	1.82	2.16	1.16	1.5	1.09	1.77
cis_mRNA_dw20k	5400	NA	n363928	MTCONS_00001849	chr1	109754802	109756393	463	+	chr1	109656537	109749403	7111	+	*		0.9124	1	0.08	0	0.15	1.12	0.93	2.19
cis_mRNA_up10k	5506	NA	n344668	MTCONS_00020890	chr14	23767526	23770466	1650	-	chr14	23775971	23782791	5725	+	*		0.9195	1	1.31	1.07	0.78	2.59	2.55	2.04
tran	62058525	NA	n366154	MTCONS_00049362	chr3	186528669	186543310	1832	-	chr3	124449213	124470145	8967	+		-2904.48	0.9997	1	2.48	4.82	3.43	3.28	3.93	3.53
tran	NA	NA	n366154	MTCONS_00004585	chr3	186528669	186543310	1832	-	chr1	31648621	31712835	6994	-		-2955.05	0.9906	1	2.48	4.82	3.43	5.43	6.82	6.16
tran	NA	NA	LTCONS_00045803	MTCONS_00008814	chr21	26734512	26734840	329	-	chr10	98510030	98510686	657	+		-555.7	0.9911	1	0.63	0.12	0.35	4.13	2.15	2.81
tran	512026	NA	n410146	MTCONS_00034856	chr19	53300661	53324922	4467	-	chr19	53836947	53860167	6513	+		-1791.85	0.9064	1	2.1	2.56	2.43	1.92	2.43	2.09
cis_mRNA_up10k	108	NA	LTCONS_00012444	MTCONS_00014333	chr11	77850806	77885023	1031	+	chr11	77811985	77850699	2455	-	*		-0.9989	-1	0.08	0	0.06	3.61	5.01	4.02
cis_mRNA_dw20k	14923	NA	n409540	MTCONS_00000678	chr1	27650365	27653016	2652	+	chr1	27561007	27635443	5138	+	*		-0.9545	-1	1.4	1.53	1.61	7.09	6.98	6.78
cis_mRNA_up10k	512	NA	n383845	MTCONS_00042960	chr20	3867915	3869231	1317	-	chr20	3869742	3907953	11798	+	*		0.9872	1	0.85	1.45	1.32	1.78	2.66	2.34
cis_mRNA_overlap	0	Lnc-CompleteIn-mRNAExon	n344852	MTCONS_00056713	chr5	139496843	139501752	2837	+	chr5	139487296	139505501	12385	+		-1246.13	0.976	1	0.18	0.45	0	1.54	1.72	1.28
cis_mRNA_up10k	297	NA	n365391	MTCONS_00037258	chr2	9344859	9346598	1369	-	chr2	9346894	9547571	7485	+	*		-0.7784	-1	0.2	0.19	0	0.85	1.12	1.25
tran	NA	NA	n384181	MTCONS_00026587	chr22	43035809	43036607	799	-	chr16	58035277	58053422	4274	+		-486.34	-0.9121	-1	0.2	0.14	0.06	1.25	1.5	1.56
tran	NA	NA	n370780	MTCONS_00014300	chr9	95909322	95910838	410	+	chr11	75297963	75382024	11247	-		-280.85	-0.8095	-1	2.24	1.1	2.04	14.6	18.81	17.71
cis_mRNA_up10k	7	NA	n406455	MTCONS_00030266	chr17	4046462	4060991	1756	+	chr17	3907735	4046456	11605	-	*		-0.9998	-1	0.26	0.25	0.33	6.88	7.09	5.65
tran	NA	NA	n377732	MTCONS_00009539	chr19	46692287	46706319	1643	-	chr10	11502054	11537045	5168	-		-878.08	-0.7835	-1	0.03	0	0.02	2.68	3.09	3.07
tran	NA	NA	n372179	MTCONS_00003844	chr10	38736775	38739473	1866	-	chr1	131337	140566	5257	-		-3912.3	-0.9951	-0.866	0.02	0	0.02	0.2	0.57	0.24
tran	NA	NA	n372179	MTCONS_00027110	chr10	38736775	38739473	1866	-	chr16	90229013	90235598	2977	+		-3791.95	-0.6389	-0.866	0.02	0	0.02	0.06	0.95	0.79
cis_mRNA_overlap	0	Lnc-AntiOverlap-mRNA	n339678	MTCONS_00051817	chr3	169512750	169514001	1250	+	chr3	169511216	169530574	2442	-		-1355.74	-0.8391	-1	1.37	1.17	0.8	0	0.27	0.31
tran	NA	NA	n375174	MTCONS_00042413	chr16	77786366	77809904	836	+	chr2	208470330	208490126	5962	-		-707.72	-0.8874	-0.866	0.13	0	0.07	1.77	1.78	1.77
tran	NA	NA	n324813	MTCONS_00015928	chr21	33450808	33453286	449	+	chr12	56914542	56989980	8281	+		-267.47	0.7939	1	0	0.36	0.08	0.72	1.14	1.06
cis_mRNA_overlap	0	Lnc-CompleteIn-mRNAIntron	n337678	MTCONS_00024501	chr15	29665806	29669160	3352	-	chr15	29412430	29862927	4960	-	*		0.8624	1	0.43	0.36	0.84	4.27	1.96	5.66
cis_mRNA_up10k	557	NA	n339150	MTCONS_00042528	chr2	220253218	220268333	1576	+	chr2	220236072	220252662	4546	-	*		-1	-1	0.02	0	0.02	0.82	1.07	0.82
cis_mRNA_overlap	0	Lnc-CompleteIn-mRNAExon	n338920	MTCONS_00041438	chr2	100889909	100898479	6689	-	chr2	100888796	100939338	15012	-		-3651.98	0.9751	1	3.7	2.45	2.34	0.28	0.14	0.08
cis_mRNA_overlap	0	Lnc-CompleteIn-mRNAExon	LTCONS_00041448	MTCONS_00041449	chr2	101887052	101891565	2443	-	chr2	101887052	101925178	6597	-		-1595.11	-0.999	-1	0.28	0.09	0.15	1.64	2.09	1.93
tran	NA	NA	LTCONS_00041448	MTCONS_00052193	chr2	101887052	101891565	2443	-	chr3	44914803	44915539	737	.		-420.09	0.9907	1	0.28	0.09	0.15	1.56	0.44	0.65
tran	NA	NA	LTCONS_00041448	MTCONS_00046277	chr2	101887052	101891565	2443	-	chr21	20230060	20230802	743	.		-433.04	0.9564	1	0.28	0.09	0.15	82.81	66.84	67.16
tran	NA	NA	n369925	MTCONS_00047652	chr7	19039116	19040901	856	-	chr22	30771653	30783401	3679	-		-742.91	-0.8041	-1	0.36	0.32	0.25	4.58	5.95	6.02
tran	NA	NA	n366426	MTCONS_00000412	chr3	39018393	39020127	472	-	chr1	16010827	16067884	9024	+		-326.52	0.9919	1	0.08	0.45	0.23	0.93	1.24	1.09
tran	140020408	NA	n366426	MTCONS_00049876	chr3	39018393	39020127	472	-	chr3	179040534	179059936	10093	+		-536.51	-0.9676	-1	0.08	0.45	0.23	2.68	2.25	2.41
tran	NA	NA	n366426	MTCONS_00010680	chr3	39018393	39020127	472	-	chr10	104838242	104954942	14991	-		-465.14	0.91	1	0.08	0.45	0.23	0.45	0.59	0.56
tran	NA	NA	n374322	MTCONS_00017712	chr14	74939815	74941018	935	-	chr12	53900311	54019780	12002	-		-827.13	-0.9846	-0.866	0.04	0.03	0.03	0.83	1.53	1.4
cis_mRNA_up10k	9297	NA	n386569	MTCONS_00004613	chr1	32697262	32707311	2323	-	chr1	32681087	32687966	1311	-	*		0.9686	1	0.19	0.28	0.33	0.44	1.19	2.21
cis_mRNA_up10k	215	NA	LTCONS_00007595	MTCONS_00004710	chr1	36851726	36852778	1053	.	chr1	36805161	36851512	3700	-	*		-0.8736	-1	0.64	0.61	0.36	15.77	16.01	16.19

cis_mRNA_up10k	144	NA	n376228	MTCONS_00036975	chr19	56905116	56910541	1599	+	chr19	56891224	56904973	6027	-	*		-0.9626	-1	0.33	0.14	0	1.17	1.41	1.46
cis_mRNA_dw20k	10483	NA	n376228	MTCONS_00035019	chr19	56905116	56910541	1599	+	chr19	56879504	56894634	6776	+	*		-0.8438	-1	0.33	0.14	0	1.61	1.63	2.01
tran	NA	NA	n378313	MTCONS_00027624	chr9	103115360	103156151	2333	+	chr16	28385134	28415186	8839	-		-2952.01	-0.8888	-1	1.75	1.61	1.78	3.95	4.2	3.57
tran	NA	NA	n408247	MTCONS_00049876	chr19	45430060	45434643	631	+	chr3	179040534	179059936	10093	+		-634.77	-1	-1	0	0.16	0.1	2.68	2.25	2.41
cis_mRNA_dw20k	17411	NA	n408247	MTCONS_00034536	chr19	45430060	45434643	631	+	chr19	45409332	45412650	1647	+	*		0.8138	1	0	0.16	0.1	0.14	0.71	0.17
cis_mRNA_overlap	0	Lnc-AntiOverlap-mRNA	n379512	MTCONS_00024678	chr15	43029401	43034389	4047	+	chr15	43033648	43213023	9214	-	*		-0.6547	-0.866	0.03	0.02	0.07	9.3	9.74	9.3
cis_mRNA_dw20k	9948	NA	LTCONS_00049424	MTCONS_00049422	chr3	129035019	129045065	4173	+	chr3	128997684	129025072	2855	+	*		0.9595	1	0.8	0.83	0.74	5.19	5.24	4.31
tran	NA	NA	n405875	MTCONS_00035939	chr7	64363620	64391955	4414	+	chr19	23400420	23433451	7034	-		-1854.6	1	1	0.88	0.7	1.18	1.84	1.59	2.25
tran	NA	NA	n405875	MTCONS_00035911	chr7	64363620	64391955	4414	+	chr19	21987482	22034892	5837	-		-1493.96	0.8885	1	0.88	0.7	1.18	0.95	0.71	1.01
tran	NA	NA	n405875	MTCONS_00033981	chr7	64363620	64391955	4414	+	chr19	21711767	21723420	4770	+		-1596.47	0.9726	1	0.88	0.7	1.18	1.93	1.78	2.74
cis_mRNA_overlap	0	Lnc-AntiOverlap-mRNA	n340205	MTCONS_00054822	chr4	120409798	120473179	981	+	chr4	120420772	120549981	7405	-	*		0.8191	1	2.76	2.55	1.36	0.11	0.04	0.01
tran	NA	NA	n333962	MTCONS_00048261	chr6	4078218	4078499	282	-	chr3	10183319	10195354	5394	+		-456.71	0.9847	1	0	0.09	0.27	6.11	6.49	6.89
cis_mRNA_overlap	0	mRNA	LTCONS_00011091	MTCONS_00011090	chr11	695614	704197	5183	+	chr11	695520	704197	1787	+		-1348.61	0.9707	1	0.97	1.49	1.81	1.02	1.62	1.73
cis_mRNA_overlap	0	mRNA	n406811	MTCONS_00033329	chr19	1356323	1378430	4170	+	chr19	1354953	1378430	4239	+		-2752.71	-0.9721	-1	1.59	1.42	1.21	4.78	7.75	9.32
cis_mRNA_dw20k	12682	NA	n386207	MTCONS_00035875	chr19	19867183	19887222	2369	-	chr19	19899903	19932573	3778	-	*		-0.9753	-1	0.38	0.54	0.86	4.89	3.95	3.11
cis_mRNA_up10k	8605	NA	n335545	MTCONS_00018284	chr12	104359654	104373763	481	+	chr12	104341724	104351050	4327	-	*		-0.8618	-0.866	0.34	0.69	0	0.88	0.88	0.92
cis_mRNA_overlap	0	Lnc-AntiOverlap-mRNA	LTCONS_00027056	MTCONS_00028366	chr16	89753016	89762800	4465	+	chr16	89762671	89768502	2951	-	*		-0.9027	-1	0.17	0.12	0.09	1.1	1.19	1.49
cis_mRNA_dw20k	1024	NA	LTCONS_00010438	MTCONS_00008660	chr10	88998426	89102315	981	-	chr10	88983430	88997403	8066	+	*		-0.9366	-1	0.09	0.2	0	0.92	0.76	1.45
cis_mRNA_overlap	0	mRNA	n407269	MTCONS_00029016	chr17	28268623	28401643	4023	+	chr17	28256793	28435470	5092	+		-1627.07	0.9522	1	0.04	0.06	0.13	0.02	0.06	0.1
cis_mRNA_overlap	0	mRNA	n409184	MTCONS_00031751	chr17	73232694	73257707	4046	-	chr17	73232684	73257764	3814	-		-2263.54	0.9961	1	0.65	1.18	2.57	0.17	0.32	0.95
tran	NA	NA	n345591	MTCONS_00000412	chr3	72790082	72790652	571	+	chr1	16010827	16067884	9024	+		-545.7	0.9783	1	0	0.18	0.06	0.93	1.24	1.09
cis_mRNA_dw20k	49	NA	n339117	MTCONS_00042413	chr2	208467342	208470282	2937	+	chr2	208470330	208490126	5962	-	*		0.9538	0.866	0.05	0.18	0.09	1.77	1.78	1.77
tran	NA	NA	n323905	MTCONS_00052157	chrX	13908106	13916860	544	-	chr3	197299019	197354752	9196	-		-678.19	0.9838	1	1.01	1.53	1.4	1.79	2.76	2.36
tran	NA	NA	n385806	MTCONS_00035915	chrX	103367013	103369317	2305	-	chr19	22165269	22193783	5010	-		-1985.56	-0.9794	-1	0.77	1.17	1.35	2.78	1	0.77
cis_mRNA_dw20k	4007	NA	n375211	MTCONS_00025685	chr16	1041085	1043057	1611	-	chr16	1031808	1037079	4532	+	*		0.9163	0.866	0.02	0.04	0.02	1.34	2.01	1.61
cis_mRNA_overlap	0	Lnc-CompleteIn-mRNAIntron	n332930	MTCONS_00035496	chr19	9642966	9643488	523	-	chr19	9636407	9649387	4891	-	*		0.9865	1	0.36	1.16	0.63	5.91	6.4	6
tran	NA	NA	n332930	MTCONS_00051716	chr19	9642966	9643488	523	-	chr3	155538816	155572410	9728	-		-460.28	0.915	1	0.36	1.16	0.63	2.4	2.77	2.66
tran	106776733	NA	n377955	MTCONS_00039148	chr2	61370690	61372087	892	-	chr2	168148819	168730621	6608	+		-860.6	-0.9909	-1	0.44	0.18	0.12	10.67	15.23	15.46
cis_mRNA_overlap	0	mRNA	LTCONS_00020130	MTCONS_00020125	chr13	44354324	44361106	6300	-	chr13	43787553	44363950	5952	-	*		0.9283	1	5.89	8.24	5.76	0.98	1.19	0.83
tran	41172	NA	n408025	MTCONS_00047097	chr22	42896585	42908566	1357	+	chr22	42949737	42970388	2668	+		-1020.38	0.9778	1	0	0.37	0.67	0.05	0.19	0.43
tran	NA	NA	LTCONS_00024227	MTCONS_00012720	chr15	90620771	90621146	376	+	chr11	113775380	113821262	6005	+		-286.73	0.9922	1	0.1	0.19	0.46	0.67	0.89	2.39
cis_mRNA_dw20k	10375	NA	n382921	MTCONS_00027411	chr16	11536047	11585611	3552	-	chr16	11595985	11621105	1101	-	*		0.976	1	0.16	0.79	0.21	0.03	0.81	0.26
cis_mRNA_up10k	1070	NA	n339759	MTCONS_00043963	chr20	62605467	62611362	2070	-	chr20	62612431	62671432	9725	+	*		0.9993	1	0.53	1	0.75	0.9	1.54	1.22
cis_mRNA_overlap	0	Lnc-AntiOverlap-mRNA	n339259	MTCONS_00050366	chr3	15087058	15090778	3721	+	chr3	15088723	15106818	3462	-	*		-0.9391	-1	1.01	1.35	0.46	8.35	6.53	9.17
cis_mRNA_up10k	414	NA	LTCONS_00047006	MTCONS_00047009	chr22	39436656	39439431	817	+	chr22	39439844	39451977	5537	+	*		0.891	1	2.07	2.31	2.25	0.47	0.56	0.5
tran	NA	NA	n346227	MTCONS_00042413	chr7	45822293	45828413	548	+	chr2	208470330	208490126	5962	-		-605.28	0.6286	0.866	0	0.18	0.15	1.77	1.78	1.77
cis_mRNA_up10k	922	NA	n375824	MTCONS_00030249	chr17	3705458	3707099	942	-	chr17	3617919	3704537	3765	-	*		0.8921	1	2.26	2.81	2.31	1.88	2.09	1.99
tran	NA	NA	n325002	MTCONS_00051716	chr20	31249676	31256636	570	+	chr3	155538816	155572410	9728	-		-596.52	-0.7293	-0.866	0.06	0	0.06	2.4	2.77	2.66
tran	NA	NA	n407372	MTCONS_00006433	chr12	66151801	66220754	1143	-	chr1	160236583	160237032	450	-		-271.96	-0.9488	-0.866	0.03	0.03	0	0	0.1	0.31
tran	NA	NA	n407372	MTCONS_00020697	chr12	66151801	66220754	1143	-	chr13	21535411	21535878	468	.		-293.43	0.7924	0.866	0.03	0.03	0	3.35	1.51	0.36
tran	NA	NA	n383557	MTCONS_00042413	chr19	55775355	55775745	391	-	chr2	208470330	208490126	5962	-		-555.32	0.9551	0.866	2.07	2.62	1.83	1.77	1.78	1.77
cis_mRNA_overlap	0	Lnc-CompleteIn-mRNAIntron	n383557	MTCONS_00036944	chr19	55775355	55775745	391	-	chr19	55773591	55791751	1904	-	*		0.9644	1	2.07	2.62	1.83	1.84	3.88	1.76
tran	NA	NA	n324705	MTCONS_00041449	chr22	22184681	22185789	477	+	chr2	101887052	101925178	6597	-		-332.17	0.9811	1	0.06	0.29	0.25	1.64	2.09	1.93
cis_mRNA_dw20k	9087	NA	n338698	MTCONS_00034504	chr19	44750631	44752527	1881	-	chr19	44716691	44741545	3108	+	*		0.8595	1	0.2	0.32	0.27	2.02	3.11	2.12
tran	NA	NA	n342792	MTCONS_00019478	chr11	125743348	125744423	1076	-	chr13	78271833	78345587	10371	+		-1080.59	1	1	0.03	0	0.06	1.68	1.6	1.76
cis_mRNA_overlap	0	Lnc-AntiOverlap-mRNA	n340788	MTCONS_00057334	chr5	180649869	180657685	2343	-	chr5	180650297	180663902	4141	+		-3957.97	0.9663	0.866	0.03	0.03	0.04	0.4	0.44	0.55
tran	NA	NA	LTCONS_00042561	MTCONS_00018225	chr2	223745026	223807923	648	-	chr12	99018030	99038644	2859	-		-653.23	-0.993	-1	1.57	1.18	0.52	0.49	0.87	1.29
cis_mRNA_overlap	0	Lnc-CompleteIn-mRNAIntron	n325909	MTCONS_00010513	chr10	96891672	96918172	421	-	chr10	96796299	96990901	2876	-	*		-0.9744	-0.866	0.09	0.08	0.08	1.2	1.71	1.59
cis_mRNA_up10k	369	NA	n379089	MTCONS_00002789	chr1	173835354	173837125	469	-	chr1	173837493	173872659	19182	+	*		0.8752	1	0	0.46	0.47	3.47	3.75	4.03
cis_mRNA_overlap	0	Lnc-CompleteIn-mRNAIntron	n342658	MTCONS_00055997	chr5	65264659	65268220	3561	+	chr5	65221180	65377350	8764	+	*		-0.963	-1	0.03	0.08	0.05	2.06	1.65	2
cis_mRNA_up10k	70	NA	n345467	MTCONS_00006456	chr1	160991202	161003036	2187	-	chr1	160969200	160991133	1302	-	*		-0.9992	-1	0.03	0.2	0.06	0.37	0	0.32
tran	NA	NA	n367739	MTCONS_00028044	chr5	45890318	45896072	563	+	chr16	70253448	70277714	4770	-		-387.25	-0.9528	-1	0.2	0	0.32	3.47	3.84	2.8
tran	NA	NA	n367739	MTCONS_00027624	chr5	45890318	45896072	563	+	chr16	28385134	28415186	8839	-		-937.44	-0.9659	-1	0.2	0	0.32	3.95	4.2	3.57
cis_mRNA_up10k	91	NA	LTCONS_00052240	MTCONS_00051164	chr3	105587983	105589603	1621	.	chr3	105374315	105587893	6468	-	*		0.9658	1	0.51	0.62	0.97	2.3	2.57	2.87
tran	NA	NA	n368295	MTCONS_00002740	chr5	67482644	67485177	924	-	chr1	169337194	169362826	8046	+		-463.52	0.9396	1	0.43	0.55	0.3	1.94	2.4	1.82
tran	NA	NA	n366571	MTCONS_00034837	chr3	14266657	14272219	1226	+	chr19	52872319	52925184	17190	+		-1046.87	0.9985	1	0.1	0.16	0	2.75	2.91	2.42
cis_mRNA_up10k	3485	NA	n378993	MTCONS_00002789	chr1	173833040	173834009	621	-	chr1	173837493	173872659	19182	+	*		0.9993	1	0	0.15	0.32	3.47	3.75	4.03
tran	NA	NA	n409402	MTCONS_00032058	chr17	44450179	44500463	587	+	chr17_ctg5_hap1	266173	381069	2068	-		-312.48	-0.8299	-1	0	1.9	3.33	0.75	0.74	0.08
cis_mRNA_up10k	767	NA	n345468	MTCONS_00006467	chr1	161102698	161109613	2822	+	chr1	161090673	161101932	3455	-	*		0.8589	1	0.13	0.19	0.06	1.76	2.6	1.73
cis_mRNA_dw20k	2324	NA	n379227	MTCONS_00028789	chr17	16342640	16367300	689	+	chr17	16318851	16340317	2810	+	*		-0.9689	-1	0.21	0.33	0	0.4	0	3.14
tran	NA	NA	n363424	MTCONS_00043963	chr1	67995313	67998012	408	+	chr20	62612431	62671432	9725	+		-315.63	-0.9707	-1	0.28	0	0.08	0.9	1.54	1.22
tran	NA	NA	n363424	MTCONS_00042413	chr1	67995313	67998012	408	+	chr2	208470330	208490126	5962	-		-371.44	-0.7206	-0.866	0.28	0	0.08	1.77	1.78	1.77
cis_mRNA_dw20k	7024	NA	n383371	MTCONS_00033690	chr19	12098432	12138553	1801	+	chr19	12075841	12091409	2853	+	*		-0.9305	-1	0.77	1.18	0.61	1.37	0.93	2.06

cis_mRNA_overlap	0	Lnc-AntiOverlap-mRNA	n383371	MTCONS_00035585	chr19	12098432	12138553	1801	+	chr19	12124713	12146826	3432	-	*		-0.9933	-1	0.77	1.18	0.61	0.49	0.41	0.54
tran	NA	NA	n338798	MTCONS_00024133	chr2	42487062	42487735	674	+	chr15	83490056	83503824	3232	+		-436.25	0.9986	1	0.27	0.74	0.82	0.18	0.38	0.4
cis_mRNA_up10k	235	NA	LTCONS_00015741	MTCONS_00017658	chr12	52242795	52245504	2710	+	chr12	52211670	52242561	4777	-	*		0.9943	1	1.04	2.1	1.41	10.12	11.51	10.47
cis_mRNA_dw20k	470	NA	n409326	MTCONS_00031929	chr17	79860777	79869340	3263	-	chr17	79869809	79876058	4407	-	*		0.6557	1	0.14	0.86	0.24	1.37	1.94	1.9
tran	NA	NA	n378468	MTCONS_00039172	chr7	124569952	124819369	2147	+	chr2	170438918	170499115	9908	+		-1534.85	-0.9449	-0.866	0.04	0.03	0.06	2.11	2.11	1.81
cis_mRNA_overlap	0	Lnc-CompleteIn-mRNAIntron	n333913	MTCONS_00057977	chr5	70528927	70530059	388	-	chr5	70328491	70585523	15856	-	*		-1	-1	0.22	0	0.23	3.97	4.43	3.95
cis_mRNA_overlap	0	Lnc-CompleteIn-mRNAExon	n408075	MTCONS_00031402	chr17	53029259	53046064	1128	-	chr17	53029251	53046064	2725	-		-515.48	0.9847	1	1.19	4.1	2.26	4.9	6.45	5.22
cis_mRNA_overlap	0	mRNA	n333026	MTCONS_00043639	chr20	47855465	47860610	865	+	chr20	47835832	47860620	3762	+		-500.6	0.9761	1	0	0.04	0.09	4.33	6.3	7.43
cis_mRNA_dw20k	4446	NA	n340791	MTCONS_00029700	chr17	61778207	61780030	1824	+	chr17	61699734	61773762	4880	+	*		-0.9776	-1	4.65	4.46	4.72	2.61	3.43	2.56
cis_mRNA_dw20k	10761	NA	n407987	MTCONS_00028368	chr16	89753076	89762772	1591	+	chr16	89773532	89787468	2832	-	*		0.9714	1	0.69	0	0.66	3.02	1.96	2.72
cis_mRNA_up10k	5959	NA	n344849	MTCONS_00056566	chr5	127412451	127413364	781	+	chr5	127419322	127525380	6973	+	*		0.8064	1	0.05	0.04	0	9.74	8.85	8.58
cis_mRNA_dw20k	11403	NA	n339968	MTCONS_00054270	chr4	42097864	42101378	1683	+	chr4	42112780	42154896	11259	-	*		-0.9502	-1	0.99	0.69	1.46	2.15	4.49	0.98
cis_mRNA_dw20k	4928	NA	n333099	MTCONS_00034536	chr19	45417577	45418151	205	+	chr19	45409332	45412650	1647	+	*		-0.9915	-1	0.87	0.39	0.78	0.14	0.71	0.17
cis_mRNA_dw20k	6442	NA	n340559	MTCONS_00028886	chr17	19246488	19281469	1322	-	chr17	19140650	19240047	4705	+	*		-0.9895	-1	0.55	0.46	0.17	0.95	1.74	3.09
cis_mRNA_up10k	306	NA	n340559	MTCONS_00028896	chr17	19246488	19281469	1322	-	chr17	19281774	19286936	3283	+	*		-0.9998	-1	0.55	0.46	0.17	1.07	1.17	1.53
tran	NA	NA	n410533	MTCONS_00041776	chr17	79476997	79479892	1837	-	chr2	130824979	130886954	10720	-		-1153.82	-0.9916	-1	7.19	8.75	17.69	5.02	3.85	0.74
tran	NA	NA	n377991	MTCONS_00050279	chr1	144284105	144311653	769	-	chr3	9878109	9885702	4133	-		-755.96	-0.7593	-1	0.6	0.37	0.33	3.88	4	4.45
cis_mRNA_dw20k	11465	NA	LTCONS_00044714	MTCONS_00044715	chr20	45103147	45113352	414	-	chr20	45124816	45142194	3877	-	*		-0.9635	-0.866	0.46	1.01	0.25	0.96	0.91	0.96
cis_mRNA_up10k	6756	NA	n341085	MTCONS_00012487	chr11	82896841	82898078	1238	-	chr11	82904833	82926321	4093	+	*		0.8303	1	0.73	1	0.78	1.11	1.6	1.46
cis_mRNA_dw20k	1985	NA	n411737	MTCONS_00028789	chr17	16342301	16373962	578	+	chr17	16318851	16340317	2810	+	*		0.9544	1	0.16	0	0.39	0.4	0	3.14
tran	NA	NA	LTCONS_00073834	MTCONS_00009847	chr9	66667681	66668398	718	.	chr10	42825274	42863493	8645	-		-506.61	0.6359	1	1.23	0.47	1.37	0.13	0.09	1.8
cis_mRNA_dw20k	31	NA	n375549	MTCONS_00030211	chr17	2591628	2592285	473	+	chr17	2592315	2615083	5748	-	*		0.9978	1	0.4	0.43	0.5	1.98	2.13	2.61
cis_mRNA_overlap	0	Lnc-CompleteIn-mRNAIntron	n339814	MTCONS_00032839	chr18	22913212	22914068	856	-	chr18	22641851	22932214	6065	-	*		-0.724	-0.866	0.04	0.04	0.07	0.76	1.2	0.58
tran	NA	NA	LTCONS_00042262	MTCONS_00022023	chr2	190787931	190789605	1675	-	chr14	21665762	21737638	14746	-		-3303.86	0.715	1	0	0.02	0.11	19.39	20.27	20.38
cis_mRNA_up10k	3410	NA	LTCONS_00044955	MTCONS_00043885	chr20	61444454	61445005	312	-	chr20	61448414	61472511	2482	+	*		0.8673	1	0.34	0.75	0.28	0.03	0.05	0
tran	NA	NA	n345360	MTCONS_00042413	chr18	45483431	45483898	468	-	chr2	208470330	208490126	5962	-		-566.58	0.9026	0.866	2.61	3.36	2.04	1.77	1.78	1.77
cis_mRNA_dw20k	327	NA	n408146	MTCONS_00002579	chr1	160185505	160232318	4183	-	chr1	160175113	160185179	2498	+	*		-0.9453	-1	4.09	4.42	4.59	1.33	1.16	0.87
cis_mRNA_dw20k	4266	NA	n408146	MTCONS_00006433	chr1	160185505	160232318	4183	-	chr1	160236583	160237032	450	-	*		0.9269	1	4.09	4.42	4.59	0	0.1	0.31
tran	NA	NA	n326676	MTCONS_00042413	chr1	22224614	22230303	1054	+	chr2	208470330	208490126	5962	-		-832.62	0.9912	0.866	0.08	0.29	0.11	1.77	1.78	1.77
cis_mRNA_overlap	0	mRNA	LTCONS_00033969	MTCONS_00033970	chr19	21579905	21593286	10335	+	chr19	21579919	21601152	5262	+	*		0.95	1	2.48	1.58	1.82	1.72	0.55	1.19
tran	2743586	NA	LTCONS_00005931	MTCONS_00002104	chr1	147574207	147578821	3331	-	chr1	144593657	144830622	7756	+		-1679.97	0.9999	1	3.48	5.27	4.36	7.98	8.75	8.37
cis_mRNA_up10k	275	NA	LTCONS_00026253	MTCONS_00027665	chr16	29910854	29917456	2040	+	chr16	29882379	29910580	3569	-	*		-0.9035	-1	2.15	1.84	3.06	23.91	31.95	19.49
cis_mRNA_overlap	0	Lnc-AntiOverlap-mRNA	LTCONS_00031121	MTCONS_00029308	chr17	40905947	40913275	3136	-	chr17	40912939	40915644	1297	+	*		-0.9926	-1	0.93	0	1.26	0.31	1.26	0.15
tran	NA	NA	n326057	MTCONS_00031911	chr10	11535951	11537392	503	+	chr17	79641591	79650954	7840	-		-319.91	0.9835	1	6.64	11.51	5.64	3.65	3.94	3.5
cis_mRNA_overlap	0	other	LTCONS_00025126	MTCONS_00024006	chr15	75192325	75199542	3545	-	chr15	75182396	75192325	4959	+	*		-0.9972	-1	16	15.22	15.31	1.66	1.95	1.94
tran	NA	NA	n345815	MTCONS_00033841	chr8	144714038	144715688	666	-	chr19	17325517	17330638	3216	+		-480.5	0.9893	1	0.27	0.45	0.34	2.25	2.56	2.41
cis_mRNA_overlap	0	Lnc-AntiOverlap-mRNA	n406840	MTCONS_00000422	chr1	16160710	16174642	2732	-	chr1	16174350	16268355	14900	+	*		0.7766	1	0.3	0.22	0.03	1.82	1.48	1.45
tran	NA	NA	n407952	MTCONS_00035911	chr7	64254771	64294025	2376	+	chr19	21987482	22034892	5837	-		-821.61	-0.8561	-1	1.83	2.25	0.92	0.95	0.71	1.01
cis_mRNA_up10k	99	NA	LTCONS_00004648	MTCONS_00004642	chr1	33336512	33338132	1480	-	chr1	33327867	33336414	3098	-	*		0.96	1	12.4	11.89	12.35	11.96	8.05	10.52
tran	NA	NA	n371340	MTCONS_00042413	chrX	47157251	47158120	477	+	chr2	208470330	208490126	5962	-		-591.55	0.9731	0.866	4.14	5.91	3.58	1.77	1.78	1.77
cis_mRNA_overlap	0	Lnc-AntiOverlap-mRNA	n406992	MTCONS_00024776	chr15	50646371	50650503	4133	+	chr15	50569389	50647605	3040	-	*		-0.6755	-1	1.72	1.76	1.31	1.16	0.93	1.2
cis_mRNA_dw20k	8	NA	n345936	MTCONS_00035875	chr19	19887452	19899896	1019	+	chr19	19899903	19932573	3778	-	*		0.9988	1	0.17	0.09	0.03	4.89	3.95	3.11
cis_mRNA_overlap	0	Lnc-CompleteIn-mRNAExon	n339003	MTCONS_00039172	chr2	170495992	170497913	1922	+	chr2	170438918	170499115	9908	+		-782.02	0.9992	0.866	2.26	2.24	1.83	2.11	2.11	1.81
cis_mRNA_overlap	0	Lnc-AntiOverlap-mRNA	n377982	MTCONS_00002579	chr1	160171989	160178659	1578	-	chr1	160175113	160185179	2498	+	*		-0.9709	-1	0.13	0.14	0.2	1.33	1.16	0.87
cis_mRNA_dw20k	16063	NA	LTCONS_00041771	MTCONS_00041776	chr2	130782206	130808917	9033	-	chr2	130824979	130886954	10720	-	*		0.9809	1	2.61	2.47	0.86	5.02	3.85	0.74
tran	9598207	NA	n377683	MTCONS_00024535	chr15	23190864	23207967	1960	-	chr15	32806173	32825942	1395	-		-579.16	0.8585	0.866	0.25	0.25	0.49	0.2	0	0.39
cis_mRNA_dw20k	1878	NA	n342431	MTCONS_00038402	chr2	96080756	96082345	1590	+	chr2	96068448	96078879	1399	+	*		0.8746	1	0.59	0.99	0.83	3.12	4.24	3.28
tran	4963329	NA	LTCONS_00023161	MTCONS_00024498	chr15	23388420	23392858	747	+	chr15	28356186	28567118	15767	-		-540.17	-0.9706	-1	1.97	1.63	2.25	2.35	2.58	2.28
cis_mRNA_dw20k	16582	NA	n377958	MTCONS_00046485	chr22	21822387	21824214	763	+	chr22	21771672	21805806	6774	+	*		-0.9981	-1	0.98	1.61	1.32	2.04	0.93	1.5
tran	NA	NA	n339477	MTCONS_00028172	chr3	123329581	123330850	1270	-	chr16	75626950	75657267	9661	-		-594.82	0.9762	0.866	0.03	0.05	0.05	3.3	3.52	3.47
cis_mRNA_dw20k	294	NA	n339477	MTCONS_00051372	chr3	123329581	123330850	1270	-	chr3	123331143	123554784	7750	-	*		-0.6682	-0.866	0.03	0.05	0.05	0.46	0.41	0.23
cis_mRNA_up10k	3432	NA	LTCONS_00027248	MTCONS_00027247	chr16	2692410	2723440	2293	-	chr16	2658107	2688979	2174	-	*		-0.9583	-1	1.78	2.21	3.05	0.2	0.19	0.02
cis_mRNA_overlap	0	AntiCompleteIn-mRNAIntron	n340662	MTCONS_00056787	chr5	140479829	140481794	581	-	chr5	140474163	140483601	3586	+	*		0.9899	1	0.06	0	0.17	3.76	3.72	3.9
cis_mRNA_up10k	113	NA	n342563	MTCONS_00050144	chr3	195382448	195384798	1672	-	chr3	195384910	195416775	3199	+	*		0.9509	1	0.58	0.4	0.33	4.53	3.56	2.34
cis_mRNA_up10k	6168	NA	n340778	MTCONS_00059046	chr5	179505357	179508661	3305	-	chr5	179338378	179499190	10687	-	*		0.9286	0.866	0.11	0.08	0.11	13.62	12.58	13.23
cis_mRNA_overlap	0	mRNA	LTCONS_00035214	MTCONS_00035211	chr19	1231625	1237990	4984	-	chr19	1228431	1238060	3192	-	*		-0.9969	-1	1.96	2.22	1.94	5.55	5.11	5.63
cis_mRNA_up10k	101	NA	n407660	MTCONS_00045578	chr21	46354641	46359828	1100	-	chr21	46359928	46398319	2294	+	*		0.9997	1	0.28	0.23	0.12	4.25	4.19	4.07
tran	179511356	NA	n364897	MTCONS_00042413	chr2	28945306	28958975	723	-	chr2	208470330	208490126	5962	-		-624.75	0.9707	0.866	0	0.16	0.04	1.77	1.78	1.77
tran	NA	NA	n406498	MTCONS_00015507	chr2	114356605	114359153	2164	-	chr12	31226779	31257730	4327	+		-1621.12	0.9904	1	0.48	0.59	0.77	1.63	1.95	2.89
tran	28479217	NA	n381199	MTCONS_00005969	chr1	120906034	120914842	515	+	chr1	149394058	149400543	5522	-		-301.14	0.9732	1	0	0.09	0.22	0.37	0.62	1.64
cis_mRNA_overlap	0	Lnc-CompleteIn-mRNAIntron	n377693	MTCONS_00018287	chr12	104424522	104426026	1389	-	chr12	104382671	104458195	2062	-	*		-1	-1	0.22	0.15	0.1	0.8	1.03	1.19
cis_mRNA_up10k	40	NA	n377939	MTCONS_00039278	chr2	177040722	177053268	984	-	chr2	177053307	177055635	1975	+	*		0.6686	1	0.24	0.52	0.19	3.31	3.54	0.49

cis_mRNA_dw20k	1985	NA	n407707	MTCONS_00028789	chr17	16342301	16373962	1191	+	chr17	16318851	16340317	2810	+	*		-0.984	-1	0.22	0.25	0.15	0.4	0	3.14
cis_mRNA_up10k	495	NA	LTCONS_00048842	MTCONS_00050853	chr3	52444527	52450253	2642	+	chr3	52435023	52444033	4062	-	*		0.9907	1	0	1.07	0.61	3.37	4.3	3.79
cis_mRNA_overlap	0	mRNA	n326279	MTCONS_00006862	chr1	197135770	197173181	756	-	chr1	197122731	197170445	8698	-	*		0.6818	1	0.27	0.35	0.64	10.82	11.69	11.71
tran	190839726	NA	n326279	MTCONS_00004052	chr1	197135770	197173181	756	-	chr1	6281253	6296045	4923	-		-569.85	0.815	1	0.27	0.35	0.64	6.45	7.45	7.75
cis_mRNA_overlap	0	mRNA	n332622	MTCONS_00013249	chr11	3752572	3765871	502	-	chr11	3719273	3819022	5445	-		-280.99	-0.9197	-1	0.09	0	0.08	2.11	2.42	2.26
tran	NA	NA	n334389	MTCONS_00012837	chr6	4078287	4078486	200	-	chr11	119019750	119034182	8465	+		-160.21	0.9392	1	1.21	2.81	1.86	1.29	1.64	1.54
cis_mRNA_overlap	0	Lnc-CompleteIn-mRNAExon	n338836	MTCONS_00040816	chr2	53889616	53891963	2347	-	chr2	53889562	54014214	9905	-		-1104.02	0.8937	1	1.78	2.72	1.7	5.36	6.05	4.64
tran	NA	NA	n370197	MTCONS_00041449	chr8	129571390	129573380	807	-	chr2	101887052	101925178	6597	-		-560.48	0.8833	1	0.26	0.64	0.34	1.64	2.09	1.93
tran	NA	NA	n338541	MTCONS_00051825	chr1	226257217	226257654	438	+	chr3	169805368	169899537	15563	-		-511.46	-0.9869	-1	2.16	2.85	2.37	1.16	0.96	1.13
tran	NA	NA	n338541	MTCONS_00015928	chr1	226257217	226257654	438	+	chr12	56914542	56989980	8281	+		-581.88	0.8443	1	2.16	2.85	2.37	0.72	1.14	1.06
cis_mRNA_up10k	230	NA	n407174	MTCONS_00013821	chr11	57479995	57508445	1792	+	chr11	57468662	57479766	3698	-	*		-0.9973	-1	6.05	6.85	5.54	2.67	2.19	2.9
cis_mRNA_overlap	0	Lnc-AntiOverlap-mRNA	LTCONS_00004224	MTCONS_00000441	chr1	16786595	16971217	25174	-	chr1	16767167	16788102	3716	+		-3159.7	-0.9498	-1	1.4	2.05	1.69	0.59	0.46	0.57
cis_mRNA_dw20k	4048	NA	n408251	MTCONS_00047449	chr22	21921957	21978323	2811	+	chr22	21982370	21985086	2108	-	*		0.9114	1	1.82	1.53	1.7	2.14	1.42	1.57
tran	NA	NA	n377252	MTCONS_00039883	chr21	47395659	47397653	1277	-	chr2	232063206	232241727	8428	+		-814.3	0.999	1	0.42	0.41	0.49	3.05	3	3.65
cis_mRNA_overlap	0	Lnc-AntiOverlap-mRNA	n378962	MTCONS_00047604	chr22	28315417	28393763	562	+	chr22	28371171	28839650	14880	-	*		0.9159	1	0.19	0.12	0	5.49	4.86	4.63
cis_mRNA_up10k	97	NA	LTCONS_00055377	MTCONS_00052382	chr4	2060867	2061096	230	.	chr4	2061192	2070816	7028	+	*		0.913	1	0.19	0.85	1.51	0.51	1.06	1.13
tran	NA	NA	n379792	MTCONS_00018846	chr16	28814097	28829149	1930	+	chr12	133494970	133532981	9230	-		-2045.55	0.8933	1	0.97	1.71	1.17	1.87	2.09	2.02
tran	NA	NA	n379792	MTCONS_00033841	chr16	28814097	28829149	1930	+	chr19	17325517	17330638	3216	+		-1730.84	0.9616	1	0.97	1.71	1.17	2.25	2.56	2.41
tran	NA	NA	n379792	MTCONS_00002255	chr16	28814097	28829149	1930	+	chr1	150266269	150283205	2889	+		-1397.32	0.981	1	0.97	1.71	1.17	9.78	10.32	10.02
cis_mRNA_overlap	0	AntiCompleteIn-mRNAIntron	LTCONS_00000895	MTCONS_00004713	chr1	36901423	36910703	634	+	chr1	36883460	36916086	1566	-	*		-0.9946	-1	0.36	1.55	0	1.61	1.14	1.84
cis_mRNA_dw20k	122	NA	n338109	MTCONS_00001821	chr1	108025756	108027521	1766	+	chr1	107682820	108025635	5024	+	*		0.9984	1	0.16	0.2	0.42	1.17	1.26	2.12
cis_mRNA_overlap	0	mRNA	n339642	MTCONS_00049651	chr3	150181245	150184205	2959	+	chr3	150126689	150184998	11162	+		-1134.7	0.9425	1	1.77	2.38	1.96	2.64	3.23	3
tran	NA	NA	n325733	MTCONS_00035915	chr13	48042776	48045454	413	-	chr19	22165269	22193783	5010	-		-203.81	-0.9135	-1	0.19	0.25	0.31	2.78	1	0.77
tran	235694	NA	n387349	MTCONS_00057977	chr5	69170991	70092798	1771	+	chr5	70328491	70585523	15856	-		-1448.62	0.9297	1	2.84	3.09	2.67	3.97	4.43	3.95
cis_mRNA_overlap	0	mRNA-AntiCompleteIn-LncIntron	n387349	MTCONS_00057969	chr5	69170991	70092798	1771	+	chr5	69770779	69881984	8211	-		-1211.69	0.998	1	2.84	3.09	2.67	3.85	4.56	3.47
cis_mRNA_overlap	0	Lnc-AntiOverlap-mRNA	n407110	MTCONS_00028090	chr16	71879899	71919174	483	+	chr16	71893582	71919632	4044	-	*		0.8531	1	0.08	0	0.07	1.73	1.28	1.45
cis_mRNA_overlap	0	Lnc-AntiOverlap-mRNA	n410042	MTCONS_00028368	chr16	89778264	89784573	1753	+	chr16	89773532	89787468	2832	-	*		-0.97	-1	0.02	0.2	0.11	3.02	1.96	2.72
cis_mRNA_up10k	5776	NA	n409745	MTCONS_00034327	chr19	39369195	39384795	1995	-	chr19	39390570	39399534	2307	+	*		-0.9967	-1	6.71	7.42	11.08	3.18	3.05	2.64
cis_mRNA_up10k	5546	NA	LTCONS_00018919	MTCONS_00018921	chr13	20437985	20527265	1970	+	chr13	20532810	20665968	10493	+	*		0.9396	1	1.29	1.81	1.77	1.9	2.83	2.45
cis_mRNA_up10k	212	NA	n406526	MTCONS_00047774	chr22	37415702	37425863	1614	+	chr22	37406905	37415491	1775	-	*		0.9621	0.866	0.34	0.3	0.3	1.68	1.22	1.04
cis_mRNA_overlap	0	Lnc-CompleteIn-mRNAIntron	n324870	MTCONS_00045141	chr21	22392807	22410718	726	+	chr21	22370623	22863909	9470	+	*		0.93	1	0.1	0.05	0.09	16.17	8.24	11.91
cis_mRNA_overlap	0	mRNA	LTCONS_00047540	MTCONS_00047541	chr22	24981523	24989035	2657	-	chr22	24981523	24989035	2890	-		-2029.88	0.9998	1	6.28	6.61	6.62	2.04	2.79	2.83
cis_mRNA_overlap	0	Lnc-CompleteIn-mRNAIntron	n342218	MTCONS_00000290	chr1	10026764	10027014	251	+	chr1	10002829	10045765	4157	+	*		0.7375	1	0.17	0.15	0	1.15	0.73	0.62
cis_mRNA_overlap	0	Lnc-AntiOverlap-mRNA	LTCONS_00031188	MTCONS_00029359	chr17	42376944	42392683	5264	-	chr17	42385758	42396042	2205	+	*		-0.7471	-1	0	0.09	0.08	2.18	0.86	1.88
tran	NA	NA	n386278	MTCONS_00049362	chr20	47551778	47552227	450	+	chr3	124449213	124470145	8967	+		-351.29	0.9048	1	14.52	19.63	18.48	3.28	3.93	3.53
tran	NA	NA	n386278	MTCONS_00039494	chr20	47551778	47552227	450	+	chr2	201980877	202038063	11977	+		-340.19	0.9488	1	14.52	19.63	18.48	0.42	0.83	0.62
tran	NA	NA	n386278	MTCONS_00004585	chr20	47551778	47552227	450	+	chr1	31648621	31712835	6994	-		-345.5	0.9615	1	14.52	19.63	18.48	5.43	6.82	6.16
cis_mRNA_overlap	0	mRNA	n346011	MTCONS_00011411	chr11	17373294	17396117	4189	+	chr11	17373749	17405078	12680	+		-2327.51	0.9361	1	1.49	2.17	1.21	6.51	7.57	4.87
cis_mRNA_up10k	8706	NA	n345908	MTCONS_00040051	chr2	242486062	242489482	617	-	chr2	242498187	242513610	2582	+	*		-0.9969	-0.866	0.29	0.05	0.05	1.27	1.9	1.96
tran	NA	NA	n335788	MTCONS_00007912	chr1	205032329	205032757	429	+	chr10	15139117	15147770	4236	+		-432.58	0.9347	1	0	0.24	0.32	0.57	0.6	0.64
cis_mRNA_dw20k	4593	NA	n375299	MTCONS_00028172	chr16	75614470	75622358	5914	-	chr16	75626950	75657267	9661	-	*		0.803	1	0.3	0.35	0.31	3.3	3.52	3.47
tran	NA	NA	n373943	MTCONS_00042413	chr13	100738209	100741196	1083	-	chr2	208470330	208490126	5962	-		-824.07	-0.6225	-0.866	0.32	0.15	1.22	1.77	1.78	1.77
tran	NA	NA	n325930	MTCONS_00018085	chr10	85661390	85662658	309	-	chr12	86354730	86651753	21531	-		-161.27	-0.9066	-1	1.43	2.67	1.16	8.52	6.12	11.7
tran	NA	NA	n323816	MTCONS_00049876	chrX	119028703	119033529	772	-	chr3	179040534	179059936	10093	+		-842.66	-0.9575	-1	4.14	5.32	5.21	2.68	2.25	2.41
tran	NA	NA	n384175	MTCONS_00018846	chr22	41581221	41593505	1375	-	chr12	133494970	133532981	9230	-		-1689.13	0.9358	1	3	3.71	3.26	1.87	2.09	2.02
cis_mRNA_dw20k	14555	NA	n378666	MTCONS_00039296	chr2	179278714	179283850	740	+	chr2	179059208	179264160	7259	+	*		0.982	1	0.4	0.49	0.53	0.25	0.46	0.49
cis_mRNA_up10k	7	NA	n406456	MTCONS_00030266	chr17	4046462	4060991	1306	+	chr17	3907735	4046456	11605	-	*		0.7559	1	18.38	19.41	18.11	6.88	7.09	5.65
cis_mRNA_dw20k	44	NA	n366694	MTCONS_00048604	chr3	43392792	43394060	1053	-	chr3	43328004	43392749	5380	+	*		-0.9949	-1	0.47	0.52	0.42	1.86	1.27	2.7
cis_mRNA_dw20k	6007	NA	n338503	MTCONS_00003247	chr1	207974867	207977425	2559	-	chr1	207925383	207968861	6335	+	*		-0.8086	-1	0.85	0.73	0.91	26.42	27.54	15.56
cis_mRNA_up10k	218	NA	n380376	MTCONS_00014359	chr11	82783164	82784236	597	+	chr11	82683907	82782947	10135	-	*		0.9054	1	2.16	3.22	3.08	7.95	8.89	8.39
tran	NA	NA	n345097	MTCONS_00022023	chrX	109085564	109158741	863	+	chr14	21665762	21737638	14746	-		-593.85	-0.9874	-1	0.34	0.12	0.04	19.39	20.27	20.38
tran	NA	NA	n337216	MTCONS_00038904	chr7	5568087	5568833	305	-	chr2	131968620	132022433	3376	+		-225.24	0.9641	1	0.53	0	0.25	1.74	0.31	0.64
tran	122367368	NA	LTCONS_00002101	MTCONS_00000547	chr1	144178760	144219394	270	+	chr1	21794885	21811393	3477	+		-166.17	-0.7279	-1	0.77	2.84	0.69	0.9	0.55	1.87
tran	NA	NA	n387246	MTCONS_00049362	chr22	24712379	24713044	488	-	chr3	124449213	124470145	8967	+		-1049.4	0.917	1	4.72	6.46	6.02	3.28	3.93	3.53
tran	NA	NA	n387246	MTCONS_00039494	chr22	24712379	24713044	488	-	chr2	201980877	202038063	11977	+		-970.4	0.9577	1	4.72	6.46	6.02	0.42	0.83	0.62
tran	NA	NA	n387246	MTCONS_00004585	chr22	24712379	24713044	488	-	chr1	31648621	31712835	6994	-		-962.8	0.9692	1	4.72	6.46	6.02	5.43	6.82	6.16
cis_mRNA_overlap	0	mRNA	n345357	MTCONS_00032370	chr18	33141618	33187473	2083	+	chr18	33141618	33298044	10946	+		-1119.01	-0.8166	-1	0.03	0	0.14	1.17	1.3	1.13
cis_mRNA_dw20k	198	NA	LTCONS_00019210	MTCONS_00019209	chr13	45607160	45609032	1873	+	chr13	45563687	45606963	4716	+	*		0.975	1	1.64	1.71	1.36	8.49	8.81	8.02
tran	NA	NA	LTCONS_00023286	MTCONS_00040816	chr15	25499878	25583387	7818	+	chr2	53889562	54014214	9905	-		-7529.49	0.8747	1	9.22	12.93	9.09	5.36	6.05	4.64
cis_mRNA_overlap	0	mRNA	n406564	MTCONS_00050522	chr3	38048987	38066278	3042	-	chr3	38048708	38071157	2804	-		-1847.15	-0.9501	-1	0.97	1.67	0.6	1.39	0.54	1.42
tran	NA	NA	n381255	MTCONS_00034476	chr1	151763318	151766877	991	+	chr19	44455373	44485875	16403	+		-725.84	-0.9282	-1	4.77	7.11	4.96	3.03	2.78	2.92
cis_mRNA_overlap	0	mRNA	n337752	MTCONS_00024766	chr15	49280967	49338630	6778	-	chr15	49280834	49338977	7394	-		-2517.71	0.9741	1	3.19	2.64	5.83	15.08	13	18.42

tran	NA	NA	n324537	MTCONS_00042064	chr6	33220538	33223522	717	+	chr2	172173912	172290520	8258	-		-607.49	0.9371	1	0	0.05	0.13	0.91	0.97	2.44
tran	NA	NA	n324537	MTCONS_00041200	chr6	33220538	33223522	717	+	chr2	85829929	85839179	8793	-		-486.75	0.9025	1	0	0.05	0.13	3.03	3.42	3.53
tran	NA	NA	n325334	MTCONS_00027624	chr2	41958199	41974683	1803	+	chr16	28385134	28415186	8839	-		-2442.08	0.9977	1	5.85	6.16	5.24	3.95	4.2	3.57
cis_mRNA_up10k	2229	NA	n381616	MTCONS_00011411	chr11	17353589	17371521	3256	+	chr11	17373749	17405078	12680	+	*		-0.9972	-1	0.11	0	0.24	6.51	7.57	4.87
tran	NA	NA	LTCONS_00070633	MTCONS_00026455	chr8	83203316	83204076	761	.	chr16	51679680	51681009	1330	+		-1021.3	0.6217	1	0.31	0.3	0.53	1.68	1.16	1.75
tran	NA	NA	n372282	MTCONS_00002255	chr10	38716834	38724184	518	+	chr1	150266269	150283205	2889	+		-506.62	0.9834	1	0.94	2.82	1.47	9.78	10.32	10.02
cis_mRNA_dw20k	16	NA	n409356	MTCONS_00049903	chr3	180701498	180707562	1401	-	chr3	180630234	180701483	9504	+	*		0.9359	1	0.83	1.31	0.57	3.54	3.61	3.4
tran	NA	NA	n338746	MTCONS_00052157	chr13	58246842	58247428	587	-	chr3	197299019	197354752	9196	-		-725.3	0.9187	1	0.06	0.28	0.11	1.79	2.76	2.36
tran	58303514	NA	n380037	MTCONS_00041200	chr2	27505301	27526416	876	-	chr2	85829929	85839179	8793	-		-880.65	-0.9778	-0.866	0.08	0.04	0.04	3.03	3.42	3.53
cis_mRNA_dw20k	3653	NA	n372960	MTCONS_00018846	chr12	133488105	133491318	2709	-	chr12	133494970	133532981	9230	-	*		0.8575	1	0.06	0.11	0.07	1.87	2.09	2.02
cis_mRNA_overlap	0	Lnc-AntiOverlap-mRNA	LTCONS_00048695	MTCONS_00050642	chr3	47205605	47286260	2989	+	chr3	47269516	47324337	3408	-	*		-0.9854	-1	1.62	1.47	1.13	0.06	0.09	0.27
cis_mRNA_dw20k	2267	NA	LTCONS_00023125	MTCONS_00021554	chr14	86098816	86101707	2892	.	chr14	85999632	86096550	9774	+	*		0.9903	1	0.58	0.91	0.06	0.87	1.4	0.36
cis_mRNA_overlap	0	AntiCompleteIn-mRNAIntron	n339428	MTCONS_00051257	chr3	112736325	112736891	567	+	chr3	112720221	112738587	5804	-	*		-0.9519	-1	0.06	0.3	0.17	0.95	0.8	0.84
cis_mRNA_dw20k	6120	NA	LTCONS_00032061	MTCONS_00032062	chr17_ctg5_hap1	1181916	1182256	341	.	chr17_ctg5_hap1	1188375	1190553	2179	.	*		-0.9966	-1	0.46	0.95	1.55	0.48	0.23	0
cis_mRNA_dw20k	9320	NA	n326635	MTCONS_00004750	chr1	38447293	38453026	2283	-	chr1	38462345	38471234	1719	-	*		-0.9821	-1	0.7	1.01	1.09	1.96	0.46	0.45
cis_mRNA_overlap	0	Lnc-AntiOverlap-mRNA	n324872	MTCONS_00045141	chr21	22303348	22392649	897	-	chr21	22370623	22863909	9470	+	*		0.9339	1	0.11	0.03	0.04	16.17	8.24	11.91
cis_mRNA_up10k	4646	NA	n346415	MTCONS_00033941	chr19	19969356	19972019	1407	-	chr19	19976664	20005965	3828	+	*		0.856	1	0.05	0.09	0.02	5.43	5.59	3.3
tran	NA	NA	n346364	MTCONS_00040072	chr1	741201	745577	252	-	chr2	243030817	243102469	1310	+		-100.45	0.8286	1	0.67	0.61	0.9	1.04	0.59	1.2
tran	NA	NA	n367653	MTCONS_00041200	chr5	29162523	29172465	732	+	chr2	85829929	85839179	8793	-		-534.33	0.6702	0.866	0.1	0.1	0.13	3.03	3.42	3.53
tran	NA	NA	n367653	MTCONS_00043639	chr5	29162523	29172465	732	+	chr20	47835832	47860620	3762	+		-788.15	0.7783	0.866	0.1	0.1	0.13	4.33	6.3	7.43
tran	45785485	NA	n326497	MTCONS_00005969	chr1	103593557	103608574	439	-	chr1	149394058	149400543	5522	-		-268.56	0.8481	1	0.54	1.11	1.36	0.37	0.62	1.64
tran	51325957	NA	n386160	MTCONS_00027511	chr16	70263707	70269272	1210	-	chr16	18816175	18937751	16148	-		-632.12	-0.8478	-1	0.7	0.21	0	2.94	3.24	4.43
cis_mRNA_overlap	0	AntiCompleteIn-mRNAIntron	n340004	MTCONS_00009717	chr10	28127445	28130140	2696	+	chr10	28101096	28288092	3879	-	*		0.9936	0.866	0.03	0.03	0.18	0.24	0.33	0.97
tran	NA	NA	n345486	MTCONS_00049362	chr1	226300133	226321383	1503	+	chr3	124449213	124470145	8967	+		-1132.04	1	1	7.55	13.88	10	3.28	3.93	3.53
tran	NA	NA	n345486	MTCONS_00039494	chr1	226300133	226321383	1503	+	chr2	201980877	202038063	11977	+		-1032.23	0.9933	1	7.55	13.88	10	0.42	0.83	0.62
cis_mRNA_dw20k	17019	NA	n342712	MTCONS_00031273	chr17	45132467	45177627	2080	-	chr17	45194645	45266692	6282	-	*		-0.9998	-1	5.32	4.75	5.22	2.59	3.41	2.75
tran	NA	NA	n369754	MTCONS_00027624	chr7	26301389	26303446	427	+	chr16	28385134	28415186	8839	-		-656.28	0.9529	1	0.51	0.56	0.09	3.95	4.2	3.57
cis_mRNA_overlap	0	mRNA	LTCONS_00033972	MTCONS_00033970	chr19	21579921	21601152	13623	+	chr19	21579919	21601152	5262	+		-2515.72	-0.995	-1	0.15	1.3	0.57	1.72	0.55	1.19
cis_mRNA_overlap	0	Lnc-CompleteIn-mRNAExon	n405416	MTCONS_00035062	chr19	57874879	57876721	732	+	chr19	57874870	57891158	6672	+		-392.29	0.7918	0.866	10.21	11.27	9.55	2.49	2.49	2.04
cis_mRNA_up10k	140	NA	n364921	MTCONS_00037937	chr2	61371541	61372104	434	-	chr2	61372243	61391964	982	+	*		0.9025	0.866	0.24	0.51	0.51	0.96	3.9	2.63
cis_mRNA_overlap	0	Lnc-CompleteIn-mRNAExon	n342215	MTCONS_00024527	chr15	31776244	32162779	2224	-	chr15	31767491	32162867	11188	-		-1717.86	0.803	1	1.5	2.62	1.53	1.54	1.83	1.72
tran	21625972	NA	n346427	MTCONS_00015507	chr12	9596155	9600808	378	-	chr12	31226779	31257730	4327	+		-300.03	-0.9983	-1	0.13	0.09	0	1.63	1.95	2.89
tran	NA	NA	n344802	MTCONS_00014245	chr1	95143531	95145163	829	+	chr11	71996538	72145597	9986	-		-705.14	0.9993	1	0.14	0.2	0.19	7.46	8.44	8.24
tran	NA	NA	n344802	MTCONS_00042413	chr1	95143531	95145163	829	+	chr2	208470330	208490126	5962	-		-668.27	0.6286	0.866	0.14	0.2	0.19	1.77	1.78	1.77
tran	NA	NA	n380353	MTCONS_00039883	chr15	40978922	40987294	718	-	chr2	232063206	232241727	8428	+		-432.06	-0.9948	-1	1.73	1.77	1.55	3.05	3	3.65
tran	NA	NA	n346520	MTCONS_00024396	chr2	114354361	114356127	513	+	chr15	102500790	102516854	2835	+		-460.6	-0.8118	-1	0.83	1.35	0.66	1.44	1.06	3.01
cis_mRNA_dw20k	4322	NA	n375114	MTCONS_00026442	chr16	50840167	50841523	747	+	chr16	50775961	50835846	8796	+	*		0.9999	1	0.05	0	0.09	0.62	0.14	0.99
cis_mRNA_overlap	0	Lnc-CompleteIn-mRNAExon	n338219	MTCONS_00020890	chr14	23776036	23780958	3530	+	chr14	23775971	23782791	5725	+		-2133.89	0.8126	1	5.6	4.32	3.61	2.59	2.55	2.04
tran	NA	NA	n383369	MTCONS_00041449	chr19	10762539	10764548	2010	-	chr2	101887052	101925178	6597	-		-1225.19	-0.999	-1	11.43	9.65	10.21	1.64	2.09	1.93
cis_mRNA_dw20k	17402	NA	n372667	MTCONS_00013380	chr11	9142141	9142948	365	-	chr11	9160349	9286956	5001	-	*		-0.7569	-1	0.53	0.1	0.19	12.18	15.82	12.71
cis_mRNA_dw20k	3563	NA	n341935	MTCONS_00017875	chr12	58012188	58015686	2443	-	chr12	58019248	58026497	4731	-	*		-0.9078	-0.866	0.01	0.01	0	7.64	7.4	7.97
tran	NA	NA	n371423	MTCONS_00057969	chrX	1780739	1781265	419	+	chr5	69770779	69881984	8211	-		-475.29	0.9965	1	0.09	0.33	0	3.85	4.56	3.47
cis_mRNA_overlap	0	Lnc-AntiOverlap-mRNA	n378000	MTCONS_00047604	chr22	28315389	28382380	846	+	chr22	28371171	28839650	14880	-	*		0.994	1	0.7	0.39	0.33	5.49	4.86	4.63
cis_mRNA_up10k	9896	NA	n375473	MTCONS_00030423	chr17	7771139	7777031	2846	-	chr17	7760000	7761244	890	-	*		-0.9933	-1	0.29	0.32	0.16	25.21	24.46	26.99
cis_mRNA_up10k	705	NA	n372342	MTCONS_00007710	chr10	3107634	3108996	1001	-	chr10	3109700	3178997	2516	+	*		0.9988	1	1.2	0.93	0.85	10.33	5.47	4.39
tran	NA	NA	n324194	MTCONS_00030957	chr7	116000775	116042110	626	-	chr17	37408059	37559422	11920	-		-547.05	0.8322	0.866	0	0.05	0.09	0.62	0.62	0.64
tran	NA	NA	n324194	MTCONS_00041200	chr7	116000775	116042110	626	-	chr2	85829929	85839179	8793	-		-692.3	0.9692	1	0	0.05	0.09	3.03	3.42	3.53
cis_mRNA_dw20k	10393	NA	n383521	MTCONS_00034576	chr19	46217645	46220639	2995	-	chr19	46195741	46207253	4196	+	*		0.9069	0.866	1.13	1.28	1.39	0.75	1.24	1.24
tran	NA	NA	n376213	MTCONS_00039494	chr19	28129391	28137384	327	-	chr2	201980877	202038063	11977	+		-382.93	0.9402	1	0	0.15	0.12	0.42	0.83	0.62
cis_mRNA_overlap	0	mRNA	n325829	MTCONS_00014003	chr11	63918390	63919879	985	-	chr11	63766028	63933606	1245	-	*		-0.948	-1	1.01	1.17	1.72	5.32	3.73	2.23
cis_mRNA_overlap	0	Lnc-CompleteIn-mRNAExon	n406462	MTCONS_00048651	chr3	45017741	45054158	1246	+	chr3	45017714	45055480	2792	+		-721.72	-0.9505	-1	5.67	4.84	6.22	1.23	1.59	1.19
tran	NA	NA	n335693	MTCONS_00026659	chr6	3495884	3496296	413	+	chr16	66914383	66925004	7056	+		-370.59	-0.891	-1	0	0.03	0.12	1.68	1.04	0.72
cis_mRNA_overlap	0	Lnc-CompleteIn-mRNAExon	n338446	MTCONS_00006891	chr1	200374083	200375228	1146	-	chr1	200367296	200379171	11654	-		-489.76	-0.9776	-1	6.16	6.15	6.64	1.71	1.76	1.55
cis_mRNA_up10k	1313	NA	n378825	MTCONS_00002789	chr1	173833039	173836181	565	-	chr1	173837493	173872659	19182	+	*		-0.979	-1	79.14	71.56	55.44	3.47	3.75	4.03
cis_mRNA_up10k	4544	NA	n345554	MTCONS_00039290	chr2	178063399	178077157	2669	-	chr2	178081700	178088736	3014	+	*		0.95	1	0.42	0.45	0.38	1.77	2.52	1.46
cis_mRNA_up10k	115	NA	n376207	MTCONS_00036975	chr19	56905087	56910539	1276	+	chr19	56891224	56904973	6027	-	*		0.9297	1	0.46	0.82	1.2	1.17	1.41	1.46
cis_mRNA_dw20k	10454	NA	n376207	MTCONS_00035019	chr19	56905087	56910539	1276	+	chr19	56879504	56894634	6776	+	*		0.8944	1	0.46	0.82	1.2	1.61	1.63	2.01
cis_mRNA_up10k	277	NA	n338527	MTCONS_00021852	chr14	103991347	103995378	4032	-	chr14	103995654	104005335	5709	+	*		0.8131	1	0.9	1.19	0.96	0.24	0.62	0.53
cis_mRNA_up10k	17	NA	n371746	MTCONS_00009629	chr10	18941165	18943952	1615	+	chr10	18799721	18941149	5873	-	*		0.9799	1	0.02	0.04	0.06	1.31	1.42	1.65
cis_mRNA_overlap	0	mRNA	n410532	MTCONS_00016276	chr12	94656297	94701451	3890	+	chr12	94542320	94701999	9202	+		-1764.12	0.8219	1	0.23	0.38	0	0.75	1.19	0.73
cis_mRNA_overlap	0	Lnc-AntiOverlap-mRNA	LTCONS_00049343	MTCONS_00051372	chr3	123304389	123349753	852	+	chr3	123331143	123554784	7750	-	*		0.8152	1	1.91	1.24	1.04	0.46	0.41	0.23

cis_mRNA_dw20k	17632	NA	n380446	MTCONS_00046485	chr22	21823437	21825558	1814	+	chr22	21771672	21805806	6774	+	*	-0.9973	-1	0.8	1.2	1.02	2.04	0.93	1.5
cis_mRNA_overlap	0	CompleteIn-LncExon	LTCONS_00047627	MTCONS_00047626	chr22	29832933	29876337	5427	-	chr22	29832933	29876337	5283	-	-2836.33	0.801	1	0.81	0.65	1.75	2.32	2.11	2.4
tran	523344	NA	n381415	MTCONS_00009717	chr10	27547691	27577753	643	+	chr10	28101096	28288092	3879	-	-369.85	0.8652	1	0.12	0.15	0.17	0.24	0.33	0.97
cis_mRNA_up10k	255	NA	LTCONS_00040881	MTCONS_00037932	chr2	61237016	61244554	1332	-	chr2	61244808	61288260	10877	+	*	-0.9973	-1	0.5	0.92	1.11	2.22	1.94	1.85
cis_mRNA_overlap	0	mRNA	n407040	MTCONS_00001881	chr1	110198698	110204331	1344	+	chr1	110198698	110208123	1290	+	-715.12	-0.7202	-1	0	0.61	0.09	0.65	0	0.1
cis_mRNA_up10k	4956	NA	n410506	MTCONS_00012979	chr11	126139040	126148027	2090	+	chr11	126152982	126169765	7744	+	*	0.9238	1	1.87	3.97	5.59	0.93	0.96	1.07
cis_mRNA_overlap	0	mRNA	n410506	MTCONS_00012973	chr11	126139040	126148027	2090	+	chr11	126138935	126148027	1976	+	-1305.25	-0.8676	-1	1.87	3.97	5.59	4.15	4.01	2.52
cis_mRNA_dw20k	6082	NA	n410506	MTCONS_00012971	chr11	126139040	126148027	2090	+	chr11	126081619	126132959	2167	+	*	0.9391	1	1.87	3.97	5.59	1.67	2.11	3.4
cis_mRNA_dw20k	4827	NA	LTCONS_00042801	MTCONS_00040891	chr2	61698128	61700243	2116	.	chr2	61705069	61766402	5878	-	*	-0.999	-1	0.68	0.74	0.95	3.64	3.39	2.24
tran	NA	NA	n371775	MTCONS_00039883	chr10	72370073	72428508	1481	+	chr2	232063206	232241727	8428	+	-842.49	0.9984	1	0.48	0.45	0.67	3.05	3	3.65
cis_mRNA_up10k	58	NA	LTCONS_00037728	MTCONS_00040660	chr2	39664543	39831216	1520	+	chr2	39475931	39664486	4808	-	*	0.9142	1	0.43	0.13	0.15	4.07	3.33	3.67
cis_mRNA_dw20k	2598	NA	n344693	MTCONS_00054181	chr4	24427776	24526490	8544	-	chr4	24529087	24586232	5998	-	*	0.8988	0.866	0.63	0.84	0.63	12.65	14.27	11.38
cis_mRNA_up10k	84	NA	n342434	MTCONS_00041384	chr2	97760672	97763200	2528	+	chr2	97749305	97760589	1693	-	*	0.7871	1	0.43	0.52	0.39	3.05	3.08	2.73
tran	2172594	NA	n409293	MTCONS_00025322	chr15	82753447	82769399	6807	+	chr15	84941992	85044504	18018	-	-5657.42	0.9793	1	7.76	6.32	4.93	10.55	9.49	7.34
tran	NA	NA	n371417	MTCONS_00007187	chrX	287992	288942	863	+	chr1	220083579	220102117	7043	-	-712.21	0.9973	1	0.04	0.01	0.07	3.64	3.24	3.95
tran	1903213	NA	n387302	MTCONS_00050144	chr3	197319987	197354752	925	-	chr3	195384910	195416775	3199	+	-620	0.9166	1	6.22	6.07	3.35	4.53	3.56	2.34
cis_mRNA_overlap	0	Lnc-CompleteIn-mRNAExon	n342072	MTCONS_00018851	chr12	133697590	133698610	1021	-	chr12	133697491	133707110	1592	-	-427.82	0.9999	1	4.11	3.15	3.38	3.26	2.12	2.41
tran	NA	NA	n383892	MTCONS_00017712	chr20	33421380	33422265	886	-	chr12	53900311	54019780	12002	-	-795.5	0.9987	1	0.12	0.29	0.25	0.83	1.53	1.4
cis_mRNA_dw20k	9788	NA	n383892	MTCONS_00044477	chr20	33421380	33422265	886	-	chr20	33432052	33460664	8075	-	*	0.8665	1	0.12	0.29	0.25	6.4	11.12	7.82
tran	NA	NA	n363580	MTCONS_00024784	chr1	8066074	8066784	410	-	chr15	50849351	50979012	10401	-	-355.33	-0.9601	-1	0	0.09	0.08	2.04	1.46	1.68
cis_mRNA_overlap	0	Lnc-CompleteIn-mRNAExon	n406642	MTCONS_00003163	chr1	204485507	204527248	10009	+	chr1	204485507	204527277	15289	+	-6146.43	0.9834	1	0.56	1.08	0.8	1.54	2.04	1.69
tran	533675	NA	n381026	MTCONS_00003844	chr1	674240	679736	683	-	chr1	131337	140566	5257	-	-464.99	0.9832	1	1.49	3.86	2.17	0.2	0.57	0.24
tran	NA	NA	n381026	MTCONS_00027110	chr1	674240	679736	683	-	chr16	90229013	90235598	2977	+	-411.42	0.8279	1	1.49	3.86	2.17	0.06	0.95	0.79
cis_mRNA_up10k	241	NA	n342446	MTCONS_00041776	chr2	130887194	130896986	1837	+	chr2	130824979	130886954	10720	-	*	0.9492	1	0.34	0.24	0.16	5.02	3.85	0.74
cis_mRNA_overlap	0	mRNA	n338918	MTCONS_00041409	chr2	99242186	99347589	1895	-	chr2	99235515	99347448	8431	-	-862.43	0.998	1	2.56	2.77	3.11	1.94	3.16	5.69
cis_mRNA_dw20k	4048	NA	n408250	MTCONS_00047449	chr22	21921957	21978323	3016	+	chr22	21982370	21985086	2108	-	*	0.9954	1	0.56	0.09	0.23	2.14	1.42	1.57
cis_mRNA_overlap	0	mRNA	n339566	MTCONS_00051532	chr3	133319450	133380690	5298	-	chr3	133318707	133380801	6137	-	-2159.27	-0.8958	-1	1.38	0	0.63	4.04	4.77	4.13
cis_mRNA_overlap	0	Lnc-AntiOverlap-mRNA	n339180	MTCONS_00026307	chr16	30784853	30787354	2502	-	chr16	30772584	30789658	8436	+	-5345.84	0.9884	1	0.11	0.22	0.15	0.85	1.43	0.98
tran	NA	NA	n345101	MTCONS_00026455	chr10	47096361	47139996	4258	-	chr16	51679680	51681009	1330	+	-747.93	-0.9995	-1	0.05	0.22	0.02	1.68	1.16	1.75
cis_mRNA_dw20k	7321	NA	n342049	MTCONS_00018555	chr12	120928131	120933728	1886	-	chr12	120941048	120966965	1634	-	*	-0.7845	-1	1.98	2.77	3.12	9.43	9.33	8.21
tran	614197	NA	n406247	MTCONS_00047425	chr22	21827287	21871780	2272	-	chr22	21061922	21213091	8306	-	-1651.25	0.8671	1	0	1.87	0.84	1.07	1.66	1.62
tran	3421057	NA	n346467	MTCONS_00002104	chr1	148251678	148262280	410	-	chr1	144593657	144830622	7756	+	-270.26	0.9917	1	2.4	3.24	2.92	7.98	8.75	8.37
cis_mRNA_overlap	0	mRNA	LTCONS_00055553	MTCONS_00055550	chr5	7890261	7901237	2101	+	chr5	7869217	7901237	3323	+	-845.15	-0.7784	-1	0.24	0.38	0.17	0.4	0.36	1.37
cis_mRNA_overlap	0	mRNA	n345180	MTCONS_00015755	chr12	52430963	52437695	1141	+	chr12	52435848	52453291	4210	+	*	0.7089	1	0.12	0.29	0.09	0.92	0.97	0.6
tran	NA	NA	n373388	MTCONS_00019478	chr12	28726259	28733275	504	+	chr13	78271833	78345587	10371	+	-534.58	-0.9099	-1	0.37	0.54	0.35	1.68	1.6	1.76
tran	NA	NA	n341861	MTCONS_00051716	chr12	24400760	24401200	441	+	chr3	155538816	155572410	9728	-	-386.99	-0.7949	-1	0.09	0	0.08	2.4	2.77	2.66
cis_mRNA_dw20k	9674	NA	n339346	MTCONS_00028366	chr16	89748969	89752998	782	-	chr16	89762671	89768502	2951	-	*	0.8051	1	0.09	0.17	0.19	1.1	1.19	1.49
cis_mRNA_dw20k	4558	NA	n406867	MTCONS_00030429	chr17	7816640	7819265	2525	-	chr17	7823822	7835317	6848	-	*	0.9659	1	1.72	2.19	1.95	1.22	2.08	1.84
cis_mRNA_dw20k	16773	NA	n376882	MTCONS_00042874	chr20	1166983	1170031	373	+	chr20	1093906	1150211	4273	+	*	0.7448	0.866	0	0.19	0.19	2.53	2.75	2.6
cis_mRNA_overlap	0	Lnc-CompleteIn-mRNAIntron	n382148	MTCONS_00022214	chr14	30084074	30085037	964	-	chr14	30045685	30398478	5282	-	*	0.9969	1	0	0.03	0.16	0.17	0.21	0.54
tran	NA	NA	n344887	MTCONS_00049362	chr6	15708733	15726229	1373	-	chr3	124449213	124470145	8967	+	-1289.98	0.9667	1	0.63	0.7	0.64	3.28	3.93	3.53
tran	NA	NA	n344887	MTCONS_00039494	chr6	15708733	15726229	1373	-	chr2	201980877	202038063	11977	+	-1223.55	0.9297	1	0.63	0.7	0.64	0.42	0.83	0.62
tran	NA	NA	n410226	MTCONS_00028474	chr6	3399752	3401315	1057	-	chr17	2211319	2228553	4379	+	-594.49	0.9975	1	1.75	1.3	0.96	1.54	1.42	1.35
cis_mRNA_up10k	2464	NA	n376342	MTCONS_00033319	chr19	1246298	1248229	673	-	chr19	1250692	1259142	3911	+	*	-0.998	-1	0.12	0.27	0.28	1.91	1.44	1.37
cis_mRNA_up10k	6643	NA	LTCONS_00037081	MTCONS_00037080	chr19	9937854	9938509	656	.	chr19	9930431	9931212	782	.	*	0.9221	1	0.11	0.4	0.1	2.75	3.34	2.33
cis_mRNA_dw20k	14191	NA	n342345	MTCONS_00035888	chr19	20318001	20320598	2598	-	chr19	20334788	20349276	4685	-	*	0.8616	0.866	0.2	0.2	0.19	5.26	5.92	4.62
tran	NA	NA	n367818	MTCONS_00034476	chr5	135802366	135844717	676	+	chr19	44455373	44485875	16403	+	-470.62	0.9545	1	3.81	0	1.12	3.03	2.78	2.92
cis_mRNA_dw20k	3562	NA	n364761	MTCONS_00042064	chr2	172167776	172170351	2258	+	chr2	172173912	172290520	8258	-	*	0.7698	1	1.5	1.69	1.78	0.91	0.97	2.44
tran	NA	NA	n369554	MTCONS_00049362	chr7	13141016	13142014	575	+	chr3	124449213	124470145	8967	+	-831.3	1	1	0.05	0.23	0.12	3.28	3.93	3.53
tran	NA	NA	n369554	MTCONS_00004585	chr7	13141016	13142014	575	+	chr1	31648621	31712835	6994	-	-889.06	0.9878	1	0.05	0.23	0.12	5.43	6.82	6.16
tran	NA	NA	LTCONS_00063770	MTCONS_00030115	chr6	26755066	26758426	3361	.	chr17	80693452	80703834	5094	+	-1851.61	-1	-1	0.38	0.41	0.35	2.63	2.55	2.71
tran	NA	NA	LTCONS_00063770	MTCONS_00034837	chr6	26755066	26758426	3361	.	chr19	52872319	52925184	17190	+	-1948.72	0.9805	1	0.38	0.41	0.35	2.75	2.91	2.42
tran	NA	NA	n323799	MTCONS_00048261	chrX	139590937	139597536	323	-	chr3	10183319	10195354	5394	+	-362.05	-0.9464	-1	1.27	1.02	0.95	6.11	6.49	6.89
tran	NA	NA	n387043	MTCONS_00049362	chr17	1143726	1150972	942	-	chr3	124449213	124470145	8967	+	-1533	-0.9486	-1	0.13	0	0.12	3.28	3.93	3.53
tran	NA	NA	n387043	MTCONS_00004585	chr17	1143726	1150972	942	-	chr1	31648621	31712835	6994	-	-1527.29	-0.8854	-1	0.13	0	0.12	5.43	6.82	6.16
tran	NA	NA	n324381	MTCONS_00058457	chr6	135068891	135070180	929	-	chr5	132028323	132073287	6288	-	-761.28	0.8612	1	0.08	0	0.09	40.31	38.91	42.38
tran	NA	NA	n377812	MTCONS_00037620	chr9	98575575	98638259	809	-	chr2	32288518	32383018	5380	+	-737.87	0.8788	1	0	0.61	0.3	2.96	3.95	3.91
tran	NA	NA	n377812	MTCONS_00019045	chr9	98575575	98638259	809	-	chr13	28712637	28847529	3366	+	-739.82	0.9999	1	0	0.61	0.3	2.72	3.24	2.97
cis_mRNA_up10k	169	NA	n338247	MTCONS_00022231	chr14	31889964	31922089	1213	+	chr14	31760633	31889796	8236	-	*	-0.9993	-0.866	0.06	0.03	0.03	0.86	1.09	1.08
cis_mRNA_up10k	8899	NA	n382897	MTCONS_00025637	chr16	432241	442960	1571	+	chr16	451858	462487	2323	+	*	0.898	1	0.04	0.1	0	2.52	2.8	1.35
cis_mRNA_up10k	292	NA	n382897	MTCONS_00027141	chr16	432241	442960	1571	+	chr16	420716	431950	3956	-	*	-0.8371	-1	0.04	0.1	0	1.28	1.22	2.12
cis_mRNA_dw20k	5936	NA	n345381	MTCONS_00044020	chr20	1416426	1416866	441	-	chr20	1422801	1448337	3580	-	*	-0.8797	-1	0	0.31	0.23	15.13	14.01	14.79
cis_mRNA_dw20k	7685	NA	n376470	MTCONS_00036865	chr19	53727171	53727904	455	+	chr19	53735588	53758156	6823	-	*	0.9177	1	0	0.08	0.07	0.93	1.03	0.98
cis_mRNA_overlap	0	Lnc-CompleteIn-mRNAExon	n337306	MTCONS_00056253	chr5	86563828	86564591	764	+	chr5	86563596	86690081	7198	+	-692.19	0.9194	1	3.79	3.1	2.55	2.72	2.65	2.36
cis_mRNA_up10k	46	NA	LTCONS_00018586	MTCONS_00016645	chr12	122231680	122241988	8559	-	chr12	122242033	122270592	8469	+	*	0.8038	1	4.16	6.52	4.24	1.66	2.14	1.96
tran	NA	NA	n326471	MTCONS_00039148	chr1	116508428	116510385	914	+	chr2	168148819	168730621	6608	+	-772.25	-0.9657	-1	0.88	0.61	0.49	10.67	15.23	15.46
tran	NA	NA	n326471	MTCONS_00041200	chr1	116508428	116510385	914	+	chr2	85829929	85839179	8793	-	-523.41	-0.9956	-1	0.88	0.61	0.49	3.03	3.42	3.53

tran	NA	NA	n385788	MTCONS_00033970	chrX	74544124	74546232	2109	+	chr19	21579919	21601152	5262	+		-1755.83	-0.8948	-1	0.83	0.99	0.97	1.72	0.55	1.19
cis_mRNA_overlap	0	mRNA	n346007	MTCONS_00038754	chr2	118594388	118603633	3105	+	chr2	118572251	118597355	10414	+		-1572.17	0.6343	0.866	0.21	0.25	0.44	0.63	0.76	0.76
cis_mRNA_up10k	5299	NA	n376431	MTCONS_00036975	chr19	56910271	56915279	340	-	chr19	56891224	56904973	6027	-	*		0.9869	0.866	0.12	0.21	0.21	1.17	1.41	1.46
cis_mRNA_up10k	203	NA	LTCONS_00045374	MTCONS_00045375	chr21	38737803	38738504	702	+	chr21	38738706	38892039	10771	+	*		0.8571	1	0.88	0.28	0.74	2.95	2.74	2.8
cis_mRNA_overlap	0	mRNA	n326766	MTCONS_00003844	chr1	131337	139570	4860	-	chr1	131337	140566	5257	-		-3305.65	-0.9951	-0.866	0.47	0.3	0.47	0.2	0.57	0.24
tran	NA	NA	n326766	MTCONS_00027110	chr1	131337	139570	4860	-	chr16	90229013	90235598	2977	+		-2167.26	-0.6389	-0.866	0.47	0.3	0.47	0.06	0.95	0.79
cis_mRNA_overlap	0	mRNA	n407261	MTCONS_00044488	chr20	33703160	33735161	2129	-	chr20	33703160	33735276	2079	-		-1204.11	-0.7331	-1	0.24	0.31	0.29	1.4	0.44	1.38
tran	3941565	NA	LTCONS_00009961	MTCONS_00010036	chr10	48000424	48004053	1636	-	chr10	51945617	51952763	2088	-		-610.3	0.9834	1	2.46	3.18	0.61	3.52	4.45	2.35
tran	NA	NA	n326310	MTCONS_00015928	chr1	180322309	180325870	1145	+	chr12	56914542	56989980	8281	+		-734.41	-0.9838	-0.866	0.06	0.03	0.03	0.72	1.14	1.06
tran	NA	NA	n373137	MTCONS_00025988	chr12	124558542	124559839	396	+	chr16	15489513	15507131	6113	+		-268.49	0.9981	1	0.48	0.55	0.34	3.06	3.56	2.27
tran	NA	NA	n377223	MTCONS_00039883	chr21	40447036	40450266	2751	-	chr2	232063206	232241727	8428	+		-1496.44	-0.6501	-1	0.11	0.18	0.1	3.05	3	3.65
cis_mRNA_overlap	0	mRNA	n406198	MTCONS_00024396	chr15	102501016	102516808	2554	+	chr15	102500790	102516854	2835	+		-1705.79	0.9285	1	5.49	3.71	7.03	1.44	1.06	3.01
tran	NA	NA	n406198	MTCONS_00014973	chr15	102501016	102516808	2554	+	chr12	73456	91263	2647	+		-1690.2	-0.912	-1	5.49	3.71	7.03	6.23	9.05	6.03
tran	NA	NA	n338260	MTCONS_00030957	chr19	999795	1002756	634	+	chr17	37408059	37559422	11920	-		-679.1	0.8447	0.866	4.16	4.01	4.29	0.62	0.62	0.64
cis_mRNA_dw20k	6891	NA	n338260	MTCONS_00035205	chr19	999795	1002756	634	+	chr19	1009646	1021141	2935	-	*		-0.9863	-1	4.16	4.01	4.29	9.37	10.85	8.68
cis_mRNA_dw20k	5227	NA	n338260	MTCONS_00033298	chr19	999795	1002756	634	+	chr19	982923	994569	2134	+	*		-0.9138	-1	4.16	4.01	4.29	0.74	1.78	0.66
tran	NA	NA	n338260	MTCONS_00058465	chr19	999795	1002756	634	+	chr5	132157833	132166769	3740	-		-807.54	0.9784	1	4.16	4.01	4.29	0.51	0.34	0.82
tran	NA	NA	n377832	MTCONS_00001977	chr2	47558519	47572128	1046	-	chr1	113929129	114239814	22242	+		-801.63	0.8456	1	1.5	2.04	2.84	0.76	0.99	1.01
tran	NA	NA	n342813	MTCONS_00049362	chr7	72693843	72698885	549	-	chr3	124449213	124470145	8967	+		-694.22	-0.9867	-1	2.6	1.71	2.39	3.28	3.93	3.53
tran	NA	NA	n342813	MTCONS_00004585	chr7	72693843	72698885	549	-	chr1	31648621	31712835	6994	-		-646.14	-0.9476	-1	2.6	1.71	2.39	5.43	6.82	6.16
cis_mRNA_dw20k	3	NA	n410027	MTCONS_00052592	chr4	15606007	15657035	3644	-	chr4	15471485	15606005	8056	+	*		-0.6363	-0.866	0.1	0.1	0.17	0.2	0.34	0.17
tran	NA	NA	n326111	MTCONS_00012720	chr10	3256008	3259599	597	+	chr11	113775380	113821262	6005	+		-470.52	0.9867	1	0.09	0.11	0.16	0.67	0.89	2.39
cis_mRNA_overlap	0	mRNA	n338213	MTCONS_00005888	chr1	145456236	145470388	1603	-	chr1	145452782	145470388	2046	-		-868.45	0.824	1	1.06	0.88	1.17	0.22	0.21	0.36
cis_mRNA_up10k	8639	NA	LTCONS_00023844	MTCONS_00024955	chr15	65588362	65588865	504	+	chr15	65550001	65579724	3673	-	*		0.9858	1	1.55	2.22	1.45	0.95	1.24	0.83
tran	NA	NA	n367438	MTCONS_00009490	chr4	183994216	183998865	554	-	chr10	7794066	7829950	4864	-		-611.29	-0.9745	-1	0.55	0.39	0.56	1.73	2.26	1.52
cis_mRNA_overlap	0	mRNA-AntiCompleteIn-LncIntron	n338397	MTCONS_00033902	chr19	18682349	18705245	1701	-	chr19	18698939	18703228	1852	+	*		0.9769	0.866	0.02	0.02	0.08	5.83	6.24	7.66
cis_mRNA_overlap	0	mRNA	n338397	MTCONS_00035829	chr19	18682349	18705245	1701	-	chr19	18703227	18717660	3102	-	*		-0.8985	-0.866	0.02	0.02	0.08	0.32	0.43	0.18
tran	NA	NA	n375707	MTCONS_00026501	chr17	36443036	36452228	798	-	chr16	55513042	55553530	6298	+		-484.17	0.6792	1	0.63	0.67	0.41	0.75	1.37	0.69
cis_mRNA_dw20k	6487	NA	n381519	MTCONS_00008660	chr10	89003889	89102315	2340	-	chr10	88983430	88997403	8066	+	*		-0.9991	-1	5.01	5.46	3.8	0.92	0.76	1.45
cis_mRNA_dw20k	1985	NA	n340529	MTCONS_00028789	chr17	16342301	16345332	983	+	chr17	16318851	16340317	2810	+	*		-0.9982	-1	30.91	31.39	23.98	0.4	0	3.14
tran	572662	NA	n410022	MTCONS_00033941	chr19	20578626	20607771	636	-	chr19	19976664	20005965	3828	+		-277.55	-0.8889	-1	0.33	0.13	0.52	5.43	5.59	3.3
tran	NA	NA	n379022	MTCONS_00042413	chr1	173834093	173837127	632	-	chr2	208470330	208490126	5962	-		-589.32	0.9885	0.866	0.42	0.79	0.48	1.77	1.78	1.77
cis_mRNA_up10k	119	NA	n376778	MTCONS_00045032	chr20	62959556	62961294	442	+	chr20	62948127	62959438	11233	-	*		0.9821	1	0.28	0.62	0.69	2.02	2.32	2.48
tran	25924	NA	n382699	MTCONS_00025322	chr15	85070427	85114026	2150	-	chr15	84941992	85044504	18018	-		-1741.78	-0.9978	-1	3.25	3.49	4.14	10.55	9.49	7.34
cis_mRNA_overlap	0	mRNA	n382954	MTCONS_00026221	chr16	29086163	29128036	2263	+	chr16	29088992	29128036	1913	+		-867.16	0.8636	1	0.02	0.04	0.07	0	0.22	0.25
tran	NA	NA	n367900	MTCONS_00037620	chr5	55832410	55833653	536	-	chr2	32288518	32383018	5380	+		-601.96	0.6108	1	0.1	0.38	0.13	2.96	3.95	3.91
cis_mRNA_up10k	72	NA	LTCONS_00025591	MTCONS_00023950	chr15	73976176	73976490	315	.	chr15	73976561	74006859	3801	+	*		-0.6758	-1	0.38	0.69	0.68	2.86	1.86	2.66
tran	19649885	NA	n364048	MTCONS_00003518	chr1	247517241	247522638	1904	+	chr1	227751220	227867357	4453	+		-919.54	0.9885	1	0.34	0.18	0.1	2.1	1.78	1.5
tran	NA	NA	n324739	MTCONS_00008032	chr22	18710726	18711888	446	-	chr10	28821293	28912041	6050	+		-579.7	0.8892	1	0.24	0.62	0.86	3.62	3.78	4.47
cis_mRNA_up10k	1031	NA	LTCONS_00007577	MTCONS_00000142	chr1	2158816	2159104	289	.	chr1	2160134	2241809	5870	+	*		0.6934	0.866	0	0.13	0.13	1.05	1.07	1.13
tran	NA	NA	n386321	MTCONS_00000634	chr22	25498414	25508659	943	-	chr1	26127068	26144764	4509	+		-908.7	-0.9894	-1	0.41	0.14	0.2	1.21	2.84	2.26
tran	NA	NA	n365154	MTCONS_00021651	chr2	54904602	54908989	352	+	chr14	93890382	94178505	20214	+		-407.1	0.9356	1	0	0.19	0.1	0.9	0.97	0.96
cis_mRNA_dw20k	13119	NA	n411587	MTCONS_00023870	chr15	67807260	67813400	304	-	chr15	67547138	67794142	3905	+	*		0.9624	1	0.09	0.36	0.43	0.52	0.58	0.63
tran	NA	NA	n324045	MTCONS_00058457	chr9	92103004	92235591	1013	-	chr5	132028323	132073287	6288	-		-771.74	0.9434	1	0.07	0	0.1	40.31	38.91	42.38
tran	NA	NA	n326137	MTCONS_00040816	chr1	242337290	242338936	567	+	chr2	53889562	54014214	9905	-		-568.42	0.9843	1	15.29	18.86	13.32	5.36	6.05	4.64
tran	NA	NA	n380216	MTCONS_00042413	chr8	104203192	104298133	551	-	chr2	208470330	208490126	5962	-		-544.55	-0.8864	-0.866	0.47	0.06	0.28	1.77	1.78	1.77
cis_mRNA_overlap	0	Lnc-CompleteIn-mRNAIntron	n343017	MTCONS_00031335	chr17	47416677	47417192	508	-	chr17	47366568	47439476	10952	-	*		0.9996	1	0	0.27	0.13	1.07	1.88	1.44
cis_mRNA_up10k	270	NA	n342010	MTCONS_00016430	chr12	107347813	107349275	1463	-	chr12	107349544	107367823	3311	+	*		-0.8871	-1	1.52	1.21	0.95	1.64	1.81	3.28
cis_mRNA_dw20k	16276	NA	n383733	MTCONS_00041776	chr2	130783572	130808704	4525	-	chr2	130824979	130886954	10720	-	*		0.9999	1	1.35	1	0.12	5.02	3.85	0.74
tran	NA	NA	n338881	MTCONS_00034837	chr2	73988396	73988908	513	-	chr19	52872319	52925184	17190	+		-408.8	0.9046	1	0.58	0.73	0.52	2.75	2.91	2.42
cis_mRNA_up10k	57	NA	n332606	MTCONS_00029146	chr17	34897565	34900681	1382	-	chr17	34900737	34946278	2853	+	*		0.9738	1	0.5	0.25	0.52	12.97	10.84	13.88
cis_mRNA_up10k	4218	NA	LTCONS_00003880	MTCONS_00000052	chr1	850358	860728	9182	-	chr1	864945	880060	3240	+	*		0.9997	1	0.97	3.9	0.57	1.63	5.67	0.95
cis_mRNA_overlap	0	mRNA	n339381	MTCONS_00048898	chr3	58291999	58304211	1531	+	chr3	58291972	58310606	7854	+		-944.14	0.9447	1	0.85	0.55	0	4.32	4.31	3.91
cis_mRNA_overlap	0	mRNA	n372533	MTCONS_00011939	chr11	61236176	61241062	857	+	chr11	61197507	61241364	1630	+		-435.07	-0.6909	-1	6.61	4.74	4.43	4.5	4.63	5.61
cis_mRNA_dw20k	14059	NA	n410486	MTCONS_00043557	chr20	44486220	44507769	2972	+	chr20	44462419	44472162	4226	+	*		-0.9988	-1	0.51	0.36	0.46	0.87	1.25	0.98
tran	NA	NA	n325714	MTCONS_00040816	chr13	61476227	61501682	708	-	chr2	53889562	54014214	9905	-		-433.62	0.9225	1	0.13	0.14	0.06	5.36	6.05	4.64
cis_mRNA_overlap	0	Lnc-CompleteIn-mRNAExon	n409307	MTCONS_00024012	chr15	75287876	75313836	3250	+	chr15	75287876	75313868	3507	+		-2085.14	-0.9074	-1	6	2.8	3.81	6.38	7.14	6.61
cis_mRNA_up10k	74	NA	LTCONS_00025660	MTCONS_00025663	chr16	691816	699262	1868	+	chr16	699335	717829	8992	+	*		-0.9802	-1	2.1	1.77	1.82	0.75	1.31	1.12
tran	NA	NA	n380971	MTCONS_00039148	chr14	27312658	27629626	568	+	chr2	168148819	168730621	6608	+		-396.16	0.903	1	0.28	0.53	0.75	10.67	15.23	15.46
tran	NA	NA	n380971	MTCONS_00041200	chr14	27312658	27629626	568	+	chr2	85829929	85839179	8793	-		-610.49	0.9622	1	0.28	0.53	0.75	3.03	3.42	3.53
tran	NA	NA	LTCONS_00029129	MTCONS_00036322	chr17	33895109	33901376	893	+	chr19	41171215	41222791	6345	-		-627.9	0.9297	1	15.53	12.73	14.04	0.79	0.37	0.42
tran	NA	NA	LTCONS_00029129	MTCONS_00004343	chr17	33895109	33901376	893	+	chr1	21067378	21113807	5598	-		-820.14	-0.9929	-1	15.53	12.73	14.04	2.09	2.16	2.12
tran	NA	NA	LTCONS_00029129	MTCONS_00015928	chr17	33895109	33901376	893	+	chr12	56914542	56989980	8281	+		-888.44	-0.9535	-1	15.53	12.73	14.04	0.72	1.14	1.06
cis_mRNA_up10k	119	NA	LTCONS_00015468	MTCONS_00015473	chr12	27676015	27676927	913	+	chr12	27677045	27854017	11564	+	*		0.9728	1	0.19	0.21	0.24	0.79	0.84	1.06
cis_mRNA_dw20k	6066	NA	n364265	MTCONS_00003351	chr1	212800569	212811889	6782	-	chr1	212781994	212794504	2777	+	*		0.8709	0.866	0.19	0.19	0.12	0.8	0.51	0.21
cis_mRNA_overlap	0	Lnc-CompleteIn-mRNAIntron	n338760	MTCONS_00037677	chr2	36750870	36751847	978	+	chr2	36582870	36778409	5804	+	*		0.9855	1	0.43	0.63	1.45	5.22	5.34	11.94
tran	703085	NA	n382702	MTCONS_00025322	chr15	85747588	85777989	2408	-	chr15	84941992	85044504	18018	-		-3565.73	-0.996	-1	1.3	1.93	3.84	10.55	9.49	7.34
cis_mRNA_up10k	231	NA	n335503	MTCONS_00018606	chr12	123011790	123012372	478	+	chr12	122989190	123011560	2352	-	*		0.9853	1	0.16	0.29	0	14.14	15.52	13.21

cis_mRNA_up10k	213	NA	n409180	MTCONS_00034490	chr19	44609492	44617336	3361	-	chr19	44617548	44639336	5456	+		-3267.05	-0.9373	-1	17.7	16.09	13.78	1.23	1.44	1.52
tran	NA	NA	n386654	MTCONS_00049362	chr11	41326847	41327450	604	+	chr3	124449213	124470145	8967	+		-1240	0.9571	1	2.49	4.58	3.83	3.28	3.93	3.53
tran	NA	NA	n386654	MTCONS_00039494	chr11	41326847	41327450	604	+	chr2	201980877	202038063	11977	+		-1152.8	0.9846	1	2.49	4.58	3.83	0.42	0.83	0.62
cis_mRNA_up10k	671	NA	n379855	MTCONS_00056566	chr5	127359719	127418652	968	-	chr5	127419322	127525380	6973	+	*		0.9998	1	0.09	0.06	0.05	9.74	8.85	8.58
cis_mRNA_dw20k	4887	NA	n345333	MTCONS_00026714	chr16	67589587	67592996	650	-	chr16	67562710	67584701	17635	+	*		0.9332	1	0.78	0.97	1.25	1.9	2.73	3.04
cis_mRNA_overlap	0	Lnc-AntiOverlap-mRNA	n342315	MTCONS_00002436	chr1	155201063	155203428	2366	-	chr1	155202886	155204238	668	+	*		-0.9337	-1	1.42	2.17	2.1	5.17	2.95	3.92
cis_mRNA_up10k	3739	NA	n342315	MTCONS_00006232	chr1	155201063	155203428	2366	-	chr1	155183518	155197325	2507	-	*		0.9603	1	1.42	2.17	2.1	1.8	2.68	2.36
cis_mRNA_dw20k	4020	NA	n377963	MTCONS_00005268	chr1	84041459	84326679	3177	-	chr1	84330698	84464833	10521	-	*		-0.905	-1	0.07	0.02	0.01	7.04	8.16	9.6
cis_mRNA_dw20k	6006	NA	n345423	MTCONS_00003247	chr1	207974866	208042495	6629	-	chr1	207925383	207968861	6335	+	*		0.948	1	0.27	0.29	0.24	26.42	27.54	15.56
cis_mRNA_up10k	108	NA	n372572	MTCONS_00014333	chr11	77850806	77852799	561	+	chr11	77811985	77850699	2455	-	*		-0.9819	-1	0.5	0.37	0.44	3.61	5.01	4.02
cis_mRNA_overlap	0	Lnc-CompleteIn-mRNAExon	n382511	MTCONS_00024678	chr15	43033744	43035546	1803	-	chr15	43033648	43213023	9214	-		-821.17	0.7046	0.866	3.68	3.86	3.18	9.3	9.74	9.3
cis_mRNA_up10k	74	NA	n409454	MTCONS_00012982	chr11	126211612	126225482	752	-	chr11	126225555	126284597	1900	+	*		-0.9736	-1	1.53	1.15	0.25	2.03	2.25	2.47
cis_mRNA_overlap	0	CompleteIn-LncIntron	n377748	MTCONS_00018580	chr12	121633051	122226808	1385	-	chr12	121862156	121905146	8398	-	*		-0.9834	-1	0.22	0.29	0.24	1.12	0.75	1.08
cis_mRNA_dw20k	5711	NA	n405418	MTCONS_00027286	chr16	3265562	3266546	985	+	chr16	3272256	3285457	4523	-	*		0.9629	1	0.14	0.1	0.06	1	0.74	0.65
cis_mRNA_dw20k	183	NA	LTCONS_00052196	MTCONS_00048792	chr3	50688742	50700924	12183	.	chr3	50654571	50688560	4386	+	*		0.9298	1	1.91	1.81	1.25	2.33	1.82	1.3
cis_mRNA_overlap	0	Lnc-CompleteIn-mRNAExon	n381233	MTCONS_00002104	chr1	144828973	144830389	1417	+	chr1	144593657	144830622	7756	+		-838.54	-0.9553	-1	11.89	10.51	11.56	7.98	8.75	8.37
tran	NA	NA	n381586	MTCONS_00034660	chr11	881476	882176	701	-	chr19	48972465	48985574	9032	+		-714.71	-0.9077	-1	0.63	0.47	0.32	3.37	5.1	5.25
cis_mRNA_dw20k	13619	NA	n381586	MTCONS_00011116	chr11	881476	882176	701	-	chr11	849826	867858	2424	+	*		0.9139	1	0.63	0.47	0.32	3.58	3.42	2.39
tran	NA	NA	n379993	MTCONS_00042413	chr5	43575425	43603206	1040	-	chr2	208470330	208490126	5962	-		-790.4	-0.6441	-0.866	1.56	1.45	2.04	1.77	1.78	1.77
tran	NA	NA	n326089	MTCONS_00035888	chr10	5266321	5295165	477	+	chr19	20334788	20349276	4685	-		-240.72	0.9981	1	0.12	0.22	0	5.26	5.92	4.62
tran	NA	NA	n385663	MTCONS_00027624	chr9	103115343	103156148	1956	+	chr16	28385134	28415186	8839	-		-2457.27	0.8536	1	0.36	0.62	0.33	3.95	4.2	3.57
tran	NA	NA	LTCONS_00070632	MTCONS_00006433	chr8	81471039	81471239	201	.	chr1	160236583	160237032	450	-		-106.93	-0.947	-1	177.64	69.48	0	0	0.1	0.31
tran	NA	NA	LTCONS_00070632	MTCONS_00020697	chr8	81471039	81471239	201	.	chr13	21535411	21535878	468	.		-113.9	1	1	177.64	69.48	0	3.35	1.51	0.36
cis_mRNA_overlap	0	mRNA	n345139	MTCONS_00011411	chr11	17401302	17403209	1144	+	chr11	17373749	17405078	12680	+		-690.57	0.9838	1	4.77	5.15	3.49	6.51	7.57	4.87
tran	NA	NA	n366364	MTCONS_00034660	chr3	184429982	184433265	672	+	chr19	48972465	48985574	9032	+		-667.37	0.8444	1	0.93	1.6	2.6	3.37	5.1	5.25
tran	3510579	NA	n407685	MTCONS_00027511	chr16	22448329	22503541	5010	+	chr16	18816175	18937751	16148	-		-2648.67	-0.9505	-1	3.59	2.54	1.43	2.94	3.24	4.43
tran	NA	NA	n384644	MTCONS_00032010	chr5	55232481	55235321	2841	-	chr17	20492220	20497653	5434	.		-1046.6	0.9877	1	0.73	2.01	0	4.2	4.21	4.19
tran	NA	NA	n372876	MTCONS_00052157	chr11	77282102	77287689	1280	-	chr3	197299019	197354752	9196	.		-1048.48	-0.912	-0.866	0.11	0.07	0.07	1.79	2.76	2.36
tran	NA	NA	n324679	MTCONS_00034660	chr22	30689344	30696387	473	+	chr19	48972465	48985574	9032	+		-535.91	0.9835	1	0.46	1.17	1.42	3.37	5.1	5.25
tran	NA	NA	n324679	MTCONS_00008384	chr22	30689344	30696387	473	+	chr10	70480968	70553348	6024	+		-467.34	0.9414	1	0.46	1.17	1.42	2.2	2.64	3.21
cis_mRNA_overlap	0	Lnc-AntiOverlap-mRNA	n377948	MTCONS_00047604	chr22	28331149	28395645	627	+	chr22	28371171	28839650	14880	-	*		-0.8752	-1	0	0.04	0.14	5.49	4.86	4.63
cis_mRNA_up10k	256	NA	n410130	MTCONS_00020276	chr13	52586523	52603780	1402	+	chr13	52506805	52586268	6893	-	*		0.9898	1	0.06	0.12	0.37	2	2.36	3.1
cis_mRNA_overlap	0	Lnc-CompleteIn-mRNAExon	n410130	MTCONS_00019354	chr13	52586523	52603780	1402	+	chr13	52586400	52608636	7544	+		-726.51	-0.8982	-1	0.06	0.12	0.37	1.24	1.15	1.09
cis_mRNA_dw20k	8554	NA	n345336	MTCONS_00028172	chr16	75614134	75618397	879	+	chr16	75626950	75657267	9661	-	*		0.6758	0.866	0.04	0.07	0.04	3.3	3.52	3.47
cis_mRNA_dw20k	13309	NA	n406625	MTCONS_00008021	chr10	27484143	27531068	3739	-	chr10	27443753	27470835	3259	+	*		0.9984	1	1.06	1.29	0.76	0.47	0.91	0
tran	122364185	NA	LTCONS_00002100	MTCONS_00000547	chr1	144175577	144219394	270	+	chr1	21794885	21811393	3477	+		-166.17	-0.7279	-1	0.77	2.84	0.69	0.9	0.55	1.87
cis_mRNA_overlap	0	Lnc-AntiOverlap-mRNA	n342332	MTCONS_00021439	chr14	74034942	74036537	1596	-	chr14	74035684	74042389	1398	+		-1302.9	0.9846	1	0.82	1.48	1.24	1.99	3.48	2.71
cis_mRNA_dw20k	1719	NA	n344825	MTCONS_00056112	chr5	71663446	71664218	772	+	chr5	71616200	71661728	7741	+	*		0.9964	1	0.51	0.93	0.87	1.02	1.33	1.26
cis_mRNA_up10k	86	NA	n376335	MTCONS_00036975	chr19	56905058	56910717	1305	+	chr19	56891224	56904973	6027	-	*		-0.9821	-1	0.97	0.43	0.14	1.17	1.41	1.46
cis_mRNA_dw20k	10425	NA	n376335	MTCONS_00035019	chr19	56905058	56910717	1305	+	chr19	56879504	56894634	6776	+	*		-0.7952	-1	0.97	0.43	0.14	1.61	1.63	2.01
tran	NA	NA	LTCONS_00028400	MTCONS_00046412	chr16	4325104	4325525	422	.	chr22	20119364	20135530	12775	+		-424.18	0.7864	1	0.18	1.32	0.32	1.54	2.16	1.99
tran	NA	NA	n384963	MTCONS_00012979	chr6	73972950	73986225	1056	+	chr11	126152982	126169765	7744	+		-1089.4	-0.8178	-1	1.31	0.86	0.72	0.93	0.96	1.07
cis_mRNA_dw20k	10733	NA	LTCONS_00027053	MTCONS_00028368	chr16	89753012	89762800	7393	+	chr16	89773532	89787468	2832	-	*		-0.8244	-1	1.87	2.77	2.61	3.02	1.96	2.72
cis_mRNA_overlap	0	Lnc-AntiOverlap-mRNA	n339808	MTCONS_00053990	chr4	2938993	2963465	1616	+	chr4	2939568	2965343	3861	-	*		0.7214	1	0.24	0.32	0.03	5.4	6.12	5.39
tran	764508	NA	n384657	MTCONS_00057969	chr5	68921203	69006272	1314	-	chr5	69770779	69881984	8211	-		-958.39	0.8118	1	5.62	5.65	5.28	3.85	4.56	3.47
cis_mRNA_overlap	0	mRNA	n407558	MTCONS_00011225	chr11	3829766	3847582	1610	+	chr11	3829240	3848095	2963	+		-1092.62	-0.9614	-1	0.89	0.35	0.94	0.12	0.34	0
cis_mRNA_dw20k	5214	NA	n341412	MTCONS_00019612	chr13	101187995	101232367	2368	-	chr13	100741269	101182782	2739	+	*		0.9838	0.866	0.1	0.09	0.09	3.99	3.65	3.57
tran	566998	NA	LTCONS_00038885	MTCONS_00038904	chr2	131390143	131401623	226	+	chr2	131968620	132022433	3376	+		-120.66	1	1	2.35	0.16	0.67	1.74	0.31	0.64
tran	133278	NA	LTCONS_00038885	MTCONS_00041793	chr2	131390143	131401623	226	+	chr2	131233939	131256866	971	-		-94.3	0.994	1	2.35	0.16	0.67	1.36	0	0.17
tran	NA	NA	n366822	MTCONS_00040619	chr4	31351906	31353347	602	+	chr2	37323735	37384351	12737	-		-624.25	0.9493	1	0.3	0.42	0.11	4.96	5.38	4.75
cis_mRNA_overlap	0	mRNA	n324637	MTCONS_00053318	chr4	118975004	119010551	576	+	chr4	118953902	119179876	6951	+	*		0.9869	0.866	0.06	0.06	0.17	1.52	1.33	2.43
cis_mRNA_up10k	7625	NA	n325188	MTCONS_00041541	chr2	107512375	107539214	1539	+	chr2	107418056	107504751	8290	-	*		-0.9663	-0.866	0	0.02	0.02	1.38	1.16	1.08
tran	NA	NA	n379147	MTCONS_00041449	chr7	144040335	144052494	568	-	chr2	101887052	101925178	6597	-		-777.46	0.9925	1	4.63	8.77	7.76	1.64	2.09	1.93
cis_mRNA_dw20k	14562	NA	n378945	MTCONS_00039296	chr2	179278721	179303866	903	+	chr2	179059208	179264160	7259	+	*		0.7444	1	1.11	1.15	1.3	0.25	0.46	0.49
tran	NA	NA	n345739	MTCONS_00027113	chr1	14778	16668	507	-	chr16	64484	67398	2407	-		-441.66	-0.9803	-1	0.34	0	0.08	2.83	4.39	4.34
cis_mRNA_up10k	9669	NA	LTCONS_00053921	MTCONS_00052327	chr4	656318	658662	1748	-	chr4	668330	675908	924	+	*		-0.9172	-1	0.82	1.32	0.8	2.19	1.41	2.78
cis_mRNA_overlap	0	mRNA	n386176	MTCONS_00028909	chr17	20224487	20305504	4204	+	chr17	20241750	20297529	4521	+		-1384.95	-0.8351	-1	0.95	1.19	0.54	1.79	1.52	1.82
cis_mRNA_up10k	3301	NA	n338432	MTCONS_00021554	chr14	85991477	85996332	1747	-	chr14	85999632	86096550	9774	+	*		0.8986	1	0.38	0.81	0.35	0.87	1.4	0.36
tran	NA	NA	n367022	MTCONS_00019166	chr4	129504154	129522555	482	-	chr13	42614826	42817094	17567	+		-304.55	0.9987	1	0.08	0.07	0.14	2.73	2.69	3.13
tran	NA	NA	n367022	MTCONS_00014101	chr4	129504154	129522555	482	-	chr11	65540198	65549058	8762	-		-603.35	-0.8107	-1	0.08	0.07	0.14	3.26	3.79	3.04
tran	NA	NA	n325325	MTCONS_00033841	chr2	43113876	43120184	786	-	chr19	17325517	17330638	3216	+		-709.04	0.9398	1	0.38	0.56	0.53	2.25	2.56	2.41
tran	NA	NA	n325325	MTCONS_00002255	chr2	43113876	43120184	786	-	chr1	150266269	150283205	2889	+		-936.67	0.9083	1	0.38	0.56	0.53	9.78	10.32	10.02
tran	NA	NA	n377691	MTCONS_00036975	chr12	66002251	66007009	874	-	chr19	56891224	56904973	6027	-		-690.85	0.9612	1	0.23	0.43	0.58	1.17	1.41	1.46
tran	NA	NA	n377691	MTCONS_00035019	chr12	66002251	66007009	874	-	chr19	56879504	56894634	6776	+		-783	0.8465	1	0.23	0.43	0.58	1.61	1.63	2.01
cis_mRNA_up10k	860	NA	n372620	MTCONS_00014722	chr11	118662550	118665346	1419	+	chr11	118618459	118661691	6170	-	*		-0.6541	-0.866	0.15	0.13	0.13	24.16	27.02	24.73

tran	NA	NA	n365365	MTCONS_00047652	chr2	2886700	2898250	464	-	chr22	30771653	30783401	3679	-		-352.78	-0.9526	-1	0.49	0.36	0.29	4.58	5.95	6.02
tran	NA	NA	n363466	MTCONS_00013723	chr1	121166923	121171134	496	+	chr11	46698581	46722200	3475	-		-343.35	0.9917	1	0.08	0.14	0.27	3.38	4.64	6.31
tran	19249425	NA	LTCONS_00031237	MTCONS_00031605	chr17	43590814	43600904	6073	-	chr17	62850328	62915584	3402	-		-1698.7	0.6499	1	1.1	1.02	1.74	1.24	0.84	1.28
tran	NA	NA	n325507	MTCONS_00055699	chr2	307912	326465	845	+	chr5	23951457	23981154	1761	+		-691.28	-0.866	-0.866	0	0.04	0.08	1.83	0.06	0.06
cis_mRNA_up10k	17	NA	n342771	MTCONS_00012278	chr11	68658747	68671303	2244	-	chr11	68671319	68708172	5138	+	*		-0.7751	-1	0.08	0.19	0.23	1.59	1.5	0.79
cis_mRNA_overlap	0	mRNA	n379304	MTCONS_00052934	chr4	68566998	68588223	1445	+	chr4	68566991	68603076	6309	+	*		-0.9815	-1	0.26	0.95	1.38	2.82	2.4	2.29
cis_mRNA_up10k	365	NA	n379034	MTCONS_00002789	chr1	173834387	173837129	583	-	chr1	173837493	173872659	19182	+	*		0.8735	1	0.59	0.61	1.74	3.47	3.75	4.03
tran	2748305	NA	n405570	MTCONS_00047703	chr22	29833004	29838118	5115	-	chr22	32586422	32600863	1651	-		-1651.63	-0.9998	-1	5.21	3.97	0.83	0.31	0.74	1.92
cis_mRNA_overlap	0	Lnc-CompleteIn-mRNAIntron	n339656	MTCONS_00044477	chr20	33434148	33435306	1159	-	chr20	33432052	33460664	8075	-	*		0.7498	1	0.3	0.96	0.94	6.4	11.12	7.82
tran	NA	NA	n381167	MTCONS_00033686	chr1	95628775	95699538	2447	-	chr19	11998621	12032411	17275	+		-1380.16	0.8374	1	1.04	1.23	1.09	1.66	3.92	3.42
tran	NA	NA	n381167	MTCONS_00072661	chr1	95628775	95699538	2447	-	chr9	40770255	40789853	5594	-		-1731.35	0.9999	1	1.04	1.23	1.09	2.05	2.71	2.23
tran	NA	NA	n381167	MTCONS_00033685	chr1	95628775	95699538	2447	-	chr19	11998607	12032411	17331	+		-1380.16	-0.8237	-1	1.04	1.23	1.09	4.11	2.39	2.73
tran	NA	NA	n381167	MTCONS_00036294	chr1	95628775	95699538	2447	-	chr19	40528842	40562147	24857	-		-1279.21	0.9958	1	1.04	1.23	1.09	6.91	8.47	7.45
cis_mRNA_overlap	0	Lnc-AntiOverlap-mRNA	n410895	MTCONS_00008486	chr10	75556272	75561157	1460	-	chr10	75544223	75561657	7284	+	*		0.9537	1	0.9	1.02	1.17	2.2	2.32	2.93
cis_mRNA_dw20k	1825	NA	n386071	MTCONS_00022588	chr14	73075742	73079803	4062	-	chr14	73081627	73360809	5528	-	*		0.9765	1	2.25	2.98	1.87	2.62	3.2	1.96
cis_mRNA_dw20k	5929	NA	LTCONS_00027282	MTCONS_00025837	chr16	3176446	3184883	5587	-	chr16	3162563	3170518	2726	+	*		1	1	2.2	2.58	2.57	2.78	4.29	4.26
tran	21467623	NA	n383218	MTCONS_00029436	chr17	66097696	66132068	11191	+	chr17	44450145	44630074	6353	+		-5325.16	-1	-1	2.28	0.65	1.12	1.06	1.58	1.43
cis_mRNA_overlap	0	Lnc-CompleteIn-mRNAExon	n410596	MTCONS_00062357	chr6	31497996	31510252	1939	-	chr6	31497996	31510252	2160	-		-1003.42	-0.9999	-1	28.67	31.25	1.76	2.04	0.24	17.6
tran	NA	NA	n410596	MTCONS_00063903	chr6	31497996	31510252	1939	-	chr6_mann_hap4	2847205	2851206	587	-		-379.76	0.9973	1	28.67	31.25	1.76	49.02	53.82	24.42
tran	NA	NA	n410596	MTCONS_00063849	chr6	31497996	31510252	1939	-	chr6_cox_hap2	3013945	3019925	1152	-		-707.13	0.866	1	28.67	31.25	1.76	0.44	1.02	0
tran	NA	NA	n338724	MTCONS_00066703	chr2	27579113	27583123	3060	+	chr7	47994874	48019266	10405	-		-4032.61	0.9933	1	5.98	6.25	6.29	7.11	8.45	8.88
tran	NA	NA	n379169	MTCONS_00036176	chr17	70026795	70034406	546	+	chr19	37305033	37328908	6621	-		-471.11	-0.9187	-1	0.08	0.28	0.43	1.21	0.73	0.71
tran	NA	NA	n379169	MTCONS_00047443	chr17	70026795	70034406	546	+	chr22	21827257	21871780	3677	-		-641.01	0.9097	1	0.08	0.28	0.43	1.84	3.15	3.17
cis_mRNA_dw20k	12008	NA	n365737	MTCONS_00039343	chr2	181940169	181941195	360	+	chr2	181845766	181928162	1543	+	*		0.9984	1	0.54	0.49	0.68	41.26	39.38	48.34
cis_mRNA_dw20k	12008	NA	n365737	MTCONS_00039342	chr2	181940169	181941195	360	+	chr2	181845766	181928162	1635	+	*		0.9896	1	0.54	0.49	0.68	20.02	15.82	26.5
cis_mRNA_overlap	0	mRNA	LTCONS_00025850	MTCONS_00025848	chr16	3331264	3341617	6960	+	chr16	3331155	3341617	5520	+		-3337.21	0.8417	1	0.91	0.89	0.78	0.36	0.2	0.12
tran	NA	NA	n367011	MTCONS_00015840	chr4	106058437	106061776	549	-	chr12	54674445	54681404	3966	+		-262.03	-0.9947	-1	0.14	0.06	0.12	6.1	8.43	6.45
tran	NA	NA	n335589	MTCONS_00060963	chr19	58904722	58906169	351	+	chr6	116575285	116759442	4648	+		-733.2	0.9906	1	0.13	0	0.29	1.8	1.59	1.96
cis_mRNA_dw20k	8717	NA	n407453	MTCONS_00039755	chr2	220110201	220115059	3118	+	chr2	220094479	220101485	2901	+	*		-1	-1	3.91	2.83	3.87	6.07	8.9	6.2
tran	NA	NA	n377403	MTCONS_00076097	chr22	46271879	46283628	805	-	chrX	103343898	103357081	6149	-		-783.47	0.6862	1	0.13	0.12	0	1.74	1.36	1.28
cis_mRNA_overlap	0	Lnc-AntiOverlap-mRNA	n407421	MTCONS_00061020	chr6	125229392	125284173	5342	-	chr6	125283144	125414026	12119	+	*		0.938	1	0.09	0.11	0.17	5.21	5.61	5.92
tran	NA	NA	n407519	MTCONS_00047278	chr6	29694378	29716826	689	-	chr22	51222100	51243982	7214	+		-406.53	0.9753	1	0	0.21	0.04	5.95	8.76	7.06
tran	NA	NA	n339695	MTCONS_00037945	chr3	172413336	172413925	590	-	chr2	62132721	62373442	11032	+		-498.39	0.8862	1	0.06	0.25	0.15	2.46	3.22	2.47
tran	NA	NA	n325789	MTCONS_00003977	chr13	29344750	29360542	913	+	chr1	2294444	2323088	9762	-		-472.7	0.9512	1	10.5	16.17	13.1	6.94	8.53	8.11
tran	NA	NA	n325789	MTCONS_00036294	chr13	29344750	29360542	913	+	chr19	40528842	40562147	24857	-		-1041.62	0.9918	1	10.5	16.17	13.1	6.91	8.47	7.45
tran	41132	NA	LTCONS_00015662	MTCONS_00015664	chr12	49523295	49580616	211	+	chr12	49621747	49667113	1771	+		-471	0.9755	1	0.42	0	0.37	1.48	0	0.99
tran	41125	NA	LTCONS_00015662	MTCONS_00015663	chr12	49523295	49580616	211	+	chr12	49621740	49667113	1660	+		-471	0.9457	1	0.42	0	0.37	6.66	1.89	4.62
tran	NA	NA	n344977	MTCONS_00031083	chr8	103698638	103742333	645	+	chr17	40190058	40196297	4596	-		-481.29	0.7908	1	0.28	0.45	0.19	2.26	2.3	1.43
cis_mRNA_up10k	110	NA	n380325	MTCONS_00013725	chr11	46867963	46895064	731	+	chr11	46765049	46867854	6900	-	*		-0.9581	-1	0.3	0.19	0.12	2.74	4.61	7.97
tran	23075125	NA	LTCONS_00005798	MTCONS_00002090	chr1	120831982	120838604	4843	-	chr1	143913728	144055455	2945	+		-1608.61	0.9982	1	1.28	1.56	1.78	0.84	1.03	1.15
tran	NA	NA	n387042	MTCONS_00036272	chr17	894694	946816	838	+	chr19	39514209	39523200	2778	-		-794.5	-0.9867	-0.866	0.04	0.04	0.15	4.48	4.56	4.1
tran	NA	NA	n387042	MTCONS_00037005	chr17	894694	946816	838	+	chr19	58201202	58220602	12336	-		-755.89	0.6719	0.866	0.04	0.04	0.15	0.3	0.16	0.34
cis_mRNA_overlap	0	mRNA	LTCONS_00034015	MTCONS_00034018	chr19	24216220	24224139	4926	+	chr19	24216220	24312694	4162	+	*		0.9895	1	1.05	1.16	0.4	2.23	2.79	0.82
cis_mRNA_up10k	6359	NA	LTCONS_00030148	MTCONS_00030144	chr17	624454	635582	1311	-	chr17	435533	618096	2781	-	*		0.9923	1	1.28	1.43	0.92	3.15	3.18	3.01
tran	1305730	NA	n406127	MTCONS_00072661	chr9	39443814	39464526	4028	-	chr9	40770255	40789853	5594	-		-1769.22	0.878	1	0.44	0.86	0.74	2.05	2.71	2.23
cis_mRNA_overlap	0	mRNA	n382224	MTCONS_00021703	chr14	96829894	96831336	1443	+	chr14	96829825	96854844	3426	+	*		-0.8205	-1	1.3	1.45	1.22	0.55	0.45	1.47
cis_mRNA_up10k	189	NA	n382224	MTCONS_00022860	chr14	96829894	96831336	1443	+	chr14	96747595	96829706	18144	-	*		0.9876	1	1.3	1.45	1.22	1.2	1.38	1.03
cis_mRNA_overlap	0	mRNA	n382224	MTCONS_00021702	chr14	96829894	96831336	1443	+	chr14	96829825	96854844	3393	+	*		0.8048	1	1.3	1.45	1.22	1.71	1.83	0.1
tran	NA	NA	n380358	MTCONS_00025783	chr11	82783146	82803380	340	+	chr16	2525125	2555778	7990	+		-247.43	0.8942	1	0	0.4	0.91	2.77	3.14	3.2
cis_mRNA_up10k	4268	NA	n335497	MTCONS_00059430	chr6	3059609	3059820	212	-	chr6	3064087	3084826	2348	+	*		-0.8231	-1	0.62	0	0.19	1.26	1.86	1.35
tran	NA	NA	n324400	MTCONS_00036294	chr6	126540032	126565078	583	+	chr19	40528842	40562147	24857	-		-367.62	0.9804	1	0.7	0.95	0.74	6.91	8.47	7.45
tran	NA	NA	n325833	MTCONS_00047711	chr11	5182847	5185114	522	+	chr22	32896532	33448559	15401	-		-395.9	0.7138	1	0.2	0.32	0.19	2.72	2.94	1.9
tran	3778770	NA	n333313	MTCONS_00036295	chr19	44341247	44351486	480	+	chr19	40528842	40562478	15125	-		-239.55	0.9716	1	0	0.2	0.21	0.26	0.41	0.47
tran	NA	NA	LTCONS_00054345	MTCONS_00033686	chr4	57198019	57211157	9096	-	chr19	11998621	12032411	17275	+		-3672.42	0.999	1	10.98	12.72	12.26	1.66	3.92	3.42
tran	NA	NA	LTCONS_00054345	MTCONS_00033685	chr4	57198019	57211157	9096	-	chr19	11998607	12032411	17331	+		-3672.42	-0.9975	-1	10.98	12.72	12.26	4.11	2.39	2.73
tran	NA	NA	LTCONS_00054345	MTCONS_00036294	chr4	57198019	57211157	9096	-	chr19	40528842	40562147	24857	-		-3694.22	0.9041	1	10.98	12.72	12.26	6.91	8.47	7.45
tran	NA	NA	LTCONS_00048126	MTCONS_00032778	chr22	22239728	22240218	491	.	chr18	11883141	11908796	4433	-		-953.53	-0.8477	-1	0.3	0.07	0	0.15	0.22	0.41
tran	1621774	NA	n387638	MTCONS_00066942	chr7	76610139	76673095	1505	+	chr7	74921877	74988366	1808	-		-768.71	0.9719	1	0.05	0.09	0.03	5.02	5.8	4.1
tran	NA	NA	LTCONS_00072235	MTCONS_00076760	chr9	14430	30786	14155	-	chrY	59354140	59358344	1999	+		-1565.18	-0.9944	-1	1.89	0.94	1.71	5.13	12.17	7.17
cis_mRNA_up10k	2185	NA	LTCONS_00025600	MTCONS_00024148	chr15	84838213	84838558	346	.	chr15	84840742	84878025	13375	+	*		-0.8484	-1	0.68	0.41	0.71	3.42	3.94	2.61
tran	NA	NA	n372399	MTCONS_00072629	chr11	18230685	18233054	2078	+	chr9	37887305	37904061	1941	-		-872.06	0.9397	1	1.57	2	2.08	8.5	8.93	9.32
tran	NA	NA	n325082	MTCONS_00003977	chr20	5360638	5361249	442	-	chr1	2294444	2323088	9762	-		-626.93	0.9874	1	18.94	27.56	24.05	6.94	8.53	8.11
tran	NA	NA	n325082	MTCONS_00039983	chr20	5360638	5361249	442	-	chr2	238536213	238694823	8109	+		-612.27	0.9562	1	18.94	27.56	24.05	1.72	3.49	3.26
cis_mRNA_overlap	0	Lnc-AntiOverlap-mRNA	n341440	MTCONS_00019701	chr13	111766159	111768025	1239	-	chr13	111767624	111958081	5346	+	*		0.6758	0.866	1.66	1.88	1.71	0	0.03	0.03
cis_mRNA_overlap	0	Lnc-AntiOverlap-mRNA	LTCONS_00050362	MTCONS_00048345	chr3	14984230	14989234	4073	-	chr3	14987670	15090876	10165	+	*		-0.9485	-1	32.66	34.99	32.63	0.35	0.25	0.4

cis_mRNA_overlap	0	Lnc-AntiOverlap-mRNA	LTCONS_00050362	MTCONS_00048346	chr3	14984230	14989234	4073	-	chr3	14988983	15090876	8622	+	*		-0.9962	-1	32.66	34.99	32.63	3.58	2.75	3.68
tran	221631842	NA	n326200	MTCONS_00003977	chr1	223954929	224006568	1530	+	chr1	2294444	2323088	9762	-		-998.37	0.9997	1	4.81	6.34	5.9	6.94	8.53	8.11
cis_mRNA_dw20k	12	NA	n337940	MTCONS_00005026	chr1	53704282	53708455	1181	+	chr1	53708466	53793821	6840	-	*		-0.8983	-1	0.15	0.16	0.37	6.68	5.43	4.05
cis_mRNA_dw20k	476	NA	n333363	MTCONS_00059455	chr6	3305107	3306062	956	+	chr6	3258996	3304632	2052	+	*		0.9968	1	0.78	0.88	0.26	1.45	1.49	0.98
tran	NA	NA	n366269	MTCONS_00047711	chr3	97910049	97915894	1123	+	chr22	32896532	33448559	15401	-		-1077.04	-0.9969	-1	0.09	0.03	0.24	2.72	2.94	1.9
tran	NA	NA	n376412	MTCONS_00072661	chr19	35339987	35351417	807	-	chr9	40770255	40789853	5594	-		-460.59	-0.9417	-1	1.23	0.83	1	2.05	2.71	2.23
tran	5177426	NA	n376412	MTCONS_00036294	chr19	35339987	35351417	807	-	chr19	40528842	40562147	24857	-		-779.81	-0.9658	-1	1.23	0.83	1	6.91	8.47	7.45
cis_mRNA_dw20k	16320	NA	n337633	MTCONS_00074021	chrX	15803839	15805712	1870	-	chrX	15756412	15787520	1212	+	*		-0.9584	-1	0.19	0.07	0.11	0.59	0.88	0.71
tran	NA	NA	n410068	MTCONS_00063764	chr10	15196721	15197346	626	-	chr6	24976618	24977395	778	.		-329.91	0.6458	1	0.17	0.16	0.1	3.55	2.27	2.2
tran	NA	NA	n369769	MTCONS_00071422	chr7	39820783	39821711	708	+	chr9	98637246	98780791	10926	+		-616.26	0.9545	1	0.76	1.68	1.55	6.78	9.15	8.15
cis_mRNA_overlap	0	Lnc-CompleteIn-mRNAIntron	n341787	MTCONS_00071788	chr9	128664233	128666749	2516	+	chr9	128508464	128729859	4118	+	*		0.9899	1	1.21	2.03	0.28	5.16	7.11	1.44
tran	NA	NA	n375451	MTCONS_00039982	chr17	12453285	12540504	2605	+	chr2	238536213	238694823	8202	+		-1326.53	0.7609	1	0.42	0.17	0.16	8.48	8.35	8.06
cis_mRNA_dw20k	6642	NA	n337939	MTCONS_00047347	chr22	19159237	19160345	1109	+	chr22	19166986	19279239	5401	-	*		0.9923	1	1.84	1.79	1.53	0.25	0.18	0
cis_mRNA_overlap	0	Lnc-CompleteIn-mRNAIntron	n324316	MTCONS_00061462	chr6	163927605	163929187	600	+	chr6	163835604	164000803	10685	+	*		0.8065	1	0.37	0.5	0.49	4	5.25	4.43
tran	NA	NA	n385437	MTCONS_00072661	chr8	122966847	123139423	822	-	chr9	40770255	40789853	5594	-		-875.68	0.9876	1	0.04	0.33	0.16	2.05	2.71	2.23
tran	NA	NA	n324498	MTCONS_00003977	chr6	53355094	53355491	356	-	chr1	2294444	2323088	9762	-		-209.82	0.954	1	0.34	0.49	0.41	6.94	8.53	8.11
tran	NA	NA	n324498	MTCONS_00036294	chr6	53355094	53355491	356	-	chr19	40528842	40562147	24857	-		-604.01	0.9906	1	0.34	0.49	0.41	6.91	8.47	7.45
cis_mRNA_dw20k	2584	NA	n410582	MTCONS_00059430	chr6	3087409	3112321	4060	+	chr6	3064087	3084826	2348	+	*		0.7868	1	0.06	0.1	0.09	1.26	1.86	1.35
cis_mRNA_up10k	186	NA	n375352	MTCONS_00027304	chr16	3661780	3693096	955	+	chr16	3629261	3661595	9627	-	*		-0.9951	-1	1.36	0.54	0.2	0.64	0.84	0.89
cis_mRNA_dw20k	622	NA	n338382	MTCONS_00002818	chr1	178449742	178450340	599	+	chr1	178340400	178449121	11191	+	*		0.9999	1	0.37	0.45	0.66	6.33	7.57	10.6
tran	NA	NA	n378968	MTCONS_00033686	chr21	25801054	25862621	2909	+	chr19	11998621	12032411	17275	+		-1292.88	0.9995	1	1.31	1.51	1.46	1.66	3.92	3.42
tran	NA	NA	n378968	MTCONS_00033685	chr21	25801054	25862621	2909	+	chr19	11998607	12032411	17331	+		-1292.88	-0.9985	-1	1.31	1.51	1.46	4.11	2.39	2.73
tran	NA	NA	n378968	MTCONS_00036294	chr21	25801054	25862621	2909	+	chr19	40528842	40562147	24857	-		-1371.65	0.8974	1	1.31	1.51	1.46	6.91	8.47	7.45
tran	43329624	NA	n407566	MTCONS_00024565	chr15	78206559	78219188	3117	-	chr15	34671269	34876936	7605	-		-1892.21	-0.9554	-1	0.01	0.04	0.03	0.83	0.41	0.66
cis_mRNA_up10k	946	NA	LTCONS_00067909	MTCONS_00065615	chr7	128114894	128115838	945	.	chr7	128116783	128144427	3837	+	*		0.9905	1	0.75	0.92	0.3	12.6	13.34	11.48
tran	NA	NA	n381140	MTCONS_00033686	chr1	64571008	64636980	2186	-	chr19	11998621	12032411	17275	+		-1015.53	0.9927	1	1.32	1.42	1.41	1.66	3.92	3.42
tran	NA	NA	n381140	MTCONS_00033685	chr1	64571008	64636980	2186	-	chr19	11998607	12032411	17331	+		-1015.53	-0.9953	-1	1.32	1.42	1.41	4.11	2.39	2.73
tran	NA	NA	n381140	MTCONS_00036294	chr1	64571008	64636980	2186	-	chr19	40528842	40562147	24857	-		-1023.3	0.8205	1	1.32	1.42	1.41	6.91	8.47	7.45
cis_mRNA_overlap	0	Lnc-CompleteIn-mRNAExon	n341106	MTCONS_00012629	chr11	102980160	103024200	3409	+	chr11	102980108	103350591	13709	+		-1511.28	0.9939	1	0	0.16	0.21	0.81	2.63	2.9
cis_mRNA_overlap	0	AntiCompleteIn-mRNAIntron	n326161	MTCONS_00003694	chr1	237499648	237527860	448	-	chr1	237205507	237997447	16662	+	*		0.9707	1	0.17	0.6	0.08	1.6	2.89	0.75
cis_mRNA_dw20k	15454	NA	LTCONS_00060612	MTCONS_00062853	chr6	79890102	79895159	3870	+	chr6	79910612	79944497	1406	-	*		-0.8883	-1	1.24	0.55	0.54	0.27	0.45	0.61
tran	143191109	NA	n381240	MTCONS_00003977	chr1	145514196	145515899	1011	-	chr1	2294444	2323088	9762	-		-1202.67	0.9996	1	1.09	1.99	1.73	6.94	8.53	8.11
cis_mRNA_overlap	0	Lnc-AntiOverlap-mRNA	n379320	MTCONS_00035138	chr19	58768639	58790174	2270	-	chr19	58739924	58776859	5782	+	*		-0.9523	-1	0.47	0.71	0.98	1.62	1.21	1.07
cis_mRNA_dw20k	1851	NA	n365108	MTCONS_00040500	chr2	26521475	26529191	2513	+	chr2	26531041	26572194	7045	-	*		0.9912	0.866	0.07	0	0.06	0.01	0	0.01
tran	NA	NA	n325585	MTCONS_00033686	chr16	61187777	61189854	558	+	chr19	11998621	12032411	17275	+		-680.14	0.7515	1	3.15	5.96	3.5	1.66	3.92	3.42
tran	NA	NA	n325585	MTCONS_00033685	chr16	61187777	61189854	558	+	chr19	11998607	12032411	17331	+		-680.14	-0.7351	-1	3.15	5.96	3.5	4.11	2.39	2.73
tran	NA	NA	n325585	MTCONS_00036294	chr16	61187777	61189854	558	+	chr19	40528842	40562147	24857	-		-705.02	0.9729	1	3.15	5.96	3.5	6.91	8.47	7.45
cis_mRNA_overlap	0	Lnc-AntiOverlap-mRNA	n338826	MTCONS_00014977	chr12	248700	258281	4913	-	chr12	175943	275884	3905	+	*		0.7488	0.866	0.05	0.04	0.05	0.65	0.54	0.88
cis_mRNA_overlap	0	Lnc-AntiOverlap-mRNA	n338826	MTCONS_00014978	chr12	248700	258281	4913	-	chr12	175971	287626	7037	+	*		-0.9625	-0.866	0.05	0.04	0.05	1.29	1.47	1.22
tran	NA	NA	n365887	MTCONS_00033686	chr3	87138430	87147851	4299	+	chr19	11998621	12032411	17275	+		-5681.54	0.9993	1	26.54	31.31	30.09	1.66	3.92	3.42
tran	NA	NA	n365887	MTCONS_00033685	chr3	87138430	87147851	4299	+	chr19	11998607	12032411	17331	+		-5681.54	-0.9981	-1	26.54	31.31	30.09	4.11	2.39	2.73
tran	NA	NA	n365887	MTCONS_00036294	chr3	87138430	87147851	4299	+	chr19	40528842	40562147	24857	-		-6581.64	0.9001	1	26.54	31.31	30.09	6.91	8.47	7.45
tran	136271	NA	n346273	MTCONS_00047443	chr22	22008050	22009208	901	+	chr22	21827257	21871780	3677	-		-947.6	0.986	1	0.79	1.51	1.68	1.84	3.15	3.17
tran	NA	NA	n333187	MTCONS_00063866	chr6	29795825	29796595	544	+	chr6_dbb_hap3	1203642	1207050	1590	+		-436.3	0.9442	1	0.14	0.06	0.09	18.36	0.43	12.42
cis_mRNA_overlap	0	mRNA	n339927	MTCONS_00033203	chr18	74072230	74207146	6031	-	chr18	74069637	74207223	8590	-		-3826.06	0.9329	0.866	2.32	1.97	1.36	1.09	1.09	0.61
tran	69561	NA	LTCONS_00036843	MTCONS_00036852	chr19	53467065	53541136	7498	-	chr19	53610696	53636173	3062	-		-1393.8	0.989	1	0.28	0.69	0.97	1.13	1.45	1.57
tran	69561	NA	LTCONS_00036843	MTCONS_00036853	chr19	53467065	53541136	7498	-	chr19	53610696	53636173	2720	-		-1197.37	-0.9867	-1	0.28	0.69	0.97	1.19	0.76	0.24
tran	NA	NA	n323887	MTCONS_00031084	chrX	41019936	41020975	835	+	chr17	40190058	40202750	4056	-		-487.28	-0.8555	-0.866	0.3	0.19	0.19	2.79	3.06	3.35
tran	NA	NA	n344694	MTCONS_00047712	chr6	114730053	114777358	2783	-	chr22	32896532	33454377	15152	-		-2464.89	-0.922	-1	0	0.01	0.02	5.15	5.06	4.49
tran	NA	NA	n380928	MTCONS_00067141	chr12	43373057	43390289	552	-	chr7	97844756	97881576	5732	-		-347.46	-0.7196	-0.866	0.16	0.06	0.06	9.65	10.64	13.13
tran	NA	NA	n346009	MTCONS_00035158	chr9	130349340	130354502	849	+	chr19	58919904	58934213	6618	+		-889.18	0.8443	1	0.13	0.3	0.23	0.37	0.67	0.39
tran	NA	NA	n346009	MTCONS_00035157	chr9	130349340	130354502	849	+	chr19	58919904	58934213	6915	+		-902.5	0.89	1	0.13	0.3	0.23	1.05	1.79	1.17
tran	NA	NA	n346009	MTCONS_00017040	chr9	130349340	130354502	849	+	chr12	6774462	6798083	4725	-		-860.24	-0.9181	-1	0.13	0.3	0.23	0.6	0	0.01
cis_mRNA_up10k	21	NA	n375715	MTCONS_00029228	chr17	38276065	38278113	1633	-	chr17	38278133	38293738	5646	+	*		-0.8827	-1	2.06	2.21	2	13.17	12.53	14.8
tran	NA	NA	n408121	MTCONS_00076615	chrX	192991	220022	2087	+	chrY	148035	171427	6735	+		-1695.85	0.9734	1	0.22	0.67	0.49	0.19	0.35	0.32
tran	NA	NA	n407042	MTCONS_00072236	chr1	14362	29370	1769	-	chr9	14430	30786	3251	-		-1280.28	0.9828	1	0.72	0	2.58	0.08	0.04	0.13
cis_mRNA_up10k	179	NA	n365462	MTCONS_00038000	chr2	65273704	65283317	1912	-	chr2	65283495	65304083	4762	+	*		0.9997	1	0.15	0.39	0.11	1.22	1.42	1.18
cis_mRNA_up10k	179	NA	n365462	MTCONS_00038002	chr2	65273704	65283317	1912	-	chr2	65283495	65314241	5957	+	*		-0.7981	-1	0.15	0.39	0.11	1.41	1.18	2.02
cis_mRNA_dw20k	1992	NA	n340810	MTCONS_00059411	chr6	3025025	3027659	2635	+	chr6	2988201	3023034	1970	+	*		0.9367	1	1.26	1.34	1.21	0.77	1.79	0.73
cis_mRNA_up10k	43	NA	LTCONS_00047364	MTCONS_00046381	chr22	19702311	19704701	2391	-	chr22	19704743	19712297	4509	+	*		0.8843	0.866	0	0.03	0.03	1.56	3.85	2.78
cis_mRNA_overlap	0	Lnc-AntiOverlap-mRNA	LTCONS_00047364	MTCONS_00046380	chr22	19702311	19704701	2391	-	chr22	19701987	19712297	3251	+	*		-0.8076	-0.866	0	0.03	0.03	13.69	0	8.12
cis_mRNA_overlap	0	mRNA	n324531	MTCONS_00062509	chr6	37619307	37622509	735	-	chr6	37599399	37666095	10963	-	*		0.9665	1	0.05	0.22	0.27	0.69	1.1	1.45
tran	NA	NA	n342231	MTCONS_00022742	chrX	75109183	75109872	690	+	chr14	89622296	90085531	8146	-		-345.44	-0.9949	-1	0.16	0.38	0.19	3.4	2.33	3.37

tran	NA	NA	n339200	MTCONS_00017912	chr16	47563101	47563827	494	+	chr12	63952662	64062424	11449	-		-447.53	0.9967	0.866	0.08	0.14	0.14	2.01	2.92	3.01
tran	NA	NA	n339200	MTCONS_00032982	chr16	47563101	47563827	494	+	chr18	45335336	45458502	17869	-		-298.56	-0.9663	-0.866	0.08	0.14	0.14	0.51	0.36	0.4
tran	NA	NA	LTCONS_00023162	MTCONS_00062442	chr15	23393042	23681407	667	+	chr6	34231228	34360518	10114	-		-652.98	0.9523	1	2.74	2.78	6.02	47.14	47.98	49.75
cis_mRNA_up10k	8569	NA	n375661	MTCONS_00030241	chr17	3608266	3608812	316	-	chr17	3566187	3599698	6213	-	*		0.9305	1	0.76	0.12	0.79	1.02	0.33	1.49
cis_mRNA_dw20k	1773	NA	n375951	MTCONS_00032467	chr18	52559875	52562337	1040	+	chr18	52385083	52558103	2294	+	*		0.9796	1	0.95	6.48	4.8	0.84	5.19	4.72
cis_mRNA_dw20k	1773	NA	n375951	MTCONS_00032468	chr18	52559875	52562337	1040	+	chr18	52385097	52558103	2284	+	*		0.9997	1	0.95	6.48	4.8	0.35	2.16	1.65
tran	NA	NA	n325644	MTCONS_00033686	chr13	102193341	102195575	1947	+	chr19	11998621	12032411	17275	+		-3161.91	0.9847	1	19.5	24.03	23.84	1.66	3.92	3.42
tran	NA	NA	n325644	MTCONS_00033685	chr13	102193341	102195575	1947	+	chr19	11998607	12032411	17331	+		-3161.91	-0.9887	-1	19.5	24.03	23.84	4.11	2.39	2.73
tran	NA	NA	n325644	MTCONS_00036294	chr13	102193341	102195575	1947	+	chr19	40528842	40562147	24857	-		-3214.31	0.7886	1	19.5	24.03	23.84	6.91	8.47	7.45
tran	NA	NA	n344511	MTCONS_00047711	chr16	65266702	65267306	605	+	chr22	32896532	33448559	15401	-		-452.75	0.9966	1	1.02	1.26	0.43	2.72	2.94	1.9
tran	85800	NA	LTCONS_00025324	MTCONS_00024148	chr15	84963824	85044102	1916	-	chr15	84840742	84878025	13375	+		-1634.34	0.9887	1	2.59	2.86	1.8	3.42	3.94	2.61
cis_mRNA_dw20k	3	NA	LTCONS_00061053	MTCONS_00063229	chr6	127587732	127609855	8819	+	chr6	127609857	127653215	2306	-	*		-0.915	-1	1.83	2.23	1.99	0.69	0	0.16
tran	NA	NA	n387143	MTCONS_00047278	chr2	114379305	114384715	511	-	chr22	51222100	51243982	7214	+		-286.86	-0.9297	-1	0.42	0.31	0.34	5.95	8.76	7.06
cis_mRNA_overlap	0	mRNA	n384200	MTCONS_00047279	chr22	51222157	51238064	1447	+	chr22	51222100	51243982	7371	+		-736.69	-0.8805	-1	3.47	1.47	4.36	2.31	2.56	1.58
tran	NA	NA	n373629	MTCONS_00066703	chr13	109962483	109964121	664	+	chr7	47994874	48019266	10405	-		-1240.69	0.9424	1	0.83	1.08	1.38	7.11	8.45	8.88
cis_mRNA_up10k	476	NA	n372244	MTCONS_00009272	chr10	128591477	128593410	1817	-	chr10	128593885	128931989	7288	+	*		0.9807	1	0.23	0.66	0.1	0.42	1.69	0.38
cis_mRNA_up10k	476	NA	n372244	MTCONS_00009271	chr10	128591477	128593410	1817	-	chr10	128593885	128931989	8294	+	*		0.9988	1	0.23	0.66	0.1	0.82	1.86	0.42
tran	NA	NA	n324484	MTCONS_00038002	chr6	71161311	71165841	397	+	chr2	65283495	65314241	5957	+		-389.68	-0.9643	-0.866	0.43	0.43	0.41	1.41	1.18	2.02
cis_mRNA_dw20k	5170	NA	n406622	MTCONS_00014238	chr11	71639768	71708643	2568	+	chr11	71713812	71753181	8276	-	*		0.9966	0.866	1.29	2.78	1.14	5.02	6.32	5.02
cis_mRNA_overlap	0	Lnc-CompleteIn-mRNAExon	n406622	MTCONS_00012320	chr11	71639768	71708643	2568	+	chr11	71639547	71714063	6955	+		-1791.54	0.9745	1	1.29	2.78	1.14	1.41	1.83	1.22
cis_mRNA_dw20k	5170	NA	n406622	MTCONS_00014239	chr11	71639768	71708643	2568	+	chr11	71713812	71791663	7467	-	*		-0.783	-1	1.29	2.78	1.14	18.38	17.19	21.2
cis_mRNA_dw20k	14759	NA	n411578	MTCONS_00042880	chr20	1305987	1359379	2479	+	chr20	1246928	1291229	6448	+	*		1	1	0.46	0.73	0.45	1.8	2.38	1.78
cis_mRNA_dw20k	15930	NA	n411578	MTCONS_00042879	chr20	1305987	1359379	2479	+	chr20	1246928	1290058	5690	+	*		-0.9933	-1	0.46	0.73	0.45	9.13	5.66	9.78
cis_mRNA_overlap	0	Lnc-CompleteIn-mRNAIntron	n339975	MTCONS_00009589	chr10	14566482	14569859	3378	-	chr10	14560554	14646331	3180	-	*		0.9108	1	0.55	0.51	0.48	9.97	7.25	7.2
tran	NA	NA	n378397	MTCONS_00013134	chr1	173834997	173836827	841	-	chr11	765030	777581	6966	-		-765.97	0.9615	1	1.09	1.5	2.76	2.55	2.94	3.34
tran	NA	NA	n378397	MTCONS_00046402	chr1	173834997	173836827	841	-	chr22	20008630	20055472	4182	+		-646.6	0.9859	1	1.09	1.5	2.76	0.97	1.3	1.8
cis_mRNA_dw20k	15510	NA	LTCONS_00059421	MTCONS_00059411	chr6	3038543	3065264	3258	+	chr6	2988201	3023034	1970	+	*		-0.9057	-1	0.24	0	0.44	0.77	1.79	0.73
cis_mRNA_up10k	135	NA	LTCONS_00062789	MTCONS_00060528	chr6	69339825	69344953	840	-	chr6	69345087	70099476	6083	+	*		0.9224	1	2.01	1.48	2.23	7.26	6.49	8.64
cis_mRNA_overlap	0	AntiCompleteIn-mRNAExon	n335652	MTCONS_00069217	chr8	145726920	145727491	572	-	chr8	145703374	145732555	6701	+		-1420.7	0.9793	1	0.39	1	0.69	1.1	1.42	1.2
cis_mRNA_dw20k	18457	NA	n408346	MTCONS_00017854	chr12	57623356	57628718	2215	+	chr12	57647174	57824682	4544	-	*		0.9085	0.866	0.11	0.92	0.45	2.5	3.35	2.5
cis_mRNA_overlap	0	Lnc-AntiOverlap-mRNA	n378123	MTCONS_00019701	chr13	111767766	111768922	493	-	chr13	111767624	111958081	5346	+		-815.83	0.6405	0.866	0.15	0.48	0.21	0	0.03	0.03
cis_mRNA_overlap	0	mRNA	n407526	MTCONS_00047279	chr22	51195514	51238065	1520	+	chr22	51222100	51243982	7371	+		-766.54	0.9747	1	2.44	2.47	1.3	2.31	2.56	1.58
cis_mRNA_up10k	104	NA	n381458	MTCONS_00008292	chr10	51780942	51827563	2177	-	chr10	51827666	51894265	5536	+	*		0.9849	1	0.33	0.29	0.48	1.78	1.18	2.8
tran	NA	NA	n371303	MTCONS_00037945	chrX	20004935	20007897	628	-	chr2	62132721	62373442	11032	+		-536.27	0.9169	1	0	0.17	0.07	2.46	3.22	2.47
cis_mRNA_dw20k	1705	NA	n384090	MTCONS_00047402	chr22	20378630	20381779	2167	+	chr22	20383483	20427848	2791	-	*		0.9852	1	3.43	4.78	2.02	0.32	0.71	0.1
cis_mRNA_dw20k	1705	NA	n384090	MTCONS_00047403	chr22	20378630	20381779	2167	+	chr22	20383483	20427848	3937	-	*		0.9355	1	3.43	4.78	2.02	2.5	2.97	0.04
tran	NA	NA	n324050	MTCONS_00033686	chr9	91506386	91524405	822	+	chr19	11998621	12032411	17275	+		-391.5	0.9484	1	3.23	4.25	3.73	1.66	3.92	3.42
tran	NA	NA	n324050	MTCONS_00033685	chr9	91506386	91524405	822	+	chr19	11998607	12032411	17331	+		-391.5	-0.9403	-1	3.23	4.25	3.73	4.11	2.39	2.73
tran	NA	NA	n324050	MTCONS_00036294	chr9	91506386	91524405	822	+	chr19	40528842	40562147	24857	-		-471.5	0.9865	1	3.23	4.25	3.73	6.91	8.47	7.45
cis_mRNA_overlap	0	Lnc-CompleteIn-mRNAExon	n342855	MTCONS_00072485	chr9	33025348	33039905	2241	-	chr9	33025348	33081406	13278	-		-1030.11	0.9449	0.866	0	0.03	0.01	0.14	0.18	0.14
tran	NA	NA	n326382	MTCONS_00009602	chr1	147843215	147843920	561	-	chr10	15147771	15210695	5166	-		-482.82	-0.9114	-1	0.33	0	0.06	0.36	0.52	0.43
tran	NA	NA	n326382	MTCONS_00034614	chr1	147843215	147843920	561	-	chr19	47249804	47266476	8769	+		-492.35	0.9387	1	0.33	0	0.06	1.15	0.57	0.86
cis_mRNA_up10k	286	NA	n386063	MTCONS_00021042	chr14	32544626	32545905	1280	-	chr14	32546190	32630855	11448	+	*		0.64	1	1.25	1.44	2.58	0.12	0.34	0.35
cis_mRNA_overlap	0	Lnc-AntiOverlap-mRNA	n386063	MTCONS_00021040	chr14	32544626	32545905	1280	-	chr14	32542224	32630855	15018	+		-2582.32	0.9912	0.866	1.25	1.44	2.58	0.17	0.17	0.31
tran	NA	NA	n387725	MTCONS_00021822	chrX	3782439	3799884	1681	-	chr14	102791829	102810753	5226	+		-1080.44	-0.9321	-1	0.94	1.77	0.75	0.58	0.34	0.84
tran	NA	NA	n387725	MTCONS_00021820	chrX	3782439	3799884	1681	-	chr14	102783531	102810753	5199	+		-1091.11	0.9971	1	0.94	1.77	0.75	0.18	0.75	0.11
cis_mRNA_overlap	0	AntiCompleteIn-mRNAIntron	n337866	MTCONS_00023841	chr15	65244342	65248504	1839	-	chr15	65211921	65251226	3440	+	*		0.9768	1	0.22	0.25	0.35	0.46	0.47	1.84
cis_mRNA_dw20k	18536	NA	n339468	MTCONS_00051343	chr3	121468132	121470030	1899	+	chr3	121488565	121553926	2476	-	*		-0.9091	-1	0.43	0.66	0.76	1.16	0.79	0
tran	NA	NA	n369612	MTCONS_00047712	chr7	125557925	125573886	837	+	chr22	32896532	33454377	15152	-		-745.01	0.8442	1	0.34	0.12	0	5.15	5.06	4.49
tran	NA	NA	n333504	MTCONS_00075496	chr7	74543562	74544386	825	+	chrX	20168029	20284780	7871	-		-417.36	0.8611	1	0.35	0.84	0.54	1.93	3.94	3.68
tran	NA	NA	n333504	MTCONS_00036591	chr7	74543562	74544386	825	+	chr19	48988524	49015089	9336	-		-495.03	0.9894	1	0.35	0.84	0.54	2.04	3.77	2.93
cis_mRNA_dw20k	2066	NA	n383026	MTCONS_00028319	chr16	87731749	87739290	6938	-	chr16	87741355	87799814	2206	-	*		0.8989	1	0.06	0.08	0.03	0.08	0.41	0
cis_mRNA_up10k	2	NA	n378950	MTCONS_00040535	chr2	27580007	27581057	536	+	chr2	27532272	27580006	7712	-	*		-0.8579	-1	0.1	0.09	0.17	0.66	1.01	0.44
cis_mRNA_overlap	0	AntiCompleteIn-mRNAIntron	LTCONS_00025416	MTCONS_00024271	chr15	91473504	91476295	2384	-	chr15	91473410	91497713	4174	+	*		-0.7434	-1	1.35	1.1	1.12	1.39	1.83	1.5
tran	NA	NA	LTCONS_00063781	MTCONS_00065329	chr6	42923995	42924739	745	.	chr7	99775538	99948836	16269	+		-568.88	0.7254	1	0.14	0.22	0.47	1.41	1.88	1.91
cis_mRNA_dw20k	12987	NA	n363372	MTCONS_00003909	chr1	1210617	1214773	389	+	chr1	1227759	1243274	8533	-	*		0.7985	1	1.23	3.09	1.48	13.98	19.76	18.13
cis_mRNA_up10k	5006	NA	n363372	MTCONS_00000083	chr1	1210617	1214773	389	+	chr1	1219778	1227752	4141	+	*		0.8787	1	1.23	3.09	1.48	0.68	1.09	0.92
cis_mRNA_overlap	0	mRNA	n363372	MTCONS_00000077	chr1	1210617	1214773	389	+	chr1	1210339	1227752	3559	+		-367.08	0.9933	1	1.23	3.09	1.48	0.36	1.37	0.37
cis_mRNA_dw20k	15358	NA	n363605	MTCONS_00000811	chr1	32814795	32816264	687	-	chr1	32757536	32799438	4973	+	*		0.9888	1	0.05	0.1	0.09	0.18	0.74	0.55
cis_mRNA_dw20k	10292	NA	n363605	MTCONS_00004620	chr1	32814795	32816264	687	-	chr1	32826555	32860062	6996	-	*		0.7458	1	0.05	0.1	0.09	1.43	2.37	1.56
cis_mRNA_overlap	0	Lnc-CompleteIn-mRNAIntron	n335719	MTCONS_00045642	chr21	47712679	47713546	868	+	chr21	47705338	47717665	1528	+	*		0.95	1	2.44	2.25	1.47	1.54	0.99	0.4
cis_mRNA_overlap	0	mRNA	n338468	MTCONS_00022858	chr14	96792025	96829678	2763	-	chr14	96747595	96800598	10098	-		-1448.5	0.9811	1	2.63	1.91	2.01	1.15	0.44	0.67
cis_mRNA_overlap	0	Lnc-CompleteIn-mRNAIntron	n337830	MTCONS_00074840	chrX	110367493	110372431	4939	+	chrX	110339375	110470582	8948	+	*		0.9922	1	10.47	15.09	7.43	17.28	20.54	13.95

cis_mRNA_overlap	0	mRNA	n346492	MTCONS_00064303	chr7	6769771	6790933	1722	+	chr7	6770022	6793507	4538	+		-993.53	0.9876	1	0.85	0.97	0.82	8.08	8.93	7.63
cis_mRNA_overlap	0	Lnc-CompleteIn-mRNAExon	n339473	MTCONS_00045300	chr21	35269911	35272163	2253	+	chr21	35014784	35272247	17062	+		-1269.56	-0.7368	-1	0.25	0.23	1.09	6.39	7.4	5.98
cis_mRNA_overlap	0	Lnc-AntiOverlap-mRNA	n375744	MTCONS_00029671	chr17	59470733	59475416	420	-	chr17	59474205	59486827	6081	+	*		0.7789	1	0	1.06	1.17	1.54	1.84	2.58
cis_mRNA_up10k	56	NA	n378143	MTCONS_00008074	chr10	31607381	31608810	456	-	chr10	31608865	31818836	7880	+	*		1	1	0.08	0.75	0.07	1.19	2.43	1.16
cis_mRNA_overlap	0	Lnc-AntiOverlap-mRNA	LTCONS_00062458	MTCONS_00060176	chr6	35225063	35227194	2132	-	chr6	35225127	35264108	5219	+		-4346.8	0.969	1	1.33	1.64	1.21	0.32	0.34	0.3
cis_mRNA_dw20k	11484	NA	n381849	MTCONS_00017288	chr12	22477346	22589990	2345	-	chr12	22601473	22698224	5350	-	*		0.8045	1	2.45	2.93	2.17	2.75	2.78	2.09
cis_mRNA_dw20k	11484	NA	n381849	MTCONS_00017287	chr12	22477346	22589990	2345	-	chr12	22601473	22672319	4825	-	*		0.9966	1	2.45	2.93	2.17	3.7	4.03	3.44
tran	869239	NA	n382701	MTCONS_00024149	chr15	85747263	85747561	299	-	chr15	84841055	84878025	11652	+		-468.13	-0.8765	-1	1.59	1.8	3.77	3.39	2.37	1.56
cis_mRNA_up10k	2008	NA	n377888	MTCONS_00000289	chr1	9739858	9747632	983	-	chr1	9749639	9789271	5938	+	*		0.8424	1	0.39	0.3	0.84	1.66	1.01	1.97
tran	18549224	NA	n377888	MTCONS_00004526	chr1	9739858	9747632	983	-	chr1	28296855	28415203	5943	-		-883.73	0.9999	1	0.39	0.3	0.84	1.48	1.22	2.89
tran	NA	NA	n339167	MTCONS_00021469	chr2	231916755	231917352	598	+	chr14	75179850	75204623	3922	+		-560.09	0.866	1	1.47	1.51	1.59	0.29	0.45	0.49
tran	NA	NA	n385992	MTCONS_00040552	chr10	75547343	75547998	656	+	chr2	27873585	27886539	5240	-		-622.25	0.6807	1	0.61	0.81	1.53	2.04	3.05	3.07
cis_mRNA_overlap	0	Lnc-CompleteIn-mRNAIntron	n385992	MTCONS_00008486	chr10	75547343	75547998	656	+	chr10	75544223	75561657	7284	+	*		0.9985	1	0.61	0.81	1.53	2.2	2.32	2.93
cis_mRNA_up10k	34	NA	LTCONS_00036172	MTCONS_00034245	chr19	37071155	37096185	2753	-	chr19	37096218	37128259	11934	+	*		0.9707	0.866	3.37	1.56	3.37	0.92	0.83	0.95
cis_mRNA_up10k	34	NA	LTCONS_00036172	MTCONS_00034244	chr19	37071155	37096185	2753	-	chr19	37096218	37128259	14238	+	*		-0.9729	-0.866	3.37	1.56	3.37	3.19	3.41	3.12
tran	NA	NA	n345581	MTCONS_00013134	chr3	5053037	5067376	907	-	chr11	765030	777581	6966	-		-845.68	1	1	0.04	0.07	0.1	2.55	2.94	3.34
cis_mRNA_dw20k	7981	NA	LTCONS_00002098	MTCONS_00002095	chr1	144166564	144219394	638	+	chr1	144146801	144158584	2483	+	*		-0.967	-1	6.91	14.4	9.25	1.52	1.26	1.38
tran	1812758	NA	LTCONS_00002098	MTCONS_00005904	chr1	144166564	144219394	638	+	chr1	146032151	146066741	4836	-		-372.31	0.9921	1	6.91	14.4	9.25	0.67	1.03	0.74
cis_mRNA_dw20k	7981	NA	LTCONS_00002098	MTCONS_00002094	chr1	144166564	144219394	638	+	chr1	144146137	144158584	2105	+	*		0.9812	1	6.91	14.4	9.25	1.16	2.07	1.6
tran	NA	NA	n380878	MTCONS_00072287	chr14	27305934	27402978	477	+	chr9	4709556	4741326	6185	-		-422.43	-0.9497	-1	0	0.21	0.08	9.84	8	9.7
tran	NA	NA	n366150	MTCONS_00033686	chr3	184264502	184274706	571	-	chr19	11998621	12032411	17275	+		-710.91	0.7832	1	1.05	1.91	1.2	1.66	3.92	3.42
tran	NA	NA	n366150	MTCONS_00064007	chr3	184264502	184274706	571	-	chr6_qbl_hap6	4499037	4514274	4383	-		-721.82	0.961	1	1.05	1.91	1.2	1.35	2.44	1.82
tran	NA	NA	n366150	MTCONS_00076535	chr3	184264502	184274706	571	-	chrX	153570168	153603006	15521	-		-447.91	0.9677	1	1.05	1.91	1.2	1.27	1.49	1.36
cis_mRNA_overlap	0	AntiCompleteIn-mRNAIntron	n325611	MTCONS_00022742	chr14	89991426	89995002	1376	+	chr14	89622296	90085531	8146	-	*		-0.9587	-1	3.79	4.33	3.96	3.4	2.33	3.37
tran	NA	NA	n385921	MTCONS_00072236	chr1	15796	18061	1101	-	chr9	14430	30786	3251	-		-865.69	0.9966	1	3.29	2.31	4.21	0.08	0.04	0.13
cis_mRNA_up10k	26	NA	n379687	MTCONS_00024573	chr15	35047285	35105124	2727	+	chr15	35043151	35047260	3065	-	*		0.9971	1	0.01	0.06	0.08	0.48	1.08	1.41
tran	NA	NA	n323796	MTCONS_00033686	chrX	145698994	145699619	436	+	chr19	11998621	12032411	17275	+		-296.62	-0.6804	-1	2.47	1.77	2.46	1.66	3.92	3.42
tran	NA	NA	n323796	MTCONS_00033685	chrX	145698994	145699619	436	+	chr19	11998607	12032411	17331	+		-296.62	0.6622	1	2.47	1.77	2.46	4.11	2.39	2.73
tran	NA	NA	n323796	MTCONS_00036294	chrX	145698994	145699619	436	+	chr19	40528842	40562147	24857	-		-276.8	-0.9443	-1	2.47	1.77	2.46	6.91	8.47	7.45
cis_mRNA_overlap	0	Lnc-CompleteIn-mRNAIntron	n339043	MTCONS_00025783	chr16	2526576	2527062	487	+	chr16	2525125	2555778	7990	+	*		0.9946	1	1.85	2.52	2.54	2.77	3.14	3.2
cis_mRNA_dw20k	11598	NA	n339043	MTCONS_00025776	chr16	2526576	2527062	487	+	chr16	2510013	2514979	2149	+	*		0.8545	1	1.85	2.52	2.54	0.24	0.35	0.48
cis_mRNA_overlap	0	Lnc-CompleteIn-mRNAIntron	n339043	MTCONS_00025780	chr16	2526576	2527062	487	+	chr16	2518074	2555778	13051	+	*		0.9997	0.866	1.85	2.52	2.54	0.68	0.88	0.88
cis_mRNA_overlap	0	Lnc-CompleteIn-mRNAExon	n341689	MTCONS_00072616	chr9	37531913	37576249	1610	-	chr9	37510386	37576360	5006	-		-1197.74	-0.6449	-0.866	1.71	1.39	1.65	1.02	1.3	1.3
tran	NA	NA	n379871	MTCONS_00074579	chr4	100175086	100196208	915	+	chrX	75392518	75414076	17491	+		-572.5	0.982	1	0.12	0.04	0	5.93	5.5	5.07
cis_mRNA_overlap	0	Lnc-AntiOverlap-mRNA	n384326	MTCONS_00049465	chr3	131044146	131100319	1768	-	chr3	130745694	131069309	2856	+	*		-0.755	-1	0	0.12	0.02	0.43	0	0.08
cis_mRNA_overlap	0	Lnc-CompleteIn-mRNAIntron	n340955	MTCONS_00013315	chr11	6491371	6492926	1556	-	chr11	6469764	6495205	4055	-	*		0.9008	1	0.24	0.41	0.34	3.59	7.62	4.34
tran	NA	NA	n346256	MTCONS_00047711	chr20	38723545	38900110	987	-	chr22	32896532	33448559	15401	-		-672.14	0.7267	1	0.32	0.6	0.29	2.72	2.94	1.9
cis_mRNA_overlap	0	Lnc-CompleteIn-mRNAIntron	n332911	MTCONS_00037945	chr2	62230727	62231282	556	+	chr2	62132721	62373442	11032	+	*		0.9667	1	0.2	0.49	0.28	2.46	3.22	2.47
tran	NA	NA	n332911	MTCONS_00032674	chr2	62230727	62231282	556	+	chr18	77794346	77830757	3161	+		-286.84	0.7131	0.866	0.2	0.49	0.28	7.23	9.01	9.01
tran	NA	NA	n380975	MTCONS_00075496	chr12	60486645	60489632	769	+	chrX	20168029	20284780	7871	-		-696.98	0.9028	1	0.61	1.04	0.81	1.93	3.94	3.68
tran	853661	NA	LTCONS_00029402	MTCONS_00029436	chr17	43592048	43596485	445	+	chr17	44450145	44630074	6353	+		-919.2	0.9839	1	5.28	7.69	6.61	1.06	1.58	1.43
tran	NA	NA	n326363	MTCONS_00067000	chr1	156368458	156377044	649	-	chr7	82993222	83218326	6098	-		-348.51	0.6292	1	0.66	0.68	0.49	0.27	3.96	0
cis_mRNA_overlap	0	Lnc-CompleteIn-mRNAIntron	n335030	MTCONS_00074629	chrX	91040488	91040722	235	+	chrX	91034260	91139166	5096	+	*		-0.9195	-1	0.91	0.66	0.81	7.71	11.75	10.77
cis_mRNA_up10k	4	NA	n341037	MTCONS_00011875	chr11	59520488	59522448	879	-	chr11	59522451	59573563	7747	+	*		0.8478	1	0.45	0.44	0.69	2.96	2.64	3.21
tran	NA	NA	LTCONS_00052236	MTCONS_00070584	chr3	94224676	94225741	1066	.	chr8	145945318	145981025	4639	-		-522.46	0.9202	1	1.02	1.53	1.41	2.64	3.84	3.13
cis_mRNA_overlap	0	Lnc-AntiOverlap-mRNA	n411119	MTCONS_00009030	chr10	111705317	111768139	1280	-	chr10	111765726	111895323	4755	+	*		0.994	1	0.16	0.3	0.05	3.16	3.58	2.94
cis_mRNA_overlap	0	mRNA	n385804	MTCONS_00074774	chrX	103173479	103187485	2293	+	chrX	103173048	103232995	6980	+	*		0.9845	1	0.56	0.59	0.44	7.98	8	6.73
tran	41433230	NA	LTCONS_00011018	MTCONS_00008657	chr10	47420433	47421231	799	.	chr10	88854460	88951383	3805	+		-349.9	-0.8374	-1	1.47	1.21	0.53	0.58	2.72	3.28
tran	NA	NA	n367322	MTCONS_00046855	chr4	9682416	9693365	261	-	chr22	31507850	31530790	4163	+		-230.09	-0.7733	-1	0.64	0.58	0.92	0.76	1.54	0.56
cis_mRNA_up10k	673	NA	n410463	MTCONS_00027649	chr16	29464412	29476301	2214	+	chr16	29464902	29465740	479	-	*		0.9061	1	1.34	0.9	1.66	32.11	30.39	39.54
cis_mRNA_up10k	8036	NA	n408068	MTCONS_00016681	chr12	123259056	123311927	2494	+	chr12	123319962	123347513	4919	+	*		0.986	1	0	0.09	0.65	5.78	6.92	9.59
cis_mRNA_up10k	7006	NA	n408068	MTCONS_00016680	chr12	123259056	123311927	2494	+	chr12	123318932	123347513	4949	+	*		0.834	1	0	0.09	0.65	12.2	14.57	15.76
cis_mRNA_overlap	0	Lnc-AntiOverlap-mRNA	n335653	MTCONS_00059430	chr6	3076992	3078808	1817	-	chr6	3064087	3084826	2348	+	*		0.964	1	0	0.05	0.02	1.26	1.86	1.35
cis_mRNA_overlap	0	mRNA	n386308	MTCONS_00047402	chr22	20383731	20398723	1975	-	chr22	20383483	20427848	2791	-		-1368.52	0.9908	1	0.3	0.55	0.07	0.32	0.71	0.1
cis_mRNA_overlap	0	mRNA	n386308	MTCONS_00047403	chr22	20383731	20398723	1975	-	chr22	20383483	20427848	3937	-		-1383.31	0.9219	1	0.3	0.55	0.07	2.5	2.97	0.04
cis_mRNA_overlap	0	Lnc-CompleteIn-mRNAIntron	n342388	MTCONS_00034454	chr19	42836016	42836379	364	+	chr19	42829577	42883002	11131	+	*		-0.9064	-0.866	0.67	0.1	0.1	8.52	10.83	12.53

cis_mRNA_overlap	0	Lnc-CompleteIn-mRNAIntron	n342388	MTCONS_00034453	chr19	42836016	42836379	364	+	chr19	42829577	42883002	11264	+	*	0.9299	0.866	0.67	0.1	0.1	10.02	8.77	8.03
cis_mRNA_up10k	815	NA	n369257	MTCONS_00060742	chr6	96461406	96462915	333	-	chr6	96463729	96663488	13026	+	*	0.6723	1	0.36	0.11	0.43	1.07	1.06	3.16
cis_mRNA_dw20k	7286	NA	n346072	MTCONS_00061545	chr6	170188941	170201611	8362	-	chr6	170151699	170181656	4339	+	*	1	1	0.04	0.07	0.01	1.58	1.61	1.55
tran	NA	NA	n345537	MTCONS_00075496	chr2	91690666	91693026	788	+	chrX	20168029	20284780	7871	-	-390.14	-0.8387	-1	0.38	0.18	0.31	1.93	3.94	3.68
cis_mRNA_overlap	0	mRNA	LTCONS_00072889	MTCONS_00072888	chr9	86534511	86536350	390	-	chr9	86451613	86536360	4778	-	-250.95	-0.9245	-1	1.44	1.12	0.87	1	1.81	1.87
tran	NA	NA	n411614	MTCONS_00064893	chr11	3379157	3400452	3411	-	chr7	64126511	64171424	5099	+	-1215.93	-0.9984	-1	2.79	2.38	6.06	1.54	1.57	0.98
tran	NA	NA	n411614	MTCONS_00034018	chr11	3379157	3400452	3411	-	chr19	24216220	24312694	4162	+	-1216	-0.9843	-1	2.79	2.38	6.06	2.23	2.79	0.82
tran	NA	NA	n367778	MTCONS_00039253	chr5	89196481	89200725	584	+	chr2	175260457	175296340	7547	+	-641.91	0.9979	1	0.13	0.24	0.23	2.63	3.2	3.11
tran	NA	NA	n367778	MTCONS_00039252	chr5	89196481	89200725	584	+	chr2	175260457	175296340	6677	+	-641.91	-0.9115	-1	0.13	0.24	0.23	1.45	0.79	1.11
cis_mRNA_overlap	0	mRNA	LTCONS_00074776	MTCONS_00074774	chrX	103173181	103227394	4061	+	chrX	103173048	103232995	6980	+	-2191.1	0.8976	1	2.57	3.06	1.98	7.98	8	6.73
cis_mRNA_overlap	0	Lnc-CompleteIn-mRNAIntron	n342660	MTCONS_00030564	chr17	15947766	15948278	508	-	chr17	15932738	16118874	10661	-	*	-0.7559	-0.866	0.07	0.33	0.46	3.18	3.18	3.1
cis_mRNA_overlap	0	mRNA	n324681	MTCONS_00046786	chr22	29695643	29704831	6477	+	chr22	29663998	29708780	9225	+	-4628.68	0.965	1	0.08	0.16	0.09	0	0.08	0.03
tran	NA	NA	n407946	MTCONS_00046404	chr15	83676702	83680393	1009	-	chr22	20008631	20055472	4110	+	-815.4	0.8486	0.866	0.3	0.3	0.6	0.97	0.79	1.13
tran	NA	NA	n407946	MTCONS_00062442	chr15	83676702	83680393	1009	-	chr6	34231228	34360518	10114	-	-970.74	0.949	0.866	0.3	0.3	0.6	47.14	47.98	49.75
tran	NA	NA	n407946	MTCONS_00046402	chr15	83676702	83680393	1009	-	chr22	20008630	20055472	4182	+	-815.4	0.9188	0.866	0.3	0.3	0.6	0.97	1.3	1.8
cis_mRNA_overlap	0	Lnc-CompleteIn-mRNAExon	n338629	MTCONS_00019952	chr13	30776775	30781018	4217	-	chr13	30776681	30881615	8283	-	-1663.86	0.9372	1	4.81	7.6	3.06	1.64	2.59	1.6
cis_mRNA_overlap	0	Lnc-CompleteIn-mRNAExon	n338629	MTCONS_00019951	chr13	30776775	30781018	4217	-	chr13	30776681	30881209	7674	-	-1581.75	0.8169	1	4.81	7.6	3.06	1.54	1.56	1.12
cis_mRNA_overlap	0	Lnc-CompleteIn-mRNAIntron	n326133	MTCONS_00003750	chr1	246922115	246929034	580	+	chr1	246887184	246946086	14034	+	*	-0.8558	-1	0.13	0	0.11	0.43	0.85	0.7
tran	NA	NA	n332900	MTCONS_00022737	chr3	12548554	12581132	565	+	chr14	89079850	89111331	4694	-	-320.41	-0.9986	-1	0.17	0	0.34	0.23	0.28	0.17
cis_mRNA_overlap	0	mRNA	LTCONS_00017286	MTCONS_00017288	chr12	22601473	22616848	4464	-	chr12	22601473	22698224	5350	-	-1670.56	0.9372	1	2.46	2.65	2.16	2.75	2.78	2.09
cis_mRNA_overlap	0	mRNA	LTCONS_00017286	MTCONS_00017287	chr12	22601473	22616848	4464	-	chr12	22601473	22672319	4825	-	-1553.53	0.9806	1	2.46	2.65	2.16	3.7	4.03	3.44
cis_mRNA_up10k	141	NA	LTCONS_00028554	MTCONS_00030321	chr17	5095318	5129775	3758	+	chr17	5078341	5095178	4755	-	*	1	1	1.37	1.61	1.48	1.77	2.52	2.12
cis_mRNA_up10k	125	NA	LTCONS_00028554	MTCONS_00030322	chr17	5095318	5129775	3758	+	chr17	5079728	5095194	8312	-	*	0.977	1	1.37	1.61	1.48	0.65	0.8	0.69
tran	148704	NA	n408123	MTCONS_00066942	chr7	75137069	75157453	1501	-	chr7	74921877	74988366	1808	-	-832.67	-0.9965	-1	5.12	4.51	6.09	5.02	5.8	4.1
tran	NA	NA	n325544	MTCONS_00032674	chr17	63609345	63623253	707	+	chr18	77794346	77830757	3161	+	-440.61	0.8322	0.866	0.05	0.14	0.09	7.23	9.01	9.01
tran	NA	NA	n367080	MTCONS_00047711	chr4	180087670	180091375	671	-	chr22	32896532	33448559	15401	-	-853.02	-0.9898	-1	0.22	0.11	0.43	2.72	2.94	1.9
tran	NA	NA	LTCONS_00066546	MTCONS_00017912	chr7	35118964	35225863	6102	-	chr12	63952662	64062424	11449	-	-2401.87	0.6996	1	4.37	4.57	5.48	2.01	2.92	3.01
cis_mRNA_up10k	155	NA	n378289	MTCONS_00047600	chr22	28315460	28318075	565	+	chr22	28247630	28315306	2987	-	*	0.9844	1	1.24	1.28	1.46	2.59	2.85	3.34
cis_mRNA_dw20k	15213	NA	n409181	MTCONS_00033385	chr19	3225483	3297073	1650	+	chr19	3185875	3210271	4428	+	*	0.9993	1	3.14	4.12	4.14	1.32	2.08	2.13
cis_mRNA_overlap	0	Lnc-CompleteIn-mRNAIntron	n385187	MTCONS_00064893	chr7	64141511	64147263	2760	+	chr7	64126511	64171424	5099	+	*	0.7511	1	0.02	0.07	0	1.54	1.57	0.98
tran	NA	NA	n339521	MTCONS_00075262	chr3	126707502	126751326	5263	+	chrX	153686623	153704519	11497	+	-4086.73	0.7664	1	5.3	6.39	8.98	3.35	4.52	4.6
tran	NA	NA	n324701	MTCONS_00033686	chr22	24007804	24009061	568	-	chr19	11998621	12032411	17275	+	-820.2	0.9977	1	18.38	22.95	22.24	1.66	3.92	3.42
tran	NA	NA	n324701	MTCONS_00033685	chr22	24007804	24009061	568	-	chr19	11998607	12032411	17331	+	-820.2	-0.9991	-1	18.38	22.95	22.24	4.11	2.39	2.73
tran	NA	NA	n324701	MTCONS_00036294	chr22	24007804	24009061	568	-	chr19	40528842	40562147	24857	-	-810.21	0.8501	1	18.38	22.95	22.24	6.91	8.47	7.45
tran	NA	NA	n345463	MTCONS_00071498	chr1	150517408	150518106	699	-	chr9	104161163	104181074	11258	+	-393.93	0.8538	1	0	0.11	0.03	4.99	6.86	6.41
tran	NA	NA	n368360	MTCONS_00031083	chr5	149719783	149720699	800	-	chr17	40190058	40196297	4596	-	-580.8	-0.9992	-0.866	0.04	0.04	0.12	2.26	2.3	1.43
tran	NA	NA	n368360	MTCONS_00031084	chr5	149719783	149720699	800	-	chr17	40190058	40202750	4056	-	-580.8	0.8761	0.866	0.04	0.04	0.12	2.79	3.06	3.35
tran	NA	NA	n380519	MTCONS_00047712	chr11	130434325	130628495	571	-	chr22	32896532	33454377	15152	-	-744.14	0.9549	1	0.39	0.34	0.27	5.15	5.06	4.49
cis_mRNA_dw20k	19	NA	n338313	MTCONS_00035598	chr19	12305853	12349425	1739	+	chr19	12349443	12405715	10448	-	*	0.7929	1	3.67	0.88	0.8	2.01	1.97	1.9
tran	128960	NA	n406598	MTCONS_00002455	chr1	155715609	155721369	2524	+	chr1	155579947	155586650	4684	+	-1677.41	0.977	1	2.54	2.66	2.55	1.22	1.56	1.32
cis_mRNA_overlap	0	Lnc-AntiOverlap-mRNA	n377659	MTCONS_00024148	chr15	84868064	84898888	2525	-	chr15	84840742	84878025	13375	+	*	-1	-1	1.47	1.06	2.09	3.42	3.94	2.61
tran	1310064	NA	n407465	MTCONS_00041782	chr2	132250386	132279149	3788	+	chr2	130908965	130940323	4092	-	-2381.63	-0.8513	-1	0.38	1.09	0.85	0.24	0	0.2
cis_mRNA_overlap	0	Lnc-CompleteIn-mRNAExon	n341836	MTCONS_00015352	chr12	14653399	14655865	2466	+	chr12	14538004	14655865	9006	+	-1005.67	0.9925	1	0.9	0	5.12	9.57	7.86	13.72
tran	NA	NA	n325869	MTCONS_00003750	chr10	122511314	122526386	2014	-	chr1	246887184	246946086	14034	+	-1456.3	-0.9359	-0.866	0.17	0.08	0.08	0.43	0.85	0.7
cis_mRNA_overlap	0	Lnc-AntiOverlap-mRNA	LTCONS_00061771	MTCONS_00059411	chr6	2985532	3019996	4379	-	chr6	2988201	3023034	1970	+	*	0.8822	1	0.01	0.02	0	0.77	1.79	0.73
tran	NA	NA	n342816	MTCONS_00017912	chr7	89748714	89754914	2018	+	chr12	63952662	64062424	11449	-	-826.3	0.998	1	1.87	2.84	2.86	2.01	2.92	3.01
cis_mRNA_overlap	0	Lnc-CompleteIn-mRNAExon	n339931	MTCONS_00032676	chr18	77853498	77854968	1471	+	chr18	77794346	77854968	4564	+	-1016.29	-0.9987	-1	0.06	0.17	0	0.39	0.3	0.43
tran	62035688	NA	LTCONS_00007023	MTCONS_00002089	chr1	206131472	206138799	5550	-	chr1	143913246	144095785	3064	+	-1766.85	0.8352	1	1.66	1.86	1.7	1.46	1.71	1.64
cis_mRNA_dw20k	616	NA	n344575	MTCONS_00003750	chr1	246946701	246955691	1686	+	chr1	246887184	246946086	14034	+	*	1	1	0.24	0.52	0.42	0.43	0.85	0.7
cis_mRNA_up10k	5609	NA	LTCONS_00063745	MTCONS_00063743	chr6	170601219	170603162	1944	-	chr6	170591202	170595611	3170	-	*	-0.9497	-1	0.74	0.71	0.68	1.52	1.58	1.8
cis_mRNA_overlap	0	Lnc-CompleteIn-mRNAIntron	n342711	MTCONS_00029436	chr17	44620700	44622821	2120	+	chr17	44450145	44630074	6353	+	*	0.9986	1	0.64	0.89	0.83	1.06	1.58	1.43
cis_mRNA_overlap	0	Lnc-AntiOverlap-mRNA	n342046	MTCONS_00018539	chr12	120639174	120650941	1170	+	chr12	120647763	120703564	5182	-	*	-0.9856	-1	0.65	0.4	0.3	0.45	1.25	1.36
tran	NA	NA	n374771	MTCONS_00010695	chr15	64165701	64168589	496	-	chr10	105623521	105678045	8971	-	-585.81	-0.9964	-1	0.39	0.38	0.36	2.12	2.32	2.61
cis_mRNA_overlap	0	mRNA	n408198	MTCONS_00036852	chr19	53611132	53636173	2659	-	chr19	53610696	53636173	3062	-	-1075.54	0.9672	1	0.52	0.85	1.18	1.13	1.45	1.57
cis_mRNA_overlap	0	mRNA	n408198	MTCONS_00036853	chr19	53611132	53636173	2659	-	chr19	53610696	53636173	2720	-	-1046.91	-0.9985	-1	0.52	0.85	1.18	1.19	0.76	0.24
cis_mRNA_dw20k	8262	NA	n337426	MTCONS_00018568	chr12	121660806	121666872	368	+	chr12	121675133	121734556	4837	-	*	-0.8787	-1	0.45	0.11	0.12	0	0.12	0.06
tran	NA	NA	n385785	MTCONS_00031132	chrX	73164159	73290211	1664	+	chr17	41102543	41117348	2197	-	-1291.21	0.959	1	0.36	0.21	0.66	7.94	6.66	8.85
cis_mRNA_up10k	169	NA	LTCONS_00062792	MTCONS_00060528	chr6	69342519	69344919	698	-	chr6	69345087	70099476	6083	+	*	0.8762	1	2.41	1.7	2.58	7.26	6.49	8.64
tran	NA	NA	LTCONS_00076831	MTCONS_00024148	chrY	27633758	27634167	410	.	chr15	84840742	84878025	13375	+	-337.7	-1	-1	5.7	4.42	7.76	3.42	3.94	2.61
tran	NA	NA	LTCONS_00042049	MTCONS_00005688	chr2	170542083	170550931	3537	-	chr1	114258385	114300735	4864	-	-1721.48	0.8675	1	2.33	2.73	1.84	3.91	4.59	3.86
cis_mRNA_overlap	0	mRNA	n336826	MTCONS_00071285	chr9	90342123	90342970	351	+	chr9	90340768	90346384	1830	+	-199.49	0.861	1	0.45	0.21	0.42	2.83	1.45	1.99

tran	NA	NA	n339548	MTCONS_00010928	chr3	129526705	129527779	1074	+	chr10	128966974	128995916	9347	-		-486.87	0.8888	1	0.07	0.08	0.02	2.03	3.32	1.15
cis_mRNA_overlap	0	AntiCompleteIn-mRNAIntron	n339548	MTCONS_00051503	chr3	129526705	129527779	1074	+	chr3	129366630	129612439	6193	-	*		-0.9843	-1	0.07	0.08	0.02	9.53	8.98	10.63
tran	NA	NA	n325063	MTCONS_00036294	chr20	12172543	12172944	280	-	chr19	40528842	40562147	24857	-		-162.47	0.9914	1	0	0.26	0.12	6.91	8.47	7.45
tran	NA	NA	n325063	MTCONS_00071498	chr20	12172543	12172944	280	-	chr9	104161163	104181074	11258	+		-333.97	0.9443	1	0	0.26	0.12	4.99	6.86	6.41
cis_mRNA_dw20k	6791	NA	n342703	MTCONS_00029287	chr17	40704456	40706766	2311	-	chr17	40687058	40697666	4727	+	*		0.9993	1	5.39	6.63	5.76	0.06	0.42	0.18
tran	NA	NA	n364863	MTCONS_00025783	chr2	6968645	6973662	799	-	chr16	2525125	2555778	7990	+		-526.23	0.8885	1	0.09	0.12	0.16	2.77	3.14	3.2
tran	NA	NA	n364863	MTCONS_00025780	chr2	6968645	6973662	799	-	chr16	2518074	2555778	13051	+		-546.07	0.822	0.866	0.09	0.12	0.16	0.68	0.88	0.88
cis_mRNA_up10k	9	NA	n375716	MTCONS_00029228	chr17	38276827	38278125	929	-	chr17	38278133	38293738	5646	+	*		-0.7378	-1	0.29	0.58	0.28	13.17	12.53	14.8
tran	NA	NA	n324916	MTCONS_00039540	chr20	61197595	61204076	551	-	chr2	204191360	204296892	8237	+		-510.14	-0.969	-1	0.18	0.1	0	7.48	9.11	9.94
tran	NA	NA	n410476	MTCONS_00063896	chr6	32938665	32949282	4899	+	chr6_mann_hap4	4403140	4406282	1380	+		-950.84	0.745	1	3.51	4.67	4.87	11.47	13.18	20.45
cis_mRNA_overlap	0	mRNA	n410476	MTCONS_00060121	chr6	32938665	32949282	4899	+	chr6	32938665	32944978	3079	+		-1999.64	0.9164	1	3.51	4.67	4.87	6	7.79	9.74
tran	NA	NA	n410476	MTCONS_00063895	chr6	32938665	32949282	4899	+	chr6_mann_hap4	4400152	4401452	1220	+		-734.19	0.6852	1	3.51	4.67	4.87	1.02	1.15	2.27
cis_mRNA_overlap	0	Lnc-AntiOverlap-mRNA	n406404	MTCONS_00073634	chr9	136890561	136896719	5734	+	chr9	136895427	136933147	5657	-	*		-0.9421	-1	1.37	1.58	1.74	2.44	2.06	1.01
cis_mRNA_overlap	0	mRNA-AntiCompleteIn-LncIntron	n410982	MTCONS_00008795	chr10	97512963	97849992	4262	-	chr10	97803159	97820687	3953	+	*		0.6213	0.866	0.05	0.05	0.04	0.24	0.99	0.1
tran	NA	NA	n325191	MTCONS_00075262	chr2	107345207	107390217	616	+	chrX	153686623	153704519	11497	+		-446.49	0.7921	1	0.11	0.25	0.53	3.35	4.52	4.6
tran	NA	NA	n386137	MTCONS_00073508	chr15	84872929	84898920	933	-	chr9	131018075	131038308	4252	-		-770.94	-0.9992	-1	0.97	2.34	1.92	12.48	11.49	11.83
tran	NA	NA	n342602	MTCONS_00003289	chr4	68919226	68938945	2777	+	chr1	210111456	210344584	12522	+		-3858.52	-0.8879	-0.866	0.12	0.02	0.02	0.88	1.64	1.29
tran	NA	NA	n342602	MTCONS_00003296	chr4	68919226	68938945	2777	+	chr1	210111538	210344584	13474	+		-3858.52	0.61	0.866	0.12	0.02	0.02	3.84	3.7	3.82
cis_mRNA_overlap	0	mRNA	LTCONS_00064454	MTCONS_00064450	chr7	23733291	23742269	1988	+	chr7	23719749	23742269	1298	+		-487.29	0.9889	1	1.75	2.01	0.99	2.5	2.61	1.59
tran	NA	NA	n408042	MTCONS_00064076	chr6	30227339	30234728	4622	+	chr6_ssto_hap7	2655403	2658749	1578	-		-1048.24	-0.6597	-0.866	0.78	0.78	0.84	11.2	3.05	0.93
tran	NA	NA	n408042	MTCONS_00063993	chr6	30227339	30234728	4622	+	chr6_qbl_hap6	2615762	2618151	921	-		-677.7	-0.9347	-0.866	0.78	0.78	0.84	12.05	17.42	2.51
cis_mRNA_overlap	0	Lnc-CompleteIn-mRNAIntron	n338444	MTCONS_00022742	chr14	89802488	89804712	2223	-	chr14	89622296	90085531	8146	-	*		-0.9354	-1	0.57	1.07	0.76	3.4	2.33	3.37
cis_mRNA_overlap	0	Lnc-CompleteIn-mRNAIntron	n407254	MTCONS_00059455	chr6	3271662	3281933	1805	+	chr6	3258996	3304632	2052	+	*		0.8601	1	0.46	0.5	0.43	1.45	1.49	0.98
tran	NA	NA	n325289	MTCONS_00069395	chr2	64566991	64581542	572	+	chr8	12933204	13462145	14928	-		-327.55	-0.7882	-0.866	0.06	0.06	0.23	8.1	10.25	6.79
tran	NA	NA	n385118	MTCONS_00032984	chr7	32768504	32794385	1766	+	chr18	45346666	45457071	23239	-		-3099.9	0.9724	1	2.39	2.44	2.35	2.93	3.26	2.83
tran	NA	NA	n383432	MTCONS_00027595	chr19	21676922	21682695	1907	+	chr16	23470492	23521815	12503	-		-1362.48	0.9989	1	0.03	0.05	0.04	0.57	0.94	0.74
cis_mRNA_dw20k	3183	NA	n345528	MTCONS_00037945	chr2	62376624	62382117	1332	+	chr2	62132721	62373442	11032	+	*		0.9959	1	0.26	0.62	0.3	2.46	3.22	2.47
tran	37962	NA	n411654	MTCONS_00029436	chr17	44668035	44834828	3945	+	chr17	44450145	44630074	6353	+		-1887.11	0.8931	1	0.09	0.29	0.15	1.06	1.58	1.43
tran	79077	NA	n411654	MTCONS_00029435	chr17	44668035	44834828	3945	+	chr17	44450145	44588959	1629	+		-880.8	0.9984	1	0.09	0.29	0.15	0.27	1.64	0.61
tran	NA	NA	n344976	MTCONS_00027594	chr7	156785276	156789232	430	-	chr16	23470492	23521815	11188	-		-426.06	0.6143	1	2.69	3.81	2.66	0.16	0.17	0.09
cis_mRNA_overlap	0	Lnc-CompleteIn-mRNAIntron	n340136	MTCONS_00008981	chr10	105342118	105343431	1314	+	chr10	105315024	105353786	5444	+	*		-0.7812	-1	0.08	0.24	0.05	0.79	0.68	1.15
cis_mRNA_dw20k	951	NA	n364046	MTCONS_00003749	chr1	246947036	246955689	1233	+	chr1	246887184	246946086	12429	+	*		0.9042	0.866	0	0.12	0.28	1.03	1.03	1.27
cis_mRNA_dw20k	13674	NA	n363829	MTCONS_00000810	chr1	32813111	32814381	406	+	chr1	32757536	32799438	5742	+	*		-0.9981	-1	0.28	0.09	0.42	4.99	5.91	4.15
cis_mRNA_up10k	31	NA	n385002	MTCONS_00060975	chr6	116999740	117001922	2183	-	chr6	117001952	117056986	5666	+	*		0.6849	1	2.53	3.2	3.39	18.06	18.14	21.74
tran	NA	NA	n365315	MTCONS_00037060	chr2	203175137	203177250	1187	+	chr19	59008116	59033259	10168	-		-812.19	-0.8589	-1	0.41	0.21	0.26	0.68	0.79	0.71
tran	11554134	NA	n406566	MTCONS_00024568	chr15	23255242	23262743	1899	+	chr15	34816876	34876936	8202	-		-1234.04	-0.9237	-0.866	0.29	0.12	0.12	3.95	5.03	5.71
tran	11408527	NA	n406566	MTCONS_00024565	chr15	23255242	23262743	1899	+	chr15	34671269	34876936	7605	-		-1230.77	0.8062	0.866	0.29	0.12	0.12	0.83	0.41	0.66
cis_mRNA_overlap	0	mRNA	LTCONS_00065332	MTCONS_00065329	chr7	99933646	99965653	4720	+	chr7	99775538	99948836	16269	+	*		-0.815	-1	13.66	12.44	10.37	1.41	1.88	1.91
tran	NA	NA	n324432	MTCONS_00022667	chr6	108551412	108584595	717	+	chr14	77249021	77279283	7871	-		-644.14	-0.9304	-1	1.25	1.22	1.27	1.82	2.33	1.8
tran	11203406	NA	n411020	MTCONS_00024568	chr15	23599895	23613471	5454	+	chr15	34816876	34876936	8202	-		-2638.07	0.9237	0.866	0.24	0.3	0.3	3.95	5.03	5.71
tran	11057799	NA	n411020	MTCONS_00024565	chr15	23599895	23613471	5454	+	chr15	34671269	34876936	7605	-		-2604.13	-0.8062	-0.866	0.24	0.3	0.3	0.83	0.41	0.66
cis_mRNA_dw20k	17202	NA	n338384	MTCONS_00021436	chr14	73989248	73991015	1379	+	chr14	73957644	73972047	3781	+	*		0.8969	1	0.08	0.23	0.36	0.11	0.46	0.47
cis_mRNA_dw20k	21	NA	n377907	MTCONS_00060502	chr6	56979709	57037194	873	-	chr6	56954828	56979689	14699	+	*		0.759	0.866	0	0.15	0.15	7.59	7.86	8.39
cis_mRNA_overlap	0	Lnc-CompleteIn-mRNAExon	n335532	MTCONS_00069892	chr8	74204581	74205868	202	-	chr8	74202840	74205907	909	-		-93.26	0.9698	1	0.89	0.4	0.24	564.66	540.71	519.77
tran	NA	NA	n366869	MTCONS_00033686	chr4	104882773	104958080	812	+	chr19	11998621	12032411	17275	+		-364.57	-0.9364	-1	17.86	16.03	17.02	1.66	3.92	3.42
tran	NA	NA	n366869	MTCONS_00033685	chr4	104882773	104958080	812	+	chr19	11998607	12032411	17331	+		-364.57	0.9275	1	17.86	16.03	17.02	4.11	2.39	2.73
cis_mRNA_up10k	4096	NA	n382957	MTCONS_00026227	chr16	29460666	29461236	571	+	chr16	29465331	29476301	6115	+	*		0.999	1	0.88	1.98	2.46	1.34	3.07	3.98
cis_mRNA_up10k	4096	NA	n382957	MTCONS_00026228	chr16	29460666	29461236	571	+	chr16	29465331	29476301	4668	+	*		0.9913	1	0.88	1.98	2.46	2.96	5.48	6.03
tran	23241471	NA	n406191	MTCONS_00065090	chr7	99918263	99933930	1518	-	chr7	76607916	76676793	3269	+		-874.24	0.9977	1	5.88	6.39	6.27	0.87	1.46	1.36
cis_mRNA_overlap	0	Lnc-AntiOverlap-mRNA	n325543	MTCONS_00031631	chr17	65102521	65136087	3085	+	chr17	65066554	65241320	13834	-	*		0.9878	1	0.04	0.06	0.07	1.17	1.69	1.81
cis_mRNA_overlap	0	Lnc-CompleteIn-mRNAIntron	n341364	MTCONS_00067805	chr7	151562747	151566014	3268	-	chr7	151253200	151574316	3405	-	*		0.964	1	0.2	0.36	0.31	0.27	0.58	0.41
tran	NA	NA	n370934	MTCONS_00022569	chr9	47192550	47210048	487	+	chr14	71188143	71276263	12575	-		-452.34	0.996	1	0.08	0.6	0.27	3.1	4.7	3.81
tran	NA	NA	n370934	MTCONS_00022568	chr9	47192550	47210048	487	+	chr14	71188143	71276226	12607	-		-452.34	-0.9407	-1	0.08	0.6	0.27	1.08	0.33	1.06
tran	NA	NA	n326344	MTCONS_00033686	chr1	166636691	166658518	1801	-	chr19	11998621	12032411	17275	+		-1474.85	0.9185	1	6.11	7.26	6.59	1.66	3.92	3.42
tran	NA	NA	n326344	MTCONS_00039983	chr1	166636691	166658518	1801	-	chr2	238536213	238694823	8109	+		-842.61	0.8781	1	6.11	7.26	6.59	1.72	3.49	3.26
tran	NA	NA	n326344	MTCONS_00033685	chr1	166636691	166658518	1801	-	chr19	11998607	12032411	17331	+		-1474.85	-0.9086	-1	6.11	7.26	6.59	4.11	2.39	2.73
tran	NA	NA	n326344	MTCONS_00036294	chr1	166636691	166658518	1801	-	chr19	40528842	40562147	24857	-		-1534.53	0.9967	1	6.11	7.26	6.59	6.91	8.47	7.45
cis_mRNA_overlap	0	mRNA	n340794	MTCONS_00059101	chr5	180682147	180684315	2168	-	chr5	180681680	180688201	3381	-		-925.32	-0.9999	-1	3.44	4.03	3.28	1.45	0.94	1.58
cis_mRNA_up10k	7133	NA	LTCONS_00076099	MTCONS_00076097	chrX	103364213	103401708	11842	-	chrX	103343898	103357081	6149	-	*		-0.9956	-1	2.29	3.1	3.39	1.74	1.36	1.28

cis_mRNA_overlap	0	mRNA	n339251	MTCONS_00026683	chr16	67198716	67203846	1543	+	chr16	67193891	67203939	4269	+		-1311.8	-0.7643	-0.866	0.87	0	0.3	0.31	1.52	0.31
cis_mRNA_dw20k	18411	NA	n345596	MTCONS_00049465	chr3	131087719	131090842	1140	+	chr3	130745694	131069309	2856	+	*		-0.9846	-0.866	0	0.03	0.03	0.43	0	0.08
tran	NA	NA	n380069	MTCONS_00015352	chr5	158527491	158542331	691	+	chr12	14538004	14655865	9006	+		-469.19	0.7815	1	1.15	0.48	1.22	9.57	7.86	13.72
cis_mRNA_overlap	0	Lnc-CompleteIn-mRNAExon	n342158	MTCONS_00063539	chr6	155574274	155577858	3087	-	chr6	155574187	155637113	6877	-		-1493.89	-0.8473	-1	1.72	1.74	1.95	0.05	0.02	0
tran	NA	NA	n326657	MTCONS_00033686	chr1	31055812	31060987	444	-	chr19	11998621	12032411	17275	+		-203.26	0.8649	1	0.09	0.7	0.28	1.66	3.92	3.42
tran	NA	NA	n326657	MTCONS_00033685	chr1	31055812	31060987	444	-	chr19	11998607	12032411	17331	+		-203.26	-0.8524	-1	0.09	0.7	0.28	4.11	2.39	2.73
cis_mRNA_dw20k	13152	NA	n338970	MTCONS_00037001	chr19	58038429	58039099	671	-	chr19	58052250	58071227	7132	-	*		-0.9355	-1	0.11	0	0.05	1.26	1.93	1.41
cis_mRNA_dw20k	9147	NA	n376322	MTCONS_00036586	chr19	48824742	48826469	916	+	chr19	48835615	48867094	2524	-	*		0.8248	1	0.08	0.21	0.24	0.08	0.22	0.59
cis_mRNA_dw20k	9147	NA	n376322	MTCONS_00036587	chr19	48824742	48826469	916	+	chr19	48835615	48867186	2145	-	*		-0.8389	-1	0.08	0.21	0.24	1.39	1.25	0.92
tran	NA	NA	n363722	MTCONS_00066703	chr1	182709783	182710939	891	-	chr7	47994874	48019266	10405	-		-1759	0.8612	1	4.61	5.28	6.97	7.11	8.45	8.88
tran	NA	NA	n374202	MTCONS_00037006	chr14	90959135	90959933	532	+	chr19	58201202	58220617	13301	-		-620.95	-0.9833	-1	0.21	0.06	0.11	4.35	5.99	5.18
cis_mRNA_up10k	234	NA	n339546	MTCONS_00045655	chr21	47874900	47878593	1529	-	chr21	47878826	47992776	10206	+	*		0.9277	1	0.18	0.5	0.26	0.56	0.73	0.66
cis_mRNA_up10k	250	NA	n339546	MTCONS_00045656	chr21	47874900	47878593	1529	-	chr21	47878842	47992776	12342	+	*		0.9796	1	0.18	0.5	0.26	0.96	1.4	1.15
cis_mRNA_dw20k	7127	NA	n340118	MTCONS_00008856	chr10	100011780	100016332	1168	+	chr10	99894381	100004654	3426	+	*		-0.9956	-1	0.12	0.11	0.19	2.68	2.72	1.08
cis_mRNA_dw20k	7127	NA	n340118	MTCONS_00008858	chr10	100011780	100016332	1168	+	chr10	99912515	100004654	3535	+	*		0.9972	1	0.12	0.11	0.19	1.69	1.59	2.09
tran	94042428	NA	n372755	MTCONS_00013134	chr11	94820008	94821880	942	-	chr11	765030	777581	6966	-		-847.57	0.9689	1	0.15	0.4	0.5	2.55	2.94	3.34
tran	NA	NA	n344670	MTCONS_00047711	chr7	19938142	20165108	4891	-	chr22	32896532	33448559	15401	-		-2642.58	-0.6636	-0.866	0.11	0.09	0.11	2.72	2.94	1.9
cis_mRNA_overlap	0	Lnc-AntiOverlap-mRNA	LTCONS_00064714	MTCONS_00066638	chr7	44078698	44082082	2100	+	chr7	44065964	44080295	4331	-	*		-0.9997	-1	0.51	0	0.82	2.81	3.87	2.1
cis_mRNA_dw20k	2994	NA	n334863	MTCONS_00059411	chr6	3026027	3028470	520	+	chr6	2988201	3023034	1970	+	*		0.9999	1	0.14	0.33	0.13	0.77	1.79	0.73
cis_mRNA_dw20k	15303	NA	n340021	MTCONS_00009913	chr10	45931684	45934607	2924	+	chr10	45949909	46090354	6388	-	*		0.8519	1	0.26	0.46	0.37	1.35	2.52	1.39
cis_mRNA_dw20k	15303	NA	n340021	MTCONS_00009914	chr10	45931684	45934607	2924	+	chr10	45949909	46090354	6250	-	*		-0.9789	-1	0.26	0.46	0.37	1.82	0.58	1.36
cis_mRNA_dw20k	1679	NA	LTCONS_00076607	MTCONS_00074898	chrX	118154499	118157091	2593	.	chrX	118139135	118152821	2152	+	*		0.9998	1	1.32	1.18	0.86	1.61	1.44	1.08
cis_mRNA_up10k	277	NA	n384324	MTCONS_00051503	chr3	129612715	129625155	1502	+	chr3	129366630	129612439	6193	-	*		-0.8391	-1	1	1.4	0.93	9.53	8.98	10.63
cis_mRNA_overlap	0	Lnc-AntiOverlap-mRNA	LTCONS_00025332	MTCONS_00024160	chr15	85181743	85197521	9047	-	chr15	85144239	85185987	15744	+		-7437.02	0.9147	1	1.75	3.17	3.07	0.48	0.74	0.62
cis_mRNA_overlap	0	mRNA	n371628	MTCONS_00076692	chrY	14774468	14800184	882	+	chrY	14774265	14972765	9712	+		-439.01	0.9999	1	4.37	0	4.35	18.82	0	18.47
cis_mRNA_overlap	0	Lnc-AntiOverlap-mRNA	n410038	MTCONS_00017778	chr12	56223392	56230030	3414	+	chr12	56214649	56224054	3520	-	*		-0.711	-1	0.27	1.26	0.37	0.88	0.18	0.31
cis_mRNA_up10k	3009	NA	n370443	MTCONS_00068295	chr8	28536529	28555991	601	-	chr8	28558999	28612901	8025	+	*		0.8614	0.866	0.17	0.01	0.17	1.71	1.4	2.03
tran	NA	NA	n411708	MTCONS_00036294	chr6	3672657	3675838	615	+	chr19	40528842	40562147	24857	-		-569.37	-0.9729	-1	7.53	5.93	6.65	6.91	8.47	7.45
tran	NA	NA	n386352	MTCONS_00073090	chr3	197784404	197807542	2642	-	chr9	99941728	99943197	1470	-		-527.05	0.9999	1	0.01	0.04	0.06	1.23	1.48	1.64
cis_mRNA_dw20k	2569	NA	LTCONS_00067875	MTCONS_00064336	chr7	11211822	11212477	656	.	chr7	11012967	11209254	3968	+	*		-0.7037	-1	0.94	1.06	0.59	16.96	12.05	17.01
cis_mRNA_up10k	4455	NA	n341640	MTCONS_00069210	chr8	145575882	145577730	1758	-	chr8	145582184	145587778	4800	+	*		-0.9929	-1	0.24	0.11	0	3.32	3.64	4.05
tran	NA	NA	n325278	MTCONS_00047712	chr2	66463942	66473086	1679	+	chr22	32896532	33454377	15152	-		-2751.81	-0.988	-1	0	0.02	0.07	5.15	5.06	4.49
cis_mRNA_overlap	0	Lnc-CompleteIn-mRNAExon	n409315	MTCONS_00076027	chrX	100870108	100872991	937	-	chrX	100870108	100872991	2148	-		-574.62	-0.9856	-1	11.88	8.9	10.48	1.43	2.11	1.85
tran	NA	NA	n323795	MTCONS_00036294	chrX	145703316	145703936	431	-	chr19	40528842	40562147	24857	-		-557.38	0.8202	1	3.36	5.37	5.17	6.91	8.47	7.45
cis_mRNA_dw20k	3110	NA	n407259	MTCONS_00017778	chr12	56146247	56211540	1148	-	chr12	56214649	56224054	3520	-	*		-0.9864	-1	2.91	3.77	3.76	0.88	0.18	0.31
cis_mRNA_up10k	254	NA	n385677	MTCONS_00071609	chr9	115232003	115248956	402	-	chr9	115249209	115392648	13883	+	*		-0.9965	-1	0.48	0.09	0.77	3.98	4.09	3.92
cis_mRNA_up10k	41	NA	n407721	MTCONS_00034245	chr19	37071155	37096178	2690	-	chr19	37096218	37128259	11934	+	*		-0.9701	-1	1.76	2.27	1.31	0.92	0.83	0.95
cis_mRNA_up10k	41	NA	n407721	MTCONS_00034244	chr19	37071155	37096178	2690	-	chr19	37096218	37128259	14238	+	*		0.9679	1	1.76	2.27	1.31	3.19	3.41	3.12
tran	NA	NA	n370556	MTCONS_00030539	chr8	118750472	118753715	772	-	chr17	15331553	15466945	10817	-		-688.2	0.9903	1	0.04	0.09	0.21	2.48	2.53	2.79
cis_mRNA_dw20k	11412	NA	n373511	MTCONS_00018509	chr12	118555475	118573735	477	-	chr12	118585146	118628356	5769	-	*		-0.7725	-1	1.42	0.87	1.41	2.25	3.01	2.75
cis_mRNA_overlap	0	Lnc-CompleteIn-mRNAIntron	n381941	MTCONS_00018538	chr12	120655867	120657920	2054	-	chr12	120647763	120703563	4570	-	*		-0.9703	-1	0.36	0.77	0.71	1.07	0.51	0.72
cis_mRNA_up10k	3733	NA	n344875	MTCONS_00063088	chr6	111808356	111872652	8999	+	chr6	111619894	111804624	11379	-	*		0.8377	0.866	0.14	0.14	0.11	1.19	1.54	0.9
cis_mRNA_dw20k	7489	NA	n344875	MTCONS_00063091	chr6	111808356	111872652	8999	+	chr6	111880140	111888521	1128	-	*		0.9449	0.866	0.14	0.14	0.11	0.91	0.96	0.81
cis_mRNA_overlap	0	Lnc-CompleteIn-mRNAIntron	n324802	MTCONS_00045300	chr21	35040004	35042534	767	+	chr21	35014784	35272247	17062	+	*		-0.9981	-1	0.19	0	0.29	6.39	7.4	5.98
tran	NA	NA	n385916	MTCONS_00076760	chr1	15603	29370	1189	-	chrY	59354140	59358344	1999	+		-975.87	0.9951	1	0.35	1.38	0.74	5.13	12.17	7.17
cis_mRNA_up10k	201	NA	n379064	MTCONS_00027703	chr16	30546394	30548465	1124	+	chr16	30541977	30546194	3033	-	*		-0.7568	-1	1.88	1.74	2.28	0.3	0.9	0.24
cis_mRNA_up10k	190	NA	n379064	MTCONS_00027704	chr16	30546394	30548465	1124	+	chr16	30541977	30546205	3041	-	*		0.9344	1	1.88	1.74	2.28	3.31	2.52	3.88
cis_mRNA_up10k	541	NA	LTCONS_00014967	MTCONS_00014875	chr11	129149651	129151314	1664	.	chr11	128834955	129149111	10066	-	*		-0.906	-1	0.91	0.57	0.85	1.26	1.84	1.59
tran	NA	NA	n365849	MTCONS_00018635	chr2	201979497	201980387	791	-	chr12	123773656	123849343	11074	-		-628.02	0.9175	1	0.05	0.08	0.04	8.24	8.78	7.39
tran	NA	NA	n365849	MTCONS_00073553	chr2	201979497	201980387	791	-	chr9	132396748	132404448	6463	-		-499.77	-0.8114	-1	0.05	0.08	0.04	3.16	3	3.84
tran	NA	NA	n365849	MTCONS_00018636	chr2	201979497	201980387	791	-	chr12	123773656	123849343	11077	-		-628.02	-0.9412	-1	0.05	0.08	0.04	3.41	2.36	4.72
tran	NA	NA	LTCONS_00035940	MTCONS_00038426	chr19	23425392	23433186	266	-	chr2	97481952	97503624	13254	+		-294.22	-0.9905	-1	0.74	1.81	1.37	0.61	0.29	0.46
tran	NA	NA	LTCONS_00035940	MTCONS_00038427	chr19	23425392	23433186	266	-	chr2	97481978	97503624	12461	+		-264.32	1	1	0.74	1.81	1.37	0.54	1.07	0.85
cis_mRNA_overlap	0	AntiCompleteIn-mRNAIntron	n339216	MTCONS_00026493	chr16	53521519	53523856	2338	-	chr16	53468351	53526530	7443	+	*		0.9277	1	0.81	0.97	1.12	1.19	1.43	2.6
cis_mRNA_up10k	1477	NA	n380073	MTCONS_00066417	chr7	27154967	27162996	2455	+	chr7	27139879	27153491	9355	-	*		0.903	1	2.35	3.4	0.99	0.62	1.18	0.51
cis_mRNA_dw20k	66	NA	LTCONS_00020702	MTCONS_00019978	chr13	31028655	31029851	1197	.	chr13	31029916	31040081	6544	-	*		0.7559	0.866	0.06	0.03	0.03	0.08	0.05	0.07
cis_mRNA_dw20k	66	NA	LTCONS_00020702	MTCONS_00019979	chr13	31028655	31029851	1197	.	chr13	31029916	31040084	7396	-	*		-0.9658	-0.866	0.06	0.03	0.03	3.6	4.01	3.9
cis_mRNA_up10k	2391	NA	n410902	MTCONS_00014596	chr11	111744780	111750181	2909	-	chr11	111651239	111742390	7596	-	*		-0.9853	-0.866	0.51	0.47	0.69	1	1	0.66
cis_mRNA_up10k	2476	NA	n410902	MTCONS_00014601	chr11	111744780	111750181	2909	-	chr11	111675818	111742305	2038	-	*		-0.9973	-1	0.51	0.47	0.69	4.54	4.68	3.37
cis_mRNA_up10k	5471	NA	n376717	MTCONS_00044739	chr20	46421066	46428516	1447	+	chr20	46282698	46415596	5087	-	*		0.9989	1	0.02	0.11	0	0.34	1.94	0.09
tran	NA	NA	n342551	MTCONS_00074579	chr3	140583917	140586625	2709	-	chrX	75392518	75414076	17491	+		-1865.78	-0.866	-0.866	0.01	0.01	0.03	5.93	5.5	5.07
cis_mRNA_up10k	6563	NA	n382109	MTCONS_00019743	chr13	113556509	113612477	2709	+	chr13	113619039	113755158	12018	+	*		0.7559	0.866	0.24	0.3	0.3	1.08	1.1	1.14
cis_mRNA_overlap	0	mRNA	n382109	MTCONS_00019741	chr13	113556509	113612477	2709	+	chr13	113548664	113755158	7614	+	*		0.863	0.866	0.24	0.3	0.3	3.06	4.03	3.54
cis_mRNA_overlap	0	Lnc-CompleteIn-mRNAExon	n345856	MTCONS_00073398	chr9	125874493	125879825	5222	-	chr9	125871758	126030961	18437	-		-3576.46	0.7393	1	0.57	0.54	1.18	6.47	6.31	6.53

tran	NA	NA	n372736	MTCONS_00026227	chr11	69159846	69167800	1147	-	chr16	29465331	29476301	6115	+		-965.59	0.9542	1	1.11	1.31	1.32	1.34	3.07	3.98
tran	NA	NA	n381362	MTCONS_00024752	chr1	228673895	228674821	927	-	chr15	48429177	48470583	7755	-		-607.48	0.9286	1	0.38	0.42	0.27	5.75	6.46	5.28
tran	NA	NA	n381362	MTCONS_00034245	chr1	228673895	228674821	927	-	chr19	37096218	37128259	11934	+		-773.41	-0.8555	-1	0.38	0.42	0.27	0.92	0.83	0.95
tran	NA	NA	n381362	MTCONS_00024751	chr1	228673895	228674821	927	-	chr15	48429177	48470583	8977	-		-530.16	0.9756	1	0.38	0.42	0.27	2.66	2.69	2.01
tran	NA	NA	n385337	MTCONS_00064984	chr8	7010239	7044422	707	+	chr7	72349936	72422865	10083	+		-451.61	-0.9966	-1	0.14	0.1	0.06	0.41	0.49	0.55
cis_mRNA_dw20k	3314	NA	n338223	MTCONS_00006036	chr1	151097097	151098018	917	+	chr1	151101331	151119142	8735	-	*		-0.9803	-1	0.39	0.07	0.31	2.36	2.5	2.42
cis_mRNA_overlap	0	mRNA	n406513	MTCONS_00072986	chr9	95059640	95087876	4423	-	chr9	95059099	95087918	5028	-		-1735.56	0.8549	1	0	0.39	0.25	0.85	1.05	0.88
tran	NA	NA	n369064	MTCONS_00037945	chr6	74364304	74365877	313	+	chr2	62132721	62373442	11032	+		-319.02	-0.9913	-1	0.13	0	0.11	2.46	3.22	2.47
cis_mRNA_up10k	161	NA	n365616	MTCONS_00040058	chr2	242671429	242673870	1867	-	chr2	242674030	242708835	3610	+	*		-0.9986	-1	1.21	1.15	1	0.22	0.24	0.28
tran	NA	NA	n365616	MTCONS_00065329	chr2	242671429	242673870	1867	-	chr7	99775538	99948836	16269	+		-2003.32	-0.7566	-1	1.21	1.15	1	1.41	1.88	1.91
tran	2316761	NA	n409170	MTCONS_00066892	chr7	75039624	75046065	1751	+	chr7	72716514	72722864	2262	-		-1198.85	-0.9872	-1	6.15	5.93	6.98	0.09	0.14	0
tran	NA	NA	n366895	MTCONS_00036294	chr4	135988388	135992533	667	+	chr19	40528842	40562147	24857	-		-313.38	-0.9401	-0.866	0.05	0	0.05	6.91	8.47	7.45
cis_mRNA_overlap	0	Lnc-AntiOverlap-mRNA	n379026	MTCONS_00024148	chr15	84869932	84874024	740	-	chr15	84840742	84878025	13375	+		-1326.23	-0.9962	-1	0.07	0	0.15	3.42	3.94	2.61
cis_mRNA_overlap	0	Lnc-CompleteIn-mRNAIntron	n337864	MTCONS_00076357	chrX	134452014	134453644	1171	-	chrX	134419588	134478619	6331	-	*		0.8758	0.866	0.03	0.03	0	2.22	2.08	1.93
cis_mRNA_up10k	44	NA	LTCONS_00075989	MTCONS_00074629	chrX	91024063	91034217	6861	-	chrX	91034260	91139166	5096	+	*		0.8691	1	1.51	2.28	1.74	7.71	11.75	10.77
tran	NA	NA	n406993	MTCONS_00021354	chr19	7537723	7538247	525	-	chr14	67999014	68056353	14579	+		-459.76	-0.6025	-0.866	0.21	0.19	0.06	0	0.12	0.12
tran	NA	NA	n406993	MTCONS_00021355	chr19	7537723	7538247	525	-	chr14	67999821	68056353	14181	+		-459.76	-0.7147	-1	0.21	0.19	0.06	1.99	2.2	2.24
tran	1428922	NA	n386306	MTCONS_00047445	chr22	20383731	20398336	2249	-	chr22	21827257	21871792	2404	-		-1411.56	-0.9909	-1	0.53	1.4	3.38	7.76	7.37	5.57
tran	1428922	NA	n386306	MTCONS_00047443	chr22	20383731	20398336	2249	-	chr22	21827257	21871780	3677	-		-1622.95	0.7441	1	0.53	1.4	3.38	1.84	3.15	3.17
tran	1503673	NA	n326384	MTCONS_00005905	chr1	147572534	147579700	537	-	chr1	146032151	146068862	5060	-		-288.1	0.9421	1	1.1	1.02	0.43	2.48	2.27	2
tran	NA	NA	n405284	MTCONS_00051424	chr6	3973650	3974855	333	-	chr3	126156444	126194841	22987	-		-387	0.8302	1	0	0.11	0.83	0.59	0.63	0.65
tran	NA	NA	n344537	MTCONS_00047711	chr2	8017836	8032435	2361	+	chr22	32896532	33448559	15401	-		-1348.18	-0.6636	-0.866	0.18	0.12	0.18	2.72	2.94	1.9
cis_mRNA_overlap	0	Lnc-CompleteIn-mRNAIntron	n340008	MTCONS_00009733	chr10	29933707	29935702	1996	-	chr10	29746276	30024864	6943	-	*		-0.7857	-1	0.14	0.09	0.15	2.49	2.79	1.5
cis_mRNA_overlap	0	Lnc-CompleteIn-mRNAIntron	n340008	MTCONS_00009734	chr10	29933707	29935702	1996	-	chr10	29746276	30025869	7018	-	*		0.9973	1	0.14	0.09	0.15	1.16	0.56	1.22
tran	NA	NA	n324855	MTCONS_00017912	chr21	25180861	25196941	540	-	chr12	63952662	64062424	11449	-		-483.37	0.7426	1	0	0.06	0.25	2.01	2.92	3.01
tran	NA	NA	n324120	MTCONS_00046404	chr9	36297039	36305276	504	+	chr22	20008631	20055472	4110	+		-451.03	-0.973	-1	0.86	1.14	0.76	0.97	0.79	1.13
tran	9840511	NA	n385204	MTCONS_00065090	chr7	66657747	66767406	1015	-	chr7	76607916	76676793	3269	+		-528.9	-0.6792	-1	1.39	0.97	1.36	0.87	1.46	1.36
tran	NA	NA	n405299	MTCONS_00064076	chr6	29855383	29858856	1839	+	chr6_ssto_hap7	2655403	2658749	1578	-		-1001.07	0.9807	0.866	0.14	0.13	0.13	11.2	3.05	0.93
tran	NA	NA	n405299	MTCONS_00063866	chr6	29855383	29858856	1839	+	chr6_dbb_hap3	1203642	1207050	1590	+		-1025.2	0.7544	0.866	0.14	0.13	0.13	18.36	0.43	12.42
tran	NA	NA	n346500	MTCONS_00005688	chr11	58897534	58902351	3890	-	chr1	114258385	114300735	4864	-		-3577.14	0.9966	1	0.11	0.22	0.09	3.91	4.59	3.86
cis_mRNA_overlap	0	Lnc-AntiOverlap-mRNA	LTCONS_00061778	MTCONS_00059411	chr6	2997616	3019996	3605	-	chr6	2988201	3023034	1970	+	*		-0.6795	-1	0.02	0.01	0.06	0.77	1.79	0.73
tran	NA	NA	n325428	MTCONS_00061304	chr2	12371894	12618842	578	+	chr6	149887450	149912879	1999	+		-287.16	-0.9736	-0.866	0.06	0	0.06	6.15	7.14	5.84
cis_mRNA_overlap	0	Lnc-AntiOverlap-mRNA	LTCONS_00034771	MTCONS_00036693	chr19	51161705	51164921	2741	+	chr19	51163129	51223153	9110	-		-4158.2	0.8632	0.866	0.04	0.1	0.1	0.9	1.91	1.4
cis_mRNA_dw20k	129	NA	LTCONS_00032021	MTCONS_00029233	chr17	38333744	38334163	420	.	chr17	38296507	38333616	2820	+	*		0.9628	1	1.36	1.9	2.6	2.31	2.52	3.44
cis_mRNA_dw20k	5279	NA	LTCONS_00032021	MTCONS_00029232	chr17	38333744	38334163	420	.	chr17	38296507	38328466	4215	+	*		0.9992	1	1.36	1.9	2.6	17.77	18.52	19.64
cis_mRNA_dw20k	3785	NA	n371132	MTCONS_00073069	chr9	99254139	99258611	1519	-	chr9	99262395	99382142	5917	-	*		-0.9325	-1	1.46	1.73	1.84	1.63	1.41	1.06
cis_mRNA_dw20k	1895	NA	n338605	MTCONS_00036295	chr19	40526292	40526948	657	+	chr19	40528842	40562478	15125	-	*		-0.8846	-1	0.21	0.15	0	0.26	0.41	0.47
tran	NA	NA	n365460	MTCONS_00072485	chr2	64503035	64503985	704	-	chr9	33025348	33081406	13278	-		-715.04	0.8934	0.866	0	0.2	0.09	0.14	0.18	0.14
cis_mRNA_overlap	0	Lnc-AntiOverlap-mRNA	n342043	MTCONS_00062853	chr6	79943435	79946510	2941	+	chr6	79910612	79944497	1406	-	*		0.992	1	0.79	0.95	1.04	0.27	0.45	0.61
tran	NA	NA	n325617	MTCONS_00033686	chr14	52905849	53062710	553	+	chr19	11998621	12032411	17275	+		-655.05	0.9988	1	2.2	2.43	2.39	1.66	3.92	3.42
tran	NA	NA	n325617	MTCONS_00033685	chr14	52905849	53062710	553	+	chr19	11998607	12032411	17331	+		-655.05	-0.9997	-1	2.2	2.43	2.39	4.11	2.39	2.73
cis_mRNA_overlap	0	mRNA	n385225	MTCONS_00065090	chr7	76668797	76682355	2332	+	chr7	76607916	76676793	3269	+	*		-0.6792	-1	0.87	0.73	0.86	0.87	1.46	1.36
tran	NA	NA	n383723	MTCONS_00047279	chr2	114368816	114384715	1231	-	chr22	51222100	51243982	7371	+		-599.95	0.9762	1	2.61	3.02	2.11	2.31	2.56	1.58
tran	NA	NA	LTCONS_00075314	MTCONS_00021822	chrX	3784555	3800907	2395	-	chr14	102791829	102810753	5226	+		-1717.51	-0.9958	-1	0.14	0.16	0.11	0.58	0.34	0.84
tran	NA	NA	LTCONS_00075314	MTCONS_00021820	chrX	3784555	3800907	2395	-	chr14	102783531	102810753	5199	+		-1712.96	0.8584	1	0.14	0.16	0.11	0.18	0.75	0.11
cis_mRNA_dw20k	153	NA	n411755	MTCONS_00063229	chr6	127587883	127609705	2541	+	chr6	127609857	127653215	2306	-	*		-0.9473	-1	2.49	4.06	3.24	0.69	0	0.16
cis_mRNA_dw20k	12996	NA	n363784	MTCONS_00003909	chr1	1210339	1214764	558	+	chr1	1227759	1243274	8533	-	*		-0.816	-1	1.2	0.47	1.08	13.98	19.76	18.13
cis_mRNA_up10k	5015	NA	n363784	MTCONS_00000083	chr1	1210339	1214764	558	+	chr1	1219778	1227752	4141	+	*		-0.8925	-1	1.2	0.47	1.08	0.68	1.09	0.92
cis_mRNA_overlap	0	Lnc-CompleteIn-mRNAExon	n363784	MTCONS_00000077	chr1	1210339	1214764	558	+	chr1	1210339	1227752	3559	+		-525.18	-0.9895	-1	1.2	0.47	1.08	0.36	1.37	0.37
tran	NA	NA	n379686	MTCONS_00032984	chr5	43578439	43603039	503	-	chr18	45346666	45457071	23239	-		-685.73	-0.994	-1	0.32	0.24	0.36	2.93	3.26	2.83
cis_mRNA_up10k	261	NA	n371171	MTCONS_00072043	chr9	135903518	135905802	567	-	chr9	135906062	135933892	3490	+	*		-0.8719	-1	0.26	0.12	0.17	4.23	5.2	4.41
cis_mRNA_dw20k	16161	NA	n380102	MTCONS_00059152	chr5	172382157	172386380	2067	-	chr5	172402540	172403441	902	.	*		-0.8808	-0.866	0.55	0.65	0.74	0.12	0.04	0.04
tran	NA	NA	n384422	MTCONS_00036294	chr4	13335037	13339924	4888	+	chr19	40528842	40562147	24857	-		-3169.39	0.8308	1	10.08	11.76	11.56	6.91	8.47	7.45
tran	NA	NA	n324418	MTCONS_00074579	chr6	113784898	113795839	507	-	chrX	75392518	75414076	17491	+		-364.22	0.9177	1	0.28	0.27	0.2	5.93	5.5	5.07
tran	NA	NA	n344801	MTCONS_00047712	chr13	56152490	56246639	5153	+	chr22	32896532	33454377	15152	-		-2863.25	-0.922	-1	0.25	0.27	0.29	5.15	5.06	4.49
cis_mRNA_up10k	631	NA	n380866	MTCONS_00016273	chr12	93960515	93965066	354	-	chr12	93965696	93970074	2385	+	*		-0.7367	-1	0.33	0	0.24	1.41	1.88	1.86
cis_mRNA_up10k	561	NA	n380866	MTCONS_00016272	chr12	93960515	93965066	354	-	chr12	93965626	93970074	1994	+	*		0.9929	1	0.33	0	0.24	11.18	8.02	10.68
tran	NA	NA	LTCONS_00059124	MTCONS_00033966	chr5	62072343	62073061	719	.	chr19	21541735	21571384	10481	+		-378.55	-0.7004	-1	0.2	0.05	0	0.46	0.49	3.14
cis_mRNA_dw20k	12065	NA	n345054	MTCONS_00071392	chr9	97067717	97068415	698	+	chr9	97021578	97055653	663	+	*		0.7915	0.866	0.05	0.05	0.11	0.7	0.33	0.93
tran	NA	NA	n338867	MTCONS_00066703	chr2	65816700	65867311	1656	-	chr7	47994874	48019266	10405	-		-2193.59	-0.9952	-1	1.44	1.34	1.29	7.11	8.45	8.88
tran	NA	NA	LTCONS_00067287	MTCONS_00017914	chr7	102909058	102920913	7371	-	chr12	64052387	64062354	5782	-		-2132.89	0.9972	1	6.84	7.61	7.56	1.34	2.18	2.06
tran	NA	NA	n364876	MTCONS_00032984	chr2	15808808	15820608	568	-	chr18	45346666	45457071	23239	-		-693.2	0.9967	1	0.04	0.15	0.02	2.93	3.26	2.83

cis_mRNA_overlap	0	Lnc-AntiOverlap-mRNA	n379303	MTCONS_00013393	chr11	9779839	9832866	2709	+	chr11	9800053	10315759	7680	-	*		-0.982	-1	0.9	0.83	0.62	2.41	2.47	3.43
tran	NA	NA	n387140	MTCONS_00047278	chr2	114368816	114384715	1482	-	chr22	51222100	51243982	7214	+		-730.24	0.9858	1	0.91	1.24	0.99	5.95	8.76	7.06
tran	NA	NA	n346041	MTCONS_00031084	chr16	21829982	21831753	632	-	chr17	40190058	40202750	4056	-		-751.26	0.9989	1	1.29	2.32	3.62	2.79	3.06	3.35
tran	NA	NA	n346079	MTCONS_00047852	chr14	99439145	99439726	488	+	chr22	39708887	39716377	1913	-		-839.75	0.981	0.866	0.08	0.07	0.07	1.31	1	0.92
tran	NA	NA	n367896	MTCONS_00076535	chr5	54317127	54319997	550	-	chrX	153570168	153603006	15521	-		-561.11	0.8936	1	2.35	4.43	4.07	1.27	1.49	1.36
cis_mRNA_dw20k	3964	NA	n345165	MTCONS_00016973	chr12	3900217	3910010	1857	-	chr12	3913973	3982608	3563	-	*		-0.9658	-1	0.44	0.37	0.28	0.83	1.11	1.25
cis_mRNA_dw20k	7841	NA	n363558	MTCONS_00003750	chr1	246953926	246955687	552	+	chr1	246887184	246946086	14034	+	*		0.9655	1	0.42	0.88	0.61	0.43	0.85	0.7
cis_mRNA_overlap	0	mRNA	n341841	MTCONS_00017230	chr12	14915441	14920865	4728	-	chr12	14917326	14924065	6304	-		-2099.81	0.9991	1	1.34	1.64	0.91	1.05	1.39	0.63
cis_mRNA_overlap	0	Lnc-CompleteIn-mRNAIntron	n342169	MTCONS_00061462	chr6	163898510	163899785	1276	+	chr6	163835604	164000803	10685	+	*		0.9849	1	0.44	1.1	0.56	4	5.25	4.43
tran	2042733	NA	n382664	MTCONS_00024149	chr15	82764079	82798323	3634	-	chr15	84841055	84878025	11652	+		-5316.94	0.9316	1	5.36	4.11	3.98	3.39	2.37	1.56
tran	NA	NA	LTCONS_00037048	MTCONS_00066703	chr19	58916679	58919763	2203	-	chr7	47994874	48019266	10405	-		-2833.42	0.9975	1	1.37	1.73	1.89	7.11	8.45	8.88
tran	NA	NA	LTCONS_00042936	MTCONS_00037945	chr20	3388848	3390736	1889	+	chr2	62132721	62373442	11032	+		-1330.47	0.9993	1	1	1.37	1.02	2.46	3.22	2.47
tran	NA	NA	LTCONS_00042936	MTCONS_00025651	chr20	3388848	3390736	1889	+	chr16	619972	634148	5304	+		-1221.11	0.8679	1	1	1.37	1.02	1.64	2.68	2.2
tran	NA	NA	LTCONS_00042936	MTCONS_00025650	chr20	3388848	3390736	1889	+	chr16	619929	634148	4474	+		-1124.61	0.7412	1	1	1.37	1.02	1.25	1.51	1.44
tran	NA	NA	n385792	MTCONS_00015840	chrX	100141957	100143628	1672	-	chr12	54674445	54681404	3966	+		-849.37	-0.6157	-0.866	0.09	0.04	0.04	6.1	8.43	6.45
tran	NA	NA	n385792	MTCONS_00015841	chrX	100141957	100143628	1672	-	chr12	54674485	54681404	1813	+		-755.95	0.967	0.866	0.09	0.04	0.04	5.16	4.1	3.72
cis_mRNA_overlap	0	Lnc-AntiOverlap-mRNA	n338343	MTCONS_00006539	chr1	165880633	166059452	3197	+	chr1	166025736	166136263	6135	-	*		0.9749	1	0.03	0.07	0	0.97	1.45	0.83
tran	NA	NA	n324963	MTCONS_00031779	chr20	48213408	48216046	733	+	chr17	73885041	73893054	3168	-		-714.23	0.9479	1	0.7	0.4	0.24	1.89	0.89	0.86
cis_mRNA_up10k	26	NA	n339172	MTCONS_00026245	chr16	29819966	29823306	2434	-	chr16	29823331	29834356	7335	+	*		0.8903	1	0.31	0.22	0.39	4.04	3.85	5.88
tran	9840511	NA	n385206	MTCONS_00065090	chr7	66752528	66767406	721	-	chr7	76607916	76676793	3269	+		-420.67	0.8501	1	0.41	0.44	0.42	0.87	1.46	1.36
cis_mRNA_overlap	0	Lnc-AntiOverlap-mRNA	LTCONS_00064494	MTCONS_00066417	chr7	27135193	27139975	4288	+	chr7	27139879	27153491	9355	-	*		0.9378	1	21.13	35.98	7.05	0.62	1.18	0.51
tran	NA	NA	n323901	MTCONS_00066260	chrX	25806377	25821582	550	+	chr7	7395856	7551405	5027	-		-282.72	-0.881	-1	0.47	0.88	0.67	3.18	0.61	0.74
tran	NA	NA	n326700	MTCONS_00033998	chr1	13856393	13859620	1007	-	chr19	23257984	23331424	6231	+		-829.06	-0.7998	-1	0.14	0	0.18	1.53	1.77	0.57
cis_mRNA_overlap	0	mRNA	n383354	MTCONS_00032674	chr18	77822752	77830757	5186	+	chr18	77794346	77830757	3161	+	*		0.739	0.866	0.24	0.33	0.53	7.23	9.01	9.01
cis_mRNA_dw20k	14922	NA	n369799	MTCONS_00064879	chr7	63831932	63836925	2028	+	chr7	63773186	63817011	9068	+	*		0.9558	1	0.02	0	0.44	0.96	0	2.84
tran	48132473	NA	n382675	MTCONS_00024565	chr15	83009408	83013526	2586	-	chr15	34671269	34876936	7605	-		-1324.8	0.958	1	0.21	0.06	0.19	0.83	0.41	0.66
tran	NA	NA	n380544	MTCONS_00047711	chr3	128679210	128684200	1065	-	chr22	32896532	33448559	15401	-		-695.88	0.6962	1	0.78	0.97	0.77	2.72	2.94	1.9
cis_mRNA_overlap	0	mRNA	n385667	MTCONS_00071518	chr9	107536633	107540044	3412	+	chr9	107526021	107540044	1984	+		-815.81	0.9187	1	0.4	0.48	0.68	1.09	1.63	1.93
cis_mRNA_up10k	8988	NA	n369262	MTCONS_00060784	chr6	105374369	105379376	412	-	chr6	105388363	105531206	5865	+	*		-0.9362	-1	0.46	0.17	0.08	2.61	2.65	2.7
tran	57971	NA	n385036	MTCONS_00063506	chr6	150319157	150326280	1881	-	chr6	150384250	150390371	1164	-		-659.46	-0.8571	-1	0.21	0.07	0.03	8.05	8.72	10.31
cis_mRNA_up10k	1975	NA	LTCONS_00004125	MTCONS_00000288	chr1	9739681	9747632	1753	-	chr1	9749606	9789271	5613	+	*		0.9961	1	0.3	1.07	0.86	2.81	5.03	4.61
tran	NA	NA	n379961	MTCONS_00047711	chr13	66715500	66806206	844	-	chr22	32896532	33448559	15401	-		-1054.35	0.9434	1	0.36	0.54	0.19	2.72	2.94	1.9
cis_mRNA_dw20k	13724	NA	n373200	MTCONS_00017231	chr12	14898794	14903603	930	-	chr12	14917326	14924065	5525	-	*		0.9817	1	1.43	2.5	1.98	0.86	1.61	1.12
cis_mRNA_up10k	8391	NA	n410514	MTCONS_00017766	chr12	56110076	56113491	841	+	chr12	56078352	56101686	4861	-	*		0.9111	1	0.12	0.19	0.36	1	2.93	3.9
cis_mRNA_dw20k	6834	NA	n363303	MTCONS_00003750	chr1	246952919	246954788	1123	+	chr1	246887184	246946086	14034	+	*		0.9924	1	0.51	0.96	0.85	0.43	0.85	0.7
cis_mRNA_dw20k	18457	NA	n408344	MTCONS_00017855	chr12	57624111	57628718	2097	+	chr12	57647174	57824682	4706	-	*		-0.9986	-1	1.5	1.2	0.55	2.19	2.63	3.4
cis_mRNA_dw20k	5082	NA	n342776	MTCONS_00012484	chr11	82901962	82904242	2281	-	chr11	82868031	82896881	6457	+	*		0.7665	1	1.1	1.37	1.39	1.91	2.17	2.8
tran	2068112	NA	LTCONS_00025268	MTCONS_00024149	chr15	82762149	82772944	7188	-	chr15	84841055	84878025	11652	+		-10220.8	-0.977	-1	1.32	1.35	1.36	3.39	2.37	1.56
tran	NA	NA	n372057	MTCONS_00066703	chr10	99582228	99601058	695	+	chr7	47994874	48019266	10405	-		-886.78	0.8807	1	0.55	0.71	1.05	7.11	8.45	8.88
tran	NA	NA	n345193	MTCONS_00024981	chr12	110667901	110668356	456	-	chr15	66184371	66546141	10164	-		-507.09	0.7832	1	0	0.08	0.07	0.6	1.46	0.83
cis_mRNA_dw20k	1199	NA	n339356	MTCONS_00048758	chr3	49760258	49762050	1793	+	chr3	49726193	49759060	5366	+	*		0.9976	1	0.1	0.07	0.15	0.67	0.52	1
tran	1619518	NA	LTCONS_00066945	MTCONS_00065090	chr7	74923996	74988399	2791	-	chr7	76607916	76676793	3269	+		-1416.49	-0.6308	-0.866	2.74	2.63	2.74	0.87	1.46	1.36
cis_mRNA_overlap	0	mRNA	LTCONS_00066945	MTCONS_00066942	chr7	74923996	74988399	2791	-	chr7	74921877	74988366	1808	-		-981.65	-0.8413	-0.866	2.74	2.63	2.74	5.02	5.8	4.1
cis_mRNA_dw20k	2297	NA	LTCONS_00076590	MTCONS_00074282	chrX	48469878	48471008	1131	.	chrX	48455874	48467582	5788	+	*		-0.6021	-1	1.47	1.41	0.94	6.19	7.54	7.57
cis_mRNA_dw20k	3349	NA	n386976	MTCONS_00030459	chr17	8261731	8263859	2129	-	chr17	8267207	8280699	8648	-	*		0.985	1	9.18	7.07	6.53	8.02	5.95	5.9
cis_mRNA_overlap	0	Lnc-AntiOverlap-mRNA	n377797	MTCONS_00045288	chr21	34636178	34638565	2228	-	chr21	34602231	34636908	2894	+		-1572.1	0.8351	1	0	0.04	0.01	0.24	0.37	0.34
cis_mRNA_overlap	0	Lnc-AntiOverlap-mRNA	n377797	MTCONS_00045287	chr21	34636178	34638565	2228	-	chr21	34602231	34636908	2848	+		-1572.1	-0.9608	-1	0	0.04	0.01	0.62	0	0.31
cis_mRNA_dw20k	16	NA	n410704	MTCONS_00030564	chr17	15902694	15932723	3636	+	chr17	15932738	16118874	10661	-	*		0.9986	0.866	4.73	4.89	2.17	3.18	3.18	3.1
cis_mRNA_overlap	0	Lnc-CompleteIn-mRNAExon	n337711	MTCONS_00004136	chr1	9986451	9987467	1017	-	chr1	9986267	10003453	4977	-		-466.23	0.9912	1	0.21	0	0.56	14.7	13.54	18.1
cis_mRNA_overlap	0	Lnc-CompleteIn-mRNAExon	n337711	MTCONS_00004137	chr1	9986451	9987467	1017	-	chr1	9986267	10003453	5077	-		-466.23	-0.9837	-1	0.21	0	0.56	5.62	5.85	4.75
cis_mRNA_up10k	4483	NA	LTCONS_00014962	MTCONS_00012832	chr11	118973031	118973569	539	.	chr11	118978051	118989668	5128	+	*		-0.8487	-1	0.69	1.13	0.43	0.13	0	1.05
cis_mRNA_dw20k	2334	NA	n337895	MTCONS_00076539	chrX	153625219	153627157	1939	-	chrX	153629490	153640486	4352	-	*		0.9677	1	0.57	1.16	0.91	1.76	2.72	2.1
cis_mRNA_dw20k	12711	NA	n339603	MTCONS_00051569	chr3	138200907	138204597	3691	+	chr3	138217307	138313129	3495	-	*		0.92	1	0.19	0.47	0.31	1.42	2.57	1.47
tran	4092951	NA	n406192	MTCONS_00065090	chr7	72476680	72514966	761	+	chr7	76607916	76676793	3269	+		-481.14	-0.9894	-1	1.01	0.49	0.65	0.87	1.46	1.36
cis_mRNA_up10k	421	NA	n373295	MTCONS_00016506	chr12	112277507	112279765	936	-	chr12	112280185	112340177	3016	+	*		-0.9321	-1	2.3	1.95	1.52	3.54	4.42	4.67
cis_mRNA_dw20k	9763	NA	n364661	MTCONS_00037927	chr2	61159963	61163467	2828	+	chr2	61108682	61150201	2589	+	*		-0.9578	-1	0.15	0.17	0.35	2.12	1.89	1.51
cis_mRNA_overlap	0	Lnc-CompleteIn-mRNAExon	n342503	MTCONS_00048799	chr3	51698597	51699166	570	+	chr3	51572720	51703332	10628	+		-364.32	-0.9994	-1	2.1	3.6	0.65	4.28	3.58	5.04
cis_mRNA_overlap	0	Lnc-CompleteIn-mRNAIntron	n337794	MTCONS_00074656	chrX	100088893	100089583	691	+	chrX	100075348	100098298	4451	+	*		0.9984	1	0.1	0.24	0.19	1.1	1.69	1.45
cis_mRNA_overlap	0	Lnc-AntiOverlap-mRNA	LTCONS_00064184	MTCONS_00066177	chr7	1609709	1629262	5927	+	chr7	1606968	1609736	848	-	*		0.9817	1	0.49	0.81	0.21	5.14	5.66	4.24
tran	NA	NA	n338641	MTCONS_00064836	chr13	33084429	33085024	596	-	chr7	55954858	56008496	3227	+		-625.81	0.9998	1	1.05	1.18	0.82	6.55	6.76	6.15
cis_mRNA_overlap	0	Lnc-CompleteIn-mRNAExon	n409264	MTCONS_00064336	chr7	11013499	11209250	2644	+	chr7	11012967	11209254	3968	+		-1226.07	-0.9379	-1	12.83	13.56	12.42	16.96	12.05	17.01

tran	NA	NA	n384428	MTCONS_00033686	chr4	14473680	14889793	2853	-	chr19	11998621	12032411	17275	+		-1280.41	0.9987	1	10.46	12.17	11.71	1.66	3.92	3.42
tran	NA	NA	n384428	MTCONS_00033685	chr4	14473680	14889793	2853	-	chr19	11998607	12032411	17331	+		-1280.41	-0.9972	-1	10.46	12.17	11.71	4.11	2.39	2.73
tran	NA	NA	n384428	MTCONS_00036294	chr4	14473680	14889793	2853	-	chr19	40528842	40562147	24857	-		-1239.65	0.9062	1	10.46	12.17	11.71	6.91	8.47	7.45
tran	NA	NA	n376433	MTCONS_00066703	chr19	58916679	58919843	2615	-	chr7	47994874	48019266	10405	-		-3136.3	0.7925	1	0.73	0.79	1.09	7.11	8.45	8.88
tran	NA	NA	n342453	MTCONS_00047712	chr16	124682	124960	278	+	chr22	32896532	33454377	15152	-		-454.82	0.6049	0.866	0.58	0.13	0.13	5.15	5.06	4.49
cis_mRNA_overlap	0	Lnc-CompleteIn-mRNAExon	n341470	MTCONS_00069549	chr8	29190580	29192682	2103	-	chr8	29190579	29208297	5533	-		-1250.95	0.9918	1	16.24	22.58	13.47	30.94	45.58	20.43
cis_mRNA_dw20k	6299	NA	n379487	MTCONS_00012486	chr11	82903179	82904704	876	-	chr11	82868137	82896881	10046	+	*		0.9998	1	0.17	0.47	0.27	3.83	5.65	4.4
cis_mRNA_dw20k	16546	NA	LTCONS_00048628	MTCONS_00050600	chr3	44519248	44523433	874	+	chr3	44539978	44552180	2507	-	*		-0.866	-0.866	1.1	1.04	0.98	1.09	1.09	1.5
cis_mRNA_overlap	0	Lnc-CompleteIn-mRNAIntron	n341853	MTCONS_00060363	chr6	43448921	43449332	411	+	chr6	43445597	43474661	3237	+	*		-0.9313	-0.866	0.28	0.08	0.08	0.53	1.1	0.89
tran	4170958	NA	n407488	MTCONS_00026789	chr16	74366304	74402153	2317	-	chr16	70147529	70195347	8044	+		-1498.36	0.9503	1	2.59	2.72	3.01	0.15	0.42	0.61
tran	NA	NA	n323875	MTCONS_00021354	chrX	48323106	48324489	450	-	chr14	67999014	68056353	14579	+		-435.38	0.8846	0.866	0	0.15	0.08	0	0.12	0.12
tran	NA	NA	n323897	MTCONS_00021354	chrX	33670156	33870273	339	+	chr14	67999014	68056353	14579	+		-382.42	-0.7712	-0.866	1.1	0.71	0	0	0.12	0.12
tran	NA	NA	n323897	MTCONS_00064984	chrX	33670156	33870273	339	+	chr7	72349936	72422865	10083	+		-245.46	-0.9692	-1	1.1	0.71	0	0.41	0.49	0.55
tran	NA	NA	n323897	MTCONS_00021355	chrX	33670156	33870273	339	+	chr14	67999821	68056353	14181	+		-382.42	-0.8574	-1	1.1	0.71	0	1.99	2.2	2.24
tran	NA	NA	n324371	MTCONS_00039983	chr6	138305909	138308632	573	-	chr2	238536213	238694823	8109	+		-811.73	0.839	1	1.62	2.22	1.83	1.72	3.49	3.26
tran	NA	NA	n324371	MTCONS_00036294	chr6	138305909	138308632	573	-	chr19	40528842	40562147	24857	-		-261.86	1	1	1.62	2.22	1.83	6.91	8.47	7.45
tran	680888	NA	n385189	MTCONS_00064920	chr7	64498732	64535091	2244	+	chr7	65215978	65229253	2515	+		-1021.68	-0.9978	-1	2.83	2.27	1.98	1.79	2.38	2.61
tran	NA	NA	n372062	MTCONS_00047712	chr10	102140800	102148112	619	+	chr22	32896532	33454377	15152	-		-642.29	-0.9955	-1	0	0.02	0.57	5.15	5.06	4.49
tran	NA	NA	n326556	MTCONS_00041121	chr1	80611637	80612894	580	-	chr2	74699085	74710421	5074	-		-570.75	-1	-1	0.06	0.48	0.12	1.35	0.28	1.2
tran	NA	NA	n324443	MTCONS_00003977	chr6	100075290	100086437	882	+	chr1	2294444	2323088	9762	-		-874.08	0.9677	1	1.56	2.6	2.09	6.94	8.53	8.11
tran	NA	NA	n324443	MTCONS_00036294	chr6	100075290	100086437	882	+	chr19	40528842	40562147	24857	-		-555.52	0.9826	1	1.56	2.6	2.09	6.91	8.47	7.45
tran	NA	NA	n345943	MTCONS_00072236	chr1	16997	29348	830	-	chr9	14430	30786	3251	-		-661.7	0.9921	1	0.04	0	0.12	0.08	0.04	0.13
cis_mRNA_overlap	0	Lnc-AntiOverlap-mRNA	LTCONS_00040515	MTCONS_00037524	chr2	27322221	27333970	1180	-	chr2	27309611	27324926	3470	+	*		0.9874	1	1.05	1.92	1.72	0.37	0.45	0.42
tran	NA	NA	n370256	MTCONS_00075496	chr8	19616323	19620042	905	+	chrX	20168029	20284780	7871	-		-449.51	-0.9193	-1	0.08	0	0.04	1.93	3.94	3.68
tran	4488469	NA	n381444	MTCONS_00010029	chr10	46737615	46762892	4231	-	chr10	51251360	51371677	5208	-		-1852.18	-0.9815	-1	2.59	2.77	2.17	1.06	0.79	3.16
tran	4488469	NA	n381444	MTCONS_00010027	chr10	46737615	46762892	4231	-	chr10	51251360	51371331	3808	-		-1630.86	0.9313	1	2.59	2.77	2.17	0.9	1.64	0.46
tran	NA	NA	n339125	MTCONS_00036272	chr16	23579994	23580903	910	+	chr19	39514209	39523200	2778	-		-560.97	-0.9114	-1	0.51	0.46	0.55	4.48	4.56	4.1
cis_mRNA_overlap	0	mRNA	n371595	MTCONS_00076692	chrY	14774298	14804152	5241	+	chrY	14774265	14972765	9712	+	*		0.9979	1	18.12	0.02	16.5	18.82	0	18.47
cis_mRNA_up10k	37	NA	n337814	MTCONS_00024893	chr15	60771380	60823166	1946	+	chr15	60743660	60771344	3273	-	*		-0.9221	-1	0.09	0.21	0.14	1.3	1.17	1.2
cis_mRNA_up10k	37	NA	n337814	MTCONS_00024889	chr15	60771380	60823166	1946	+	chr15	60711808	60771344	7308	-	*		0.8463	1	0.09	0.21	0.14	0.31	0.62	0.6
cis_mRNA_overlap	0	mRNA	n408367	MTCONS_00059430	chr6	3063572	3066298	648	+	chr6	3064087	3084826	2348	+	*		0.9854	1	0.95	1.59	1.15	1.26	1.86	1.35
cis_mRNA_overlap	0	Lnc-AntiOverlap-mRNA	n410148	MTCONS_00029926	chr17	74730197	74733493	2072	-	chr17	74733490	74775758	3503	+	*		-0.9775	-1	5.45	6.47	3.93	1.54	1.37	1.66
cis_mRNA_dw20k	15251	NA	n342048	MTCONS_00018538	chr12	120565074	120632513	8608	-	chr12	120647763	120703563	4570	-	*		-0.9876	-1	10.61	12.25	11.41	1.07	0.51	0.72
cis_mRNA_dw20k	9836	NA	LTCONS_00027573	MTCONS_00027576	chr16	21888253	21892223	2867	-	chr16	21902058	21910427	1747	-	*		-0.7637	-1	0.63	0.68	0.95	1.04	0.7	0.61
cis_mRNA_overlap	0	mRNA-AntiCompleteIn-LncIntron	n381538	MTCONS_00008795	chr10	97678695	97849986	2950	-	chr10	97803159	97820687	3953	+	*		0.7913	1	0.03	0.04	0	0.24	0.99	0.1
cis_mRNA_dw20k	5882	NA	n384337	MTCONS_00051598	chr3	141655549	141657389	1841	-	chr3	141663270	141747507	9431	-	*		-0.9926	-1	0.61	0.38	0.26	1.49	2.04	2.49
cis_mRNA_dw20k	3	NA	n410905	MTCONS_00066417	chr7	27135713	27139877	775	+	chr7	27139879	27153491	9355	-	*		0.6613	1	38.64	40.29	7.96	0.62	1.18	0.51
tran	NA	NA	n383439	MTCONS_00064893	chr19	23299777	23332763	3202	+	chr7	64126511	64171424	5099	+		-1035.06	0.7222	1	0.48	0.94	0.33	1.54	1.57	0.98
cis_mRNA_overlap	0	mRNA	n383439	MTCONS_00033998	chr19	23299777	23332763	3202	+	chr19	23257984	23331424	6231	+		-1114.55	0.8145	1	0.48	0.94	0.33	1.53	1.77	0.57
tran	NA	NA	n378532	MTCONS_00037006	chr2	7057163	7058810	588	-	chr19	58201202	58220617	13301	-		-587.69	0.9887	1	0.52	2.45	1.75	4.35	5.99	5.18
cis_mRNA_dw20k	2569	NA	n383499	MTCONS_00036154	chr19	36810618	36822610	1898	-	chr19	36825178	36870284	8211	-	*		0.8072	1	0.95	1.18	0.49	0.17	0.37	0.15
cis_mRNA_dw20k	2569	NA	n383499	MTCONS_00036153	chr19	36810618	36822610	1898	-	chr19	36825178	36870284	7852	-	*		0.9897	1	0.95	1.18	0.49	2.91	2.96	2.71
cis_mRNA_overlap	0	Lnc-AntiOverlap-mRNA	n375748	MTCONS_00029672	chr17	59470838	59477096	970	-	chr17	59474205	59486827	6316	+		-2028.89	0.9063	1	6.69	2.77	5.55	1.75	0.55	0.94
cis_mRNA_up10k	5985	NA	LTCONS_00028394	MTCONS_00025836	chr16	3156111	3156579	469	.	chr16	3162563	3170518	2260	+	*		0.8321	1	0	0.22	0.14	1.45	2.44	1.55
cis_mRNA_up10k	5985	NA	LTCONS_00028394	MTCONS_00025837	chr16	3156111	3156579	469	.	chr16	3162563	3170518	2726	+	*		0.9394	1	0	0.22	0.14	2.78	4.29	4.26
cis_mRNA_overlap	0	Lnc-CompleteIn-mRNAExon	n340064	MTCONS_00010226	chr10	73818793	73848767	5304	-	chr10	73818787	73849024	5944	-		-3592.75	0.994	1	0.24	32.36	45.07	2.44	2.76	2.83
cis_mRNA_overlap	0	mRNA	n340064	MTCONS_00010228	chr10	73818793	73848767	5304	-	chr10	73818787	73849909	6820	-		-3655.43	0.833	1	0.24	32.36	45.07	0.06	0.11	0.32
cis_mRNA_up10k	7066	NA	n380208	MTCONS_00066417	chr7	27160556	27162269	989	+	chr7	27139879	27153491	9355	-	*		-0.9882	-0.866	0.74	0.47	0.74	0.62	1.18	0.51
cis_mRNA_dw20k	16221	NA	n411612	MTCONS_00062835	chr6	75994730	76001580	1297	+	chr6	76017800	76044494	3756	-	*		0.8295	1	0.19	0.22	0.1	0.45	1.19	0.24
cis_mRNA_up10k	168	NA	n337888	MTCONS_00076495	chrX	152864799	152865337	539	+	chrX	152853036	152864632	1631	-	*		-0.9975	-1	2.65	4.44	4.67	2.12	1.46	1.31
cis_mRNA_up10k	644	NA	n374790	MTCONS_00024048	chr15	77578764	77712518	317	-	chr15	77713161	77781298	10875	+	*		0.8695	1	1.13	0.23	0.16	2.39	2.21	1.99
cis_mRNA_up10k	627	NA	n374790	MTCONS_00024047	chr15	77578764	77712518	317	-	chr15	77713144	77781298	7244	+	*		-0.9615	-1	1.13	0.23	0.16	0.79	1.29	1.55
tran	NA	NA	LTCONS_00073836	MTCONS_00038426	chr9	67311158	67312019	862	.	chr2	97481952	97503624	13254	+		-733.67	-0.8672	-1	0	0.23	0.22	0.61	0.29	0.46
tran	52060935	NA	LTCONS_00073836	MTCONS_00072360	chr9	67311158	67312019	862	.	chr9	15167067	15250224	7033	-		-666.75	0.7779	1	0	0.23	0.22	2.46	3.34	2.75
tran	NA	NA	LTCONS_00073836	MTCONS_00038427	chr9	67311158	67312019	862	.	chr2	97481978	97503624	12461	+		-733.67	0.9259	1	0	0.23	0.22	0.54	1.07	0.85
tran	13242327	NA	n344662	MTCONS_00029069	chr17	43622980	43662302	428	-	chr17	30334413	30380654	3751	+		-388.01	0.8581	1	0.71	0.35	0.76	1.86	1.79	1.97
cis_mRNA_up10k	7	NA	LTCONS_00012937	MTCONS_00014816	chr11	124670346	124676641	2525	+	chr11	124635844	124670340	7137	-	*		0.9991	1	0.41	0.51	0.33	1.58	1.72	1.45
tran	NA	NA	n378156	MTCONS_00035197	chr10	31596922	31607861	320	-	chr19	812518	823297	3947	-		-236.97	-0.9305	-1	0.75	1.43	0.96	16.71	12.57	14.07
tran	NA	NA	n410154	MTCONS_00076600	chr5	133307566	133340657	2071	-	chrX	80185011	80186350	1340	.		-693.02	-0.7778	-1	24.96	27.68	37.3	0.11	0.06	0.05
cis_mRNA_dw20k	226	NA	n341742	MTCONS_00071498	chr9	104181299	104185086	3788	+	chr9	104161163	104181074	11258	+	*		0.8688	1	0.23	0.33	0.26	4.99	6.86	6.41
cis_mRNA_dw20k	49	NA	n381420	MTCONS_00009733	chr10	29745065	29746228	1164	+	chr10	29746276	30024864	6943	-	*		-0.99	-1	0.06	0.05	0.16	2.49	2.79	1.5
cis_mRNA_dw20k	49	NA	n381420	MTCONS_00009734	chr10	29745065	29746228	1164	+	chr10	29746276	30025869	7018	-	*		0.6351	1	0.06	0.05	0.16	1.16	0.56	1.22
tran	NA	NA	n346055	MTCONS_00074579	chr6	127185939	127194415	1017	-	chrX	75392518	75414076	17491	+		-814.55	-0.982	-1	0.07	0.08	0.1	5.93	5.5	5.07
tran	NA	NA	n381447	MTCONS_00015841	chr10	47133297	47133836	540	-	chr12	54674485	54681404	1813	+		-320.23	0.9818	1	0.23	0.1	0	5.16	4.1	3.72
tran	NA	NA	n367973	MTCONS_00074629	chr5	142869420	142910915	560	-	chrX	91034260	91139166	5096	+		-705.63	0.8419	1	0.2	0.36	0.24	7.71	11.75	10.77

cis_mRNA_overlap	0	mRNA	n411657	MTCONS_00032778	chr18	11883472	11908796	2992	-	chr18	11883141	11908796	4433	-		-1691.34	0.7844	1	0	0.07	0.08	0.15	0.22	0.41
cis_mRNA_overlap	0	Lnc-CompleteIn-mRNAExon	n341107	MTCONS_00012630	chr11	103024204	103041679	1948	+	chr11	102980109	103350591	13729	+		-883.32	0.9871	1	0.75	0.19	0.2	3.25	1.64	1.94
cis_mRNA_overlap	0	mRNA	LTCONS_00025849	MTCONS_00025852	chr16	3331264	3341617	5167	+	chr16	3333487	3349196	2470	+		-1743.32	0.8966	1	0.22	0.21	0.29	0.52	0.27	0.73
cis_mRNA_dw20k	13	NA	n339281	MTCONS_00028102	chr16	72127834	72146802	4226	+	chr16	72146814	72342088	9157	-	*		0.995	1	5.75	5.1	10.16	2.63	2.59	4.46
tran	NA	NA	n324286	MTCONS_00026227	chr6	169951339	169952123	694	-	chr16	29465331	29476301	6115	+		-652.97	0.968	1	2.18	2.71	2.77	1.34	3.07	3.98
cis_mRNA_dw20k	17354	NA	n407766	MTCONS_00043960	chr20	62584737	62588338	3571	+	chr20	62557618	62567384	5611	+	*		0.9854	1	0.47	0.7	0.59	1.43	1.94	1.62
cis_mRNA_overlap	0	Lnc-CompleteIn-mRNAIntron	n387266	MTCONS_00048032	chr22	50171538	50173958	2421	-	chr22	50166930	50221198	4589	-	*		0.9694	1	1.55	1.94	2.14	4.1	4.42	4.82
cis_mRNA_overlap	0	Lnc-CompleteIn-mRNAIntron	n387266	MTCONS_00048033	chr22	50171538	50173958	2421	-	chr22	50166930	50221199	4197	-	*		-0.9504	-1	1.55	1.94	2.14	11.38	10.68	9.56
tran	NA	NA	LTCONS_00003817	MTCONS_00076760	chr1	14050	29381	2735	-	chrY	59354140	59358344	1999	+		-1487.05	-0.9147	-1	1	0.39	0.6	5.13	12.17	7.17
cis_mRNA_overlap	0	mRNA	n364044	MTCONS_00003750	chr1	246939995	246956015	1859	+	chr1	246887184	246946086	14034	+		-1196.48	0.9828	1	0.35	1	0.88	0.43	0.85	0.7
tran	NA	NA	n324623	MTCONS_00071498	chr6	100135	101639	751	+	chr9	104161163	104181074	11258	+		-846.03	0.7181	1	1.44	2.43	1.49	4.99	6.86	6.41
tran	NA	NA	n407951	MTCONS_00033967	chr7	64254771	64294025	2585	+	chr19	21541735	21571384	6244	+		-908.75	0.7104	1	0.79	1.56	1.47	0.34	0.87	0.43
tran	NA	NA	n386555	MTCONS_00072236	chr1	16858	29370	1148	-	chr9	14430	30786	3251	-		-888.96	0.9965	1	0.71	0.09	1.29	0.08	0.04	0.13
tran	NA	NA	LTCONS_00024395	MTCONS_00076760	chr15	102500501	102516854	3942	+	chrY	59354140	59358344	1999	+		-1611.79	-0.9988	-1	0.17	0.14	0.16	5.13	12.17	7.17
cis_mRNA_dw20k	18458	NA	n409234	MTCONS_00034722	chr19	50180409	50191704	1566	+	chr19	50145343	50161952	4862	+	*		0.855	1	6.16	6.56	5.61	0.61	0.97	0.58
cis_mRNA_overlap	0	Lnc-AntiOverlap-mRNA	n371651	MTCONS_00076692	chrY	14774286	14775639	745	-	chrY	14774265	14972765	9712	+		-950.4	0.9849	1	0.05	0	0.04	18.82	0	18.47
cis_mRNA_dw20k	791	NA	n380962	MTCONS_00073634	chr9	136892273	136894637	802	+	chr9	136895427	136933147	5657	-	*		-0.9899	-1	0	0.13	0.33	2.44	2.06	1.01
cis_mRNA_overlap	0	mRNA	LTCONS_00063235	MTCONS_00063229	chr6	127609857	127664849	2756	-	chr6	127609857	127653215	2306	-		-840.7	-0.9682	-1	0.67	1.28	1	0.69	0	0.16
cis_mRNA_overlap	0	Lnc-AntiOverlap-mRNA	n410040	MTCONS_00017778	chr12	56223392	56230030	3070	+	chr12	56214649	56224054	3520	-	*		0.9964	1	0.16	0.01	0.05	0.88	0.18	0.31
cis_mRNA_up10k	2850	NA	n344978	MTCONS_00068295	chr8	28480295	28556150	1885	+	chr8	28558999	28612901	8025	+	*		-0.8614	-0.866	0.02	0.04	0.02	1.71	1.4	2.03
tran	NA	NA	n324648	MTCONS_00039539	chr22	46819191	46823030	372	-	chr2	204103164	204171345	11508	+		-556.15	-0.9971	-1	1.27	2.44	2.9	0.99	0.69	0.61
cis_mRNA_overlap	0	Lnc-AntiOverlap-mRNA	LTCONS_00025310	MTCONS_00024148	chr15	84866139	84898920	4502	-	chr15	84840742	84878025	13375	+	*		0.9998	1	0.95	1.27	0.41	3.42	3.94	2.61
cis_mRNA_dw20k	18313	NA	n378624	MTCONS_00045359	chr21	38593720	38610045	520	+	chr21	38445479	38575408	6952	+	*		0.885	1	0.14	0.62	0.37	63.33	78.9	63.6
tran	NA	NA	n326404	MTCONS_00024149	chr1	144087482	144093754	814	-	chr15	84841055	84878025	11652	+		-506.38	0.9477	1	0.23	0.17	0.16	3.39	2.37	1.56
tran	NA	NA	n385714	MTCONS_00031084	chr9	138363011	138372564	875	-	chr17	40190058	40202750	4056	-		-691.55	0.96	1	0	0.11	0.15	2.79	3.06	3.35
tran	NA	NA	n410247	MTCONS_00071498	chr6	3591246	3592810	549	-	chr9	104161163	104181074	11258	+		-586.67	-1	-1	0.77	0	0.18	4.99	6.86	6.41
tran	NA	NA	n381522	MTCONS_00022667	chr10	89084496	89102315	1111	-	chr14	77249021	77279283	7871	-		-968	0.9925	1	0.36	0.41	0.35	1.82	2.33	1.8
cis_mRNA_overlap	0	Lnc-CompleteIn-mRNAExon	n381443	MTCONS_00009913	chr10	45950066	45952578	2513	-	chr10	45949909	46090354	6388	-		-1614.89	0.8714	1	1.5	2.1	1.81	1.35	2.52	1.39
cis_mRNA_overlap	0	Lnc-CompleteIn-mRNAExon	n381443	MTCONS_00009914	chr10	45950066	45952578	2513	-	chr10	45949909	46090354	6250	-		-1603.34	-0.9861	-1	1.5	2.1	1.81	1.82	0.58	1.36
cis_mRNA_up10k	410	NA	n379945	MTCONS_00009602	chr10	15211104	15221737	2785	+	chr10	15147771	15210695	5166	-	*		0.9999	1	0.56	1.18	0.84	0.36	0.52	0.43
cis_mRNA_up10k	410	NA	n379945	MTCONS_00009601	chr10	15211104	15221737	2785	+	chr10	15145428	15210695	1702	-	*		-0.8726	-1	0.56	1.18	0.84	3.75	3.36	3.39
tran	5959878	NA	LTCONS_00024238	MTCONS_00024149	chr15	90837902	90838450	217	+	chr15	84841055	84878025	11652	+		-361.85	0.9056	1	0.81	0.37	0.36	3.39	2.37	1.56
cis_mRNA_overlap	0	Lnc-AntiOverlap-mRNA	n379300	MTCONS_00035688	chr19	14247991	14282071	2856	+	chr19	14258549	14276912	8632	-	*		0.6758	0.866	0.01	0.03	0.03	0.71	1.15	0.81
cis_mRNA_overlap	0	Lnc-AntiOverlap-mRNA	n379300	MTCONS_00035689	chr19	14247991	14282071	2856	+	chr19	14258549	14316997	7878	-	*		-0.6025	-0.866	0.01	0.03	0.03	1.09	1.07	0.94
tran	NA	NA	n406138	MTCONS_00017912	chr7	102815462	102920759	3698	-	chr12	63952662	64062424	11449	-		-1673.87	0.7243	1	0.31	0.4	0.73	2.01	2.92	3.01
tran	NA	NA	n340982	MTCONS_00030540	chr11	17439193	17447011	924	-	chr17	15331553	15466945	10874	-		-725.42	-0.9969	-1	0.08	0	0.07	0.45	1.09	0.58
cis_mRNA_overlap	0	Lnc-CompleteIn-mRNAIntron	n324912	MTCONS_00043908	chr20	61909873	61918318	533	+	chr20	61904165	61921232	5348	+	*		-0.9336	-1	0.07	0.38	0.56	4.37	4.18	3.76
cis_mRNA_overlap	0	Lnc-CompleteIn-mRNAIntron	n324912	MTCONS_00043905	chr20	61909873	61918318	533	+	chr20	61904152	61921232	3347	+	*		-0.9731	-1	0.07	0.38	0.56	8.85	7.25	5.13
tran	NA	NA	n325895	MTCONS_00033686	chr10	107239344	107252103	450	-	chr19	11998621	12032411	17275	+		-247.42	-0.7403	-1	0.34	0.06	0.31	1.66	3.92	3.42
tran	NA	NA	n325895	MTCONS_00033685	chr10	107239344	107252103	450	-	chr19	11998607	12032411	17331	+		-247.42	0.7236	1	0.34	0.06	0.31	4.11	2.39	2.73
tran	NA	NA	n325895	MTCONS_00036294	chr10	107239344	107252103	450	-	chr19	40528842	40562147	24857	-		-222.99	-0.9689	-1	0.34	0.06	0.31	6.91	8.47	7.45
tran	1352225	NA	n407899	MTCONS_00038601	chr2	110656009	110683202	2552	+	chr2	109150836	109303785	4719	+		-1225.8	-1	-1	0.46	0.48	0.32	3.17	3.08	3.86
tran	93641231	NA	n345550	MTCONS_00041120	chr2	168351651	168394068	1044	+	chr2	74699085	74710421	5373	-		-838.24	0.9953	1	1.02	1.42	1.57	2.19	2.58	2.8
tran	NA	NA	n345550	MTCONS_00051598	chr2	168351651	168394068	1044	+	chr3	141663270	141747507	9431	-		-808.94	0.9803	1	1.02	1.42	1.57	1.49	2.04	2.49
cis_mRNA_dw20k	3550	NA	n339865	MTCONS_00032971	chr18	44383187	44383857	671	-	chr18	44387406	44486283	7035	-	*		-0.9393	-1	0.11	0.64	0.24	2.33	2.15	2.23
cis_mRNA_dw20k	14760	NA	n339865	MTCONS_00032974	chr18	44383187	44383857	671	-	chr18	44398616	44486293	11003	-	*		0.9941	1	0.11	0.64	0.24	1.36	1.79	1.42
cis_mRNA_overlap	0	Lnc-CompleteIn-mRNAIntron	n337954	MTCONS_00046404	chr22	20012193	20012441	249	+	chr22	20008631	20055472	4110	+	*		0.9849	1	0.17	0	0.45	0.97	0.79	1.13
cis_mRNA_dw20k	5	NA	n379110	MTCONS_00065917	chr7	149564861	149570934	2974	-	chr7	149535413	149564857	9254	+	*		-0.9485	-1	4.02	3.45	2.95	1.73	2.3	2.42
cis_mRNA_up10k	842	NA	n406845	MTCONS_00008074	chr10	31605457	31608024	2213	-	chr10	31608865	31818836	7880	+	*		-0.7453	-1	0.41	0.32	0.62	1.19	2.43	1.16
cis_mRNA_up10k	98	NA	n369049	MTCONS_00062673	chr6	47277791	47280162	383	+	chr6	47199268	47277694	5159	-	*		-0.7999	-1	0	0.55	0.36	7.06	5.48	6.96
tran	NA	NA	n325607	MTCONS_00037001	chr14	100485068	100492267	739	+	chr19	58052250	58071227	7132	-		-659.4	0.6732	0.866	0	0.09	0.09	1.26	1.93	1.41
tran	NA	NA	n325607	MTCONS_00028316	chr14	100485068	100492267	739	+	chr16	87731658	87800405	7775	-		-724.85	-0.9912	-0.866	0	0.09	0.09	0.79	0.73	0.72
cis_mRNA_dw20k	88	NA	n369399	MTCONS_00063088	chr6	111616704	111619807	325	+	chr6	111619894	111804624	11379	-	*		0.9957	1	0.24	0.45	0	1.19	1.54	0.9
tran	NA	NA	n332848	MTCONS_00007143	chr2	232189116	232189629	514	+	chr1	213165404	213188836	5075	-		-551.81	-0.7293	-0.866	0	0.26	0.37	2.09	2.09	2.06
tran	NA	NA	n406144	MTCONS_00047278	chr8	158345	182318	5788	-	chr22	51222100	51243982	7214	+		-2534.86	0.8943	1	3.59	6.11	5.63	5.95	8.76	7.06
tran	NA	NA	LTCONS_00069238	MTCONS_00047279	chr8	155778	182318	8285	-	chr22	51222100	51243982	7371	+		-3313.15	-0.9988	-1	0.28	0.19	0.49	2.31	2.56	1.58
tran	NA	NA	n408336	MTCONS_00033686	chr20	5451842	5457780	3799	+	chr19	11998621	12032411	17275	+		-2122.83	0.9687	1	1.72	2.1	1.93	1.66	3.92	3.42
tran	NA	NA	n408336	MTCONS_00039983	chr20	5451842	5457780	3799	+	chr2	238536213	238694823	8109	+		-2753.44	0.9417	1	1.72	2.1	1.93	1.72	3.49	3.26
tran	NA	NA	n408336	MTCONS_00033685	chr20	5451842	5457780	3799	+	chr19	11998607	12032411	17331	+		-2122.83	-0.9624	-1	1.72	2.1	1.93	4.11	2.39	2.73
tran	NA	NA	n408336	MTCONS_00036294	chr20	5451842	5457780	3799	+	chr19	40528842	40562147	24857	-		-2059.24	0.9722	1	1.72	2.1	1.93	6.91	8.47	7.45

tran	NA	NA	n371860	MTCONS_00072360	chr10	38064299	38073059	712	-	chr9	15167067	15250224	7033	-		-656.71	0.7694	1	0.72	1.07	1.06	2.46	3.34	2.75
tran	NA	NA	n381782	MTCONS_00047279	chr11	118873673	118874303	631	-	chr22	51222100	51243982	7371	+		-459.81	-0.9871	-1	0.06	0	0.15	2.31	2.56	1.58
cis_mRNA_overlap	0	Lnc-AntiOverlap-mRNA	n342280	MTCONS_00024160	chr15	85177461	85188902	1903	-	chr15	85144239	85185987	15744	+		-2969.14	0.8975	1	0.13	0.79	0.21	0.48	0.74	0.62
cis_mRNA_dw20k	7231	NA	n378535	MTCONS_00061545	chr6	170188886	170198921	2418	-	chr6	170151699	170181656	4339	+	*		0.9998	1	0.88	1.03	0.72	1.58	1.61	1.55
tran	NA	NA	n367704	MTCONS_00041018	chr5	7373230	7379903	577	+	chr2	69684995	69870977	20533	-		-562.6	0.9148	0.866	0.1	0.18	0.1	1.51	2.79	2.03
cis_mRNA_dw20k	2060	NA	n406954	MTCONS_00075219	chrX	152965947	152990201	1643	-	chrX	152954921	152963888	6707	+	*		-0.9689	-1	16.09	11.98	14.13	0.74	1.37	0.9
tran	1507467	NA	LTCONS_00005930	MTCONS_00005904	chr1	147574207	147578137	2587	-	chr1	146032151	146066741	4836	-		-1344.89	0.9084	1	1.28	1.78	1.57	0.67	1.03	0.74
tran	NA	NA	n384940	MTCONS_00036294	chr6	46459789	46501215	1342	+	chr19	40528842	40562147	24857	-		-786.44	0.814	1	9.18	13.04	12.7	6.91	8.47	7.45
cis_mRNA_up10k	23	NA	n341029	MTCONS_00013726	chr11	46867978	46895947	1504	+	chr11	46765049	46867956	6870	-	*		-0.7627	-1	0	0.06	0.02	44.6	32.91	33.05
tran	NA	NA	LTCONS_00052282	MTCONS_00011534	chr3	156432178	156433270	1093	.	chr11	28129798	28156930	1565	+		-1012.74	0.9998	1	0.03	0.21	0.11	0.47	0.54	0.5
cis_mRNA_up10k	121	NA	n369533	MTCONS_00065151	chr7	86954666	86974831	468	-	chr7	86974951	87023016	2596	+	*		-0.9872	-1	0.53	0	0.29	0.09	0.58	0.38
cis_mRNA_dw20k	8714	NA	n374280	MTCONS_00021126	chr14	45552347	45552901	467	-	chr14	45431403	45543634	6414	+	*		-0.9924	-0.866	0	0.07	0.07	5.73	5.08	4.98
tran	NA	NA	n376908	MTCONS_00066562	chr20	46683233	46692077	815	+	chr7	36363280	36406878	4088	-		-502.22	-0.9366	-1	0.39	0.29	0.12	0.52	0.53	1.03
cis_mRNA_up10k	130	NA	n338573	MTCONS_00036177	chr19	37341832	37403930	584	+	chr19	37305033	37341703	6388	-	*		-0.9722	-1	1.26	0.88	1.1	0	1.26	0.79
cis_mRNA_overlap	0	Lnc-AntiOverlap-mRNA	n407440	MTCONS_00076539	chrX	153626571	153630680	2155	+	chrX	153629490	153640486	4352	-		-2572	0.9994	1	9.7	10.69	10.02	1.76	2.72	2.1
tran	NA	NA	LTCONS_00073815	MTCONS_00015840	chr9	4944474	4944708	235	.	chr12	54674445	54681404	3966	+		-454.6	0.6804	1	0.36	0.68	0.65	6.1	8.43	6.45
tran	NA	NA	n377638	MTCONS_00033686	chr14	62037256	62125389	1632	+	chr19	11998621	12032411	17275	+		-864.31	0.9569	1	17.4	21.17	19.34	1.66	3.92	3.42
tran	NA	NA	n377638	MTCONS_00039983	chr14	62037256	62125389	1632	+	chr2	238536213	238694823	8109	+		-2027.43	0.9261	1	17.4	21.17	19.34	1.72	3.49	3.26
tran	NA	NA	n377638	MTCONS_00033685	chr14	62037256	62125389	1632	+	chr19	11998607	12032411	17331	+		-864.31	-0.9495	-1	17.4	21.17	19.34	4.11	2.39	2.73
tran	NA	NA	n377638	MTCONS_00036294	chr14	62037256	62125389	1632	+	chr19	40528842	40562147	24857	-		-973.81	0.9815	1	17.4	21.17	19.34	6.91	8.47	7.45
cis_mRNA_up10k	9259	NA	n346546	MTCONS_00070538	chr8	145168558	145169578	926	+	chr8	145153521	145159300	2318	-	*		-0.9347	-1	0.13	0.53	0.43	1.48	0.92	1.24
cis_mRNA_dw20k	15053	NA	n342597	MTCONS_00008287	chr10	51723005	51724217	1213	+	chr10	51623350	51707953	2944	+	*		0.8211	1	0.89	1.23	0.75	11.96	13.28	5.17
cis_mRNA_overlap	0	Lnc-AntiOverlap-mRNA	LTCONS_00072191	MTCONS_00073766	chr9	140083054	140084826	1232	+	chr9	140069236	140083057	4059	-	*		-0.9436	-1	14.54	10.45	12.85	1.29	1.91	1.73
tran	NA	NA	n344625	MTCONS_00033686	chr5	25433945	25445978	426	-	chr19	11998621	12032411	17275	+		-601.45	0.9953	1	0	0.08	0.07	1.66	3.92	3.42
tran	NA	NA	n344625	MTCONS_00033685	chr5	25433945	25445978	426	-	chr19	11998607	12032411	17331	+		-601.45	-0.9974	-1	0	0.08	0.07	4.11	2.39	2.73
cis_mRNA_overlap	0	Lnc-CompleteIn-mRNAIntron	n385467	MTCONS_00070424	chr8	141111257	141113775	2519	-	chr8	140740088	141467861	6770	-	*		-0.9333	-1	0.04	0.03	0.09	1.17	1.25	1.09
cis_mRNA_up10k	1249	NA	LTCONS_00063847	MTCONS_00063846	chr6_cox_hap2	2223297	2224174	766	-	chr6_cox_hap2	2220561	2222049	1123	-	*		-0.9352	-1	4.35	4.19	4.48	5.57	9.55	5.07
cis_mRNA_dw20k	382	NA	n340144	MTCONS_00009062	chr10	114590928	114592210	1283	+	chr10	114206756	114590547	13265	+	*		0.8429	1	0.08	0.17	0.1	2.59	3.51	3.26
tran	NA	NA	n325943	MTCONS_00015352	chr10	79723605	79730766	520	+	chr12	14538004	14655865	9006	+		-554.45	-0.9589	-0.866	0.07	0.07	0.06	9.57	7.86	13.72
tran	NA	NA	n325943	MTCONS_00062442	chr10	79723605	79730766	520	+	chr6	34231228	34360518	10114	-		-288.08	-0.949	-0.866	0.07	0.07	0.06	47.14	47.98	49.75
tran	NA	NA	n325943	MTCONS_00015351	chr10	79723605	79730766	520	+	chr12	14537918	14655865	9095	+		-554.45	0.9755	0.866	0.07	0.07	0.06	7.33	5.93	1.25
tran	NA	NA	n325943	MTCONS_00027704	chr10	79723605	79730766	520	+	chr16	30541977	30546205	3041	-		-546.34	-0.8158	-0.866	0.07	0.07	0.06	3.31	2.52	3.88
tran	NA	NA	n325281	MTCONS_00003977	chr2	66407985	66409279	1024	+	chr1	2294444	2323088	9762	-		-522.3	0.7234	1	11.78	14.03	11.85	6.94	8.53	8.11
tran	NA	NA	n325281	MTCONS_00036294	chr2	66407985	66409279	1024	+	chr19	40528842	40562147	24857	-		-481.18	0.9491	1	11.78	14.03	11.85	6.91	8.47	7.45
cis_mRNA_overlap	0	mRNA	n411603	MTCONS_00026601	chr16	58534047	58547523	3261	+	chr16	58534038	58547540	3285	+		-1954.85	-0.8448	-1	8.34	7.56	9.25	19.52	22.09	19.51
cis_mRNA_dw20k	6236	NA	n411603	MTCONS_00027906	chr16	58534047	58547523	3261	+	chr16	58553758	58663759	8522	-	*		-0.9184	-1	8.34	7.56	9.25	0.94	2.63	0.59
cis_mRNA_overlap	0	Lnc-AntiOverlap-mRNA	n375746	MTCONS_00029671	chr17	59470821	59474207	1225	-	chr17	59474205	59486827	6081	+	*		-0.7793	-1	1.45	0.59	0.5	1.54	1.84	2.58
tran	NA	NA	n324722	MTCONS_00031083	chr22	18974408	18975991	375	+	chr17	40190058	40196297	4596	-		-485.67	0.8623	1	0.31	0.57	0.09	2.26	2.3	1.43
tran	NA	NA	n324722	MTCONS_00036272	chr22	18974408	18975991	375	+	chr19	39514209	39523200	2778	-		-261.78	0.9178	1	0.31	0.57	0.09	4.48	4.56	4.1
tran	NA	NA	LTCONS_00032015	MTCONS_00017475	chr17	27530199	27531596	1398	.	chr12	44187526	44200178	3217	-		-2555.79	0.9328	0.866	0	0.02	0.02	0.86	1.4	1.71
tran	NA	NA	LTCONS_00032015	MTCONS_00017474	chr17	27530199	27531596	1398	.	chr12	44187526	44200178	3110	-		-2582.97	-0.989	-0.866	0	0.02	0.02	2.6	2.23	2.16
tran	NA	NA	LTCONS_00042852	MTCONS_00047279	chr2	243160378	243160804	427	.	chr22	51222100	51243982	7371	+		-889.5	0.7082	1	0.09	0.65	0.08	2.31	2.56	1.58
cis_mRNA_overlap	0	AntiCompleteIn-mRNAIntron	n341585	MTCONS_00070213	chr8	110656344	110660313	3075	+	chr8	110586396	110704053	2994	-	*		-0.9484	-1	0.01	0.02	0	0.7	0.66	0.85
tran	NA	NA	n342329	MTCONS_00076002	chr1	172721726	172722064	339	+	chrX	99880885	99891854	5056	-		-207.18	-0.7397	-1	0.04	0	0.52	2.12	2.85	1.88
tran	70516935	NA	n375912	MTCONS_00032674	chr18	7272467	7277412	786	+	chr18	77794346	77830757	3161	+		-654.9	0.9529	0.866	0.14	0.43	0.34	7.23	9.01	9.01
tran	NA	NA	n382500	MTCONS_00074579	chr15	38431798	38519066	2469	-	chrX	75392518	75414076	17491	+		-3172.11	-0.9555	-1	16.42	16.62	17.28	5.93	5.5	5.07
tran	1389103	NA	n406511	MTCONS_00036154	chr19	35417807	35436076	2664	+	chr19	36825178	36870284	8211	-		-1209.33	-0.9759	-1	3.05	2.64	3.23	0.17	0.37	0.15
tran	1424818	NA	n386309	MTCONS_00047445	chr22	20383731	20402440	2489	-	chr22	21827257	21871792	2404	-		-1496.84	1	1	1.25	1.13	0.59	7.76	7.37	5.57
tran	1424818	NA	n386309	MTCONS_00047443	chr22	20383731	20402440	2489	-	chr22	21827257	21871780	3677	-		-1799.41	-0.6505	-1	1.25	1.13	0.59	1.84	3.15	3.17
tran	NA	NA	n378985	MTCONS_00036294	chr6	132223103	132241705	2305	+	chr19	40528842	40562147	24857	-		-1201.9	0.9846	1	0.03	0.05	0.04	6.91	8.47	7.45
tran	NA	NA	n405467	MTCONS_00047279	chr2	54756359	54756978	620	+	chr22	51222100	51243982	7371	+		-334.35	-0.9991	-1	0.82	0.7	1.26	2.31	2.56	1.58
cis_mRNA_dw20k	1224	NA	n406609	MTCONS_00071422	chr9	98782014	98784037	1721	-	chr9	98637246	98780791	10926	+	*		0.8972	1	0.08	0.14	0.09	6.78	9.15	8.15
cis_mRNA_overlap	0	Lnc-CompleteIn-mRNAIntron	n326399	MTCONS_00005872	chr1	144940123	144941802	727	-	chr1	144890575	145076193	5495	-	*		0.9149	1	0.45	0.68	0.8	2.51	2.93	3.95
cis_mRNA_overlap	0	Lnc-CompleteIn-mRNAIntron	n326399	MTCONS_00005871	chr1	144940123	144941802	727	-	chr1	144890575	145039994	4848	-	*		0.9699	1	0.45	0.68	0.8	7.16	7.39	7.68
cis_mRNA_dw20k	6342	NA	n366219	MTCONS_00048904	chr3	58419678	58468999	309	+	chr3	58318617	58413337	4519	+	*		-0.7495	-1	0.13	0	0.12	1.12	2.46	2.11
cis_mRNA_dw20k	6342	NA	n366219	MTCONS_00048902	chr3	58419678	58468999	309	+	chr3	58318615	58413337	4515	+	*		0.655	1	0.13	0	0.12	2.04	0.9	1.05
cis_mRNA_up10k	907	NA	n340011	MTCONS_00008074	chr10	31605457	31607959	2503	-	chr10	31608865	31818836	7880	+	*		0.9445	1	2.37	2.48	2.31	1.19	2.43	1.16
cis_mRNA_overlap	0	Lnc-AntiOverlap-mRNA	n407997	MTCONS_00041120	chr2	74710200	74721691	2820	+	chr2	74699085	74710421	5373	-	*		0.999	1	1.32	1.92	2.32	2.19	2.58	2.8
cis_mRNA_dw20k	3	NA	LTCONS_00061058	MTCONS_00063229	chr6	127587881	127609855	18321	+	chr6	127609857	127653215	2306	-	*		-0.986	-1	1.64	2.12	2.09	0.69	0	0.16
tran	NA	NA	n339024	MTCONS_00033686	chr2	176047421	176048342	922	+	chr19	11998621	12032411	17275	+		-772.98	-0.9859	-1	0.08	0	0.03	1.66	3.92	3.42
tran	NA	NA	n339024	MTCONS_00033685	chr2	176047421	176048342	922	+	chr19	11998607	12032411	17331	+		-772.98	0.9815	1	0.08	0	0.03	4.11	2.39	2.73
tran	NA	NA	n324119	MTCONS_00034284	chr9	36322947	36324969	388	-	chr19	37996748	38034296	8612	+		-382.8	-0.8492	-1	0	0.45	0.18	0.74	0.63	0.64
cis_mRNA_dw20k	11890	NA	n382935	MTCONS_00027594	chr16	23457791	23458603	813	-	chr16	23470492	23521815	11188	-	*		0.8675	1	0.57	1.03	0.26	0.16	0.17	0.09

tran	NA	NA	n382935	MTCONS_00073553	chr16	23457791	23458603	813	-	chr9	132396748	132404448	6463	-		-1046.6	-0.8981	-1	0.57	1.03	0.26	3.16	3	3.84
cis_mRNA_overlap	0	Lnc-CompleteIn-mRNAIntron	n387175	MTCONS_00042714	chr2	240115027	240117153	2127	-	chr2	239969864	240322671	8689	-	*		0.8186	1	0.16	0.09	0.07	0.67	0.61	0.41
cis_mRNA_overlap	0	Lnc-CompleteIn-mRNAIntron	n387175	MTCONS_00042715	chr2	240115027	240117153	2127	-	chr2	239969864	240322706	8438	-	*		0.9201	1	0.16	0.09	0.07	2.75	2.54	2.25
tran	NA	NA	n325077	MTCONS_00039982	chr20	5436521	5438648	358	-	chr2	238536213	238694823	8202	+		-306.77	0.9748	1	0.11	0.1	0	8.48	8.35	8.06
cis_mRNA_dw20k	1418	NA	n386493	MTCONS_00070921	chr9	35406752	35451103	2513	+	chr9	35161900	35405335	6519	+	*		0.9966	1	0	0.02	0.04	46.3	48.85	50.76
cis_mRNA_overlap	0	Lnc-CompleteIn-mRNAIntron	n370223	MTCONS_00067939	chr8	1398398	1398683	233	+	chr8	1010002	1656642	12652	+	*		0.9989	1	0	0.17	0.33	0.75	0.85	0.93
cis_mRNA_overlap	0	mRNA	n406445	MTCONS_00033325	chr19	1269267	1274809	3095	+	chr19	1269267	1280544	5142	+		-2404.43	0.9564	1	0.27	0.53	0.5	1.2	2.24	1.83
tran	NA	NA	LTCONS_00064399	MTCONS_00018627	chr7	19338948	19431991	374	+	chr12	123636661	123717823	8479	-		-217.6	-0.9913	-1	1.35	0.19	1.21	2.3	2.5	2.35
tran	NA	NA	LTCONS_00064399	MTCONS_00018626	chr7	19338948	19431991	374	+	chr12	123636661	123717699	8134	-		-217.6	-0.7508	-1	1.35	0.19	1.21	0.48	1.15	1
tran	NA	NA	n338547	MTCONS_00063987	chr1	227581292	227618736	1971	+	chr6_qbl_hap6	4472090	4476522	542	+		-309.95	0.8478	0.866	0.03	0	0.03	542	445.43	490.75
tran	NA	NA	n338547	MTCONS_00063842	chr1	227581292	227618736	1971	+	chr6_cox_hap2	4683664	4688093	542	+		-309.95	0.8478	0.866	0.03	0	0.03	542	445.43	490.75
tran	NA	NA	n383699	MTCONS_00037060	chr2	110744640	110752147	1725	+	chr19	59008116	59033259	10168	-		-2380.92	0.9841	1	0.6	1.32	0.67	0.68	0.79	0.71
cis_mRNA_overlap	0	Lnc-AntiOverlap-mRNA	n341937	MTCONS_00017879	chr12	58120054	58122131	1492	+	chr12	58118994	58132147	4978	-	*		0.9766	1	0.49	0.36	0.62	4.48	3.05	5.12
tran	9840511	NA	n385208	MTCONS_00065090	chr7	66757424	66767406	743	-	chr7	76607916	76676793	3269	+		-394.67	-0.8205	-1	3.97	2.88	3.66	0.87	1.46	1.36
tran	NA	NA	n371446	MTCONS_00033686	chrX	49155629	49157930	386	+	chr19	11998621	12032411	17275	+		-437.63	-0.8429	-1	3.1	2.55	2.95	1.66	3.92	3.42
tran	NA	NA	n371446	MTCONS_00033685	chrX	49155629	49157930	386	+	chr19	11998607	12032411	17331	+		-437.63	0.8295	1	3.1	2.55	2.95	4.11	2.39	2.73
tran	NA	NA	n371446	MTCONS_00036294	chrX	49155629	49157930	386	+	chr19	40528842	40562147	24857	-		-417.3	-0.9967	-1	3.1	2.55	2.95	6.91	8.47	7.45
tran	NA	NA	n406569	MTCONS_00072236	chr2	114341230	114356613	1958	+	chr9	14430	30786	3251	-		-1424.24	0.9481	1	12.65	7.35	14.61	0.08	0.04	0.13
tran	NA	NA	n378904	MTCONS_00046404	chr1	173834662	173836867	796	-	chr22	20008631	20055472	4110	+		-628.47	-1	-1	1.27	1.85	0.76	0.97	0.79	1.13
cis_mRNA_overlap	0	Lnc-AntiOverlap-mRNA	n386637	MTCONS_00008663	chr10	88998426	89102315	820	-	chr10	89101180	89131137	11860	+		-1054.96	0.9663	1	0.9	0.19	0.59	2.47	1.97	2.37
cis_mRNA_overlap	0	Lnc-AntiOverlap-mRNA	n386637	MTCONS_00008664	chr10	88998426	89102315	820	-	chr10	89101180	89131137	10665	+		-1054.96	-0.9537	-1	0.9	0.19	0.59	0	0.45	0.07
tran	NA	NA	n339280	MTCONS_00033686	chr3	32303234	32305396	2159	+	chr19	11998621	12032411	17275	+		-1174.08	0.7873	1	3.75	4.91	3.96	1.66	3.92	3.42
tran	NA	NA	n339280	MTCONS_00033685	chr3	32303234	32305396	2159	+	chr19	11998607	12032411	17331	+		-1174.08	-0.772	-1	3.75	4.91	3.96	4.11	2.39	2.73
tran	NA	NA	n339280	MTCONS_00036294	chr3	32303234	32305396	2159	+	chr19	40528842	40562147	24857	-		-1105.62	0.9844	1	3.75	4.91	3.96	6.91	8.47	7.45
cis_mRNA_dw20k	11392	NA	n373510	MTCONS_00018509	chr12	118555262	118573755	828	-	chr12	118585146	118628356	5769	-	*		-0.9979	-1	0.74	0.49	0.59	2.25	3.01	2.75
cis_mRNA_dw20k	994	NA	n410558	MTCONS_00066911	chr7	73097898	73112542	1426	+	chr7	73113535	73134020	2334	-	*		-0.9764	-1	0.66	0.17	0.31	8.76	9.93	9.37
cis_mRNA_overlap	0	Lnc-CompleteIn-mRNAExon	n339828	MTCONS_00032339	chr18	31329600	31331159	1560	+	chr18	31220863	31331159	12135	+		-690.6	0.8806	1	4.71	6.91	7.96	11.33	12.08	14.46
cis_mRNA_overlap	0	Lnc-CompleteIn-mRNAExon	n339828	MTCONS_00032340	chr18	31329600	31331159	1560	+	chr18	31220966	31331159	15102	+		-690.6	0.9699	1	4.71	6.91	7.96	1.41	1.47	1.54
cis_mRNA_dw20k	8050	NA	n340707	MTCONS_00029415	chr17	43921243	43972139	447	-	chr17	43861646	43913194	2415	+	*		0.9837	1	0.79	0.27	0	0.06	0.03	0
cis_mRNA_up10k	1481	NA	n375685	MTCONS_00028835	chr17	16936688	16944627	764	-	chr17	16946107	17088937	3851	+	*		-0.7244	-1	0.14	0.51	0.42	9.66	7.4	9.53
cis_mRNA_overlap	0	Lnc-CompleteIn-mRNAIntron	n340885	MTCONS_00029974	chr17	75975719	75978584	2865	+	chr17	75954761	76105096	10491	+	*		-0.8369	-1	0.32	0.34	0.35	1.05	0.95	0.36
cis_mRNA_overlap	0	Lnc-CompleteIn-mRNAIntron	n340958	MTCONS_00011315	chr11	8081585	8090920	2020	+	chr11	8040765	8127748	6694	+	*		0.9396	1	0.09	0.05	0	4.54	3.56	3.24
cis_mRNA_up10k	1640	NA	n341471	MTCONS_00069549	chr8	29209936	29210687	752	-	chr8	29190579	29208297	5533	-	*		0.9759	1	0.62	0.96	0.09	30.94	45.58	20.43
cis_mRNA_overlap	0	Lnc-AntiOverlap-mRNA	n378793	MTCONS_00037899	chr2	56402686	56412048	1623	-	chr2	56410912	56613309	4074	+	*		0.9852	1	0.04	0.02	0.19	3.05	1.82	6.14
cis_mRNA_overlap	0	Lnc-AntiOverlap-mRNA	n378793	MTCONS_00037900	chr2	56402686	56412048	1623	-	chr2	56410913	56613309	3996	+	*		0.6139	1	0.04	0.02	0.19	1.56	0.41	1.6
tran	NA	NA	n376307	MTCONS_00064836	chr19	35704060	35706780	872	+	chr7	55954858	56008496	3227	+		-568.76	-0.998	-1	0.3	0.22	0.42	6.55	6.76	6.15
tran	NA	NA	n376307	MTCONS_00038867	chr19	35704060	35706780	872	+	chr2	128848754	128953644	11141	+		-878.96	0.9934	1	0.3	0.22	0.42	0.94	0.93	0.95
cis_mRNA_up10k	144	NA	n369505	MTCONS_00065151	chr7	86954666	86974808	707	-	chr7	86974951	87023016	2596	+	*		0.9919	1	2.33	2.84	2.69	0.09	0.58	0.38
tran	NA	NA	n342217	MTCONS_00050042	chrX	52861124	52861635	512	-	chr3	186501977	186507686	1927	+		-238.85	0.8722	1	0.73	1.24	0.65	41.15	47.74	31.34
cis_mRNA_overlap	0	Lnc-CompleteIn-mRNAExon	n338615	MTCONS_00003750	chr1	246935607	246939075	3458	+	chr1	246887184	246946086	14034	+		-1699.44	0.9833	1	0.61	0.93	0.87	0.43	0.85	0.7
cis_mRNA_overlap	0	mRNA	n341790	MTCONS_00071878	chr9	131174045	131198079	4593	+	chr9	131181775	131199635	3117	+		-2614.7	-0.9691	-1	1.56	2.87	2.36	0.27	0.08	0.11
cis_mRNA_up10k	141	NA	n379469	MTCONS_00041079	chr2	73511662	73518113	5127	+	chr2	73480220	73511522	9122	-	*		0.9177	0.866	0.04	0.07	0.09	0.52	0.59	0.59
cis_mRNA_up10k	11	NA	n379469	MTCONS_00041080	chr2	73511662	73518113	5127	+	chr2	73480220	73511652	9021	-	*		0.803	0.866	0.04	0.07	0.09	0.14	0.14	0.17
cis_mRNA_up10k	27	NA	n376215	MTCONS_00034472	chr19	44326209	44331358	2066	-	chr19	44331384	44357322	6804	+	*		0.9942	0.866	0.09	0.26	0.11	2.32	2.71	2.32
cis_mRNA_overlap	0	mRNA	n377916	MTCONS_00004620	chr1	32826872	32829824	2759	-	chr1	32826555	32860062	6996	-		-1995.6	0.8826	1	0.62	1.05	0.87	1.43	2.37	1.56
cis_mRNA_dw20k	18997	NA	n383509	MTCONS_00034284	chr19	38053292	38054681	1390	-	chr19	37996748	38034296	8612	+	*		-0.6264	-1	0.8	1.18	0.83	0.74	0.63	0.64
tran	NA	NA	n335160	MTCONS_00075496	chr7	73645996	73646353	358	+	chrX	20168029	20284780	7871	-		-571.1	0.9625	1	1.78	3.46	2.81	1.93	3.94	3.68
tran	3039367	NA	LTCONS_00011021	MTCONS_00010029	chr10	48208864	48211994	3131	.	chr10	51251360	51371677	5208	-		-1419.94	-0.9769	-1	2.65	2.91	2.1	1.06	0.79	3.16
tran	3039367	NA	LTCONS_00011021	MTCONS_00010027	chr10	48208864	48211994	3131	.	chr10	51251360	51371331	3808	-		-1438.31	0.9395	1	2.65	2.91	2.1	0.9	1.64	0.46
cis_mRNA_overlap	0	mRNA	n410146	MTCONS_00036822	chr19	53300661	53324922	4467	-	chr19	53299146	53360982	6301	-		-1919.6	-0.9979	-1	2.1	2.56	2.43	0.79	0.52	0.58
tran	1533157	NA	n379335	MTCONS_00033967	chr19	19945686	20008579	4287	-	chr19	21541735	21571384	6244	+		-2929.25	-0.9886	-1	0.36	0.2	0.31	0.34	0.87	0.43
cis_mRNA_overlap	0	Lnc-AntiOverlap-mRNA	n409499	MTCONS_00059411	chr6	3020390	3025005	4616	-	chr6	2988201	3023034	1970	+	*		0.9994	0.866	0.05	0.12	0.05	0.77	1.79	0.73
tran	NA	NA	LTCONS_00067917	MTCONS_00037060	chr7	148787954	148789503	1550	.	chr19	59008116	59033259	10168	-		-1266.92	1	1	1.24	1.72	1.37	0.68	0.79	0.71
tran	NA	NA	LTCONS_00067917	MTCONS_00041121	chr7	148787954	148789503	1550	.	chr2	74699085	74710421	5074	-		-1621.86	-0.9909	-1	1.24	1.72	1.37	1.35	0.28	1.2
tran	NA	NA	LTCONS_00067917	MTCONS_00016577	chr7	148787954	148789503	1550	.	chr12	117176000	117291436	5847	+		-1291.62	0.9415	1	1.24	1.72	1.37	10.92	12.07	11.58
tran	NA	NA	LTCONS_00067917	MTCONS_00036605	chr7	148787954	148789503	1550	.	chr19	49251172	49257074	4531	-		-1222.96	0.9904	1	1.24	1.72	1.37	0.23	0.45	0.26
tran	674439	NA	n342353	MTCONS_00034018	chr19	23521418	23541782	975	-	chr19	24216220	24312694	4162	+		-346.51	-0.7862	-1	1.03	0.55	1.09	2.23	2.79	0.82

cis_mRNA_dw20k	140	NA	LTCONS_00063502	MTCONS_00063506	chr6	150314724	150384111	870	-	chr6	150384250	150390371	1164	-	*		0.8337	1	7.85	8.9	9.13	8.05	8.72	10.31
tran	NA	NA	LTCONS_00032002	MTCONS_00063987	chr17	14608571	14608893	323	.	chr6_qbl_hap6	4472090	4476522	542	+		-658.4	-0.9933	-1	1.73	3.36	2.76	542	445.43	490.75
tran	NA	NA	LTCONS_00032002	MTCONS_00063842	chr17	14608571	14608893	323	.	chr6_cox_hap2	4683664	4688093	542	+		-658.4	-0.9933	-1	1.73	3.36	2.76	542	445.43	490.75
tran	NA	NA	n326616	MTCONS_00067000	chr1	48339974	48400130	645	-	chr7	82993222	83218326	6098	-		-370.4	0.9675	1	0.07	0.22	0	0.27	3.96	0
cis_mRNA_dw20k	30	NA	n338425	MTCONS_00002943	chr1	185261516	185263504	1987	-	chr1	185126482	185261487	4541	+	*		0.7594	1	0.17	0.05	0	0.55	0.52	0
tran	NA	NA	n382143	MTCONS_00039982	chr14	24439148	24453530	2221	+	chr2	238536213	238694823	8202	+		-2170.59	-0.9662	-1	3.31	4.78	6.04	8.48	8.35	8.06
cis_mRNA_up10k	415	NA	n373299	MTCONS_00016505	chr12	112277578	112279771	1016	-	chr12	112280185	112331416	1636	+	*		0.6271	1	1.41	0.8	0.84	5.27	2.6	4.99
cis_mRNA_up10k	1661	NA	n338183	MTCONS_00005686	chr1	114304454	114307432	2975	-	chr1	114237411	114302794	6385	-	*		-0.9998	-1	2.41	2.17	0.36	2.17	2.29	3.04
cis_mRNA_dw20k	4188	NA	n342187	MTCONS_00061572	chr6	170886169	170888915	2747	+	chr6	170863421	170881982	1931	+	*		-0.9374	-1	0.19	0.14	0.15	1.79	6.2	3.91
tran	NA	NA	n369925	MTCONS_00041120	chr7	19039116	19040901	856	-	chr2	74699085	74710421	5373	-		-490.63	-0.9506	-1	0.36	0.32	0.25	2.19	2.58	2.8
tran	NA	NA	n369925	MTCONS_00050190	chr7	19039116	19040901	856	-	chr3	197518118	197612907	7657	+		-651.1	-0.9576	-1	0.36	0.32	0.25	4.44	4.46	4.68
tran	NA	NA	n345810	MTCONS_00075496	chr18	55297591	55309277	647	+	chrX	20168029	20284780	7871	-		-639.62	0.9998	1	0.45	0.92	0.85	1.93	3.94	3.68
cis_mRNA_dw20k	2377	NA	n406348	MTCONS_00064249	chr7	5111691	5112854	579	+	chr7	5085452	5109315	8484	+	*		0.8895	1	0	0.06	0.11	1.75	1.77	1.93
tran	NA	NA	n372985	MTCONS_00050190	chr12	19705712	19706887	1047	+	chr3	197518118	197612907	7657	+		-880.55	0.7293	1	0.15	0.32	0.37	4.44	4.46	4.68
cis_mRNA_overlap	0	mRNA	LTCONS_00074777	MTCONS_00074774	chrX	103173181	103227394	6816	+	chrX	103173048	103232995	6980	+		-2985.13	0.7241	1	2.19	2.58	2.04	7.98	8	6.73
cis_mRNA_overlap	0	Lnc-AntiOverlap-mRNA	n379534	MTCONS_00069866	chr8	70746346	70749605	828	+	chr8	70579284	70748090	10041	-	*		0.8847	1	0.74	0.16	0	7.39	7.33	7.22
tran	NA	NA	n374322	MTCONS_00040552	chr14	74939815	74941018	935	-	chr2	27873585	27886539	5240	-		-519.59	-0.9999	-0.866	0.04	0.03	0.03	2.04	3.05	3.07
cis_mRNA_overlap	0	Lnc-CompleteIn-mRNAExon	n385198	MTCONS_00064920	chr7	65216092	65228661	1674	+	chr7	65215978	65229253	2515	+		-797.24	-0.8943	-1	0.41	0.32	0.12	1.79	2.38	2.61
tran	NA	NA	LTCONS_00032069	MTCONS_00036996	chr17_gl000205_random	39005	40208	828	+	chr19	57973345	57988938	12739	-		-492.03	0.928	1	0.29	1.38	0.27	1.19	1.44	1.03
cis_mRNA_dw20k	5188	NA	n374534	MTCONS_00025367	chr15	89045175	89049018	3775	-	chr15	89054205	89089912	2879	-	*		-0.9957	-1	0.18	0.13	0.27	0.32	0.44	0
cis_mRNA_dw20k	5188	NA	n374534	MTCONS_00025366	chr15	89045175	89049018	3775	-	chr15	89054205	89089912	2949	-	*		0.9982	1	0.18	0.13	0.27	0.34	0	0.83
cis_mRNA_dw20k	15989	NA	n380287	MTCONS_00011493	chr11	22850958	22942196	627	+	chr11	22689654	22834970	2277	+	*		0.901	1	0	0.16	0.26	0.28	0.41	0.88
cis_mRNA_dw20k	15989	NA	n380287	MTCONS_00011491	chr11	22850958	22942196	627	+	chr11	22688160	22834970	2335	+	*		-0.8463	-1	0	0.16	0.26	0.58	0.52	0
tran	NA	NA	n378313	MTCONS_00032984	chr9	103115360	103156151	2333	+	chr18	45346666	45457071	23239	-		-3038.04	-0.9983	-1	1.75	1.61	1.78	2.93	3.26	2.83
tran	NA	NA	n408247	MTCONS_00066666	chr19	45430060	45434643	631	+	chr7	44605372	44621849	8184	-		-558.8	0.9845	1	0	0.16	0.1	5.65	8.18	7.64
tran	NA	NA	n326192	MTCONS_00039983	chr1	228795029	228797952	403	+	chr2	238536213	238694823	8109	+		-448.86	-0.9985	-1	2.6	1.14	1.41	1.72	3.49	3.26
tran	NA	NA	n326192	MTCONS_00071498	chr1	228795029	228797952	403	+	chr9	104161163	104181074	11258	+		-448.06	-0.9983	-1	2.6	1.14	1.41	4.99	6.86	6.41
cis_mRNA_overlap	0	mRNA	LTCONS_00042027	MTCONS_00042029	chr2	167260069	167350786	16710	-	chr2	167260069	167351002	9814	-		-4106.65	0.9873	1	0.3	0.43	1	0.44	0.46	1.55
tran	NA	NA	n325169	MTCONS_00050191	chr2	113986510	113989532	745	+	chr3	197518135	197612907	7748	+		-621.51	-0.878	-1	7.67	13.31	10.53	0.27	0	0.26
tran	NA	NA	n325169	MTCONS_00060684	chr2	113986510	113989532	745	+	chr6	87861490	87984049	24321	+		-623.82	0.9885	1	7.67	13.31	10.53	0.6	0.76	0.66
cis_mRNA_overlap	0	Lnc-CompleteIn-mRNAExon	n340517	MTCONS_00030540	chr17	15332273	15333318	1046	-	chr17	15331553	15466945	10874	-		-810.81	0.758	1	0.65	0.78	0.76	0.45	1.09	0.58
cis_mRNA_up10k	42	NA	n346431	MTCONS_00034248	chr19	37176623	37178315	562	-	chr19	37178356	37212226	2948	+	*		0.7343	1	2.03	2.09	1	1.62	2.25	1.4
cis_mRNA_dw20k	12041	NA	n375682	MTCONS_00030539	chr17	15317202	15319513	338	-	chr17	15331553	15466945	10817	-	*		-0.9958	-1	0.47	0.43	0.31	2.48	2.53	2.79
tran	192197	NA	n405875	MTCONS_00064893	chr7	64363620	64391955	4414	+	chr7	64126511	64171424	5099	+		-1366.71	-0.9444	-1	0.88	0.7	1.18	1.54	1.57	0.98
tran	NA	NA	n405875	MTCONS_00034018	chr7	64363620	64391955	4414	+	chr19	24216220	24312694	4162	+		-1268.53	-0.9949	-1	0.88	0.7	1.18	2.23	2.79	0.82
cis_mRNA_dw20k	5724	NA	n372955	MTCONS_00015664	chr12	49672836	49676768	2535	-	chr12	49621747	49667113	1771	+	*		-0.9701	-1	0.23	0.34	0.29	1.48	0	0.99
cis_mRNA_dw20k	5724	NA	n372955	MTCONS_00015663	chr12	49672836	49676768	2535	-	chr12	49621740	49667113	1660	+	*		-0.9908	-1	0.23	0.34	0.29	6.66	1.89	4.62
cis_mRNA_dw20k	9064	NA	n338899	MTCONS_00036656	chr19	50341902	50354933	2149	-	chr19	50363996	50370844	3874	-	*		0.9892	0.866	0.13	0.13	0.27	1.31	1.43	2.07
cis_mRNA_dw20k	11641	NA	n410156	MTCONS_00048766	chr3	50126352	50156392	3041	+	chr3	49977474	50114712	2490	+	*		-0.9397	-1	4.7	4.39	3.2	3.28	3.68	4.05
cis_mRNA_overlap	0	mRNA	n345277	MTCONS_00022707	chr14	81881291	81893558	938	-	chr14	81726254	81902826	11672	-	*		0.9803	1	0.08	0.24	0.1	3.27	3.84	3.45
tran	1908095	NA	n345955	MTCONS_00072888	chr9	88444454	88464907	1497	+	chr9	86451613	86536360	4778	-		-641.98	-0.9802	-1	2.18	1.44	1.17	1	1.81	1.87
cis_mRNA_up10k	153	NA	n341984	MTCONS_00016273	chr12	93936240	93965544	1655	-	chr12	93965696	93970074	2385	+	*		-0.8048	-1	2.74	2.28	2.57	1.41	1.88	1.86
cis_mRNA_up10k	83	NA	n341984	MTCONS_00016272	chr12	93936240	93965544	1655	-	chr12	93965626	93970074	1994	+	*		0.9745	1	2.74	2.28	2.57	11.18	8.02	10.68
tran	NA	NA	n368027	MTCONS_00031083	chr5	10521850	10527001	799	+	chr17	40190058	40196297	4596	-		-783.2	0.6672	1	1.15	1.43	1.09	2.26	2.3	1.43
cis_mRNA_overlap	0	mRNA	n383353	MTCONS_00032676	chr18	77806900	77839191	1346	+	chr18	77794346	77854968	4564	+		-820.98	0.8845	1	0.2	0.15	0.34	0.39	0.3	0.43
cis_mRNA_up10k	135	NA	LTCONS_00062791	MTCONS_00060528	chr6	69339825	69344953	3895	-	chr6	69345087	70099476	6083	+	*		0.9702	1	1.75	1.55	1.9	7.26	6.49	8.64
tran	NA	NA	LTCONS_00047500	MTCONS_00033686	chr22	24005496	24008641	493	-	chr19	11998621	12032411	17275	+		-561.71	-0.9848	-1	3.28	1.94	2.45	1.66	3.92	3.42
tran	NA	NA	LTCONS_00047500	MTCONS_00033685	chr22	24005496	24008641	493	-	chr19	11998607	12032411	17331	+		-561.71	0.9803	1	3.28	1.94	2.45	4.11	2.39	2.73
tran	NA	NA	LTCONS_00047500	MTCONS_00036294	chr22	24005496	24008641	493	-	chr19	40528842	40562147	24857	-		-533.71	-0.9515	-1	3.28	1.94	2.45	6.91	8.47	7.45
cis_mRNA_up10k	9933	NA	n385806	MTCONS_00076097	chrX	103367013	103369317	2305	-	chrX	103343898	103357081	6149	-	*		-0.9896	-1	0.77	1.17	1.35	1.74	1.36	1.28
cis_mRNA_overlap	0	mRNA	n337402	MTCONS_00071930	chr9	131710297	131719290	881	+	chr9	131711049	131770312	7540	+		-561.88	0.7704	1	0.4	0.51	0.11	1.22	1.41	1.2
cis_mRNA_up10k	324	NA	n370935	MTCONS_00072720	chr9	67340517	67343501	394	+	chr9	67323534	67340194	5721	-	*		-0.9556	-0.866	0.98	0.98	0.96	3.46	2.62	5.4
cis_mRNA_up10k	245	NA	n378604	MTCONS_00063088	chr6	111804868	111824814	915	+	chr6	111619894	111804624	11379	-	*		0.8581	1	0.46	0.47	0.24	1.19	1.54	0.9
cis_mRNA_overlap	0	Lnc-CompleteIn-mRNAIntron	n337882	MTCONS_00024981	chr15	66319582	66321724	2142	-	chr15	66184371	66546141	10164	-	*		-0.8994	-1	0.06	0.03	0.04	0.6	1.46	0.83
cis_mRNA_overlap	0	Lnc-CompleteIn-mRNAIntron	n337882	MTCONS_00024980	chr15	66319582	66321724	2142	-	chr15	66184371	66546119	9509	-	*		0.7559	0.866	0.06	0.03	0.04	0.07	0.04	0.07
cis_mRNA_overlap	0	Lnc-AntiOverlap-mRNA	n405511	MTCONS_00026227	chr16	29476289	29478899	1478	-	chr16	29465331	29476301	6115	+	*		0.9999	1	0.05	0.21	0.29	1.34	3.07	3.98
cis_mRNA_overlap	0	Lnc-AntiOverlap-mRNA	n405511	MTCONS_00026228	chr16	29476289	29478899	1478	-	chr16	29465331	29476301	4668	+	*		0.9865	1	0.05	0.21	0.29	2.96	5.48	6.03
cis_mRNA_dw20k	15391	NA	n385202	MTCONS_00064940	chr7	65841032	65865395	4423	-	chr7	65670259	65825642	3351	+	*		0.8566	1	0.23	0.24	0.26	2.53	3.7	3.96
tran	NA	NA	LTCONS_00064611	MTCONS_00017914	chr7	32766662	32802650	11793	+	chr12	64052387	64062354	5782	-		-5093.07	0.7036	1	0.87	1.39	0.94	1.34	2.18	2.06
tran	NA	NA	LTCONS_00012573	MTCONS_00026228	chr11	93921564	93922676	1113	+	chr16	29465331	29476301	4668	+		-956.53	0.7792	1	0.54	0.62	0.92	2.96	5.48	6.03
cis_mRNA_dw20k	19247	NA	n406668	MTCONS_00069195	chr8	145162629	145173218	7119	+	chr8	145133434	145143383	7286	+	*		0.9035	1	0.5	0.63	0.54	0.72	1.15	1.02
cis_mRNA_up10k	3330	NA	n406668	MTCONS_00070538	chr8	145162629	145173218	7119	+	chr8	145153521	145159300	2318	-	*		-0.9907	-1	0.5	0.63	0.54	1.48	0.92	1.24

cis_mRNA_overlap	0	Lnc-CompleteIn-mRNAExon	n339259	MTCONS_00048345	chr3	15087058	15090778	3721	+	chr3	14987670	15090876	10165	+		-1841.7	-0.9475	-1	1.01	1.35	0.46	0.35	0.25	0.4
cis_mRNA_overlap	0	Lnc-CompleteIn-mRNAExon	n339259	MTCONS_00048346	chr3	15087058	15090778	3721	+	chr3	14988983	15090876	8622	+		-1773.4	-0.8468	-1	1.01	1.35	0.46	3.58	2.75	3.68
cis_mRNA_overlap	0	Lnc-AntiOverlap-mRNA	LTCONS_00034255	MTCONS_00036177	chr19	37288420	37320621	2460	+	chr19	37305033	37341703	6388	-	*		-0.978	-1	2.73	1.72	1.9	0	1.26	0.79
cis_mRNA_overlap	0	mRNA	LTCONS_00073078	MTCONS_00073080	chr9	99649607	99776566	12300	-	chr9	99695812	99776004	2620	-		-1871.33	-0.9989	-1	3.48	3.89	3.99	2.1	1.41	1.19
tran	NA	NA	n346227	MTCONS_00034284	chr7	45822293	45828413	548	+	chr19	37996748	38034296	8612	+		-594.56	-0.9973	-1	0	0.18	0.15	0.74	0.63	0.64
tran	NA	NA	n385995	MTCONS_00023576	chr10	88965591	89102315	1328	-	chr15	42841004	42863691	5541	+		-1326.55	-0.9951	-1	3.63	4.38	4.62	2.08	2	1.96
cis_mRNA_dw20k	14209	NA	n385995	MTCONS_00008657	chr10	88965591	89102315	1328	-	chr10	88854460	88951383	3805	+	*		0.9993	1	3.63	4.38	4.62	0.58	2.72	3.28
cis_mRNA_overlap	0	mRNA	LTCONS_00034016	MTCONS_00034018	chr19	24216220	24224139	4796	+	chr19	24216220	24312694	4162	+	*		-0.9994	-1	3.74	3.52	4.39	2.23	2.79	0.82
cis_mRNA_up10k	6690	NA	n345324	MTCONS_00028316	chr16	87807094	87848932	2721	-	chr16	87731658	87800405	7775	-	*		0.9245	1	0.03	0.02	0.01	0.79	0.73	0.72
tran	NA	NA	n335787	MTCONS_00022667	chr6	119312344	119312956	613	-	chr14	77249021	77279283	7871	-		-617.1	-0.9265	-1	0.18	0.05	0.27	1.82	2.33	1.8
cis_mRNA_dw20k	3264	NA	n338142	MTCONS_00005657	chr1	112313292	112315191	1401	-	chr1	112318454	112532197	2356	-	*		0.9983	1	3.03	5.61	2.79	2.05	3.93	2.01
cis_mRNA_up10k	535	NA	n375398	MTCONS_00027072	chr16	89883185	89894373	2063	+	chr16	89894907	89939169	7585	+	*		-0.8705	-1	0.49	0.07	0.37	2.73	3.62	3.39
cis_mRNA_dw20k	3619	NA	n326011	MTCONS_00009833	chr10	38104305	38113065	712	-	chr10	38116683	38147369	7634	-	*		0.9696	1	0.2	0	0.23	0.42	0	0.66
cis_mRNA_dw20k	3619	NA	n326011	MTCONS_00009832	chr10	38104305	38113065	712	-	chr10	38116683	38147369	6884	-	*		0.9078	1	0.2	0	0.23	0.65	0	1.37
cis_mRNA_overlap	0	Lnc-CompleteIn-mRNAIntron	n342168	MTCONS_00061462	chr6	163894452	163895192	741	+	chr6	163835604	164000803	10685	+	*		0.9562	1	0.39	0.97	0.74	4	5.25	4.43
cis_mRNA_overlap	0	Lnc-AntiOverlap-mRNA	n342767	MTCONS_00014025	chr11	64418767	64423783	907	+	chr11	64373626	64490777	6715	-		-1255.54	-0.9906	-0.866	0.04	0.04	0	16.48	17.27	21.82
cis_mRNA_overlap	0	Lnc-AntiOverlap-mRNA	n342767	MTCONS_00014024	chr11	64418767	64423783	907	+	chr11	64373626	64490660	6631	-		-1254.59	-0.9918	-0.866	0.04	0.04	0	15.39	15.82	18.49
cis_mRNA_overlap	0	mRNA	n341661	MTCONS_00072405	chr9	21324051	21335352	732	-	chr9	21328775	21335429	5277	-		-424.58	0.9974	1	0.45	0.57	0.67	0.5	0.78	0.96
tran	NA	NA	n368295	MTCONS_00076002	chr5	67482644	67485177	924	-	chrX	99880885	99891854	5056	-		-440.84	0.9533	1	0.43	0.55	0.3	2.12	2.85	1.88
tran	NA	NA	n376745	MTCONS_00027230	chr20	48788959	48790099	688	+	chr16	2227098	2246516	8560	-		-649.98	0.9999	1	0	0.61	0.19	1.84	2.67	2.09
cis_mRNA_dw20k	1728	NA	LTCONS_00076589	MTCONS_00074282	chrX	48469309	48469700	392	.	chrX	48455874	48467582	5788	+	*		0.9839	1	1.94	2.13	2.18	6.19	7.54	7.57
cis_mRNA_dw20k	789	NA	n410030	MTCONS_00017028	chr12	6648694	6665249	2798	-	chr12	6666037	6716670	11091	-	*		0.8923	1	0	2.08	1.72	2.81	3.72	3.18
cis_mRNA_overlap	0	Lnc-AntiOverlap-mRNA	n339103	MTCONS_00039537	chr2	204102184	204104694	2511	-	chr2	204103065	204171345	11657	+		-2407.64	-0.926	-1	2.64	1.88	2.21	0.61	1.2	1.16
tran	NA	NA	n363424	MTCONS_00018627	chr1	67995313	67998012	408	+	chr12	123636661	123717823	8479	-		-250.35	-0.866	-1	0.28	0	0.08	2.3	2.5	2.35
tran	NA	NA	n363424	MTCONS_00018626	chr1	67995313	67998012	408	+	chr12	123636661	123717699	8134	-		-250.35	-0.9978	-1	0.28	0	0.08	0.48	1.15	1
tran	NA	NA	n407251	MTCONS_00063981	chr6	31938952	31949223	1805	+	chr6_qbl_hap6	3241000	3242626	423	+		-350.6	-0.7386	-1	4.06	4.56	3.85	0.18	0.12	3.54
cis_mRNA_overlap	0	Lnc-AntiOverlap-mRNA	n342055	MTCONS_00018635	chr12	123848415	123850334	1920	+	chr12	123773656	123849343	11074	-	*		-0.9999	-1	0.36	0.25	0.54	8.24	8.78	7.39
cis_mRNA_overlap	0	Lnc-AntiOverlap-mRNA	n342055	MTCONS_00018636	chr12	123848415	123850334	1920	+	chr12	123773656	123849343	11077	-	*		0.9972	1	0.36	0.25	0.54	3.41	2.36	4.72
tran	NA	NA	n342456	MTCONS_00065329	chr16	2710350	2723375	1449	-	chr7	99775538	99948836	16269	+		-1604.35	0.9419	1	2.52	3.84	4.68	1.41	1.88	1.91
cis_mRNA_dw20k	73	NA	n341648	MTCONS_00072270	chr9	3218302	3223008	4705	-	chr9	3223080	3525995	4469	-	*		0.8343	1	4.37	4.59	3.59	3.02	3.42	2.88
tran	NA	NA	n374137	MTCONS_00072661	chr14	41423822	41432050	392	+	chr9	40770255	40789853	5594	-		-242.63	-0.9809	-1	5.68	3.65	5.52	2.05	2.71	2.23
tran	NA	NA	n374137	MTCONS_00036294	chr14	41423822	41432050	392	+	chr19	40528842	40562147	24857	-		-652.24	-0.9619	-1	5.68	3.65	5.52	6.91	8.47	7.45
cis_mRNA_dw20k	12648	NA	n341933	MTCONS_00017854	chr12	57628694	57634527	2010	-	chr12	57647174	57824682	4544	-	*		0.7793	0.866	0.63	0.78	0.37	2.5	3.35	2.5
tran	NA	NA	n385030	MTCONS_00033686	chr6	144175206	144177090	1885	+	chr19	11998621	12032411	17275	+		-2837.4	0.76	1	4.87	5.16	4.91	1.66	3.92	3.42
tran	NA	NA	n385030	MTCONS_00033685	chr6	144175206	144177090	1885	+	chr19	11998607	12032411	17331	+		-2837.4	-0.7439	-1	4.87	5.16	4.91	4.11	2.39	2.73
tran	NA	NA	n411585	MTCONS_00061304	chr15	67807260	67814182	793	-	chr6	149887450	149912879	1999	+		-562.43	-0.9178	-1	0.1	0	0.25	6.15	7.14	5.84
tran	599548	NA	n408016	MTCONS_00066942	chr7	74306894	74322330	1323	+	chr7	74921877	74988366	1808	-		-685.19	0.9414	1	0.75	0.88	0.71	5.02	5.8	4.1
cis_mRNA_dw20k	5170	NA	n406623	MTCONS_00014238	chr11	71639768	71708643	2706	+	chr11	71713812	71753181	8276	-	*		-0.9653	-0.866	0.45	0.18	0.55	5.02	6.32	5.02
cis_mRNA_overlap	0	mRNA	n406623	MTCONS_00012320	chr11	71639768	71708643	2706	+	chr11	71639547	71714063	6955	+		-1914.41	-0.999	-1	0.45	0.18	0.55	1.41	1.83	1.22
cis_mRNA_dw20k	5170	NA	n406623	MTCONS_00014239	chr11	71639768	71708643	2706	+	chr11	71713812	71791663	7467	-	*		0.8824	1	0.45	0.18	0.55	18.38	17.19	21.2
tran	NA	NA	n368305	MTCONS_00033686	chr5	72236631	72251181	588	-	chr19	11998621	12032411	17275	+		-480.84	-0.8043	-1	0.52	0.28	0.47	1.66	3.92	3.42
tran	NA	NA	n368305	MTCONS_00033685	chr5	72236631	72251181	588	-	chr19	11998607	12032411	17331	+		-480.84	0.7895	1	0.52	0.28	0.47	4.11	2.39	2.73
tran	NA	NA	n342874	MTCONS_00047712	chr9	102902688	102904291	1604	+	chr22	32896532	33454377	15152	-		-932.62	-0.8411	-1	0.93	1.08	1.16	5.15	5.06	4.49
cis_mRNA_overlap	0	Lnc-CompleteIn-mRNAExon	n334398	MTCONS_00029672	chr17	59479134	59480524	281	+	chr17	59474205	59486827	6316	+		-225.88	0.7835	1	0.17	0	0.16	1.75	0.55	0.94
cis_mRNA_up10k	59	NA	n408075	MTCONS_00029598	chr17	53029259	53046064	1128	-	chr17	53046122	53241777	6384	+	*		-0.9903	-1	1.19	4.1	2.26	1.38	0.34	1.13
tran	NA	NA	LTCONS_00063776	MTCONS_00050191	chr6	27685776	27686189	414	.	chr3	197518135	197612907	7748	+		-376.19	0.9454	1	2.07	1.71	1.94	0.27	0	0.26
tran	NA	NA	LTCONS_00063776	MTCONS_00041121	chr6	27685776	27686189	414	.	chr2	74699085	74710421	5074	-		-247.96	0.9726	1	2.07	1.71	1.94	1.35	0.28	1.2
cis_mRNA_dw20k	7208	NA	n346355	MTCONS_00066638	chr7	44054297	44058757	274	-	chr7	44065964	44080295	4331	-	*		-0.8212	-1	0.07	0	2.06	2.81	3.87	2.1
cis_mRNA_overlap	0	Lnc-CompleteIn-mRNAIntron	n337957	MTCONS_00047374	chr22	19973144	19973425	282	-	chr22	19957343	19974747	4429	-	*		0.9963	1	1.31	2.51	6.1	11.05	11.26	12.3
cis_mRNA_overlap	0	Lnc-CompleteIn-mRNAIntron	n341752	MTCONS_00073226	chr9	111843400	111845685	2286	-	chr9	111777415	111882241	7989	-	*		-0.9938	-1	1.08	0.82	0.96	2.37	2.81	2.53
cis_mRNA_overlap	0	Lnc-CompleteIn-mRNAIntron	n324803	MTCONS_00045298	chr21	35039924	35079053	2632	+	chr21	35014784	35210810	5434	+	*		-0.8998	-1	0.2	0.29	0.24	8.72	6.57	8.7
cis_mRNA_overlap	0	Lnc-CompleteIn-mRNAIntron	n341690	MTCONS_00072616	chr9	37564532	37567353	2817	-	chr9	37510386	37576360	5006	-	*		0.6547	0.866	0.36	0.41	0.37	1.02	1.3	1.3
cis_mRNA_dw20k	11403	NA	LTCONS_00014953	MTCONS_00014297	chr11	75125689	75126844	1156	.	chr11	75138246	75144242	3585	-	*		-0.9862	-1	1.52	1.75	1.3	3.08	1.51	3.9
cis_mRNA_dw20k	6595	NA	LTCONS_00014953	MTCONS_00014296	chr11	75125689	75126844	1156	.	chr11	75133438	75144250	2102	-	*		-0.9071	-1	1.52	1.75	1.3	8.16	0.9	8.83
tran	NA	NA	n405300	MTCONS_00063841	chr6	33080293	33096890	805	+	chr6_cox_hap2	4488020	4499840	2170	+		-535.07	0.6496	1	0.89	1.9	0.79	2.32	2.6	0.09
tran	NA	NA	n369813	MTCONS_00003750	chr7	89123841	89126206	466	+	chr1	246887184	246946086	14034	+		-340.43	-0.9958	-1	0.16	0	0.07	0.43	0.85	0.7

cis_mRNA_overlap	0	AntiCompleteIn-mRNAIntron	n341621	MTCONS_00070424	chr8	140943416	140946180	2759	+	chr8	140740088	141467861	6770	-	*		0.866	0.866	0.01	0.01	0	1.17	1.25	1.09
cis_mRNA_dw20k	6291	NA	LTCONS_00070629	MTCONS_00069892	chr8	74193058	74196550	3493	.	chr8	74202840	74205907	909	-	*		-0.8847	-0.866	0.01	0.02	0.02	564.66	540.71	519.77
cis_mRNA_overlap	0	mRNA-AntiCompleteIn-LncIntron	n379409	MTCONS_00035733	chr19	15050246	16748905	1493	+	chr19	15355596	15443247	6845	-	*		0.9827	1	0.05	0.08	0.06	0.68	0.87	0.71
cis_mRNA_overlap	0	mRNA-AntiCompleteIn-LncIntron	n379409	MTCONS_00035727	chr19	15050246	16748905	1493	+	chr19	15225699	15236589	2672	-	*		-0.7963	-1	0.05	0.08	0.06	1.23	1.09	1.1
cis_mRNA_overlap	0	mRNA-AntiCompleteIn-LncIntron	n379409	MTCONS_00035735	chr19	15050246	16748905	1493	+	chr19	15362357	15442705	4857	-	*		0.8457	1	0.05	0.08	0.06	0.67	1.29	1.19
tran	NA	NA	n381013	MTCONS_00072236	chr1	16858	19759	1577	-	chr9	14430	30786	3251	-		-1191.58	0.9434	1	1.88	1.34	5.44	0.08	0.04	0.13
cis_mRNA_up10k	9229	NA	n323808	MTCONS_00076339	chrX	134058543	134060320	867	-	chrX	134021662	134049315	2214	-	*		0.9769	1	0.33	0.52	0.07	1.3	1.94	0.89
cis_mRNA_overlap	0	Lnc-AntiOverlap-mRNA	n375747	MTCONS_00029671	chr17	59470827	59476780	463	-	chr17	59474205	59486827	6081	+		-704.24	-0.979	-1	3.21	3.13	2.29	1.54	1.84	2.58
cis_mRNA_overlap	0	Lnc-AntiOverlap-mRNA	n341085	MTCONS_00012486	chr11	82896841	82898078	1238	-	chr11	82868137	82896881	10046	+	*		0.9907	1	0.73	1	0.78	3.83	5.65	4.4
tran	NA	NA	n367813	MTCONS_00026228	chr5	127999671	128078522	462	+	chr16	29465331	29476301	4668	+		-373	-0.7225	-1	0.08	0.07	0	2.96	5.48	6.03
cis_mRNA_up10k	979	NA	n342019	MTCONS_00018380	chr12	110435224	110437401	1709	-	chr12	110385867	110434246	2400	-	*		0.9984	1	0.38	0.4	0.52	1.14	1.17	1.5
cis_mRNA_overlap	0	Lnc-AntiOverlap-mRNA	n373506	MTCONS_00016506	chr12	112331318	112333002	478	-	chr12	112280185	112340177	3016	+	*		-0.9715	-1	0.16	0.07	0	3.54	4.42	4.67
cis_mRNA_up10k	590	NA	n368957	MTCONS_00059455	chr6	3255350	3258407	965	+	chr6	3258996	3304632	2052	+	*		0.8799	1	0.48	0.7	0.29	1.45	1.49	0.98
tran	NA	NA	n325818	MTCONS_00041121	chr11	130628527	130675876	584	+	chr2	74699085	74710421	5074	-		-413.28	-0.8343	-1	0.11	0.2	0.17	1.35	0.28	1.2
cis_mRNA_overlap	0	Lnc-CompleteIn-mRNAIntron	n337941	MTCONS_00005027	chr1	53771125	53774157	3033	-	chr1	53708466	53793821	6966	-	*		0.9796	1	0.33	0.53	0.47	6.87	9.69	8.34
tran	NA	NA	n373586	MTCONS_00017040	chr13	24072935	24077013	552	+	chr12	6774462	6798083	4725	-		-665.06	-0.8956	-1	0	0.24	0.13	0.6	0	0.01
cis_mRNA_dw20k	12101	NA	n333016	MTCONS_00022901	chr14	100744716	100745353	638	+	chr14	100757453	100772860	7834	-	*		0.9358	1	8.51	9.62	10.6	8.45	9.34	12.71
cis_mRNA_overlap	0	mRNA	n407166	MTCONS_00065151	chr7	86974951	86989425	1264	+	chr7	86974951	87023016	2596	+		-526.06	-0.9999	-1	1.34	0.63	0.93	0.09	0.58	0.38
tran	NA	NA	n326676	MTCONS_00034630	chr1	22224614	22230303	1054	+	chr19	47852627	47887580	9026	+		-927.4	0.9329	1	0.08	0.29	0.11	0.79	1.81	1.28
tran	NA	NA	n326335	MTCONS_00033686	chr1	168507368	168514697	1457	-	chr19	11998621	12032411	17275	+		-998.68	0.9023	1	0.92	1.39	1.1	1.66	3.92	3.42
tran	NA	NA	n326335	MTCONS_00039983	chr1	168507368	168514697	1457	-	chr2	238536213	238694823	8109	+		-659.29	0.8587	1	0.92	1.39	1.1	1.72	3.49	3.26
tran	NA	NA	n326335	MTCONS_00033685	chr1	168507368	168514697	1457	-	chr19	11998607	12032411	17331	+		-998.68	-0.8915	-1	0.92	1.39	1.1	4.11	2.39	2.73
cis_mRNA_dw20k	8522	NA	LTCONS_00033969	MTCONS_00033967	chr19	21579905	21593286	10335	+	chr19	21541735	21571384	6244	+	*		-0.8095	-1	2.48	1.58	1.82	0.34	0.87	0.43
cis_mRNA_up10k	5602	NA	n380372	MTCONS_00070507	chr8	144821566	144824305	582	+	chr8	144806032	144815965	8804	-	*		0.9486	1	0.03	0	0.21	2.94	2.83	3.08
tran	1507467	NA	LTCONS_00005931	MTCONS_00005904	chr1	147574207	147578821	3331	-	chr1	146032151	146066741	4836	-		-1652.9	0.9462	1	3.48	5.27	4.36	0.67	1.03	0.74
tran	NA	NA	n342845	MTCONS_00036294	chr8	73973659	73975440	1782	-	chr19	40528842	40562147	24857	-		-1362.32	0.9915	1	1.63	2.65	2.1	6.91	8.47	7.45
tran	NA	NA	n371452	MTCONS_00026035	chrX	57667298	57680873	380	+	chr16	19079136	19096290	8456	+		-231.68	-0.9984	-1	0.1	0.09	0	2.57	2.63	4.11
cis_mRNA_dw20k	4167	NA	n382508	MTCONS_00023576	chr15	42867857	42965295	6032	+	chr15	42841004	42863691	5541	+	*		-0.9789	-1	0.1	0.28	0.47	2.08	2	1.96
cis_mRNA_overlap	0	Lnc-CompleteIn-mRNAIntron	n342690	MTCONS_00029070	chr17	30366993	30367713	721	+	chr17	30334858	30380654	1766	+	*		-0.9177	-0.866	0.25	0.19	0.29	0.49	0.95	0.49
cis_mRNA_overlap	0	Lnc-CompleteIn-mRNAIntron	n342690	MTCONS_00029069	chr17	30366993	30367713	721	+	chr17	30334413	30380654	3751	+	*		0.9707	1	0.25	0.19	0.29	1.86	1.79	1.97
cis_mRNA_up10k	592	NA	n340122	MTCONS_00010593	chr10	101380812	101381673	862	+	chr10	101370220	101380221	2786	-	*		-0.8518	-1	0.29	0.19	0.15	1.1	1.27	1.86
cis_mRNA_overlap	0	mRNA	n408339	MTCONS_00008663	chr10	89102168	89103331	1164	+	chr10	89101180	89131137	11860	+		-983.91	0.9888	1	8.69	6.5	8.59	2.47	1.97	2.37
cis_mRNA_overlap	0	mRNA	n408339	MTCONS_00008664	chr10	89102168	89103331	1164	+	chr10	89101180	89131137	10665	+		-983.91	-0.9945	-1	8.69	6.5	8.59	0	0.45	0.07
cis_mRNA_dw20k	12818	NA	n375641	MTCONS_00029925	chr17	74788575	74799876	591	+	chr17	74733070	74775758	2844	+	*		0.9374	1	0.25	0.06	0	3.4	2.52	1.42
tran	NA	NA	n381595	MTCONS_00066703	chr11	3402191	3419300	1241	+	chr7	47994874	48019266	10405	-		-2076.96	-0.998	-1	3.45	1.33	0.86	7.11	8.45	8.88
cis_mRNA_overlap	0	mRNA	n345032	MTCONS_00070442	chr8	142278107	142318404	3339	-	chr8	142220870	142318462	3889	-	*		0.8805	1	0	0.06	0.02	2.09	2.44	2.36
tran	NA	NA	n345815	MTCONS_00072360	chr8	144714038	144715688	666	-	chr9	15167067	15250224	7033	-		-684.02	0.9978	1	0.27	0.45	0.34	2.46	3.34	2.75
tran	NA	NA	n345815	MTCONS_00070766	chr8	144714038	144715688	666	-	chr9	15422782	15466745	6896	+		-517.46	0.9527	1	0.27	0.45	0.34	13.4	15.38	13.59
tran	NA	NA	n336822	MTCONS_00067430	chr8	54870538	54870827	290	+	chr7	121713598	121784344	8375	-		-368.1	0.9786	1	0.19	0.23	0.68	1.12	1.14	1.19
cis_mRNA_overlap	0	Lnc-CompleteIn-mRNAIntron	n336822	MTCONS_00068491	chr8	54870538	54870827	290	+	chr8	54792567	54871863	1534	+	*		-0.6228	-1	0.19	0.23	0.68	1.2	0.1	0
tran	NA	NA	n385922	MTCONS_00072236	chr1	17233	29370	806	-	chr9	14430	30786	3251	-		-674.89	-0.87	-1	1.47	1.7	1.45	0.08	0.04	0.13
tran	NA	NA	n325693	MTCONS_00039982	chr13	74024981	74028022	825	+	chr2	238536213	238694823	8202	+		-424.36	-0.993	-1	7.01	7.67	8.6	8.48	8.35	8.06
tran	NA	NA	n365929	MTCONS_00070791	chr3	27862979	27932792	543	+	chr9	20658090	21002254	12800	+		-301.13	0.9806	1	1.81	1.29	2.59	2.44	2.4	2.47
cis_mRNA_dw20k	9824	NA	n370915	MTCONS_00072451	chr9	27529917	27536721	1421	+	chr9	27546544	27573867	3341	-	*		0.9629	1	0.05	0.07	0.09	0.47	1.97	2.49
cis_mRNA_overlap	0	mRNA	n326666	MTCONS_00004515	chr1	27859426	27861820	441	-	chr1	27860756	27930587	6376	-	*		-0.9929	-1	0.09	0	0.08	1.21	1.69	1.32
tran	7514831	NA	n379281	MTCONS_00008663	chr10	81564241	81586350	829	-	chr10	89101180	89131137	11860	+		-1186.31	-0.9988	-1	0.26	1.19	0.49	2.47	1.97	2.37
tran	7514831	NA	n379281	MTCONS_00008664	chr10	81564241	81586350	829	-	chr10	89101180	89131137	10665	+		-1186.31	0.9955	1	0.26	1.19	0.49	0	0.45	0.07
tran	NA	NA	n339477	MTCONS_00026493	chr3	123329581	123330850	1270	-	chr16	53468351	53526530	7443	+		-973.34	0.6314	0.866	0.03	0.05	0.05	1.19	1.43	2.6
tran	NA	NA	n339477	MTCONS_00026494	chr3	123329581	123330850	1270	-	chr16	53468351	53526530	7508	+		-973.34	0.866	0.866	0.03	0.05	0.05	0.36	1.1	0.73
tran	NA	NA	LTCONS_00028411	MTCONS_00015840	chr16	55753698	55754347	650	.	chr12	54674445	54681404	3966	+		-1192.3	0.6924	1	0	0.09	0.08	6.1	8.43	6.45
tran	33173747	NA	n364897	MTCONS_00037945	chr2	28945306	28958975	723	-	chr2	62132721	62373442	11032	+		-501.41	0.9734	1	0	0.16	0.04	2.46	3.22	2.47
tran	NA	NA	n364897	MTCONS_00030322	chr2	28945306	28958975	723	-	chr17	5079728	5095194	8312	-		-525.5	0.9998	1	0	0.16	0.04	0.65	0.8	0.69
cis_mRNA_dw20k	14727	NA	LTCONS_00063802	MTCONS_00060963	chr6	116774168	116774655	488	.	chr6	116575285	116759442	4648	+	*		-0.9983	-1	0.08	0.21	0	1.8	1.59	1.96
cis_mRNA_overlap	0	Lnc-CompleteIn-mRNAIntron	n339838	MTCONS_00032383	chr18	33954221	33955232	1011	+	chr18	33877674	34360026	5428	+	*		0.8228	1	0.28	0.16	0.34	1.86	1.76	2.6
cis_mRNA_overlap	0	Lnc-CompleteIn-mRNAIntron	n339838	MTCONS_00032382	chr18	33954221	33955232	1011	+	chr18	33877674	34360026	4864	+	*		0.9631	1	0.28	0.16	0.34	2.68	2.17	3.36
cis_mRNA_dw20k	17796	NA	n366032	MTCONS_00050042	chr3	186525481	186535910	540	+	chr3	186501977	186507686	1927	+	*		-0.9544	-1	0.14	0.06	0.18	41.15	47.74	31.34
tran	NA	NA	n326279	MTCONS_00032674	chr1	197135770	197173181	756	-	chr18	77794346	77830757	3161	+		-432.61	0.6673	0.866	0.27	0.35	0.64	7.23	9.01	9.01

tran	NA	NA	n384426	MTCONS_00003977	chr4	14472092	14473364	1273	-	chr1	2294444	2323088	9762	-		-1227.37	0.9893	1	7.81	9.25	8.68	6.94	8.53	8.11
tran	NA	NA	n384426	MTCONS_00039983	chr4	14472092	14473364	1273	-	chr2	238536213	238694823	8109	+		-1257	0.9599	1	7.81	9.25	8.68	1.72	3.49	3.26
tran	NA	NA	n384426	MTCONS_00036294	chr4	14472092	14473364	1273	-	chr19	40528842	40562147	24857	-		-672.06	0.9567	1	7.81	9.25	8.68	6.91	8.47	7.45
tran	NA	NA	LTCONS_00069236	MTCONS_00047278	chr8	155778	167642	9202	-	chr22	51222100	51243982	7214	+		-3301.62	0.9134	1	0.28	0.58	0.51	5.95	8.76	7.06
cis_mRNA_overlap	0	AntiCompleteIn-mRNAIntron	n385245	MTCONS_00065329	chr7	99927419	99930239	929	-	chr7	99775538	99948836	16269	+	*		0.7161	1	1.58	1.75	2.32	1.41	1.88	1.91
cis_mRNA_overlap	0	Lnc-CompleteIn-mRNAExon	n341479	MTCONS_00069574	chr8	30885536	30887165	1630	-	chr8	30877206	30890694	13200	-		-617.08	-0.9965	-1	0.22	0.99	0.82	2.01	1.65	1.7
cis_mRNA_overlap	0	mRNA	n345161	MTCONS_00017854	chr12	57745329	57816967	8502	-	chr12	57647174	57824682	4544	-	*		-0.866	-0.866	0.21	0.19	0.23	2.5	3.35	2.5
cis_mRNA_overlap	0	Lnc-AntiOverlap-mRNA	LTCONS_00072259	MTCONS_00070686	chr9	2452958	2621867	1656	-	chr9	2621474	2656905	6168	+	*		0.7206	0.866	0.7	1.48	0.7	6.17	7.07	3.92
tran	NA	NA	n385736	MTCONS_00021822	chrX	3735571	3747433	1242	-	chr14	102791829	102810753	5226	+		-807.22	-0.9877	-1	7.3	8.66	4.69	0.58	0.34	0.84
tran	NA	NA	n385736	MTCONS_00021820	chrX	3735571	3747433	1242	-	chr14	102783531	102810753	5199	+		-807.22	0.8233	1	7.3	8.66	4.69	0.18	0.75	0.11
cis_mRNA_dw20k	11419	NA	n405704	MTCONS_00030144	chr17	197721	424115	5068	-	chr17	435533	618096	2781	-	*		-0.803	-1	0.06	0	0.08	3.15	3.18	3.01
tran	NA	NA	n338541	MTCONS_00037006	chr1	226257217	226257654	438	+	chr19	58201202	58220617	13301	-		-573.73	0.9738	1	2.16	2.85	2.37	4.35	5.99	5.18
cis_mRNA_up10k	170	NA	n338717	MTCONS_00037524	chr2	27307990	27309442	1453	-	chr2	27309611	27324926	3470	+	*		0.9951	1	0.76	0.83	0.81	0.37	0.45	0.42
cis_mRNA_up10k	112	NA	n378962	MTCONS_00047600	chr22	28315417	28393763	562	+	chr22	28247630	28315306	2987	-	*		-0.9997	-1	0.19	0.12	0	2.59	2.85	3.34
tran	NA	NA	n387141	MTCONS_00047278	chr2	114368816	114384715	1388	-	chr22	51222100	51243982	7214	+		-686.61	0.9347	1	2.43	3.77	2.49	5.95	8.76	7.06
cis_mRNA_dw20k	5947	NA	n338973	MTCONS_00041855	chr2	144695635	144696173	539	-	chr2	144702119	145090106	4128	-	*		0.9054	1	0.55	1.06	1.04	1.48	2.51	2.04
tran	NA	NA	n325819	MTCONS_00047711	chr11	130609643	130626921	569	+	chr22	32896532	33448559	15401	-		-389.69	0.6636	0.866	0.06	0.29	0.06	2.72	2.94	1.9
cis_mRNA_dw20k	7217	NA	n406196	MTCONS_00066638	chr7	43980494	44058748	6466	-	chr7	44065964	44080295	4331	-	*		0.9996	1	0.37	0.42	0.34	2.81	3.87	2.1
cis_mRNA_dw20k	2499	NA	n335098	MTCONS_00017019	chr12	6645713	6646179	246	+	chr12	6648677	6665502	2962	-	*		0.996	1	0.34	0.31	0.15	2.55	2.53	2.24
cis_mRNA_up10k	2727	NA	n410042	MTCONS_00027065	chr16	89778264	89784573	1753	+	chr16	89787299	89809138	7047	+	*		-0.9646	-1	0.02	0.2	0.11	0.61	0.42	0.47
tran	NA	NA	n379701	MTCONS_00015840	chr18	29992145	29993199	857	+	chr12	54674445	54681404	3966	+		-1753.41	-0.8148	-1	1	0.42	0.59	6.1	8.43	6.45
tran	659367	NA	n408306	MTCONS_00035603	chr19	11784813	11797384	2000	+	chr19	12456750	12476545	5643	-		-937.36	-0.9993	-1	1.15	0.8	1.1	1.5	1.79	1.53
cis_mRNA_up10k	1492	NA	n410440	MTCONS_00031282	chr17	45900638	45908907	1898	-	chr17	45884647	45899147	4371	-	*		0.9799	1	0.24	0.33	0.41	1.38	1.67	1.79
cis_mRNA_up10k	8095	NA	n410440	MTCONS_00031281	chr17	45900638	45908907	1898	-	chr17	45884647	45892544	2846	-	*		0.9	1	0.24	0.33	0.41	0.63	0.68	1.06
cis_mRNA_dw20k	422	NA	n342192	MTCONS_00050191	chr3	197613328	197615303	1975	+	chr3	197518135	197612907	7748	+	*		-1	-1	0.49	0.73	0.5	0.27	0	0.26
cis_mRNA_overlap	0	Lnc-AntiOverlap-mRNA	LTCONS_00034770	MTCONS_00036693	chr19	51161641	51164921	2943	+	chr19	51163129	51223153	9110	-		-4158.2	0.9592	1	0.06	0.1	0.09	0.9	1.91	1.4
tran	NA	NA	n324712	MTCONS_00031779	chr22	20006237	20009256	576	-	chr17	73885041	73893054	3168	-		-613.92	0.8989	1	0.13	0.06	0	1.89	0.89	0.86
cis_mRNA_overlap	0	Lnc-AntiOverlap-mRNA	n324712	MTCONS_00046402	chr22	20006237	20009256	576	-	chr22	20008630	20055472	4182	+	*		-0.9869	-1	0.13	0.06	0	0.97	1.3	1.8
cis_mRNA_overlap	0	mRNA	n406343	MTCONS_00024160	chr15	85174691	85185694	4509	+	chr15	85144239	85185987	15744	+		-3058.16	0.9151	1	1.2	1.48	1.46	0.48	0.74	0.62
cis_mRNA_up10k	2013	NA	n381052	MTCONS_00000289	chr1	9732486	9747627	951	-	chr1	9749639	9789271	5938	+	*		-0.985	-1	0.29	0.61	0	1.66	1.01	1.97
cis_mRNA_overlap	0	AntiCompleteIn-mRNAIntron	n379953	MTCONS_00047351	chr22	19402955	19409368	3627	+	chr22	19318224	19419872	4944	-	*		0.9653	1	0.43	0.49	0.61	1.13	1.19	1.91
cis_mRNA_overlap	0	Lnc-CompleteIn-mRNAIntron	n341088	MTCONS_00014386	chr11	83212282	83213964	1683	-	chr11	83194882	84028487	2684	-	*		0.9778	1	0.02	0.19	0.26	1.02	1.75	2.42
cis_mRNA_up10k	65	NA	n380182	MTCONS_00066417	chr7	27153555	27161818	339	+	chr7	27139879	27153491	9355	-	*		0.6964	1	0.79	0.88	0	0.62	1.18	0.51
cis_mRNA_dw20k	1774	NA	n346011	MTCONS_00011406	chr11	17373294	17396117	4189	+	chr11	17298286	17371521	5510	+	*		-0.9742	-1	1.49	2.17	1.21	2.08	1.8	2.1
cis_mRNA_dw20k	1774	NA	n346011	MTCONS_00011405	chr11	17373294	17396117	4189	+	chr11	17298286	17371521	1885	+	*		-0.7251	-0.866	1.49	2.17	1.21	1.26	1.26	2.41
cis_mRNA_dw20k	15649	NA	n344500	MTCONS_00074726	chrX	102024113	102167269	4060	+	chrX	102000889	102008465	5271	+	*		-0.8889	-1	0.38	0.45	0.4	2.21	2.04	2.09
cis_mRNA_overlap	0	Lnc-AntiOverlap-mRNA	n342590	MTCONS_00009733	chr10	29698465	29776785	877	+	chr10	29746276	30024864	6943	-	*		-0.9102	-1	0.37	0.15	0.51	2.49	2.79	1.5
cis_mRNA_overlap	0	Lnc-AntiOverlap-mRNA	n342590	MTCONS_00009734	chr10	29698465	29776785	877	+	chr10	29746276	30025869	7018	-	*		0.9512	1	0.37	0.15	0.51	1.16	0.56	1.22
tran	NA	NA	n323816	MTCONS_00004693	chrX	119028703	119033529	772	-	chr1	35641973	35658743	11272	-		-708.92	0.8754	1	4.14	5.32	5.21	15.29	21.29	17.95
tran	NA	NA	n323816	MTCONS_00004694	chrX	119028703	119033529	772	-	chr1	35641973	35658743	8868	-		-708.92	0.9272	1	4.14	5.32	5.21	4.59	5.32	4.99
cis_mRNA_overlap	0	Lnc-CompleteIn-mRNAExon	LTCONS_00025847	MTCONS_00025848	chr16	3331155	3341617	5393	+	chr16	3331155	3341617	5520	+		-3090.88	-0.9042	-1	0.3	0.35	0.48	0.36	0.2	0.12
cis_mRNA_overlap	0	Lnc-CompleteIn-mRNAIntron	n385142	MTCONS_00064706	chr7	43157495	43202786	1839	+	chr7	43152125	43606182	9921	+	*		-0.9028	-1	0.11	0.2	0.25	4.68	4.6	4.36
cis_mRNA_dw20k	239	NA	LTCONS_00050599	MTCONS_00050600	chr3	44538373	44539740	1368	-	chr3	44539978	44552180	2507	-	*		0.822	0.866	1.81	1.93	2.02	1.09	1.09	1.5
tran	NA	NA	n345097	MTCONS_00024048	chrX	109085564	109158741	863	+	chr15	77713161	77781298	10875	+		-732.26	0.949	1	0.34	0.12	0.04	2.39	2.21	1.99
cis_mRNA_dw20k	13074	NA	n342607	MTCONS_00008657	chr10	88964456	88965038	583	-	chr10	88854460	88951383	3805	+	*		0.9985	1	0.32	0.75	0.9	0.58	2.72	3.28
cis_mRNA_dw20k	15643	NA	LTCONS_00074730	MTCONS_00074726	chrX	102024107	102219401	6708	+	chrX	102000889	102008465	5271	+	*		-0.9701	-1	1.69	2.18	1.93	2.21	2.04	2.09
tran	NA	NA	n381255	MTCONS_00074629	chr1	151763318	151766877	991	+	chrX	91034260	91139166	5096	+		-823.95	0.7389	1	4.77	7.11	4.96	7.71	11.75	10.77
cis_mRNA_dw20k	4788	NA	n345062	MTCONS_00072077	chr9	137029881	137030348	468	+	chr9	137000890	137025094	3436	+	*		0.7982	1	0.08	0	0.07	1.82	0.82	1.11
tran	NA	NA	n346504	MTCONS_00072661	chr5	45749057	45753300	668	+	chr9	40770255	40789853	5594	-		-390.85	0.8881	1	0.33	0.69	0.58	2.05	2.71	2.23
tran	NA	NA	n346504	MTCONS_00036294	chr5	45749057	45753300	668	+	chr19	40528842	40562147	24857	-		-663.66	0.9224	1	0.33	0.69	0.58	6.91	8.47	7.45
cis_mRNA_overlap	0	mRNA	LTCONS_00034471	MTCONS_00034472	chr19	44331384	44348207	7613	+	chr19	44331384	44357322	6804	+	*		0.682	0.866	2.91	3.08	3.04	2.32	2.71	2.32
cis_mRNA_overlap	0	Lnc-CompleteIn-mRNAExon	n381616	MTCONS_00011406	chr11	17353589	17371521	3256	+	chr11	17298286	17371521	5510	+		-1552.2	0.8718	1	0.11	0	0.24	2.08	1.8	2.1
cis_mRNA_overlap	0	mRNA	n381616	MTCONS_00011405	chr11	17353589	17371521	3256	+	chr11	17298286	17371521	1885	+		-849.01	0.8891	0.866	0.11	0	0.24	1.26	1.26	2.41
cis_mRNA_up10k	6819	NA	n408048	MTCONS_00050191	chr3	197477261	197511317	3824	+	chr3	197518135	197612907	7748	+	*		-0.7787	-1	4.55	5.86	5.42	0.27	0	0.26
cis_mRNA_overlap	0	mRNA	LTCONS_00027503	MTCONS_00027501	chr16	18494434	18496023	1590	-	chr16	18487752	18496838	2954	-		-1602.14	-0.9999	-1	0.76	1	1.15	0.46	0.17	0
cis_mRNA_overlap	0	Lnc-CompleteIn-mRNAIntron	n341437	MTCONS_00019701	chr13	111774999	111797169	1362	+	chr13	111767624	111958081	5346	+	*		-0.9774	-0.866	0.69	0.51	0.55	0	0.03	0.03
cis_mRNA_overlap	0	Lnc-AntiOverlap-mRNA	n411099	MTCONS_00012123	chr11	65336691	65337884	1194	-	chr11	65337856	65341749	1807	+	*		-0.6821	-1	1.17	1.66	1.18	1.26	0.39	0.58
tran	NA	NA	n323994	MTCONS_00005688	chr9	112401576	112407320	635	+	chr1	114258385	114300735	4864	-		-780.9	1	1	1.94	3.09	1.85	3.91	4.59	3.86

cis_mRNA_overlap	0	Lnc-AntiOverlap-mRNA	LTCONS_00015884	MTCONS_00017778	chr12	56223345	56230045	3517	+	chr12	56214649	56224054	3520	-	*	0.9964	1	1.62	1.42	1.44	0.88	0.18	0.31
cis_mRNA_overlap	0	Lnc-CompleteIn-mRNAIntron	n342914	MTCONS_00060710	chr6	90279820	90280172	353	+	chr6	90271332	90343500	6390	+	*	0.8058	0.866	0	0.1	0.1	2.57	3.98	3.14
tran	NA	NA	n380037	MTCONS_00066666	chr2	27505301	27526416	876	-	chr7	44605372	44621849	8184	-	-895.26	-0.9793	-0.866	0.08	0.04	0.04	5.65	8.18	7.64
cis_mRNA_overlap	0	Lnc-AntiOverlap-mRNA	n379069	MTCONS_00066177	chr7	1609725	1629257	3839	+	chr7	1606968	1609736	848	-	*	0.9998	1	0.41	0.62	0.07	5.14	5.66	4.24
cis_mRNA_dw20k	6961	NA	n345527	MTCONS_00037927	chr2	61157161	61167156	2794	+	chr2	61108682	61150201	2589	+	*	0.9967	1	0.79	0.66	0.5	2.12	1.89	1.51
cis_mRNA_dw20k	10638	NA	n339839	MTCONS_00032915	chr18	34809542	34812101	2557	+	chr18	34822738	35146000	4288	-	*	-0.9713	-1	0.29	0.43	0.5	1.39	1.04	1
cis_mRNA_overlap	0	CompleteIn-LncExon	n410678	MTCONS_00030241	chr17	3566187	3599698	6285	-	chr17	3566187	3599698	6213	-	-3510.14	-1	-1	0.17	0.42	0	1.02	0.33	1.49
tran	NA	NA	n386138	MTCONS_00073508	chr15	84873539	84898842	467	-	chr9	131018075	131038308	4252	-	-399.57	-0.794	-1	0.39	1.48	0.45	12.48	11.49	11.83
cis_mRNA_overlap	0	Lnc-CompleteIn-mRNAIntron	n381775	MTCONS_00014673	chr11	116941486	116942325	840	-	chr11	116713726	116969056	6344	-	*	0.7198	0.866	0.21	0.08	0.08	1.76	1.35	0.32
cis_mRNA_overlap	0	Lnc-CompleteIn-mRNAIntron	n381775	MTCONS_00014672	chr11	116941486	116942325	840	-	chr11	116713726	116969051	6519	-	*	-0.9891	-0.866	0.21	0.08	0.08	1.11	2.65	2.94
tran	NA	NA	n387427	MTCONS_00066703	chr6	35352370	35353406	734	-	chr7	47994874	48019266	10405	-	-601.35	0.9988	1	20.53	24.78	25.81	7.11	8.45	8.88
tran	NA	NA	n409645	MTCONS_00021822	chrX	3735576	3761935	1791	-	chr14	102791829	102810753	5226	+	-1275.16	0.9043	1	2.76	2.53	2.79	0.58	0.34	0.84
tran	NA	NA	n409645	MTCONS_00021820	chrX	3735576	3761935	1791	-	chr14	102783531	102810753	5199	+	-1275.16	-1	-1	2.76	2.53	2.79	0.18	0.75	0.11
cis_mRNA_overlap	0	Lnc-CompleteIn-mRNAIntron	n332375	MTCONS_00069395	chr8	13133760	13133963	204	-	chr8	12933204	13462145	14928	-	*	0.9257	1	1.31	1.59	0.59	8.1	10.25	6.79
cis_mRNA_overlap	0	AntiCompleteIn-mRNAIntron	n325612	MTCONS_00022741	chr14	89991368	89995001	2026	+	chr14	89622296	90085506	8118	-	*	-0.6078	-0.866	0.05	0.05	0.09	0.88	1.31	0.81
tran	NA	NA	n337808	MTCONS_00065329	chr1	28870972	28873054	2083	+	chr7	99775538	99948836	16269	+	-1448.58	0.8666	1	0.38	0.48	0.6	1.41	1.88	1.91
tran	NA	NA	n325680	MTCONS_00033686	chr13	79276554	79277821	428	+	chr19	11998621	12032411	17275	+	-188.57	-0.9977	-1	3.35	2.12	2.31	1.66	3.92	3.42
tran	NA	NA	n325680	MTCONS_00036294	chr13	79276554	79277821	428	+	chr19	40528842	40562147	24857	-	-184.77	-0.8497	-1	3.35	2.12	2.31	6.91	8.47	7.45
cis_mRNA_up10k	1938	NA	n369394	MTCONS_00060707	chr6	89830998	89853352	702	+	chr6	89855289	89875286	7845	+	*	-0.6719	-0.866	0.1	0.05	0.05	2.01	2.37	2.09
tran	NA	NA	n369754	MTCONS_00032984	chr7	26301389	26303446	427	+	chr18	45346666	45457071	23239	-	-688.92	0.7478	1	0.51	0.56	0.09	2.93	3.26	2.83
tran	27722695	NA	LTCONS_00007623	MTCONS_00002054	chr1	148858789	148859503	715	.	chr1	121107062	121136095	7425	+	-1208.8	-0.9905	-1	0.35	0.37	0.54	3.58	3.06	1.43
cis_mRNA_dw20k	8538	NA	LTCONS_00033972	MTCONS_00033967	chr19	21579921	21601152	13623	+	chr19	21541735	21571384	6244	+	*	0.9781	1	0.15	1.3	0.57	0.34	0.87	0.43
cis_mRNA_dw20k	1700	NA	n378844	MTCONS_00047445	chr22	21821680	21825558	2593	+	chr22	21827257	21871792	2404	-	*	-0.8004	-1	0	0.34	0.45	7.76	7.37	5.57
cis_mRNA_dw20k	1700	NA	n378844	MTCONS_00047443	chr22	21821680	21825558	2593	+	chr22	21827257	21871780	3677	-	*	0.9751	1	0	0.34	0.45	1.84	3.15	3.17
tran	NA	NA	n364921	MTCONS_00034629	chr2	61371541	61372104	434	-	chr19	47852538	47887580	5991	+	-508.45	0.9177	0.866	0.24	0.51	0.51	0.13	0.4	0.58
tran	NA	NA	n364921	MTCONS_00043334	chr2	61371541	61372104	434	-	chr20	34042961	34107003	15551	+	-488.27	0.9286	0.866	0.24	0.51	0.51	1.02	1.18	1.12
tran	NA	NA	n364921	MTCONS_00034630	chr2	61371541	61372104	434	-	chr19	47852627	47887580	9026	+	-513.68	0.8545	0.866	0.24	0.51	0.51	0.79	1.81	1.28
tran	NA	NA	LTCONS_00016475	MTCONS_00039983	chr12	111051832	111068271	2207	+	chr2	238536213	238694823	8109	+	-2214.84	0.8287	1	1.7	1.82	1.74	1.72	3.49	3.26
tran	NA	NA	LTCONS_00016475	MTCONS_00036294	chr12	111051832	111068271	2207	+	chr19	40528842	40562147	24857	-	-1295.92	0.9999	1	1.7	1.82	1.74	6.91	8.47	7.45
cis_mRNA_up10k	902	NA	n378885	MTCONS_00008074	chr10	31596658	31607964	564	-	chr10	31608865	31818836	7880	+	*	0.7693	1	0.22	0.24	0.18	1.19	2.43	1.16
cis_mRNA_dw20k	5573	NA	n368383	MTCONS_00059154	chr5	177387472	177392855	4735	-	chr5	177398427	177399290	864	.	*	0.9449	0.866	0.01	0	0.01	0.13	0.09	0.15
tran	NA	NA	n325017	MTCONS_00074579	chr20	25679854	25681397	320	+	chrX	75392518	75414076	17491	+	-160.35	0.8998	1	0.25	0.23	0	5.93	5.5	5.07
cis_mRNA_dw20k	7305	NA	n383369	MTCONS_00033616	chr19	10762539	10764548	2010	-	chr19	10713117	10755235	3504	+	*	0.9987	1	11.43	9.65	10.21	5.84	3.82	4.36
cis_mRNA_dw20k	7305	NA	n383369	MTCONS_00033615	chr19	10762539	10764548	2010	-	chr19	10713117	10755235	3761	+	*	-0.9998	-1	11.43	9.65	10.21	7.47	10.15	9.26
cis_mRNA_up10k	5837	NA	n375399	MTCONS_00027072	chr16	89883571	89889071	1807	+	chr16	89894907	89939169	7585	+	*	0.8551	1	0.22	0.88	0.39	2.73	3.62	3.39
tran	NA	NA	n372816	MTCONS_00031084	chr11	18230678	18235486	922	+	chr17	40190058	40202750	4056	-	-817.07	0.9739	1	2.89	3.08	3.57	2.79	3.06	3.35
cis_mRNA_up10k	3890	NA	n376812	MTCONS_00043190	chr20	23337245	23338763	855	-	chr20	23342652	23355092	6394	+	*	-0.9879	-1	0.04	0	0.15	0.52	0.56	0.21
cis_mRNA_up10k	84	NA	n378000	MTCONS_00047600	chr22	28315389	28382380	846	+	chr22	28247630	28315306	2987	-	*	-0.854	-1	0.7	0.39	0.33	2.59	2.85	3.34
cis_mRNA_overlap	0	Lnc-AntiOverlap-mRNA	n380092	MTCONS_00066417	chr7	27147396	27161792	509	+	chr7	27139879	27153491	9355	-	*	1	1	0.21	0.85	0.08	0.62	1.18	0.51
cis_mRNA_overlap	0	Lnc-AntiOverlap-mRNA	LTCONS_00036197	MTCONS_00034284	chr19	37988014	37999167	2103	-	chr19	37996748	38034296	8612	+	*	0.831	1	1.8	1.53	1.7	0.74	0.63	0.64
cis_mRNA_dw20k	1492	NA	n411624	MTCONS_00059455	chr6	3306123	3309396	2429	+	chr6	3258996	3304632	2052	+	*	0.8201	1	0.4	0.47	0.36	1.45	1.49	0.98
cis_mRNA_dw20k	5436	NA	LTCONS_00027280	MTCONS_00025836	chr16	3175953	3184883	1019	-	chr16	3162563	3170518	2260	+	*	-0.6305	-1	3.12	0.43	0.63	1.45	2.44	1.55
cis_mRNA_dw20k	5436	NA	LTCONS_00027280	MTCONS_00025837	chr16	3175953	3184883	1019	-	chr16	3162563	3170518	2726	+	*	-0.9988	-1	3.12	0.43	0.63	2.78	4.29	4.26
tran	NA	NA	n340853	MTCONS_00050190	chr17	73556195	73556850	656	+	chr3	197518118	197612907	7657	+	-681.14	0.7921	1	0.36	0.64	0.77	4.44	4.46	4.68
tran	NA	NA	n340853	MTCONS_00075262	chr17	73556195	73556850	656	+	chrX	153686623	153704519	11497	+	-556.28	0.9668	1	0.36	0.64	0.77	3.35	4.52	4.6
tran	NA	NA	n367382	MTCONS_00065208	chr4	124454586	124457636	546	-	chr7	92076700	92120647	2433	+	-621.14	-0.9904	-1	0.24	0.26	0.07	1.29	0.97	2.29
cis_mRNA_overlap	0	CompleteIn-LncIntron	n324000	MTCONS_00071543	chr9	108418160	108481900	2055	+	chr9	108456825	108476786	526	+	*	0.6025	0.866	0.02	0.15	0	1.09	1.09	0.12
cis_mRNA_overlap	0	Lnc-CompleteIn-mRNAIntron	n337681	MTCONS_00024507	chr15	30020390	30021735	1346	-	chr15	29992333	30114706	7008	-	*	0.8214	0.866	0.23	0.16	0.23	4.13	3.45	5.04
cis_mRNA_overlap	0	Lnc-CompleteIn-mRNAIntron	n337681	MTCONS_00024508	chr15	30020390	30021735	1346	-	chr15	29992333	30261111	6881	-	*	-0.9843	-0.866	0.23	0.16	0.23	2.17	3.13	2.35
cis_mRNA_overlap	0	mRNA	n375814	MTCONS_00029671	chr17	59475313	59476820	1394	+	chr17	59474205	59486827	6081	+	-1223.69	0.9941	1	1.6	1.96	3.54	1.54	1.84	2.58
tran	NA	NA	n364207	MTCONS_00031631	chr1	146644430	146645134	604	-	chr17	65066554	65241320	13834	-	-382.01	0.9843	0.866	0.06	0.11	0.11	1.17	1.69	1.81
cis_mRNA_overlap	0	mRNA	n339283	MTCONS_00028105	chr16	72317200	72325315	1003	-	chr16	72152996	72318932	5259	-	*	0.8356	1	5.71	7.3	8.35	1.94	2.04	3.44
cis_mRNA_dw20k	3911	NA	n337815	MTCONS_00024893	chr15	60737702	60739750	2048	-	chr15	60743660	60771344	3273	-	*	-0.9362	-1	0.27	0.47	0.36	1.3	1.17	1.2
cis_mRNA_overlap	0	mRNA	n337815	MTCONS_00024889	chr15	60737702	60739750	2048	-	chr15	60711808	60771344	7308	-	*	0.866	1	0.27	0.47	0.36	0.31	0.62	0.6
cis_mRNA_overlap	0	mRNA	n410145	MTCONS_00036823	chr19	53300661	53324922	4712	-	chr19	53299146	53360982	6056	-	-1999.12	0.9165	1	2.32	2.27	2.68	0.1	0.06	0.14
tran	NA	NA	n326545	MTCONS_00036294	chr1	83814047	84099267	3177	-	chr19	40528842	40562147	24857	-	-1550.51	0.9115	1	2.28	2.85	2.69	6.91	8.47	7.45
cis_mRNA_dw20k	5642	NA	n382707	MTCONS_00025367	chr15	89045149	89048564	3416	-	chr15	89054205	89089912	2879	-	*	0.7803	1	0.18	0.49	0.14	0.32	0.44	0
cis_mRNA_dw20k	5642	NA	n382707	MTCONS_00025366	chr15	89045149	89048564	3416	-	chr15	89054205	89089912	2949	-	*	-0.8663	-1	0.18	0.49	0.14	0.34	0	0.83
cis_mRNA_overlap	0	Lnc-CompleteIn-mRNAIntron	n370221	MTCONS_00067939	chr8	1304795	1306100	911	+	chr8	1010002	1656642	12652	+	*	0.8586	1	0.31	0.32	0.49	0.75	0.85	0.93

cis_mRNA_dw20k	5630	NA	n337480	MTCONS_00047374	chr22	19948785	19951714	295	+	chr22	19957343	19974747	4429	-	*		0.9527	1	0	0.5	1.11	11.05	11.26	12.3
cis_mRNA_overlap	0	mRNA	n338323	MTCONS_00035688	chr19	14272220	14294451	1451	-	chr19	14258549	14276912	8632	-		-1177.53	-0.9911	-1	1.46	1.14	1.43	0.71	1.15	0.81
cis_mRNA_up10k	5818	NA	n409416	MTCONS_00068443	chr8	42128820	42190171	4187	+	chr8	42195988	42229331	1261	+	*		-1	-1	0.12	0.5	0.1	24.41	20.83	24.63
cis_mRNA_dw20k	5265	NA	n369616	MTCONS_00065671	chr7	129780903	129782245	576	+	chr7	129710338	129775639	6441	+	*		0.6396	0.866	0.06	0.12	0.06	8.26	11.47	10.89
cis_mRNA_up10k	117	NA	LTCONS_00021396	MTCONS_00022569	chr14	71276379	71283598	2784	+	chr14	71188143	71276263	12575	-	*		0.9484	1	5.64	6.39	5.75	3.1	4.7	3.81
cis_mRNA_up10k	154	NA	LTCONS_00021396	MTCONS_00022568	chr14	71276379	71283598	2784	+	chr14	71188143	71276226	12607	-	*		-0.9936	-1	5.64	6.39	5.75	1.08	0.33	1.06
tran	NA	NA	n325704	MTCONS_00005688	chr13	63458505	63548145	1878	-	chr1	114258385	114300735	4864	-		-940.26	-0.7617	-1	0.09	0.07	0.14	3.91	4.59	3.86
tran	NA	NA	n385675	MTCONS_00043564	chr9	111922746	111923757	1012	-	chr20	44486220	44515128	8833	+		-868.59	0.9999	1	0.17	0.38	0.47	0.68	0.9	1
cis_mRNA_dw20k	14826	NA	n383835	MTCONS_00042880	chr20	1306054	1359378	2373	+	chr20	1246928	1291229	6448	+	*		-0.9734	-1	0.11	0	0.15	1.8	2.38	1.78
cis_mRNA_dw20k	15997	NA	n383835	MTCONS_00042879	chr20	1306054	1359378	2373	+	chr20	1246928	1290058	5690	+	*		0.9936	1	0.11	0	0.15	9.13	5.66	9.78
cis_mRNA_overlap	0	Lnc-CompleteIn-mRNAIntron	n341587	MTCONS_00070242	chr8	116580684	116581247	564	-	chr8	116420662	116681303	9969	-	*		-0.9389	-1	0.13	0.24	0.06	1.36	1.06	2.05
cis_mRNA_dw20k	2796	NA	n369093	MTCONS_00063088	chr6	111598892	111617099	663	+	chr6	111619894	111804624	11379	-	*		0.8918	0.866	1.27	1.81	1.27	1.19	1.54	0.9
tran	NA	NA	n369777	MTCONS_00041121	chr7	47622655	47630846	1257	+	chr2	74699085	74710421	5074	-		-798.59	0.9235	1	0.25	0.05	0.15	1.35	0.28	1.2
cis_mRNA_dw20k	6362	NA	LTCONS_00024026	MTCONS_00024021	chr15	75638984	75648130	8535	+	chr15	75628374	75632623	1063	+	*		0.9926	1	1.3	1.88	1.95	0.35	0.89	1.05
cis_mRNA_up10k	4662	NA	LTCONS_00067933	MTCONS_00069250	chr8	685887	869722	13543	+	chr8	561770	681226	6135	-	*		-0.9483	-1	0.96	1.11	0.85	2.47	2.25	3
cis_mRNA_overlap	0	Lnc-AntiOverlap-mRNA	n345720	MTCONS_00008897	chr10	102729268	102730478	1064	-	chr10	102729270	102746027	5403	+	*		-0.9903	-1	0.13	0.03	0.15	0.49	1.01	0.48
tran	NA	NA	n338097	MTCONS_00047711	chr1	95123089	95285834	1328	-	chr22	32896532	33448559	15401	-		-1409.09	0.8263	1	0.3	0.77	0.14	2.72	2.94	1.9
tran	NA	NA	n383185	MTCONS_00073553	chr17	43295637	43299132	1556	-	chr9	132396748	132404448	6463	-		-1691.91	0.9908	1	0.31	0.3	0.5	3.16	3	3.84
tran	NA	NA	n363715	MTCONS_00015352	chr1	173978405	173985344	448	-	chr12	14538004	14655865	9006	+		-363.64	0.9723	1	0.76	0.54	0.98	9.57	7.86	13.72
tran	NA	NA	n363715	MTCONS_00031083	chr1	173978405	173985344	448	-	chr17	40190058	40196297	4596	-		-316.95	-0.8857	-1	0.76	0.54	0.98	2.26	2.3	1.43
tran	NA	NA	n326036	MTCONS_00034245	chr10	27260141	27263858	868	-	chr19	37096218	37128259	11934	+		-738.81	-0.9494	-1	0.66	0.73	0.58	0.92	0.83	0.95
tran	NA	NA	n326036	MTCONS_00034244	chr10	27260141	27263858	868	-	chr19	37096218	37128259	14238	+		-738.81	0.9465	1	0.66	0.73	0.58	3.19	3.41	3.12
tran	2071657	NA	n409293	MTCONS_00024149	chr15	82753447	82769399	6807	+	chr15	84841055	84878025	11652	+		-5029.16	0.9984	1	7.76	6.32	4.93	3.39	2.37	1.56
tran	NA	NA	n326405	MTCONS_00037005	chr1	144084411	144086911	666	-	chr19	58201202	58220602	12336	-		-626.52	0.9764	1	0.36	0.18	0.49	0.3	0.16	0.34
cis_mRNA_dw20k	1418	NA	n385507	MTCONS_00070921	chr9	35406752	35483026	2863	+	chr9	35161900	35405335	6519	+	*		0.963	1	0	0.01	0.03	46.3	48.85	50.76
tran	7513874	NA	n381511	MTCONS_00008663	chr10	81585662	81587307	1646	+	chr10	89101180	89131137	11860	+		-1386.59	0.989	1	8.47	7.08	8	2.47	1.97	2.37
tran	7513874	NA	n381511	MTCONS_00008664	chr10	81585662	81587307	1646	+	chr10	89101180	89131137	10665	+		-1386.59	-0.9813	-1	8.47	7.08	8	0	0.45	0.07
cis_mRNA_overlap	0	Lnc-AntiOverlap-mRNA	LTCONS_00001055	MTCONS_00004873	chr1	44457280	44462284	3136	+	chr1	44462060	44497134	6000	-	*		-0.9622	-1	2.62	2.79	3.01	0.6	0.39	0.29
tran	NA	NA	n371417	MTCONS_00022667	chrX	287992	288942	863	+	chr14	77249021	77279283	7871	-		-771.48	-0.8822	-1	0.04	0.01	0.07	1.82	2.33	1.8
tran	47756189	NA	n382658	MTCONS_00024565	chr15	82633124	82637131	2456	-	chr15	34671269	34876936	7605	-		-1253.43	-0.9832	-1	0.69	1.08	0.91	0.83	0.41	0.66
cis_mRNA_up10k	5867	NA	n384115	MTCONS_00046590	chr22	24385938	24401899	2070	-	chr22	24407765	24574596	7448	+	*		-0.9691	-1	0.05	0.03	0.01	1.13	1.62	1.81
tran	NA	NA	n383892	MTCONS_00037001	chr20	33421380	33422265	886	-	chr19	58052250	58071227	7132	-		-807.16	0.8223	1	0.12	0.29	0.25	1.26	1.93	1.41
cis_mRNA_up10k	119	NA	LTCONS_00025869	MTCONS_00027304	chr16	3661713	3708195	5669	+	chr16	3629261	3661595	9627	-	*		0.7643	1	2.94	3.06	3.66	0.64	0.84	0.89
cis_mRNA_dw20k	11624	NA	n375550	MTCONS_00030214	chr17	2615677	2616453	695	+	chr17	2628076	2652895	611	-	*		-0.8009	-1	0.78	1.71	0.88	1.68	0.84	1.1
cis_mRNA_dw20k	16072	NA	n375550	MTCONS_00030215	chr17	2615677	2616453	695	+	chr17	2632524	2652894	1607	-	*		0.6868	1	0.78	1.71	0.88	0.4	0.88	0.81
tran	NA	NA	n367743	MTCONS_00050368	chr5	54359007	54362058	567	+	chr3	15111560	15140295	7253	-		-486.63	0.6989	1	0.13	0.22	0.23	1.04	1.13	1.57
tran	NA	NA	n375515	MTCONS_00047279	chr17	81189540	81190888	611	+	chr22	51222100	51243982	7371	+		-354.83	0.9977	1	2.62	3.59	0.53	2.31	2.56	1.58
tran	41915799	NA	n407936	MTCONS_00008658	chr10	46897641	46939145	3290	+	chr10	88854943	88951383	3782	+		-1251.58	0.9222	1	0.11	0.07	0.09	6.11	2.97	3.4
cis_mRNA_dw20k	12	NA	n375850	MTCONS_00029926	chr17	74775769	74778303	2433	-	chr17	74733490	74775758	3503	+	*		-0.9483	-1	0.26	0.42	0.24	1.54	1.37	1.66
tran	NA	NA	n324160	MTCONS_00039982	chr9	6037360	6056714	499	+	chr2	238536213	238694823	8202	+		-244.24	-0.9662	-1	0	0.07	0.13	8.48	8.35	8.06
cis_mRNA_up10k	5822	NA	n339009	MTCONS_00027161	chr16	783055	785525	2162	+	chr16	771946	777234	4844	-	*		-0.7448	-0.866	0.49	0.49	0.52	0.46	0.61	0.39
cis_mRNA_up10k	5822	NA	n339009	MTCONS_00027160	chr16	783055	785525	2162	+	chr16	771946	777234	4194	-	*		0.648	0.866	0.49	0.49	0.52	0.17	0.55	0.64
cis_mRNA_dw20k	10441	NA	n339009	MTCONS_00025670	chr16	783055	785525	2162	+	chr16	765173	772615	5743	+	*		-0.7696	-0.866	0.49	0.49	0.52	2.01	2.58	1.7
tran	NA	NA	n344767	MTCONS_00066703	chr1	91297428	91357295	1615	+	chr7	47994874	48019266	10405	-		-973.96	0.9997	1	1.89	2.56	2.75	7.11	8.45	8.88
tran	NA	NA	n341861	MTCONS_00009062	chr12	24400760	24401200	441	+	chr10	114206756	114590547	13265	+		-399.66	-0.7777	-1	0.09	0	0.08	2.59	3.51	3.26
tran	NA	NA	LTCONS_00063898	MTCONS_00062306	chr6_mann_hap4	1502623	1505473	236	-	chr6	30152232	30181191	3201	-		-189.35	0.7703	1	1.56	0	2.22	5.31	5.24	6.25
cis_mRNA_overlap	0	Lnc-CompleteIn-mRNAExon	n407211	MTCONS_00073398	chr9	125871773	125877756	5984	-	chr9	125871758	126030961	18437	-		-4208.74	-1	-1	1.9	2.13	1.81	6.47	6.31	6.53
cis_mRNA_overlap	0	Lnc-CompleteIn-mRNAIntron	n337645	MTCONS_00003974	chr1	2253784	2255494	1711	-	chr1	2252696	2323029	1605	-	*		0.9333	0.866	0.04	0.37	0.04	0.23	0.45	0.31
cis_mRNA_overlap	0	mRNA	n339061	MTCONS_00042256	chr2	190611390	190613707	1696	-	chr2	190611295	190627994	2640	-		-731.7	0.9956	1	0.15	0.36	0.29	3.65	4.5	4.29
tran	41525329	NA	n372015	MTCONS_00008663	chr10	47571665	47575852	473	+	chr10	89101180	89131137	11860	+		-288.47	0.982	1	0.08	0	0.05	2.47	1.97	2.37
tran	41525329	NA	n372015	MTCONS_00008664	chr10	47571665	47575852	473	+	chr10	89101180	89131137	10665	+		-288.47	-0.9725	-1	0.08	0	0.05	0	0.45	0.07
cis_mRNA_overlap	0	Lnc-AntiOverlap-mRNA	n408286	MTCONS_00060710	chr6	90341943	90348216	5514	-	chr6	90271332	90343500	6390	+		-2935.2	0.9304	1	1.44	2.46	2.19	2.57	3.98	3.14
cis_mRNA_overlap	0	mRNA	n380180	MTCONS_00067939	chr8	687651	1087777	811	+	chr8	1010002	1656642	12652	+	*		-0.9884	-1	0.92	0.81	0.66	0.75	0.85	0.93
tran	NA	NA	n367818	MTCONS_00074629	chr5	135802366	135844717	676	+	chrX	91034260	91139166	5096	+		-577.23	-0.9985	-1	3.81	0	1.12	7.71	11.75	10.77
tran	NA	NA	n376313	MTCONS_00041121	chr19	37168290	37177738	908	+	chr2	74699085	74710421	5074	-		-564.25	-0.9099	-1	0.29	0.8	0.56	1.35	0.28	1.2
cis_mRNA_dw20k	2539	NA	LTCONS_00036145	MTCONS_00036154	chr19	36803588	36822640	4013	-	chr19	36825178	36870284	8211	-	*		0.6695	1	1.58	1.66	1.08	0.17	0.37	0.15
cis_mRNA_dw20k	2539	NA	LTCONS_00036145	MTCONS_00036153	chr19	36803588	36822640	4013	-	chr19	36825178	36870284	7852	-	*		0.998	1	1.58	1.66	1.08	2.91	2.96	2.71
tran	NA	NA	n383880	MTCONS_00033686	chr20	25165310	25177508	2158	-	chr19	11998621	12032411	17275	+		-1104.73	0.9967	1	5.2	5.57	5.46	1.66	3.92	3.42
tran	NA	NA	n383880	MTCONS_00033685	chr20	25165310	25177508	2158	-	chr19	11998607	12032411	17331	+		-1104.73	-0.9944	-1	5.2	5.57	5.46	4.11	2.39	2.73
tran	NA	NA	n383880	MTCONS_00036294	chr20	25165310	25177508	2158	-	chr19	40528842	40562147	24857	-		-1146.38	0.9188	1	5.2	5.57	5.46	6.91	8.47	7.45
cis_mRNA_overlap	0	Lnc-CompleteIn-mRNAIntron	n386655	MTCONS_00011646	chr11	43918853	43921422	2570	+	chr11	43902357	43941906	1561	+	*		0.9664	1	0.58	0.35	0.23	2.64	1.87	0.85
cis_mRNA_overlap	0	Lnc-AntiOverlap-mRNA	LTCONS_00072255	MTCONS_00070686	chr9	2421216	2621755	4815	-	chr9	2621474	2656905	6168	+	*		0.774	1	0.31	1.64	0.18	6.17	7.07	3.92
tran	44866458	NA	n332776	MTCONS_00033296	chr19	45842441	45843009	569	+	chr19	925746	975984	6143	+		-662.38	0.972	1	0.21	0	0.06	9.89	7.25	7.38

cis_mRNA_overlap	0	Lnc-CompleteIn-mRNAIntron	n385327	MTCONS_00069250	chr8	590408	594400	3993	-	chr8	561770	681226	6135	-	*		-0.9584	-0.866	1.17	1.17	0.47	2.47	2.25	3
cis_mRNA_overlap	0	mRNA	LTCONS_00041143	MTCONS_00041144	chr2	75889337	75904999	12571	-	chr2	75889337	75938111	6743	-		-2746.31	1	1	0.1	0.12	0.11	0.07	0.09	0.08
cis_mRNA_dw20k	1473	NA	n341321	MTCONS_00067545	chr7	131178723	131183518	3769	-	chr7	131184990	131241390	6269	-	*		-0.6628	-0.866	0.02	0.02	0.05	1.05	2.25	0.73
cis_mRNA_overlap	0	CompleteIn-LncExon	n407661	MTCONS_00033966	chr19	21541735	21571384	11721	+	chr19	21541735	21571384	10481	+		-4197.7	-0.8726	-1	8.98	6.5	3.99	0.46	0.49	3.14
cis_mRNA_overlap	0	AntiCompleteIn-mRNAIntron	n345570	MTCONS_00042485	chr2	218820768	218857691	1295	+	chr2	218664512	218876680	12054	-	*		-0.9898	-1	1.66	1.91	1.49	2.5	1.4	2.92
tran	NA	NA	n324381	MTCONS_00024980	chr6	135068891	135070180	929	-	chr15	66184371	66546119	9509	-		-719.01	0.9948	0.866	0.08	0	0.09	0.07	0.04	0.07
tran	NA	NA	n334772	MTCONS_00064076	chr6	31323945	31324734	544	+	chr6_ssto_hap7	2655403	2658749	1578	-		-1147.1	0.7298	1	0.34	0.31	0.06	11.2	3.05	0.93
tran	NA	NA	n380362	MTCONS_00069395	chr11	82783160	82784425	825	+	chr8	12933204	13462145	14928	-		-649.31	0.9709	1	1.69	1.99	1.26	8.1	10.25	6.79
tran	NA	NA	n387533	MTCONS_00039983	chr6	2370339	2376003	1980	+	chr2	238536213	238694823	8109	+		-1911.84	0.9178	1	7.98	10.5	9.23	1.72	3.49	3.26
tran	NA	NA	n387533	MTCONS_00036294	chr6	2370339	2376003	1980	+	chr19	40528842	40562147	24857	-		-1363.68	0.9854	1	7.98	10.5	9.23	6.91	8.47	7.45
cis_mRNA_overlap	0	Lnc-AntiOverlap-mRNA	n377812	MTCONS_00071422	chr9	98575575	98638259	809	-	chr9	98637246	98780791	10926	+	*		0.9951	1	0	0.61	0.3	6.78	9.15	8.15
cis_mRNA_dw20k	2515	NA	n342030	MTCONS_00016578	chr12	117293950	117295968	2019	-	chr12	117176090	117291436	6271	+	*		0.9757	1	5.39	6.88	3.71	1.62	2.27	1.29
tran	17399374	NA	n345939	MTCONS_00024148	chr15	102277398	102284705	237	-	chr15	84840742	84878025	13375	+		-153.91	0.9123	1	0.36	0.49	0.32	3.42	3.94	2.61
cis_mRNA_overlap	0	Lnc-AntiOverlap-mRNA	n406374	MTCONS_00011457	chr11	19732480	19736146	3667	-	chr11	19734881	20143147	10928	+	*		0.9917	1	2.29	2.61	1.87	0.24	0.31	0.09
tran	NA	NA	n345983	MTCONS_00021624	chr4	42287839	42294639	1096	-	chr14	91580357	91692629	3655	+		-1089.14	0.943	1	0.31	0.2	0.37	1.69	1.28	2.45
cis_mRNA_up10k	8047	NA	n342995	MTCONS_00066417	chr7	27161537	27168379	2021	+	chr7	27139879	27153491	9355	-	*		0.9882	0.866	0.19	0.35	0.19	0.62	1.18	0.51
tran	NA	NA	n324570	MTCONS_00035162	chr6	21630686	21631982	543	-	chr19	58976821	58996322	13062	+		-445.61	-0.9091	-1	3.13	5	2.03	0.64	0.59	0.79
cis_mRNA_dw20k	3713	NA	n340148	MTCONS_00010764	chr10	116184204	116187157	2954	-	chr10	116190869	116527986	7269	-	*		-0.8786	-0.866	0.08	0.07	0.07	16.22	19.85	23.17
cis_mRNA_dw20k	3713	NA	n340148	MTCONS_00010760	chr10	116184204	116187157	2954	-	chr10	116190869	116392281	7618	-	*		0.8687	0.866	0.08	0.07	0.07	26.22	21.88	24.03
cis_mRNA_up10k	6439	NA	n380146	MTCONS_00069250	chr8	687664	704258	965	+	chr8	561770	681226	6135	-	*		-0.9584	-0.866	0.48	0.48	0.27	2.47	2.25	3
tran	NA	NA	n326471	MTCONS_00017912	chr1	116508428	116510385	914	+	chr12	63952662	64062424	11449	-		-608.39	-0.9751	-1	0.88	0.61	0.49	2.01	2.92	3.01
cis_mRNA_overlap	0	mRNA	LTCONS_00027159	MTCONS_00027161	chr16	771946	777219	4747	-	chr16	771946	777234	4844	-		-3784.03	-0.9958	-1	0.4	0.19	0.54	0.46	0.61	0.39
cis_mRNA_overlap	0	Lnc-AntiOverlap-mRNA	LTCONS_00027159	MTCONS_00025670	chr16	771946	777219	4747	-	chr16	765173	772615	5743	+	*		-0.9985	-1	0.4	0.19	0.54	2.01	2.58	1.7
tran	NA	NA	n383178	MTCONS_00004524	chr17	40894172	40895176	1005	-	chr1	28296855	28406960	5892	-		-886.75	-0.7439	-0.866	0.11	0.13	0.13	1.69	1.24	0.27
cis_mRNA_overlap	0	Lnc-CompleteIn-mRNAExon	n341808	MTCONS_00073668	chr9	138898385	138900389	1998	-	chr9	138898319	138944231	7520	-		-1335.96	0.7637	1	0.1	0.09	0.22	0.57	0.34	0.66
tran	NA	NA	LTCONS_00028869	MTCONS_00022356	chr17	18601292	18607785	789	+	chr14	50882427	50999404	7199	-		-471.9	0.9851	1	2.9	4.1	6.34	2.85	2.95	3.05
cis_mRNA_overlap	0	Lnc-CompleteIn-mRNAIntron	n339936	MTCONS_00007684	chr10	1147572	1149291	1720	+	chr10	1102764	1180847	7236	+	*		-0.8122	-1	0.18	0.38	0.2	0.11	0.08	0.09
tran	NA	NA	n325341	MTCONS_00033686	chr2	37824980	37827693	384	+	chr19	11998621	12032411	17275	+		-532.41	0.8646	1	7.29	9.51	7.98	1.66	3.92	3.42
tran	NA	NA	n325341	MTCONS_00033685	chr2	37824980	37827693	384	+	chr19	11998607	12032411	17331	+		-532.41	-0.852	-1	7.29	9.51	7.98	4.11	2.39	2.73
tran	NA	NA	n325341	MTCONS_00036294	chr2	37824980	37827693	384	+	chr19	40528842	40562147	24857	-		-543.33	0.9992	1	7.29	9.51	7.98	6.91	8.47	7.45
tran	NA	NA	n410478	MTCONS_00033686	chr6	4226965	4230624	1773	+	chr19	11998621	12032411	17275	+		-1076.3	-0.7677	-1	0.52	0.32	0.49	1.66	3.92	3.42
tran	NA	NA	n410478	MTCONS_00033685	chr6	4226965	4230624	1773	+	chr19	11998607	12032411	17331	+		-1076.3	0.7518	1	0.52	0.32	0.49	4.11	2.39	2.73
tran	NA	NA	n410066	MTCONS_00066703	chr19	36980529	36981942	1414	+	chr7	47994874	48019266	10405	-		-1699.93	0.9983	1	0.98	1.11	1.14	7.11	8.45	8.88
tran	2105359	NA	LTCONS_00064616	MTCONS_00066544	chr7	32852367	32861205	399	+	chr7	34966563	35076785	6648	-		-193.48	-0.9672	-1	1.42	1.16	1.24	0.22	0.67	0.43
cis_mRNA_dw20k	5940	NA	n369617	MTCONS_00065671	chr7	129781578	129782455	392	+	chr7	129710338	129775639	6441	+	*		-0.9855	-0.866	0.1	0.09	0.09	8.26	11.47	10.89
tran	NA	NA	n325567	MTCONS_00002053	chr17	15650705	15661668	413	-	chr1	121107062	121136095	7428	+		-390	0.9705	1	0.09	0.17	0	4.79	4.92	4.73
cis_mRNA_overlap	0	Lnc-AntiOverlap-mRNA	n338916	MTCONS_00038426	chr2	97480968	97482303	902	-	chr2	97481952	97503624	13254	+	*		0.9958	1	1.26	0.54	0.98	0.61	0.29	0.46
cis_mRNA_overlap	0	Lnc-AntiOverlap-mRNA	n338916	MTCONS_00038427	chr2	97480968	97482303	902	-	chr2	97481978	97503624	12461	+	*		-0.9747	-1	1.26	0.54	0.98	0.54	1.07	0.85
cis_mRNA_overlap	0	Lnc-AntiOverlap-mRNA	n339545	MTCONS_00046270	chr21	47649441	47655367	1943	+	chr21	47654955	47706207	6873	-	*		-0.9219	-1	0.34	0.65	0.52	3.95	2.85	3.7
cis_mRNA_overlap	0	Lnc-AntiOverlap-mRNA	n339545	MTCONS_00046271	chr21	47649441	47655367	1943	+	chr21	47654955	47706264	6826	-	*		0.98	1	0.34	0.65	0.52	2.73	4.66	4.2
cis_mRNA_up10k	68	NA	n385196	MTCONS_00064920	chr7	65187621	65215911	2000	-	chr7	65215978	65229253	2515	+	*		-0.9856	-1	0.92	0.67	0.48	1.79	2.38	2.61
tran	NA	NA	n384439	MTCONS_00033686	chr4	39529639	39596327	2787	+	chr19	11998621	12032411	17275	+		-1320.83	0.9965	1	26.87	29.11	28.8	1.66	3.92	3.42
tran	NA	NA	n384439	MTCONS_00033685	chr4	39529639	39596327	2787	+	chr19	11998607	12032411	17331	+		-1320.83	-0.9982	-1	26.87	29.11	28.8	4.11	2.39	2.73
tran	NA	NA	n384439	MTCONS_00036294	chr4	39529639	39596327	2787	+	chr19	40528842	40562147	24857	-		-1331.77	0.8412	1	26.87	29.11	28.8	6.91	8.47	7.45
tran	139337786	NA	n326310	MTCONS_00000972	chr1	180322309	180325870	1145	+	chr1	40974409	40984524	4743	+		-991.62	0.9379	0.866	0.06	0.03	0.03	1.1	0.56	0.75
tran	NA	NA	n326310	MTCONS_00042922	chr1	180322309	180325870	1145	+	chr20	2854142	3028899	10710	+		-1039.14	-0.8934	-0.866	0.06	0.03	0.03	0.54	0.94	0.76
tran	NA	NA	n406198	MTCONS_00072236	chr15	102501016	102516808	2554	+	chr9	14430	30786	3251	-		-1710.84	0.9944	1	5.49	3.71	7.03	0.08	0.04	0.13
cis_mRNA_up10k	8526	NA	n410111	MTCONS_00013219	chr11	3022152	3078681	2819	-	chr11	2965660	3013627	2500	-	*		0.9948	1	0.44	0	0.88	45.18	41.78	47.56
tran	NA	NA	n377832	MTCONS_00026493	chr2	47558519	47572128	1046	-	chr16	53468351	53526530	7443	+		-933.48	0.9683	1	1.5	2.04	2.84	1.19	1.43	2.6
cis_mRNA_up10k	62	NA	n377928	MTCONS_00070686	chr9	2422702	2621413	3108	-	chr9	2621474	2656905	6168	+	*		0.8359	1	0.35	0.68	0.27	6.17	7.07	3.92
tran	NA	NA	LTCONS_00040928	MTCONS_00047712	chr2	64479550	64484740	410	-	chr22	32896532	33454377	15152	-		-298.28	-0.9945	-1	0.66	0.67	1.07	5.15	5.06	4.49
cis_mRNA_up10k	91	NA	n380333	MTCONS_00063088	chr6	111804714	111814206	2191	+	chr6	111619894	111804624	11379	-	*		0.8918	0.866	0.21	0.42	0.21	1.19	1.54	0.9
cis_mRNA_overlap	0	Lnc-AntiOverlap-mRNA	n379774	MTCONS_00025836	chr16	3160462	3165599	1218	-	chr16	3162563	3170518	2260	+	*		0.9596	1	0.3	0.73	0.46	1.45	2.44	1.55
cis_mRNA_overlap	0	Lnc-AntiOverlap-mRNA	n379774	MTCONS_00025837	chr16	3160462	3165599	1218	-	chr16	3162563	3170518	2726	+	*		0.7944	1	0.3	0.73	0.46	2.78	4.29	4.26
tran	209261	NA	n385219	MTCONS_00066942	chr7	74702377	74712617	1008	-	chr7	74921877	74988366	1808	-		-508.48	-0.981	-1	2.33	2.27	2.48	5.02	5.8	4.1
cis_mRNA_overlap	0	Lnc-CompleteIn-mRNAExon	n384936	MTCONS_00060313	chr6	42855731	42857596	1866	+	chr6	42846215	42857611	5611	+		-1369.96	-0.9837	-1	14.61	16.78	17.3	23.39	22.38	21.81
cis_mRNA_dw20k	6172	NA	n338646	MTCONS_00040275	chr2	8861264	8862816	1553	-	chr2	8868987	8977805	7243	-	*		0.772	1	1.83	1.56	1.91	45.94	44	57.02

cis_mRNA_dw20k	6172	NA	n338646	MTCONS_00040276	chr2	8861264	8862816	1553	-	chr2	8868987	8977805	7240	-	*		-0.9923	-1	1.83	1.56	1.91	126.23	129.47	125.85
cis_mRNA_up10k	231	NA	n410007	MTCONS_00016672	chr12	122989190	123011560	2430	-	chr12	123011790	123076557	4953	+	*		0.8681	1	1.91	2.4	1.99	0.78	1.05	0.95
tran	NA	NA	n375707	MTCONS_00064836	chr17	36443036	36452228	798	-	chr7	55954858	56008496	3227	+		-489.37	0.9796	1	0.63	0.67	0.41	6.55	6.76	6.15
tran	NA	NA	n375707	MTCONS_00043333	chr17	36443036	36452228	798	-	chr20	34042961	34107003	15277	+		-593.88	0.6338	1	0.63	0.67	0.41	0.44	1.32	0.42
cis_mRNA_overlap	0	mRNA	LTCONS_00065333	MTCONS_00065329	chr7	99933665	99948836	1969	+	chr7	99775538	99948836	16269	+		-1378.53	-0.9954	-1	15.05	7.01	5.48	1.41	1.88	1.91
cis_mRNA_overlap	0	Lnc-CompleteIn-mRNAIntron	n324429	MTCONS_00060832	chr6	109179550	109196352	1785	+	chr6	109169633	109295352	3299	+	*		0.9414	0.866	0.1	0.09	0.09	1.36	0.67	0.31
cis_mRNA_dw20k	6790	NA	n342905	MTCONS_00016578	chr12	117298225	117318837	1104	-	chr12	117176090	117291436	6271	+	*		0.7958	1	5.03	5.2	2.64	1.62	2.27	1.29
cis_mRNA_up10k	2043	NA	n364212	MTCONS_00006035	chr1	151120885	151123810	1722	-	chr1	151101331	151118843	8339	-	*		-0.8151	-1	0.48	0.39	0.36	1.14	1.26	1.75
tran	71647530	NA	n371757	MTCONS_00010695	chr10	33973683	33975992	452	+	chr10	105623521	105678045	8971	-		-484.74	0.864	1	0	0.08	0.09	2.12	2.32	2.61
cis_mRNA_dw20k	4010	NA	n377561	MTCONS_00046786	chr22	29712789	29715727	1225	-	chr22	29663998	29708780	9225	+	*		0.819	1	0	0.16	0.15	0	0.08	0.03
cis_mRNA_up10k	962	NA	n338137	MTCONS_00005657	chr1	112533158	112541464	3067	+	chr1	112318454	112532197	2356	-	*		0.9922	1	0.07	0.18	0.05	2.05	3.93	2.01
tran	NA	NA	n324739	MTCONS_00051424	chr22	18710726	18711888	446	-	chr3	126156444	126194841	22987	-		-537.51	0.9982	1	0.24	0.62	0.86	0.59	0.63	0.65
cis_mRNA_up10k	434	NA	n373301	MTCONS_00016505	chr12	112277583	112279752	614	-	chr12	112280185	112331416	1636	+	*		-0.9693	-1	0	0.35	0.12	5.27	2.6	4.99
cis_mRNA_up10k	31	NA	n339049	MTCONS_00039355	chr2	182752578	182756390	3808	-	chr2	182756420	182795554	5840	+	*		-0.8982	-1	0.1	0.16	0.07	8.35	6.72	12.87
tran	NA	NA	n323977	MTCONS_00010695	chr9	124203070	124205006	297	-	chr10	105623521	105678045	8971	-		-407.06	-0.8504	-1	0.61	0.5	0.49	2.12	2.32	2.61
tran	NA	NA	n323977	MTCONS_00031084	chr9	124203070	124205006	297	-	chr17	40190058	40202750	4056	-		-424.85	-0.892	-1	0.61	0.5	0.49	2.79	3.06	3.35
cis_mRNA_up10k	19	NA	LTCONS_00069531	MTCONS_00068295	chr8	28553718	28558981	4062	-	chr8	28558999	28612901	8025	+	*		0.9986	1	0.07	0	0.13	1.71	1.4	2.03
cis_mRNA_dw20k	14588	NA	n342001	MTCONS_00062835	chr6	76001574	76003213	1638	-	chr6	76017800	76044494	3756	-	*		0.671	0.866	0.08	0.08	0.06	0.45	1.19	0.24
tran	NA	NA	n383030	MTCONS_00000817	chr16	89262802	89264585	1072	-	chr1	33004756	33071548	5997	+		-952.43	0.9417	1	0.28	0.11	0.32	3.16	2.9	3.42
cis_mRNA_overlap	0	Lnc-CompleteIn-mRNAIntron	n341786	MTCONS_00071788	chr9	128519873	128520523	651	+	chr9	128508464	128729859	4118	+	*		0.9751	1	3.69	6.72	1.1	5.16	7.11	1.44
tran	NA	NA	n324045	MTCONS_00029926	chr9	92103004	92235591	1013	-	chr17	74733490	74775758	3503	+		-632.83	0.9919	1	0.07	0	0.1	1.54	1.37	1.66
tran	NA	NA	n385216	MTCONS_00032984	chr7	74233343	74233767	425	-	chr18	45346666	45457071	23239	-		-536.11	-0.9865	-1	0.63	0.49	0.64	2.93	3.26	2.83
tran	NA	NA	n385216	MTCONS_00033998	chr7	74233343	74233767	425	-	chr19	23257984	23331424	6231	+		-503.49	-0.6986	-1	0.63	0.49	0.64	1.53	1.77	0.57
cis_mRNA_overlap	0	Lnc-CompleteIn-mRNAIntron	n385216	MTCONS_00066926	chr7	74233343	74233767	425	-	chr7	74225225	74268229	1582	-	*		0.9469	1	0.63	0.49	0.64	0.41	0.28	0.49
cis_mRNA_overlap	0	mRNA	n345034	MTCONS_00072593	chr9	36422448	36485775	6515	-	chr9	36336217	36487396	5231	-	*		-0.9201	-0.866	0.08	0.07	0.07	0.39	1.05	1.48
tran	NA	NA	n385737	MTCONS_00021822	chrX	3744677	3761898	1498	-	chr14	102791829	102810753	5226	+		-1104.12	0.9979	1	3.11	2.3	3.81	0.58	0.34	0.84
tran	NA	NA	n385737	MTCONS_00021820	chrX	3744677	3761898	1498	-	chr14	102783531	102810753	5199	+		-1152	-0.928	-1	3.11	2.3	3.81	0.18	0.75	0.11
cis_mRNA_overlap	0	Lnc-CompleteIn-mRNAIntron	n341503	MTCONS_00068491	chr8	54811569	54821517	2239	+	chr8	54792567	54871863	1534	+	*		-0.9972	-0.866	0.14	0.2	0.2	1.2	0.1	0
cis_mRNA_up10k	91	NA	n385315	MTCONS_00066059	chr7	155434168	155437113	2946	-	chr7	155437203	155574179	10358	+	*		0.9992	1	1.53	2.09	1.45	1.7	2.91	1.46
cis_mRNA_overlap	0	mRNA	n346238	MTCONS_00002090	chr1	143916238	144094428	463	+	chr1	143913728	144055455	2945	+		-262.57	-0.9915	-1	4.27	3.7	3.49	0.84	1.03	1.15
tran	NA	NA	n408895	MTCONS_00045359	chrX	74960373	74962914	2542	-	chr21	38445479	38575408	6952	+		-1209.71	-1	-1	2.08	0	2.06	63.33	78.9	63.6
tran	NA	NA	n377415	MTCONS_00041019	chr22	20850211	20853786	497	+	chr2	69684995	69870977	20536	-		-441.4	0.9058	1	0.3	0.13	0.08	1.88	1.83	1.75
cis_mRNA_overlap	0	mRNA	LTCONS_00062508	MTCONS_00062509	chr6	37599399	37609970	10349	-	chr6	37599399	37666095	10963	-		-6494.44	0.9988	1	0.27	0.66	0.94	0.69	1.1	1.45
cis_mRNA_dw20k	13922	NA	n338652	MTCONS_00036311	chr19	40804388	40805930	1543	-	chr19	40819851	40854302	9201	-	*		0.9359	0.866	0.36	0.22	0.31	0.29	0.18	0.29
tran	NA	NA	n325682	MTCONS_00074579	chr13	78259455	78264443	376	-	chrX	75392518	75414076	17491	+		-220.22	-0.987	-1	1.38	1.89	2.8	5.93	5.5	5.07
tran	NA	NA	n378207	MTCONS_00037006	chr2	7053758	7058798	579	-	chr19	58201202	58220617	13301	-		-558.1	0.9392	1	0	0.17	0.14	4.35	5.99	5.18
cis_mRNA_dw20k	6775	NA	n339841	MTCONS_00032383	chr18	34366800	34408805	975	-	chr18	33877674	34360026	5428	+	*		-0.9704	-1	3.64	4.11	2.77	1.86	1.76	2.6
cis_mRNA_dw20k	6775	NA	n339841	MTCONS_00032382	chr18	34366800	34408805	975	-	chr18	33877674	34360026	4864	+	*		-0.9961	-1	3.64	4.11	2.77	2.68	2.17	3.36
cis_mRNA_up10k	440	NA	LTCONS_00011009	MTCONS_00009660	chr10	25012718	25013669	952	.	chr10	24872538	25012279	7254	-	*		-0.9726	-1	0.26	0.14	0.1	5.28	6.15	6.89
tran	1884496	NA	LTCONS_00071259	MTCONS_00072888	chr9	88420855	88433628	739	+	chr9	86451613	86536360	4778	-		-359.53	0.8527	1	0	0.25	0.59	1	1.81	1.87
tran	15800535	NA	n384068	MTCONS_00047712	chr22	17082801	17095998	1573	+	chr22	32896532	33454377	15152	-		-1603.29	-0.941	-1	0	0.05	0.11	5.15	5.06	4.49
tran	NA	NA	LTCONS_00029129	MTCONS_00016974	chr17	33895109	33901376	893	+	chr12	3918027	3982294	4757	-		-732.85	-0.8469	-0.866	15.53	12.73	14.04	1.23	1.35	1.23
tran	NA	NA	LTCONS_00029129	MTCONS_00032674	chr17	33895109	33901376	893	+	chr18	77794346	77830757	3161	+		-577	-0.884	-0.866	15.53	12.73	14.04	7.23	9.01	9.01
tran	61956	NA	n377902	MTCONS_00035599	chr19	12305830	12318512	495	+	chr19	12380467	12405714	4813	-		-251.98	-0.8489	-1	0.26	0.2	0.42	0.93	1.46	0.77
cis_mRNA_overlap	0	mRNA	n335503	MTCONS_00016670	chr12	123011790	123012372	478	+	chr12	123011756	123112317	8357	+	*		-0.8552	-1	0.16	0.29	0	0.77	0.55	0.78
tran	NA	NA	n374538	MTCONS_00072720	chr15	21365491	21366478	343	+	chr9	67323534	67340194	5721	-		-209.44	0.9093	1	0.8	0.1	1.13	3.46	2.62	5.4
tran	NA	NA	n333362	MTCONS_00010695	chr6	3110289	3111244	956	+	chr10	105623521	105678045	8971	-		-748.7	0.9014	1	2.02	2.4	2.49	2.12	2.32	2.61
tran	NA	NA	n333362	MTCONS_00032674	chr6	3110289	3111244	956	+	chr18	77794346	77830757	3161	+		-842.21	0.9836	0.866	2.02	2.4	2.49	7.23	9.01	9.01
tran	NA	NA	n325299	MTCONS_00074579	chr2	60431234	60433655	2005	-	chrX	75392518	75414076	17491	+		-2751.42	0.9649	1	2.36	2.22	1.83	5.93	5.5	5.07
tran	NA	NA	n324464	MTCONS_00074579	chr6	82579722	82580593	520	-	chrX	75392518	75414076	17491	+		-376.93	-0.9549	-1	0	0.2	0.26	5.93	5.5	5.07
cis_mRNA_overlap	0	Lnc-CompleteIn-mRNAIntron	n383698	MTCONS_00041565	chr2	110339915	110342057	2143	-	chr2	110309462	110371783	2742	-	*		0.7074	0.866	0.03	0.19	0.03	0.55	1.37	1.15
cis_mRNA_overlap	0	Lnc-CompleteIn-mRNAIntron	n383698	MTCONS_00041562	chr2	110339915	110342057	2143	-	chr2	110300281	110371783	5074	-	*		0.8874	0.866	0.03	0.19	0.03	0.9	1.29	1.08
tran	NA	NA	n383698	MTCONS_00036294	chr2	110339915	110342057	2143	-	chr19	40528842	40562147	24857	-		-2085.42	0.9401	0.866	0.03	0.19	0.03	6.91	8.47	7.45
cis_mRNA_overlap	0	mRNA	n410565	MTCONS_00051522	chr3	132276982	132441303	7788	-	chr3	132276713	132379140	4761	-		-2383.37	-0.9911	-1	1.38	1.02	1.13	0	0.39	0.32
tran	1428564	NA	n386307	MTCONS_00047445	chr22	20383731	20398694	2359	-	chr22	21827257	21871792	2404	-		-1445.07	0.9988	1	2.74	2.12	0	7.76	7.37	5.57
tran	1428564	NA	n386307	MTCONS_00047443	chr22	20383731	20398694	2359	-	chr22	21827257	21871780	3677	-		-1706.47	-0.6847	-1	2.74	2.12	0	1.84	3.15	3.17
cis_mRNA_up10k	6661	NA	n385516	MTCONS_00072629	chr9	37910721	37912138	1418	-	chr9	37887305	37904061	1941	-	*		-0.9961	-1	3.1	2.8	2.43	8.5	8.93	9.32
cis_mRNA_up10k	113	NA	n379294	MTCONS_00049231	chr3	112021325	112051638	1097	+	chr3	112051750	112087729	8452	+	*		0.8115	1	0.27	0.49	0.43	0	0.06	0.01
cis_mRNA_overlap	0	mRNA	LTCONS_00033569	MTCONS_00033571	chr19	9434448	9457687	7977	+	chr19	9434448	9493293	3220	+	*		-0.9964	-1	1.28	1.25	1.08	0.28	0.51	1.27
tran	NA	NA	n372506	MTCONS_00072661	chr11	34714000	34723562	627	+	chr9	40770255	40789853	5594	-		-400.13	-0.982	-1	8.06	4.18	7.73	2.05	2.71	2.23
tran	NA	NA	n372506	MTCONS_00036294	chr11	34714000	34723562	627	+	chr19	40528842	40562147	24857	-		-833.49	-0.9635	-1	8.06	4.18	7.73	6.91	8.47	7.45
cis_mRNA_overlap	0	Lnc-CompleteIn-mRNAExon	n341245	MTCONS_00066703	chr7	47994962	47996911	1948	-	chr7	47994874	48019266	10405	-		-987.54	0.9986	1	0	0.51	0.72	7.11	8.45	8.88
cis_mRNA_up10k	421	NA	n373296	MTCONS_00016505	chr12	112277507	112279765	1410	-	chr12	112280185	112331416	1636	+	*		-0.7897	-1	6.85	8.24	7.83	5.27	2.6	4.99
tran	203735080	NA	n325347	MTCONS_00039983	chr2	34756150	34801134	1109	+	chr2	238536213	238694823	8109	+		-1188.61	0.9316	1	4.23	6.22	5.28	1.72	3.49	3.26
cis_mRNA_overlap	0	CompleteIn-LncIntron	n377748	MTCONS_00018568	chr12	121633051	122226808	1385	-	chr12	121675133	121734556	4837	-	*		0.9707	1	0.22	0.29	0.24	0	0.12	0.06

cis_mRNA_dw20k	71	NA	n408333	MTCONS_00041080	chr2	73461364	73480150	1883	+	chr2	73480220	73511652	9021	-	*	1	1	0.17	0.17	0.62	0.14	0.14	0.17
cis_mRNA_overlap	0	Lnc-AntiOverlap-mRNA	LTCONS_00060931	MTCONS_00063147	chr6	114292659	114380952	2180	+	chr6	114376325	114663480	3597	-	*	0.8899	1	0.23	0.39	0	0.62	2.51	0.25
cis_mRNA_dw20k	17320	NA	n377371	MTCONS_00047851	chr22	39688993	39691568	1778	+	chr22	39708887	39715821	2491	-	*	-0.927	-1	0.16	0.19	0.12	1.65	0.88	1.88
tran	NA	NA	n385791	MTCONS_00047711	chrX	91354536	91360178	2006	-	chr22	32896532	33448559	15401	-	-3394.33	0.6636	0.866	0.02	0.06	0.02	2.72	2.94	1.9
cis_mRNA_up10k	1489	NA	n324717	MTCONS_00046404	chr22	19994110	20007143	1857	-	chr22	20008631	20055472	4110	+	*	-0.9878	-1	1.15	1.27	1.09	0.97	0.79	1.13
tran	NA	NA	n345589	MTCONS_00042922	chr3	52341571	52344151	691	+	chr20	2854142	3028899	10710	+	-625.34	-0.946	-1	0.31	0	0.23	0.54	0.94	0.76
tran	NA	NA	n385663	MTCONS_00032984	chr9	103115343	103156148	1956	+	chr18	45346666	45457071	23239	-	-2494.76	0.9916	1	0.36	0.62	0.33	2.93	3.26	2.83
cis_mRNA_overlap	0	Lnc-AntiOverlap-mRNA	LTCONS_00034772	MTCONS_00036693	chr19	51161757	51164921	2728	+	chr19	51163129	51223153	9110	-	-4158.2	0.9785	1	0	0.16	0.05	0.9	1.91	1.4
tran	NA	NA	n366364	MTCONS_00043564	chr3	184429982	184433265	672	+	chr20	44486220	44515128	8833	+	-672.87	0.9471	1	0.93	1.6	2.6	0.68	0.9	1
tran	NA	NA	n324679	MTCONS_00060502	chr22	30689344	30696387	473	+	chr6	56954828	56979689	14699	+	-564.71	0.8981	1	0.46	1.17	1.42	7.59	7.86	8.39
cis_mRNA_overlap	0	mRNA	LTCONS_00059422	MTCONS_00059430	chr6	3038543	3065264	3270	+	chr6	3064087	3084826	2348	+	*	0.9935	1	0.09	0.79	0.11	1.26	1.86	1.35
tran	NA	NA	n326173	MTCONS_00033686	chr1	232911758	232922346	1008	+	chr19	11998621	12032411	17275	+	-960.02	0.9985	1	7.26	9.6	8.96	1.66	3.92	3.42
tran	NA	NA	n326173	MTCONS_00039983	chr1	232911758	232922346	1008	+	chr2	238536213	238694823	8109	+	-590.84	0.9891	1	7.26	9.6	8.96	1.72	3.49	3.26
tran	NA	NA	n326173	MTCONS_00033685	chr1	232911758	232922346	1008	+	chr19	11998607	12032411	17331	+	-960.02	-0.9968	-1	7.26	9.6	8.96	4.11	2.39	2.73
tran	NA	NA	n384856	MTCONS_00063866	chr6	29894106	29897502	2516	+	chr6_dbb_hap3	1203642	1207050	1590	+	-1168.45	-0.9901	-1	0.2	0.25	0.21	18.36	0.43	12.42
tran	NA	NA	n377178	MTCONS_00017231	chr21	44232380	44237997	1172	+	chr12	14917326	14924065	5525	-	-1116.21	0.9497	1	0.27	0.35	0.32	0.86	1.61	1.12
cis_mRNA_dw20k	12791	NA	n341634	MTCONS_00070492	chr8	144638999	144640423	1425	-	chr8	144653213	144660563	5041	-	*	0.6577	0.866	0.02	0.02	0	0.93	1.75	0.72
cis_mRNA_dw20k	33	NA	n338721	MTCONS_00036504	chr19	46271645	46272943	1299	+	chr19	46272975	46296060	6532	-	*	-0.9713	-0.866	0.05	0.05	0.02	3.3	3.03	4.12
cis_mRNA_overlap	0	mRNA	n409199	MTCONS_00051502	chr3	129366635	129407575	5631	-	chr3	129366630	129514363	6277	-	-2551.14	-0.9607	-1	0.07	0.1	0	2.73	1.8	3.5
cis_mRNA_overlap	0	mRNA	n409199	MTCONS_00051503	chr3	129366635	129407575	5631	-	chr3	129366630	129612439	6193	-	-2544.59	-0.9993	-1	0.07	0.1	0	9.53	8.98	10.63
cis_mRNA_overlap	0	mRNA	n407958	MTCONS_00038868	chr2	128848782	128953249	10932	+	chr2	128848754	129002860	8052	+	-4015.68	-0.9991	-1	3.11	2.19	2.63	5.13	5.81	5.46
tran	NA	NA	n342622	MTCONS_00012008	chr4	146298061	146299109	1021	+	chr11	63448922	63527363	4860	+	-483.58	0.8329	1	0.04	0.06	0	3.71	5.78	3.38
tran	NA	NA	n342622	MTCONS_00012009	chr4	146298061	146299109	1021	+	chr11	63448922	63527363	2586	+	-477.74	0.8043	1	0.04	0.06	0	0.21	0.53	0.18
cis_mRNA_overlap	0	mRNA	n406602	MTCONS_00071788	chr9	128509617	128729655	2632	+	chr9	128508464	128729859	4118	+	-1149.65	0.8918	1	3.1	11.28	0.51	5.16	7.11	1.44
cis_mRNA_overlap	0	Lnc-CompleteIn-mRNAIntron	n342150	MTCONS_00063441	chr6	146216172	146218558	2386	-	chr6	146205856	146285534	7297	-	*	-0.7763	-1	0.72	0.69	0.63	2.64	3.98	4.03
tran	NA	NA	n341779	MTCONS_00064303	chr9	125578331	125579531	1201	-	chr7	6770022	6793507	4538	+	-784.53	0.8799	1	5.33	5.55	4.52	8.08	8.93	7.63
cis_mRNA_overlap	0	Lnc-AntiOverlap-mRNA	n387676	MTCONS_00070067	chr8	94752339	94753047	709	+	chr8	94740413	94753252	8515	-	*	0.739	0.866	0	0.09	0.09	4.18	5.05	4.45
tran	NA	NA	n325348	MTCONS_00005688	chr2	34462359	34713911	567	+	chr1	114258385	114300735	4864	-	-408.9	-0.75	-1	0.07	0	0.26	3.91	4.59	3.86
cis_mRNA_overlap	0	mRNA	n324003	MTCONS_00071526	chr9	108252918	108406897	1313	+	chr9	108210308	108315359	8283	+	*	-0.993	-1	0.03	0.05	0	13.03	12.07	13.98
cis_mRNA_dw20k	17354	NA	n342655	MTCONS_00030144	chr17	414737	418180	3444	-	chr17	435533	618096	2781	-	*	0.8122	1	0.12	0.37	0.03	3.15	3.18	3.01
cis_mRNA_overlap	0	Lnc-CompleteIn-mRNAIntron	n338032	MTCONS_00047711	chr22	33256006	33256421	415	-	chr22	32896532	33448559	15401	-	*	0.9914	1	0.28	0.34	0.16	2.72	2.94	1.9
tran	NA	NA	n386062	MTCONS_00070791	chr14	27791206	28142425	498	-	chr9	20658090	21002254	12800	+	-270.5	-0.822	-0.866	0.08	0.08	0	2.44	2.4	2.47
tran	NA	NA	n324495	MTCONS_00075262	chr6	53713506	53719779	850	-	chrX	153686623	153704519	11497	+	-728.42	0.9271	1	0.04	0.08	0.11	3.35	4.52	4.6
cis_mRNA_dw20k	11476	NA	n409109	MTCONS_00033499	chr19	7694671	7696510	429	+	chr19	7660788	7683196	4391	+	*	-0.9988	-1	2.36	0.81	1.44	2.28	3.26	2.82
cis_mRNA_dw20k	11476	NA	n409109	MTCONS_00033500	chr19	7694671	7696510	429	+	chr19	7660788	7683196	4137	+	*	0.9991	1	2.36	0.81	1.44	5.38	3.67	4.43
cis_mRNA_dw20k	3252	NA	n378779	MTCONS_00047445	chr22	21823045	21824006	496	+	chr22	21827257	21871792	2404	-	*	0.8574	1	0.68	0.23	0	7.76	7.37	5.57
cis_mRNA_dw20k	3252	NA	n378779	MTCONS_00047443	chr22	21823045	21824006	496	+	chr22	21827257	21871780	3677	-	*	-0.9474	-1	0.68	0.23	0	1.84	3.15	3.17
tran	NA	NA	n405665	MTCONS_00017912	chr7	35129456	35147346	3914	-	chr12	63952662	64062424	11449	-	-1520.76	0.9988	1	1.7	2.43	2.55	2.01	2.92	3.01
cis_mRNA_overlap	0	mRNA	n410504	MTCONS_00011817	chr11	57479995	57586652	6251	+	chr11	57479995	57510883	3285	+	*	-0.6425	-1	22.59	20.06	22.39	1.01	2.43	2.27
cis_mRNA_overlap	0	Lnc-AntiOverlap-mRNA	n411602	MTCONS_00029974	chr17	76103483	76107880	3188	-	chr17	75954761	76105096	10491	+	*	-0.9253	-1	0.05	0.07	0.09	1.05	0.95	0.36
tran	NA	NA	n323836	MTCONS_00067725	chrX	73417021	73428635	654	-	chr7	148766716	148787869	3158	-	-457.88	0.8489	1	0.45	0.52	0.25	2.25	2.68	2.11
tran	NA	NA	n366822	MTCONS_00017772	chr4	31351906	31353347	602	+	chr12	56214649	56223225	3578	-	-425.43	0.9671	1	0.3	0.42	0.11	4.85	5.95	4.15
cis_mRNA_dw20k	153	NA	n411756	MTCONS_00063229	chr6	127587983	127609705	2271	+	chr6	127609857	127653215	2306	-	*	0.7051	1	7.15	3.43	7	0.69	0	0.16
cis_mRNA_dw20k	11068	NA	n410586	MTCONS_00041120	chr2	74682150	74688018	2112	+	chr2	74699085	74710421	5373	-	*	0.9836	1	2.52	3.61	3.87	2.19	2.58	2.8
cis_mRNA_up10k	6126	NA	n381037	MTCONS_00003963	chr1	1586823	1590469	335	-	chr1	1570328	1580698	2728	-	*	-0.9482	-1	3.83	1.96	2.61	0	1.01	0.98
cis_mRNA_dw20k	3193	NA	n341223	MTCONS_00066562	chr7	36359490	36360088	599	+	chr7	36363280	36406878	4088	-	*	0.9071	1	0.18	0.39	0.66	0.52	0.53	1.03
cis_mRNA_dw20k	15720	NA	n409410	MTCONS_00065299	chr7	99245813	99277621	1986	-	chr7	99214214	99230094	4743	+	*	-0.8914	-1	0.11	0.05	0	0.07	0.13	0.59
tran	NA	NA	n326082	MTCONS_00039982	chr10	6700684	6707314	499	+	chr2	238536213	238694823	8202	+	-412.23	0.9948	1	0.22	0.19	0.08	8.48	8.35	8.06
cis_mRNA_overlap	0	Lnc-CompleteIn-mRNAIntron	n381381	MTCONS_00007684	chr10	1136095	1138119	2025	+	chr10	1102764	1180847	7236	+	*	-0.7559	-0.866	0.25	0.36	0.25	0.11	0.08	0.09
cis_mRNA_overlap	0	Lnc-CompleteIn-mRNAIntron	n381381	MTCONS_00007683	chr10	1136095	1138119	2025	+	chr10	1095059	1180847	7831	+	*	0.9857	0.866	0.25	0.36	0.25	1.25	1.57	1.18
cis_mRNA_overlap	0	mRNA	n407398	MTCONS_00059411	chr6	2988201	2991405	2809	+	chr6	2988201	3023034	1970	+	*	-0.6542	-1	0.99	0.93	1.29	0.77	1.79	0.73
tran	7514831	NA	LTCONS_00010375	MTCONS_00008663	chr10	81451258	81586350	1153	-	chr10	89101180	89131137	11860	+	-1365.25	0.982	0.866	0.63	0	0.63	2.47	1.97	2.37
tran	7514831	NA	LTCONS_00010375	MTCONS_00008664	chr10	81451258	81586350	1153	-	chr10	89101180	89131137	10665	+	-1365.25	-0.9895	-0.866	0.63	0	0.63	0	0.45	0.07
cis_mRNA_dw20k	5668	NA	LTCONS_00042804	MTCONS_00041037	chr2	70370117	70371350	1234	.	chr2	70377017	70418151	2478	-	*	-0.8359	-1	0.8	0.75	1.2	0.66	1.35	0.27
cis_mRNA_dw20k	5668	NA	LTCONS_00042804	MTCONS_00041039	chr2	70370117	70371350	1234	.	chr2	70377017	70418170	2598	-	*	0.9172	1	0.8	0.75	1.2	0.46	0	0.94
tran	NA	NA	n367022	MTCONS_00064836	chr4	129504154	129522555	482	-	chr7	55954858	56008496	3227	+	-592.27	-0.9774	-1	0.08	0.07	0.14	6.55	6.76	6.15
tran	NA	NA	n367022	MTCONS_00034245	chr4	129504154	129522555	482	-	chr19	37096218	37128259	11934	+	-605.24	0.7825	1	0.08	0.07	0.14	0.92	0.83	0.95
cis_mRNA_up10k	115	NA	n369985	MTCONS_00065151	chr7	86953598	86974837	1699	-	chr7	86974951	87023016	2596	+	*	-0.8085	-0.866	0.29	0.23	0.29	0.09	0.58	0.38
cis_mRNA_up10k	8432	NA	LTCONS_00060037	MTCONS_00060038	chr6	30882041	30883274	999	+	chr6	30891705	30896237	3402	+	*	0.9815	1	3.08	6.38	9.57	0	1.24	1.83
tran	201204915	NA	n365365	MTCONS_00039539	chr2	2886700	2898250	464	-	chr2	204103164	204171345	11508	+	-537.57	0.9886	1	0.49	0.36	0.29	0.99	0.69	0.61
tran	NA	NA	n365365	MTCONS_00075262	chr2	2886700	2898250	464	-	chrX	153686623	153704519	11497	+	-392.16	-0.9568	-1	0.49	0.36	0.29	3.35	4.52	4.6
tran	NA	NA	n379304	MTCONS_00021157	chr4	68566998	68588223	1445	+	chr14	50778997	50801593	9871	+	-1133.65	-0.9933	-1	0.26	0.95	1.38	2.94	2.59	2.26
tran	NA	NA	n325731	MTCONS_00066948	chr13	49536817	49538736	787	+	chr7	75162587	75268569	8101	-	-676.93	0.9222	1	0.21	0.27	0.32	19.24	19.91	22.78
cis_mRNA_up10k	1717	NA	n344963	MTCONS_00065448	chr7	104622581	104652888	5285	-	chr7	104654604	104756825	8989	+	*	0.9793	1	0.64	0.73	0.49	3.18	3.55	2.88

cis_mRNA_overlap	0	mRNA	n411728	MTCONS_00017252	chr12	16701306	16761148	3767	-	chr12	16698067	16763417	7285	-		-1361.36	0.9994	1	1.02	13.3	0.23	0.24	2.45	0
tran	1399410	NA	n384091	MTCONS_00047445	chr22	20383731	20427848	2113	-	chr22	21827257	21871792	2404	-		-1388.28	0.8498	1	0.42	0.15	0.02	7.76	7.37	5.57
tran	1399410	NA	n384091	MTCONS_00047443	chr22	20383731	20427848	2113	-	chr22	21827257	21871780	3677	-		-1590.94	-0.952	-1	0.42	0.15	0.02	1.84	3.15	3.17
cis_mRNA_dw20k	9421	NA	n340863	MTCONS_00031779	chr17	73872453	73875621	3169	+	chr17	73885041	73893054	3168	-	*		0.8785	1	0.02	0.01	0	1.89	0.89	0.86
tran	599548	NA	n408017	MTCONS_00066942	chr7	74306894	74322330	1220	+	chr7	74921877	74988366	1808	-		-641.85	0.8563	1	0.09	0.39	0.08	5.02	5.8	4.1
cis_mRNA_overlap	0	mRNA	n337463	MTCONS_00016685	chr12	123465703	123466268	270	+	chr12	123459264	123467462	4048	+		-252.01	0.6934	0.866	0.48	0.59	0.15	0.36	0.37	0.36
cis_mRNA_dw20k	18828	NA	n380700	MTCONS_00016892	chr12	133180601	133182850	921	-	chr12	133135899	133161774	4985	+	*		-0.8557	-1	0.04	0.14	0	0.57	0.48	0.87
cis_mRNA_up10k	174	NA	n381167	MTCONS_00001694	chr1	95628775	95699538	2447	-	chr1	95699711	95714855	5383	+	*		-0.973	-1	1.04	1.23	1.09	1.87	1.7	1.79
tran	NA	NA	n381167	MTCONS_00052301	chr1	95628775	95699538	2447	-	chr4	53227	166058	11726	+		-2432.31	0.9999	1	1.04	1.23	1.09	12.85	15.72	13.56
cis_mRNA_overlap	0	mRNA	n346356	MTCONS_00031600	chr17	62774792	62793418	4276	-	chr17	62773976	62833309	6100	-		-3436.89	-0.9088	-1	0.38	0.89	0.73	0.6	0.47	0.56
cis_mRNA_overlap	0	mRNA	n346356	MTCONS_00031596	chr17	62774792	62793418	4276	-	chr17	62745750	62833302	8207	-		-3371.03	0.8236	1	0.38	0.89	0.73	0.27	0.83	0.35
cis_mRNA_dw20k	13197	NA	n363822	MTCONS_00000745	chr1	29459862	29461446	1166	+	chr1	29241056	29446666	6169	+	*		1	1	1.68	1.35	1.76	12.99	10.13	13.69
cis_mRNA_dw20k	13197	NA	n363822	MTCONS_00000738	chr1	29459862	29461446	1166	+	chr1	29213598	29446666	6002	+	*		-0.9183	-1	1.68	1.35	1.76	3.41	5.41	0.87
cis_mRNA_dw20k	13543	NA	n366565	MTCONS_00052179	chr3	197374634	197381275	4887	-	chr3	197394817	197476612	10290	-	*		0.6214	1	1.11	0.89	1.08	0.51	0.09	0.1
cis_mRNA_up10k	1301	NA	LTCONS_00031997	MTCONS_00030272	chr17	4168574	4170448	1875	.	chr17	4074203	4167274	5289	-	*		0.9975	0.866	0.51	1.1	0.46	1.13	1.37	1.13
cis_mRNA_up10k	360	NA	LTCONS_00031997	MTCONS_00030268	chr17	4168574	4170448	1875	.	chr17	4061027	4168215	14872	-	*		-0.899	-1	0.51	1.1	0.46	0.09	0.08	0.1
tran	NA	NA	n377403	MTCONS_00025856	chr22	46271879	46283628	805	-	chr16	3355385	3370044	7080	+		-765.26	-0.9839	-1	0.13	0.12	0	1.44	1.97	3.51
cis_mRNA_dw20k	3237	NA	n369916	MTCONS_00066233	chr7	5653430	5656062	635	-	chr7	5659298	5821361	6224	-	*		-0.6355	-0.866	0.46	0.26	0.26	1.76	1.86	2.33
cis_mRNA_dw20k	3237	NA	n369916	MTCONS_00066232	chr7	5653430	5656062	635	-	chr7	5659298	5821361	6053	-	*		0.6719	0.866	0.46	0.26	0.26	3.06	3.04	2.97
cis_mRNA_dw20k	6834	NA	n410630	MTCONS_00004686	chr1	35441300	35444307	1887	-	chr1	35451140	35497334	5720	-	*		-0.9867	-1	0.22	0.73	0.44	0.29	0.22	0.27
tran	NA	NA	n407519	MTCONS_00069240	chr6	29694378	29716826	689	-	chr8	155778	182318	8373	-		-433.75	0.8383	1	0	0.21	0.04	0	0.17	0.12
tran	24980893	NA	n339695	MTCONS_00052179	chr3	172413336	172413925	590	-	chr3	197394817	197476612	10290	-		-605.44	-0.8612	-1	0.06	0.25	0.15	0.51	0.09	0.1
cis_mRNA_overlap	0	Lnc-AntiOverlap-mRNA	LTCONS_00015662	MTCONS_00017591	chr12	49523295	49580616	211	+	chr12	49521565	49582861	1546	-		-475	-0.6819	-1	0.42	0	0.37	0.45	0.92	0.86
tran	NA	NA	n344977	MTCONS_00074663	chr8	103698638	103742333	645	+	chrX	100353189	100425597	10070	+		-691.07	0.9741	1	0.28	0.45	0.19	0.96	1.83	0.83
tran	NA	NA	n379182	MTCONS_00014144	chr3	62249550	62304567	733	-	chr11	66448399	66496836	12763	-		-783.86	0.9011	0.866	1.3	1.69	1	2.42	2.66	2.42
tran	NA	NA	n379182	MTCONS_00014146	chr3	62249550	62304567	733	-	chr11	66448399	66497268	13286	-		-783.86	-0.9368	-1	1.3	1.69	1	3.01	2.92	3.02
tran	NA	NA	n406199	MTCONS_00055677	chr6	58246159	58287724	770	-	chr5	21459589	21503711	2614	+		-472.86	-0.8398	-1	1.6	0.26	1.23	1.13	1.46	1.39
tran	NA	NA	n406199	MTCONS_00055679	chr6	58246159	58287724	770	-	chr5	21459589	21589481	5313	+		-510.89	-0.9988	-1	1.6	0.26	1.23	0.4	0.53	0.43
tran	NA	NA	n371252	MTCONS_00076657	chr9	33503824	33509337	349	-	chrY	4898547	4981558	13244	+		-333.61	0.7261	1	0.91	0	0.14	1.57	0.93	1.47
cis_mRNA_dw20k	10178	NA	n408035	MTCONS_00071845	chr9	130882972	130890712	1493	-	chr9	130853465	130872795	5263	+	*		-0.9922	-1	10.8	9.88	10.56	1.66	2.97	2.15
cis_mRNA_up10k	9330	NA	n410641	MTCONS_00043944	chr20	62289163	62330051	5772	+	chr20	62339380	62370513	4399	+	*		0.9079	1	1.56	1.59	1.25	5.04	5.29	4.78
cis_mRNA_up10k	9318	NA	n410641	MTCONS_00043939	chr20	62289163	62330051	5772	+	chr20	62339368	62370513	4481	+	*		0.8284	1	1.56	1.59	1.25	0.07	0.19	0
tran	NA	NA	n325442	MTCONS_00001490	chr2	11415092	11423213	1061	-	chr1	78245281	78506196	5315	+		-811.67	0.7619	1	0.19	0.18	0.41	0.7	0	1.01
tran	NA	NA	n368642	MTCONS_00008781	chr6	94416801	94456471	4603	+	chr10	97515409	97637023	12236	+		-3415.4	0.866	0.866	0.06	0.02	0.04	0.09	0	0.09
tran	NA	NA	n325702	MTCONS_00008780	chr13	66844520	66852109	611	+	chr10	97515409	97637023	12392	+		-671.85	-0.8462	-1	0.35	0.29	0.27	0	0.01	0.04
tran	NA	NA	n324400	MTCONS_00052869	chr6	126540032	126565078	583	+	chr4	56914805	57200064	10656	+		-525.72	-0.9849	-1	0.7	0.95	0.74	19.73	16.31	18.62
cis_mRNA_overlap	0	Lnc-CompleteIn-mRNAIntron	n342313	MTCONS_00022261	chr14	36263047	36265529	2014	-	chr14	36007514	36278519	8136	-	*		-0.7336	-1	0.26	0.16	0.52	1.02	4.11	0.92
tran	NA	NA	n375566	MTCONS_00028040	chr17	18315789	18317623	484	+	chr16	70010190	70099871	6665	-		-606.49	0.9441	1	0.3	0.43	0.35	3.74	5.39	4.86
tran	NA	NA	n375566	MTCONS_00000611	chr17	18315789	18317623	484	+	chr1	24968184	24999772	5383	+		-579.03	0.9999	1	0.3	0.43	0.35	1.94	3.41	2.52
cis_mRNA_up10k	86	NA	n385605	MTCONS_00072873	chr9	84304628	84370473	1381	+	chr9	84198597	84304543	4217	-	*		0.9935	1	0.11	0.25	0.03	10	12.21	8.09
cis_mRNA_overlap	0	Lnc-AntiOverlap-mRNA	LTCONS_00070747	MTCONS_00072323	chr9	9389313	9442239	231	+	chr9	8314246	9414824	8434	-	*		0.8354	1	5.85	3.61	3.07	0.58	0.52	0.37
cis_mRNA_overlap	0	mRNA	n377745	MTCONS_00004234	chr1	16888818	16931427	4295	-	chr1	16888816	16940154	7419	-		-2178.21	-0.9695	-1	0.69	0.86	0.96	5.07	3	0.07
cis_mRNA_overlap	0	mRNA	n377745	MTCONS_00004233	chr1	16888818	16931427	4295	-	chr1	16888816	16940154	6606	-		-2160.94	0.9003	1	0.69	0.86	0.96	0	0.91	3.95
cis_mRNA_dw20k	12145	NA	n340427	MTCONS_00055777	chr5	35723089	35746487	1751	-	chr5	35617989	35710945	4486	+	*		-0.9515	-0.866	0.04	0.09	0.04	1.03	0.42	1.31
cis_mRNA_dw20k	12145	NA	n340427	MTCONS_00055775	chr5	35723089	35746487	1751	-	chr5	35617982	35710945	4508	+	*		0.9996	0.866	0.04	0.09	0.04	0.35	0.94	0.33
cis_mRNA_overlap	0	Lnc-AntiOverlap-mRNA	LTCONS_00054345	MTCONS_00052869	chr4	57198019	57211157	9096	-	chr4	56914805	57200064	10656	+	*		-0.8936	-1	10.98	12.72	12.26	19.73	16.31	18.62
tran	57031962	NA	LTCONS_00054345	MTCONS_00052301	chr4	57198019	57211157	9096	-	chr4	53227	166058	11726	+		-9427.13	0.8528	1	10.98	12.72	12.26	12.85	15.72	13.56
cis_mRNA_overlap	0	mRNA	n411139	MTCONS_00065415	chr7	102004308	102067129	2911	+	chr7	102004308	102067211	2895	+		-1852.17	-0.9804	-1	2.68	3.03	2.58	3.4	2.23	3.42
tran	NA	NA	n345068	MTCONS_00072875	chrX	64295724	64628987	2946	+	chr9	84248380	84304524	2608	-		-1599.67	-0.7165	-0.866	0.03	0.03	0.07	7.35	6.58	6.28
tran	NA	NA	LTCONS_00065888	MTCONS_00016474	chr7	145812302	145943603	19610	+	chr12	111051832	111068271	2328	+		-2268.08	-0.9942	-1	2.75	2.99	2.43	1.08	0.6	1.52
tran	NA	NA	n367913	MTCONS_00052870	chr5	71870013	71876804	591	-	chr4	56914919	57200064	10678	+		-407.51	0.9706	1	0.18	0.29	0	0.92	2.25	0
tran	NA	NA	LTCONS_00072235	MTCONS_00075293	chr9	14430	30786	14155	-	chrX	155251143	155255371	1784	+		-1404.58	0.9106	1	1.89	0.94	1.71	3.66	0	1.57
tran	NA	NA	LTCONS_00072235	MTCONS_00003821	chr9	14430	30786	14155	-	chr1	14050	29439	3053	-		-2282.84	-0.9965	-1	1.89	0.94	1.71	0.47	0.66	0.49
cis_mRNA_overlap	0	Lnc-CompleteIn-mRNAIntron	n381464	MTCONS_00010147	chr10	65005260	65007599	2340	-	chr10	64926164	65281636	9235	-	*		-0.9288	-1	0.92	1.19	1.21	0.45	0.29	0.17
cis_mRNA_overlap	0	Lnc-CompleteIn-mRNAIntron	n326269	MTCONS_00003079	chr1	201522903	201524552	775	+	chr1	201508186	201796103	13957	+	*		0.9986	1	0.05	0.08	0	0.39	0.58	0
cis_mRNA_overlap	0	Lnc-CompleteIn-mRNAIntron	n326269	MTCONS_00003080	chr1	201522903	201524552	775	+	chr1	201508188	201796103	13964	+	*		0.9928	1	0.05	0.08	0	2.09	2.13	1.98
cis_mRNA_overlap	0	mRNA	n409093	MTCONS_00015709	chr12	50794592	50855272	1727	+	chr12	50794592	50873788	6354	+		-755.62	-0.9991	-1	0	0.12	0.17	0.07	0.03	0.01
tran	NA	NA	n344848	MTCONS_00030178	chr5	126190650	126192242	957	+	chr17	1472556	1532487	9223	-		-890.35	0.8911	1	0.12	0.3	0.37	0.48	0.69	1.17
cis_mRNA_dw20k	244	NA	LTCONS_00073702	MTCONS_00073708	chr9	139296372	139300963	2989	-	chr9	139301206	139305054	1265	-	*		-0.8281	-1	3.09	3.34	3.41	2.2	2.13	1.92
tran	NA	NA	n326200	MTCONS_00052869	chr1	223954929	224006568	1530	+	chr4	56914805	57200064	10656	+		-841.25	-0.9046	-1	4.81	6.34	5.9	19.73	16.31	18.62
cis_mRNA_up10k	2393	NA	n369921	MTCONS_00064311	chr7	7590643	7603904	1300	-	chr7	7606296	7652468	9535	+	*		0.9381	1	0.17	0	0.19	2.77	2.33	3.11
tran	NA	NA	n376412	MTCONS_00052301	chr19	35339987	35351417	807	-	chr4	53227	166058	11726	+		-503.9	-0.9323	-1	1.23	0.83	1	12.85	15.72	13.56
cis_mRNA_dw20k	1546	NA	LTCONS_00000259	MTCONS_00000258	chr1	7831311	7841499	4043	+	chr1	7764871	7829766	5397	+	*		0.7699	1	1.6	1.64	1.38	9.79	17.55	7.59
cis_mRNA_dw20k	1546	NA	LTCONS_00000259	MTCONS_00000256	chr1	7831311	7841499	4043	+	chr1	7764855	7829766	5444	+	*		-0.8288	-1	1.6	1.64	1.38	8.01	2.17	10.66
cis_mRNA_up10k	1379	NA	n346443	MTCONS_00048087	chr22	51022806	51035501	2577	+	chr22	51006098	51021428	5612	-	*		-0.6446	-1	0.49	0.16	0.45	1.24	2.06	2
cis_mRNA_dw20k	656	NA	n370880	MTCONS_00071845	chr9	130873450	130880972	3801	-	chr9	130853465	130872795	5263	+	*		0.8286	1	0.82	1.43	1.38	1.66	2.97	2.15

tran	NA	NA	n345134	MTCONS_00035590	chr11	12093738	12094453	713	+	chr19	12242097	12267577	9341	-		-677.01	0.956	1	0.15	0.29	0.36	0.74	1.45	1.48
cis_mRNA_up10k	5596	NA	n407976	MTCONS_00047226	chr22	50609160	50618724	3018	+	chr22	50624319	50656144	12945	+	*		0.9508	1	0	0.14	0.29	0.87	1.16	1.25
cis_mRNA_overlap	0	Lnc-AntiOverlap-mRNA	n378951	MTCONS_00067458	chr7	124569975	124784620	3252	+	chr7	124461305	124570037	5083	-	*		0.866	1	0.02	0.03	0.06	0.08	0.23	0.29
tran	NA	NA	n375451	MTCONS_00068694	chr17	12453285	12540504	2605	+	chr8	79578282	79624487	15452	+		-1308.51	-0.642	-1	0.42	0.17	0.16	3.66	3.69	3.86
tran	NA	NA	n387365	MTCONS_00046828	chr5	81436374	81437039	666	+	chr22	30999407	31027721	7200	+		-1328.11	0.9952	1	4.6	5.69	5.51	1.69	2.32	2.28
tran	NA	NA	n370462	MTCONS_00054075	chr8	38721756	38725271	1173	-	chr4	8363185	8431038	7806	-		-748.99	-0.6449	-0.866	0.39	0.33	0.33	0.11	0.43	0.17
cis_mRNA_overlap	0	Lnc-AntiOverlap-mRNA	n377596	MTCONS_00061930	chr6	4125589	4129180	3592	+	chr6	4115927	4135831	1605	-	*		-0.998	-1	0.03	0	0.01	0	0.18	0.11
tran	NA	NA	n385437	MTCONS_00052301	chr8	122966847	123139423	822	-	chr4	53227	166058	11726	+		-948.27	0.983	1	0.04	0.33	0.16	12.85	15.72	13.56
tran	NA	NA	n326039	MTCONS_00067475	chr10	26977035	26982389	880	+	chr7	127010302	127032875	5908	-		-461.14	-0.9978	-1	0.09	0.37	0.26	0.78	0.6	0.66
tran	1588452	NA	n383681	MTCONS_00041393	chr2	96472813	96492729	3115	-	chr2	98081180	98160993	24447	-		-2021.62	-0.9959	-1	0.08	0.17	0.13	1.57	0.94	1.17
tran	1588452	NA	n383681	MTCONS_00041394	chr2	96472813	96492729	3115	-	chr2	98081180	98206428	25798	-		-2021.62	0.9983	1	0.08	0.17	0.13	2.83	3.88	3.36
cis_mRNA_dw20k	19	NA	n371284	MTCONS_00071845	chr9	130872813	130880957	3254	-	chr9	130853465	130872795	5263	+	*		0.8754	1	0.89	1.12	1.08	1.66	2.97	2.15
cis_mRNA_overlap	0	Lnc-CompleteIn-mRNAExon	LTCONS_00064775	MTCONS_00064777	chr7	48128355	48145906	3148	+	chr7	48128355	48148727	5326	+		-2159.55	0.953	1	3.38	4.81	4.26	2.57	5.07	4.84
cis_mRNA_overlap	0	Lnc-CompleteIn-mRNAExon	LTCONS_00064775	MTCONS_00064780	chr7	48128355	48145906	3148	+	chr7	48128355	48148727	5211	+		-2157.19	0.9513	1	3.38	4.81	4.26	0.96	1.54	1.49
tran	NA	NA	n375694	MTCONS_00053148	chr17	26557602	26561264	3088	-	chr4	91048528	92018810	14378	+		-3480.03	1	1	0.06	0	0.04	0.99	0.93	0.97
cis_mRNA_overlap	0	Lnc-AntiOverlap-mRNA	n380573	MTCONS_00016685	chr12	123413548	123466196	6482	-	chr12	123459264	123467462	4048	+		-6775.52	0.9997	0.866	0.64	1	0.63	0.36	0.37	0.36
tran	NA	NA	n381240	MTCONS_00052869	chr1	145514196	145515899	1011	-	chr4	56914805	57200064	10656	+		-1228.38	-0.9052	-1	1.09	1.99	1.73	19.73	16.31	18.62
cis_mRNA_overlap	0	Lnc-CompleteIn-mRNAIntron	n385698	MTCONS_00071890	chr9	131247780	131250064	2285	+	chr9	131218285	131263722	5539	+	*		0.8438	1	0.27	0.23	0.3	0.04	0	0.9
cis_mRNA_overlap	0	AntiCompleteIn-mRNAIntron	n346215	MTCONS_00061403	chr6	158719303	158724405	487	-	chr6	158653184	158932914	12054	+	*		-0.8107	-1	0.23	0.07	0	2.74	2.92	4.12
cis_mRNA_overlap	0	AntiCompleteIn-mRNAIntron	n346215	MTCONS_00061406	chr6	158719303	158724405	487	-	chr6	158703267	158932914	12048	+	*		-0.8998	-1	0.23	0.07	0	6.18	6.46	7.12
cis_mRNA_overlap	0	Lnc-CompleteIn-mRNAIntron	n384667	MTCONS_00057959	chr5	69428359	69430659	2301	-	chr5	69416727	69497870	3769	-	*		0.9971	1	0.48	0.66	0.81	0.16	0.28	0.41
cis_mRNA_overlap	0	Lnc-CompleteIn-mRNAIntron	n384667	MTCONS_00057960	chr5	69428359	69430659	2301	-	chr5	69416727	69521622	3590	-	*		-0.946	-1	0.48	0.66	0.81	1.21	0.65	0.55
tran	135026925	NA	n381256	MTCONS_00004232	chr1	151967007	152015250	4109	+	chr1	16888816	16940083	7373	-		-2148.47	0.9091	0.866	0.07	0.12	0.12	3.93	6.2	5.25
tran	135026854	NA	n381256	MTCONS_00004234	chr1	151967007	152015250	4109	+	chr1	16888816	16940154	7419	-		-2124.28	-0.8124	-0.866	0.07	0.12	0.12	5.07	3	0.07
tran	135026854	NA	n381256	MTCONS_00004233	chr1	151967007	152015250	4109	+	chr1	16888816	16940154	6606	-		-2077.26	0.6782	0.866	0.07	0.12	0.12	0	0.91	3.95
tran	NA	NA	n365887	MTCONS_00052301	chr3	87138430	87147851	4299	+	chr4	53227	166058	11726	+		-2164.74	0.8479	1	26.54	31.31	30.09	12.85	15.72	13.56
cis_mRNA_overlap	0	mRNA	n405981	MTCONS_00052109	chr3	195686792	195717150	2491	-	chr3	195684854	195717166	4032	-		-1621.08	-0.9999	-1	0.62	0.87	0.91	1.88	1.04	0.89
tran	NA	NA	n333187	MTCONS_00063948	chr6	29795825	29796595	544	+	chr6_mcf_hap5	2701469	2704679	1429	-		-434.56	-0.8664	-1	0.14	0.06	0.09	1.3	5.31	1.92
tran	NA	NA	n334975	MTCONS_00041342	chr6	481970	482301	332	+	chr2	96487344	96658969	20870	-		-224.66	0.9942	1	34.04	38.11	42.28	2.25	2.77	3.55
cis_mRNA_overlap	0	Lnc-CompleteIn-mRNAIntron	n381471	MTCONS_00010316	chr10	79317424	79320001	2578	-	chr10	78629359	79397577	12156	-	*		0.7856	1	0.12	0.22	0.21	0.57	1.15	0.74
cis_mRNA_overlap	0	Lnc-CompleteIn-mRNAIntron	n381471	MTCONS_00010321	chr10	79317424	79320001	2578	-	chr10	78644635	79397577	6115	-	*		-0.9959	-0.866	0.12	0.22	0.21	0.6	0.59	0.59
cis_mRNA_dw20k	16604	NA	n407587	MTCONS_00035589	chr19	12203078	12225494	3769	+	chr19	12242097	12267577	9332	-	*		-0.9887	-1	0.57	0.73	0.31	1.84	1.63	2.04
cis_mRNA_dw20k	5262	NA	n345059	MTCONS_00073277	chr9	114967942	114975454	1131	-	chr9	114980715	115059964	7078	-	*		-0.8103	-0.866	0.06	0.08	0.06	6.11	4.65	5.25
tran	NA	NA	n380928	MTCONS_00076657	chr12	43373057	43390289	552	-	chrY	4898547	4981558	13244	+		-376.14	0.6205	0.866	0.16	0.06	0.06	1.57	0.93	1.47
tran	NA	NA	n338841	MTCONS_00014144	chr2	54684398	54856341	2164	+	chr11	66448399	66496836	12763	-		-1477.99	-0.9773	-0.866	29.74	26.28	30.73	2.42	2.66	2.42
tran	NA	NA	n338841	MTCONS_00014146	chr2	54684398	54856341	2164	+	chr11	66448399	66497268	13286	-		-1479.02	0.9925	1	29.74	26.28	30.73	3.01	2.92	3.02
cis_mRNA_overlap	0	mRNA	n382927	MTCONS_00027553	chr16	21458834	21468260	2543	-	chr16	21448105	21531804	14985	-	*		0.9986	1	0.78	1.13	0.87	2.77	4.17	3.06
cis_mRNA_dw20k	15424	NA	n345981	MTCONS_00031600	chr17	62745938	62758553	2139	-	chr17	62773976	62833309	6100	-	*		-0.9045	-1	0.53	0.76	0.69	0.6	0.47	0.56
cis_mRNA_overlap	0	Lnc-CompleteIn-mRNAExon	n345981	MTCONS_00031596	chr17	62745938	62758553	2139	-	chr17	62745750	62833302	8207	-		-1362.63	0.8177	1	0.53	0.76	0.69	0.27	0.83	0.35
tran	NA	NA	n382512	MTCONS_00002444	chr15	43432319	43433465	1147	-	chr1	155278539	155290457	1359	+		-702.2	0.9109	1	0.03	0.01	0	88.27	78.59	55.17
tran	NA	NA	n382512	MTCONS_00002443	chr15	43432319	43433465	1147	-	chr1	155278539	155200457	1449	+		-731.9	0.7639	1	0.03	0.01	0	25.56	25.4	14.17
tran	NA	NA	n326341	MTCONS_00036204	chr1	166999227	167010352	418	-	chr19	38120564	38147191	7291	-		-500.53	0.9605	1	0.54	1.88	1.09	3.37	3.84	3.44
tran	NA	NA	n376630	MTCONS_00053148	chr20	24911284	24913619	2156	-	chr4	91048528	92018810	14378	+		-1161.98	-0.7559	-0.866	0.41	0.48	0.48	0.99	0.93	0.97
tran	NA	NA	n384441	MTCONS_00076657	chr4	39640760	39653681	1050	+	chrY	4898547	4981558	13244	+		-916.92	0.8898	1	0.87	0.75	0.8	1.57	0.93	1.47
tran	NA	NA	n326115	MTCONS_00052870	chr10	2533995	2569817	320	+	chr4	56914919	57200064	10678	+		-184.75	0.9506	1	1.02	1.65	0.94	0.92	2.25	0
cis_mRNA_overlap	0	AntiCompleteIn-mRNAIntron	n325230	MTCONS_00041432	chr2	100191148	100234378	3115	+	chr2	100161762	100759037	10115	-	*		0.9193	1	0.24	0.39	0.22	0.59	0.85	0.34
cis_mRNA_overlap	0	Lnc-CompleteIn-mRNAIntron	n339200	MTCONS_00026400	chr16	47563101	47563827	494	+	chr16	47495015	47735448	8267	+	*		0.9968	0.866	0.08	0.14	0.14	2.44	3.35	3.27
tran	NA	NA	LTCONS_00023162	MTCONS_00069358	chr15	23393042	23681407	667	+	chr8	10958954	11060192	12778	-		-639.81	0.9199	1	2.74	2.78	6.02	1	2.1	3.72
cis_mRNA_overlap	0	mRNA	n386361	MTCONS_00054466	chr4	76648698	76649795	999	-	chr4	76567933	76649795	4391	-		-557.27	-0.7975	-1	1.58	1.67	1.93	13.02	10.97	10.58
cis_mRNA_overlap	0	mRNA	n386361	MTCONS_00054467	chr4	76648698	76649795	999	-	chr4	76567933	76649795	4490	-		-551.12	-0.9561	-1	1.58	1.67	1.93	3.75	3.5	3.27
tran	NA	NA	n325644	MTCONS_00052301	chr13	102193341	102195575	1947	+	chr4	53227	166058	11726	+		-945.44	0.7177	1	19.5	24.03	23.84	12.85	15.72	13.56
tran	NA	NA	LTCONS_00013840	MTCONS_00052870	chr11	58646457	58673199	5255	-	chr4	56914919	57200064	10678	+		-4110.56	-0.9975	-1	1.34	0.89	1.74	0.92	2.25	0
tran	NA	NA	LTCONS_00013840	MTCONS_00016474	chr11	58646457	58673199	5255	-	chr12	111051832	111068271	2328	+		-991.64	1	1	1.34	0.89	1.74	1.08	0.6	1.52
cis_mRNA_overlap	0	mRNA	LTCONS_00061053	MTCONS_00061054	chr6	127587732	127609855	8819	+	chr6	127587732	127609855	2983	+		-1371.39	0.979	1	1.83	2.23	1.99	1.45	1.92	1.55
tran	18882164	NA	n326498	MTCONS_00001552	chr1	103590357	103600943	418	+	chr1	84629892	84708194	8478	+		-180.21	0.9884	1	0.09	0.42	0.16	0.82	0.99	0.88
tran	NA	NA	n367738	MTCONS_00008381	chr5	45035301	45098749	820	+	chr10	70359450	70460551	14554	+		-674.12	-0.8511	-1	0.05	0.15	0	0.81	0.75	1.1
tran	NA	NA	n367738	MTCONS_00008382	chr5	45035301	45098749	820	+	chr10	70360234	70460551	13766	+		-674.12	0.9966	1	0.05	0.15	0	5.71	6.44	5.21

cis_mRNA_overlap	0	Lnc-CompleteIn-mRNAIntron	n324484	MTCONS_00060538	chr6	71161311	71165841	397	+	chr6	71123107	71270947	6303	+	*	0.9763	0.866	0.43	0.43	0.41	1.09	1.41	0
cis_mRNA_overlap	0	Lnc-CompleteIn-mRNAIntron	n324484	MTCONS_00060539	chr6	71161311	71165841	397	+	chr6	71123107	71270947	6254	+	*	-0.7146	-0.866	0.43	0.43	0.41	0.52	0	0.72
tran	NA	NA	n325608	MTCONS_00073450	chr14	100485068	100488842	610	+	chr9	130475494	130477939	1451	-	-536.92	0.9349	1	0.3	0.11	0.21	1.88	1.17	1.78
cis_mRNA_overlap	0	Lnc-AntiOverlap-mRNA	n407803	MTCONS_00027103	chr16	90106169	90114033	1349	-	chr16	90093690	90111416	3396	+	-1566.86	0.9989	1	0	0.03	0.06	1.32	1.78	2.17
cis_mRNA_overlap	0	Lnc-AntiOverlap-mRNA	n407803	MTCONS_00027102	chr16	90106169	90114033	1349	-	chr16	90092242	90111416	4899	+	*	0.9729	1	0	0.03	0.06	0.84	1.08	1.18
cis_mRNA_up10k	25	NA	n406664	MTCONS_00008471	chr10	74894282	74927853	1826	-	chr10	74927877	75002575	6155	+	*	0.6286	0.866	0.17	0.19	0.07	1.08	1.09	1.08
tran	NA	NA	n376479	MTCONS_00037544	chr19	13781870	13789989	773	-	chr2	27651458	27667164	3792	+	-496.62	0.9848	0.866	0.34	0.99	0.46	0.88	1.48	0.88
cis_mRNA_overlap	0	Lnc-AntiOverlap-mRNA	n377577	MTCONS_00061930	chr6	4131253	4134818	3566	+	chr6	4115927	4135831	1605	-	*	0.999	1	0.1	0.17	0.14	0	0.18	0.11
cis_mRNA_up10k	122	NA	n338274	MTCONS_00002418	chr1	155017693	155023641	1580	-	chr1	155023762	155035324	2964	+	*	-0.9349	-1	0	0.02	0.09	0.99	0.45	0
cis_mRNA_dw20k	16215	NA	n408346	MTCONS_00015936	chr12	57623356	57628718	2215	+	chr12	57522282	57607142	15650	+	*	0.9361	1	0.11	0.92	0.45	1.36	2.31	2.06
cis_mRNA_dw20k	16215	NA	n408346	MTCONS_00015939	chr12	57623356	57628718	2215	+	chr12	57546864	57607142	15612	+	*	0.8162	0.866	0.11	0.92	0.45	0.58	0.9	0.9
cis_mRNA_overlap	0	Lnc-AntiOverlap-mRNA	LTCONS_00055934	MTCONS_00057854	chr5	56204939	56213386	1723	+	chr5	56212638	56248020	8061	-	-1325.1	0.8185	1	0.15	0.51	0.61	0.07	0.11	0.24
tran	NA	NA	n385312	MTCONS_00049454	chr7	155022391	155023284	894	-	chr3	130612835	130727146	10239	+	-815.94	0.9469	1	0.61	1.35	1.01	1.22	1.47	1.43
tran	48493074	NA	LTCONS_00056074	MTCONS_00055678	chr5	70015606	70164996	16778	+	chr5	21459589	21522533	925	+	-553.5	-0.9887	-1	0.4	1.71	3.75	1.01	0.76	0.03
tran	517737	NA	LTCONS_00056074	MTCONS_00057959	chr5	70015606	70164996	16778	+	chr5	69416727	69497870	3769	-	-1922.39	0.9948	1	0.4	1.71	3.75	0.16	0.28	0.41
cis_mRNA_dw20k	16856	NA	n371303	MTCONS_00075490	chrX	20004935	20007897	628	-	chrX	20024752	20135114	4110	-	*	0.9693	1	0	0.17	0.07	5.44	7.08	6.47
tran	NA	NA	n324050	MTCONS_00052301	chr9	91506386	91524405	822	+	chr4	53227	166058	11726	+	-939.42	0.9631	1	3.23	4.25	3.73	12.85	15.72	13.56
cis_mRNA_dw20k	17316	NA	n342448	MTCONS_00038916	chr2	132291181	132300824	1128	-	chr2	132250386	132273866	8919	+	*	0.9989	1	0.06	0.17	0	3.6	4.17	3.23
cis_mRNA_dw20k	3650	NA	n410677	MTCONS_00076512	chrX	153059904	153063967	946	+	chrX	153067616	153096099	5276	-	*	-0.9669	-1	1.5	0.5	1.09	13.23	16.13	13.73
cis_mRNA_dw20k	3650	NA	n410677	MTCONS_00076511	chrX	153059904	153063967	946	+	chrX	153067616	153096099	3866	-	*	0.9451	1	1.5	0.5	1.09	14.2	8.57	13.66
cis_mRNA_dw20k	17705	NA	n341664	MTCONS_00072430	chr9	23500689	23672385	4805	-	chr9	23690089	23850763	4231	-	*	-0.8697	-1	0.67	0.79	0.84	72.38	68.88	58.29
cis_mRNA_overlap	0	Lnc-CompleteIn-mRNAIntron	n325435	MTCONS_00037317	chr2	11739038	11740905	1170	+	chr2	11622954	11783475	12886	+	*	0.9782	1	2.06	2.89	2.91	1.34	1.5	1.55
cis_mRNA_dw20k	15744	NA	n372861	MTCONS_00013503	chr11	18283800	18284297	404	-	chr11	18300040	18343799	5623	-	*	-0.9999	-1	0.09	0	0.08	1.06	1.27	1.08
cis_mRNA_dw20k	5476	NA	n340067	MTCONS_00008471	chr10	75008050	75036742	2842	+	chr10	74927877	75002575	6155	+	*	0.9774	0.866	0.2	0.38	0.24	1.08	1.09	1.08
cis_mRNA_dw20k	5476	NA	n340067	MTCONS_00008469	chr10	75008050	75036742	2842	+	chr10	74927877	75002575	3816	+	*	-0.9977	-1	0.2	0.38	0.24	1.01	0.59	0.89
tran	NA	NA	n369231	MTCONS_00041393	chr6	41349175	41350213	937	-	chr2	98081180	98160993	24447	-	-712.31	0.7787	0.866	0.17	0.13	0.17	1.57	0.94	1.17
tran	NA	NA	n369231	MTCONS_00041394	chr6	41349175	41350213	937	-	chr2	98081180	98206428	25798	-	-712.31	-0.8633	-0.866	0.17	0.13	0.17	2.83	3.88	3.36
cis_mRNA_dw20k	5289	NA	n342564	MTCONS_00052109	chr3	195676060	195679566	3507	-	chr3	195684854	195717166	4032	-	*	-0.6956	-1	1.3	1.34	1.65	1.88	1.04	0.89
tran	NA	NA	n406442	MTCONS_00000658	chr15	45803334	45848928	1927	+	chr1	26758868	26804903	12663	+	-1115.87	0.9438	0.866	0.02	0.03	0.02	1.49	2.56	1.85
tran	NA	NA	n406442	MTCONS_00000659	chr15	45803334	45848928	1927	+	chr1	26758868	26804903	8592	+	-1137.27	0.9936	0.866	0.02	0.03	0.02	0.68	1.25	0.6
cis_mRNA_overlap	0	mRNA	n345248	MTCONS_00022035	chr14	21911321	21924249	2257	-	chr14	21853281	21924404	8666	-	*	-0.9719	-1	0.24	0.22	0.3	3.28	4.26	2.14
cis_mRNA_up10k	5865	NA	n345248	MTCONS_00022033	chr14	21911321	21924249	2257	-	chr14	21853281	21905457	8557	-	*	-0.9852	-1	0.24	0.22	0.3	4.14	4.3	2.25
tran	NA	NA	n325867	MTCONS_00052872	chr10	122675049	122730203	943	-	chr4	57081966	57200064	10458	+	-550.61	0.7519	1	58.79	77.67	79.58	15.47	18.33	26.84
tran	NA	NA	n325867	MTCONS_00041342	chr10	122675049	122730203	943	-	chr2	96487344	96658969	20870	-	-1590.3	0.8497	1	58.79	77.67	79.58	2.25	2.77	3.55
tran	NA	NA	n325867	MTCONS_00041341	chr10	122675049	122730203	943	-	chr2	96487344	96549806	19081	-	-1590.3	-0.6028	-1	58.79	77.67	79.58	6.48	6.43	5.38
cis_mRNA_dw20k	18421	NA	LTCONS_00045965	MTCONS_00045292	chr21	34949869	34960620	2945	-	chr21	34915350	34931449	7968	+	*	0.8595	0.866	1.68	2.58	2.14	1.83	1.98	1.83
cis_mRNA_up10k	2892	NA	n337889	MTCONS_00000967	chr1	40938727	40940020	1294	+	chr1	40942911	40962620	3475	+	*	-0.9841	-1	0.43	0.34	0.29	0.53	0.91	1.31
cis_mRNA_up10k	2892	NA	n337889	MTCONS_00000968	chr1	40938727	40940020	1294	+	chr1	40942911	40962620	3452	+	*	-0.955	-1	0.43	0.34	0.29	0.33	0.52	0.83
tran	NA	NA	n383667	MTCONS_00052872	chr2	89065419	89079081	1803	+	chr4	57081966	57200064	10458	+	-1867.39	-0.975	-1	1.27	1.26	0.83	15.47	18.33	26.84
tran	7408264	NA	n383667	MTCONS_00041342	chr2	89065419	89079081	1803	+	chr2	96487344	96658969	20870	-	-971.4	-0.9254	-1	1.27	1.26	0.83	2.25	2.77	3.55
tran	7408264	NA	n383667	MTCONS_00041341	chr2	89065419	89079081	1803	+	chr2	96487344	96549806	19081	-	-971.4	0.9998	1	1.27	1.26	0.83	6.48	6.43	5.38
cis_mRNA_dw20k	3616	NA	n337634	MTCONS_00000066	chr1	995114	1001833	974	+	chr1	955460	991499	8883	+	*	-0.964	-1	1.85	2.08	1.43	6.72	6.5	8.79
cis_mRNA_dw20k	3616	NA	n337634	MTCONS_00000069	chr1	995114	1001833	974	+	chr1	969154	991499	8181	+	*	-1	-1	1.85	2.08	1.43	13.18	11.59	16.19
cis_mRNA_up10k	66	NA	LTCONS_00023004	MTCONS_00021916	chr14	105880755	105886121	4873	-	chr14	105886186	105937068	2863	+	*	0.998	1	1.33	1.35	1.22	9.06	9.17	8.66
tran	NA	NA	n387328	MTCONS_00052108	chr5	1568637	1579695	1026	-	chr3	195684854	195717166	5790	-	-609.69	-0.9994	-1	1.45	2.68	1.82	3.52	2.83	3.29
tran	NA	NA	n325485	MTCONS_00052872	chr2	2817043	2821170	574	-	chr4	57081966	57200064	10458	+	-556.25	0.8413	1	0.5	0.69	0.75	15.47	18.33	26.84
cis_mRNA_dw20k	15402	NA	n386068	MTCONS_00021295	chr14	62594747	62597232	2486	+	chr14	62279426	62579346	13406	+	*	-0.9996	-1	0.37	0	0.6	1.67	2.01	1.48
cis_mRNA_dw20k	15402	NA	n386068	MTCONS_00021294	chr14	62594747	62597232	2486	+	chr14	62279243	62579346	14469	+	*	0.8144	1	0.37	0	0.6	4.6	4.59	4.82
cis_mRNA_dw20k	5179	NA	n380550	MTCONS_00015240	chr12	8940870	8946932	809	+	chr12	8850493	8935692	5471	+	*	0.862	1	1.7	0.25	0.69	6.49	4.17	5.97
cis_mRNA_dw20k	113	NA	LTCONS_00073839	MTCONS_00071093	chr9	69852020	69852511	492	.	chr9	69844168	69851908	6749	+	*	0.8858	1	1.27	0.75	2.63	1.74	1.03	2.04
cis_mRNA_overlap	0	Lnc-AntiOverlap-mRNA	n378246	MTCONS_00041102	chr2	74612630	74619360	578	+	chr2	74579851	74619214	12873	-	*	0.8419	1	0.16	0	0.23	2.72	2.64	3.07
cis_mRNA_overlap	0	mRNA	n340920	MTCONS_00030051	chr17	79410850	79428992	5166	+	chr17	79373540	79433733	12320	+	-4681.18	0.9747	1	0.89	1.48	1.14	0.48	0.98	0.79
cis_mRNA_dw20k	6125	NA	n345078	MTCONS_00075464	chrX	18903153	18903852	700	-	chrX	18909976	19003200	6401	-	*	-0.9678	-1	0	0.05	0.14	0.7	0.48	0.32
tran	1297184	NA	LTCONS_00008242	MTCONS_00009929	chr10	47708809	47727808	586	+	chr10	46321034	46411626	3201	-	-311.96	-0.7622	-1	0.95	1.14	1.16	0.4	0.3	0.02
tran	1368820	NA	LTCONS_00008242	MTCONS_00009927	chr10	47708809	47727808	586	+	chr10	46321034	46339990	2246	-	-311.96	-0.7033	-1	0.95	1.14	1.16	0.74	0.63	0.13
cis_mRNA_overlap	0	Lnc-AntiOverlap-mRNA	n340855	MTCONS_00059713	chr6	13614343	13615387	1045	-	chr6	13574792	13614875	3970	+	-1069.8	0.8443	1	0.94	1.35	1.5	1.33	1.41	1.68
cis_mRNA_dw20k	2287	NA	n369509	MTCONS_00064216	chr7	2477398	2487484	2066	+	chr7	2443223	2475112	2973	+	*	0.9923	1	0.31	0.47	0.52	0.24	1.08	1.19
cis_mRNA_dw20k	2287	NA	n369509	MTCONS_00064218	chr7	2477398	2487484	2066	+	chr7	2443259	2475112	2951	+	*	-0.9028	-1	0.31	0.47	0.52	0.38	0.24	0
tran	NA	NA	n326479	MTCONS_00052399	chr1	113164435	113193924	704	-	chr4	2828574	2842823	8847	+	-698	0.9112	1	0.1	2.15	0.99	2.19	2.46	2.41
tran	NA	NA	n385992	MTCONS_00048727	chr10	75547343	75547998	656	+	chr3	48956233	49053477	15105	+	-590.62	-0.9904	-1	0.61	0.81	1.53	0.47	0.46	0.34
tran	NA	NA	n345581	MTCONS_00042372	chr3	5053037	5067376	907	-	chr2	203637784	203736841	8428	-	-508.21	-0.9563	-1	0.04	0.07	0.1	1.46	1.33	1.29
tran	3354814	NA	LTCONS_00002098	MTCONS_00005933	chr1	144166564	144219394	638	+	chr1	147574207	147610081	4489	-	-378.5	-0.9522	-0.866	6.91	14.4	9.25	0.08	0	0.08
tran	3782910	NA	LTCONS_00002098	MTCONS_00005954	chr1	144166564	144219394	638	+	chr1	148002303	148025848	4803	-	-375.86	-1	-1	6.91	14.4	9.25	1.19	0.97	1.12
tran	1084817	NA	LTCONS_00002098	MTCONS_00002114	chr1	144166564	144219394	638	+	chr1	145304210	145370255	11091	+	-390.08	0.9928	1	6.91	14.4	9.25	0.52	1.07	0.63

tran	3787384	NA	LTCONS_00002098	MTCONS_00005956	chr1	144166564	144219394	638	+	chr1	148006777	148025848	3003	-		-375.86	0.9697	1	6.91	14.4	9.25	12.58	18.82	15.88
cis_mRNA_overlap	0	mRNA	n381582	MTCONS_00011076	chr11	559477	560106	535	+	chr11	556469	564719	2618	+	*		0.9933	1	3.53	2.33	3.96	0.12	0	0.19
cis_mRNA_overlap	0	mRNA	n407441	MTCONS_00031597	chr17	62745780	62778117	2862	-	chr17	62745750	62833311	8616	-		-1995.71	-0.809	-1	0.61	0.5	1.09	1.92	1.98	1.9
tran	NA	NA	n380878	MTCONS_00000611	chr14	27305934	27402978	477	+	chr1	24968184	24999772	5383	+		-325.72	0.9999	1	0	0.21	0.08	1.94	3.41	2.52
tran	NA	NA	n366377	MTCONS_00025854	chr3	189846778	189847483	497	+	chr16	3355344	3370044	4078	+		-543.36	0.785	1	4.25	3.43	4.63	1.18	1.11	2.09
cis_mRNA_dw20k	13583	NA	n380381	MTCONS_00013887	chr11	61263912	61267548	554	+	chr11	61281130	61310455	6862	-	*		-0.8825	-0.866	0	0.06	0.06	0.17	0.08	0
tran	NA	NA	n323796	MTCONS_00052301	chrX	145698994	145699619	436	+	chr4	53227	166058	11726	+		-416.51	-0.9743	-1	2.47	1.77	2.46	12.85	15.72	13.56
cis_mRNA_overlap	0	mRNA	LTCONS_00076652	MTCONS_00076655	chrY	4868267	4880883	10535	+	chrY	4868267	5106271	7871	+	*		0.9687	1	6.17	1.49	7.59	2.78	1.04	4.25
cis_mRNA_overlap	0	mRNA	LTCONS_00076652	MTCONS_00076654	chrY	4868267	4880883	10535	+	chrY	4868267	4981558	12618	+	*		0.8658	1	6.17	1.49	7.59	4.85	4.11	6.56
tran	118199	NA	n386353	MTCONS_00052299	chr4	206389	249771	2605	+	chr4	53198	88191	2956	+		-847.16	0.852	0.866	2.04	1.54	0.99	0.57	0.52	0.52
cis_mRNA_up10k	147	NA	LTCONS_00021865	MTCONS_00021868	chr14	104601667	104602591	925	+	chr14	104602737	104649911	12164	+	*		-0.8971	-1	0.54	0.31	0.41	0.41	0.74	0.46
cis_mRNA_up10k	128	NA	LTCONS_00021865	MTCONS_00021866	chr14	104601667	104602591	925	+	chr14	104602718	104649911	9613	+	*		0.9871	1	0.54	0.31	0.41	0.69	0.45	0.52
tran	839917	NA	LTCONS_00027646	MTCONS_00027690	chr16	29435949	29436215	267	-	chr16	30276131	30346695	10819	-		-163.96	0.9848	1	1.49	1.91	2.11	0.35	0.68	0.98
cis_mRNA_overlap	0	mRNA	LTCONS_00067799	MTCONS_00067796	chr7	151105479	151108082	2231	-	chr7	151075540	151108082	8772	-		-2049.77	0.926	1	8.43	4.58	7.21	5.4	4.41	4.75
tran	NA	NA	n324605	MTCONS_00008780	chr6	5796738	5815452	412	+	chr10	97515409	97637023	12392	+		-263.75	0.9696	1	0	0.08	0.17	0	0.01	0.04
tran	NA	NA	n326363	MTCONS_00076654	chr1	156368458	156377044	649	-	chrY	4868267	4981558	12618	+		-545.66	-0.9795	-1	0.66	0.68	0.49	4.85	4.11	6.56
cis_mRNA_overlap	0	Lnc-AntiOverlap-mRNA	n323961	MTCONS_00071890	chr9	131232752	131249390	1846	-	chr9	131218285	131263722	5539	+	*		0.7473	1	0.05	0	0.07	0.04	0	0.9
cis_mRNA_overlap	0	mRNA	n385307	MTCONS_00065941	chr7	150097790	150109557	2581	+	chr7	150102113	150109813	4351	+		-1947.69	-0.9245	-0.866	0	0.13	0.08	0.13	0.11	0.11
cis_mRNA_overlap	0	mRNA	n385307	MTCONS_00065938	chr7	150097790	150109557	2581	+	chr7	150076398	150109813	3635	+		-1807.4	0.9381	1	0	0.13	0.08	0	0.59	0.18
tran	NA	NA	n333290	MTCONS_00076657	chr8	104927741	104933211	527	+	chrY	4898547	4981558	13244	+		-495.69	-0.9491	-1	0.72	2.63	1.58	1.57	0.93	1.47
tran	NA	NA	n325437	MTCONS_00076655	chr2	11456746	11459838	373	+	chrY	4868267	5106271	7871	+		-515.16	-0.9092	-1	35.49	41.58	35.16	2.78	1.04	4.25
tran	NA	NA	n325927	MTCONS_00054075	chr10	86943157	86947694	2029	-	chr4	8363185	8431038	7806	-		-1621.35	0.8595	1	0	0.03	0.02	0.11	0.43	0.17
cis_mRNA_dw20k	12367	NA	n338026	MTCONS_00024028	chr15	75660496	75661920	1425	+	chr15	75638984	75648130	5040	+	*		-0.9729	-1	0.66	0.56	1.09	0.92	1.21	0.49
cis_mRNA_dw20k	12367	NA	n338026	MTCONS_00024027	chr15	75660496	75661920	1425	+	chr15	75638984	75648130	4379	+	*		-0.9989	-1	0.66	0.56	1.09	0.86	0.9	0.62
cis_mRNA_dw20k	1620	NA	n370583	MTCONS_00069113	chr8	142208034	142209262	337	-	chr8	142151718	142206415	8077	+	*		0.9907	1	0.12	0.21	0.1	1.19	1.39	1.1
cis_mRNA_dw20k	17384	NA	n345046	MTCONS_00072431	chr9	23647973	23672706	2294	-	chr9	23690089	23850763	4143	-	*		-0.9379	-1	2.54	3.12	2.06	28.12	26.44	28.36
cis_mRNA_overlap	0	Lnc-CompleteIn-mRNAIntron	n382507	MTCONS_00023549	chr15	42100589	42102741	1608	+	chr15	42064664	42120056	9643	+	*		0.9901	1	0.22	0.35	0.44	0.88	1.26	1.41
cis_mRNA_up10k	249	NA	n378769	MTCONS_00006898	chr1	200993077	200996918	2497	+	chr1	200938461	200992829	9378	-	*		0.846	1	2.24	1.7	1.56	10.18	9.7	8.54
tran	NA	NA	n369892	MTCONS_00000710	chr7	158383260	158383964	556	+	chr1	28696093	28827167	6521	+		-476.28	-0.9751	-1	0.31	0.24	0.23	0.72	1.19	1.43
tran	3174688	NA	LTCONS_00073835	MTCONS_00071093	chr9	66668650	66669481	832	.	chr9	69844168	69851908	6749	+		-505.06	0.9143	1	0.52	0.13	1.29	1.74	1.03	2.04
cis_mRNA_dw20k	5490	NA	n341314	MTCONS_00067477	chr7	126999844	127004813	4970	-	chr7	127010302	127032875	6352	-	*		0.8192	0.866	0.53	0.72	0.86	0.25	0.25	0.37
cis_mRNA_dw20k	5490	NA	n341314	MTCONS_00067476	chr7	126999844	127004813	4970	-	chr7	127010302	127032875	5903	-	*		0.9133	1	0.53	0.72	0.86	0.39	0.9	0.91
tran	30568440	NA	n342388	MTCONS_00035590	chr19	42836016	42836379	364	+	chr19	12242097	12267577	9341	-		-520.01	-0.9994	-0.866	0.67	0.1	0.1	0.74	1.45	1.48
tran	30568440	NA	n342388	MTCONS_00035588	chr19	42836016	42836379	364	+	chr19	12242097	12267577	7026	-		-520.01	0.7608	0.866	0.67	0.1	0.1	1.78	1.28	1.61
tran	NA	NA	n342388	MTCONS_00068694	chr19	42836016	42836379	364	+	chr8	79578282	79624487	15452	+		-529.06	-0.6156	-0.866	0.67	0.1	0.1	3.66	3.69	3.86
tran	1088787	NA	n381222	MTCONS_00002114	chr1	144212643	144215424	709	+	chr1	145304210	145370255	11091	+		-438.52	0.9689	1	27.38	37.88	31.87	0.52	1.07	0.63
cis_mRNA_overlap	0	Lnc-AntiOverlap-mRNA	n407804	MTCONS_00027104	chr16	90109310	90114191	1391	-	chr16	90096312	90111416	4405	+		-2338.23	0.9955	1	0	0.26	0.17	0.98	1.75	1.55
cis_mRNA_overlap	0	mRNA	n387128	MTCONS_00041342	chr2	96592962	96657541	1951	-	chr2	96487344	96658969	20870	-		-1105.15	-1	-1	2.02	1.71	1.25	2.25	2.77	3.55
cis_mRNA_overlap	0	mRNA	n345612	MTCONS_00052066	chr3	194192715	194206447	1834	-	chr3	194123403	194207754	9002	-	*		0.9853	0.866	0.11	0.09	0	1.1	1.1	0.69
cis_mRNA_up10k	3916	NA	LTCONS_00069947	MTCONS_00069944	chr8	80946421	81086273	7122	-	chr8	80829722	80942506	2062	-	*		0.754	1	1.85	2.51	2.71	0.74	0.79	1.17
tran	NA	NA	LTCONS_00073876	MTCONS_00073892	chrUn_gl000219	77630	99629	2030	-	chrUn_gl000241	16000	34973	964	-		-344.06	0.8793	1	0.14	1.13	4.49	0.11	1.74	2.55
tran	NA	NA	LTCONS_00073876	MTCONS_00073893	chrUn_gl000219	77630	99629	2030	-	chrUn_gl000241	16000	36863	837	-		-307.94	0.8644	1	0.14	1.13	4.49	0	4.89	7.03
cis_mRNA_overlap	0	Lnc-CompleteIn-mRNAIntron	n340270	MTCONS_00053698	chr4	169763543	169765898	2356	+	chr4	169552768	169849608	4979	+	*		0.9845	1	0.12	0.19	0.14	0.64	2.2	1.33
cis_mRNA_overlap	0	Lnc-AntiOverlap-mRNA	LTCONS_00015661	MTCONS_00017590	chr12	49521569	49579494	874	+	chr12	49521565	49582861	1630	-		-1859.7	0.9992	1	0.61	0.67	0.51	15.14	18.9	7.8
tran	NA	NA	n325081	MTCONS_00052870	chr20	5360638	5374332	1542	-	chr4	56914919	57200064	10678	+		-1453.84	-0.9055	-1	1.69	1.31	3.21	0.92	2.25	0
cis_mRNA_up10k	7689	NA	n340930	MTCONS_00011084	chr11	565660	568420	1965	-	chr11	576108	612518	6188	+	*		-0.9975	-1	0.31	0	0.28	1.17	1.67	1.18
cis_mRNA_dw20k	15901	NA	n345015	MTCONS_00068792	chr8	96088313	96095554	1701	+	chr8	96037221	96072413	3385	+	*		-0.993	-1	0.12	0.15	0.08	0.71	0.37	1.01
tran	48926573	NA	n387357	MTCONS_00055679	chr5	70516053	70555172	660	-	chr5	21459589	21589481	5313	+		-424.94	-0.7465	-1	15.41	2.63	3.99	0.4	0.53	0.43
cis_mRNA_dw20k	6712	NA	n4864	MTCONS_00000710	chr1	28833878	28834083	206	+	chr1	28696093	28827167	6521	+	*		-0.8696	-1	288.14	270.73	201.9	0.72	1.19	1.43
tran	NA	NA	n371707	MTCONS_00053148	chr10	85926986	85931832	1855	-	chr4	91048528	92018810	14378	+		-2076.25	-0.9286	-1	0.21	0.3	0.27	0.99	0.93	0.97
tran	NA	NA	n378631	MTCONS_00055883	chr1	143744199	143744717	307	-	chr5	49962220	50142704	8405	+		-272.68	0.9215	1	1.37	0.72	0.82	1.02	0.71	0.87
tran	NA	NA	n324701	MTCONS_00052301	chr22	24007804	24009061	568	-	chr4	53227	166058	11726	+		-319.36	0.7884	1	18.38	22.95	22.24	12.85	15.72	13.56
cis_mRNA_overlap	0	mRNA	n385798	MTCONS_00074678	chrX	100740408	100788446	3205	+	chrX	100740359	100771939	3421	+		-1504.73	-0.7472	-1	0.25	0.31	0.33	1.52	1.41	0.24
tran	NA	NA	n325250	MTCONS_00000612	chr2	75000190	75005481	763	-	chr1	24968184	24999772	7455	+		-385.85	-0.7418	-1	0.85	0.84	0.75	1.92	2.59	2.79
cis_mRNA_up10k	295	NA	LTCONS_00055397	MTCONS_00054496	chr4	78740873	78741433	561	.	chr4	78634506	78740579	8935	-	*		-0.9847	-1	0	0.12	0.06	1.31	0.82	0.99
tran	NA	NA	n343078	MTCONS_00063070	chr11	117162062	117162865	804	+	chr6	109782987	109804477	6409	-		-664.8	-0.6865	-1	2.98	2.96	2.65	0	0.3	0.37
tran	NA	NA	LTCONS_00035172	MTCONS_00003818	chr19	60022	73583	9134	-	chr1	14050	29381	9122	-		-5341.84	0.9295	1	0.6	1.1	1.99	2.41	2.88	3.09
tran	NA	NA	LTCONS_00035172	MTCONS_00003819	chr19	60022	73583	9134	-	chr1	14050	29437	7973	-		-4934.8	0.9665	1	0.6	1.1	1.99	5.14	7.76	9.61
cis_mRNA_dw20k	4764	NA	n385078	MTCONS_00064295	chr7	6703605	6714110	1410	+	chr7	6676932	6698842	8192	+	*		-0.9993	-1	0.91	1.98	1.16	6.89	6.18	6.7
cis_mRNA_overlap	0	Lnc-AntiOverlap-mRNA	n335504	MTCONS_00010147	chr10	65281169	65370072	675	+	chr10	64926164	65281636	9235	-	*		0.9006	1	0.06	0.05	0	0.45	0.29	0.17
cis_mRNA_overlap	0	CompleteIn-LncIntron	n381248	MTCONS_00005957	chr1	147930761	148176401	476	-	chr1	148016192	148026022	1241	-	*		0.8533	1	0.31	1.18	0	0.95	0.97	0.89
tran	NA	NA	n324772	MTCONS_00030180	chr21	45837996	45841351	674	+	chr17	1472556	1533118	9141	-		-653.95	-0.9917	-1	0.08	1.15	0.82	0.47	0.15	0.21
cis_mRNA_overlap	0	Lnc-AntiOverlap-mRNA	n379572	MTCONS_00069120	chr8	142400039	142402674	1380	-	chr8	142402119	142442685	2982	+	*		0.9316	1	0.08	0.09	0.16	1.25	2.36	3.62

cis_mRNA_overlap	0	Lnc-AntiOverlap-mRNA	LTCONS_00065583	MTCONS_00067457	chr7	124569927	124785288	2938	+	chr7	124461305	124570037	5214	-	*		-0.9874	-1	0	0.17	0.15	0.82	0.6	0.66
cis_mRNA_dw20k	9975	NA	n379061	MTCONS_00008471	chr10	75012549	75014107	762	+	chr10	74927877	75002575	6155	+	*		-0.7936	-0.866	1.36	1.02	1.9	1.08	1.09	1.08
cis_mRNA_overlap	0	AntiCompleteIn-mRNAIntron	n346107	MTCONS_00047902	chr22	42665997	42674031	3073	+	chr22	42555898	42739561	7507	-	*		0.9673	1	0.29	0.18	0	5.32	4.82	1.72
cis_mRNA_overlap	0	AntiCompleteIn-mRNAIntron	n346107	MTCONS_00047901	chr22	42665997	42674031	3073	+	chr22	42555898	42680076	7674	-	*		-0.982	-1	0.29	0.18	0	2.36	2.62	3.63
cis_mRNA_overlap	0	Lnc-CompleteIn-mRNAIntron	n345302	MTCONS_00023649	chr15	47829760	47841473	568	+	chr15	47473246	48071485	14335	+	*		0.6708	0.866	0.58	1.18	0.41	0.1	0.1	0.01
cis_mRNA_overlap	0	Lnc-CompleteIn-mRNAIntron	n345302	MTCONS_00023651	chr15	47829760	47841473	568	+	chr15	47476077	48071485	11465	+	*		0.8949	1	0.58	1.18	0.41	1.34	1.62	0.86
cis_mRNA_overlap	0	mRNA	n385915	MTCONS_00003818	chr1	14407	29370	3517	-	chr1	14050	29381	9122	-		-2772.42	0.954	1	1.14	3.07	5.69	2.41	2.88	3.09
cis_mRNA_overlap	0	mRNA	n385915	MTCONS_00003819	chr1	14407	29370	3517	-	chr1	14050	29437	7973	-	*		0.9827	1	1.14	3.07	5.69	5.14	7.76	9.61
cis_mRNA_dw20k	4730	NA	n374036	MTCONS_00021295	chr14	62584075	62595131	863	+	chr14	62279426	62579346	13406	+	*		0.7741	0.866	0.18	0.18	0.15	1.67	2.01	1.48
cis_mRNA_dw20k	4730	NA	n374036	MTCONS_00021293	chr14	62584075	62595131	863	+	chr14	62279243	62579346	13802	+	*		-0.9967	-0.866	0.18	0.18	0.15	1.36	1.47	2.58
cis_mRNA_dw20k	4730	NA	n374036	MTCONS_00021294	chr14	62584075	62595131	863	+	chr14	62279243	62579346	14469	+	*		-0.9993	-0.866	0.18	0.18	0.15	4.6	4.59	4.82
cis_mRNA_up10k	7217	NA	n346478	MTCONS_00074247	chrX	47415406	47418281	1768	-	chrX	47425497	47431424	2878	+	*		0.9963	1	0.18	0.2	0.17	0.86	1.22	0.61
cis_mRNA_up10k	5572	NA	LTCONS_00066770	MTCONS_00066765	chr7	56107807	56119284	3098	-	chr7	56077796	56102236	3179	-	*		-0.9744	-1	1.07	1.48	1.72	1.57	0.66	0.49
cis_mRNA_overlap	0	mRNA	LTCONS_00066770	MTCONS_00066768	chr7	56107807	56119284	3098	-	chr7	56077796	56119284	3358	-	*		0.9144	1	1.07	1.48	1.72	0.73	0.78	0.92
cis_mRNA_up10k	1056	NA	n384198	MTCONS_00048109	chr22	51222225	51227600	673	+	chr22	51203433	51221170	5239	-	*		0.6287	1	0	0.08	0.81	0.52	0.77	0.79
tran	NA	NA	LTCONS_00042049	MTCONS_00010010	chr2	170542083	170550931	3537	-	chr10	50641266	50818344	30532	-		-1931.22	-0.9212	-1	2.33	2.73	1.84	1.11	0.69	1.21
tran	NA	NA	LTCONS_00042049	MTCONS_00008781	chr2	170542083	170550931	3537	-	chr10	97515409	97637023	12236	+		-3672.01	-0.8354	-0.866	2.33	2.73	1.84	0.09	0	0.09
tran	NA	NA	LTCONS_00042049	MTCONS_00010011	chr2	170542083	170550931	3537	-	chr10	50641266	50818350	30643	-		-1931.22	0.9878	1	2.33	2.73	1.84	1.27	1.58	1.05
cis_mRNA_dw20k	10325	NA	n375785	MTCONS_00030272	chr17	4063018	4063879	808	+	chr17	4074203	4167274	5289	-	*		0.9016	0.866	0.36	0.83	0	1.13	1.37	1.13
cis_mRNA_overlap	0	AntiCompleteIn-mRNAExon	n375785	MTCONS_00030268	chr17	4063018	4063879	808	+	chr17	4061027	4168215	14872	-		-1782.66	-0.9971	-1	0.36	0.83	0	0.09	0.08	0.1
tran	NA	NA	n325063	MTCONS_00052869	chr20	12172543	12172944	280	-	chr4	56914805	57200064	10656	+		-328.23	-0.9879	-1	0	0.26	0.12	19.73	16.31	18.62
cis_mRNA_overlap	0	mRNA	n342576	MTCONS_00054093	chr4	10452511	10459032	2623	-	chr4	10440363	10459050	8306	-	*		0.8427	1	1.68	0	2.19	0.24	0	0.92
cis_mRNA_overlap	0	Lnc-CompleteIn-mRNAExon	n387658	MTCONS_00065941	chr7	150102377	150109557	3244	+	chr7	150102113	150109813	4351	+		-2479.51	0.6896	0.866	3.61	2.59	3.36	0.13	0.11	0.11
cis_mRNA_overlap	0	mRNA	n387658	MTCONS_00065938	chr7	150102377	150109557	3244	+	chr7	150076398	150109813	3635	+		-2209.84	-0.9979	-1	3.61	2.59	3.36	0	0.59	0.18
cis_mRNA_up10k	980	NA	n381852	MTCONS_00015446	chr12	26097790	26110985	2084	-	chr12	26111964	26225956	5785	+	*		-0.6411	-1	0.01	0.34	0	1.52	1.25	2.97
cis_mRNA_overlap	0	mRNA	n341313	MTCONS_00067477	chr7	126986844	127013619	1478	-	chr7	127010302	127032875	6352	-	*		-0.7107	-0.866	0.2	0.12	0.09	0.25	0.25	0.37
cis_mRNA_overlap	0	mRNA	n341313	MTCONS_00067476	chr7	126986844	127013619	1478	-	chr7	127010302	127032875	5903	-	*		-0.9689	-1	0.2	0.12	0.09	0.39	0.9	0.91
tran	4826475	NA	LTCONS_00000543	MTCONS_00004234	chr1	21766628	21792645	552	+	chr1	16888816	16940154	7419	-		-414.96	-0.9527	-1	0.79	0.98	2.3	5.07	3	0.07
tran	4826475	NA	LTCONS_00000543	MTCONS_00004233	chr1	21766628	21792645	552	+	chr1	16888816	16940154	6606	-		-414.96	0.9944	1	0.79	0.98	2.3	0	0.91	3.95
tran	40503245	NA	LTCONS_00027651	MTCONS_00028039	chr16	29502549	29506946	682	-	chr16	70010190	70099851	3062	-		-367.49	0.9979	1	3.15	4.32	3.01	0.28	0.52	0.23
tran	NA	NA	n375716	MTCONS_00074936	chr17	38276827	38278125	929	-	chrX	122993620	123047829	8359	+		-828.93	-0.6665	-1	0.29	0.58	0.28	2.31	2	3.66
tran	NA	NA	n375716	MTCONS_00074935	chr17	38276827	38278125	929	-	chrX	122993486	123047829	7457	+		-828.93	0.8406	1	0.29	0.58	0.28	1.36	1.71	0.9
cis_mRNA_overlap	0	mRNA	n336938	MTCONS_00073562	chr9	132658075	132658914	840	-	chr9	132654142	132805491	5878	-	*		-0.9999	-1	0.19	0.13	0.04	4.11	4.38	4.77
cis_mRNA_dw20k	9960	NA	LTCONS_00008473	MTCONS_00008470	chr10	75012534	75014196	1568	+	chr10	74927877	75002575	3742	+	*		0.9537	1	1.12	1.08	0.54	0.76	0.72	0.65
cis_mRNA_overlap	0	Lnc-AntiOverlap-mRNA	n339118	MTCONS_00042430	chr2	210895457	210929080	2197	+	chr2	210890310	211020369	6136	-	*		-0.9128	-1	0.42	0.7	0.33	0.64	0.59	0.72
cis_mRNA_overlap	0	CompleteIn-LncIntron	LTCONS_00005945	MTCONS_00005957	chr1	147906022	148176401	1228	-	chr1	148016192	148026022	1241	-	*		0.9562	1	2.92	3.29	2.57	0.95	0.97	0.89
tran	NA	NA	n342440	MTCONS_00069239	chr2	114363444	114365485	2020	-	chr8	155778	182318	8477	-		-1033.7	-0.9865	-1	1.16	1.04	0.88	0.1	0.22	0.31
tran	NA	NA	n342440	MTCONS_00069241	chr2	114363444	114365485	2020	-	chr8	155778	182318	8251	-		-1033.7	0.9686	1	1.16	1.04	0.88	0.1	0.08	0
cis_mRNA_overlap	0	mRNA	n345623	MTCONS_00052388	chr4	2561696	2599219	4766	+	chr4	2536492	2734302	5767	+	*		0.6433	0.866	0.11	0.14	0.11	2.78	3.06	1.55
cis_mRNA_overlap	0	Lnc-AntiOverlap-mRNA	n410982	MTCONS_00008780	chr10	97512963	97849992	4262	-	chr10	97515409	97637023	12392	+	*		-0.9707	-0.866	0.05	0.05	0.04	0	0.01	0.04
tran	NA	NA	n408042	MTCONS_00063947	chr6	30227339	30234728	4622	+	chr6_mcf_hap5	2617058	2704679	1476	-		-998.31	-0.6744	-0.866	0.78	0.78	0.84	4.6	12.78	2.22
tran	NA	NA	n323965	MTCONS_00020638	chr9	131060454	131061946	966	+	chr13	111546754	111567460	6922	-		-698.53	-0.6751	-1	0.92	0.9	1.07	8.43	10.86	8.1
tran	NA	NA	n324643	MTCONS_00030178	chr22	48769920	48773655	1016	+	chr17	1472556	1532487	9223	-		-916.95	0.9902	1	0	0.03	0.07	0.48	0.69	1.17
tran	NA	NA	n385118	MTCONS_00006068	chr7	32768504	32794385	1766	+	chr1	151740719	151763010	4208	-		-1272.17	-0.9588	-1	2.39	2.44	2.35	1.08	0.72	1.16
cis_mRNA_overlap	0	mRNA	LTCONS_00057963	MTCONS_00057959	chr5	69427437	69498377	12524	-	chr5	69416727	69497870	3769	-	*		0.9661	1	0.86	1.35	1.55	0.16	0.28	0.41
cis_mRNA_overlap	0	mRNA	LTCONS_00057963	MTCONS_00057960	chr5	69427437	69498377	12524	-	chr5	69416727	69521622	3590	-	*		-0.9896	-1	0.86	1.35	1.55	1.21	0.65	0.55
tran	NA	NA	LTCONS_00047494	MTCONS_00066824	chr22	23980675	24059610	3339	-	chr7	65425535	65447301	2097	-		-1412.79	0.8653	1	1.72	1.21	1.36	1.41	0.66	1.23
tran	NA	NA	n366528	MTCONS_00035590	chr3	149735043	149740537	959	-	chr19	12242097	12267577	9341	-		-850.44	0.98	1	0.76	1.07	1.17	0.74	1.45	1.48
cis_mRNA_dw20k	2366	NA	n371161	MTCONS_00071845	chr9	130875160	130877568	2022	-	chr9	130853465	130872795	5263	+	*		0.9999	1	0.66	0.91	0.75	1.66	2.97	2.15
cis_mRNA_overlap	0	AntiCompleteIn-mRNAIntron	n323983	MTCONS_00073336	chr9	119450705	119459126	1299	+	chr9	119187492	120177521	4963	-	*		0.9582	1	0	0.1	0.17	3.67	6.62	7.11
cis_mRNA_overlap	0	AntiCompleteIn-mRNAIntron	n378274	MTCONS_00019086	chr13	32599451	32605776	1115	-	chr13	32420865	32873866	13531	+	*		0.8812	1	0.28	0.26	0.16	11.68	10.17	9.2
cis_mRNA_up10k	597	NA	LTCONS_00037019	MTCONS_00037014	chr19	58447336	58448318	983	-	chr19	58411293	58446740	13084	-	*		0.987	1	3.27	3.54	2.18	3.94	3.96	3.31
cis_mRNA_overlap	0	Lnc-AntiOverlap-mRNA	n338246	MTCONS_00006141	chr1	153895879	153907635	1120	+	chr1	153901977	153918703	7927	-	*		0.9894	1	0.22	0.31	0.36	1.2	1.86	2.48
cis_mRNA_overlap	0	mRNA	LTCONS_00054148	MTCONS_00054151	chr4	17801804	17812381	7578	-	chr4	17801804	17812414	4936	-		-2449.56	0.9993	1	0.97	1.04	1.37	0.98	1.06	1.56
tran	NA	NA	n371184	MTCONS_00030178	chr9	139506546	139507780	703	-	chr17	1472556	1532487	9223	-		-847.75	0.8709	1	0.99	1.18	1.24	0.48	0.69	1.17
tran	NA	NA	n371184	MTCONS_00037317	chr9	139506546	139507780	703	-	chr2	11622954	11783475	12886	+		-502.11	1	1	0.99	1.18	1.24	1.34	1.5	1.55
tran	NA	NA	n384937	MTCONS_00076654	chr6	42929295	42929645	351	-	chrY	4868267	4981558	12618	+		-472.46	0.9959	1	2.28	1.87	2.94	4.85	4.11	6.56
tran	104716716	NA	n366869	MTCONS_00052301	chr4	104882773	104958080	812	+	chr4	53227	166058	11726	+		-931.33	-0.9722	-1	17.86	16.03	17.02	12.85	15.72	13.56

cis_mRNA_overlap	0	AntiCompleteIn-mRNAIntron	n340553	MTCONS_00058217	chr5	94871015	94872172	1157	+	chr5	94799529	94890714	7708	-	*		0.9869	1	1.33	1.81	1.34	0.3	0.72	0.38
cis_mRNA_overlap	0	mRNA	n405547	MTCONS_00027553	chr16	21529230	21531765	2536	-	chr16	21448105	21531804	14985	-	*		0.9874	1	3.49	4.14	3.72	2.77	4.17	3.06
cis_mRNA_overlap	0	mRNA	n407060	MTCONS_00010122	chr10	62629198	62760736	4373	-	chr10	62629144	62761185	4378	-		-1916.35	-0.957	-1	0	0.87	0.37	1.43	0	1.21
cis_mRNA_overlap	0	mRNA	n407060	MTCONS_00010120	chr10	62629198	62760736	4373	-	chr10	62629144	62704565	4406	-		-1926.2	0.982	1	0	0.87	0.37	0.92	1.53	1.28
cis_mRNA_dw20k	14094	NA	LTCONS_00070642	MTCONS_00070549	chr8	145560759	145561717	959	.	chr8	145575810	145582212	5331	-	*		-0.8646	-1	0.33	0.17	0.16	0.6	0.93	1.33
cis_mRNA_dw20k	14094	NA	LTCONS_00070642	MTCONS_00070550	chr8	145560759	145561717	959	.	chr8	145575810	145582217	5012	-	*		-0.7466	-1	0.33	0.17	0.16	2.2	2.35	2.75
tran	48222599	NA	n409585	MTCONS_00055679	chr5	69812079	69851150	610	-	chr5	21459589	21589481	5313	+		-406.55	0.7349	1	0	2.61	2.38	0.4	0.53	0.43
cis_mRNA_dw20k	4941	NA	LTCONS_00007586	MTCONS_00004234	chr1	16883644	16883876	233	.	chr1	16888816	16940154	7419	-	*		0.9112	0.866	3.51	3.51	2.8	5.07	3	0.07
cis_mRNA_dw20k	4941	NA	LTCONS_00007586	MTCONS_00004233	chr1	16883644	16883876	233	.	chr1	16888816	16940154	6606	-	*		-0.9755	-0.866	3.51	3.51	2.8	0	0.91	3.95
tran	NA	NA	n366183	MTCONS_00012799	chr3	20387157	20389501	415	+	chr11	118309253	118409866	27510	+		-522.52	0.8037	1	0.09	0.17	0.33	1.69	2.23	2.28
cis_mRNA_up10k	8102	NA	n342817	MTCONS_00065318	chr7	99637173	99639311	2138	+	chr7	99647412	99667594	7058	+	*		-0.9783	-1	11.61	11.97	10.63	0.07	0.02	0.13
cis_mRNA_dw20k	19231	NA	n406385	MTCONS_00066642	chr7	44078698	44082082	1663	+	chr7	44101312	44122129	15429	-	*		0.9811	0.866	1	1.27	2.32	0.06	0.06	0.14
cis_mRNA_dw20k	19231	NA	n406385	MTCONS_00066641	chr7	44078698	44082082	1663	+	chr7	44101312	44122129	13583	-	*		0.7068	1	1	1.27	2.32	0.53	0.78	0.8
cis_mRNA_up10k	108	NA	LTCONS_00075982	MTCONS_00074607	chrX	84479215	84498804	329	-	chrX	84498911	84528368	5915	+	*		0.9333	1	1.09	1.43	0.65	8.93	12.06	7.95
tran	NA	NA	n338021	MTCONS_00054074	chr22	31282046	31283901	1856	-	chr4	8363185	8431038	7874	-		-1562.68	0.8514	1	0.52	0.54	0.33	0.26	0.47	0.12
cis_mRNA_overlap	0	Lnc-AntiOverlap-mRNA	n340069	MTCONS_00008471	chr10	74994301	74995396	1095	-	chr10	74927877	75002575	6155	+	*		-0.9135	-0.866	0.77	0.64	0.86	1.08	1.09	1.08
tran	9067728	NA	n385197	MTCONS_00064844	chr7	65216092	65228661	1765	+	chr7	56119378	56148365	4952	+		-967.06	0.844	1	0.75	0.22	0.25	0.91	0.6	0.78
cis_mRNA_up10k	9272	NA	LTCONS_00047222	MTCONS_00047226	chr22	50609160	50615048	1414	+	chr22	50624319	50656144	12945	+	*		0.974	0.866	1.04	1.12	1.12	0.87	1.16	1.25
cis_mRNA_overlap	0	Lnc-CompleteIn-mRNAExon	n407262	MTCONS_00019863	chr13	25502893	25542607	6477	-	chr13	25502893	25542627	7000	-		-2860.45	-0.9134	-1	0.28	0	0.01	1.45	2.32	1.94
tran	NA	NA	n324281	MTCONS_00074936	chr6	170846326	170847963	806	+	chrX	122993620	123047829	8359	+		-631.35	-0.9404	-1	0.18	0.24	0.12	2.31	2	3.66
tran	NA	NA	n324281	MTCONS_00074935	chr6	170846326	170847963	806	+	chrX	122993486	123047829	7457	+		-631.35	0.9969	1	0.18	0.24	0.12	1.36	1.71	0.9
cis_mRNA_overlap	0	Lnc-CompleteIn-mRNAIntron	n382010	MTCONS_00019086	chr13	32524332	32527563	3232	+	chr13	32420865	32873866	13531	+	*		0.9136	1	0.36	0.31	0.15	11.68	10.17	9.2
tran	NA	NA	n372755	MTCONS_00066642	chr11	94820008	94821880	942	-	chr7	44101312	44122129	15429	-		-763.26	0.7206	0.866	0.15	0.4	0.5	0.06	0.06	0.14
cis_mRNA_dw20k	19231	NA	LTCONS_00064714	MTCONS_00066642	chr7	44078698	44082082	2100	+	chr7	44101312	44122129	15429	-	*		0.7878	0.866	0.51	0	0.82	0.06	0.06	0.14
cis_mRNA_dw20k	18598	NA	n334599	MTCONS_00061762	chr6	2928327	2929772	424	-	chr6	2948369	2971622	1445	-	*		0.9924	1	2.34	2.05	2.49	3.49	2.57	4.24
tran	NA	NA	n345283	MTCONS_00000711	chr14	100701760	100704777	473	-	chr1	28696093	28827167	6497	+		-478.6	-0.9981	-1	0	0.43	0.07	0.36	0	0.28
tran	NA	NA	n345283	MTCONS_00000709	chr14	100701760	100704777	473	-	chr1	28696093	28827167	6525	+		-478.6	-0.9836	-1	0	0.43	0.07	1.23	1.11	1.19
tran	NA	NA	n345283	MTCONS_00070589	chr14	100701760	100704777	473	-	chr8	145997260	146012823	3278	-		-483.17	0.9983	1	0	0.43	0.07	2.17	4.08	2.59
tran	NA	NA	n332381	MTCONS_00064648	chr10	38680896	38691746	399	-	chr7	35840627	35946723	4370	+		-254.12	0.8324	1	0.16	0.48	0.21	10.74	11.69	11.39
tran	NA	NA	n332381	MTCONS_00064649	chr10	38680896	38691746	399	-	chr7	35870348	35946723	4827	+		-236.3	0.9295	1	0.16	0.48	0.21	0.81	1.47	1.14
tran	NA	NA	n371374	MTCONS_00028040	chrX	13998185	14006939	544	-	chr16	70010190	70099871	6665	-		-335.4	0.8377	1	8.85	10.1	9.05	3.74	5.39	4.86
cis_mRNA_overlap	0	Lnc-CompleteIn-mRNAIntron	n324256	MTCONS_00064216	chr7	2443924	2454011	2067	+	chr7	2443223	2475112	2973	+	*		0.9425	1	4.06	4.74	5.26	0.24	1.08	1.19
cis_mRNA_overlap	0	Lnc-CompleteIn-mRNAIntron	n324256	MTCONS_00064218	chr7	2443924	2454011	2067	+	chr7	2443259	2475112	2951	+	*		-0.9742	-1	4.06	4.74	5.26	0.38	0.24	0
tran	53522370	NA	LTCONS_00027481	MTCONS_00028041	chr16	16485934	16487821	1762	-	chr16	70010190	70099880	4224	-		-3109.16	-0.8752	-1	0.16	0.21	0.45	0.45	0.3	0.21
cis_mRNA_overlap	0	Lnc-CompleteIn-mRNAExon	n341640	MTCONS_00070549	chr8	145575882	145577730	1758	-	chr8	145575810	145582212	5331	-		-1453.68	-0.9947	-1	0.24	0.11	0	0.6	0.93	1.33
cis_mRNA_overlap	0	mRNA	n341640	MTCONS_00070550	chr8	145575882	145577730	1758	-	chr8	145575810	145582217	5012	-		-1457.48	-0.9539	-1	0.24	0.11	0	2.2	2.35	2.75
cis_mRNA_dw20k	5821	NA	LTCONS_00027202	MTCONS_00025719	chr16	1826236	1832854	6348	-	chr16	1756175	1820416	5702	+	*		0.891	1	0.3	0.28	0.4	14.36	13.02	15.29
cis_mRNA_dw20k	5821	NA	LTCONS_00027202	MTCONS_00025718	chr16	1826236	1832854	6348	-	chr16	1756175	1820416	5699	+	*		0.8371	1	0.3	0.28	0.4	4.63	2.84	5.45
cis_mRNA_dw20k	438	NA	n790	MTCONS_00056736	chr5	139930090	139937036	869	-	chr5	139781399	139929653	11917	+	*		0.8233	0.866	13.41	14.13	13.72	0.28	0.46	0.46
cis_mRNA_dw20k	7636	NA	n790	MTCONS_00056734	chr5	139930090	139937036	869	-	chr5	139781399	139922455	11259	+	*		-0.8233	-0.866	13.41	14.13	13.72	0.52	0.43	0.43
cis_mRNA_up10k	261	NA	n338447	MTCONS_00006898	chr1	200993089	200997920	3495	+	chr1	200938461	200992829	9378	-	*		0.9746	1	4.95	4.79	2.67	10.18	9.7	8.54
cis_mRNA_dw20k	161	NA	n335595	MTCONS_00012351	chr11	71950462	71951269	808	-	chr11	71935164	71950302	9040	+	*		0.9815	1	0	0.34	0.67	1.3	1.74	1.95
cis_mRNA_overlap	0	Lnc-AntiOverlap-mRNA	n405703	MTCONS_00030180	chr17	1481377	1489848	3298	+	chr17	1472556	1533118	9141	-	*		0.9999	1	0.08	0.03	0.04	0.47	0.15	0.21
tran	NA	NA	n365203	MTCONS_00001489	chr2	100107781	100125484	473	+	chr1	78245231	78345227	6485	+		-573.15	0.9272	1	2.17	3.34	2.79	0.21	1.95	1.75
tran	NA	NA	n365203	MTCONS_00001491	chr2	100107781	100125484	473	+	chr1	78245281	78506196	5323	+		-573.15	-0.9636	-1	2.17	3.34	2.79	3.7	0	0.84
tran	NA	NA	n365203	MTCONS_00001488	chr2	100107781	100125484	473	+	chr1	78245231	78345227	6487	+		-573.15	-0.9874	-1	2.17	3.34	2.79	3.27	1.06	1.79
cis_mRNA_overlap	0	AntiCompleteIn-mRNAIntron	n381571	MTCONS_00010885	chr10	126392597	126403327	1629	+	chr10	126307784	126480439	6313	-	*		0.9253	1	0.06	0.17	0.16	2.05	2.93	2.53
cis_mRNA_up10k	7770	NA	n325254	MTCONS_00041040	chr2	70483570	70503314	577	+	chr2	70436575	70475801	5391	-	*		0.6941	1	0.07	0.28	0.05	3.43	3.73	2.02
cis_mRNA_up10k	7733	NA	n325254	MTCONS_00041042	chr2	70483570	70503314	577	+	chr2	70436575	70475838	9359	-	*		0.9496	1	0.07	0.28	0.05	11.26	12.26	10.62
cis_mRNA_up10k	7770	NA	n325254	MTCONS_00041041	chr2	70483570	70503314	577	+	chr2	70436575	70475801	9189	-	*		0.6336	1	0.07	0.28	0.05	5.94	6.21	3.3
cis_mRNA_overlap	0	mRNA	n341166	MTCONS_00014916	chr11	133792471	133807995	2004	-	chr11	133779074	133826908	4475	-		-1552.33	-0.9756	-1	0.38	0.23	0.18	0.23	0.63	0.64
cis_mRNA_overlap	0	mRNA	n341166	MTCONS_00014917	chr11	133792471	133807995	2004	-	chr11	133779074	133826975	4588	-		-1565.91	-0.9786	-1	0.38	0.23	0.18	0.24	1.2	1.24
cis_mRNA_overlap	0	mRNA	n335184	MTCONS_00064382	chr7	18535449	18630114	656	+	chr7	18535166	18710425	6579	+		-337.9	0.9971	1	0.18	0.2	0.06	0.67	0.7	0.22
cis_mRNA_overlap	0	mRNA	n341178	MTCONS_00066222	chr7	5428137	5451779	1332	-	chr7	5390483	5463458	6938	-		-1376.49	-0.9931	-1	0.11	0.05	0.09	2.13	3.02	2.33
cis_mRNA_overlap	0	mRNA	n341178	MTCONS_00066221	chr7	5428137	5451779	1332	-	chr7	5390483	5463452	9012	-		-1368.26	-0.9807	-1	0.11	0.05	0.09	0.94	1.28	0.99
cis_mRNA_overlap	0	mRNA	n385916	MTCONS_00003821	chr1	15603	29370	1189	-	chr1	14050	29439	3053	-	*		0.9587	1	0.35	1.38	0.74	0.47	0.66	0.49
cis_mRNA_overlap	0	mRNA	n411750	MTCONS_00031179	chr17	42282401	42298994	4997	-	chr17	42282261	42296982	4881	-		-2681	-1	-1	4.62	4.89	3.82	0.98	0.81	1.46
tran	NA	NA	n365033	MTCONS_00010010	chr2	214141276	214148929	1727	-	chr10	50641266	50818344	30532	-		-1714.73	-0.6746	-1	1.31	1.33	0.82	1.11	0.69	1.21
tran	NA	NA	n365033	MTCONS_00010011	chr2	214141276	214148929	1727	-	chr10	50641266	50818350	30643	-		-1714.73	0.8328	1	1.31	1.33	0.82	1.27	1.58	1.05
tran	NA	NA	LTCONS_00054666	MTCONS_00000612	chr4	103340075	103352403	256	-	chr1	24968184	24999772	7455	+		-217.26	-0.9982	-1	1.25	0.75	0.55	1.92	2.59	2.79
tran	NA	NA	n368846	MTCONS_00076654	chr6	28805646	28806783	709	-	chrY	4868267	4981558	12618	+		-664.74	-0.9844	-1	0.21	0.32	0.08	4.85	4.11	6.56
cis_mRNA_dw20k	5289	NA	n343016	MTCONS_00000710	chr1	28832455	28837404	2346	+	chr1	28696093	28827167	6521	+	*		0.9841	1	3.41	3.88	4.34	0.72	1.19	1.43
cis_mRNA_up10k	116	NA	n409543	MTCONS_00052066	chr3	194207869	194209274	1123	+	chr3	194123403	194207754	9002	-	*		-0.866	-0.866	0	0.03	0.06	1.1	1.1	0.69
cis_mRNA_dw20k	2282	NA	LTCONS_00073496	MTCONS_00071843	chr9	130875076	130881071	3102	-	chr9	130830430	130872795	4789	+	*		0.9404	1	0.38	0.23	0.58	0.29	0.26	0.55

cis_mRNA_overlap	0	Lnc-AntiOverlap-mRNA	n341361	MTCONS_00065991	chr7	150928545	150930322	1778	-	chr7	150929572	150936055	4916	+		-1886.5	0.8866	1	0.17	0.12	0.28	1.54	0.36	1.96
cis_mRNA_overlap	0	Lnc-AntiOverlap-mRNA	n341361	MTCONS_00065993	chr7	150928545	150930322	1778	-	chr7	150929584	150936055	3938	+		-1855.9	-0.9939	-1	0.17	0.12	0.28	1.18	1.48	0.75
cis_mRNA_overlap	0	Lnc-CompleteIn-mRNAIntron	n338634	MTCONS_00019086	chr13	32632895	32633647	748	+	chr13	32420865	32873866	13531	+	*		0.9948	1	0.34	0.13	0.04	11.68	10.17	9.2
tran	NA	NA	n325514	MTCONS_00076657	chr19	48349271	48356854	500	+	chrY	4898547	4981558	13244	+		-603.34	-0.9943	-1	5.07	7.15	5.61	1.57	0.93	1.47
cis_mRNA_up10k	91	NA	n338469	MTCONS_00021710	chr14	96967985	96968630	646	-	chr14	96968720	97033464	4387	+	*		0.7319	1	1.35	2.15	1.26	5.78	6.07	4.72
cis_mRNA_up10k	17	NA	n340071	MTCONS_00010272	chr10	75255282	75256029	424	+	chr10	75203544	75255266	2443	-	*		0.8591	1	0.09	0.16	0.24	7.79	8.48	8.5
cis_mRNA_overlap	0	other	n340071	MTCONS_00010268	chr10	75255282	75256029	424	+	chr10	75196537	75255282	3192	-	*		0.9883	1	0.09	0.16	0.24	0	1.74	5.24
cis_mRNA_up10k	3428	NA	n375998	MTCONS_00032694	chr18	716089	717930	1447	-	chr18	670324	712662	4304	-	*		-0.9418	-1	0.12	0.24	0.06	0.5	0.17	1.07
tran	NA	NA	n368195	MTCONS_00037317	chr5	172700826	172708051	382	+	chr2	11622954	11783475	12886	+		-363.21	0.9959	1	0.72	0.91	1	1.34	1.5	1.55
cis_mRNA_up10k	62	NA	n370121	MTCONS_00064313	chr7	7592410	7606237	871	-	chr7	7606298	7652468	9329	+	*		-0.9933	-1	1.31	1.96	2.42	2.13	1.2	0.78
tran	NA	NA	n381362	MTCONS_00076654	chr1	228673895	228674821	927	-	chrY	4868267	4981558	12618	+		-828.63	-0.9993	-1	0.38	0.42	0.27	4.85	4.11	6.56
tran	432999	NA	LTCONS_00066937	MTCONS_00065027	chr7	74601105	74655051	5145	-	chr7	74072015	74168107	3624	+		-1711.21	-0.7988	-0.866	0.73	0.86	0.53	1.28	1.18	1.28
cis_mRNA_overlap	0	Lnc-CompleteIn-mRNAExon	n409649	MTCONS_00004416	chr1	24292937	24306953	2373	-	chr1	24290088	24306953	12176	-	*		-0.9994	-1	2.5	1.45	1.3	0.6	1.52	1.61
cis_mRNA_overlap	0	Lnc-CompleteIn-mRNAExon	n378410	MTCONS_00004234	chr1	16892935	16905862	1069	-	chr1	16888816	16940154	7419	-		-567.62	0.972	1	2.96	2.81	2.2	5.07	3	0.07
cis_mRNA_overlap	0	Lnc-CompleteIn-mRNAExon	n378410	MTCONS_00004233	chr1	16892935	16905862	1069	-	chr1	16888816	16940154	6606	-		-567.62	-0.9994	-1	2.96	2.81	2.2	0	0.91	3.95
tran	NA	NA	n325508	MTCONS_00052870	chr2	296059	299379	590	-	chr4	56914919	57200064	10678	+		-345.62	0.9759	1	0.81	1	0.76	0.92	2.25	0
cis_mRNA_up10k	8886	NA	n369064	MTCONS_00062825	chr6	74364304	74365877	313	+	chr6	74303102	74355419	3412	-	*		0.9417	1	0.13	0	0.11	1.77	1.15	1.48
tran	NA	NA	n342333	MTCONS_00039956	chr1	179559507	179560440	934	+	chr2	236402710	237040438	10783	+		-520.25	-0.9943	-1	0.04	0.17	0.14	9.24	6.85	7.15
tran	NA	NA	n342333	MTCONS_00039959	chr1	179559507	179560440	934	+	chr2	236402736	237040438	10769	+		-549.77	0.8123	1	0.04	0.17	0.14	0.44	2.66	0.91
tran	48426206	NA	n384673	MTCONS_00055679	chr5	70015686	70150219	2014	+	chr5	21459589	21589481	5313	+		-957.72	0.9791	1	3.19	4.56	3.76	0.4	0.53	0.43
cis_mRNA_overlap	0	mRNA	n408111	MTCONS_00067465	chr7	126078652	126883569	3630	-	chr7	126078421	126892491	4099	-		-1709.91	-0.9078	-1	0.38	0	0.19	0	0.3	0.27
tran	NA	NA	n408337	MTCONS_00069239	chr17	81174666	81188573	2256	+	chr8	155778	182318	8477	-		-1230.32	-0.9527	-1	0.16	0.04	0.02	0.1	0.22	0.31
tran	NA	NA	n408337	MTCONS_00069241	chr17	81174666	81188573	2256	+	chr8	155778	182318	8251	-		-1230.32	0.7488	1	0.16	0.04	0.02	0.1	0.08	0
cis_mRNA_dw20k	18421	NA	n376820	MTCONS_00043213	chr20	25225790	25227480	590	-	chr20	25176325	25207370	2293	+	*		-0.9966	-1	0.19	0.23	0	5.28	4.95	8.63
cis_mRNA_dw20k	18421	NA	n376820	MTCONS_00043212	chr20	25225790	25227480	590	-	chr20	25176325	25207370	2826	+	*		-0.9878	-1	0.19	0.23	0	4.21	4.17	8.99
cis_mRNA_dw20k	12953	NA	LTCONS_00070643	MTCONS_00070547	chr8	145562506	145562858	353	.	chr8	145575810	145582212	5259	-	*		0.8802	1	0	1.01	0.4	0.02	0.46	0.39
tran	78788325	NA	n366895	MTCONS_00052870	chr4	135988388	135992533	667	+	chr4	56914919	57200064	10678	+		-780.32	-0.9136	-0.866	0.05	0	0.05	0.92	2.25	0
tran	78788325	NA	n366895	MTCONS_00052869	chr4	135988388	135992533	667	+	chr4	56914805	57200064	10656	+		-780.32	0.9481	0.866	0.05	0	0.05	19.73	16.31	18.62
tran	NA	NA	LTCONS_00076586	MTCONS_00058317	chrX	40794221	40794472	252	.	chr5	114539713	114632458	16163	-		-455.4	0.8145	1	0	1.21	0.75	0.99	1.62	1.02
tran	NA	NA	n386230	MTCONS_00069239	chr19	59086766	59110850	1036	+	chr8	155778	182318	8477	-		-742.84	0.9797	1	4.23	5.58	7.64	0.1	0.22	0.31
tran	NA	NA	n386230	MTCONS_00069241	chr19	59086766	59110850	1036	+	chr8	155778	182318	8251	-		-742.84	-0.9772	-1	4.23	5.58	7.64	0.1	0.08	0
tran	1319398	NA	n326384	MTCONS_00005908	chr1	147572534	147579700	537	-	chr1	146214653	146253137	3614	-		-282.3	-0.7632	-1	1.1	1.02	0.43	0	1.09	1.44
cis_mRNA_overlap	0	mRNA	n326384	MTCONS_00005932	chr1	147572534	147579700	537	-	chr1	147574207	147589414	3854	-	*		-0.7524	-1	1.1	1.02	0.43	1.39	1.56	1.61
cis_mRNA_overlap	0	Lnc-CompleteIn-mRNAIntron	n340086	MTCONS_00010321	chr10	79214867	79216481	1615	-	chr10	78644635	79397577	6115	-	*		-0.8322	-0.866	0.02	0.06	0.11	0.6	0.59	0.59
cis_mRNA_overlap	0	Lnc-CompleteIn-mRNAIntron	n332851	MTCONS_00069873	chr8	71032114	71037863	660	-	chr8	71021222	71315888	9572	-	*		0.9949	1	0.72	0.4	0.49	6.71	4.59	5.38
cis_mRNA_overlap	0	Lnc-CompleteIn-mRNAIntron	n332851	MTCONS_00069871	chr8	71032114	71037863	660	-	chr8	71021222	71158306	10331	-	*		-0.7862	-1	0.72	0.4	0.49	1.79	2.75	1.9
cis_mRNA_overlap	0	Lnc-CompleteIn-mRNAIntron	n332851	MTCONS_00069872	chr8	71032114	71037863	660	-	chr8	71021222	71315834	9311	-	*		-0.8195	-1	0.72	0.4	0.49	2.91	4.72	3.22
cis_mRNA_overlap	0	Lnc-CompleteIn-mRNAExon	n384541	MTCONS_00053706	chr4	170642341	170644334	1994	+	chr4	170541677	170644392	6255	+		-920.59	-0.987	-1	0.41	0	0.6	3.07	4.06	2.23
tran	19205831	NA	n408023	MTCONS_00031600	chr17	43513266	43568146	4914	-	chr17	62773976	62833309	6100	-		-3616.28	0.9747	1	0.88	0.54	0.85	0.6	0.47	0.56
tran	NA	NA	n408023	MTCONS_00032055	chr17	43513266	43568146	4914	-	chr17_ctg5_hap1	126502	183185	3623	-		-2637.14	-1	-1	0.88	0.54	0.85	0.19	0.91	0.26
tran	19177605	NA	n408023	MTCONS_00031596	chr17	43513266	43568146	4914	-	chr17	62745750	62833302	8207	-		-3530.14	-0.9986	-1	0.88	0.54	0.85	0.27	0.83	0.35
tran	NA	NA	n373553	MTCONS_00010010	chr13	38109077	38125673	3314	+	chr10	50641266	50818344	30532	-		-1929.65	0.9988	1	0.07	0.01	0.08	1.11	0.69	1.21
tran	NA	NA	n373553	MTCONS_00008781	chr13	38109077	38125673	3314	+	chr10	97515409	97637023	12236	+		-3382.96	0.9912	0.866	0.07	0.01	0.08	0.09	0	0.09
tran	NA	NA	n373553	MTCONS_00010011	chr13	38109077	38125673	3314	+	chr10	50641266	50818350	30643	-		-1929.65	-0.9573	-1	0.07	0.01	0.08	1.27	1.58	1.05
cis_mRNA_dw20k	2087	NA	n382098	MTCONS_00019629	chr13	103532449	103548383	2312	+	chr13	103498162	103530363	6986	+	*		0.9594	1	0.03	0.18	0.08	0.43	0.55	0.5
cis_mRNA_dw20k	2087	NA	n382098	MTCONS_00019627	chr13	103532449	103548383	2312	+	chr13	103497031	103530363	7672	+	*		0.989	1	0.03	0.18	0.08	0.69	1.1	0.77
tran	NA	NA	n334776	MTCONS_00001490	chr16	84649611	84650115	505	-	chr1	78245281	78506196	5315	+		-620.62	0.7839	1	1.55	1.14	6.2	0.7	0	1.01
cis_mRNA_up10k	476	NA	n340822	MTCONS_00061930	chr6	4136306	4157619	2277	+	chr6	4115927	4135831	1605	-	*		0.9781	1	0	0.14	0.06	0	0.18	0.11
cis_mRNA_overlap	0	mRNA	LTCONS_00056073	MTCONS_00056070	chr5	69734407	69746189	5552	+	chr5	69696747	69746189	4977	+		-2509.55	0.9878	1	2.13	1.93	2.64	1.43	0.98	4.43
cis_mRNA_overlap	0	Lnc-CompleteIn-mRNAIntron	n325225	MTCONS_00041431	chr2	100705115	100705776	236	-	chr2	100161762	100721866	10064	-	*		-0.9878	-0.866	0.18	0.16	0.16	0	0.15	0.18
cis_mRNA_overlap	0	Lnc-CompleteIn-mRNAIntron	n380532	MTCONS_00018781	chr12	130904080	130905564	415	-	chr12	130880680	131200847	5180	-	*		0.8514	1	0.18	0	0.58	3.76	0.05	4.67
cis_mRNA_overlap	0	Lnc-CompleteIn-mRNAIntron	n380532	MTCONS_00018783	chr12	130904080	130905564	415	-	chr12	130880680	131200847	7309	-	*		0.9994	1	0.18	0	0.58	1.09	0	3.2
cis_mRNA_overlap	0	Lnc-CompleteIn-mRNAIntron	n380532	MTCONS_00018782	chr12	130904080	130905564	415	-	chr12	130880680	131200847	4784	-	*		0.9827	1	0.18	0	0.58	1.13	0	2.38
cis_mRNA_dw20k	10668	NA	n380532	MTCONS_00018787	chr12	130904080	130905564	415	-	chr12	130916231	131200847	3321	-	*		0.9981	1	0.18	0	0.58	0.5	0	1.37
tran	NA	NA	n405299	MTCONS_00063948	chr6	29855383	29858856	1839	+	chr6_mcf_hap5	2701469	2704679	1429	-		-941.9	-0.6192	-0.866	0.14	0.13	0.13	1.3	5.31	1.92
tran	3384603	NA	n326378	MTCONS_00002112	chr1	148754857	148757132	566	+	chr1	145293371	145370255	10498	+		-357.43	-0.9985	-1	0.45	0.24	0.53	0.61	1.47	0.36

tran	3384603	NA	n326378	MTCONS_00002111	chr1	148754857	148757132	566	+	chr1	145293371	145370255	5674	+		-351.2	-1	-1	0.45	0.24	0.53	1.1	2.25	0.67
cis_mRNA_up10k	919	NA	LTCONS_00037118	MTCONS_00035094	chr19	58137688	58140167	2480	.	chr19	58141085	58155498	4332	+	*		0.949	1	0.75	1.08	1.47	0.72	0.93	1.99
cis_mRNA_up10k	4369	NA	LTCONS_00037118	MTCONS_00035098	chr19	58137688	58140167	2480	.	chr19	58144535	58155498	5172	+	*		-0.9811	-1	0.75	1.08	1.47	2.38	2.3	2.1
tran	NA	NA	n323974	MTCONS_00076654	chr9	127545854	127547840	509	-	chrY	4868267	4981558	12618	+		-371.11	-0.98	-1	2.94	3.05	2.03	4.85	4.11	6.56
cis_mRNA_up10k	1490	NA	n410457	MTCONS_00024302	chr15	93426073	93441977	1593	+	chr15	93443466	93571283	9837	+	*		-0.9985	-1	16.8	13.42	11.16	2.8	3.85	4.42
cis_mRNA_up10k	1484	NA	n410457	MTCONS_00024301	chr15	93426073	93441977	1593	+	chr15	93443460	93537587	5962	+	*		0.9981	1	16.8	13.42	11.16	1.77	1.26	0.99
tran	NA	NA	n407017	MTCONS_00064010	chr6	33286335	33290793	2356	-	chr6_qbl_hap6	4518473	4522985	2666	-		-1295.42	0.986	1	0.81	0	0.45	2.11	0.25	1.56
cis_mRNA_dw20k	5130	NA	n411686	MTCONS_00061760	chr6	2940611	2943240	2539	+	chr6	2948369	2969631	1923	-	*		0.9361	1	0.13	0.26	0.27	0.76	1.03	1.22
cis_mRNA_dw20k	5130	NA	n411686	MTCONS_00061761	chr6	2940611	2943240	2539	+	chr6	2948369	2971602	1556	-	*		-0.8919	-1	0.13	0.26	0.27	3.81	2.94	2.04
cis_mRNA_overlap	0	mRNA	n337800	MTCONS_00074704	chrX	101854454	101905073	964	+	chrX	101854096	101972671	3991	+	*		0.9948	1	7.26	7.43	6.74	7.77	9.15	5.09
cis_mRNA_up10k	103	NA	n383723	MTCONS_00038737	chr2	114368816	114384715	1231	-	chr2	114384817	114402022	3224	+	*		0.9364	1	2.61	3.02	2.11	0.03	0.13	0
cis_mRNA_overlap	0	Lnc-CompleteIn-mRNAIntron	n324190	MTCONS_00067466	chr7	126787075	126792049	4975	-	chr7	126078421	126893160	4100	-	*		-0.9787	-1	0.04	0.02	0.05	0.56	1	0.49
cis_mRNA_overlap	0	Lnc-CompleteIn-mRNAIntron	n339870	MTCONS_00032435	chr18	46091166	46094713	3548	+	chr18	46065427	46389588	6259	+	*		-0.9656	-0.866	0.21	0.21	0.15	0.45	0.59	0.97
cis_mRNA_overlap	0	Lnc-CompleteIn-mRNAIntron	n339870	MTCONS_00032434	chr18	46091166	46094713	3548	+	chr18	46065427	46389588	6765	+	*		0.8808	0.866	0.21	0.21	0.15	1.9	2.08	1.7
tran	NA	NA	n342601	MTCONS_00066765	chr10	75346970	75347274	305	+	chr7	56077796	56102236	3179	-		-554.4	-0.9992	-1	0.26	1.48	1.64	1.57	0.66	0.49
tran	NA	NA	n342601	MTCONS_00066768	chr10	75346970	75347274	305	+	chr7	56077796	56119284	3358	-		-554.4	0.7748	1	0.26	1.48	1.64	0.73	0.78	0.92
cis_mRNA_overlap	0	Lnc-CompleteIn-mRNAExon	n411755	MTCONS_00061054	chr6	127587883	127609705	2541	+	chr6	127587732	127609855	2983	+		-1104.95	0.9569	1	2.49	4.06	3.24	1.45	1.92	1.55
cis_mRNA_overlap	0	mRNA	n385240	MTCONS_00067128	chr7	97595908	97601638	698	-	chr7	97481372	97602630	4004	-		-576.8	-0.9898	-1	0.7	0	1.46	1.09	1.26	0.78
tran	NA	NA	n323869	MTCONS_00052870	chrX	56661549	56667616	713	+	chr4	56914919	57200064	10678	+		-1079.52	-0.9767	-1	1.04	0.78	1.11	0.92	2.25	0
tran	43574882	NA	n384422	MTCONS_00052869	chr4	13335037	13339924	4888	+	chr4	56914805	57200064	10656	+		-3899.61	-0.8172	-1	10.08	11.76	11.56	19.73	16.31	18.62
cis_mRNA_overlap	0	CompleteIn-LncIntron	LTCONS_00052103	MTCONS_00052108	chr3	195666277	195731964	5295	-	chr3	195684854	195717166	5790	-	*		-0.7559	-0.866	0.02	0.04	0.04	3.52	2.83	3.29
cis_mRNA_overlap	0	CompleteIn-LncIntron	LTCONS_00052103	MTCONS_00052109	chr3	195666277	195731964	5295	-	chr3	195684854	195717166	4032	-	*		-0.9901	-0.866	0.02	0.04	0.04	1.88	1.04	0.89
tran	NA	NA	n345054	MTCONS_00074936	chr9	97067717	97068415	698	+	chrX	122993620	123047829	8359	+		-620.29	0.9845	0.866	0.05	0.05	0.11	2.31	2	3.66
tran	NA	NA	n345054	MTCONS_00074935	chr9	97067717	97068415	698	+	chrX	122993486	123047829	7457	+		-620.29	-0.9025	-0.866	0.05	0.05	0.11	1.36	1.71	0.9
tran	NA	NA	n345054	MTCONS_00076654	chr9	97067717	97068415	698	+	chrY	4868267	4981558	12618	+		-600.54	0.9557	0.866	0.05	0.05	0.11	4.85	4.11	6.56
cis_mRNA_overlap	0	Lnc-AntiOverlap-mRNA	LTCONS_00038184	MTCONS_00041103	chr2	74611627	74627755	6911	+	chr2	74579851	74619214	15274	-	*		0.8771	1	1.01	1.67	1.09	2.11	2.89	2.56
cis_mRNA_up10k	4445	NA	n339076	MTCONS_00025940	chr16	11032736	11033901	1164	-	chr16	11038345	11276074	6871	+	*		0.986	1	2.44	2.5	3.11	1.14	1.47	2.43
tran	NA	NA	n383885	MTCONS_00064290	chr20	25990435	26002424	1548	+	chr7	6617065	6648357	10434	+		-1426.05	0.6218	0.866	0.24	0.26	0.24	1.94	2.89	2.74
cis_mRNA_up10k	64	NA	n369919	MTCONS_00064313	chr7	7589540	7606235	2663	-	chr7	7606298	7652468	9329	+	*		0.9978	1	0.76	0.67	0.64	2.13	1.2	0.78
cis_mRNA_dw20k	12912	NA	n324612	MTCONS_00061800	chr6	3127977	3140989	1506	-	chr6	3153900	3227968	1818	-	*		-0.8794	-1	0.11	0.09	0.13	5.88	6	2.15
cis_mRNA_dw20k	12912	NA	n324612	MTCONS_00061799	chr6	3127977	3140989	1506	-	chr6	3153900	3227968	1726	-	*		0.9747	1	0.11	0.09	0.13	409	378.03	480.82
cis_mRNA_overlap	0	mRNA	n324249	MTCONS_00064285	chr7	6621773	6627705	1116	+	chr7	6617058	6648357	4557	+	*		-0.9994	-1	0.09	0.2	0.22	0.42	0.05	0
tran	NA	NA	n383080	MTCONS_00058320	chr17	19349247	19350178	932	-	chr5	114597548	114632458	7737	-		-657.31	-0.9853	-1	237.5	255.74	171.91	1.06	1.05	1.29
cis_mRNA_overlap	0	mRNA	n378866	MTCONS_00004232	chr1	16931913	16940029	2743	-	chr1	16888816	16940083	7373	-	*		0.9768	1	3.2	3.53	3.33	3.93	6.2	5.25
tran	NA	NA	n387140	MTCONS_00069240	chr2	114368816	114384715	1482	-	chr8	155778	182318	8373	-		-836.5	0.8666	1	0.91	1.24	0.99	0	0.17	0.12
tran	1450680	NA	n405453	MTCONS_00065027	chr7	72569026	72621336	3622	+	chr7	74072015	74168107	3624	+		-1577.38	0.9887	0.866	33.92	26.95	32.8	1.28	1.18	1.28
cis_mRNA_dw20k	18014	NA	n406931	MTCONS_00071845	chr9	130890808	130892913	2106	+	chr9	130853465	130872795	5263	+	*		0.9492	1	5.68	7.95	5.83	1.66	2.97	2.15
cis_mRNA_dw20k	939	NA	n410808	MTCONS_00011078	chr11	565657	568457	2303	-	chr11	558978	564719	2784	+	*		0.9545	1	0.26	0.32	0.45	2.54	2.55	3.33
cis_mRNA_up10k	7652	NA	n410808	MTCONS_00011083	chr11	565657	568457	2303	-	chr11	576108	612518	6552	+	*		0.9998	1	0.26	0.32	0.45	0.96	1.09	1.35
cis_mRNA_overlap	0	mRNA	n386451	MTCONS_00067127	chr7	97503667	97601638	929	-	chr7	97481372	97601638	2696	-		-546.83	0.9938	1	0.16	0.18	0.24	0.75	1.08	3.08
tran	NA	NA	LTCONS_00002192	MTCONS_00036202	chr1	148555673	148566637	2241	+	chr19	38120564	38147153	8329	-		-1311.36	0.9856	1	3.47	5.06	4.55	0.76	1.01	0.97
tran	NA	NA	LTCONS_00002192	MTCONS_00036203	chr1	148555673	148566637	2241	+	chr19	38120564	38147153	7953	-		-1289.17	-0.9385	-1	3.47	5.06	4.55	1.7	0.89	1.4
tran	NA	NA	LTCONS_00002192	MTCONS_00036204	chr1	148555673	148566637	2241	+	chr19	38120564	38147191	7291	-		-1289.17	0.8314	1	3.47	5.06	4.55	3.37	3.84	3.44
cis_mRNA_dw20k	4503	NA	LTCONS_00017091	MTCONS_00015240	chr12	8940194	8941443	1224	-	chr12	8850493	8935692	5471	+	*		-0.9889	-1	6.24	7.46	6.33	6.49	4.17	5.97
cis_mRNA_dw20k	15207	NA	n341745	MTCONS_00071534	chr9	108418650	108420264	1615	+	chr9	108320411	108403444	7109	+	*		0.7756	0.866	0.04	0.02	0.04	0.55	0.33	0.94
tran	NA	NA	n387326	MTCONS_00066823	chr4	144480625	144482612	1988	+	chr7	65425535	65447301	3593	-		-1145.74	-0.9965	-1	1.04	1.26	1.54	1.42	1.31	1.12
tran	NA	NA	n387326	MTCONS_00066825	chr4	144480625	144482612	1988	+	chr7	65425535	65447379	2361	-		-1013.56	0.9764	1	1.04	1.26	1.54	0	0.34	0.54
tran	NA	NA	n324963	MTCONS_00069358	chr20	48213408	48216046	733	+	chr8	10958954	11060192	12778	-		-914.47	-0.96	-1	0.7	0.4	0.24	1	2.1	3.72
tran	NA	NA	n365438	MTCONS_00067477	chr2	45401699	45402931	706	-	chr7	127010302	127032875	6352	-		-442.6	0.866	0.866	0.05	0	0.1	0.25	0.25	0.37
cis_mRNA_dw20k	18531	NA	n381375	MTCONS_00007494	chr1	243219612	243265046	3838	-	chr1	243283576	243419303	12649	-	*		0.7751	0.866	0.59	0.7	0.59	1.07	1.25	0.75
cis_mRNA_dw20k	18531	NA	n381375	MTCONS_00007493	chr1	243219612	243265046	3838	-	chr1	243283576	243419273	12031	-	*		-0.7327	-0.866	0.59	0.7	0.59	3.06	2.9	3.43
tran	64385662	NA	n326700	MTCONS_00001490	chr1	13856393	13859620	1007	-	chr1	78245281	78506196	5315	+		-531.44	0.9959	1	0.14	0	0.18	0.7	0	1.01
tran	NA	NA	n339406	MTCONS_00010010	chr3	99898411	99900574	2157	+	chr10	50641266	50818344	30532	-		-2290.16	-0.9796	-1	0.65	1.06	0.4	1.11	0.69	1.21
tran	NA	NA	n339406	MTCONS_00008781	chr3	99898411	99900574	2157	+	chr10	97515409	97637023	12236	+		-1106.61	-0.927	-0.866	0.65	1.06	0.4	0.09	0	0.09
tran	NA	NA	n339406	MTCONS_00010011	chr3	99898411	99900574	2157	+	chr10	50641266	50818350	30643	-		-2290.16	0.9991	1	0.65	1.06	0.4	1.27	1.58	1.05
tran	158607893	NA	n342454	MTCONS_00037317	chr2	170391367	170391969	470	-	chr2	11622954	11783475	12886	+		-421.79	1	1	0.24	0.44	0.5	1.34	1.5	1.55
tran	NA	NA	n342454	MTCONS_00074664	chr2	170391367	170391969	470	-	chrX	100353217	100425597	10299	+		-408.71	-0.9981	-1	0.24	0.44	0.5	0.9	0.61	0.55
tran	NA	NA	n365012	MTCONS_00030178	chr2	177494893	177502290	838	-	chr17	1472556	1532487	9223	-		-746.08	-0.8123	-1	1.02	0.76	0.72	0.48	0.69	1.17
cis_mRNA_overlap	0	mRNA	n410136	MTCONS_00072471	chr9	32972604	33001574	2164	-	chr9	32968411	33001639	6188	-		-1265.26	0.9299	1	1.38	1.81	0	0.91	0.99	0.85
cis_mRNA_overlap	0	Lnc-CompleteIn-mRNAIntron	n341758	MTCONS_00073277	chr9	114991482	114992595	606	-	chr9	114980715	115095964	7078	-	*		-0.9979	-1	0.18	0.66	0.49	6.11	4.65	5.25
tran	NA	NA	n366854	MTCONS_00001490	chr4	62009375	62019680	546	+	chr1	78245281	78506196	5315	+		-313.38	0.9996	1	0.82	0.72	0.87	0.7	0	1.01
cis_mRNA_dw20k	51	NA	n371350	MTCONS_00076014	chrX	100258821	100262956	3210	+	chrX	100263006	100307105	4402	-	*		-0.6547	-0.866	0.48	0.48	0.54	1.43	2.91	1.06
cis_mRNA_dw20k	4083	NA	n342574	MTCONS_00054075	chr4	8357038	8359103	2066	-	chr4	8363185	8431038	7806	-	*		0.7387	1	0.68	1.32	1.23	0.11	0.43	0.17
cis_mRNA_dw20k	1564	NA	LTCONS_00000260	MTCONS_00000257	chr1	7831329	7838585	6458	+	chr1	7764855	7829766	5074	+	*		-0.9607	-1	1.43	1.57	1.66	12.08	9	8.55

cis_mRNA_dw20k	16970	NA	n408344	MTCONS_00015939	chr12	57624111	57628718	2097	+	chr12	57546864	57607142	15612	+	*		-0.743	-0.866	1.5	1.2	0.55	0.58	0.9	0.9
cis_mRNA_dw20k	16970	NA	n408344	MTCONS_00015937	chr12	57624111	57628718	2097	+	chr12	57522282	57607142	16992	+	*		-0.9835	-1	1.5	1.2	0.55	1.27	1.67	2.11
cis_mRNA_dw20k	2772	NA	n345919	MTCONS_00000415	chr1	16115910	16126782	2000	-	chr1	16084305	16113139	3838	+	*		-0.8327	-1	0.09	0.05	0.02	0.35	0.36	0.81
cis_mRNA_dw20k	2772	NA	n345919	MTCONS_00000419	chr1	16115910	16126782	2000	-	chr1	16085255	16113139	3682	+	*		-0.8541	-1	0.09	0.05	0.02	1.58	1.63	2.34
cis_mRNA_overlap	0	mRNA	n323963	MTCONS_00071870	chr9	131084558	131087828	583	+	chr9	131084558	131096357	8649	+		-403.13	0.8119	1	0.06	0.23	0.28	0.84	1.25	2.75
cis_mRNA_overlap	0	mRNA	n323963	MTCONS_00071871	chr9	131084558	131087828	583	+	chr9	131084558	131096357	7985	+		-426.47	0.9445	1	0.06	0.23	0.28	0.59	1.05	1.56
cis_mRNA_dw20k	1976	NA	n370618	MTCONS_00068298	chr8	28915363	28922437	6644	+	chr8	28747903	28913388	6431	+	*		0.9999	1	2.78	3.45	3.47	1.55	2.72	2.74
cis_mRNA_dw20k	385	NA	n341568	MTCONS_00070133	chr8	101928753	101930297	1545	-	chr8	101930681	101966843	4952	-	*		-0.9796	-1	2.89	2.82	2.66	1.24	1.33	2.07
cis_mRNA_up10k	7601	NA	n323956	MTCONS_00071911	chr9	131291200	131307181	1959	+	chr9	131314781	131396015	7974	+	*		-0.983	-1	0.09	0.05	0	0	0.65	2.32
tran	NA	NA	n323941	MTCONS_00069239	chr9	136584405	136585564	280	+	chr8	155778	182318	8477	-		-345.46	0.9993	1	0	0.14	0.26	0.1	0.22	0.31
tran	NA	NA	n323941	MTCONS_00069241	chr9	136584405	136585564	280	+	chr8	155778	182318	8251	-		-345.46	-0.9295	-1	0	0.14	0.26	0.1	0.08	0
tran	NA	NA	n364085	MTCONS_00010010	chr1	25589071	25593390	2904	-	chr10	50641266	50818344	30532	-		-1595.61	-0.8363	-1	5.22	5.59	4.31	1.11	0.69	1.21
tran	NA	NA	n364085	MTCONS_00008781	chr1	25589071	25593390	2904	-	chr10	97515409	97637023	12236	+		-3279.49	-0.7231	-0.866	5.22	5.59	4.31	0.09	0	0.09
tran	NA	NA	n364085	MTCONS_00010011	chr1	25589071	25593390	2904	-	chr10	50641266	50818350	30643	-		-1614.06	0.9439	1	5.22	5.59	4.31	1.27	1.58	1.05
cis_mRNA_dw20k	8387	NA	n379832	MTCONS_00059085	chr5	180258027	180261779	671	+	chr5	180270165	180288363	9165	-	*		0.9484	1	0.18	1.09	0.31	1.9	2.74	2.27
cis_mRNA_up10k	454	NA	LTCONS_00017328	MTCONS_00015444	chr12	26097790	26111511	6952	-	chr12	26111964	26225956	5742	+	*		0.9689	1	1.21	1.82	2.64	0.34	0.67	2
cis_mRNA_up10k	2597	NA	n340918	MTCONS_00030050	chr17	79369409	79370944	1536	-	chr17	79373540	79433733	10731	+	*		-0.9449	-1	0.05	0.04	0.1	0.09	0.17	0
cis_mRNA_up10k	2597	NA	n340918	MTCONS_00030052	chr17	79369409	79370944	1536	-	chr17	79373540	79433733	10515	+	*		0.9922	1	0.05	0.04	0.1	0.24	0.02	0.79
tran	NA	NA	n365972	MTCONS_00000415	chr3	119814477	119817634	545	+	chr1	16084305	16113139	3838	+		-546.37	-0.8327	-1	1.29	1.21	1.15	0.35	0.36	0.81
tran	NA	NA	n365972	MTCONS_00000419	chr3	119814477	119817634	545	+	chr1	16085255	16113139	3682	+		-546.37	-0.8541	-1	1.29	1.21	1.15	1.58	1.63	2.34
cis_mRNA_dw20k	7314	NA	LTCONS_00011027	MTCONS_00010146	chr10	64917370	64918851	1482	.	chr10	64926164	65281611	9104	-	*		0.8632	1	0.67	1.23	0.65	0.79	0.94	0.62
cis_mRNA_overlap	0	Lnc-CompleteIn-mRNAExon	n385796	MTCONS_00074680	chrX	100740408	100788446	3842	+	chrX	100740395	100790972	6386	+		-1881.29	1	1	3.5	3.98	2.65	1.46	1.59	1.23
cis_mRNA_dw20k	10178	NA	n408034	MTCONS_00071843	chr9	130882972	130890712	1586	-	chr9	130830430	130872795	4789	+	*		0.6629	1	1.92	1.39	1.99	0.29	0.26	0.55
cis_mRNA_overlap	0	Lnc-AntiOverlap-mRNA	n340166	MTCONS_00009200	chr10	123921860	123923892	2033	-	chr10	123923056	124014084	3933	+	*		0.9901	1	0.22	0.32	0.35	2.54	3.27	3.68
cis_mRNA_overlap	0	Lnc-AntiOverlap-mRNA	n340166	MTCONS_00009198	chr10	123921860	123923892	2033	-	chr10	123923007	124014084	3793	+	*		0.9146	1	0.22	0.32	0.35	5.51	5.53	5.56
cis_mRNA_overlap	0	Lnc-AntiOverlap-mRNA	n340166	MTCONS_00009199	chr10	123921860	123923892	2033	-	chr10	123923007	124014084	3841	+	*		-0.9952	-1	0.22	0.32	0.35	2.17	1.22	0.77
cis_mRNA_overlap	0	mRNA	n345737	MTCONS_00065941	chr7	150102420	150109537	4820	+	chr7	150102113	150109813	4351	+		-3112.32	-0.822	-0.866	0.17	0.24	0.2	0.13	0.11	0.11
cis_mRNA_overlap	0	mRNA	n345737	MTCONS_00065938	chr7	150102420	150109537	4820	+	chr7	150076398	150109813	3635	+		-2660.75	0.9903	1	0.17	0.24	0.2	0	0.59	0.18
cis_mRNA_overlap	0	Lnc-CompleteIn-mRNAExon	n410134	MTCONS_00072472	chr9	32972604	33001639	1885	-	chr9	32968411	33002977	7531	-		-1123.9	0.9943	1	2.04	1.91	1.87	0.15	0.11	0.09
cis_mRNA_overlap	0	mRNA	n411640	MTCONS_00019684	chr13	111267807	111292342	2594	+	chr13	111267807	111292343	2896	+		-1597.23	-0.7644	-1	3.43	3.86	2.19	4.66	4.32	4.7
cis_mRNA_dw20k	3458	NA	n339363	MTCONS_00050833	chr3	51985865	51987918	2052	-	chr3	51991375	52008646	5144	-	*		0.8062	1	0.52	1.05	0.71	0.62	0.79	0.78
cis_mRNA_dw20k	3458	NA	n339363	MTCONS_00050832	chr3	51985865	51987918	2052	-	chr3	51991375	52008646	6302	-	*		0.9498	1	0.52	1.05	0.71	0.27	0.82	0.62
tran	NA	NA	n364940	MTCONS_00070570	chr2	75146682	75151973	763	-	chr8	145672727	145688245	4458	-		-588.06	-0.909	-1	0.14	0.41	0	2.52	2.31	3.06
tran	NA	NA	n364940	MTCONS_00001490	chr2	75146682	75151973	763	-	chr1	78245281	78506196	5315	+		-466.82	-0.9993	-1	0.14	0.41	0	0.7	0	1.01
tran	NA	NA	n367819	MTCONS_00000612	chr5	135802366	135855987	427	+	chr1	24968184	24999772	7455	+		-336.1	-0.9965	-1	1.12	0.43	0.31	1.92	2.59	2.79
tran	NA	NA	n382641	MTCONS_00006068	chr15	75797279	75797803	525	+	chr1	151740719	151763010	4208	-		-329.21	-0.988	-1	0.79	1.84	0.77	1.08	0.72	1.16
tran	NA	NA	n324417	MTCONS_00041342	chr6	113856435	113861316	524	-	chr2	96487344	96658969	20870	-		-852.42	-0.9211	-1	72.57	72.48	63.61	2.25	2.77	3.55
tran	NA	NA	n324417	MTCONS_00041341	chr6	113856435	113861316	524	-	chr2	96487344	96549806	19081	-		-852.42	0.9995	1	72.57	72.48	63.61	6.48	6.43	5.38
cis_mRNA_overlap	0	Lnc-AntiOverlap-mRNA	n378226	MTCONS_00041102	chr2	74612624	74619031	766	+	chr2	74579851	74619214	12873	-	*		0.995	1	0.28	0.21	1.04	2.72	2.64	3.07
cis_mRNA_up10k	7258	NA	n378090	MTCONS_00004232	chr1	16947340	16971167	5934	-	chr1	16888816	16940083	7373	-	*		0.9659	1	3.7	6.08	4.54	3.93	6.2	5.25
tran	41328032	NA	n363832	MTCONS_00001489	chr1	36916215	36917200	640	+	chr1	78245231	78345227	6485	+		-697.4	0.8471	1	9.26	12.05	10.31	0.21	1.95	1.75
tran	41328082	NA	n363832	MTCONS_00001491	chr1	36916215	36917200	640	+	chr1	78245281	78506196	5323	+		-697.4	-0.9017	-1	9.26	12.05	10.31	3.7	0	0.84
tran	41328032	NA	n363832	MTCONS_00001488	chr1	36916215	36917200	640	+	chr1	78245231	78345227	6487	+		-697.4	-0.9443	-1	9.26	12.05	10.31	3.27	1.06	1.79
tran	14307623	NA	n384428	MTCONS_00052301	chr4	14473680	14889793	2853	-	chr4	53227	166058	11726	+		-4300.93	0.8554	1	10.46	12.17	11.71	12.85	15.72	13.56
cis_mRNA_up10k	88	NA	LTCONS_00028404	MTCONS_00027552	chr16	21531887	21534054	2168	.	chr16	21448105	21531800	16151	-	*		-0.8897	-1	1.52	1.47	0.62	0.37	0.41	0.45
cis_mRNA_up10k	64	NA	n409646	MTCONS_00049596	chr3	142719687	142720309	623	-	chr3	142720372	142779959	11896	+	*		-0.6474	-1	0.53	0.5	0.97	4.21	5.44	4.02
cis_mRNA_overlap	0	mRNA	n339265	MTCONS_00050395	chr3	17199899	17782399	6608	-	chr3	17198654	17784240	6422	-		-2802.59	0.7384	1	0.52	0.55	0.38	2.99	3.55	2.89
tran	31351383	NA	n341586	MTCONS_00068694	chr8	110975869	110977604	1735	-	chr8	79578282	79624487	15452	+		-892.39	-0.9972	-1	0.27	0.26	0.13	3.66	3.69	3.86
cis_mRNA_up10k	7544	NA	n407877	MTCONS_00050692	chr3	48730884	48754711	1375	-	chr3	48673896	48723341	17215	-	*		0.9124	1	2.4	2.03	2.99	1.22	1	1.29
cis_mRNA_dw20k	2412	NA	n378569	MTCONS_00034272	chr19	37742828	37747421	462	+	chr19	37708321	37740417	8180	+	*		-0.9333	-1	0.15	0.08	0	0.07	0.42	0.51
cis_mRNA_dw20k	2412	NA	n378569	MTCONS_00034273	chr19	37742828	37747421	462	+	chr19	37709374	37740417	8724	+	*		0.9989	1	0.15	0.08	0	1.19	0.96	0.65
tran	NA	NA	n324565	MTCONS_00027552	chr6	25150046	25164871	577	+	chr16	21448105	21531800	16151	-		-429.17	-0.9881	-1	0.97	0.78	0.45	0.37	0.41	0.45
cis_mRNA_overlap	0	mRNA	LTCONS_00018788	MTCONS_00018781	chr12	130944708	131200833	9488	-	chr12	130880680	131200847	5180	-	*		0.9358	1	1.65	0.02	3.42	3.76	0.05	4.67
cis_mRNA_overlap	0	mRNA	LTCONS_00018788	MTCONS_00018783	chr12	130944708	131200833	9488	-	chr12	130880680	131200847	7309	-	*		0.9875	1	1.65	0.02	3.42	1.09	0	3.2
cis_mRNA_overlap	0	mRNA	LTCONS_00018788	MTCONS_00018782	chr12	130944708	131200833	9488	-	chr12	130880680	131200847	4784	-	*		1	1	1.65	0.02	3.42	1.13	0	2.38
cis_mRNA_overlap	0	mRNA	LTCONS_00018788	MTCONS_00018787	chr12	130944708	131200833	9488	-	chr12	130916231	131200847	3321	-	*		0.9914	1	1.65	0.02	3.42	0.5	0	1.37
cis_mRNA_overlap	0	mRNA	n410459	MTCONS_00024296	chr15	93426526	93441977	1497	+	chr15	93425950	93571283	12699	+		-775.65	0.649	1	0.93	2.34	2.23	6.23	6.72	6.29
cis_mRNA_overlap	0	mRNA	n407859	MTCONS_00020279	chr13	52706779	52733996	2395	-	chr13	52706741	52728944	2577	-		-949.14	0.9901	1	1.06	1.44	0.53	1.5	2.25	0.86
tran	NA	NA	n323875	MTCONS_00044626	chrX	48323106	48324489	450	-	chr20	39804376	39946230	12652	-		-475.2	-0.974	-1	0	0.15	0.08	0.06	0	0.04
tran	13829721	NA	n323897	MTCONS_00074257	chrX	33670156	33870273	339	+	chrX	47699993	47785604	13752	+		-401.4	-0.99	-1	1.1	0.71	0	1.06	1.28	1.52
tran	NA	NA	n323897	MTCONS_00000419	chrX	33670156	33870273	339	+	chr1	16085255	16113139	3682	+		-241.88	-0.9558	-1	1.1	0.71	0	1.58	1.63	2.34
tran	NA	NA	n373653	MTCONS_00000612	chr13	30937313	30947169	469	-	chr1	24968184	24999772	7455	+		-353.66	0.7166	1	0.22	0.24	0.55	1.92	2.59	2.79
tran	NA	NA	n372062	MTCONS_00027552	chr10	102140800	102148112	619	+	chr16	21448105	21531800	16151	-		-811.58	0.8811	1	0	0.02	0.57	0.37	0.41	0.45
tran	NA	NA	n377094	MTCONS_00010010	chr21	27722099	27722953	516	-	chr10	50641266	50818344	30532	-		-478.17	-0.9856	-1	0.07	0.2	0	1.11	0.69	1.21
tran	NA	NA	n377094	MTCONS_00010011	chr21	27722099	27722953	516	-	chr10	50641266	50818350	30643	-		-478.17	0.9973	1	0.07	0.2	0	1.27	1.58	1.05
tran	NA	NA	n326556	MTCONS_00039909	chr1	80611637	80612894	580	-	chr2	233561975	233729859	18457	+		-486.54	0.61	0.866	0.06	0.48	0.12	0.84	0.89	0.89
tran	NA	NA	n324443	MTCONS_00052301	chr6	100075290	100086437	882	+	chr4	53227	166058	11726	+		-920.17	0.9568	1	1.56	2.6	2.09	12.85	15.72	13.56

cis_mRNA_overlap	0	Lnc-AntiOverlap-mRNA	LTCONS_00065581	MTCONS_00067457	chr7	124569927	124742104	721	+	chr7	124461305	124570037	5214	-	*	0.9753	1	0.2	0	0.01	0.82	0.6	0.66
cis_mRNA_dw20k	13059	NA	n382926	MTCONS_00027555	chr16	21443347	21445776	2430	-	chr16	21458834	21531790	5591	-	*	0.9388	1	0.44	0.66	0.31	8.59	9.43	6.81
cis_mRNA_overlap	0	Lnc-AntiOverlap-mRNA	n342405	MTCONS_00020279	chr13	52703027	52706859	3433	+	chr13	52706741	52728944	2577	-	*	-0.8423	-0.866	0.01	0.01	0.02	1.5	2.25	0.86
tran	NA	NA	LTCONS_00024397	MTCONS_00003818	chr15	102500790	102516854	3720	+	chr1	14050	29381	9122	-	-2741.21	0.9923	1	3.21	4.43	4.72	2.41	2.88	3.09
tran	NA	NA	n370256	MTCONS_00056622	chr8	19616323	19620042	905	+	chr5	133451298	133483920	3550	+	-491.14	-0.9449	-1	0.08	0	0.04	0	0.05	0.04
tran	NA	NA	n370256	MTCONS_00056621	chr8	19616323	19620042	905	+	chr5	133451298	133483920	3163	+	-469.75	-0.9177	-1	0.08	0	0.04	0	0.08	0.01
tran	NA	NA	n367820	MTCONS_00052870	chr5	136070616	136090375	564	+	chr4	56914919	57200064	10678	+	-351.35	0.998	1	0.33	0.44	0.27	0.92	2.25	0
cis_mRNA_overlap	0	mRNA	n405993	MTCONS_00016879	chr12	132568828	132610885	1539	+	chr12	132568828	132615587	4881	+	-994.36	0.9099	0.866	0.3	0.35	0.23	0.81	0.81	0.79
cis_mRNA_up10k	4337	NA	LTCONS_00067904	MTCONS_00065318	chr7	99642325	99643076	752	.	chr7	99647412	99667594	7058	+	*	0.9587	1	0.77	0.26	0.98	0.07	0.02	0.13
cis_mRNA_dw20k	18566	NA	LTCONS_00067904	MTCONS_00067185	chr7	99642325	99643076	752	.	chr7	99661641	99679371	8777	-	*	-0.991	-1	0.77	0.26	0.98	0.21	0.72	0.11
cis_mRNA_dw20k	18566	NA	LTCONS_00067904	MTCONS_00067184	chr7	99642325	99643076	752	.	chr7	99661641	99679371	8936	-	*	0.7703	1	0.77	0.26	0.98	1.69	1.63	2.42
cis_mRNA_dw20k	18566	NA	LTCONS_00067904	MTCONS_00067183	chr7	99642325	99643076	752	.	chr7	99661641	99679371	4083	-	*	0.8121	1	0.77	0.26	0.98	3.1	3.05	3.39
cis_mRNA_dw20k	7924	NA	n373373	MTCONS_00016892	chr12	133169697	133170155	292	-	chr12	133135899	133161774	4985	+	*	0.9594	1	0.56	0.38	0.75	0.57	0.48	0.87
tran	356454	NA	LTCONS_00027573	MTCONS_00027552	chr16	21888253	21892223	2867	-	chr16	21448105	21531800	16151	-	-1796.72	0.9295	1	0.63	0.68	0.95	0.37	0.41	0.45
tran	8383909	NA	LTCONS_00027573	MTCONS_00027690	chr16	21888253	21892223	2867	-	chr16	30276131	30346695	10819	-	-1562.35	0.919	1	0.63	0.68	0.95	0.35	0.68	0.98
tran	NA	NA	n340598	MTCONS_00047230	chr5	118463897	118464783	885	+	chr22	50639408	50656144	7863	+	-716.97	0.9559	0.866	0	0.18	0.18	1.68	2.41	2.19
tran	NA	NA	n340598	MTCONS_00047226	chr5	118463897	118464783	885	+	chr22	50624319	50656144	12945	+	-716.97	0.974	0.866	0	0.18	0.18	0.87	1.16	1.25
tran	NA	NA	n378867	MTCONS_00035588	chr2	179278695	179296068	793	+	chr19	12242097	12267577	7026	-	-641.35	-0.9942	-1	0.55	1.13	0.69	1.78	1.28	1.61
cis_mRNA_dw20k	5882	NA	n384337	MTCONS_00051602	chr3	141655549	141657389	1841	-	chr3	141663270	141868386	9883	-	*	0.9608	1	0.61	0.38	0.26	0.62	0.48	0.28
tran	85027510	NA	n325438	MTCONS_00041342	chr2	11456631	11459835	399	+	chr2	96487344	96658969	20870	-	-480.3	-0.9714	-1	0.69	0.27	0	2.25	2.77	3.55
tran	85027510	NA	n325438	MTCONS_00041341	chr2	11456631	11459835	399	+	chr2	96487344	96549806	19081	-	-480.3	0.8207	1	0.69	0.27	0	6.48	6.43	5.38
tran	NA	NA	n377190	MTCONS_00040484	chr21	46708063	46709414	542	+	chr2	25591401	25896503	11005	-	-328.94	0.9577	1	6.58	10.2	8.71	0.09	0.48	0.22
cis_mRNA_overlap	0	Lnc-CompleteIn-mRNAIntron	n407043	MTCONS_00061760	chr6	2965323	2967412	571	-	chr6	2948369	2969631	1923	-	*	-0.8879	-1	0.32	0.27	0	0.76	1.03	1.22
cis_mRNA_overlap	0	Lnc-CompleteIn-mRNAIntron	n407043	MTCONS_00061761	chr6	2965323	2967412	571	-	chr6	2948369	2971602	1556	-	*	0.933	1	0.32	0.27	0	3.81	2.94	2.04
cis_mRNA_overlap	0	CompleteIn-LncIntron	LTCONS_00052104	MTCONS_00052109	chr3	195666277	195731964	5860	-	chr3	195684854	195717166	4032	-	*	0.7317	1	0.06	0.05	0	1.88	1.04	0.89
cis_mRNA_up10k	3406	NA	n377550	MTCONS_00048087	chr22	51024833	51025888	536	+	chr22	51006098	51021428	5612	-	*	0.9995	1	0.2	0.47	0.46	1.24	2.06	2
cis_mRNA_overlap	0	Lnc-AntiOverlap-mRNA	n338112	MTCONS_00001838	chr1	109233371	109235146	1776	-	chr1	109234927	109245998	7550	+	*	0.963	1	0.19	0.51	0.36	1.18	1.97	1.41
cis_mRNA_overlap	0	Lnc-AntiOverlap-mRNA	n338112	MTCONS_00001840	chr1	109233371	109235146	1776	-	chr1	109234932	109245998	6959	+	*	-0.9452	-1	0.19	0.51	0.36	0.39	0.05	0.31
cis_mRNA_overlap	0	Lnc-AntiOverlap-mRNA	n338195	MTCONS_00048088	chr22	51009428	51010882	1455	+	chr22	51006098	51021476	6017	-	*	0.9898	1	0.1	0.17	0.19	5.18	6.44	7.13
cis_mRNA_overlap	0	Lnc-AntiOverlap-mRNA	n338195	MTCONS_00048092	chr22	51009428	51010882	1455	+	chr22	51006098	51021486	5859	-	*	-0.9958	-1	0.1	0.17	0.19	0.39	0.12	0
tran	1598671	NA	n383680	MTCONS_00041393	chr2	96472813	96482510	2951	-	chr2	98081180	98160993	24447	-	-1611.86	-0.9996	-1	2.92	3.82	3.47	1.57	0.94	1.17
tran	1598671	NA	n383680	MTCONS_00041394	chr2	96472813	96482510	2951	-	chr2	98081180	98206428	25798	-	-1611.86	0.9926	1	2.92	3.82	3.47	2.83	3.88	3.36
tran	NA	NA	n340058	MTCONS_00001551	chr10	71785390	71785990	601	-	chr1	84629892	84708194	9069	+	-251.24	0.9923	1	0.55	0.77	0.54	4.59	5.93	4.31
tran	NA	NA	n340058	MTCONS_00001553	chr10	71785390	71785990	601	-	chr1	84629909	84708194	8413	+	-251.24	-0.8846	-1	0.55	0.77	0.54	0.49	0.48	0.5
tran	NA	NA	n340058	MTCONS_00001554	chr10	71785390	71785990	601	-	chr1	84629990	84708194	8389	+	-251.24	0.9792	1	0.55	0.77	0.54	3.1	3.16	3.08
tran	1044608	NA	LTCONS_00071007	MTCONS_00072644	chr9	40332907	40341454	4202	+	chr9	39072764	39288300	5226	-	-1683.98	0.9975	1	1.02	0.86	1.36	0.54	0.41	0.92
cis_mRNA_overlap	0	Lnc-AntiOverlap-mRNA	LTCONS_00010545	MTCONS_00008780	chr10	97635325	97667908	840	-	chr10	97515409	97637023	12392	+	*	0.9996	1	3.03	3.21	3.68	0	0.01	0.04
tran	NA	NA	n378156	MTCONS_00001489	chr10	31596922	31607861	320	-	chr1	78245231	78345227	6485	+	-328.73	0.8047	1	0.75	1.43	0.96	0.21	1.95	1.75
tran	NA	NA	n378156	MTCONS_00001491	chr10	31596922	31607861	320	-	chr1	78245281	78506196	5323	+	-319.98	-0.8665	-1	0.75	1.43	0.96	3.7	0	0.84
cis_mRNA_dw20k	17228	NA	n337773	MTCONS_00004406	chr1	24086872	24104777	1074	-	chr1	24122004	24127294	2123	-	*	-0.9742	-1	1.31	0.89	1.67	1.51	1.94	1.36
tran	NA	NA	n367973	MTCONS_00076657	chr5	142869420	142910915	560	-	chrY	4898547	4981558	13244	+	-709.23	-0.9953	-1	0.2	0.36	0.24	1.57	0.93	1.47
tran	NA	NA	n324286	MTCONS_00069358	chr6	169951339	169952123	694	-	chr8	10958954	11060192	12778	-	-610.13	0.8572	1	2.18	2.71	2.77	1	2.1	3.72
cis_mRNA_overlap	0	Lnc-CompleteIn-mRNAIntron	n324286	MTCONS_00063722	chr6	169951339	169952123	694	-	chr6	169857303	170102168	9247	-	*	0.9355	1	2.18	2.71	2.77	2.61	2.98	3.27
tran	NA	NA	LTCONS_00003817	MTCONS_00075293	chr1	14050	29381	2735	-	chrX	155251143	155255371	1784	+	-1362.23	0.9954	1	1	0.39	0.6	3.66	0	1.57
cis_mRNA_overlap	0	Lnc-CompleteIn-mRNAExon	LTCONS_00003817	MTCONS_00003821	chr1	14050	29381	2735	-	chr1	14050	29439	3053	-	-1827.14	-0.8222	-1	1	0.39	0.6	0.47	0.66	0.49
cis_mRNA_overlap	0	Lnc-CompleteIn-mRNAIntron	n5263	MTCONS_00067357	chr7	108150668	108151827	1159	-	chr7	108110121	108166638	5368	-	*	-0.7721	-1	0.36	0.94	0.49	1.38	1.12	1.16
cis_mRNA_overlap	0	Lnc-CompleteIn-mRNAIntron	n323960	MTCONS_00071890	chr9	131240316	131241218	800	+	chr9	131218285	131263722	5539	+	*	0.7665	1	0.11	0	0.16	0.04	0	0.9
cis_mRNA_overlap	0	Lnc-AntiOverlap-mRNA	n341878	MTCONS_00015480	chr12	28341263	28343671	2409	-	chr12	28343477	28703209	2690	+	*	0.9799	1	0.58	0.48	0.39	0.94	0.61	0
tran	NA	NA	n371495	MTCONS_00008382	chrX	46183253	46187066	753	-	chr10	70360234	70460551	13766	+	-639.08	0.8938	1	0.19	0.22	0.05	5.71	6.44	5.21
tran	NA	NA	n345916	MTCONS_00003819	chr2	114341441	114352674	287	+	chr1	14050	29437	7973	-	-274.41	0.9124	1	0	0.08	0.38	5.14	7.76	9.61
tran	NA	NA	LTCONS_00024395	MTCONS_00075293	chr15	102500501	102516854	3942	+	chrX	155251143	155255371	1784	+	-1461.33	0.9632	1	0.17	0.14	0.16	3.66	0	1.57
tran	NA	NA	LTCONS_00024395	MTCONS_00003821	chr15	102500501	102516854	3942	+	chr1	14050	29439	3053	-	-2140.8	-0.9719	-1	0.17	0.14	0.16	0.47	0.66	0.49
tran	NA	NA	n325247	MTCONS_00052870	chr2	75020893	75023305	1266	+	chr4	56914919	57200064	10678	+	-917.05	0.937	1	8.18	8.72	8.14	0.92	2.25	0
tran	NA	NA	n324558	MTCONS_00004419	chr6	28724042	28724822	537	-	chr1	24290088	24306953	8967	-	-607.01	-0.9505	-1	0.21	0.01	0.4	0.16	0.79	0
tran	NA	NA	n405980	MTCONS_00052109	chr5	1572073	1594646	1152	-	chr3	195684854	195717166	4032	-	-770.92	-0.9446	-1	1.2	1.33	1.44	1.88	1.04	0.89
cis_mRNA_dw20k	3	NA	LTCONS_00063235	MTCONS_00061054	chr6	127609857	127664849	2756	-	chr6	127587732	127609855	2983	+	*	0.9332	1	0.67	1.28	1	1.45	1.92	1.55
cis_mRNA_overlap	0	Lnc-AntiOverlap-mRNA	n370741	MTCONS_00072431	chr9	23829671	23849912	1427	+	chr9	23690089	23850763	4143	-	*	-0.9934	-0.866	0.15	0.2	0.15	28.12	26.44	28.36

tran	NA	NA	n326650	MTCONS_00064290	chr1	32587382	32588851	687	-	chr7	6617065	6648357	10434	+		-861.14	-0.7723	-1	1.42	0.74	1.27	1.94	2.89	2.74
tran	NA	NA	n385081	MTCONS_00008781	chr7	8384553	8387652	3100	+	chr10	97515409	97637023	12236	+		-3421.28	0.9245	0.866	0.61	0.48	0.56	0.09	0	0.09
cis_mRNA_overlap	0	other	n343053	MTCONS_00056736	chr5	139929653	139937678	1948	-	chr5	139781399	139929653	11917	+	*		0.9688	0.866	2.77	3.52	3.78	0.28	0.46	0.46
cis_mRNA_dw20k	7199	NA	n343053	MTCONS_00056734	chr5	139929653	139937678	1948	-	chr5	139781399	139922455	11259	+	*		-0.9688	-0.866	2.77	3.52	3.78	0.52	0.43	0.43
cis_mRNA_up10k	986	NA	n380720	MTCONS_00015445	chr12	26107588	26110979	741	-	chr12	26111964	26225956	5648	+	*		0.9583	1	0.18	0.7	0.49	0.68	0.98	0.94
cis_mRNA_overlap	0	Lnc-CompleteIn-mRNAIntron	n341849	MTCONS_00060306	chr6	42751234	42759057	2739	+	chr6	42713996	42836296	9285	+	*		-1	-1	1.33	1.92	1.22	1.3	1.05	1.35
cis_mRNA_overlap	0	Lnc-CompleteIn-mRNAIntron	n341849	MTCONS_00060307	chr6	42751234	42759057	2739	+	chr6	42714017	42836296	6704	+	*		0.9412	1	1.33	1.92	1.22	0.59	1.25	0
tran	1041235	NA	n379473	MTCONS_00030180	chr17	2574352	2577029	885	-	chr17	1472556	1533118	9141	-		-871.51	-0.9754	-1	4.78	6.31	5.71	0.47	0.15	0.21
tran	NA	NA	n326366	MTCONS_00071871	chr1	155421802	155423819	471	+	chr9	131084558	131096357	7985	+		-725.86	-0.9851	-1	0.9	0.8	0.74	0.59	1.05	1.56
cis_mRNA_dw20k	7440	NA	n381805	MTCONS_00014916	chr11	133766330	133771635	5306	-	chr11	133779074	133826908	4475	-	*		0.9794	1	2.22	2.68	2.82	0.23	0.63	0.64
cis_mRNA_dw20k	7440	NA	n381805	MTCONS_00014917	chr11	133766330	133771635	5306	-	chr11	133779074	133826975	4588	-	*		0.9821	1	2.22	2.68	2.82	0.24	1.2	1.24
tran	NA	NA	n366295	MTCONS_00052395	chr3	119817722	119824125	608	+	chr4	2819681	2842823	9784	+		-524.28	-0.9577	-1	0.21	0.33	0.12	4.86	4.44	4.94
cis_mRNA_dw20k	68	NA	n344995	MTCONS_00068298	chr8	28913455	28922296	3981	+	chr8	28747903	28913388	6431	+	*		0.9982	1	2.48	3.15	3.21	1.55	2.72	2.74
cis_mRNA_overlap	0	mRNA	n409275	MTCONS_00061762	chr6	2948369	2964501	2270	-	chr6	2948369	2971622	1445	-	*		0.9983	1	0.07	0.06	0.08	3.49	2.57	4.24
cis_mRNA_overlap	0	AntiCompleteIn-mRNAExon	n341807	MTCONS_00073664	chr9	138716695	138798984	1234	+	chr9	138700333	138799283	8930	-		-2433.53	-0.9072	-0.866	0.03	0	0.03	6.68	10.18	4.12
tran	NA	NA	n340982	MTCONS_00069240	chr11	17439193	17447011	924	-	chr8	155778	182318	8373	-		-779.82	-0.8009	-1	0.08	0	0.07	0	0.17	0.12
cis_mRNA_up10k	64	NA	n339631	MTCONS_00049596	chr3	142719749	142720309	561	-	chr3	142720372	142779959	11896	+	*		0.7821	1	1.04	1.29	0.32	4.21	5.44	4.02
tran	NA	NA	n324356	MTCONS_00052301	chr6	143313041	143319476	554	+	chr4	53227	166058	11726	+		-459.18	-0.986	-1	1.62	1.37	1.6	12.85	15.72	13.56
cis_mRNA_dw20k	18343	NA	n408131	MTCONS_00062268	chr6	28346598	28367544	4343	-	chr6	28385886	28411332	9324	-	*		-0.9364	-1	4.95	4.54	3.51	0.97	1	1.02
tran	NA	NA	n325895	MTCONS_00052301	chr10	107239344	107252103	450	-	chr4	53227	166058	11726	+		-525.75	-0.9899	-1	0.34	0.06	0.31	12.85	15.72	13.56
cis_mRNA_dw20k	13468	NA	LTCONS_00052167	MTCONS_00052177	chr3	197358479	197381350	9141	-	chr3	197394817	197463843	10135	-	*		0.9899	0.866	1.83	1.83	1.95	1.53	1.86	3.69
tran	NA	NA	n345550	MTCONS_00051602	chr2	168351651	168394068	1044	+	chr3	141663270	141868386	9883	-		-808.94	-0.9365	-1	1.02	1.42	1.57	0.62	0.48	0.28
cis_mRNA_dw20k	5526	NA	LTCONS_00070644	MTCONS_00070572	chr8	145670009	145670953	945	.	chr8	145676478	145687915	1965	-	*		0.86	1	0.94	0.38	0.4	10.32	8.33	9.4
cis_mRNA_dw20k	5526	NA	LTCONS_00070644	MTCONS_00070573	chr8	145670009	145670953	945	.	chr8	145676478	145688245	11117	-	*		-0.9999	-1	0.94	0.38	0.4	1.71	2.94	2.92
cis_mRNA_overlap	0	Lnc-CompleteIn-mRNAIntron	n340919	MTCONS_00030051	chr17	79374890	79376779	1890	+	chr17	79373540	79433733	12320	+	*		0.9775	1	0.49	0.98	0.89	0.48	0.98	0.79
cis_mRNA_dw20k	14647	NA	n410267	MTCONS_00061930	chr6	4098867	4101281	1333	+	chr6	4115927	4135831	1605	-	*		-0.8668	-1	0.43	0	0.37	0	0.18	0.11
tran	NA	NA	n342872	MTCONS_00014146	chr9	99687295	99688122	828	-	chr11	66448399	66497268	13286	-		-807.18	0.604	1	0.13	0.12	0.38	3.01	2.92	3.02
cis_mRNA_overlap	0	Lnc-CompleteIn-mRNAExon	n365498	MTCONS_00041393	chr2	98104748	98105300	450	-	chr2	98081180	98160993	24447	-		-770.6	-0.9512	-1	0	0.97	0.91	1.57	0.94	1.17
cis_mRNA_dw20k	11609	NA	n365498	MTCONS_00041398	chr2	98104748	98105300	450	-	chr2	98116908	98160997	8825	-	*		0.612	1	0	0.97	0.91	2.69	3.03	2.72
cis_mRNA_overlap	0	Lnc-CompleteIn-mRNAExon	n365498	MTCONS_00041394	chr2	98104748	98105300	450	-	chr2	98081180	98206428	25798	-		-770.6	0.8948	1	0	0.97	0.91	2.83	3.88	3.36
cis_mRNA_overlap	0	mRNA	n336894	MTCONS_00001551	chr1	84630580	84644903	218	+	chr1	84629892	84708194	9069	+		-115.18	0.7768	1	7.68	8.14	5.98	4.59	5.93	4.31
cis_mRNA_overlap	0	mRNA	n336894	MTCONS_00001553	chr1	84630580	84644903	218	+	chr1	84629909	84708194	8413	+	*		-0.9492	-1	7.68	8.14	5.98	0.49	0.48	0.5
cis_mRNA_overlap	0	mRNA	n336894	MTCONS_00001554	chr1	84630580	84644903	218	+	chr1	84629990	84708194	8389	+		-115.18	0.8247	1	7.68	8.14	5.98	3.1	3.16	3.08
cis_mRNA_overlap	0	Lnc-AntiOverlap-mRNA	n381901	MTCONS_00018119	chr12	90102732	90105728	2997	+	chr12	89981826	90102733	7180	-	*		0.7434	1	20.59	16.22	20.04	1.2	0	0.25
cis_mRNA_overlap	0	Lnc-AntiOverlap-mRNA	n381901	MTCONS_00018120	chr12	90102732	90105728	2997	+	chr12	89981826	90107256	11234	-		-5826.07	0.6789	1	20.59	16.22	20.04	3.19	2.59	2.66
cis_mRNA_overlap	0	Lnc-AntiOverlap-mRNA	n381901	MTCONS_00018118	chr12	90102732	90105728	2997	+	chr12	89981826	90102733	7267	-	*		0.9957	1	20.59	16.22	20.04	56.68	49.58	56.49
tran	1073978	NA	LTCONS_00002099	MTCONS_00002112	chr1	144172386	144219394	270	+	chr1	145293371	145370255	10498	+		-156.59	0.9832	1	0.77	2.84	0.69	0.61	1.47	0.36
tran	1073978	NA	LTCONS_00002099	MTCONS_00002111	chr1	144172386	144219394	270	+	chr1	145293371	145370255	5674	+		-156.59	0.9729	1	0.77	2.84	0.69	1.1	2.25	0.67
cis_mRNA_overlap	0	mRNA	n406144	MTCONS_00069240	chr8	158345	182318	5788	-	chr8	155778	182318	8373	-		-2833.73	0.994	1	3.59	6.11	5.63	0	0.17	0.12
cis_mRNA_overlap	0	Lnc-CompleteIn-mRNAIntron	n325226	MTCONS_00041431	chr2	100652786	100658932	562	-	chr2	100161762	100721866	10064	-	*		-0.7258	-1	0.07	0.06	0	0	0.15	0.18
tran	11172099	NA	n366979	MTCONS_00052872	chr4	45724760	45909868	437	-	chr4	57081966	57200064	10458	+		-268.39	0.8406	1	8.12	11.47	12.52	15.47	18.33	26.84
cis_mRNA_overlap	0	Lnc-CompleteIn-mRNAIntron	n409287	MTCONS_00061800	chr6	3164345	3180755	1895	-	chr6	3153900	3227968	1818	-	*		-0.9565	-1	0.32	0.2	0.57	5.88	6	2.15
cis_mRNA_overlap	0	Lnc-CompleteIn-mRNAIntron	n409287	MTCONS_00061799	chr6	3164345	3180755	1895	-	chr6	3153900	3227968	1726	-	*		0.9997	1	0.32	0.2	0.57	409	378.03	480.82
cis_mRNA_dw20k	2426	NA	n380364	MTCONS_00011078	chr11	567144	568457	816	-	chr11	558978	564719	2784	+	*		0.9926	1	0	0.08	0.56	2.54	2.55	3.33
cis_mRNA_up10k	7652	NA	n380364	MTCONS_00011083	chr11	567144	568457	816	-	chr11	576108	612518	6552	+	*		0.9799	1	0	0.08	0.56	0.96	1.09	1.35
cis_mRNA_overlap	0	Lnc-CompleteIn-mRNAIntron	n342827	MTCONS_00067809	chr7	151973010	151974416	1406	-	chr7	151831996	152133112	17048	-	*		-0.9814	-1	0.1	0	0.07	2.67	3.52	3.07
cis_mRNA_overlap	0	Lnc-CompleteIn-mRNAIntron	n342827	MTCONS_00067810	chr7	151973010	151974416	1406	-	chr7	151831996	152133649	16864	-	*		-0.9736	-1	0.1	0	0.07	2.73	4.52	3.63
tran	130634125	NA	LTCONS_00005930	MTCONS_00004232	chr1	147574207	147578137	2587	-	chr1	16888816	16940083	7373	-		-1363.45	1	1	1.28	1.78	1.57	3.93	6.2	5.25
cis_mRNA_dw20k	2314	NA	LTCONS_00005930	MTCONS_00005935	chr1	147574207	147578137	2587	-	chr1	147580450	147610081	2781	-	*		0.9385	1	1.28	1.78	1.57	2.6	3.12	2.74
cis_mRNA_overlap	0	mRNA	LTCONS_00005930	MTCONS_00005933	chr1	147574207	147578137	2587	-	chr1	147574207	147610081	4489	-		-1325.71	-0.8164	-0.866	1.28	1.78	1.57	0.08	0	0.08
tran	424167	NA	LTCONS_00005930	MTCONS_00005954	chr1	147574207	147578137	2587	-	chr1	148002303	148025848	4803	-		-1332.06	-0.9556	-1	1.28	1.78	1.57	1.19	0.97	1.12
tran	2203953	NA	LTCONS_00005930	MTCONS_00002114	chr1	147574207	147578137	2587	-	chr1	145304210	145370255	11091	+		-1363.31	0.9108	1	1.28	1.78	1.57	0.52	1.07	0.63
tran	NA	NA	n384940	MTCONS_00052869	chr6	46459789	46501215	1342	+	chr4	56914805	57200064	10656	+		-1292.18	-0.7998	-1	9.18	13.04	12.7	19.73	16.31	18.62
tran	NA	NA	n325150	MTCONS_00052872	chr2	123539367	123541768	801	+	chr4	57081966	57200064	10458	+		-770.95	0.821	1	0.49	0.8	0.88	15.47	18.33	26.84
tran	26880399	NA	n325150	MTCONS_00041342	chr2	123539367	123541768	801	+	chr2	96487344	96658969	20870	-		-450.42	0.9034	1	0.49	0.8	0.88	2.25	2.77	3.55
tran	26989562	NA	n325150	MTCONS_00041341	chr2	123539367	123541768	801	+	chr2	96487344	96549806	19081	-		-454.86	-0.6884	-1	0.49	0.8	0.88	6.48	6.43	5.38
cis_mRNA_overlap	0	mRNA	n385923	MTCONS_00003821	chr1	17606	29370	868	-	chr1	14050	29439	3053	-		-718.59	0.8303	1	0	0.14	0.09	0.47	0.66	0.49
cis_mRNA_overlap	0	mRNA	n386034	MTCONS_00015242	chr12	8931627	8935685	1887	+	chr12	8850518	8935692	5961	+	*		0.9231	1	5.96	7.32	5.74	12.29	13.55	11.03
cis_mRNA_dw20k	469	NA	n407188	MTCONS_00061800	chr6	3118926	3153432	1616	+	chr6	3153900	3227968	1818	-	*		-0.7628	-1	1.94	1.04	2.36	5.88	6	2.15

cis_mRNA_dw20k	469	NA	n407188	MTCONS_00061799	chr6	3118926	3153432	1616	+	chr6	3153900	3227968	1726	-	*		0.9079	1	1.94	1.04	2.36	409	378.03	480.82
cis_mRNA_up10k	402	NA	n374280	MTCONS_00021130	chr14	45552347	45552901	467	-	chr14	45553302	45584892	5743	+	*		0.9758	0.866	0	0.07	0.07	2.56	4.35	3.94
cis_mRNA_up10k	402	NA	n374280	MTCONS_00021131	chr14	45552347	45552901	467	-	chr14	45553302	45584892	5430	+	*		-0.8496	-0.866	0	0.07	0.07	0.47	0.3	0.11
cis_mRNA_up10k	402	NA	n374280	MTCONS_00021129	chr14	45552347	45552901	467	-	chr14	45553302	45584892	3519	+	*		0.8093	0.866	0	0.07	0.07	3.84	4.45	5.33
cis_mRNA_up10k	8408	NA	n323957	MTCONS_00071911	chr9	131290645	131306374	1887	+	chr9	131314781	131396015	7974	+	*		0.9515	1	0	0.05	0.09	0	0.65	2.32
tran	NA	NA	n338331	MTCONS_00030180	chr1	161929297	161929914	615	+	chr17	1472556	1533118	9141	-		-414.32	0.7696	1	4.02	3.86	3.99	0.47	0.15	0.21
cis_mRNA_overlap	0	mRNA	n380577	MTCONS_00013887	chr11	61306988	61309731	1831	-	chr11	61281130	61310455	6862	-	*		-0.9007	-1	0	0.21	0.22	0.17	0.08	0
cis_mRNA_overlap	0	Lnc-CompleteIn-mRNAIntron	n340420	MTCONS_00055679	chr5	21581201	21582529	1329	+	chr5	21459589	21589481	5313	+	*		-0.8351	-1	0.08	0	0.02	0.4	0.53	0.43
tran	NA	NA	n325146	MTCONS_00052869	chr2	126584146	126592032	624	+	chr4	56914805	57200064	10656	+		-576.48	-0.9966	-1	1.54	3.35	2.26	19.73	16.31	18.62
cis_mRNA_up10k	2106	NA	n335584	MTCONS_00056211	chr5	79947242	79947865	624	-	chr5	79949970	80185496	4192	+	*		0.9128	1	1477.55	2123.6	1931.89	0	0.51	0.17
tran	NA	NA	n344961	MTCONS_00030180	chr7	95306193	95308564	831	+	chr17	1472556	1533118	9141	-		-817.8	-0.9979	-1	0.9	1.34	1.23	0.47	0.15	0.21
cis_mRNA_overlap	0	Lnc-AntiOverlap-mRNA	n338283	MTCONS_00033492	chr19	7597616	7601370	3755	-	chr19	7587496	7626657	6923	+	*		-0.8511	-1	1.96	2.02	1.67	0.08	0	0.12
cis_mRNA_overlap	0	Lnc-AntiOverlap-mRNA	n338283	MTCONS_00033493	chr19	7597616	7601370	3755	-	chr19	7587496	7626657	7355	+	*		-0.6759	-1	1.96	2.02	1.67	0.49	0.2	0.51
tran	4396764	NA	n385540	MTCONS_00072644	chr9	43685063	43890529	2392	+	chr9	39072764	39288300	5226	-		-1599.84	-0.9058	-1	2.31	3.15	1.84	0.54	0.41	0.92
cis_mRNA_overlap	0	mRNA	n385540	MTCONS_00071029	chr9	43685063	43890529	2392	+	chr9	43684758	43821492	4720	+		-1434.8	-0.8231	-1	2.31	3.15	1.84	1.47	1.24	3.8
cis_mRNA_dw20k	1244	NA	LTCONS_00072191	MTCONS_00073770	chr9	140083054	140084826	1232	+	chr9	140086069	140100097	2655	-	*		0.9968	1	14.54	10.45	12.85	1.14	0.76	1.01
tran	NA	NA	n324416	MTCONS_00041342	chr6	113856446	113860536	511	-	chr2	96487344	96658969	20870	-		-816.25	0.9198	1	1.77	15.3	19.65	2.25	2.77	3.55
tran	NA	NA	n324416	MTCONS_00041341	chr6	113856446	113860536	511	-	chr2	96487344	96549806	19081	-		-816.25	-0.7169	-1	1.77	15.3	19.65	6.48	6.43	5.38
cis_mRNA_overlap	0	Lnc-CompleteIn-mRNAIntron	n386571	MTCONS_00004913	chr1	46111452	46112357	906	-	chr1	46092955	46152943	3774	-	*		-0.9676	-1	0.63	0.68	0.66	3.12	2.22	2.78
cis_mRNA_overlap	0	Lnc-CompleteIn-mRNAIntron	n386571	MTCONS_00004915	chr1	46111452	46112357	906	-	chr1	46092955	46153796	3637	-	*		-0.9568	-1	0.63	0.68	0.66	3.07	0	0.38
cis_mRNA_overlap	0	Lnc-AntiOverlap-mRNA	n409629	MTCONS_00067793	chr7	151106322	151110132	702	+	chr7	151074148	151108086	6920	-	*		0.9897	1	0.31	0.07	0	5.57	4.44	4.34
tran	NA	NA	n368759	MTCONS_00069620	chr6	111599135	111599945	557	+	chr8	38266059	38326352	9657	-		-563.53	-0.9821	-1	1.98	1.61	1.3	1.01	1.48	1.67
tran	NA	NA	n368759	MTCONS_00008780	chr6	111599135	111599945	557	+	chr10	97515409	97637023	12392	+		-488.55	-0.9454	-1	1.98	1.61	1.3	0	0.01	0.04
cis_mRNA_overlap	0	Lnc-AntiOverlap-mRNA	n341363	MTCONS_00067793	chr7	151106322	151112174	2569	+	chr7	151074148	151108086	6920	-	*		-0.8018	-1	1	1.15	1.45	5.57	4.44	4.34
cis_mRNA_dw20k	11220	NA	n368391	MTCONS_00059086	chr5	180255984	180258946	2659	-	chr5	180270165	180288363	9238	-	*		-0.9752	-1	0.35	0.43	0.3	0.63	0.39	0.96
cis_mRNA_dw20k	18394	NA	LTCONS_00030389	MTCONS_00030393	chr17	7191544	7197546	4229	-	chr17	7215939	7233357	7328	-	*		0.9824	1	0.97	2.19	1.41	0.53	1.14	0.85
tran	NA	NA	n384848	MTCONS_00046828	chr6	27793690	27794715	588	+	chr22	30999407	31027721	7200	+		-427.62	0.9161	1	1	2.17	1.64	1.69	2.32	2.28
tran	NA	NA	n323898	MTCONS_00047226	chrX	33654546	33870318	495	+	chr22	50624319	50656144	12945	+		-479	0.8386	1	1.36	1.37	1.4	0.87	1.16	1.25
cis_mRNA_overlap	0	mRNA	n341542	MTCONS_00068696	chr8	79640608	79642045	1438	+	chr8	79578282	79645002	10955	+	*		-0.9966	-1	1.83	1.25	1.38	2.2	2.69	2.62
cis_mRNA_up10k	86	NA	n370906	MTCONS_00072430	chr9	23850848	23946157	903	+	chr9	23690089	23850763	4231	-	*		0.8452	1	0.16	0.07	0.04	72.38	68.88	58.29
tran	NA	NA	n366846	MTCONS_00008780	chr4	57488955	57492377	3323	+	chr10	97515409	97637023	12392	+		-3943.1	0.8555	1	0.61	0.72	0.76	0	0.01	0.04
tran	NA	NA	n326088	MTCONS_00052869	chr10	5297996	5303199	692	-	chr4	56914805	57200064	10656	+		-419.05	-0.7598	-1	19.7	23.04	22.98	19.73	16.31	18.62
tran	NA	NA	n378985	MTCONS_00052869	chr6	132223103	132241705	2305	+	chr4	56914805	57200064	10656	+		-1960.38	-0.9801	-1	0.03	0.05	0.04	19.73	16.31	18.62
cis_mRNA_up10k	122	NA	LTCONS_00006198	MTCONS_00002417	chr1	155017668	155023641	1510	-	chr1	155023762	155035324	3719	+	*		-0.7469	-1	0.42	0.67	0.44	1.57	0.55	0.81
cis_mRNA_up10k	115	NA	LTCONS_00006198	MTCONS_00002410	chr1	155017668	155023641	1510	-	chr1	155023755	155035324	3652	+	*		0.9873	1	0.42	0.67	0.44	0.29	1.75	0.64
tran	51125	NA	LTCONS_00055410	MTCONS_00055104	chr4	165837756	165838050	295	.	chr4	165889174	165898818	2592	-		-144.27	1	1	0.83	0	0.13	4.2	0	0.64
tran	NA	NA	LTCONS_00018856	MTCONS_00001489	chr12	8116219	8117218	1000	.	chr1	78245231	78345227	6485	+		-474.69	0.9945	0.866	0	0.03	0.03	0.21	1.95	1.75
tran	NA	NA	LTCONS_00018856	MTCONS_00001488	chr12	8116219	8117218	1000	.	chr1	78245231	78345227	6487	+		-474.69	-0.946	-0.866	0	0.03	0.03	3.27	1.06	1.79
tran	NA	NA	LTCONS_00018856	MTCONS_00012799	chr12	8116219	8117218	1000	.	chr11	118309253	118409866	27510	+		-825.51	0.9971	0.866	0	0.03	0.03	1.69	2.23	2.28
cis_mRNA_up10k	9263	NA	n345352	MTCONS_00032166	chr18	8695855	8696125	271	-	chr18	8705387	8832775	7747	+	*		0.7107	0.866	0	0.14	0.14	0.53	0.68	1.08
tran	NA	NA	n324054	MTCONS_00052179	chr9	88436882	88501231	530	+	chr3	197394817	197476612	10290	-		-515.29	-0.8763	-1	0.15	0.19	0.17	0.51	0.09	0.1
tran	NA	NA	n378509	MTCONS_00052177	chr15	55609382	55611243	866	-	chr3	197394817	197463843	10135	-		-522.83	0.7885	1	1.52	2.51	2.84	1.53	1.86	3.69
tran	38277	NA	n383372	MTCONS_00035590	chr19	12305853	12318387	347	+	chr19	12242097	12267577	9341	-		-224.79	0.7861	1	0	0.11	0.32	0.74	1.45	1.48
cis_mRNA_dw20k	15877	NA	n411584	MTCONS_00017797	chr12	56596288	56615715	5563	+	chr12	56631591	56652162	9141	-	*		0.9674	1	0.85	1.02	0.88	5.82	6.5	6.1
tran	NA	NA	LTCONS_00055344	MTCONS_00016474	chr4	187761862	187813160	8225	-	chr12	111051832	111068271	2328	+		-1237.22	0.908	1	8.21	8.11	8.9	1.08	0.6	1.52
cis_mRNA_overlap	0	Lnc-CompleteIn-mRNAIntron	n345474	MTCONS_00006673	chr1	173980078	173986779	1573	-	chr1	173899872	173991508	11790	-	*		-0.8033	-1	0.42	0.34	0.35	2.4	4.18	2.93
cis_mRNA_overlap	0	Lnc-CompleteIn-mRNAIntron	n345474	MTCONS_00006674	chr1	173980078	173986779	1573	-	chr1	173899872	173991518	11797	-	*		0.6729	1	0.42	0.34	0.35	1.65	0	1.47
cis_mRNA_overlap	0	mRNA	LTCONS_00061058	MTCONS_00061054	chr6	127587881	127609855	18321	+	chr6	127587732	127609855	2983	+		-1342.34	0.7052	1	1.64	2.12	2.09	1.45	1.92	1.55
tran	NA	NA	n324674	MTCONS_00008780	chr22	31053158	31069092	361	-	chr10	97515409	97637023	12392	+		-295.79	0.9922	1	0.42	0.48	0.89	0	0.01	0.04
cis_mRNA_dw20k	9160	NA	n324674	MTCONS_00046829	chr22	31053158	31069092	361	-	chr22	30999407	31043999	4636	+	*		-0.7424	-1	0.42	0.48	0.89	1.13	0.82	0.74
tran	NA	NA	n325077	MTCONS_00041342	chr20	5436521	5438648	358	-	chr2	96487344	96658969	20870	-		-198.35	-0.9472	-1	0.11	0.1	0	2.25	2.77	3.55
cis_mRNA_up10k	217	NA	LTCONS_00041825	MTCONS_00038966	chr2	135622097	135676176	3479	-	chr2	135676392	135716996	11918	+	*		0.8866	1	0.79	0.92	0.77	3.71	4.08	3.22
cis_mRNA_overlap	0	Lnc-CompleteIn-mRNAExon	n369674	MTCONS_00066721	chr7	50518334	50521012	2566	-	chr7	50511447	50521151	6698	-		-1278.47	-0.9686	-1	0.16	0.28	0	0.13	0.1	0.25
cis_mRNA_up10k	247	NA	n369674	MTCONS_00066719	chr7	50518334	50521012	2566	-	chr7	50511447	50518088	5182	-	*		0.822	0.866	0.16	0.28	0	0.56	0.8	0.56
cis_mRNA_dw20k	14647	NA	n406399	MTCONS_00067682	chr7	143509061	143533810	2527	-	chr7	143548456	143582466	5714	-	*		0.7677	1	0.13	0.27	0.1	38.86	39.48	36.31
cis_mRNA_dw20k	3537	NA	LTCONS_00011028	MTCONS_00010147	chr10	64919063	64922628	3566	.	chr10	64926164	65281636	9235	-	*		-0.9392	-1	0.73	0.82	0.83	0.45	0.29	0.17
tran	NA	NA	n324708	MTCONS_00076014	chr22	21998803	22000680	346	+	chrX	100263006	100307105	4402	-		-301.27	0.986	1	0.11	0.31	0	1.43	2.91	1.06
tran	NA	NA	n324708	MTCONS_00076013	chr22	21998803	22000680	346	+	chrX	100263006	100307105	4435	-		-301.27	-0.9491	-1	0.11	0.31	0	1.54	0.4	1.59
cis_mRNA_dw20k	1870	NA	n346311	MTCONS_00011078	chr11	566588	568437	1352	-	chr11	558978	564719	2784	+	*		-0.999	-1	0.63	0.59	0	2.54	2.55	3.33
cis_mRNA_up10k	7672	NA	n346311	MTCONS_00011083	chr11	566588	568437	1352	-	chr11	576108	612518	6552	+	*		-0.9619	-1	0.63	0.59	0	0.96	1.09	1.35
tran	NA	NA	n332853	MTCONS_00070785	chr13	50613139	50613550	412	-	chr9	19230566	19376252	10584	+		-431.5	0.7559	1	0.09	0.25	0.08	1.1	1.36	0.45
tran	NA	NA	n332853	MTCONS_00070786	chr13	50613139	50613550	412	-	chr9	19230719	19376252	10160	+		-431.5	-0.7466	-1	0.09	0.25	0.08	1.7	1.55	2.1
tran	NA	NA	n385983	MTCONS_00041103	chr10	24736419	24736989	571	-	chr2	74579851	74619214	15274	-		-638.82	0.9893	1	0.94	1.14	1.03	2.11	2.89	2.56

cis_mRNA_overlap	0	mRNA	LTCONS_00036205	MTCONS_00036202	chr19	38120564	38149835	11008	-	chr19	38120564	38147153	8329	-		-3810.96	-1	-1	0.56	0.23	0.28	0.76	1.01	0.97
cis_mRNA_overlap	0	CompleteIn-LncExon	LTCONS_00036205	MTCONS_00036203	chr19	38120564	38149835	11008	-	chr19	38120564	38147153	7953	-		-3710	0.8622	1	0.56	0.23	0.28	1.7	0.89	1.4
cis_mRNA_overlap	0	CompleteIn-LncExon	LTCONS_00036205	MTCONS_00036204	chr19	38120564	38149835	11008	-	chr19	38120564	38147191	7291	-		-3574.55	-0.7195	-1	0.56	0.23	0.28	3.37	3.84	3.44
tran	NA	NA	n344837	MTCONS_00030178	chr5	95338604	95339260	657	-	chr17	1472556	1532487	9223	-		-916.89	0.999	1	0.12	0.17	0.31	0.48	0.69	1.17
tran	NA	NA	n371446	MTCONS_00052301	chrX	49155629	49157930	386	+	chr4	53227	166058	11726	+		-248.08	-0.9996	-1	3.1	2.55	2.95	12.85	15.72	13.56
cis_mRNA_overlap	0	mRNA	LTCONS_00054029	MTCONS_00054030	chr4	5899181	5930272	8131	-	chr4	5899181	5969276	10312	-		-4321.71	-0.9855	-1	0.4	0.57	0.26	0.99	0.85	1.2
cis_mRNA_up10k	6431	NA	n332964	MTCONS_00061735	chr6	2859578	2859971	394	+	chr6	2833733	2853148	2613	-	*		0.9733	1	0	0.18	0.17	0	0.07	0.05
cis_mRNA_dw20k	11571	NA	n384790	MTCONS_00059085	chr5	180256959	180258595	1637	-	chr5	180270165	180288363	9165	-	*		0.8661	1	3.7	5.53	5.39	1.9	2.74	2.27
tran	NA	NA	n339280	MTCONS_00052301	chr3	32303234	32305396	2159	+	chr4	53227	166058	11726	+		-1816.51	0.9976	1	3.75	4.91	3.96	12.85	15.72	13.56
tran	NA	NA	n333910	MTCONS_00055677	chr6	26853161	26856999	272	-	chr5	21459589	21503711	2614	+		-203.71	-0.9994	-1	0.96	0	0.17	1.13	1.46	1.39
cis_mRNA_overlap	0	Lnc-AntiOverlap-mRNA	n346304	MTCONS_00065941	chr7	150101540	150104574	2666	-	chr7	150102113	150109813	4351	+	*		0.9449	0.866	0.13	0.07	0.09	0.13	0.11	0.11
cis_mRNA_overlap	0	AntiCompleteIn-mRNAIntron	n346304	MTCONS_00065938	chr7	150101540	150104574	2666	-	chr7	150076398	150109813	3635	+	*		-0.9165	-1	0.13	0.07	0.09	0	0.59	0.18
cis_mRNA_up10k	49	NA	LTCONS_00066560	MTCONS_00064648	chr7	35837627	35840579	2953	-	chr7	35840627	35946723	4370	+	*		-0.7735	-1	1.24	1.05	1.23	10.74	11.69	11.39
cis_mRNA_dw20k	3585	NA	n385926	MTCONS_00000066	chr1	995083	997436	673	+	chr1	955460	991499	8883	+	*		-0.8518	-1	0.11	0.2	0.05	6.72	6.5	8.79
cis_mRNA_dw20k	3585	NA	n385926	MTCONS_00000069	chr1	995083	997436	673	+	chr1	969154	991499	8181	+	*		-0.9579	-1	0.11	0.2	0.05	13.18	11.59	16.19
tran	NA	NA	n376789	MTCONS_00035589	chr20	5489720	5491696	887	-	chr19	12242097	12267577	9332	-		-691.68	0.9792	1	0.05	0.04	0.07	1.84	1.63	2.04
cis_mRNA_overlap	0	mRNA	n410669	MTCONS_00074936	chrX	122994017	123047829	7542	+	chrX	122993620	123047829	8359	+		-3544.81	-0.9051	-1	3.05	4.39	2.08	2.31	2	3.66
cis_mRNA_overlap	0	mRNA	n410669	MTCONS_00074935	chrX	122994017	123047829	7542	+	chrX	122993486	123047829	7457	+		-3392.23	0.9855	1	3.05	4.39	2.08	1.36	1.71	0.9
tran	48977561	NA	n387356	MTCONS_00055678	chr5	70500093	70516131	296	-	chr5	21459589	21522533	925	+		-221.22	0.925	1	12.38	4.96	0	1.01	0.76	0.03
tran	978472	NA	n387356	MTCONS_00057960	chr5	70500093	70516131	296	-	chr5	69416727	69521622	3590	-		-209.04	0.9642	1	12.38	4.96	0	1.21	0.65	0.55
cis_mRNA_up10k	224	NA	n386645	MTCONS_00011084	chr11	573809	575885	2077	-	chr11	576108	612518	6188	+	*		-0.9564	-1	2.21	1.83	2.09	1.17	1.67	1.18
tran	NA	NA	n373228	MTCONS_00052301	chr12	51365312	51366728	621	-	chr4	53227	166058	11726	+		-421.14	1	1	1	2.13	1.27	12.85	15.72	13.56
tran	NA	NA	n381152	MTCONS_00062880	chr1	83439567	83451891	1521	-	chr6	83874592	83903012	3126	-		-650.82	-0.9996	-1	0.07	0.08	0.16	0.36	0.31	0
cis_mRNA_dw20k	8137	NA	n373537	MTCONS_00016892	chr12	133169910	133172672	596	-	chr12	133135899	133161774	4985	+	*		0.9978	1	0.12	0.11	0.17	0.57	0.48	0.87
cis_mRNA_dw20k	9	NA	n363609	MTCONS_00000961	chr1	40932412	40938148	2382	-	chr1	40915760	40932404	5056	+	*		-0.7891	-1	1.92	2.29	1.8	0.67	0.53	1.41
tran	NA	NA	n366154	MTCONS_00046828	chr3	186528669	186543310	1832	-	chr22	30999407	31027721	7200	+		-1475.7	0.8392	1	2.48	4.82	3.43	1.69	2.32	2.28
tran	NA	NA	n379335	MTCONS_00053148	chr19	19945686	20008579	4287	-	chr4	91048528	92018810	14378	+		-2158.83	0.9997	1	0.36	0.2	0.31	0.99	0.93	0.97
tran	NA	NA	LTCONS_00067917	MTCONS_00053913	chr7	148787954	148789503	1550	.	chr4	432920	493550	7083	-		-1031.47	0.9916	1	1.24	1.72	1.37	1.42	1.97	1.5
cis_mRNA_dw20k	10353	NA	n377749	MTCONS_00008470	chr10	75012927	75014107	635	+	chr10	74927877	75002575	3742	+	*		0.975	1	0.26	0.22	0	0.76	0.72	0.65
tran	NA	NA	n387512	MTCONS_00076657	chr6	3266750	3267543	592	+	chrY	4898547	4981558	13244	+		-770.24	0.8664	1	3.63	2.72	3.06	1.57	0.93	1.47
cis_mRNA_dw20k	37	NA	n406455	MTCONS_00030268	chr17	4046462	4060991	1756	+	chr17	4061027	4168215	14872	-	*		0.9177	1	0.26	0.25	0.33	0.09	0.08	0.1
tran	NA	NA	n326616	MTCONS_00076654	chr1	48339974	48400130	645	-	chrY	4868267	4981558	12618	+		-488.94	-0.9083	-1	0.07	0.22	0	4.85	4.11	6.56
cis_mRNA_overlap	0	Lnc-CompleteIn-mRNAIntron	n325649	MTCONS_00020532	chr13	96622724	96629877	692	-	chr13	96453458	96705736	9294	-	*		0.9767	1	0.59	1.11	0.35	2.26	2.51	2.23
cis_mRNA_up10k	382	NA	n372797	MTCONS_00012950	chr11	125461043	125462350	469	-	chr11	125462731	125546150	10232	+	*		-0.9971	-1	0.48	0.87	0.43	1.33	1.16	1.37
tran	NA	NA	n372797	MTCONS_00041102	chr11	125461043	125462350	469	-	chr2	74579851	74619214	12873	-		-301.59	-0.7197	-1	0.48	0.87	0.43	2.72	2.64	3.07
cis_mRNA_overlap	0	Lnc-CompleteIn-mRNAExon	n337723	MTCONS_00075730	chrX	53659450	53713673	1036	-	chrX	53559011	53713709	15124	-		-700.76	-0.9392	-1	6.32	2.02	4.26	11.17	11.75	11.63
cis_mRNA_overlap	0	Lnc-AntiOverlap-mRNA	n340602	MTCONS_00056524	chr5	121772192	121814782	1424	-	chr5	121672519	121799805	3572	+	*		0.6904	0.866	0.02	0	0.02	1.63	0.98	1.14
cis_mRNA_overlap	0	Lnc-AntiOverlap-mRNA	n340602	MTCONS_00056522	chr5	121772192	121814782	1424	-	chr5	121647799	121799805	3672	+	*		-0.8756	-0.866	0.02	0	0.02	1.08	1.54	0.65
tran	NA	NA	n324813	MTCONS_00062262	chr21	33450808	33453286	449	+	chr6	28346545	28367544	7547	-		-268.32	0.9928	1	0	0.36	0.08	1.28	1.49	1.35
tran	NA	NA	n324813	MTCONS_00062263	chr21	33450808	33453286	449	+	chr6	28346545	28367580	4610	-		-268.32	-0.8737	-1	0	0.36	0.08	2.59	2.1	2.26
tran	NA	NA	n373032	MTCONS_00074936	chr12	54536059	54537698	566	+	chrX	122993620	123047829	8359	+		-489.62	-0.9793	-1	0.85	1.36	0	2.31	2	3.66
tran	NA	NA	n373032	MTCONS_00074935	chr12	54536059	54537698	566	+	chrX	122993486	123047829	7457	+		-489.62	0.9979	1	0.85	1.36	0	1.36	1.71	0.9
cis_mRNA_up10k	566	NA	n376782	MTCONS_00042911	chr20	2671873	2672959	486	-	chr20	2673524	2740754	2713	+	*		0.9684	1	0.2	0.49	0	5.74	7.39	2.96
cis_mRNA_overlap	0	mRNA	n410692	MTCONS_00043784	chr20	57226309	57254582	4146	+	chr20	57226147	57292108	6637	+	*		0.8788	1	0.02	0.67	0.69	1.94	2.32	2.7
cis_mRNA_overlap	0	mRNA	n410692	MTCONS_00043783	chr20	57226309	57254582	4146	+	chr20	57226147	57267661	2972	+	*		0.9973	1	0.02	0.67	0.69	3.13	4.6	4.78
cis_mRNA_dw20k	6089	NA	n408032	MTCONS_00059883	chr6	26421619	26430816	2483	+	chr6	26402465	26415531	4914	+	*		-0.997	-1	0.07	0.08	0.1	1.72	1.19	0.4
cis_mRNA_dw20k	6089	NA	n408032	MTCONS_00059884	chr6	26421619	26430816	2483	+	chr6	26402465	26415531	4935	+	*		0.9998	1	0.07	0.08	0.1	0	0.63	2.01
tran	NA	NA	n380222	MTCONS_00000612	chr8	90768463	90769592	696	-	chr1	24968184	24999772	7455	+		-485.3	0.9919	1	0.54	1.15	1.22	1.92	2.59	2.79
cis_mRNA_overlap	0	Lnc-AntiOverlap-mRNA	n342187	MTCONS_00063751	chr6	170886169	170888915	2747	+	chr6	170887587	170893780	3997	-	*		-0.9998	-1	0.19	0.14	0.15	0.25	3.26	2.72
cis_mRNA_overlap	0	Lnc-CompleteIn-mRNAIntron	n333537	MTCONS_00012369	chr11	73019305	73020007	703	+	chr11	73018523	73081227	6278	+	*		-0.7368	-1	0.73	0.36	0.38	1.39	1.58	1.44
tran	130634125	NA	n378336	MTCONS_00005933	chr1	16892131	16940083	3076	-	chr1	147574207	147610081	4489	-		-1678.35	0.9784	0.866	0.5	0.32	0.55	0.08	0	0.08
tran	NA	NA	n345810	MTCONS_00047230	chr18	55297591	55309277	647	+	chr22	50639408	50656144	7863	+		-705.02	0.9873	1	0.45	0.92	0.85	1.68	2.41	2.19
tran	NA	NA	n374322	MTCONS_00069359	chr14	74939815	74941018	935	-	chr8	10958954	11060192	12629	-		-766.1	0.8765	0.866	0.04	0.03	0.03	2.61	2.34	2.47
tran	NA	NA	n374322	MTCONS_00069358	chr14	74939815	74941018	935	-	chr8	10958954	11060192	12778	-		-766.1	-0.8059	-0.866	0.04	0.03	0.03	1	2.1	3.72
tran	NA	NA	n344905	MTCONS_00001552	chr6	56757552	56765419	798	-	chr1	84629892	84708194	8478	+		-649.78	0.9993	1	1.25	1.72	1.4	0.82	0.99	0.88
cis_mRNA_up10k	23	NA	LTCONS_00072197	MTCONS_00073770	chr9	140100119	140113844	5847	+	chr9	140086069	140100097	2655	-	*		-0.9771	-1	1.73	2.24	2	1.14	0.76	1.01
tran	NA	NA	n378313	MTCONS_00006068	chr9	103115360	103156151	2333	+	chr1	151740719	151763010	4208	-		-1542.38	1	1	1.75	1.61	1.78	1.08	0.72	1.16
tran	NA	NA	n378313	MTCONS_00006067	chr9	103115360	103156151	2333	+	chr1	151740719	151763010	4227	-		-1544.15	-0.9525	-1	1.75	1.61	1.78	5.39	6.43	4.51
tran	NA	NA	n326192	MTCONS_00027553	chr1	228795029	228797952	403	+	chr16	21448105	21531804	14985	-		-460.1	-0.7807	-1	2.6	1.14	1.41	2.77	4.17	3.06
tran	47969606	NA	LTCONS_00057966	MTCONS_00055678	chr5	69492138	69587380	3360	-	chr5	21459589	21522533	925	+		-628.86	0.7136	1	9.1	4.28	4.15	1.01	0.76	0.03
cis_mRNA_overlap	0	mRNA	LTCONS_00057966	MTCONS_00057959	chr5	69492138	69587380	3360	-	chr5	69416727	69497870	3769	-	*		-0.866	-1	9.1	4.28	4.15	0.16	0.28	0.41
cis_mRNA_overlap	0	mRNA	LTCONS_00057966	MTCONS_00057960	chr5	69492138	69587380	3360	-	chr5	69416727	69521622	3590	-	*		0.993	1	9.1	4.28	4.15	1.21	0.65	0.55
tran	NA	NA	n363298	MTCONS_00010010	chr1	95940293	95944912	2755	+	chr10	50641266	50818344	30532	-		-1613.35	-0.7655	-1	0.27	0.3	0.13	1.11	0.69	1.21

tran	NA	NA	n363298	MTCONS_00010011	chr1	95940293	95944912	2755	+	chr10	50641266	50818350	30643	-		-1613.35	0.8981	1	0.27	0.3	0.13	1.27	1.58	1.05
cis_mRNA_overlap	0	AntiCompleteIn-mRNAExon	LTCONS_00003514	MTCONS_00007287	chr1	227506375	227507232	858	+	chr1	227243429	227507232	9095	-		-1999.4	-0.9677	-1	0.21	0.08	0.26	1.4	1.89	1.39
cis_mRNA_overlap	0	AntiCompleteIn-mRNAExon	LTCONS_00003514	MTCONS_00007283	chr1	227506375	227507232	858	+	chr1	227177465	227507232	12052	-		-1999.4	0.9996	1	0.21	0.08	0.26	0.62	0.39	0.72
tran	NA	NA	n381972	MTCONS_00021582	chr12	133485301	133487790	645	+	chr14	89029253	89058791	7381	+		-602.34	-0.9428	-1	0.51	0.41	0.15	0.08	0.23	0.34
tran	NA	NA	n381972	MTCONS_00070588	chr12	133485301	133487790	645	+	chr8	145997260	146006382	3241	-		-612.7	-0.9959	-1	0.51	0.41	0.15	1.44	1.59	2.22
cis_mRNA_dw20k	5289	NA	n408890	MTCONS_00000711	chr1	28832455	28865708	2858	+	chr1	28696093	28827167	6497	+	*		0.9307	1	1.32	1.05	1.17	0.36	0	0.28
cis_mRNA_dw20k	5289	NA	n408890	MTCONS_00000709	chr1	28832455	28865708	2858	+	chr1	28696093	28827167	6525	+	*		0.9679	1	1.32	1.05	1.17	1.23	1.11	1.19
cis_mRNA_overlap	0	mRNA	n385795	MTCONS_00074680	chrX	100740408	100771939	2703	+	chrX	100740395	100790972	6386	+		-1369.72	0.8448	1	0.37	1.05	0.27	1.46	1.59	1.23
cis_mRNA_dw20k	10892	NA	n339222	MTCONS_00042763	chr2	242421371	242423364	1901	-	chr2	242434255	242448112	2765	-	*		-0.9928	-1	0.27	0.74	0.34	6.38	5.36	6.11
tran	NA	NA	n342898	MTCONS_00070588	chr12	66308500	66308797	293	+	chr8	145997260	146006382	3241	-		-497.97	0.945	1	4.17	5.89	7.66	1.44	1.59	2.22
cis_mRNA_dw20k	7278	NA	LTCONS_00014546	MTCONS_00014549	chr11	105865549	105869117	3569	-	chr11	105876394	105892992	5482	-	*		0.8608	1	1.14	0.88	0.89	1.53	0.36	0.99
cis_mRNA_dw20k	7278	NA	LTCONS_00014546	MTCONS_00014547	chr11	105865549	105869117	3569	-	chr11	105876394	105892992	5486	-	*		-0.6566	-1	1.14	0.88	0.89	2.6	3.25	2.71
cis_mRNA_overlap	0	mRNA	n341046	MTCONS_00012058	chr11	64037534	64055562	1274	+	chr11	64052914	64056972	2703	+		-1035.91	0.9941	1	0.13	0.16	0.42	2.35	2.42	2.68
cis_mRNA_overlap	0	mRNA	n341046	MTCONS_00012054	chr11	64037534	64055562	1274	+	chr11	64052267	64056972	2920	+		-1115.69	0.6883	1	0.13	0.16	0.42	1.1	1.77	1.89
tran	NA	NA	n323905	MTCONS_00054075	chrX	13908106	13916860	544	-	chr4	8363185	8431038	7806	-		-319.78	0.8096	1	1.01	1.53	1.4	0.11	0.43	0.17
tran	NA	NA	LTCONS_00047500	MTCONS_00052301	chr22	24005496	24008641	493	-	chr4	53227	166058	11726	+		-303.17	-0.9128	-1	3.28	1.94	2.45	12.85	15.72	13.56
cis_mRNA_dw20k	1412	NA	n371099	MTCONS_00071129	chr9	72009222	72013753	1872	-	chr9	71940460	72007811	3794	+	*		-0.983	-1	0.06	0.03	0	1.11	2.01	2.47
cis_mRNA_dw20k	1412	NA	n371099	MTCONS_00071128	chr9	72009222	72013753	1872	-	chr9	71939488	72007811	3673	+	*		0.9947	1	0.06	0.03	0	0.73	0.5	0.17
cis_mRNA_dw20k	5850	NA	n338094	MTCONS_00024180	chr15	86298435	86299468	592	-	chr15	86162295	86292586	10014	+	*		-0.919	-1	0	0.11	0.06	3.76	0.39	3.15
cis_mRNA_dw20k	5850	NA	n338094	MTCONS_00024177	chr15	86298435	86299468	592	-	chr15	85923827	86292586	13274	+	*		-0.9975	-1	0	0.11	0.06	2.52	1.17	1.7
cis_mRNA_overlap	0	Lnc-CompleteIn-mRNAExon	n337962	MTCONS_00005065	chr1	55559717	55613437	4245	-	chr1	55531964	55680438	10450	-		-2151.73	-0.9998	-1	1.37	0.18	2.49	12.17	13.87	10.67
cis_mRNA_overlap	0	Lnc-CompleteIn-mRNAExon	n337962	MTCONS_00005066	chr1	55559717	55613437	4245	-	chr1	55531964	55681039	11568	-		-2148.49	-0.9427	-1	1.37	0.18	2.49	0.62	0.76	0.59
cis_mRNA_overlap	0	Lnc-CompleteIn-mRNAExon	n337962	MTCONS_00005067	chr1	55559717	55613437	4245	-	chr1	55531964	55681045	11060	-		-2074.69	0.9695	1	1.37	0.18	2.49	1.38	1.08	2.09
tran	NA	NA	n370935	MTCONS_00069239	chr9	67340517	67343501	394	+	chr8	155778	182318	8477	-		-349.37	-0.822	-0.866	0.98	0.98	0.96	0.1	0.22	0.31
tran	NA	NA	n370935	MTCONS_00069241	chr9	67340517	67343501	394	+	chr8	155778	182318	8251	-		-349.37	0.982	0.866	0.98	0.98	0.96	0.1	0.08	0
cis_mRNA_overlap	0	Lnc-AntiOverlap-mRNA	n377826	MTCONS_00041103	chr2	74612651	74620023	1273	+	chr2	74579851	74619214	15274	-	*		0.9664	1	0	0.4	0.14	2.11	2.89	2.56
tran	NA	NA	n368560	MTCONS_00053148	chr5	87484995	87485986	420	-	chr4	91048528	92018810	14378	+		-555.08	-0.7936	-1	0.27	0.42	0.41	0.99	0.93	0.97
cis_mRNA_dw20k	2412	NA	n342366	MTCONS_00034272	chr19	37742828	37754515	1699	+	chr19	37708321	37740417	8180	+	*		-0.955	-1	0.25	0.14	0.04	0.07	0.42	0.51
cis_mRNA_dw20k	2412	NA	n342366	MTCONS_00034273	chr19	37742828	37754515	1699	+	chr19	37709374	37740417	8724	+	*		0.9936	1	0.25	0.14	0.04	1.19	0.96	0.65
tran	NA	NA	LTCONS_00024227	MTCONS_00065068	chr15	90620771	90621146	376	+	chr7	75831172	75916605	7637	+		-248.81	0.8336	1	0.1	0.19	0.46	1.07	1.51	1.64
cis_mRNA_overlap	0	Lnc-AntiOverlap-mRNA	LTCONS_00064611	MTCONS_00066513	chr7	32766662	32802650	11793	+	chr7	32625309	32768322	1911	-	*		-0.7644	-1	0.87	1.39	0.94	0.81	0.29	0.41
tran	NA	NA	LTCONS_00012573	MTCONS_00002260	chr11	93921564	93922676	1113	+	chr1	150459876	150486361	5394	+		-660.56	0.9209	1	0.54	0.62	0.92	0.33	0.54	0.7
cis_mRNA_overlap	0	CompleteIn-LncIntron	n387352	MTCONS_00056071	chr5	69199603	69770097	5137	+	chr5	69696973	69746189	4716	+	*		0.9312	1	3.49	4.16	3.52	0	1.66	0.67
cis_mRNA_overlap	0	Lnc-CompleteIn-mRNAExon	n385794	MTCONS_00074678	chrX	100740408	100754031	1861	+	chrX	100740359	100771939	3421	+		-1036.31	-0.8662	-1	0.1	0.65	1.07	1.52	1.41	0.24
tran	NA	NA	n324705	MTCONS_00027553	chr22	22184681	22185789	477	+	chr16	21448105	21531804	14985	-		-499.48	0.7737	1	0.06	0.29	0.25	2.77	4.17	3.06
tran	NA	NA	n376792	MTCONS_00037013	chr20	11849692	11859217	659	-	chr19	58411293	58429710	12199	-		-650.52	0.9778	1	0.06	0.1	0.17	0.52	0.55	0.7
tran	NA	NA	LTCONS_00042561	MTCONS_00036578	chr2	223745026	223807923	648	-	chr19	48710992	48759203	5393	-		-326.01	-0.9817	-1	1.57	1.18	0.52	0.82	1.03	1.21
cis_mRNA_overlap	0	Lnc-AntiOverlap-mRNA	n380405	MTCONS_00015444	chr12	26107741	26112698	1121	-	chr12	26111964	26225956	5742	+	*		0.8302	1	0	0.08	0.11	0.34	0.67	2
cis_mRNA_dw20k	1029	NA	n326368	MTCONS_00006188	chr1	154924113	154928413	4120	-	chr1	154929441	154943223	7183	-	*		0.9787	1	0.11	0.09	0.12	1.95	0	2.26
cis_mRNA_dw20k	1029	NA	n326368	MTCONS_00006189	chr1	154924113	154928413	4120	-	chr1	154929441	154943229	7186	-	*		-0.9463	-1	0.11	0.09	0.12	0.01	2.01	0
cis_mRNA_overlap	0	Lnc-CompleteIn-mRNAExon	n410542	MTCONS_00029890	chr17	73975298	74002080	3330	+	chr17	73975251	74002080	3959	+		-2550.96	-0.9594	-0.866	1.58	0	1.58	1.01	2.63	1.48
tran	NA	NA	n325852	MTCONS_00035589	chr10	131457098	131457689	495	+	chr19	12242097	12267577	9332	-		-482.48	0.9153	1	0.18	0.16	0.31	1.84	1.63	2.04
tran	NA	NA	n376745	MTCONS_00024928	chr20	48788959	48790099	688	+	chr15	64457716	64648442	8298	-		-660.23	-0.9982	-1	0	0.61	0.19	2.13	0.22	1.64
tran	NA	NA	n376745	MTCONS_00024930	chr20	48788959	48790099	688	+	chr15	64457716	64648442	8274	-		-660.23	0.9858	1	0	0.61	0.19	2.41	4.69	3.46
tran	NA	NA	n368643	MTCONS_00008780	chr6	94416801	94486199	2620	+	chr10	97515409	97637023	12392	+		-2720.11	0.9608	1	0.13	0.16	0.19	0	0.01	0.04
tran	NA	NA	n338461	MTCONS_00041102	chr14	95625023	95646268	783	+	chr2	74579851	74619214	12873	-		-860.16	-0.8818	-1	3.02	3.32	2.84	2.72	2.64	3.07
cis_mRNA_overlap	0	Lnc-CompleteIn-mRNAExon	n407570	MTCONS_00055678	chr5	21459589	21497305	703	+	chr5	21459589	21522533	925	+		-438.25	0.7082	1	28.79	19.83	19.67	1.01	0.76	0.03
tran	47919423	NA	n407570	MTCONS_00057960	chr5	21459589	21497305	703	+	chr5	69416727	69521622	3590	-		-443.14	0.9921	1	28.79	19.83	19.67	1.21	0.65	0.55
tran	NA	NA	LTCONS_00076589	MTCONS_00020679	chrX	48469309	48469700	392	.	chr13	114109147	114145035	4320	-		-373.61	-0.7856	-1	1.94	2.13	2.18	0.76	0.67	0.29
tran	NA	NA	LTCONS_00076589	MTCONS_00020678	chrX	48469309	48469700	392	.	chr13	114109147	114145033	10788	-		-373.61	0.9952	1	1.94	2.13	2.18	1.59	1.73	1.79
tran	NA	NA	n405979	MTCONS_00002444	chr7	76099337	76104317	4981	-	chr1	155278539	155290457	1359	+		-856.67	-0.9292	-1	0.16	0.26	0.32	88.27	78.59	55.17
tran	NA	NA	n405979	MTCONS_00002443	chr7	76099337	76104317	4981	-	chr1	155278539	155290457	1449	+		-913.83	-0.7932	-1	0.16	0.26	0.32	25.56	25.4	14.17
tran	NA	NA	n324002	MTCONS_00052872	chr9	108386244	108394054	371	-	chr4	57081966	57200064	10458	+		-208.86	0.9982	1	1.56	1.81	2.85	15.47	18.33	26.84
tran	NA	NA	n324002	MTCONS_00041342	chr9	108386244	108394054	371	-	chr2	96487344	96658969	20870	-		-442.63	0.9748	1	1.56	1.81	2.85	2.25	2.77	3.55
cis_mRNA_overlap	0	AntiCompleteIn-mRNAIntron	n324002	MTCONS_00071535	chr9	108386244	108394054	371	-	chr9	108335600	108403444	7801	+	*		-0.9832	-1	1.56	1.81	2.85	0.19	0.15	0.08
tran	NA	NA	n324002	MTCONS_00041341	chr9	108386244	108394054	371	-	chr2	96487344	96549806	19081	-		-442.63	-0.9897	-1	1.56	1.81	2.85	6.48	6.43	5.38
cis_mRNA_overlap	0	mRNA	n407569	MTCONS_00055677	chr5	21459589	21474565	1648	+	chr5	21459589	21503711	2614	+	*		-0.7189	-1	1.69	0.62	1.6	1.13	1.46	1.39
cis_mRNA_overlap	0	mRNA	n407569	MTCONS_00055679	chr5	21459589	21474565	1648	+	chr5	21459589	21589481	5313	+		-892.85	-0.9893	-1	1.69	0.62	1.6	0.4	0.53	0.43
cis_mRNA_up10k	143	NA	n410116	MTCONS_00038961	chr2	135624203	135676176	1405	-	chr2	135676318	135716996	7397	+	*		-0.9983	-1	3.03	1.73	2.47	2.8	3.32	3.05

cis_mRNA_overlap	0	AntiCompleteIn-mRNAIntron	n377921	MTCONS_00041103	chr2	74612613	74614153	584	+	chr2	74579851	74619214	15274	-	*		0.9208	1	0	0.68	0.15	2.11	2.89	2.56
tran	NA	NA	n374137	MTCONS_00052301	chr14	41423822	41432050	392	+	chr4	53227	166058	11726	+		-283.44	-0.9858	-1	5.68	3.65	5.52	12.85	15.72	13.56
tran	NA	NA	n385030	MTCONS_00052301	chr6	144175206	144177090	1885	+	chr4	53227	166058	11726	+		-893.2	0.9937	1	4.87	5.16	4.91	12.85	15.72	13.56
cis_mRNA_overlap	0	mRNA	n336988	MTCONS_00023550	chr15	42111270	42111610	341	+	chr15	42064664	42120056	10042	+		-273.83	0.9309	1	0.12	0.53	0.21	1.16	1.54	1.37
cis_mRNA_overlap	0	mRNA	n336988	MTCONS_00023545	chr15	42111270	42111610	341	+	chr15	41952533	42120056	24009	+		-273.83	0.9976	1	0.12	0.53	0.21	1.64	2.17	1.72
cis_mRNA_up10k	8667	NA	n336988	MTCONS_00023556	chr15	42111270	42111610	341	+	chr15	42120276	42140436	6812	+	*		0.8286	1	0.12	0.53	0.21	3.68	5.04	4.7
tran	NA	NA	n411585	MTCONS_00031389	chr15	67807260	67814182	793	-	chr17	49039439	49124242	7991	-		-706.46	-0.9976	-1	0.1	0	0.25	13.85	14.82	11.95
tran	NA	NA	n385959	MTCONS_00055882	chr1	149576161	149576700	540	+	chr5	49962091	50142704	8119	+		-389.32	0.8901	1	0.75	0.28	0.16	0.82	0.79	0.74
cis_mRNA_overlap	0	Lnc-AntiOverlap-mRNA	n340563	MTCONS_00058231	chr5	96120481	96121703	993	+	chr5	96096514	96271606	7562	-	*		-0.9696	-1	0.18	0	0.06	0	0.63	0.57
tran	47900757	NA	n406552	MTCONS_00055678	chr5	69423289	69521622	1016	-	chr5	21459589	21522533	925	+		-480.84	0.9569	1	7.54	4.26	1.19	1.01	0.76	0.03
cis_mRNA_overlap	0	mRNA	n406552	MTCONS_00057959	chr5	69423289	69521622	1016	-	chr5	69416727	69497870	3769	-	*		-0.9991	-1	7.54	4.26	1.19	0.16	0.28	0.41
cis_mRNA_overlap	0	mRNA	n406552	MTCONS_00057960	chr5	69423289	69521622	1016	-	chr5	69416727	69521622	3590	-		-526.23	0.9347	1	7.54	4.26	1.19	1.21	0.65	0.55
cis_mRNA_overlap	0	AntiCompleteIn-mRNAIntron	n384179	MTCONS_00047902	chr22	42665759	42670867	1241	+	chr22	42555898	42739561	7507	-	*		0.9685	1	0.08	0.05	0	5.32	4.82	1.72
cis_mRNA_overlap	0	AntiCompleteIn-mRNAIntron	n384179	MTCONS_00047901	chr22	42665759	42670867	1241	+	chr22	42555898	42680076	7674	-	*		-0.9829	-1	0.08	0.05	0	2.36	2.62	3.63
cis_mRNA_dw20k	1970	NA	n340791	MTCONS_00031552	chr17	61778207	61780030	1824	+	chr17	61781999	61819330	1402	-	*		0.9238	1	4.65	4.46	4.72	0.26	0.16	0.42
cis_mRNA_overlap	0	Lnc-AntiOverlap-mRNA	LTCONS_00050602	MTCONS_00048630	chr3	44598986	44666429	315	-	chr3	44596625	44614246	4108	+	*		0.9996	1	1.52	0.58	1.02	1.3	0.94	1.1
tran	84548925	NA	n326768	MTCONS_00001552	chr1	79414	80968	1319	-	chr1	84629892	84708194	8478	+		-654.36	-0.8961	-1	0.55	0.3	0.36	0.82	0.99	0.88
cis_mRNA_overlap	0	Lnc-AntiOverlap-mRNA	LTCONS_00010542	MTCONS_00008780	chr10	97512963	97667703	4481	-	chr10	97515409	97637023	12392	+	*		0.9985	1	0.34	0.37	0.44	0	0.01	0.04
cis_mRNA_overlap	0	mRNA-AntiCompleteIn-LncIntron	n379409	MTCONS_00035725	chr19	15050246	16748905	1493	+	chr19	15071211	15090703	3128	-	*		-0.9762	-1	0.05	0.08	0.06	3.23	3.07	3.21
cis_mRNA_dw20k	1564	NA	LTCONS_00000262	MTCONS_00000257	chr1	7831329	7841499	4268	+	chr1	7764855	7829766	5074	+	*		-0.8779	-1	0	0.44	1.05	12.08	9	8.55
cis_mRNA_overlap	0	Lnc-AntiOverlap-mRNA	n342082	MTCONS_00062928	chr6	88273570	88275290	1721	+	chr6	88222436	88299735	3653	-	*		-0.8885	-0.866	0	0.02	0.02	4.09	3.35	3.69
cis_mRNA_dw20k	11485	NA	n385674	MTCONS_00073235	chr9	111921538	111922745	1208	-	chr9	111934229	112083021	5486	-	*		-0.6333	-1	0.52	0.53	0.44	0.82	0.27	0.86
tran	3175771	NA	LTCONS_00073834	MTCONS_00071093	chr9	66667681	66668398	718	.	chr9	69844168	69851908	6749	+		-480.18	0.989	1	1.23	0.47	1.37	1.74	1.03	2.04
tran	NA	NA	n325818	MTCONS_00001489	chr11	130628527	130675876	584	+	chr1	78245231	78345227	6485	+		-325.11	0.9741	1	0.11	0.2	0.17	0.21	1.95	1.75
tran	NA	NA	n325818	MTCONS_00001488	chr11	130628527	130675876	584	+	chr1	78245231	78345227	6487	+		-325.72	-1	-1	0.11	0.2	0.17	3.27	1.06	1.79
tran	NA	NA	n377737	MTCONS_00053148	chr11	3495285	3498281	2574	-	chr4	91048528	92018810	14378	+		-2895.04	-0.8773	-1	0.04	0.13	0.11	0.99	0.93	0.97
tran	130634125	NA	LTCONS_00005931	MTCONS_00004232	chr1	147574207	147578821	3331	-	chr1	16888816	16940083	7373	-		-1721.76	0.9946	1	3.48	5.27	4.36	3.93	6.2	5.25
cis_mRNA_dw20k	1630	NA	LTCONS_00005931	MTCONS_00005935	chr1	147574207	147578821	3331	-	chr1	147580450	147610081	2781	-	*		0.9687	1	3.48	5.27	4.36	2.6	3.12	2.74
cis_mRNA_overlap	0	mRNA	LTCONS_00005931	MTCONS_00005933	chr1	147574207	147578821	3331	-	chr1	147574207	147610081	4489	-		-1679.01	-0.8708	-0.866	3.48	5.27	4.36	0.08	0	0.08
tran	423483	NA	LTCONS_00005931	MTCONS_00005954	chr1	147574207	147578821	3331	-	chr1	148002303	148025848	4803	-		-1641.82	-0.9806	-1	3.48	5.27	4.36	1.19	0.97	1.12
tran	2203953	NA	LTCONS_00005931	MTCONS_00002114	chr1	147574207	147578821	3331	-	chr1	145304210	145370255	11091	+		-1750.45	0.948	1	3.48	5.27	4.36	0.52	1.07	0.63
tran	427957	NA	LTCONS_00005931	MTCONS_00005956	chr1	147574207	147578821	3331	-	chr1	148006777	148025848	3003	-		-1459.48	0.9991	1	3.48	5.27	4.36	12.58	18.82	15.88
tran	48163685	NA	LTCONS_00027569	MTCONS_00028041	chr16	21845895	21846506	612	-	chr16	70010190	70099880	4224	-		-367.31	0.8262	1	3.15	3.07	2.1	0.45	0.3	0.21
cis_mRNA_overlap	0	mRNA	n410507	MTCONS_00038961	chr2	135676392	135716915	6909	+	chr2	135676318	135716996	7397	+		-2558.74	-0.8854	-1	0.84	0.35	0.83	2.8	3.32	3.05
tran	NA	NA	n365192	MTCONS_00020679	chr2	85190235	85194331	930	+	chr13	114109147	114145035	4320	-		-556.62	0.6681	1	0.43	0.11	0.1	0.76	0.67	0.29
cis_mRNA_dw20k	8087	NA	n324280	MTCONS_00063752	chr6	170876210	170879501	758	+	chr6	170887587	170893780	3206	-	*		-0.9996	-1	0.85	0.99	1.1	5.65	4.29	3.11
cis_mRNA_up10k	387	NA	n384745	MTCONS_00056736	chr5	139780100	139781013	914	-	chr5	139781399	139929653	11917	+	*		0.9745	0.866	0	3.71	2.84	0.28	0.46	0.46
cis_mRNA_up10k	387	NA	n384745	MTCONS_00056734	chr5	139780100	139781013	914	-	chr5	139781399	139922455	11259	+	*		-0.9745	-0.866	0	3.71	2.84	0.52	0.43	0.43
cis_mRNA_dw20k	203	NA	n338211	MTCONS_00022032	chr14	21852451	21853079	629	+	chr14	21853281	21905457	8850	-	*		0.6812	1	0.17	0.11	0.1	2.61	2.53	1.83
cis_mRNA_dw20k	5427	NA	n372975	MTCONS_00015240	chr12	8941118	8948420	1785	+	chr12	8850493	8935692	5471	+	*		0.7083	1	7.09	1.19	1.51	6.49	4.17	5.97
cis_mRNA_dw20k	16875	NA	n407843	MTCONS_00026724	chr16	67708436	67753273	1729	-	chr16	67679030	67691562	4488	+	*		0.8761	1	0.61	0.57	2.28	0.97	0.96	0.98
tran	NA	NA	LTCONS_00025902	MTCONS_00067128	chr16	5289760	5517249	380	+	chr7	97481372	97602630	4004	-		-391.67	0.8755	1	1.64	5.29	0.75	1.09	1.26	0.78
tran	NA	NA	n345168	MTCONS_00010010	chr12	8145284	8169776	531	-	chr10	50641266	50818344	30532	-		-490.3	0.7591	1	0.07	0.06	0.12	1.11	0.69	1.21
tran	NA	NA	n345168	MTCONS_00010011	chr12	8145284	8169776	531	-	chr10	50641266	50818350	30643	-		-490.3	-0.8938	-1	0.07	0.06	0.12	1.27	1.58	1.05
tran	NA	NA	n407210	MTCONS_00058320	chr7	23530007	23531031	1025	+	chr5	114597548	114632458	7737	-		-706.03	-0.8376	-1	7.98	9.37	6.97	1.06	1.05	1.29
cis_mRNA_overlap	0	mRNA	LTCONS_00061056	MTCONS_00061055	chr6	127587881	127609855	7701	+	chr6	127587732	127609855	2714	+		-1242.11	-0.8879	-1	1.35	1.52	1.18	1.11	0	1.17
cis_mRNA_overlap	0	CompleteIn-LncExon	LTCONS_00061056	MTCONS_00061057	chr6	127587881	127609855	7701	+	chr6	127587881	127609855	2395	+		-1077.67	0.9782	1	1.35	1.52	1.18	1.07	2.13	0.58
tran	NA	NA	n336822	MTCONS_00014548	chr8	54870538	54870827	290	+	chr11	105876394	105892992	5127	-		-350.47	-0.8446	-1	0.19	0.23	0.68	2.08	1.78	1.58
tran	NA	NA	n325693	MTCONS_00052872	chr13	74024981	74028022	825	+	chr4	57081966	57200064	10458	+		-470.62	0.9835	1	7.01	7.67	8.6	15.47	18.33	26.84
tran	9816574	NA	LTCONS_00066689	MTCONS_00064648	chr7	45763296	45808617	2300	-	chr7	35840627	35946723	4370	+		-1046.06	-0.9784	-1	5.59	4.55	4.67	10.74	11.69	11.39
tran	9816574	NA	LTCONS_00066689	MTCONS_00064649	chr7	45763296	45808617	2300	-	chr7	35870348	35946723	4827	+		-1054.43	-0.9139	-1	5.59	4.55	4.67	0.81	1.47	1.14
tran	NA	NA	n324094	MTCONS_00008780	chr9	45048388	45052528	931	+	chr10	97515409	97637023	12392	+		-984.8	0.9918	1	0.45	0.56	0.75	0	0.01	0.04
tran	NA	NA	n365929	MTCONS_00005218	chr3	27862979	27932792	543	+	chr1	75667816	75843119	7850	-		-316.86	-0.9835	-1	1.81	1.29	2.59	18.73	24.06	14.51
cis_mRNA_overlap	0	mRNA	n410458	MTCONS_00024296	chr15	93426073	93441977	1787	+	chr15	93425950	93571283	12699	+		-970.62	-0.9194	-1	7.19	5.49	6.35	6.23	6.72	6.29
tran	NA	NA	n381926	MTCONS_00030179	chr12	100597447	100615569	748	-	chr17	1472556	1532487	9190	-		-589.26	0.9726	1	0.59	0.71	0.45	2.11	2.49	1.92
cis_mRNA_overlap	0	Lnc-CompleteIn-mRNAExon	n385304	MTCONS_00067737	chr7	149244245	149321881	3583	-	chr7	149239631	149321881	9298	-		-2192.88	0.6309	1	2.49	3.53	2.5	1.03	1.34	1.29
tran	1450695	NA	n406149	MTCONS_00065028	chr7	72569012	72621336	3454	+	chr7	74072030	74175022	5221	+		-1717.57	-0.9996	-1	84.42	80.2	73.02	24.02	25.02	26.56
cis_mRNA_up10k	568	NA	n339955	MTCONS_00009482	chr10	7453561	7455429	1868	+	chr10	7200572	7452994	7877	-	*		0.9917	1	0.07	0.18	0.21	1.63	1.81	1.9
cis_mRNA_up10k	642	NA	n339955	MTCONS_00009480	chr10	7453561	7455429	1868	+	chr10	7200572	7452920	8014	-	*		0.9986	1	0.07	0.18	0.21	1.18	1.57	1.71
cis_mRNA_dw20k	13600	NA	n341936	MTCONS_00015970	chr12	58017197	58018656	1460	-	chr12	57998112	58003598	2375	+	*		-0.9899	-1	12.15	12.95	13.33	2.34	1.05	0.75
cis_mRNA_overlap	0	Lnc-AntiOverlap-mRNA	n341936	MTCONS_00015973	chr12	58017197	58018656	1460	-	chr12	58005162	58020030	6160	+	*		0.9814	1	12.15	12.95	13.33	0.74	1.49	1.61

cis_mRNA_dw20k	21	NA	n341936	MTCONS_00015976	chr12	58017197	58018656	1460	-	chr12	58013573	58017177	1512	+	*		0.9996	1	12.15	12.95	13.33	1.97	2.47	2.68
cis_mRNA_overlap	0	Lnc-CompleteIn-mRNAIntron	n336268	MTCONS_00059811	chr6	25439997	25440485	489	+	chr6	25279509	25620758	5608	+	*		-0.7597	-0.866	0.08	0.14	0.14	1.37	0.07	0.93
cis_mRNA_overlap	0	Lnc-CompleteIn-mRNAIntron	n336268	MTCONS_00059810	chr6	25439997	25440485	489	+	chr6	25279318	25620758	5824	+	*		0.9972	0.866	0.08	0.14	0.14	0.49	1.69	1.59
cis_mRNA_up10k	628	NA	n372465	MTCONS_00011059	chr11	287702	288773	749	+	chr11	289400	298192	6936	+	*		0.996	1	0	0.13	0.04	0.55	1.62	0.79
tran	NA	NA	n368205	MTCONS_00074936	chr5	179085079	179090159	588	+	chrX	122993620	123047829	8359	+		-545.23	0.9919	1	1.29	1.1	1.72	2.31	2	3.66
tran	NA	NA	n368205	MTCONS_00074935	chr5	179085079	179090159	588	+	chrX	122993486	123047829	7457	+		-545.23	-0.99	-1	1.29	1.1	1.72	1.36	1.71	0.9
cis_mRNA_dw20k	2801	NA	n377939	MTCONS_00039270	chr2	177040722	177053268	984	-	chr2	177017071	177037922	4463	+	*		0.9212	1	0.24	0.52	0.19	1.57	2.39	0.7
cis_mRNA_dw20k	2801	NA	n377939	MTCONS_00039272	chr2	177040722	177053268	984	-	chr2	177021750	177037922	4387	+	*		0.9035	1	0.24	0.52	0.19	0.99	1.46	0.41
cis_mRNA_dw20k	2801	NA	n377939	MTCONS_00039271	chr2	177040722	177053268	984	-	chr2	177021750	177037922	2734	+	*		0.9129	1	0.24	0.52	0.19	4.47	6.46	2.2
tran	NA	NA	n326331	MTCONS_00052081	chr1	171142663	171143447	658	+	chr3	194995354	195163817	13560	-		-320.44	0.9795	1	0.06	0.2	0.05	0.3	0.79	0.12
tran	NA	NA	n326279	MTCONS_00069239	chr1	197135770	197173181	756	-	chr8	155778	182318	8477	-		-420.3	0.9215	1	0.27	0.35	0.64	0.1	0.22	0.31
tran	NA	NA	n326279	MTCONS_00069241	chr1	197135770	197173181	756	-	chr8	155778	182318	8251	-		-420.3	-0.9999	-1	0.27	0.35	0.64	0.1	0.08	0
tran	NA	NA	n334389	MTCONS_00001489	chr6	4078287	4078486	200	-	chr1	78245231	78345227	6485	+		-160.92	0.8647	1	1.21	2.81	1.86	0.21	1.95	1.75
tran	NA	NA	n334389	MTCONS_00001491	chr6	4078287	4078486	200	-	chr1	78245281	78506196	5323	+		-160.92	-0.9158	-1	1.21	2.81	1.86	3.7	0	0.84
tran	NA	NA	n334389	MTCONS_00001488	chr6	4078287	4078486	200	-	chr1	78245231	78345227	6487	+		-160.92	-0.9549	-1	1.21	2.81	1.86	3.27	1.06	1.79
cis_mRNA_overlap	0	mRNA	LTCONS_00069236	MTCONS_00069240	chr8	155778	167642	9202	-	chr8	155778	182318	8373	-		-3781.84	0.9979	1	0.28	0.58	0.51	0	0.17	0.12
tran	NA	NA	n370197	MTCONS_00030180	chr8	129571390	129573380	807	-	chr17	1472556	1533118	9141	-		-667.73	-0.7845	-1	0.26	0.64	0.34	0.47	0.15	0.21
cis_mRNA_overlap	0	Lnc-CompleteIn-mRNAIntron	n337907	MTCONS_00004914	chr1	46103195	46104558	1362	-	chr1	46092955	46152943	3819	-	*		0.9856	1	0.38	0.56	0.68	1.03	1.55	2.18
cis_mRNA_dw20k	7724	NA	n409679	MTCONS_00024919	chr15	63879909	63893026	1722	-	chr15	63900749	64126180	16300	-	*		-0.9561	-1	0.24	0.4	0.34	23.02	21.16	21.33
tran	NA	NA	n325476	MTCONS_00052870	chr2	3983120	3999535	2454	-	chr4	56914919	57200064	10678	+		-1928	-0.9614	-1	0.62	0.54	0.77	0.92	2.25	0
cis_mRNA_dw20k	4489	NA	n340024	MTCONS_00008252	chr10	49651879	49653859	1981	-	chr10	49514684	49647391	5838	+	*		-0.9473	-1	0.57	0.7	1.59	23.98	21.75	18.8
cis_mRNA_dw20k	4489	NA	n340024	MTCONS_00008254	chr10	49651879	49653859	1981	-	chr10	49514701	49647391	6010	+	*		-0.9937	-1	0.57	0.7	1.59	5.5	5.06	3.65
cis_mRNA_up10k	7984	NA	n409152	MTCONS_00051602	chr3	141876369	141944449	10920	-	chr3	141663270	141868386	9883	-	*		-0.9947	-1	1.8	1.88	2.05	0.62	0.48	0.28
cis_mRNA_overlap	0	Lnc-CompleteIn-mRNAExon	n407311	MTCONS_00007513	chr1	245003940	245010243	5045	-	chr1	244999078	245027879	20106	-		-2246.49	0.9456	1	1.51	1.9	1.58	0.75	1	0.87
cis_mRNA_overlap	0	Lnc-CompleteIn-mRNAExon	n407311	MTCONS_00007514	chr1	245003940	245010243	5045	-	chr1	244999078	245027879	20163	-		-2246.49	0.9269	1	1.51	1.9	1.58	0.62	0.81	0.72
cis_mRNA_overlap	0	Lnc-CompleteIn-mRNAExon	n410563	MTCONS_00035590	chr19	12242168	12267546	3415	-	chr19	12242097	12267577	9341	-		-1775.75	0.7408	1	0	0.07	0.25	0.74	1.45	1.48
cis_mRNA_dw20k	1295	NA	n344971	MTCONS_00067477	chr7	126986884	127009008	7167	-	chr7	127010302	127032875	6352	-	*		0.7924	0.866	0.24	0.32	0.37	0.25	0.25	0.37
cis_mRNA_dw20k	1295	NA	n344971	MTCONS_00067476	chr7	126986884	127009008	7167	-	chr7	127010302	127032875	5903	-	*		0.9308	1	0.24	0.32	0.37	0.39	0.9	0.91
cis_mRNA_up10k	121	NA	LTCONS_00007576	MTCONS_00000065	chr1	954932	955334	403	.	chr1	955454	991499	7444	+	*		0.8377	1	1.24	1.39	1.28	16.17	17.27	17.03
cis_mRNA_overlap	0	CompleteIn-LncIntron	LTCONS_00004224	MTCONS_00004232	chr1	16786595	16971217	25174	-	chr1	16888816	16940083	7373	-	*		0.9879	1	1.4	2.05	1.69	3.93	6.2	5.25
tran	NA	NA	n377457	MTCONS_00054075	chr22	36068836	36085068	391	+	chr4	8363185	8431038	7806	-		-350.62	-0.9734	-1	0.3	0	0.18	0.11	0.43	0.17
tran	NA	NA	n325366	MTCONS_00005218	chr2	23451605	23457675	541	-	chr1	75667816	75843119	7850	-		-506.5	0.9822	1	3.44	4.61	3	18.73	24.06	14.51
tran	NA	NA	n387141	MTCONS_00069240	chr2	114368816	114384715	1388	-	chr8	155778	182318	8373	-		-770.85	0.7535	1	2.43	3.77	2.49	0	0.17	0.12
cis_mRNA_overlap	0	Lnc-CompleteIn-mRNAIntron	n383831	MTCONS_00039960	chr2	236916212	236918759	2548	+	chr2	236578619	237040438	11253	+	*		0.9993	1	0.12	0.22	0.29	3.31	3.81	4.21
cis_mRNA_overlap	0	Lnc-CompleteIn-mRNAIntron	n324260	MTCONS_00064200	chr7	1715250	1751313	1013	+	chr7	1705351	1787590	4362	+	*		-0.6127	-0.866	0.03	0.03	0.16	1.05	0.7	0.64
cis_mRNA_overlap	0	Lnc-CompleteIn-mRNAIntron	n324260	MTCONS_00064201	chr7	1715250	1751313	1013	+	chr7	1709935	1787590	3962	+	*		0.9887	0.866	0.03	0.03	0.16	1.52	0.99	4.28
cis_mRNA_overlap	0	AntiCompleteIn-mRNAIntron	n337641	MTCONS_00075464	chrX	18917183	18921344	2627	+	chrX	18909976	19003200	6401	-	*		0.8171	0.866	0.01	0.01	0	0.7	0.48	0.32
tran	2806534	NA	n408331	MTCONS_00009928	chr10	49218159	49239658	2139	-	chr10	46321034	46411626	5158	-		-1196.39	0.9791	1	0.67	0.7	0.64	5.39	8.7	3.83
tran	NA	NA	n387513	MTCONS_00076657	chr6	3267102	3267543	442	+	chrY	4898547	4981558	13244	+		-682.79	-0.6571	-1	15.89	23.17	22.78	1.57	0.93	1.47
tran	NA	NA	n346329	MTCONS_00073892	chr9	68428124	68433566	1248	-	chrUn_gl000241	16000	34973	964	-		-312.57	0.9954	1	0.17	1.22	1.97	0.11	1.74	2.55
tran	NA	NA	n346329	MTCONS_00073893	chr9	68428124	68433566	1248	-	chrUn_gl000241	16000	36863	837	-		-295.63	0.992	1	0.17	1.22	1.97	0	4.89	7.03
cis_mRNA_overlap	0	CompleteIn-LncIntron	n387349	MTCONS_00056070	chr5	69170991	70092798	1771	+	chr5	69696747	69746189	4977	+	*		-0.8713	-1	2.84	3.09	2.67	1.43	0.98	4.43
tran	NA	NA	LTCONS_00073267	MTCONS_00000415	chr9	114389202	114393569	777	-	chr1	16084305	16113139	3838	+		-834.05	0.6924	1	0.78	0.88	0.91	0.35	0.36	0.81
tran	NA	NA	LTCONS_00073267	MTCONS_00000419	chr9	114389202	114393569	777	-	chr1	16085255	16113139	3682	+		-834.05	0.7206	1	0.78	0.88	0.91	1.58	1.63	2.34
cis_mRNA_up10k	139	NA	n370120	MTCONS_00064311	chr7	7591941	7606158	923	-	chr7	7606296	7652468	9535	+	*		0.9006	0.866	0.18	0.01	0.18	2.77	2.33	3.11
cis_mRNA_up10k	141	NA	n370120	MTCONS_00064312	chr7	7591941	7606158	923	-	chr7	7606298	7652468	8819	+	*		-0.8486	-0.866	0.18	0.01	0.18	0.34	0.51	0.43
cis_mRNA_up10k	4594	NA	n341043	MTCONS_00011986	chr11	62529072	62534182	771	+	chr11	62538775	62557965	3517	+	*		0.9985	1	0.98	11.59	15.79	0.62	1	1.19
tran	NA	NA	n386278	MTCONS_00046828	chr20	47551778	47552227	450	+	chr22	30999407	31027721	7200	+		-307.86	0.9873	1	14.52	19.63	18.48	1.69	2.32	2.28
cis_mRNA_dw20k	255	NA	n384574	MTCONS_00057355	chr5	470626	473080	1742	-	chr5	473334	524549	2772	-	*		-0.9243	-1	0.65	0.21	0	0	0.01	0.03
cis_mRNA_up10k	103	NA	n387142	MTCONS_00038738	chr2	114379305	114384715	768	-	chr2	114384817	114402022	3834	+	*		-0.9939	-1	1.28	0.8	0.24	0.57	0.86	1.09
cis_mRNA_overlap	0	mRNA	n346081	MTCONS_00074678	chrX	100740395	100753377	6264	+	chrX	100740359	100771939	3421	+		-2215.39	0.9457	1	0.47	0.43	0.37	1.52	1.41	0.24
cis_mRNA_dw20k	18211	NA	n371093	MTCONS_00072750	chr9	69175590	69181270	2429	-	chr9	69199480	69262593	6928	-	*		0.9756	1	0.06	0.03	0.08	1.12	1.01	1.28
cis_mRNA_dw20k	13213	NA	n406456	MTCONS_00030272	chr17	4046462	4060991	1306	+	chr17	4074203	4167274	5289	-	*		0.9804	0.866	18.38	19.41	18.11	1.13	1.37	1.13
cis_mRNA_dw20k	37	NA	n406456	MTCONS_00030268	chr17	4046462	4060991	1306	+	chr17	4061027	4168215	14872	-	*		-0.9475	-1	18.38	19.41	18.11	0.09	0.08	0.1
tran	NA	NA	LTCONS_00072234	MTCONS_00003818	chr9	14430	30646	15054	-	chr1	14050	29381	9122	-		-6198.37	0.9682	1	6.12	8.1	10.34	2.41	2.88	3.09
tran	NA	NA	LTCONS_00072234	MTCONS_00003819	chr9	14430	30646	15054	-	chr1	14050	29437	7973	-		-5504.14	0.9909	1	6.12	8.1	10.34	5.14	7.76	9.61
cis_mRNA_up10k	5883	NA	n363316	MTCONS_00000121	chr1	1535819	1543166	1048	+	chr1	1549048	1566006	5568	+	*		-0.8893	-1	0.64	0.26	0.18	0.1	0.22	0.4
cis_mRNA_overlap	0	Lnc-CompleteIn-mRNAIntron	n325618	MTCONS_00020679	chr13	114113136	114117525	514	-	chr13	114109147	114145035	4320	-	*		-0.9836	-0.866	0.07	0.07	0.32	0.76	0.67	0.29

cis_mRNA_overlap	0	Lnc-CompleteIn-mRNAIntron	n325618	MTCONS_00020678	chr13	114113136	114117525	514	-	chr13	114109147	114145033	10788	-	*		0.7313	0.866	0.07	0.07	0.32	1.59	1.73	1.79
tran	NA	NA	n345097	MTCONS_00042372	chrX	109085564	109158741	863	+	chr2	203637784	203736841	8428	-		-720.08	0.9994	1	0.34	0.12	0.04	1.46	1.33	1.29
cis_mRNA_overlap	0	mRNA	n345764	MTCONS_00031600	chr17	62796440	62833211	1549	-	chr17	62773976	62833309	6100	-		-1288.68	-1	-1	0	0.19	0.06	0.6	0.47	0.56
tran	NA	NA	n345764	MTCONS_00032055	chr17	62796440	62833211	1549	-	chr17_ctg5_hap1	126502	183185	3623	-		-1206.68	0.9746	1	0	0.19	0.06	0.19	0.91	0.26
cis_mRNA_overlap	0	mRNA	n345764	MTCONS_00031596	chr17	62796440	62833211	1549	-	chr17	62745750	62833302	8207	-		-1299.64	0.9836	1	0	0.19	0.06	0.27	0.83	0.35
cis_mRNA_overlap	0	mRNA	LTCONS_00019865	MTCONS_00019863	chr13	25509380	25542635	3437	-	chr13	25502893	25542627	7000	-	*		0.9816	1	0.23	1.06	0.84	1.45	2.32	1.94
tran	1073978	NA	LTCONS_00002101	MTCONS_00002112	chr1	144178760	144219394	270	+	chr1	145293371	145370255	10498	+		-156.59	0.9832	1	0.77	2.84	0.69	0.61	1.47	0.36
tran	1995260	NA	LTCONS_00002101	MTCONS_00005907	chr1	144178760	144219394	270	+	chr1	146214653	146252722	2842	-		-147.07	-0.6115	-1	0.77	2.84	0.69	2.39	2.28	3.27
tran	1073978	NA	LTCONS_00002101	MTCONS_00002111	chr1	144178760	144219394	270	+	chr1	145293371	145370255	5674	+		-156.59	0.9729	1	0.77	2.84	0.69	1.1	2.25	0.67
tran	6286364	NA	n387246	MTCONS_00046828	chr22	24712379	24713044	488	-	chr22	30999407	31027721	7200	+		-1011.5	0.9822	1	4.72	6.46	6.02	1.69	2.32	2.28
cis_mRNA_up10k	7768	NA	n339096	MTCONS_00017053	chr12	7087683	7089096	1414	+	chr12	7074486	7079916	2177	-	*		-0.9982	-1	2.43	2.17	2.52	0.53	0.75	0.43
cis_mRNA_up10k	7768	NA	n339096	MTCONS_00017054	chr12	7087683	7089096	1414	+	chr12	7074486	7079916	1899	-	*		-0.9017	-1	2.43	2.17	2.52	3.78	4.41	2.6
cis_mRNA_dw20k	2512	NA	n370619	MTCONS_00068299	chr8	28915899	28922445	6335	+	chr8	28747908	28913388	6396	+	*		0.9609	1	1.23	1.97	1.57	3.25	3.93	3.39
cis_mRNA_dw20k	2351	NA	n370619	MTCONS_00069541	chr8	28915899	28922445	6335	+	chr8	28924795	28968618	5745	-	*		0.9583	1	1.23	1.97	1.57	0.41	1.07	0.54
tran	NA	NA	n406898	MTCONS_00032056	chr17	62780959	62833302	2922	-	chr17_ctg5_hap1	128247	183173	5339	-		-2243.69	-0.9371	-1	0.71	0.9	1.7	1.21	0.61	0.04
tran	NA	NA	LTCONS_00023286	MTCONS_00052870	chr15	25499878	25583387	7818	+	chr4	56914919	57200064	10678	+		-4463.09	0.9253	1	9.22	12.93	9.09	0.92	2.25	0
tran	126763547	NA	n381255	MTCONS_00000611	chr1	151763318	151766877	991	+	chr1	24968184	24999772	5383	+		-610.18	0.9463	1	4.77	7.11	4.96	1.94	3.41	2.52
tran	NA	NA	n324537	MTCONS_00037317	chr6	33220538	33223522	717	+	chr2	11622954	11783475	12886	+		-660.01	0.9106	1	0	0.05	0.13	1.34	1.5	1.55
cis_mRNA_overlap	0	Lnc-CompleteIn-mRNAExon	n378046	MTCONS_00004234	chr1	16920057	16935145	488	-	chr1	16888816	16940154	7419	-		-331.18	0.9743	1	1.98	1.46	1.13	5.07	3	0.07
cis_mRNA_overlap	0	Lnc-CompleteIn-mRNAExon	n378046	MTCONS_00004233	chr1	16920057	16935145	488	-	chr1	16888816	16940154	6606	-		-331.18	-0.9089	-1	1.98	1.46	1.13	0	0.91	3.95
tran	NA	NA	n324547	MTCONS_00063834	chr6	30857714	30868006	1176	+	chr6_cox_hap2	2371882	2380839	3740	+		-872.16	0.9843	1	0.15	0.13	0	1.95	1.44	0.27
cis_mRNA_up10k	107	NA	LTCONS_00042498	MTCONS_00042494	chr2	219519520	219524344	2485	-	chr2	219501914	219519414	8923	-	*		-0.8712	-1	1.05	0.83	0.96	0.79	1.11	1.07
cis_mRNA_up10k	650	NA	n408048	MTCONS_00052179	chr3	197477261	197511317	3824	+	chr3	197394817	197476612	10290	-	*		-0.9507	-1	4.55	5.86	5.42	0.51	0.09	0.1
cis_mRNA_overlap	0	Lnc-CompleteIn-mRNAExon	n340760	MTCONS_00057237	chr5	176724685	176727213	2528	+	chr5	176560760	176728189	13291	+		-1499.39	0.9878	0.866	0	0.02	0.02	1.03	1.08	1.09
tran	16308385	NA	LTCONS_00027503	MTCONS_00027225	chr16	18494434	18496023	1590	-	chr16	2138617	2186050	14619	-		-1661.42	0.9766	1	0.76	1	1.15	1.27	2.41	3.94
cis_mRNA_dw20k	17589	NA	n408883	MTCONS_00028688	chr17	7833663	7835267	748	-	chr17	7788468	7816075	9655	+	*		0.8951	1	1.68	1.76	1.41	0.95	1.25	0.78
tran	NA	NA	n323994	MTCONS_00010010	chr9	112401576	112407320	635	+	chr10	50641266	50818344	30532	-		-826.7	-0.9932	-1	1.94	3.09	1.85	1.11	0.69	1.21
tran	NA	NA	n323994	MTCONS_00058482	chr9	112401576	112407320	635	+	chr5	133621084	133676107	2857	-		-756.95	0.9934	1	1.94	3.09	1.85	1.39	1.73	1.31
tran	NA	NA	n323994	MTCONS_00010011	chr9	112401576	112407320	635	+	chr10	50641266	50818350	30643	-		-826.7	0.9356	1	1.94	3.09	1.85	1.27	1.58	1.05
cis_mRNA_up10k	67	NA	LTCONS_00030048	MTCONS_00030051	chr17	79372799	79373474	464	+	chr17	79373540	79433733	12320	+	*		-0.8046	-1	1.17	0.48	1.15	0.48	0.98	0.79
tran	NA	NA	n342914	MTCONS_00068281	chr6	90279820	90280172	353	+	chr8	27348645	27402486	6475	+		-430.56	0.682	0.866	0	0.1	0.1	0.26	0.38	0.77
cis_mRNA_overlap	0	CompleteIn-LncExon	LTCONS_00054149	MTCONS_00054150	chr4	17801804	17812414	10261	-	chr4	17801804	17812414	4921	-		-2642.91	-0.9866	-1	1.5	2.24	2.03	1.28	1.04	1.07
cis_mRNA_overlap	0	AntiCompleteIn-mRNAIntron	LTCONS_00007600	MTCONS_00001023	chr1	43876648	43877437	790	.	chr1	43855556	43919040	13202	+	*		-0.9455	-1	0.5	0.74	0.81	1.12	0.9	0.66
cis_mRNA_overlap	0	AntiCompleteIn-mRNAIntron	LTCONS_00007600	MTCONS_00001021	chr1	43876648	43877437	790	.	chr1	43855556	43919040	14646	+	*		0.9912	1	0.5	0.74	0.81	0.87	1.17	1.33
cis_mRNA_up10k	3185	NA	n363373	MTCONS_00000121	chr1	1535819	1545864	2219	+	chr1	1549048	1566006	5568	+	*		0.9472	1	0.06	0.08	0.28	0.1	0.22	0.4
tran	NA	NA	n376186	MTCONS_00003818	chr19	60951	70966	1137	-	chr1	14050	29381	9122	-		-848.07	0.9346	1	0.1	0.64	1.55	2.41	2.88	3.09
tran	NA	NA	n376186	MTCONS_00003819	chr19	60951	70966	1137	-	chr1	14050	29437	7973	-		-848.07	0.97	1	0.1	0.64	1.55	5.14	7.76	9.61
cis_mRNA_overlap	0	mRNA	n385920	MTCONS_00003818	chr1	16858	17751	717	-	chr1	14050	29381	9122	-	*		0.8944	1	3.81	5.81	10.9	2.41	2.88	3.09
cis_mRNA_overlap	0	mRNA	n385920	MTCONS_00003819	chr1	16858	17751	717	-	chr1	14050	29437	7973	-		-603.04	0.9409	1	3.81	5.81	10.9	5.14	7.76	9.61
tran	NA	NA	LTCONS_00013841	MTCONS_00052870	chr11	58646457	58673215	5165	-	chr4	56914919	57200064	10678	+		-4085.78	0.809	0.866	2.51	2.51	1.97	0.92	2.25	0
tran	NA	NA	LTCONS_00013841	MTCONS_00052872	chr11	58646457	58673215	5165	-	chr4	57081966	57200064	10458	+		-4085.78	-0.9703	-0.866	2.51	2.51	1.97	15.47	18.33	26.84
tran	NA	NA	LTCONS_00013841	MTCONS_00027552	chr11	58646457	58673215	5165	-	chr16	21448105	21531800	16151	-		-4080.81	-0.866	-0.866	2.51	2.51	1.97	0.37	0.41	0.45
tran	NA	NA	LTCONS_00013841	MTCONS_00016474	chr11	58646457	58673215	5165	-	chr12	111051832	111068271	2328	+		-967.93	-0.8532	-0.866	2.51	2.51	1.97	1.08	0.6	1.52
cis_mRNA_dw20k	9517	NA	n337635	MTCONS_00000065	chr1	1001015	1002363	1348	-	chr1	955454	991499	7444	+	*		0.9875	1	0.57	1.01	0.85	16.17	17.27	17.03
cis_mRNA_overlap	0	mRNA	n410133	MTCONS_00072472	chr9	32972604	33001639	1995	-	chr9	32968411	33002977	7531	-		-1175.29	-0.7984	-1	1.78	2.1	6.04	0.15	0.11	0.09
tran	NA	NA	n367653	MTCONS_00076654	chr5	29162523	29172465	732	+	chrY	4868267	4981558	12618	+		-568.96	0.9557	0.866	0.1	0.1	0.13	4.85	4.11	6.56
cis_mRNA_overlap	0	AntiCompleteIn-mRNAExon	n339355	MTCONS_00048725	chr3	49021339	49021925	586	-	chr3	48956206	49053477	15010	+		-1274	0.7924	0.866	1.19	0.8	0.95	0.01	0	0.01
cis_mRNA_overlap	0	AntiCompleteIn-mRNAExon	n339355	MTCONS_00048726	chr3	49021339	49021925	586	-	chr3	48956233	49053477	15713	+		-1274	-0.9991	-1	1.19	0.8	0.95	1.89	2.86	2.45
tran	NA	NA	n326497	MTCONS_00030178	chr1	103593557	103608574	439	-	chr17	1472556	1532487	9223	-		-489.67	0.9031	1	0.54	1.11	1.36	0.48	0.69	1.17
tran	NA	NA	n324620	MTCONS_00001552	chr6	2582169	2585234	834	+	chr1	84629892	84708194	8478	+		-398.23	0.9656	1	0.69	1.48	1.15	0.82	0.99	0.88
tran	NA	NA	n382975	MTCONS_00052870	chr16	32162610	32163874	1161	-	chr4	56914919	57200064	10678	+		-722.07	-0.9931	-1	1.28	1.12	1.35	0.92	2.25	0
tran	NA	NA	n345486	MTCONS_00046828	chr1	226300133	226321383	1503	+	chr22	30999407	31027721	7200	+		-1097.59	0.8273	1	7.55	13.88	10	1.69	2.32	2.28
tran	NA	NA	n325680	MTCONS_00052869	chr13	79276554	79277821	428	+	chr4	56914805	57200064	10656	+		-593.2	0.8367	1	3.35	2.12	2.31	19.73	16.31	18.62
cis_mRNA_up10k	2195	NA	n342662	MTCONS_00056211	chr5	79946861	79947776	916	-	chr5	79949970	80185496	4192	+	*		0.918	1	887.39	1252.04	1139.58	0	0.51	0.17
cis_mRNA_dw20k	1834	NA	LTCONS_00005884	MTCONS_00002112	chr1	145372088	145377867	5360	-	chr1	145293371	145370255	10498	+	*		0.9852	1	2.56	3.7	2.5	0.61	1.47	0.36
cis_mRNA_dw20k	1834	NA	LTCONS_00005884	MTCONS_00002111	chr1	145372088	145377867	5360	-	chr1	145293371	145370255	5674	+	*		0.9755	1	2.56	3.7	2.5	1.1	2.25	0.67
cis_mRNA_overlap	0	mRNA	LTCONS_00016475	MTCONS_00016476	chr12	111051832	111068271	2207	+	chr12	111051832	111080554	2438	+	*		0.866	1	1.7	1.82	1.74	0.79	0.99	0.95
cis_mRNA_overlap	0	Lnc-CompleteIn-mRNAIntron	LTCONS_00010017	MTCONS_00010010	chr10	50802466	50817151	14686	-	chr10	50641266	50818344	30532	-	*		-0.9995	-1	2.61	3.08	2.52	1.11	0.69	1.21
cis_mRNA_overlap	0	mRNA	LTCONS_00010017	MTCONS_00010013	chr10	50802466	50817151	14686	-	chr10	50720556	50818343	7004	-	*		0.8251	1	2.61	3.08	2.52	1.11	1.17	0.97

cis_mRNA_overlap	0	Lnc-CompleteIn-mRNAIntron	LTCONS_00010017	MTCONS_00010011	chr10	50802466	50817151	14686 -	chr10	50641266	50818350	30643 -	*		0.9622	1	2.61	3.08	2.52	1.27	1.58	1.05
cis_mRNA_overlap	0	Lnc-CompleteIn-mRNAIntron	n323984	MTCONS_00073336	chr9	119264393	119268359	751 -	chr9	119187492	120177521	4963 -	*		0.9851	1	0	0.09	0.13	3.67	6.62	7.11
cis_mRNA_up10k	25	NA	n369870	MTCONS_00067796	chr7	151108106	151110811	1527 +	chr7	151075540	151108082	8772 -	*		0.986	1	0.76	0.12	0.24	5.4	4.41	4.75
tran	27711965	NA	n385532	MTCONS_00072762	chr9	42671887	42714943	1253 +	chr9	70426907	70490171	6813 -		-525.06	-0.6342	-1	1.03	2.59	0.85	0.07	0.06	0.19
tran	NA	NA	n372816	MTCONS_00074514	chr11	18230678	18235486	922 +	chrX	70338311	70363507	8225 +		-771.22	-0.9732	-1	2.89	3.08	3.57	1.54	1.22	0.88
cis_mRNA_dw20k	8591	NA	n341935	MTCONS_00015970	chr12	58012188	58015686	2443 -	chr12	57998112	58003598	2375 +	*		0.6458	0.866	0.01	0.01	0	2.34	1.05	0.75
cis_mRNA_overlap	0	Lnc-AntiOverlap-mRNA	n341935	MTCONS_00015973	chr12	58012188	58015686	2443 -	chr12	58005162	58020030	6160 +	*		-0.6061	-0.866	0.01	0.01	0	0.74	1.49	1.61
cis_mRNA_overlap	0	Lnc-AntiOverlap-mRNA	n341935	MTCONS_00015974	chr12	58012188	58015686	2443 -	chr12	58005162	58020030	5668 +	*		-0.9375	-0.866	0.01	0.01	0	0.97	0.85	1.19
cis_mRNA_overlap	0	Lnc-AntiOverlap-mRNA	n341935	MTCONS_00015976	chr12	58012188	58015686	2443 -	chr12	58013573	58017177	1512 +	*		-0.7281	-0.866	0.01	0.01	0	1.97	2.47	2.68
tran	12665420	NA	n346288	MTCONS_00055677	chr5	34169130	34176069	665 -	chr5	21459589	21503711	2614 +		-366.47	-0.9982	-1	1.15	0.89	0.93	1.13	1.46	1.39
cis_mRNA_up10k	3896	NA	n406634	MTCONS_00006188	chr1	154947118	154951725	924 +	chr1	154929441	154943223	7183 -	*		-0.994	-1	0.62	1.89	0.21	1.95	0	2.26
cis_mRNA_up10k	3890	NA	n406634	MTCONS_00006189	chr1	154947118	154951725	924 +	chr1	154929441	154943229	7186 -	*		0.9732	1	0.62	1.89	0.21	0.01	2.01	0
cis_mRNA_overlap	0	mRNA	LTCONS_00016686	MTCONS_00016685	chr12	123459264	123467462	4600 +	chr12	123459264	123467462	4048 +		-3069.81	-0.6628	-0.866	1.12	1.04	1.42	0.36	0.37	0.36
cis_mRNA_overlap	0	mRNA	n333356	MTCONS_00061565	chr6	170657335	170697497	393 +	chr6	170603269	170716538	6726 +		-251.37	0.7807	1	0.2	0.54	0.53	0.27	0.56	0.37
cis_mRNA_overlap	0	mRNA	n333356	MTCONS_00061564	chr6	170657335	170697497	393 +	chr6	170602852	170716538	7458 +		-269.13	-0.7924	-1	0.2	0.54	0.53	2.16	1.69	1.99
tran	NA	NA	n339565	MTCONS_00004416	chr3	133372474	133372980	507 +	chr1	24290088	24306953	12176 -		-581.88	-1	-1	0.43	0.13	0.1	0.6	1.52	1.61
cis_mRNA_overlap	0	Lnc-CompleteIn-mRNAIntron	n385938	MTCONS_00001408	chr1	70385005	70386000	996 +	chr1	70032973	70446623	2700 +	*		-0.9946	-1	0.04	0.03	0	0.34	0.54	0.92
tran	NA	NA	n367382	MTCONS_00010010	chr4	124454586	124457636	546 -	chr10	50641266	50818344	30532 -		-608.46	-0.7186	-1	0.24	0.26	0.07	1.11	0.69	1.21
tran	NA	NA	n367382	MTCONS_00010011	chr4	124454586	124457636	546 -	chr10	50641266	50818350	30643 -		-608.46	0.8651	1	0.24	0.26	0.07	1.27	1.58	1.05
cis_mRNA_dw20k	14717	NA	n324000	MTCONS_00071534	chr9	108418160	108481900	2055 +	chr9	108320411	108403444	7109 +	*		-0.8472	-1	0.02	0.15	0	0.55	0.33	0.94
cis_mRNA_dw20k	276	NA	LTCONS_00014958	MTCONS_00014548	chr11	105871095	105876119	5025 .	chr11	105876394	105892992	5127 -	*		-0.803	-0.866	0.14	0.14	0.19	2.08	1.78	1.58
tran	NA	NA	n382987	MTCONS_00000659	chr16	35036459	35037041	583 -	chr1	26758868	26804903	8592 +		-344.18	0.948	1	6.34	7.22	5.69	0.68	1.25	0.6
tran	2891236	NA	LTCONS_00005962	MTCONS_00002112	chr1	148261490	148262364	247 -	chr1	145293371	145370255	10498 +		-140.75	0.9873	1	2.57	3.89	2.48	0.61	1.47	0.36
tran	2891236	NA	LTCONS_00005962	MTCONS_00002111	chr1	148261490	148262364	247 -	chr1	145293371	145370255	5674 +		-140.75	0.9782	1	2.57	3.89	2.48	1.1	2.25	0.67
tran	NA	NA	n364207	MTCONS_00064777	chr1	146644430	146645134	604 -	chr7	48128355	48148727	5326 +		-650.12	0.9965	0.866	0.06	0.11	0.11	2.57	5.07	4.84
tran	NA	NA	n364207	MTCONS_00064780	chr1	146644430	146645134	604 -	chr7	48128355	48148727	5211 +		-652.27	0.997	0.866	0.06	0.11	0.11	0.96	1.54	1.49
cis_mRNA_overlap	0	Lnc-AntiOverlap-mRNA	n379344	MTCONS_00068299	chr8	28779448	28922453	6111 -	chr8	28747908	28913388	6396 +	*		0.9808	0.866	0.03	0.04	0.03	3.25	3.93	3.39
cis_mRNA_overlap	0	Lnc-AntiOverlap-mRNA	n379344	MTCONS_00068300	chr8	28779448	28922453	6111 -	chr8	28747908	28913388	7232 +	*		-0.6379	-0.866	0.03	0.04	0.03	1.09	1.04	1.32
cis_mRNA_dw20k	2343	NA	n379344	MTCONS_00069541	chr8	28779448	28922453	6111 -	chr8	28924795	28968618	5745 -	*		0.9826	0.866	0.03	0.04	0.03	0.41	1.07	0.54
cis_mRNA_dw20k	2343	NA	n379344	MTCONS_00069543	chr8	28779448	28922453	6111 -	chr8	28924795	29120611	8704 -	*		-0.8735	-0.866	0.03	0.04	0.03	1.77	0.99	2.51
cis_mRNA_dw20k	4730	NA	n406352	MTCONS_00021293	chr14	62584075	62600903	6635 +	chr14	62279243	62579346	13802 +	*		0.9994	1	0.36	0.44	0.99	1.36	1.47	2.58
cis_mRNA_up10k	794	NA	LTCONS_00004087	MTCONS_00000257	chr1	7753184	7764062	9510 -	chr1	7764855	7829766	5074 +	*		-0.873	-1	1.42	1.74	2.2	12.08	9	8.55
tran	NA	NA	n380997	MTCONS_00069239	chr12	47599684	47610227	654 -	chr8	155778	182318	8477 -		-468.26	-0.9006	-1	0.06	0.05	0	0.1	0.22	0.31
cis_mRNA_up10k	4095	NA	LTCONS_00066629	MTCONS_00066627	chr7	43950362	43966064	420 -	chr7	43915501	43946268	3650 -	*		1	1	1.4	0.39	1.01	2.09	1.06	1.69
tran	NA	NA	n326545	MTCONS_00052869	chr1	83814047	84099267	3177 -	chr4	56914805	57200064	10656 +		-2432.64	-0.9014	-1	2.28	2.85	2.69	19.73	16.31	18.62
tran	NA	NA	n325478	MTCONS_00058317	chr2	3139534	3140575	435 +	chr5	114539713	114632458	16163 -		-419.15	-0.8701	-1	0.17	0	0.08	0.99	1.62	1.02
tran	NA	NA	n325478	MTCONS_00021581	chr2	3139534	3140575	435 +	chr14	89029253	89058791	7562 +		-445.53	0.8768	1	0.17	0	0.08	1.14	0.98	1.13
cis_mRNA_overlap	0	mRNA	LTCONS_00012904	MTCONS_00012906	chr11	123380598	123382134	922 +	chr11	123380598	123499110	8796 +	*		1	1	0.35	0.7	0.27	0.96	1.54	0.82
cis_mRNA_overlap	0	mRNA	LTCONS_00012904	MTCONS_00012905	chr11	123380598	123382134	922 +	chr11	123380598	123499110	8137 +	*		0.8745	1	0.35	0.7	0.27	2.34	3.11	0.98
cis_mRNA_overlap	0	Lnc-CompleteIn-mRNAIntron	LTCONS_00012904	MTCONS_00012903	chr11	123380598	123382134	922 +	chr11	123351521	123499110	8070 +	*		-0.6794	-1	0.35	0.7	0.27	0.51	0.4	2.46
cis_mRNA_dw20k	7481	NA	n332925	MTCONS_00061055	chr6	127617335	127620368	619 +	chr6	127587732	127609855	2714 +	*		0.9108	1	0.06	0	0.11	1.11	0	1.17
cis_mRNA_dw20k	7481	NA	n332925	MTCONS_00061057	chr6	127617335	127620368	619 +	chr6	127587881	127609855	2395 +	*		-0.9877	-1	0.06	0	0.11	1.07	2.13	0.58
cis_mRNA_overlap	0	Lnc-CompleteIn-mRNAExon	n381061	MTCONS_00004234	chr1	16888923	16890082	1160 -	chr1	16888816	16940154	7419 -		-715.1	0.986	1	2.27	1	0	5.07	3	0.07
cis_mRNA_overlap	0	Lnc-CompleteIn-mRNAExon	n381061	MTCONS_00004233	chr1	16888923	16890082	1160 -	chr1	16888816	16940154	6606 -		-715.1	-0.9322	-1	2.27	1	0	0	0.91	3.95
cis_mRNA_overlap	0	mRNA	LTCONS_00052303	MTCONS_00052301	chr4	124369	213298	1668 +	chr4	53227	166058	11726 +	*		-0.7009	-1	3.13	2.47	2.48	12.85	15.72	13.56
tran	NA	NA	n324896	MTCONS_00041342	chr21	18129204	18179796	1117 -	chr2	96487344	96658969	20870 -		-638.03	0.9961	1	14.17	15.18	17.31	2.25	2.77	3.55
tran	NA	NA	n324896	MTCONS_00041341	chr21	18129204	18179796	1117 -	chr2	96487344	96549806	19081 -		-638.03	-0.961	-1	14.17	15.18	17.31	6.48	6.43	5.38
tran	NA	NA	n326563	MTCONS_00027552	chr1	73947639	73953485	320 +	chr16	21448105	21531800	16151 -		-179.16	0.9542	1	0	0.08	0.35	0.37	0.41	0.45
cis_mRNA_up10k	125	NA	n342502	MTCONS_00050672	chr3	48481667	48485616	561 +	chr3	48473413	48481543	1669 -	*		0.8887	1	172.05	141.57	188.82	0.37	0.35	0.44
cis_mRNA_up10k	58	NA	n342502	MTCONS_00050673	chr3	48481667	48485616	561 +	chr3	48473413	48481610	1741 -	*		0.9633	1	172.05	141.57	188.82	2.05	1.74	2.5
cis_mRNA_dw20k	3250	NA	LTCONS_00071898	MTCONS_00071890	chr9	131266971	131282508	5261 +	chr9	131218285	131263722	5539 +	*		0.9794	1	3.85	3.84	3.88	0.04	0	0.9
cis_mRNA_overlap	0	AntiCompleteIn-mRNAIntron	n386452	MTCONS_00067127	chr7	97595944	97596474	531 +	chr7	97481372	97601638	2696 -	*		-0.6662	-1	0.91	0.13	0.06	0.75	1.08	3.08
cis_mRNA_overlap	0	Lnc-CompleteIn-mRNAIntron	n337722	MTCONS_00074432	chrX	56312985	56315265	2281 +	chrX	56258814	56318181	2901 +	*		-0.9889	-1	0.11	0	0.64	0.33	0.48	0
tran	NA	NA	n325704	MTCONS_00010010	chr13	63458505	63548145	1878 -	chr10	50641266	50818344	30532 -		-939.71	0.8343	1	0.09	0.07	0.14	1.11	0.69	1.21
tran	NA	NA	n325704	MTCONS_00010011	chr13	63458505	63548145	1878 -	chr10	50641266	50818350	30643 -		-939.71	-0.9427	-1	0.09	0.07	0.14	1.27	1.58	1.05
tran	132347653	NA	n345565	MTCONS_00041102	chr2	206966866	206967332	467 -	chr2	74579851	74619214	12873 -		-283.78	0.9817	1	0.08	0	0.22	2.72	2.64	3.07
tran	NA	NA	n409405	MTCONS_00075621	chr12	98847619	98850923	3305 -	chrX	46465414	46618804	3431 -		-1687.9	0.7771	1	1.37	1.41	2.44	9.36	11.84	13.08
tran	NA	NA	n409405	MTCONS_00075622	chr12	98847619	98850923	3305 -	chrX	46465414	46618804	2894 -		-1561.81	-0.9943	-1	1.37	1.41	2.44	2.31	2.19	1.51
tran	NA	NA	n325200	MTCONS_00074936	chr2	105127013	105127802	515 +	chrX	122993620	123047829	8359 +		-594.43	0.876	1	0.07	0	0.11	2.31	2	3.66
tran	NA	NA	n325200	MTCONS_00074935	chr2	105127013	105127802	515 +	chrX	122993486	123047829	7457 +		-594.43	-0.9726	-1	0.07	0	0.11	1.36	1.71	0.9

cis_mRNA_up10k	128	NA	n340705	MTCONS_00029392	chr17	43238061	43238894	834	-	chr17	43239021	43248063	2774	+	*		0.9738	1	1.8	2.26	1.95	0.27	0.46	0.29
cis_mRNA_up10k	128	NA	n340705	MTCONS_00029391	chr17	43238061	43238894	834	-	chr17	43239021	43248063	2318	+	*		0.9894	1	1.8	2.26	1.95	0.04	0.15	0.09
tran	145839	NA	n386880	MTCONS_00024177	chr15	85752617	85777989	558	-	chr15	85923827	86292586	13274	+		-490.94	0.9995	1	3.68	0.85	2.04	2.52	1.17	1.7
cis_mRNA_overlap	0	CompleteIn-LncExon	LTCONS_00024026	MTCONS_00024025	chr15	75638984	75648130	8535	+	chr15	75638984	75648130	4354	+		-3447.04	0.9928	1	1.3	1.88	1.95	0.15	1	1.25
cis_mRNA_dw20k	10657	NA	n366784	MTCONS_00055139	chr4	170838912	170897053	4365	-	chr4	170907709	170947599	6532	-	*		0.9596	1	0.09	0.13	0.06	1.5	1.78	0.91
tran	NA	NA	n326405	MTCONS_00037014	chr1	144084411	144086911	666	-	chr19	58411293	58446740	13084	-		-612.55	-0.8313	-1	0.36	0.18	0.49	3.94	3.96	3.31
cis_mRNA_overlap	0	mRNA	n409550	MTCONS_00041103	chr2	74588281	74619214	4451	-	chr2	74579851	74619214	15274	-		-3232.6	0.983	1	1.39	4.2	2.56	2.11	2.89	2.56
tran	NA	NA	n324349	MTCONS_00019861	chr6	148023532	148316766	773	+	chr13	25498815	25542635	10084	-		-371.25	-0.9989	-1	0.74	0.8	0.83	1.04	0.89	0.8
cis_mRNA_overlap	0	Lnc-CompleteIn-mRNAIntron	n333015	MTCONS_00061386	chr6	157297613	157298235	623	+	chr6	157098955	157532937	11020	+	*		0.9825	1	0.23	0.75	0.26	0.16	0.85	0.33
cis_mRNA_overlap	0	Lnc-CompleteIn-mRNAIntron	n333015	MTCONS_00061387	chr6	157297613	157298235	623	+	chr6	157098955	157532937	10882	+	*		-0.9187	-1	0.23	0.75	0.26	1.68	1.07	1.41
tran	1602822	NA	n387302	MTCONS_00052109	chr3	197319987	197354752	925	-	chr3	195684854	195717166	4032	-		-607.76	0.6526	1	6.22	6.07	3.35	1.88	1.04	0.89
tran	NA	NA	n326324	MTCONS_00008781	chr1	173438987	173440608	775	-	chr10	97515409	97637023	12236	+		-422.75	-0.8187	-0.866	0.37	1.22	0.86	0.09	0	0.09
tran	NA	NA	n410487	MTCONS_00063751	chr12	108296927	108297548	622	-	chr6	170887587	170893780	3997	-		-375.89	-0.9787	-1	1.61	0.99	1.22	0.25	3.26	2.72
cis_mRNA_up10k	869	NA	n385310	MTCONS_00067809	chr7	152133980	152140098	853	+	chr7	151831996	152133112	17048	-	*		-0.9306	-1	0.08	0	0.07	2.67	3.52	3.07
cis_mRNA_up10k	332	NA	n385310	MTCONS_00067810	chr7	152133980	152140098	853	+	chr7	151831996	152133649	16864	-	*		-0.9164	-1	0.08	0	0.07	2.73	4.52	3.63
cis_mRNA_overlap	0	mRNA	n381970	MTCONS_00016878	chr12	132602691	132613429	4335	+	chr12	132568828	132615587	6250	+	*		0.8265	1	0.6	0.88	0.83	0	0.07	0.02
cis_mRNA_dw20k	8321	NA	n381970	MTCONS_00016875	chr12	132602691	132613429	4335	+	chr12	132568828	132594371	2225	+	*		0.9951	1	0.6	0.88	0.83	0.12	0.25	0.24
cis_mRNA_up10k	443	NA	n371759	MTCONS_00009798	chr10	35104695	35105314	302	+	chr10	34398476	35104253	5881	-	*		-0.8698	-1	0.8	0.36	0.12	0.56	0.64	0.99
cis_mRNA_up10k	4600	NA	n386563	MTCONS_00004234	chr1	16944753	16952994	1404	-	chr1	16888816	16940154	7419	-	*		-0.9179	-1	4.37	4.4	5.96	5.07	3	0.07
cis_mRNA_up10k	4600	NA	n386563	MTCONS_00004233	chr1	16944753	16952994	1404	-	chr1	16888816	16940154	6606	-	*		0.979	1	4.37	4.4	5.96	0	0.91	3.95
cis_mRNA_overlap	0	AntiCompleteIn-mRNAIntron	n341770	MTCONS_00073336	chr9	119467561	119469216	1652	+	chr9	119187492	120177521	4963	-	*		-0.9221	-1	2.28	1.79	1.29	3.67	6.62	7.11
tran	225831	NA	n346467	MTCONS_00005954	chr1	148251678	148262280	410	-	chr1	148002303	148025848	4803	-		-246.46	-0.9416	-1	2.4	3.24	2.92	1.19	0.97	1.12
tran	2881424	NA	n346467	MTCONS_00002114	chr1	148251678	148262280	410	-	chr1	145304210	145370255	11091	+		-245.52	0.8915	1	2.4	3.24	2.92	0.52	1.07	0.63
cis_mRNA_overlap	0	mRNA	n344872	MTCONS_00060306	chr6	42727241	42778139	6853	+	chr6	42713996	42836296	9285	+	*		-0.9567	-1	0.44	0.53	0.37	1.3	1.05	1.35
cis_mRNA_overlap	0	mRNA	n344872	MTCONS_00060307	chr6	42727241	42778139	6853	+	chr6	42714017	42836296	6704	+	*		0.9992	1	0.44	0.53	0.37	0.59	1.25	0
tran	NA	NA	n369700	MTCONS_00027552	chr7	113134743	113138753	433	-	chr16	21448105	21531800	16151	-		-205.19	-0.9608	-1	0.26	0.23	0.22	0.37	0.41	0.45
cis_mRNA_up10k	162	NA	n411697	MTCONS_00004843	chr1	43232916	43241413	899	+	chr1	43212006	43232755	4264	-	*		0.968	1	13.53	12.4	11.97	1.03	0.89	0.75
cis_mRNA_dw20k	566	NA	n406867	MTCONS_00028689	chr17	7816640	7819265	2525	-	chr17	7788484	7816075	8129	+	*		-0.9573	-1	1.72	2.19	1.95	0.67	0.43	0.49
cis_mRNA_overlap	0	Lnc-CompleteIn-mRNAExon	n337970	MTCONS_00005088	chr1	57460452	57462521	2070	-	chr1	57460452	57890453	5757	-		-934.95	0.8979	1	1.24	2.05	1.92	0.52	0.66	0.58
cis_mRNA_up10k	63	NA	n411152	MTCONS_00062001	chr6	8435856	8785678	1183	+	chr6	8412187	8435794	2970	-	*		0.9911	1	1.67	1.14	1.84	1.11	0.79	1.15
tran	NA	NA	n365234	MTCONS_00030178	chr2	121343387	121362263	950	+	chr17	1472556	1532487	9223	-		-778.55	0.9902	1	0	0.03	0.07	0.48	0.69	1.17
tran	NA	NA	n344887	MTCONS_00046828	chr6	15708733	15726229	1373	-	chr22	30999407	31027721	7200	+		-1275.31	0.6539	1	0.63	0.7	0.64	1.69	2.32	2.28
tran	NA	NA	n410226	MTCONS_00012799	chr6	3399752	3401315	1057	-	chr11	118309253	118409866	27510	+		-937.68	-0.9334	-1	1.75	1.3	0.96	1.69	2.23	2.28
cis_mRNA_overlap	0	AntiCompleteIn-mRNAExon	n341789	MTCONS_00071843	chr9	130870856	130871223	368	-	chr9	130830430	130872795	4789	+		-800.5	-0.6326	-1	0.74	1.73	0.66	0.29	0.26	0.55
tran	NA	NA	n367818	MTCONS_00000611	chr5	135802366	135844717	676	+	chr1	24968184	24999772	5383	+		-355.22	-0.9377	-1	3.81	0	1.12	1.94	3.41	2.52
cis_mRNA_overlap	0	Lnc-CompleteIn-mRNAExon	n341180	MTCONS_00064295	chr7	6694495	6696063	1569	+	chr7	6676932	6698842	8192	+		-1371.96	-0.8026	-1	8.53	9.93	9.71	6.89	6.18	6.7
tran	1677754	NA	n410076	MTCONS_00035588	chr19	13945330	13947103	1774	-	chr19	12242097	12267577	7026	-		-1459.83	-0.8856	-1	0.68	1.37	1.21	1.78	1.28	1.61
cis_mRNA_overlap	0	Lnc-CompleteIn-mRNAExon	n409323	MTCONS_00036581	chr19	48711343	48752890	5198	-	chr19	48710992	48759248	5508	-		-2185.99	-0.9897	-1	0.4	0.13	0.49	1.39	2.14	0.93
tran	NA	NA	n383880	MTCONS_00052301	chr20	25165310	25177508	2158	-	chr4	53227	166058	11726	+		-2675.04	0.8709	1	5.2	5.57	5.46	12.85	15.72	13.56
cis_mRNA_overlap	0	CompleteIn-LncIntron	LTCONS_00005947	MTCONS_00005957	chr1	147906748	148176401	1258	-	chr1	148016192	148026022	1241	-	*		-0.9873	-1	0.09	0	0.98	0.95	0.97	0.89
cis_mRNA_overlap	0	mRNA	n324278	MTCONS_00063752	chr6	170887186	170888790	439	-	chr6	170887587	170893780	3206	-	*		-0.8975	-1	0.35	0.39	0.69	5.65	4.29	3.11
tran	NA	NA	n346262	MTCONS_00008781	chr6	6493144	6587623	2936	-	chr10	97515409	97637023	12236	+		-3002.79	-0.9707	-0.866	0	0.04	0.01	0.09	0	0.09
cis_mRNA_dw20k	2190	NA	n366169	MTCONS_00052179	chr3	197387436	197392628	1471	-	chr3	197394817	197476612	10290	-	*		0.6972	1	0.19	0.02	0.15	0.51	0.09	0.1
cis_mRNA_overlap	0	mRNA	n409281	MTCONS_00061762	chr6	2940298	2956709	1895	-	chr6	2948369	2971622	1445	-	*		-0.9005	-1	0.06	0.07	0	3.49	2.57	4.24
tran	NA	NA	n375756	MTCONS_00004419	chr17	66805273	66820947	815	-	chr1	24290088	24306953	8967	-		-402.42	0.9825	1	0.03	0.08	0	0.16	0.79	0
cis_mRNA_up10k	235	NA	LTCONS_00060496	MTCONS_00062754	chr6	56708719	56713357	4639	+	chr6	56322747	56708485	17654	-	*		-0.797	-0.866	0.23	0.18	0.23	15.07	15.76	15.49
tran	142027496	NA	n364823	MTCONS_00041432	chr2	242786532	242787188	571	+	chr2	100161762	100759037	10115	-		-447.08	0.9797	1	0.9	1.23	0.75	0.59	0.85	0.34
cis_mRNA_up10k	67	NA	LTCONS_00030047	MTCONS_00030050	chr17	79369012	79373474	565	+	chr17	79373540	79433733	10731	+	*		0.8486	0.866	1.34	2.11	1.34	0.09	0.17	0
cis_mRNA_up10k	67	NA	LTCONS_00030047	MTCONS_00030052	chr17	79369012	79373474	565	+	chr17	79373540	79433733	10515	+	*		-0.7206	-0.866	1.34	2.11	1.34	0.24	0.02	0.79
cis_mRNA_up10k	67	NA	LTCONS_00030047	MTCONS_00030051	chr17	79369012	79373474	565	+	chr17	79373540	79433733	12320	+	*		0.7892	0.866	1.34	2.11	1.34	0.48	0.98	0.79
tran	NA	NA	n380362	MTCONS_00071534	chr11	82783160	82784425	825	+	chr9	108320411	108403444	7109	+		-392.81	-0.9984	-1	1.69	1.99	1.26	0.55	0.33	0.94
tran	48187687	NA	n384669	MTCONS_00055679	chr5	69777167	69881629	1021	-	chr5	21459589	21589481	5313	+		-604.31	0.9975	1	2.3	3.92	2.78	0.4	0.53	0.43
tran	NA	NA	LTCONS_00072731	MTCONS_00005218	chr9	69080245	69108793	1120	-	chr1	75667816	75843119	7850	-		-716.35	-0.8976	-0.866	1.28	0.95	1.28	18.73	24.06	14.51
cis_mRNA_dw20k	3879	NA	n370669	MTCONS_00069213	chr8	145703379	145705868	1128	+	chr8	145691413	145699501	3137	+	*		0.9355	1	0.1	0.15	0.2	27.95	29.94	39.45
cis_mRNA_dw20k	3879	NA	n370669	MTCONS_00069215	chr8	145703379	145705868	1128	+	chr8	145691507	145699501	3401	+	*		-0.9925	-1	0.1	0.15	0.2	4.66	4.42	4.05
cis_mRNA_up10k	1056	NA	n384199	MTCONS_00048109	chr22	51222225	51227781	857	+	chr22	51203433	51221170	5239	-	*		0.9956	1	1.22	1.95	2.1	0.52	0.77	0.79
tran	NA	NA	n384841	MTCONS_00055678	chr6	26839266	26924333	1200	-	chr5	21459589	21522533	925	+		-558.85	0.8895	1	1.76	0.91	0.5	1.01	0.76	0.03
cis_mRNA_up10k	430	NA	n378828	MTCONS_00052066	chr3	194208183	194239008	2680	+	chr3	194123403	194207754	9002	-	*		-0.9969	-0.866	0.36	0.31	0.88	1.1	1.1	0.69
cis_mRNA_up10k	430	NA	n378828	MTCONS_00052067	chr3	194208183	194239008	2680	+	chr3	194123403	194207754	7564	-	*		0.9897	1	0.36	0.31	0.88	19.99	19.49	21.65
cis_mRNA_dw20k	2291	NA	n346188	MTCONS_00064216	chr7	2477402	2483484	381	+	chr7	2443223	2475112	2973	+	*		0.9944	0.866	0	0.1	0.1	0.24	1.08	1.19
cis_mRNA_dw20k	2291	NA	n346188	MTCONS_00064218	chr7	2477402	2483484	381	+	chr7	2443259	2475112	2951	+	*		-0.7811	-0.866	0	0.1	0.1	0.38	0.24	0
tran	NA	NA	n408162	MTCONS_00075293	chr12	87984	91263	1221	+	chrX	155251143	155255371	1784	+		-901.32	-0.9932	-1	4.36	13.26	10.36	3.66	0	1.57
cis_mRNA_dw20k	3634	NA	LTCONS_00011041	MTCONS_00009208	chr10	124195504	124195745	242	.	chr10	124134588	124191871	5558	+	*		0.8903	1	0.18	1.75	0	0.96	1.21	0.67
cis_mRNA_dw20k	3634	NA	LTCONS_00011041	MTCONS_00009206	chr10	124195504	124195745	242	.	chr10	124134094	124191871	6543	+	*		0.9683	1	0.18	1.75	0	3.4	4.56	2.79

cis_mRNA_up10k	111	NA	LTCONS_00076776	MTCONS_00076655	chrY	4858044	4868157	6834	-	chrY	4868267	5106271	7871	+	*	0.9564	1	1.48	0.02	1.82	2.78	1.04	4.25
cis_mRNA_up10k	111	NA	LTCONS_00076776	MTCONS_00076654	chrY	4858044	4868157	6834	-	chrY	4868267	4981558	12618	+	*	0.8421	1	1.48	0.02	1.82	4.85	4.11	6.56
cis_mRNA_overlap	0	Lnc-CompleteIn-mRNAIntron	n386466	MTCONS_00069358	chr8	10980891	10982327	1437	-	chr8	10958954	11060192	12778	-	*	0.9157	0.866	0.24	0.24	0.28	1	2.1	3.72
cis_mRNA_overlap	0	Lnc-CompleteIn-mRNAIntron	n383035	MTCONS_00027104	chr16	90107060	90108062	1003	+	chr16	90096312	90111416	4405	+	*	0.866	1	1.55	2.89	1.92	0.98	1.75	1.55
tran	NA	NA	n366302	MTCONS_00067129	chr3	125485334	125505694	355	+	chr7	97481372	97602642	3059	-	-339.37	-0.9971	-1	0.33	1.2	0.59	2.64	1.36	2.17
tran	NA	NA	LTCONS_00028869	MTCONS_00062613	chr17	18601292	18607785	789	+	chr6	43489259	43497289	3911	-	-505.16	0.9888	1	2.9	4.1	6.34	0.33	0.37	0.52
tran	NA	NA	LTCONS_00028869	MTCONS_00065317	chr17	18601292	18607785	789	+	chr7	99647409	99667594	8056	+	-835.2	-0.9851	-1	2.9	4.1	6.34	0.58	0.51	0.44
cis_mRNA_overlap	0	Lnc-CompleteIn-mRNAIntron	n338800	MTCONS_00037755	chr2	42815140	42816614	1475	+	chr2	42795601	42984294	5825	+	*	0.7196	0.866	0.21	0.15	0.21	1.65	1.05	3.16
cis_mRNA_overlap	0	Lnc-CompleteIn-mRNAIntron	n338800	MTCONS_00037753	chr2	42815140	42816614	1475	+	chr2	42795268	42984294	5608	+	*	-0.8238	-0.866	0.21	0.15	0.21	6.33	7.9	4.26
tran	NA	NA	n371268	MTCONS_00023550	chr9	87653271	87655849	778	-	chr15	42064664	42120056	10042	+	-509.33	-0.8948	-0.866	0.18	0.04	0.04	1.16	1.54	1.37
tran	NA	NA	n371268	MTCONS_00023549	chr9	87653271	87655849	778	-	chr15	42064664	42120056	9643	+	-513.86	-0.9616	-0.866	0.18	0.04	0.04	0.88	1.26	1.41
tran	NA	NA	n371268	MTCONS_00012799	chr9	87653271	87655849	778	-	chr11	118309253	118409866	27510	+	-758.88	-0.9971	-0.866	0.18	0.04	0.04	1.69	2.23	2.28
cis_mRNA_dw20k	255	NA	n386371	MTCONS_00057355	chr5	470933	473080	1293	-	chr5	473334	524549	2772	-	*	0.9864	1	0	0.11	0.23	0	0.01	0.03
tran	NA	NA	n325341	MTCONS_00052301	chr2	37824980	37827693	384	+	chr4	53227	166058	11726	+	-217.46	0.9976	1	7.29	9.51	7.98	12.85	15.72	13.56
cis_mRNA_overlap	0	Lnc-AntiOverlap-mRNA	n410066	MTCONS_00036162	chr19	36980529	36981942	1414	+	chr19	36936021	36980534	5098	-	*	0.9999	1	0.98	1.11	1.14	0.83	1.42	1.57
cis_mRNA_overlap	0	Lnc-AntiOverlap-mRNA	n342409	MTCONS_00036581	chr19	48758932	48761448	1952	+	chr19	48710992	48759248	5508	-	*	0.9479	1	0.92	1.19	0.9	1.39	2.14	0.93
tran	NA	NA	n377066	MTCONS_00034273	chr21	16120066	16126186	743	-	chr19	37709374	37740417	8724	+	-631.32	-0.9218	-1	0.08	0.09	0.3	1.19	0.96	0.65
tran	NA	NA	n378428	MTCONS_00055882	chr1	144325552	144340593	405	-	chr5	49962091	50142704	8119	+	-205.21	0.8641	1	0.58	0.41	0.38	0.82	0.79	0.74
cis_mRNA_overlap	0	mRNA	n341283	MTCONS_00067233	chr7	100806996	100808145	1150	-	chr7	100805790	100813234	2820	-	-1097.86	0.9477	1	14.91	17.51	22.61	0.54	0.77	0.91
cis_mRNA_overlap	0	mRNA	n341283	MTCONS_00067235	chr7	100806996	100808145	1150	-	chr7	100805790	100813451	3958	-	-1117.01	0.7591	0.866	14.91	17.51	22.61	0.95	1.04	1.04
tran	NA	NA	n325705	MTCONS_00052870	chr13	63300903	63303986	790	-	chr4	56914919	57200064	10678	+	-803.74	0.8979	1	5.69	5.86	4.93	0.92	2.25	0
cis_mRNA_up10k	111	NA	n380927	MTCONS_00021915	chr14	105883221	105886076	2279	-	chr14	105886186	105937068	8305	+	*	-0.9996	-1	0.54	0.25	0.41	4.25	5.6	4.89
cis_mRNA_up10k	111	NA	n380927	MTCONS_00021914	chr14	105883221	105886076	2279	-	chr14	105886186	105937068	9547	+	*	-0.9962	-1	0.54	0.25	0.41	1.06	1.69	1.39
tran	39363582	NA	n384439	MTCONS_00052301	chr4	39529639	39596327	2787	+	chr4	53227	166058	11726	+	-3812.75	0.778	1	26.87	29.11	28.8	12.85	15.72	13.56
cis_mRNA_dw20k	11358	NA	n372857	MTCONS_00011060	chr11	309549	311060	878	-	chr11	289400	298192	6281	+	*	0.9912	1	0.89	1.02	0.86	1.05	1.71	0.75
cis_mRNA_dw20k	11358	NA	n372857	MTCONS_00011056	chr11	309549	311060	878	-	chr11	289076	298192	6835	+	*	0.9905	1	0.89	1.02	0.86	0.99	1.27	0.86
cis_mRNA_up10k	9017	NA	n377554	MTCONS_00048088	chr22	51030492	51033971	914	+	chr22	51006098	51021476	6017	-	*	0.7707	0.866	0.54	0.54	0.64	5.18	6.44	7.13
cis_mRNA_up10k	9007	NA	n377554	MTCONS_00048092	chr22	51030492	51033971	914	+	chr22	51006098	51021486	5859	-	*	-0.737	-0.866	0.54	0.54	0.64	0.39	0.12	0
tran	NA	NA	n337760	MTCONS_00048727	chr15	51213504	51214133	630	+	chr3	48956233	49053477	15105	+	-616.78	-0.9967	-1	0	0.05	0.31	0.47	0.46	0.34
cis_mRNA_dw20k	4680	NA	n341567	MTCONS_00070132	chr8	101924988	101926002	534	-	chr8	101930681	101965623	4245	-	*	0.899	1	0.56	0.25	0.81	6.84	4.82	6.86
cis_mRNA_overlap	0	Lnc-CompleteIn-mRNAExon	n381793	MTCONS_00012906	chr11	123496111	123498473	2363	+	chr11	123380598	123499110	8796	+	-1526.01	-0.9882	-1	0.33	0.05	0.34	0.96	1.54	0.82
cis_mRNA_overlap	0	Lnc-CompleteIn-mRNAExon	n381793	MTCONS_00012905	chr11	123496111	123498473	2363	+	chr11	123380598	123499110	8137	+	-1480.71	-0.795	-1	0.33	0.05	0.34	2.34	3.11	0.98
tran	NA	NA	n367438	MTCONS_00035589	chr4	183994216	183998865	554	-	chr19	12242097	12267577	9332	-	-592.11	0.8973	1	0.55	0.39	0.56	1.84	1.63	2.04
cis_mRNA_dw20k	2064	NA	LTCONS_00041071	MTCONS_00041073	chr2	73169165	73298443	4560	-	chr2	73300506	73340146	6359	-	*	-0.7476	-1	0.44	0.48	0.81	1.69	1.59	1.56
cis_mRNA_dw20k	2064	NA	LTCONS_00041071	MTCONS_00041075	chr2	73169165	73298443	4560	-	chr2	73300506	73340146	6225	-	*	0.7508	1	0.44	0.48	0.81	0.5	0.63	0.67
cis_mRNA_overlap	0	mRNA	n371450	MTCONS_00074431	chrX	56316470	56325981	1347	+	chrX	56258806	56319269	9570	+	-716.72	-0.9592	-0.866	0.1	0	0.1	0.51	1.06	0.67
cis_mRNA_overlap	0	mRNA	n371450	MTCONS_00074432	chrX	56316470	56325981	1347	+	chrX	56258814	56318181	2901	+	*	-0.7406	-0.866	0.1	0	0.1	0.33	0.48	0
tran	NA	NA	n406926	MTCONS_00067127	chr12	8509560	8543348	661	-	chr7	97481372	97601638	2696	-	-496.62	0.6468	1	0	0.52	0.55	0.75	1.08	3.08
tran	NA	NA	n345954	MTCONS_00073892	chr20	29611871	29652524	1811	+	chrUn_gl000241	16000	34973	964	-	-382.51	0.9627	1	0.09	1.08	2.4	0.11	1.74	2.55
tran	NA	NA	n345954	MTCONS_00073893	chr20	29611871	29652524	1811	+	chrUn_gl000241	16000	36863	837	-	-378.2	0.954	1	0.09	1.08	2.4	0	4.89	7.03
tran	NA	NA	n325918	MTCONS_00041393	chr10	91665226	91707110	1860	-	chr2	98081180	98160993	24447	-	-901.31	-0.9066	-1	0.09	0.21	0.12	1.57	0.94	1.17
tran	NA	NA	n325918	MTCONS_00041394	chr10	91665226	91707110	1860	-	chr2	98081180	98206428	25798	-	-901.31	0.9592	1	0.09	0.21	0.12	2.83	3.88	3.36
tran	NA	NA	n325004	MTCONS_00052869	chr20	29472900	29480644	499	-	chr4	56914805	57200064	10656	+	-250.44	-0.9443	-1	8.41	14.77	12.34	19.73	16.31	18.62
tran	48201462	NA	n386388	MTCONS_00055679	chr5	69790942	69881629	1252	-	chr5	21459589	21589481	5313	+	-769.3	-0.6971	-1	8.45	4.32	4.44	0.4	0.53	0.43
cis_mRNA_overlap	0	Lnc-CompleteIn-mRNAIntron	n337053	MTCONS_00014146	chr11	66485915	66486182	268	-	chr11	66448399	66497268	13286	-	*	0.6698	1	0.77	0.7	1.24	3.01	2.92	3.02
cis_mRNA_up10k	2364	NA	n369920	MTCONS_00064311	chr7	7590483	7603933	1545	-	chr7	7606296	7652468	9535	+	*	-0.8268	-0.866	0.29	0.29	0	2.77	2.33	3.11
cis_mRNA_up10k	2366	NA	n369920	MTCONS_00064313	chr7	7590483	7603933	1545	-	chr7	7606298	7652468	9329	+	*	0.7396	0.866	0.29	0.29	0	2.13	1.2	0.78
tran	NA	NA	n386321	MTCONS_00044626	chr22	25498414	25508659	943	-	chr20	39804376	39946230	12652	-	-726.06	0.8773	1	0.41	0.14	0.2	0.06	0	0.04
tran	NA	NA	n378905	MTCONS_00014549	chr2	75767865	75769832	1101	+	chr11	105876394	105892992	5482	-	-571.69	-0.8932	-1	0.19	0.73	0.67	1.53	0.36	0.99
cis_mRNA_dw20k	942	NA	LTCONS_00063315	MTCONS_00063319	chr6	135605098	135622821	2126	-	chr6	135623762	135818903	4090	-	*	0.9962	1	1.45	3.58	3.74	1.46	2.93	3.21
tran	NA	NA	n365154	MTCONS_00018412	chr2	54904602	54908989	352	+	chr12	111889928	112038572	7176	-	-389.73	-0.9476	-1	0	0.19	0.1	3.54	3.26	3.31
cis_mRNA_overlap	0	mRNA	n324045	MTCONS_00072941	chr9	92103004	92235591	1013	-	chr9	91975013	92110713	7640	-	*	-0.9985	-1	0.07	0	0.1	0.34	0.62	0.19
cis_mRNA_overlap	0	mRNA	n324045	MTCONS_00072942	chr9	92103004	92235591	1013	-	chr9	91975013	92112916	7554	-	*	-0.9872	-1	0.07	0	0.1	0.27	0.51	0.08
tran	NA	NA	n385216	MTCONS_00036675	chr7	74233343	74233767	425	-	chr19	50489537	50529105	3738	-	-265.48	0.8545	1	0.63	0.49	0.64	1.87	1.54	2.31
tran	NA	NA	n385216	MTCONS_00036673	chr7	74233343	74233767	425	-	chr19	50489537	50528921	3771	-	-265.48	0.8686	1	0.63	0.49	0.64	1.87	1.39	2.45
tran	NA	NA	n382018	MTCONS_00074936	chr13	41793282	41794134	853	+	chrX	122993620	123047829	8359	+	-647.93	-0.7185	-1	0.23	0.55	0.19	2.31	2	3.66
tran	NA	NA	n382018	MTCONS_00074935	chr13	41793282	41794134	853	+	chrX	122993486	123047829	7457	+	-647.93	0.8774	1	0.23	0.55	0.19	1.36	1.71	0.9
cis_mRNA_dw20k	584	NA	n377665	MTCONS_00028686	chr17	7816658	7819589	2831	-	chr17	7788123	7816075	7388	+	*	-0.9077	-1	0.11	0.18	0.38	18.09	14.96	13.21
cis_mRNA_dw20k	10279	NA	n346258	MTCONS_00052108	chr3	195665538	195674576	443	-	chr3	195684854	195717166	5790	-	*	0.9658	1	0.94	0.62	0.76	3.52	2.83	3.29
cis_mRNA_dw20k	7441	NA	n384792	MTCONS_00059086	chr5	180258663	180262725	1558	+	chr5	180270165	180288363	9238	-	*	-1	-1	0.94	1.29	0.45	0.63	0.39	0.96
tran	NA	NA	n325768	MTCONS_00005218	chr13	30402814	30403619	435	-	chr1	75667816	75843119	7850	-	-739.48	0.9746	1	0.75	1.93	0.38	18.73	24.06	14.51
tran	47551077	NA	n384662	MTCONS_00055679	chr5	69140557	69416666	1357	+	chr5	21459589	21589481	5313	+	-811.65	-0.8064	-1	3.11	0.24	0.82	0.4	0.53	0.43

cis_mRNA_overlap	0	AntiCompleteIn-mRNAIntron	n383602	MTCONS_00037512	chr2	27234637	27237480	1966	-	chr2	27193282	27250087	2068	+	*	0.7786	1	0.94	2.01	1.31	4.94	5.35	5.34
cis_mRNA_overlap	0	AntiCompleteIn-mRNAIntron	n383602	MTCONS_00037513	chr2	27234637	27237480	1966	-	chr2	27193282	27250087	2686	+	*	0.9887	1	0.94	2.01	1.31	0.79	2.13	1.43
cis_mRNA_overlap	0	AntiCompleteIn-mRNAIntron	n383602	MTCONS_00037511	chr2	27234637	27237480	1966	-	chr2	27193282	27250087	4016	+	*	0.985	1	0.94	2.01	1.31	3.83	5.76	4.79
cis_mRNA_overlap	0	Lnc-CompleteIn-mRNAIntron	n336291	MTCONS_00010886	chr10	126467360	126467666	307	-	chr10	126307784	126480448	6254	-	*	-0.9999	-1	0.39	1.07	0.35	1.02	0	1.1
cis_mRNA_dw20k	10852	NA	LTCONS_00037011	MTCONS_00037013	chr19	58370253	58400442	407	-	chr19	58411293	58429710	12199	-	*	-0.9999	-1	0.91	0.86	0.63	0.52	0.55	0.7
cis_mRNA_up10k	4350	NA	n344941	MTCONS_00066222	chr7	5467807	5476343	2937	-	chr7	5390483	5463458	6938	-	*	0.9931	1	0.02	0.05	0.03	2.13	3.02	2.33
cis_mRNA_up10k	4356	NA	n344941	MTCONS_00066221	chr7	5467807	5476343	2937	-	chr7	5390483	5463452	9012	-	*	0.9807	1	0.02	0.05	0.03	0.94	1.28	0.99
tran	NA	NA	n325929	MTCONS_00041342	chr10	86029716	86044395	2685	+	chr2	96487344	96658969	20870	-	-1393.2	0.9922	1	26.37	26.69	27.48	2.25	2.77	3.55
cis_mRNA_dw20k	1566	NA	LTCONS_00022932	MTCONS_00022934	chr14	102690686	102693495	2510	-	chr14	102695060	102771531	2845	-	*	-0.8986	-1	0.63	0.56	0.97	1.39	1.94	0.99
cis_mRNA_dw20k	1566	NA	LTCONS_00022932	MTCONS_00022935	chr14	102690686	102693495	2510	-	chr14	102695060	102771531	2842	-	*	0.9969	1	0.63	0.56	0.97	1.45	1.39	2.06
cis_mRNA_dw20k	1566	NA	LTCONS_00022932	MTCONS_00022933	chr14	102690686	102693495	2510	-	chr14	102695060	102771531	2052	-	*	0.7231	1	0.63	0.56	0.97	1.11	0.21	1.25
tran	NA	NA	n372533	MTCONS_00068694	chr11	61236176	61241062	857	+	chr8	79578282	79624487	15452	+	-687.66	-0.7138	-1	6.61	4.74	4.43	3.66	3.69	3.86
cis_mRNA_up10k	4525	NA	LTCONS_00004249	MTCONS_00004234	chr1	16944678	16971208	3228	-	chr1	16888816	16940154	7419	-	*	-0.9112	-0.866	1.79	1.79	2.09	5.07	3	0.07
cis_mRNA_up10k	4525	NA	LTCONS_00004249	MTCONS_00004233	chr1	16944678	16971208	3228	-	chr1	16888816	16940154	6606	-	*	0.9755	0.866	1.79	1.79	2.09	0	0.91	3.95
tran	NA	NA	LTCONS_00055712	MTCONS_00052872	chr5	27472376	27479317	1984	+	chr4	57081966	57200064	10458	+	-1713.37	-0.7632	-1	10.54	7.22	6.81	15.47	18.33	26.84
cis_mRNA_up10k	4250	NA	n369045	MTCONS_00062586	chr6	42423793	42424759	386	+	chr6	42192598	42419544	7869	-	*	-0.9809	-1	0.2	0.18	0	0	0.32	1.1
cis_mRNA_up10k	4755	NA	n369045	MTCONS_00062588	chr6	42423793	42424759	386	+	chr6	42235641	42419039	2316	-	*	0.6174	1	0.2	0.18	0	1.64	0.98	0.94
tran	NA	NA	n380971	MTCONS_00000612	chr14	27312658	27629626	568	+	chr1	24968184	24999772	7455	+	-387.89	0.965	1	0.28	0.53	0.75	1.92	2.59	2.79
tran	NA	NA	n384068	MTCONS_00027552	chr22	17082801	17095998	1573	+	chr16	21448105	21531800	16151	-	-1373.49	0.9986	1	0	0.05	0.11	0.37	0.41	0.45
cis_mRNA_dw20k	16426	NA	LTCONS_00036191	MTCONS_00034271	chr19	37756842	37792580	376	-	chr19	37708283	37740417	8340	+	*	0.9082	1	0.49	0	0.71	0.34	0.25	0.54
tran	38254	NA	n377902	MTCONS_00035589	chr19	12305830	12318512	495	+	chr19	12242097	12267577	9332	-	-293.19	0.9636	1	0.26	0.2	0.42	1.84	1.63	2.04
tran	NA	NA	n406148	MTCONS_00073892	chr20	29611879	29634007	946	+	chrUn_gl000241	16000	34973	964	-	-320.27	0.9772	1	0.21	3.98	8.05	0.11	1.74	2.55
tran	NA	NA	n406148	MTCONS_00073893	chr20	29611879	29634007	946	+	chrUn_gl000241	16000	36863	837	-	-291.84	0.9703	1	0.21	3.98	8.05	0	4.89	7.03
tran	NA	NA	n325157	MTCONS_00052872	chr2	118656985	118660419	580	-	chr4	57081966	57200064	10458	+	-351.98	0.8542	1	10.08	11.23	11.64	15.47	18.33	26.84
tran	NA	NA	n386654	MTCONS_00046828	chr11	41326847	41327450	604	+	chr22	30999407	31027721	7200	+	-1208.1	0.9538	1	2.49	4.58	3.83	1.69	2.32	2.28
cis_mRNA_overlap	0	Lnc-CompleteIn-mRNAIntron	n371549	MTCONS_00074703	chrX	101892938	101894686	395	+	chrX	101854096	101914831	7080	+	*	0.9014	1	2.44	4.39	4.12	0.32	0.76	0.52
tran	NA	NA	n325279	MTCONS_00076655	chr2	66407997	66409620	905	+	chrY	4868267	5106271	7871	+	-349.85	-0.9483	-1	7.79	8.46	7.66	2.78	1.04	4.25
tran	NA	NA	n383698	MTCONS_00019863	chr2	110339915	110342057	2143	-	chr13	25502893	25542627	7000	-	-1969.65	0.8273	0.866	0.03	0.19	0.03	1.45	2.32	1.94
tran	NA	NA	n383698	MTCONS_00052301	chr2	110339915	110342057	2143	-	chr4	53227	166058	11726	+	-1386.52	0.9714	0.866	0.03	0.19	0.03	12.85	15.72	13.56
cis_mRNA_dw20k	10208	NA	n375787	MTCONS_00030272	chr17	4063095	4063996	843	+	chr17	4074203	4167274	5289	-	*	0.9254	0.866	0.46	0.67	0.33	1.13	1.37	1.13
cis_mRNA_overlap	0	AntiCompleteIn-mRNAExon	n375787	MTCONS_00030268	chr17	4063095	4063996	843	+	chr17	4061027	4168215	14872	-	-1851.76	-0.9909	-1	0.46	0.67	0.33	0.09	0.08	0.1
tran	NA	NA	n372506	MTCONS_00052301	chr11	34714000	34723562	627	+	chr4	53227	166058	11726	+	-411.6	-0.9867	-1	8.06	4.18	7.73	12.85	15.72	13.56
tran	NA	NA	n369336	MTCONS_00069240	chr6	170920275	170921789	280	-	chr8	155778	182318	8373	-	-238.31	-0.7666	-1	0.15	0	0.14	0	0.17	0.12
tran	NA	NA	n325347	MTCONS_00052869	chr2	34756150	34801134	1109	+	chr4	56914805	57200064	10656	+	-1191.11	-0.9733	-1	4.23	6.22	5.28	19.73	16.31	18.62
tran	NA	NA	n324195	MTCONS_00076657	chr7	115998442	116041973	468	-	chrY	4898547	4981558	13244	+	-641.19	-0.9886	-1	0.16	0.46	0.25	1.57	0.93	1.47
cis_mRNA_overlap	0	Lnc-CompleteIn-mRNAIntron	n332852	MTCONS_00032436	chr18	46282065	46282694	630	+	chr18	46065427	46389588	5746	+	*	-0.89	-1	0	0.26	0.21	8.79	5.18	7.4
cis_mRNA_dw20k	16927	NA	n337991	MTCONS_00001410	chr1	70607065	70610043	2979	+	chr1	70033190	70590139	8430	+	*	0.8458	1	0.93	1.32	0.97	0.34	0.73	0.58
cis_mRNA_overlap	0	Lnc-AntiOverlap-mRNA	n340821	MTCONS_00061930	chr6	4135660	4146285	635	+	chr6	4115927	4135831	1605	-	*	0.9226	0.866	0	0.05	0.05	0	0.18	0.11
cis_mRNA_up10k	8432	NA	LTCONS_00052183	MTCONS_00052177	chr3	197472274	197476666	3570	-	chr3	197394817	197463843	10135	-	*	-0.9979	-1	1.55	1.42	0.95	1.53	1.86	3.69
tran	NA	NA	n386236	MTCONS_00052872	chr2	56400669	56412905	2863	-	chr4	57081966	57200064	10458	+	-1641.87	0.7065	1	20.96	26.6	26.71	15.47	18.33	26.84
tran	40074440	NA	n386236	MTCONS_00041342	chr2	56400669	56412905	2863	-	chr2	96487344	96658969	20870	-	-2452.74	0.8128	1	20.96	26.6	26.71	2.25	2.77	3.55
tran	NA	NA	n385663	MTCONS_00006068	chr9	103115343	103156148	1956	+	chr1	151740719	151763010	4208	-	-1277.1	-0.997	-1	0.36	0.62	0.33	1.08	0.72	1.16
tran	NA	NA	n385663	MTCONS_00006067	chr9	103115343	103156148	1956	+	chr1	151740719	151763010	4227	-	-1277.1	0.9282	1	0.36	0.62	0.33	5.39	6.43	4.51
cis_mRNA_dw20k	19231	NA	LTCONS_00064718	MTCONS_00066642	chr7	44079067	44082082	1710	+	chr7	44101312	44122129	15429	-	*	-0.9981	-0.866	0.65	0.61	0.07	0.06	0.06	0.14
cis_mRNA_dw20k	19231	NA	LTCONS_00064718	MTCONS_00066641	chr7	44079067	44082082	1710	+	chr7	44101312	44122129	13583	-	*	-0.6067	-1	0.65	0.61	0.07	0.53	0.78	0.8
tran	NA	NA	n326173	MTCONS_00052301	chr1	232911758	232922346	1008	+	chr4	53227	166058	11726	+	-753.34	0.8579	1	7.26	9.6	8.96	12.85	15.72	13.56
tran	NA	NA	n384856	MTCONS_00063948	chr6	29894106	29897502	2516	+	chr6_mcf_hap5	2701469	2704679	1429	-	-1053.62	0.9989	1	0.2	0.25	0.21	1.3	5.31	1.92
tran	NA	NA	n375659	MTCONS_00020680	chr17	79482373	79494105	922	+	chr13	114109147	114145063	4196	-	-905.08	-0.9076	-1	2.73	1.79	2.44	0	0.29	0.2
tran	2886472	NA	LTCONS_00005961	MTCONS_00002112	chr1	148256726	148262364	247	-	chr1	145293371	145370255	10498	+	-140.75	0.9873	1	2.57	3.89	2.48	0.61	1.47	0.36
tran	2886472	NA	LTCONS_00005961	MTCONS_00002111	chr1	148256726	148262364	247	-	chr1	145293371	145370255	5674	+	-140.75	0.9782	1	2.57	3.89	2.48	1.1	2.25	0.67
cis_mRNA_dw20k	13278	NA	LTCONS_00063836	MTCONS_00063834	chr6_cox_hap2	2394116	2395144	911	+	chr6_cox_hap2	2371882	2380839	3740	+	*	0.9793	1	4.15	3.71	0	1.95	1.44	0.27
cis_mRNA_overlap	0	mRNA	n410592	MTCONS_00061729	chr6	2828801	2841057	1939	-	chr6	2828801	2845430	2629	-	-814.57	0.9078	1	0.04	0.08	0.1	0.02	0.04	0.09
tran	1073978	NA	LTCONS_00002100	MTCONS_00002112	chr1	144175577	144219394	270	+	chr1	145293371	145370255	10498	+	-156.59	0.9832	1	0.77	2.84	0.69	0.61	1.47	0.36
tran	1073978	NA	LTCONS_00002100	MTCONS_00002111	chr1	144175577	144219394	270	+	chr1	145293371	145370255	5674	+	-156.59	0.9729	1	0.77	2.84	0.69	1.1	2.25	0.67
tran	1073978	NA	LTCONS_00002096	MTCONS_00002112	chr1	144162886	144219394	270	+	chr1	145293371	145370255	10498	+	-156.59	0.9832	1	0.77	2.84	0.69	0.61	1.47	0.36
tran	1073978	NA	LTCONS_00002096	MTCONS_00002111	chr1	144162886	144219394	270	+	chr1	145293371	145370255	5674	+	-156.59	0.9729	1	0.77	2.84	0.69	1.1	2.25	0.67
tran	NA	NA	n384963	MTCONS_00034272	chr6	73972950	73986225	1056	+	chr19	37708321	37740417	8180	+	-1173.37	-0.9994	-1	1.31	0.86	0.72	0.07	0.42	0.51
tran	NA	NA	n384963	MTCONS_00034273	chr6	73972950	73986225	1056	+	chr19	37709374	37740417	8724	+	-1173.37	0.9287	1	1.31	0.86	0.72	1.19	0.96	0.65
tran	NA	NA	n325348	MTCONS_00008781	chr2	34462359	34713911	567	+	chr10	97515409	97637023	12236	+	-780	0.7081	0.866	0.07	0	0.26	0.09	0	0.09
cis_mRNA_overlap	0	CompleteIn-LncIntron	n324003	MTCONS_00071534	chr9	108252918	108406897	1313	+	chr9	108320411	108403444	7109	+	*	-0.999	-1	0.03	0.05	0	0.55	0.33	0.94

tran	NA	NA	n376540	MTCONS_00052870	chr20	22255046	22263124	4392	-	chr4	56914919	57200064	10678	+		-2167.33	-0.8974	-1	1.95	1.89	2.22	0.92	2.25	0
cis_mRNA_overlap	0	mRNA	n344541	MTCONS_00016879	chr12	132589591	132615587	4985	+	chr12	132568828	132615587	4881	+		-2575.41	-0.866	-0.866	0.02	0.08	0.14	0.81	0.81	0.79
cis_mRNA_overlap	0	mRNA	n342433	MTCONS_00041342	chr2	96602559	96604956	2398	-	chr2	96487344	96658969	20870	-	*		0.855	1	4.68	5.73	5.85	2.25	2.77	3.55
tran	NA	NA	n334456	MTCONS_00072750	chr5	72743514	72743830	317	-	chr9	69199480	69262593	6928	-		-250.48	-0.9756	-1	0.48	1.14	0.04	1.12	1.01	1.28
tran	NA	NA	n324495	MTCONS_00012799	chr6	53713506	53719779	850	-	chr11	118309253	118409866	27510	+		-747.55	0.9342	1	0.04	0.08	0.11	1.69	2.23	2.28
cis_mRNA_dw20k	10216	NA	n379076	MTCONS_00008471	chr10	75012790	75014102	548	+	chr10	74927877	75002575	6155	+	*		-0.866	-0.866	0.28	0.26	0.27	1.08	1.09	1.08
cis_mRNA_dw20k	10216	NA	n379076	MTCONS_00008469	chr10	75012790	75014102	548	+	chr10	74927877	75002575	3816	+	*		0.9707	1	0.28	0.26	0.27	1.01	0.59	0.89
cis_mRNA_overlap	0	Lnc-AntiOverlap-mRNA	n410117	MTCONS_00046828	chr22	30972612	31003000	2693	-	chr22	30999407	31027721	7200	+	*		0.9971	1	0.28	0.7	0.64	1.69	2.32	2.28
cis_mRNA_overlap	0	mRNA	n406187	MTCONS_00028041	chr16	70010202	70099851	4182	-	chr16	70010190	70099880	4224	-		-2006.02	-0.9875	-1	0.03	0.13	0.16	0.45	0.3	0.21
cis_mRNA_up10k	77	NA	n407212	MTCONS_00006298	chr1	156252704	156262234	2226	+	chr1	156213112	156252628	6094	-	*		0.9594	0.866	0.68	0.99	0.77	1.31	1.94	1.31
cis_mRNA_up10k	77	NA	n407212	MTCONS_00006301	chr1	156252704	156262234	2226	+	chr1	156213112	156252628	8897	-	*		0.9982	1	0.68	0.99	0.77	0	0.93	0.32
cis_mRNA_up10k	77	NA	n407212	MTCONS_00006299	chr1	156252704	156262234	2226	+	chr1	156213112	156252628	10188	-	*		0.9995	1	0.68	0.99	0.77	0.71	0.93	0.78
cis_mRNA_overlap	0	Lnc-CompleteIn-mRNAExon	n411756	MTCONS_00061054	chr6	127587983	127609705	2271	+	chr6	127587732	127609855	2983	+		-992.96	-0.986	-1	7.15	3.43	7	1.45	1.92	1.55
cis_mRNA_overlap	0	Lnc-CompleteIn-mRNAIntron	n341079	MTCONS_00014343	chr11	79059322	79060897	1576	-	chr11	78364328	79151695	13449	-	*		-0.9886	-1	0	0.12	0.06	1.69	0.63	1.02
tran	NA	NA	n341223	MTCONS_00069620	chr7	36359490	36360088	599	+	chr8	38266059	38326352	9657	-		-597.17	0.9516	1	0.18	0.39	0.66	1.01	1.48	1.67
tran	NA	NA	n341223	MTCONS_00008780	chr7	36359490	36360088	599	+	chr10	97515409	97637023	12392	+		-509.36	0.9782	1	0.18	0.39	0.66	0	0.01	0.04
tran	NA	NA	n326082	MTCONS_00052872	chr10	6700684	6707314	499	+	chr4	57081966	57200064	10458	+		-407.42	-0.9992	-1	0.22	0.19	0.08	15.47	18.33	26.84
tran	47345810	NA	n386385	MTCONS_00055679	chr5	68935290	69006272	1469	-	chr5	21459589	21589481	5313	+		-821.12	0.9911	1	3.22	4.55	3.35	0.4	0.53	0.43
cis_mRNA_overlap	0	AntiCompleteIn-mRNAIntron	n407713	MTCONS_00010147	chr10	65224989	65226322	1334	+	chr10	64926164	65281636	9235	-	*		-0.8594	-1	0.29	0.33	0.81	0.45	0.29	0.17
tran	NA	NA	n379147	MTCONS_00030180	chr7	144040335	144052494	568	-	chr17	1472556	1533118	9141	-		-786.49	-0.9983	-1	4.63	8.77	7.76	0.47	0.15	0.21
cis_mRNA_overlap	0	mRNA	n341510	MTCONS_00068552	chr8	61712995	61777685	5901	+	chr8	61592077	61780598	11545	+		-3175.34	0.9769	1	1.62	1.26	1.14	2.76	2.13	2.11
tran	NA	NA	n333001	MTCONS_00076654	chr13	103492685	103493041	357	+	chrY	4868267	4981558	12618	+		-372.51	1	1	0.78	0.48	1.49	4.85	4.11	6.56
cis_mRNA_overlap	0	Lnc-CompleteIn-mRNAExon	n345739	MTCONS_00003821	chr1	14778	16668	507	-	chr1	14050	29439	3053	-		-436.15	-0.749	-1	0.34	0	0.08	0.47	0.66	0.49
tran	NA	NA	n340044	MTCONS_00054075	chr10	64099347	64134886	2621	-	chr4	8363185	8431038	7806	-		-2530.81	-0.7777	-1	0.12	0.07	0.08	0.11	0.43	0.17
cis_mRNA_overlap	0	mRNA	n341249	MTCONS_00064817	chr7	54624663	54639419	3029	+	chr7	54610002	54639419	3912	+	*		1	1	0.48	1.84	0.71	3.43	7.75	4.15
tran	NA	NA	n324422	MTCONS_00010013	chr6	110254418	110256681	465	+	chr10	50720556	50818343	7004	-		-345.46	-0.926	-1	0.16	0	0.25	1.11	1.17	0.97
tran	NA	NA	n371287	MTCONS_00022641	chr9	136125807	136126767	761	-	chr14	75370491	75389974	5464	-		-514.1	0.8928	0.866	0.09	0.13	0.13	1.4	1.85	2.22
tran	NA	NA	n371287	MTCONS_00022643	chr9	136125807	136126767	761	-	chr14	75370491	75439203	4850	-		-514.03	0.9033	0.866	0.09	0.13	0.13	0.52	1.31	0.97
tran	NA	NA	n371287	MTCONS_00022642	chr9	136125807	136126767	761	-	chr14	75370491	75390014	5536	-		-520.32	-0.8733	-0.866	0.09	0.13	0.13	18.41	16.39	14.47
tran	NA	NA	n325325	MTCONS_00069359	chr2	43113876	43120184	786	-	chr8	10958954	11060192	12629	-		-813.24	-0.9407	-1	0.38	0.56	0.53	2.61	2.34	2.47
cis_mRNA_overlap	0	mRNA	n385241	MTCONS_00067128	chr7	97595908	97601638	650	-	chr7	97481372	97602630	4004	-		-545.1	0.9093	1	0.42	1.57	0	1.09	1.26	0.78
cis_mRNA_overlap	0	mRNA	LTCONS_00058235	MTCONS_00058231	chr5	96178473	96271612	2179	-	chr5	96096514	96271606	7562	-		-1188.72	0.993	1	0.36	0.64	0.58	0	0.63	0.57
tran	NA	NA	n376354	MTCONS_00062927	chr19	21747057	21748673	270	-	chr6	88222436	88299735	3594	-		-326.82	0.9967	1	0.13	0.69	0	1.38	2.21	1.08
cis_mRNA_dw20k	18440	NA	n370715	MTCONS_00072750	chr9	69174214	69181041	3182	-	chr9	69199480	69262593	6928	-	*		-0.8558	-1	0.04	0.42	0	1.12	1.01	1.28
tran	NA	NA	n333749	MTCONS_00058317	chr16	2012062	2012526	238	+	chr5	114539713	114632458	16163	-		-412.9	-0.9306	-1	0.54	0	0.32	0.99	1.62	1.02
cis_mRNA_up10k	514	NA	n376781	MTCONS_00042911	chr20	2651191	2673011	464	-	chr20	2673524	2740754	2713	+	*		-0.9174	-1	0.11	0	0.15	5.74	7.39	2.96
tran	NA	NA	n324051	MTCONS_00001491	chr9	90814769	90815213	445	-	chr1	78245281	78506196	5323	+		-492.09	0.6957	1	0.32	0	0.31	3.7	0	0.84
cis_mRNA_dw20k	14911	NA	n368599	MTCONS_00059085	chr5	180253868	180255255	430	-	chr5	180270165	180288363	9165	-	*		0.9873	1	0.09	0.47	0.31	1.9	2.74	2.27
cis_mRNA_overlap	0	mRNA	n342897	MTCONS_00017592	chr12	49521755	49580621	1344	-	chr12	49521565	49582861	1631	-		-818.86	-0.9929	-1	6.63	9.13	40.44	37.22	30.19	1.36
cis_mRNA_overlap	0	mRNA	n405876	MTCONS_00067458	chr7	124462440	124570037	4202	-	chr7	124461305	124570037	5083	-		-1596.25	-0.8988	-1	0.42	0.31	0.07	0.08	0.23	0.29
cis_mRNA_overlap	0	Lnc-CompleteIn-mRNAExon	n407929	MTCONS_00023588	chr15	43477466	43488229	2424	+	chr15	43425687	43489383	6585	+		-1341.38	-0.8027	-1	0.61	0.23	0.6	0	0.58	0.36
cis_mRNA_overlap	0	Lnc-CompleteIn-mRNAExon	n407929	MTCONS_00023590	chr15	43477466	43488229	2424	+	chr15	43425707	43489383	6551	+		-1341.49	1	1	0.61	0.23	0.6	1.13	0.79	1.12
tran	122969	NA	LTCONS_00038439	MTCONS_00041393	chr2	97935463	97958212	16109	+	chr2	98081180	98160993	24447	-		-7062.57	-0.9639	-1	5.84	7.84	6.64	1.57	0.94	1.17
tran	122969	NA	LTCONS_00038439	MTCONS_00041394	chr2	97935463	97958212	16109	+	chr2	98081180	98206428	25798	-		-7302.27	0.9928	1	5.84	7.84	6.64	2.83	3.88	3.36
cis_mRNA_dw20k	1554	NA	n339223	MTCONS_00042763	chr2	242428943	242432702	3751	-	chr2	242434255	242448112	2765	-	*		-0.9999	-1	1.02	1.64	1.19	6.38	5.36	6.11
cis_mRNA_dw20k	1214	NA	n337463	MTCONS_00018622	chr12	123465703	123466268	270	+	chr12	123467481	123635402	7379	-	*		0.9951	1	0.48	0.59	0.15	1.08	1.18	0.53
cis_mRNA_dw20k	1214	NA	n337463	MTCONS_00018621	chr12	123465703	123466268	270	+	chr12	123467481	123635400	8506	-	*		-0.9782	-1	0.48	0.59	0.15	0.94	0.38	1.66
cis_mRNA_dw20k	11018	NA	n410895	MTCONS_00010288	chr10	75556272	75561157	1460	-	chr10	75572174	75634355	3863	-	*		0.8322	0.866	0.9	1.02	1.17	0.84	0.91	0.91
cis_mRNA_overlap	0	Lnc-CompleteIn-mRNAIntron	n332511	NM_004924_dup1	chr19	39203997	39204367	371	+	chr19	39138267	39221171	3954	+	*		0.7996	1	0.94	0.67	0.66	0.49	0.31	0
cis_mRNA_overlap	0	mRNA	n410019	MTCONS_00026015	chr16	16411466	16444465	6295	+	chr16	16411466	16455520	6703	+		-4786.61	0.9692	1	0.59	1.38	4.63	1.22	2.12	3.35
cis_mRNA_overlap	0	mRNA	n410019	MTCONS_00026017	chr16	16411466	16444465	6295	+	chr16	16411466	16456337	7223	+		-4987.45	0.9876	1	0.59	1.38	4.63	0.42	0.45	1.36
cis_mRNA_overlap	0	Lnc-CompleteIn-mRNAExon	n410019	MTCONS_00026008	chr16	16411466	16444465	6295	+	chr16	16410889	16444465	9515	+	*		-0.6858	-1	0.59	1.38	4.63	2.81	0.82	0.71
cis_mRNA_overlap	0	mRNA	n410019	MTCONS_00026016	chr16	16411466	16444465	6295	+	chr16	16411466	16456337	5068	+	*		0.9876	1	0.59	1.38	4.63	0.21	0.31	3.36
cis_mRNA_overlap	0	Lnc-CompleteIn-mRNAExon	n410019	MTCONS_00026011	chr16	16411466	16444465	6295	+	chr16	16410889	16444465	10499	+	*		0.9837	1	0.59	1.38	4.63	4.7	5.11	5.83
cis_mRNA_overlap	0	mRNA	n410019	MTCONS_00026002	chr16	16411466	16444465	6295	+	chr16	16402869	16441369	10176	+	*		0.999	1	0.59	1.38	4.63	0.47	0.71	1.48
cis_mRNA_overlap	0	mRNA	n410019	MTCONS_00026009	chr16	16411466	16444465	6295	+	chr16	16410889	16444465	10413	+	*		0.9621	1	0.59	1.38	4.63	1.82	2.65	3.68
cis_mRNA_overlap	0	mRNA	n410019	MTCONS_00026004	chr16	16411466	16444465	6295	+	chr16	16402869	16441369	8303	+	*		0.9938	1	0.59	1.38	4.63	0	0.06	0.2
cis_mRNA_overlap	0	mRNA	n410019	MTCONS_00026007	chr16	16411466	16444465	6295	+	chr16	16403022	16444465	12506	+		-5240.41	0.9156	1	0.59	1.38	4.63	1.84	2.4	2.83
tran	NA	NA	n409501	MTCONS_00059974	chr8	117886663	117889107	2010	+	chr6	28234788	28286363	24577	+		-1545.82	0.9997	1	0.1	0.33	0.05	0.06	0.39	0
tran	NA	NA	n378469	MTCONS_00026486	chr17	66097708	66111659	707	+	chr16	53175052	53364300	14535	+		-374.73	-0.8943	-0.866	0.15	0.73	0.47	0.21	0.18	0.18
cis_mRNA_overlap	0	Lnc-CompleteIn-mRNAExon	n338963	MTCONS_00038955	chr2	135206960	135212192	5231	+	chr2	134877422	135212270	8785	+		-2969.42	0.9974	1	7.93	9.09	7.51	3.35	4.44	2.81

cis_mRNA_overlap	0	Lnc-CompleteIn-mRNAExon	n338963	MTCONS_00038957	chr2	135206960	135212192	5231	+	chr2	134877422	135212270	8661	+		-2956.11	0.9703	1	7.93	9.09	7.51	4.08	4.53	3.66
cis_mRNA_up10k	51	NA	LTCONS_00019252	MTCONS_00020179	chr13	46626959	46687932	2413	+	chr13	46531334	46626909	5373	-	*		-0.752	-1	2.91	2.98	2.45	0.85	0.01	1.08
cis_mRNA_up10k	51	NA	LTCONS_00019252	MTCONS_00020178	chr13	46626959	46687932	2413	+	chr13	46531334	46626909	5370	-	*		-0.9995	-1	2.91	2.98	2.45	0.84	0.7	2.11
tran	NA	NA	n346456	MTCONS_00068764	chr15	41455552	41456526	668	-	chr8	94712691	94749484	14768	+		-860.95	-0.9183	-1	0.11	0.05	0	0.49	0.62	0.63
tran	NA	NA	n346456	MTCONS_00068761	chr15	41455552	41456526	668	-	chr8	94712686	94749484	11773	+		-860.95	0.891	0.866	0.11	0.05	0	0.04	0.02	0.02
tran	NA	NA	n346456	MTCONS_00068763	chr15	41455552	41456526	668	-	chr8	94712691	94749484	10038	+		-1194.21	0.982	1	0.11	0.05	0	0.54	0.44	0.4
cis_mRNA_dw20k	262	NA	LTCONS_00030560	NM_000676_dup1	chr17	15879471	15903081	1784	-	chr17	15848231	15879210	1885	+	*		-0.9762	-0.866	1.79	2.3	2.45	0.09	0.05	0.05
cis_mRNA_up10k	8745	NA	n410566	MTCONS_00036834	chr19	53430388	53445847	2334	-	chr19	53407555	53421644	4538	-	*		0.8803	1	0.16	0.34	0.1	1.6	1.65	1.49
tran	145488229	NA	n367049	MTCONS_00052416	chr4	148530703	148538396	568	-	chr4	2965178	3042475	6358	+		-624.6	1	1	0.13	0.41	0.23	1.41	1.74	1.53
cis_mRNA_up10k	3226	NA	n338421	MTCONS_00006787	chr1	184727266	184730133	2868	+	chr1	184659578	184724041	6645	-	*		0.8246	1	0.04	0.01	0.14	2.05	1.19	2.31
tran	NA	NA	n380358	MTCONS_00025973	chr11	82783146	82803380	340	+	chr16	15004214	15045993	14649	+		-264.92	0.9509	1	0	0.4	0.91	2.44	3.88	4.45
cis_mRNA_overlap	0	mRNA	n374940	MTCONS_00024265	chr15	91446090	91446964	371	+	chr15	91445629	91466202	7575	+		-385.96	0.7193	1	0.43	0.58	1.96	0.83	1.35	1.48
cis_mRNA_overlap	0	mRNA	n374940	MTCONS_00024264	chr15	91446090	91446964	371	+	chr15	91445629	91466202	7320	+		-385.96	-0.7993	-1	0.43	0.58	1.96	1.41	0.65	0.3
cis_mRNA_up10k	457	NA	n374940	MTCONS_00024269	chr15	91446090	91446964	371	+	chr15	91447420	91466202	8894	+	*		0.9268	1	0.43	0.58	1.96	1.38	1.7	2.08
tran	123123189	NA	LTCONS_00070747	MTCONS_00071976	chr9	9389313	9442239	231	+	chr9	132565427	132575224	7596	+		-223.52	0.9671	1	5.85	3.61	3.07	2.42	2.38	2.35
tran	NA	NA	n381086	MTCONS_00024456	chr1	26551811	26556330	1077	+	chr15	23281011	23378337	7671	-		-780.41	0.9265	1	0.16	0.09	0.44	0.06	0	0.11
cis_mRNA_up10k	3180	NA	n366950	NM_000683_dup1	chr4	3760475	3765117	4525	-	chr4	3768296	3770253	1958	+	*		0.982	1	2.43	3.09	2.31	1.98	2.4	1.77
tran	NA	NA	n324241	MTCONS_00048053	chr7	13860505	13865175	359	-	chr22	50691921	50700089	3929	-		-490.22	-0.7381	-1	3.33	3.24	2.38	2.12	6.79	8.18
tran	11020034	NA	LTCONS_00026233	MTCONS_00027494	chr16	29494866	29495623	758	+	chr16	18451937	18474833	4867	-		-581.85	0.9716	1	0.14	0.39	0.3	8.39	13.37	12.7
tran	NA	NA	LTCONS_00074732	MTCONS_00005308	chrX	102025998	102047742	1022	+	chr1	86810132	86862025	9802	-		-951.88	0.9798	1	0.93	0.94	0.97	1.4	1.65	1.98
tran	NA	NA	n368666	MTCONS_00057412	chr6	35307829	35308188	320	-	chr5	1571958	1586534	12677	-		-292.96	0.8268	1	3.64	4.54	3.7	2.97	3.83	3.5
cis_mRNA_overlap	0	mRNA	n409655	MTCONS_00031439	chr17	56078280	56084707	4722	-	chr17	56066398	56084707	13635	-		-2180.4	0.7837	1	1.19	2.88	2.35	2.32	3.83	2.43
cis_mRNA_overlap	0	mRNA	n409655	MTCONS_00031438	chr17	56078280	56084707	4722	-	chr17	56066398	56084707	15778	-		-2225.43	0.9579	1	1.19	2.88	2.35	0.3	0.81	0.52
cis_mRNA_up10k	3543	NA	n386231	MTCONS_00037070	chr19	59086766	59095763	3342	+	chr19	59080367	59083224	2740	-	*		0.9782	1	8.68	10.79	10.38	1.93	3.43	2.85
cis_mRNA_dw20k	4165	NA	n410934	NM_000704_dup1	chr19	36031640	36036931	1627	-	chr19	36041095	36054560	3559	-	*		0.7936	1	0.74	0.73	0.88	0.02	0	0.03
tran	NA	NA	n410934	MTCONS_00030317	chr19	36031640	36036931	1627	-	chr17	5005757	5017891	5800	-		-1477.57	-0.8609	-1	0.74	0.73	0.88	0.94	1.31	0.65
cis_mRNA_overlap	0	mRNA	n324126	MTCONS_00070895	chr9	34655662	34671295	665	+	chr9	34646573	34661974	3264	+	*		0.9991	1	0.16	0.21	0.44	2.75	2.98	3.8
tran	NA	NA	n375451	MTCONS_00005308	chr17	12453285	12540504	2605	+	chr1	86810132	86862025	9802	-		-1708.42	-0.8424	-1	0.42	0.17	0.16	1.4	1.65	1.98
cis_mRNA_dw20k	8485	NA	n381754	MTCONS_00014441	chr11	93454680	93455031	352	+	chr11	93463515	93474703	1516	-	*		0.9953	1	21.22	25.27	16.72	0.72	1	0.28
cis_mRNA_dw20k	8485	NA	n381754	MTCONS_00014443	chr11	93454680	93455031	352	+	chr11	93463515	93474703	4660	-	*		0.9551	1	21.22	25.27	16.72	4.2	4.56	2.78
cis_mRNA_dw20k	8485	NA	n381754	MTCONS_00014446	chr11	93454680	93455031	352	+	chr11	93463515	93474703	1953	-	*		0.9086	1	21.22	25.27	16.72	9.91	10.27	5.09
cis_mRNA_overlap	0	mRNA	n410257	MTCONS_00059522	chr6	4043970	4046384	1360	+	chr6	4021533	4065268	6805	+	*		0.9999	1	0.38	0.61	0.5	1.15	2.92	2.05
cis_mRNA_overlap	0	mRNA	n410257	MTCONS_00059521	chr6	4043970	4046384	1360	+	chr6	4021533	4065268	5114	+	*		0.9594	1	0.38	0.61	0.5	2.7	6.01	3.59
tran	NA	NA	n635	MTCONS_00033625	chr2	47562454	47562653	200	+	chr19	10764937	10803102	6693	+		-153.59	0.893	1	6.17	4.14	7.7	3.02	2.82	4.17
cis_mRNA_overlap	0	Lnc-CompleteIn-mRNAIntron	n339939	NM_018702_dup1	chr10	1657287	1659814	2338	-	chr10	1223253	1779670	8426	-	*		0.9998	1	0.1	0.25	0.04	0.32	0.96	0.04
cis_mRNA_dw20k	3261	NA	LTCONS_00048848	MTCONS_00050861	chr3	52570621	52574586	316	+	chr3	52577846	52719610	9589	-	*		-0.9764	-0.866	2.37	1.79	2.54	0.46	0.56	0.46
cis_mRNA_dw20k	3261	NA	LTCONS_00048848	MTCONS_00050864	chr3	52570621	52574586	316	+	chr3	52577846	52719956	9457	-	*		-0.9894	-1	2.37	1.79	2.54	0.5	0.73	0.48
cis_mRNA_dw20k	3261	NA	LTCONS_00048848	MTCONS_00050866	chr3	52570621	52574586	316	+	chr3	52577846	52719959	9528	-	*		0.8643	1	2.37	1.79	2.54	1.52	1.34	1.93
cis_mRNA_dw20k	3261	NA	LTCONS_00048848	MTCONS_00050867	chr3	52570621	52574586	316	+	chr3	52577846	52719959	9399	-	*		-0.9536	-1	2.37	1.79	2.54	0.76	0.93	0.59
cis_mRNA_dw20k	1286	NA	n380573	MTCONS_00018622	chr12	123413548	123466196	6482	-	chr12	123467481	123635402	7379	-	*		0.6371	1	0.64	1	0.63	1.08	1.18	0.53
cis_mRNA_dw20k	1286	NA	n380573	MTCONS_00018621	chr12	123413548	123466196	6482	-	chr12	123467481	123635400	8506	-	*		-0.8409	-1	0.64	1	0.63	0.94	0.38	1.66
tran	NA	NA	n332817	MTCONS_00048052	chr6	3448891	3449539	445	-	chr22	50691921	50700089	3899	-		-436.81	0.9886	1	0.27	0.37	0	5.65	5.82	4.44
cis_mRNA_overlap	0	Lnc-AntiOverlap-mRNA	n407638	MTCONS_00053779	chr4	183060159	183065668	4953	-	chr4	183065063	183724267	11238	+	*		0.9854	1	1.89	1.22	3.81	2.06	1.26	3.2
cis_mRNA_overlap	0	Lnc-CompleteIn-mRNAExon	n408057	MTCONS_00045933	chr21	34106210	34144169	4012	-	chr21	34105977	34144169	8478	-		-1669.16	-0.9992	-1	4	4.03	1.4	1.92	1.84	3.26
tran	357211	NA	LTCONS_00036843	MTCONS_00034863	chr19	53467065	53541136	7498	-	chr19	53898346	53962049	4462	+		-2001.8	-0.9475	-1	0.28	0.69	0.97	2.13	1.31	1.22
tran	394092	NA	LTCONS_00036843	MTCONS_00034869	chr19	53467065	53541136	7498	-	chr19	53935227	53962049	4648	+		-2172.19	-0.9076	-1	0.28	0.69	0.97	0.67	0.62	0.43
tran	NA	NA	n380729	MTCONS_00009585	chr14	19655782	19656886	413	+	chr10	13672872	13933777	19431	-		-277.47	-0.9994	-1	0.09	0	0.17	0.11	0.14	0.08
tran	NA	NA	n380729	MTCONS_00009583	chr14	19655782	19656886	413	+	chr10	13672872	13752203	21653	-		-354.82	0.9401	1	0.09	0	0.17	0.51	0.44	0.76
tran	372168	NA	n383551	MTCONS_00034859	chr19	53471505	53496784	2996	-	chr19	53868951	53890918	7088	+		-1352.11	-0.877	-1	1.57	5.61	2.54	0.53	0.34	0.4
cis_mRNA_up10k	185	NA	n370541	MTCONS_00068883	chr8	104493481	104511823	2585	-	chr8	104512007	105267094	8765	+	*		0.7655	1	1.4	1.03	1.48	3.51	3.05	5.46
tran	5584756	NA	n382317	MTCONS_00023326	chr15	23282265	23311039	3358	-	chr15	28895794	28930558	6311	+		-1743.1	0.9989	1	0.02	1.31	1.62	1.14	3.28	3.65
cis_mRNA_overlap	0	mRNA	n382317	MTCONS_00024457	chr15	23282265	23311039	3358	-	chr15	23281011	23378337	7754	-	*		0.9897	1	0.02	1.31	1.62	0	1.64	2.44
tran	5521531	NA	n382317	MTCONS_00023315	chr15	23282265	23311039	3358	-	chr15	28832569	28930558	8121	+		-1900.29	0.8287	1	0.02	1.31	1.62	0.03	0.14	0.43
cis_mRNA_overlap	0	Lnc-CompleteIn-mRNAIntron	n340301	MTCONS_00057364	chr5	763495	764229	722	-	chr5	745253	892939	3119	-	*		-0.8967	-1	0.85	0.64	0.53	1.65	2.26	4.08
cis_mRNA_overlap	0	mRNA	n346116	MTCONS_00057417	chr5	1571958	1594584	3650	-	chr5	1574556	1594678	11153	-		-1786.25	0.9963	1	0.27	0.12	0.07	1.14	0.84	0.78
cis_mRNA_overlap	0	mRNA	n346116	MTCONS_00057415	chr5	1571958	1594584	3650	-	chr5	1571958	1594678	1490	-		-766.35	0.8836	1	0.27	0.12	0.07	1.96	1.74	1.27
tran	NA	NA	n334378	MTCONS_00001031	chr19	5208250	5210593	266	-	chr1	43996547	44089953	7503	+		-200.94	0.7736	1	0	0.16	0.15	2.63	3.15	2.79
tran	NA	NA	n334378	MTCONS_00001028	chr19	5208250	5210593	266	-	chr1	43996540	44089953	7516	+		-200.94	0.9723	1	0	0.16	0.15	1.64	5.53	4.38
tran	NA	NA	n334378	MTCONS_00001027	chr19	5208250	5210593	266	-	chr1	43996540	44089953	7477	+		-200.94	-0.9889	-1	0	0.16	0.15	0.46	0.18	0.24
tran	NA	NA	n334378	MTCONS_00001026	chr19	5208250	5210593	266	-	chr1	43996537	44089953	7468	+		-200.94	-0.9068	-1	0	0.16	0.15	0.53	0	0.25
cis_mRNA_up10k	178	NA	n341248	MTCONS_00066731	chr7	50850753	50853616	2863	+	chr7	50657480	50850576	5538	-	*		0.9999	0.866	0.02	0.02	0.06	0.83	0.86	2.49
cis_mRNA_up10k	31	NA	n341248	MTCONS_00066737	chr7	50850753	50853616	2863	+	chr7	50657480	50850723	5611	-	*		-0.8532	-0.866	0.02	0.02	0.06	0.99	1.11	0.88
cis_mRNA_up10k	178	NA	n341248	MTCONS_00066732	chr7	50850753	50853616	2863	+	chr7	50657480	50850576	5087	-	*		0.8486	0.866	0.02	0.02	0.06	1.63	0.82	2.35
cis_mRNA_up10k	93	NA	n341248	MTCONS_00066733	chr7	50850753	50853616	2863	+	chr7	50657480	50850661	5642	-	*		0.9978	0.866	0.02	0.02	0.06	1.19	1.17	1.44
cis_mRNA_overlap	0	mRNA	n364378	MTCONS_00002194	chr1	148556023	148557204	733	+	chr1	148556011	148596267	5017	+		-513.27	-0.678	-1	3.15	4.54	3.19	4.57	4.23	4.3
cis_mRNA_overlap	0	mRNA	n364378	MTCONS_00002190	chr1	148556023	148557204	733	+	chr1	148555358	148596267	5492	+		-508.15	0.9997	0.866	3.15	4.54	3.19	0.27	0.36	0.27
cis_mRNA_dw20k	3520	NA	n341680	MTCONS_00070896	chr9	34665493	34669254	3759	+	chr9	34646573	34661974	5657	+	*		0.9998	1	0.72	1.12	0.99	5.05	6.8	6.2
tran	NA	NA	n339200	MTCONS_00048053	chr16	47563101	47563827	494	+	chr22	50691921	50700089	3929	-		-339.31	0.9757	0.866	0.08	0.14	0.14	2.12	6.79	8.18

tran	NA	NA	LTCONS_00013840	MTCONS_00026094	chr11	58646457	58673199	5255	-	chr16	22445951	22518194	21000	+		-4427.29	-0.9662	-1	1.34	0.89	1.74	3.82	4.56	3.59
cis_mRNA_overlap	0	Lnc-AntiOverlap-mRNA	n406370	NM_130786_dup1	chr19	58863336	58866549	2130	+	chr19	58858172	58864865	1766	-	*		0.9441	1	1.57	1.25	2.12	2.56	2.53	3.26
cis_mRNA_dw20k	16405	NA	n345847	MTCONS_00035075	chr19	58024239	58030244	3104	+	chr19	57999079	58007835	4051	+	*		-0.8824	-1	0.67	1.06	0.96	0.29	0	0.2
tran	NA	NA	n382914	MTCONS_00072843	chr16	3087252	3087800	549	-	chr9	79226141	79307110	10092	-		-319.2	-0.6567	-1	0.07	0.06	0.24	1.17	2.82	0.87
tran	NA	NA	n325608	MTCONS_00039113	chr14	100485068	100488842	610	+	chr2	162480845	162841786	11838	+		-504.21	-0.9991	-1	0.3	0.11	0.21	0.8	1.76	1.22
cis_mRNA_up10k	1678	NA	n376687	MTCONS_00044214	chr20	16555756	16559931	1402	+	chr20	16252439	16554079	5301	-	*		0.8669	1	0.15	0.18	0.35	0.97	1.28	1.47
tran	NA	NA	n325730	MTCONS_00012040	chr13	49993070	49999586	731	-	chr11	63996107	63997566	1327	+		-368.07	-0.9924	-1	0.25	0.13	0.14	0.67	1.87	1.62
cis_mRNA_overlap	0	mRNA	n325598	MTCONS_00024455	chr15	23281071	23375031	2491	-	chr15	23281011	23378259	7914	-		-1467.78	-0.956	-1	0.56	0.02	0.03	0	4.18	2.86
cis_mRNA_overlap	0	mRNA	n325598	MTCONS_00024454	chr15	23281071	23375031	2491	-	chr15	23281011	23378259	8982	-		-1539.13	-1	-1	0.56	0.02	0.03	0.35	4.57	4.5
cis_mRNA_up10k	31	NA	n341560	MTCONS_00068764	chr8	94358695	94712661	1914	-	chr8	94712691	94749484	14768	+	*		0.7963	1	0.07	0.08	0.1	0.49	0.62	0.63
cis_mRNA_up10k	26	NA	n341560	MTCONS_00068761	chr8	94358695	94712661	1914	-	chr8	94712686	94749484	11773	+	*		-0.7559	-0.866	0.07	0.08	0.1	0.04	0.02	0.02
cis_mRNA_up10k	31	NA	n341560	MTCONS_00068763	chr8	94358695	94712661	1914	-	chr8	94712691	94749484	10038	+	*		-0.9078	-1	0.07	0.08	0.1	0.54	0.44	0.4
cis_mRNA_dw20k	18046	NA	n381584	MTCONS_00011098	chr11	856880	858707	1828	-	chr11	818901	838835	13012	+	*		-0.7053	-1	1	1.01	0.88	0.04	0	0.05
tran	NA	NA	n338263	MTCONS_00000234	chr19	2610153	2611860	1707	-	chr1	6640063	6649340	7313	+		-1665.28	-0.9889	-0.866	0.02	0.12	0.02	0.58	0	0.69
tran	NA	NA	n338263	MTCONS_00000237	chr19	2610153	2611860	1707	-	chr1	6640063	6649340	5957	+		-1665.28	0.6911	0.866	0.02	0.12	0.02	0.55	2.21	1.8
tran	NA	NA	n338263	MTCONS_00000235	chr19	2610153	2611860	1707	-	chr1	6640063	6649340	6555	+		-1665.28	0.9738	0.866	0.02	0.12	0.02	1.23	1.84	1.04
cis_mRNA_overlap	0	Lnc-AntiOverlap-mRNA	n377656	MTCONS_00053779	chr4	183062803	183065619	2260	-	chr4	183065063	183724267	11238	+	*		-0.9871	-1	0.39	0.87	0	2.06	1.26	3.2
cis_mRNA_dw20k	9433	NA	n407979	MTCONS_00000059	chr1	910579	917473	3040	-	chr1	896282	901147	3204	+	*		-0.9639	-1	0.02	0	0.01	1.36	2.05	1.54
cis_mRNA_dw20k	9433	NA	n407979	MTCONS_00000057	chr1	910579	917473	3040	-	chr1	896197	901147	3658	+	*		-0.8837	-1	0.02	0	0.01	0.77	1.74	0.81
cis_mRNA_dw20k	9433	NA	n407979	MTCONS_00000060	chr1	910579	917473	3040	-	chr1	896282	901147	3083	+	*		0.9972	1	0.02	0	0.01	1.2	0.58	0.93
cis_mRNA_overlap	0	Lnc-AntiOverlap-mRNA	n409147	MTCONS_00030101	chr17	80347086	80376513	4505	-	chr17	80375909	80400611	3446	+	*		0.7492	1	0.41	0.29	0.83	0.76	0.32	0.82
tran	NA	NA	LTCONS_00022338	MTCONS_00062598	chr14	50320337	50368213	288	-	chr6	43004263	43027309	7588	-		-254.15	-0.8672	-1	6.04	5	13.16	0.14	0.23	0.08
cis_mRNA_overlap	0	mRNA	n339619	MTCONS_00049565	chr3	141121660	141161439	649	+	chr3	141106243	141168942	8427	+		-337.79	0.9392	1	0.5	0.25	0.77	1.31	1.11	2.35
cis_mRNA_overlap	0	mRNA	n339619	MTCONS_00049564	chr3	141121660	141161439	649	+	chr3	141103456	141168942	10025	+		-347.11	0.9544	1	0.5	0.25	0.77	0.22	0.13	0.25
tran	NA	NA	n373455	MTCONS_00005308	chr12	128433497	128435791	1280	+	chr1	86810132	86862025	9802	-		-881.22	0.9039	1	1.95	2.24	2.3	1.4	1.65	1.98
cis_mRNA_dw20k	7303	NA	LTCONS_00025416	MTCONS_00024263	chr15	91473504	91476295	2384	-	chr15	91445629	91466202	9482	+	*		-0.8529	-1	1.35	1.1	1.12	1.5	2.03	1.72
tran	25648284	NA	n336640	MTCONS_00059974	chr6	2584945	2586505	726	-	chr6	28234788	28286363	24577	+		-794.07	0.9078	1	0.12	0.22	0	0.06	0.39	0
cis_mRNA_overlap	0	Lnc-CompleteIn-mRNAExon	n341577	MTCONS_00068883	chr8	105080740	105161076	516	+	chr8	104512007	105267094	8765	+		-303.38	0.9676	1	19.01	14.32	25.38	3.51	3.05	5.46
cis_mRNA_up10k	75	NA	n382029	MTCONS_00020179	chr13	46626983	46675482	3508	+	chr13	46531334	46626909	5373	-	*		-0.9918	-1	0.52	0.8	0.38	0.85	0.01	1.08
cis_mRNA_up10k	75	NA	n382029	MTCONS_00020178	chr13	46626983	46675482	3508	+	chr13	46531334	46626909	5370	-	*		-0.8118	-1	0.52	0.8	0.38	0.84	0.7	2.11
cis_mRNA_overlap	0	mRNA	n387328	MTCONS_00057414	chr5	1568637	1579695	1026	-	chr5	1571958	1594646	6957	-		-546.06	0.9774	1	1.45	2.68	1.82	0.71	2.32	0.86
cis_mRNA_overlap	0	mRNA	n387328	MTCONS_00057412	chr5	1568637	1579695	1026	-	chr5	1571958	1586534	12677	-	*		0.9361	1	1.45	2.68	1.82	2.97	3.83	3.5
cis_mRNA_overlap	0	Lnc-CompleteIn-mRNAIntron	n332870	MTCONS_00023313	chr15	28839838	28840249	412	+	chr15	28832569	28930558	8205	+	*		-0.9999	-1	0.84	1.32	2.08	0.1	0.06	0
cis_mRNA_overlap	0	Lnc-CompleteIn-mRNAIntron	n332870	MTCONS_00023315	chr15	28839838	28840249	412	+	chr15	28832569	28930558	8121	+	*		0.9923	1	0.84	1.32	2.08	0.03	0.14	0.43
cis_mRNA_overlap	0	Lnc-AntiOverlap-mRNA	n378912	MTCONS_00054564	chr4	85887538	85932430	6434	+	chr4	85589145	85887544	15720	-	*		-0.866	-1	0.35	0.4	0.42	0.28	0.21	0
cis_mRNA_overlap	0	AntiCompleteIn-mRNAIntron	n341880	MTCONS_00017391	chr12	30908008	30933673	1552	+	chr12	30862388	30976321	8243	-	*		-0.9978	-1	0.04	0	0.02	0.68	1.38	0.99
cis_mRNA_up10k	176	NA	n341880	MTCONS_00017384	chr12	30908008	30933673	1552	+	chr12	30862388	30907833	4815	-	*		0.9542	1	0.04	0	0.02	1.42	1.07	1.34
cis_mRNA_up10k	121	NA	n341880	MTCONS_00017388	chr12	30908008	30933673	1552	+	chr12	30862388	30907888	5378	-	*		-0.982	-1	0.04	0	0.02	2.21	3.65	2.69
tran	NA	NA	LTCONS_00019264	MTCONS_00035110	chr13	47730192	47844471	400	+	chr19	58330978	58380311	10514	+		-345.18	0.823	1	8.8	1.87	0.93	0.09	0.07	0.03
cis_mRNA_dw20k	17294	NA	n410743	MTCONS_00035631	chr19	12786531	12792701	1248	-	chr19	12809994	12835433	6132	-	*		0.9737	1	2.68	1.76	3.14	1.11	0.64	1.17
cis_mRNA_dw20k	17294	NA	n410743	MTCONS_00035630	chr19	12786531	12792701	1248	-	chr19	12809994	12834810	6281	-	*		-0.7805	-1	2.68	1.76	3.14	0.77	0.78	0.55
cis_mRNA_overlap	0	Lnc-AntiOverlap-mRNA	n339711	MTCONS_00044730	chr20	45919358	45920745	1388	+	chr20	45837599	45985925	6010	-	*		-0.9714	-1	0.07	0.15	0.11	2.72	1.02	1.51
cis_mRNA_overlap	0	Lnc-AntiOverlap-mRNA	n339711	MTCONS_00044731	chr20	45919358	45920745	1388	+	chr20	45837599	45985927	5866	-	*		1	1	0.07	0.15	0.11	2.87	5.03	3.94
cis_mRNA_overlap	0	mRNA-AntiCompleteIn-LncIntron	n379687	NM_005159_dup1	chr15	35047285	35105124	2727	+	chr15	35080297	35087927	3693	-	*		0.7506	1	0.01	0.06	0.08	0.07	0.08	0.27
cis_mRNA_overlap	0	Lnc-CompleteIn-mRNAIntron	n339377	MTCONS_00048882	chr3	57886576	57887872	1297	+	chr3	57741919	57916105	6759	+	*		-1	-1	0.16	0.26	0.24	0.88	0.34	0.45
cis_mRNA_dw20k	11164	NA	LTCONS_00047513	MTCONS_00046580	chr22	24235751	24244301	2881	-	chr22	24200003	24224588	5746	+	*		-0.7954	-0.866	0.09	0	0.09	0.91	1.45	1.24
cis_mRNA_dw20k	4690	NA	LTCONS_00047513	MTCONS_00046581	chr22	24235751	24244301	2881	-	chr22	24200003	24231062	6811	+	*		-0.9974	-0.866	0.09	0	0.09	1.01	1.47	0.97
cis_mRNA_dw20k	11164	NA	LTCONS_00047513	MTCONS_00046582	chr22	24235751	24244301	2881	-	chr22	24200041	24224588	5921	+	*		-0.8881	-0.866	0.09	0	0.09	3.82	6.71	5.15
cis_mRNA_dw20k	4690	NA	LTCONS_00047513	MTCONS_00046579	chr22	24235751	24244301	2881	-	chr22	24199901	24231062	11486	+	*		-0.7017	-0.866	0.09	0	0.09	0.37	1.06	0.88
tran	99712	NA	LTCONS_00027646	MTCONS_00027653	chr16	29435949	29436215	267	-	chr16	29535926	29625059	11433	-		-178.01	-0.8668	-1	1.49	1.91	2.11	0.61	0.48	0
tran	99712	NA	LTCONS_00027646	MTCONS_00027654	chr16	29435949	29436215	267	-	chr16	29535926	29625059	11402	-		-178.01	0.9869	1	1.49	1.91	2.11	2.15	2.38	2.58
tran	NA	NA	n380975	MTCONS_00052416	chr12	60486645	60489632	769	+	chr4	2965178	3042475	6358	+		-694.68	0.9933	1	0.61	1.04	0.81	1.41	1.74	1.53
tran	NA	NA	n372028	MTCONS_00024457	chr10	69609270	69610353	871	+	chr15	23281011	23378337	7754	-		-569.22	0.8453	1	0.55	0.56	0.61	0	1.64	2.44
cis_mRNA_overlap	0	Lnc-AntiOverlap-mRNA	n406446	MTCONS_00022114	chr14	24025198	24028787	1239	+	chr14	24028683	24048373	7750	-	*		-0.9815	-1	6.08	5.39	6.57	0.76	1.29	0.02
tran	430867	NA	n381059	MTCONS_00000469	chr1	16804750	16817579	1122	-	chr1	17248445	17299508	8051	+		-1035.87	-0.7747	-0.866	1.13	0.96	0.96	0.8	1.03	1.44
tran	430867	NA	n381059	MTCONS_00000467	chr1	16804750	16817579	1122	-	chr1	17248445	17299508	6964	+		-1037.46	-0.843	-0.866	1.13	0.96	0.96	0.05	0.17	0.31
cis_mRNA_overlap	0	AntiCompleteIn-mRNAIntron	n344531	MTCONS_00017391	chr12	30948615	30963541	3145	+	chr12	30862388	30976321	8243	-	*		0.9644	1	0	0.05	0.01	0.68	1.38	0.99

cis_mRNA_overlap	0	Lnc-AntiOverlap-mRNA	n338026	MTCONS_00025136	chr15	75660496	75661920	1425	+	chr15	75647592	75748154	11092	-		-2645.98	0.9999	1	0.66	0.56	1.09	0.06	0.04	0.14
cis_mRNA_up10k	226	NA	n338395	MTCONS_00002857	chr1	179922319	179923575	1257	-	chr1	179923800	180084015	13434	+	*		-0.8083	-1	0.7	0.66	0.89	1.79	2.47	1.55
cis_mRNA_up10k	192	NA	n338395	MTCONS_00002856	chr1	179922319	179923575	1257	-	chr1	179923766	180084015	13718	+	*		0.6607	1	0.7	0.66	0.89	4.07	3.58	4.09
tran	NA	NA	n342388	MTCONS_00025134	chr19	42836016	42836379	364	+	chr15	75647592	75744087	12970	-		-297.3	-0.9838	-0.866	0.67	0.1	0.1	2.31	2.94	2.82
cis_mRNA_overlap	0	Lnc-AntiOverlap-mRNA	LTCONS_00027480	MTCONS_00026012	chr16	16425410	16470714	2012	-	chr16	16410889	16464173	5350	+	*		0.9936	1	0.06	0.25	0.17	2.69	4.03	3.6
cis_mRNA_overlap	0	Lnc-AntiOverlap-mRNA	LTCONS_00027480	MTCONS_00026019	chr16	16425410	16470714	2012	-	chr16	16456592	16470859	1890	+	*		-0.9909	-1	0.06	0.25	0.17	3.17	2.91	3.05
cis_mRNA_up10k	3686	NA	LTCONS_00022336	NM_001663_dup1	chr14	50320337	50356051	269	-	chr14	50359736	50363772	3939	+	*		-0.7945	-1	4.08	2.78	0	9.99	10.95	11.04
tran	NA	NA	n381333	MTCONS_00057417	chr1	205831207	205865215	2594	+	chr5	1574556	1594678	11153	-		-1287.62	-0.6286	-0.866	0.32	0.32	2.29	1.14	0.84	0.78
cis_mRNA_dw20k	5940	NA	n374898	MTCONS_00023326	chr15	28936497	28944750	375	+	chr15	28895794	28930558	6311	+	*		-0.9906	-0.866	0.62	0.19	0.19	1.14	3.28	3.65
cis_mRNA_dw20k	5940	NA	n374898	MTCONS_00023316	chr15	28936497	28944750	375	+	chr15	28834591	28930558	5989	+	*		0.6286	0.866	0.62	0.19	0.19	1.67	1.01	1.56
cis_mRNA_dw20k	5940	NA	n374898	MTCONS_00023313	chr15	28936497	28944750	375	+	chr15	28832569	28930558	8205	+	*		0.803	0.866	0.62	0.19	0.19	0.1	0.06	0
cis_mRNA_dw20k	5940	NA	n374898	MTCONS_00023315	chr15	28936497	28944750	375	+	chr15	28832569	28930558	8121	+	*		-0.7125	-0.866	0.62	0.19	0.19	0.03	0.14	0.43
tran	NA	NA	n324919	MTCONS_00035736	chr20	60735569	60737659	461	+	chr19	15462350	15529852	8576	-		-566.98	-0.6363	-0.866	0.82	0.82	0.58	0.39	0.53	0.56
cis_mRNA_dw20k	2820	NA	n407588	MTCONS_00046465	chr22	21356211	21364663	2616	+	chr22	21321447	21353392	7819	+	*		-0.7831	-1	0.39	0.36	0.13	0	0.21	0.29
cis_mRNA_overlap	0	Lnc-AntiOverlap-mRNA	n339662	MTCONS_00043350	chr20	34738335	34743655	2701	-	chr20	34742580	34820799	6433	+	*		-0.9999	-1	0.52	0.76	0.65	10.22	9.66	9.91
cis_mRNA_overlap	0	CompleteIn-LncExon	LTCONS_00002595	MTCONS_00002600	chr1	161068130	161070194	1540	+	chr1	161068151	161070194	984	+		-637.07	-0.9679	-1	1.3	2.88	2.09	1.13	0.33	0.91
cis_mRNA_overlap	0	mRNA	LTCONS_00002595	MTCONS_00002599	chr1	161068130	161070194	1540	+	chr1	161068151	161070194	1780	+		-879.93	-0.9992	-1	1.3	2.88	2.09	1.48	0.08	0.73
cis_mRNA_overlap	0	mRNA	LTCONS_00002595	MTCONS_00002597	chr1	161068130	161070194	1540	+	chr1	161068151	161070194	1509	+		-868.63	0.9707	1	1.3	2.88	2.09	1.44	1.58	1.54
cis_mRNA_dw20k	10899	NA	n381258	MTCONS_00006273	chr1	155596547	155618335	1287	-	chr1	155629233	155827026	4852	-	*		0.992	1	2.92	3.48	2.79	0.94	2.08	0.87
tran	NA	NA	n367778	MTCONS_00071211	chr5	89196481	89200725	584	+	chr9	82186862	82348271	11916	+		-597.67	0.9168	1	0.13	0.24	0.23	1.15	1.34	1.25
cis_mRNA_up10k	88	NA	n381277	NM_000696_dup1	chr1	165667987	165679197	1208	+	chr1	165631449	165667900	2500	-	*		0.9729	0.866	0.03	0.03	0	12.14	12.62	10.63
tran	25339053	NA	n410087	MTCONS_00059973	chr6	2880257	2895736	2186	+	chr6	28234788	28286363	24413	+		-1724.36	-0.8878	-1	0.14	0.39	0.29	1.46	1.05	1.39
cis_mRNA_overlap	0	Lnc-CompleteIn-mRNAExon	n335714	MTCONS_00071212	chr9	82186892	82187391	500	+	chr9	82186868	82348271	11300	+		-279.86	-0.9018	-1	0.14	0	0.42	0.76	0.92	0.7
cis_mRNA_up10k	177	NA	LTCONS_00050264	MTCONS_00048204	chr3	9427509	9439178	1748	-	chr3	9439354	9519901	8247	+	*		-0.8493	-1	6	0.35	3.99	1.48	2.11	2.02
cis_mRNA_up10k	177	NA	LTCONS_00050264	MTCONS_00048203	chr3	9427509	9439178	1748	-	chr3	9439354	9519901	7357	+	*		0.972	1	6	0.35	3.99	1.73	1.35	1.68
cis_mRNA_up10k	177	NA	LTCONS_00050264	MTCONS_00048205	chr3	9427509	9439178	1748	-	chr3	9439354	9519901	7795	+	*		-0.9822	-1	6	0.35	3.99	0	2.67	1.39
cis_mRNA_up10k	173	NA	LTCONS_00050264	MTCONS_00048198	chr3	9427509	9439178	1748	-	chr3	9439350	9519901	9023	+	*		-0.9431	-1	6	0.35	3.99	0.66	1.55	0.68
cis_mRNA_up10k	173	NA	LTCONS_00050264	MTCONS_00048199	chr3	9427509	9439178	1748	-	chr3	9439350	9519901	7232	+	*		0.9902	1	6	0.35	3.99	1.19	0.97	1.14
tran	NA	NA	n367080	MTCONS_00036834	chr4	180087670	180091375	671	-	chr19	53407555	53421644	4538	-		-617.97	-0.9994	-1	0.22	0.11	0.43	1.6	1.65	1.49
cis_mRNA_up10k	1009	NA	n386327	MTCONS_00048200	chr3	9433389	9438342	2606	-	chr3	9439350	9519901	7913	+	*		-0.9989	-1	1.29	1.98	2.11	1.78	0.32	0.14
cis_mRNA_overlap	0	Lnc-AntiOverlap-mRNA	n407565	MTCONS_00006254	chr1	155531772	155533735	1964	+	chr1	155304682	155658823	12763	-		-2349.64	0.9174	1	1.28	1.71	1.4	0.11	0.72	0.5
cis_mRNA_dw20k	2	NA	LTCONS_00047430	MTCONS_00046465	chr22	21353393	21357476	3870	-	chr22	21321447	21353392	7819	+	*		-0.7738	-1	2	1.97	1.7	0	0.21	0.29
tran	NA	NA	n325250	MTCONS_00048053	chr2	75000190	75005481	763	-	chr22	50691921	50700089	3929	-		-434.59	-0.7414	-1	0.85	0.84	0.75	2.12	6.79	8.18
tran	NA	NA	n364842	MTCONS_00057412	chr2	3616113	3622828	2541	-	chr5	1571958	1586534	12677	-		-2579.22	0.9937	1	1.85	2.7	2.46	2.97	3.83	3.5
cis_mRNA_dw20k	10186	NA	n375216	NM_001116_dup1	chr16	3993168	4002465	4042	-	chr16	4012650	4166186	7727	-	*		0.8945	1	0	0.48	0.39	5.09	6.17	5.52
cis_mRNA_overlap	0	Lnc-AntiOverlap-mRNA	n324514	MTCONS_00062599	chr6	43016080	43019686	679	+	chr6	43004263	43027309	8306	-		-902.78	-0.9153	-1	0.16	0	0.1	1.44	2.25	2.04
cis_mRNA_overlap	0	mRNA	n411666	MTCONS_00027354	chr16	4746511	4784378	2667	-	chr16	4745890	4781575	5148	-	*		0.9939	1	2.1	2.05	3.07	0.7	0.57	1.36
cis_mRNA_overlap	0	mRNA	n411666	MTCONS_00027361	chr16	4746511	4784378	2667	-	chr16	4745890	4784378	4808	-		-2135.33	-0.9749	-1	2.1	2.05	3.07	3.18	3.64	1.96
cis_mRNA_overlap	0	Lnc-AntiOverlap-mRNA	n406598	MTCONS_00006275	chr1	155715609	155721369	2524	+	chr1	155718230	155827026	7922	-		-2350.35	0.9177	1	2.54	2.66	2.55	0.9	2.49	1.64
cis_mRNA_overlap	0	AntiCompleteIn-mRNAIntron	n406598	MTCONS_00006272	chr1	155715609	155721369	2524	+	chr1	155629233	155826991	5022	-	*		-0.9982	-1	2.54	2.66	2.55	1.86	1.04	1.74
cis_mRNA_overlap	0	Lnc-AntiOverlap-mRNA	n406598	MTCONS_00006276	chr1	155715609	155721369	2524	+	chr1	155719419	155827078	7846	-		-2147.54	-0.767	-1	2.54	2.66	2.55	3.01	1.62	2.01
cis_mRNA_overlap	0	Lnc-AntiOverlap-mRNA	n406598	MTCONS_00006274	chr1	155715609	155721369	2524	+	chr1	155718230	155733619	5186	-		-2350.35	0.9246	1	2.54	2.66	2.55	0.92	1.52	1.19
tran	NA	NA	n372245	MTCONS_00024455	chr10	130803350	130804436	892	-	chr15	23281011	23378259	7914	-		-785.97	0.997	1	0.62	1.14	0.94	0	4.18	2.86
tran	NA	NA	LTCONS_00048358	MTCONS_00062598	chr3	15771428	15780228	371	+	chr6	43004263	43027309	7588	-		-322.98	0.8128	1	3.49	3.51	2.46	0.14	0.23	0.08
tran	NA	NA	n374450	MTCONS_00071977	chr14	66908663	66927208	967	-	chr9	132565427	132575224	8828	+		-820.63	-0.9735	-1	0.04	0.1	0.03	1.15	0.99	1.24
tran	NA	NA	n325293	MTCONS_00025135	chr2	64166988	64168884	563	-	chr15	75647592	75744087	13925	-		-386.13	0.61	0.866	0.58	0.84	0.58	2.36	3.13	3.02
cis_mRNA_overlap	0	mRNA	n406512	MTCONS_00058990	chr5	176946790	176981548	4611	-	chr5	176945424	176981548	5254	-		-3126.6	0.9955	1	4.16	5.27	4.99	0.61	1.2	1
cis_mRNA_up10k	2824	NA	n406512	MTCONS_00058983	chr5	176946790	176981548	4611	-	chr5	176928901	176943967	8478	-	*		0.9995	1	4.16	5.27	4.99	0.45	1.09	0.91
cis_mRNA_overlap	0	mRNA	n406512	MTCONS_00058993	chr5	176946790	176981548	4611	-	chr5	176945424	176981548	5048	-		-3037.9	1	1	4.16	5.27	4.99	0.42	0.62	0.57
cis_mRNA_up10k	1507	NA	n406512	MTCONS_00058984	chr5	176946790	176981548	4611	-	chr5	176928901	176945284	8525	-	*		-0.9614	-1	4.16	5.27	4.99	0.09	0.05	0.07
cis_mRNA_overlap	0	Lnc-CompleteIn-mRNAExon	n405975	MTCONS_00022628	chr14	74751980	74769767	3029	-	chr14	74751789	74769767	8842	-		-1979.78	0.9397	1	1.94	2.31	2.26	2.02	3.53	2.84
cis_mRNA_dw20k	4165	NA	n338522	NM_000704_dup1	chr19	36031640	36036931	1627	-	chr19	36041095	36054560	3559	-	*		0.7936	1	0.74	0.73	0.88	0.02	0	0.03
tran	NA	NA	n338522	MTCONS_00030317	chr19	36031640	36036931	1627	-	chr17	5005757	5017891	5800	-		-1477.57	-0.8609	-1	0.74	0.73	0.88	0.94	1.31	0.65
cis_mRNA_up10k	133	NA	LTCONS_00052267	MTCONS_00049348	chr3	123751154	123752040	887	.	chr3	123752172	124238922	6988	+	*		-0.8504	-0.866	0.92	1.02	1.11	0.01	0.01	0
cis_mRNA_up10k	133	NA	LTCONS_00052267	MTCONS_00049349	chr3	123751154	123752040	887	.	chr3	123752172	124445213	16237	+	*		-0.9559	-1	0.92	1.02	1.11	6.21	4.27	3.77
cis_mRNA_up10k	5627	NA	n410006	MTCONS_00051150	chr3	101237711	101242731	2295	+	chr3	100940797	101232085	22429	-	*		0.8227	0.866	0.03	0.07	0.03	3.86	4.44	3.09
tran	NA	NA	n367711	MTCONS_00026180	chr5	10479483	10482421	483	+	chr16	28829772	28848814	10249	+		-581.23	0.9494	1	0.08	0.5	0.43	0.37	0.61	0.5
cis_mRNA_up10k	6384	NA	n334529	MTCONS_00017800	chr12	56733916	56734193	278	+	chr12	56700351	56727533	10333	-	*		-0.9575	-1	0.14	0	0.03	0	0.88	0.46
cis_mRNA_up10k	6080	NA	n334529	MTCONS_00017806	chr12	56733916	56734193	278	+	chr12	56708258	56727837	6723	-	*		-0.9255	-1	0.14	0	0.03	2.29	3.61	2.87
cis_mRNA_up10k	6080	NA	n334529	MTCONS_00017803	chr12	56733916	56734193	278	+	chr12	56700351	56727837	10052	-	*		0.913	1	0.14	0	0.03	1.29	0.95	1.15

cis_mRNA_up10k	6080	NA	n334529	MTCONS_00017801	chr12	56733916	56734193	278	+	chr12	56700351	56727837	10262	-	*	-0.9008	-1	0.14	0	0.03	1.08	1.96	1.42
cis_mRNA_up10k	7354	NA	n386372	MTCONS_00057417	chr5	1602031	1634120	1188	-	chr5	1574556	1594678	11153	-	*	0.9835	1	2.14	1.59	1.31	1.14	0.84	0.78
cis_mRNA_up10k	7354	NA	n386372	MTCONS_00057415	chr5	1602031	1634120	1188	-	chr5	1571958	1594678	1490	-	*	0.9242	1	2.14	1.59	1.31	1.96	1.74	1.27
tran	124580930	NA	LTCONS_00000543	MTCONS_00002140	chr1	21766628	21792645	552	+	chr1	146373574	146406546	2884	+	-445.49	0.63	1	0.79	0.98	2.3	0.8	1.2	1.22
tran	124580930	NA	LTCONS_00000543	MTCONS_00002142	chr1	21766628	21792645	552	+	chr1	146373574	146411106	2761	+	-445.49	0.9725	1	0.79	0.98	2.3	0	0.3	0.86
tran	11080446	NA	LTCONS_00027651	MTCONS_00027486	chr16	29502549	29506946	682	-	chr16	18411784	18422104	1093	-	-314.02	0.7835	1	3.15	4.32	3.01	1.84	2.57	0
tran	6954708	NA	LTCONS_00027651	MTCONS_00026095	chr16	29502549	29506946	682	-	chr16	22445951	22547842	9825	+	-344.85	0.9998	1	3.15	4.32	3.01	0.05	0.59	0
tran	14293854	NA	LTCONS_00027651	MTCONS_00027452	chr16	29502549	29506946	682	-	chr16	15198163	15208696	2656	-	-340.4	0.9964	1	3.15	4.32	3.01	3.1	3.94	2.9
tran	11079629	NA	LTCONS_00027651	MTCONS_00027487	chr16	29502549	29506946	682	-	chr16	18411784	18422921	1802	-	-311.46	0.9588	1	3.15	4.32	3.01	2.84	3.33	2.54
tran	13061181	NA	LTCONS_00027651	MTCONS_00026003	chr16	29502549	29506946	682	-	chr16	16402869	16441369	9463	+	-403.65	-0.6592	-1	3.15	4.32	3.01	0.04	0	0.37
tran	6954708	NA	LTCONS_00027651	MTCONS_00026100	chr16	29502549	29506946	682	-	chr16	22452709	22547842	10437	+	-353.17	0.8928	1	3.15	4.32	3.01	1.7	1.96	1.4
tran	NA	NA	n334773	MTCONS_00009585	chr5	138609821	138615278	678	+	chr10	13672872	13933777	19431	-	-474.29	0.9799	1	0.84	1.17	0.15	0.11	0.14	0.08
tran	NA	NA	n334773	MTCONS_00009583	chr5	138609821	138615278	678	+	chr10	13672872	13752203	21653	-	-614.46	-0.9936	-1	0.84	1.17	0.15	0.51	0.44	0.76
cis_mRNA_overlap	0	Lnc-CompleteIn-mRNAIntron	n375056	MTCONS_00026939	chr16	85316564	85319569	1566	+	chr16	85203298	85710002	9937	+	*	-0.8626	-0.866	0.16	0.09	0.16	1.85	2.27	1.42
tran	48712184	NA	n323965	MTCONS_00071212	chr9	131060454	131061946	966	+	chr9	82186868	82348271	11300	+	-733.73	-0.7823	-1	0.92	0.9	1.07	0.76	0.92	0.7
cis_mRNA_dw20k	262	NA	LTCONS_00030561	NM_000676_dup1	chr17	15879471	15903081	1548	-	chr17	15848231	15879210	1885	+	*	0.9994	0.866	1.45	0.94	0.96	0.09	0.05	0.05
tran	NA	NA	n326710	MTCONS_00058855	chr1	8200837	8203176	477	-	chr5	168081512	168272122	16496	-	-513.61	0.7884	1	1.49	1.86	1.47	5.1	5.78	3.77
tran	NA	NA	n326710	MTCONS_00058857	chr1	8200837	8203176	477	-	chr5	168081512	168728133	28723	-	-513.61	0.83	1	1.49	1.86	1.47	8.82	9.47	7.85
cis_mRNA_overlap	0	Lnc-CompleteIn-mRNAIntron	n405912	MTCONS_00034863	chr19	53935227	53947925	2932	+	chr19	53898346	53962049	4462	+	*	-0.6453	-1	4.16	4.35	6.12	2.13	1.31	1.22
cis_mRNA_overlap	0	mRNA	n405912	MTCONS_00034869	chr19	53935227	53947925	2932	+	chr19	53935227	53962049	4648	+	*	-0.9939	-1	4.16	4.35	6.12	0.67	0.62	0.43
cis_mRNA_overlap	0	mRNA	n372355	MTCONS_00009573	chr10	13565924	13570545	281	-	chr10	13482875	13570914	4186	-	-168.43	0.9843	0.866	0.16	0.13	0	0.84	0.84	0.48
cis_mRNA_dw20k	12514	NA	n338246	MTCONS_00006147	chr1	153895879	153907635	1120	+	chr1	153920148	153936035	9385	-	*	0.9798	1	0.22	0.31	0.36	1.16	1.75	1.87
cis_mRNA_dw20k	12514	NA	n338246	MTCONS_00006146	chr1	153895879	153907635	1120	+	chr1	153920148	153936035	5191	-	*	0.9996	1	0.22	0.31	0.36	0.93	1.19	1.35
tran	32638	NA	LTCONS_00003941	MTCONS_00003951	chr1	1592930	1626095	7616	-	chr1	1658732	1677500	7140	-	-4120.5	0.9089	1	0.97	1.42	1.04	5.61	6.84	6.28
cis_mRNA_overlap	0	mRNA	LTCONS_00003941	MTCONS_00003942	chr1	1592930	1626095	7616	-	chr1	1592930	1677500	7291	-	-4335.65	0.9955	1	0.97	1.42	1.04	5.98	11.09	6.27
tran	32638	NA	LTCONS_00003941	MTCONS_00003952	chr1	1592930	1626095	7616	-	chr1	1658732	1678937	11788	-	-5236.78	0.7866	1	0.97	1.42	1.04	0.23	0.68	0.57
cis_mRNA_dw20k	18269	NA	n406566	MTCONS_00024457	chr15	23255242	23262743	1899	+	chr15	23281011	23378337	7754	-	*	-0.9469	-0.866	0.29	0.12	0.12	0	1.64	2.44
cis_mRNA_dw20k	18269	NA	n406566	MTCONS_00024455	chr15	23255242	23262743	1899	+	chr15	23281011	23378259	7914	-	*	-0.9511	-0.866	0.29	0.12	0.12	0	4.18	2.86
cis_mRNA_dw20k	18269	NA	n406566	MTCONS_00024454	chr15	23255242	23262743	1899	+	chr15	23281011	23378259	8982	-	*	-0.9999	-0.866	0.29	0.12	0.12	0.35	4.57	4.5
cis_mRNA_overlap	0	Lnc-CompleteIn-mRNAIntron	n340074	MTCONS_00054390	chr4	66533081	66533967	887	-	chr4	66196389	66535746	4197	-	*	0.8567	1	0.36	0.29	0.14	1.78	0.73	0.47
cis_mRNA_overlap	0	Lnc-CompleteIn-mRNAIntron	n340074	MTCONS_00054389	chr4	66533081	66533967	887	-	chr4	66196389	66535746	4533	-	*	-0.8154	-1	0.36	0.29	0.14	0.09	0.66	0.74
cis_mRNA_overlap	0	Lnc-CompleteIn-mRNAIntron	n340074	MTCONS_00054386	chr4	66533081	66533967	887	-	chr4	66185032	66535746	8137	-	*	0.8739	1	0.36	0.29	0.14	1.08	0.25	0
tran	8037486	NA	n405547	MTCONS_00027659	chr16	21529230	21531765	2536	-	chr16	29569250	29625058	2159	-	-1324.39	0.9943	1	3.49	4.14	3.72	0.67	1.06	0.77
cis_mRNA_overlap	0	Lnc-AntiOverlap-mRNA	n382933	MTCONS_00026095	chr16	22430867	22449036	2492	-	chr16	22445951	22547842	9825	+	*	-0.9356	-1	0.56	0.33	0.73	0.05	0.59	0
cis_mRNA_overlap	0	Lnc-AntiOverlap-mRNA	n382933	MTCONS_00026094	chr16	22430867	22449036	2492	-	chr16	22445951	22518194	21000	+	*	-0.9784	-1	0.56	0.33	0.73	3.82	4.56	3.59
cis_mRNA_up10k	3674	NA	n382933	MTCONS_00026100	chr16	22430867	22449036	2492	-	chr16	22452709	22547842	10437	+	*	-0.9919	-1	0.56	0.33	0.73	1.7	1.96	1.4
cis_mRNA_overlap	0	Lnc-AntiOverlap-mRNA	n382933	MTCONS_00026096	chr16	22430867	22449036	2492	-	chr16	22445951	22553889	11744	+	*	-0.9811	-1	0.56	0.33	0.73	0.06	0.09	0.05
cis_mRNA_overlap	0	AntiCompleteIn-mRNAIntron	n336220	MTCONS_00070523	chr8	144997132	144998828	1697	+	chr8	144989185	145060635	15516	-	*	0.9964	1	0.31	0.13	0.22	1.75	0.93	1.4
cis_mRNA_overlap	0	AntiCompleteIn-mRNAExon	n336220	MTCONS_00070520	chr8	144997132	144998828	1697	+	chr8	144989185	145028088	14926	-	-4350.1	0.9827	1	0.31	0.13	0.22	4.81	2.08	3.89
cis_mRNA_overlap	0	AntiCompleteIn-mRNAExon	n336220	MTCONS_00070519	chr8	144997132	144998828	1697	+	chr8	144989185	145028088	14938	-	-4350.1	-0.8807	-1	0.31	0.13	0.22	0.94	3.27	3.19
cis_mRNA_overlap	0	AntiCompleteIn-mRNAIntron	n336220	MTCONS_00070522	chr8	144997132	144998828	1697	+	chr8	144989185	145060635	15528	-	*	-0.9615	-1	0.31	0.13	0.22	0.36	1.43	1.16
cis_mRNA_overlap	0	AntiCompleteIn-mRNAExon	n336220	MTCONS_00070521	chr8	144997132	144998828	1697	+	chr8	144989185	145028195	15064	-	-4350.1	-0.926	-1	0.31	0.13	0.22	2.5	4.2	2.75
cis_mRNA_dw20k	19684	NA	n339663	MTCONS_00043350	chr20	34840482	34842636	2153	+	chr20	34742580	34820799	6433	+	*	-0.9986	-1	0.24	0.44	0.36	10.22	9.66	9.91
cis_mRNA_overlap	0	Lnc-AntiOverlap-mRNA	n407758	MTCONS_00052757	chr4	40044537	40058819	1613	-	chr4	40057780	40169531	20063	+	*	0.9226	1	0.02	0.12	0.08	0.34	0.46	0.37
cis_mRNA_up10k	6409	NA	n371246	MTCONS_00072168	chr9	139736279	139736848	472	+	chr9	139743256	139756563	7103	+	*	-0.9757	-1	0.8	1.52	0.36	0.11	0.08	0.15
cis_mRNA_dw20k	627	NA	n371246	MTCONS_00072155	chr9	139736279	139736848	472	+	chr9	139690790	139735653	5389	+	*	-0.9001	-1	0.8	1.52	0.36	0.63	0.5	1.09
cis_mRNA_dw20k	627	NA	n371246	MTCONS_00072157	chr9	139736279	139736848	472	+	chr9	139690790	139735653	5811	+	*	-0.9832	-1	0.8	1.52	0.36	1.52	0.96	1.67
cis_mRNA_overlap	0	Lnc-CompleteIn-mRNAIntron	n324304	MTCONS_00061510	chr6	168337887	168348030	449	+	chr6	168227585	168372728	7816	+	*	0.9898	1	0.42	0.15	0.08	5.38	3.81	3.71
cis_mRNA_up10k	79	NA	n341602	MTCONS_00068985	chr8	126036509	126104005	4103	-	chr8	126104083	126379743	1492	+	*	0.845	1	6.46	5.83	5.71	2.57	2.43	2.15
cis_mRNA_up10k	79	NA	n341602	MTCONS_00068986	chr8	126036509	126104005	4103	-	chr8	126104083	126379764	1262	+	*	-0.9416	-1	6.46	5.83	5.71	2.67	3.66	4.55
cis_mRNA_overlap	0	CompleteIn-LncExon	n410097	MTCONS_00071442	chr9	100000708	100139577	6930	+	chr9	100000708	100139577	6379	+	-3418.94	-0.8851	-1	1.41	1.31	0.7	0.25	0.58	0.82
cis_mRNA_overlap	0	mRNA	n410097	MTCONS_00071439	chr9	100000708	100139577	6930	+	chr9	100000708	100059596	3098	+	-1817.27	-0.9632	-1	1.41	1.31	0.7	0.41	0.56	0.79
tran	NA	NA	LTCONS_00032053	MTCONS_00031250	chr17_ctg5_hap1	1322427	1425920	6311	+	chr17	44107264	44270272	5284	-	-2858.69	-0.8272	-1	4.07	3.85	1.25	0	0.45	0.72
tran	NA	NA	LTCONS_00032053	MTCONS_00031258	chr17_ctg5_hap1	1322427	1425920	6311	+	chr17	44228558	44302867	3448	-	-1816.03	0.9882	1	4.07	3.85	1.25	3.61	2.98	0.9

tran	NA	NA	n324281	MTCONS_00037130	chr6	170846326	170847963	806	+	chr1_gl000191_random	31266	50281	11132	-		-397.74	-0.9834	-1	0.18	0.24	0.12	0.16	0	0.47
tran	NA	NA	n324281	MTCONS_00037133	chr6	170846326	170847963	806	+	chr1_gl000191_random	31266	50281	13961	-		-420.87	0.9015	1	0.18	0.24	0.12	3.12	3.19	2.36
cis_mRNA_dw20k	9219	NA	n339546	MTCONS_00045653	chr21	47874900	47878593	1529	-	chr21	47846027	47865682	4642	+	*		0.8171	1	0.18	0.5	0.26	1.22	1.67	1.58
cis_mRNA_overlap	0	mRNA	n375738	MTCONS_00031440	chr17	56066423	56067684	883	-	chr17	56066398	56084707	11419	-		-501.76	0.9185	0.866	1.13	0.68	0	1.19	1.19	0.56
cis_mRNA_overlap	0	Lnc-CompleteIn-mRNAIntron	n340021	NM_000698_dup1	chr10	45931684	45934607	2924	+	chr10	45869629	45941565	2554	+	*		0.8358	0.866	0.26	0.46	0.37	0.07	0.16	0.07
cis_mRNA_overlap	0	mRNA	n386099	MTCONS_00023318	chr15	28878269	28887567	1559	+	chr15	28834608	28930558	5759	+		-917.05	-0.7982	-1	1.56	0.86	1.83	1.34	1.42	0.84
cis_mRNA_overlap	0	mRNA	n386099	MTCONS_00023317	chr15	28878269	28887567	1559	+	chr15	28834608	28930558	6711	+		-916.19	-0.996	-1	1.56	0.86	1.83	0.91	1.41	0.63
cis_mRNA_overlap	0	Lnc-AntiOverlap-mRNA	LTCONS_00027583	MTCONS_00026095	chr16	22430867	22449036	3958	-	chr16	22445951	22547842	9825	+	*		0.712	1	0.8	1	0.01	0.05	0.59	0
cis_mRNA_overlap	0	Lnc-AntiOverlap-mRNA	LTCONS_00027583	MTCONS_00026094	chr16	22430867	22449036	3958	-	chr16	22445951	22518194	21000	+	*		0.8103	1	0.8	1	0.01	3.82	4.56	3.59
cis_mRNA_up10k	3674	NA	LTCONS_00027583	MTCONS_00026100	chr16	22430867	22449036	3958	-	chr16	22452709	22547842	10437	+	*		0.9582	1	0.8	1	0.01	1.7	1.96	1.4
cis_mRNA_overlap	0	Lnc-AntiOverlap-mRNA	LTCONS_00027583	MTCONS_00026096	chr16	22430867	22449036	3958	-	chr16	22445951	22553889	11744	+	*		0.8183	1	0.8	1	0.01	0.06	0.09	0.05
cis_mRNA_overlap	0	mRNA	LTCONS_00027845	MTCONS_00027838	chr16	53726539	53737787	3834	-	chr16	53632320	53737771	7692	-	*		1	1	2.43	2.77	2.71	0	1.18	0.97
cis_mRNA_overlap	0	mRNA	LTCONS_00027845	MTCONS_00027840	chr16	53726539	53737787	3834	-	chr16	53632320	53737780	7599	-	*		-0.9724	-1	2.43	2.77	2.71	0.33	0	0.13
cis_mRNA_overlap	0	CompleteIn-LncExon	LTCONS_00027845	MTCONS_00027844	chr16	53726539	53737787	3834	-	chr16	53726539	53737780	3135	-		-1806.12	-0.966	-1	2.43	2.77	2.71	1.48	0.16	0.71
cis_mRNA_overlap	0	AntiCompleteIn-mRNAIntron	n407093	MTCONS_00044730	chr20	45947246	45949498	2253	+	chr20	45837599	45985925	6010	-	*		0.9988	1	0.05	0.02	0.03	2.72	1.02	1.51
cis_mRNA_overlap	0	AntiCompleteIn-mRNAIntron	n407093	MTCONS_00044731	chr20	45947246	45949498	2253	+	chr20	45837599	45985927	5866	-	*		-0.981	-1	0.05	0.02	0.03	2.87	5.03	3.94
tran	NA	NA	n4806	MTCONS_00033625	chr2	47562454	47562653	200	+	chr19	10764937	10803102	6693	+		-153.59	0.893	1	6.17	4.14	7.7	3.02	2.82	4.17
tran	1439942	NA	LTCONS_00027481	MTCONS_00025979	chr16	16485934	16487821	1762	-	chr16	15019851	15045993	5075	+		-1735.25	0.731	1	0.16	0.21	0.45	2.75	4.76	5.1
tran	41470	NA	LTCONS_00027481	MTCONS_00026007	chr16	16485934	16487821	1762	-	chr16	16403022	16444465	12506	+		-1795.56	0.9059	1	0.16	0.21	0.45	1.84	2.4	2.83
tran	NA	NA	n340408	MTCONS_00049348	chr5	14144544	14419987	4998	+	chr3	123752172	124238922	6988	+		-2883.92	-0.6443	-0.866	14.04	15.87	16.29	0.01	0.01	0
tran	NA	NA	n340408	MTCONS_00049349	chr5	14144544	14419987	4998	+	chr3	123752172	124445213	16237	+		-2891.32	-0.9998	-1	14.04	15.87	16.29	6.21	4.27	3.77
cis_mRNA_overlap	0	Lnc-AntiOverlap-mRNA	LTCONS_00027202	MTCONS_00025724	chr16	1826236	1832854	6348	-	chr16	1823145	1826845	1606	+	*		-0.8695	-1	0.3	0.28	0.4	1.36	1.82	1.09
cis_mRNA_overlap	0	Lnc-AntiOverlap-mRNA	LTCONS_00027202	MTCONS_00025723	chr16	1826236	1832854	6348	-	chr16	1823145	1826709	3212	+	*		0.9944	1	0.3	0.28	0.4	2.74	2.48	3.45
cis_mRNA_overlap	0	Lnc-AntiOverlap-mRNA	LTCONS_00027202	MTCONS_00025728	chr16	1826236	1832854	6348	-	chr16	1823169	1826709	2770	+	*		-0.7076	-1	0.3	0.28	0.4	1.31	2.3	1.18
cis_mRNA_overlap	0	Lnc-AntiOverlap-mRNA	LTCONS_00027202	MTCONS_00025729	chr16	1826236	1832854	6348	-	chr16	1823188	1826845	1799	+	*		-0.6375	-1	0.3	0.28	0.4	0.01	0.76	0
cis_mRNA_overlap	0	Lnc-AntiOverlap-mRNA	LTCONS_00027202	MTCONS_00025726	chr16	1826236	1832854	6348	-	chr16	1823145	1828554	1842	+	*		0.9703	1	0.3	0.28	0.4	1.81	1.37	2.49
cis_mRNA_up10k	7728	NA	n346123	MTCONS_00072114	chr9	138354460	138364246	5586	-	chr9	138371973	138380741	4852	+	*		-0.9586	-1	0.19	0.13	0.26	1.32	2.53	0.84
cis_mRNA_overlap	0	Lnc-AntiOverlap-mRNA	n338342	MTCONS_00035736	chr19	15489887	15491657	1771	+	chr19	15462350	15529852	8576	-		-2724.98	0.834	1	0.02	0.05	0.12	0.39	0.53	0.56
cis_mRNA_dw20k	2901	NA	n405952	MTCONS_00027086	chr16	90038988	90063028	2929	+	chr16	90015151	90036088	6984	+	*		-0.9959	-1	0.92	0.5	0.8	0.85	1.34	1.03
cis_mRNA_overlap	0	mRNA	n335595	NM_005169_dup1	chr11	71950462	71951269	808	-	chr11	71950121	71955220	1699	-	*		0.9644	1	0	0.34	0.67	16.46	18.08	22.45
cis_mRNA_overlap	0	Lnc-CompleteIn-mRNAExon	n407986	MTCONS_00027057	chr16	89753076	89762772	1725	+	chr16	89753016	89762800	3021	+		-1405.09	-0.9804	-1	1.4	0.2	2.42	0.91	3.21	0
cis_mRNA_overlap	0	mRNA	n407986	MTCONS_00027055	chr16	89753076	89762772	1725	+	chr16	89753016	89762800	3125	+		-1421.27	0.9541	1	1.4	0.2	2.42	1.64	0.88	1.81
cis_mRNA_up10k	117	NA	LTCONS_00070187	MTCONS_00068881	chr8	104493465	104511823	3852	-	chr8	104511939	105267094	8752	+	*		-0.9824	-1	1.39	2.16	3.88	2.72	2.34	1.92
cis_mRNA_up10k	9	NA	LTCONS_00050267	MTCONS_00048200	chr3	9427509	9439342	2893	-	chr3	9439350	9519901	7913	+	*		0.985	1	1.03	0.59	0.42	1.78	0.32	0.14
cis_mRNA_dw20k	9113	NA	n384095	MTCONS_00046468	chr22	21362504	21368576	2095	-	chr22	21336558	21353392	4669	+	*		0.9193	1	0.61	2.03	1.08	8.2	10.24	9.6
cis_mRNA_overlap	0	Lnc-AntiOverlap-mRNA	n345533	MTCONS_00038172	chr2	74211337	74252962	2984	-	chr2	74212780	74335391	10093	+	*		0.8955	1	0.1	0.09	0.07	1.71	1.51	1.44
cis_mRNA_overlap	0	mRNA	LTCONS_00002193	MTCONS_00002194	chr1	148555673	148575210	7680	+	chr1	148556011	148596267	5017	+	*		-0.9986	-1	4	4.94	4.7	4.57	4.23	4.3
cis_mRNA_overlap	0	mRNA	LTCONS_00002193	MTCONS_00002190	chr1	148555673	148575210	7680	+	chr1	148555358	148596267	5492	+	*		0.6975	0.866	4	4.94	4.7	0.27	0.36	0.27
cis_mRNA_dw20k	6504	NA	n410151	MTCONS_00055015	chr4	150999426	151178608	4344	+	chr4	151185111	151936861	10301	-	*		-0.9984	-1	0.77	1.53	2.2	1.72	1.18	0.79
cis_mRNA_dw20k	6504	NA	n410151	MTCONS_00055016	chr4	150999426	151178608	4344	+	chr4	151185111	151936861	10587	-	*		0.9905	1	0.77	1.53	2.2	0.8	1.15	1.65
tran	908010	NA	n334856	MTCONS_00039126	chr2	167159708	167163041	345	-	chr2	166095912	166251699	11673	+		-188.12	-0.9428	-1	0	0.21	0.1	3.11	2.45	3
cis_mRNA_overlap	0	Lnc-CompleteIn-mRNAExon	n375739	MTCONS_00031440	chr17	56066423	56070937	443	-	chr17	56066398	56084707	11419	-		-283.02	-0.797	-0.866	0.13	0.27	0.36	1.19	1.19	0.56
tran	NA	NA	n373751	MTCONS_00033625	chr13	45943762	45957973	790	+	chr19	10764937	10803102	6693	+		-794.57	0.6742	1	0.44	0.23	0.46	3.02	2.82	4.17
tran	NA	NA	n373751	MTCONS_00033622	chr13	45943762	45957973	790	+	chr19	10764937	10796526	4284	+		-800.04	0.7772	1	0.44	0.23	0.46	2.24	1.34	4.42
cis_mRNA_overlap	0	Lnc-AntiOverlap-mRNA	LTCONS_00016739	MTCONS_00018662	chr12	125396431	125398212	642	+	chr12	125396186	125399587	994	-		-1006.16	0.9999	1	0.11	0.21	0.15	8.92	11.43	9.96
cis_mRNA_overlap	0	Lnc-AntiOverlap-mRNA	LTCONS_00016739	MTCONS_00018659	chr12	125396431	125398212	642	+	chr12	125396186	125399587	1222	-		-1190.61	-0.9493	-1	0.11	0.21	0.15	37.89	26.14	36.74
cis_mRNA_overlap	0	Lnc-AntiOverlap-mRNA	LTCONS_00016739	MTCONS_00018657	chr12	125396431	125398212	642	+	chr12	125396186	125399587	1906	-		-1367.9	0.8737	1	0.11	0.21	0.15	9.5	10.21	10.11
cis_mRNA_up10k	503	NA	n366855	MTCONS_00054391	chr4	66536248	66559104	422	+	chr4	66461388	66535746	7167	-	*		-0.7735	-1	0.47	0.21	0.59	0.95	0.96	0.77
cis_mRNA_overlap	0	Lnc-CompleteIn-mRNAIntron	n405691	NM_001110_dup1	chr15	58983302	58985324	1518	-	chr15	58888510	59042177	3927	-	*		0.9378	1	0.09	0.15	0.06	19.9	21.14	17.66

cis_mRNA_overlap	0	mRNA	n383172	MTCONS_00030911	chr17	36364603	36421858	1281	-	chr17	36345066	36421858	6939	-		-734.39	0.9725	1	0.26	0.46	0.55	2.16	2.5	2.53
tran	NA	NA	n409649	MTCONS_00037129	chr1	24292937	24306953	2373	-	chr1_gl000191_random	31266	50281	14340	-		-1075.72	0.8975	1	2.5	1.45	1.3	0.24	0.13	0
cis_mRNA_overlap	0	Lnc-CompleteIn-mRNAExon	n381279	MTCONS_00006573	chr1	168048781	168049929	1149	-	chr1	168047875	168106218	8901	-		-670.81	0.9962	1	0.31	0.77	1.12	0	0.29	0.45
cis_mRNA_overlap	0	Lnc-CompleteIn-mRNAExon	n381279	MTCONS_00006575	chr1	168048781	168049929	1149	-	chr1	168047875	168106991	8892	-		-670.81	-0.9664	-1	0.31	0.77	1.12	1.04	0.89	0.6
cis_mRNA_overlap	0	Lnc-AntiOverlap-mRNA	n366746	MTCONS_00054391	chr4	66535679	66559103	1176	+	chr4	66461388	66535746	7167	-	*		0.7712	1	0.38	0.6	0.28	0.95	0.96	0.77
tran	640166	NA	LTCONS_00072758	MTCONS_00071098	chr9	70195031	70216674	481	-	chr9	70856839	70890198	1245	+		-221.29	0.7334	1	0.87	2.04	0.72	6.27	6.65	4.8
cis_mRNA_up10k	1740	NA	n386230	MTCONS_00037071	chr19	59086766	59110850	1036	+	chr19	59080367	59085027	3036	-	*		0.8171	1	4.23	5.58	7.64	0.29	0.88	0.9
cis_mRNA_up10k	3543	NA	n386230	MTCONS_00037066	chr19	59086766	59110850	1036	+	chr19	59070842	59083224	5736	-	*		0.9975	1	4.23	5.58	7.64	0.09	0.1	0.12
cis_mRNA_overlap	0	Lnc-CompleteIn-mRNAExon	n375477	MTCONS_00031441	chr17	56066403	56070840	4056	-	chr17	56066398	56084707	16943	-		-1982.06	0.6131	0.866	0.79	0.79	0	0.41	2.27	0.09
cis_mRNA_overlap	0	Lnc-CompleteIn-mRNAExon	n375477	MTCONS_00031437	chr17	56066403	56070840	4056	-	chr17	56066398	56084707	16946	-		-1982.78	-0.9753	-0.866	0.79	0.79	0	0.51	0.01	2.17
cis_mRNA_overlap	0	mRNA	n375477	MTCONS_00031440	chr17	56066403	56070840	4056	-	chr17	56066398	56084707	11419	-		-1930.31	1	1	0.79	0.79	0	1.19	1.19	0.56
tran	7860265	NA	n384929	MTCONS_00059972	chr6	36125846	36163947	1098	-	chr6	28234788	28265582	10295	+		-513.88	-0.9387	-0.866	0.17	0.11	0.17	1.65	2.25	1.86
tran	7839484	NA	n384929	MTCONS_00059974	chr6	36125846	36163947	1098	-	chr6	28234788	28286363	24577	+		-651.04	-0.9897	-0.866	0.17	0.11	0.17	0.06	0.39	0
tran	7839484	NA	n384929	MTCONS_00059973	chr6	36125846	36163947	1098	-	chr6	28234788	28286363	24413	+		-651.04	0.9872	0.866	0.17	0.11	0.17	1.46	1.05	1.39
cis_mRNA_overlap	0	AntiCompleteIn-mRNAIntron	n379502	MTCONS_00029757	chr17	65871895	65881544	4429	-	chr17	65821780	65982197	13331	+	*		-0.9488	-1	0.05	0.03	0.04	4.49	5.53	4.71
cis_mRNA_up10k	4	NA	n338301	MTCONS_00006423	chr1	160231527	160234228	2555	+	chr1	160185476	160231524	4176	-	*		-0.687	-1	0.19	0.37	0.15	1.91	1.73	4.32
cis_mRNA_overlap	0	Lnc-AntiOverlap-mRNA	n338301	MTCONS_00006426	chr1	160231527	160234228	2555	+	chr1	160185476	160232379	7881	-	*		-0.9906	-1	0.19	0.37	0.15	3.48	1.5	3.56
cis_mRNA_up10k	4	NA	n338301	MTCONS_00006424	chr1	160231527	160234228	2555	+	chr1	160185476	160231524	7858	-	*		0.999	1	0.19	0.37	0.15	2.26	5.31	1.78
cis_mRNA_dw20k	11671	NA	n379362	MTCONS_00026189	chr16	28897204	28936455	1175	+	chr16	28857855	28885534	5438	+	*		0.9859	1	0.29	0.66	0.43	0.71	1.11	0.8
tran	NA	NA	n406266	MTCONS_00002203	chr5	85578262	85593362	2633	+	chr1	148739442	148758311	3223	+		-1246.08	-0.9989	-1	0.03	0.01	0	0	0.17	0.24
tran	NA	NA	n406266	MTCONS_00002195	chr5	85578262	85593362	2633	+	chr1	148556011	148758311	4653	+		-1357.54	0.9955	1	0.03	0.01	0	2.02	1.53	1.37
tran	NA	NA	n334145	MTCONS_00071858	chr6	3966863	3967145	283	-	chr9	130965633	130989314	5488	+		-201.37	0.998	1	0.4	1.2	0.65	3.63	5.85	4.45
cis_mRNA_overlap	0	Lnc-AntiOverlap-mRNA	n378606	MTCONS_00045937	chr21	34100450	34115433	3523	+	chr21	34105977	34144169	8461	-	*		0.9241	1	1.04	0.62	0.29	1.95	1.54	0
tran	NA	NA	n325428	MTCONS_00030316	chr2	12371894	12618842	578	+	chr17	5005757	5016033	8346	-		-529.57	-0.9563	-0.866	0.06	0	0.06	2.38	3.18	2.62
tran	NA	NA	n325428	MTCONS_00030317	chr2	12371894	12618842	578	+	chr17	5005757	5017891	5800	-		-511.64	-0.8988	-0.866	0.06	0	0.06	0.94	1.31	0.65
cis_mRNA_dw20k	4455	NA	n326378	MTCONS_00002201	chr1	148754857	148757132	566	+	chr1	148737657	148750403	3372	+	*		-0.9694	-1	0.45	0.24	0.53	2.92	4.44	2.88
cis_mRNA_up10k	4394	NA	n384582	MTCONS_00057414	chr5	1599039	1634120	2228	-	chr5	1571958	1594646	6957	-	*		0.9566	1	0.3	0.46	0.36	0.71	2.32	0.86
tran	86074830	NA	n326743	MTCONS_00005308	chr1	730175	735303	581	+	chr1	86810132	86862025	9802	-		-697.03	1	1	2.33	2.5	2.73	1.4	1.65	1.98
cis_mRNA_overlap	0	Lnc-AntiOverlap-mRNA	LTCONS_00045262	MTCONS_00045937	chr21	34100420	34106208	5026	+	chr21	34105977	34144169	8461	-	*		-0.9901	-1	0.9	1.06	1.37	1.95	1.54	0
tran	25648259	NA	n335022	MTCONS_00059973	chr6	2585741	2586530	543	-	chr6	28234788	28286363	24413	+		-682.73	-0.9872	-1	0	0.28	0.09	1.46	1.05	1.39
tran	NA	NA	n365460	MTCONS_00053417	chr2	64503035	64503985	704	-	chr4	128930387	128960982	5347	+		-558.64	0.9999	1	0	0.2	0.09	0.1	0.64	0.35
tran	NA	NA	n365460	MTCONS_00053413	chr2	64503035	64503985	704	-	chr4	128886414	128960982	5837	+		-558.64	-0.8658	-1	0	0.2	0.09	1.17	0.39	0.44
tran	NA	NA	n365460	MTCONS_00053415	chr2	64503035	64503985	704	-	chr4	128886446	128960982	5850	+		-558.64	0.999	1	0	0.2	0.09	0.72	1.19	0.95
cis_mRNA_overlap	0	Lnc-CompleteIn-mRNAIntron	n342085	MTCONS_00060714	chr6	90550659	90551267	609	+	chr6	90539619	90581374	6394	+	*		0.6934	0.866	0.12	0.11	0.11	0.89	0.81	0.57
tran	NA	NA	n326645	MTCONS_00024457	chr1	35088882	35089743	720	+	chr15	23281011	23378337	7754	-		-572.96	-0.9469	-0.866	0.18	0.14	0.14	0	1.64	2.44
tran	NA	NA	n326645	MTCONS_00024455	chr1	35088882	35089743	720	+	chr15	23281011	23378259	7914	-		-572.96	-0.9511	-0.866	0.18	0.14	0.14	0	4.18	2.86
cis_mRNA_overlap	0	Lnc-CompleteIn-mRNAIntron	n340709	MTCONS_00058811	chr5	158398049	158398508	459	-	chr5	158122910	158527345	5870	-	*		0.9443	1	0.58	1.27	1.47	4.98	5.18	5.4
cis_mRNA_overlap	0	AntiCompleteIn-mRNAIntron	n342698	MTCONS_00056752	chr5	140227822	140229558	1736	-	chr5	140170812	140391929	14785	+		-2560.52	-0.972	-1	1.01	0.81	1.12	0.97	1.17	0.94
cis_mRNA_overlap	0	AntiCompleteIn-mRNAIntron	n342698	MTCONS_00056753	chr5	140227822	140229558	1736	-	chr5	140170812	140391929	15222	+		-2557.92	-0.9595	-1	1.01	0.81	1.12	0.16	0.39	0.14
cis_mRNA_dw20k	13079	NA	LTCONS_00027090	MTCONS_00027081	chr16	90038988	90052096	1823	+	chr16	90015026	90025910	1068	+	*		0.8598	1	0.62	0.6	0.65	2.72	1.85	2.83
cis_mRNA_up10k	219	NA	n375822	MTCONS_00031942	chr17	79995797	79997235	527	+	chr17	79993720	79995579	870	-	*		0.992	1	0.07	0.13	0	0.18	0.22	0.15
cis_mRNA_overlap	0	Lnc-CompleteIn-mRNAIntron	n339595	MTCONS_00044274	chr20	20389471	20390912	1442	-	chr20	20370068	20693266	9632	-	*		-0.86	-1	0.07	0.15	0.21	1.94	1.85	0.79
cis_mRNA_overlap	0	Lnc-CompleteIn-mRNAIntron	n339595	MTCONS_00044276	chr20	20389471	20390912	1442	-	chr20	20370068	20693266	9771	-	*		0.8246	1	0.07	0.15	0.21	0.45	0.46	2.32
cis_mRNA_overlap	0	Lnc-CompleteIn-mRNAIntron	n339595	MTCONS_00044275	chr20	20389471	20390912	1442	-	chr20	20370068	20693266	10173	-	*		0.9527	1	0.07	0.15	0.21	0	0.54	0.63
cis_mRNA_dw20k	6981	NA	n410080	MTCONS_00030104	chr17	80407591	80408707	672	-	chr17	80376219	80400611	2236	+	*		0.9893	1	4.21	5.26	4.48	1.89	2.86	2
cis_mRNA_dw20k	6981	NA	n410080	MTCONS_00030100	chr17	80407591	80408707	672	-	chr17	80375909	80400611	4570	+	*		0.8276	1	4.21	5.26	4.48	0.13	0.32	0.28
cis_mRNA_dw20k	6981	NA	n410080	MTCONS_00030105	chr17	80407591	80408707	672	-	chr17	80376245	80400611	3607	+	*		0.9875	1	4.21	5.26	4.48	0.34	0.39	0.36
cis_mRNA_overlap	0	mRNA	LTCONS_00002192	MTCONS_00002194	chr1	148555673	148566637	2241	+	chr1	148556011	148596267	5017	+	*		-0.9924	-1	3.47	5.06	4.55	4.57	4.23	4.3
cis_mRNA_overlap	0	mRNA	LTCONS_00002192	MTCONS_00002190	chr1	148555673	148566637	2241	+	chr1	148555358	148596267	5492	+		-1384.82	0.7467	0.866	3.47	5.06	4.55	0.27	0.36	0.27
tran	NA	NA	LTCONS_00042936	MTCONS_00003951	chr20	3388848	3390736	1889	+	chr1	1658732	1677500	7140	-		-1333.41	0.8643	1	1	1.37	1.02	5.61	6.84	6.28
tran	NA	NA	LTCONS_00042936	MTCONS_00003952	chr20	3388848	3390736	1889	+	chr1	1658732	1678937	11788	-		-1333.71	0.7231	1	1	1.37	1.02	0.23	0.68	0.57
tran	NA	NA	LTCONS_00042936	MTCONS_00000237	chr20	3388848	3390736	1889	+	chr1	6640063	6649340	5957	+		-1251.64	0.725	1	1	1.37	1.02	0.55	2.21	1.8
cis_mRNA_up10k	59	NA	n373563	MTCONS_00020447	chr13	79980444	79998466	2469	+	chr13	79889027	79980386	4748	-	*		0.9591	1	0.06	0.05	0.34	2.82	2.45	3.61
cis_mRNA_up10k	460	NA	n373563	MTCONS_00020445	chr13	79980444	79998466	2469	+	chr13	79889027	79979985	3112	-	*		0.9949	1	0.06	0.05	0.34	5	4.87	5.79
cis_mRNA_up10k	59	NA	n373563	MTCONS_00020446	chr13	79980444	79998466	2469	+	chr13	79889027	79980386	5403	-	*		-0.9667	-1	0.06	0.05	0.34	3.29	3.46	2.88

cis_mRNA_up10k	59	NA	n373563	MTCONS_00020448	chr13	79980444	79998466	2469	+	chr13	79889027	79980386	3504	-	*		-0.6773	-1	0.06	0.05	0.34	0.46	0.66	0.41
tran	NA	NA	n379661	MTCONS_00024457	chr17	48840212	48844918	940	-	chr15	23281011	23378337	7754	-		-588.39	0.8935	1	0.19	0.25	0.42	0	1.64	2.44
cis_mRNA_overlap	0	CompleteIn-LncIntron	LTCONS_00021153	NM_001663_dup1	chr14	50329151	50374627	289	+	chr14	50359736	50363772	3939	+	*		0.7329	1	40.35	40.96	43	9.99	10.95	11.04
tran	NA	NA	n380184	MTCONS_00022628	chr5	153822557	153825382	569	-	chr14	74751789	74769767	8842	-		-696.25	0.9011	1	0.29	0.66	0.34	2.02	3.53	2.84
tran	NA	NA	n380544	MTCONS_00026094	chr3	128679210	128684200	1065	-	chr16	22445951	22518194	21000	+		-716.26	0.983	1	0.78	0.97	0.77	3.82	4.56	3.59
cis_mRNA_overlap	0	mRNA	n363948	MTCONS_00002194	chr1	148556480	148558168	336	+	chr1	148556011	148596267	5017	+		-285.08	-0.7506	-1	2.52	3.81	2.7	4.57	4.23	4.3
cis_mRNA_overlap	0	mRNA	n363948	MTCONS_00002190	chr1	148556480	148558168	336	+	chr1	148555358	148596267	5492	+		-274.41	0.9917	0.866	2.52	3.81	2.7	0.27	0.36	0.27
tran	NA	NA	LTCONS_00025610	MTCONS_00014443	chr15	93363768	93364554	787	.	chr11	93463515	93474703	4660	-		-472.89	0.6564	0.866	1.05	1.45	1.05	4.2	4.56	2.78
tran	NA	NA	LTCONS_00025610	MTCONS_00071858	chr15	93363768	93364554	787	.	chr9	130965633	130989314	5488	+		-802.92	0.9309	0.866	1.05	1.45	1.05	3.63	5.85	4.45
tran	NA	NA	LTCONS_00025610	MTCONS_00006148	chr15	93363768	93364554	787	.	chr1	153920148	153940210	10729	-		-747.73	0.9966	0.866	1.05	1.45	1.05	0.71	0.82	0.72
cis_mRNA_overlap	0	mRNA	n386788	MTCONS_00023318	chr15	28896567	28902471	1138	+	chr15	28834608	28930558	5759	+	*		0.9934	1	0.21	0.28	0	1.34	1.42	0.84
cis_mRNA_overlap	0	mRNA	n386788	MTCONS_00023317	chr15	28896567	28902471	1138	+	chr15	28834608	28930558	6711	+		-846.1	0.9037	1	0.21	0.28	0	0.91	1.41	0.63
cis_mRNA_overlap	0	Lnc-AntiOverlap-mRNA	n338753	MTCONS_00037633	chr2	32581787	32583096	1310	-	chr2	32582096	32843966	15784	+	*		0.9787	1	0.53	0.65	0.83	4.36	4.85	6.68
tran	15960787	NA	LTCONS_00007648	MTCONS_00003143	chr1	219784048	219785802	1755	.	chr1	203764700	203823262	5223	+		-3349.3	-0.991	-1	0.12	0.02	0	0.35	0.59	0.69
cis_mRNA_overlap	0	Lnc-AntiOverlap-mRNA	n338702	NM_004036_dup1	chr2	25048479	25049586	1108	+	chr2	25042038	25142055	4397	-	*		0.9371	1	0.7	0.61	1.19	7.54	7.17	7.94
cis_mRNA_overlap	0	Lnc-AntiOverlap-mRNA	n339674	MTCONS_00043445	chr20	39726969	39766640	1458	-	chr20	39765575	39804377	5768	+	*		0.976	1	0.12	0.24	0.07	2.37	3	1.77
cis_mRNA_dw20k	16523	NA	n368173	NM_004045_dup1	chr5	151098090	151105861	1781	+	chr5	151122383	151138210	489	-	*		0.9935	1	0.55	0.67	0.62	33.46	39.16	37.36
cis_mRNA_overlap	0	Lnc-CompleteIn-mRNAIntron	LTCONS_00057369	MTCONS_00057364	chr5	795720	824714	2410	-	chr5	745253	892939	3119	-	*		0.9848	1	0.69	0.79	1.99	1.65	2.26	4.08
cis_mRNA_overlap	0	mRNA	n384306	MTCONS_00049352	chr3	123752542	123883532	2096	+	chr3	123752180	124445213	16268	+	*		0.6319	1	0.74	1.56	0.76	2.75	7.9	7.13
cis_mRNA_overlap	0	mRNA	n384306	MTCONS_00049350	chr3	123752542	123883532	2096	+	chr3	123752172	124445213	16273	+	*		-0.834	-1	0.74	1.56	0.76	0.25	0.04	0.13
cis_mRNA_overlap	0	mRNA	n384306	MTCONS_00049351	chr3	123752542	123883532	2096	+	chr3	123752180	124445213	16226	+	*		-0.9363	-1	0.74	1.56	0.76	7.25	7.17	7.22
cis_mRNA_up10k	13	NA	LTCONS_00050266	MTCONS_00048204	chr3	9427509	9439342	3145	-	chr3	9439354	9519901	8247	+	*		-0.7019	-1	2.33	1.12	2.17	1.48	2.11	2.02
cis_mRNA_up10k	13	NA	LTCONS_00050266	MTCONS_00048203	chr3	9427509	9439342	3145	-	chr3	9439354	9519901	7357	+	*		1	1	2.33	1.12	2.17	1.73	1.35	1.68
cis_mRNA_up10k	13	NA	LTCONS_00050266	MTCONS_00048205	chr3	9427509	9439342	3145	-	chr3	9439354	9519901	7795	+	*		-0.9109	-1	2.33	1.12	2.17	0	2.67	1.39
cis_mRNA_up10k	9	NA	LTCONS_00050266	MTCONS_00048198	chr3	9427509	9439342	3145	-	chr3	9439350	9519901	9023	+	*		-0.9948	-1	2.33	1.12	2.17	0.66	1.55	0.68
cis_mRNA_up10k	9	NA	LTCONS_00050266	MTCONS_00048199	chr3	9427509	9439342	3145	-	chr3	9439350	9519901	7232	+	*		0.9953	1	2.33	1.12	2.17	1.19	0.97	1.14
cis_mRNA_up10k	181	NA	n384212	MTCONS_00048202	chr3	9431591	9439174	1987	-	chr3	9439354	9519901	10675	+	*		0.9996	1	6.16	12.68	5.26	2.36	3.27	2.2
cis_mRNA_up10k	181	NA	n384212	MTCONS_00048206	chr3	9431591	9439174	1987	-	chr3	9439354	9519901	7674	+	*		-0.9747	-1	6.16	12.68	5.26	0.73	0	1.1
cis_mRNA_up10k	177	NA	n384212	MTCONS_00048201	chr3	9431591	9439174	1987	-	chr3	9439350	9519901	7418	+	*		0.8714	1	6.16	12.68	5.26	3.71	4.3	2.87
cis_mRNA_dw20k	5006	NA	LTCONS_00027094	MTCONS_00027086	chr16	90041093	90052096	4591	+	chr16	90015151	90036088	6984	+	*		0.8608	1	0.76	1.22	1.15	0.85	1.34	1.03
tran	12922392	NA	n364049	MTCONS_00007394	chr1	247537325	247538837	1000	+	chr1	234526822	234614934	14726	-		-1143.96	-0.8605	-1	0.71	0.68	0.63	1.36	1.66	1.71
cis_mRNA_overlap	0	mRNA	LTCONS_00002189	MTCONS_00002190	chr1	148555358	148575210	8395	+	chr1	148555358	148596267	5492	+	*		0.9707	0.866	0.34	0.7	0.22	0.27	0.36	0.27
cis_mRNA_dw20k	9950	NA	n381809	MTCONS_00015011	chr12	1615336	1616484	1149	+	chr12	1100697	1605387	9580	+	*		0.8776	1	0.02	0	0.03	1.27	0.84	2.77
cis_mRNA_dw20k	9950	NA	n381809	MTCONS_00015014	chr12	1615336	1616484	1149	+	chr12	1107541	1605387	9599	+	*		-0.9999	-1	0.02	0	0.03	1.32	2.99	0.54
tran	NA	NA	n324056	MTCONS_00025134	chr9	84258080	84259960	599	+	chr15	75647592	75744087	12970	-		-646.73	0.7939	1	0.21	0.3	0.23	2.31	2.94	2.82
tran	NA	NA	n324056	MTCONS_00025132	chr9	84258080	84259960	599	+	chr15	75647592	75728820	12606	-		-646.73	0.96	1	0.21	0.3	0.23	0.41	1.06	0.72
tran	641958	NA	LTCONS_00072757	MTCONS_00071097	chr9	70195031	70214882	6126	-	chr9	70856839	70890198	6527	+		-2985.58	0.8343	1	5.05	7.36	5.64	3.69	4.36	4.21
cis_mRNA_dw20k	19829	NA	LTCONS_00037039	NM_130786_dup1	chr19	58826039	58838344	520	-	chr19	58858172	58864865	1766	-	*		0.717	1	19.91	13.06	22.16	2.56	2.53	3.26
cis_mRNA_overlap	0	mRNA	n341058	MTCONS_00012274	chr11	68228239	68350597	1846	+	chr11	68228491	68382952	5882	+		-879.72	-0.9911	-1	3.01	4.46	3.79	0.64	0.35	0.45
cis_mRNA_overlap	0	mRNA	n342435	MTCONS_00034863	chr19	53901195	53930574	2010	+	chr19	53898346	53962049	4462	+	*		-0.9684	-1	1.23	1.52	1.67	2.13	1.31	1.22
cis_mRNA_up10k	4654	NA	n342435	MTCONS_00034869	chr19	53901195	53930574	2010	+	chr19	53935227	53962049	4648	+	*		-0.8744	-1	1.23	1.52	1.67	0.67	0.62	0.43
cis_mRNA_overlap	0	Lnc-AntiOverlap-mRNA	LTCONS_00073652	MTCONS_00072110	chr9	138354460	138372564	5225	-	chr9	138371483	138380741	4801	+	*		0.951	1	0.05	0.26	0.4	1.88	3.04	5.45
cis_mRNA_overlap	0	Lnc-AntiOverlap-mRNA	LTCONS_00073652	MTCONS_00072112	chr9	138354460	138372564	5225	-	chr9	138371532	138380741	5118	+	*		0.949	1	0.05	0.26	0.4	0.59	1.43	1.52
cis_mRNA_overlap	0	Lnc-AntiOverlap-mRNA	LTCONS_00073652	MTCONS_00072111	chr9	138354460	138372564	5225	-	chr9	138371532	138380741	4762	+	*		0.9974	1	0.05	0.26	0.4	0.59	1.36	1.75
cis_mRNA_up10k	1181	NA	n339393	MTCONS_00051031	chr3	71634094	71636560	2467	+	chr3	71001290	71632914	9781	-	*		-0.6547	-0.866	0.03	0.19	0.23	0.28	0.28	0.21
tran	NA	NA	n341586	MTCONS_00005308	chr8	110975869	110977604	1735	-	chr1	86810132	86862025	9802	-		-1401.07	-0.9286	-1	0.27	0.26	0.13	1.4	1.65	1.98
tran	NA	NA	n373939	MTCONS_00026094	chr13	98714196	98716549	643	-	chr16	22445951	22518194	21000	+		-806.67	0.9992	1	0.11	0.15	0.1	3.82	4.56	3.59
cis_mRNA_overlap	0	AntiCompleteIn-mRNAIntron	n379138	MTCONS_00051031	chr3	71338920	71355004	700	+	chr3	71001290	71632914	9781	-	*		0.803	0.866	0.05	0.02	0	0.28	0.28	0.21
tran	NA	NA	n337397	MTCONS_00022628	chrX	153297825	153402578	487	-	chr14	74751789	74769767	8842	-		-665.44	0.8401	0.866	1.87	2.16	1.87	2.02	3.53	2.84
tran	NA	NA	n337397	MTCONS_00022629	chrX	153297825	153402578	487	-	chr14	74751789	74769767	5311	-		-665.44	0.6318	0.866	1.87	2.16	1.87	0.39	0.46	0.05
cis_mRNA_overlap	0	mRNA	n324689	NM_005160_dup1	chr22	26036612	26043417	5052	+	chr22	25960861	26125259	9056	+	*		0.9057	1	0.22	0.35	0.3	26.1	34.64	28.04
tran	1000176	NA	n382926	MTCONS_00026094	chr16	21443347	21445776	2430	-	chr16	22445951	22518194	21000	+		-1641.02	0.9892	1	0.44	0.66	0.31	3.82	4.56	3.59
cis_mRNA_up10k	3029	NA	n384580	MTCONS_00057414	chr5	1597674	1634120	3699	-	chr5	1571958	1594646	6957	-	*		-0.8529	-1	0.57	0.1	0.29	0.71	2.32	0.86
cis_mRNA_overlap	0	mRNA	n408079	MTCONS_00015004	chr12	1100404	1605099	8472	+	chr12	1096227	1605387	13060	+		-4479.59	-0.9887	-1	2.83	2.47	1.77	0.05	0.06	0.1
cis_mRNA_overlap	0	mRNA	n405658	MTCONS_00024457	chr15	23282265	23378259	6075	-	chr15	23281011	23378337	7754	-	*		0.9794	1	0	9.29	10.73	0	1.64	2.44
tran	7643704	NA	LTCONS_00027573	MTCONS_00027653	chr16	21888253	21892223	2867	-	chr16	29535926	29625059	11433	-		-1559.69	-0.9983	-1	0.63	0.68	0.95	0.61	0.48	0
tran	7643704	NA	LTCONS_00027573	MTCONS_00027654	chr16	21888253	21892223	2867	-	chr16	29535926	29625059	11402	-		-1582.69	0.9139	1	0.63	0.68	0.95	2.15	2.38	2.58
cis_mRNA_dw20k	1754	NA	n406533	MTCONS_00048690	chr3	47053032	47054957	1012	+	chr3	47021115	47051279	11094	+	*		1	1	0.03	0.06	0.19	0.41	0.68	1.8
cis_mRNA_dw20k	1754	NA	n406533	MTCONS_00048691	chr3	47053032	47054957	1012	+	chr3	47021115	47051279	9566	+	*		0.887	1	0.03	0.06	0.19	0.42	0.87	1.15
cis_mRNA_dw20k	1754	NA	n406533	MTCONS_00048688	chr3	47053032	47054957	1012	+	chr3	47020590	47051279	12000	+	*		-0.929	-1	0.03	0.06	0.19	1.36	0.71	0.13
cis_mRNA_up10k	5656	NA	LTCONS_00048578	MTCONS_00048585	chr3	42623617	42636490	1197	+	chr3	42642145	42690806	9597	+	*		-0.8701	-1	1.32	0.96	1.56	0.13	0.15	0
cis_mRNA_up10k	5656	NA	LTCONS_00048578	MTCONS_00048586	chr3	42623617	42636490	1197	+	chr3	42642145	42690806	7936	+	*		0.9987	1	1.32	0.96	1.56	0.5	0.3	0.61
tran	NA	NA	LTCONS_00063951	MTCONS_00062362	chr6_mcf_hap5	2996967	2999422	339	-	chr6	31606805	31619760	3704	-		-264.22	0.9763	0.866	5.34	4.08	4.08	13.45	11.17	10.5
tran	NA	NA	LTCONS_00063951	MTCONS_00062367	chr6_mcf_hap5	2996967	2999422	339	-	chr6	31606805	31620477	3651	-		-264.22	0.9777	0.866	5.34	4.08	4.08	11.02	9.75	9.39

tran	NA	NA	LTCONS_00063951	MTCONS_00062364	chr6_mcf_hap5	2996967	2999422	339	-	chr6	31606805	31620477	4376	-		-248.28	-0.9398	-0.866	5.34	4.08	4.08	1.04	1.68	2.02
cis_mRNA_dw20k	10186	NA	n375218	NM_001116_dup1	chr16	3995947	4002465	4040	-	chr16	4012650	4166186	7727	-	*		-0.8443	-1	0.72	0.36	0.39	5.09	6.17	5.52
tran	NA	NA	LTCONS_00022341	MTCONS_00062598	chr14	50329259	50373431	294	-	chr6	43004263	43027309	7588	-		-260.51	0.8102	1	12258.43	12409.24	1711.39	0.14	0.23	0.08
cis_mRNA_overlap	0	Lnc-AntiOverlap-mRNA	n341742	NM_000035_dup1	chr9	104181299	104185086	3788	+	chr9	104182842	104198062	2426	-	*		0.7313	0.866	0.23	0.33	0.26	0	0.01	0.01
cis_mRNA_up10k	1937	NA	n345960	MTCONS_00025982	chr16	15016583	15019124	442	+	chr16	15021060	15045993	3803	+	*		-0.9811	-1	0.46	0.34	0.74	0.95	1.8	0
cis_mRNA_up10k	728	NA	n345960	MTCONS_00025980	chr16	15016583	15019124	442	+	chr16	15019851	15045993	4109	+	*		-0.903	-1	0.46	0.34	0.74	6.55	7.4	6.17
cis_mRNA_overlap	0	mRNA	n345960	MTCONS_00025974	chr16	15016583	15019124	442	+	chr16	15004367	15019746	4708	+		-428.01	-0.9936	-1	0.46	0.34	0.74	2.01	2.13	1.51
cis_mRNA_overlap	0	Lnc-AntiOverlap-mRNA	n407766	MTCONS_00045018	chr20	62584737	62588338	3571	+	chr20	62587985	62601221	7281	-	*		0.9978	1	0.47	0.7	0.59	2.14	3.21	2.76
cis_mRNA_overlap	0	Lnc-AntiOverlap-mRNA	n338990	NM_130786_dup1	chr19	58859117	58866549	1718	+	chr19	58858172	58864865	1766	-	*		0.9548	1	4.99	4.19	6.56	2.56	2.53	3.26
tran	NA	NA	n379352	MTCONS_00027523	chr4	110351119	110354973	957	-	chr16	19712510	19729544	7301	-		-767.78	0.933	1	1.48	1.41	1.09	1.77	1.34	0.93
cis_mRNA_overlap	0	mRNA	n405980	MTCONS_00057417	chr5	1572073	1594646	1152	-	chr5	1574556	1594678	11153	-		-788.64	-0.9494	-1	1.2	1.33	1.44	1.14	0.84	0.78
cis_mRNA_overlap	0	mRNA	n405980	MTCONS_00057415	chr5	1572073	1594646	1152	-	chr5	1571958	1594678	1490	-		-714.49	-0.9678	-1	1.2	1.33	1.44	1.96	1.74	1.27
tran	NA	NA	LTCONS_00056230	MTCONS_00036834	chr5	81563058	81569517	465	+	chr19	53407555	53421644	4538	-		-752.8	0.8858	1	1.13	1.47	1.01	1.6	1.65	1.49
tran	NA	NA	n380867	MTCONS_00041955	chr12	50305763	50314301	617	+	chr2	160171377	160568994	12663	-		-353.28	-0.9912	-1	2.56	3.22	3.31	0.13	0.07	0.05
tran	NA	NA	n380867	MTCONS_00041953	chr12	50305763	50314301	617	+	chr2	160171377	160568985	12678	-		-354.28	0.8047	1	2.56	3.22	3.31	1.67	1.88	2.36
cis_mRNA_overlap	0	Lnc-CompleteIn-mRNAIntron	n341490	MTCONS_00069681	chr8	42385192	42388316	3124	-	chr8	42328619	42396301	898	-	*		0.9989	1	2.5	3.05	2.57	1.68	2.57	1.75
cis_mRNA_overlap	0	Lnc-CompleteIn-mRNAIntron	n341490	MTCONS_00069677	chr8	42385192	42388316	3124	-	chr8	42273985	42396651	4153	-	*		-0.9876	-1	2.5	3.05	2.57	1.57	0	1.13
cis_mRNA_up10k	5140	NA	n346028	MTCONS_00025981	chr16	15013775	15014712	384	+	chr16	15019851	15045993	4475	+	*		0.8312	1	0.22	1.21	1.34	1.18	1.69	2.64
cis_mRNA_overlap	0	mRNA	n346028	MTCONS_00025973	chr16	15013775	15014712	384	+	chr16	15004214	15045993	14649	+		-430.29	0.9852	1	0.22	1.21	1.34	2.44	3.88	4.45
cis_mRNA_up10k	5140	NA	n346028	MTCONS_00025979	chr16	15013775	15014712	384	+	chr16	15019851	15045993	5075	+	*		0.9996	1	0.22	1.21	1.34	2.75	4.76	5.1
cis_mRNA_up10k	7389	NA	n385713	MTCONS_00072110	chr9	138354571	138364095	2921	-	chr9	138371483	138380741	4801	+	*		0.9984	1	0	0.03	0.08	1.88	3.04	5.45
cis_mRNA_up10k	7438	NA	n385713	MTCONS_00072112	chr9	138354571	138364095	2921	-	chr9	138371532	138380741	5118	+	*		0.837	1	0	0.03	0.08	0.59	1.43	1.52
cis_mRNA_up10k	7438	NA	n385713	MTCONS_00072111	chr9	138354571	138364095	2921	-	chr9	138371532	138380741	4762	+	*		0.946	1	0	0.03	0.08	0.59	1.36	1.75
cis_mRNA_up10k	8	NA	LTCONS_00039016	NM_001616_dup1	chr2	148600360	148602563	2204	+	chr2	148602570	148688393	5244	+	*		0.9802	1	2.49	2.71	2.54	21.18	27.75	23.86
cis_mRNA_dw20k	3433	NA	LTCONS_00070907	MTCONS_00070896	chr9	34665406	34682348	2161	+	chr9	34646573	34661974	5657	+	*		0.9185	1	0.69	1.39	0.9	5.05	6.8	6.2
cis_mRNA_overlap	0	Lnc-AntiOverlap-mRNA	n385714	MTCONS_00072110	chr9	138363011	138372564	875	-	chr9	138371483	138380741	4801	+	*		0.8949	1	0	0.11	0.15	1.88	3.04	5.45
cis_mRNA_overlap	0	Lnc-AntiOverlap-mRNA	n385714	MTCONS_00072112	chr9	138363011	138372564	875	-	chr9	138371532	138380741	5118	+	*		0.9851	1	0	0.11	0.15	0.59	1.43	1.52
cis_mRNA_overlap	0	Lnc-AntiOverlap-mRNA	n385714	MTCONS_00072111	chr9	138363011	138372564	875	-	chr9	138371532	138380741	4762	+	*		0.9971	1	0	0.11	0.15	0.59	1.36	1.75
cis_mRNA_up10k	462	NA	n386461	MTCONS_00067823	chr7	154795143	154797412	2270	+	chr7	154735400	154794682	3712	-	*		0.9646	1	4.49	4.3	4.44	1.31	1.13	1.22
cis_mRNA_dw20k	5557	NA	n339588	MTCONS_00043095	chr20	18303196	18339944	2490	-	chr20	18269121	18297640	2943	+	*		-0.8768	-1	0.04	0.06	0.07	2.24	2.01	1.2
cis_mRNA_dw20k	5557	NA	n339588	MTCONS_00043099	chr20	18303196	18339944	2490	-	chr20	18276237	18297640	5207	+	*		0.9831	1	0.04	0.06	0.07	0.48	0.69	0.73
cis_mRNA_up10k	5901	NA	n407742	NM_000031_dup1	chr9	116169518	116172955	2399	-	chr9	116148592	116163618	3183	-	*		0.9707	0.866	0.49	0.23	0.23	4	3.82	3.76
cis_mRNA_dw20k	8502	NA	n381443	NM_000698_dup1	chr10	45950066	45952578	2513	-	chr10	45869629	45941565	2554	+	*		0.8562	0.866	1.5	2.1	1.81	0.07	0.16	0.07
cis_mRNA_overlap	0	mRNA	n406246	MTCONS_00034859	chr19	53868968	53889841	6094	+	chr19	53868951	53890918	7088	+		-2451.06	0.763	1	2.08	1.79	2.07	0.53	0.34	0.4
cis_mRNA_up10k	198	NA	LTCONS_00070644	MTCONS_00070565	chr8	145670009	145670953	945	.	chr8	145648999	145669812	6734	-	*		-0.9568	-1	0.94	0.38	0.4	2.43	3.47	3.13
cis_mRNA_up10k	6195	NA	LTCONS_00070644	MTCONS_00070562	chr8	145670009	145670953	945	.	chr8	145648999	145663815	5605	-	*		-0.9441	-1	0.94	0.38	0.4	0.1	0.32	0.24
tran	NA	NA	n387169	MTCONS_00057412	chr2	170183043	170183659	617	+	chr5	1571958	1586534	12677	-		-1392.6	0.9993	1	6.22	8.04	7.4	2.97	3.83	3.5
cis_mRNA_overlap	0	Lnc-AntiOverlap-mRNA	n338840	MTCONS_00015044	chr12	2785167	2800284	2190	-	chr12	2162416	2807115	13528	+	*		-0.9734	-1	0.02	0	0.06	0.24	0.39	0.11
cis_mRNA_overlap	0	Lnc-AntiOverlap-mRNA	n338840	MTCONS_00015043	chr12	2785167	2800284	2190	-	chr12	2162416	2807115	13621	+	*		-0.9994	-1	0.02	0	0.06	0.07	0.11	0
tran	NA	NA	n325153	MTCONS_00022628	chr2	122264506	122269086	650	+	chr14	74751789	74769767	8842	-		-363.48	0.9789	1	0	0.11	0.04	2.02	3.53	2.84
cis_mRNA_overlap	0	Lnc-AntiOverlap-mRNA	n410077	MTCONS_00030104	chr17	80400463	80408707	2028	-	chr17	80376219	80400611	2236	+	*		0.724	1	0.51	0.77	0.72	1.89	2.86	2
cis_mRNA_overlap	0	Lnc-AntiOverlap-mRNA	n410077	MTCONS_00030100	chr17	80400463	80408707	2028	-	chr17	80375909	80400611	4570	+	*		0.9998	1	0.51	0.77	0.72	0.13	0.32	0.28
cis_mRNA_overlap	0	Lnc-AntiOverlap-mRNA	n410077	MTCONS_00030105	chr17	80400463	80408707	2028	-	chr17	80376245	80400611	3607	+	*		0.8977	1	0.51	0.77	0.72	0.34	0.39	0.36
cis_mRNA_dw20k	545	NA	n341097	MTCONS_00014441	chr11	93459820	93462971	3152	-	chr11	93463515	93474703	1516	-	*		0.9506	1	2.42	2.5	1.6	0.72	1	0.28
cis_mRNA_dw20k	545	NA	n341097	MTCONS_00014443	chr11	93459820	93462971	3152	-	chr11	93463515	93474703	4660	-	*		0.9937	1	2.42	2.5	1.6	4.2	4.56	2.78
cis_mRNA_dw20k	545	NA	n341097	MTCONS_00014446	chr11	93459820	93462971	3152	-	chr11	93463515	93474703	1953	-	*		0.9998	1	2.42	2.5	1.6	9.91	10.27	5.09
cis_mRNA_overlap	0	Lnc-AntiOverlap-mRNA	LTCONS_00027204	MTCONS_00025725	chr16	1826236	1832921	6298	-	chr16	1823145	1826958	2918	+	*		0.9806	1	1.12	1.39	1.63	1.43	1.83	2.55
cis_mRNA_overlap	0	mRNA	n410096	MTCONS_00071442	chr9	100000708	100139577	6348	+	chr9	100000708	100139577	6379	+		-3228.83	0.9912	1	0.1	0.48	0.92	0.25	0.58	0.82
cis_mRNA_overlap	0	mRNA	n410096	MTCONS_00071439	chr9	100000708	100139577	6348	+	chr9	100000708	100059596	3098	+		-1787.3	0.9969	1	0.1	0.48	0.92	0.41	0.56	0.79
cis_mRNA_overlap	0	mRNA	n338293	MTCONS_00035440	chr19	8166027	8537075	1702	-	chr19	8130287	8212385	9246	-	*		-0.9851	-1	0.29	0.22	0.31	0.26	0.68	0.24
cis_mRNA_overlap	0	mRNA	n338293	MTCONS_00035442	chr19	8166027	8537075	1702	-	chr19	8130287	8215127	9911	-	*		0.9481	1	0.29	0.22	0.31	0.62	0.19	1.07
tran	977875	NA	LTCONS_00005930	MTCONS_00002194	chr1	147574207	147578137	2587	-	chr1	148556011	148596267	5017	+		-1336.49	-0.9724	-1	1.28	1.78	1.57	4.57	4.23	4.3
tran	977222	NA	LTCONS_00005930	MTCONS_00002190	chr1	147574207	147578137	2587	-	chr1	148555358	148596267	5492	+		-1368.9	0.8164	0.866	1.28	1.78	1.57	0.27	0.36	0.27
tran	NA	NA	n410215	MTCONS_00073041	chr8	48100930	48104439	3510	+	chr9	98205264	98271030	8274	-		-1941.74	-0.9883	-1	2.17	0.5	2.03	0.56	1.15	0.7
tran	NA	NA	n410215	MTCONS_00073042	chr8	48100930	48104439	3510	+	chr9	98205264	98271065	9738	-		-2015.76	-1	-1	2.17	0.5	2.03	0.23	0.35	0.24
cis_mRNA_overlap	0	mRNA	n345547	MTCONS_00041955	chr2	160561651	160567363	942	-	chr2	160171377	160568994	12663	-	*		-0.9608	-1	0	0.07	0.14	0.13	0.07	0.05
cis_mRNA_overlap	0	mRNA	n345547	MTCONS_00041953	chr2	160561651	160567363	942	-	chr2	160171377	160568985	12678	-	*		0.9754	1	0	0.07	0.14	1.67	1.88	2.36
cis_mRNA_overlap	0	Lnc-AntiOverlap-mRNA	n340147	NM_000684_dup1	chr10	115800691	115804503	3811	-	chr10	115803806	115806667	2862	+	*		-0.9878	-0.866	0.03	0	0.03	0.06	0.26	0.02

tran	NA	NA	n376908	MTCONS_00003507	chr20	46683233	46692077	815	+	chr1	227058269	227110171	9828	+		-630.44	-0.9555	-1	0.39	0.29	0.12	0.25	0.37	0.44
tran	NA	NA	n376908	MTCONS_00003503	chr20	46683233	46692077	815	+	chr1	227057647	227110171	10475	+		-630.44	-0.9735	-1	0.39	0.29	0.12	0.15	0.19	0.22
tran	NA	NA	n367794	MTCONS_00052416	chr5	101941339	101941901	391	+	chr4	2965178	3042475	6358	+		-281.24	0.9533	1	0	0.19	0.12	1.41	1.74	1.53
tran	NA	NA	LTCONS_00047464	MTCONS_00000237	chr22	22674152	22675668	1517	-	chr1	6640063	6649340	5957	+		-1677.37	0.9124	1	1.28	1.99	1.55	0.55	2.21	1.8
cis_mRNA_overlap	0	mRNA	n386097	MTCONS_00023318	chr15	28834638	28836269	1023	+	chr15	28834608	28930558	5759	+	*		-0.9999	-1	1.49	1.37	2.34	1.34	1.42	0.84
cis_mRNA_overlap	0	mRNA	n386097	MTCONS_00023317	chr15	28834638	28836269	1023	+	chr15	28834608	28930558	6711	+	*		-0.8412	-1	1.49	1.37	2.34	0.91	1.41	0.63
cis_mRNA_up10k	59	NA	n373562	MTCONS_00020447	chr13	79980444	79997087	1753	+	chr13	79889027	79980386	4748	-	*		0.9708	1	0.16	0.14	0.38	2.82	2.45	3.61
cis_mRNA_up10k	460	NA	n373562	MTCONS_00020445	chr13	79980444	79997087	1753	+	chr13	79889027	79979985	3112	-	*		0.9984	1	0.16	0.14	0.38	5	4.87	5.79
cis_mRNA_up10k	59	NA	n373562	MTCONS_00020446	chr13	79980444	79997087	1753	+	chr13	79889027	79980386	5403	-	*		-0.9772	-1	0.16	0.14	0.38	3.29	3.46	2.88
cis_mRNA_up10k	59	NA	n373562	MTCONS_00020448	chr13	79980444	79997087	1753	+	chr13	79889027	79980386	3504	-	*		-0.7096	-1	0.16	0.14	0.38	0.46	0.66	0.41
tran	278806	NA	n381066	MTCONS_00000469	chr1	16959590	16969640	596	-	chr1	17248445	17299508	8051	+		-536.78	0.956	1	0.19	1.54	2.37	0.8	1.03	1.44
tran	278806	NA	n381066	MTCONS_00000470	chr1	16959590	16969640	596	-	chr1	17248445	17299508	8543	+		-536.78	0.7897	0.866	0.19	1.54	2.37	1.31	1.31	2.13
cis_mRNA_overlap	0	mRNA	LTCONS_00057363	MTCONS_00057364	chr5	709662	851250	7985	-	chr5	745253	892939	3119	-	*		0.9998	1	1.17	1.67	3.01	1.65	2.26	4.08
cis_mRNA_dw20k	11008	NA	LTCONS_00057363	MTCONS_00057374	chr5	709662	851250	7985	-	chr5	862257	892939	7131	-	*		0.9411	1	1.17	1.67	3.01	1.46	1.65	1.79
cis_mRNA_dw20k	7392	NA	LTCONS_00057363	MTCONS_00057371	chr5	709662	851250	7985	-	chr5	858641	892939	5789	-	*		0.9288	1	1.17	1.67	3.01	0.14	0.34	0.47
cis_mRNA_dw20k	19882	NA	n386154	MTCONS_00027457	chr16	15188268	15203054	2598	+	chr16	15222935	15225560	2027	-	*		-0.9916	-1	0.25	0.34	0.47	6.04	5.29	3.48
tran	NA	NA	n369174	MTCONS_00022629	chr6	277864	286279	721	-	chr14	74751789	74769767	5311	-		-735.53	0.7606	1	0.15	0.32	0.11	0.39	0.46	0.05
tran	NA	NA	n324054	MTCONS_00012040	chr9	88436882	88501231	530	+	chr11	63996107	63997566	1327	+		-502.89	0.9477	1	0.15	0.19	0.17	0.67	1.87	1.62
cis_mRNA_overlap	0	Lnc-CompleteIn-mRNAIntron	n325683	MTCONS_00020416	chr13	77747856	77749650	342	-	chr13	77616731	77901236	17064	-	*		0.9104	1	0	0.42	0.21	2.31	2.78	2.36
cis_mRNA_overlap	0	Lnc-CompleteIn-mRNAIntron	n325683	MTCONS_00020417	chr13	77747856	77749650	342	-	chr13	77616731	77901236	17259	-	*		-0.996	-1	0	0.42	0.21	6.41	4.42	5.26
cis_mRNA_overlap	0	Lnc-CompleteIn-mRNAIntron	n325683	MTCONS_00020414	chr13	77747856	77749650	342	-	chr13	77616731	77901215	17340	-	*		0.975	1	0	0.42	0.21	1.82	3.01	2.18
cis_mRNA_overlap	0	Lnc-CompleteIn-mRNAIntron	n338476	MTCONS_00033929	chr19	19614692	19615463	375	+	chr19	19516727	19619821	5476	+	*		-0.6791	-1	0.1	0.09	0.28	2.21	4.13	1.77
cis_mRNA_dw20k	7909	NA	LTCONS_00007630	MTCONS_00002403	chr1	154973498	154974407	910	.	chr1	154955770	154965590	2589	+	*		0.9848	1	0	0.07	0.03	0.06	0.39	0.15
cis_mRNA_dw20k	7909	NA	LTCONS_00007630	MTCONS_00002401	chr1	154973498	154974407	910	.	chr1	154955770	154965590	1495	+	*		-0.9837	-1	0	0.07	0.03	1.72	1.12	1.56
cis_mRNA_dw20k	7909	NA	LTCONS_00007630	MTCONS_00002399	chr1	154973498	154974407	910	.	chr1	154955770	154965590	1650	+	*		0.979	1	0	0.07	0.03	1.43	3.11	2.45
tran	NA	NA	n339024	MTCONS_00009569	chr2	176047421	176048342	922	+	chr10	13482875	13570598	7728	-		-443.02	-0.9067	-1	0.08	0	0.03	1.22	2.68	1.57
cis_mRNA_dw20k	17590	NA	n364539	MTCONS_00038436	chr2	97948299	97957475	2757	+	chr2	97778898	97930710	5878	+	*		-0.9364	-1	2.13	3.23	3.38	10.85	9.7	8.75
cis_mRNA_dw20k	17590	NA	n364539	MTCONS_00038432	chr2	97948299	97957475	2757	+	chr2	97777789	97930710	5308	+	*		0.9968	1	2.13	3.23	3.38	0.35	0.39	0.4
cis_mRNA_dw20k	17590	NA	n364539	MTCONS_00038434	chr2	97948299	97957475	2757	+	chr2	97778872	97930710	7270	+	*		-0.9905	-1	2.13	3.23	3.38	2.95	2.22	1.97
cis_mRNA_dw20k	17590	NA	n364539	MTCONS_00038435	chr2	97948299	97957475	2757	+	chr2	97778898	97930710	9268	+	*		-0.9787	-1	2.13	3.23	3.38	7.65	7.2	6.99
tran	58143213	NA	n345900	MTCONS_00067268	chr7	44073613	44076409	437	-	chr7	102219621	102251963	7267	-		-372	0.9725	1	1.47	2.59	2.25	0.51	0.92	0.89
tran	58043872	NA	n345900	MTCONS_00067261	chr7	44073613	44076409	437	-	chr7	102120280	102257205	6445	-		-386.89	0.9233	1	1.47	2.59	2.25	0.91	2.28	1.39
cis_mRNA_overlap	0	Lnc-AntiOverlap-mRNA	LTCONS_00045263	MTCONS_00045937	chr21	34100420	34115515	3872	+	chr21	34105977	34144169	8461	-	*		-0.8974	-1	0.23	0.89	1.3	1.95	1.54	0
cis_mRNA_overlap	0	Lnc-CompleteIn-mRNAIntron	n340626	MTCONS_00030865	chr17	34884342	34886197	1856	-	chr17	34851504	34891404	5790	-	*		-0.8204	-1	0.32	0.76	0.28	0.46	0.19	0.94
cis_mRNA_overlap	0	Lnc-CompleteIn-mRNAIntron	n340626	MTCONS_00030866	chr17	34884342	34886197	1856	-	chr17	34851504	34891404	5923	-	*		0.9995	1	0.32	0.76	0.28	0.65	2.76	0.54
cis_mRNA_overlap	0	Lnc-AntiOverlap-mRNA	LTCONS_00072544	MTCONS_00070897	chr9	34661799	34666208	3400	-	chr9	34646620	34661974	4578	+	*		-1	-1	1.6	2.07	0.88	0.38	0.21	0.64
tran	58040059	NA	n406597	MTCONS_00067260	chr7	44068486	44080222	1527	-	chr7	102120280	102252017	6534	-		-1286.75	0.9966	1	1.64	2.03	3.62	0.57	0.73	1.97
cis_mRNA_up10k	9638	NA	n406597	MTCONS_00066634	chr7	44068486	44080222	1527	-	chr7	43998541	44058849	4268	-	*		0.8369	1	1.64	2.03	3.62	0.11	0.16	0.18
cis_mRNA_overlap	0	Lnc-AntiOverlap-mRNA	n338987	MTCONS_00041952	chr2	160471807	160473593	1494	+	chr2	160171377	160491797	12332	-	*		-0.9907	-0.866	0.46	0.44	0.46	1.42	2.17	1.53
cis_mRNA_overlap	0	Lnc-CompleteIn-mRNAIntron	n339074	MTCONS_00042297	chr2	198115585	198167243	1066	-	chr2	197851386	198175523	7384	-	*		-0.8171	-1	0.17	0.21	0	0.65	0.05	0.86
tran	NA	NA	LTCONS_00038412	MTCONS_00047690	chr2	96683858	96688981	2492	+	chr22	32041675	32058206	498	-		-483.81	0.7847	1	1.65	2.08	2.32	0.33	0.36	1.85
cis_mRNA_overlap	0	Lnc-CompleteIn-mRNAIntron	LTCONS_00057365	MTCONS_00057364	chr5	749201	751112	1912	-	chr5	745253	892939	3119	-	*		-0.8783	-1	1.15	1.06	1.02	1.65	2.26	4.08
cis_mRNA_dw20k	5295	NA	n342469	MTCONS_00026181	chr16	28854108	28857678	1621	-	chr16	28833270	28848814	5525	+	*		0.9076	1	29.86	30.87	28.89	0.13	0.34	0.11
cis_mRNA_overlap	0	Lnc-AntiOverlap-mRNA	LTCONS_00027586	MTCONS_00026095	chr16	22430867	22449081	3744	-	chr16	22445951	22547842	9825	+	*		-0.8513	-1	1.39	0.52	2.65	0.05	0.59	0
cis_mRNA_overlap	0	Lnc-AntiOverlap-mRNA	LTCONS_00027586	MTCONS_00026094	chr16	22430867	22449081	3744	-	chr16	22445951	22518194	21000	+	*		-0.921	-1	1.39	0.52	2.65	3.82	4.56	3.59
cis_mRNA_up10k	3629	NA	LTCONS_00027586	MTCONS_00026100	chr16	22430867	22449081	3744	-	chr16	22452709	22547842	10437	+	*		-0.9979	-1	1.39	0.52	2.65	1.7	1.96	1.4
cis_mRNA_overlap	0	Lnc-AntiOverlap-mRNA	LTCONS_00027586	MTCONS_00026096	chr16	22430867	22449081	3744	-	chr16	22445951	22553889	11744	+	*		-0.9263	-1	1.39	0.52	2.65	0.06	0.09	0.05
tran	NA	NA	n410541	MTCONS_00035490	chr16	89284111	89295965	3481	+	chr19	9522659	9546244	4290	-		-1689.73	-0.772	-1	1.28	2.23	2.61	3.08	3.02	2.38
tran	NA	NA	n410541	MTCONS_00035489	chr16	89284111	89295965	3481	+	chr19	9522659	9546244	3985	-		-1698.56	-0.9595	-1	1.28	2.23	2.61	2.04	1.48	0.83
cis_mRNA_up10k	4213	NA	n345532	MTCONS_00038172	chr2	74208236	74208568	333	-	chr2	74212780	74335391	10093	+	*		-0.8271	-1	0	0.11	0.53	1.71	1.51	1.44
tran	375118	NA	LTCONS_00036842	MTCONS_00034858	chr19	53467065	53493457	7496	-	chr19	53868574	53890918	6325	+		-2657.34	0.8333	1	0.39	0.46	0	0.26	0.81	0
tran	375495	NA	LTCONS_00036842	MTCONS_00034860	chr19	53467065	53493457	7496	-	chr19	53868951	53890918	6302	+		-2643.66	-0.9746	-1	0.39	0.46	0	1.25	1.01	1.67
tran	45422	NA	LTCONS_00036842	MTCONS_00034836	chr19	53467065	53493457	7496	-	chr19	53407555	53421644	4538	-		-2162.71	0.9858	1	0.39	0.46	0	1.6	1.65	1.49
tran	544030	NA	n410146	MTCONS_00034859	chr19	53300661	53324922	4467	-	chr19	53868951	53890918	7088	+		-1913.16	-0.9993	-1	2.1	2.56	2.43	0.53	0.34	0.4
cis_mRNA_dw20k	751	NA	n379099	MTCONS_00045938	chr21	34100426	34105227	2945	+	chr21	34105977	34144169	12472	-	*		-0.7338	-1	2.2	1.48	1.63	2.04	2.4	2.08
cis_mRNA_up10k	173	NA	LTCONS_00050265	MTCONS_00048200	chr3	9427509	9439178	3910	-	chr3	9439350	9519901	7913	+	*		-0.9714	-1	1.16	2.72	3.52	1.78	0.32	0.14
tran	NA	NA	LTCONS_00067917	MTCONS_00025135	chr7	148787954	148789503	1550	.	chr15	75647592	75744087	13925	-		-1621.89	0.7962	1	1.24	1.72	1.37	2.36	3.13	3.02
tran	NA	NA	n387512	MTCONS_00071858	chr6	3266750	3267543	592	+	chr9	130965633	130989314	5488	+		-787.95	-0.9569	-1	3.63	2.72	3.06	3.63	5.85	4.45
cis_mRNA_dw20k	11894	NA	n339360	MTCONS_00050802	chr3	50371526	50372548	1022	+	chr3	50384441	50388486	1630	-	*		0.9522	0.866	0.03	0.09	0.03	1.37	1.48	1.32

tran	NA	NA	n370780	MTCONS_00020416	chr9	95909322	95910838	410	+	chr13	77616731	77901236	17064	-		-279.08	-0.9977	-1	2.24	1.1	2.04	2.31	2.78	2.36
tran	NA	NA	n370780	MTCONS_00020417	chr9	95909322	95910838	410	+	chr13	77616731	77901236	17259	-		-279.08	0.9012	1	2.24	1.1	2.04	6.41	4.42	5.26
tran	NA	NA	n375174	MTCONS_00012040	chr16	77786366	77809904	836	+	chr11	63996107	63997566	1327	+		-516.8	-0.9326	-1	0.13	0	0.07	0.67	1.87	1.62
tran	NA	NA	n382143	MTCONS_00005308	chr14	24439148	24453530	2221	+	chr1	86810132	86862025	9802	-		-922.79	0.9923	1	3.31	4.78	6.04	1.4	1.65	1.98
cis_mRNA_overlap	0	mRNA	n332501	MTCONS_00073581	chr9	134473665	134497278	473	-	chr9	134452157	134615260	6097	-		-389.37	-0.7324	-1	0	0.09	0.01	5.91	0.87	1.91
cis_mRNA_overlap	0	mRNA	n332501	MTCONS_00073579	chr9	134473665	134497278	473	-	chr9	134452157	134615258	6209	-		-389.37	0.692	1	0	0.09	0.01	0.31	4.59	3.95
tran	NA	NA	n379844	MTCONS_00065636	chr12	22754359	22771801	429	+	chr7	128430115	128462217	2569	+		-268.22	-0.6673	-0.866	0.45	0.32	0.32	0.13	0.5	0.21
tran	NA	NA	n379844	MTCONS_00065633	chr12	22754359	22771801	429	+	chr7	128430115	128462217	19038	+		-271.26	-0.7986	-0.866	0.45	0.32	0.32	3.48	4.42	3.85
tran	NA	NA	n379844	MTCONS_00065635	chr12	22754359	22771801	429	+	chr7	128430115	128462217	3660	+		-271.26	-0.8061	-0.866	0.45	0.32	0.32	0.79	2.1	1.32
cis_mRNA_overlap	0	mRNA	n340076	MTCONS_00008504	chr10	76672544	76944815	2287	+	chr10	76586245	76792639	7808	+	*		0.9441	1	0.51	0.77	0.62	0.34	0.8	0.39
cis_mRNA_overlap	0	mRNA	n340076	MTCONS_00008502	chr10	76672544	76944815	2287	+	chr10	76585268	76792639	8001	+	*		-0.9266	-1	0.51	0.77	0.62	0.44	0.26	0.43
cis_mRNA_overlap	0	mRNA	n340076	MTCONS_00008501	chr10	76672544	76944815	2287	+	chr10	76585227	76792639	8591	+	*		0.9985	1	0.51	0.77	0.62	0.47	0.98	0.71
cis_mRNA_overlap	0	Lnc-CompleteIn-mRNAExon	n338783	MTCONS_00040669	chr2	40324916	40328505	3590	-	chr2	40322403	40680022	23810	-	*		0.917	1	7.79	6.38	4.43	5.48	5.43	3.37
cis_mRNA_dw20k	17411	NA	n408247	NM_000041_dup1	chr19	45430060	45434643	631	+	chr19	45409039	45412650	1180	+	*		0.8731	1	0	0.16	0.1	1.2	2.27	1.38
tran	NA	NA	LTCONS_00021149	MTCONS_00062599	chr14	50320334	50379335	298	+	chr6	43004263	43027309	8306	-		-392.26	0.9971	1	1.51	3.97	3.51	1.44	2.25	2.04
cis_mRNA_dw20k	12951	NA	n333962	MTCONS_00059517	chr6	4078218	4078499	282	-	chr6	4021470	4065268	6935	+	*		0.9883	1	0	0.09	0.27	0.3	0.52	0.77
cis_mRNA_dw20k	12951	NA	n333962	MTCONS_00059520	chr6	4078218	4078499	282	-	chr6	4021533	4065268	5119	+	*		0.9863	1	0	0.09	0.27	0	0.34	0.71
cis_mRNA_dw20k	12951	NA	n333962	MTCONS_00059519	chr6	4078218	4078499	282	-	chr6	4021504	4065268	5220	+	*		-0.8831	-1	0	0.09	0.27	3.37	1.24	0.59
cis_mRNA_dw20k	12951	NA	n333962	MTCONS_00059518	chr6	4078218	4078499	282	-	chr6	4021470	4065268	4742	+	*		0.8802	1	0	0.09	0.27	5.42	6.03	6.21
tran	NA	NA	n366535	MTCONS_00057417	chr3	166896245	166901168	267	-	chr5	1574556	1594678	11153	-		-176.82	-0.9967	-1	0.16	0.56	0.69	1.14	0.84	0.78
tran	NA	NA	n366535	MTCONS_00057415	chr3	166896245	166901168	267	-	chr5	1571958	1594678	1490	-		-176.82	-0.8813	-1	0.16	0.56	0.69	1.96	1.74	1.27
cis_mRNA_up10k	396	NA	LTCONS_00028390	MTCONS_00025627	chr16	326153	330211	4059	.	chr16	330606	337216	2802	+	*		0.9592	1	0.49	1.07	0.8	6.39	8.71	8.23
cis_mRNA_up10k	396	NA	LTCONS_00028390	NM_001176_dup1	chr16	326153	330211	4059	.	chr16	330606	333003	942	+	*		-0.9261	-1	0.49	1.07	0.8	80.29	66.85	68.28
cis_mRNA_up10k	396	NA	LTCONS_00028390	MTCONS_00025626	chr16	326153	330211	4059	.	chr16	330606	337216	3956	+	*		0.9537	1	0.49	1.07	0.8	13.63	18.36	14.88
cis_mRNA_up10k	396	NA	LTCONS_00028390	MTCONS_00025628	chr16	326153	330211	4059	.	chr16	330606	337216	3827	+	*		0.9897	1	0.49	1.07	0.8	7.5	9.94	8.5
cis_mRNA_up10k	212	NA	LTCONS_00028390	MTCONS_00027131	chr16	326153	330211	4059	.	chr16	318302	325942	3122	-	*		-0.943	-1	0.49	1.07	0.8	4.01	0.01	3.08
cis_mRNA_up10k	240	NA	LTCONS_00028390	MTCONS_00027129	chr16	326153	330211	4059	.	chr16	318302	325914	4568	-	*		0.9752	1	0.49	1.07	0.8	1.06	1.51	1.39
tran	NA	NA	n341492	MTCONS_00073041	chr8	48102395	48104885	2491	+	chr9	98205264	98271030	8274	-		-1286.55	-0.9757	-1	2.22	1.13	2.21	0.56	1.15	0.7
tran	NA	NA	n341492	MTCONS_00073042	chr8	48102395	48104885	2491	+	chr9	98205264	98271065	9738	-		-1241.09	-0.9977	-1	2.22	1.13	2.21	0.23	0.35	0.24
cis_mRNA_overlap	0	mRNA	LTCONS_00027056	MTCONS_00027052	chr16	89753016	89762800	4465	+	chr16	89753012	89762800	2666	+		-2136.98	-0.9984	-1	0.17	0.12	0.09	3.04	3.81	4.38
cis_mRNA_overlap	0	Lnc-AntiOverlap-mRNA	n379088	MTCONS_00052098	chr3	195634947	195638128	2096	+	chr3	195590236	195636450	5573	-	*		0.9936	1	0	0.03	0.01	2.23	3.28	2.47
cis_mRNA_overlap	0	Lnc-AntiOverlap-mRNA	n379088	MTCONS_00052097	chr3	195634947	195638128	2096	+	chr3	195590236	195636450	5052	-	*		-0.8891	-1	0	0.03	0.01	1.26	0	0.31
cis_mRNA_dw20k	649	NA	n383057	MTCONS_00030452	chr17	8123949	8127361	2093	-	chr17	8128009	8151413	7897	-	*		-0.992	-1	1.08	0.78	0.45	0.6	0.77	0.89
cis_mRNA_up10k	9460	NA	n373788	MTCONS_00020444	chr13	79989444	79999377	3368	+	chr13	79889027	79979985	3758	-	*		0.999	1	0.28	0.5	0.73	3.36	3.82	4.38
cis_mRNA_up10k	391	NA	n341696	NM_001163_dup1	chr9	72287665	72288629	965	+	chr9	72042449	72287275	6584	-	*		-0.9924	-1	0.11	0.03	0	19.25	21.19	22.4
cis_mRNA_up10k	177	NA	n384210	MTCONS_00048200	chr3	9430537	9439174	1834	-	chr3	9439350	9519901	7913	+	*		0.833	1	5.43	3.91	1.18	1.78	0.32	0.14
cis_mRNA_dw20k	2788	NA	n408905	NM_001619_dup1	chr11	67056816	67069955	2223	+	chr11	67033905	67054029	3444	+	*		0.9982	1	0.91	2.51	2.58	20.25	23.88	24.31
cis_mRNA_overlap	0	Lnc-CompleteIn-mRNAIntron	n333833	MTCONS_00026276	chr16	30214329	30214825	497	+	chr16	30205627	30216563	6546	+	*		0.9894	1	1.13	1.64	1.41	0.07	0.38	0.28
cis_mRNA_dw20k	5106	NA	n333833	MTCONS_00026274	chr16	30214329	30214825	497	+	chr16	30205499	30209224	1172	+	*		-0.9853	-1	1.13	1.64	1.41	0.22	0.07	0.16
cis_mRNA_overlap	0	Lnc-CompleteIn-mRNAIntron	n333833	MTCONS_00026277	chr16	30214329	30214825	497	+	chr16	30205627	30216563	5004	+	*		0.9096	1	1.13	1.64	1.41	0.68	1.6	1.56
tran	NA	NA	n385806	MTCONS_00005308	chrX	103367013	103369317	2305	-	chr1	86810132	86862025	9802	-		-1043.6	0.9568	1	0.77	1.17	1.35	1.4	1.65	1.98
cis_mRNA_overlap	0	Lnc-CompleteIn-mRNAIntron	n339315	MTCONS_00026943	chr16	85625872	85629315	3443	+	chr16	85588698	85710002	8662	+	*		0.8732	1	0.1	0.26	0.11	0.55	0.83	0.7
cis_mRNA_overlap	0	Lnc-CompleteIn-mRNAIntron	n340725	MTCONS_00058857	chr5	168581858	168583220	1361	-	chr5	168081512	168728133	28723	-	*		-0.9971	-1	0.03	0.02	0.05	8.82	9.47	7.85
cis_mRNA_up10k	4247	NA	n339759	MTCONS_00045018	chr20	62605467	62611362	2070	-	chr20	62587985	62601221	7281	-	*		0.9918	1	0.53	1	0.75	2.14	3.21	2.76
cis_mRNA_overlap	0	mRNA	n410193	MTCONS_00030905	chr17	36351796	36413256	1652	-	chr17	36336491	36421858	4845	-	*		-0.8672	-1	1.99	2.21	1.23	0.11	0	0.16
cis_mRNA_overlap	0	mRNA	n410193	MTCONS_00030907	chr17	36351796	36413256	1652	-	chr17	36340450	36413290	3041	-		-948.19	-0.9942	-1	1.99	2.21	1.23	1.56	1.2	2.31
cis_mRNA_overlap	0	mRNA	n410193	MTCONS_00030908	chr17	36351796	36413256	1652	-	chr17	36340806	36421858	2973	-	*		0.8059	1	1.99	2.21	1.23	1.04	1.88	0.82
tran	NA	NA	n325002	MTCONS_00012040	chr20	31249676	31256636	570	+	chr11	63996107	63997566	1327	+		-450.37	-0.6611	-0.866	0.06	0	0.06	0.67	1.87	1.62
cis_mRNA_dw20k	17589	NA	n407026	MTCONS_00068762	chr8	94767072	94831460	3365	+	chr8	94712686	94749484	11199	+	*		-0.8946	-1	0.89	1.11	0.37	0.19	0.07	0.24
cis_mRNA_overlap	0	mRNA	n345971	MTCONS_00026012	chr16	16411889	16470859	2001	+	chr16	16410889	16464173	5350	+	*		0.9234	1	0.16	0.22	0.18	2.69	4.03	3.6
cis_mRNA_overlap	0	mRNA	n345971	MTCONS_00026019	chr16	16411889	16470859	2001	+	chr16	16456592	16470859	1890	+		-1490.42	-0.9894	-1	0.16	0.22	0.18	3.17	2.91	3.05
tran	6982038	NA	n382958	MTCONS_00026096	chr16	29535926	29542213	5316	-	chr16	22445951	22553889	11744	+		-2698.57	0.9113	1	0.81	0.84	0.76	0.06	0.09	0.05
tran	NA	NA	LTCONS_00042561	MTCONS_00071101	chr2	223745026	223807923	648	-	chr9	70856839	70920000	12104	+		-683.8	-0.9338	-1	1.57	1.18	0.52	0.5	0.77	0.89
tran	NA	NA	n370632	MTCONS_00002201	chr8	99057943	99059896	771	+	chr1	148737657	148750403	3372	+		-505.44	0.7315	1	2.33	2.59	1.66	2.92	4.44	2.88
cis_mRNA_overlap	0	Lnc-CompleteIn-mRNAIntron	n335787	MTCONS_00063191	chr6	119312344	119312956	613	-	chr6	119282658	119399939	8703	-	*		-0.9758	-1	0.18	0.05	0.27	0.92	2.06	0.62
cis_mRNA_overlap	0	Lnc-CompleteIn-mRNAIntron	n335787	MTCONS_00063185	chr6	119312344	119312956	613	-	chr6	119280990	119399909	3894	-	*		0.9871	1	0.18	0.05	0.27	1.16	0.41	1.43
tran	58054314	NA	n379903	MTCONS_00067260	chr7	44059560	44065967	920	+	chr7	102120280	102252017	6534	-		-1692.2	1	1	0.12	0.14	0.31	0.57	0.73	1.97
cis_mRNA_up10k	712	NA	n379903	MTCONS_00066634	chr7	44059560	44065967	920	+	chr7	43998541	44058849	4268	-	*		0.7837	1	0.12	0.14	0.31	0.11	0.16	0.18
tran	NA	NA	n325852	MTCONS_00030556	chr10	131457098	131457689	495	+	chr17	15531265	15587618	2412	-		-468.52	-0.9755	-1	0.18	0.16	0.31	0.84	1.28	0
tran	283158	NA	n380553	MTCONS_00000464	chr1	16944751	16957401	1765	-	chr1	17240558	17299508	6328	+		-1455.48	-0.9837	-1	0.45	1.61	0	0.99	0.38	1.06
tran	291045	NA	n380553	MTCONS_00000470	chr1	16944751	16957401	1765	-	chr1	17248445	17299508	8543	+		-1457	-0.7159	-0.866	0.45	1.61	0	1.31	1.31	2.13
cis_mRNA_up10k	668	NA	n383188	MTCONS_00031250	chr17	44270939	44274080	513	+	chr17	44107264	44270272	5284	-	*		-0.9448	-1	2.69	1.06	0.97	0	0.45	0.72
cis_mRNA_overlap	0	AntiCompleteIn-mRNAIntron	n383188	MTCONS_00031258	chr17	44270939	44274080	513	+	chr17	44228558	44302867	3448	-	*		0.7132	1	2.69	1.06	0.97	3.61	2.98	0.9

cis_mRNA_overlap	0	Lnc-CompleteIn-mRNAIntron	n338550	MTCONS_00003544	chr1	228405269	228405735	467	+	chr1	228395861	228566579	25513	+	*	0.9997	1	0.56	0.81	0.36	1.39	2.28	0.61
cis_mRNA_overlap	0	Lnc-CompleteIn-mRNAIntron	n338550	MTCONS_00003540	chr1	228405269	228405735	467	+	chr1	228390713	228548952	31828	+	*	0.8963	0.866	0.56	0.81	0.36	0.08	0.09	0.08
cis_mRNA_overlap	0	mRNA	n337166	MTCONS_00001530	chr1	82418511	82418858	348	+	chr1	82266655	82462214	8715	+	*	-0.8985	-0.866	0.45	0.31	0.2	0.22	0.25	0.25
cis_mRNA_overlap	0	Lnc-AntiOverlap-mRNA	LTCONS_00028550	MTCONS_00030316	chr17	5015311	5018818	1536	+	chr17	5005757	5016033	8346	-	*	-0.9442	-1	1.99	1.62	1.77	2.38	3.18	2.62
cis_mRNA_overlap	0	mRNA	n408077	MTCONS_00015011	chr12	1100404	1605099	9321	+	chr12	1100697	1605387	9580	+	-4524.73	0.9461	1	1.63	1.18	2.05	1.27	0.84	2.77
cis_mRNA_overlap	0	mRNA	n408077	MTCONS_00015005	chr12	1100404	1605099	9321	+	chr12	1100395	1580100	3491	+	*	-0.9413	-1	1.63	1.18	2.05	0.18	1.06	0
cis_mRNA_overlap	0	mRNA	n408077	MTCONS_00015014	chr12	1100404	1605099	9321	+	chr12	1107541	1605387	9599	+	-4537.13	-0.9826	-1	1.63	1.18	2.05	1.32	2.99	0.54
cis_mRNA_overlap	0	Lnc-CompleteIn-mRNAIntron	n339072	MTCONS_00042296	chr2	198093723	198095300	1578	-	chr2	197851386	198175523	8559	-	*	-0.9344	-1	0.09	0.04	0.06	1.15	1.55	1.53
tran	NA	NA	n363424	MTCONS_00042514	chr1	67995313	67998012	408	+	chr2	220074444	220094364	9699	-	-308.79	-0.9583	-1	0.28	0	0.08	2.36	3.58	2.92
cis_mRNA_overlap	0	mRNA	n339810	MTCONS_00052430	chr4	3294762	3424259	3161	+	chr4	3295114	3442384	7095	+	-2215.98	0.9959	1	2.22	3.16	7.26	0.25	0.35	1.31
cis_mRNA_overlap	0	mRNA	n339810	MTCONS_00052427	chr4	3294762	3424259	3161	+	chr4	3294745	3455002	9751	+	-2472.96	0.9949	1	2.22	3.16	7.26	0.91	1.04	1.37
cis_mRNA_overlap	0	mRNA	n339810	MTCONS_00052429	chr4	3294762	3424259	3161	+	chr4	3294762	3451691	8401	+	-2474.18	0.7553	1	2.22	3.16	7.26	1.37	1.77	1.85
tran	NA	NA	n368305	MTCONS_00026277	chr5	72236631	72251181	588	-	chr16	30205627	30216563	5004	+	-392.97	-0.6895	-1	0.52	0.28	0.47	0.68	1.6	1.56
tran	NA	NA	LTCONS_00063776	MTCONS_00022628	chr6	27685776	27686189	414	.	chr14	74751789	74769767	8842	-	-252.02	-0.9783	-1	2.07	1.71	1.94	2.02	3.53	2.84
cis_mRNA_up10k	1947	NA	n383460	MTCONS_00034118	chr19	35085572	35166604	1998	-	chr19	35168550	35178704	4289	+	*	0.9902	1	0.14	0.05	0.02	1.8	1.01	0.91
cis_mRNA_dw20k	4928	NA	n333099	NM_000041_dup1	chr19	45417577	45418151	205	+	chr19	45409039	45412650	1180	+	*	-0.9998	-1	0.87	0.39	0.78	1.2	2.27	1.38
cis_mRNA_overlap	0	Lnc-CompleteIn-mRNAIntron	n340110	MTCONS_00010526	chr10	97152266	97152995	730	-	chr10	97071315	97321171	7420	-	*	0.6279	1	0.35	0.36	0.57	0.75	0.89	0.91
cis_mRNA_overlap	0	mRNA-AntiCompleteIn-LncIntron	n379409	MTCONS_00035746	chr19	15050246	16748905	1493	+	chr19	15530816	15560773	8795	-	*	-0.9558	-1	0.05	0.08	0.06	2.81	1.43	1.99
cis_mRNA_overlap	0	mRNA-AntiCompleteIn-LncIntron	n379409	MTCONS_00035740	chr19	15050246	16748905	1493	+	chr19	15490773	15529838	3761	-	*	0.9998	1	0.05	0.08	0.06	5.28	7.59	6.09
cis_mRNA_overlap	0	mRNA-AntiCompleteIn-LncIntron	n379409	MTCONS_00035747	chr19	15050246	16748905	1493	+	chr19	15530816	15560777	8400	-	*	0.8945	1	0.05	0.08	0.06	0	1.4	1.04
cis_mRNA_overlap	0	mRNA-AntiCompleteIn-LncIntron	n379409	MTCONS_00035739	chr19	15050246	16748905	1493	+	chr19	15484221	15529852	5526	-	*	0.9722	1	0.05	0.08	0.06	3.84	5.47	4.72
tran	28141897	NA	n385531	MTCONS_00071101	chr9	42669321	42714943	2231	+	chr9	70856839	70920000	12104	+	-1009.27	0.8277	1	1.89	1.97	2.4	0.5	0.77	0.89
cis_mRNA_up10k	382	NA	n376348	MTCONS_00033625	chr19	10762541	10764556	1895	-	chr19	10764937	10803102	6693	+	*	-0.8566	-1	0.16	0.44	0	3.02	2.82	4.17
cis_mRNA_up10k	382	NA	n376348	MTCONS_00033622	chr19	10762541	10764556	1895	-	chr19	10764937	10796526	4284	+	*	-0.9243	-1	0.16	0.44	0	2.24	1.34	4.42
cis_mRNA_overlap	0	mRNA	n408146	MTCONS_00006427	chr1	160185505	160232318	4183	-	chr1	160185476	160232432	4167	-	-2174.49	-0.955	-1	4.09	4.42	4.59	2.91	2.37	1.55
cis_mRNA_overlap	0	mRNA	n408146	MTCONS_00006425	chr1	160185505	160232318	4183	-	chr1	160185476	160232379	7491	-	-2493.08	0.9994	1	4.09	4.42	4.59	1.69	2.4	2.82
cis_mRNA_dw20k	15199	NA	n382141	MTCONS_00022112	chr14	23982558	24013485	2524	+	chr14	24028683	24048373	8044	-	*	0.9746	1	0.03	0.09	0.07	3.55	6.3	4.84
cis_mRNA_dw20k	15199	NA	n382141	MTCONS_00022116	chr14	23982558	24013485	2524	+	chr14	24028683	24048685	8371	-	*	-0.7795	-1	0.03	0.09	0.07	0.31	0.07	0.3
cis_mRNA_overlap	0	Lnc-AntiOverlap-mRNA	n339541	MTCONS_00045608	chr21	46926024	46929082	2908	-	chr21	46825054	46934632	6440	+	*	0.8555	1	0.15	0.09	0.07	0.52	0.4	0.07
tran	977191	NA	LTCONS_00005931	MTCONS_00002194	chr1	147574207	147578821	3331	-	chr1	148556011	148596267	5017	+	-1666.14	-0.9437	-1	3.48	5.27	4.36	4.57	4.23	4.3
tran	976538	NA	LTCONS_00005931	MTCONS_00002190	chr1	147574207	147578821	3331	-	chr1	148555358	148596267	5492	+	-1636.84	0.8708	0.866	3.48	5.27	4.36	0.27	0.36	0.27
cis_mRNA_overlap	0	Lnc-CompleteIn-mRNAIntron	n406943	MTCONS_00001794	chr1	104112026	104114008	1983	+	chr1	104068326	104122152	5138	+	*	0.9662	1	0.52	0.79	0.2	1.34	2.04	1.02
tran	3431744	NA	LTCONS_00027571	MTCONS_00027487	chr16	21854664	21855150	375	-	chr16	18411784	18422921	1802	-	-187.36	-0.8114	-1	0.87	0.78	2.91	2.84	3.33	2.54
tran	597560	NA	LTCONS_00027571	MTCONS_00026100	chr16	21854664	21855150	375	-	chr16	22452709	22547842	10437	+	-205.49	-0.9026	-1	0.87	0.78	2.91	1.7	1.96	1.4
cis_mRNA_overlap	0	Lnc-AntiOverlap-mRNA	LTCONS_00057062	MTCONS_00058811	chr5	158526963	158527384	422	+	chr5	158122910	158527345	5870	-	-812.4	0.852	0.866	0	0.08	0.08	4.98	5.18	5.4
cis_mRNA_up10k	135	NA	LTCONS_00057062	MTCONS_00058809	chr5	158526963	158527384	422	+	chr5	158122910	158526829	5285	-	*	-0.9025	-0.866	0	0.08	0.08	5.07	0.14	2.27
cis_mRNA_overlap	0	Lnc-AntiOverlap-mRNA	LTCONS_00057062	MTCONS_00058812	chr5	158526963	158527384	422	+	chr5	158122910	158527345	5828	-	-812.4	0.9721	0.866	0	0.08	0.08	8.43	12.07	11.18
cis_mRNA_up10k	135	NA	LTCONS_00057062	MTCONS_00058810	chr5	158526963	158527384	422	+	chr5	158122910	158526829	5381	-	*	-0.9884	-0.866	0	0.08	0.08	3.79	1.52	1.89
cis_mRNA_dw20k	6818	NA	n377982	MTCONS_00006427	chr1	160171989	160178659	1578	-	chr1	160185476	160232432	4167	-	*	-0.963	-1	0.13	0.14	0.2	2.91	2.37	1.55
cis_mRNA_dw20k	6818	NA	n377982	MTCONS_00006425	chr1	160171989	160178659	1578	-	chr1	160185476	160232379	7491	-	*	0.8586	1	0.13	0.14	0.2	1.69	2.4	2.82
cis_mRNA_dw20k	19867	NA	n340924	MTCONS_00030104	chr17	80420477	80422131	1655	+	chr17	80376219	80400611	2236	+	*	0.958	1	0.42	0.91	0.61	1.89	2.86	2
cis_mRNA_dw20k	19867	NA	n340924	MTCONS_00030100	chr17	80420477	80422131	1655	+	chr17	80375909	80400611	4570	+	*	0.8998	1	0.42	0.91	0.61	0.13	0.32	0.28
cis_mRNA_dw20k	19867	NA	n340924	MTCONS_00030105	chr17	80420477	80422131	1655	+	chr17	80376245	80400611	3607	+	*	0.9999	1	0.42	0.91	0.61	0.34	0.39	0.36
cis_mRNA_overlap	0	mRNA	n375478	MTCONS_00031441	chr17	56066405	56072211	4722	-	chr17	56066398	56084707	16943	-	-2358.01	0.9944	1	0.01	0.04	0	0.41	2.27	0.09
cis_mRNA_overlap	0	mRNA	n375478	MTCONS_00031437	chr17	56066405	56072211	4722	-	chr17	56066398	56084707	16946	-	-2358.01	-0.8355	-1	0.01	0.04	0	0.51	0.01	2.17
cis_mRNA_overlap	0	mRNA	n375478	MTCONS_00031440	chr17	56066405	56072211	4722	-	chr17	56066398	56084707	11419	-	-2278.5	0.6934	0.866	0.01	0.04	0	1.19	1.19	0.56
cis_mRNA_dw20k	17788	NA	n405510	MTCONS_00026276	chr16	30234350	30256932	3455	-	chr16	30205627	30216563	6546	+	*	0.9807	1	0.91	1.28	1.23	0.07	0.38	0.28
cis_mRNA_dw20k	17788	NA	n405510	MTCONS_00026277	chr16	30234350	30256932	3455	-	chr16	30205627	30216563	5004	+	*	0.9963	1	0.91	1.28	1.23	0.68	1.6	1.56
cis_mRNA_up10k	177	NA	n382906	NM_001089_dup1	chr16	2390923	2412667	1749	+	chr16	2325879	2390747	6623	-	*	0.9128	0.866	0.05	0.16	0.16	47.48	50.18	52.06
tran	NA	NA	n373063	MTCONS_00000103	chr12	76687753	76691515	406	+	chr1	1385069	1405538	3856	+	-262.87	-0.9999	-1	0.28	0.02	0.08	0.07	0.21	0.18
cis_mRNA_dw20k	4001	NA	n409262	MTCONS_00049349	chr3	124449213	124468119	6583	+	chr3	123752172	124445213	16237	+	*	-0.8529	-1	0.45	0.56	0.81	6.21	4.27	3.77
cis_mRNA_up10k	52	NA	LTCONS_00037078	MTCONS_00035491	chr19	9546295	9546637	343	.	chr19	9522659	9546244	3982	-	*	0.9568	1	1.15	0.1	0.31	0.4	0.25	0.32
cis_mRNA_up10k	243	NA	n384003	MTCONS_00045933	chr21	34144411	34170014	1188	+	chr21	34105977	34144169	8478	-	*	0.9966	1	0.27	0.24	0.45	1.92	1.84	3.26
cis_mRNA_up10k	44	NA	n379485	MTCONS_00028074	chr16	71496181	71499844	2793	+	chr16	71481500	71496138	3216	-	*	0.9757	1	0.1	0.19	0.21	0	0.43	0.7
cis_mRNA_up10k	48	NA	n379485	MTCONS_00028071	chr16	71496181	71499844	2793	+	chr16	71481500	71496134	3228	-	*	-0.9934	-1	0.1	0.19	0.21	1.7	0.65	0.22
cis_mRNA_up10k	44	NA	n379485	MTCONS_00028076	chr16	71496181	71499844	2793	+	chr16	71481500	71496138	2787	-	*	-0.7482	-1	0.1	0.19	0.21	2.43	2.25	1.31

cis_mRNA_up10k	44	NA	n379485	MTCONS_00028073	chr16	71496181	71499844	2793	+	chr16	71481500	71496138	3166	-	*	0.8941	1	0.1	0.19	0.21	0.94	2.01	3.6
tran	NA	NA	LTCONS_00020698	MTCONS_00003945	chr13	22202574	22202881	308	.	chr1	1592930	1681887	14284	-	-197.72	0.9764	1	0	0.12	0.23	0	0.05	0.07
tran	NA	NA	LTCONS_00020698	MTCONS_00003944	chr13	22202574	22202881	308	.	chr1	1592930	1678937	10207	-	-197.72	0.9071	1	0	0.12	0.23	1.3	1.69	1.72
cis_mRNA_overlap	0	mRNA	n386098	MTCONS_00023316	chr15	28878269	28886727	1946	+	chr15	28834591	28930558	5989	+	-1132.25	-0.7214	-1	0.53	1.19	1.1	1.67	1.01	1.56
cis_mRNA_dw20k	13020	NA	n334389	MTCONS_00059522	chr6	4078287	4078486	200	-	chr6	4021533	4065268	6805	+	*	0.9931	1	1.21	2.81	1.86	1.15	2.92	2.05
cis_mRNA_dw20k	13020	NA	n334389	MTCONS_00059521	chr6	4078287	4078486	200	-	chr6	4021533	4065268	5114	+	*	0.9883	1	1.21	2.81	1.86	2.7	6.01	3.59
cis_mRNA_overlap	0	Lnc-CompleteIn-mRNAIntron	n338832	MTCONS_00015016	chr12	1420758	1422780	2020	+	chr12	1110167	1580100	3443	+	*	-0.878	-1	0.14	0.25	0.17	6.74	5.13	5.59
cis_mRNA_up10k	4122	NA	n379792	MTCONS_00026182	chr16	28814097	28829149	1930	+	chr16	28833270	28848814	7574	+	*	0.977	1	0.97	1.71	1.17	0.97	1.33	0.99
cis_mRNA_up10k	624	NA	n379792	MTCONS_00026180	chr16	28814097	28829149	1930	+	chr16	28829772	28848814	10249	+	*	0.9531	1	0.97	1.71	1.17	0.37	0.61	0.5
tran	NA	NA	n387513	MTCONS_00071858	chr6	3267102	3267543	442	+	chr9	130965633	130989314	5488	+	-680.03	0.8106	1	15.89	23.17	22.78	3.63	5.85	4.45
cis_mRNA_dw20k	10632	NA	n343020	NM_001678_dup1	chr17	7571720	7590863	2586	-	chr17	7554254	7561089	3332	+	*	0.9363	1	1.24	1.46	1.07	1.15	1.6	1.1
cis_mRNA_dw20k	4271	NA	n372894	MTCONS_00015011	chr12	1609657	1613590	2244	+	chr12	1100697	1605387	9580	+	*	0.7528	1	0.07	0	0.08	1.27	0.84	2.77
cis_mRNA_dw20k	4271	NA	n372894	MTCONS_00015014	chr12	1609657	1613590	2244	+	chr12	1107541	1605387	9599	+	*	-0.9797	-1	0.07	0	0.08	1.32	2.99	0.54
cis_mRNA_overlap	0	Lnc-AntiOverlap-mRNA	LTCONS_00072545	MTCONS_00070897	chr9	34661799	34666208	3751	-	chr9	34646620	34661974	4578	+	*	0.8853	1	1.25	0.91	1.32	0.38	0.21	0.64
cis_mRNA_up10k	2177	NA	n369186	MTCONS_00059517	chr6	4016675	4019294	2368	-	chr6	4021470	4065268	6935	+	*	-0.9572	-1	1.15	1.07	1.04	0.3	0.52	0.77
cis_mRNA_up10k	2240	NA	n369186	MTCONS_00059520	chr6	4016675	4019294	2368	-	chr6	4021533	4065268	5119	+	*	-0.9608	-1	1.15	1.07	1.04	0	0.34	0.71
cis_mRNA_up10k	2211	NA	n369186	MTCONS_00059519	chr6	4016675	4019294	2368	-	chr6	4021504	4065268	5220	+	*	0.9991	1	1.15	1.07	1.04	3.37	1.24	0.59
cis_mRNA_up10k	2177	NA	n369186	MTCONS_00059518	chr6	4016675	4019294	2368	-	chr6	4021470	4065268	4742	+	*	-0.9989	-1	1.15	1.07	1.04	5.42	6.03	6.21
tran	NA	NA	n375755	MTCONS_00026094	chr17	66793605	66796516	1147	-	chr16	22445951	22518194	21000	+	-1079.66	-0.9172	-1	0.06	0.04	0.09	3.82	4.56	3.59
tran	NA	NA	n343085	MTCONS_00033625	chr2	47562454	47562653	200	+	chr19	10764937	10803102	6693	+	-153.59	0.893	1	6.17	4.14	7.7	3.02	2.82	4.17
cis_mRNA_overlap	0	Lnc-AntiOverlap-mRNA	n407082	MTCONS_00072153	chr9	139698379	139703300	2386	-	chr9	139690790	139735653	5626	+	*	-0.9992	-1	0.09	0.24	0.22	0.52	0.19	0.22
cis_mRNA_overlap	0	Lnc-AntiOverlap-mRNA	n407082	MTCONS_00072159	chr9	139698379	139703300	2386	-	chr9	139690790	139735653	5120	+	*	0.6191	1	0.09	0.24	0.22	0	0.42	0.01
tran	NA	NA	LTCONS_00073869	MTCONS_00071099	chrUn_gl000211	6316	35709	299	-	chr9	70856839	70890198	1157	+	-145.86	0.9638	1	0.9	1.04	1.32	0.67	0.75	1.8
tran	NA	NA	LTCONS_00073869	MTCONS_00071103	chrUn_gl000211	6316	35709	299	-	chr9	70856839	70920000	7940	+	-153.91	-0.9585	-1	0.9	1.04	1.32	1.07	1.06	0.87
tran	131620867	NA	n378046	MTCONS_00002195	chr1	16920057	16935145	488	-	chr1	148556011	148758311	4653	+	-304.38	0.9877	1	1.98	1.46	1.13	2.02	1.53	1.37
tran	3245797	NA	LTCONS_00027503	MTCONS_00027460	chr16	18494434	18496023	1590	-	chr16	15226560	15248638	3000	-	-1590.64	0.9876	1	0.76	1	1.15	0.74	0.85	0.97
tran	3448442	NA	LTCONS_00027503	MTCONS_00025973	chr16	18494434	18496023	1590	-	chr16	15004214	15045993	14649	+	-1595.28	0.9937	1	0.76	1	1.15	2.44	3.88	4.45
tran	3245797	NA	LTCONS_00027503	MTCONS_00027459	chr16	18494434	18496023	1590	-	chr16	15226560	15248638	4201	-	-1608.81	0.9929	1	0.76	1	1.15	0.73	1.33	1.56
tran	2053066	NA	LTCONS_00027503	MTCONS_00026002	chr16	18494434	18496023	1590	-	chr16	16402869	16441369	10176	+	-1632.56	0.9104	1	0.76	1	1.15	0.47	0.71	1.48
tran	2053066	NA	LTCONS_00027503	MTCONS_00026004	chr16	18494434	18496023	1590	-	chr16	16402869	16441369	8303	+	-1628.36	0.9361	1	0.76	1	1.15	0	0.06	0.2
tran	2049970	NA	LTCONS_00027503	MTCONS_00026007	chr16	18494434	18496023	1590	-	chr16	16403022	16444465	12506	+	-1626.62	0.9984	1	0.76	1	1.15	1.84	2.4	2.83
tran	NA	NA	n326280	MTCONS_00059974	chr1	197043410	197173162	930	-	chr6	28234788	28286363	24577	+	-606.38	-0.9671	-1	0.17	0	0.28	0.06	0.39	0
cis_mRNA_up10k	4390	NA	n342632	MTCONS_00057414	chr5	1599035	1634096	1033	-	chr5	1571958	1594646	6957	-	*	-0.6422	-1	2.68	1.46	1.58	0.71	2.32	0.86
tran	NA	NA	n380037	MTCONS_00000237	chr2	27505301	27526416	876	-	chr1	6640063	6649340	5957	+	-636.35	-0.9715	-0.866	0.08	0.04	0.04	0.55	2.21	1.8
cis_mRNA_overlap	0	AntiCompleteIn-mRNAIntron	n339712	MTCONS_00044732	chr20	45981305	45983377	1809	+	chr20	45837599	45986661	6675	-	*	-0.8299	-1	0.02	0.03	0.07	0.59	0.16	0
tran	NA	NA	LTCONS_00013841	MTCONS_00026094	chr11	58646457	58673215	5165	-	chr16	22445951	22518194	21000	+	-4324.31	0.6835	0.866	2.51	2.51	1.97	3.82	4.56	3.59
cis_mRNA_overlap	0	Lnc-CompleteIn-mRNAIntron	n385465	MTCONS_00070371	chr8	131307601	131308779	1179	-	chr8	131064254	131370830	6504	-	*	0.9948	1	0.77	0.6	0.74	14.47	9.5	13.11
tran	NA	NA	n324066	MTCONS_00027523	chr9	74676464	74678845	583	-	chr16	19712510	19729544	7301	-	-595.01	-0.9999	-1	0.06	0.17	0.27	1.77	1.34	0.93
tran	NA	NA	n324066	MTCONS_00027519	chr9	74676464	74678845	583	-	chr16	19712510	19729016	7445	-	-595.01	0.9532	1	0.06	0.17	0.27	0	1.57	1.96
cis_mRNA_dw20k	17026	NA	n386160	NM_001605_dup1	chr16	70263707	70269272	1210	-	chr16	70286297	70323412	3344	-	*	0.9801	1	0.7	0.21	0	45.27	40.89	40.37
tran	NA	NA	LTCONS_00033972	MTCONS_00057414	chr19	21579921	21601152	13623	+	chr5	1571958	1594646	6957	-	-7460.93	0.9597	1	0.15	1.3	0.57	0.71	2.32	0.86
tran	NA	NA	n325387	MTCONS_00057412	chr2	20127098	20170071	746	-	chr5	1571958	1586534	12677	-	-1057.26	-0.9876	-1	0.96	0.48	0.73	2.97	3.83	3.5
cis_mRNA_up10k	390	NA	n383369	MTCONS_00033627	chr19	10762539	10764548	2010	-	chr19	10764937	10803102	7237	+	*	0.9939	1	11.43	9.65	10.21	0.87	0.24	0.5
cis_mRNA_up10k	81	NA	n384485	NM_000671_dup1	chr4	100010011	100023944	754	+	chr4	99992129	100009931	2644	-	*	0.8816	1	0.85	0.11	0.9	69.32	65.03	73.99
tran	NA	NA	n371423	MTCONS_00071212	chrX	1780739	1781265	419	+	chr9	82186868	82348271	11300	+	-437.6	1	1	0.09	0.33	0	0.76	0.92	0.7
cis_mRNA_up10k	4046	NA	n406634	MTCONS_00002398	chr1	154947118	154951725	924	+	chr1	154955770	154965590	3033	+	*	-0.7056	-1	0.62	1.89	0.21	1.61	1.59	2.34
cis_mRNA_dw20k	20	NA	LTCONS_00016686	MTCONS_00018622	chr12	123459264	123467462	4600	+	chr12	123467481	123635402	7379	-	*	-0.9983	-1	1.12	1.04	1.42	1.08	1.18	0.53
cis_mRNA_dw20k	20	NA	LTCONS_00016686	MTCONS_00018621	chr12	123459264	123467462	4600	+	chr12	123467481	123635400	8506	-	*	0.9688	1	1.12	1.04	1.42	0.94	0.38	1.66
cis_mRNA_overlap	0	mRNA	n410015	MTCONS_00023318	chr15	28899588	28930410	2947	+	chr15	28834608	28930558	5759	+	*	0.912	1	1.62	1.96	1.31	1.34	1.42	0.84
cis_mRNA_overlap	0	mRNA	n410015	MTCONS_00023317	chr15	28899588	28930410	2947	+	chr15	28834608	28930558	6711	+	-1759.75	0.9909	1	1.62	1.96	1.31	0.91	1.41	0.63
cis_mRNA_dw20k	19103	NA	n383032	MTCONS_00027086	chr16	90055190	90067195	1277	+	chr16	90015151	90036088	6984	+	*	-0.8717	-1	0.32	0.09	0.13	0.85	1.34	1.03
tran	NA	NA	LTCONS_00000021	MTCONS_00026094	chr1	704025	735303	1352	+	chr16	22445951	22518194	21000	+	-683.93	0.6835	0.866	0.04	0.04	0	3.82	4.56	3.59
tran	86074830	NA	LTCONS_00000021	MTCONS_00005308	chr1	704025	735303	1352	+	chr1	86810132	86862025	9802	-	-1012.71	-0.903	-0.866	0.04	0.04	0	1.4	1.65	1.98
tran	131849361	NA	n381061	MTCONS_00002203	chr1	16888923	16890082	1160	-	chr1	148739442	148758311	3223	+	-718.62	-0.986	-1	2.27	1	0	0	0.17	0.24
cis_mRNA_up10k	94	NA	n410607	MTCONS_00041692	chr2	122407230	122409114	1799	+	chr2	122095352	122407137	10671	-	*	1	1	0.37	0.49	0.75	0	0.18	0.57
cis_mRNA_up10k	94	NA	n410607	MTCONS_00041693	chr2	122407230	122409114	1799	+	chr2	122095352	122407137	8156	-	*	0.9991	1	0.37	0.49	0.75	4.74	5.78	8.5
cis_mRNA_up10k	22	NA	n368817	MTCONS_00059517	chr6	4019077	4021449	209	-	chr6	4021470	4065268	6935	+	*	-0.847	-0.866	0.42	0.19	0.19	0.3	0.52	0.77
cis_mRNA_up10k	85	NA	n368817	MTCONS_00059520	chr6	4019077	4021449	209	-	chr6	4021533	4065268	5119	+	*	-0.8536	-0.866	0.42	0.19	0.19	0	0.34	0.71
cis_mRNA_up10k	85	NA	n368817	MTCONS_00059522	chr6	4019077	4021449	209	-	chr6	4021533	4065268	6805	+	*	-0.8709	-0.866	0.42	0.19	0.19	1.15	2.92	2.05
cis_mRNA_up10k	56	NA	n368817	MTCONS_00059519	chr6	4019077	4021449	209	-	chr6	4021504	4065268	5220	+	*	0.9747	0.866	0.42	0.19	0.19	3.37	1.24	0.59
cis_mRNA_up10k	22	NA	n368817	MTCONS_00059518	chr6	4019077	4021449	209	-	chr6	4021470	4065268	4742	+	*	-0.9761	-0.866	0.42	0.19	0.19	5.42	6.03	6.21
cis_mRNA_up10k	85	NA	n368817	MTCONS_00059521	chr6	4019077	4021449	209	-	chr6	4021533	4065268	5114	+	*	-0.7078	-0.866	0.42	0.19	0.19	2.7	6.01	3.59
cis_mRNA_overlap	0	Lnc-CompleteIn-mRNAIntron	n339394	MTCONS_00051030	chr3	71240669	71241371	703	-	chr3	71001290	71355136	9322	-	*	-0.8551	-0.866	0	0.09	0.09	0.27	0	0.14
cis_mRNA_dw20k	11093	NA	n377610	MTCONS_00059517	chr6	4076360	4079955	3596	+	chr6	4021470	4065268	6935	+	*	-0.9789	-1	0.36	0.34	0.29	0.3	0.52	0.77
cis_mRNA_dw20k	11093	NA	n377610	MTCONS_00059520	chr6	4076360	4079955	3596	+	chr6	4021533	4065268	5119	+	*	-0.9763	-1	0.36	0.34	0.29	0	0.34	0.71
cis_mRNA_dw20k	11093	NA	n377610	MTCONS_00059519	chr6	4076360	4079955	3596	+	chr6	4021504	4065268	5220	+	*	0.8573	1	0.36	0.34	0.29	3.37	1.24	0.59

cis_mRNA_dw20k	11093	NA	n377610	MTCONS_00059518	chr6	4076360	4079955	3596	+	chr6	4021470	4065268	4742	+	*	-0.8541	-1	0.36	0.34	0.29	5.42	6.03	6.21
tran	NA	NA	n383185	MTCONS_00000234	chr17	43295637	43299132	1556	-	chr1	6640063	6649340	7313	+	-1671.92	0.657	1	0.31	0.3	0.5	0.58	0	0.69
tran	NA	NA	n383185	MTCONS_00000235	chr17	43295637	43299132	1556	-	chr1	6640063	6649340	6555	+	-1671.92	-0.7155	-1	0.31	0.3	0.5	1.23	1.84	1.04
tran	NA	NA	n370268	MTCONS_00006576	chr8	23316673	23336893	376	+	chr1	168047875	168106991	9211	-	-397.04	0.9532	1	0.31	0.19	0.37	1.35	0.92	2
tran	NA	NA	n325817	MTCONS_00026094	chr12	9980445	9987361	979	-	chr16	22445951	22518194	21000	+	-787.62	-0.7469	-1	0.04	0.03	0.13	3.82	4.56	3.59
cis_mRNA_up10k	926	NA	n345322	MTCONS_00026943	chr16	85198653	85587773	14805	+	chr16	85588698	85710002	8662	+	*	0.8859	0.866	0.07	0.08	0.08	0.55	0.83	0.7
cis_mRNA_up10k	1459	NA	n345322	MTCONS_00026944	chr16	85198653	85587773	14805	+	chr16	85589231	85710002	8243	+	*	-0.9589	-0.866	0.07	0.08	0.08	0.39	0.22	0.15
cis_mRNA_overlap	0	mRNA	n345322	MTCONS_00026942	chr16	85198653	85587773	14805	+	chr16	85496443	85710002	7793	+	*	0.9526	0.866	0.07	0.08	0.08	0.23	0.65	0.84
cis_mRNA_up10k	15	NA	n339346	MTCONS_00027052	chr16	89748969	89752998	782	-	chr16	89753012	89762800	2666	+	*	0.9695	1	0.09	0.17	0.19	3.04	3.81	4.38
cis_mRNA_up10k	71	NA	n375994	MTCONS_00033241	chr18	77712626	77716325	3504	+	chr18	77659871	77712556	6635	-	*	-0.6775	-1	1.04	2.19	1.08	0.71	0.61	0.63
cis_mRNA_overlap	0	mRNA	n406650	MTCONS_00036773	chr19	52359056	52391189	938	-	chr19	52343169	52391006	4010	-	-500.03	-0.8573	-1	0.99	0.54	0.56	1.21	1.72	1.44
cis_mRNA_overlap	0	Lnc-AntiOverlap-mRNA	n377940	MTCONS_00003951	chr1	1656054	1663343	1808	+	chr1	1658732	1677500	7140	-	*	0.9904	1	0.1	0.16	0.14	5.61	6.84	6.28
cis_mRNA_overlap	0	AntiCompleteIn-mRNAIntron	n377940	MTCONS_00003942	chr1	1656054	1663343	1808	+	chr1	1592930	1677500	7291	-	-2388.43	0.788	1	0.1	0.16	0.14	5.98	11.09	6.27
cis_mRNA_overlap	0	Lnc-AntiOverlap-mRNA	n377940	MTCONS_00003952	chr1	1656054	1663343	1808	+	chr1	1658732	1678937	11788	-	-3840.29	0.9953	1	0.1	0.16	0.14	0.23	0.68	0.57
cis_mRNA_dw20k	2	NA	n337964	MTCONS_00046465	chr22	21353393	21356015	1941	-	chr22	21321447	21353392	7819	+	*	0.8976	1	0	0.27	0.84	0	0.21	0.29
tran	NA	NA	n376313	MTCONS_00025135	chr19	37168290	37177738	908	+	chr15	75647592	75744087	13925	-	-592.45	0.9369	1	0.29	0.8	0.56	2.36	3.13	3.02
cis_mRNA_dw20k	9113	NA	n384096	MTCONS_00046468	chr22	21362504	21368576	1952	-	chr22	21336558	21353392	4669	+	*	-0.8676	-1	1.79	1.12	1.64	8.2	10.24	9.6
cis_mRNA_up10k	75	NA	n382030	MTCONS_00020180	chr13	46626983	46675482	3218	+	chr13	46531334	46626909	5318	-	*	-0.8796	-1	1.64	0.25	1.39	8.49	9.34	9.02
cis_mRNA_up10k	90	NA	n382030	MTCONS_00020175	chr13	46626983	46675482	3218	+	chr13	46531334	46626894	5297	-	*	-0.9874	-1	1.64	0.25	1.39	3.27	4.95	3.29
tran	1156281	NA	n365816	MTCONS_00038437	chr2	96614276	96622618	313	-	chr2	97778898	97930710	5409	+	-189.57	-0.989	-1	0.4	0	0.15	1.76	2.26	2.14
tran	NA	NA	n369554	MTCONS_00057412	chr7	13141016	13142014	575	+	chr5	1571958	1586534	12677	-	-915.12	0.9661	1	0.05	0.23	0.12	2.97	3.83	3.5
cis_mRNA_overlap	0	Lnc-AntiOverlap-mRNA	n410948	MTCONS_00031460	chr17	56402811	56431088	2483	+	chr17	56378592	56406188	6710	-	*	0.9746	1	0.12	0.03	0	2.23	1.71	1.7
cis_mRNA_overlap	0	Lnc-AntiOverlap-mRNA	n410948	MTCONS_00031461	chr17	56402811	56431088	2483	+	chr17	56378592	56406191	6893	-	*	-0.7281	-1	0.12	0.03	0	4.22	4.33	6.2
cis_mRNA_overlap	0	mRNA	n410735	MTCONS_00017391	chr12	30862486	30907448	4399	-	chr12	30862388	30976321	8243	-	*	-0.9484	-1	3.89	2.9	3.17	0.68	1.38	0.99
cis_mRNA_overlap	0	mRNA	n410735	MTCONS_00017384	chr12	30862486	30907448	4399	-	chr12	30862388	30907833	4815	-	-1871.7	0.8471	1	3.89	2.9	3.17	1.42	1.07	1.34
cis_mRNA_overlap	0	mRNA	n410735	MTCONS_00017388	chr12	30862486	30907448	4399	-	chr12	30862388	30907888	5378	-	*	-0.9018	-1	3.89	2.9	3.17	2.21	3.65	2.69
tran	NA	NA	n386655	MTCONS_00029943	chr11	43918853	43921422	2570	+	chr17	75136995	75213183	5530	+	-1533.99	-0.9818	-1	0.58	0.35	0.23	0.29	0.53	0.78
tran	NA	NA	n386655	MTCONS_00029944	chr11	43918853	43921422	2570	+	chr17	75136995	75213183	5400	+	-1533.99	0.9418	1	0.58	0.35	0.23	0.14	0.09	0
tran	NA	NA	n386655	MTCONS_00029942	chr11	43918853	43921422	2570	+	chr17	75136995	75213183	4961	+	-1529.77	-0.9761	-1	0.58	0.35	0.23	2.36	3.51	4.83
cis_mRNA_overlap	0	Lnc-CompleteIn-mRNAIntron	n339970	MTCONS_00009569	chr10	13568765	13570185	1421	-	chr10	13482875	13570598	7728	-	*	0.9844	1	0.2	0.4	0.28	1.22	2.68	1.57
tran	NA	NA	n387043	MTCONS_00057412	chr17	1143726	1150972	942	-	chr5	1571958	1586534	12677	-	-1309.56	-0.8321	-1	0.13	0	0.12	2.97	3.83	3.5
tran	NA	NA	n375756	MTCONS_00037130	chr17	66805273	66820947	815	-	chr1_gl000191_random	31266	50281	11132	-	-402.95	-0.9475	-1	0.03	0.08	0	0.16	0	0.47
tran	NA	NA	n375756	MTCONS_00037133	chr17	66805273	66820947	815	-	chr1_gl000191_random	31266	50281	13961	-	-417.83	0.8305	1	0.03	0.08	0	3.12	3.19	2.36
tran	NA	NA	n339417	MTCONS_00036834	chr3	108895291	108896212	922	-	chr19	53407555	53421644	4538	-	-729.87	0.9522	0.866	0.04	0.04	0.03	1.6	1.65	1.49
cis_mRNA_up10k	622	NA	n340474	MTCONS_00030316	chr17	5016654	5018299	1646	+	chr17	5005757	5016033	8346	-	*	0.9822	1	0.67	1.74	1.17	2.38	3.18	2.62
cis_mRNA_overlap	0	mRNA	n324559	MTCONS_00059974	chr6	28237538	28245351	1628	+	chr6	28234788	28286363	24577	+	*	0.9992	1	0.99	1.2	0.94	0.06	0.39	0
tran	NA	NA	LTCONS_00039063	MTCONS_00047690	chr2	153574407	153619183	16236	+	chr22	32041675	32058206	498	-	-427.09	0.9747	1	0.84	0.89	1.04	0.33	0.36	1.85
cis_mRNA_overlap	0	Lnc-CompleteIn-mRNAIntron	n340710	MTCONS_00058811	chr5	158470618	158472793	2176	-	chr5	158122910	158527345	5870	-	*	0.8908	1	1.47	1.48	1.83	4.98	5.18	5.4
tran	NA	NA	n385788	MTCONS_00057414	chrX	74544124	74546232	2109	+	chr5	1571958	1594646	6957	-	-922.55	0.6616	1	0.83	0.99	0.97	0.71	2.32	0.86
tran	NA	NA	n385788	MTCONS_00057412	chrX	74544124	74546232	2109	+	chr5	1571958	1586534	12677	-	-991.34	0.9624	1	0.83	0.99	0.97	2.97	3.83	3.5
cis_mRNA_dw20k	3433	NA	LTCONS_00070908	MTCONS_00070897	chr9	34665406	34682348	1805	+	chr9	34646620	34661974	4578	+	*	-0.8681	-1	0.65	1.1	0.58	0.38	0.21	0.64
cis_mRNA_up10k	2240	NA	LTCONS_00061918	MTCONS_00059522	chr6	3999741	4019294	6007	-	chr6	4021533	4065268	6805	+	*	0.9995	1	0.38	0.63	0.5	1.15	2.92	2.05
cis_mRNA_up10k	2240	NA	LTCONS_00061918	MTCONS_00059521	chr6	3999741	4019294	6007	-	chr6	4021533	4065268	5114	+	*	0.9719	1	0.38	0.63	0.5	2.7	6.01	3.59
cis_mRNA_up10k	35	NA	LTCONS_00019251	MTCONS_00020180	chr13	46626943	46678982	7881	+	chr13	46531334	46626909	5318	-	*	0.9817	1	0.61	0.81	0.77	8.49	9.34	9.02
cis_mRNA_up10k	50	NA	LTCONS_00019251	MTCONS_00020175	chr13	46626943	46678982	7881	+	chr13	46531334	46626894	5297	-	*	0.6625	1	0.61	0.81	0.77	3.27	4.95	3.29
cis_mRNA_overlap	0	Lnc-AntiOverlap-mRNA	LTCONS_00016740	MTCONS_00018665	chr12	125396432	125398212	413	+	chr12	125396186	125399587	2134	-	-917.4	0.9924	1	0.09	0.24	0	11.06	13.12	9.14
cis_mRNA_overlap	0	mRNA	LTCONS_00028869	MTCONS_00028872	chr17	18601292	18607785	789	+	chr17	18601328	18639702	2763	+	-519.49	-0.9981	-1	2.9	4.1	6.34	1.64	1.24	0.65
cis_mRNA_overlap	0	mRNA	LTCONS_00028869	MTCONS_00028870	chr17	18601292	18607785	789	+	chr17	18601292	18639702	2701	+	-519.49	0.9751	1	2.9	4.1	6.34	0.3	0.41	1.1
cis_mRNA_overlap	0	mRNA	LTCONS_00028869	MTCONS_00028871	chr17	18601292	18607785	789	+	chr17	18601292	18639702	2452	+	-510.92	0.9833	1	2.9	4.1	6.34	0	0.29	0.57
tran	3013683	NA	LTCONS_00028869	MTCONS_00030549	chr17	18601292	18607785	789	+	chr17	15531265	15587610	2250	-	-505.54	0.9882	1	2.9	4.1	6.34	1.2	1.68	3.52
cis_mRNA_overlap	0	Lnc-CompleteIn-mRNAIntron	n338833	MTCONS_00015011	chr12	1526670	1529046	2354	+	chr12	1100697	1605387	9580	+	*	-0.8561	-1	0.09	0.21	0.04	1.27	0.84	2.77
cis_mRNA_overlap	0	Lnc-CompleteIn-mRNAIntron	n338833	MTCONS_00015005	chr12	1526670	1529046	2354	+	chr12	1100395	1580100	3491	+	*	0.9915	1	0.09	0.21	0.04	0.18	1.06	0
cis_mRNA_overlap	0	Lnc-CompleteIn-mRNAIntron	n338833	MTCONS_00015014	chr12	1526670	1529046	2354	+	chr12	1107541	1605387	9599	+	*	0.9996	1	0.09	0.21	0.04	1.32	2.99	0.54
cis_mRNA_dw20k	11249	NA	n371268	MTCONS_00071247	chr9	87653271	87655849	778	-	chr9	87283366	87642023	8936	+	*	-0.7505	-0.866	0.18	0.04	0.04	3.66	6.46	12.25
cis_mRNA_dw20k	11249	NA	n371268	MTCONS_00071250	chr9	87653271	87655849	778	-	chr9	87283389	87642023	8994	+	*	-0.7664	-0.866	0.18	0.04	0.04	2.12	2.52	3.27
cis_mRNA_dw20k	11249	NA	n371268	MTCONS_00071249	chr9	87653271	87655849	778	-	chr9	87283366	87642023	9434	+	*	-0.9636	-0.866	0.18	0.04	0.04	0	1.6	2.21
cis_mRNA_overlap	0	Lnc-AntiOverlap-mRNA	LTCONS_00044241	MTCONS_00043095	chr20	18251196	18287944	2296	-	chr20	18269121	18297640	2943	+	*	-0.6711	-0.866	0	0.01	0.01	2.24	2.01	1.2

cis_mRNA_overlap	0	Lnc-AntiOverlap-mRNA	LTCONS_00044241	MTCONS_00043099	chr20	18251196	18287944	2296	-	chr20	18276237	18297640	5207	+	*		0.9888	0.866	0	0.01	0.01	0.48	0.69	0.73
cis_mRNA_up10k	8952	NA	n338191	MTCONS_00048068	chr22	50922417	50924864	2447	+	chr22	50883309	50913466	8090	-	*		0.7506	1	0.01	0	0.12	0.65	0.39	0.74
cis_mRNA_up10k	8954	NA	n338191	MTCONS_00048065	chr22	50922417	50924864	2447	+	chr22	50883309	50913464	8166	-	*		-0.9742	-1	0.01	0	0.12	9.87	10.75	7.87
cis_mRNA_dw20k	16792	NA	n339769	NM_007100_dup1	chr4	647012	649434	1388	-	chr4	666225	668127	364	-	*		0.9097	1	1.23	2.1	1.63	56.29	61.61	60.8
tran	NA	NA	n373137	MTCONS_00026094	chr12	124558542	124559839	396	+	chr16	22445951	22518194	21000	+		-552.32	0.8847	1	0.48	0.55	0.34	3.82	4.56	3.59
tran	89443312	NA	n385523	MTCONS_00071858	chr9	41516046	41522322	1022	+	chr9	130965633	130989314	5488	+		-682.36	-0.9742	-1	0.6	0.09	0.52	3.63	5.85	4.45
cis_mRNA_dw20k	4729	NA	n370391	MTCONS_00069063	chr8	133865865	133867309	1091	+	chr8	133787604	133861137	6467	+	*		-0.9993	-1	0.13	0.03	0.09	0.3	0.6	0.43
tran	NA	NA	n342813	MTCONS_00057412	chr7	72693843	72698885	549	-	chr5	1571958	1586534	12677	-		-665.37	-0.9092	-1	2.6	1.71	2.39	2.97	3.83	3.5
cis_mRNA_dw20k	262	NA	LTCONS_00030559	NM_000676_dup1	chr17	15879471	15899465	4660	-	chr17	15848231	15879210	1885	+	*		-0.9847	-0.866	0.53	0.75	0.8	0.09	0.05	0.05
tran	NA	NA	LTCONS_00038175	MTCONS_00047690	chr2	74345615	74355819	10205	+	chr22	32041675	32058206	498	-		-462.18	-0.8784	-1	2.9	1.84	0.75	0.33	0.36	1.85
cis_mRNA_dw20k	10225	NA	LTCONS_00038175	MTCONS_00038172	chr2	74345615	74355819	10205	+	chr2	74212780	74335391	10093	+	*		0.9613	1	2.9	1.84	0.75	1.71	1.51	1.44
cis_mRNA_up10k	1017	NA	n384209	MTCONS_00048204	chr3	9391374	9438338	3252	-	chr3	9439354	9519901	8247	+	*		0.8483	1	1.3	1.78	1.47	1.48	2.11	2.02
cis_mRNA_up10k	1017	NA	n384209	MTCONS_00048203	chr3	9391374	9438338	3252	-	chr3	9439354	9519901	7357	+	*		-0.9724	-1	1.3	1.78	1.47	1.73	1.35	1.68
cis_mRNA_up10k	1017	NA	n384209	MTCONS_00048205	chr3	9391374	9438338	3252	-	chr3	9439354	9519901	7795	+	*		0.9819	1	1.3	1.78	1.47	0	2.67	1.39
cis_mRNA_up10k	1013	NA	n384209	MTCONS_00048198	chr3	9391374	9438338	3252	-	chr3	9439350	9519901	9023	+	*		0.9437	1	1.3	1.78	1.47	0.66	1.55	0.68
cis_mRNA_up10k	1013	NA	n384209	MTCONS_00048199	chr3	9391374	9438338	3252	-	chr3	9439350	9519901	7232	+	*		-0.9905	-1	1.3	1.78	1.47	1.19	0.97	1.14
cis_mRNA_up10k	552	NA	n364545	MTCONS_00041697	chr2	122407688	122409113	1340	+	chr2	122159576	122407137	5666	-	*		0.9968	1	2.22	2.23	1.89	1.36	1.39	1.13
tran	NA	NA	n370977	MTCONS_00052761	chr9	95432687	95435900	1022	+	chr4	40097159	40169531	19264	+		-730.43	-0.9986	-1	0.62	0.47	0.65	2.88	4.06	2.73
tran	NA	NA	n370977	MTCONS_00052756	chr9	95432687	95435900	1022	+	chr4	40057780	40169531	19726	+		-730.43	-0.9118	-1	0.62	0.47	0.65	0.51	0.65	0.34
cis_mRNA_up10k	286	NA	n370540	MTCONS_00068883	chr8	104493481	104511722	3020	-	chr8	104512007	105267094	8765	+	*		0.9331	1	0.34	0.22	0.45	3.51	3.05	5.46
tran	NA	NA	n323954	MTCONS_00024457	chr9	131292919	131304614	605	+	chr15	23281011	23378337	7754	-		-410.76	1	1	0.35	0.41	0.44	0	1.64	2.44
cis_mRNA_up10k	4376	NA	n346143	MTCONS_00057417	chr5	1599053	1634050	2160	-	chr5	1574556	1594678	11153	-	*		-0.8064	-1	0.19	0.28	0.53	1.14	0.84	0.78
cis_mRNA_up10k	4376	NA	n346143	MTCONS_00057415	chr5	1599053	1634050	2160	-	chr5	1571958	1594678	1490	-	*		-0.9983	-1	0.19	0.28	0.53	1.96	1.74	1.27
tran	NA	NA	n342320	MTCONS_00062598	chr14	50329254	50329627	374	-	chr6	43004263	43027309	7588	-		-332.14	-0.922	-1	87866.47	74580.45	88036.16	0.14	0.23	0.08
tran	NA	NA	n338881	MTCONS_00022629	chr2	73988396	73988908	513	-	chr14	74751789	74769767	5311	-		-563.25	0.822	1	0.58	0.73	0.52	0.39	0.46	0.05
cis_mRNA_dw20k	12931	NA	n375319	MTCONS_00026944	chr16	85722932	85733297	1507	-	chr16	85589231	85710002	8243	+	*		-0.7251	-0.866	0.02	0.02	0.08	0.39	0.22	0.15
cis_mRNA_dw20k	12931	NA	n375319	MTCONS_00026939	chr16	85722932	85733297	1507	-	chr16	85203298	85710002	9937	+	*		-0.8694	-0.866	0.02	0.02	0.08	1.85	2.27	1.42
cis_mRNA_dw20k	12931	NA	n375319	MTCONS_00026942	chr16	85722932	85733297	1507	-	chr16	85496443	85710002	7793	+	*		0.7399	0.866	0.02	0.02	0.08	0.23	0.65	0.84
cis_mRNA_dw20k	3433	NA	LTCONS_00070909	MTCONS_00070896	chr9	34665406	34682348	1978	+	chr9	34646573	34661974	5657	+	*		0.9847	1	1.31	1.7	1.63	5.05	6.8	6.2
cis_mRNA_overlap	0	mRNA	n333473	MTCONS_00026484	chr16	53241944	53256701	792	+	chr16	53164987	53364300	14530	+	*		0.9561	1	1.57	1.79	1.37	0.11	0.3	0.06
cis_mRNA_up10k	6162	NA	n332606	MTCONS_00030865	chr17	34897565	34900681	1382	-	chr17	34851504	34891404	5790	-	*		0.8154	1	0.5	0.25	0.52	0.46	0.19	0.94
cis_mRNA_up10k	6162	NA	n332606	MTCONS_00030866	chr17	34897565	34900681	1382	-	chr17	34851504	34891404	5923	-	*		-0.9997	-1	0.5	0.25	0.52	0.65	2.76	0.54
cis_mRNA_overlap	0	Lnc-AntiOverlap-mRNA	LTCONS_00037011	MTCONS_00035110	chr19	58370253	58400442	407	-	chr19	58330978	58380311	10514	+		-384.57	0.9864	1	0.91	0.86	0.63	0.09	0.07	0.03
cis_mRNA_overlap	0	Lnc-AntiOverlap-mRNA	LTCONS_00037011	MTCONS_00035108	chr19	58370253	58400442	407	-	chr19	58330634	58380311	10914	+	*		-0.9933	-1	0.91	0.86	0.63	0.13	0.14	0.3
tran	NA	NA	n326734	MTCONS_00036834	chr1	1353365	1355498	1210	-	chr19	53407555	53421644	4538	-		-685.85	0.9982	1	0.26	0.35	0	1.6	1.65	1.49
cis_mRNA_overlap	0	mRNA	n334024	MTCONS_00035238	chr19	1612197	1619134	395	-	chr19	1609230	1652896	3307	-		-381.28	0.9512	1	0.31	0.1	0	1.93	0.83	0.82
tran	NA	NA	n384325	MTCONS_00057412	chr3	129612715	129627754	3495	+	chr5	1571958	1586534	12677	-		-1709.48	0.9995	1	0.63	0.91	0.81	2.97	3.83	3.5
tran	NA	NA	n325586	MTCONS_00005308	chr16	31624492	31626173	723	+	chr1	86810132	86862025	9802	-		-290.1	0.9182	1	1.05	1.06	1.29	1.4	1.65	1.98
cis_mRNA_overlap	0	Lnc-CompleteIn-mRNAIntron	n325109	MTCONS_00042650	chr2	234438881	234440744	1864	-	chr2	234383819	234475421	8561	-	*		-0.9384	-0.866	0.22	0.25	0.25	3.14	1.8	2.27
cis_mRNA_overlap	0	Lnc-CompleteIn-mRNAIntron	n325109	MTCONS_00042649	chr2	234438881	234440744	1864	-	chr2	234383819	234475420	5700	-	*		-0.8171	-0.866	0.22	0.25	0.25	2.35	1.78	2.11
cis_mRNA_overlap	0	Lnc-CompleteIn-mRNAIntron	n325109	MTCONS_00042648	chr2	234438881	234440744	1864	-	chr2	234383819	234475415	10399	-	*		-0.6934	-0.866	0.22	0.25	0.25	0.33	0.29	0.17
cis_mRNA_dw20k	2901	NA	n405951	MTCONS_00027086	chr16	90038988	90063028	3168	+	chr16	90015151	90036088	6984	+	*		0.9768	1	1.25	1.7	1.5	0.85	1.34	1.03
cis_mRNA_dw20k	19988	NA	n339246	MTCONS_00048200	chr3	9539888	9541253	1223	-	chr3	9439350	9519901	7913	+	*		0.9962	1	3.34	2	1.67	1.78	0.32	0.14
tran	58144302	NA	n346040	MTCONS_00067263	chr7	44001284	44027449	960	-	chr7	102171750	102213068	8121	-		-778.69	0.9984	1	0.05	0.29	0	3.11	4.57	2.68
tran	58246262	NA	n346040	MTCONS_00067271	chr7	44001284	44027449	960	-	chr7	102273710	102312182	5303	-		-770.37	-0.7744	-1	0.05	0.29	0	2.57	2.14	4.19
cis_mRNA_overlap	0	mRNA	LTCONS_00047379	NM_001670_dup1	chr22	19985279	20009331	5165	-	chr22	19957402	20004309	4036	-	*		0.8648	1	0	0.23	0.15	11.11	12.43	11.35
cis_mRNA_overlap	0	mRNA	n386029	MTCONS_00014995	chr12	1011722	1016190	4469	+	chr12	861708	1021255	11428	+	*		0.7233	1	0.35	0.68	0.67	0.38	1.4	0.65
cis_mRNA_overlap	0	mRNA	n386029	MTCONS_00014993	chr12	1011722	1016190	4469	+	chr12	861708	1021255	15207	+		-2175.58	0.6393	1	0.35	0.68	0.67	0.61	0.87	0.65
cis_mRNA_overlap	0	mRNA	n386029	MTCONS_00014990	chr12	1011722	1016190	4469	+	chr12	861697	1021255	11508	+	*		0.7897	1	0.35	0.68	0.67	0.55	0.83	0.65
tran	NA	NA	n324195	MTCONS_00071858	chr7	115998442	116041973	468	-	chr9	130965633	130989314	5488	+		-639.5	0.997	1	0.16	0.46	0.25	3.63	5.85	4.45
cis_mRNA_overlap	0	Lnc-CompleteIn-mRNAIntron	LTCONS_00069682	MTCONS_00069678	chr8	42382332	42396651	8741	-	chr8	42273985	42396777	3791	-	*		0.8971	1	4.98	4.62	5.41	1.37	0.41	1.51
cis_mRNA_overlap	0	Lnc-CompleteIn-mRNAExon	n342783	NM_000019_dup1	chr11	107992258	108018275	1517	+	chr11	107992258	108018895	2138	+	*		-0.9955	-1	44.15	37.1	51.93	2.52	3.34	1.87
tran	3725623	NA	n381233	MTCONS_00002194	chr1	144828973	144830389	1417	+	chr1	148556011	148596267	5017	+		-797.24	0.8139	1	11.89	10.51	11.56	4.57	4.23	4.3
tran	3724970	NA	n381233	MTCONS_00002190	chr1	144828973	144830389	1417	+	chr1	148555358	148596267	5492	+		-797.24	-0.9734	-0.866	11.89	10.51	11.56	0.27	0.36	0.27
cis_mRNA_overlap	0	mRNA	n410095	MTCONS_00071442	chr9	100000708	100059596	3218	+	chr9	100000708	100139577	6379	+		-1792.76	-1	-1	0.79	0.48	0.26	0.25	0.58	0.82
cis_mRNA_overlap	0	CompleteIn-LncExon	n410095	MTCONS_00071439	chr9	100000708	100059596	3218	+	chr9	100000708	100059596	3098	+		-1494.42	-0.9762	-1	0.79	0.48	0.26	0.41	0.56	0.79
cis_mRNA_dw20k	6054	NA	n338562	MTCONS_00007397	chr1	234510108	234520769	1329	+	chr1	234526822	234614937	9527	-	*		-0.866	-1	0.33	0.35	0.32	0.11	0	0.55
cis_mRNA_dw20k	6054	NA	n338562	MTCONS_00007393	chr1	234510108	234520769	1329	+	chr1	234526822	234614934	9792	-	*		0.9992	1	0.33	0.35	0.32	1.56	2.23	1.17
cis_mRNA_dw20k	6054	NA	n338562	MTCONS_00007395	chr1	234510108	234520769	1329	+	chr1	234526822	234614937	9891	-	*		0.9924	1	0.33	0.35	0.32	1.34	1.63	1.11
cis_mRNA_dw20k	6054	NA	n338562	MTCONS_00007396	chr1	234510108	234520769	1329	+	chr1	234526822	234614937	8693	-	*		0.9499	1	0.33	0.35	0.32	0.41	0.62	0.08
tran	NA	NA	n345115	MTCONS_00026094	chr10	47051926	47052306	381	+	chr16	22445951	22518194	21000	+		-227.36	0.9955	1	12.05	14.37	11.66	3.82	4.56	3.59
cis_mRNA_dw20k	11608	NA	n375401	NM_001116_dup1	chr16	3980005	4001043	2605	-	chr16	4012650	4166186	7727	-	*		1	1	0.27	0.32	0.29	5.09	6.17	5.52
tran	NA	NA	LTCONS_00028400	MTCONS_00052416	chr16	4325104	4325525	422	.	chr4	2965178	3042475	6358	+		-360.46	0.9678	1	0.18	1.32	0.32	1.41	1.74	1.53

cis_mRNA_overlap	0	AntiCompleteIn-mRNAIntron	n324818	MTCONS_00045906	chr21	32687310	32688140	831	+	chr21	32490734	32716501	7145	-	*		-0.6078	-0.866	1.83	1.9	1.4	3.43	2.55	3.43
cis_mRNA_overlap	0	AntiCompleteIn-mRNAIntron	n324818	MTCONS_00045907	chr21	32687310	32688140	831	+	chr21	32490734	32719412	7716	-	*		-0.9999	-1	1.83	1.9	1.4	0.17	0.11	0.5
cis_mRNA_dw20k	2856	NA	n387676	MTCONS_00068764	chr8	94752339	94753047	709	+	chr8	94712691	94749484	14768	+	*		0.9979	0.866	0	0.09	0.09	0.49	0.62	0.63
cis_mRNA_dw20k	2856	NA	n387676	MTCONS_00068761	chr8	94752339	94753047	709	+	chr8	94712686	94749484	11773	+	*		-1	-1	0	0.09	0.09	0.04	0.02	0.02
cis_mRNA_dw20k	2856	NA	n387676	MTCONS_00068763	chr8	94752339	94753047	709	+	chr8	94712691	94749484	10038	+	*		-0.9608	-0.866	0	0.09	0.09	0.54	0.44	0.4
cis_mRNA_dw20k	19338	NA	n339368	MTCONS_00050862	chr3	52529371	52558509	7909	+	chr3	52577846	52719612	9627	-	*		-0.9953	-1	0.01	0.05	0.06	3.32	3.15	3.08
cis_mRNA_dw20k	19338	NA	n339368	MTCONS_00050865	chr3	52529371	52558509	7909	+	chr3	52577846	52719956	9513	-	*		-0.9882	-1	0.01	0.05	0.06	0.89	0.66	0.54
cis_mRNA_up10k	488	NA	n369881	MTCONS_00067823	chr7	154795169	154797375	1954	+	chr7	154735400	154794682	3712	-	*		0.9997	1	0.35	0.12	0.24	1.31	1.13	1.22
tran	1173943	NA	n342433	MTCONS_00038435	chr2	96602559	96604956	2398	-	chr2	97778898	97930710	9268	+		-935.78	-0.9752	-1	4.68	5.73	5.85	7.65	7.2	6.99
cis_mRNA_up10k	1714	NA	n339808	MTCONS_00052414	chr4	2938993	2963465	1616	+	chr4	2965178	3042475	2862	+	*		0.9892	1	0.24	0.32	0.03	2.4	2.63	0.91
cis_mRNA_up10k	1714	NA	n339808	MTCONS_00052415	chr4	2938993	2963465	1616	+	chr4	2965178	3042475	1793	+	*		0.9975	1	0.24	0.32	0.03	1.15	1.46	0.55
cis_mRNA_overlap	0	Lnc-CompleteIn-mRNAIntron	n408157	MTCONS_00049352	chr3	123813558	123883533	2410	+	chr3	123752180	124445213	16268	+	*		-0.7118	-1	1.27	0.34	1.14	2.75	7.9	7.13
cis_mRNA_overlap	0	Lnc-CompleteIn-mRNAIntron	n408157	MTCONS_00049350	chr3	123813558	123883533	2410	+	chr3	123752172	124445213	16273	+	*		0.8886	1	1.27	0.34	1.14	0.25	0.04	0.13
cis_mRNA_overlap	0	Lnc-CompleteIn-mRNAIntron	n408157	MTCONS_00049351	chr3	123813558	123883533	2410	+	chr3	123752180	124445213	16226	+	*		0.9687	1	1.27	0.34	1.14	7.25	7.17	7.22
cis_mRNA_overlap	0	Lnc-AntiOverlap-mRNA	n410075	MTCONS_00030101	chr17	80400463	80408707	2077	-	chr17	80375909	80400611	3446	+	*		0.9656	1	1.01	0	1.6	0.76	0.32	0.82
tran	58137717	NA	n378636	MTCONS_00067268	chr7	44079122	44081905	2590	+	chr7	102219621	102251963	7267	-		-4075.2	0.9798	1	7.79	12.63	11.31	0.51	0.92	0.89
cis_mRNA_dw20k	2462	NA	n381037	MTCONS_00003942	chr1	1586823	1590469	335	-	chr1	1592930	1677500	7291	-	*		-0.7977	-1	3.83	1.96	2.61	5.98	11.09	6.27
cis_mRNA_overlap	0	AntiCompleteIn-mRNAIntron	n345503	MTCONS_00038956	chr2	134883534	134965157	10381	-	chr2	134877422	135212270	8782	+	*		0.8812	1	0.09	0.2	0.15	1.97	2.62	2.03
cis_mRNA_overlap	0	AntiCompleteIn-mRNAIntron	n345503	MTCONS_00038954	chr2	134883534	134965157	10381	-	chr2	134877422	135212270	8658	+	*		0.9407	1	0.09	0.2	0.15	1.77	2.73	2.6
cis_mRNA_up10k	6554	NA	LTCONS_00067267	MTCONS_00067257	chr7	102219621	102224650	4919	-	chr7	102113453	102213068	1770	-	*		0.9903	1	0.65	1.6	1.19	1.69	2.45	2.03
cis_mRNA_overlap	0	mRNA	LTCONS_00067267	MTCONS_00067268	chr7	102219621	102224650	4919	-	chr7	102219621	102251963	7267	-		-3764.43	0.929	1	0.65	1.6	1.19	0.51	0.92	0.89
cis_mRNA_overlap	0	Lnc-CompleteIn-mRNAIntron	LTCONS_00067267	MTCONS_00067261	chr7	102219621	102224650	4919	-	chr7	102120280	102257205	6445	-		-3439.18	0.9689	1	0.65	1.6	1.19	0.91	2.28	1.39
tran	16745771	NA	n380201	MTCONS_00070565	chr8	128808279	128903229	844	+	chr8	145648999	145669812	6734	-		-947.04	0.9739	1	0.57	1.6	1.49	2.43	3.47	3.13
cis_mRNA_overlap	0	Lnc-CompleteIn-mRNAExon	n340697	MTCONS_00057031	chr5	154135135	154183768	2630	+	chr5	154135007	154197215	7161	+		-1676.57	-0.993	-1	2.07	3	4.01	10.07	4.97	1.31
cis_mRNA_up10k	73	NA	n377426	NM_005160_dup1	chr22	25957850	25960789	2332	+	chr22	25960861	26125259	9056	+	*		0.9538	1	0	0.02	0.01	26.1	34.64	28.04
cis_mRNA_up10k	9231	NA	n334153	MTCONS_00059517	chr6	4012036	4012240	205	-	chr6	4021470	4065268	6935	+	*		-0.997	-1	0.43	0.2	0	0.3	0.52	0.77
cis_mRNA_up10k	9294	NA	n334153	MTCONS_00059520	chr6	4012036	4012240	205	-	chr6	4021533	4065268	5119	+	*		-0.9979	-1	0.43	0.2	0	0	0.34	0.71
cis_mRNA_up10k	9265	NA	n334153	MTCONS_00059519	chr6	4012036	4012240	205	-	chr6	4021504	4065268	5220	+	*		0.9669	1	0.43	0.2	0	3.37	1.24	0.59
cis_mRNA_up10k	9231	NA	n334153	MTCONS_00059518	chr6	4012036	4012240	205	-	chr6	4021470	4065268	4742	+	*		-0.9653	-1	0.43	0.2	0	5.42	6.03	6.21
cis_mRNA_overlap	0	mRNA	n340690	NM_004045_dup1	chr5	151121877	151131987	4341	-	chr5	151122383	151138210	489	-	*		-0.9384	-1	0.4	0.24	0.34	33.46	39.16	37.36
cis_mRNA_up10k	124	NA	LTCONS_00045037	MTCONS_00044064	chr20	3154347	3157613	3267	.	chr20	3143263	3154224	4781	-	*		-0.9533	-1	0.75	0.83	0.59	0.01	0	0.33
cis_mRNA_up10k	103	NA	LTCONS_00045037	MTCONS_00044065	chr20	3154347	3157613	3267	.	chr20	3143263	3154245	4246	-	*		0.8078	1	0.75	0.83	0.59	6.61	8.19	6.45
cis_mRNA_overlap	0	Lnc-AntiOverlap-mRNA	n338221	MTCONS_00022113	chr14	24030306	24037282	3243	+	chr14	24028683	24048373	7641	-	*		-0.9452	-1	0.09	0.14	0.2	1.74	1.6	0.8
cis_mRNA_overlap	0	Lnc-AntiOverlap-mRNA	n338221	MTCONS_00022115	chr14	24030306	24037282	3243	+	chr14	24028683	24048442	7987	-	*		0.9942	1	0.09	0.14	0.2	0.52	1.66	2.6
cis_mRNA_overlap	0	Lnc-AntiOverlap-mRNA	n340765	MTCONS_00031439	chr17	56084050	56085165	1116	+	chr17	56066398	56084707	13635	-		-1144.91	-0.9959	-1	1.76	1.46	1.71	2.32	3.83	2.43
cis_mRNA_overlap	0	Lnc-AntiOverlap-mRNA	n340765	MTCONS_00031438	chr17	56084050	56085165	1116	+	chr17	56066398	56084707	15778	-		-1144.91	-0.9587	-1	1.76	1.46	1.71	0.3	0.81	0.52
tran	NA	NA	n324051	MTCONS_00026276	chr9	90814769	90815213	445	-	chr16	30205627	30216563	6546	+		-502.76	-0.766	-1	0.32	0	0.31	0.07	0.38	0.28
cis_mRNA_up10k	23	NA	n379921	MTCONS_00053779	chr4	183064331	183065041	554	-	chr4	183065063	183724267	11238	+	*		0.9781	1	0.58	0.14	0.88	2.06	1.26	3.2
cis_mRNA_dw20k	4754	NA	LTCONS_00038439	MTCONS_00038437	chr2	97935463	97958212	16109	+	chr2	97778898	97930710	5409	+	*		0.9185	1	5.84	7.84	6.64	1.76	2.26	2.14
cis_mRNA_up10k	7623	NA	n342881	MTCONS_00072168	chr9	139702413	139735634	2746	-	chr9	139743256	139756563	7103	+	*		0.9966	1	0.46	0.41	0.51	0.11	0.08	0.15
cis_mRNA_overlap	0	AntiCompleteIn-mRNAExon	n342881	MTCONS_00072155	chr9	139702413	139735634	2746	-	chr9	139690790	139735653	5389	+	*		0.9516	1	0.46	0.41	0.51	0.63	0.5	1.09
cis_mRNA_overlap	0	AntiCompleteIn-mRNAExon	n342881	MTCONS_00072157	chr9	139702413	139735634	2746	-	chr9	139690790	139735653	5811	+		-6672.11	0.9487	1	0.46	0.41	0.51	1.52	0.96	1.67
cis_mRNA_up10k	4043	NA	n374817	NM_000057_dup1	chr15	91221600	91256537	386	-	chr15	91260579	91358686	4528	+	*		0.906	1	0.4	0.36	0.27	0.49	0.4	0.36
cis_mRNA_overlap	0	mRNA	n335198	NM_000912_dup1	chr8	54163578	54164257	317	-	chr8	54138276	54164194	4959	-	*		1	1	0.42	0.38	0.74	30.27	26.5	62.27
cis_mRNA_up10k	120	NA	n363904	NM_001839_dup1	chr1	95392854	95433017	1635	+	chr1	95362507	95392735	2066	-	*		0.9838	1	0.05	0.1	0	47.36	51.4	39.59
cis_mRNA_up10k	680	NA	LTCONS_00062790	NM_001704_dup1	chr6	69339825	69344953	5023	-	chr6	69345632	70099403	5637	+	*		0.9985	1	0.45	1.25	1.78	3.8	5.14	5.86
cis_mRNA_dw20k	14987	NA	n341057	NM_002496_dup1	chr11	67819100	67821228	2129	+	chr11	67798084	67804114	804	+	*		0.9965	1	0.16	0.31	0.17	50.69	61.13	52.3
cis_mRNA_up10k	7193	NA	n409697	NM_005282_dup1	chr19	46112658	46142675	1974	-	chr19	46093022	46105466	3048	-	*		-0.9135	-0.866	0.22	0	0.13	0.03	0.08	0.03
cis_mRNA_dw20k	493	NA	n338963	NM_002410_dup1	chr2	135206960	135212192	5231	+	chr2	135011830	135206468	2421	+	*		0.8449	1	7.93	9.09	7.51	14	14.83	11.25
cis_mRNA_dw20k	12127	NA	LTCONS_00019252	NM_002298_dup1	chr13	46626959	46687932	2413	+	chr13	46700058	46756459	3790	-	*		-0.9999	-1	2.91	2.98	2.45	0.54	0.5	0.83
cis_mRNA_dw20k	7697	NA	n5382	NM_002073_dup1	chr22	23474917	23475187	271	-	chr22	23412669	23467221	3208	+	*		-0.9329	-1	0.3	0.42	0.14	42.25	42.02	44.86
cis_mRNA_up10k	9640	NA	n379407	NM_001299_dup1	chr19	11637552	11639940	2276	+	chr19	11649579	11661138	1586	+	*		0.8985	1	0.02	0.03	0.04	0.28	0.4	0.41
cis_mRNA_dw20k	18052	NA	LTCONS_00059120	NM_002202_dup1	chr5	50708615	50710674	2060	.	chr5	50678958	50690564	2718	+	*		-0.8251	-1	1.47	1.23	1.14	157.32	168.18	214.48
cis_mRNA_overlap	0	Lnc-AntiOverlap-mRNA	n379808	NM_001992_dup1	chr5	75987264	76012040	600	-	chr5	76011868	76031595	3810	+	*		-0.9671	-1	0	0.06	0.05	15.21	11.92	13.25
cis_mRNA_up10k	2712	NA	n380543	NM_002386_dup1	chr16	89978527	89981576	2464	+	chr16	89984287	89987385	3099	+	*		0.9256	1	1.36	2.48	1.97	3.32	4.4	4.3

cis_mRNA_overlap	0	Lnc-AntiOverlap-mRNA	n407967	NM_001212_dup1	chr17	5322961	5336340	1615	+	chr17	5336099	5342471	1163	-	*		-0.7873	-1	0.86	1.14	0.11	94.23	79.64	96.35
cis_mRNA_overlap	0	Lnc-AntiOverlap-mRNA	n407967	NM_002532_dup1	chr17	5322961	5336340	1615	+	chr17	5289346	5323059	2495	-	*		0.9638	1	0.86	1.14	0.11	15.93	16.66	15.23
cis_mRNA_overlap	0	mRNA	LTCONS_00072556	NM_004629_dup1	chr9	35073825	35080023	2622	-	chr9	35073835	35080013	2631	-		-1730.48	-0.985	-1	0.64	1.66	1.22	4.5	3.16	3.94
cis_mRNA_dw20k	2930	NA	n341948	NM_002076_dup1	chr12	65103238	65104293	1056	+	chr12	65107222	65153226	5128	-	*		0.9888	1	0.03	0.26	0.17	17.49	22.34	19.81
cis_mRNA_dw20k	9315	NA	n364037	NM_000254_dup1	chr1	237076595	237085551	1130	+	chr1	236958581	237067281	10558	+	*		0.89	1	0.02	0.03	0.09	5.96	6.2	6.38
cis_mRNA_overlap	0	mRNA-AntiCompleteIn-LncIntron	n341074	NM_001293_dup1	chr11	77184416	77467427	3683	+	chr11	77327196	77348851	1373	-	*		0.9814	1	0.09	0.07	0.05	40.54	39.77	38.21
cis_mRNA_overlap	0	other	n337940	NM_002370_dup1	chr1	53704282	53708455	1181	+	chr1	53692564	53704282	756	-	*		-0.8436	-1	0.15	0.16	0.37	8.75	7.92	7.3
cis_mRNA_overlap	0	mRNA	LTCONS_00059410	NM_000904_dup1	chr6	2988201	3018206	3975	+	chr6	3000067	3019994	1139	+	*		-0.6423	-0.866	0.17	0.18	0.18	10.31	10.17	9.55
cis_mRNA_dw20k	191	NA	n345604	NM_002268_dup1	chr3	160212796	160217772	4163	-	chr3	160217962	160283376	3802	-	*		0.9993	1	1.17	1.3	1.04	23.81	25.43	22.39
cis_mRNA_dw20k	4008	NA	LTCONS_00030940	NM_005937_dup1	chr17	36890063	36908470	7190	-	chr17	36861873	36886056	7500	+	*		0.7206	1	0.6	0.85	0.87	9	9.4	10.79
cis_mRNA_up10k	3585	NA	n409369	NM_002477_dup1	chr4	666225	668127	310	-	chr4	671711	675817	661	+	*		-0.9857	-1	12.64	10.73	17.61	1.82	1.99	0.48
tran	NA	NA	LTCONS_00063839	NM_001320_dup1	chr6_cox_hap2	3146839	3147525	360	+	chr6_ssto_hap7	2964462	2968648	1128	+		-208.49	-0.8589	-1	20.96	15.89	13.39	48.3	49	51.99
cis_mRNA_dw20k	7910	NA	n380646	NM_004558_dup1	chr19	5836244	5839733	2701	+	chr19	5823818	5828335	1104	+	*		0.803	1	0.33	0.45	0.44	0	0.09	0.03
cis_mRNA_up10k	5443	NA	n364173	NM_001938_dup1	chr1	93792165	93806036	2186	-	chr1	93811478	93828149	3209	+	*		-0.7687	-1	0.76	0	0.62	24.26	26.9	26.4
cis_mRNA_dw20k	10478	NA	n325842	NM_005539_dup1	chr10	134607461	134628918	2619	-	chr10	134351353	134596984	2938	+	*		0.6384	1	0.09	0.2	0.07	15.37	15.43	13.36
cis_mRNA_dw20k	457	NA	n384393	NM_005017_dup1	chr3	195961240	195964785	3546	-	chr3	195965241	196014584	1589	-	*		0.9798	1	2.86	3.2	3.61	3.86	4.34	5.61
cis_mRNA_overlap	0	Lnc-AntiOverlap-mRNA	n383814	NM_000091_dup1	chr2	228092486	228189880	1956	-	chr2	228029281	228179508	8097	+	*		0.7664	0.866	0.51	0.43	0.66	0.03	0.02	0.03
cis_mRNA_dw20k	8143	NA	n365108	NM_000183_dup1	chr2	26521475	26529191	2513	+	chr2	26467616	26513333	2188	+	*		0.9153	1	0.07	0	0.06	16.09	13.86	14.93
cis_mRNA_up10k	133	NA	n373843	NM_002010_dup1	chr13	22225659	22245083	494	-	chr13	22245215	22278640	4530	+	*		-0.7113	-1	1.44	1.45	1.15	1.93	1.44	2.09
cis_mRNA_overlap	0	Lnc-CompleteIn-mRNAIntron	n382172	NM_002028_dup1	chr14	65453948	65455990	2043	+	chr14	65453507	65529370	2819	+	*		0.7114	1	0.57	0.6	0.75	6.99	7.64	7.73
cis_mRNA_dw20k	14649	NA	n338129	NM_002232_dup1	chr1	111197952	111199662	1711	-	chr1	111214310	111217655	3346	-	*		0.9946	1	0.33	0.89	0.17	0.36	0.95	0.28
cis_mRNA_up10k	6366	NA	LTCONS_00047364	NM_000407_dup1	chr22	19702311	19704701	2391	-	chr22	19711066	19712297	958	+	*		0.9873	0.866	0	0.03	0.03	0	6.41	5.32
cis_mRNA_dw20k	15243	NA	n345778	NM_002199_dup1	chr4	185286377	185293634	2002	-	chr4	185308876	185395726	2286	-	*		-0.9984	-0.866	0.02	0.02	0	2.68	2.63	3.42
cis_mRNA_up10k	614	NA	n372244	NM_001380_dup1	chr10	128591477	128593410	1817	-	chr10	128594023	129250780	6751	+	*		0.9926	1	0.23	0.66	0.1	0.5	1.38	0.39
cis_mRNA_dw20k	5269	NA	n406622	NM_006185_dup1	chr11	71639768	71708643	2568	+	chr11	71713911	71791573	7182	-	*		0.9685	1	1.29	2.78	1.14	19.49	22.04	18.21
cis_mRNA_dw20k	762	NA	n341717	NM_005226_dup1	chr9	91620686	91625315	4630	-	chr9	91606362	91619925	4206	+	*		-0.8938	-1	3.11	1.26	1.6	0.02	0.07	0.04
cis_mRNA_dw20k	3782	NA	n370586	NM_138465_dup1	chr8	144362882	144363898	533	-	chr8	144349607	144359101	1343	+	*		-0.9918	-1	0.81	0.97	0.72	1.72	1.15	2.23
cis_mRNA_up10k	607	NA	LTCONS_00005667	NM_006135_dup1	chr1	113153070	113161469	1240	-	chr1	113162075	113214241	2740	+	*		0.9934	1	1.69	1.72	1.67	62.36	64.42	60.3
cis_mRNA_dw20k	9683	NA	n372731	NM_004561_dup1	chr11	65574367	65585604	762	-	chr11	65554529	65564685	2993	+	*		-0.9995	-1	0.61	0.86	0.17	0.01	0	0.03
cis_mRNA_overlap	0	Lnc-AntiOverlap-mRNA	n342855	NM_001539_dup1	chr9	33025348	33039905	2241	-	chr9	33025209	33039062	1538	+	*		-0.8219	-1	0	0.03	0.01	327.74	286.81	291.62
cis_mRNA_dw20k	2801	NA	n383633	NM_000251_dup1	chr2	47713160	47715691	2000	-	chr2	47630263	47710360	3145	+	*		0.9235	0.866	0.07	0.02	0.02	34.19	31.4	29.64
cis_mRNA_overlap	0	mRNA	n323786	NM_005334_dup1	chrX	153215504	153216581	341	-	chrX	153213008	153236819	8426	-	*		-0.976	-1	0.7	0	0.21	8.19	9.83	9.69
cis_mRNA_overlap	0	Lnc-CompleteIn-mRNAIntron	n338263	NM_052847_dup1	chr19	2610153	2611860	1707	-	chr19	2511218	2702746	4246	-	*		0.9888	0.866	0.02	0.12	0.02	0.77	1.02	0.81
cis_mRNA_overlap	0	Lnc-AntiOverlap-mRNA	n339468	NM_004487_dup1	chr3	121468132	121470030	1899	+	chr3	121382048	121468602	11185	-	*		0.9517	1	0.43	0.66	0.76	4.97	5.19	5.49
cis_mRNA_overlap	0	mRNA	n410579	NM_000904_dup1	chr6	3001363	3023034	2360	+	chr6	3000067	3019994	1139	+	*		-0.6423	-0.866	0.03	0.09	0.09	10.31	10.17	9.55
cis_mRNA_up10k	151	NA	n340589	NM_004475_dup1	chr17	27224865	27225801	937	+	chr17	27206357	27224715	2632	-	*		0.9861	1	0.04	0.03	0.17	38.89	36.86	45.36
cis_mRNA_dw20k	9880	NA	n345976	NM_014213_dup1	chr2	176999524	177001878	789	-	chr2	176987413	176989645	1885	+	*		0.7819	1	0.35	0.32	0	0.22	0.08	0.02
cis_mRNA_up10k	5750	NA	LTCONS_00067182	NM_001185_dup1	chr7	99579484	99588629	1334	-	chr7	99564350	99573735	1265	-	*		-0.9996	-1	1.25	0.78	1.05	0	0.05	0.02
cis_mRNA_dw20k	338	NA	n387060	NM_002067_dup1	chr19	3121807	3123994	2188	+	chr19	3094408	3121470	1615	+	*		0.8846	1	17.84	17.22	18.41	16.57	16.35	19.49
cis_mRNA_dw20k	7303	NA	LTCONS_00025416	NM_006122_dup1	chr15	91473504	91476295	2384	-	chr15	91447420	91466202	6279	+	*		-0.9974	-0.866	1.35	1.1	1.12	4.9	5.45	5.45
cis_mRNA_dw20k	223	NA	n364426	NM_005373_dup1	chr1	43820357	43823559	407	-	chr1	43803475	43820135	3645	+	*		-0.6934	-0.866	0	0.06	0.08	0.03	0.03	0
cis_mRNA_up10k	5424	NA	n364172	NM_001938_dup1	chr1	93791325	93806055	2278	-	chr1	93811478	93828149	3209	+	*		0.6685	1	0.91	1.21	0.92	24.26	26.9	26.4
cis_mRNA_overlap	0	Lnc-AntiOverlap-mRNA	n409277	NM_000904_dup1	chr6	2997616	3013741	2270	-	chr6	3000067	3019994	1139	+	*		0.9748	1	0.47	0.38	0.24	10.31	10.17	9.55
cis_mRNA_overlap	0	Lnc-CompleteIn-mRNAIntron	n342197	NM_006148_dup1	chr17	37071930	37073090	1161	+	chr17	37026112	37078023	4109	+	*		0.9394	1	0.42	0.59	0.86	17.07	17.31	21.99
cis_mRNA_overlap	0	Lnc-CompleteIn-mRNAIntron	n341766	NM_002581_dup1	chr9	118935545	118937821	2277	+	chr9	118916071	119164600	10970	+	*		0.9952	1	0.14	0.04	0.39	17.46	10.62	28.91
cis_mRNA_overlap	0	Lnc-AntiOverlap-mRNA	n342529	NM_001236_dup1	chr21	37504065	37528605	1499	-	chr21	37507263	37518860	1112	+	*		0.8386	0.866	0.02	0.06	0.06	0.03	0.14	0.08
cis_mRNA_up10k	8933	NA	n325581	NM_002616_dup1	chr17	8064685	8068086	2082	-	chr17	8043788	8055753	4709	-	*		-0.998	-1	0.37	0.33	0.42	3.67	3.95	3.23
cis_mRNA_dw20k	16860	NA	LTCONS_00014946	NM_005528_dup1	chr11	64018612	64018921	310	.	chr11	63997753	64001753	1285	+	*		0.9696	1	0.65	0.71	1.04	2.63	3.21	4.14
cis_mRNA_up10k	4882	NA	n366150	NM_004443_dup1	chr3	184264502	184274706	571	-	chr3	184279587	184300196	4220	+	*		-0.7342	-1	1.05	1.91	1.2	6.08	3.84	4.17
cis_mRNA_dw20k	1052	NA	n384140	NM_004147_dup1	chr22	31831223	31834552	3330	+	chr22	31795539	31830172	1450	+	*		0.9963	1	5.38	5.14	5.21	48.45	43.86	44.83
cis_mRNA_dw20k	17718	NA	n339043	NM_001761_dup1	chr16	2526576	2527062	487	+	chr16	2479395	2508859	4287	+	*		0.7781	1	1.85	2.52	2.54	6.12	6.54	7.35
cis_mRNA_overlap	0	mRNA	n333074	NM_001415_dup1	chrX	24096519	24096950	432	+	chrX	24073065	24096927	3465	+	*		0.8403	1	0.4	0.91	0	55.48	55.65	48.3
cis_mRNA_up10k	9900	NA	n407057	NM_001299_dup1	chr19	11616744	11639680	1574	-	chr19	11649579	11661138	1586	+	*		-0.9927	-1	0.45	0.29	0.25	0.28	0.4	0.41
cis_mRNA_dw20k	14697	NA	n345250	NM_005249_dup1	chr14	29253567	29265164	2519	-	chr14	29236287	29238871	2585	+	*		-0.9926	-1	2.54	3	2.91	0.14	0	0.01
cis_mRNA_up10k	152	NA	n340627	NM_002715_dup1	chr5	133562101	133563505	1405	+	chr5	133532148	133561950	2643	-	*		0.9999	1	0.64	0.54	0.46	101.1	96.29	92.63
cis_mRNA_dw20k	9503	NA	n408257	NM_004545_dup1	chr14	92524896	92572965	6837	-	chr14	92582467	92588153	420	-	*		0.8611	1	0.24	0	0.26	119.7	108.62	134.3
cis_mRNA_overlap	0	Lnc-AntiOverlap-mRNA	n410698	NM_012202_dup1	chr11	62457734	62477091	2096	-	chr11	62475114	62476678	903	+	*		0.9962	1	0.36	0.19	0.04	69.06	67.1	65.83
cis_mRNA_dw20k	1810	NA	n375484	NM_001303_dup1	chr17	14113805	14121239	1537	+	chr17	13972719	14111996	3002	+	*		0.9954	1	0.65	0.93	0.83	4.05	5.64	5.21

cis_mRNA_overlap	0	Lnc-CompleteIn-mRNAIntron	n342388	NM_001410_dup1	chr19	42836016	42836379	364	+	chr19	42829761	42882921	10966	+	*		-0.9982	-0.866	0.67	0.1	0.1	0.51	1.07	1.11
cis_mRNA_dw20k	13387	NA	LTCONS_00025593	NM_004378_dup1	chr15	78653958	78657576	3619	.	chr15	78632666	78640572	772	+	*		0.8564	1	0.94	0.46	0.27	519.33	487.26	382.44
cis_mRNA_overlap	0	Lnc-AntiOverlap-mRNA	n407804	NM_001481_dup1	chr16	90109310	90114191	1391	-	chr16	90089008	90111379	3185	+	*		0.852	1	0	0.26	0.17	0.3	1.81	0.54
cis_mRNA_dw20k	153	NA	LTCONS_00002595	NM_012394_dup1	chr1	161068130	161070194	1540	+	chr1	161070346	161087866	642	-	*		-0.9516	-1	1.3	2.88	2.09	33.86	29.91	30.78
cis_mRNA_up10k	50	NA	LTCONS_00047082	NM_002490_dup1	chr22	42486937	42532702	2097	+	chr22	42481530	42486888	1184	-	*		-0.7445	-1	1.54	2.1	2.16	34.89	33.43	28.72
cis_mRNA_overlap	0	AntiCompleteIn-mRNAIntron	LTCONS_00042733	NM_002081_dup1	chr2	241392855	241395468	2614	-	chr2	241375115	241407495	3686	+	*		0.9969	0.866	0	0.06	0.06	30.46	33.18	33.44
cis_mRNA_dw20k	1020	NA	n380161	NM_006896_dup1	chr7	27179983	27192316	687	+	chr7	27193335	27196296	2018	-	*		0.9972	0.866	0.28	0.28	0	0.12	0.11	0
cis_mRNA_overlap	0	mRNA-AntiCompleteIn-LncIntron	n380161	NM_024014_dup1	chr7	27179983	27192316	687	+	chr7	27185202	27187393	802	-	*		0.7695	0.866	0.28	0.28	0	1.03	0.57	0.32
cis_mRNA_overlap	0	mRNA-AntiCompleteIn-LncIntron	n380161	NM_019102_dup1	chr7	27179983	27192316	687	+	chr7	27180671	27183287	1657	-	*		0.9832	0.866	0.28	0.28	0	128.47	116.76	68
cis_mRNA_up10k	9585	NA	n380161	NM_002141_dup1	chr7	27179983	27192316	687	+	chr7	27168126	27170399	1728	-	*		0.9964	0.866	0.28	0.28	0	9.4	9.17	6.96
cis_mRNA_up10k	8830	NA	n407391	NM_001032392_dup1	chr2	87257798	87303536	2201	-	chr2	87237587	87248969	3011	-	*		-0.923	-1	2.58	2.37	3.88	0.14	0.23	0.05
cis_mRNA_up10k	2655	NA	n340567	NM_001270_dup1	chr5	98264892	98266713	1822	+	chr5	98190908	98262238	6457	-	*		0.9985	1	2.14	2.33	1.69	8.76	8.92	8.27
cis_mRNA_up10k	298	NA	n363610	NM_001255_dup1	chr1	43820355	43824329	661	-	chr1	43824626	43828874	1675	+	*		1	1	0.05	0.12	0	0.08	0.19	0
cis_mRNA_dw20k	221	NA	n363610	NM_005373_dup1	chr1	43820355	43824329	661	-	chr1	43803475	43820135	3645	+	*		0.8142	0.866	0.05	0.12	0	0.03	0.03	0
cis_mRNA_up10k	2917	NA	n376923	NM_001958_dup1	chr20	62133421	62137060	778	+	chr20	62119366	62130505	1841	-	*		-0.6653	-0.866	0.18	0.08	0.08	119.25	142.89	124.3
cis_mRNA_dw20k	8243	NA	n342869	NM_005226_dup1	chr9	91628167	91793555	2160	-	chr9	91606362	91619925	4206	+	*		0.9978	1	0	0.44	0.15	0.02	0.07	0.04
cis_mRNA_overlap	0	AntiCompleteIn-mRNAIntron	LTCONS_00005670	NM_006135_dup1	chr1	113164315	113194788	1598	-	chr1	113162075	113214241	2740	+	*		-0.9608	-1	0.21	0.15	0.23	62.36	64.42	60.3
cis_mRNA_overlap	0	AntiCompleteIn-mRNAIntron	n342452	NM_004482_dup1	chr2	166624403	166624724	316	+	chr2	166604313	166650803	3267	-	*		0.907	1	0.66	1.27	1.31	0.01	0.1	0.18
cis_mRNA_up10k	8566	NA	LTCONS_00028856	NM_002018_dup1	chr17	18170620	18172142	1499	+	chr17	18148150	18162055	4163	-	*		0.9723	1	0.49	0.86	0.85	14.72	18.82	17.73
cis_mRNA_dw20k	17720	NA	LTCONS_00014943	NM_002556_dup1	chr11	59323905	59324152	248	.	chr11	59341871	59383617	5083	-	*		-0.9583	-1	4.42	2.63	0.91	6.1	6.35	7.09
cis_mRNA_dw20k	882	NA	n383944	NM_001794_dup1	chr20	60513180	60515672	2493	+	chr20	59827559	60512299	3060	+	*		1	1	3.66	5.18	2.87	4.75	7.01	3.55
cis_mRNA_up10k	331	NA	n368118	NM_004365_dup1	chr5	89705933	89712311	3347	+	chr5	89689528	89705603	982	-	*		0.9937	1	0.21	0.25	0.2	13.1	14.42	12.52
cis_mRNA_dw20k	18575	NA	n336826	NM_004938_dup1	chr9	90342123	90342970	351	+	chr9	90112756	90323549	5938	+	*		0.758	1	0.45	0.21	0.42	27.54	22.72	23.83
cis_mRNA_overlap	0	Lnc-AntiOverlap-mRNA	n342703	NM_000413_dup1	chr17	40704456	40706766	2311	-	chr17	40703984	40707232	2231	+	*		0.9998	1	5.39	6.63	5.76	0.41	0.63	0.48
tran	216210	NA	LTCONS_00046631	NM_000496_dup1	chr22	25844045	25857644	2741	+	chr22	25615612	25627836	763	+		-534.29	-0.8561	-0.866	1.35	2.24	1.78	0.05	0.04	0.04
cis_mRNA_dw20k	1415	NA	n338398	NM_001249_dup1	chr14	74429933	74431758	1826	-	chr14	74433172	74486026	2057	-	*		0.9999	1	1.21	1.56	1.35	1.4	1.57	1.47
cis_mRNA_up10k	2561	NA	n377216	NM_006134_dup1	chr21	34854841	34856673	560	-	chr21	34821448	34852281	2332	-	*		-0.6654	-0.866	0.09	0.26	0.26	14.73	11.41	14.02
cis_mRNA_up10k	686	NA	n375882	NM_005603_dup1	chr18	55471012	55474049	2870	+	chr18	55313658	55470327	5938	-	*		-1	-1	0.05	0.05	0.09	0.01	0.01	0
cis_mRNA_dw20k	13888	NA	n363829	NM_004964_dup1	chr1	32813111	32814381	406	+	chr1	32757708	32799224	2091	+	*		-0.9618	-1	0.28	0.09	0.42	7.03	8	5.09
cis_mRNA_dw20k	1959	NA	n384937	NM_000287_dup1	chr6	42929295	42929645	351	-	chr6	42931603	42946981	3503	-	*		-0.9184	-1	2.28	1.87	2.94	5.93	6.88	5.6
cis_mRNA_dw20k	18582	NA	n338905	NM_004533_dup1	chr19	50988164	50990120	1957	+	chr19	50936160	50969583	3598	+	*		0.8856	0.866	0.91	2.05	1.44	0.01	0.03	0.01
cis_mRNA_dw20k	1703	NA	n384640	NM_181501_dup1	chr5	52251187	52255037	3851	+	chr5	52084136	52249485	4796	+	*		1	1	0.12	0.22	0.2	0.56	1.21	1.08
cis_mRNA_up10k	9467	NA	n411617	NM_001757_dup1	chr21	37406841	37432819	3251	-	chr21	37442285	37445462	1245	+	*		-0.9826	-1	1.76	2.58	2.12	9.15	7.67	8.26
cis_mRNA_dw20k	2889	NA	n338803	NM_006184_dup1	chr19	49429428	49429701	274	+	chr19	49403307	49426540	2579	+	*		0.9944	1	0.15	1.5	0.67	28.79	35.32	31.91
cis_mRNA_up10k	704	NA	n380069	NM_024007_dup1	chr5	158527491	158542331	691	+	chr5	158122923	158526788	5274	-	*		0.7142	1	1.15	0.48	1.22	16.97	16.26	19.88
cis_mRNA_dw20k	11152	NA	n409087	NM_004052_dup1	chr10	133747960	133770053	1836	+	chr10	133781204	133795435	1534	-	*		0.9442	1	1.22	2.6	1.12	38.89	39.44	38.54
cis_mRNA_up10k	6548	NA	n363643	NM_001938_dup1	chr1	93770668	93804931	600	-	chr1	93811478	93828149	3209	+	*		-0.7415	-1	0.07	0	0.06	24.26	26.9	26.4
tran	NA	NA	LTCONS_00064001	NM_005510_dup1	chr6_qbl_hap6	3231774	3232430	657	-	chr6_ssto_hap7	3270298	3272742	1590	-		-507.44	0.8352	1	1.75	2.29	2.5	6.04	6.15	6.59
cis_mRNA_up10k	853	NA	n375723	NM_003204_dup1	chr17	46124254	46124834	343	-	chr17	46125686	46138907	4874	+	*		0.999	1	0.92	1.05	0.41	58.54	62.28	47.05
cis_mRNA_up10k	136	NA	n373511	NM_002567_dup1	chr12	118555475	118573735	477	-	chr12	118573870	118583390	1491	+	*		0.9918	1	1.42	0.87	1.41	425.12	351.86	413.77
cis_mRNA_dw20k	257	NA	n335595	NM_001567_dup1	chr11	71950462	71951269	808	-	chr11	71935882	71950206	4725	+	*		0.9996	1	0	0.34	0.67	6.27	7.7	9.24
cis_mRNA_dw20k	1645	NA	n341310	NM_005302_dup1	chr7	124382366	124384470	2104	-	chr7	124386114	124405681	2958	-	*		0.9779	1	0.02	0.04	0.06	0.55	0.92	1.09
cis_mRNA_dw20k	3027	NA	LTCONS_00020702	NM_002128_dup1	chr13	31028655	31029851	1197	.	chr13	31032877	31040081	3428	-	*		0.9701	0.866	0.06	0.03	0.03	87.11	77.6	74.39
cis_mRNA_overlap	0	Lnc-CompleteIn-mRNAIntron	n335499	NM_004155_dup1	chr6	2892877	2893088	212	-	chr6	2887504	2903545	4145	-	*		-0.9856	-1	0.42	0.38	0.74	24.24	25.78	20.22
cis_mRNA_dw20k	2206	NA	n325514	NM_000554_dup1	chr19	48349271	48356854	500	+	chr19	48325099	48347066	4482	+	*		0.9787	1	5.07	7.15	5.61	2.24	2.42	2.32
cis_mRNA_overlap	0	AntiCompleteIn-mRNAIntron	n385230	NM_000722_dup1	chr7	81638493	81659271	2651	+	chr7	81579418	82073031	3822	-	*		0.8793	0.866	0	0.01	0.01	4.61	7.8	6.28
cis_mRNA_overlap	0	Lnc-AntiOverlap-mRNA	n387059	NM_001280_dup1	chr19	1267471	1270259	1355	-	chr19	1269267	1273171	1397	+	*		0.9128	1	0.05	0.02	0	182.34	172.83	140.39
cis_mRNA_dw20k	10023	NA	n339982	NM_000812_dup1	chr4	47438484	47440426	1940	+	chr4	47033295	47428462	2226	+	*		0.9935	1	0.04	0	0.28	0.02	0	0.97
cis_mRNA_overlap	0	Lnc-CompleteIn-mRNAExon	n410437	NM_004927_dup1	chr11	64889716	64894841	1950	+	chr11	64889716	64894841	2101	+	*		0.9517	1	1.02	0.96	0	20.23	19.58	18.43
tran	NA	NA	LTCONS_00071082	NM_004477_dup1	chr9	68726485	68767301	858	+	chr4	190861974	190884359	1043	+		-334.32	-0.9894	-1	1.49	1.64	0.35	30.28	29.1	33.66
cis_mRNA_up10k	4730	NA	n406211	NM_005952_dup1	chr16	56709649	56711653	2005	+	chr16	56716382	56718108	404	+	*		0.9784	1	0	0.05	0.06	1.05	1.9	2.38
cis_mRNA_dw20k	10700	NA	n346069	NM_004155_dup1	chr6	2854300	2876805	1989	-	chr6	2887504	2903545	4145	-	*		-0.8612	-1	0.03	0	0.04	24.24	25.78	20.22
cis_mRNA_up10k	137	NA	n367536	NM_002199_dup1	chr4	185395862	185398943	1321	+	chr4	185308876	185395726	2286	-	*		0.8529	1	0.04	0	0.07	2.68	2.63	3.42
cis_mRNA_up10k	445	NA	n367006	NM_001203_dup1	chr4	95664951	95678684	511	-	chr4	95679128	96079601	5560	+	*		0.7131	0.866	0	0.07	0.07	0.2	0.49	0.28
cis_mRNA_dw20k	19144	NA	n342316	NM_004558_dup1	chr19	5847478	5858250	1188	+	chr19	5823818	5828335	1104	+	*		-0.9449	-0.866	0.03	0	0.03	0	0.09	0.03
cis_mRNA_overlap	0	Lnc-AntiOverlap-mRNA	n409778	NM_002379_dup1	chr1	31191619	31199593	2050	+	chr1	31184124	31196432	3830	-	*		0.9921	1	0.13	0.15	0.12	0.29	0.34	0.25
cis_mRNA_dw20k	5346	NA	n410901	NM_000112_dup1	chr5	149372686	149380171	3988	-	chr5	149340300	149367341	8082	+	*		-0.7766	-1	0.49	0.45	0.29	5.53	6.1	6.23

cis_mRNA_dw20k	3912	NA	n382098	NM_000123_dup1	chr13	103532449	103548383	2312	+	chr13	103498191	103528538	4091	+	*		-0.8773	-1	0.03	0.18	0.08	7.15	6.16	6.38
tran	NA	NA	LTCONS_00048125	NM_198256_dup1	chr22	21623559	21623959	401	.	chr2	11584501	11606297	3240	-		-187.79	-0.9893	-1	0.09	0	1.17	6.61	7.11	4.88
cis_mRNA_up10k	74	NA	n363648	NM_001938_dup1	chr1	93804252	93811405	969	-	chr1	93811478	93828149	3209	+	*		0.8026	1	0	1.35	0.32	24.26	26.9	26.4
cis_mRNA_dw20k	9656	NA	n366667	NM_006232_dup1	chr3	184096018	184097565	1434	+	chr3	184081194	184086363	821	+	*		0.9999	1	0.07	0.02	0	12.88	11.73	11.3
cis_mRNA_up10k	287	NA	n339328	NM_002461_dup1	chr16	88729781	88753594	923	+	chr16	88718348	88729495	1795	-	*		0.9992	1	2.74	2.85	1.53	245	258.29	152
cis_mRNA_overlap	0	mRNA	n335671	NM_005319_dup1	chr6	26055935	26056500	566	-	chr6	26055968	26056699	732	-	*		0.9793	1	0	0.73	2.2	15.11	18.5	21.75
cis_mRNA_dw20k	442	NA	n385229	NM_000722_dup1	chr7	81575760	81578977	3218	-	chr7	81579418	82073031	3822	-	*		0.9678	1	3.5	5.57	4.12	4.61	7.8	6.28
cis_mRNA_dw20k	19947	NA	n338259	NM_001194_dup1	chr19	637105	637537	433	-	chr19	589893	617159	3408	+	*		-0.9395	-0.866	0.09	0.08	0.08	2.95	3.41	3.25
cis_mRNA_overlap	0	Lnc-AntiOverlap-mRNA	n339044	NM_004424_dup1	chr16	2285690	2286914	1225	-	chr16	2273567	2285743	2573	+	*		-0.9259	-1	0.23	0.26	0.2	10.85	10.58	12.42
cis_mRNA_up10k	2377	NA	n370776	NM_005384_dup1	chr9	94188520	94189426	474	+	chr9	94171327	94186144	2085	-	*		-0.983	-1	0.08	0	0.28	8.99	9.75	8.06
cis_mRNA_overlap	0	mRNA	n332576	NM_002601_dup1	chr2	232643434	232645989	991	-	chr2	232597147	232645974	1118	-	*		-0.9968	-1	0.77	0.74	0.99	29.78	30.21	28.06
cis_mRNA_overlap	0	Lnc-CompleteIn-mRNAIntron	n324792	NM_002240_dup1	chr21	39268225	39271570	1598	-	chr21	38996785	39288696	2485	-	*		0.9189	0.866	0	0.04	0.04	0.26	0.7	0.99
cis_mRNA_dw20k	4616	NA	n408156	NM_006218_dup1	chr3	178957537	178977679	2127	-	chr3	178866311	178952922	3724	+	*		-0.9955	-1	0.21	0.27	0	8.55	8.06	9.64
tran	NA	NA	LTCONS_00042936	NM_000554_dup1	chr20	3388848	3390736	1889	+	chr19	48325099	48347066	4482	+		-1373.31	0.9165	1	1	1.37	1.02	2.24	2.42	2.32
tran	NA	NA	LTCONS_00042936	NM_000761_dup1	chr20	3388848	3390736	1889	+	chr15	75041184	75048941	3127	+		-1089.76	-0.9811	-1	1	1.37	1.02	0.09	0.05	0.08
tran	NA	NA	LTCONS_00042936	NM_005540_dup1	chr20	3388848	3390736	1889	+	chr1	38326369	38412729	4454	-		-996.35	0.9858	1	1	1.37	1.02	3.26	4.28	3.49
cis_mRNA_overlap	0	mRNA	LTCONS_00031118	NM_001991_dup1	chr17	40852278	40857101	3759	-	chr17	40852293	40897071	4646	-		-2137.92	0.9337	1	1.82	2.6	3.96	6.48	7.09	7.37
cis_mRNA_up10k	6881	NA	LTCONS_00075255	NM_001183_dup1	chrX	153647388	153650098	2034	+	chrX	153656978	153664862	2099	+	*		0.9403	1	1.66	1.82	1.27	54.39	60.19	50.46
cis_mRNA_overlap	0	Lnc-AntiOverlap-mRNA	n378044	NM_005378_dup1	chr2	16076387	16081704	1313	-	chr2	16080683	16087129	2605	+	*		-0.9789	-0.866	0.03	0.03	0.02	3.34	3.77	5.34
cis_mRNA_overlap	0	Lnc-AntiOverlap-mRNA	LTCONS_00064494	NM_006735_dup1	chr7	27135193	27139975	4288	+	chr7	27139973	27142394	1778	-	*		0.9731	1	21.13	35.98	7.05	0.72	1.95	0.24
cis_mRNA_dw20k	693	NA	n375535	NM_002126_dup1	chr17	53403118	53405152	1763	-	chr17	53342321	53402426	5599	+	*		-0.8778	-1	0.08	0.04	0.07	1.79	2.83	2.51
cis_mRNA_dw20k	1070	NA	n344618	NM_001523_dup1	chr19	52192594	52215296	5779	+	chr19	52216365	52227221	2087	-	*		-0.9999	-1	0.5	0.69	0.79	0.39	0.27	0.21
cis_mRNA_overlap	0	AntiCompleteIn-mRNAIntron	LTCONS_00004125	NM_005026_dup1	chr1	9739681	9747632	1753	-	chr1	9711790	9789172	5411	+	*		-0.836	-1	0.3	1.07	0.86	5.92	4.15	5.55
cis_mRNA_overlap	0	mRNA	n409338	NM_002027_dup1	chr8	42911442	42940932	1609	+	chr8	42911442	42940931	1694	+	*		0.986	1	3.94	2.61	1.61	30.77	29.52	27.83
cis_mRNA_dw20k	3064	NA	n364035	NM_000254_dup1	chr1	237070344	237091621	7360	+	chr1	236958581	237067281	10558	+	*		-0.9428	-1	0.23	0.15	0.14	5.96	6.2	6.38
cis_mRNA_overlap	0	Lnc-AntiOverlap-mRNA	n410865	NM_002318_dup1	chr8	23193721	23223638	4374	+	chr8	23154410	23261722	3810	-	*		-0.9449	-0.866	0.02	0.02	0.03	0.13	0.17	0.05
cis_mRNA_up10k	7654	NA	n342500	NM_001296_dup1	chr3	42842342	42843311	970	-	chr3	42850964	42908775	2960	+	*		-0.9979	-0.866	0.47	0.46	0.33	0.01	0.01	0.07
cis_mRNA_up10k	1710	NA	n367358	NM_004354_dup1	chr4	78076012	78076648	344	-	chr4	78078357	78091213	5474	+	*		0.9692	1	0.34	0.1	0.2	12.98	9.76	11.8
cis_mRNA_up10k	6855	NA	LTCONS_00014962	NM_002105_dup1	chr11	118973031	118973569	539	.	chr11	118964584	118966177	1594	-	*		-0.997	-1	0.69	1.13	0.43	2.07	1.56	2.29
cis_mRNA_up10k	903	NA	n379376	NM_024007_dup1	chr5	158527690	158544486	2664	+	chr5	158122923	158526788	5274	-	*		-0.79	-1	1.71	2.16	1.59	16.97	16.26	19.88
cis_mRNA_up10k	7312	NA	n342742	NM_004420_dup1	chr11	1594477	1623308	1626	+	chr11	1575281	1587166	4455	-	*		0.9193	1	2.65	2.18	1.59	3.67	2.51	2.23
tran	NA	NA	LTCONS_00002189	NM_005022_dup1	chr1	148555358	148575210	8395	+	chr17	4848947	4851825	807	-		-1334.26	-0.765	-1	0.34	0.7	0.22	129	127.67	139.27
cis_mRNA_up10k	2465	NA	n339593	NM_002196_dup1	chr20	20344335	20346301	1967	+	chr20	20348765	20351593	2829	+	*		0.9973	1	0	0.02	0.08	1.57	1.76	2.63
cis_mRNA_up10k	111	NA	LTCONS_00034761	NM_004977_dup1	chr19	50832744	50837000	4257	+	chr19	50818765	50832634	3176	-	*		-0.9639	-1	0.26	0.53	0.38	0.39	0.34	0.38
cis_mRNA_up10k	6555	NA	n365084	NM_002539_dup1	chr2	10595007	10597698	2359	+	chr2	10580508	10588453	2062	-	*		-0.921	-1	0.11	0	0.27	99.31	99.74	77.45
cis_mRNA_dw20k	5546	NA	LTCONS_00074293	NM_181532_dup1	chrX	48694093	48706251	293	+	chrX	48687283	48688548	1266	+	*		-0.9411	-1	1.66	1.26	0.87	0.19	0.28	0.3
cis_mRNA_overlap	0	Lnc-AntiOverlap-mRNA	n339879	NM_001080467_dup1	chr18	47340393	47377429	1615	+	chr18	47349156	47721451	9520	-	*		-0.9967	-1	0.04	0.02	0	0.13	0.62	1.27
cis_mRNA_dw20k	3425	NA	LTCONS_00045058	NM_004535_dup1	chr20	62877030	62878115	1086	.	chr20	62795827	62873606	5535	+	*		-0.9747	-1	0.74	0.8	0.9	16.59	15.35	14.42
cis_mRNA_up10k	15	NA	LTCONS_00001675	NM_001839_dup1	chr1	95392749	95433017	5380	+	chr1	95362507	95392735	2066	-	*		0.9828	1	3.6	4.16	3.05	47.36	51.4	39.59
cis_mRNA_overlap	0	Lnc-CompleteIn-mRNAIntron	n342935	NM_001353_dup1	chr10	5015558	5017273	1198	+	chr10	5005454	5020158	1379	+	*		-0.8844	-1	3.1	5.61	9.59	104.55	74.47	67.94
cis_mRNA_dw20k	13308	NA	n344585	NM_001731_dup1	chr12	92367962	92520747	9659	-	chr12	92534054	92539673	4680	-	*		0.9671	1	0.07	0.1	0.09	4.38	5.24	4.76
cis_mRNA_up10k	6100	NA	n410560	NM_002408_dup1	chr14	50065415	50081390	1965	+	chr14	50087489	50090199	2711	+	*		0.9999	1	0.06	0	0.17	11.04	10.58	11.93
cis_mRNA_up10k	439	NA	n409487	NM_006196_dup1	chr2	70189395	70314147	2386	-	chr2	70314585	70316335	1751	+	*		0.8636	1	1.5	2.03	1.03	14.64	14.66	14.07
cis_mRNA_overlap	0	Lnc-AntiOverlap-mRNA	n378757	NM_005253_dup1	chr2	28607276	28617539	3001	-	chr2	28615779	28637516	3991	+	*		-0.9897	-1	0.17	0.35	0.36	0.29	0.21	0.19
cis_mRNA_dw20k	1265	NA	n342697	NM_002622_dup1	chr5	139554736	139623371	824	+	chr5	139624635	139682689	1324	-	*		0.6286	1	17.44	15.44	17.28	46.26	43.46	43.72
cis_mRNA_dw20k	99	NA	n410711	NM_004146_dup1	chr19	14640379	14676792	1198	+	chr19	14676890	14682889	546	-	*		-0.994	-1	2.2	2.69	1.22	121.93	118.29	133.96
cis_mRNA_overlap	0	Lnc-AntiOverlap-mRNA	n341297	NM_000441_dup1	chr7	107297075	107302156	3687	-	chr7	107301080	107358254	4930	+	*		0.7559	0.866	0	0.03	0.02	0.04	0.05	0.04
cis_mRNA_dw20k	11433	NA	n339125	NM_005003_dup1	chr16	23579994	23580903	910	+	chr16	23592335	23607639	667	-	*		0.9599	1	0.51	0.46	0.55	113.95	103.33	116.37
cis_mRNA_overlap	0	CompleteIn-LncExon	n407108	NM_005340_dup1	chr5	130494875	130501034	971	-	chr5	130494875	130501034	782	-	*		-0.9694	-1	0.52	1.21	1.16	278.99	210.57	232.12
cis_mRNA_overlap	0	Lnc-CompleteIn-mRNAIntron	n326188	NM_004481_dup1	chr1	230362460	230388012	818	+	chr1	230202956	230417875	4459	+	*		0.7731	1	0.22	0.2	0.23	13.09	13.06	14.05
cis_mRNA_dw20k	97	NA	n410905	NM_006735_dup1	chr7	27135713	27139877	775	+	chr7	27139973	27142394	1778	-	*		0.7476	1	38.64	40.29	7.96	0.72	1.95	0.24
cis_mRNA_dw20k	412	NA	n410736	NM_002586_dup1	chr6	32148745	32152099	1673	-	chr6	32152510	32157963	3213	-	*		0.9721	1	0.79	2.55	1.36	22.77	24.45	22.93
cis_mRNA_dw20k	6506	NA	n338195	NM_005198_dup1	chr22	51009428	51010882	1455	+	chr22	51017387	51021428	1629	-	*		0.9016	1	0.1	0.17	0.19	6.78	8.31	10.8
cis_mRNA_up10k	1007	NA	n340492	NM_000747_dup1	chr17	7323422	7347400	1975	+	chr17	7348406	7360932	2437	+	*		0.9793	1	0.65	0.6	0.8	5.66	5.57	7.62
cis_mRNA_overlap	0	AntiCompleteIn-mRNAIntron	n341771	NM_002581_dup1	chr9	119048108	119051048	1854	-	chr9	118916071	119164600	10970	+	*		-0.8599	-1	0.06	0.12	0.05	17.46	10.62	28.91
cis_mRNA_dw20k	16690	NA	n339281	NM_020995_dup1	chr16	72127834	72146802	4226	+	chr16	72097125	72111145	1242	+	*		0.993	0.866	5.75	5.1	10.16	0.03	0.03	0.05
cis_mRNA_up10k	72	NA	n409200	NM_001284_dup1	chr5	115163894	115177548	4446	-	chr5	115177619	115249778	1289	+	*		0.9408	1	1.5	0.94	1.21	36.85	33.69	34.22
cis_mRNA_dw20k	9656	NA	n366740	NM_006232_dup1	chr3	184096018	184097565	335	-	chr3	184081194	184086363	821	+	*		0.848	1	0.24	0.21	0.11	12.88	11.73	11.3
cis_mRNA_overlap	0	Lnc-AntiOverlap-mRNA	LTCONS_00017004	NM_001242_dup1	chr12	6548167	6560780	3430	-	chr12	6554051	6560884	1320	+		-1614.17	-0.9052	-1	1.57	1.5	1.75	0.08	0.14	0.05

cis_mRNA_up10k	562	NA	n367620	NM_002199_dup1	chr4	185396287	185407002	417	-	chr4	185308876	185395726	2286	-	*	-0.9387	-1	0.09	0.15	0	2.68	2.63	3.42
cis_mRNA_dw20k	3880	NA	n337810	NM_000911_dup1	chr1	29194087	29198224	1764	+	chr1	29138654	29190208	1774	+	*	0.9701	1	0.2	0.62	0.14	0.12	0.21	0.07
cis_mRNA_up10k	105	NA	n338088	NM_001938_dup1	chr1	93730202	93811374	1004	-	chr1	93811478	93828149	3209	+	*	-0.9993	-1	0.33	0	0.05	24.26	26.9	26.4
cis_mRNA_dw20k	8475	NA	n366214	NM_000387_dup1	chr3	48885397	48885882	407	+	chr3	48894356	48936426	1892	-	*	0.9403	1	0	0.31	0.24	12.66	13.37	12.99
cis_mRNA_up10k	8514	NA	LTCONS_00035854	NM_004386_dup1	chr19	19311418	19314269	2319	-	chr19	19322782	19363061	6406	+	*	-0.7924	-1	0.6	0.88	0.7	18.14	15.87	15.95
cis_mRNA_up10k	8019	NA	LTCONS_00035854	NM_005919_dup1	chr19	19311418	19314269	2319	-	chr19	19256376	19303400	1952	-	*	-0.9359	-0.866	0.6	0.88	0.7	0.17	0.13	0.17
cis_mRNA_up10k	203	NA	n373384	NM_006143_dup1	chr12	12849323	12853012	1588	+	chr12	12813995	12849121	1743	-	*	0.9994	1	0.54	0.6	0.33	10.65	10.89	9.62
cis_mRNA_up10k	5722	NA	n378803	NM_002227_dup1	chr1	65437908	65458695	2804	-	chr1	65298906	65432187	5053	-	*	0.9977	0.866	0.11	0.12	0.12	17.19	22.22	21.84
cis_mRNA_overlap	0	AntiCompleteIn-mRNAIntron	n387169	NM_004525_dup1	chr2	170183043	170183659	617	+	chr2	169983619	170219122	15735	-	*	0.9985	1	6.22	8.04	7.4	0.08	0.18	0.14
cis_mRNA_up10k	4527	NA	LTCONS_00027204	NM_002513_dup1	chr16	1826236	1832921	6298	-	chr16	1820321	1821710	1014	-	*	0.9998	1	1.12	1.39	1.63	19.73	22.56	24.92
cis_mRNA_up10k	46	NA	n373038	NM_005967_dup1	chr12	57480963	57482632	1279	+	chr12	57482677	57489259	2711	+	*	0.9678	1	0	0.07	0.17	14.45	15.54	20.57
cis_mRNA_up10k	6433	NA	n368271	NM_002202_dup1	chr5	50671224	50672526	432	-	chr5	50678958	50690564	2718	+	*	0.9951	1	7.48	8.17	9.91	157.32	168.18	214.48
cis_mRNA_overlap	0	Lnc-AntiOverlap-mRNA	n342734	NM_002116_dup1	chr6	29867168	29911213	2259	-	chr6	29910309	29913661	1549	+	*	-0.9997	-0.866	0.27	0.26	0.27	39.79	99.26	38.06
cis_mRNA_up10k	738	NA	n381117	NM_004559_dup1	chr1	43144357	43147329	649	+	chr1	43148066	43168020	1546	+	*	-0.9678	-1	1.07	1.12	1.25	416.37	391.38	366.67
cis_mRNA_dw20k	396	NA	n363455	NM_006496_dup1	chr1	110138847	110140403	596	+	chr1	110091186	110138452	4730	+	*	-0.6429	-1	1.05	1.12	0.71	41.69	38.71	41.74
cis_mRNA_overlap	0	Lnc-AntiOverlap-mRNA	n341029	NM_002334_dup1	chr11	46867978	46895947	1504	+	chr11	46878268	46940173	8227	-	*	0.9449	0.866	0	0.06	0.02	0.01	0.02	0.01
cis_mRNA_dw20k	570	NA	n338931	NM_006236_dup1	chr2	105474040	105475032	993	+	chr2	105471969	105473471	1503	+	*	0.8224	1	1.1	1.68	0.98	1.01	1.07	0.86
cis_mRNA_overlap	0	Lnc-CompleteIn-mRNAExon	n338609	NM_005288_dup1	chr13	27332962	27333964	1003	-	chr13	27328908	27334922	4863	-	*	0.9936	1	0.04	0.36	0.1	0.24	0.38	0.25
cis_mRNA_overlap	0	Lnc-AntiOverlap-mRNA	n340538	NM_001388_dup1	chr17	18010648	18013777	3130	-	chr17	17991283	18011292	1888	+	*	0.6547	0.866	0.01	0.01	0	3.63	3.87	3.57
cis_mRNA_overlap	0	Lnc-AntiOverlap-mRNA	LTCONS_00060918	NM_001527_dup1	chr6	114290865	114320962	1572	+	chr6	114257320	114292359	6656	-	*	0.6176	1	0.38	0.02	0.01	26.77	26.54	24.98
cis_mRNA_dw20k	11658	NA	n324660	NM_005297_dup1	chr22	41090475	41095186	1142	-	chr22	41075182	41078818	2423	+	*	-0.9843	-0.866	0.03	0.03	0.08	3.49	3.86	1.89
cis_mRNA_dw20k	8265	NA	n383544	NM_001523_dup1	chr19	52206207	52208101	1072	+	chr19	52216365	52227221	2087	-	*	-0.9934	-1	0	0.13	0.23	0.39	0.27	0.21
cis_mRNA_up10k	168	NA	n366949	NM_002111_dup1	chr4	3065198	3076241	515	-	chr4	3076408	3245687	13475	+	*	0.9996	1	0	0.07	0.13	4.95	5.67	6.35
cis_mRNA_dw20k	5582	NA	n338345	NM_005956_dup1	chr14	64932306	64935981	1158	+	chr14	64854759	64926725	3448	+	*	0.9109	1	1.45	0.77	1.28	10.92	9.53	10.05
cis_mRNA_up10k	3250	NA	n341282	NM_002319_dup1	chr7	100187025	100201661	2537	-	chr7	100171634	100183776	3201	-	*	-0.9611	-1	0.05	0.09	0.17	3.74	3.68	2.74
cis_mRNA_overlap	0	Lnc-CompleteIn-mRNAExon	n336033	NM_001697_dup1	chr21	35279646	35281515	241	-	chr21	35275757	35288158	806	-	*	0.9593	1	0.55	0	0.16	195.26	180.19	188.37
cis_mRNA_dw20k	6609	NA	n341041	NM_002032_dup1	chr11	61721451	61725149	639	-	chr11	61731757	61735132	1229	-	*	0.9411	1	0.4	0.31	0.2	39.89	38.93	38.62
cis_mRNA_overlap	0	Lnc-CompleteIn-mRNAExon	n341139	NM_014619_dup1	chr11	120531028	120856969	2869	+	chr11	120531028	120856969	2869	+	*	1	1	0.11	0.18	0.21	0.11	0.18	0.21
cis_mRNA_dw20k	1113	NA	n410816	NM_001916_dup1	chr8	145153540	145159138	1685	-	chr8	145149960	145152428	1210	+	*	0.9959	1	1.76	2.35	2.45	45.46	50.42	50.68
cis_mRNA_dw20k	13837	NA	LTCONS_00012161	NM_001532_dup1	chr11	66115284	66116156	873	+	chr11	66129992	66139291	2515	-	*	0.8731	1	1.18	1.34	0.8	4.44	6.66	3.69
cis_mRNA_up10k	1073	NA	n376503	NM_005178_dup1	chr19	45248302	45250906	2447	-	chr19	45251978	45263301	1864	+	*	-0.9059	-1	0.04	0.01	0	0.35	0.39	0.46
cis_mRNA_dw20k	819	NA	n340956	NM_000391_dup1	chr11	6627526	6633179	5392	-	chr11	6633997	6640692	3533	-	*	0.9462	1	1.03	1.13	1.56	0.1	0.28	0.47
cis_mRNA_dw20k	7723	NA	n376880	NM_001958_dup1	chr20	62110073	62111644	1340	-	chr20	62119366	62130505	1841	-	*	0.9839	1	2.42	3.86	2.46	119.25	142.89	124.3
tran	NA	NA	LTCONS_00057341	NM_001187_dup1	chr5	180708367	180730650	3529	+	chr21	11057796	11098937	932	-	-508.89	-0.8555	-1	2.4	3.98	2.61	0.15	0.07	0.1
cis_mRNA_dw20k	4599	NA	n340546	NM_004365_dup1	chr5	89684285	89684930	646	-	chr5	89689528	89705603	982	-	*	-0.9608	-1	0.34	0.21	0.5	13.1	14.42	12.52
cis_mRNA_overlap	0	Lnc-CompleteIn-mRNAIntron	n383603	NM_177983_dup1	chr2	27615490	27616443	954	-	chr2	27604061	27632496	2253	-	*	-0.9977	-1	0.45	0.58	0.53	82.74	74.12	77.95
cis_mRNA_dw20k	12136	NA	n332789	NM_002332_dup1	chr12	57619277	57619686	410	+	chr12	57522282	57607142	14905	+	*	0.7201	1	1.18	3.2	0.88	5.28	5.63	3.59
cis_mRNA_up10k	221	NA	LTCONS_00038541	NM_006236_dup1	chr2	105469943	105471749	1604	+	chr2	105471969	105473471	1503	+	*	0.7675	1	0.86	1.45	0.81	1.01	1.07	0.86
cis_mRNA_overlap	0	CompleteIn-LncExon	LTCONS_00059035	NM_005520_dup1	chr5	179041176	179061747	4585	-	chr5	179041179	179050722	2249	-	-1243.5	-0.998	-1	0.33	0.95	0.81	115.27	109.56	111.19
cis_mRNA_up10k	89	NA	LTCONS_00027143	NM_005009_dup1	chr16	433523	447104	1219	-	chr16	447192	450754	1007	+	*	0.8634	1	1	1.67	0.87	11.17	12.32	9.15
cis_mRNA_overlap	0	Lnc-CompleteIn-mRNAIntron	n333368	NM_001500_dup1	chr6	1950463	1952398	575	-	chr6	1624041	2245846	1672	-	*	0.9109	0.866	0	0.06	0.06	1.78	2.26	2.6
cis_mRNA_dw20k	8566	NA	n338257	NM_001319_dup1	chr19	1989902	1990369	468	+	chr19	1941161	1981337	2905	+	*	0.9646	1	0.88	1.39	2.37	27.05	27.35	30.59
cis_mRNA_up10k	116	NA	n373510	NM_002567_dup1	chr12	118555262	118573755	828	-	chr12	118573870	118583390	1491	+	*	0.8804	1	0.74	0.49	0.59	425.12	351.86	413.77
cis_mRNA_overlap	0	mRNA-AntiCompleteIn-LncIntron	n379691	NM_001757_dup1	chr21	37426162	37481988	2124	-	chr21	37442285	37445462	1245	+	*	-0.9183	-0.866	0.05	0.06	0.06	9.15	7.67	8.26
cis_mRNA_dw20k	11226	NA	LTCONS_00072544	NM_000155_dup1	chr9	34661799	34666208	3400	-	chr9	34646635	34650574	1335	+	*	0.9829	1	1.6	2.07	0.88	7.77	8.11	6.61
cis_mRNA_dw20k	2919	NA	n345587	NM_002207_dup1	chr3	37864199	37867773	1138	+	chr3	37493813	37861281	3962	+	*	0.8715	1	1.29	0.53	1.9	3.55	3.31	6.23
cis_mRNA_dw20k	10488	NA	n340715	NM_000212_dup1	chr17	45400564	45402367	1686	-	chr17	45331208	45390077	4894	+	*	0.9919	1	0.06	0.11	0.09	0.08	0.15	0.13
cis_mRNA_dw20k	6467	NA	n337934	NM_000098_dup1	chr1	53686335	53689930	3596	+	chr1	53662101	53679869	3094	+	*	0.9103	1	0.09	0.04	0.13	5.11	4.59	5.13
cis_mRNA_overlap	0	Lnc-AntiOverlap-mRNA	n380141	NM_001527_dup1	chr6	114291118	114314627	568	+	chr6	114257320	114292359	6656	-	*	-0.9965	-1	0	0.19	0.9	26.77	26.54	24.98
cis_mRNA_dw20k	4793	NA	n342487	NM_001570_dup1	chr3	10290219	10322900	4688	+	chr3	10206563	10285427	3469	+	*	0.9037	1	4.02	4.52	3.65	2.12	2.31	1.24
cis_mRNA_up10k	3636	NA	n407077	NM_001524_dup1	chr17	40341105	40346255	2693	-	chr17	40336078	40337470	577	-	*	-0.8523	-1	0.22	0.1	0.58	0.06	0.23	0
cis_mRNA_up10k	390	NA	n372797	NM_152713_dup1	chr11	125461043	125462350	469	-	chr11	125462739	125491280	2803	+	*	-0.6509	-1	0.48	0.87	0.43	20.19	19.9	23.13
cis_mRNA_up10k	404	NA	LTCONS_00048138	NM_001429_dup1	chr22	41487747	41488211	465	.	chr22	41488614	41576081	8761	+	*	0.9723	1	2.43	4.65	3.98	7.77	9.91	8.82
cis_mRNA_up10k	197	NA	LTCONS_00010431	NM_004329_dup1	chr10	88493609	88516200	787	-	chr10	88516396	88684945	3616	+	*	0.994	1	1	0.66	0.85	14.89	14.02	14.59
cis_mRNA_dw20k	3098	NA	n375055	NM_004483_dup1	chr16	81110924	81112455	1077	-	chr16	81115552	81129980	1538	-	*	-0.9611	-1	0.1	0.09	0.12	23.51	24.66	22.67
cis_mRNA_overlap	0	mRNA-AntiCompleteIn-LncIntron	n409659	NM_000867_dup1	chr2	231921578	232037540	3155	+	chr2	231972947	231989824	2235	-	*	-0.8486	-1	0.16	0.56	1.36	0.11	0.06	0.05
cis_mRNA_up10k	8415	NA	n345810	NM_004539_dup1	chr18	55297591	55309277	647	+	chr18	55267891	55289177	2868	-	*	0.9982	1	0.45	0.92	0.85	35.13	60.56	58.35

cis_mRNA_overlap	0	mRNA	n341553	NM_005181_dup1	chr8	86132845	86361265	2403	+	chr8	86351056	86361269	1753	+	*		0.9538	1	0.09	0.34	0.24	0	0.06	0.02
cis_mRNA_dw20k	15258	NA	LTCONS_00062522	NM_002062_dup1	chr6	39070777	39082941	7466	-	chr6	39016557	39055520	3123	+	*		0.997	1	2.16	2.25	2.92	0.6	0.61	0.88
cis_mRNA_overlap	0	Lnc-CompleteIn-mRNAIntron	n338399	NM_001249_dup1	chr14	74468337	74470121	1774	-	chr14	74433172	74486026	2057	-	*		0.9901	1	0	0.07	0.02	1.4	1.57	1.47
cis_mRNA_up10k	2526	NA	n377100	NM_006134_dup1	chr21	34854806	34856565	666	-	chr21	34821448	34852281	2332	-	*		-0.6996	-1	0.14	0.33	0.32	14.73	11.41	14.02
cis_mRNA_dw20k	7850	NA	n372336	NM_004052_dup1	chr10	133772474	133773355	808	+	chr10	133781204	133795435	1534	-	*		0.9537	1	5.45	5.97	4.46	38.89	39.44	38.54
cis_mRNA_up10k	8072	NA	n368301	NM_005909_dup1	chr5	71393374	71395047	1354	-	chr5	71403118	71505851	11996	+	*		-0.858	-0.866	0.05	0.05	0.07	375.23	378.22	372.4
cis_mRNA_dw20k	8785	NA	n381766	NM_000855_dup1	chr11	106544752	106549126	4375	-	chr11	106557910	106889171	2954	-	*		0.9981	1	8.63	12.21	3.18	10.46	13.54	4.54
cis_mRNA_dw20k	12340	NA	n374167	NM_005252_dup1	chr14	75761276	75763354	697	+	chr14	75745481	75748937	2158	+	*		-0.9155	-1	0.36	0.19	0.23	2.05	3.91	2.79
cis_mRNA_overlap	0	Lnc-CompleteIn-mRNAIntron	n324869	NM_004540_dup1	chr21	22504129	22715010	435	+	chr21	22370633	22912517	5002	+	*		-0.8914	-1	0.09	0.16	0.39	38.47	34.42	32.24
cis_mRNA_dw20k	13736	NA	LTCONS_00007599	NM_005373_dup1	chr1	43833870	43834994	1125	.	chr1	43803475	43820135	3645	+	*		-0.9484	-0.866	0.67	0.78	1.01	0.03	0.03	0
tran	NA	NA	LTCONS_00042561	NM_000887_dup1	chr2	223745026	223807923	648	-	chr16	31366509	31394318	4649	+		-395.22	0.9892	1	1.57	1.18	0.52	0.05	0.03	0.01
cis_mRNA_dw20k	9780	NA	n340455	NM_020310_dup1	chr17	2275157	2277575	2419	-	chr17	2287354	2304258	4842	-	*		-0.9799	-0.866	0.03	0.01	0.01	3.63	4.2	4.08
cis_mRNA_up10k	97	NA	LTCONS_00052240	NM_170662_dup1	chr3	105587983	105589603	1621	.	chr3	105377109	105587887	3976	-	*		0.9891	1	0.51	0.62	0.97	3.39	3.79	4.46
cis_mRNA_up10k	982	NA	n384568	NM_004477_dup1	chr4	190784615	190860993	1644	-	chr4	190861974	190884359	1043	+	*		-0.8603	-1	0.04	0.15	0	30.28	29.1	33.66
cis_mRNA_dw20k	12032	NA	n408882	NM_000840_dup1	chr7	86506223	86669550	6597	-	chr7	86273230	86494192	4260	+	*		0.6417	1	8.96	6.35	9.45	5.42	4.62	32.13
cis_mRNA_overlap	0	Lnc-CompleteIn-mRNAIntron	n342212	NM_033223_dup1	chr15	27721418	27723354	1937	+	chr15	27216429	27778373	2004	+	*		0.9964	1	0	0.03	0.01	0.51	2.37	1.27
cis_mRNA_up10k	9042	NA	n340921	NM_012140_dup1	chr17	79668354	79670330	1977	-	chr17	79679371	79688042	1885	+	*		-0.9721	-1	4.76	4.66	4.37	3.21	3.23	4.57
cis_mRNA_overlap	0	mRNA	LTCONS_00054670	NM_005908_dup1	chr4	103552643	103652127	6783	-	chr4	103552643	103682151	3311	-		-1758.75	-0.999	-1	3.35	2.76	3.76	4.88	5.92	4.27
cis_mRNA_overlap	0	mRNA	LTCONS_00075749	NM_004463_dup1	chrX	54471738	54477717	5590	-	chrX	54471887	54522599	4275	-	*		0.9998	1	0.34	0.61	0.52	4.15	5.43	4.98
cis_mRNA_dw20k	5269	NA	n406623	NM_006185_dup1	chr11	71639768	71708643	2706	+	chr11	71713911	71791573	7182	-	*		-0.9975	-1	0.45	0.18	0.55	19.49	22.04	18.21
cis_mRNA_up10k	843	NA	n380206	NM_024007_dup1	chr5	158527630	158534225	568	+	chr5	158122923	158526788	5274	-	*		0.9981	1	2.2	2.13	2.65	16.97	16.26	19.88
cis_mRNA_dw20k	4869	NA	n339148	NM_000784_dup1	chr2	219684884	219687242	2359	+	chr2	219646472	219680016	2293	+	*		0.6069	0.866	0.01	0	0.01	0.36	0.31	0.67
cis_mRNA_up10k	147	NA	n339723	NM_001719_dup1	chr20	55841853	55858060	1172	+	chr20	55743809	55841707	4031	-	*		0.9787	1	0.06	0.16	0.03	12.87	20.63	7.3
cis_mRNA_overlap	0	CompleteIn-LncIntron	n379409	NM_005370_dup1	chr19	15050246	16748905	1493	+	chr19	16222490	16244445	2177	+	*		0.9012	1	0.05	0.08	0.06	8.22	8.92	8.73
cis_mRNA_overlap	0	mRNA-AntiCompleteIn-LncIntron	n379409	NM_000435_dup1	chr19	15050246	16748905	1493	+	chr19	15270444	15311792	8071	-	*		0.9707	1	0.05	0.08	0.06	0.16	0.38	0.28
cis_mRNA_overlap	0	Lnc-AntiOverlap-mRNA	n408352	NM_033133_dup1	chr17	40128439	40169715	1989	-	chr17	40118759	40129754	5222	+	*		0.9938	1	0.15	0.14	0	12.12	12.06	11.79
cis_mRNA_up10k	161	NA	n379311	NM_005892_dup1	chr17	43289616	43299132	1685	-	chr17	43299292	43324685	3963	+	*		0.803	0.866	0	0.04	0.04	0.54	0.56	0.59
cis_mRNA_dw20k	2720	NA	n375549	NM_000430_dup1	chr17	2591628	2592285	473	+	chr17	2496923	2588909	5614	+	*		-0.9824	-1	0.4	0.43	0.5	115.47	112.23	108.52
cis_mRNA_dw20k	1335	NA	n339646	NM_002563_dup1	chr3	152557179	152559228	2050	+	chr3	152552736	152555845	3110	+	*		0.9959	1	0.18	0.52	0.6	0.2	0.54	0.67
cis_mRNA_dw20k	13830	NA	n408146	NM_001231_dup1	chr1	160185505	160232318	4183	-	chr1	160160285	160171676	1958	+	*		0.9425	0.866	4.09	4.42	4.59	0.02	0.05	0.05
cis_mRNA_overlap	0	AntiCompleteIn-mRNAIntron	n326676	NM_005529_dup1	chr1	22224614	22230303	1054	+	chr1	22148737	22263750	14288	-	*		0.9912	0.866	0.08	0.29	0.11	0.03	0.08	0.03
cis_mRNA_up10k	986	NA	n376426	NM_001425_dup1	chr19	48824029	48827644	2750	-	chr19	48828629	48833810	829	+	*		0.9608	1	0.16	0.17	0.13	0.39	0.43	0.35
cis_mRNA_overlap	0	mRNA	n410670	NM_004507_dup1	chr7	48002885	48019222	2408	-	chr7	48002885	48019222	3017	-	*		0.9231	1	0.28	0.15	0.38	1.74	1.58	2.33
cis_mRNA_overlap	0	Lnc-AntiOverlap-mRNA	n340706	NM_005892_dup1	chr17	43315395	43319101	2003	-	chr17	43299292	43324685	3963	+	*		0.9999	1	1.1	1.22	1.39	0.54	0.56	0.59
cis_mRNA_overlap	0	Lnc-CompleteIn-mRNAIntron	n340298	NM_005245_dup1	chr4	187543069	187544347	1279	-	chr4	187508937	187644987	14764	-	*		0.8857	1	0.11	0.22	0.34	4.71	4.8	9.72
cis_mRNA_overlap	0	Lnc-CompleteIn-mRNAIntron	n340644	NM_001903_dup1	chr5	138157628	138157842	215	+	chr5	138089107	138270723	3761	+	*		-0.7724	-1	0	0.19	0.18	62.15	46.81	57.33
cis_mRNA_dw20k	10604	NA	n410025	NM_002631_dup1	chr1	10490804	10502872	1431	+	chr1	10459085	10480201	1937	+	*		-0.9353	-1	1.71	1.9	2.06	233.37	219.7	171.34
cis_mRNA_dw20k	2298	NA	n367102	NM_000203_dup1	chr4	1000614	1002952	1706	+	chr4	980785	998317	2186	+	*		0.9213	1	1.11	1.83	1.75	3.3	4.57	3.96
cis_mRNA_dw20k	314	NA	n377982	NM_001231_dup1	chr1	160171989	160178659	1578	-	chr1	160160285	160171676	1958	+	*		0.61	0.866	0.13	0.14	0.2	0.02	0.05	0.05
cis_mRNA_up10k	1066	NA	n373232	NM_005967_dup1	chr12	57477184	57481612	1383	-	chr12	57482677	57489259	2711	+	*		0.921	1	0	0.07	0.13	14.45	15.54	20.57
cis_mRNA_overlap	0	Lnc-AntiOverlap-mRNA	LTCONS_00064724	NM_000290_dup1	chr7	44105184	44111845	6662	+	chr7	44102326	44105186	857	-	*		-0.9998	-1	5.69	8.03	6.13	0.54	0.18	0.48
cis_mRNA_dw20k	19275	NA	n332816	NM_001710_dup1	chr6	31939135	31939783	445	-	chr6	31913721	31919861	2627	+	*		0.866	0.866	1.17	1.9	1.9	0	0.02	0.01
cis_mRNA_dw20k	896	NA	n341936	NM_001478_dup1	chr12	58017197	58018656	1460	-	chr12	58019551	58026985	3010	-	*		0.976	1	12.15	12.95	13.33	9.76	10.56	10.66
tran	NA	NA	LTCONS_00057342	NM_001187_dup1	chr5	180708367	180748813	1909	+	chr21	11057796	11098937	932	-		-536.74	0.9697	1	3.46	2.28	2.45	0.15	0.07	0.1
cis_mRNA_dw20k	981	NA	n341923	NM_001417_dup1	chr12	53436973	53448222	2861	-	chr12	53400062	53435993	4028	+	*		0.9973	1	0.89	1.32	0.72	157.67	166.76	152.78
cis_mRNA_up10k	131	NA	n339241	NM_006141_dup1	chr16	66785655	66788646	2897	+	chr16	66754799	66785525	4336	-	*		0.9984	1	0.22	0.2	0.24	82.7	80.53	85.35
cis_mRNA_dw20k	15079	NA	n340464	NM_002203_dup1	chr5	52405687	52410955	2354	+	chr5	52285156	52390609	7869	+	*		0.8867	1	0.09	0.1	0.17	0.01	0.1	0.17
cis_mRNA_overlap	0	Lnc-AntiOverlap-mRNA	n377693	NM_002629_dup1	chr12	104424522	104426026	1389	-	chr12	104424519	104426269	1745	+	*		-0.9778	-1	0.22	0.15	0.1	0	0.08	0.2
cis_mRNA_dw20k	2897	NA	n377939	NM_006898_dup1	chr2	177040722	177053268	984	-	chr2	177028805	177037826	2299	+	*		0.9239	1	0.24	0.52	0.19	14.39	22.87	5.61
cis_mRNA_dw20k	1996	NA	n385222	NM_001540_dup1	chr7	75935609	75937653	2045	+	chr7	75931875	75933614	897	+	*		-0.7753	-1	0	0.06	0.01	4.75	4.15	4.28
cis_mRNA_dw20k	9423	NA	LTCONS_00015879	NM_001487_dup1	chr12	56122913	56125045	2133	+	chr12	56109818	56113491	578	+	*		-0.6124	-1	3.07	4.05	3.91	38.24	29.82	38.21
cis_mRNA_dw20k	4210	NA	n339715	NM_004975_dup1	chr20	47980423	47984296	3870	-	chr20	47988505	48099181	3756	-	*		0.9994	1	0	0.45	0.59	0.58	1.28	1.46
cis_mRNA_dw20k	557	NA	n340640	NM_005937_dup1	chr17	36886612	36891297	2326	+	chr17	36861873	36886056	7500	+	*		0.9619	1	0.75	0.92	1.11	9	9.4	10.79
cis_mRNA_overlap	0	mRNA	n335098	NM_002046_dup1	chr12	6645713	6646179	246	+	chr12	6643657	6647536	1310	+	*		0.7589	1	0.34	0.31	0.15	1086.79	1023.06	1007.08
cis_mRNA_overlap	0	Lnc-AntiOverlap-mRNA	n338346	NM_005956_dup1	chr14	64907089	64908956	1868	-	chr14	64854759	64926725	3448	+	*		-0.9957	-1	2.11	3.04	2.77	10.92	9.53	10.05
tran	NA	NA	LTCONS_00073267	NM_006166_dup1	chr9	114389202	114393569	777	-	chr12	104510858	104532040	3482	-		-649.87	-0.9854	-1	0.78	0.88	0.91	8.32	7.33	7.27
cis_mRNA_dw20k	18550	NA	n410581	NM_000904_dup1	chr6	3038543	3063437	3277	+	chr6	3000067	3019994	1139	+	*		1	1	0.19	0.16	0.03	10.31	10.17	9.55
cis_mRNA_overlap	0	Lnc-AntiOverlap-mRNA	n409121	NM_001482_dup1	chr15	45670898	45672321	1424	+	chr15	45653322	45670980	2586	-	*		-0.9915	-1	2.3	2.43	3.13	5.16	4.33	2.21

cis_mRNA_up10k	8711	NA	n345908	NM_032515_dup1	chr2	242486062	242489482	617	-	chr2	242498192	242513553	2601	+	*		-0.9968	-0.866	0.29	0.05	0.05	3.11	12.21	13.09
cis_mRNA_dw20k	7087	NA	n372719	NM_006725_dup1	chr11	60794935	60810810	3206	-	chr11	60739115	60787849	3289	+	*		0.9107	0.866	0.91	1.22	1.44	0.03	0.04	0.04
cis_mRNA_up10k	409	NA	n338664	NM_002539_dup1	chr2	10588861	10590831	1971	+	chr2	10580508	10588453	2062	-	*		0.6843	1	0.51	0.65	0.47	99.31	99.74	77.45
cis_mRNA_dw20k	5141	NA	n384175	NM_001429_dup1	chr22	41581221	41593505	1375	-	chr22	41488614	41576081	8761	+	*		0.9899	1	3	3.71	3.26	7.77	9.91	8.82
cis_mRNA_dw20k	18244	NA	n345142	NM_004551_dup1	chr11	47624358	47632686	1279	+	chr11	47600562	47606115	959	+	*		0.9919	1	0.25	0.57	0.46	43.21	48.2	47.07
cis_mRNA_dw20k	11226	NA	LTCONS_00072545	NM_000155_dup1	chr9	34661799	34666208	3751	-	chr9	34646635	34650574	1335	+	*		-0.7844	-1	1.25	0.91	1.32	7.77	8.11	6.61
cis_mRNA_dw20k	10947	NA	n346464	NM_004048_dup1	chr15	45021303	45025042	1930	+	chr15	45003685	45010357	987	+	*		0.9975	1	20.05	21.89	18.3	16.04	22.34	8.37
cis_mRNA_dw20k	9311	NA	n405868	NM_004074_dup1	chr11	63753325	63765892	2502	+	chr11	63742079	63744015	507	+	*		-0.9924	-1	4.77	4.13	4.59	178.84	184.45	181.04
cis_mRNA_up10k	3133	NA	n376501	NM_002250_dup1	chr19	44288541	44290302	1269	-	chr19	44270685	44285409	2240	-	*		0.9286	0.866	0.03	0.03	0	0.09	0.06	0.01
cis_mRNA_up10k	3190	NA	n376463	NM_001425_dup1	chr19	48824722	48825440	425	+	chr19	48828629	48833810	829	+	*		0.9177	1	0.09	0.16	0.08	0.39	0.43	0.35
cis_mRNA_dw20k	4943	NA	n365884	NM_000387_dup1	chr3	48885390	48889414	3447	+	chr3	48894356	48936426	1892	-	*		0.9843	1	0.47	0.68	0.6	12.66	13.37	12.99
cis_mRNA_dw20k	520	NA	n381616	NM_005013_dup1	chr11	17353589	17371521	3256	+	chr11	17298286	17353070	1612	+	*		0.9508	1	0.11	0	0.24	13.3	12.18	18.73
cis_mRNA_overlap	0	Lnc-AntiOverlap-mRNA	n377718	NM_000744_dup1	chr20	61991340	62002529	3264	+	chr20	61974665	61992695	5487	-	*		-0.9607	-1	0.59	0.49	0.38	12.16	13.45	13.94
cis_mRNA_overlap	0	mRNA-AntiCompleteIn-LncIntron	n342296	NM_002506_dup1	chr1	115825655	115910884	2801	+	chr1	115828537	115880857	1049	-	*		-0.7559	-0.866	0.02	0.06	0.08	0.03	0.03	0
cis_mRNA_dw20k	5945	NA	n380037	NM_002437_dup1	chr2	27505301	27526416	876	-	chr2	27532360	27545969	1007	-	*		-0.8467	-0.866	0.08	0.04	0.04	10.15	10.8	11.54
cis_mRNA_overlap	0	Lnc-CompleteIn-mRNAIntron	n340028	NM_000124_dup1	chr10	50726991	50729600	2610	-	chr10	50664491	50747147	7006	-	*		0.9344	1	0.22	0.42	0.23	7.41	7.46	7.43
cis_mRNA_up10k	3798	NA	n338985	NM_006186_dup1	chr2	157193084	157198632	5467	-	chr2	157180944	157189287	3531	-	*		0.9979	1	0.2	0.4	0	0.47	0.83	0.02
cis_mRNA_up10k	3476	NA	n344839	NM_001270_dup1	chr5	98265713	98271477	1411	+	chr5	98190908	98262238	6457	-	*		-0.9808	-1	0.59	0.58	0.79	8.76	8.92	8.27
cis_mRNA_overlap	0	Lnc-AntiOverlap-mRNA	n407743	NM_000520_dup1	chr15	72668454	72671129	2676	+	chr15	72635778	72668520	2437	-	*		-0.8945	-1	0.61	0.46	0.35	8.57	8.75	9.7
cis_mRNA_dw20k	15623	NA	n369455	NM_000287_dup1	chr6	42909008	42915981	1889	-	chr6	42931603	42946981	3503	-	*		-0.8948	-1	0.02	0	0.06	5.93	6.88	5.6
cis_mRNA_up10k	5003	NA	n380092	NM_006735_dup1	chr7	27147396	27161792	509	+	chr7	27139973	27142394	1778	-	*		0.9931	1	0.21	0.85	0.08	0.72	1.95	0.24
cis_mRNA_overlap	0	CompleteIn-LncExon	n406634	NM_001826_dup1	chr1	154947118	154951725	924	+	chr1	154947118	154951725	816	+	*		-0.9296	-1	0.62	1.89	0.21	7.53	7.18	8.01
cis_mRNA_overlap	0	Lnc-CompleteIn-mRNAIntron	n338545	NM_002221_dup1	chr1	226920314	226922612	2299	-	chr1	226819391	226926876	6162	-	*		0.6286	0.866	0.03	0.03	0	0.08	0.03	0.02
cis_mRNA_up10k	287	NA	n386164	NM_002461_dup1	chr16	88729781	88741913	2429	+	chr16	88718348	88729495	1795	-	*		0.622	1	3.32	3.58	3.31	245	258.29	152
cis_mRNA_dw20k	11770	NA	LTCONS_00069156	NM_138465_dup1	chr8	144370870	144383103	10873	+	chr8	144349607	144359101	1343	+		-1290.45	-0.9549	-1	0.58	0.62	0.57	1.72	1.15	2.23
cis_mRNA_up10k	285	NA	n379387	NM_001731_dup1	chr12	92539957	92583436	5699	+	chr12	92534054	92539673	4680	-	*		-0.9978	-1	0.02	0	0.01	4.38	5.24	4.76
cis_mRNA_up10k	253	NA	n365196	NM_002665_dup1	chr2	88045926	88047354	315	+	chr2	88047606	88058995	3016	+	*		0.6864	1	0.51	0.92	1.02	0.95	0.96	1.14
cis_mRNA_dw20k	4	NA	LTCONS_00024026	NM_006715_dup1	chr15	75638984	75648130	8535	+	chr15	75648133	75660941	3254	-	*		0.9973	1	1.3	1.88	1.95	6.37	9.28	9.93
cis_mRNA_overlap	0	Lnc-CompleteIn-mRNAIntron	n335680	NM_001500_dup1	chr6	1949489	1949978	490	-	chr6	1624041	2245846	1672	-	*		0.9109	0.866	0	0.14	0.14	1.78	2.26	2.6
cis_mRNA_dw20k	2671	NA	n386477	NM_005941_dup1	chr8	89044244	89046790	2547	-	chr8	89049460	89339717	6335	-	*		0.9932	1	5.57	7.83	7.02	6.49	9.74	8.92
cis_mRNA_up10k	493	NA	n371188	NM_000718_dup1	chr9	140767616	140771749	1223	-	chr9	140772241	141019076	9790	+	*		0.8091	1	0	0.08	0.11	1.14	1.47	3.17
cis_mRNA_overlap	0	Lnc-CompleteIn-mRNAIntron	n341721	NM_001698_dup1	chr9	94035854	94039649	3796	-	chr9	93976097	94124206	1588	-	*		-0.9238	-1	0.28	0.24	0.3	13.16	13.92	11.56
cis_mRNA_overlap	0	CompleteIn-LncExon	n405870	NM_198256_dup1	chr2	11584501	11606297	3311	-	chr2	11584501	11606297	3240	-	*		-1	-1	1.36	1.19	1.94	6.61	7.11	4.88
cis_mRNA_up10k	613	NA	n341048	NM_014205_dup1	chr11	64885782	64886583	800	+	chr11	64883875	64885170	1296	-	*		-0.9941	-1	3.4	4.73	1.92	1.8	0.83	2.54
cis_mRNA_dw20k	13827	NA	n338235	NM_005249_dup1	chr14	29252697	29254990	1652	-	chr14	29236287	29238871	2585	+	*		-0.8963	-1	0	0.04	0.02	0.14	0	0.01
cis_mRNA_overlap	0	mRNA	n410436	NM_004927_dup1	chr11	64889655	64894841	2078	+	chr11	64889716	64894841	2101	+	*		0.9874	1	0.26	0.25	0.24	20.23	19.58	18.43
cis_mRNA_overlap	0	AntiCompleteIn-mRNAIntron	n340992	NM_002224_dup1	chr6	33599094	33601413	1970	-	chr6	33589156	33664351	9034	+	*		0.8474	0.866	0.83	1.17	0.53	0.06	0.06	0.02
cis_mRNA_dw20k	7924	NA	n383543	NM_001523_dup1	chr19	52196593	52208442	1893	+	chr19	52216365	52227221	2087	-	*		-0.9192	-1	0	0.14	0.45	0.39	0.27	0.21
cis_mRNA_overlap	0	Lnc-CompleteIn-mRNAExon	n333214	NM_005959_dup1	chr11	92714830	92715208	379	+	chr11	92702789	92715948	1662	+	*		-0.9608	-1	0.08	0.19	0	0.03	0	0.09
cis_mRNA_dw20k	4016	NA	n385639	NM_002486_dup1	chr9	100440045	100441883	1839	-	chr9	100395705	100436030	5381	+	*		0.7462	1	1.05	1.53	1.39	17.3	18.43	17.34
cis_mRNA_dw20k	391	NA	n339849	NM_002647_dup1	chr18	39661838	39663348	1511	+	chr18	39535199	39661448	3069	+	*		0.9739	1	5.41	6.67	5.93	15.01	16.97	15.41
cis_mRNA_overlap	0	CompleteIn-LncExon	n406590	NM_002626_dup1	chr21	45719925	45747261	3401	+	chr21	45719930	45747256	2913	+	*		0.9465	1	0.44	0.31	0.7	43.79	37.28	47.77
cis_mRNA_up10k	4233	NA	n382897	NM_005009_dup1	chr16	432241	442960	1571	+	chr16	447192	450754	1007	+	*		0.9632	1	0.04	0.1	0	11.17	12.32	9.15
cis_mRNA_overlap	0	Lnc-AntiOverlap-mRNA	n342865	NM_021965_dup1	chr9	70970081	70972776	2058	-	chr9	70971815	71145977	3338	+	*		0.9966	1	0.02	0.03	0.04	0	0.03	0.07
cis_mRNA_dw20k	14833	NA	LTCONS_00070908	NM_000155_dup1	chr9	34665406	34682348	1805	+	chr9	34646635	34650574	1335	+	*		0.7616	1	0.65	1.1	0.58	7.77	8.11	6.61
cis_mRNA_overlap	0	Lnc-CompleteIn-mRNAIntron	n383178	NM_001991_dup1	chr17	40894172	40895176	1005	-	chr17	40852293	40897071	4646	-	*		0.9515	0.866	0.11	0.13	0.13	6.48	7.09	7.37
cis_mRNA_overlap	0	Lnc-CompleteIn-mRNAIntron	n383035	NM_001481_dup1	chr16	90107060	90108062	1003	+	chr16	90089008	90111379	3185	+	*		0.9925	1	1.55	2.89	1.92	0.3	1.81	0.54
cis_mRNA_overlap	0	Lnc-AntiOverlap-mRNA	n411545	NM_005534_dup1	chr21	34804793	34852316	3127	-	chr21	34775202	34810302	2234	+	*		-0.9819	-1	3.33	5.12	5.82	14.09	13.32	13.24
cis_mRNA_up10k	160	NA	n380319	NM_001500_dup1	chr6	2246005	2398898	744	+	chr6	1624041	2245846	1672	-	*		0.8461	1	0.06	0.07	0.21	1.78	2.26	2.6
cis_mRNA_dw20k	4892	NA	n409096	NM_004502_dup1	chr17	46669654	46679703	439	+	chr17	46684594	46688383	1367	-	*		0.7574	0.866	0.78	0.46	0.46	12.19	9.9	5.36
cis_mRNA_up10k	8719	NA	n379718	NM_002461_dup1	chr16	88738213	88746008	4477	+	chr16	88718348	88729495	1795	-	*		0.732	1	4.58	6.8	4.05	245	258.29	152
tran	NA	NA	LTCONS_00073832	NM_002009_dup1	chr9	66544872	66549674	4803	.	chr15	49715375	49779523	3936	+		-1246.54	-0.7146	-0.866	0.76	0.4	0.5	0.04	0.08	0.04
cis_mRNA_up10k	9065	NA	n377554	NM_005198_dup1	chr22	51030492	51033971	914	+	chr22	51017387	51021428	1629	-	*		0.9262	0.866	0.54	0.54	0.64	6.78	8.31	10.8
cis_mRNA_dw20k	6418	NA	n368955	NM_000904_dup1	chr6	3026411	3027751	1268	+	chr6	3000067	3019994	1139	+	*		0.9202	1	0.71	0.47	0.27	10.31	10.17	9.55
cis_mRNA_up10k	8546	NA	n410111	NM_005969_dup1	chr11	3022152	3078681	2819	-	chr11	2965660	3013607	2518	-	*		-0.9997	-1	0.44	0	0.88	7.45	7.82	7.11
cis_mRNA_up10k	8451	NA	LTCONS_00059409	NM_000904_dup1	chr6	2988201	2991617	1921	+	chr6	3000067	3019994	1139	+	*		0.9139	1	0.92	0.59	0.33	10.31	10.17	9.55

cis_mRNA_dw20k	16449	NA	n410616	NM_002586_dup1	chr6	32121776	32136062	2728	+	chr6	32152510	32157963	3213	-	*	0.9624	1	0.9	1.96	1.28	22.77	24.45	22.93
cis_mRNA_overlap	0	Lnc-AntiOverlap-mRNA	n340457	NM_002202_dup1	chr5	50672932	50679087	2258	-	chr5	50678958	50690564	2718	+	*	0.7435	1	27.02	33.79	34.94	157.32	168.18	214.48
cis_mRNA_overlap	0	Lnc-AntiOverlap-mRNA	n378411	NM_001236_dup1	chr21	37513705	37523008	4651	-	chr21	37507263	37518860	1112	+	*	-0.9449	-1	0.43	0.35	0.37	0.03	0.14	0.08
cis_mRNA_overlap	0	Lnc-CompleteIn-mRNAIntron	n325768	NM_007106_dup1	chr13	30402814	30403619	435	-	chr13	30338545	30424820	4346	-	*	0.7748	1	0.75	1.93	0.38	15.9	16.66	11.33
cis_mRNA_dw20k	7221	NA	n339135	NM_001520_dup1	chr16	27458990	27464714	2615	-	chr16	27471934	27561251	7107	-	*	0.9883	1	0.15	0.24	0.19	4.49	7.99	6.51
cis_mRNA_dw20k	2375	NA	n337950	NM_006901_dup1	chr15	72114640	72115963	1323	-	chr15	72118337	72410422	8704	-	*	0.9538	1	10.01	12.07	10.76	13.43	16	15.05
cis_mRNA_overlap	0	CompleteIn-LncExon	LTCONS_00015156	NM_002075_dup1	chr12	6937232	6956606	6843	+	chr12	6949375	6956557	1923	+	-1572.17	0.9498	1	2.47	3.34	4.19	0.27	0.65	0.75
cis_mRNA_dw20k	439	NA	LTCONS_00029129	NM_000286_dup1	chr17	33895109	33901376	893	+	chr17	33901814	33905656	2683	-	*	0.982	1	15.53	12.73	14.04	2.39	1.99	2.11
cis_mRNA_up10k	7683	NA	n333362	NM_004332_dup1	chr6	3110289	3111244	956	+	chr6	3118926	3153432	1548	+	*	-0.9581	-1	2.02	2.4	2.49	18.48	14.95	12
cis_mRNA_overlap	0	mRNA	LTCONS_00052337	NM_000203_dup1	chr4	977910	983116	3360	+	chr4	980785	998317	2186	+	*	-0.8544	-0.866	0.09	0.05	0.09	3.3	4.57	3.96
cis_mRNA_dw20k	1351	NA	n341856	NM_000921_dup1	chr12	20835231	20837006	1776	+	chr12	20522197	20833881	4124	+	*	0.8881	1	0.14	0.28	0.07	0.44	0.54	0.13
cis_mRNA_dw20k	14976	NA	n381047	NM_004402_dup1	chr1	3816968	3833877	1055	+	chr1	3773845	3801993	3028	+	*	0.7104	1	0	0.3	0.26	2.63	2.76	2.65
cis_mRNA_dw20k	9843	NA	n342783	NM_002519_dup1	chr11	107992258	108018275	1517	+	chr11	108028117	108093365	5938	-	*	-0.9906	-1	44.15	37.1	51.93	5.39	6.08	4.92
cis_mRNA_overlap	0	Lnc-CompleteIn-mRNAIntron	n385044	NM_000876_dup1	chr6	160514114	160517244	3131	+	chr6	160390131	160527583	9091	+	*	0.9489	1	0.6	0.41	0.24	12.27	11.7	9.92
cis_mRNA_overlap	0	mRNA	n342920	NM_002356_dup1	chr6	114178524	114182980	1611	+	chr6	114178527	114184652	4291	+	*	-0.9179	-1	68.79	81.49	92.86	112.24	108.31	87.37
cis_mRNA_overlap	0	mRNA	n410592	NM_030666_dup1	chr6	2828801	2841057	1939	-	chr6	2833733	2842081	1293	-	*	0.9611	1	0.04	0.08	0.1	1.33	2.11	2.16
cis_mRNA_dw20k	4059	NA	n340184	NM_001203_dup1	chr4	96083659	96088750	5092	-	chr4	95679128	96079601	5560	+	*	0.9489	1	0	0.68	0.38	0.2	0.49	0.28
cis_mRNA_dw20k	2448	NA	n334226	NM_002386_dup1	chr16	89989832	90001180	296	+	chr16	89984287	89987385	3099	+	*	-0.9903	-1	0.68	0.12	0.25	3.32	4.4	4.3
cis_mRNA_dw20k	9108	NA	n333732	NM_002645_dup1	chr11	17096684	17099015	347	-	chr11	17108122	17191354	8290	-	*	0.8417	1	0.13	0	0.11	3.57	2.22	2.67
cis_mRNA_dw20k	9443	NA	LTCONS_00023127	NM_001823_dup1	chr14	103976302	103976554	253	.	chr14	103985996	103989170	1431	-	*	-0.7993	-1	0.17	0.3	1.63	113.48	102.37	97.07
cis_mRNA_dw20k	7631	NA	n409333	NM_001856_dup1	chr1	32095463	32110218	1820	-	chr1	32117848	32169768	5584	-	*	0.994	0.866	8.58	13.09	13.7	0.03	0.11	0.11
cis_mRNA_overlap	0	Lnc-CompleteIn-mRNAExon	n334456	NM_004472_dup1	chr5	72743514	72743830	317	-	chr5	72742083	72744352	2270	-	*	0.9996	1	0.48	1.14	0.04	0.43	0.66	0.26
cis_mRNA_dw20k	2262	NA	n342277	NM_002053_dup1	chr1	89488180	89515726	1359	+	chr1	89517987	89531043	3050	-	*	0.7107	0.866	0.03	0	0.11	0.01	0	0.01
cis_mRNA_up10k	3270	NA	n338139	NM_002557_dup1	chr1	111973668	111975690	2023	-	chr1	111956937	111970399	2242	-	*	0.9997	1	2.53	3.65	2.44	0.89	1.43	0.83
cis_mRNA_dw20k	1622	NA	n341264	NM_000722_dup1	chr7	81575760	81577797	2037	-	chr7	81579418	82073031	3822	-	*	0.9867	1	0.65	0.77	0.73	4.61	7.8	6.28
cis_mRNA_up10k	5650	NA	n407799	NM_001947_dup1	chr3	52096110	52099128	3019	-	chr3	52082935	52090461	3235	-	*	-0.9996	-0.866	0.01	0.01	0	6.4	6.38	7.02
cis_mRNA_up10k	579	NA	n372620	NM_004397_dup1	chr11	118662550	118665346	1419	+	chr11	118618472	118661972	6227	-	*	0.9988	0.866	0.15	0.13	0.13	7.21	4.81	4.67
cis_mRNA_up10k	17	NA	n342771	NM_002180_dup1	chr11	68658747	68671303	2244	-	chr11	68671319	68708069	3954	+	*	0.9954	1	0.08	0.19	0.23	3.31	4.37	4.95
cis_mRNA_overlap	0	Lnc-CompleteIn-mRNAIntron	n341767	NM_002581_dup1	chr9	118954208	118954799	592	+	chr9	118916071	119164600	10970	+	*	0.9744	1	0.62	0.34	0.83	17.46	10.62	28.91
cis_mRNA_overlap	0	Lnc-CompleteIn-mRNAIntron	n325094	NM_002712_dup1	chr2	242107060	242108335	822	+	chr2	242089902	242122439	1293	+	*	-0.8667	-1	0.13	0.04	0.19	59.87	61.13	51.16
cis_mRNA_overlap	0	CompleteIn-LncExon	n409159	NM_001357_dup1	chr1	182808439	182857117	4673	+	chr1	182808439	182857117	4536	+	*	0.8775	1	4.97	4.87	4.34	78.33	74.55	72.12
cis_mRNA_overlap	0	Lnc-CompleteIn-mRNAIntron	n342929	NM_000876_dup1	chr6	160515069	160517244	2176	+	chr6	160390131	160527583	9091	+	*	-0.9923	-1	0.71	1.02	1.58	12.27	11.7	9.92
cis_mRNA_up10k	5773	NA	n410036	NM_000419_dup1	chr17	42472645	42580957	7037	-	chr17	42449550	42466873	3334	-	*	0.9026	1	0.74	1.09	1.03	0.68	0.82	0.74
cis_mRNA_up10k	5084	NA	n375214	NM_020982_dup1	chr16	3056586	3057374	569	-	chr16	3062457	3064506	2050	+	*	0.9994	1	0.06	0.18	0	0.12	0.26	0.04
cis_mRNA_dw20k	12431	NA	n363822	NM_005626_dup1	chr1	29459862	29461446	1166	+	chr1	29473876	29508637	2535	-	*	-0.9993	-1	1.68	1.35	1.76	11.77	13.07	11.38
cis_mRNA_overlap	0	Lnc-CompleteIn-mRNAIntron	n1315	NM_014865_dup1	chr12	6619388	6619717	330	+	chr12	6604240	6641132	4785	+	*	-0.9347	-1	693.33	668.85	604.82	2.59	3.24	3.68
cis_mRNA_dw20k	7460	NA	n410630	NM_007167_dup1	chr1	35441300	35444307	1887	-	chr1	35451766	35497569	5145	-	*	0.9913	1	0.22	0.73	0.44	4.17	4.87	4.39
cis_mRNA_dw20k	14760	NA	n367269	NM_021973_dup1	chr4	174430799	174432893	1606	+	chr4	174447652	174451378	2368	-	*	-0.9538	-1	0.04	0	0.06	0.03	0.63	0.01
cis_mRNA_overlap	0	Lnc-AntiOverlap-mRNA	LTCONS_00015662	NM_006009_dup1	chr12	49523295	49580616	211	+	chr12	49578582	49582861	1666	-	-475	0.9555	1	0.42	0	0.37	9214.39	8055.59	8750.73
cis_mRNA_dw20k	11574	NA	n385045	NM_021977_dup1	chr6	160887587	160932156	1968	-	chr6	160769425	160876014	5624	+	*	0.9767	1	0.61	0.78	0.71	0.03	0.08	0.05
cis_mRNA_dw20k	5251	NA	n338946	NM_002830_dup1	chr2	120740287	120742475	2189	+	chr2	120517207	120735037	3943	+	*	0.8982	1	2.93	3.54	3.74	6.97	7.31	7.94
cis_mRNA_overlap	0	Lnc-AntiOverlap-mRNA	LTCONS_00050427	NM_002948_dup1	chr3	23933136	23959321	3215	-	chr3	23958639	23962334	1993	+	*	0.9909	1	0.84	0.96	0.56	297.8	317.71	270.34
cis_mRNA_up10k	140	NA	LTCONS_00032024	NM_005440_dup1	chr17	41176517	41177119	603	.	chr17	41177258	41184058	4152	+	*	-0.7894	-1	0.49	0.06	0.22	22.62	26.41	22.66
cis_mRNA_overlap	0	Lnc-CompleteIn-mRNAIntron	n325442	NM_004850_dup1	chr2	11415092	11423213	1061	-	chr2	11321778	11484711	6401	-	*	0.7805	1	0.19	0.18	0.41	11.84	11.8	11.86
cis_mRNA_dw20k	8341	NA	n340579	NM_014819_dup1	chr5	108572835	108662070	5455	-	chr5	108670410	108745675	4869	-	*	0.9831	1	0.15	0.16	0.21	110.65	119.59	136.99
cis_mRNA_up10k	1087	NA	LTCONS_00072556	NM_007126_dup1	chr9	35073825	35080023	2622	-	chr9	35056065	35072739	3859	-	*	-0.8715	-1	0.64	1.66	1.22	150.7	142.84	149.92
cis_mRNA_overlap	0	Lnc-CompleteIn-mRNAIntron	n338350	NM_004831_dup1	chr19	16697719	16698796	1077	-	chr19	16685718	16739015	3184	-	*	0.9794	1	0.03	0.06	0.09	1.66	1.75	1.94
cis_mRNA_overlap	0	Lnc-AntiOverlap-mRNA	n338903	NM_004836_dup1	chr2	88838238	88875128	1747	+	chr2	88856259	88927094	4648	-	*	-0.8771	-0.866	0.04	0	0.04	8.47	10.06	7
cis_mRNA_overlap	0	CompleteIn-LncExon	n410803	NM_003307_dup1	chr21	45773484	45862964	5879	+	chr21	45773484	45862964	5876	+	*	0.822	0.866	0.01	0	0.01	0.14	0	0.06
cis_mRNA_up10k	1369	NA	LTCONS_00032043	NM_003857_dup1	chr17	74068752	74069524	773	.	chr17	74070892	74073573	1298	+	*	-0.9589	-0.866	1.28	0.94	1.42	0.05	0.15	0.05
cis_mRNA_up10k	5516	NA	n378526	NM_021959_dup1	chr6	29987547	30029417	1497	-	chr6	30034932	30038110	1664	+	*	-0.9999	-1	0.78	0.85	0.4	30.52	29.64	35.95
cis_mRNA_overlap	0	Lnc-AntiOverlap-mRNA	n410951	NM_003168_dup1	chr17	56406966	56431088	2201	+	chr17	56422539	56429563	1489	-	*	-0.9999	-1	0.29	0	0.31	30.25	32.93	30.03
cis_mRNA_overlap	0	Lnc-CompleteIn-mRNAIntron	n341376	NM_014671_dup1	chr7	157026561	157028829	2267	+	chr7	156931655	157062066	5181	+	*	-0.7639	-1	0.6	0.39	0.59	18.48	20.08	19.59

cis_mRNA_overlap	0	Lnc-CompleteIn-mRNAExon	n339248	NM_014229_dup1	chr3	10857917	10980146	1991	+	chr3	10857917	10980146	1991	+	*		1	1	1.21	1.73	1.25	1.21	1.73	1.25
cis_mRNA_dw20k	7628	NA	n339305	NM_022041_dup1	chr16	81421430	81424413	2984	+	chr16	81348571	81413803	4544	+	*		0.9218	1	4.55	5.99	5.71	7.18	7.67	7.4
cis_mRNA_up10k	1548	NA	n345672	NM_006026_dup1	chr3	129036667	129037497	601	+	chr3	129033614	129035120	1507	-	*		0.8697	1	1.34	0.67	1.27	50.7	43.88	46.77
tran	NA	NA	LTCONS_00045050	NM_002952_dup1	chr20	44269093	44269403	311	.	chr17	19349226	19350188	961	-		-652.4	0.9112	1	0.52	0.23	0.12	123.55	109.01	82.13
cis_mRNA_up10k	379	NA	LTCONS_00065120	NM_012301_dup1	chr7	79083268	79100524	12779	+	chr7	77646374	79082890	6880	-	*		0.9993	1	0.77	1.01	1.1	1.34	2.15	2.51
cis_mRNA_overlap	0	mRNA	n326561	NM_004582_dup1	chr1	76249597	76257629	369	+	chr1	76251886	76260764	1496	+	*		0.9903	1	0.42	0.48	0.38	57.54	64.13	50.37
cis_mRNA_up10k	9	NA	n378066	NM_004866_dup1	chr5	77654625	77656331	1479	-	chr5	77656339	77776562	6245	+	*		-0.9989	-1	1.1	1.3	2.03	48.96	48.52	47.26
cis_mRNA_up10k	3484	NA	LTCONS_00073702	NM_003086_dup1	chr9	139296372	139300963	2989	-	chr9	139270029	139292889	4650	-	*		0.9749	1	3.09	3.34	3.41	3.65	4.32	4.81
cis_mRNA_dw20k	15508	NA	n337886	NM_012421_dup1	chr1	40722100	40723639	1540	-	chr1	40627041	40706593	6250	+	*		-0.9766	-1	16.96	17.31	12.84	13.71	13.18	15
cis_mRNA_overlap	0	Lnc-CompleteIn-mRNAIntron	n410068	NM_004808_dup1	chr10	15196721	15197346	626	-	chr10	15147771	15210695	5004	-	*		0.9316	1	0.17	0.16	0.1	2.84	2.4	1.93
cis_mRNA_up10k	140	NA	n339311	NM_003791_dup1	chr16	84150656	84151176	521	+	chr16	84087368	84150517	4347	-	*		0.9199	1	1.64	1.17	1.02	14.19	9.96	3.65
cis_mRNA_dw20k	10272	NA	n406872	NM_002887_dup1	chr5	167956582	167957639	1058	+	chr5	167913463	167946311	2140	+	*		-0.9884	-1	77.45	73.74	76.27	53.72	57.1	55.25
cis_mRNA_overlap	0	Lnc-CompleteIn-mRNAIntron	n339768	NM_006315_dup1	chr4	744880	745418	539	+	chr4	699573	764428	5666	+	*		-0.9014	-1	0.27	0.13	0.67	18.24	19.51	17.57
cis_mRNA_dw20k	11122	NA	n345338	NM_005792_dup1	chr16	82168723	82170646	625	-	chr16	82181767	82203829	1101	-	*		-0.9564	-1	0.12	0.16	0.05	15.89	13.37	17.42
cis_mRNA_dw20k	2800	NA	n372243	NM_000375_dup1	chr10	127473652	127474348	309	-	chr10	127477147	127511837	1354	-	*		0.9544	1	0.52	0.24	0.23	33.11	31.33	30.44
tran	NA	NA	LTCONS_00063790	NM_001000_dup1	chr6	74082662	74083035	374	.	chrX	118920469	118925606	401	-		-743	0.9037	1	0.93	0.85	0.37	634.24	543.34	467.3
cis_mRNA_overlap	0	mRNA	n410582	NM_003804_dup1	chr6	3087409	3112321	4060	+	chr6	3077058	3115421	3864	+	*		0.9986	1	0.06	0.1	0.09	2.9	3.6	3.46
cis_mRNA_dw20k	11269	NA	n335677	NM_014641_dup1	chr6	30655827	30656316	490	-	chr6	30667584	30685458	7368	-	*		-0.9933	-1	0.97	0	1.3	4.36	5.21	4.22
cis_mRNA_overlap	0	Lnc-CompleteIn-mRNAIntron	n338090	NM_003567_dup1	chr1	94060502	94062345	1844	-	chr1	94027348	94147385	3171	-	*		1	1	0	0.03	0.02	0.49	0.73	0.65
cis_mRNA_up10k	262	NA	n409709	NM_002912_dup1	chr6	111804675	111899383	2195	+	chr6	111620234	111804414	10701	-	*		-0.7941	-1	5.76	2.81	3.94	9.89	11.81	9.9
cis_mRNA_overlap	0	Lnc-AntiOverlap-mRNA	n410990	NM_003827_dup1	chr19	47987539	48004854	656	+	chr19	47990891	48018497	1842	-	*		-0.8961	-1	0.45	0	0.22	11.82	15.36	12.12
cis_mRNA_dw20k	661	NA	n381240	NM_005105_dup1	chr1	145514196	145515899	1011	-	chr1	145507557	145513536	4960	+	*		0.9502	1	1.09	1.99	1.73	41.46	43.98	42.55
cis_mRNA_up10k	9092	NA	n381240	NM_003637_dup1	chr1	145514196	145515899	1011	-	chr1	145524990	145543868	5170	+	*		0.9936	1	1.09	1.99	1.73	0.23	0.34	0.32
cis_mRNA_up10k	294	NA	n381256	NM_002966_dup1	chr1	151967007	152015250	4109	+	chr1	151955386	151966714	1055	-	*		0.9105	0.866	0.07	0.12	0.12	30.77	50.82	65.07
cis_mRNA_overlap	0	mRNA-AntiCompleteIn-LncIntron	n381256	NM_005620_dup1	chr1	151967007	152015250	4109	+	chr1	152004982	152009511	594	-	*		0.6658	0.866	0.07	0.12	0.12	0	0.06	0.28
cis_mRNA_dw20k	3038	NA	n345712	NM_004833_dup1	chr1	159026258	159029238	1942	-	chr1	159032275	159046647	1484	-	*		0.9747	1	0.04	0.21	0.06	0.51	0.87	0.63
cis_mRNA_overlap	0	Lnc-AntiOverlap-mRNA	n409329	NM_003769_dup1	chr12	120884241	120901556	4346	+	chr12	120899471	120907558	1163	-	*		0.6818	1	0.55	1.76	2.1	36.45	36.5	39.12
cis_mRNA_dw20k	2935	NA	n338835	NM_001042603_dup1	chr12	383123	386289	3167	-	chr12	389223	498620	10835	-	*		0.9816	1	1.3	1.71	1.87	8.12	8.74	9.25
cis_mRNA_overlap	0	Lnc-CompleteIn-mRNAExon	n339927	NM_014643_dup1	chr18	74072230	74207146	6031	-	chr18	74069637	74207146	8626	-	*		-0.9845	-1	2.32	1.97	1.36	0	0.14	0.27
cis_mRNA_overlap	0	Lnc-AntiOverlap-mRNA	n380219	NM_014729_dup1	chr8	60031599	60032590	692	+	chr8	59717977	60031767	4131	-	*		0.9885	1	0.42	0.29	0.65	6.59	5.82	9.29
cis_mRNA_dw20k	2685	NA	n372462	NM_003904_dup1	chr11	116645826	116646592	598	-	chr11	116649276	116658739	1778	-	*		0.9653	1	11.9	9.24	10.71	124.11	78.03	114.51
cis_mRNA_dw20k	3514	NA	n408154	NM_003857_dup1	chr17	74077086	74099652	4822	-	chr17	74070892	74073573	1298	+	*		-0.8771	-0.866	1.19	0	2.29	0.05	0.15	0.05
cis_mRNA_dw20k	7457	NA	n406530	NM_014238_dup1	chr17	25958174	25976586	1597	+	chr17	25799036	25950718	4489	+	*		0.7046	0.866	0.04	0.04	0	1.17	1.67	0.99
cis_mRNA_up10k	35	NA	n386361	NM_003715_dup1	chr4	76648698	76649795	999	-	chr4	76649829	76735366	3831	+	*		-0.8762	-1	1.58	1.67	1.93	22.07	21.15	20.76
cis_mRNA_dw20k	2664	NA	n375951	NM_004163_dup1	chr18	52559875	52562337	1040	+	chr18	52495840	52557212	1336	+	*		0.7741	1	0.95	6.48	4.8	0.48	2.54	0.62
cis_mRNA_dw20k	4608	NA	n325400	NM_003385_dup1	chr2	17842313	17844906	586	-	chr2	17721807	17837706	2005	+	*		-0.8878	-0.866	0.06	0	0.06	2.91	4.01	1.97
cis_mRNA_overlap	0	Lnc-CompleteIn-mRNAIntron	n332704	NM_005885_dup1	chr5	10366505	10366746	242	+	chr5	10353828	10435491	4611	+	*		-0.9841	-1	0.18	0	0.63	36.1	39.46	31.9
cis_mRNA_up10k	130	NA	LTCONS_00029614	NM_003647_dup1	chr17	54903823	54911331	7509	+	chr17	54911460	54946036	7768	+	*		-0.8286	-0.866	0.11	0.04	0.04	4.43	4.86	5.41
cis_mRNA_dw20k	7798	NA	n335619	NM_002755_dup1	chr15	66791679	66792425	343	-	chr15	66679211	66783882	2586	+	*		0.8793	1	0	0.44	0.2	11.89	13.48	13.34
cis_mRNA_up10k	124	NA	n365904	NM_003458_dup1	chr3	49586739	49591799	2603	-	chr3	49591922	49708982	15959	+	*		0.8512	0.866	0.14	0.14	0.28	2.68	2.26	3.06
cis_mRNA_dw20k	1618	NA	n409172	NM_012288_dup1	chr6	52284994	52360583	6839	+	chr6	52362200	52441862	7057	-	*		0.979	1	3.35	4.02	4.27	0.31	0.43	0.53
cis_mRNA_overlap	0	mRNA	n336906	NM_003827_dup1	chr19	47995313	48010399	717	-	chr19	47990891	48018497	1842	-	*		0.9017	1	0	0.18	0.09	11.82	15.36	12.12
cis_mRNA_dw20k	19798	NA	n386409	NM_001163391_dup1	chr6	28334982	28336930	1117	+	chr6	28356727	28367544	3519	-	*		-0.9958	-1	2.86	2.46	2.02	3.01	3.41	3.73
cis_mRNA_dw20k	6469	NA	n364831	NM_012293_dup1	chr2	1624282	1629191	1641	-	chr2	1635659	1748291	6821	-	*		-0.9999	-1	0.81	1.01	1.04	10.74	10.29	10.23
cis_mRNA_dw20k	9177	NA	n335652	NM_004260_dup1	chr8	145726920	145727491	572	-	chr8	145736667	145743210	3816	-	*		0.9709	1	0.39	1	0.69	1.13	1.91	1.68
cis_mRNA_dw20k	11812	NA	n344962	NM_006304_dup1	chr7	96266552	96306268	4822	+	chr7	96318079	96339203	498	-	*		-0.8289	-0.866	0.1	0.13	0.1	28.81	28.34	29.41
tran	NA	NA	LTCONS_00055968	NM_014875_dup1	chr5	60452705	60453538	834	+	chr1	200520625	200589862	7277	-		-611.79	-0.8299	-1	2.23	1.83	1.61	0.14	0.17	0.42
cis_mRNA_dw20k	8229	NA	n363714	NM_003326_dup1	chr1	173143439	173144642	690	-	chr1	173152870	173176471	3493	-	*		1	1	0.05	0.05	0	0.17	0.17	0.13
cis_mRNA_up10k	5273	NA	n323786	NM_002910_dup1	chrX	153215504	153216581	341	-	chrX	153200722	153210232	1471	-	*		-0.9704	-1	0.7	0	0.21	2.03	3.74	2.86
cis_mRNA_dw20k	17180	NA	n344896	NM_002754_dup1	chr6	36125021	36147484	1405	+	chr6	36098262	36107842	1888	+	*		-0.8109	-0.866	0.02	0	0.02	0.11	0.28	0.21
cis_mRNA_overlap	0	Lnc-AntiOverlap-mRNA	n378371	NM_006516_dup1	chr1	43424720	43449029	1124	+	chr1	43391046	43424847	3670	-	*		0.9973	1	0.24	0.06	0.18	10.81	9.12	10.36
cis_mRNA_overlap	0	AntiCompleteIn-mRNAIntron	n383193	NM_003110_dup1	chr17	45976152	45995938	1714	-	chr17	45973516	46006323	3112	+	*		0.9987	1	0.12	0.59	0.31	0.69	1.08	0.83
cis_mRNA_overlap	0	Lnc-CompleteIn-mRNAIntron	n382014	NM_005780_dup1	chr13	40100016	40107556	7541	-	chr13	39917029	40177356	2158	-	*		0.9943	1	0.09	0.34	0.43	6.91	19.43	21.85
cis_mRNA_dw20k	1028	NA	LTCONS_00045965	NM_145858_dup1	chr21	34949869	34960620	2945	-	chr21	34961647	35014160	1711	-	*		-0.9982	-1	1.68	2.58	2.14	17.93	13.66	15.97
cis_mRNA_overlap	0	Lnc-AntiOverlap-mRNA	n379282	NM_002841_dup1	chr3	62249717	62355005	502	-	chr3	61547243	62280573	6648	+	*		-0.9755	-1	0.23	0	0.16	5.23	10.45	5.69
cis_mRNA_dw20k	8786	NA	n346502	NM_006056_dup1	chr2	232371883	232379086	637	-	chr2	232387871	232395182	3274	-	*		0.9987	1	0.27	0.35	0.47	0.06	0.1	0.15
cis_mRNA_dw20k	1589	NA	n340903	NM_003447_dup1	chr6	28058929	28063493	3711	+	chr6	28048482	28057341	2378	+	*		0.9576	1	0.22	0.16	0.21	0.75	0.52	0.65
cis_mRNA_up10k	8235	NA	n342529	NM_005128_dup1	chr21	37504065	37528605	1499	-	chr21	37536839	37667034	7689	+	*		-0.9997	-0.866	0.02	0.06	0.06	3.44	2.4	2.43

cis_mRNA_up10k	933	NA	n339824	NM_004775_dup1	chr18	29265618	29266786	1169	+	chr18	29202209	29264686	4814	-	*	0.9986	1	1.5	1.42	2.11	4.95	4.75	5.93
cis_mRNA_overlap	0	mRNA	n325581	NM_014232_dup1	chr17	8064685	8068086	2082	-	chr17	8062465	8066293	2155	-	*	-0.969	-1	0.37	0.33	0.42	9.05	10.17	8.48
cis_mRNA_overlap	0	mRNA	n340549	NM_005654_dup1	chr5	92929723	92930317	595	+	chr5	92919043	92929804	3210	+	*	0.9657	1	8.38	14.7	3.74	4.15	9.33	2.99
cis_mRNA_dw20k	5663	NA	LTCONS_00063820	NM_002793_dup1	chr6	170834086	170838542	4457	.	chr6	170844204	170862417	917	-	*	0.8806	1	0.16	0.1	0.14	130.2	114.86	118.37
cis_mRNA_dw20k	11528	NA	n381849	NM_014802_dup1	chr12	22477346	22589990	2345	-	chr12	22601517	22697452	4408	-	*	0.9873	1	2.45	2.93	2.17	7.89	9.5	7.43
cis_mRNA_overlap	0	Lnc-CompleteIn-mRNAExon	n335583	NM_002945_dup1	chr17	1800648	1801341	694	+	chr17	1733273	1802848	4345	+	*	-0.9817	-1	0.87	0.15	0.98	1.19	1.45	1.07
cis_mRNA_dw20k	6344	NA	n340855	NM_005493_dup1	chr6	13614343	13615387	1045	-	chr6	13621730	13711796	3113	-	*	0.9896	1	0.94	1.35	1.5	25.88	26.47	26.86
cis_mRNA_dw20k	9198	NA	n406535	NM_006013_dup1	chrX	153639877	153650063	1919	+	chrX	153626571	153630680	2318	+	*	-0.9757	-1	0.85	1.09	1.44	162.75	145.92	134.65
cis_mRNA_up10k	4737	NA	n373576	NM_004800_dup1	chr13	100143729	100148992	2796	-	chr13	100153728	100215278	2391	+	*	-0.9986	-1	1.87	2.65	2.26	60.98	58.32	59.53
cis_mRNA_up10k	4703	NA	n342828	NM_004800_dup1	chr13	100139216	100149026	1002	-	chr13	100153728	100215278	2391	+	*	0.8388	0.866	0.02	0	0.02	60.98	58.32	59.53
cis_mRNA_overlap	0	Lnc-AntiOverlap-mRNA	n325630	NM_003749_dup1	chr13	110433655	110440912	684	+	chr13	110406184	110438914	6998	-	*	0.996	1	0.32	0.63	0.8	1.57	2.41	3.05
cis_mRNA_dw20k	15238	NA	n380381	NM_004200_dup1	chr11	61263912	61267548	554	+	chr11	61282785	61348298	4532	-	*	-0.6682	-0.866	0	0.06	0.06	4.6	3.91	4.45
cis_mRNA_up10k	1373	NA	n379095	NM_005047_dup1	chr9	123606578	123607554	884	+	chr9	123578331	123605206	3387	-	*	0.9879	1	1.34	0.55	0	12.76	10.61	8.03
cis_mRNA_dw20k	8035	NA	n383805	NM_021141_dup1	chr2	217079050	217081397	2348	+	chr2	216974020	217071016	3430	+	*	0.8262	1	24.28	25.86	26.84	148.41	148.89	155.61
tran	NA	NA	LTCONS_00052236	NM_003034_dup1	chr3	94224676	94225741	1066	.	chr12	22346325	22487648	9714	-	-501.25	0.9977	1	1.02	1.53	1.41	4.04	5.1	4.92
cis_mRNA_overlap	0	AntiCompleteIn-mRNAIntron	n325437	NM_004850_dup1	chr2	11456746	11459838	373	+	chr2	11321778	11484711	6401	-	*	-0.9589	-1	35.49	41.58	35.16	11.84	11.8	11.86
cis_mRNA_dw20k	5448	NA	n367843	NM_005110_dup1	chr5	179720245	179722253	393	+	chr5	179727700	179780315	3011	-	*	0.9811	1	1.22	0.96	0.84	3.54	2.75	2
cis_mRNA_dw20k	6770	NA	n374597	NM_003134_dup1	chr15	40338158	40350627	2087	+	chr15	40327891	40331389	1126	-	*	-0.8435	-1	0.14	0.22	0.23	143.07	138.25	130.17
cis_mRNA_up10k	334	NA	n338395	NM_014810_dup1	chr1	179922319	179923575	1257	-	chr1	179923908	180084015	13456	+	*	0.8098	1	0.7	0.66	0.89	3.35	2.51	3.65
cis_mRNA_dw20k	3104	NA	n408068	NM_003677_dup1	chr12	123259056	123311927	2494	+	chr12	123237371	123255953	3061	+	*	0.8365	1	0	0.09	0.65	23.1	25.7	27.03
cis_mRNA_dw20k	5542	NA	n367426	NM_021973_dup1	chr4	174374164	174442111	3118	-	chr4	174447652	174451378	2368	-	*	0.9996	0.866	0.1	0.18	0.1	0.03	0.63	0.01
cis_mRNA_overlap	0	Lnc-AntiOverlap-mRNA	n335653	NM_003804_dup1	chr6	3076992	3078808	1817	-	chr6	3077058	3115421	3864	+	*	0.9011	1	0	0.05	0.02	2.9	3.6	3.46
cis_mRNA_dw20k	1725	NA	n339158	NM_005687_dup1	chr2	223431624	223434438	2815	-	chr2	223436162	223520827	2233	-	*	0.9999	1	1.61	1.79	1.89	27.04	28.97	29.99
cis_mRNA_up10k	963	NA	n342630	NM_004168_dup1	chr5	214013	217394	2881	-	chr5	218356	256815	2390	+	*	-0.98	-1	0.51	0.57	0.43	30.64	30.17	32.06
cis_mRNA_dw20k	18345	NA	n407588	NM_004173_dup1	chr22	21356211	21364663	2616	+	chr22	21383007	21386146	2290	-	*	-0.9086	-1	0.39	0.36	0.13	0.22	0.45	0.67
cis_mRNA_overlap	0	mRNA	LTCONS_00017286	NM_014802_dup1	chr12	22601473	22616848	4464	-	chr12	22601517	22697452	4408	-	-1529.74	0.9051	1	2.46	2.65	2.16	7.89	9.5	7.43
tran	NA	NA	LTCONS_00015661	NM_006000_dup1	chr12	49521569	49579494	874	+	chr2	220115001	220118638	1478	-	-1199.61	0.9537	1	0.61	0.67	0.51	0.49	0.92	0.25
cis_mRNA_up10k	3218	NA	n380211	NM_003295_dup1	chr13	45918514	45955352	856	+	chr13	45911304	45915297	829	-	*	-0.9929	-1	0.43	0.93	1.14	358.65	311.99	280.6
cis_mRNA_overlap	0	Lnc-CompleteIn-mRNAIntron	n334588	NM_005768_dup1	chr12	7110682	7115781	646	-	chr12	7085347	7125842	2297	-	*	0.8993	1	0.34	0.36	0.1	10.12	11.71	8.52
cis_mRNA_up10k	2708	NA	n380213	NM_005749_dup1	chr17	48944120	48986284	547	+	chr17	48939584	48941413	1830	-	*	0.9913	1	0	1.54	0.97	1.71	2.23	2.1
cis_mRNA_dw20k	17345	NA	n346486	NM_003546_dup1	chr6	27821498	27823582	1141	+	chr6	27840926	27841289	364	-	*	-0.8438	-1	0.09	0.08	0.14	0.64	1.76	0.1
cis_mRNA_dw20k	16042	NA	n346486	NM_003533_dup1	chr6	27821498	27823582	1141	+	chr6	27839623	27840099	477	-	*	-0.9956	-1	0.09	0.08	0.14	2.13	2.29	0
cis_mRNA_dw20k	12362	NA	n407467	NM_022817_dup1	chr2	239133754	239140318	1959	-	chr2	239152679	239197207	6342	-	*	0.9244	1	0.63	0.35	0.8	0.54	0.44	0.8
cis_mRNA_up10k	165	NA	n338175	NM_003090_dup1	chr15	101835624	101845942	2590	+	chr15	101821715	101835460	1059	-	*	0.9896	1	0.6	0.62	0.51	5.69	5.71	5.07
cis_mRNA_up10k	26	NA	n364842	NM_001011_dup1	chr2	3616113	3622828	2541	-	chr2	3622853	3628509	728	+	*	-0.9392	-1	1.85	2.7	2.46	306.02	264	288.82
cis_mRNA_dw20k	890	NA	n379692	NM_004604_dup1	chr16	31052374	31054276	1417	+	chr16	31044903	31051485	1386	+	*	0.9942	1	0.42	1.28	1.69	6.35	7.43	7.74
cis_mRNA_overlap	0	Lnc-CompleteIn-mRNAIntron	n324796	NM_006052_dup1	chr21	38617427	38627156	608	-	chr21	38595726	38639833	3251	-	*	-0.9957	-0.866	0	0.11	0.11	13.23	12.08	11.95
cis_mRNA_dw20k	2123	NA	n368174	NM_004779_dup1	chr5	154258474	154263999	1464	+	chr5	154238198	154256352	2483	+	*	-0.9743	-0.866	0.13	0.18	0.13	27.41	25.84	27.04
cis_mRNA_overlap	0	Lnc-CompleteIn-mRNAIntron	n324772	NM_003307_dup1	chr21	45837996	45841351	674	+	chr21	45773484	45862964	5876	+	*	-0.9908	-1	0.08	1.15	0.82	0.14	0	0.06
cis_mRNA_dw20k	1463	NA	n345676	NM_014606_dup1	chr4	89631148	89643579	714	+	chr4	89513647	89629686	4894	+	*	-0.9177	-1	0.08	0	0.18	5.06	5.66	4.92
cis_mRNA_overlap	0	mRNA	LTCONS_00009126	NM_003054_dup1	chr10	119028886	119042599	7016	+	chr10	119000584	119038941	3872	+	-1636.63	0.8158	1	0.12	0.13	0.09	0.51	2.05	0.13
cis_mRNA_dw20k	19373	NA	LTCONS_00038264	NM_021103_dup1	chr2	85153171	85192994	4478	+	chr2	85132763	85133799	482	+	*	0.8103	1	0.72	0.71	0.88	5776.14	4858.31	6303.2
cis_mRNA_up10k	106	NA	n367847	NM_013232_dup1	chr5	269973	271631	522	-	chr5	271736	315089	1135	+	*	0.699	0.866	0.21	0.13	0.13	32.56	27.82	31.34
cis_mRNA_overlap	0	mRNA	n379198	NM_014803_dup1	chr10	97889813	97917094	1579	+	chr10	97889472	97923517	8295	+	*	0.9643	1	7.78	6.33	6.02	4.7	4.33	4.06
cis_mRNA_overlap	0	mRNA	n340740	NM_003862_dup1	chr5	170846660	170883653	1012	+	chr5	170846667	170884630	1982	+	*	0.9996	1	0.28	0.08	0.36	10.58	8.66	11.25
cis_mRNA_overlap	0	mRNA	LTCONS_00066770	NM_004577_dup1	chr7	56107807	56119284	3098	-	chr7	56078744	56119268	2118	-	*	-0.9927	-1	1.07	1.48	1.72	5.93	4.45	3.11
cis_mRNA_overlap	0	Lnc-AntiOverlap-mRNA	n406964	NM_005854_dup1	chr17	40905947	40913275	3257	-	chr17	40913212	40915059	785	+	*	-0.9982	-1	0.95	2.06	0.72	1.75	1.19	1.82
cis_mRNA_up10k	4718	NA	n374073	NM_004239_dup1	chr14	92511120	92516990	701	-	chr14	92434243	92506403	8088	-	*	-0.6038	-1	0.36	0.19	0.18	8.41	8.45	8.89
cis_mRNA_dw20k	901	NA	n377056	NM_006936_dup1	chr21	46222492	46224632	1031	+	chr21	46225532	46238044	1807	-	*	0.8637	1	0	0.38	0.12	34.13	36.09	35.67
cis_mRNA_dw20k	5790	NA	n386202	NM_003437_dup1	chr19	12305853	12349425	1627	+	chr19	12273872	12300064	3018	+	*	-0.9894	-1	0	1.46	0.28	11.7	10.47	11.65
cis_mRNA_dw20k	3506	NA	n407187	NM_003804_dup1	chr6	3118926	3153432	1844	+	chr6	3077058	3115421	3864	+	*	0.9963	1	0.17	0.7	0.64	2.9	3.6	3.46
cis_mRNA_overlap	0	Lnc-CompleteIn-mRNAExon	n342407	NM_003009_dup1	chr19	48281921	48287938	796	+	chr19	48281842	48287939	876	+	*	0.7654	1	25.85	1.83	29.49	205.79	203.68	212.66
cis_mRNA_overlap	0	Lnc-AntiOverlap-mRNA	n378306	NM_002815_dup1	chr17	30748305	30772564	3030	-	chr17	30771502	30808042	1581	+	*	0.8111	1	0.01	0.06	0	27.32	27.72	26.25
cis_mRNA_up10k	353	NA	n374988	NM_014862_dup1	chr15	80695741	80696340	477	-	chr15	80696692	80890278	6559	+	*	-0.9253	-1	0.9	0.85	0.63	21.25	22.32	23.25
cis_mRNA_overlap	0	Lnc-CompleteIn-mRNAIntron	n338801	NM_004718_dup1	chr2	42583031	42584366	1336	-	chr2	42577642	42588356	1117	-	*	-0.9827	-1	0.44	0.5	0.73	96.7	96.51	81.88
cis_mRNA_dw20k	392	NA	n384600	NM_005885_dup1	chr5	10435882	10437961	2080	+	chr5	10353828	10435491	4611	+	*	0.9923	1	40.4	42.1	37.03	36.1	39.46	31.9
cis_mRNA_up10k	765	NA	n364377	NM_005399_dup1	chr1	146644893	146648626	448	+	chr1	146626685	146644129	5423	-	*	0.7148	1	1.26	1.45	2.98	20.18	22.2	22.65
cis_mRNA_up10k	120	NA	n375736	NM_003488_dup1	chr17	55156601	55162434	405	-	chr17	55162553	55198710	3968	+	*	-0.9986	-1	0.09	0.17	0.51	23.7	22.65	16.1
cis_mRNA_up10k	1656	NA	LTCONS_00058189	NM_005654_dup1	chr5	92744992	92917388	3765	-	chr5	92919043	92929804	3210	+	*	0.804	1	1.84	2.11	1.01	4.15	9.33	2.99
cis_mRNA_dw20k	6364	NA	n344717	NM_003145_dup1	chr1	155959025	155972476	2741	-	chr1	155978839	155990758	1124	-	*	0.9922	0.866	0.06	0.02	0.06	95.41	92.21	95.91
cis_mRNA_up10k	78	NA	n376545	NM_006282_dup1	chr20	43592442	43595043	2058	-	chr20	43595120	43708594	6331	+	*	-0.9125	-1	0.08	0.05	0.01	7.54	7.83	7.9
cis_mRNA_dw20k	11432	NA	n384692	NM_006909_dup1	chr5	80533384	80597378	2064	-	chr5	80256558	80521953	4078	+	*	-0.6826	-1	0.59	0.55	0.94	0.64	2.53	0.32

cis_mRNA_dw20k	19710	NA	n406833	NM_014760_dup1	chr3	10342615	10362858	1599	-	chr3	10290177	10322906	4907	+	*		-0.9969	-1	0.64	1.16	0.49	2.8	2.54	2.91
cis_mRNA_up10k	83	NA	LTCONS_00042795	NM_003108_dup1	chr2	5832069	5832717	649	.	chr2	5832799	5841517	8719	+	*		0.9953	1	25.56	12.15	19.36	102.47	56.03	77.09
cis_mRNA_up10k	2306	NA	n386398	NM_005654_dup1	chr5	92877578	92916738	3165	-	chr5	92919043	92929804	3210	+	*		0.9772	1	1.33	2.47	0.63	4.15	9.33	2.99
cis_mRNA_overlap	0	Lnc-AntiOverlap-mRNA	n377907	NM_004282_dup1	chr6	56979709	57037194	873	-	chr6	57037104	57050013	2053	+	*		-0.978	-0.866	0	0.15	0.15	3.09	2.68	2.77
cis_mRNA_dw20k	5511	NA	n324137	NM_000459_dup1	chr9	27235682	27272791	2913	-	chr9	27109147	27230172	4759	+	*		-0.866	-0.866	0.08	0.01	0.01	0	0.01	0.02
cis_mRNA_dw20k	7468	NA	n333535	NM_002883_dup1	chr22	41625501	41634148	1566	-	chr22	41641615	41682216	3021	-	*		0.6432	1	0.07	0.19	0.21	38.7	39.14	47.85
cis_mRNA_up10k	5138	NA	n340966	NM_000990_dup1	chr11	8697265	8698858	1592	-	chr11	8703995	8711419	4879	+	*		-0.9758	-1	0.51	0.52	0.55	37.27	34.59	31.28
cis_mRNA_dw20k	18153	NA	n377897	NM_005819_dup1	chr1	180918835	180924023	1440	-	chr1	180942175	180992046	4536	-	*		-0.9797	-1	0.48	0.64	0.32	10.67	10.15	11.76
cis_mRNA_up10k	602	NA	n379244	NM_003143_dup1	chr7	141423702	141437575	670	-	chr7	141438176	141450257	628	+	*		0.9711	1	0.09	0	0.17	91.69	85.66	93.76
cis_mRNA_overlap	0	mRNA	n410497	NM_004623_dup1	chr1	55107427	55208328	6470	+	chr1	55181495	55208330	2395	+	*		0.9634	1	0	0.04	0.03	7.63	8.22	7.93
cis_mRNA_up10k	662	NA	n376260	NM_002866_dup1	chr19	18315535	18316371	281	+	chr19	18307611	18314874	1501	-	*		-0.8137	-1	0.15	0.13	0.26	29.43	31.16	28.73
cis_mRNA_dw20k	12842	NA	n372307	NM_005479_dup1	chr10	99094513	99098753	1138	+	chr10	99079022	99081672	2651	+	*		-0.9925	-1	0.25	0.59	0.69	3.62	2.6	2.48
cis_mRNA_dw20k	5708	NA	n375219	NM_001098814_dup1	chr16	4230069	4233668	519	-	chr16	4239375	4292081	4214	-	*		0.9245	1	0.14	0.2	0.13	0.01	0.02	0
cis_mRNA_overlap	0	CompleteIn-LncExon	n408020	NM_001003681_dup1	chr22	35653445	35691800	4251	+	chr22	35653445	35691800	4136	+	*		-0.9998	-1	0.45	0.55	0.78	2.33	2.25	2.08
cis_mRNA_overlap	0	Lnc-CompleteIn-mRNAExon	n341602	NM_014846_dup1	chr8	126036509	126104005	4103	-	chr8	126036502	126104061	4166	-	*		-0.9978	-1	6.46	5.83	5.71	0	1.45	1.87
cis_mRNA_overlap	0	Lnc-AntiOverlap-mRNA	n384245	NM_005875_dup1	chr3	40214644	40351182	2099	-	chr3	40351173	40353915	1012	+	*		0.9223	1	0	0.07	0.09	14.5	16.2	18.5
cis_mRNA_dw20k	4210	NA	n340913	NM_000199_dup1	chr17	78174029	78178870	4412	-	chr17	78183079	78194199	2768	-	*		0.9889	1	0.51	0.57	0.55	2.19	2.55	2.48
cis_mRNA_up10k	501	NA	n365085	NM_004850_dup1	chr2	11485211	11489408	831	+	chr2	11321778	11484711	6401	-	*		-0.9762	-1	0.09	0.16	0.08	11.84	11.8	11.86
cis_mRNA_up10k	352	NA	n378845	NM_002912_dup1	chr6	111804765	111897944	784	+	chr6	111620234	111804414	10701	-	*		-0.7099	-1	2.18	1.32	1.55	9.89	11.81	9.9
cis_mRNA_up10k	2041	NA	n379717	NM_005654_dup1	chr5	92877578	92917003	2868	-	chr5	92919043	92929804	3210	+	*		0.9982	1	0.2	0.64	0.06	4.15	9.33	2.99
cis_mRNA_up10k	2404	NA	n379627	NM_005853_dup1	chr16	54952798	54962708	643	-	chr16	54965111	54968397	2064	+	*		-0.9998	-1	1.62	0	0.91	2.1	3.22	2.57
cis_mRNA_dw20k	6622	NA	n339341	NM_003420_dup1	chr3	44708904	44710856	1953	+	chr3	44690233	44702283	2649	+	*		0.9775	1	3.5	4.86	4.52	9.54	11	10.95
cis_mRNA_dw20k	2886	NA	n407589	NM_006767_dup1	chr22	21356211	21357561	1263	+	chr22	21336558	21353326	4315	+	*		-0.822	-1	0.69	1.4	0.63	12.35	10.86	14.92
cis_mRNA_dw20k	8072	NA	LTCONS_00027272	NM_020982_dup1	chr16	3072577	3074415	1605	-	chr16	3062457	3064506	2050	+	*		-0.9508	-1	0.33	0.17	0.34	0.12	0.26	0.04
cis_mRNA_overlap	0	mRNA-AntiCompleteIn-LncIntron	LTCONS_00026311	NM_004765_dup1	chr16	30885353	30906541	2760	+	chr16	30899116	30905399	858	-	*		-0.9762	-1	1.06	1.51	1.03	30.23	22.01	33.42
cis_mRNA_up10k	7403	NA	n346123	NM_014811_dup1	chr9	138354460	138364246	5586	-	chr9	138371648	138380739	4932	+	*		-0.9808	-1	0.19	0.13	0.26	0.55	1.17	0.19
cis_mRNA_overlap	0	Lnc-AntiOverlap-mRNA	n338342	NM_005858_dup1	chr19	15489887	15491657	1771	+	chr19	15464332	15490612	3543	-	*		0.9839	1	0.02	0.05	0.12	5.33	5.95	6.68
cis_mRNA_up10k	130	NA	LTCONS_00031418	NM_003647_dup1	chr17	54903823	54911331	7509	-	chr17	54911460	54946036	7768	+	*		0.8981	1	2.84	3.41	3.51	4.43	4.86	5.41
cis_mRNA_overlap	0	Lnc-CompleteIn-mRNAIntron	n325144	NM_004807_dup1	chr2	129054535	129055620	357	-	chr2	129023054	129076171	3952	-	*		0.976	1	0.11	0.7	0.1	101.92	136.33	90.93
cis_mRNA_up10k	4703	NA	n373697	NM_004800_dup1	chr13	100139216	100149026	1002	-	chr13	100153728	100215278	2391	+	*		0.8388	0.866	0.02	0	0.02	60.98	58.32	59.53
cis_mRNA_dw20k	14622	NA	n325935	NM_003019_dup1	chr10	81679934	81682875	1424	+	chr10	81697496	81708861	1283	-	*		-0.8881	-1	0.46	0.38	0.26	0.35	0.64	0.7
cis_mRNA_up10k	115	NA	LTCONS_00059110	NM_007118_dup1	chr5	14142134	14143715	1582	.	chr5	14143829	14509458	10227	+	*		0.9791	1	0.63	0.52	0.76	12.29	11.23	15.07
cis_mRNA_dw20k	6037	NA	n405458	NM_003348_dup1	chr12	93771746	93796052	3776	+	chr12	93802088	93836026	2552	-	*		0.8163	1	16.58	17.05	16.9	65.74	68.41	66.07
cis_mRNA_overlap	0	AntiCompleteIn-mRNAExon	LTCONS_00016739	NM_021009_dup1	chr12	125396431	125398212	642	+	chr12	125396192	125399587	2584	-		-1377.4	0.9705	1	0.11	0.21	0.15	418.23	433.62	427.65
cis_mRNA_up10k	5967	NA	n378089	NM_021959_dup1	chr6	30003207	30028966	1706	-	chr6	30034932	30038110	1664	+	*		0.7465	1	0.09	0.05	0.1	30.52	29.64	35.95
cis_mRNA_up10k	469	NA	LTCONS_00033254	NM_004775_dup1	chr18	29265154	29265516	363	.	chr18	29202209	29264686	4814	-	*		-0.9974	-1	0.86	0.88	0.67	4.95	4.75	5.93
cis_mRNA_dw20k	11432	NA	n384691	NM_006909_dup1	chr5	80533384	80597378	2730	-	chr5	80256558	80521953	4078	+	*		-0.9973	-1	0.48	0.12	0.58	0.64	2.53	0.32
cis_mRNA_up10k	1227	NA	n376820	NM_002862_dup1	chr20	25225790	25227480	590	-	chr20	25228706	25278648	4131	+	*		-0.7076	-1	0.19	0.23	0	68.96	63.78	69.59
cis_mRNA_up10k	1724	NA	n375821	NM_003955_dup1	chr17	76357881	76360723	247	+	chr17	76352859	76356158	2734	-	*		0.7609	1	0.14	0.16	0	2.72	5.07	2
tran	NA	NA	LTCONS_00073887	NM_003440_dup1	chrUn_gl000223	1	25645	1722	-	chr12	133657037	133684260	3067	+		-915.19	-0.9535	-1	0.62	0.66	0.65	7.16	6.24	6.72
cis_mRNA_dw20k	19094	NA	LTCONS_00067915	NM_004911_dup1	chr7	148680849	148681061	213	.	chr7	148700154	148725782	2952	-	*		-0.8185	-1	1.03	1.31	0.37	32.45	31.16	32.68
cis_mRNA_up10k	4326	NA	n377376	NM_014246_dup1	chr22	46937392	46944093	2949	+	chr22	46756731	46933067	11389	-	*		1	1	0.02	0.03	0.02	0.03	0.05	0.03
cis_mRNA_dw20k	9510	NA	n410072	NM_006315_dup1	chr4	773937	775636	1700	-	chr4	699573	764428	5666	+	*		0.9883	1	9.19	11.85	8.51	18.24	19.51	17.57
cis_mRNA_up10k	5050	NA	n375619	NM_004645_dup1	chr17	55043460	55048783	669	+	chr17	55015560	55038411	2634	-	*		-0.9998	-1	0.11	0.1	0	12.97	13.53	17.9
cis_mRNA_overlap	0	Lnc-CompleteIn-mRNAExon	n335661	NM_005811_dup1	chr12	56137097	56143584	1144	+	chr12	56137064	56146665	4260	+	*		-0.9551	-1	0.08	0.24	0	12.75	11.47	14.64
cis_mRNA_dw20k	4057	NA	n335660	NM_003347_dup1	chr22	21982379	21982768	390	-	chr22	21921957	21978323	2907	+	*		-0.9827	-1	3.33	1.18	2.52	26.63	29.32	28.08
cis_mRNA_overlap	0	mRNA	n324177	NM_014671_dup1	chr7	156951686	156985171	4049	+	chr7	156931655	157062066	5181	+	*		0.8189	1	0.11	0.25	0.13	18.48	20.08	19.59
cis_mRNA_up10k	87	NA	n338716	NM_000321_dup1	chr13	48870649	48877797	1143	-	chr13	48877883	49056026	4772	+	*		-0.7823	-1	0.11	0.08	0.01	11.6	12.34	12.41
cis_mRNA_up10k	117	NA	n372812	NM_003141_dup1	chr11	4415042	4432109	835	+	chr11	4406127	4414926	1929	-	*		0.9992	1	0	0.12	0.08	9.06	9.63	9.46
cis_mRNA_overlap	0	mRNA	n326348	NM_006917_dup1	chr1	165411778	165431650	971	-	chr1	165370159	165414592	2195	-	*		0.7123	1	0.12	0.04	0.11	28.2	17.45	19.22
cis_mRNA_dw20k	5723	NA	n409778	NM_006762_dup1	chr1	31191619	31199593	2050	+	chr1	31205315	31230683	2240	-	*		-0.866	-1	0.13	0.15	0.12	0.15	0.13	0.23
cis_mRNA_up10k	789	NA	n375477	NM_007146_dup1	chr17	56066403	56070840	4056	-	chr17	56048910	56065615	4633	-	*		0.9962	0.866	0.79	0.79	0	17.06	17.33	14.52
cis_mRNA_dw20k	18005	NA	n384929	NM_002754_dup1	chr6	36125846	36163947	1098	-	chr6	36098262	36107842	1888	+	*		-0.8109	-0.866	0.17	0.11	0.17	0.11	0.28	0.21
cis_mRNA_up10k	1866	NA	LTCONS_00033210	NM_007345_dup1	chr18	74516930	74534251	4811	-	chr18	74536116	74682683	8124	+	*		0.9959	1	0.72	1.08	1.15	1.54	1.63	1.66
cis_mRNA_up10k	7789	NA	n376347	NM_004230_dup1	chr19	10349736	10361691	288	-	chr19	10332109	10341948	3584	-	*		-0.9912	-1	0	0.26	0.13	5.18	4.27	4.62
cis_mRNA_dw20k	12958	NA	n338063	NM_002872_dup1	chr22	37602686	37608353	1682	-	chr22	37621310	37640305	1468	-	*		0.9165	1	0.51	0	0.39	0.05	0	0.02
cis_mRNA_up10k	178	NA	n406618	NM_007373_dup1	chr10	112658488	112679124	2118	-	chr10	112679301	112773425	3947	+	*		0.7579	1	1.91	2.19	1.79	39.44	39.58	36.47
cis_mRNA_overlap	0	Lnc-AntiOverlap-mRNA	n379329	NM_003877_dup1	chr12	93936239	93965544	3325	-	chr12	93963598	93969978	2210	+	*		-0.947	-1	0.31	0.4	0.29	1.36	1.24	1.47
cis_mRNA_dw20k	8822	NA	n377653	NM_006056_dup1	chr2	232373137	232379050	1282	-	chr2	232387871	232395182	3274	-	*		-0.9947	-1	0.71	0.55	0.41	0.06	0.1	0.15
cis_mRNA_dw20k	1719	NA	n344704	NM_003683_dup1	chr21	45225701	45238644	3132	-	chr21	45209418	45223983	1807	+	*		0.859	0.866	0	0.01	0.01	15.06	15.67	16.31
cis_mRNA_overlap	0	mRNA	LTCONS_00033278	NM_005860_dup1	chr19	680761	683419	2117	+	chr19	676389	683392	2511	+		-1562.1	-0.6515	-1	3.89	2.82	4.07	3.34	3.35	3.18
cis_mRNA_up10k	7	NA	n341308	NM_003941_dup1	chr7	123389122	123391476	2109	+	chr7	123321989	123389116	4430	-	*		-0.7329	-1	1.26	1.06	0.26	6.44	7.48	7.62

cis_mRNA_overlap	0	Lnc-AntiOverlap-mRNA	n324777	NM_005049_dup1	chr21	45544588	45549600	2323	-	chr21	45527208	45551063	3260	+	*		-0.797	-1	0.04	0.01	0	5.75	5.85	6.35
cis_mRNA_overlap	0	Lnc-CompleteIn-mRNAIntron	n324176	NM_014671_dup1	chr7	156986777	156989032	546	+	chr7	156931655	157062066	5181	+	*		0.9543	0.866	0	0.06	0.06	18.48	20.08	19.59
cis_mRNA_up10k	6350	NA	n364428	NM_014652_dup1	chr1	44404330	44406129	364	-	chr1	44412478	44433694	4027	+	*		-1	-1	1.18	0.59	0.77	5.03	8.16	7.23
cis_mRNA_dw20k	4484	NA	n368598	NM_005649_dup1	chr5	178121721	178134039	835	-	chr5	178138522	178157703	2555	-	*		-0.9914	-0.866	0	0.04	0.04	4.63	3.3	3.08
cis_mRNA_dw20k	3718	NA	n375822	NM_005052_dup1	chr17	79995797	79997235	527	+	chr17	79989532	79992080	1060	+	*		-0.9837	-1	0.07	0.13	0	71.65	66.39	83.82
cis_mRNA_dw20k	12912	NA	n324612	NM_001069_dup1	chr6	3127977	3140989	1506	-	chr6	3153900	3157783	1621	-	*		0.9859	1	0.11	0.09	0.13	725.91	676.38	816.6
cis_mRNA_dw20k	1014	NA	n337746	NM_013309_dup1	chr15	45771811	45773663	1853	-	chr15	45774676	45815002	4287	-	*		0.9019	1	4.74	4.35	3.94	3.78	3.72	2.92
cis_mRNA_overlap	0	Lnc-AntiOverlap-mRNA	n379272	NM_007136_dup1	chr3	113933238	113955051	3023	+	chr3	113953478	113956425	2948	-	*		0.9042	0.866	0.07	0.14	0.11	0.01	0.03	0.03
tran	NA	NA	LTCONS_00076603	NM_003932_dup1	chrX	92543004	92543282	279	.	chr22	41220599	41252687	3196	-		-562.2	-0.9623	-1	0.16	0	0.26	85.16	87.58	81.13
cis_mRNA_dw20k	10509	NA	LTCONS_00037048	NM_001009_dup1	chr19	58916679	58919763	2203	-	chr19	58898636	58906171	741	+	*		-0.9423	-1	1.37	1.73	1.89	379.13	344.28	290.48
cis_mRNA_dw20k	1984	NA	n387326	NM_003601_dup1	chr4	144480625	144482612	1988	+	chr4	144434616	144478642	7926	+	*		-0.9454	-1	1.04	1.26	1.54	31.32	31.15	30.04
cis_mRNA_dw20k	268	NA	LTCONS_00031118	NM_003632_dup1	chr17	40852278	40857101	3759	-	chr17	40834632	40852011	5454	+	*		0.813	1	1.82	2.6	3.96	21.42	27.12	27.51
cis_mRNA_overlap	0	Lnc-AntiOverlap-mRNA	n379750	NM_003703_dup1	chr4	2936626	2949400	1999	+	chr4	2939663	2965118	2918	-	*		-0.888	-1	0.08	0.17	0.25	0.45	0.42	0.11
cis_mRNA_dw20k	12893	NA	n372056	NM_005479_dup1	chr10	99094564	99102955	557	+	chr10	99079022	99081672	2651	+	*		-0.9954	-0.866	0	0.06	0.06	3.62	2.6	2.48
cis_mRNA_overlap	0	AntiCompleteIn-mRNAIntron	n340502	NM_004822_dup1	chr17	9074385	9082435	5649	-	chr17	8924859	9147317	5954	+	*		-0.6547	-0.866	0.01	0.01	0.02	1.38	0.86	0.73
cis_mRNA_dw20k	17713	NA	n376476	NM_000455_dup1	chr19	1246146	1248181	928	-	chr19	1205798	1228434	3276	+	*		-0.8985	-0.866	0.37	0.51	0.62	10.95	10.65	10.65
cis_mRNA_dw20k	4902	NA	n339329	NM_001142864_dup1	chr16	88775365	88776845	1481	-	chr16	88781746	88851372	7833	-	*		0.768	0.866	0	0.02	0.02	0.8	1.36	2.4
cis_mRNA_overlap	0	CompleteIn-LncExon	n407779	NM_000380_dup1	chr9	100437191	100459691	1700	-	chr9	100437191	100459691	1469	-	*		0.9238	1	1.73	2.04	1.27	7.08	7.62	6.9
cis_mRNA_overlap	0	mRNA	n335523	NM_003901_dup1	chr10	72575718	72580258	552	+	chr10	72575704	72640946	5783	+	*		0.984	1	0.15	0.19	0.06	9.97	10.12	9.02
cis_mRNA_up10k	3971	NA	n370082	NM_003439_dup1	chr7	99605931	99609249	266	+	chr7	99613219	99635403	5494	+	*		0.7718	1	0.12	0.28	0.23	18.68	19.8	18.74
cis_mRNA_overlap	0	Lnc-AntiOverlap-mRNA	n339342	NM_014240_dup1	chr3	45719657	45730374	2300	-	chr3	45636323	45722755	6284	+	*		-0.9497	-0.866	0.04	0.12	0.04	2	1.83	2.08
cis_mRNA_up10k	128	NA	n369815	NM_006980_dup1	chr7	91510143	91515201	308	+	chr7	91502021	91510016	2163	-	*		-0.6611	-0.866	0	0.12	0.12	8.4	7.68	8.25
cis_mRNA_overlap	0	AntiCompleteIn-mRNAExon	n337862	NM_001078171_dup1	chrX	134166392	134167544	1153	-	chrX	134166333	134167576	1244	+	*		0.6177	0.866	1.03	1.19	1.19	48.63	50.34	59.83
cis_mRNA_overlap	0	Lnc-AntiOverlap-mRNA	n407266	NM_014860_dup1	chr2	27851863	27873713	1727	+	chr2	27873679	27886449	4261	-	*		-0.8572	-1	0.88	0.65	2.2	6.46	7.96	5.6
cis_mRNA_overlap	0	Lnc-AntiOverlap-mRNA	n410673	NM_004855_dup1	chr15	55647421	55790782	4838	-	chr15	55611133	55647846	2197	+	*		0.7973	0.866	0.98	1.57	1.95	1.55	1.55	2.38
cis_mRNA_up10k	3051	NA	n375745	NM_005994_dup1	chr17	59470821	59474207	1126	-	chr17	59477257	59486827	3380	+	*		0.8285	1	4.5	3.49	4.81	40.7	36.8	53.42
cis_mRNA_overlap	0	mRNA	n407163	NM_005439_dup1	chr12	6857158	6862636	1888	-	chr12	6857158	6862082	1391	-	*		0.9847	1	28.05	31.1	32.02	38.99	42.84	43.06
cis_mRNA_overlap	0	Lnc-AntiOverlap-mRNA	n339674	NM_003286_dup1	chr20	39726969	39766640	1458	-	chr20	39657462	39753127	3734	+	*		-0.9961	-1	0.12	0.24	0.07	4.91	4.35	5.06
cis_mRNA_overlap	0	Lnc-CompleteIn-mRNAIntron	n324175	NM_014671_dup1	chr7	156998749	157002296	292	+	chr7	156931655	157062066	5181	+	*		0.9543	0.866	0	0.25	0.25	18.48	20.08	19.59
cis_mRNA_dw20k	3506	NA	n407189	NM_003804_dup1	chr6	3118926	3153432	1776	+	chr6	3077058	3115421	3864	+	*		0.8113	1	2.21	4.37	2.73	2.9	3.6	3.46
cis_mRNA_dw20k	3154	NA	n372317	NM_007373_dup1	chr10	112776578	112779659	589	+	chr10	112679301	112773425	3947	+	*		0.9453	1	1.81	2.38	0.83	39.44	39.58	36.47
cis_mRNA_dw20k	4986	NA	n345476	NM_005819_dup1	chr1	180931250	180937190	990	-	chr1	180942175	180992046	4536	-	*		-0.9999	-1	0.14	0.16	0.1	10.67	10.15	11.76
cis_mRNA_dw20k	19526	NA	n341630	NM_005672_dup1	chr8	143783670	143808391	5684	-	chr8	143761874	143764145	1023	+	*		0.843	0.866	0.16	0.03	0.1	0.31	0.03	0.03
cis_mRNA_up10k	225	NA	LTCONS_00060465	NM_012288_dup1	chr6	52442086	52448866	2547	+	chr6	52362200	52441862	7057	-	*		0.9226	1	1.23	1.34	1.35	0.31	0.43	0.53
cis_mRNA_dw20k	5054	NA	n345574	NM_004846_dup1	chr2	233438973	233448354	2542	+	chr2	233415357	233433920	1009	+	*		0.9674	1	11.72	11.17	12.75	18.25	16.13	19.84
cis_mRNA_up10k	9944	NA	n339363	NM_004704_dup1	chr3	51985865	51987918	2052	-	chr3	51967446	51975922	1543	-	*		-0.7874	-1	0.52	1.05	0.71	14.03	12.8	12.83
cis_mRNA_up10k	3227	NA	n325553	NM_003559_dup1	chr17	36959384	36964273	2141	-	chr17	36921944	36956158	5732	-	*		-0.7719	-0.866	0.63	1.05	0.63	110.95	102.61	126.07
cis_mRNA_overlap	0	Lnc-AntiOverlap-mRNA	n339984	NM_003380_dup1	chr10	17256245	17271983	1777	-	chr10	17270258	17279592	2136	+	*		0.7137	1	0.56	0.85	0.59	71.31	79.91	78.3
cis_mRNA_overlap	0	AntiCompleteIn-mRNAIntron	n382641	NM_002833_dup1	chr15	75797279	75797803	525	+	chr15	75759462	75871625	3938	-	*		-0.8466	-1	0.79	1.84	0.77	25.97	24.66	27.55
cis_mRNA_up10k	8326	NA	n406891	NM_003116_dup1	chr20	34146507	34195484	6579	-	chr20	34203809	34208965	1452	+	*		0.9984	1	0.26	0.52	0.38	1.08	1.59	1.34
cis_mRNA_dw20k	2376	NA	n380484	NM_003959_dup1	chr12	123349882	123351568	482	+	chr12	123320039	123347507	4473	+	*		-0.6788	-1	0.23	0.5	0.28	10.14	7.34	7.49
cis_mRNA_dw20k	9786	NA	n364661	NM_002908_dup1	chr2	61159963	61163467	2828	+	chr2	61108752	61150178	2592	+	*		0.8719	1	0.15	0.17	0.35	0.22	0.51	0.73
cis_mRNA_up10k	3523	NA	LTCONS_00035978	NM_003430_dup1	chr19	23581791	23598903	3263	-	chr19	23540498	23578269	5396	-	*		-0.938	-0.866	0	0.08	0.08	30.6	27.78	26.25
cis_mRNA_dw20k	18786	NA	LTCONS_00037039	NM_021089_dup1	chr19	58826039	58838344	520	-	chr19	58790318	58807254	2210	+	*		0.9492	1	19.91	13.06	22.16	12.39	12.09	12.73
cis_mRNA_dw20k	10509	NA	n376433	NM_001009_dup1	chr19	58916679	58919843	2615	-	chr19	58898636	58906171	741	+	*		-0.9702	-1	0.73	0.79	1.09	379.13	344.28	290.48
cis_mRNA_up10k	468	NA	n341701	NM_006914_dup1	chr9	77109925	77111785	1861	-	chr9	77112252	77302117	3604	+	*		0.8963	0.866	0.02	0	0.02	0.17	0.08	0.13
cis_mRNA_overlap	0	Lnc-CompleteIn-mRNAExon	n407172	NM_002853_dup1	chr5	34905366	34915731	4524	-	chr5	34905365	34915780	4683	-	*		-0.9334	-1	3.55	4.34	2.38	3.6	3.54	4.72
cis_mRNA_dw20k	4017	NA	n410560	NM_001001_dup1	chr14	50065415	50081390	1965	+	chr14	50085406	50087349	523	-	*		-0.9805	-1	0.06	0	0.17	90.94	93.59	88.55
cis_mRNA_up10k	1975	NA	LTCONS_00027854	NM_005853_dup1	chr16	54944636	54963137	13772	-	chr16	54965111	54968397	2064	+	*		-0.8161	-0.866	1.38	0.86	0.86	2.1	3.22	2.57
cis_mRNA_overlap	0	CompleteIn-LncExon	n410711	NM_138501_dup1	chr19	14640379	14676792	1198	+	chr19	14640379	14676792	1166	+	*		0.9942	1	2.2	2.69	1.22	16.78	19.29	13.43
cis_mRNA_overlap	0	Lnc-AntiOverlap-mRNA	LTCONS_00072071	NM_007371_dup1	chr9	136890561	136896816	5342	+	chr9	136895427	136933141	5654	-		-2808.1	-0.9005	-1	0.4	0.29	0.42	5.37	5.88	4.72
cis_mRNA_up10k	9009	NA	n371595	NM_004654_dup1	chrY	14774298	14804152	5241	+	chrY	14813160	14972957	10048	+	*		0.9991	1	18.12	0.02	16.5	0.92	0	0.88
cis_mRNA_up10k	292	NA	LTCONS_00037727	NM_003618_dup1	chr2	39664510	39718360	17022	+	chr2	39476422	39664219	4113	-	*		0.9998	1	1.56	1.43	1.41	11.92	10.88	10.75
cis_mRNA_overlap	0	Lnc-CompleteIn-mRNAIntron	n323896	NM_004615_dup1	chrX	38514528	38543855	1180	+	chrX	38420731	38548172	1806	+	*		-0.9755	-1	0.09	0.08	0.11	47.59	56.88	39.27

cis_mRNA_overlap	0	Lnc-AntiOverlap-mRNA	n340615	NM_005732_dup1	chr5	131973740	131991846	407	-	chr5	131892616	131980313	6597	+	*		0.9967	1	0.09	0	0.08	7.47	6.14	7.44
cis_mRNA_overlap	0	mRNA	n410154	NM_003374_dup1	chr5	133307566	133340657	2071	-	chr5	133307566	133340824	1993	-	*		-0.8809	-1	24.96	27.68	37.3	103.82	93.21	87.7
tran	NA	NA	LTCONS_00007598	NM_021109_dup1	chr1	42965540	42966010	471	.	chrX	12993226	12995346	629	+		-896	-0.8553	-1	0.72	1.24	0.71	1685.05	1379.36	2033.33
tran	NA	NA	LTCONS_00007598	NM_004202_dup1	chr1	42965540	42966010	471	.	chrY	15815447	15817904	1669	+		-662.93	-0.9768	-1	0.72	1.24	0.71	4.27	0	5.62
cis_mRNA_overlap	0	mRNA	LTCONS_00025849	NM_005741_dup1	chr16	3331264	3341617	5167	+	chr16	3333487	3341460	3286	+		-2149.4	0.9293	1	0.22	0.21	0.29	9.46	9.37	9.56
cis_mRNA_up10k	2361	NA	n410203	NM_004687_dup1	chr17	56597611	56606778	2379	-	chr17	56566893	56595251	5829	-	*		0.9309	1	0.6	1.58	1.36	50	52.1	50.93
cis_mRNA_overlap	0	Lnc-CompleteIn-mRNAExon	n339281	NM_014003_dup1	chr16	72127834	72146802	4226	+	chr16	72127615	72146811	4454	+	*		-0.9839	-1	5.75	5.1	10.16	1.69	1.94	1.11
cis_mRNA_up10k	262	NA	LTCONS_00060873	NM_002912_dup1	chr6	111804675	111872652	8047	+	chr6	111620234	111804414	10701	-	*		-0.8683	-1	0.04	0	0.02	9.89	11.81	9.9
cis_mRNA_overlap	0	Lnc-CompleteIn-mRNAExon	n343053	NM_001035235_dup1	chr5	139929653	139937678	1948	-	chr5	139929653	139937678	1948	-	*		1	1	2.77	3.52	3.78	2.77	3.52	3.78
cis_mRNA_overlap	0	CompleteIn-LncExon	n407053	NM_003876_dup1	chr17	21101263	21117908	1688	-	chr17	21101263	21117908	1396	-	*		-0.9998	-1	2.24	2.13	2	8.21	8.95	9.77
cis_mRNA_overlap	0	CompleteIn-LncIntron	LTCONS_00019713	NM_005986_dup1	chr13	112517175	112742572	2735	+	chr13	112721913	112726020	4108	+	*		0.7696	0.866	1.23	1.8	0.92	0.01	0.01	0
cis_mRNA_overlap	0	mRNA	n341140	NM_005422_dup1	chr11	121061481	121062195	715	+	chr11	120973375	121061515	6468	+	*		0.9944	1	0.81	0.64	1.17	0.85	0.81	0.99
cis_mRNA_overlap	0	mRNA	n379254	NM_014803_dup1	chr10	97889492	97915776	729	+	chr10	97889472	97923517	8295	+	*		0.9452	1	0.25	0.23	0.18	4.7	4.33	4.06
cis_mRNA_up10k	3523	NA	LTCONS_00035975	NM_003430_dup1	chr19	23581791	23598903	2997	-	chr19	23540498	23578269	5396	-	*		0.9885	1	1	0.83	0.67	30.6	27.78	26.25
cis_mRNA_up10k	128	NA	n366214	NM_004157_dup1	chr3	48885397	48885882	407	+	chr3	48788093	48885270	2381	-	*		0.9663	1	0	0.31	0.24	28.23	29.23	28.77
cis_mRNA_up10k	146	NA	n337731	NM_003758_dup1	chr15	44826703	44829121	1851	-	chr15	44829266	44855001	2558	+	*		-0.917	-1	7.09	8.19	8.46	20.44	20.27	20.05
cis_mRNA_overlap	0	Lnc-CompleteIn-mRNAIntron	n340982	NM_000352_dup1	chr11	17439193	17447011	924	-	chr11	17414432	17498449	4978	-	*		-0.9771	-1	0.08	0	0.07	0.75	1.63	1.04
cis_mRNA_overlap	0	mRNA	n408131	NM_001163391_dup1	chr6	28346598	28367544	4343	-	chr6	28356727	28367544	3519	-	*		-0.953	-1	4.95	4.54	3.51	3.01	3.41	3.73
cis_mRNA_overlap	0	Lnc-AntiOverlap-mRNA	n324372	NM_006290_dup1	chr6	138186503	138188724	1025	-	chr6	138188581	138204449	4432	+	*		-0.8591	-1	0.24	0.22	0.15	0.3	0.37	0.4
cis_mRNA_overlap	0	Lnc-AntiOverlap-mRNA	n342140	NM_006290_dup1	chr6	138144812	138189370	1959	-	chr6	138188581	138204449	4432	+	*		-0.7313	-0.866	0.11	0.11	0.05	0.3	0.37	0.4
cis_mRNA_overlap	0	mRNA	LTCONS_00076700	NM_004202_dup1	chrY	15815403	16031352	7653	+	chrY	15815447	15817904	1669	+		-961.97	0.9803	1	4.84	0.25	5.02	4.27	0	5.62
cis_mRNA_up10k	1684	NA	n340943	NM_005706_dup1	chr11	2419371	2421840	2470	-	chr11	2423523	2425106	1424	+	*		-0.9837	-1	0.36	0.2	0.43	12.6	15.53	10.06
cis_mRNA_up10k	13	NA	LTCONS_00027204	NM_012225_dup1	chr16	1826236	1832921	6298	-	chr16	1832933	1839192	1382	+	*		0.9188	1	1.12	1.39	1.63	12.4	13.15	17.02
tran	NA	NA	LTCONS_00069238	NM_000984_dup1	chr8	155778	182318	8285	-	chr17	27047000	27051374	970	+		-581.47	-0.9934	-1	0.28	0.19	0.49	213.54	227.03	156.83
cis_mRNA_dw20k	3398	NA	n375029	NM_003223_dup1	chr16	4295826	4303790	2093	-	chr16	4307187	4323001	2140	-	*		-0.9489	-1	0	0.11	0.01	2.53	2.1	2.36
cis_mRNA_dw20k	2720	NA	n406850	NM_005727_dup1	chr1	46654353	46685977	2936	-	chr1	46640749	46651634	1629	+	*		-0.9375	-0.866	0.23	0.31	0.31	0.45	0.23	0.11
cis_mRNA_up10k	6563	NA	n409287	NM_001069_dup1	chr6	3164345	3180755	1895	-	chr6	3153900	3157783	1621	-	*		0.9995	1	0.32	0.2	0.57	725.91	676.38	816.6
cis_mRNA_up10k	7286	NA	n405879	NM_006049_dup1	chr15	66797431	66841822	3277	+	chr15	66782666	66790146	785	-	*		-0.7747	-1	8.96	7.59	2.77	3.42	4.62	4.85
cis_mRNA_overlap	0	Lnc-AntiOverlap-mRNA	n339534	NM_005049_dup1	chr21	45550940	45553342	2403	-	chr21	45527208	45551063	3260	+	*		-0.9878	-0.866	0.26	0.26	0.25	5.75	5.85	6.35
cis_mRNA_dw20k	8465	NA	n345053	NM_005077_dup1	chr9	84189583	84190134	552	-	chr9	84198598	84303596	3264	-	*		-0.7526	-0.866	0.54	0.18	0.18	0.98	1.46	2.44
cis_mRNA_overlap	0	Lnc-AntiOverlap-mRNA	n339563	NM_003571_dup1	chr3	133052421	133131725	2703	-	chr3	133118790	133194056	1626	+	*		1	1	0.04	0.07	0.05	0	0.06	0.02
cis_mRNA_dw20k	469	NA	n407188	NM_001069_dup1	chr6	3118926	3153432	1616	+	chr6	3153900	3157783	1621	-	*		0.9306	1	1.94	1.04	2.36	725.91	676.38	816.6
cis_mRNA_dw20k	3529	NA	n340817	NM_001050_dup1	chr17	71171622	71172772	1151	+	chr17	71161160	71168094	2996	+	*		0.998	1	2.59	3.77	4.96	7.88	10.67	12.93
cis_mRNA_dw20k	2716	NA	LTCONS_00007617	NM_004892_dup1	chr1	145119637	145121819	2183	.	chr1	145096407	145116922	1821	+	*		-0.9891	-1	1.93	2.23	2.04	37.08	34.66	36.52
cis_mRNA_dw20k	10146	NA	n410557	NM_004596_dup1	chr19	41281442	41302849	1394	+	chr19	41256759	41271297	1629	+	*		0.7198	1	0	0.61	0.08	7.83	10.25	9.84
cis_mRNA_dw20k	14671	NA	n379545	NM_003161_dup1	chr17	58042457	58074333	1603	+	chr17	57970443	58027787	5319	+	*		0.9999	0.866	0	0.02	0.02	12.23	12.98	12.99
cis_mRNA_up10k	352	NA	n382027	NM_003295_dup1	chr13	45915648	45965616	2029	-	chr13	45911304	45915297	829	-	*		0.9963	1	1.2	0.97	0.76	358.65	311.99	280.6
cis_mRNA_overlap	0	Lnc-AntiOverlap-mRNA	LTCONS_00045634	NM_003906_dup1	chr21	47649145	47663412	314	+	chr21	47655048	47705236	6113	-		-516.06	0.9314	1	0.58	0.87	0.12	5.65	6.7	5.23
cis_mRNA_overlap	0	CompleteIn-LncExon	LTCONS_00072191	NM_003731_dup1	chr9	140083054	140084826	1232	+	chr9	140083054	140084822	941	+		-771.63	-0.9497	-1	14.54	10.45	12.85	8.49	11.19	8.8
cis_mRNA_dw20k	7553	NA	n371571	NM_006306_dup1	chrX	53388441	53393518	395	-	chrX	53401070	53449618	9725	-	*		0.8547	1	0.78	1.6	1.39	13.15	14.81	13.58
cis_mRNA_dw20k	9175	NA	n410676	NM_006942_dup1	chr17	7465309	7482324	4304	+	chr17	7491498	7493488	1394	-	*		0.9826	1	0.49	0.23	0.52	0.07	0.02	0.09
cis_mRNA_dw20k	12225	NA	n410676	NM_004860_dup1	chr17	7465309	7482324	4304	+	chr17	7494548	7518215	2987	-	*		0.9046	1	0.49	0.23	0.52	44.67	43.63	45.75
cis_mRNA_up10k	1947	NA	n378806	NM_005047_dup1	chr9	123607152	123616651	3519	+	chr9	123578331	123605206	3387	-	*		0.9321	1	2.7	0.58	0	12.76	10.61	8.03
cis_mRNA_overlap	0	CompleteIn-LncExon	n410200	NM_003809_dup1	chr17	7452375	7461207	1625	+	chr17	7452375	7461207	1386	+	*		-0.9858	-1	0.08	0.06	0	0.42	0.77	1.29
tran	NA	NA	LTCONS_00063847	NM_003897_dup1	chr6_cox_hap2	2223297	2224174	766	-	chr6	30710976	30712327	1240	-		-506.6	-0.9857	-1	4.35	4.19	4.48	2.01	2.18	1.76
cis_mRNA_up10k	2154	NA	n375239	NM_006043_dup1	chr16	22823087	22823707	273	-	chr16	22825860	22928087	1968	+	*		-0.8717	-1	0.15	0.27	0.14	1.84	1.39	2.39
tran	NA	NA	LTCONS_00042852	NM_000984_dup1	chr2	243160378	243160804	427	.	chr17	27047000	27051374	970	+		-758.8	0.6601	1	0.09	0.65	0.08	213.54	227.03	156.83
cis_mRNA_dw20k	4175	NA	n338287	NM_145185_dup1	chr19	7983537	7984042	506	-	chr19	7968765	7979363	3372	+	*		0.831	1	0.29	0.34	0.79	7.94	9.17	9.87
cis_mRNA_dw20k	1433	NA	n384848	NM_021968_dup1	chr6	27793690	27794715	588	+	chr6	27791903	27792258	356	+	*		-0.9993	-1	1	2.17	1.64	192.8	147.36	166.42
cis_mRNA_dw20k	17246	NA	n384848	NM_003509_dup1	chr6	27793690	27794715	588	+	chr6	27775977	27776445	469	+	*		-0.8965	-1	1	2.17	1.64	12.97	8.81	8.86
cis_mRNA_dw20k	2997	NA	LTCONS_00070621	NM_003350_dup1	chr8	48977450	48980118	2669	.	chr8	48920995	48974454	1524	+	*		0.9551	1	1.21	1.51	1.25	58.18	61.64	59.61
cis_mRNA_up10k	1959	NA	n340361	NM_003966_dup1	chr5	9548191	9550410	2220	+	chr5	9035138	9546233	11808	-	*		0.9841	1	0.03	0.01	0	0.72	0.47	0.23
cis_mRNA_overlap	0	Lnc-AntiOverlap-mRNA	n378509	NM_004855_dup1	chr15	55609382	55611243	866	-	chr15	55611133	55647846	2197	+	*		0.6934	0.866	1.52	2.51	2.84	1.55	1.55	2.38
cis_mRNA_dw20k	11896	NA	n386173	NM_014695_dup1	chr17	16690206	16707819	3901	+	chr17	16593639	16678311	5828	+	*		0.7386	1	0.52	0.84	0.87	0.26	0.31	0.47
cis_mRNA_dw20k	9380	NA	n340956	NM_003737_dup1	chr11	6627526	6633179	5392	-	chr11	6642558	6677074	10750	-	*		0.8985	1	1.03	1.13	1.56	26.21	28.18	29.52
cis_mRNA_up10k	170	NA	n379351	NM_032727_dup1	chr10	104958490	105036751	1552	-	chr10	105036920	105050108	3231	+	*		0.85	1	2.14	1.97	1.63	312.16	291.86	287.72
cis_mRNA_overlap	0	mRNA	n338375	NM_003285_dup1	chr1	175375845	175712906	709	-	chr1	175291935	175712572	5190	-	*		-0.9958	-1	0.41	0.33	0.37	1.79	2.29	2.08
cis_mRNA_dw20k	3369	NA	n379823	NM_003641_dup1	chr11	318640	319615	366	+	chr11	313991	315272	717	+	*		0.8133	1	0.21	0.62	0.31	0	0.05	0.04
cis_mRNA_up10k	4474	NA	n346049	NM_001049_dup1	chr14	38662413	38672731	4372	+	chr14	38677204	38682268	4291	+	*		-0.9286	-1	0.02	0.04	0.01	0.04	0.03	0.06

cis_mRNA_up10k	1571	NA	n409082	NM_006983_dup1	chr1	1550795	1565990	3121	+	chr1	1567560	1570030	1240	+	*	-0.9844	-1	3.64	1.71	4.16	0.39	0.72	0.38
cis_mRNA_up10k	87	NA	n378425	NM_000321_dup1	chr13	48870649	48877797	1143	-	chr13	48877883	49056026	4772	+	*	-0.7823	-1	0.11	0.08	0.01	11.6	12.34	12.41
cis_mRNA_overlap	0	mRNA	n377720	NM_014803_dup1	chr10	97915100	97923517	8269	+	chr10	97889472	97923517	8295	+	*	0.8793	1	10.26	9.82	6.8	4.7	4.33	4.06
cis_mRNA_dw20k	1052	NA	n380909	NM_007371_dup1	chr9	136890608	136894376	1068	+	chr9	136895427	136933141	5654	-	*	0.9863	1	0.76	1.04	0.56	5.37	5.88	4.72
cis_mRNA_up10k	4944	NA	n5726	NM_006049_dup1	chr15	66795089	66795395	307	-	chr15	66782666	66790146	785	-	*	-0.6581	-1	1.57	1.55	1.17	3.42	4.62	4.85
cis_mRNA_overlap	0	Lnc-CompleteIn-mRNAIntron	n339113	NM_003812_dup1	chr2	207470896	207473546	2651	+	chr2	207308368	207482685	3056	+	*	0.9949	1	0.71	0.73	1.25	3.3	3.71	6.14
cis_mRNA_up10k	433	NA	n367312	NM_005663_dup1	chr4	2011391	2043485	1945	-	chr4	1984443	2010959	2463	-	*	0.9985	1	0.4	0.21	0.22	9.09	8.46	8.53
cis_mRNA_dw20k	7840	NA	n373180	NM_003440_dup1	chr12	133692099	133698651	4867	+	chr12	133657037	133684260	3067	+	*	0.9864	1	0.03	0	0.02	7.16	6.24	6.72
cis_mRNA_dw20k	11697	NA	n407869	NM_014751_dup1	chr8	125500735	125551329	1148	-	chr8	125563025	125740730	5010	-	*	0.9213	1	0.78	1.81	0.46	0.26	0.44	0
cis_mRNA_up10k	108	NA	n363353	NM_014813_dup1	chr1	113554309	113615724	2456	-	chr1	113615831	113667342	4015	+	*	1	1	1.04	1.5	1.19	9.25	10.13	9.54
cis_mRNA_overlap	0	Lnc-CompleteIn-mRNAIntron	n326140	NM_002924_dup1	chr1	241325916	241327512	530	-	chr1	240938817	241520478	2440	-	*	-0.9706	-1	0.07	0	0.19	3.42	4.54	2.61
cis_mRNA_overlap	0	Lnc-AntiOverlap-mRNA	n378100	NM_003906_dup1	chr21	47649161	47662913	2280	+	chr21	47655048	47705236	6113	-	*	0.9963	1	0.63	0.7	0.59	5.65	6.7	5.23
cis_mRNA_overlap	0	Lnc-CompleteIn-mRNAExon	n342469	NM_003321_dup1	chr16	28854108	28857678	1621	-	chr16	28853732	28857729	2071	-	*	-0.9995	-1	29.86	30.87	28.89	33	30.8	35.35
cis_mRNA_dw20k	11848	NA	n376434	NM_001009_dup1	chr19	58918018	58919467	1357	-	chr19	58898636	58906171	741	+	*	-0.9617	-1	0.19	0.21	0.34	379.13	344.28	290.48
cis_mRNA_up10k	151	NA	n364719	NM_005415_dup1	chr2	113402607	113403284	579	+	chr2	113403434	113421402	3379	+	*	0.7204	1	0.06	0.23	0	25.5	25.62	21.66
tran	142144	NA	LTCONS_00036842	NM_006969_dup1	chr19	53467065	53493457	7496	-	chr19	53300661	53324922	4594	-	-1952.52	0.9671	1	0.39	0.46	0	1.06	1.6	0.22
tran	NA	NA	LTCONS_00067917	NM_004230_dup1	chr7	148787954	148789503	1550	.	chr19	10332109	10341948	3584	-	-1101.15	-0.9245	-1	1.24	1.72	1.37	5.18	4.27	4.62
cis_mRNA_overlap	0	Lnc-CompleteIn-mRNAExon	n342487	NM_014760_dup1	chr3	10290219	10322900	4688	+	chr3	10290177	10322906	4907	+	*	-0.9897	-1	4.02	4.52	3.65	2.8	2.54	2.91
cis_mRNA_up10k	90	NA	n339762	NM_005131_dup1	chr18	268148	270278	2127	+	chr18	214520	268059	2120	-	*	0.9934	1	0.24	0.27	0.29	7.36	8.35	9.34
tran	NA	NA	LTCONS_00032002	NM_022551_dup1	chr17	14608571	14608893	323	.	chr3	101295064	101296275	549	-	-638	-0.9799	-1	1.73	3.36	2.76	0.46	0.35	0.37
cis_mRNA_up10k	204	NA	n408128	NM_014828_dup1	chr14	21927179	21945132	2884	-	chr14	21945335	21967319	4524	+	*	0.8291	1	0.86	1.39	1.29	16.07	17.71	16.53
tran	58162542	NA	LTCONS_00032034	NM_003585_dup1	chr17	58193961	58200248	6288	.	chr17	6011	31420	1073	-	-1316.85	-0.8105	-1	0.28	0.44	0.25	21.9	16.04	37.02
cis_mRNA_overlap	0	Lnc-CompleteIn-mRNAExon	n337723	NM_031407_dup1	chrX	53659450	53713673	1036	-	chrX	53559063	53713673	14734	-	*	-0.9151	-1	6.32	2.02	4.26	11.25	16.73	11.76
cis_mRNA_up10k	6481	NA	n340646	NM_005859_dup1	chr5	139482507	139487228	2769	-	chr5	139493708	139499001	5294	+	*	-0.7963	-1	0.2	0.08	0.02	2.05	2.06	2.19
cis_mRNA_overlap	0	mRNA	n410671	NM_006250_dup1	chr12	10998448	11324224	1582	-	chr12	11033560	11036874	673	-	*	0.997	1	5.24	4.97	4.93	0.5	0.09	0.07
cis_mRNA_dw20k	2981	NA	LTCONS_00045860	NM_016940_dup1	chr21	30366867	30375100	6339	-	chr21	30378080	30391685	1705	-	*	0.9599	1	1.74	1.81	1.88	4.45	5.26	7.73
tran	NA	NA	LTCONS_00045860	NM_000978_dup1	chr21	30366867	30375100	6339	-	chr17	37006321	37010053	577	-	-356.18	-0.9319	-1	1.74	1.81	1.88	492.66	488.18	465.18
cis_mRNA_overlap	0	AntiCompleteIn-mRNAIntron	n338349	NM_004831_dup1	chr19	16698072	16700220	2149	+	chr19	16685718	16739015	3184	-	*	-0.9824	-1	0.14	0.13	0.07	1.66	1.75	1.94
cis_mRNA_overlap	0	Lnc-AntiOverlap-mRNA	n340183	NM_003728_dup1	chr4	96470280	96473371	3091	+	chr4	96083655	96470361	9879	-	*	0.9356	1	5.16	6.16	3.93	8.45	11.98	7.39
cis_mRNA_up10k	384	NA	n380222	NM_003821_dup1	chr8	90768463	90769592	696	-	chr8	90769975	90803292	2585	+	*	-0.998	-1	0.54	1.15	1.22	1	0.85	0.82
tran	NA	NA	LTCONS_00032069	NM_005730_dup1	chr17_gl000205_random	39005	40208	828	+	chr12	58213710	58240747	5020	-	-993.35	0.6302	1	0.29	1.38	0.27	3.47	3.6	2.75
cis_mRNA_dw20k	15298	NA	LTCONS_00072197	NM_003731_dup1	chr9	140100119	140113844	5847	+	chr9	140083054	140084822	941	+	*	0.8994	1	1.73	2.24	2	8.49	11.19	8.8
cis_mRNA_dw20k	19077	NA	n408350	NM_006743_dup1	chrX	48455880	48463582	2091	+	chrX	48432836	48436804	1588	+	*	0.9073	1	3.72	0.96	3.12	137.03	114.88	123.67
cis_mRNA_up10k	796	NA	LTCONS_00021149	NM_004713_dup1	chr14	50320334	50379335	298	+	chr14	50250532	50319539	4121	-	*	0.7441	1	1.51	3.97	3.51	19.69	20.36	19.79
cis_mRNA_overlap	0	Lnc-AntiOverlap-mRNA	n342800	NM_004289_dup1	chr7	26223684	26224429	746	-	chr7	26191847	26226757	3711	+	*	-0.6682	-1	4.68	7.07	4.99	2.31	1.49	1.57
cis_mRNA_dw20k	13002	NA	n333962	NM_003913_dup1	chr6	4078218	4078499	282	-	chr6	4021569	4065217	7482	+	*	-0.944	-1	0	0.09	0.27	3.34	2.43	1.89
cis_mRNA_overlap	0	Lnc-AntiOverlap-mRNA	n410952	NM_003168_dup1	chr17	56414563	56431088	2451	+	chr17	56422539	56429563	1489	-	*	-0.6855	-1	0.04	0	0.23	30.25	32.93	30.03
cis_mRNA_overlap	0	Lnc-CompleteIn-mRNAExon	n341240	NM_033224_dup1	chr7	44915900	44918584	2685	-	chr7	44915505	44924960	9074	-	*	0.9834	1	0.29	0.54	0.67	1.4	1.69	1.75
cis_mRNA_overlap	0	Lnc-CompleteIn-mRNAExon	n335545	NM_003211_dup1	chr12	104359654	104373763	481	+	chr12	104359593	104382656	3244	+	*	-0.9738	-1	0.34	0.69	0	35.13	31.02	36.81
cis_mRNA_dw20k	10742	NA	LTCONS_00027056	NM_004913_dup1	chr16	89753016	89762800	4465	+	chr16	89773541	89787394	2749	-	*	-0.9915	-1	0.17	0.12	0.09	5.97	7.76	9.47
cis_mRNA_dw20k	5952	NA	n381378	NM_003431_dup1	chr1	247285278	247313252	2031	-	chr1	247319203	247335318	1674	-	*	0.8084	1	1.88	1.84	0.39	134.63	89.42	60.84
cis_mRNA_overlap	0	mRNA-AntiCompleteIn-LncIntron	n381512	NM_003019_dup1	chr10	81664654	81710783	1961	+	chr10	81697496	81708861	1283	-	*	-0.7674	-1	0.12	0.1	0.02	0.35	0.64	0.7
cis_mRNA_up10k	154	NA	n385266	NM_003941_dup1	chr7	123389269	123391032	1764	+	chr7	123321989	123389116	4430	-	*	0.6082	1	3.29	3.32	4.52	6.44	7.48	7.62
cis_mRNA_overlap	0	AntiCompleteIn-mRNAIntron	n340719	NM_003110_dup1	chr17	45976151	45995938	1715	-	chr17	45973516	46006323	3112	+	*	0.7744	0.866	0	0.02	0.02	0.69	1.08	0.83
cis_mRNA_dw20k	8663	NA	n372823	NM_004200_dup1	chr11	61267237	61274123	961	+	chr11	61282785	61348298	4532	-	*	0.7967	1	0.15	0	0.03	4.6	3.91	4.45
cis_mRNA_overlap	0	Lnc-AntiOverlap-mRNA	n405217	NM_014742_dup1	chr20	30684322	30703341	3456	-	chr20	30697309	30755061	3978	+	*	-0.9867	-1	0.39	0.27	0.34	10.4	12.37	11.5
cis_mRNA_up10k	1143	NA	LTCONS_00040533	NM_003353_dup1	chr2	27532272	27545982	1767	-	chr2	27530265	27531130	615	-	*	0.9912	1	0.51	1.04	0.95	0.53	1.19	1.17
cis_mRNA_dw20k	15595	NA	n385202	NM_003596_dup1	chr7	65841032	65865395	4423	-	chr7	65670259	65825438	2051	+	*	0.9866	1	0.23	0.24	0.26	7.23	8.71	10.33
tran	NA	NA	LTCONS_00012573	NM_005719_dup1	chr11	93921564	93922676	1113	+	chr12	110872706	110888158	884	-	-1247.91	0.993	1	0.54	0.62	0.92	142.01	142.98	152.6
cis_mRNA_overlap	0	Lnc-CompleteIn-mRNAIntron	n338804	NM_005708_dup1	chr13	94287787	94288466	680	+	chr13	93879078	95060274	7103	+	*	0.9924	1	0.43	1.16	0.24	3.51	12.64	2.7
cis_mRNA_overlap	0	Lnc-CompleteIn-mRNAIntron	n341262	NM_012301_dup1	chr7	78802389	78804340	1952	-	chr7	77646374	79082890	6880	-	*	0.9996	1	0	0.06	0.09	1.34	2.15	2.51
cis_mRNA_up10k	8704	NA	n342823	NM_003143_dup1	chr7	141408153	141429473	2084	+	chr7	141438176	141450257	628	+	*	-0.9872	-1	0.02	0.05	0	91.69	85.66	93.76
cis_mRNA_up10k	3260	NA	n375220	NM_001098814_dup1	chr16	4295340	4303751	2428	-	chr16	4239375	4292081	4214	-	*	0.9934	1	0.03	0.05	0	0.01	0.02	0
cis_mRNA_up10k	3981	NA	n370079	NM_003439_dup1	chr7	99597001	99609239	356	+	chr7	99613219	99635403	5494	+	*	-0.9996	-1	0.36	0	0.33	18.68	19.8	18.74

cis_mRNA_overlap	0	Lnc-CompleteIn-mRNAExon	n340532	NM_006909_dup1	chr5	80256558	80521586	3711	+	chr5	80256558	80521953	4078	+	*		-0.9959	-1	0.39	0	0.41	0.64	2.53	0.32
cis_mRNA_dw20k	8667	NA	n345330	NM_004765_dup1	chr16	30816811	30890450	1837	-	chr16	30899116	30905399	858	-	*		0.7376	1	1.93	1.9	2.74	30.23	22.01	33.42
cis_mRNA_up10k	71	NA	n340921	NM_002949_dup1	chr17	79668354	79670330	1977	-	chr17	79670400	79674556	1015	+	*		0.983	1	4.76	4.66	4.37	7.92	7	5.74
cis_mRNA_overlap	0	Lnc-AntiOverlap-mRNA	n340921	NM_004712_dup1	chr17	79668354	79670330	1977	-	chr17	79650962	79669151	2972	+	*		-0.9967	-1	4.76	4.66	4.37	25.88	26.73	30.64
cis_mRNA_up10k	2091	NA	n406432	NM_005654_dup1	chr5	92746502	92916953	1184	-	chr5	92919043	92929804	3210	+	*		0.8833	1	1.75	2.61	0.35	4.15	9.33	2.99
cis_mRNA_overlap	0	mRNA	n409672	NM_006513_dup1	chr1	109756515	109760113	3181	+	chr1	109756515	109780804	1939	+	*		-0.6885	-1	6.54	6.57	10.62	201.33	179.97	173.31
cis_mRNA_up10k	8297	NA	n372214	NM_016195_dup1	chr10	91446851	91453071	806	-	chr10	91461367	91534700	6310	+	*		-0.7308	-1	0.28	0	0.22	1.09	1.79	1.72
cis_mRNA_up10k	5302	NA	n405468	NM_005399_dup1	chr1	146649430	146651528	2099	+	chr1	146626685	146644129	5423	-	*		0.9945	1	2.11	2.48	2.51	20.18	22.2	22.65
cis_mRNA_dw20k	6105	NA	n406594	NM_003921_dup1	chr1	85715637	85725355	3390	-	chr1	85731459	85742587	3105	-	*		1	1	10.07	11.41	9.84	4.94	5.17	4.9
cis_mRNA_up10k	1332	NA	n365374	NM_003108_dup1	chr2	5816755	5831468	840	-	chr2	5832799	5841517	8719	+	*		0.903	1	0.65	0.31	0.32	102.47	56.03	77.09
cis_mRNA_dw20k	1691	NA	n386292	NM_006052_dup1	chr21	38592889	38594036	1148	+	chr21	38595726	38639833	3251	-	*		-0.8545	-1	0	0.1	0.25	13.23	12.08	11.95
cis_mRNA_overlap	0	Lnc-AntiOverlap-mRNA	n410783	NM_004218_dup1	chr19	8439260	8455575	1022	-	chr19	8455205	8469318	1629	+	*		-0.9783	-1	2.24	1.85	1.05	10.23	11.19	12.11
cis_mRNA_up10k	554	NA	n375834	NM_003488_dup1	chr17	55149782	55162000	238	-	chr17	55162553	55198710	3968	+	*		-0.9992	-1	0	0.08	0.45	23.7	22.65	16.1
cis_mRNA_dw20k	11224	NA	n372815	NM_000352_dup1	chr11	17402306	17403209	788	+	chr11	17414432	17498449	4978	-	*		0.996	1	0.31	1.73	0.89	0.75	1.63	1.04
cis_mRNA_dw20k	15293	NA	n382141	NM_003917_dup1	chr14	23982558	24013485	2524	+	chr14	24028777	24037279	2876	-	*		0.9543	1	0.03	0.09	0.07	3.48	6.11	6.02
cis_mRNA_overlap	0	Lnc-AntiOverlap-mRNA	LTCONS_00031121	NM_005854_dup1	chr17	40905947	40913275	3136	-	chr17	40913212	40915059	785	+	*		0.9882	1	0.93	0	1.26	1.75	1.19	1.82
cis_mRNA_overlap	0	CompleteIn-LncExon	LTCONS_00073483	NM_003863_dup1	chr9	130697374	130700785	2346	-	chr9	130697374	130700763	1546	-		-1096.06	0.9397	1	2.68	3.53	4.07	2.48	2.81	3.56
cis_mRNA_up10k	3312	NA	n384227	NM_003298_dup1	chr3	14984294	14985925	1632	-	chr3	14989236	15090780	8330	+	*		-0.6876	-1	14.48	13.63	13.78	2.85	5.08	3.02
cis_mRNA_up10k	339	NA	n383620	NM_003618_dup1	chr2	39664557	39828484	2517	+	chr2	39476422	39664219	4113	-	*		0.9668	1	1.75	1.57	1.47	11.92	10.88	10.75
cis_mRNA_dw20k	457	NA	n339390	NM_007114_dup1	chr3	69066555	69068522	1968	-	chr3	69068978	69101484	6879	-	*		0.8504	1	0.3	0.37	0.16	10.16	11.38	9.91
cis_mRNA_dw20k	17185	NA	n409644	NM_001042603_dup1	chr12	368155	372039	1154	-	chr12	389223	498620	10835	-	*		0.9306	1	0	0.03	0.14	8.12	8.74	9.25
cis_mRNA_dw20k	10500	NA	n363432	NM_005482_dup1	chr1	77533605	77544167	497	+	chr1	77554666	77685132	4626	-	*		-0.7881	-1	1.35	1.16	1.81	6.59	7.44	6.49
cis_mRNA_overlap	0	AntiCompleteIn-mRNAExon	n338387	NM_006029_dup1	chr14	74178486	74180998	2513	+	chr14	74178486	74181128	2643	-	*		0.7424	0.866	0.03	0.04	0.04	42.19	48.54	44.19
cis_mRNA_dw20k	12939	NA	n383066	NM_014695_dup1	chr17	16691249	16707819	3763	+	chr17	16593639	16678311	5828	+	*		-0.9851	-1	0.66	0.51	0.28	0.26	0.31	0.47
cis_mRNA_overlap	0	Lnc-AntiOverlap-mRNA	n380942	NM_003877_dup1	chr12	93959360	93965174	618	-	chr12	93963598	93969978	2210	+	*		0.9004	1	0.79	0.75	1.12	1.36	1.24	1.47
cis_mRNA_overlap	0	Lnc-AntiOverlap-mRNA	n346312	NM_002952_dup1	chr17	19349405	19350190	732	+	chr17	19349226	19350188	961	-	*		-0.8733	-1	0.25	0.58	0.66	123.55	109.01	82.13
cis_mRNA_up10k	791	NA	n375478	NM_007146_dup1	chr17	56066405	56072211	4722	-	chr17	56048910	56065615	4633	-	*		0.7535	1	0.01	0.04	0	17.06	17.33	14.52
tran	4963329	NA	LTCONS_00023161	NM_004667_dup1	chr15	23388420	23392858	747	+	chr15	28356186	28567295	15334	-		-540.17	0.9855	1	1.97	1.63	2.25	10.35	10.25	10.5
cis_mRNA_dw20k	3159	NA	n342787	NM_004716_dup1	chr11	117066329	117072630	1242	-	chr11	117075788	117102811	3484	-	*		0.9482	1	0	0.26	0.05	10.76	11.4	11.07
cis_mRNA_dw20k	19248	NA	n345569	NM_000998_dup1	chr2	217385437	217394308	1053	+	chr2	217363520	217366190	420	+	*		0.9916	0.866	0.08	0.04	0.04	1189.3	1019.04	991.25
cis_mRNA_overlap	0	Lnc-CompleteIn-mRNAExon	n409262	NM_000373_dup1	chr3	124449213	124468119	6583	+	chr3	124449213	124468120	6738	+	*		-0.9417	-1	0.45	0.56	0.81	1.07	0.5	0.13
cis_mRNA_overlap	0	mRNA	n332874	NM_005831_dup1	chr17	46933166	46933632	467	+	chr17	46908372	46942229	3282	+	*		-0.9948	-1	0.32	0.44	0.36	3.23	2.98	3.17
cis_mRNA_dw20k	8133	NA	n379485	NM_006961_dup1	chr16	71496181	71499844	2793	+	chr16	71507976	71523254	2743	-	*		0.8778	1	0.1	0.19	0.21	1.41	1.54	1.76
cis_mRNA_overlap	0	Lnc-AntiOverlap-mRNA	n410949	NM_003168_dup1	chr17	56406299	56431088	2278	+	chr17	56422539	56429563	1489	-	*		0.9328	1	1.19	1.61	0.88	30.25	32.93	30.03
cis_mRNA_dw20k	7792	NA	n409679	NM_003922_dup1	chr15	63879909	63893026	1722	-	chr15	63900817	64126147	15197	-	*		0.8437	1	0.24	0.4	0.34	10.64	13.1	10.91
cis_mRNA_dw20k	7390	NA	n371524	NM_003604_dup1	chrX	107963202	107968338	4659	-	chrX	107975727	107979607	3881	-	*		0.9762	1	0.03	0.01	0.06	0.03	0.02	0.07
cis_mRNA_dw20k	5013	NA	LTCONS_00007576	NM_005101_dup1	chr1	954932	955334	403	.	chr1	948847	949920	667	+	*		-0.9904	-1	1.24	1.39	1.28	1.63	0.94	1.36
cis_mRNA_overlap	0	Lnc-CompleteIn-mRNAExon	n408251	NM_003347_dup1	chr22	21921957	21978323	2811	+	chr22	21921957	21978323	2907	+	*		-0.9896	-1	1.82	1.53	1.7	26.63	29.32	28.08
cis_mRNA_overlap	0	mRNA	LTCONS_00016271	NM_003877_dup1	chr12	93964039	93970074	2883	+	chr12	93963598	93969978	2210	+		-1072.56	0.9604	1	2.13	1.82	2.22	1.36	1.24	1.47
cis_mRNA_up10k	6805	NA	n373022	NM_003482_dup1	chr12	49455911	49457826	811	+	chr12	49412758	49449107	19419	-	*		1	1	0	0.04	0.08	2.04	2.37	2.71
cis_mRNA_overlap	0	Lnc-AntiOverlap-mRNA	n378317	NM_014240_dup1	chr3	45720724	45730626	955	-	chr3	45636323	45722755	6284	+	*		0.9884	1	0.06	0	0.11	2	1.83	2.08
cis_mRNA_dw20k	3615	NA	n339642	NM_014779_dup1	chr3	150181245	150184205	2959	+	chr3	150126788	150177631	3901	+	*		0.9966	1	1.77	2.38	1.96	1.18	1.35	1.22
cis_mRNA_dw20k	4582	NA	n335098	NM_014865_dup1	chr12	6645713	6646179	246	+	chr12	6604240	6641132	4785	+	*		-0.8837	-1	0.34	0.31	0.15	2.59	3.24	3.68
tran	NA	NA	LTCONS_00018887	NM_000992_dup1	chr12	95861359	95861645	287	.	chr3	52027644	52029958	695	-		-613.4	-0.9224	-1	0.28	0.39	0.51	210.65	192.69	189.2
cis_mRNA_up10k	212	NA	n406526	NM_003312_dup1	chr22	37415702	37425863	1614	+	chr22	37406906	37415491	1128	-	*		-0.9862	-0.866	0.34	0.3	0.3	1.23	1.65	1.74
cis_mRNA_dw20k	13015	NA	LTCONS_00047540	NM_004175_dup1	chr22	24981523	24989035	2657	-	chr22	24951618	24968509	1385	+	*		-0.6529	-1	6.28	6.61	6.62	39.34	39.09	37.89
cis_mRNA_overlap	0	Lnc-CompleteIn-mRNAExon	n339510	NM_021964_dup1	chr3	124945219	124945675	456	-	chr3	124944513	125094198	9543	-	*		-0.8989	-1	1.08	0.74	2.29	10.28	10.84	9.94
cis_mRNA_overlap	0	Lnc-AntiOverlap-mRNA	n339179	NM_004765_dup1	chr16	30887233	30906541	1125	+	chr16	30899116	30905399	858	-	*		-0.9991	-1	1.42	2.65	1.03	30.23	22.01	33.42
cis_mRNA_overlap	0	Lnc-CompleteIn-mRNAExon	n339291	NM_003149_dup1	chr3	36422097	36589496	2963	+	chr3	36422097	36589496	2963	+	*		1	1	0.53	1.43	0.64	0.53	1.43	0.64
cis_mRNA_up10k	692	NA	n379350	NM_001046_dup1	chr5	127276118	127418792	1380	-	chr5	127419483	127525380	6860	+	*		0.9957	1	0.97	0.88	1.61	0.36	0.35	0.78
cis_mRNA_up10k	7687	NA	n386301	NM_003906_dup1	chr21	47712922	47714711	827	+	chr21	47655048	47705236	6113	-	*		0.9055	1	0.71	0.93	0.26	5.65	6.7	5.23
cis_mRNA_up10k	2276	NA	n369186	NM_003913_dup1	chr6	4016675	4019294	2368	-	chr6	4021569	4065217	7482	+	*		0.9939	1	1.15	1.07	1.04	3.34	2.43	1.89
cis_mRNA_up10k	8409	NA	n378109	NM_021959_dup1	chr6	30002712	30026524	700	-	chr6	30034932	30038110	1664	+	*		0.9996	1	0.02	0	0.12	30.52	29.64	35.95
cis_mRNA_overlap	0	Lnc-AntiOverlap-mRNA	n339096	NM_005768_dup1	chr12	7087683	7089096	1414	+	chr12	7085347	7125842	2297	-	*		-0.9624	-1	2.43	2.17	2.52	10.12	11.71	8.52
cis_mRNA_dw20k	5770	NA	n375403	NM_003223_dup1	chr16	4295967	4301418	1192	-	chr16	4307187	4323001	2140	-	*		0.9999	1	0.22	0	0.13	2.53	2.1	2.36
cis_mRNA_up10k	5977	NA	n378352	NM_021959_dup1	chr6	30022378	30028956	3294	-	chr6	30034932	30038110	1664	+	*		0.9792	1	0.33	0.29	0.41	30.52	29.64	35.95
cis_mRNA_up10k	1180	NA	n364851	NM_003108_dup1	chr2	5742637	5831620	468	-	chr2	5832799	5841517	8719	+	*		0.895	1	0.08	0	0.07	102.47	56.03	77.09

cis_mRNA_up10k	121	NA	n365884	NM_004157_dup1	chr3	48885390	48889414	3447	+	chr3	48788093	48885270	2381	-	*		0.9959	1	0.47	0.68	0.6	28.23	29.23	28.77
cis_mRNA_overlap	0	mRNA	LTCONS_00042498	NM_001105537_dup1	chr2	219519520	219524344	2485	-	chr2	219502639	219524261	5923	-	*		-0.8091	-0.866	1.05	0.83	0.96	0.64	0.79	0.79
cis_mRNA_dw20k	3850	NA	n380037	NM_003353_dup1	chr2	27505301	27526416	876	-	chr2	27530265	27531130	615	-	*		-0.9996	-0.866	0.08	0.04	0.04	0.53	1.19	1.17
cis_mRNA_dw20k	6984	NA	n345527	NM_002908_dup1	chr2	61157161	61167156	2794	+	chr2	61108752	61150178	2592	+	*		-0.9904	-1	0.79	0.66	0.5	0.22	0.51	0.73
tran	NA	NA	LTCONS_00007600	NM_014631_dup1	chr1	43876648	43877437	790	.	chr10	105353784	105615164	11247	-		-1406.9	0.9274	1	0.5	0.74	0.81	4.26	4.52	4.86
cis_mRNA_up10k	96	NA	n363878	NM_005482_dup1	chr1	77685227	77686671	311	+	chr1	77554666	77685132	4626	-	*		-0.6428	-1	0.39	0.35	0.81	6.59	7.44	6.49
tran	NA	NA	LTCONS_00032061	NM_001029_dup1	chr17_ctg5_hap1	1181916	1182256	341	.	chr12	56435686	56438007	682	+		-739.6	0.9959	1	0.46	0.95	1.55	127.6	132.07	139.67
cis_mRNA_overlap	0	Lnc-CompleteIn-mRNAIntron	n386977	NM_006041_dup1	chr17	14207057	14209061	2005	+	chr17	14204506	14249492	2032	+	*		0.9566	1	3.86	4.48	3.16	3.82	4.11	2.68
cis_mRNA_overlap	0	CompleteIn-LncExon	LTCONS_00029268	NM_005801_dup1	chr17	39845127	39850630	4766	+	chr17	39845127	39847898	1326	+		-742.25	0.9396	1	5.35	4.87	4.44	45.85	43.93	36.93
cis_mRNA_up10k	258	NA	n377824	NM_003295_dup1	chr13	45915554	45944051	744	+	chr13	45911304	45915297	829	-	*		0.8044	0.866	0.36	0.36	0.15	358.65	311.99	280.6
cis_mRNA_dw20k	2274	NA	n335622	NM_002804_dup1	chr11	47437352	47438047	696	-	chr11	47440320	47448024	1580	-	*		0.8572	1	0.31	0.24	0.19	94.84	94.74	93.69
cis_mRNA_dw20k	17745	NA	n375097	NM_003041_dup1	chr16	31519835	31520850	880	+	chr16	31494439	31502091	2273	+	*		-0.9963	-1	0.49	0.52	0.4	0.08	0.07	0.13
cis_mRNA_dw20k	4316	NA	LTCONS_00011035	NM_003061_dup1	chr10	98749489	98753480	3992	.	chr10	98757795	98945683	7931	-	*		0.9325	1	1.05	1.16	1.32	7.53	7.84	13.34
cis_mRNA_dw20k	2219	NA	n346398	NM_007371_dup1	chr9	136890612	136893209	2178	+	chr9	136895427	136933141	5654	-	*		0.9883	1	0.53	1.24	0	5.37	5.88	4.72
cis_mRNA_overlap	0	Lnc-AntiOverlap-mRNA	LTCONS_00024835	NM_004855_dup1	chr15	55608294	55611339	2007	-	chr15	55611133	55647846	2197	+	*		-0.9529	-0.866	1.76	1.67	1.47	1.55	1.55	2.38
cis_mRNA_up10k	5649	NA	n383684	NM_005735_dup1	chr2	98286209	98319519	1356	+	chr2	98272402	98280561	2258	-	*		0.9435	1	0.26	0.19	0.43	28.7	28.5	28.84
tran	NA	NA	LTCONS_00016475	NM_005431_dup1	chr12	111051832	111068271	2207	+	chr7	152343571	152373250	3085	-		-909.71	0.9248	1	1.7	1.82	1.74	0.82	1.19	1.07
cis_mRNA_up10k	1197	NA	LTCONS_00010017	NM_003055_dup1	chr10	50802466	50817151	14686	-	chr10	50818347	50820766	2420	+	*		0.9831	1	2.61	3.08	2.52	75.8	88.94	69.21
cis_mRNA_dw20k	1487	NA	n341975	NM_004664_dup1	chr12	81186299	81189685	3387	-	chr12	81191171	81331694	1230	-	*		0.9975	1	23.1	32.07	16.8	22.67	31.11	15.05
cis_mRNA_overlap	0	mRNA	n340111	NM_014803_dup1	chr10	97889829	97965044	1594	+	chr10	97889472	97923517	8295	+	*		0.9074	0.866	0.35	0.28	0.28	4.7	4.33	4.06
cis_mRNA_overlap	0	Lnc-AntiOverlap-mRNA	n343025	NM_005778_dup1	chr3	50137036	50138421	1386	-	chr3	50126352	50156393	3116	+	*		-0.9707	-0.866	0.03	0	0.04	30.15	30.34	30.15
cis_mRNA_overlap	0	Lnc-CompleteIn-mRNAExon	n340774	NM_014757_dup1	chr5	179159851	179204277	5713	+	chr5	179159851	179204287	5723	+	*		0.9427	1	0	0.03	0.02	3.76	4.6	4.07
cis_mRNA_up10k	302	NA	n364207	NM_005399_dup1	chr1	146644430	146645134	604	-	chr1	146626685	146644129	5423	-	*		0.9853	0.866	0.06	0.11	0.11	20.18	22.2	22.65
cis_mRNA_dw20k	6214	NA	n345663	NM_003059_dup1	chr5	131686112	131701412	2243	+	chr5	131630145	131679899	2192	+	*		-0.9347	-0.866	0.13	0.1	0.1	0.41	0.91	0.73
cis_mRNA_overlap	0	CompleteIn-LncExon	n410145	NM_006969_dup1	chr19	53300661	53324922	4712	-	chr19	53300661	53324922	4594	-	*		-0.9592	-1	2.32	2.27	2.68	1.06	1.6	0.22
cis_mRNA_overlap	0	AntiCompleteIn-mRNAIntron	n340759	NM_003488_dup1	chr17	55169506	55173788	4282	-	chr17	55162553	55198710	3968	+	*		-0.6063	-0.866	0	0.01	0.01	23.7	22.65	16.1
cis_mRNA_up10k	771	NA	n339233	NM_003670_dup1	chr3	5018474	5020327	1854	-	chr3	5021097	5026866	3035	+	*		0.9148	1	0.04	0.19	0.02	0.21	0.34	0.08
tran	NA	NA	LTCONS_00063865	NM_002931_dup1	chr6_cox_hap2	4621622	4622011	390	.	chr6_mcf_hap5	4650047	4654463	1744	+		-300.3	-0.9405	-1	1.28	2.46	1.77	13.25	12.32	12.58
cis_mRNA_up10k	121	NA	n368817	NM_003913_dup1	chr6	4019077	4021449	209	-	chr6	4021569	4065217	7482	+	*		0.9297	0.866	0.42	0.19	0.19	3.34	2.43	1.89
cis_mRNA_dw20k	2685	NA	n340381	NM_005885_dup1	chr5	10438175	10440499	2325	+	chr5	10353828	10435491	4611	+	*		0.9766	1	2.36	2.56	1.76	36.1	39.46	31.9
cis_mRNA_dw20k	3136	NA	n369093	NM_002912_dup1	chr6	111598892	111617099	663	+	chr6	111620234	111804414	10701	-	*		1	0.866	1.27	1.81	1.27	9.89	11.81	9.9
cis_mRNA_dw20k	11144	NA	n377610	NM_003913_dup1	chr6	4076360	4079955	3596	+	chr6	4021569	4065217	7482	+	*		0.9254	1	0.36	0.34	0.29	3.34	2.43	1.89
cis_mRNA_overlap	0	Lnc-AntiOverlap-mRNA	n407229	NM_001070_dup1	chr17	40731526	40761445	3778	-	chr17	40761358	40767256	1951	+	*		-0.9993	-1	1.81	1.65	1.24	14.5	15.14	17.1
cis_mRNA_dw20k	12848	NA	n408126	NM_002929_dup1	chr13	114451484	114454062	2579	-	chr13	114321594	114438637	2100	+	*		-0.9707	-1	0.07	0	0.14	0.08	0.1	0.03
cis_mRNA_up10k	262	NA	LTCONS_00060877	NM_002912_dup1	chr6	111804675	111899383	2131	+	chr6	111620234	111804414	10701	-	*		0.7163	1	0	0.58	0.42	9.89	11.81	9.9
cis_mRNA_up10k	6618	NA	n345225	NM_000321_dup1	chr13	48863195	48871266	1406	-	chr13	48877883	49056026	4772	+	*		-0.9795	-1	0.07	0.02	0	11.6	12.34	12.41
cis_mRNA_up10k	5927	NA	n405444	NM_007112_dup1	chr1	155183616	155197325	2036	-	chr1	155165379	155177690	3147	-	*		0.9995	1	0.22	0.52	0	5.1	6.43	4.01
cis_mRNA_dw20k	13331	NA	n342072	NM_003440_dup1	chr12	133697590	133698610	1021	-	chr12	133657037	133684260	3067	+	*		0.9501	1	4.11	3.15	3.38	7.16	6.24	6.72
cis_mRNA_up10k	5879	NA	n326324	NM_004905_dup1	chr1	173438987	173440608	775	-	chr1	173446486	173457946	1670	+	*		0.9097	1	0.37	1.22	0.86	19.53	20.77	19.77
cis_mRNA_overlap	0	Lnc-CompleteIn-mRNAExon	n335579	NM_014752_dup1	chr11	74676920	74688085	366	+	chr11	74660292	74690076	2708	+	*		0.8682	0.866	0.13	0.13	0.12	16.09	14.79	13.47
cis_mRNA_overlap	0	CompleteIn-LncExon	n408250	NM_003347_dup1	chr22	21921957	21978323	3016	+	chr22	21921957	21978323	2907	+	*		-0.9831	-1	0.56	0.09	0.23	26.63	29.32	28.08
cis_mRNA_up10k	1550	NA	n364852	NM_003108_dup1	chr2	5758459	5831250	1058	-	chr2	5832799	5841517	8719	+	*		0.9502	1	0.15	0	0.11	102.47	56.03	77.09
cis_mRNA_dw20k	2076	NA	n341048	NM_003273_dup1	chr11	64885782	64886583	800	+	chr11	64879341	64883707	1586	+	*		0.9933	1	3.4	4.73	1.92	70.45	77.06	65.54
cis_mRNA_dw20k	3828	NA	LTCONS_00037119	NM_021089_dup1	chr19	58811081	58816490	5410	.	chr19	58790318	58807254	2210	+	*		-1	-1	4.96	5.06	4.85	12.39	12.09	12.73
cis_mRNA_dw20k	7717	NA	n342191	NM_004267_dup1	chr3	142849528	142851286	1759	+	chr3	142838668	142841812	3043	+	*		0.9961	1	0.14	0.11	0.3	2.65	2.32	7.31
cis_mRNA_dw20k	6763	NA	n406867	NM_004732_dup1	chr17	7816640	7819265	2525	-	chr17	7826027	7832753	1608	-	*		-0.9871	-1	1.72	2.19	1.95	0.27	0	0.1
cis_mRNA_dw20k	12133	NA	n337645	NM_003036_dup1	chr1	2253784	2255494	1711	-	chr1	2160134	2241652	5707	+	*		0.822	0.866	0.04	0.37	0.04	1.57	1.64	1.61
cis_mRNA_dw20k	4308	NA	LTCONS_00034312	NM_002812_dup1	chr19	38878771	38880568	1798	+	chr19	38865190	38874464	1539	+	*		-0.9667	-1	0.77	0.96	0.72	121.76	120.06	123.11
cis_mRNA_overlap	0	Lnc-AntiOverlap-mRNA	LTCONS_00000774	NM_004814_dup1	chr1	31756623	31764094	4297	+	chr1	31731956	31769644	1638	-	*		0.9151	1	0.12	0.18	0.05	34.67	34.7	34.28
cis_mRNA_dw20k	17865	NA	n376342	NM_000455_dup1	chr19	1246298	1248229	673	-	chr19	1205798	1228434	3276	+	*		-0.9984	-0.866	0.12	0.27	0.28	10.95	10.65	10.65
cis_mRNA_overlap	0	mRNA	n325907	NM_005063_dup1	chr10	102123362	102133115	2551	+	chr10	102106772	102124588	5468	+	*		-0.92	-0.866	0.01	0.01	0.03	212.72	264.6	133.22
cis_mRNA_dw20k	13388	NA	n340905	NM_006298_dup1	chr6	28138623	28140299	1677	+	chr6	28109716	28125236	5362	+	*		0.6983	0.866	0.54	0.54	0.48	10.26	11.13	9.96
cis_mRNA_up10k	3713	NA	n323799	NM_005634_dup1	chrX	139590937	139597536	323	-	chrX	139585152	139587225	2074	-	*		-0.7711	-1	1.27	1.02	0.95	0.15	0.18	0.34
cis_mRNA_overlap	0	mRNA-AntiCompleteIn-LncIntron	LTCONS_00030294	NM_002798_dup1	chr17	4688580	4710311	465	-	chr17	4699457	4701790	826	+	*		0.9709	1	3.14	0.3	1.21	119.05	118.35	118.41
cis_mRNA_overlap	0	CompleteIn-LncIntron	LTCONS_00019714	NM_005986_dup1	chr13	112517175	112764886	5604	+	chr13	112721913	112726020	4108	+	*		0.7559	0.866	0.46	0.72	0.33	0.01	0.01	0
cis_mRNA_dw20k	4	NA	LTCONS_00041143	NM_014763_dup1	chr2	75889337	75904999	12571	-	chr2	75873909	75889334	7827	+	*		0.9078	1	0.1	0.12	0.11	3.85	4.85	3.95

cis_mRNA_overlap	0	Lnc-AntiOverlap-mRNA	n379970	NM_003703_dup1	chr4	2937273	2943596	3006	+	chr4	2939663	2965118	2918	-	*		-0.9172	-1	0.93	1.09	1.27	0.45	0.42	0.11
cis_mRNA_dw20k	9767	NA	n345568	NM_021141_dup1	chr2	217080782	217084704	1936	+	chr2	216974020	217071016	3430	+	*		0.9929	1	7.47	7.64	8.37	148.41	148.89	155.61
cis_mRNA_overlap	0	CompleteIn-LncExon	n409669	NM_003137_dup1	chr6	35800811	35888957	4390	-	chr6	35800811	35888957	4362	-	*		-0.9477	-1	4.42	2.55	0.53	31.53	32.03	34.21
cis_mRNA_up10k	65	NA	n370208	NM_003844_dup1	chr8	23082744	23088426	1125	-	chr8	23048970	23082680	1750	-	*		-0.7559	-0.866	0.06	0.03	0.05	0	0.02	0.02
cis_mRNA_dw20k	17054	NA	LTCONS_00028555	NM_004505_dup1	chr17	5095377	5138931	1584	+	chr17	5031687	5078324	7971	+	*		0.777	1	0.78	0.93	0.41	0.17	0.77	0.11
cis_mRNA_dw20k	10753	NA	n339129	NM_021141_dup1	chr2	217081768	217084913	3146	+	chr2	216974020	217071016	3430	+	*		0.9058	1	27.78	30.07	32.58	148.41	148.89	155.61
cis_mRNA_overlap	0	Lnc-CompleteIn-mRNAExon	n342494	NM_003791_dup1	chr16	84087371	84150511	4338	-	chr16	84087368	84150517	4347	-	*		-0.997	-1	0	2.85	8.58	14.19	9.96	3.65
cis_mRNA_up10k	5429	NA	n340469	NM_002798_dup1	chr17	4690328	4694029	2826	-	chr17	4699457	4701790	826	+	*		0.9998	1	5.89	5.41	5.44	119.05	118.35	118.41
cis_mRNA_dw20k	2260	NA	n370008	NM_003941_dup1	chr7	123315994	123319730	1031	-	chr7	123321989	123389116	4430	-	*		0.8629	1	0.24	0.28	0.34	6.44	7.48	7.62
cis_mRNA_dw20k	6101	NA	n346026	NM_006315_dup1	chr4	770528	775635	4025	-	chr4	699573	764428	5666	+	*		0.9926	1	11.69	14.9	10.71	18.24	19.51	17.57
cis_mRNA_dw20k	687	NA	n339254	NM_004625_dup1	chr3	13858242	13859396	1155	-	chr3	13860082	13921618	1714	-	*		0.9917	1	0	0.14	0.03	0.08	0.26	0.14
cis_mRNA_dw20k	4436	NA	n346007	NM_006773_dup1	chr2	118594388	118603633	3105	+	chr2	118572255	118589953	3765	+	*		-0.9432	-1	0.21	0.25	0.44	28.01	25.95	23.73
cis_mRNA_up10k	6248	NA	n338992	NM_001009_dup1	chr19	58879170	58892389	1853	-	chr19	58898636	58906171	741	+	*		-0.8514	-1	0	0.26	0.29	379.13	344.28	290.48
cis_mRNA_up10k	2067	NA	n375270	NM_005853_dup1	chr16	54958849	54963045	641	-	chr16	54965111	54968397	2064	+	*		0.9895	1	1.39	1.61	1.51	2.1	3.22	2.57
cis_mRNA_dw20k	14222	NA	n341236	NM_002787_dup1	chr7	42940871	42942239	1369	-	chr7	42956460	42971805	1466	-	*		0.9981	1	0.41	0.19	0.2	100.95	92.92	93.82
cis_mRNA_overlap	0	mRNA	n333425	NM_001029_dup1	chr12	56436232	56438028	410	+	chr12	56435686	56438007	682	+	*		0.9175	1	0	0.19	0.26	127.6	132.07	139.67
cis_mRNA_up10k	113	NA	LTCONS_00055393	NM_004898_dup1	chr4	56413109	56414155	1047	.	chr4	56298660	56412997	5801	-	*		-0.7954	-1	0.5	0.4	0.15	1.18	1.74	1.82
cis_mRNA_overlap	0	mRNA	n341283	NM_003378_dup1	chr7	100806996	100808145	1150	-	chr7	100805790	100808852	2553	-	*		0.9491	1	14.91	17.51	22.61	47.92	48.64	82.94
cis_mRNA_dw20k	11053	NA	n333018	NM_022551_dup1	chr3	101280746	101284012	500	+	chr3	101295064	101296275	549	-	*		-0.9638	-1	0	0.42	0.24	0.46	0.35	0.37
cis_mRNA_overlap	0	Lnc-AntiOverlap-mRNA	n341898	NM_003463_dup1	chr6	64280910	64282308	1399	-	chr6	64281920	64293489	5082	+	*		0.9936	1	0.12	0	0.09	11.42	0.7	9.92
cis_mRNA_overlap	0	Lnc-CompleteIn-mRNAIntron	n325836	NM_021134_dup1	chr11	1968902	1971275	722	+	chr11	1968502	1977839	742	+	*		-0.9443	-0.866	0.45	0.45	0.31	30.32	27.21	36.49
cis_mRNA_dw20k	18497	NA	n333550	NM_004579_dup1	chr11	64537502	64538113	409	-	chr11	64556609	64570713	2955	-	*		0.9514	1	1.67	3.39	2.45	2.72	3.46	3.26
cis_mRNA_up10k	331	NA	n378555	NM_002912_dup1	chr6	111804744	111898363	1106	+	chr6	111620234	111804414	10701	-	*		0.6504	1	0.19	1.82	1.51	9.89	11.81	9.9
cis_mRNA_overlap	0	mRNA	n409439	NM_006917_dup1	chr1	165406165	165414592	785	-	chr1	165370159	165414592	2195	-	*		0.997	1	1.1	0.39	0.56	28.2	17.45	19.22
cis_mRNA_up10k	75	NA	LTCONS_00059151	NM_003862_dup1	chr5	170845981	170846593	613	.	chr5	170846667	170884630	1982	+	*		0.9397	1	1.25	0.54	2.18	10.58	8.66	11.25
cis_mRNA_up10k	301	NA	n380333	NM_002912_dup1	chr6	111804714	111814206	2191	+	chr6	111620234	111804414	10701	-	*		1	0.866	0.21	0.42	0.21	9.89	11.81	9.9
tran	NA	NA	LTCONS_00032068	NM_005730_dup1	chr17_gl000205_random	39001	40208	1208	+	chr12	58213710	58240747	5020	-		-1469.79	0.9927	1	22.7	23.16	4.54	3.47	3.6	2.75
cis_mRNA_overlap	0	Lnc-CompleteIn-mRNAIntron	n383003	NM_004594_dup1	chr16	67295831	67297687	1857	+	chr16	67282855	67306094	3735	+	*		0.992	1	0.22	0.65	0.24	0.79	1.52	0.92
cis_mRNA_up10k	377	NA	n380136	NM_003295_dup1	chr13	45915673	45952755	563	+	chr13	45911304	45915297	829	-	*		0.9756	1	1.16	0.69	0	358.65	311.99	280.6
cis_mRNA_dw20k	3238	NA	LTCONS_00037102	NM_004596_dup1	chr19	41274534	41274922	389	.	chr19	41256759	41271297	1629	+	*		-0.9984	-1	0.99	0.27	0.35	7.83	10.25	9.84
cis_mRNA_up10k	223	NA	n407088	NM_004747_dup1	chr10	79686570	79689583	3014	+	chr10	79550549	79686348	7479	-	*		0.8076	1	0.37	0.42	0.26	12.25	13.93	12.03
cis_mRNA_up10k	124	NA	n374497	NM_003134_dup1	chr15	40331512	40359491	2317	+	chr15	40327891	40331389	1126	-	*		-0.8415	-1	0.1	0.35	0.38	143.07	138.25	130.17
cis_mRNA_up10k	1031	NA	LTCONS_00007577	NM_003036_dup1	chr1	2158816	2159104	289	.	chr1	2160134	2241652	5707	+	*		0.9042	0.866	0	0.13	0.13	1.57	1.64	1.61
cis_mRNA_dw20k	1517	NA	n372447	NM_014752_dup1	chr11	74691592	74695088	1588	+	chr11	74660292	74690076	2708	+	*		0.9084	1	1.44	1.41	1.13	16.09	14.79	13.47
cis_mRNA_dw20k	2204	NA	n378407	NM_007371_dup1	chr9	136890617	136893224	2300	+	chr9	136895427	136933141	5654	-	*		-0.9994	-1	5.14	3.81	7.05	5.37	5.88	4.72
cis_mRNA_dw20k	1548	NA	n382966	NM_004604_dup1	chr16	31053032	31054276	1131	+	chr16	31044903	31051485	1386	+	*		-0.9935	-1	0.86	0.37	0.31	6.35	7.43	7.74
cis_mRNA_up10k	355	NA	n373462	NM_014718_dup1	chr12	7281677	7282613	512	-	chr12	7282967	7311541	4013	+	*		0.997	1	0.34	0.53	0.32	59.23	72.64	56.41
cis_mRNA_dw20k	13315	NA	n340618	NM_003885_dup1	chr17	30831585	30832662	1078	-	chr17	30814105	30818271	3854	+	*		-0.6023	-0.866	0.03	0.03	0	50.38	46.86	50.92
cis_mRNA_dw20k	10555	NA	n341810	NM_021138_dup1	chr9	139831621	139832440	820	-	chr9	139780965	139821067	2277	+	*		-0.9489	-1	0.61	0.52	0.74	3.29	3.58	3.16
cis_mRNA_dw20k	4116	NA	LTCONS_00032018	NM_002815_dup1	chr17	30812157	30814084	1928	.	chr17	30771502	30808042	1581	+	*		-0.9831	-1	1.2	1.17	1.5	27.32	27.72	26.25
cis_mRNA_up10k	22	NA	LTCONS_00032018	NM_003885_dup1	chr17	30812157	30814084	1928	.	chr17	30814105	30818271	3854	+	*		0.6659	1	1.2	1.17	1.5	50.38	46.86	50.92
cis_mRNA_overlap	0	Lnc-AntiOverlap-mRNA	n380038	NM_003703_dup1	chr4	2937273	2952794	3860	+	chr4	2939663	2965118	2918	-	*		-0.7241	-1	0.47	0.87	0.98	0.45	0.42	0.11
cis_mRNA_dw20k	4550	NA	n381057	NM_006511_dup1	chr1	15992766	15995535	2770	+	chr1	15986364	15988217	1854	+	*		0.9448	1	3.74	4.17	4.04	3.59	4.43	3.93
cis_mRNA_overlap	0	AntiCompleteIn-mRNAIntron	n377763	NM_014629_dup1	chr8	1774313	1784348	1501	-	chr8	1772149	1906807	5591	+	*		-0.9449	-1	0.04	0.05	0.06	1.76	1.67	1.31
cis_mRNA_dw20k	10828	NA	n376502	NM_003425_dup1	chr19	44387820	44405949	1232	-	chr19	44416776	44439411	3903	-	*		0.9939	1	0.35	0.41	0.42	3.14	4.3	4.33
cis_mRNA_overlap	0	Lnc-AntiOverlap-mRNA	n409180	NM_013398_dup1	chr19	44609492	44617336	3361	-	chr19	44598482	44612479	2483	+	*		-0.908	-1	17.7	16.09	13.78	0.89	1.75	1.97
cis_mRNA_up10k	262	NA	n409708	NM_002912_dup1	chr6	111804675	111923497	4651	+	chr6	111620234	111804414	10701	-	*		0.9754	1	0	0.68	0.16	9.89	11.81	9.9
cis_mRNA_up10k	2042	NA	LTCONS_00073484	NM_003863_dup1	chr9	130702804	130707636	4741	-	chr9	130697374	130700763	1546	-	*		0.9512	1	0.38	0.56	0.69	2.48	2.81	3.56
cis_mRNA_up10k	2422	NA	n375011	NM_005853_dup1	chr16	54952778	54962690	921	-	chr16	54965111	54968397	2064	+	*		-0.9973	-1	0.62	0	0.32	2.1	3.22	2.57
cis_mRNA_up10k	80	NA	n341793	NM_003934_dup1	chr9	133452741	133454881	2141	-	chr9	133454960	133513739	3157	+	*		0.9998	1	1.2	1.61	1.31	3.54	5.34	3.99
cis_mRNA_up10k	615	NA	n409454	NM_006278_dup1	chr11	126211612	126225482	752	-	chr11	126226096	126284533	1743	+	*		-0.9796	-1	1.53	1.15	0.25	0.46	0.47	0.56
cis_mRNA_dw20k	7010	NA	n408333	NM_006062_dup1	chr2	73461364	73480150	1883	+	chr2	73441366	73454355	2567	+	*		0.8077	0.866	0.17	0.17	0.62	9.01	10.03	10.73
cis_mRNA_dw20k	13771	NA	n342783	NM_003478_dup1	chr11	107992258	108018275	1517	+	chr11	107879408	107978488	6387	+	*		0.8515	0.866	44.15	37.1	51.93	12.49	12.27	12.49
cis_mRNA_overlap	0	Lnc-AntiOverlap-mRNA	n405483	NM_005105_dup1	chr1	145509820	145516076	6257	-	chr1	145507557	145513536	4960	+	*		-0.9446	-1	0.32	0.21	0.24	41.46	43.98	42.55
cis_mRNA_up10k	8915	NA	n405483	NM_003637_dup1	chr1	145509820	145516076	6257	-	chr1	145524990	145543868	5170	+	*		-0.9954	-1	0.32	0.21	0.24	0.23	0.34	0.32
cis_mRNA_up10k	179	NA	n385423	NM_003821_dup1	chr8	90729656	90769797	1430	-	chr8	90769975	90803292	2585	+	*		-0.9006	-1	0.72	0.75	0.79	1	0.85	0.82
cis_mRNA_overlap	0	Lnc-AntiOverlap-mRNA	n377923	NM_006516_dup1	chr1	43424775	43442099	3075	+	chr1	43391046	43424847	3670	-	*		-0.9664	-0.866	0.07	0.34	0.07	10.81	9.12	10.36
cis_mRNA_dw20k	6291	NA	n338562	NM_005646_dup1	chr1	234510108	234520769	1329	-	chr1	234527059	234614849	5130	-	*		-0.9855	-1	0.33	0.35	0.32	1.54	1.39	1.68
cis_mRNA_up10k	124	NA	n411590	NM_003134_dup1	chr15	40331512	40359710	2819	+	chr15	40327891	40331389	1126	-	*		0.955	1	0.11	0.04	0	143.07	138.25	130.17
cis_mRNA_up10k	122	NA	LTCONS_00024721	NM_003758_dup1	chr15	44824202	44829145	4659	-	chr15	44829266	44855001	2558	+	*		0.8858	1	1.09	0.95	0.93	20.44	20.27	20.05
cis_mRNA_overlap	0	mRNA	n410130	NM_021645_dup1	chr13	52586523	52603780	1402	+	chr13	52598827	52607736	5529	+	*		0.821	1	0.06	0.12	0.37	9.83	10.56	10.82

cis_mRNA_up10k	224	NA	n372419	NM_012193_dup1	chr11	86666663	86711987	1207	+	chr11	86656717	86666440	7394	-	*		0.9336	1	0	0.13	0.28	0.87	1.03	2.14
cis_mRNA_up10k	121	NA	n342902	NM_003211_dup1	chr12	104343980	104359473	1714	-	chr12	104359593	104382656	3244	+	*		0.9705	1	0.41	0.21	0.42	35.13	31.02	36.81
cis_mRNA_dw20k	5112	NA	n339128	NM_021141_dup1	chr2	217076127	217078242	2116	+	chr2	216974020	217071016	3430	+	*		0.9696	1	25.49	26.9	30.05	148.41	148.89	155.61
cis_mRNA_dw20k	15894	NA	n345379	NM_004798_dup1	chr20	30938707	30945410	2778	-	chr20	30865454	30922814	6116	+	*		0.8853	0.866	0.04	0.07	0.07	20.73	25.67	29.97
cis_mRNA_up10k	2104	NA	LTCONS_00028400	NM_003223_dup1	chr16	4325104	4325525	422	.	chr16	4307187	4323001	2140	-	*		-0.9581	-1	0.18	1.32	0.32	2.53	2.1	2.36
cis_mRNA_overlap	0	AntiCompleteIn-mRNAIntron	n324818	NM_003253_dup1	chr21	32687310	32688140	831	+	chr21	32490736	32931290	7198	-	*		-0.9681	-1	1.83	1.9	1.4	1.6	1.17	2.31
cis_mRNA_up10k	178	NA	n335716	NM_003592_dup1	chr7	148394642	148395756	1115	-	chr7	148395933	148498202	3209	+	*		0.8627	1	1.11	0.14	0.37	23.24	22.35	22.98
cis_mRNA_dw20k	3301	NA	n342785	NM_003904_dup1	chr11	116644515	116645976	1460	-	chr11	116649276	116658739	1778	-	*		0.9981	1	30.1	21.64	27.85	124.11	78.03	114.51
cis_mRNA_overlap	0	mRNA	n324637	NM_004784_dup1	chr4	118975004	119010551	576	+	chr4	118955500	119179789	5947	+	*		0.9605	0.866	0.06	0.06	0.17	2.42	1.99	3.49
cis_mRNA_overlap	0	mRNA	LTCONS_00038262	NM_021103_dup1	chr2	85131182	85133799	1641	+	chr2	85132763	85133799	482	+		-265.71	0.9851	1	1.66	1.35	1.99	5776.14	4858.31	6303.2
cis_mRNA_up10k	9330	NA	n334153	NM_003913_dup1	chr6	4012036	4012240	205	-	chr6	4021569	4065217	7482	+	*		0.9944	1	0.43	0.2	0	3.34	2.43	1.89
cis_mRNA_up10k	1502	NA	n333488	NM_006049_dup1	chr15	66791647	66791946	300	-	chr15	66782666	66790146	785	-	*		-0.9973	-1	3.23	1.01	0.34	3.42	4.62	4.85
cis_mRNA_dw20k	5439	NA	LTCONS_00033253	NM_006938_dup1	chr18	19215646	19216562	917	.	chr18	19192260	19210208	1600	+	*		-0.8437	-1	0.77	1.41	0.9	33.79	28.95	30.36
cis_mRNA_dw20k	4533	NA	n406621	NM_014760_dup1	chr3	10327438	10335133	1482	+	chr3	10290177	10322906	4907	+	*		-0.9993	-1	0.05	0.19	0	2.8	2.54	2.91
cis_mRNA_overlap	0	Lnc-CompleteIn-mRNAExon	n339894	NM_004852_dup1	chr18	55154943	55158529	3586	+	chr18	55102917	55158530	16122	+	*		0.6355	1	2.15	4.78	2.33	6.01	11.83	11.17
tran	771594	NA	LTCONS_00031237	NM_014834_dup1	chr17	43590814	43600904	6073	-	chr17	44372497	44415160	5177	+		-2298.67	-0.9801	-1	1.1	1.02	1.74	1.46	1.81	0.66
cis_mRNA_overlap	0	Lnc-AntiOverlap-mRNA	n341403	NM_003747_dup1	chr8	9412657	9414030	1374	-	chr8	9413445	9639856	9599	+	*		0.9989	1	3.22	2.42	3.61	37.03	26.04	41.43
cis_mRNA_up10k	262	NA	LTCONS_00060874	NM_002912_dup1	chr6	111804675	111872652	5775	+	chr6	111620234	111804414	10701	-	*		-0.8683	-1	0.02	0	0.01	9.89	11.81	9.9
cis_mRNA_up10k	2759	NA	n409156	NM_007170_dup1	chr1	45959598	45965751	2041	-	chr1	45809555	45956840	3076	-	*		-0.9387	-0.866	0.13	0	0.2	0.19	0.24	0.19
cis_mRNA_overlap	0	Lnc-AntiOverlap-mRNA	n410895	NM_015037_dup1	chr10	75556272	75561157	1460	-	chr10	75545605	75561551	5835	+	*		0.9988	1	0.9	1.02	1.17	8.11	8.87	9.67
cis_mRNA_up10k	170	NA	n339683	NM_016470_dup1	chr20	42839600	42854667	1441	+	chr20	42825136	42839431	1572	-	*		0.9982	1	0.45	0.29	0.18	16.45	14.88	14.02
cis_mRNA_dw20k	1113	NA	n381720	NM_015459_dup1	chr11	63391556	63395325	3770	-	chr11	63396437	63439083	2256	-	*		0.7163	1	1.27	1.88	1.96	1.69	1.81	2.36
cis_mRNA_up10k	66	NA	LTCONS_00019252	NM_015070_dup1	chr13	46626959	46687932	2413	+	chr13	46536314	46626894	6409	-	*		-0.8986	-1	2.91	2.98	2.45	0.42	0.36	0.47
cis_mRNA_overlap	0	Lnc-CompleteIn-mRNAIntron	n338202	NM_006699_dup1	chr1	117963932	117966317	2385	+	chr1	117910085	118068320	5388	+	*		0.8319	1	0.65	0.67	0.9	17.43	19.3	20.45
cis_mRNA_overlap	0	Lnc-CompleteIn-mRNAExon	n410624	NM_006857_dup1	chr2	70121075	70132368	1352	+	chr2	70121075	70132368	1438	+	*		0.9734	1	2.59	2.94	2.19	19.23	23.29	17.22
cis_mRNA_dw20k	11673	NA	LTCONS_00035773	NM_015260_dup1	chr19	17002836	17007813	2706	-	chr19	16940218	16991164	5129	+	*		0.9555	1	2.54	3.79	3.11	0.15	0.64	0.24
cis_mRNA_up10k	60	NA	n381186	NM_007204_dup1	chr1	112287939	112298131	2767	-	chr1	112298190	112310199	3510	+	*		-0.9925	-1	0.04	0.05	0.02	11.37	11.17	12.08
cis_mRNA_overlap	0	CompleteIn-LncIntron	n387042	NM_013337_dup1	chr17	894694	946816	838	+	chr17	900357	905407	1690	+	*		0.9964	0.866	0.04	0.04	0.15	18.31	18.67	22.17
cis_mRNA_up10k	205	NA	n370193	NM_007218_dup1	chr8	125474740	125486804	5639	-	chr8	125487008	125500859	3312	+	*		0.9929	0.866	0.52	0.47	0.52	16.82	16.55	16.86
cis_mRNA_dw20k	4339	NA	n341687	NM_012166_dup1	chr9	37485959	37506551	1562	+	chr9	37510889	37576250	4575	-	*		-0.8401	-1	2.93	3.06	2.59	4.28	4.02	4.35
cis_mRNA_dw20k	12080	NA	LTCONS_00030148	NM_015721_dup1	chr17	624454	635582	1311	-	chr17	647661	655501	3741	-	*		0.9672	1	1.28	1.43	0.92	3.92	4.43	3.45
cis_mRNA_dw20k	404	NA	n411757	NM_016395_dup1	chr15	65871096	65903627	2461	-	chr15	65822827	65870693	3208	+	*		-0.9457	-1	10.82	11.19	7.96	206.66	198	218.43
cis_mRNA_up10k	4384	NA	n410641	NM_015894_dup1	chr20	62289163	62330051	5772	+	chr20	62271061	62284780	2255	-	*		-0.9975	-1	1.56	1.59	1.25	190.09	181.92	232.64
cis_mRNA_up10k	4135	NA	n381272	NM_016371_dup1	chr1	162751901	162756362	4462	+	chr1	162760496	162782608	1519	+	*		0.9985	1	0.42	0.86	0.2	22.81	25.29	21.83
cis_mRNA_up10k	263	NA	n380358	NM_014488_dup1	chr11	82783146	82803380	340	+	chr11	82692478	82782884	1602	-	*		0.9321	1	0	0.4	0.91	1.91	3.34	3.77
cis_mRNA_up10k	3319	NA	n339111	NM_015161_dup1	chr16	18816175	18819185	3011	-	chr16	18802991	18812857	2264	-	*		-0.9408	-1	1.37	1.43	0	150.88	136.45	174.68
cis_mRNA_up10k	504	NA	n345281	NM_016417_dup1	chr14	95998848	96000820	1072	-	chr14	96001323	96011055	1171	+	*		-0.7914	-1	0.61	0.62	0.78	31.76	29.73	28.7
cis_mRNA_dw20k	12145	NA	n381749	NM_007173_dup1	chr11	86534419	86648860	5341	+	chr11	86511491	86522275	3806	+	*		-0.8198	-1	0.13	0.27	0.15	2.04	1.91	1.95
cis_mRNA_dw20k	1908	NA	n333163	NM_006690_dup1	chr20	33866711	33866919	209	-	chr20	33814539	33864804	4333	+	*		0.9879	1	2.12	1.64	2.94	20.21	19.6	22.29
cis_mRNA_overlap	0	mRNA	n338321	NM_014992_dup1	chr14	59655450	59797337	1711	+	chr14	59655399	59836471	4256	+	*		0.9959	1	5.26	6.83	4.36	34.42	37.08	33.37
tran	8058209	NA	LTCONS_00026233	NM_130464_dup1	chr16	29494866	29495623	758	+	chr16	21413455	21436658	3617	-		-1682.4	-0.9347	-0.866	0.14	0.39	0.3	0.88	0.72	0.72
cis_mRNA_overlap	0	Lnc-AntiOverlap-mRNA	n407756	NM_016333_dup1	chr16	2787077	2802601	845	-	chr16	2802330	2821413	9353	+	*		0.9882	1	0	0.12	0.04	7.73	11.58	8.46
cis_mRNA_overlap	0	mRNA	n405969	NM_014937_dup1	chr10	121485609	121551720	950	+	chr10	121485609	121588659	4932	+	*		0.8248	1	0.9	0.13	0.41	19.84	18.58	19.73
cis_mRNA_up10k	4154	NA	LTCONS_00073702	NM_015160_dup1	chr9	139296372	139300963	2989	-	chr9	139305116	139318213	2080	+	*		0.9723	1	3.09	3.34	3.41	17.72	19.02	20.01
cis_mRNA_dw20k	14145	NA	n407074	NM_015144_dup1	chr16	87362942	87425708	3364	-	chr16	87439852	87525460	6911	-	*		0.9728	1	0	0.79	2.32	5.43	5.45	5.6
cis_mRNA_dw20k	7044	NA	n340558	NM_015681_dup1	chr17	19238462	19239440	967	-	chr17	19246483	19266046	928	-	*		-0.9846	-1	0.11	0.17	0.03	3.26	2.65	3.7
cis_mRNA_overlap	0	AntiCompleteIn-mRNAExon	n337633	NM_007220_dup1	chrX	15803839	15805712	1870	-	chrX	15756412	15805748	6032	+	*		-0.9767	-1	0.19	0.07	0.11	1.34	1.78	1.55
cis_mRNA_overlap	0	Lnc-AntiOverlap-mRNA	n410934	NM_032635_dup1	chr19	36031640	36036931	1627	-	chr19	36036545	36038428	851	+	*		0.6765	1	0.74	0.73	0.88	34.48	33.31	34.72
cis_mRNA_overlap	0	Lnc-AntiOverlap-mRNA	n340374	NM_012073_dup1	chr5	10264709	10267258	2546	-	chr5	10250282	10266501	3403	+	*		0.7955	1	0.03	0	0.01	124.5	109.92	123.48
cis_mRNA_up10k	7950	NA	n409775	NM_014049_dup1	chr3	128580351	128590384	1104	-	chr3	128598333	128631957	2575	+	*		-0.9966	-1	0.53	0.48	0.35	5.99	6.16	6.84
cis_mRNA_overlap	0	mRNA	n341084	NM_007173_dup1	chr11	86511597	86663830	2138	+	chr11	86511491	86522275	3806	+	*		-0.8515	-1	0.08	0.13	0.09	2.04	1.91	1.95
cis_mRNA_dw20k	1985	NA	n411738	NM_016113_dup1	chr17	16342301	16344525	635	+	chr17	16318856	16340317	2808	+	*		-0.8671	-1	2.82	0.63	0	1.4	2.29	4.24
cis_mRNA_up10k	38	NA	LTCONS_00042823	NM_015630_dup1	chr2	149402297	149402523	227	.	chr2	149402560	149545136	3641	+	*		-0.9668	-1	0.76	0.35	0.68	17.95	20.18	18.91
cis_mRNA_overlap	0	Lnc-CompleteIn-mRNAIntron	n340002	NM_021252_dup1	chr10	27810962	27811539	574	+	chr10	27793249	27829099	2790	+	*		-0.896	-1	0	0.35	0.11	57.82	56.07	56.56
cis_mRNA_overlap	0	mRNA	n380955	NM_013244_dup1	chr12	87120982	87232905	500	-	chr12	86373037	87232681	1934	-	*		0.8307	1	2.59	1.67	2.74	25.77	16.18	45.46
cis_mRNA_overlap	0	Lnc-AntiOverlap-mRNA	n379320	NM_014480_dup1	chr19	58768639	58790174	2270	-	chr19	58740070	58775010	3512	+	*		-0.9623	-1	0.47	0.71	0.98	12.91	10.57	9.63
cis_mRNA_overlap	0	Lnc-CompleteIn-mRNAIntron	n338996	NM_014900_dup1	chr2	165685630	165688230	2601	-	chr2	165541256	165697928	4923	-	*		0.9878	1	0.01	0	0.02	0.04	0	0.11
cis_mRNA_up10k	32	NA	n378128	NM_019026_dup1	chr1	165738166	165744413	2150	+	chr1	165693528	165738135	4446	-	*		0.9975	1	0.05	0.5	0.12	16.36	17.33	16.44

cis_mRNA_overlap	0	AntiCompleteIn-mRNAIntron	n341831	NM_015987_dup1	chr12	13132771	13137572	1739	+	chr12	13127798	13153243	1209	-	*		0.9442	1	0.34	0.52	0.16	0.67	1.4	0.49
cis_mRNA_overlap	0	mRNA	n324655	NM_014351_dup1	chr22	44210408	44223288	545	-	chr22	44220387	44258378	2465	-	*		0.7982	1	2.65	1.98	2.42	34.92	31.1	31.34
cis_mRNA_up10k	266	NA	n406461	NM_012383_dup1	chr9	77676116	77703133	1450	-	chr9	77703398	77762114	1313	+	*		0.9996	1	0.91	1.07	1.89	7.51	7.88	10.23
cis_mRNA_up10k	179	NA	n365462	NM_015147_dup1	chr2	65273704	65283317	1912	-	chr2	65283495	65314142	5862	+	*		0.9765	1	0.15	0.39	0.11	4.82	6.15	4.11
cis_mRNA_dw20k	569	NA	n407775	NM_019070_dup1	chr19	19040010	19050444	1981	-	chr19	19030484	19039442	1836	+	*		0.9311	1	1.1	1.09	1.41	30.37	29.67	31.45
cis_mRNA_dw20k	14869	NA	n382914	NM_016639_dup1	chr16	3087252	3087800	549	-	chr16	3070313	3072384	1033	+	*		-0.881	-1	0.07	0.06	0.24	1.33	1.65	1.03
cis_mRNA_overlap	0	mRNA	LTCONS_00013489	NM_012139_dup1	chr11	17809595	18034313	4050	-	chr11	17809595	18034637	1494	-		-969.21	-0.8828	-1	1.35	1.61	1.54	16.57	15.13	16.14
cis_mRNA_up10k	7387	NA	n344576	NM_014049_dup1	chr3	128578560	128590947	2555	+	chr3	128598333	128631957	2575	+	*		-0.966	-1	0.2	0.13	0.04	5.99	6.16	6.84
cis_mRNA_dw20k	2702	NA	n406664	NM_015901_dup1	chr10	74894282	74927853	1826	-	chr10	74870210	74891581	2104	+	*		-0.9997	-1	0.17	0.19	0.07	0.44	0.4	0.68
cis_mRNA_overlap	0	AntiCompleteIn-mRNAIntron	n340985	NM_012139_dup1	chr11	17816331	17818197	1867	+	chr11	17809595	18034637	1494	-	*		-0.8276	-1	0.06	0.24	0.21	16.57	15.13	16.14
cis_mRNA_dw20k	16593	NA	LTCONS_00058944	NM_014613_dup1	chr5	175953667	175964942	5615	-	chr5	175875356	175937075	4515	+	*		0.9449	1	2.5	3.62	2.59	14.51	17.1	15.54
cis_mRNA_up10k	12	NA	LTCONS_00014948	NM_016938_dup1	chr11	65640416	65641079	664	.	chr11	65633912	65640405	2068	-	*		0.9381	1	0	0.1	0.15	0.25	0.7	1.51
cis_mRNA_overlap	0	mRNA	n410129	NM_178238_dup1	chr7	99933688	99965454	3007	+	chr7	99955626	99965454	1438	+	*		-0.9938	-1	4.54	2.99	3.71	1.39	1.99	1.77
cis_mRNA_overlap	0	mRNA	n341850	NM_015349_dup1	chr6	42714696	42836128	6511	+	chr6	42788794	42836296	6515	+	*		-0.8178	-1	2.48	1.88	1.75	0	0.4	1.47
cis_mRNA_dw20k	3125	NA	n408346	NM_007224_dup1	chr12	57623356	57628718	2215	+	chr12	57610578	57620232	1804	+	*		0.948	1	0.11	0.92	0.45	2.39	5.29	2.73
cis_mRNA_up10k	277	NA	n338923	NM_001102426_dup1	chr2	101768122	101771872	2475	+	chr2	101623690	101767846	4147	-	*		-0.9291	-1	0.3	0.44	0.25	1.59	1.01	2.45
tran	NA	NA	LTCONS_00055968	NM_012388_dup1	chr5	60452705	60453538	834	+	chr15	45879417	45902393	3959	+		-732.84	0.9999	1	2.23	1.83	1.61	18.22	17.27	16.77
tran	NA	NA	LTCONS_00055968	NM_014395_dup1	chr5	60452705	60453538	834	+	chr4	100737981	100791347	2938	+		-594.37	-0.9959	-1	2.23	1.83	1.61	0.12	0.2	0.23
cis_mRNA_up10k	59	NA	n335667	NM_014346_dup1	chr22	47157804	47158460	657	-	chr22	47158518	47571342	3793	+	*		-0.9981	-1	7.52	6.05	6.62	4.77	4.98	4.91
cis_mRNA_dw20k	5470	NA	n369453	NM_006703_dup1	chr6	34247383	34249098	1642	-	chr6	34254567	34360457	2410	-	*		0.8052	1	0.17	0.16	0.39	18.76	14.39	21.32
cis_mRNA_up10k	118	NA	n381340	NM_014053_dup1	chr1	213029947	213031480	941	-	chr1	213031597	213072705	5939	+	*		-0.9969	-1	0.97	0	1.65	16.71	20.97	12.76
cis_mRNA_overlap	0	AntiCompleteIn-mRNAIntron	n323972	NM_015635_dup1	chr9	128072221	128078018	2102	-	chr9	128024111	128127290	6889	+	*		0.8435	1	0.48	0.39	0.38	10.65	10.11	9.24
cis_mRNA_overlap	0	Lnc-AntiOverlap-mRNA	n379906	NM_017423_dup1	chr4	174087220	174090078	593	-	chr4	174089904	174245118	4307	+	*		0.9766	0.866	0.06	0.06	0	5.59	5.36	4.57
cis_mRNA_up10k	13	NA	n338103	NM_012243_dup1	chr1	100433823	100435528	1704	-	chr1	100435540	100489006	2149	+	*		0.8305	1	0.26	0.19	0.16	2.73	2.65	2.26
cis_mRNA_up10k	6562	NA	n382902	NM_006453_dup1	chr16	2014997	2015503	231	+	chr16	2022064	2028751	2594	+	*		-0.8329	-1	5.76	3.38	5.33	10.86	13.17	12.49
cis_mRNA_up10k	49	NA	n381826	NM_015509_dup1	chr12	8232081	8234759	2679	-	chr12	8234807	8250373	2592	+	*		-0.6022	-0.866	0.07	0	0.07	92.99	93.45	89.99
cis_mRNA_up10k	3762	NA	LTCONS_00063781	NM_018960_dup1	chr6	42923995	42924739	745	.	chr6	42928500	42931618	1068	+	*		-0.7247	-1	0.14	0.22	0.47	0.43	0.27	0.26
cis_mRNA_dw20k	16988	NA	LTCONS_00063781	NM_006586_dup1	chr6	42923995	42924739	745	.	chr6	42896860	42907008	1765	+	*		0.7934	1	0.14	0.22	0.47	19.22	19.91	20.05
cis_mRNA_up10k	89	NA	LTCONS_00072793	NM_015110_dup1	chr9	72830975	72873790	1499	-	chr9	72873878	72969789	5965	+	*		0.9809	1	0.53	0.55	0.87	6.92	7.28	8.33
cis_mRNA_dw20k	6043	NA	n367047	NM_014487_dup1	chr4	142161893	142199943	1037	-	chr4	142142049	142155851	1891	+	*		0.992	0.866	0.03	0.15	0.03	12.36	15.01	11.94
cis_mRNA_dw20k	3591	NA	n344996	NM_006571_dup1	chr8	30044745	30055524	4144	+	chr8	30013813	30041155	1126	+	*		0.9066	1	2.43	2.72	2.51	29.08	33.37	31.91
cis_mRNA_dw20k	4536	NA	n377856	NM_014296_dup1	chr3	15298958	15300460	564	+	chr3	15247733	15294423	4368	+	*		0.9959	1	0.27	0.65	0.91	11.09	12.12	13.09
cis_mRNA_up10k	90	NA	n382029	NM_015070_dup1	chr13	46626983	46675482	3508	+	chr13	46536314	46626894	6409	-	*		-0.9905	-1	0.52	0.8	0.38	0.42	0.36	0.47
cis_mRNA_up10k	1775	NA	n373433	NM_006825_dup1	chr12	106643487	106646559	722	+	chr12	106631659	106641713	3036	-	*		-0.9962	-1	0.75	0.86	1.11	27.87	27.23	26.2
cis_mRNA_dw20k	1056	NA	n340475	NM_016041_dup1	chr17	5374577	5376558	1982	-	chr17	5377613	5389497	1127	-	*		-0.6651	-1	12.19	12.24	11.56	17.03	15.94	17.22
cis_mRNA_overlap	0	mRNA	LTCONS_00074785	NM_017416_dup1	chrX	103810725	103905212	2645	+	chrX	103810996	105011822	2985	+		-1187.02	0.9987	1	0.18	0.73	0.1	0.95	3.94	0.3
cis_mRNA_overlap	0	Lnc-CompleteIn-mRNAIntron	n324359	NM_016485_dup1	chr6	142495920	142497981	913	+	chr6	142468410	142542085	3244	+	*		-0.6875	-0.866	0.16	0.14	0.14	18.01	20.65	18.65
cis_mRNA_dw20k	3216	NA	n342631	NM_007277_dup1	chr5	470626	473178	2553	-	chr5	443334	467411	2764	+	*		-0.9709	-1	1.98	2.17	1.89	10.18	9.97	10.42
cis_mRNA_up10k	3371	NA	n410994	NM_007059_dup1	chr19	47990891	48018515	1516	-	chr19	47978400	47987521	1688	-	*		-0.9272	-1	1.5	0.98	3.61	3.8	4.19	3.47
cis_mRNA_overlap	0	Lnc-CompleteIn-mRNAIntron	n384202	NM_182916_dup1	chr3	3171714	3172360	647	+	chr3	3168600	3190707	2276	+	*		0.8545	1	0.73	0.15	0.4	10.26	5.07	9.93
cis_mRNA_overlap	0	Lnc-CompleteIn-mRNAIntron	n385992	NM_015037_dup1	chr10	75547343	75547998	656	+	chr10	75545605	75561551	5835	+	*		0.9552	1	0.61	0.81	1.53	8.11	8.87	9.67
cis_mRNA_up10k	6067	NA	n410743	NM_016145_dup1	chr19	12786531	12792701	1248	-	chr19	12778881	12780465	861	-	*		0.8489	1	2.68	1.76	3.14	57.67	56.22	64.89
cis_mRNA_up10k	1934	NA	n368610	NM_014051_dup1	chr6	52529199	52533951	3554	+	chr6	52535884	52551386	1015	+	*		-0.879	-1	0.89	1.02	0.69	98.84	86.4	101.19
cis_mRNA_overlap	0	mRNA	n339112	NM_015092_dup1	chr16	18849783	18861683	2016	-	chr16	18816175	18937726	16065	-	*		0.9984	1	1.86	1.83	0.96	5.2	5.07	3.83
cis_mRNA_dw20k	11673	NA	n410180	NM_012453_dup1	chr7	72950683	72971602	1694	-	chr7	72983274	72993013	3041	-	*		0.7259	1	0.9	1.88	0.86	9.14	9.26	8.8
cis_mRNA_overlap	0	Lnc-CompleteIn-mRNAExon	n342089	NM_006581_dup1	chr6	96656045	96663486	7441	+	chr6	96463845	96663488	12798	+	*		-0.9998	-1	13.97	11.14	14.19	10.57	12.02	10.49
cis_mRNA_dw20k	19626	NA	LTCONS_00076614	NM_013364_dup1	chrX	152248452	152250131	1680	.	chrX	152224766	152228827	3751	+	*		0.9801	1	1.21	1.59	0.86	18.22	24.89	15.32
cis_mRNA_up10k	118	NA	LTCONS_00055395	NM_017426_dup1	chr4	77069772	77070058	287	.	chr4	77035817	77069655	2354	-	*		-0.9907	-1	0.14	0.39	0.89	11.98	11.01	9.84
cis_mRNA_overlap	0	Lnc-AntiOverlap-mRNA	n386699	NM_016546_dup1	chr12	7261167	7273623	2086	+	chr12	7247146	7261869	3398	-	*		0.8273	1	0.16	0.4	0.34	1.75	3.13	2.05
cis_mRNA_up10k	170	NA	n378602	NM_016470_dup1	chr20	42839600	42854667	1441	+	chr20	42825136	42839431	1572	-	*		0.9982	1	0.45	0.29	0.18	16.45	14.88	14.02
cis_mRNA_overlap	0	mRNA	n408019	NM_012263_dup1	chr22	43435523	43485434	1876	-	chr22	43435522	43485434	1773	-	*		0.9996	1	0	0.32	0.37	14.12	15.6	15.89
cis_mRNA_overlap	0	Lnc-CompleteIn-mRNAIntron	n381344	NM_012414_dup1	chr1	220439521	220441057	1537	-	chr1	220321610	220445843	7329	-	*		0.9008	1	1.55	1.38	1.17	19.17	17.82	17.59
cis_mRNA_up10k	42	NA	n342587	NM_014317_dup1	chr10	26984020	26986554	2535	-	chr10	26986595	27035727	1619	+	*		0.9825	1	0.24	0.18	0.1	3.12	2.7	2.41
cis_mRNA_dw20k	9716	NA	LTCONS_00011032	NM_019054_dup1	chr10	88960937	88961773	837	.	chr10	88854953	88951222	3404	+	*		-0.998	-1	0.09	0.04	0.19	4.61	5.1	3.32
cis_mRNA_up10k	27	NA	LTCONS_00047878	NM_014460_dup1	chr22	41947710	41956988	448	-	chr22	41957014	41972670	2530	+	*		0.8248	1	1.44	1.56	1.21	5.73	7.24	5.54
cis_mRNA_up10k	110	NA	LTCONS_00046742	NM_012399_dup1	chr22	28315364	28404569	4861	+	chr22	28247657	28315255	2925	-	*		0.9588	1	0.02	0.04	0	12.68	14.11	12.22
cis_mRNA_overlap	0	mRNA	n408220	NM_012459_dup1	chr11	111955539	111957522	1140	-	chr11	111955524	111957522	822	-	*		-0.7918	-1	7.16	9.24	8.64	48.87	45.85	48.52
cis_mRNA_up10k	931	NA	n369257	NM_006581_dup1	chr6	96461406	96462915	333	-	chr6	96463845	96663488	12798	+	*		-0.9867	-1	0.36	0.11	0.43	10.57	12.02	10.49
cis_mRNA_overlap	0	Lnc-CompleteIn-mRNAIntron	n324919	NM_198935_dup1	chr20	60735569	60737659	461	+	chr20	60718822	60757566	4548	+	*		0.7844	0.866	0.82	0.82	0.58	20.71	21.97	19.96
cis_mRNA_up10k	1697	NA	n376177	NM_171999_dup1	chr18	76736573	76738579	1352	-	chr18	76740275	76758194	4775	+	*		-0.9799	-1	0.4	0.23	0.42	0.45	0.87	0.27
cis_mRNA_overlap	0	Lnc-CompleteIn-mRNAIntron	n341072	NM_015516_dup1	chr11	76498677	76500442	1766	+	chr11	76494285	76509198	2710	+	*		0.7107	0.866	0.29	0.37	0.37	2.27	2.42	2.82

cis_mRNA_up10k	3916	NA	LTCONS_00069947	NM_014018_dup1	chr8	80946421	81086273	7122	-	chr8	80831095	80942506	707	-	*	-0.9962	-1	1.85	2.51	2.71	16.52	15.49	15.02
cis_mRNA_overlap	0	Lnc-AntiOverlap-mRNA	LTCONS_00015661	NM_006082_dup1	chr12	49521569	49579494	874	+	chr12	49521565	49525304	1753	-	-1833.46	-0.9292	-1	0.61	0.67	0.51	525.26	415.17	570.76
cis_mRNA_up10k	1076	NA	n377450	NM_014941_dup1	chr22	31365262	31375327	5016	+	chr22	31322600	31364187	4467	-	*	-0.9999	-1	4.16	5.44	3.51	4.86	4.34	5.11
cis_mRNA_dw20k	5155	NA	n409080	NM_006634_dup1	chr2	85825670	85829822	1486	-	chr2	85811531	85820516	670	+	*	-0.9285	-1	0.99	0.2	1.92	0.11	0.15	0.1
cis_mRNA_dw20k	7399	NA	n345975	NM_006355_dup1	chr6	25992929	25999173	1261	+	chr6	25963071	25985531	3286	+	*	0.9599	0.866	2.1	0.67	0.09	0.05	0.04	0.04
cis_mRNA_up10k	53	NA	n374977	NM_005744_dup1	chr15	72757151	72766615	709	-	chr15	72766667	72878896	5251	+	*	-0.9953	-1	0.1	0.27	0	24.75	23.24	26.01
cis_mRNA_dw20k	13676	NA	n371707	NM_014394_dup1	chr10	85926986	85931832	1855	-	chr10	85899185	85913311	2449	+	*	-0.9643	-1	0.21	0.3	0.27	69.4	65.43	65.72
cis_mRNA_dw20k	3162	NA	n365392	NM_198182_dup1	chr2	10145573	10149991	951	-	chr2	10091792	10142412	3605	+	*	0.9922	1	0.04	0.03	0	2.22	2.18	2.11
cis_mRNA_overlap	0	mRNA	n409377	NM_006476_dup1	chr11	118272104	118280562	1391	+	chr11	118272104	118280995	1343	+	*	0.9042	1	0.27	0.1	0.17	80.87	75.71	79.87
cis_mRNA_up10k	4153	NA	n345941	NM_006547_dup1	chr7	23514147	23515208	983	+	chr7	23349828	23509995	4159	-	*	-0.9961	-1	0.22	0.1	0.26	12.59	14.33	11.73
cis_mRNA_overlap	0	Lnc-AntiOverlap-mRNA	n338222	NM_021004_dup1	chr14	24408822	24423375	1845	-	chr14	24422965	24438488	1278	+	*	-0.9412	-1	1.79	0.6	0.92	2.12	3.22	2.59
cis_mRNA_dw20k	1830	NA	n341295	NM_181581_dup1	chr7	107220797	107262285	1209	+	chr7	107204432	107218968	2250	+	*	0.9571	1	2.64	2.16	2.73	2.51	2.11	2.81
cis_mRNA_up10k	5525	NA	n379061	NM_015190_dup1	chr10	75012549	75014107	762	+	chr10	75002582	75007025	1437	-	*	0.9993	1	1.36	1.02	1.9	33.49	31.17	36.72
cis_mRNA_overlap	0	Lnc-AntiOverlap-mRNA	n338522	NM_032635_dup1	chr19	36031640	36036931	1627	-	chr19	36036545	36038428	851	+	*	0.6765	1	0.74	0.73	0.88	34.48	33.31	34.72
cis_mRNA_overlap	0	mRNA	n405970	NM_014937_dup1	chr10	121578786	121588659	3146	+	chr10	121485609	121588659	4932	+	*	0.722	1	171.2	61.78	85.15	19.84	18.58	19.73
cis_mRNA_up10k	87	NA	LTCONS_00018585	NM_019034_dup1	chr12	122231680	122241988	4133	-	chr12	122215660	122231594	2435	-	*	-0.6823	-1	0.59	0.34	0.37	1.43	1.98	1.5
cis_mRNA_dw20k	13843	NA	LTCONS_00046508	NM_022044_dup1	chr22	22012430	22016874	4445	+	chr22	21996542	21998588	874	+	*	-0.9489	-1	1.15	1.83	1.58	4.76	4.41	4.43
cis_mRNA_overlap	0	Lnc-CompleteIn-mRNAIntron	n342317	NM_152280_dup1	chr1	155839565	155841887	2321	+	chr1	155829260	155854990	5280	+	*	0.9989	1	1.12	2.18	2.08	105.65	149.85	143.49
cis_mRNA_overlap	0	mRNA	n336938	NM_015033_dup1	chr9	132658075	132658914	840	-	chr9	132649466	132805473	5425	-	*	-0.9234	-1	0.19	0.13	0.04	1.65	2.57	2.88
cis_mRNA_up10k	315	NA	LTCONS_00025570	NM_015307_dup1	chr15	29863241	29867504	4264	.	chr15	29412455	29862927	4707	-	*	0.6386	1	0.27	0.17	0.78	14.05	6.93	14.18
cis_mRNA_up10k	101	NA	n385425	NM_016023_dup1	chr8	92080139	92082324	2078	-	chr8	92082424	92099323	3298	+	*	-0.8455	-1	19.09	18.06	20.72	8.63	9.36	8.55
cis_mRNA_up10k	84	NA	LTCONS_00008473	NM_016065_dup1	chr10	75012534	75014196	1568	+	chr10	75008601	75012451	2634	-	*	-0.7379	-1	1.12	1.08	0.54	32.87	33.85	34.18
cis_mRNA_dw20k	2908	NA	n341946	NM_007235_dup1	chr12	64845371	64846681	1311	-	chr12	64798153	64842464	3948	+	*	0.9599	1	0.03	0.07	0.05	25.81	31.92	27.32
cis_mRNA_dw20k	2650	NA	n371273	NM_016481_dup1	chr9	100660035	100664123	2128	-	chr9	100666772	100684852	1646	-	*	0.9672	1	0.87	1.01	0.57	5.4	5.41	5.26
cis_mRNA_up10k	3329	NA	n376545	NM_006809_dup1	chr20	43592442	43595043	2058	-	chr20	43570771	43589114	2049	-	*	-0.9713	-1	0.08	0.05	0.01	4.78	5.18	6.68
cis_mRNA_up10k	7533	NA	LTCONS_00027255	NM_016333_dup1	chr16	2787077	2794798	1298	-	chr16	2802330	2821413	9353	+	*	-0.8606	-1	0.23	0.2	0.21	7.73	11.58	8.46
cis_mRNA_dw20k	8830	NA	n383328	NM_013305_dup1	chr18	44247785	44250252	2468	-	chr18	44259081	44337039	2623	-	*	0.9078	1	0.46	0.55	0.45	0.18	0.21	0.15
cis_mRNA_dw20k	11430	NA	n410108	NM_006391_dup1	chr11	9481103	9482245	1143	-	chr11	9406169	9469674	6192	+	*	0.9701	1	2.76	3.1	2.9	31.46	34.4	32.01
cis_mRNA_dw20k	2323	NA	n379194	NM_016113_dup1	chr17	16342639	16345280	889	+	chr17	16318856	16340317	2808	+	*	-0.7943	-1	4.66	2.18	1.93	1.4	2.29	4.24
cis_mRNA_dw20k	17285	NA	n378187	NM_015094_dup1	chr22	21823036	21824224	788	+	chr22	21771693	21805752	6798	+	*	0.9793	1	0.83	1.27	1.11	9.58	11.42	11.1
cis_mRNA_dw20k	4281	NA	n341753	NM_014334_dup1	chr9	111892575	111895301	2727	-	chr9	111899581	111929571	1189	-	*	0.9966	1	17.26	16.61	15.58	16.98	16.14	15.15
cis_mRNA_up10k	7239	NA	n338803	NM_014475_dup1	chr19	49429428	49429701	274	+	chr19	49436939	49448226	1081	+	*	0.9977	1	0.15	1.5	0.67	0.2	0.47	0.32
cis_mRNA_overlap	0	Lnc-CompleteIn-mRNAIntron	n340372	NM_012073_dup1	chr5	10252773	10253000	227	+	chr5	10250282	10266501	3403	+	*	-0.9029	-1	0	0.35	0.17	124.5	109.92	123.48
cis_mRNA_up10k	255	NA	n342003	NM_001031701_dup1	chr12	104235229	104238442	674	+	chr12	104166081	104234975	7214	-	*	0.9219	1	0.05	0.1	0.14	2.2	2.47	2.49
cis_mRNA_overlap	0	Lnc-AntiOverlap-mRNA	n384245	NM_015460_dup1	chr3	40214644	40351182	2099	-	chr3	39851303	40301812	4870	+	*	0.8793	1	0	0.07	0.09	2.28	3.18	4.95
cis_mRNA_up10k	236	NA	n341082	NM_014488_dup1	chr11	82783119	82784646	554	+	chr11	82692478	82782884	1602	-	*	-0.9947	-1	4.33	0.56	0	1.91	3.34	3.77
cis_mRNA_dw20k	15111	NA	n365077	NM_006826_dup1	chr2	9705818	9708996	525	+	chr2	9724106	9771106	2166	-	*	-0.7484	-0.866	0	0.06	0.06	347.5	310.64	335.59
cis_mRNA_dw20k	7187	NA	n340069	NM_015190_dup1	chr10	74994301	74995396	1095	-	chr10	75002582	75007025	1437	-	*	0.9803	1	0.77	0.64	0.86	33.49	31.17	36.72
cis_mRNA_dw20k	5186	NA	LTCONS_00012874	NM_014352_dup1	chr11	120195838	120205573	7400	+	chr11	120110951	120190653	2965	+	*	-0.9286	-0.866	0.66	0.78	0.98	0.01	0.01	0
cis_mRNA_up10k	759	NA	n380891	NM_013381_dup1	chr12	72649461	72665771	403	-	chr12	72666529	73059422	5552	+	*	0.9948	1	0.04	0.2	0.09	0.38	0.84	0.48
cis_mRNA_up10k	7612	NA	LTCONS_00063797	NM_016021_dup1	chr6	90070230	90071631	1402	.	chr6	90036344	90062619	4342	-	*	-0.983	-1	0	0.05	0.07	19.83	19.72	19.63
cis_mRNA_dw20k	2422	NA	n332617	NM_013356_dup1	chr22	38471400	38471723	324	+	chr22	38474144	38479170	1896	-	*	-0.7848	-1	0.21	2.67	0	0.24	0.15	0.44
cis_mRNA_dw20k	7127	NA	n340118	NM_014472_dup1	chr10	100011780	100016332	1168	+	chr10	99894381	100004654	3371	+	*	0.9721	1	0.12	0.11	0.19	9.14	8	11.26
cis_mRNA_up10k	1095	NA	n372755	NM_015036_dup1	chr11	94820008	94821880	942	-	chr11	94822974	94865815	4693	+	*	0.9962	1	0.15	0.4	0.5	9.6	14.93	16.29
cis_mRNA_dw20k	15255	NA	LTCONS_00025332	NM_014300_dup1	chr15	85181743	85197521	9047	-	chr15	85212775	85259674	1383	-	*	-0.9184	-1	1.75	3.17	3.07	59.24	50.97	54.72
cis_mRNA_dw20k	5052	NA	n375510	NM_012121_dup1	chr17	71272639	71274712	1164	+	chr17	71279763	71308143	3101	-	*	0.8384	1	0.06	0.11	0.05	4.38	4.69	3.71
cis_mRNA_dw20k	194	NA	LTCONS_00027272	NM_016639_dup1	chr16	3072577	3074415	1605	-	chr16	3070313	3072384	1033	+	*	-0.8993	-1	0.33	0.17	0.34	1.33	1.65	1.03
cis_mRNA_up10k	9156	NA	n371891	NM_015901_dup1	chr10	74857802	74861055	3005	-	chr10	74870210	74891581	2104	+	*	-0.9245	-1	0.58	0.83	0.33	0.44	0.4	0.68
cis_mRNA_overlap	0	Lnc-AntiOverlap-mRNA	n338342	NM_014371_dup1	chr19	15489887	15491657	1771	+	chr19	15490859	15529833	2114	-	*	-0.9583	-1	0.02	0.05	0.12	31.17	29.19	27.6
cis_mRNA_overlap	0	mRNA	n325254	NM_016297_dup1	chr2	70483570	70503314	577	+	chr2	70485231	70508317	5377	+	*	-0.8153	-1	0.07	0.28	0.05	22.17	21.58	23.27
cis_mRNA_overlap	0	Lnc-CompleteIn-mRNAExon	n338059	NM_006078_dup1	chr22	36956917	36958962	2046	-	chr22	36956916	37098690	4523	-	*	0.9554	1	0.94	1.58	1.17	2.08	3.03	2.67
cis_mRNA_overlap	0	CompleteIn-LncExon	n407029	NM_016062_dup1	chr16	66965958	66968320	849	-	chr16	66965958	66968320	693	-	*	-0.8401	-1	1.6	1.29	1.03	50.64	50.65	60.09
cis_mRNA_overlap	0	Lnc-CompleteIn-mRNAIntron	n382920	NM_014015_dup1	chr16	11029866	11031993	2128	-	chr16	11022748	11036257	1501	-	*	0.6251	1	1.58	1.54	2.13	13.9	13.72	13.92
cis_mRNA_overlap	0	Lnc-CompleteIn-mRNAIntron	n340536	NM_012446_dup1	chr5	80813191	80815724	2534	-	chr5	80715672	81047072	1948	-	*	0.998	1	0.03	0.06	0.17	17.79	20.6	28.05
cis_mRNA_dw20k	19370	NA	n341682	NM_015297_dup1	chr9	35001910	35003660	1751	+	chr9	34958192	34982541	5847	+	*	0.8116	1	0.44	0.5	0.25	7.07	9.77	6.39
cis_mRNA_dw20k	2253	NA	n339864	NM_013305_dup1	chr18	44247785	44256829	2042	-	chr18	44259081	44337039	2623	-	*	-0.866	-0.866	0.02	0	0.02	0.18	0.21	0.15
cis_mRNA_dw20k	1384	NA	n341759	NM_015258_dup1	chr9	115913265	115926417	1740	+	chr9	115927800	115983641	4336	-	*	0.996	1	1.18	2.03	2.35	4.51	5.42	5.92
cis_mRNA_overlap	0	Lnc-CompleteIn-mRNAExon	n339452	NM_007038_dup1	chr21	28290242	28292870	2629	-	chr21	28290231	28339439	9663	-	*	0.9018	1	0.07	0.13	0.29	0.2	0.28	0.32
cis_mRNA_overlap	0	Lnc-AntiOverlap-mRNA	n338180	NM_006594_dup1	chr1	114399257	114443859	1733	+	chr1	114437677	114447741	2484	-	*	0.7538	1	0.63	0.9	0.95	11.01	11.36	12.81
cis_mRNA_up10k	1448	NA	n343050	NM_014941_dup1	chr22	31365634	31375381	7105	+	chr22	31322600	31364187	4467	-	*	-0.8942	-1	7.57	7.87	6.74	4.86	4.34	5.11

cis_mRNA_up10k	157	NA	n339815	NM_016930_dup1	chr4	4543931	4712665	2404	+	chr4	4420695	4543775	2158	-	*		0.9264	1	0.08	0.24	0.21	11.55	12.71	12.09
cis_mRNA_dw20k	8137	NA	n377412	NM_006440_dup1	chr22	19847802	19854904	610	+	chr22	19863040	19929359	1964	-	*		-0.9226	-0.866	0.06	0.05	0.05	6.01	6.19	6.12
cis_mRNA_up10k	5341	NA	n377412	NM_053004_dup1	chr22	19847802	19854904	610	+	chr22	19775934	19842462	1519	-	*		0.9725	0.866	0.06	0.05	0.05	2.61	1.8	1.54
cis_mRNA_dw20k	1535	NA	n337863	NM_012199_dup1	chr1	36391433	36395210	3778	+	chr1	36348810	36389899	7478	+	*		0.9886	1	4.65	5.01	3.96	7.44	8.22	6.58
cis_mRNA_up10k	2676	NA	n346481	NM_015565_dup1	chr21	30367952	30370413	1114	-	chr21	30300466	30365277	7756	-	*		-0.9934	-1	0.46	0	0.47	13.3	15.38	12.95
cis_mRNA_dw20k	11788	NA	n407230	NM_014367_dup1	chr3	122140748	122233786	7012	-	chr3	122103023	122128961	838	+	*		0.6876	0.866	0.43	0.52	0.52	10.99	11.56	13.34
cis_mRNA_up10k	151	NA	n374529	NM_007347_dup1	chr15	51132073	51200796	2398	-	chr15	51200946	51298097	6682	+	*		0.9537	1	0	0.16	0.05	4.24	4.74	4.53
cis_mRNA_up10k	4462	NA	n410585	NM_012067_dup1	chr1	19619741	19622230	2490	-	chr1	19609057	19615280	1241	-	*		0.8623	1	1.25	1.64	1.41	0.27	0.46	0.44
cis_mRNA_up10k	65	NA	LTCONS_00017018	NM_016497_dup1	chr12	6602535	6603358	824	-	chr12	6601316	6602471	677	-	*		0.9784	1	1.52	0.87	2.05	55.16	52.03	60.5
cis_mRNA_up10k	6539	NA	n341452	NM_016353_dup1	chr8	16988690	17007298	2290	+	chr8	17013836	17080241	4012	+	*		-0.9625	-1	0.2	0.11	0.08	13.95	17.26	20.5
cis_mRNA_up10k	8355	NA	n409319	NM_053006_dup1	chr22	19023795	19109967	4369	-	chr22	19118321	19120136	1816	+	*		0.9449	0.866	0	0.58	0.87	0.04	0.05	0.05
cis_mRNA_up10k	6626	NA	n326172	NM_019090_dup1	chr1	232927771	232934013	3574	+	chr1	232940638	232946092	5455	+	*		-0.9903	-1	0.01	0.03	0.02	1.22	0.85	1.08
cis_mRNA_overlap	0	Lnc-AntiOverlap-mRNA	LTCONS_00029039	NM_015986_dup1	chr17	29035714	29119437	3516	+	chr17	29109702	29151778	2941	-	*		-0.9608	-1	2.01	1.93	1.43	2.53	2.71	2.98
cis_mRNA_up10k	568	NA	n369064	NM_012434_dup1	chr6	74364304	74365877	313	+	chr6	74303102	74363737	3314	-	*		0.8399	1	0.13	0	0.11	8.38	7.29	7.65
tran	NA	NA	LTCONS_00030629	NM_014501_dup1	chr17	18578482	18585572	6734	-	chr19	55912650	55919325	1191	-		-1833.78	-0.9482	-1	2.17	2.86	2.38	78.46	71.74	74.51
cis_mRNA_overlap	0	Lnc-CompleteIn-mRNAIntron	n324647	NM_014346_dup1	chr22	47223366	47225693	568	+	chr22	47158518	47571342	3793	+	*		0.9599	1	0	0.18	0.17	4.77	4.98	4.91
cis_mRNA_dw20k	3110	NA	n410072	NM_006651_dup1	chr4	773937	775636	1700	-	chr4	778745	819945	2140	-	*		0.999	1	9.19	11.85	8.51	7.89	9.27	7.63
cis_mRNA_overlap	0	Lnc-AntiOverlap-mRNA	n339734	NM_198976_dup1	chr20	57560742	57562496	1755	-	chr20	57556311	57570188	2257	+	*		-0.8269	-1	0.02	0.05	0	14.87	14.7	18.34
cis_mRNA_up10k	604	NA	n384929	NM_015695_dup1	chr6	36125846	36163947	1098	-	chr6	36164550	36200567	6023	+	*		-0.9712	-0.866	0.17	0.11	0.17	4.88	6.21	5.21
cis_mRNA_overlap	0	Lnc-CompleteIn-mRNAIntron	n334776	NM_021149_dup1	chr16	84649611	84650115	505	-	chr16	84599202	84651669	1827	-	*		0.9657	1	1.55	1.14	6.2	48.89	41.38	63.77
cis_mRNA_up10k	243	NA	n380500	NM_006317_dup1	chr5	17169365	17217508	2381	-	chr5	17217750	17276943	1797	+	*		0.9028	1	1.55	1.46	0.8	245.27	225.39	207.54
cis_mRNA_overlap	0	Lnc-CompleteIn-mRNAIntron	n380532	NM_015347_dup1	chr12	130904080	130905564	415	-	chr12	130880681	131002410	6322	-	*		0.7929	1	0.18	0	0.58	0.98	0	1.08
cis_mRNA_up10k	126	NA	LTCONS_00045056	NM_015894_dup1	chr20	62284905	62287583	2679	.	chr20	62271061	62284780	2255	-	*		0.9963	1	0.94	0.93	1.07	190.09	181.92	232.64
cis_mRNA_dw20k	164	NA	LTCONS_00034771	NM_016148_dup1	chr19	51161705	51164921	2741	+	chr19	51165084	51220195	6643	-	*		0.9028	0.866	0.04	0.1	0.1	0.66	0.95	1.17
cis_mRNA_up10k	6969	NA	n345307	NM_015042_dup1	chr15	64762013	64784651	3422	-	chr15	64791619	64978267	8618	+	*		0.9832	1	0.04	0.06	0.07	6.45	7.76	8.01
cis_mRNA_up10k	2390	NA	n338259	NM_020637_dup1	chr19	637105	637537	433	-	chr19	639926	643604	513	+	*		-0.6857	-0.866	0.09	0.08	0.08	0.07	0.13	0.32
cis_mRNA_up10k	3371	NA	n410995	NM_007059_dup1	chr19	47990891	48018515	1663	-	chr19	47978400	47987521	1688	-	*		-0.9718	-1	0.77	0.55	1.21	3.8	4.19	3.47
cis_mRNA_overlap	0	Lnc-AntiOverlap-mRNA	n324355	NM_016108_dup1	chr6	143329254	143400412	1900	-	chr6	143382023	143661441	1385	+	*		0.9742	1	0.09	0.07	0.05	17.22	16.22	13.88
cis_mRNA_overlap	0	Lnc-AntiOverlap-mRNA	LTCONS_00009330	NM_015722_dup1	chr10	135138303	135139037	378	+	chr10	135138928	135150475	983	-	*		-0.979	-1	2.97	2.52	2.11	3.6	6.22	11.22
cis_mRNA_overlap	0	Lnc-CompleteIn-mRNAExon	n335717	NM_016337_dup1	chr14	100610216	100610573	358	+	chr14	100531751	100610573	1842	+	*		-0.9681	-1	5.26	3.8	8.29	29.75	29.91	27.89
cis_mRNA_up10k	4445	NA	n339076	NM_015226_dup1	chr16	11032736	11033901	1164	-	chr16	11038345	11276046	6891	+	*		-0.79	-1	2.44	2.5	3.11	2.3	1.18	0.68
cis_mRNA_dw20k	2827	NA	n383718	NM_012455_dup1	chr2	113963503	113966973	3471	+	chr2	113931560	113960677	5039	+	*		0.8417	1	0.35	0.49	0.56	0.13	0.15	0.26
cis_mRNA_dw20k	5332	NA	n379303	NM_015055_dup1	chr11	9779839	9832866	2709	+	chr11	9685628	9774508	4848	+	*		0.9948	1	0.9	0.83	0.62	2.61	2.48	1.75
cis_mRNA_dw20k	66	NA	n384190	NM_016426_dup1	chr22	46726772	46730183	1150	+	chr22	46692638	46726707	3112	+	*		0.9575	0.866	0.52	0.33	0.6	0.05	0.03	0.05
cis_mRNA_dw20k	951	NA	n333501	NM_012396_dup1	chr1	201433396	201433672	277	-	chr1	201434622	201438299	1516	-	*		-0.9999	-0.866	0	0.13	0.13	6.26	5.12	5.14
cis_mRNA_overlap	0	mRNA	n410210	NM_001025930_dup1	chr3	9851644	9878040	4160	+	chr3	9851644	9878040	3662	+	*		0.9956	0.866	0.13	0.13	0.14	0.16	0.19	0.45
cis_mRNA_up10k	594	NA	LTCONS_00042936	NM_001009984_dup1	chr20	3388848	3390736	1889	+	chr20	3229948	3388255	6855	-	*		-0.9779	-1	1	1.37	1.02	21.67	20.35	21.32
cis_mRNA_dw20k	11690	NA	LTCONS_00032032	NM_012329_dup1	chr17	53457973	53458285	313	.	chr17	53469974	53499341	2723	-	*		0.9517	1	0.77	0.7	1.03	75.78	72.19	79.48
cis_mRNA_up10k	480	NA	LTCONS_00012826	NM_016146_dup1	chr11	118877069	118888762	9015	+	chr11	118889241	118894384	1200	+	*		0.7746	1	3.04	3.59	3.76	53.96	55.91	67.4
cis_mRNA_dw20k	7509	NA	LTCONS_00005696	NM_006594_dup1	chr1	114419050	114430169	5496	-	chr1	114437677	114447741	2484	-	*		-0.8137	-1	0.08	0.02	0	11.01	11.36	12.81
cis_mRNA_up10k	107	NA	n372056	NM_012083_dup1	chr10	99094564	99102955	557	+	chr10	99092254	99094458	2205	-	*		-0.9813	-0.866	0	0.06	0.06	6.43	5.35	5.57
cis_mRNA_up10k	119	NA	LTCONS_00010961	NM_006426_dup1	chr10	133996726	134000296	2053	-	chr10	134000414	134019280	2719	+	*		-0.9845	-1	2.27	2.3	2.13	68.91	66.18	74.18
cis_mRNA_dw20k	18413	NA	n409415	NM_013270_dup1	chr3	46710485	46735194	5224	-	chr3	46753606	46759373	1347	-	*		0.965	1	0	0.24	0.15	0	0.16	0.14
cis_mRNA_overlap	0	Lnc-CompleteIn-mRNAIntron	n341075	NM_024684_dup1	chr11	77576824	77578177	1354	+	chr11	77532208	77583398	504	+	*		0.803	0.866	0.2	0.18	0.23	1.92	1.62	1.92
cis_mRNA_up10k	381	NA	n339342	NM_014016_dup1	chr3	45719657	45730374	2300	-	chr3	45730754	45786900	3707	+	*		0.9441	0.866	0.04	0.12	0.04	11.65	12.62	11.16
cis_mRNA_overlap	0	Lnc-CompleteIn-mRNAExon	n409355	NM_006089_dup1	chrX	18257433	18372844	3139	-	chrX	18257433	18372844	4198	-	*		-1	-1	0.53	0.9	1.3	1.4	0.82	0.21
cis_mRNA_dw20k	4787	NA	n384332	NM_144717_dup1	chr3	136734713	136735851	1139	+	chr3	136676707	136729927	2047	+	*		0.7995	1	0.19	0.35	0.12	0.39	0.4	0.31
cis_mRNA_dw20k	12777	NA	LTCONS_00004217	NM_018994_dup1	chr1	16555130	16563783	4611	-	chr1	16576559	16678948	2976	-	*		-0.6514	-0.866	4.61	4.04	6.95	11.19	12.18	11.19
cis_mRNA_dw20k	2178	NA	LTCONS_00051824	NM_014373_dup1	chr3	169805368	169817286	10407	-	chr3	169755735	169803191	2019	+	*		0.6934	0.866	0.12	0.17	0.17	0.1	0.14	0.11
cis_mRNA_dw20k	426	NA	n340251	NM_007080_dup1	chr4	147111638	147114339	2702	+	chr4	147096835	147111213	787	+	*		-0.999	-1	0.28	0.56	0.33	13.34	10.49	12.96
cis_mRNA_dw20k	5127	NA	n342776	NM_015885_dup1	chr11	82901962	82904242	2281	-	chr11	82868137	82896836	5913	+	*		-0.978	-1	1.1	1.37	1.39	12.48	11.49	11.11
cis_mRNA_overlap	0	Lnc-AntiOverlap-mRNA	n323963	NM_015679_dup1	chr9	131084558	131087828	583	+	chr9	131071396	131084697	1439	-	*		0.9862	1	0.06	0.23	0.28	20.97	22.82	23.95
cis_mRNA_dw20k	2359	NA	n370618	NM_015254_dup1	chr8	28915363	28922437	6644	+	chr8	28924795	29120610	8766	-	*		0.9943	1	2.78	3.45	3.47	0.51	1.71	1.91
cis_mRNA_dw20k	10795	NA	n345633	NM_006345_dup1	chr4	42100345	42101382	1038	+	chr4	41992523	42089551	3261	+	*		0.9403	1	2.41	2.19	1.87	60.87	60.67	58.29
cis_mRNA_dw20k	2403	NA	LTCONS_00023124	NM_013231_dup1	chr14	86096672	86097268	597	.	chr14	85996488	86094270	7179	+	*		0.98	1	0.55	1.4	0.22	2.11	4.79	1.87
cis_mRNA_up10k	231	NA	n341630	NM_016647_dup1	chr8	143783670	143808391	5684	-	chr8	143808621	143818350	2264	+	*		0.9437	1	0.16	0.03	0.1	11.75	1.86	10.24
cis_mRNA_dw20k	1955	NA	n408255	NM_016246_dup1	chr19	49298319	49314320	2080	-	chr19	49316274	49339934	1237	-	*		0.9171	1	3.22	5.08	4.2	0.55	1.71	0.73
cis_mRNA_up10k	271	NA	n374300	NM_015549_dup1	chr14	65170511	65170923	286	-	chr14	65171193	65211060	4400	+	*		-0.7806	-0.866	0	0.13	0.13	5.14	2.72	4.25
cis_mRNA_dw20k	2780	NA	n332918	NM_013441_dup1	chr1	24865207	24865735	529	+	chr1	24829387	24862428	1700	+	*		0.9209	1	22.17	21.97	25.37	3.58	2.33	5.18
cis_mRNA_dw20k	18452	NA	LTCONS_00048139	NM_016272_dup1	chr22	41809538	41811041	1504	.	chr22	41829492	41843027	4565	-	*		0.9927	1	0.41	0.9	0.6	4.23	4.96	4.59
cis_mRNA_dw20k	964	NA	n342503	NM_015106_dup1	chr3	51698597	51699166	570	+	chr3	51575596	51697634	4727	+	*		0.9992	1	2.1	3.6	0.65	5.06	5.61	4.45
cis_mRNA_up10k	143	NA	n339879	NM_006111_dup1	chr18	47340393	47377429	1615	+	chr18	47309874	47340251	1934	-	*		0.9905	1	0.04	0.02	0	15.35	14.11	13.35

cis_mRNA_up10k	64	NA	n409646	NM_001080415_dup1	chr3	142719687	142720309	623	-	chr3	142720372	142779567	7464	+	*	-0.8833	-1	0.53	0.5	0.97	13.56	14.54	12.65
cis_mRNA_dw20k	13511	NA	LTCONS_00074394	NM_018969_dup1	chrX	53123307	53145163	524	+	chrX	53078506	53109797	4252	+	*	-0.8769	-1	0	0.33	0.16	17.37	15.53	17.36
cis_mRNA_dw20k	6124	NA	n339573	NM_016201_dup1	chr3	134066130	134068067	1938	-	chr3	134074190	134093406	4984	-	*	0.9081	1	0.23	0.32	0.11	19.22	19.23	18.28
cis_mRNA_overlap	0	mRNA	LTCONS_00018788	NM_015347_dup1	chr12	130944708	131200833	9488	-	chr12	130880681	131002410	6322	-	*	0.8945	1	1.65	0.02	3.42	0.98	0	1.08
cis_mRNA_dw20k	12951	NA	LTCONS_00040515	NM_007046_dup1	chr2	27322221	27333970	1180	-	chr2	27301435	27309271	3938	+	*	-0.8432	-1	1.05	1.92	1.72	0.83	0.49	0.74
cis_mRNA_overlap	0	Lnc-CompleteIn-mRNAIntron	n340967	NM_015055_dup1	chr11	9766574	9768012	1439	+	chr11	9685628	9774508	4848	+	*	0.9512	1	0.31	0.2	0.06	2.61	2.48	1.75
cis_mRNA_overlap	0	Lnc-CompleteIn-mRNAExon	n342048	NM_006836_dup1	chr12	120565074	120632513	8608	-	chr12	120565014	120632513	8668	-	*	-0.9494	-1	10.61	12.25	11.41	1.67	0.97	1.53
cis_mRNA_dw20k	9398	NA	LTCONS_00062945	NM_006813_dup1	chr6	89804276	89827800	5263	-	chr6	89790429	89794879	2133	+	*	0.9911	1	0.91	0.65	0.79	8.76	6.3	7.34
cis_mRNA_up10k	153	NA	LTCONS_00052265	NM_019069_dup1	chr3	122135034	122136011	978	.	chr3	122130700	122134882	4183	-	*	-0.8322	-0.866	1.01	1.28	1.16	2.71	2.44	2.71
cis_mRNA_dw20k	16221	NA	n411612	NM_015687_dup1	chr6	75994730	76001580	1297	+	chr6	76017800	76203496	4622	-	*	0.8589	1	0.19	0.22	0.1	0.03	0.11	0
cis_mRNA_overlap	0	CompleteIn-LncExon	n410474	NM_014321_dup1	chr16	46723558	46732306	1687	+	chr16	46723558	46732306	1628	+	*	-0.9603	-1	0.27	0.67	0	3.91	3.17	4.03
cis_mRNA_overlap	0	Lnc-AntiOverlap-mRNA	n339210	NM_001080437_dup1	chr2	242014096	242034916	2330	-	chr2	241938255	242033643	6834	+	*	0.9983	1	0.41	1.01	0.74	0.02	0.04	0.03
cis_mRNA_dw20k	4540	NA	n364246	NM_012396_dup1	chr1	201424770	201430083	1929	-	chr1	201434622	201438299	1516	-	*	-0.7659	-1	0.01	0.07	0.03	6.26	5.12	5.14
cis_mRNA_dw20k	5496	NA	n338478	NM_015965_dup1	chr19	19644508	19647142	2635	-	chr19	19627019	19639013	542	+	*	-0.6538	-1	0	0.07	0.06	426.21	363.37	422.23
cis_mRNA_dw20k	2046	NA	LTCONS_00032669	NM_006701_dup1	chr18	77724582	77730822	2160	+	chr18	77732867	77748532	1387	-	*	-0.9994	-1	0.86	1.3	0.68	30.01	26.84	31.12
cis_mRNA_dw20k	2319	NA	n379213	NM_016113_dup1	chr17	16342635	16345301	1016	+	chr17	16318856	16340317	2808	+	*	-0.9598	-1	15.39	13.01	11.16	1.4	2.29	4.24
cis_mRNA_overlap	0	AntiCompleteIn-mRNAIntron	n326650	NM_012316_dup1	chr1	32587382	32588851	687	-	chr1	32573644	32642169	7373	+	*	-0.9347	-1	1.42	0.74	1.27	18.68	21.6	20.27
cis_mRNA_overlap	0	Lnc-AntiOverlap-mRNA	n324535	NM_006703_dup1	chr6	34355460	34360244	4785	+	chr6	34254567	34360457	2410	-	*	0.9832	1	0.04	0	0.05	18.76	14.39	21.32
cis_mRNA_up10k	4410	NA	n342492	NM_012091_dup1	chr16	75661630	75681577	1997	-	chr16	75632247	75657221	3545	-	*	-0.9796	-1	41.56	44.64	30.17	3.83	3.52	4.3
cis_mRNA_dw20k	9093	NA	LTCONS_00059143	NM_015669_dup1	chr5	140526796	140528097	1302	.	chr5	140514800	140517704	2905	+	*	0.9871	1	1.82	0.83	0.91	19.98	4.22	8.03
cis_mRNA_overlap	0	mRNA	LTCONS_00023646	NM_012388_dup1	chr15	45879327	45902393	3961	+	chr15	45879417	45902393	3959	+	-1475.46	0.7849	1	3.44	3.37	1.55	18.22	17.27	16.77
cis_mRNA_overlap	0	Lnc-CompleteIn-mRNAIntron	n385583	NM_015110_dup1	chr9	72935053	72937759	2707	+	chr9	72873878	72969789	5965	+	*	0.9649	1	0.58	0.87	1.17	6.92	7.28	8.33
cis_mRNA_dw20k	518	NA	n339347	NM_025010_dup1	chr3	47388823	47390563	1741	+	chr3	47324330	47388306	4724	+	*	0.9153	1	2.2	2.76	2.3	9.15	9.93	9.58
cis_mRNA_dw20k	2313	NA	n373936	NM_007084_dup1	chr13	95357954	95359564	1436	-	chr13	95361876	95364389	2514	-	*	0.9464	1	0.05	0.03	0.04	0.56	0.12	0.47
cis_mRNA_up10k	9056	NA	n411552	NM_018992_dup1	chr16	2708390	2723440	8336	-	chr16	2732495	2759031	2463	+	*	0.9939	1	1.55	1.96	2.78	10.78	11.12	12.25
cis_mRNA_overlap	0	AntiCompleteIn-mRNAExon	n339131	NM_014494_dup1	chr16	24788314	24788648	334	-	chr16	24741049	24837548	8423	+	*	-0.9999	-0.866	0.12	0.11	0.11	4.44	6.58	6.61
cis_mRNA_up10k	462	NA	n386461	NM_007349_dup1	chr7	154795143	154797412	2270	+	chr7	154735400	154794682	3712	-	*	0.9646	1	4.49	4.3	4.44	1.31	1.13	1.22
cis_mRNA_dw20k	2025	NA	n376626	NM_013248_dup1	chr20	23337432	23338753	505	-	chr20	23331373	23335408	1117	+	*	-0.9941	-1	0	0.07	0.13	9.96	9.88	9.78
cis_mRNA_dw20k	8803	NA	n337076	NM_003926_dup1	chr19	1556692	1567876	494	-	chr19	1576678	1592652	2559	-	*	0.6475	1	0	0.61	0.71	20.68	20.87	24.05
cis_mRNA_overlap	0	Lnc-AntiOverlap-mRNA	LTCONS_00034289	NM_016536_dup1	chr19	38037568	38078928	4299	+	chr19	38055155	38085673	2276	-	*	-0.9244	-1	0.21	0.02	0	2.48	2.82	3.11
cis_mRNA_dw20k	192	NA	n339255	NM_014463_dup1	chr3	14240060	14242620	1998	+	chr3	14220228	14239869	695	+	*	-0.9806	-1	0.56	0.76	0.15	42.84	42.38	45.66
cis_mRNA_dw20k	2500	NA	n344995	NM_015254_dup1	chr8	28913455	28922296	3981	+	chr8	28924795	29120610	8766	-	*	0.9983	1	2.48	3.15	3.21	0.51	1.71	1.91
cis_mRNA_up10k	64	NA	n339631	NM_001080415_dup1	chr3	142719749	142720309	561	-	chr3	142720372	142779567	7464	+	*	0.957	1	1.04	1.29	0.32	13.56	14.54	12.65
cis_mRNA_dw20k	14324	NA	n385994	NM_019054_dup1	chr10	88965545	89102315	1420	-	chr10	88854953	88951222	3404	+	*	-0.9944	-1	0.25	0.21	0.44	4.61	5.1	3.32
cis_mRNA_overlap	0	Lnc-AntiOverlap-mRNA	n410687	NM_013353_dup1	chr1	151138498	151142773	1986	+	chr1	151142463	151148547	1322	-	*	-0.7997	-0.866	1.1	1.59	2.34	0.24	0.05	0.05
cis_mRNA_dw20k	17863	NA	n370407	NM_015117_dup1	chr8	144499834	144501963	1075	+	chr8	144519825	144623620	3277	-	*	0.7852	1	0.85	1.94	1.55	1.64	2.11	1.65
cis_mRNA_up10k	4063	NA	n377310	NM_014941_dup1	chr22	31368249	31375377	5561	+	chr22	31322600	31364187	4467	-	*	-0.9609	-1	2.68	3.85	2.62	4.86	4.34	5.11
cis_mRNA_dw20k	811	NA	n341453	NM_016353_dup1	chr8	17081051	17083288	2238	+	chr8	17013836	17080241	4012	+	*	0.974	1	8.3	9.14	11.06	13.95	17.26	20.5
cis_mRNA_dw20k	5832	NA	n382654	NM_014272_dup1	chr15	79044449	79045714	391	+	chr15	79051545	79103773	5490	-	*	0.9999	0.866	0	0.09	0.09	1.37	2.4	2.42
cis_mRNA_up10k	112	NA	n369049	NM_014452_dup1	chr6	47277791	47280162	383	+	chr6	47199268	47277680	3634	-	*	0.9975	1	0	0.55	0.36	8.56	12.36	11.29
cis_mRNA_dw20k	6801	NA	n368097	NM_016218_dup1	chr5	74902446	74906582	770	+	chr5	74807657	74895646	4512	+	*	0.9877	1	0.47	0.43	0.54	9.67	9.18	11.41
cis_mRNA_up10k	32	NA	n338141	NM_007204_dup1	chr1	112296397	112298159	1763	-	chr1	112298190	112310199	3510	+	*	-0.8578	-1	0.11	0.25	0.05	11.37	11.17	12.08
cis_mRNA_up10k	6804	NA	n5543	NM_014450_dup1	chr9	35657750	35658014	265	-	chr9	35649295	35650947	1238	-	*	0.9905	0.866	8145.1	7808.43	7749.68	0.06	0.03	0.03
cis_mRNA_up10k	80	NA	n342005	NM_015275_dup1	chr12	105500702	105501413	712	-	chr12	105501492	105562908	5808	+	*	0.6313	0.866	0.86	1.25	0.86	16.05	16.79	12.44
cis_mRNA_overlap	0	Lnc-CompleteIn-mRNAExon	n338703	NM_014971_dup1	chr2	25380063	25382004	1942	+	chr2	25264973	25382004	7458	+	*	-0.9999	-1	5.35	4.26	5.77	67.83	76.92	64.54
cis_mRNA_dw20k	6303	NA	LTCONS_00076583	NM_014271_dup1	chrX	29980319	29986271	5953	.	chrX	28605681	29974017	2679	+	*	0.9976	1	1.52	1.43	1.63	7.57	6.74	8.87
cis_mRNA_overlap	0	Lnc-AntiOverlap-mRNA	LTCONS_00031760	NM_015971_dup1	chr17	73262212	73267303	3171	-	chr17	73257749	73262457	1415	+	*	0.9999	1	2.47	2.24	2.63	25.02	23.78	25.93
cis_mRNA_dw20k	8714	NA	n374280	NM_015091_dup1	chr14	45552347	45552901	467	-	chr14	45431416	45543634	6242	+	*	0.7807	0.866	0	0.07	0.07	0	0.32	0.87
cis_mRNA_overlap	0	mRNA	n338573	NM_003419_dup1	chr19	37341832	37403930	584	+	chr19	37341267	37370471	3113	+	*	-0.9839	-1	1.26	0.88	1.1	0.19	0.96	0.39
cis_mRNA_dw20k	446	NA	n338331	NM_007348_dup1	chr1	161929297	161929914	615	+	chr1	161736084	161928852	2488	+	*	-0.7814	-1	4.02	3.86	3.99	15	16.75	16.39
cis_mRNA_up10k	341	NA	n379545	NM_016125_dup1	chr17	58042457	58074333	1603	+	chr17	58029723	58042117	2016	-	*	-1	-1	0	0.02	0.02	2.07	2.01	2.01
cis_mRNA_up10k	403	NA	n377662	NM_014991_dup1	chr4	85887946	85928168	1594	+	chr4	85590685	85887544	14325	-	*	0.9372	1	0.62	0.75	0.66	13.48	15.98	15.03
cis_mRNA_dw20k	1461	NA	n345433	NM_006559_dup1	chr1	32510932	32526453	1306	+	chr1	32479491	32509472	2685	+	*	0.9999	1	3.2	3.11	3.02	29.42	27.27	25.24
cis_mRNA_overlap	0	Lnc-AntiOverlap-mRNA	LTCONS_00072191	NM_013366_dup1	chr9	140083054	140084826	1232	+	chr9	140069236	140083057	2700	-	*	0.9884	1	14.54	10.45	12.85	2.59	2.26	2.41
cis_mRNA_up10k	5246	NA	n386454	NM_016068_dup1	chr7	100893616	100895601	1986	-	chr7	100882893	100888371	739	-	*	0.6557	1	3.8	3.67	4.54	109.91	97.61	110.68
cis_mRNA_dw20k	4813	NA	n344473	NM_015957_dup1	chr11	34736325	34899030	977	+	chr11	34903842	34937939	1259	-	*	-0.9987	-1	3.44	0.76	2.61	5.73	6.91	6.15
cis_mRNA_dw20k	9865	NA	n371709	NM_016046_dup1	chr10	99178541	99185802	894	-	chr10	99195666	99205768	1150	-	*	0.823	1	3.43	3.63	3.75	9.11	9.27	11.39
cis_mRNA_dw20k	2302	NA	n378322	NM_006703_dup1	chr6	34247482	34252266	4785	+	chr6	34254567	34360457	2410	-	*	-0.9973	-1	0.71	0.94	0.61	18.76	14.39	21.32
cis_mRNA_dw20k	468	NA	n408239	NM_022006_dup1	chr19	35645672	35660788	1589	+	chr19	35634154	35645205	713	+	*	-0.9739	-1	0	0.06	0.04	8	5.28	5.59
cis_mRNA_dw20k	2740	NA	n374961	NM_007280_dup1	chr15	41590239	41598727	5196	-	chr15	41601466	41624819	1236	-	*	0.7146	0.866	0.04	0.02	0.02	0.23	0.05	0.18

cis_mRNA_dw20k	16155	NA	n374961	NM_007236_dup1	chr15	41590239	41598727	5196	-	chr15	41523437	41574085	3230	+	*		-0.7715	-0.866	0.04	0.02	0.02	26.65	31.61	28.41
cis_mRNA_overlap	0	Lnc-CompleteIn-mRNAIntron	n338637	NM_016030_dup1	chr2	3454789	3457703	2915	+	chr2	3383446	3483342	2524	+	*		0.6934	1	0.3	0.28	0.49	13.73	13.43	13.79
cis_mRNA_overlap	0	mRNA	LTCONS_00030389	NM_015982_dup1	chr17	7191544	7197546	4229	-	chr17	7191571	7197876	1583	-		-1269.25	0.9999	1	0.97	2.19	1.41	1.23	1.47	1.32
cis_mRNA_up10k	82	NA	n384189	NM_016426_dup1	chr22	46691041	46692557	1517	-	chr22	46692638	46726707	3112	+	*		-0.9905	-0.866	0.69	0.96	0.73	0.05	0.03	0.05
cis_mRNA_up10k	97	NA	n339194	NM_001040445_dup1	chr2	239334020	239335530	1511	-	chr2	239335626	239360891	6833	+	*		-0.9999	-1	1.19	0.79	0.7	7.5	7.98	8.08
cis_mRNA_dw20k	1426	NA	n333525	NM_006330_dup1	chr8	54955499	54957513	416	-	chr8	54958938	55014577	2574	-	*		0.6311	1	0.28	0	0.25	58.12	49.98	50.54
cis_mRNA_dw20k	2292	NA	n346168	NM_015279_dup1	chr12	65277103	65368537	1151	+	chr12	65218352	65274812	7838	+	*		-0.9943	-1	0.09	0.31	0.21	7.97	6.89	7.28
cis_mRNA_dw20k	9231	NA	n379823	NM_006435_dup1	chr11	318640	319615	366	+	chr11	308107	309410	730	+	*		-0.6887	-0.866	0.21	0.62	0.31	0.2	0.04	0.04
cis_mRNA_dw20k	7365	NA	LTCONS_00007578	NM_007033_dup1	chr1	2344247	2346219	1973	.	chr1	2323214	2336883	3102	+	*		0.9712	1	0.63	0.76	0.92	17.13	19.29	20.41
cis_mRNA_overlap	0	Lnc-CompleteIn-mRNAExon	n342411	NM_007084_dup1	chr13	95364042	95364270	229	-	chr13	95361876	95364389	2514	-	*		0.9207	1	0.32	0	0.14	0.56	0.12	0.47
tran	750094	NA	LTCONS_00036205	NM_003419_dup1	chr19	38120564	38149835	11008	-	chr19	37341267	37370471	3113	+		-1640.99	-0.7941	-1	0.56	0.23	0.28	0.19	0.96	0.39
cis_mRNA_overlap	0	mRNA	LTCONS_00036205	NM_014898_dup1	chr19	38120564	38149835	11008	-	chr19	38123389	38146313	4611	-		-2322.41	0.9619	1	0.56	0.23	0.28	18.32	16.36	17.16
cis_mRNA_overlap	0	mRNA	n341038	NM_015533_dup1	chr11	61114590	61116612	1639	+	chr11	61100654	61116231	4241	+	*		-0.8773	-0.866	0.02	0.02	0	1.72	1.24	2.24
cis_mRNA_dw20k	1388	NA	n338325	NM_016029_dup1	chr14	60606871	60610109	3239	+	chr14	60611496	60632211	1392	-	*		0.8584	1	4.83	4.73	4.97	61.54	51.56	62.52
cis_mRNA_up10k	514	NA	n380308	NM_014488_dup1	chr11	82783397	82784754	860	+	chr11	82692478	82782884	1602	-	*		0.961	1	1.2	2.07	2.88	1.91	3.34	3.77
tran	NA	NA	LTCONS_00063644	NM_058182_dup1	chr6	166778407	166796486	4211	-	chr21	35747749	35761452	900	+		-728.82	-0.7826	-1	0.85	1.26	1.18	10.14	7.84	9.68
cis_mRNA_overlap	0	Lnc-AntiOverlap-mRNA	n338987	NM_013450_dup1	chr2	160471807	160473593	1494	+	chr2	160175490	160473059	8145	-	*		-0.9898	-0.866	0.46	0.44	0.46	31.36	37.71	30.21
cis_mRNA_overlap	0	Lnc-AntiOverlap-mRNA	n407869	NM_007218_dup1	chr8	125500735	125551329	1148	-	chr8	125487008	125500859	3312	+	*		-0.994	-1	0.78	1.81	0.46	16.82	16.55	16.86
cis_mRNA_up10k	253	NA	n372581	NM_014488_dup1	chr11	82783136	82784917	706	+	chr11	82692478	82782884	1602	-	*		0.9878	1	0	0.25	0.27	1.91	3.34	3.77
cis_mRNA_up10k	222	NA	n378478	NM_014904_dup1	chr10	119806335	119969663	3268	+	chr10	119764427	119806114	6061	-	*		0.9881	1	0.21	0.2	0.25	14.48	13.89	15.61
cis_mRNA_dw20k	3468	NA	n408887	NM_006351_dup1	chr19	7985867	7988136	1447	+	chr19	7991603	8008708	2000	-	*		-0.9985	-1	0	2.11	0.38	16.63	15.21	16.3
cis_mRNA_overlap	0	mRNA	n341790	NM_016174_dup1	chr9	131174045	131198079	4593	+	chr9	131182759	131199630	2689	+	*		0.9855	1	1.56	2.87	2.36	5.71	7.29	6.92
cis_mRNA_dw20k	2325	NA	n379199	NM_016113_dup1	chr17	16342641	16367300	1143	+	chr17	16318856	16340317	2808	+	*		-0.9957	-1	0.49	0.44	0.27	1.4	2.29	4.24
cis_mRNA_up10k	491	NA	n410014	NM_014587_dup1	chr16	1025761	1031318	1751	-	chr16	1031808	1036979	3049	+	*		-0.9673	-1	4.99	5.16	4.88	1.13	0.8	1.66
cis_mRNA_dw20k	15256	NA	n409540	NM_015023_dup1	chr1	27650365	27653016	2652	+	chr1	27561007	27635110	4802	+	*		-0.8901	-1	1.4	1.53	1.61	2.23	2.1	1.57
cis_mRNA_up10k	477	NA	n377749	NM_016065_dup1	chr10	75012927	75014107	635	+	chr10	75008601	75012451	2634	-	*		-0.7905	-1	0.26	0.22	0	32.87	33.85	34.18
cis_mRNA_dw20k	11894	NA	n339360	NM_006545_dup1	chr3	50371526	50372548	1022	+	chr3	50384441	50388486	1672	-	*		0.9927	0.866	0.03	0.09	0.03	6.57	7.1	6.49
cis_mRNA_up10k	5859	NA	n339360	NM_007275_dup1	chr3	50371526	50372548	1022	+	chr3	50362341	50365668	1682	-	*		-0.8605	-0.866	0.03	0.09	0.03	21.13	19.01	20.05
cis_mRNA_dw20k	3137	NA	n337988	NM_005744_dup1	chr15	72882032	72884186	2155	+	chr15	72766667	72878896	5251	+	*		-0.9636	-1	5.74	6.64	5.5	24.75	23.24	26.01
cis_mRNA_dw20k	3870	NA	n408908	NM_012134_dup1	chr1	201857797	201861715	1375	+	chr1	201865584	201915716	3967	-	*		0.881	0.866	0.47	1.56	2.54	0.01	0.02	0.02
cis_mRNA_dw20k	591	NA	n341167	NM_015261_dup1	chr11	134020575	134021747	1173	-	chr11	134022337	134094426	5605	-	*		-0.9595	-1	0.57	0.41	0.83	2.51	2.53	2.35
cis_mRNA_overlap	0	Lnc-AntiOverlap-mRNA	n342219	NM_007280_dup1	chr15	41576230	41601901	1350	+	chr15	41601466	41624819	1236	-	*		-0.982	-1	0.78	1.22	0.82	0.23	0.05	0.18
cis_mRNA_dw20k	2146	NA	n342219	NM_007236_dup1	chr15	41576230	41601901	1350	+	chr15	41523437	41574085	3230	+	*		0.9624	1	0.78	1.22	0.82	26.65	31.61	28.41
cis_mRNA_dw20k	1564	NA	n338419	NM_015101_dup1	chr1	183900822	183903403	2581	-	chr1	183904966	184006863	5182	-	*		0.9602	1	0.42	0.55	0.81	3.83	4.01	7.01
cis_mRNA_overlap	0	Lnc-CompleteIn-mRNAIntron	n337678	NM_015307_dup1	chr15	29665806	29669160	3352	-	chr15	29412455	29862927	4707	-	*		0.6246	1	0.43	0.36	0.84	14.05	6.93	14.18
cis_mRNA_dw20k	1915	NA	n339353	NM_016479_dup1	chr3	48505605	48507283	1679	-	chr3	48509197	48541661	2166	-	*		-0.9948	-1	0.51	0.45	0.4	8.16	9.95	10.98
cis_mRNA_overlap	0	CompleteIn-LncIntron	n410671	NM_023922_dup1	chr12	10998448	11324224	1582	-	chr12	11090853	11091806	954	-	*		0.6718	1	5.24	4.97	4.93	1.48	1.45	1.19
cis_mRNA_up10k	2440	NA	LTCONS_00022969	NM_015656_dup1	chr14	104601279	104602621	1343	-	chr14	104605060	104647235	6757	+	*		0.9908	1	0.49	0.76	0.32	17.05	19.7	14.35
cis_mRNA_overlap	0	mRNA	n340076	NM_012330_dup1	chr10	76672544	76944815	2287	+	chr10	76586379	76792639	8550	+	*		0.9414	1	0.51	0.77	0.62	1.04	1.64	1.1
cis_mRNA_overlap	0	Lnc-CompleteIn-mRNAIntron	n341611	NM_015137_dup1	chr8	133010124	133011947	1822	+	chr8	132916356	133025889	5417	+	*		0.9333	1	0.45	0.5	0.44	7.06	7.56	6.56
cis_mRNA_up10k	7888	NA	n385144	NM_019082_dup1	chr7	44622024	44623270	1247	+	chr7	44605403	44614137	2485	-	*		-0.8669	-1	0.92	1.07	0.8	10.13	9.93	12.66
cis_mRNA_dw20k	9528	NA	n338899	NM_007254_dup1	chr19	50341902	50354933	2149	-	chr19	50364460	50370822	1721	-	*		0.6179	0.866	0.13	0.13	0.27	4.86	4.14	4.99
cis_mRNA_overlap	0	Lnc-AntiOverlap-mRNA	n338899	NM_017432_dup1	chr19	50341902	50354933	2149	-	chr19	50354416	50363999	1884	+	*		-0.8175	-0.866	0.13	0.13	0.27	45.49	51.4	41.18
cis_mRNA_overlap	0	Lnc-AntiOverlap-mRNA	LTCONS_00011091	NM_021008_dup1	chr11	695614	704197	5183	+	chr11	644225	695740	2716	-	*		0.9246	1	0.97	1.49	1.81	35.31	36.74	40.51
cis_mRNA_up10k	57	NA	n409692	NM_016422_dup1	chr11	10562830	10621479	614	+	chr11	10533225	10562774	4084	-	*		0.9972	1	0.06	0.05	0.27	12.65	12.03	17.01
cis_mRNA_dw20k	3974	NA	n386971	NM_016080_dup1	chr17	655900	658576	2677	+	chr17	662549	685571	1837	-	*		-0.9114	-1	0.36	0.38	0.33	55.44	50.92	56.74
cis_mRNA_up10k	400	NA	n386971	NM_015721_dup1	chr17	655900	658576	2677	+	chr17	647661	655501	3741	-	*		0.9904	1	0.36	0.38	0.33	3.92	4.43	3.45
cis_mRNA_up10k	87	NA	n344535	NM_019059_dup1	chr7	22862507	22922065	7144	+	chr7	22852251	22862421	729	-	*		0.7046	0.866	0.05	0.04	0.04	69.26	54.75	65.42
cis_mRNA_dw20k	17787	NA	n341857	NM_016072_dup1	chr12	21689123	21757554	2902	-	chr12	21654699	21671337	3272	+	*		0.838	1	0	0.14	0.16	9.02	9.93	11.6
cis_mRNA_dw20k	243	NA	n385777	NM_013346_dup1	chrX	70279099	70280546	1448	-	chrX	70280788	70288231	642	-	*		0.9949	1	50.78	49.03	53	48.39	45.71	50.78
cis_mRNA_overlap	0	mRNA	n407095	NM_016401_dup1	chr11	86014398	86056985	1079	+	chr11	86013253	86056985	1137	+	*		0.9944	1	0.08	0	0.34	37.15	36.47	41.66
cis_mRNA_dw20k	4107	NA	n375211	NM_014587_dup1	chr16	1041085	1043057	1611	-	chr16	1031808	1036979	3049	+	*		-0.7918	-0.866	0.02	0.04	0.02	1.13	0.8	1.66
cis_mRNA_overlap	0	Lnc-CompleteIn-mRNAExon	n337962	NM_015306_dup1	chr1	55559717	55613437	4245	-	chr1	55532032	55681039	10802	-	*		0.8695	1	1.37	0.18	2.49	9.43	9.41	10.13
cis_mRNA_up10k	286	NA	n337402	NM_014908_dup1	chr9	131710297	131719290	881	+	chr9	131707809	131710012	2204	-	*		-0.9999	-1	0.4	0.51	0.11	6.97	6.56	7.97
cis_mRNA_overlap	0	Lnc-AntiOverlap-mRNA	n408025	NM_015703_dup1	chr22	42896585	42908566	1357	+	chr22	42904341	42915829	5455	-	*		0.9175	1	0	0.37	0.67	8.08	8.5	10.35
cis_mRNA_up10k	151	NA	n374518	NM_007223_dup1	chr15	40213243	40217099	2729	+	chr15	40092931	40213093	2816	-	*		0.8675	1	0.07	0.22	0.01	37.7	43.83	19.57
cis_mRNA_up10k	1070	NA	n339759	NM_012469_dup1	chr20	62605467	62611362	2070	-	chr20	62612431	62664453	3101	+	*		0.8874	1	0.53	1	0.75	24.17	27.57	24.2
cis_mRNA_overlap	0	Lnc-AntiOverlap-mRNA	n341149	NM_014026_dup1	chr11	126210853	126225431	1460	-	chr11	126173647	126215644	1479	+	*		0.6919	1	0.64	0.88	0.87	2.65	3.87	2.91
cis_mRNA_dw20k	6244	NA	n340788	NM_006098_dup1	chr5	180649869	180657685	2343	-	chr5	180663928	180670906	1109	-	*		-0.8911	-0.866	0.03	0.03	0.04	537.41	496.85	448.17
cis_mRNA_dw20k	404	NA	n411758	NM_016395_dup1	chr15	65871096	65903474	2703	-	chr15	65822827	65870693	3208	+	*		0.9998	1	2.16	1.98	2.39	206.66	198	218.43

cis_mRNA_overlap	0	mRNA	LTCONS_00034161	NM_022006_dup1	chr19	35639044	35645207	3674	+	chr19	35634154	35645205	713	+		-566.1	-0.8724	-1	2.7	5.03	3.68	8	5.28	5.59
cis_mRNA_dw20k	3094	NA	n338142	NM_007204_dup1	chr1	112313292	112315191	1401	-	chr1	112298190	112310199	3510	+	*		-0.725	-1	3.03	5.61	2.79	11.37	11.17	12.08
cis_mRNA_up10k	161	NA	n365489	NM_144563_dup1	chr2	88986204	88991016	1749	-	chr2	88991176	89050452	1834	+	*		0.8109	0.866	0.05	0.22	0.12	2.4	2.5	2.5
cis_mRNA_up10k	9264	NA	LTCONS_00025596	NM_015154_dup1	chr15	81291468	81293179	1712	.	chr15	81268095	81282205	4243	-	*		-0.7524	-1	0.09	0.07	0.24	11.76	12.4	11.57
cis_mRNA_overlap	0	Lnc-CompleteIn-mRNAExon	n340631	NM_015288_dup1	chr5	133861798	133918918	6463	+	chr5	133861798	133918918	6463	+	*		1	1	0.07	0.26	0.1	0.07	0.26	0.1
cis_mRNA_up10k	4656	NA	n332690	NM_015417_dup1	chr20	3766757	3767130	374	-	chr20	3758151	3762102	1587	-	*		-0.9767	-1	5.94	3.52	4.27	0.2	0.34	0.27
cis_mRNA_up10k	9121	NA	n342456	NM_018992_dup1	chr16	2710350	2723375	1449	-	chr16	2732495	2759031	2463	+	*		0.9096	1	2.52	3.84	4.68	10.78	11.12	12.25
tran	NA	NA	LTCONS_00067896	NM_007209_dup1	chr7	66071463	66072190	728	.	chr9	127620158	127624240	460	-		-940.2	-0.9873	-1	0.69	1.8	1.02	575.09	489.51	537.51
cis_mRNA_up10k	19	NA	n338747	NM_007059_dup1	chr19	47987539	47999639	1753	+	chr19	47978400	47987521	1688	-	*		0.9928	1	0.39	0.61	0.27	3.8	4.19	3.47
cis_mRNA_dw20k	13022	NA	n341933	NM_014925_dup1	chr12	57628694	57634527	2010	-	chr12	57647548	57704246	3974	-	*		0.9928	1	0.63	0.78	0.37	5.26	5.89	3.44
cis_mRNA_overlap	0	Lnc-CompleteIn-mRNAIntron	n338203	NM_006699_dup1	chr1	118036137	118037628	1492	+	chr1	117910085	118068320	5388	+	*		0.8693	1	0.78	0.8	0.91	17.43	19.3	20.45
cis_mRNA_up10k	1026	NA	n345167	NM_016546_dup1	chr12	7262894	7275421	3761	+	chr12	7247146	7261869	3398	-	*		0.9971	1	0.04	0.11	0.05	1.75	3.13	2.05
cis_mRNA_up10k	52	NA	n370213	NM_015169_dup1	chr8	67331824	67341212	4366	-	chr8	67341263	67342968	1706	+	*		0.923	1	0.04	0.06	0.07	2.33	2.41	2.58
cis_mRNA_dw20k	16573	NA	n340020	NM_007021_dup1	chr10	45306472	45455137	3300	-	chr10	45471709	45474330	2060	-	*		0.8177	1	0.21	0.13	0.09	3.98	3.69	1.34
cis_mRNA_overlap	0	Lnc-AntiOverlap-mRNA	LTCONS_00034290	NM_016536_dup1	chr19	38038835	38078928	6569	+	chr19	38055155	38085673	2276	-	*		-0.9388	-1	0.35	0.16	0.13	2.48	2.82	3.11
cis_mRNA_up10k	116	NA	LTCONS_00007135	NM_014053_dup1	chr1	213025450	213031482	2577	-	chr1	213031597	213072705	5939	+	*		0.9935	1	0.87	1.21	0.66	16.71	20.97	12.76
cis_mRNA_dw20k	17347	NA	n367424	NM_007281_dup1	chr4	174284949	174291953	1089	-	chr4	174309299	174320617	903	-	*		-0.8066	-1	0.49	0.37	0.5	40.23	57	8.28
cis_mRNA_overlap	0	mRNA-AntiCompleteIn-LncIntron	n379409	NM_006387_dup1	chr19	15050246	16748905	1493	+	chr19	16628700	16653263	4095	-	*		0.9948	1	0.05	0.08	0.06	1.23	1.69	1.34
cis_mRNA_overlap	0	mRNA-AntiCompleteIn-LncIntron	n379409	NM_006844_dup1	chr19	15050246	16748905	1493	+	chr19	15225785	15236577	2298	-	*		0.9811	1	0.05	0.08	0.06	8.48	12.35	10.43
cis_mRNA_dw20k	396	NA	n375549	NM_015229_dup1	chr17	2591628	2592285	473	+	chr17	2592680	2614927	5224	-	*		0.8024	1	0.4	0.43	0.5	5.27	6.36	6.51
cis_mRNA_overlap	0	Lnc-CompleteIn-mRNAIntron	n339814	NM_015461_dup1	chr18	22913212	22914068	856	-	chr18	22641888	22932214	4971	-	*		-0.6075	-0.866	0.04	0.04	0.07	3.48	6.56	2.98
cis_mRNA_dw20k	10807	NA	n345496	NM_018158_dup1	chr2	27928653	27989051	2722	+	chr2	27886338	27917847	2970	+	*		0.9267	1	0.34	0.49	0.42	12.41	14.42	14.2
cis_mRNA_overlap	0	AntiCompleteIn-mRNAExon	LTCONS_00044955	NM_007346_dup1	chr20	61444454	61445005	312	-	chr20	61436177	61445352	2420	+		-719.88	-0.7512	-1	0.34	0.75	0.28	16.83	16.44	18.23
cis_mRNA_overlap	0	mRNA	n408146	NM_015726_dup1	chr1	160185505	160232318	4183	-	chr1	160185505	160232350	3883	-	*		-0.9996	-1	4.09	4.42	4.59	1.32	1.19	1.13
cis_mRNA_dw20k	7438	NA	n369004	NM_006355_dup1	chr6	25992968	26002137	4186	+	chr6	25963071	25985531	3286	+	*		1	1	0.16	0.07	0.07	0.05	0.04	0.04
cis_mRNA_overlap	0	Lnc-CompleteIn-mRNAExon	n342240	NM_021992_dup1	chrX	101768611	101771673	639	-	chrX	101768610	101771699	666	-	*		0.9972	1	128.3	108.97	111.51	46.99	22.55	27.57
cis_mRNA_dw20k	3353	NA	LTCONS_00032008	NM_007202_dup1	chr17	19799934	19805397	5464	.	chr17	19808749	19881129	2917	-	*		0.9872	1	0.2	0.32	0.26	7.85	8.35	8.03
cis_mRNA_up10k	27	NA	n338211	NM_007192_dup1	chr14	21852451	21853079	629	+	chr14	21819631	21852425	4684	-	*		0.7465	1	0.17	0.11	0.1	49.53	48.68	45.21
cis_mRNA_overlap	0	Lnc-AntiOverlap-mRNA	LTCONS_00006032	NM_006818_dup1	chr1	151023381	151032562	3336	-	chr1	151032151	151040973	2158	+	*		-0.9361	-1	2.66	3.9	2.16	223.4	211.5	241.49
cis_mRNA_dw20k	14934	NA	n377747	NM_144563_dup1	chr2	89065385	89106126	5395	+	chr2	88991176	89050452	1834	+	*		0.7168	0.866	3.8	3.96	4.37	2.4	2.5	2.5
cis_mRNA_dw20k	7772	NA	n345182	NM_015026_dup1	chr12	62999134	63014175	4107	+	chr12	62860597	62991363	10379	+	*		0.9621	1	1.57	1.73	1.21	5.02	5.57	4.57
cis_mRNA_dw20k	8387	NA	LTCONS_00015883	NM_014182_dup1	chr12	56223345	56230045	5392	+	chr12	56211806	56214959	1334	+	*		0.9935	1	1.25	1.29	1.87	8.09	8.21	8.75
cis_mRNA_up10k	2082	NA	n339988	NM_006587_dup1	chr4	47842140	47846356	4215	-	chr4	47596018	47840059	4942	-	*		-0.9979	-1	3.28	3.08	3.53	0.1	0.14	0.06
cis_mRNA_dw20k	6847	NA	n377982	NM_015726_dup1	chr1	160171989	160178659	1578	-	chr1	160185505	160232350	3883	-	*		-0.8249	-1	0.13	0.14	0.2	1.32	1.19	1.13
cis_mRNA_overlap	0	Lnc-AntiOverlap-mRNA	LTCONS_00037872	NM_014614_dup1	chr2	54197976	54284448	747	+	chr2	54091204	54197977	7099	-	*		0.8908	0.866	2.5	0.83	1.59	5.95	5.17	5.17
cis_mRNA_dw20k	1842	NA	n410160	NM_015910_dup1	chr2	63344986	63346677	1692	-	chr2	63348518	63815867	3326	-	*		0.8344	1	0.04	0.05	0.02	1.11	1.6	1.03
cis_mRNA_up10k	192	NA	n341599	NM_007218_dup1	chr8	125485051	125486817	1764	-	chr8	125487008	125500859	3312	+	*		0.8535	1	0.71	0.68	0.76	16.82	16.55	16.86
cis_mRNA_overlap	0	Lnc-AntiOverlap-mRNA	n408116	NM_001080437_dup1	chr2	242026509	242041747	4566	-	chr2	241938255	242033643	6834	+	*		0.9744	1	1.19	1.79	1.37	0.02	0.04	0.03
cis_mRNA_dw20k	16636	NA	n377958	NM_015094_dup1	chr22	21822387	21824214	763	+	chr22	21771693	21805752	6798	+	*		0.951	1	0.98	1.61	1.32	9.58	11.42	11.1
cis_mRNA_overlap	0	Lnc-AntiOverlap-mRNA	n342787	NM_001040455_dup1	chr11	117066329	117072630	1242	-	chr11	117049939	117068161	3919	+	*		0.8017	1	0	0.26	0.05	1.79	2.95	2.68
cis_mRNA_dw20k	4401	NA	n384507	NM_014278_dup1	chr4	128758922	128761883	2962	+	chr4	128703453	128754522	3266	+	*		0.934	1	4.74	5.68	6.23	20.27	27.47	27.54
cis_mRNA_dw20k	13243	NA	n408276	NM_015533_dup1	chr11	61129473	61136975	1725	+	chr11	61100654	61116231	4241	+	*		0.9879	1	0.08	0	0.13	1.72	1.24	2.24
cis_mRNA_up10k	9056	NA	LTCONS_00027248	NM_018992_dup1	chr16	2692410	2723440	2293	-	chr16	2732495	2759031	2463	+	*		0.9932	1	1.78	2.21	3.05	10.78	11.12	12.25
cis_mRNA_overlap	0	Lnc-CompleteIn-mRNAExon	n341541	NM_016010_dup1	chr8	79630315	79631995	1678	+	chr8	79578282	79632000	3352	+	*		0.9955	1	8.62	6.5	9.11	24.39	21.22	24.71
cis_mRNA_up10k	1284	NA	n345544	NM_015702_dup1	chr2	150445613	150455210	2159	+	chr2	150426147	150444330	1437	-	*		-0.8961	-1	0.06	0.04	0.16	71.38	75.03	68.74
cis_mRNA_dw20k	10015	NA	LTCONS_00048842	NM_015512_dup1	chr3	52444527	52450253	2642	+	chr3	52350335	52434513	13110	+	*		0.994	1	0	1.07	0.61	0.67	1	0.89
cis_mRNA_up10k	229	NA	LTCONS_00063815	NM_012419_dup1	chr6	153452617	153453461	845	.	chr6	153332026	153452389	1472	-	*		-0.998	-1	0.42	1.19	0.87	8.04	7.79	7.88
cis_mRNA_overlap	0	Lnc-AntiOverlap-mRNA	n338717	NM_007046_dup1	chr2	27307990	27309442	1453	-	chr2	27301435	27309271	3938	+	*		-0.8738	-1	0.76	0.83	0.81	0.83	0.49	0.74
cis_mRNA_overlap	0	Lnc-AntiOverlap-mRNA	n378962	NM_001145418_dup1	chr22	28315417	28393763	562	+	chr22	28374002	29075853	11795	-	*		0.9152	1	0.19	0.12	0	10.67	9.4	8.94
cis_mRNA_up10k	129	NA	n378317	NM_014016_dup1	chr3	45720724	45730626	955	-	chr3	45730754	45786900	3707	+	*		-0.9909	-1	0.06	0	0.11	11.65	12.62	11.16
cis_mRNA_up10k	8725	NA	n345686	NM_014280_dup1	chr1	28568266	28573567	688	-	chr1	28526789	28559542	1765	-	*		0.7977	1	0.42	0.43	0.05	51.19	53.79	49.65
cis_mRNA_overlap	0	Lnc-AntiOverlap-mRNA	n406196	NM_015983_dup1	chr7	43980494	44058748	6466	-	chr7	43966035	43995735	3982	+	*		-0.9193	-1	0.37	0.42	0.34	3.47	3.29	3.96
cis_mRNA_overlap	0	Lnc-CompleteIn-mRNAIntron	n326396	NM_014455_dup1	chr1	145636538	145638085	446	+	chr1	145611036	145688776	1675	+	*		-0.9638	-1	0.17	0.08	0	18.83	19.91	20.23
cis_mRNA_up10k	459	NA	n370120	NM_019005_dup1	chr7	7591941	7606158	923	-	chr7	7606616	7647112	3325	+	*		0.7815	0.866	0.18	0.01	0.18	27.83	23.44	25.06

cis_mRNA_dw20k	7907	NA	n346033	NM_022044_dup1	chr22	22006494	22007888	385	+	chr22	21996542	21998588	874	+	*		0.9993	1	2.01	1.28	1.35	4.76	4.41	4.43
cis_mRNA_dw20k	8178	NA	n371329	NM_015884_dup1	chrX	21911718	21920026	7619	+	chrX	21857656	21903541	4525	+	*		-0.982	-1	0.3	0.49	0.35	4.55	4.28	4.53
cis_mRNA_overlap	0	CompleteIn-LncExon	n408119	NM_001033088_dup1	chr15	90808895	90815443	1760	+	chr15	90808895	90815443	1349	+	*		-0.9206	-0.866	213.54	215.22	217.81	35.01	35.01	25.17
cis_mRNA_dw20k	14678	NA	n371497	NM_014008_dup1	chrX	49121664	49123331	1519	-	chrX	49091927	49106987	2319	+	*		-0.9078	-1	0.11	0.04	0.06	7.9	8.53	8.11
cis_mRNA_dw20k	3896	NA	n373805	NM_015567_dup1	chr13	88335766	88349465	9590	+	chr13	88324870	88331871	4447	+	*		0.9869	1	1.51	1.66	2.28	3.03	3.9	5.55
tran	NA	NA	LTCONS_00055412	NM_006601_dup1	chr4	170712130	170712793	664	.	chr12	57057125	57082078	1916	-		-1127.7	0.6955	1	0.05	0.04	0	72.84	65.89	65.43
cis_mRNA_dw20k	2519	NA	n339096	NM_006331_dup1	chr12	7087683	7089096	1414	+	chr12	7079944	7085165	1070	+	*		0.9213	1	2.43	2.17	2.52	7.13	6.08	8.77
cis_mRNA_overlap	0	AntiCompleteIn-mRNAIntron	n384036	NM_144991_dup1	chr21	45937098	45938859	970	+	chr21	45917775	46131495	3967	-	*		-0.9942	-1	0.26	0.3	0.33	0.06	0.02	0
cis_mRNA_up10k	3623	NA	n340760	NM_013237_dup1	chr5	176724685	176727213	2528	+	chr5	176730835	176733950	1191	+	*		0.7082	0.866	0	0.02	0.02	30.85	32.38	36.53
cis_mRNA_dw20k	13059	NA	LTCONS_00067562	NM_014149_dup1	chr7	134850440	134855532	3755	-	chr7	134868590	134896316	4619	-	*		0.9999	1	1.44	1.68	1.52	3.73	4.29	3.91
cis_mRNA_overlap	0	AntiCompleteIn-mRNAIntron	n338746	NM_001040429_dup1	chr13	58246842	58247428	587	-	chr13	58205789	58303065	8009	+	*		0.7804	1	0.06	0.28	0.11	1.93	5.78	5.15
cis_mRNA_overlap	0	Lnc-CompleteIn-mRNAExon	n407178	NM_013349_dup1	chr1	212606229	212619721	888	+	chr1	212606229	212619721	949	+	*		-0.7809	-1	2.63	3.1	2.53	15.8	15.36	17.39
cis_mRNA_overlap	0	Lnc-CompleteIn-mRNAIntron	n342914	NM_014942_dup1	chr6	90279820	90280172	353	+	chr6	90142897	90343403	5190	+	*		0.7036	0.866	0	0.1	0.1	8.42	12.71	9.55
cis_mRNA_dw20k	19027	NA	n337877	NM_016037_dup1	chr1	38509523	38511025	1503	-	chr1	38478384	38490497	2050	+	*		-0.8546	-1	3.89	2.55	4.08	21	21.16	20.74
cis_mRNA_dw20k	1985	NA	n407698	NM_016113_dup1	chr17	16342301	16345340	1013	+	chr17	16318856	16340317	2808	+	*		-0.7578	-1	7.73	4.65	4.56	1.4	2.29	4.24
cis_mRNA_dw20k	4547	NA	LTCONS_00023125	NM_013231_dup1	chr14	86098816	86101707	2892	.	chr14	85996488	86094270	7179	+	*		0.8376	1	0.58	0.91	0.06	2.11	4.79	1.87
cis_mRNA_up10k	176	NA	LTCONS_00025582	NM_015617_dup1	chr15	55881225	55882354	1130	.	chr15	55838221	55881050	1260	-	*		-0.8566	-1	2.26	1.8	1.69	3.39	3.81	4.69
cis_mRNA_overlap	0	Lnc-CompleteIn-mRNAIntron	n381775	NM_025164_dup1	chr11	116941486	116942325	840	-	chr11	116714118	116968993	6069	-	*		-0.9042	-0.866	0.21	0.08	0.08	3.3	3.46	3.58
cis_mRNA_overlap	0	Lnc-CompleteIn-mRNAIntron	n385465	NM_018482_dup1	chr8	131307601	131308779	1179	-	chr8	131064353	131414217	6042	-	*		-0.9583	-1	0.77	0.6	0.74	3.63	7.8	5.47
cis_mRNA_overlap	0	Lnc-CompleteIn-mRNAExon	n341331	NM_015135_dup1	chr7	135242688	135333497	6237	+	chr7	135242662	135333501	6267	+	*		-0.9932	-1	3.56	2.36	8.37	12.26	14.19	7.95
cis_mRNA_up10k	136	NA	n374590	NM_007223_dup1	chr15	40213228	40218031	3565	+	chr15	40092931	40213093	2816	-	*		-0.6954	-0.866	0.04	0	0.04	37.7	43.83	19.57
cis_mRNA_up10k	2315	NA	n366062	NM_016474_dup1	chr3	14689701	14690939	446	-	chr3	14693253	14714166	2960	+	*		0.682	0.866	0	0.08	0.08	7.05	7.56	7.17
cis_mRNA_dw20k	13185	NA	n376419	NM_016536_dup1	chr19	38038730	38041971	2547	-	chr19	38055155	38085673	2276	-	*		-0.8937	-1	2.86	0.1	0.06	2.48	2.82	3.11
cis_mRNA_dw20k	1985	NA	n407694	NM_016113_dup1	chr17	16342301	16345340	1123	+	chr17	16318856	16340317	2808	+	*		-0.8226	-1	27.41	13.74	11.49	1.4	2.29	4.24
cis_mRNA_overlap	0	Lnc-AntiOverlap-mRNA	n339141	NM_015202_dup1	chr16	27719346	27730125	957	-	chr16	27561468	27791692	6623	+	*		0.8278	0.866	0	0.03	0.03	1.79	2.27	2
cis_mRNA_up10k	223	NA	n376605	NM_012384_dup1	chr20	62258603	62260060	582	+	chr20	62218955	62258381	4210	-	*		0.9483	1	0.07	1.4	0	2.75	2.82	2.71
cis_mRNA_dw20k	11002	NA	n376605	NM_015894_dup1	chr20	62258603	62260060	582	+	chr20	62271061	62284780	2255	-	*		-0.6583	-1	0.07	1.4	0	190.09	181.92	232.64
cis_mRNA_overlap	0	mRNA	n406466	NM_017444_dup1	chr8	141521397	141527252	2377	+	chr8	141521401	141527252	2500	+	*		0.6744	1	0.33	0.15	0.31	1.39	1.31	1.32
cis_mRNA_overlap	0	Lnc-AntiOverlap-mRNA	n410795	NM_016536_dup1	chr19	38042273	38078248	2510	+	chr19	38055155	38085673	2276	-	*		-0.9536	-1	5.18	1.28	0.41	2.48	2.82	3.11
cis_mRNA_overlap	0	mRNA	LTCONS_00044959	NM_006602_dup1	chr20	61482941	61492241	6975	-	chr20	61472467	61493115	2456	-	*		0.973	1	1.81	2.48	2.03	0.68	1.3	1.01
cis_mRNA_overlap	0	Lnc-AntiOverlap-mRNA	n378000	NM_001145418_dup1	chr22	28315389	28382380	846	+	chr22	28374002	29075853	11795	-	*		0.9942	1	0.7	0.39	0.33	10.67	9.4	8.94
cis_mRNA_up10k	394	NA	n409207	NM_014159_dup1	chr3	47205860	47285606	2540	+	chr3	47057898	47205467	8433	-	*		0.8532	1	0.51	0.63	0.32	17.39	18.01	17.31
cis_mRNA_overlap	0	Lnc-AntiOverlap-mRNA	n341912	NM_015086_dup1	chr12	49392150	49412988	1294	+	chr12	49388933	49393088	3744	-	*		0.7628	1	0.16	0.25	0.17	0.03	0.07	0.06
cis_mRNA_dw20k	2325	NA	n379167	NM_016113_dup1	chr17	16342641	16367300	794	+	chr17	16318856	16340317	2808	+	*		0.7413	0.866	0	0.05	0.05	1.4	2.29	4.24
cis_mRNA_up10k	7792	NA	n338136	NM_007204_dup1	chr1	112282463	112290399	2110	+	chr1	112298190	112310199	3510	+	*		-0.9982	-1	3.3	3.43	2.96	11.37	11.17	12.08
cis_mRNA_up10k	202	NA	n378541	NM_016470_dup1	chr20	42839632	42854667	1563	+	chr20	42825136	42839431	1572	-	*		-0.7928	-1	0.14	0.15	0.39	16.45	14.88	14.02
cis_mRNA_overlap	0	Lnc-AntiOverlap-mRNA	n411089	NM_016256_dup1	chr16	5083703	5092972	1237	+	chr16	5074845	5083942	2209	-	*		0.9734	1	0.11	0.31	0.38	2.08	2.88	2.9
cis_mRNA_overlap	0	Lnc-AntiOverlap-mRNA	n338768	NM_006449_dup1	chr2	37898911	37899815	905	+	chr2	37870743	37899326	3981	-	*		0.7836	1	0.31	0	0.42	12.03	10.83	19.94
cis_mRNA_dw20k	2069	NA	n339365	NM_017442_dup1	chr3	52251032	52253028	1997	-	chr3	52255096	52260179	3870	-	*		0.8062	1	0.02	0.06	0.03	0.05	0.16	0.14
cis_mRNA_overlap	0	mRNA	n342404	NM_015131_dup1	chr2	29117536	29171084	3500	+	chr2	29117533	29171080	3501	+	*		-0.9981	-1	8.97	0.02	4.65	3.96	13.69	8.13
cis_mRNA_dw20k	12026	NA	n344460	NM_006092_dup1	chr7	30423846	30452118	5954	+	chr7	30464143	30518393	4496	-	*		0.9726	1	0.06	0.04	0.1	0.18	0.11	0.24
cis_mRNA_dw20k	17686	NA	n380446	NM_015094_dup1	chr22	21823437	21825558	1814	+	chr22	21771693	21805752	6798	+	*		0.9546	1	0.8	1.2	1.02	9.58	11.42	11.1
cis_mRNA_dw20k	513	NA	n385512	NM_012166_dup1	chr9	37505587	37510377	4791	+	chr9	37510889	37576250	4575	-	*		-0.671	-1	5.94	5.95	5.01	4.28	4.02	4.35
cis_mRNA_overlap	0	Lnc-CompleteIn-mRNAIntron	n385675	NM_014334_dup1	chr9	111922746	111923757	1012	-	chr9	111899581	111929571	1189	-	*		-0.9626	-1	0.17	0.38	0.47	16.98	16.14	15.15
cis_mRNA_dw20k	13495	NA	LTCONS_00074393	NM_018969_dup1	chrX	53123291	53156872	4610	+	chrX	53078506	53109797	4252	+	*		0.6776	1	1.83	1.03	1.21	17.37	15.53	17.36
cis_mRNA_overlap	0	Lnc-AntiOverlap-mRNA	n341573	NM_015902_dup1	chr8	103268620	103270049	1430	+	chr8	103264501	103424917	10884	-	*		-0.9982	-1	0.02	0	0.04	13.8	15.43	11.79
cis_mRNA_overlap	0	AntiCompleteIn-mRNAIntron	n325817	NM_016523_dup1	chr12	9980445	9987361	979	-	chr12	9980077	9997604	1239	+	*		0.7037	1	0.04	0.03	0.13	0.17	0.03	0.2
cis_mRNA_dw20k	2733	NA	n374607	NM_007280_dup1	chr15	41576212	41598734	7481	+	chr15	41601466	41624819	1236	-	*		-0.997	-1	0.87	5.12	1.74	0.23	0.05	0.18
cis_mRNA_dw20k	2128	NA	n374607	NM_007236_dup1	chr15	41576212	41598734	7481	+	chr15	41523437	41574085	3230	+	*		0.9868	1	0.87	5.12	1.74	26.65	31.61	28.41
cis_mRNA_up10k	9573	NA	n341171	NM_013393_dup1	chr7	2291405	2294621	3201	-	chr7	2273926	2281833	1601	-	*		-0.994	-1	0.08	0.16	0	12.58	11.65	13.95
cis_mRNA_dw20k	7209	NA	n338918	NM_014044_dup1	chr2	99242186	99347589	1895	-	chr2	99225042	99234978	1143	+	*		0.9363	1	2.56	2.77	3.11	22.18	22.25	24.3
cis_mRNA_up10k	3541	NA	LTCONS_00045862	NM_015565_dup1	chr21	30368817	30375094	5731	-	chr21	30300466	30365277	7756	-	*		0.9932	1	1.53	2.04	1.52	13.3	15.38	12.95
cis_mRNA_dw20k	5016	NA	n408286	NM_014611_dup1	chr6	90341943	90348216	5514	-	chr6	90353231	90529442	17400	-	*		0.9985	1	1.44	2.46	2.19	1.98	3.34	3.05
cis_mRNA_overlap	0	Lnc-AntiOverlap-mRNA	n408286	NM_014942_dup1	chr6	90341943	90348216	5514	-	chr6	90142897	90343403	5190	+	*		0.8618	1	1.44	2.46	2.19	8.42	12.71	9.55
cis_mRNA_dw20k	7949	NA	n377697	NM_018419_dup1	chr20	62669493	62671131	1466	+	chr20	62679079	62680979	1705	-	*		0.9409	1	0.1	0.06	0.21	0.04	0	0.07

cis_mRNA_overlap	0	Lnc-AntiOverlap-mRNA	n408315	NM_016145_dup1	chr19	12777618	12786646	1436	+	chr19	12778881	12780465	861	-	*		0.9997	1	0.69	0.63	1.05	57.67	56.22	64.89
cis_mRNA_dw20k	2070	NA	n373804	NM_015567_dup1	chr13	88333940	88340538	4483	+	chr13	88324870	88331871	4447	+	*		0.9706	1	1.54	1.88	2.15	3.03	3.9	5.55
cis_mRNA_overlap	0	Lnc-CompleteIn-mRNAIntron	n337921	NM_019073_dup1	chr1	48844751	48847025	2275	-	chr1	48761044	48937876	5004	-	*		0.7449	1	0.21	0.48	0.23	1.14	1.49	1.4
cis_mRNA_up10k	6168	NA	n406952	NM_016449_dup1	chr22	23980675	24059610	3416	-	chr22	23950639	23974508	1298	-	*		0.9585	1	3.8	4.75	3.18	0.11	0.17	0
cis_mRNA_up10k	177	NA	n338247	NM_015473_dup1	chr14	31889964	31922089	1213	+	chr14	31760994	31889788	7843	-	*		-0.8	-0.866	0.06	0.03	0.03	2.14	2.69	3.53
cis_mRNA_up10k	1240	NA	LTCONS_00052266	NM_019069_dup1	chr3	122136121	122137344	1224	.	chr3	122130700	122134882	4183	-	*		-0.8351	-0.866	0.34	0.83	0.61	2.71	2.44	2.71
cis_mRNA_up10k	87	NA	LTCONS_00018586	NM_019034_dup1	chr12	122231680	122241988	8559	-	chr12	122215660	122231594	2435	-	*		0.9962	1	4.16	6.52	4.24	1.43	1.98	1.5
cis_mRNA_dw20k	5163	NA	n366298	NM_012430_dup1	chr3	122998557	122999473	579	+	chr3	122920774	122993395	3429	+	*		0.9975	1	0.06	0	0.23	5.77	5.23	6.89
tran	NA	NA	LTCONS_00061918	NM_058182_dup1	chr6	3999741	4019294	6007	-	chr21	35747749	35761452	900	+		-799.46	-0.9522	-1	0.38	0.63	0.5	10.14	7.84	9.68
cis_mRNA_overlap	0	mRNA	n338404	NM_016545_dup1	chr1	181060245	181062035	1791	+	chr1	181057638	181060400	2350	+	*		-0.9741	-0.866	0.21	0.26	0.26	1.47	0.92	1.05
cis_mRNA_overlap	0	mRNA	n409088	NM_014167_dup1	chr12	82746083	82752584	1546	-	chr12	82746083	82752199	1617	-	*		-0.846	-1	1.99	2.58	1.55	4.43	4.37	5.55
cis_mRNA_dw20k	3111	NA	n346026	NM_006651_dup1	chr4	770528	775635	4025	-	chr4	778745	819945	2140	-	*		0.997	1	11.69	14.9	10.71	7.89	9.27	7.63
tran	NA	NA	LTCONS_00028869	NM_015092_dup1	chr17	18601292	18607785	789	+	chr16	18816175	18937726	16065	-		-802	-0.9652	-1	2.9	4.1	6.34	5.2	5.07	3.83
cis_mRNA_dw20k	6019	NA	n369617	NM_014997_dup1	chr7	129781578	129782455	392	+	chr7	129710349	129775560	6438	+	*		0.9997	0.866	0.1	0.09	0.09	10.86	9.41	9.45
cis_mRNA_overlap	0	Lnc-CompleteIn-mRNAIntron	n385272	NM_014390_dup1	chr7	127637562	127640130	2569	+	chr7	127292202	127732659	3508	+	*		0.9231	1	0.15	0.21	0.18	62.14	65.3	62.58
cis_mRNA_dw20k	6754	NA	n342537	NM_144991_dup1	chr21	45905454	45911022	1406	+	chr21	45917775	46131495	3967	-	*		-0.9865	-1	0.17	0.49	0.56	0.06	0.02	0
cis_mRNA_dw20k	6638	NA	LTCONS_00055402	NM_015693_dup1	chr4	128644571	128647776	3206	.	chr4	128554087	128637934	3275	+	*		0.9738	1	1.76	1.96	2.02	3.74	4.19	4.54
cis_mRNA_dw20k	3341	NA	n338916	NM_020184_dup1	chr2	97480968	97482303	902	-	chr2	97426639	97477628	4800	+	*		-0.9731	-1	1.26	0.54	0.98	3.16	4.08	3.32
cis_mRNA_dw20k	8614	NA	n372857	NM_021034_dup1	chr11	309549	311060	878	-	chr11	319673	320914	668	-	*		0.9406	1	0.89	1.02	0.86	0.05	0.1	0
cis_mRNA_dw20k	2684	NA	n368635	NM_013375_dup1	chr6	26602961	26606885	2861	+	chr6	26597171	26600278	2264	+	*		-0.9177	-0.866	0.16	0.22	0.12	11.63	10.08	11.63
cis_mRNA_overlap	0	Lnc-CompleteIn-mRNAIntron	n326388	NM_016334_dup1	chr1	147452927	147453997	905	+	chr1	147400506	147465753	2116	+	*		-0.9754	-1	0.36	0.43	0.26	7.62	6.67	8.24
cis_mRNA_overlap	0	AntiCompleteIn-mRNAExon	n333836	NM_138284_dup1	chr13	21296610	21297237	628	-	chr13	21277482	21297237	1861	+	*		1	0.866	0.29	0.05	0.05	5.57	3.94	3.93
cis_mRNA_dw20k	19436	NA	n384936	NM_015349_dup1	chr6	42855731	42857596	1866	+	chr6	42788794	42836296	6515	+	*		0.8267	1	14.61	16.78	17.3	0	0.4	1.47
cis_mRNA_overlap	0	mRNA	n406580	NM_012416_dup1	chr9	6011019	6015640	3566	-	chr9	6011019	6015618	4600	-	*		1	1	3.58	3.05	5.67	30.55	29.86	33.34
cis_mRNA_dw20k	7036	NA	LTCONS_00001217	NM_016491_dup1	chr1	54691091	54692196	1106	+	chr1	54665840	54684056	1483	+	*		0.99	1	0.03	0.14	0.17	15.13	18.13	19.71
cis_mRNA_dw20k	18654	NA	n364212	NM_013353_dup1	chr1	151120885	151123810	1722	-	chr1	151142463	151148547	1322	-	*		0.9707	0.866	0.48	0.39	0.36	0.24	0.05	0.05
cis_mRNA_up10k	89	NA	n370006	NM_016200_dup1	chr7	117808702	117823998	777	-	chr7	117824086	117844093	12520	+	*		-0.8374	-0.866	0	0.04	0.04	7.59	6.54	5.27
cis_mRNA_dw20k	3345	NA	n345144	NM_002689_dup1	chr11	65068432	65076128	1757	-	chr11	65029432	65065088	2477	+	*		1	1	0.04	0.18	0.11	1.08	1.67	1.37
cis_mRNA_dw20k	6635	NA	n339464	NM_018944_dup1	chr21	33632115	33633896	1782	-	chr21	33640530	33651376	1571	-	*		0.9982	0.866	0.04	0.04	0.02	2.64	2.65	2.5
cis_mRNA_overlap	0	mRNA	LTCONS_00031497	NM_016125_dup1	chr17	58029650	58036835	2334	-	chr17	58029723	58042117	2016	-		-735.51	-0.7519	-0.866	1.39	2	1.59	2.07	2.01	2.01
cis_mRNA_up10k	4863	NA	n372447	NM_006656_dup1	chr11	74691592	74695088	1588	+	chr11	74699950	74718743	2748	+	*		-0.9971	-1	1.44	1.41	1.13	4.88	4.89	5.63
cis_mRNA_dw20k	14588	NA	n342001	NM_015687_dup1	chr6	76001574	76003213	1638	-	chr6	76017800	76203496	4622	-	*		0.7107	0.866	0.08	0.08	0.06	0.03	0.11	0
cis_mRNA_up10k	325	NA	n407036	NM_006808_dup1	chr9	101978707	101984246	2989	-	chr9	101984570	101992901	562	+	*		0.9191	1	0.37	0.3	0.48	49.87	46.66	50.96
cis_mRNA_dw20k	14485	NA	n407463	NM_030758_dup1	chr22	31318295	31322640	2133	+	chr22	31090793	31303811	4340	+	*		0.8696	1	0.06	0.09	0.07	3.4	4.27	4.09
cis_mRNA_dw20k	1052	NA	n370538	NM_015420_dup1	chr8	104456732	104457916	969	-	chr8	104426942	104455681	2642	+	*		-0.9942	-1	0.07	0.13	0	16.47	15.01	18.99
cis_mRNA_up10k	6692	NA	n383917	NM_016470_dup1	chr20	42846122	42852330	4103	+	chr20	42825136	42839431	1572	-	*		-0.7708	-0.866	0.18	0.18	0.22	16.45	14.88	14.02
cis_mRNA_dw20k	13059	NA	n406654	NM_014149_dup1	chr7	134850532	134855532	1862	-	chr7	134868590	134896316	4619	-	*		-0.9184	-1	1.29	1.01	1.1	3.73	4.29	3.91
cis_mRNA_up10k	345	NA	n375319	NM_016095_dup1	chr16	85722932	85733297	1507	-	chr16	85711280	85722588	1180	-	*		-0.8546	-0.866	0.02	0.02	0.08	3.23	2.43	1.69
cis_mRNA_up10k	462	NA	n378461	NM_016470_dup1	chr20	42839892	42853618	695	+	chr20	42825136	42839431	1572	-	*		-0.8482	-1	0.29	0.3	0.36	16.45	14.88	14.02
cis_mRNA_dw20k	2448	NA	n341810	NM_018998_dup1	chr9	139831621	139832440	820	-	chr9	139834887	139839173	2345	-	*		0.9203	1	0.61	0.52	0.74	24.24	24.18	27.24
cis_mRNA_dw20k	2343	NA	n379236	NM_016113_dup1	chr17	16342659	16345056	821	+	chr17	16318856	16340317	2808	+	*		-0.9574	-1	8.7	6.58	4.98	1.4	2.29	4.24
cis_mRNA_dw20k	17877	NA	n405259	NM_015669_dup1	chr5	140535580	140537990	2411	+	chr5	140514800	140517704	2905	+	*		0.9893	1	0.27	0.19	0.22	19.98	4.22	8.03
cis_mRNA_overlap	0	Lnc-CompleteIn-mRNAIntron	n338760	NM_016441_dup1	chr2	36750870	36751847	978	+	chr2	36583370	36778278	5611	+	*		0.9998	1	0.43	0.63	1.45	21.91	28.35	52.12
tran	NA	NA	LTCONS_00059446	NM_006087_dup1	chr6	3154674	3177868	476	+	chr19	6494330	6502330	2287	-		-955.8	0.9347	1	0	0.41	0.34	19.99	20.53	20.26
cis_mRNA_dw20k	6278	NA	n374165	NM_015351_dup1	chr14	71148354	71166840	493	+	chr14	71108504	71142077	5217	+	*		-0.9466	-0.866	0.23	0.14	0.14	9.77	10.75	11.23
cis_mRNA_up10k	200	NA	n376604	NM_012384_dup1	chr20	62258580	62260177	801	+	chr20	62218955	62258381	4210	-	*		-0.99	-1	2.5	1.29	2.87	2.75	2.82	2.71
cis_mRNA_dw20k	10885	NA	n376604	NM_015894_dup1	chr20	62258580	62260177	801	+	chr20	62271061	62284780	2255	-	*		0.7833	1	2.5	1.29	2.87	190.09	181.92	232.64
cis_mRNA_dw20k	5550	NA	n346040	NM_015983_dup1	chr7	44001284	44027449	960	-	chr7	43966035	43995735	3982	+	*		-0.8123	-1	0.05	0.29	0	3.47	3.29	3.96
cis_mRNA_up10k	134	NA	n386706	NM_015987_dup1	chr12	13153376	13157762	4387	+	chr12	13127798	13153243	1209	-	*		0.9442	1	0.6	0.74	0.46	0.67	1.4	0.49
cis_mRNA_overlap	0	AntiCompleteIn-mRNAIntron	n341280	NM_014916_dup1	chr7	97752600	97755814	3214	-	chr7	97736197	97838945	8946	+	*		0.7185	0.866	0.51	0.62	0.62	8.49	8.64	9.02
cis_mRNA_dw20k	15393	NA	n382962	NM_006110_dup1	chr16	30278914	30346695	4300	-	chr16	30362087	30366682	3421	-	*		0.9793	1	3.73	3.69	5.17	36.48	34.49	42.93
cis_mRNA_dw20k	1642	NA	n345457	NM_006699_dup1	chr1	118069961	118071491	1531	+	chr1	117910085	118068320	5388	+	*		0.957	1	10.52	11.67	11.8	17.43	19.3	20.45
cis_mRNA_dw20k	5269	NA	n377214	NM_015565_dup1	chr21	30291160	30295198	1132	-	chr21	30300466	30365277	7756	-	*		0.6109	0.866	0.28	0.28	0.25	13.3	15.38	12.95
cis_mRNA_dw20k	167	NA	n341826	NM_016059_dup1	chr6	36819837	36822439	2603	-	chr6	36822605	36842800	1737	-	*		-0.9978	-1	0.68	0.63	0.89	37.78	38.33	33.95
cis_mRNA_overlap	0	Lnc-CompleteIn-mRNAIntron	n325364	NM_014971_dup1	chr2	25280686	25285575	466	+	chr2	25264973	25382004	7458	+	*		-0.9835	-1	0.08	0	0.14	67.83	76.92	64.54
cis_mRNA_overlap	0	mRNA	n377748	NM_019034_dup1	chr12	121633051	122226808	1385	-	chr12	122215660	122231594	2435	-	*		0.9866	1	0.22	0.29	0.24	1.43	1.98	1.5
cis_mRNA_dw20k	15091	NA	n375386	NM_012213_dup1	chr16	83964877	83967981	2279	+	chr16	83932730	83949787	2195	+	*		0.8168	1	0.11	0.04	0.09	5.92	5.24	5.35
cis_mRNA_up10k	9648	NA	n375457	NM_016538_dup1	chr17	79885705	79888628	1894	+	chr17	79869815	79876058	1731	-	*		0.8914	1	1.42	1.26	1.56	3.84	2.67	3.86
cis_mRNA_up10k	6185	NA	n345589	NM_015512_dup1	chr3	52341571	52344151	691	+	chr3	52350335	52434513	13110	+	*		-0.8949	-1	0.31	0	0.23	0.67	1	0.89
cis_mRNA_dw20k	19292	NA	n336301	NM_007375_dup1	chr1	11104840	11107151	682	-	chr1	11072679	11085549	4217	+	*		0.8122	1	0	0.1	0.09	7.07	7.76	7.3
cis_mRNA_overlap	0	Lnc-AntiOverlap-mRNA	n377948	NM_001145418_dup1	chr22	28331149	28395645	627	+	chr22	28374002	29075853	11795	-	*		-0.8744	-1	0	0.04	0.14	10.67	9.4	8.94
cis_mRNA_dw20k	5003	NA	n369512	NM_007342_dup1	chr7	23245632	23247664	1999	+	chr7	23221446	23240630	1797	+	*		-1	-1	0.02	0.09	0.28	18.85	16.88	11.29
cis_mRNA_overlap	0	Lnc-CompleteIn-mRNAExon	n335611	NM_012455_dup1	chr2	113960078	113960494	417	+	chr2	113931560	113960677	5039	+	*		0.7074	1	0	0.27	0.31	0.13	0.15	0.26

cis_mRNA_overlap	0	Lnc-AntiOverlap-mRNA	n341554	NM_007013_dup1	chr8	87345503	87355543	1486	-	chr8	87354994	87480181	4108	+	*		-0.9924	-1	0.14	0.11	0.12	7.03	7.55	7.32
cis_mRNA_overlap	0	Lnc-AntiOverlap-mRNA	n408278	NM_015541_dup1	chr3	66271168	66429351	2226	+	chr3	66429221	66550845	4762	-	*		-0.9029	-1	0.3	0.24	0.65	2.98	3.65	2.43
cis_mRNA_overlap	0	CompleteIn-LncExon	n408049	NM_006554_dup1	chr2	177134123	177202753	1592	+	chr2	177134123	177202753	1388	+	*		0.8812	1	2.42	1.9	1.79	56.66	52.58	46.04
cis_mRNA_up10k	6542	NA	n369908	NM_013321_dup1	chr7	2360640	2393863	407	-	chr7	2291405	2354099	4727	-	*		1	1	0	0.34	0.17	2.34	2.42	2.38
cis_mRNA_dw20k	13851	NA	n345336	NM_012091_dup1	chr16	75614134	75618397	879	+	chr16	75632247	75657221	3545	-	*		-0.8012	-0.866	0.04	0.07	0.04	3.83	3.52	4.3
cis_mRNA_up10k	488	NA	n369881	NM_007349_dup1	chr7	154795169	154797375	1954	+	chr7	154735400	154794682	3712	-	*		0.9997	1	0.35	0.12	0.24	1.31	1.13	1.22
cis_mRNA_overlap	0	mRNA	n338711	NM_014166_dup1	chr13	48627461	48669198	950	-	chr13	48650092	48669240	2022	-	*		-0.9933	-1	7.82	8.42	5.69	20.34	19.64	21.78
cis_mRNA_dw20k	4146	NA	LTCONS_00043220	NM_025176_dup1	chr20	25388323	25429192	3225	+	chr20	25433337	25566153	4973	-	*		0.9453	1	0.41	0.7	0.11	2.03	3.53	1.63
cis_mRNA_up10k	502	NA	n380278	NM_014488_dup1	chr11	82783385	82784642	658	+	chr11	82692478	82782884	1602	-	*		-0.9777	-1	0.8	0.54	0.35	1.91	3.34	3.77
cis_mRNA_overlap	0	Lnc-CompleteIn-mRNAIntron	n341079	NM_001098816_dup1	chr11	79059322	79060897	1576	-	chr11	78364328	79151695	13548	-	*		0.9961	1	0	0.12	0.06	0.01	2.99	1.27
cis_mRNA_up10k	174	NA	n379000	NM_012399_dup1	chr22	28315428	28382330	837	+	chr22	28247657	28315255	2925	-	*		-0.7947	-1	0.2	0.11	0.64	12.68	14.11	12.22
cis_mRNA_up10k	7224	NA	n372872	NM_006819_dup1	chr11	63944169	63946364	607	-	chr11	63953587	63972020	2201	+	*		-0.7479	-1	0.18	0.77	0.32	80.4	77.34	77.65
cis_mRNA_overlap	0	Lnc-CompleteIn-mRNAIntron	n381381	NM_014023_dup1	chr10	1136095	1138119	2025	+	chr10	1102776	1178237	4611	+	*		0.8359	0.866	0.25	0.36	0.25	11.15	12.19	9.88
cis_mRNA_overlap	0	mRNA	n340697	NM_015315_dup1	chr5	154135135	154183768	2630	+	chr5	154092462	154197165	6593	+	*		0.8608	1	2.07	3	4.01	2.74	8.51	8.6
cis_mRNA_up10k	157	NA	n338432	NM_013231_dup1	chr14	85991477	85996332	1747	-	chr14	85996488	86094270	7179	+	*		0.9999	1	0.38	0.81	0.35	2.11	4.79	1.87
cis_mRNA_up10k	6409	NA	n409173	NM_007342_dup1	chr7	23145353	23215038	3306	+	chr7	23221446	23240630	1797	+	*		-1	-1	4.65	5.12	6.47	18.85	16.88	11.29
cis_mRNA_up10k	9539	NA	n333749	NM_006453_dup1	chr16	2012062	2012526	238	+	chr16	2022064	2028751	2594	+	*		-0.9429	-1	0.54	0	0.32	10.86	13.17	12.49
cis_mRNA_up10k	7315	NA	n381883	NM_007235_dup1	chr12	64616117	64790839	1360	+	chr12	64798153	64842464	3948	+	*		0.9599	1	0.05	0.09	0.07	25.81	31.92	27.32
cis_mRNA_dw20k	2740	NA	n374608	NM_007280_dup1	chr15	41578132	41598727	8656	+	chr15	41601466	41624819	1236	-	*		0.9194	1	10.33	5.39	7.2	0.23	0.05	0.18
cis_mRNA_dw20k	4048	NA	n374608	NM_007236_dup1	chr15	41578132	41598727	8656	+	chr15	41523437	41574085	3230	+	*		-0.9495	-1	10.33	5.39	7.2	26.65	31.61	28.41
cis_mRNA_up10k	143	NA	LTCONS_00043479	NM_016470_dup1	chr20	42839573	42854809	1494	+	chr20	42825136	42839431	1572	-	*		-0.9602	-1	1.11	1.56	1.6	16.45	14.88	14.02
cis_mRNA_overlap	0	CompleteIn-LncExon	n410036	NM_001002909_dup1	chr17	42472645	42580957	7037	-	chr17	42472645	42580957	7019	-	*		0.8531	1	0.74	1.09	1.03	5.14	6.09	5.46
cis_mRNA_dw20k	6070	NA	n338158	NM_025225_dup1	chr22	44349517	44351200	1684	-	chr22	44319619	44343448	2805	+	*		0.8916	1	0.35	0.37	0.18	9.27	12.06	6.86
cis_mRNA_dw20k	2652	NA	n325800	NM_022459_dup1	chr13	21344909	21348817	1623	-	chr13	21351468	21476913	9885	-	*		-0.9989	-1	0.09	0.04	0.07	4.58	5.35	4.92
cis_mRNA_overlap	0	Lnc-CompleteIn-mRNAIntron	n383815	NM_025243_dup1	chr2	228573101	228575446	2346	-	chr2	228549926	228582745	3767	-	*		-0.8998	-0.866	0.03	0.01	0.03	0.32	0.41	0.25
cis_mRNA_dw20k	743	NA	n338963	NM_030923_dup1	chr2	135206960	135212192	5231	+	chr2	135212934	135476571	1901	-	*		0.9805	1	7.93	9.09	7.51	0.13	0.4	0.11
cis_mRNA_up10k	266	NA	LTCONS_00030148	NM_024792_dup1	chr17	624454	635582	1311	-	chr17	635847	646075	2080	+	*		0.9786	1	1.28	1.43	0.92	2.25	2.46	2.02
cis_mRNA_overlap	0	mRNA-AntiCompleteIn-LncIntron	n408109	NM_024070_dup1	chr7	99798278	99869855	3550	-	chr7	99816871	99819111	1569	+	*		-0.6437	-0.866	1.62	1.99	0	0.02	0	0.02
cis_mRNA_dw20k	546	NA	n406459	NM_020191_dup1	chr3	139076433	139108522	3202	-	chr3	139062861	139075888	1123	+	*		0.9482	1	2.33	3.6	3.93	18	18.76	19.51
cis_mRNA_dw20k	2842	NA	LTCONS_00025597	NM_022566_dup1	chr15	81299186	81302795	3610	.	chr15	81293295	81296345	3051	+	*		0.9735	1	1.65	1.22	1.45	12.02	9.4	11.34
cis_mRNA_overlap	0	Lnc-CompleteIn-mRNAIntron	n338959	NM_022103_dup1	chr19	56979088	56987270	3593	-	chr19	56950693	56988770	3860	-	*		0.9952	1	0.47	0.26	0	2.58	1.65	0
cis_mRNA_dw20k	11720	NA	n372358	NM_020848_dup1	chr10	30280163	30290010	2153	-	chr10	30301729	30348488	9300	-	*		0.737	0.866	0	0.03	0.03	0.18	0.31	0.22
cis_mRNA_overlap	0	Lnc-AntiOverlap-mRNA	LTCONS_00050221	NM_020873_dup1	chr3	3742498	3864550	2302	-	chr3	3841121	3889387	3823	+	*		0.8784	1	0.76	0.56	0.23	1.23	0.67	0.55
cis_mRNA_up10k	2076	NA	n335557	NM_024077_dup1	chr9	91926209	91931337	234	+	chr9	91933412	91974561	3467	+	*		-0.9987	-1	0.09	0	0.12	14.71	15.09	14.55
cis_mRNA_dw20k	1130	NA	LTCONS_00054345	NM_020722_dup1	chr4	57198019	57211157	9096	-	chr4	57036361	57196890	7013	+	*		-0.8233	-1	10.98	12.72	12.26	4.19	0	3.37
cis_mRNA_up10k	4778	NA	n382899	NM_022773_dup1	chr16	1025761	1030913	3222	-	chr16	903634	1020984	2606	-	*		0.7471	1	5.71	7.1	7.27	2.4	2.76	3.97
tran	NA	NA	LTCONS_00048126	NM_023075_dup1	chr22	22239728	22240218	491	.	chr18	11883470	11908641	2806	-		-984.1	0.6805	0.866	0.3	0.07	0	3.93	3.93	3.4
cis_mRNA_overlap	0	Lnc-AntiOverlap-mRNA	n410951	NM_017763_dup1	chr17	56406966	56431088	2201	+	chr17	56431037	56494931	4558	-	*		0.6724	1	0.29	0	0.31	0.01	0	0.06
cis_mRNA_up10k	4364	NA	n381086	NM_022778_dup1	chr1	26551811	26556330	1077	+	chr1	26560693	26605299	3935	+	*		-0.9956	-1	0.16	0.09	0.44	9.87	10.28	8.86
cis_mRNA_up10k	352	NA	LTCONS_00059149	NM_024594_dup1	chr5	168006965	168007787	823	.	chr5	167982628	168006614	3387	-	*		0.9849	1	0.52	0.91	0.39	82.17	95.93	72.73
cis_mRNA_up10k	1213	NA	n369044	NM_033502_dup1	chr6	42420213	42423732	354	+	chr6	42192669	42419001	7557	-	*		-0.9678	-1	1.11	1.61	1.19	0.57	0.09	0.38
cis_mRNA_up10k	569	NA	n365511	NM_024093_dup1	chr2	105950392	105953445	1853	-	chr2	105954013	105961984	887	+	*		0.9468	1	0.17	0.62	0.42	10.9	13.29	12.95
cis_mRNA_dw20k	2767	NA	n339357	NM_022064_dup1	chr3	49761728	49823960	4455	-	chr3	49726990	49758962	4255	+	*		-0.7002	-1	2.6	0.35	0.39	6.67	8.43	7.1
cis_mRNA_dw20k	3630	NA	n339616	NM_022131_dup1	chr3	140290548	140294071	3524	+	chr3	139654027	140286919	4882	+	*		0.9942	1	17.22	22.28	22.73	19.86	24.59	25.71
cis_mRNA_up10k	7007	NA	n338225	NM_017999_dup1	chr14	24608654	24609653	741	+	chr14	24616659	24629870	3556	+	*		0.9333	1	0.05	0.09	0.04	8.2	8.52	7.84
cis_mRNA_overlap	0	mRNA	n407074	NM_024735_dup1	chr16	87362942	87425708	3364	-	chr16	87362942	87417394	3616	-	*		-0.7893	-1	0	0.79	2.32	11.6	9.9	9.81
cis_mRNA_up10k	568	NA	LTCONS_00007591	NM_024674_dup1	chr1	26736004	26736702	699	.	chr1	26737269	26756219	4014	+	*		0.8263	1	1.34	1.65	0.69	2.42	5.56	1.92
cis_mRNA_dw20k	3609	NA	n363491	NM_018122_dup1	chr1	173831290	173833079	853	+	chr1	173793797	173827682	3348	+	*		-0.8055	-1	0.43	0.58	0.38	4.13	3.98	4.81
cis_mRNA_up10k	5637	NA	n407976	NM_025204_dup1	chr22	50609160	50618724	3018	+	chr22	50624360	50638027	2315	+	*		-0.9171	-1	0	0.14	0.29	3.27	3.2	2.59
cis_mRNA_dw20k	6314	NA	n409085	NM_017669_dup1	chrX	71401526	71418194	1250	+	chrX	71424507	71458858	4207	-	*		0.6628	0.866	0.75	0.95	0	0.01	0.03	0.01
cis_mRNA_dw20k	13937	NA	n370073	NM_022906_dup1	chr7	66800449	66805315	789	+	chr7	66767616	66786513	2135	+	*		0.9437	1	0.22	0.1	0.44	9.37	7.42	10.43
cis_mRNA_overlap	0	CompleteIn-LncExon	n406655	NM_024033_dup1	chr7	134850532	134855532	1609	-	chr7	134850532	134855532	1572	-	*		0.972	1	2.62	2.84	3.23	0.67	0.87	2.13
cis_mRNA_dw20k	6886	NA	n342463	NM_025216_dup1	chr2	219765536	219795358	1008	-	chr2	219745255	219758651	2375	+	*		-0.8773	-1	0.07	0	0.09	0.03	0.04	0.01
cis_mRNA_up10k	946	NA	LTCONS_00067909	NM_018396_dup1	chr7	128114894	128115838	945	.	chr7	128116783	128142978	2182	+	*		-0.9338	-1	0.75	0.92	0.3	6.98	6.64	7.21
cis_mRNA_dw20k	472	NA	n378802	NM_017971_dup1	chr1	1334902	1336805	1135	+	chr1	1337276	1342693	733	-	*		0.6898	1	1.07	0	0.13	54.11	48.39	53.33
cis_mRNA_up10k	4970	NA	n409151	NM_030973_dup1	chr19	50310124	50316567	1660	-	chr19	50321536	50340237	2343	+	*		-0.9197	-1	0.37	0	0.16	3.64	4.69	4.64
cis_mRNA_dw20k	3548	NA	n345507	NM_001031716_dup1	chr2	192556795	192583257	5397	+	chr2	192542861	192553248	3849	+	*		-0.9934	-0.866	0.03	0.03	0.01	0.62	0.64	0.78
cis_mRNA_dw20k	1384	NA	n340240	NM_020724_dup1	chr4	141782330	141785342	1813	-	chr4	141786725	142054616	4661	-	*		0.9755	1	10.78	13.09	13.59	11.76	14.37	15.99
cis_mRNA_overlap	0	Lnc-AntiOverlap-mRNA	n340244	NM_022475_dup1	chr4	145564074	145567174	1129	-	chr4	145567148	145659881	3555	+	*		-0.8833	-1	0.44	0	0.3	0.06	0.1	0.09

cis_mRNA_up10k	1644	NA	n369238	NM_018214_dup1	chr6	53610490	53658135	1072	-	chr6	53659778	53788919	3180	+	*	0.9109	1	0.7	1.26	1.04	2.35	3.22	2.55
cis_mRNA_overlap	0	Lnc-CompleteIn-mRNAIntron	n340037	NM_001080512_dup1	chr10	60273109	60274558	1450	+	chr10	60272904	60588845	3119	+	*	0.8608	1	0.02	0.09	0.11	0.41	0.51	0.74
cis_mRNA_overlap	0	mRNA	n332366	NM_030973_dup1	chr19	50339125	50339784	379	+	chr19	50321536	50340237	2343	+	*	0.9816	1	0.39	0.72	0.64	3.64	4.69	4.64
cis_mRNA_up10k	8883	NA	n382914	NM_022468_dup1	chr16	3087252	3087800	549	-	chr16	3096682	3110727	3554	+	*	0.9901	1	0.07	0.06	0.24	0.06	0.05	0.1
cis_mRNA_overlap	0	Lnc-CompleteIn-mRNAExon	n406622	NM_018320_dup1	chr11	71639768	71708643	2568	+	chr11	71639768	71708643	2606	+	*	-0.9996	-1	1.29	2.78	1.14	4.27	3.17	4.42
cis_mRNA_dw20k	3432	NA	n367209	NM_021227_dup1	chr4	109592411	109602985	4819	+	chr4	109571741	109588980	1080	+	*	0.9676	1	0.18	0.24	0.31	48.93	49.91	50.36
cis_mRNA_up10k	9472	NA	LTCONS_00025605	NM_022767_dup1	chr15	89154721	89155056	336	.	chr15	89164527	89175512	3116	+	*	-0.8658	-1	0.47	2.46	0.42	3.01	2.12	3.97
cis_mRNA_dw20k	459	NA	n386409	NM_024493_dup1	chr6	28334982	28336930	1117	+	chr6	28317691	28334524	2295	+	*	-0.9998	-1	2.86	2.46	2.02	6.66	7.46	8.4
cis_mRNA_overlap	0	Lnc-AntiOverlap-mRNA	n408346	NM_020142_dup1	chr12	57623356	57628718	2215	+	chr12	57628686	57631121	1177	-	*	0.8771	1	0.11	0.92	0.45	0.57	0.97	0.92
tran	NA	NA	LTCONS_00055968	NM_024646_dup1	chr5	60452705	60453538	834	+	chr1	53192131	53293014	8143	+	-709.5	-0.9394	-1	2.23	1.83	1.61	26.29	27.31	29.3
cis_mRNA_dw20k	6325	NA	n379814	NM_024541_dup1	chr10	103596053	103599032	2272	+	chr10	103605356	103815932	4101	-	*	-0.9893	-1	0.04	0.11	0.16	3.6	3.39	3.14
cis_mRNA_dw20k	7374	NA	n341837	NM_021943_dup1	chr6	38129773	38132113	1839	+	chr6	37787307	38122400	3334	+	*	0.9917	1	0.04	0.1	0.13	6.24	6.46	6.52
cis_mRNA_up10k	4868	NA	n384964	NM_030568_dup1	chr6	73977774	74001059	3358	+	chr6	73951037	73972907	1134	-	*	-0.9877	-1	1.56	1.85	1.79	2.01	1.3	1.55
cis_mRNA_overlap	0	CompleteIn-LncExon	n410461	NM_022100_dup1	chr1	174982094	174992591	2237	-	chr1	174982094	174992591	2145	-	*	-0.6912	-0.866	0.28	0.28	1.36	9.69	11.7	9.03
cis_mRNA_up10k	3575	NA	n411745	NM_024579_dup1	chr1	150237799	150241609	2130	-	chr1	150245183	150253335	527	+	*	-0.7213	-1	0.56	0.49	1.36	0.07	0.32	0
cis_mRNA_dw20k	6012	NA	n325867	NM_018117_dup1	chr10	122675049	122730203	943	-	chr10	122610687	122669038	4734	+	*	-0.6024	-1	58.79	77.67	79.58	16.69	16.51	12.68
cis_mRNA_overlap	0	Lnc-AntiOverlap-mRNA	n383026	NM_020655_dup1	chr16	87731749	87739290	6938	-	chr16	87636499	87731762	3997	+	*	-1	-1	0.06	0.08	0.03	25.45	23.37	28.48
cis_mRNA_dw20k	7898	NA	n341087	NM_021825_dup1	chr11	82962569	82964609	2041	-	chr11	82972506	82997377	1574	-	*	0.8286	1	1.09	1.78	2.01	30.83	31.59	34.29
cis_mRNA_overlap	0	Lnc-AntiOverlap-mRNA	LTCONS_00035120	NM_025027_dup1	chr19	58513729	58522652	7554	+	chr19	58488440	58514714	4226	-	*	0.9899	1	2.71	2.65	2.61	5.96	5.66	5.33
cis_mRNA_overlap	0	mRNA-AntiCompleteIn-LncIntron	n379282	NM_020685_dup1	chr3	62249717	62355005	502	-	chr3	62305396	62319321	768	+	*	0.9936	1	0.23	0	0.16	45.18	35.65	41.32
cis_mRNA_dw20k	11209	NA	LTCONS_00037116	NM_021216_dup1	chr19	57146758	57147171	414	.	chr19	57106664	57135550	3133	+	*	0.999	1	1.76	1.26	0.83	19.6	13.8	7.98
cis_mRNA_overlap	0	Lnc-CompleteIn-mRNAIntron	n340026	NM_021226_dup1	chr10	49717904	49719721	1818	-	chr10	49654079	49813138	2749	-	*	0.8844	0.866	0.06	0.17	0.17	8.31	11.46	14.22
cis_mRNA_dw20k	7923	NA	n376436	NM_017703_dup1	chr19	9903552	9913021	1197	+	chr19	9920943	9929731	1852	-	*	-0.6286	-0.866	0.15	0.16	0.16	5.74	5.67	5.32
cis_mRNA_dw20k	15366	NA	n337634	NM_017891_dup1	chr1	995114	1001833	974	+	chr1	1017198	1051736	2104	-	*	-0.9973	-1	1.85	2.08	1.43	3.15	2.88	3.82
cis_mRNA_overlap	0	Lnc-CompleteIn-mRNAExon	n338468	NM_018036_dup1	chr14	96792025	96829678	2763	-	chr14	96747595	96829678	11099	-	*	-0.924	-1	2.63	1.91	2.01	4.95	7.18	6.07
cis_mRNA_up10k	5814	NA	n363595	NM_020317_dup1	chr1	25579798	25594376	777	-	chr1	25568740	25573985	1465	-	*	-0.8918	-1	0.18	0.49	0.51	1.64	1.41	1.18
cis_mRNA_up10k	841	NA	LTCONS_00073020	NM_017561_dup1	chr9	97091766	97094200	2435	-	chr9	97080478	97090926	2561	-	*	0.8223	1	2.25	1.31	0.66	0.51	0.5	0.13
cis_mRNA_dw20k	11400	NA	n370125	NM_022373_dup1	chr7	35654455	35660870	815	-	chr7	35672269	35734772	2911	-	*	-0.961	-1	0.41	0.19	0.17	5.35	5.57	5.69
cis_mRNA_dw20k	4917	NA	n377676	NM_024821_dup1	chr22	42227219	42230669	2989	-	chr22	42196678	42222303	1275	+	*	-0.799	-1	0.26	0.34	0.13	3.04	2.47	3.05
cis_mRNA_dw20k	4160	NA	LTCONS_00045034	NM_024325_dup1	chr20	2456071	2458304	2234	.	chr20	2462463	2489778	3632	-	*	0.7643	1	0.05	0	0.01	5.06	4.82	5.02
cis_mRNA_dw20k	15492	NA	n370201	NM_018440_dup1	chr8	81806189	81864554	1789	+	chr8	81880045	82024303	10744	-	*	0.9495	1	0.25	0.28	0.17	5.1	6.79	3.74
cis_mRNA_up10k	155	NA	n364340	NM_020883_dup1	chr1	45672404	45683543	320	+	chr1	45482074	45672250	5838	-	*	0.9633	1	0	0.34	0.22	12.36	14.31	14.13
cis_mRNA_dw20k	2655	NA	n384770	NM_024594_dup1	chr5	167975500	167979974	4475	-	chr5	167982628	168006614	3387	-	*	0.999	1	63.46	73.15	55.68	82.17	95.93	72.73
cis_mRNA_dw20k	82	NA	LTCONS_00015230	NM_018088_dup1	chr12	8332805	8373775	4572	+	chr12	8373856	8380214	2516	-	*	-0.9796	-1	8.1	4.93	7.04	0	0.07	0.01
cis_mRNA_up10k	386	NA	LTCONS_00059106	NM_024337_dup1	chr5	3595295	3595783	489	.	chr5	3596168	3601517	1858	+	*	0.857	1	0.15	0	0.41	46.88	39.41	48.16
cis_mRNA_dw20k	16876	NA	n386033	NM_018088_dup1	chr12	8332805	8356981	1182	+	chr12	8373856	8380214	2516	-	*	0.8928	1	0.58	1.11	0.88	0	0.07	0.01
cis_mRNA_overlap	0	CompleteIn-LncExon	LTCONS_00051731	NM_020307_dup1	chr3	156862626	156881750	17150	-	chr3	156865585	156878482	2625	-	-1369.83	0.8739	1	2.81	3.23	2.48	8.22	8.39	6.58
cis_mRNA_up10k	6369	NA	LTCONS_00006171	NM_017582_dup1	chr1	154537488	154538658	1171	-	chr1	154521051	154531120	3223	-	*	-0.988	-1	1.47	1.09	0.77	25.59	26.79	28.53
cis_mRNA_dw20k	2337	NA	n335453	NM_022830_dup1	chr11	62334412	62340181	673	-	chr11	62342517	62359109	2826	-	*	-0.9999	-1	0.28	0.11	0.26	6.66	8.39	6.89
cis_mRNA_up10k	29	NA	LTCONS_00037085	NM_024074_dup1	chr19	16771071	16771910	840	.	chr19	16771938	16799830	1639	+	*	0.985	1	0.93	1.35	1.02	3.78	5.47	3.84
cis_mRNA_up10k	104	NA	LTCONS_00037085	NM_024104_dup1	chr19	16771071	16771910	840	.	chr19	16756472	16770968	1390	-	*	0.8324	1	0.93	1.35	1.02	18.16	18.81	18.64
cis_mRNA_dw20k	1825	NA	n340727	NM_020223_dup1	chr7	302535	303422	516	+	chr7	192969	300711	2751	+	*	0.9959	1	0.65	0.79	1.22	18.96	19.59	22.94
cis_mRNA_dw20k	14183	NA	n375684	NM_020653_dup1	chr17	16438939	16439449	385	-	chr17	16453631	16472520	4278	-	*	-0.737	-0.866	0	0.09	0.09	6.03	5.95	5.77
cis_mRNA_dw20k	3725	NA	LTCONS_00063792	NM_031469_dup1	chr6	80417093	80418900	1808	.	chr6	80341000	80413369	4646	+	*	0.9832	1	0.78	0.75	0.88	63.83	57.82	72.94
cis_mRNA_dw20k	4047	NA	n344955	NM_030796_dup1	chr7	55532613	55534260	1469	-	chr7	55538306	55640200	2944	-	*	0.6988	0.866	0.61	1.12	0.61	13.95	15.31	10.02
cis_mRNA_dw20k	4126	NA	n342116	NM_020755_dup1	chr6	122757934	122760368	2433	-	chr6	122764493	122792952	3128	-	*	0.6135	1	8.08	7.81	7.82	557	540.59	555.13
cis_mRNA_overlap	0	mRNA	n340194	NM_025212_dup1	chr4	105390671	105416058	3130	-	chr4	105393330	105412467	761	-	*	0.8569	1	7.47	9.1	7.99	13.9	16.88	16.29
cis_mRNA_dw20k	13784	NA	LTCONS_00064960	NM_022906_dup1	chr7	66800296	66823124	3917	+	chr7	66767616	66786513	2135	+	*	-0.9378	-0.866	0.49	0.61	0.49	9.37	7.42	10.43
cis_mRNA_up10k	8746	NA	LTCONS_00002595	NM_030916_dup1	chr1	161068130	161070194	1540	+	chr1	161040781	161059385	3502	-	*	-0.866	-0.866	1.3	2.88	2.09	0.01	0	0.01
cis_mRNA_overlap	0	mRNA	LTCONS_00069457	NM_025232_dup1	chr8	21995533	21996852	1102	-	chr8	21995533	21999448	1694	-	-851.56	0.9982	1	0	0.09	0.57	0.51	0.59	1.34
cis_mRNA_overlap	0	Lnc-CompleteIn-mRNAIntron	n341414	NM_017884_dup1	chr8	10660588	10662320	1733	-	chr8	10622884	10697299	1047	-	*	-0.6926	-1	0.1	0.09	0.14	2.02	2.51	1.99
cis_mRNA_dw20k	2015	NA	n378464	NM_024734_dup1	chr14	95624097	95646262	723	+	chr14	95648276	95786245	12750	-	*	0.9285	1	0.11	0.64	0.45	0.23	0.36	0.27
cis_mRNA_overlap	0	mRNA	n407939	NM_024063_dup1	chr15	45694519	45713616	2609	+	chr15	45694519	45713617	2580	+	*	0.9889	0.866	0.97	1.08	1.66	3.08	3.08	3.75
cis_mRNA_up10k	424	NA	n377667	NM_021035_dup1	chr20	47895179	47905797	504	+	chr20	47862437	47894756	7371	-	*	-0.8465	-1	4	2.66	0	3.56	4.29	4.43
cis_mRNA_up10k	1062	NA	n386327	NM_001080517_dup1	chr3	9433389	9438342	2606	-	chr3	9439403	9519838	6827	+	*	0.7778	1	1.29	1.98	2.11	4.63	5.05	6.47
cis_mRNA_overlap	0	Lnc-AntiOverlap-mRNA	n407565	NM_018489_dup1	chr1	155531772	155533735	1964	+	chr1	155305052	155532324	11774	-	*	0.9853	1	1.28	1.71	1.4	9.49	10.74	9.63
cis_mRNA_overlap	0	mRNA	LTCONS_00026293	NM_024031_dup1	chr16	30662180	30663801	1495	+	chr16	30662241	30667734	2118	+	*	0.6852	1	0.75	0.8	0.74	2.46	2.48	2.24
cis_mRNA_dw20k	9925	NA	n341227	NM_020192_dup1	chr7	39622404	39622930	527	+	chr7	39606003	39612480	871	+	*	-0.9951	-1	0.35	1.8	0.25	20.6	15.86	21.55
cis_mRNA_dw20k	578	NA	LTCONS_00031371	NM_022167_dup1	chr17	48439089	48440294	1206	-	chr17	48423393	48438512	3567	+	*	0.9953	1	0.44	0.55	0.34	4.79	5.01	4.51
cis_mRNA_up10k	434	NA	n376010	NM_020648_dup1	chr18	9318628	9334332	780	-	chr18	9334765	9402418	3756	+	*	0.9748	1	0.56	0.43	0	3.61	3.31	2.92

cis_mRNA_overlap	0	mRNA	n326134	NM_022366_dup1	chr1	246714169	246715408	967	-	chr1	246703863	246729565	1786	-	*	-0.9999	-1	0	0.07	0.03	4	3.43	3.75
cis_mRNA_dw20k	6735	NA	n365311	NM_001031716_dup1	chr2	192559982	192563100	2784	+	chr2	192542861	192553248	3849	+	*	0.9919	1	0.02	0.03	0.06	0.62	0.64	0.78
cis_mRNA_dw20k	1703	NA	n341836	NM_018179_dup1	chr12	14653399	14655865	2466	+	chr12	14518611	14651697	4661	+	*	0.9878	1	0.9	0	5.12	7.24	7.19	12.07
cis_mRNA_dw20k	4151	NA	n345559	NM_018471_dup1	chr2	187378238	187406872	3337	+	chr2	187350885	187374088	2134	+	*	-0.9999	-1	0.72	0.91	0.88	62.54	57.89	58.69
cis_mRNA_up10k	1946	NA	n365510	NM_024093_dup1	chr2	105950391	105952068	1588	-	chr2	105954013	105961984	887	+	*	0.8462	1	0.05	0.99	0.38	10.9	13.29	12.95
cis_mRNA_dw20k	7597	NA	LTCONS_00037096	NM_024509_dup1	chr19	36443693	36445464	1772	.	chr19	36428022	36436097	2515	+	*	-0.9305	-1	1.48	1.79	1.31	5.27	5.14	5.55
cis_mRNA_up10k	41	NA	n375789	NM_020795_dup1	chr17	7308233	7311462	829	+	chr17	7311502	7323179	4642	+	*	0.9975	1	4.36	5.97	4.53	10.22	12.3	10.59
cis_mRNA_dw20k	4953	NA	n341011	NM_017583_dup1	chr11	35835882	35839556	3675	+	chr11	35684353	35830930	4337	+	*	0.934	1	0.91	1.22	0.85	24.92	26.27	23.6
cis_mRNA_dw20k	2222	NA	n374805	NM_022480_dup1	chr15	86296037	86300336	808	-	chr15	86302557	86338189	3683	-	*	-0.9673	-1	0.09	0.12	0.04	3.29	2.53	3.8
cis_mRNA_up10k	3758	NA	n366670	NM_022149_dup1	chr3	184433593	184445372	555	+	chr3	184428155	184429836	1682	-	*	-0.9824	-1	0.22	0.65	1.18	47.16	44.03	42.04
cis_mRNA_up10k	5238	NA	LTCONS_00038264	NM_020122_dup1	chr2	85153171	85192994	4478	+	chr2	85198231	85286595	7553	+	*	0.7397	1	0.72	0.71	0.88	9.36	9.08	9.46
cis_mRNA_dw20k	15649	NA	n374036	NM_031914_dup1	chr14	62584075	62595131	863	+	chr14	62462541	62568427	3137	+	*	-0.7777	-0.866	0.18	0.18	0.15	2.01	1.52	2.29
cis_mRNA_dw20k	3140	NA	n381885	NM_018448_dup1	chr12	67711527	67713732	2206	+	chr12	67663061	67708388	5908	+	*	-0.9191	-1	7.77	9.31	7.58	75.24	71.68	78.58
cis_mRNA_up10k	6841	NA	LTCONS_00044657	NM_022358_dup1	chr20	43323577	43367648	760	-	chr20	43374488	43380954	2532	+	*	-0.9979	-1	1.94	2.01	1.09	0.01	0	0.07
cis_mRNA_up10k	350	NA	n407268	NM_017906_dup1	chr6	10671651	10694839	539	-	chr6	10695188	10709972	1497	+	*	0.8943	1	1.88	1.76	4.29	16.88	15.45	18.4
cis_mRNA_overlap	0	Lnc-CompleteIn-mRNAIntron	n340722	NM_001122679_dup1	chr5	167555861	167557427	1566	+	chr5	166711843	167691162	9645	+	*	0.9481	1	0.02	0.1	0	2.83	4.6	1.12
cis_mRNA_dw20k	11746	NA	n411109	NM_018463_dup1	chr12	2945982	2968961	877	+	chr12	2921787	2934237	2362	+	*	0.9998	1	0.41	0.7	0.14	6.49	6.78	6.2
cis_mRNA_overlap	0	Lnc-AntiOverlap-mRNA	n379581	NM_024886_dup1	chr10	104209595	104216051	1287	+	chr10	104209594	104211300	1469	-	*	-0.9993	-1	14.23	12.46	11.43	0.09	0.13	0.15
cis_mRNA_dw20k	1129	NA	LTCONS_00033741	NM_024323_dup1	chr19	13976111	13992040	926	+	chr19	13993168	14016909	2420	-	*	-0.96	-1	1.42	2.43	1.67	0.79	0.77	0.78
cis_mRNA_dw20k	5358	NA	n378505	NM_018122_dup1	chr1	173833039	173837127	822	-	chr1	173793797	173827682	3348	+	*	-0.9852	-1	3.38	4.07	2.05	4.13	3.98	4.81
cis_mRNA_up10k	708	NA	n345131	NM_020693_dup1	chr11	117668683	117676368	2880	-	chr11	117298489	117667976	6899	-	*	-0.9959	-1	0.05	0.04	0.01	0.29	0.43	0.71
cis_mRNA_up10k	4776	NA	n377827	NM_017940_dup1	chr1	16944757	16961054	2763	-	chr1	16890412	16939982	4339	-	*	0.9035	1	0.82	0	2.1	10.25	8.47	10.74
cis_mRNA_overlap	0	Lnc-AntiOverlap-mRNA	n411135	NM_017770_dup1	chr6	11043991	11079377	1615	+	chr6	10980992	11044624	4074	-	*	0.9258	1	0.15	0.18	0.13	0.97	1.38	0.96
cis_mRNA_dw20k	7550	NA	n341373	NM_022458_dup1	chr7	156462735	156466021	3286	-	chr7	156473570	156685902	4890	-	*	0.866	1	0.31	0.27	0.2	14.82	13.77	13.56
cis_mRNA_dw20k	1733	NA	n384454	NM_018475_dup1	chr4	56294074	56297591	3518	-	chr4	56262090	56292342	1965	+	*	0.7364	1	0.79	0	0.6	15.95	12.81	13.06
cis_mRNA_up10k	387	NA	n338226	NM_020832_dup1	chr1	151252500	151254405	622	-	chr1	151254791	151264381	4508	+	*	0.9982	1	0.18	0.59	0.21	3.8	4.54	3.9
cis_mRNA_overlap	0	Lnc-AntiOverlap-mRNA	LTCONS_00030561	NM_017775_dup1	chr17	15879471	15903081	1548	-	chr17	15902694	15932894	3505	+	*	-0.9721	-1	1.45	0.94	0.96	17.16	18.46	18.1
cis_mRNA_overlap	0	mRNA	n341052	NM_024036_dup1	chr11	66624589	66627946	1554	+	chr11	66624876	66627946	2528	+	*	-0.998	-1	1.91	1.87	1.56	28.9	29.94	34.72
cis_mRNA_overlap	0	Lnc-AntiOverlap-mRNA	n342041	NM_017934_dup1	chr6	79787562	79790141	2580	+	chr6	79644136	79788011	11966	-	*	-0.9524	-1	1.11	1.42	1.51	22.36	21.19	20.01
cis_mRNA_dw20k	16946	NA	n405260	NM_018933_dup1	chr5	140613938	140617101	3164	+	chr5	140593509	140596993	3485	+	*	0.9849	1	0.87	0.3	0.39	1.07	0.76	0.86
cis_mRNA_up10k	8047	NA	n405260	NM_018935_dup1	chr5	140613938	140617101	3164	+	chr5	140625147	140627802	2656	+	*	0.6631	1	0.87	0.3	0.39	8.87	4.24	8.59
cis_mRNA_up10k	429	NA	n338246	NM_020699_dup1	chr1	153895879	153907635	1120	+	chr1	153777201	153895451	7478	-	*	0.9911	1	0.22	0.31	0.36	6.96	7.57	8.12
cis_mRNA_dw20k	8866	NA	n339672	NM_030919_dup1	chr20	37590568	37590754	187	-	chr20	37554955	37581703	2429	+	*	0.9999	0.866	0.24	1.11	1.1	0.42	0.49	0.49
cis_mRNA_overlap	0	Lnc-CompleteIn-mRNAExon	n338934	NM_031895_dup1	chr19	54490741	54493468	2723	+	chr19	54466290	54493469	8751	+	*	0.9848	0.866	0.02	0.02	0.21	1.24	1.12	1.77
cis_mRNA_overlap	0	CompleteIn-LncExon	n405881	NM_017846_dup1	chr1	28879529	28905057	2015	+	chr1	28879529	28905057	1867	+	*	-0.9422	-1	2.21	2.79	1.63	12.45	9.38	13.18
cis_mRNA_dw20k	16228	NA	n365563	NM_024770_dup1	chr2	172101203	172157685	603	-	chr2	172173912	172291312	8135	-	*	0.827	1	0.06	0.28	0	1.15	1.4	0.41
cis_mRNA_dw20k	10665	NA	n385218	NM_030798_dup1	chr7	74440801	74445619	4819	+	chr7	74456283	74489699	2378	-	*	-0.6982	-1	16.38	16.45	15.45	11.19	8.9	11.75
cis_mRNA_dw20k	16380	NA	n346376	NM_021633_dup1	chr1	202830879	202843851	1726	+	chr1	202860230	202896371	3338	-	*	-0.9186	-1	1.1	0.76	0.53	19.13	21.89	21.91
cis_mRNA_dw20k	1893	NA	n364686	NM_018221_dup1	chr2	74378365	74381319	2915	+	chr2	74383211	74405995	1329	-	*	-0.9732	-1	2.64	2.72	2	11.4	11.29	11.62
cis_mRNA_dw20k	16839	NA	n369945	NM_022373_dup1	chr7	35654246	35655431	1091	-	chr7	35672269	35734772	2911	-	*	0.9864	1	0.57	0.77	0.82	5.35	5.57	5.69
cis_mRNA_dw20k	1019	NA	n383019	NM_024731_dup1	chr16	84696934	84700665	3732	+	chr16	84682131	84695916	2169	+	*	1	1	7.82	7.12	8.9	8.11	7.59	8.92
cis_mRNA_overlap	0	Lnc-AntiOverlap-mRNA	n333535	NM_031488_dup1	chr22	41625501	41634148	1566	-	chr22	41601313	41627275	3192	+	*	0.6969	1	0.07	0.19	0.21	13.57	13.69	14.53
cis_mRNA_dw20k	3597	NA	n377897	NM_020950_dup1	chr1	180918835	180924023	1440	-	chr1	180882313	180915239	4155	+	*	0.9934	1	0.48	0.64	0.32	0.27	0.31	0.21
cis_mRNA_dw20k	6537	NA	LTCONS_00007586	NM_017940_dup1	chr1	16883644	16883876	233	.	chr1	16890412	16939982	4339	-	*	-0.667	-0.866	3.51	3.51	2.8	10.25	8.47	10.74
cis_mRNA_dw20k	7502	NA	LTCONS_00014963	NM_024769_dup1	chr11	122933303	122935519	2217	.	chr11	122943020	123066007	2643	-	*	0.9604	1	0.9	0.76	0.99	1.16	0.66	2.04
cis_mRNA_dw20k	9148	NA	n376322	NM_018273_dup1	chr19	48824742	48826469	916	+	chr19	48835616	48867186	2249	-	*	-0.9523	-1	0.08	0.21	0.24	1.22	1.05	0.9
cis_mRNA_dw20k	2015	NA	n378603	NM_024734_dup1	chr14	95643820	95646262	2323	+	chr14	95648276	95786245	12750	-	*	-0.9838	-1	0.23	0.08	0.16	0.23	0.36	0.27
cis_mRNA_overlap	0	Lnc-CompleteIn-mRNAIntron	n341336	NM_194071_dup1	chr7	137665746	137668441	2696	-	chr7	137559725	137686846	7455	-	*	0.9177	0.866	0.09	0.12	0.12	0.82	1.12	1
cis_mRNA_up10k	5809	NA	n407262	NM_018451_dup1	chr13	25502893	25542607	6477	-	chr13	25456412	25497085	5173	-	*	-0.6889	-1	0.28	0	0.01	9.84	11.8	10.26
cis_mRNA_dw20k	8745	NA	n369972	NM_022906_dup1	chr7	66795257	66829655	4561	-	chr7	66767616	66786513	2135	+	*	-0.9378	-0.866	0.05	0.1	0.05	9.37	7.42	10.43
cis_mRNA_dw20k	10640	NA	LTCONS_00059107	NM_024337_dup1	chr5	3612156	3618566	6411	.	chr5	3596168	3601517	1858	+	*	0.8376	1	0.63	0.46	0.97	46.88	39.41	48.16
cis_mRNA_dw20k	6810	NA	n378704	NM_018122_dup1	chr1	173834491	173837127	897	-	chr1	173793797	173827682	3348	+	*	-0.9496	-1	1.47	2.1	0.76	4.13	3.98	4.81
cis_mRNA_up10k	783	NA	n383561	NM_022103_dup1	chr19	56989552	57006803	661	+	chr19	56950693	56988770	3860	-	*	0.8045	1	7.87	0.42	0	2.58	1.65	0
cis_mRNA_overlap	0	Lnc-CompleteIn-mRNAIntron	n338025	NM_024686_dup1	chr1	84444801	84449983	2921	-	chr1	84335057	84464833	3648	-	*	0.9998	1	0.36	0.39	0.5	10.74	10.93	11.72
cis_mRNA_dw20k	5230	NA	n369735	NM_018641_dup1	chr7	2479469	2483692	3365	+	chr7	2443259	2474240	2093	+	*	-0.9602	-1	0.55	0.86	0.73	1.41	0.78	1.2
cis_mRNA_up10k	62	NA	LTCONS_00050267	NM_001080517_dup1	chr3	9427509	9439342	2893	-	chr3	9439403	9519838	6827	+	*	-0.8503	-1	1.03	0.59	0.42	4.63	5.05	6.47
cis_mRNA_overlap	0	mRNA	n341488	NM_021623_dup1	chr8	38758829	38826184	1071	+	chr8	38758753	38831431	5641	+	*	-0.6611	-1	0.28	0.11	0.26	0.62	0.92	0.89
cis_mRNA_dw20k	1902	NA	LTCONS_00073496	NM_025072_dup1	chr9	130875076	130881071	3102	-	chr9	130882972	130890474	2064	-	*	0.9089	1	0.38	0.23	0.58	8.86	8.82	11.92
cis_mRNA_up10k	779	NA	n410071	NM_024602_dup1	chr1	45477805	45481341	1458	+	chr1	45468220	45477027	3628	-	*	0.9102	1	5.19	5.25	5.54	11.18	12.72	13.95
cis_mRNA_up10k	5432	NA	n377412	NM_024627_dup1	chr22	19847802	19854904	610	+	chr22	19833661	19842371	6635	-	*	-0.9745	-0.866	0.06	0.05	0.05	5.03	6.4	6.82
cis_mRNA_overlap	0	CompleteIn-LncExon	n408896	NM_022103_dup1	chr19	56950693	56988770	4462	-	chr19	56950693	56988770	3860	-	*	0.9585	1	3.23	1.27	0.03	2.58	1.65	0
cis_mRNA_dw20k	6520	NA	n338688	NM_020905_dup1	chr2	18728655	18729470	814	-	chr2	18735989	18741959	1600	-	*	0.9835	1	0.13	0	0.04	8.58	7.17	7.83

cis_mRNA_overlap	0	mRNA	n325994	NM_018590_dup1	chr10	43674865	43710887	1974	+	chr10	43633934	43680750	3724	+	*	-0.9792	-1	0.05	0.14	0.09	10.53	8.65	9.36
cis_mRNA_dw20k	3203	NA	n383244	NM_024591_dup1	chr17	78977135	78978830	1696	+	chr17	78965641	78973933	1664	+	*	0.999	1	0.92	0.85	0.93	9.34	8.98	9.37
cis_mRNA_up10k	5262	NA	n387059	NM_017914_dup1	chr19	1267471	1270259	1355	-	chr19	1275520	1279243	895	+	*	-0.944	-1	0.05	0.02	0	4.08	5.91	6.07
cis_mRNA_dw20k	2986	NA	n409649	NM_017761_dup1	chr1	24292937	24306953	2373	-	chr1	24286301	24289952	2417	+	*	-0.9995	-1	2.5	1.45	1.3	14.07	24.37	26.29
cis_mRNA_up10k	4697	NA	LTCONS_00004251	NM_017940_dup1	chr1	16944678	16971217	4412	-	chr1	16890412	16939982	4339	-	*	-0.995	-1	2.54	5.34	2.17	10.25	8.47	10.74
cis_mRNA_overlap	0	mRNA	n406513	NM_017948_dup1	chr9	95059640	95087876	4423	-	chr9	95059640	95087876	4319	-	*	-0.9517	-1	0	0.39	0.25	3.86	2.97	3.02
cis_mRNA_dw20k	16574	NA	n365497	NM_025190_dup1	chr2	98104121	98104688	362	-	chr2	98121261	98206428	5986	-	*	0.991	1	2.61	1.3	2.67	6.52	5.45	6.76
cis_mRNA_dw20k	12385	NA	n339982	NM_017845_dup1	chr4	47438484	47440426	1940	+	chr4	47452810	47465676	1412	-	*	0.9961	1	0.04	0	0.28	5.64	5.36	6.58
cis_mRNA_overlap	0	AntiCompleteIn-mRNAIntron	n378101	NM_025155_dup1	chr11	73594907	73597748	2176	-	chr11	73588033	73638779	1585	+	*	0.9896	1	0.04	0.08	0.02	9.68	12.72	7.09
cis_mRNA_up10k	46	NA	n375788	NM_020795_dup1	chr17	7308194	7311457	1260	+	chr17	7311502	7323179	4642	+	*	0.9347	1	0.61	1.4	1.01	10.22	12.3	10.59
cis_mRNA_overlap	0	mRNA	LTCONS_00059016	NM_020666_dup1	chr5	178029138	178054073	7526	-	chr5	178029665	178054054	2507	-	-1061.69	0.6478	1	2.13	2.77	2.14	5.42	5.87	5.79
cis_mRNA_up10k	69	NA	n340232	NM_031296_dup1	chr4	140372572	140374893	2322	-	chr4	140374961	140397255	3876	+	*	-0.9997	-1	1.75	1.37	1.38	8.17	9.66	9.58
cis_mRNA_up10k	147	NA	LTCONS_00054702	NM_025212_dup1	chr4	105412613	105416227	974	-	chr4	105393330	105412467	761	-	*	0.8447	1	2.9	4.88	3.49	13.9	16.88	16.29
cis_mRNA_up10k	119	NA	n407922	NM_001037806_dup1	chr12	50222326	50234937	3088	+	chr12	50184929	50222208	4900	-	*	-0.7757	-1	1.02	0.75	0.98	12.38	13.87	13.52
cis_mRNA_up10k	3833	NA	n378997	NM_017561_dup1	chr9	97094758	97123230	2162	+	chr9	97080478	97090926	2561	-	*	-0.8349	-1	0.11	0.23	0.32	0.51	0.5	0.13
cis_mRNA_dw20k	11084	NA	n380023	NM_020734_dup1	chr12	8940870	8948385	1972	+	chr12	8850518	8929787	5830	+	*	-0.6429	-1	9.63	3.05	1.75	14.78	14.81	16.08
cis_mRNA_overlap	0	Lnc-AntiOverlap-mRNA	n379594	NM_030981_dup1	chr11	66037303	66045996	2792	-	chr11	66036056	66044963	1955	+	*	0.707	1	0.88	1.16	1.22	48.98	51.21	70.25
cis_mRNA_dw20k	12522	NA	n367170	NM_024592_dup1	chr4	56251873	56253958	1487	+	chr4	56212388	56239352	4089	+	*	-0.9898	-1	0.01	0.06	0	2.39	1.49	2.78
cis_mRNA_overlap	0	mRNA	n326706	NM_020780_dup1	chr1	11592175	11595999	1285	+	chr1	11539295	11597640	5214	+	*	1	1	0.22	0.45	0.15	1.37	3	0.86
cis_mRNA_dw20k	2033	NA	n380102	NM_001031711_dup1	chr5	172382157	172386380	2067	-	chr5	172261223	172380125	2949	+	*	0.9822	1	0.55	0.65	0.74	24.88	26.3	28.81
tran	NA	NA	LTCONS_00059124	NM_030940_dup1	chr5	62072343	62073061	719	.	chr9	88879461	88897490	2004	-	-1374	0.8703	1	0.2	0.05	0	51.72	48.5	40.75
cis_mRNA_dw20k	12064	NA	n345054	NM_017561_dup1	chr9	97067717	97068415	698	+	chr9	97080478	97090926	2561	-	*	-0.9997	-0.866	0.05	0.05	0.11	0.51	0.5	0.13
cis_mRNA_up10k	109	NA	LTCONS_00071771	NM_030978_dup1	chr9	127629512	127631376	1865	+	chr9	127631484	127639727	1034	+	*	0.9987	1	0.04	0	1.33	14.96	14.56	19.21
cis_mRNA_up10k	2211	NA	n324249	NM_024067_dup1	chr7	6621773	6627705	1116	+	chr7	6629915	6648355	1913	+	*	0.6783	1	0.09	0.2	0.22	6.52	6.64	7.9
cis_mRNA_overlap	0	Lnc-AntiOverlap-mRNA	n323991	NM_021218_dup1	chr9	115453057	115484956	1223	+	chr9	115448778	115480387	1532	-	*	0.9552	1	0.11	0.1	0.04	21.78	20.28	18.22
cis_mRNA_overlap	0	mRNA	n409297	NM_022773_dup1	chr16	903635	1020984	2912	-	chr16	903634	1020984	2606	-	*	-0.7686	-1	0.92	0.26	0.15	2.4	2.76	3.97
cis_mRNA_dw20k	9915	NA	LTCONS_00005702	NM_022836_dup1	chr1	114466622	114472114	2267	-	chr1	114447915	114456708	3788	+	*	-0.9979	-1	0	0.22	0.19	1.84	1.54	1.6
cis_mRNA_overlap	0	Lnc-AntiOverlap-mRNA	n380054	NM_024307_dup1	chr16	30107751	30116777	2990	+	chr16	30116131	30124878	1098	-	*	-0.9516	-1	1.83	1.21	1.82	1.12	2.16	1.46
cis_mRNA_overlap	0	AntiCompleteIn-mRNAIntron	n384183	NM_173050_dup1	chr22	43671961	43679830	1945	+	chr22	43599229	43739394	3868	-	*	1	1	0.15	0.16	0.3	14.06	14.18	16.19
cis_mRNA_up10k	4223	NA	n339172	NM_024516_dup1	chr16	29819966	29823306	2434	-	chr16	29827528	29833816	3825	+	*	0.8486	0.866	0.31	0.22	0.39	4.3	4.3	5.8
cis_mRNA_dw20k	2948	NA	n340720	NM_018129_dup1	chr17	46029621	46029874	254	+	chr17	46018889	46026674	3465	+	*	0.9834	1	0	0.3	0.15	4.68	9.88	6.45
cis_mRNA_overlap	0	Lnc-CompleteIn-mRNAIntron	n326317	NM_152663_dup1	chr1	178794735	178802277	1489	+	chr1	178694300	178889238	5834	+	*	0.6528	0.866	0.14	0.15	0.15	6.85	7.19	8.57
cis_mRNA_up10k	256	NA	n373502	NM_020244_dup1	chr12	102089780	102091162	1247	-	chr12	102091417	102122846	1567	+	*	-0.9096	-1	0.14	0.13	0.17	0.13	0.35	0
cis_mRNA_dw20k	18444	NA	n378365	NM_019091_dup1	chr2	179388226	179402395	655	+	chr2	179345199	179369783	2470	+	*	-0.9975	-1	0.14	0	0.42	10.88	11.83	9.43
cis_mRNA_dw20k	343	NA	n385667	NM_018376_dup1	chr9	107536633	107540044	3412	+	chr9	107526451	107536291	1752	+	*	0.9534	1	0.4	0.48	0.68	1.4	1.8	2.12
cis_mRNA_dw20k	12510	NA	LTCONS_00044957	NM_018270_dup1	chr20	61444454	61445010	437	-	chr20	61427805	61431945	1636	+	*	-0.9182	-1	0.73	0.84	1.6	12.09	11.78	11.47
cis_mRNA_overlap	0	mRNA	LTCONS_00051824	NM_024947_dup1	chr3	169805368	169817286	10407	-	chr3	169805368	169899537	12672	-	-3940.62	0.7693	0.866	0.12	0.17	0.17	4.04	5.12	4.42
cis_mRNA_dw20k	2593	NA	n374603	NM_020857_dup1	chr15	41198765	41200046	968	+	chr15	41186628	41196173	3895	+	*	0.9039	1	0.55	0.82	0.88	12.02	13.29	15.03
cis_mRNA_dw20k	3140	NA	n386524	NM_022486_dup1	chr9	114796205	114799922	3030	-	chr9	114803061	114937556	2996	-	*	0.9999	1	0.86	0.89	0.88	6.17	8.9	8.02
cis_mRNA_overlap	0	Lnc-CompleteIn-mRNAIntron	n339743	NM_020386_dup1	chr3	192959568	192961761	2194	+	chr3	192958918	192988644	1062	+	*	0.9996	1	0.7	1.2	0.63	1.79	3.32	1.63
cis_mRNA_dw20k	11786	NA	n340129	NM_018243_dup1	chr4	77971553	77973268	1716	-	chr4	77870895	77959768	5555	+	*	0.9912	1	2.51	2.22	2.8	39.08	36.26	40.84
cis_mRNA_dw20k	1382	NA	n345526	NM_022055_dup1	chr2	47739991	47746533	1466	-	chr2	47747914	47797470	1425	-	*	0.7166	1	0.38	0.54	0.55	2.9	3.18	4.11
cis_mRNA_overlap	0	mRNA	LTCONS_00057369	NM_024786_dup1	chr5	795720	824714	2410	-	chr5	795720	851101	2606	-	-1351.23	0.9372	1	0.69	0.79	1.99	0.7	0.87	1.11
cis_mRNA_overlap	0	mRNA	n342042	NM_017934_dup1	chr6	79654800	79675673	1435	-	chr6	79644136	79788011	11966	-	*	-0.9969	-1	1.85	3.94	6.72	22.36	21.19	20.01
cis_mRNA_up10k	2597	NA	n340918	NM_001080519_dup1	chr17	79369409	79370944	1536	-	chr17	79373540	79433358	10164	+	*	-0.8064	-1	0.05	0.04	0.1	3.16	3.41	3.07
cis_mRNA_dw20k	16012	NA	n345476	NM_020950_dup1	chr1	180931250	180937190	990	-	chr1	180882313	180915239	4155	+	*	0.9972	1	0.14	0.16	0.1	0.27	0.31	0.21
cis_mRNA_up10k	108	NA	n363607	NM_025079_dup1	chr1	37920480	37940012	1395	-	chr1	37940119	37949978	2716	+	*	-0.9981	-1	0.11	0.15	0.08	0.05	0	0.08
cis_mRNA_dw20k	18165	NA	n363607	NM_022756_dup1	chr1	37920480	37940012	1395	-	chr1	37958176	37980364	2147	-	*	-0.9994	-1	0.11	0.15	0.08	55.72	49.86	59.58
cis_mRNA_up10k	243	NA	n338348	NM_024881_dup1	chr19	16683435	16686151	2715	+	chr19	16660648	16683193	5093	-	*	0.9149	1	0.6	0.94	0.74	6.22	7.5	7.22
cis_mRNA_overlap	0	mRNA	n377781	NM_017940_dup1	chr1	16888923	16892211	1955	-	chr1	16890412	16939982	4339	-	*	0.9096	1	5.16	3.64	7.41	10.25	8.47	10.74
cis_mRNA_up10k	6658	NA	n373776	NM_020866_dup1	chr13	70689282	70705153	594	+	chr13	70274725	70682625	4150	-	*	0.9937	1	0.08	0	0.17	2.11	1.13	3.76
cis_mRNA_overlap	0	Lnc-CompleteIn-mRNAIntron	n325553	NM_017748_dup1	chr17	36959384	36964273	2141	-	chr17	36957446	36981589	2330	-	*	-0.7025	-0.866	0.63	1.05	0.63	1.36	1.03	2.29
cis_mRNA_up10k	59	NA	n385513	NM_024345_dup1	chr9	37789213	37800732	2096	-	chr9	37800790	37867666	7915	+	*	0.9991	1	0.49	0.81	0.36	3.1	3.16	3.08
cis_mRNA_dw20k	7324	NA	n378198	NM_018122_dup1	chr1	173835005	173836178	242	-	chr1	173793797	173827682	3348	+	*	-0.9635	-1	0.2	0.35	0	4.13	3.98	4.81
cis_mRNA_overlap	0	Lnc-CompleteIn-mRNAIntron	n387672	NM_018688_dup1	chr8	22497884	22499722	1839	-	chr8	22477931	22526661	1863	-	*	0.8435	1	0.04	0.07	0.15	5.32	5.67	5.77
cis_mRNA_up10k	2054	NA	n342453	NM_022450_dup1	chr16	124682	124960	278	+	chr16	108058	122629	2992	-	*	-0.8335	-0.866	0.58	0.13	0.13	0.35	0.91	0.6
cis_mRNA_dw20k	17014	NA	n342453	NM_024571_dup1	chr16	124682	124960	278	+	chr16	103829	107669	1086	+	*	0.805	0.866	0.58	0.13	0.13	27.45	25	21.37
cis_mRNA_dw20k	5146	NA	LTCONS_00074394	NM_022117_dup1	chrX	53123307	53145163	524	+	chrX	53111542	53118162	2833	+	*	0.967	1	0	0.33	0.16	13.13	19.9	14.86
cis_mRNA_dw20k	18379	NA	n372216	NM_030971_dup1	chr10	102819376	102820683	855	-	chr10	102790996	102800998	3090	+	*	0.9704	1	0.33	0.3	0.26	30.87	29.98	26.57
cis_mRNA_overlap	0	Lnc-AntiOverlap-mRNA	n377834	NM_022140_dup1	chr5	111496223	111499973	2969	+	chr5	111498315	111755010	4707	-	*	-0.9762	-1	7.06	8.79	4.53	14.86	13.58	19.77
cis_mRNA_up10k	90	NA	n407172	NM_018321_dup1	chr5	34905366	34915731	4524	-	chr5	34915820	34925787	1273	+	*	-0.9196	-1	3.55	4.34	2.38	19.19	18.65	19.36
cis_mRNA_overlap	0	mRNA	n406826	NM_152305_dup1	chr3	119187785	119213554	3675	+	chr3	119187785	119213555	3533	+	*	-0.965	-1	0.96	0.73	0.85	5.99	7.65	6.39
cis_mRNA_dw20k	11837	NA	n324443	NM_021620_dup1	chr6	100075290	100086437	882	+	chr6	100054650	100063454	3204	+	*	0.9999	1	1.56	2.6	2.09	0.03	0.05	0.04

cis_mRNA_dw20k	2797	NA	n365789	NM_018263_dup1	chr2	25956629	25959457	265	-	chr2	25962253	26101312	7173	-	*		0.9928	0.866	0	0.14	0.14	0.46	0.66	0.69
tran	NA	NA	LTCONS_00055396	NM_019116_dup1	chr4	78033818	78034343	526	.	chr16	23568862	23585710	4931	+		-1034.8	-0.8632	-1	0.07	0.06	0	27.84	29.36	30.29
cis_mRNA_up10k	1027	NA	n337728	NM_024758_dup1	chr1	15912631	15930121	2845	-	chr1	15899152	15911605	2196	-	*		-0.9027	-1	0.05	0.08	0.18	0.59	0.54	0.51
cis_mRNA_dw20k	10807	NA	n341049	NM_031904_dup1	chr11	65191802	65193997	1988	+	chr11	65154041	65180996	3752	+	*		-1	-1	0.48	0.34	0.04	1.62	1.87	2.4
cis_mRNA_overlap	0	Lnc-CompleteIn-mRNAIntron	n340594	NM_020796_dup1	chr5	115887966	115890185	2220	-	chr5	115779251	115910551	6860	-	*		0.7515	1	0.65	0.58	0.3	8.55	3.85	3.04
cis_mRNA_dw20k	3720	NA	n384430	NM_025205_dup1	chr4	17629879	17633666	3788	+	chr4	17616273	17626160	1281	+	*		0.9045	1	5.79	6.32	5.32	20.07	20.52	17
cis_mRNA_dw20k	2746	NA	n383499	NM_020917_dup1	chr19	36810618	36822610	1898	-	chr19	36825355	36870105	7493	-	*		-0.8808	-1	0.95	1.18	0.49	8.69	7.95	8.91
cis_mRNA_dw20k	2173	NA	n342251	NM_018207_dup1	chr1	33607472	33608831	1360	-	chr1	33611003	33647671	3841	-	*		0.8665	1	0.03	0.12	0.23	1.42	1.99	2.03
cis_mRNA_dw20k	803	NA	n339177	NM_023931_dup1	chr16	30538239	30541977	1951	+	chr16	30542779	30546194	2371	-	*		0.7042	1	0.07	0.13	0.08	2.06	2.6	2.54
cis_mRNA_dw20k	5469	NA	n367986	NM_030613_dup1	chr5	178365678	178368084	2287	-	chr5	178322916	178360210	2405	+	*		-0.9351	-1	0.43	0.44	0.37	2.03	1.84	2.24
cis_mRNA_overlap	0	Lnc-CompleteIn-mRNAExon	n341045	NM_001144936_dup1	chr11	63527369	63529844	2476	-	chr11	63527364	63536113	5582	-	*		0.8011	1	25.08	18.64	21.18	23.13	15.21	23.1
cis_mRNA_overlap	0	Lnc-CompleteIn-mRNAExon	n384826	NM_001031713_dup1	chr6	13786977	13790645	3669	-	chr6	13786781	13814792	5471	-	*		-0.8275	-1	0.31	0.82	0.07	4.01	3.88	4.79
cis_mRNA_dw20k	17354	NA	n407766	NM_025219_dup1	chr20	62584737	62588338	3571	+	chr20	62526455	62567384	5293	+	*		-0.9424	-1	0.47	0.7	0.59	37.29	35.03	36.8
tran	NA	NA	LTCONS_00070763	NM_017867_dup1	chr9	14993325	15019722	888	+	chr4	170650619	170679093	1216	-		-385.79	-0.9976	-1	1.04	0.82	0.71	17.65	20.38	21.39
cis_mRNA_overlap	0	Lnc-CompleteIn-mRNAExon	n408094	NM_001039618_dup1	chr11	85368608	85376182	4618	-	chr11	85368608	85376182	7575	-	*		0.9721	1	1.23	1.65	0.74	7.5	8.65	6.93
cis_mRNA_overlap	0	AntiCompleteIn-mRNAIntron	n385625	NM_022755_dup1	chr9	95393149	95395650	2502	+	chr9	95375466	95432547	4401	-	*		0.9944	1	0.32	0.61	0.47	3.75	4.16	4
cis_mRNA_up10k	398	NA	LTCONS_00017004	NM_018009_dup1	chr12	6548167	6560780	3430	-	chr12	6561177	6571488	1818	+	*		0.9983	1	1.57	1.5	1.75	0.19	0.16	0.25
cis_mRNA_overlap	0	mRNA	n409517	NM_021174_dup1	chr8	22462145	22477983	3809	+	chr8	22462539	22477984	3992	+	*		-0.9966	-1	20.81	19.8	23.53	5.88	6.1	4.96
cis_mRNA_dw20k	6974	NA	n364251	NM_030952_dup1	chr1	205251239	205264218	490	-	chr1	205271191	205290883	3401	-	*		0.997	1	0	0.17	0.19	1.07	1.38	1.45
cis_mRNA_up10k	1743	NA	LTCONS_00059143	NM_018939_dup1	chr5	140526796	140528097	1302	.	chr5	140529839	140532868	3030	+	*		0.9986	1	1.82	0.83	0.91	0.73	0.27	0.28
cis_mRNA_overlap	0	Lnc-CompleteIn-mRNAIntron	n406838	NM_020133_dup1	chr6	161581164	161583014	1851	-	chr6	161551057	161695107	7879	-	*		-0.9969	-1	2.01	2.08	2.22	20.74	20.29	19.02
cis_mRNA_dw20k	16404	NA	n376626	NM_022080_dup1	chr20	23337432	23338753	505	-	chr20	23355156	23402156	3871	-	*		0.9374	1	0	0.07	0.13	67.31	85.18	87.94
cis_mRNA_up10k	731	NA	n383560	NM_022103_dup1	chr19	56989500	57006803	1523	+	chr19	56950693	56988770	3860	-	*		0.9988	1	15.57	9.36	0.16	2.58	1.65	0
cis_mRNA_up10k	8745	NA	n345207	NM_017520_dup1	chr13	20179732	20199044	1950	+	chr13	20207788	20247599	4268	+	*		-0.8778	-0.866	0.02	0.02	0.03	20.26	20.03	19.78
cis_mRNA_up10k	552	NA	n379300	NM_018154_dup1	chr19	14247991	14282071	2856	+	chr19	14230321	14247440	1731	-	*		1	1	0.01	0.03	0.03	0	0.02	0.02
cis_mRNA_dw20k	5357	NA	n378839	NM_018122_dup1	chr1	173833038	173837127	542	-	chr1	173793797	173827682	3348	+	*		-0.9441	-1	2	3.45	0.46	4.13	3.98	4.81
cis_mRNA_overlap	0	mRNA	n325099	NM_030768_dup1	chr2	239084070	239128434	1975	-	chr2	239079043	239112324	1443	-	*		-0.9498	-1	0.74	1.01	1.14	11.79	10.35	10.34
cis_mRNA_dw20k	12075	NA	n408131	NM_024493_dup1	chr6	28346598	28367544	4343	-	chr6	28317691	28334524	2295	+	*		-0.9806	-1	4.95	4.54	3.51	6.66	7.46	8.4
cis_mRNA_up10k	6221	NA	n324912	NM_020882_dup1	chr20	61909873	61918318	533	+	chr20	61924538	61962285	4172	+	*		-0.942	-1	0.07	0.38	0.56	0.03	0.02	0
cis_mRNA_overlap	0	mRNA	n410323	NM_018061_dup1	chr1	109234935	109244422	3753	+	chr1	109234932	109244518	3711	+	*		-0.9912	-1	0.88	1.21	0.19	4.92	4.73	5.68
cis_mRNA_dw20k	16003	NA	n340518	NM_017775_dup1	chr17	15948896	15949795	460	-	chr17	15902694	15932894	3505	+	*		0.9518	1	0.16	0.45	0.29	17.16	18.46	18.1
cis_mRNA_up10k	35	NA	LTCONS_00067903	NM_022900_dup1	chr7	94138734	94139136	403	.	chr7	94139170	94186331	3882	+	*		0.9589	1	1.33	1.91	1.19	58.28	65.86	51.94
cis_mRNA_dw20k	3736	NA	n340009	NM_018109_dup1	chr10	30591756	30594995	3235	-	chr10	30598730	30638267	5620	-	*		0.8842	1	5.12	5.2	4.6	18.03	19.54	16.91
cis_mRNA_overlap	0	mRNA	n406850	NM_017739_dup1	chr1	46654353	46685977	2936	-	chr1	46654353	46664121	2736	-	*		0.6651	0.866	0.23	0.31	0.31	20.97	21.73	24.53
cis_mRNA_dw20k	9432	NA	n364458	NM_030980_dup1	chr1	156681159	156682982	1020	-	chr1	156692413	156697705	2051	-	*		0.8339	1	0.14	0.43	0.24	7.58	9.56	9.31
cis_mRNA_dw20k	5233	NA	n386684	NM_017857_dup1	chr11	67085310	67159158	850	+	chr11	67070919	67080078	2878	+	*		-0.9992	-1	2.31	1.64	1.23	0.21	0.42	0.53
cis_mRNA_overlap	0	mRNA	LTCONS_00031760	NM_020679_dup1	chr17	73262212	73267303	3171	-	chr17	73262309	73267303	1412	-		-1030.24	0.9621	1	2.47	2.24	2.63	1.18	1.03	1.46
cis_mRNA_up10k	1648	NA	n345547	NM_022826_dup1	chr2	160561651	160567363	942	-	chr2	160569010	160625084	3468	+	*		-0.9856	-1	0	0.07	0.14	22.67	22.02	20.82
cis_mRNA_up10k	402	NA	n374280	NM_017922_dup1	chr14	45552347	45552901	467	-	chr14	45553302	45584804	2868	+	*		-0.9939	-0.866	0	0.07	0.07	10.03	8.2	7.95
cis_mRNA_overlap	0	mRNA	LTCONS_00004245	NM_017940_dup1	chr1	16922285	16940154	8129	-	chr1	16890412	16939982	4339	-	*		-0.986	-1	4.16	4.45	3.99	10.25	8.47	10.74
cis_mRNA_dw20k	18439	NA	n378671	NM_019091_dup1	chr2	179388221	179400579	529	+	chr2	179345199	179369783	2470	+	*		-0.9956	-1	0.2	0	0.64	10.88	11.83	9.43
cis_mRNA_overlap	0	mRNA-AntiCompleteIn-LncIntron	LTCONS_00062252	NM_025231_dup1	chr6	28083464	28104626	998	-	chr6	28092387	28097857	1276	+	*		0.9094	1	2.82	2.21	2.49	14.05	12.28	13.78
cis_mRNA_up10k	9419	NA	n346546	NM_030974_dup1	chr8	145168558	145169578	926	+	chr8	145153536	145159140	1816	-	*		0.931	1	0.13	0.53	0.43	3.07	4.81	3.8
cis_mRNA_overlap	0	Lnc-CompleteIn-mRNAIntron	n386571	NM_021639_dup1	chr1	46111452	46112357	906	-	chr1	46092976	46152302	3738	-	*		0.9821	1	0.63	0.68	0.66	6.66	11	10.01
cis_mRNA_overlap	0	Lnc-AntiOverlap-mRNA	n338416	NM_024496_dup1	chr14	77494429	77495418	990	+	chr14	77490886	77495034	4149	-	*		-0.9755	-1	0.32	0.2	0.35	15.08	16.34	14.23
cis_mRNA_up10k	60	NA	LTCONS_00011515	NM_018362_dup1	chr11	27528385	27699354	2058	+	chr11	27515965	27528326	4852	-	*		0.9618	1	0.15	0.04	0.05	76.96	72.56	74.13
cis_mRNA_up10k	6988	NA	LTCONS_00023107	NM_018453_dup1	chr14	35015930	35017031	1102	.	chr14	34985135	35008943	1348	-	*		0.9403	1	39.74	66.96	37.15	31.61	32.47	31
cis_mRNA_up10k	271	NA	n407988	NM_022140_dup1	chr5	111755280	111756677	1398	+	chr5	111498315	111755010	4707	-	*		0.9817	1	2.98	2.97	4.52	14.86	13.58	19.77
cis_mRNA_overlap	0	AntiCompleteIn-mRNAExon	n340696	NM_198321_dup1	chr5	153798764	153799256	493	-	chr5	153570295	153800544	5956	+	*		-0.8939	-1	0.08	0.21	0	2.48	1.59	5.93
cis_mRNA_up10k	8767	NA	n409227	NM_030916_dup1	chr1	161068151	161070138	1088	+	chr1	161040781	161059385	3502	-	*		0.8976	0.866	4.73	4.49	4.92	0.01	0	0.01
cis_mRNA_dw20k	2622	NA	n381831	NM_020734_dup1	chr12	8932408	8935685	644	+	chr12	8850518	8929787	5830	+	*		-0.7388	-1	0.8	0.46	0.32	14.78	14.81	16.08
cis_mRNA_overlap	0	mRNA	LTCONS_00057363	NM_024786_dup1	chr5	709662	851250	7985	-	chr5	795720	851101	2606	-		-1915.48	0.9873	1	1.17	1.67	3.01	0.7	0.87	1.11
cis_mRNA_overlap	0	Lnc-CompleteIn-mRNAExon	n334779	NM_018326_dup1	chr7	150270658	150271040	383	+	chr7	150264458	150271041	1965	+	*		0.995	1	0.05	0	0.19	0.06	0.05	0.11
cis_mRNA_dw20k	15018	NA	n344861	NM_198321_dup1	chr5	153815561	153823040	1762	-	chr5	153570295	153800544	5956	+	*		-0.6859	-1	0.04	0.27	0.03	2.48	1.59	5.93
cis_mRNA_up10k	2131	NA	n374511	NM_015059_dup1	chr15	62929371	62937380	2214	-	chr15	62939510	63136830	11650	+	*		-0.9351	-0.866	0.31	0.23	0.23	1.69	2.44	2.86
cis_mRNA_dw20k	763	NA	n384189	NM_017931_dup1	chr22	46691041	46692557	1517	-	chr22	46663861	46690279	2616	+	*		0.9999	1	0.69	0.96	0.73	4.01	4.96	4.14
tran	NA	NA	LTCONS_00063916	NM_030652_dup1	chr6_mann_hap4	3478570	3478878	309	.	chr6_ssto_hap7	3480109	3483790	1292	+		-205.42	-0.9998	-1	0.82	1.69	0	0.25	0	0.47
cis_mRNA_dw20k	4118	NA	n364975	NM_025181_dup1	chr2	114464370	114467816	2370	-	chr2	114471933	114514400	2848	-	*		0.871	1	5.13	6.17	6.38	10.82	11.6	12.92
cis_mRNA_overlap	0	Lnc-CompleteIn-mRNAIntron	n338476	NM_017660_dup1	chr19	19614692	19615463	375	+	chr19	19496642	19619741	5672	+	*		-0.9528	-1	0.1	0.09	0.28	0.81	0.99	0.48

cis_mRNA_overlap	0	Lnc-AntiOverlap-mRNA	n341460	NM_021174_dup1	chr8	22471421	22474170	2750	-	chr8	22462539	22477984	3992	+	*		-0.8268	-1	1.33	1.25	1.36	5.88	6.1	4.96
cis_mRNA_dw20k	5359	NA	n378107	NM_018122_dup1	chr1	173833040	173836181	745	-	chr1	173793797	173827682	3348	+	*		-0.9997	-1	12.96	13.62	9.38	4.13	3.98	4.81
cis_mRNA_overlap	0	mRNA	n406921	NM_001031716_dup1	chr2	192542798	192553248	3448	+	chr2	192542861	192553248	3849	+	*		-0.998	-1	0.52	0.49	0	0.62	0.64	0.78
tran	NA	NA	LTCONS_00052254	NM_020675_dup1	chr3	117009298	117028869	19572	.	chr2	169727401	169746944	1346	-		-594.3	0.8745	0.866	0.62	0.74	0.74	3.79	5.13	4.48
cis_mRNA_dw20k	3553	NA	LTCONS_00033007	NM_017653_dup1	chr18	46521050	46566620	4103	-	chr18	46570172	46987079	2628	-	*		0.8937	1	2.3	2.48	2.38	17.64	18.47	18.36
cis_mRNA_overlap	0	mRNA-AntiCompleteIn-LncIntron	n408110	NM_024070_dup1	chr7	99798278	99869855	2862	-	chr7	99816871	99819111	1569	+	*		-0.9784	-0.866	7.06	10.05	6.23	0.02	0	0.02
cis_mRNA_overlap	0	Lnc-AntiOverlap-mRNA	n339106	NM_017759_dup1	chr2	206950356	206951576	1221	+	chr2	206858445	206950906	14136	-	*		-0.6358	-1	4.04	2.66	3.93	3.11	3.79	3.72
cis_mRNA_dw20k	5008	NA	n406641	NM_001031723_dup1	chr4	100799495	100815703	4234	-	chr4	100820710	100867879	2766	-	*		0.6985	1	4.34	5.1	4.46	11	12.34	12.19
cis_mRNA_dw20k	6981	NA	n378904	NM_018122_dup1	chr1	173834662	173836867	796	-	chr1	173793797	173827682	3348	+	*		-0.9248	-1	1.27	1.85	0.76	4.13	3.98	4.81
cis_mRNA_dw20k	5340	NA	n338325	NM_022495_dup1	chr14	60606871	60610109	3239	+	chr14	60558629	60601532	3932	+	*		0.9728	1	4.83	4.73	4.97	17.34	16.41	17.91
cis_mRNA_overlap	0	Lnc-AntiOverlap-mRNA	n338257	NM_017797_dup1	chr19	1989902	1990369	468	+	chr19	1985447	2015702	2618	-	*		0.9998	1	0.88	1.39	2.37	26.45	27.15	28.6
cis_mRNA_dw20k	2202	NA	n339828	NM_030632_dup1	chr18	31329600	31331159	1560	+	chr18	31158541	31327399	7642	+	*		0.9247	1	4.71	6.91	7.96	5.33	6.51	8.86
cis_mRNA_up10k	107	NA	n376192	NM_024106_dup1	chr19	9649409	9650402	857	+	chr19	9638665	9649303	2320	-	*		-0.9998	-1	2.6	1.36	1.6	2.08	2.64	2.52
cis_mRNA_dw20k	19763	NA	n385926	NM_017891_dup1	chr1	995083	997436	673	+	chr1	1017198	1051736	2104	-	*		-0.9373	-1	0.11	0.2	0.05	3.15	2.88	3.82
cis_mRNA_dw20k	19557	NA	n410525	NM_017871_dup1	chr1	1215816	1227409	2960	+	chr1	1246965	1260046	2135	-	*		0.9918	1	0.6	0.82	1.19	13.51	14.3	16.59
cis_mRNA_up10k	287	NA	n383928	NM_021035_dup1	chr20	47895042	47905793	615	+	chr20	47862437	47894756	7371	-	*		0.9239	1	9.34	13.19	17.31	3.56	4.29	4.43
cis_mRNA_dw20k	4485	NA	n409178	NM_017921_dup1	chr17	79506911	79519429	3849	-	chr17	79523913	79604138	4381	-	*		0.984	1	2.46	3.07	5.6	12.77	12.78	14.44
cis_mRNA_dw20k	3023	NA	n363609	NM_023070_dup1	chr1	40932412	40938148	2382	-	chr1	40916337	40929390	2031	+	*		-0.8383	-1	1.92	2.29	1.8	1.83	1.76	2.05
tran	NA	NA	LTCONS_00064247	NM_018053_dup1	chr7	5013616	5044200	8765	+	chr1	28286504	28294604	2158	+		-1644.21	-0.7924	-0.866	0.99	0.78	0.78	0.03	0.55	0.23
cis_mRNA_up10k	47	NA	n337934	NM_017887_dup1	chr1	53686335	53689930	3596	+	chr1	53679771	53686289	1085	-	*		0.9998	1	0.09	0.04	0.13	20.9	18.65	22.57
cis_mRNA_dw20k	4027	NA	n338598	NM_018012_dup1	chr1	245870454	245872733	2280	+	chr1	245318287	245866428	7287	+	*		0.8311	1	5.53	5.04	5.69	6.02	5.51	7.75
cis_mRNA_overlap	0	mRNA	n407041	NM_022361_dup1	chr6	105605775	105624130	1389	-	chr6	105605775	105627858	1848	-	*		0.9774	0.866	0.05	0.1	0.05	0	0.27	0.06
cis_mRNA_up10k	226	NA	LTCONS_00050265	NM_001080517_dup1	chr3	9427509	9439178	3910	-	chr3	9439403	9519838	6827	+	*		0.8833	1	1.16	2.72	3.52	4.63	5.05	6.47
cis_mRNA_dw20k	19879	NA	LTCONS_00063502	NM_025218_dup1	chr6	150314724	150384111	870	-	chr6	150285143	150294846	3142	+		-400.45	0.9744	1	7.85	8.9	9.13	2.36	2.47	2.54
cis_mRNA_up10k	6929	NA	n379573	NM_017940_dup1	chr1	16946910	16951432	2866	+	chr1	16890412	16939982	4339	-	*		-0.9882	-1	0.29	0.38	0.24	10.25	8.47	10.74
cis_mRNA_up10k	9178	NA	n407232	NM_025222_dup1	chr3	52321836	52329272	2792	+	chr3	52288438	52312659	4292	-	*		-0.9083	-1	0.1	0.14	0.09	71.87	64.09	82.85
cis_mRNA_overlap	0	AntiCompleteIn-mRNAIntron	n326715	NM_018198_dup1	chr1	6707284	6710055	1686	+	chr1	6694228	6761966	3293	-	*		-0.8872	-0.866	0.02	0	0.02	12.37	13.92	11.04
cis_mRNA_up10k	167	NA	n406113	NM_019116_dup1	chr16	23533334	23568696	3869	-	chr16	23568862	23585710	4931	+	*		0.9542	1	0.93	2.44	2.59	27.84	29.36	30.29
cis_mRNA_overlap	0	Lnc-AntiOverlap-mRNA	n381337	NM_019605_dup1	chr1	210404804	210407466	800	-	chr1	210406195	210416460	2028	+	*		0.9608	1	0	0.08	0.04	0.03	0.11	0.09
cis_mRNA_overlap	0	mRNA	n378336	NM_017940_dup1	chr1	16892131	16940083	3076	-	chr1	16890412	16939982	4339	-	*		1	1	0.5	0.32	0.55	10.25	8.47	10.74
cis_mRNA_up10k	5155	NA	n338740	NM_024572_dup1	chr2	31366725	31371824	2255	-	chr2	31133333	31361571	2712	-	*		0.9853	1	0.02	0.03	0.01	0.4	0.53	0.33
cis_mRNA_overlap	0	Lnc-AntiOverlap-mRNA	n340864	NM_032478_dup1	chr17	73893141	73896229	1419	+	chr17	73894724	73901181	1575	-	*		-0.9989	-1	0.15	0.07	0.13	14.9	17.36	15.62
cis_mRNA_overlap	0	Lnc-AntiOverlap-mRNA	n410952	NM_017763_dup1	chr17	56414563	56431088	2451	+	chr17	56431037	56494931	4558	-	*		1	1	0.04	0	0.23	0.01	0	0.06
cis_mRNA_dw20k	9826	NA	n386971	NM_024792_dup1	chr17	655900	658576	2677	+	chr17	635847	646075	2080	+	*		0.9961	1	0.36	0.38	0.33	2.25	2.46	2.02
cis_mRNA_dw20k	9003	NA	n335545	NM_031302_dup1	chr12	104359654	104373763	481	+	chr12	104382765	104443915	1877	-	*		-0.9973	-1	0.34	0.69	0	0.76	0.72	0.79
cis_mRNA_up10k	1365	NA	n342613	NM_001031723_dup1	chr4	100869243	100871512	1843	-	chr4	100820710	100867879	2766	-	*		-0.9997	-1	2.06	1.72	1.75	11	12.34	12.19
cis_mRNA_up10k	2560	NA	n369907	NM_017781_dup1	chr7	1019650	1020276	417	-	chr7	1022835	1029276	2304	+	*		0.866	0.866	0	0.08	0.08	0	0.04	0.08
cis_mRNA_up10k	7834	NA	n338074	NM_022566_dup1	chr15	81283145	81285462	2317	-	chr15	81293295	81296345	3051	+	*		0.7007	0.866	0.04	0.01	0.01	12.02	9.4	11.34
cis_mRNA_dw20k	4914	NA	n341857	NM_030572_dup1	chr12	21689123	21757554	2902	-	chr12	21679256	21684210	583	+	*		0.7788	1	0	0.14	0.16	0	0.06	0.23
cis_mRNA_up10k	304	NA	n376012	NM_020648_dup1	chr18	9322059	9334462	420	-	chr18	9334765	9402418	3756	+	*		-0.826	-0.866	0.34	0.93	0.93	3.61	3.31	2.92
cis_mRNA_up10k	230	NA	n384210	NM_001080517_dup1	chr3	9430537	9439174	1834	-	chr3	9439403	9519838	6827	+	*		-0.9901	-1	5.43	3.91	1.18	4.63	5.05	6.47
cis_mRNA_up10k	965	NA	n408905	NM_017857_dup1	chr11	67056816	67069955	2223	+	chr11	67070919	67080078	2878	+	*		0.9529	1	0.91	2.51	2.58	0.21	0.42	0.53
cis_mRNA_overlap	0	Lnc-CompleteIn-mRNAIntron	n332930	NM_024106_dup1	chr19	9642966	9643488	523	-	chr19	9638665	9649303	2320	-	*		0.8756	1	0.36	1.16	0.63	2.08	2.64	2.52
cis_mRNA_up10k	3490	NA	n406668	NM_030974_dup1	chr8	145162629	145173218	7119	+	chr8	145153536	145159140	1816	-	*		0.9921	1	0.5	0.63	0.54	3.07	4.81	3.8
cis_mRNA_overlap	0	Lnc-CompleteIn-mRNAIntron	n340053	NM_018475_dup1	chr4	56264559	56265055	497	+	chr4	56262090	56292342	1965	+	*		-0.9854	-1	0.3	0.62	0.54	15.95	12.81	13.06
cis_mRNA_overlap	0	Lnc-AntiOverlap-mRNA	n339259	NM_022497_dup1	chr3	15087058	15090778	3721	+	chr3	15090019	15106816	4566	-	*		0.8783	1	1.01	1.35	0.46	4.51	6.74	4.05
cis_mRNA_up10k	6201	NA	n342823	NM_001080392_dup1	chr7	141408153	141429473	2084	+	chr7	141356528	141401953	7296	-	*		-0.8995	-1	0.02	0.05	0	7.69	7.48	8.55
tran	NA	NA	LTCONS_00042561	NM_018241_dup1	chr2	223745026	223807923	648	-	chr4	148538539	148556679	2980	+		-310.38	-0.9916	-1	1.57	1.18	0.52	14.51	15.95	17.48
cis_mRNA_dw20k	4835	NA	n326011	NM_021045_dup1	chr10	38104305	38113065	712	-	chr10	38117899	38146486	4883	-	*		0.9366	1	0.2	0	0.23	8.47	0.04	15.67
cis_mRNA_up10k	125	NA	n366596	NM_025222_dup1	chr3	52312783	52314314	224	+	chr3	52288438	52312659	4292	-	*		0.9804	1	0.39	0.35	0.52	71.87	64.09	82.85
cis_mRNA_up10k	4770	NA	n380553	NM_017940_dup1	chr1	16944751	16957401	1765	-	chr1	16890412	16939982	4339	-	*		-0.9977	-1	0.45	1.61	0	10.25	8.47	10.74
cis_mRNA_dw20k	4323	NA	n341015	NM_020929_dup1	chr11	40128794	40131431	2638	-	chr11	40135753	40315664	4054	-	*		0.9999	1	2.16	3.74	1.9	14	22.35	12.81
cis_mRNA_overlap	0	Lnc-CompleteIn-mRNAExon	n407652	NM_020717_dup1	chrX	50334643	50557044	6007	-	chrX	50334635	50557044	9568	-	*		-0.847	-1	0.24	0.18	0.04	0.04	0.12	0.14
cis_mRNA_dw20k	19468	NA	n339103	NM_001114132_dup1	chr2	204102184	204104694	2511	-	chr2	203879602	204082717	9058	+	*		-0.9561	-1	2.64	1.88	2.21	3.87	4.43	4.34
cis_mRNA_dw20k	1626	NA	n338526	NM_018040_dup1	chr1	217600340	217602209	1870	-	chr1	217603834	217804409	2336	-	*		0.9623	1	0.79	1.12	1.18	5.54	6.35	6.91
cis_mRNA_dw20k	7113	NA	n409672	NM_020775_dup1	chr1	109756515	109760113	3181	+	chr1	109656585	109749403	7032	+	*		0.9904	1	6.54	6.57	10.62	1.9	2.18	3.7
cis_mRNA_dw20k	2190	NA	n411689	NM_031490_dup1	chr16	48389596	48396910	2549	+	chr16	48278211	48387407	4342	+	*		0.8155	1	1.62	1.71	3.14	16.72	18.76	19.97
cis_mRNA_overlap	0	Lnc-AntiOverlap-mRNA	n1175	NM_020866_dup1	chr13	70682044	70705678	1236	+	chr13	70274725	70682625	4150	-	*		0.9036	1	0.13	0	0.17	2.11	1.13	3.76
cis_mRNA_dw20k	10893	NA	n340516	NM_017775_dup1	chr17	15943786	15944587	802	+	chr17	15902694	15932894	3505	+	*		0.9634	0.866	0.04	0.08	0.08	17.16	18.46	18.1

cis_mRNA_up10k	1882	NA	n381508	NM_020338_dup1	chr10	80822813	80826911	862	-	chr10	80828792	81076285	7555	+	*	0.9914	1	0.2	0.53	0	15.01	22.76	7.51
cis_mRNA_overlap	0	mRNA	n406623	NM_018320_dup1	chr11	71639768	71708643	2706	+	chr11	71639768	71708643	2606	+	*	0.9881	1	0.45	0.18	0.55	4.27	3.17	4.42
cis_mRNA_dw20k	1828	NA	n333026	NM_021035_dup1	chr20	47855465	47860610	865	+	chr20	47862437	47894756	7371	-	*	0.9059	1	0	0.04	0.09	3.56	4.29	4.43
cis_mRNA_overlap	0	mRNA	n333026	NM_017895_dup1	chr20	47855465	47860610	865	+	chr20	47835832	47860614	2695	+	*	-0.9689	-1	0	0.04	0.09	4.85	4.28	3.99
cis_mRNA_dw20k	18842	NA	LTCONS_00014953	NM_030792_dup1	chr11	75125689	75126844	1156	.	chr11	75145685	75236599	3174	-	*	-0.9994	-1	1.52	1.75	1.3	11.02	9.43	12.74
cis_mRNA_dw20k	5261	NA	LTCONS_00006641	NM_018122_dup1	chr1	173832942	173838020	841	-	chr1	173793797	173827682	3348	+	*	-0.9903	-1	9.12	9.27	4.96	4.13	3.98	4.81
cis_mRNA_dw20k	6812	NA	LTCONS_00037095	NM_024509_dup1	chr19	36442908	36443513	606	.	chr19	36428022	36436097	2515	+	*	-0.8991	-1	1.57	2.53	1.19	5.27	5.14	5.55
cis_mRNA_overlap	0	mRNA-AntiCompleteIn-LncIntron	n379409	NM_021241_dup1	chr19	15050246	16748905	1493	+	chr19	15532318	15560762	4168	-	*	-0.9707	-1	0.05	0.08	0.06	4.69	4.09	4.63
cis_mRNA_overlap	0	mRNA-AntiCompleteIn-LncIntron	n379409	NM_022904_dup1	chr19	15050246	16748905	1493	+	chr19	15562438	15575382	3290	-	*	-1	-1	0.05	0.08	0.06	0.06	0.03	0.05
cis_mRNA_overlap	0	mRNA-AntiCompleteIn-LncIntron	n379409	NM_024881_dup1	chr19	15050246	16748905	1493	+	chr19	16660648	16683193	5093	-	*	0.8756	1	0.05	0.08	0.06	6.22	7.5	7.22
cis_mRNA_dw20k	7568	NA	n379409	NM_024104_dup1	chr19	15050246	16748905	1493	+	chr19	16756472	16770968	1390	-	*	0.8966	1	0.05	0.08	0.06	18.16	18.81	18.64
cis_mRNA_up10k	4408	NA	n385981	NM_031455_dup1	chr10	13048111	13141713	969	-	chr10	12938625	13043704	2737	-	*	0.9014	1	0.17	0.43	0.31	0.81	1.73	0.93
cis_mRNA_up10k	9247	NA	n323808	NM_019556_dup1	chrX	134058543	134060320	867	-	chrX	134021662	134049297	2358	-	*	-0.9774	-1	0.33	0.52	0.07	2.21	1.93	3.15
cis_mRNA_dw20k	10771	NA	n408352	NM_031421_dup1	chr17	40128439	40169715	1989	-	chr17	40086888	40117669	2084	+	*	0.6325	1	0.15	0.14	0	1.12	0.96	0.94
cis_mRNA_up10k	97	NA	LTCONS_00042830	NM_030650_dup1	chr2	176867114	176872199	5086	.	chr2	176790410	176867018	5765	-	*	0.9881	1	2.03	2.27	1.52	21.4	22.8	19.73
cis_mRNA_overlap	0	mRNA	n326162	NM_018072_dup1	chr1	236710307	236715946	1537	-	chr1	236712305	236767841	8484	-	*	-0.8322	-1	0.32	0.25	0.46	6.8	7.37	6.71
cis_mRNA_dw20k	9279	NA	n409520	NM_030919_dup1	chr20	37590981	37668366	3342	+	chr20	37554955	37581703	2429	+	*	-0.8255	-0.866	0.98	0.45	0.75	0.42	0.49	0.49
cis_mRNA_dw20k	13208	NA	n375641	NM_024311_dup1	chr17	74788575	74799876	591	+	chr17	74733783	74775368	2538	+	*	-0.9862	-1	0.25	0.06	0	3.09	5.32	6.75
cis_mRNA_dw20k	15193	NA	LTCONS_00006032	NM_021222_dup1	chr1	151023381	151032562	3336	-	chr1	150980973	151008189	2977	+	*	1	1	2.66	3.9	2.16	9.8	10.43	9.55
cis_mRNA_up10k	8831	NA	n368448	NM_022132_dup1	chr5	70868036	70874285	1962	+	chr5	70883115	70954531	3674	+	*	0.8761	1	0.94	1	1.12	3.22	4.32	4.63
cis_mRNA_dw20k	7420	NA	n324526	NM_020737_dup1	chr6	40347283	40351954	539	-	chr6	40359373	40555126	3144	-	*	0.8498	0.866	0.07	0.25	0.25	2	4.37	3.12
cis_mRNA_up10k	13	NA	LTCONS_00045040	NM_020689_dup1	chr20	19192055	19193278	1224	.	chr20	19193290	19703541	3889	+	*	0.9731	1	0.43	0.52	0.15	8.98	11.87	5.47
cis_mRNA_up10k	38	NA	n369417	NM_021243_dup1	chr6	139348761	139349782	245	+	chr6	139349819	139364439	845	+	*	0.9589	1	0.17	0	0.31	20.98	17.49	21.8
cis_mRNA_dw20k	11925	NA	n369973	NM_022906_dup1	chr7	66798437	66800523	1264	-	chr7	66767616	66786513	2135	+	*	-0.7695	-0.866	0.03	0.03	0	9.37	7.42	10.43
cis_mRNA_up10k	366	NA	n372465	NM_025092_dup1	chr11	287702	288773	749	+	chr11	289138	295688	3268	+	*	0.996	1	0	0.13	0.04	4.88	6.57	5.26
cis_mRNA_overlap	0	AntiCompleteIn-mRNAIntron	n384259	NM_018398_dup1	chr3	54666153	54673884	3136	-	chr3	54156693	55108584	3675	+	*	-0.9288	-1	0.03	0.18	0.08	12.59	7.32	9.08
cis_mRNA_overlap	0	Lnc-AntiOverlap-mRNA	n410949	NM_017763_dup1	chr17	56406299	56431088	2278	+	chr17	56431037	56494931	4558	-	*	-0.8986	-1	1.19	1.61	0.88	0.01	0	0.06
cis_mRNA_overlap	0	Lnc-CompleteIn-mRNAIntron	n339838	NM_025135_dup1	chr18	33954221	33955232	1011	+	chr18	33877702	34360018	4942	+	*	-0.8811	-1	0.28	0.16	0.34	6.49	6.66	5.92
cis_mRNA_dw20k	17016	NA	LTCONS_00048842	NM_020163_dup1	chr3	52444527	52450253	2642	+	chr3	52467268	52479043	4700	-	*	0.8229	0.866	0	1.07	0.61	0.01	0.03	0.01
cis_mRNA_up10k	428	NA	n367674	NM_022909_dup1	chr5	68477525	68484948	436	-	chr5	68485375	68506184	1389	+	*	0.9428	1	0.09	0	0.08	2.45	2.1	2.3
cis_mRNA_dw20k	12408	NA	n366630	NM_017945_dup1	chr3	112315410	112322287	6675	+	chr3	112280895	112303003	2940	+	*	0.9538	0.866	0.04	0.04	0.05	25.24	26.77	30.21
cis_mRNA_dw20k	17277	NA	n409273	NM_018143_dup1	chr17	39981334	39992523	1646	-	chr17	40009799	40021629	2326	-	*	-0.7565	-1	49.86	39.52	49.36	11.17	12.66	12.22
cis_mRNA_dw20k	221	NA	n340024	NM_021226_dup1	chr10	49651879	49653859	1981	-	chr10	49654079	49813138	2749	-	*	0.9029	1	0.57	0.7	1.59	8.31	11.46	14.22
cis_mRNA_dw20k	7589	NA	n365183	NM_018221_dup1	chr2	74375288	74375623	210	+	chr2	74383211	74405995	1329	-	*	-0.8958	-1	0.52	0.89	0.39	11.4	11.29	11.62
cis_mRNA_overlap	0	Lnc-CompleteIn-mRNAIntron	n405607	NM_018064_dup1	chr6	88410020	88410933	914	-	chr6	88384578	88411985	1932	-	*	0.7617	0.866	5.4	6.19	5.4	8.51	8.94	7.68
cis_mRNA_dw20k	4381	NA	n377457	NM_030641_dup1	chr22	36068836	36085068	391	+	chr22	36044424	36064456	10139	+	*	-0.9177	-0.866	0.3	0	0.18	0.04	0.05	0.04
cis_mRNA_dw20k	10660	NA	LTCONS_00000895	NM_031280_dup1	chr1	36901423	36910703	634	+	chr1	36921362	36930040	965	-	*	0.9786	1	0.36	1.55	0	36	37.57	35.97
cis_mRNA_up10k	74	NA	n379316	NM_018362_dup1	chr11	27528399	27699354	2324	+	chr11	27515965	27528326	4852	-	*	-0.9938	-1	0	0.11	0.06	76.96	72.56	74.13
cis_mRNA_dw20k	317	NA	n378245	NM_022140_dup1	chr5	111496226	111497999	1367	+	chr5	111498315	111755010	4707	-	*	-0.9844	-1	1.36	1.37	0.94	14.86	13.58	19.77
cis_mRNA_overlap	0	Lnc-CompleteIn-mRNAIntron	n342218	NM_022787_dup1	chr1	10026764	10027014	251	+	chr1	10003486	10045556	3781	+	*	-0.7689	-1	0.17	0.15	0	2.51	3.16	3.38
cis_mRNA_up10k	4463	NA	n345365	NM_017742_dup1	chr18	60181761	60186196	1640	-	chr18	60190658	60245818	5543	+	*	0.9777	1	0.04	0.1	0.08	2.36	2.63	2.49
cis_mRNA_up10k	287	NA	n383927	NM_021035_dup1	chr20	47895042	47905793	677	+	chr20	47862437	47894756	7371	-	*	-0.9964	-1	60.18	51.44	50.75	3.56	4.29	4.43
cis_mRNA_overlap	0	Lnc-CompleteIn-mRNAIntron	n325618	NM_001014283_dup1	chr13	114113136	114117525	514	-	chr13	114110134	114145023	3029	-	*	0.6419	0.866	0.07	0.07	0.32	7.82	6.62	8.09
cis_mRNA_dw20k	4756	NA	LTCONS_00019210	NM_018559_dup1	chr13	45607160	45609032	1873	+	chr13	45563687	45602405	1393	+	*	-0.8452	-1	1.64	1.71	1.36	5.02	3.41	5.7
cis_mRNA_overlap	0	Lnc-AntiOverlap-mRNA	LTCONS_00006257	NM_018116_dup1	chr1	155584813	155588107	3295	-	chr1	155580007	155585141	2446	+	*	0.9858	1	0.14	0.18	0.23	3.7	3.98	4.65
cis_mRNA_overlap	0	AntiCompleteIn-mRNAIntron	n341829	NM_030640_dup1	chr12	12632120	12632530	411	+	chr12	12626216	12715448	6312	-	*	0.7615	1	0.56	0.34	0.33	11.03	10.54	9.43
cis_mRNA_up10k	175	NA	n411528	NM_018332_dup1	chr16	70349543	70380650	1471	-	chr16	70380824	70407284	2903	+	*	0.8782	1	0.34	0.38	0.7	12.56	14.56	16.09
cis_mRNA_overlap	0	Lnc-CompleteIn-mRNAIntron	n338815	NM_024766_dup1	chr2	44622449	44623532	1078	+	chr2	44589043	44999729	1553	+	*	0.9338	1	0.07	0.12	0.2	3.79	4.75	5.15
cis_mRNA_up10k	112	NA	LTCONS_00054149	NM_022346_dup1	chr4	17801804	17812414	10261	-	chr4	17812525	17846488	4664	+	*	0.9868	1	1.5	2.24	2.03	0.01	0.08	0.05
cis_mRNA_up10k	4696	NA	n346415	NM_021047_dup1	chr19	19969356	19972019	1407	-	chr19	19976714	20004293	2329	+	*	0.9942	1	0.05	0.09	0.02	5.11	6.41	3.69
cis_mRNA_up10k	88	NA	n342088	NM_024641_dup1	chr6	96023059	96025326	2268	-	chr6	96025413	96057326	4574	+	*	0.9441	1	3.56	4.61	2.46	6.51	7.11	6.35
cis_mRNA_overlap	0	Lnc-CompleteIn-mRNAIntron	n340734	NM_022897_dup1	chr5	170507601	170508562	955	+	chr5	170288896	170727019	4566	+	*	0.9009	1	0.04	0.17	0.03	3.64	4.2	3.1
cis_mRNA_overlap	0	AntiCompleteIn-mRNAIntron	n340004	NM_018076_dup1	chr10	28127445	28130140	2696	+	chr10	28101097	28287977	3572	-	*	0.9934	0.866	0.03	0.03	0.18	1.24	1.01	2.85
cis_mRNA_up10k	159	NA	n407267	NM_017906_dup1	chr6	10671651	10695030	686	-	chr6	10695188	10709972	1497	+	*	0.9438	1	0.4	0.33	0.72	16.88	15.45	18.4
cis_mRNA_up10k	3575	NA	n411747	NM_024579_dup1	chr1	150237799	150241609	2192	-	chr1	150245183	150253335	527	+	*	-0.8756	-1	1.79	1.7	1.97	0.07	0.32	0
cis_mRNA_dw20k	9022	NA	n344930	NM_017909_dup1	chr6	151713943	151716968	1661	+	chr6	151725989	151773316	1911	-	*	0.9521	1	1.02	1.33	1.21	14.01	14.99	14.9

cis_mRNA_up10k	1267	NA	n344930	NM_020861_dup1	chr6	151713943	151716968	1661	+	chr6	151685250	151712677	3086	-	*		0.9364	1	1.02	1.33	1.21	1.99	2.24	2.23
cis_mRNA_up10k	4514	NA	n345863	NM_018088_dup1	chr12	8384727	8395419	1338	-	chr12	8373856	8380214	2516	-	*		0.7326	1	0.09	0.26	0.23	0	0.07	0.01
cis_mRNA_dw20k	12757	NA	n410112	NM_022120_dup1	chr1	40204517	40222441	4368	+	chr1	40235197	40237020	1824	-	*		0.9991	0.866	1.32	1.11	1.31	0.42	0.33	0.42
cis_mRNA_dw20k	10597	NA	n345573	NM_025139_dup1	chr2	232220510	232225661	780	+	chr2	232063342	232209914	2218	+	*		-0.6776	-0.866	0	0.04	0.04	3.91	3.3	3.77
cis_mRNA_up10k	130	NA	n338080	NM_020243_dup1	chr22	39066473	39077825	1325	-	chr22	39077954	39080765	1378	+	*		-0.9972	-1	1.26	1.41	1.2	32.99	28.88	34.12
cis_mRNA_overlap	0	Lnc-AntiOverlap-mRNA	n378874	NM_021035_dup1	chr20	47894715	47905797	1075	+	chr20	47862437	47894756	7371	-	*		-0.9887	-0.866	0.29	0.1	0.1	3.56	4.29	4.43
cis_mRNA_up10k	1428	NA	n374979	NM_024776_dup1	chr15	77578760	77712556	296	-	chr15	77400471	77577333	11576	-	*		0.7352	1	0.27	0.37	0	1.27	2.38	1.22
cis_mRNA_overlap	0	Lnc-CompleteIn-mRNAIntron	n339983	NM_020453_dup1	chr4	47552735	47556650	3916	+	chr4	47487410	47595503	6550	+	*		0.9621	1	0.1	0.21	0.36	1.3	1.75	3.96
cis_mRNA_dw20k	918	NA	n377988	NM_017971_dup1	chr1	1334910	1336359	1149	+	chr1	1337276	1342693	733	-	*		0.9884	1	7.44	5.36	6.85	54.11	48.39	53.33
cis_mRNA_dw20k	6162	NA	n410506	NM_024556_dup1	chr11	126139040	126148027	2090	+	chr11	126081619	126132879	2090	+	*		-0.9295	-1	1.87	3.97	5.59	8.18	6.79	6.67
cis_mRNA_overlap	0	Lnc-AntiOverlap-mRNA	n341348	NM_018646_dup1	chr7	142569836	142572152	2317	+	chr7	142568960	142583477	2894	-	*		0.6934	0.866	0.19	0.31	0.28	0.01	0.03	0.01
cis_mRNA_overlap	0	Lnc-CompleteIn-mRNAIntron	n339582	NM_018327_dup1	chr20	13093137	13095336	2200	+	chr20	12989627	13147411	3845	+	*		1	1	0.02	0.01	0	0.04	0.02	0
cis_mRNA_overlap	0	AntiCompleteIn-mRNAIntron	n338327	NM_022495_dup1	chr14	60567052	60568435	1240	-	chr14	60558629	60601532	3932	+	*		0.8544	1	0.11	0.1	0.18	17.34	16.41	17.91
cis_mRNA_up10k	914	NA	n369777	NM_022748_dup1	chr7	47622655	47630846	1257	+	chr7	47314752	47621742	7618	-	*		-0.9988	-1	0.25	0.05	0.15	0.31	0.66	0.47
cis_mRNA_overlap	0	CompleteIn-LncExon	LTCONS_00024026	NM_024608_dup1	chr15	75638984	75648130	8535	+	chr15	75639331	75647588	1892	+		-1608.94	-0.9985	-1	1.3	1.88	1.95	0.9	0.71	0.7
cis_mRNA_dw20k	6371	NA	LTCONS_00024026	NM_017828_dup1	chr15	75638984	75648130	8535	+	chr15	75628374	75632614	925	+	*		-0.9976	-1	1.3	1.88	1.95	10.77	9.2	8.86
cis_mRNA_dw20k	5130	NA	LTCONS_00074393	NM_022117_dup1	chrX	53123291	53156872	4610	+	chrX	53111542	53118162	2833	+	*		-0.835	-1	1.83	1.03	1.21	13.13	19.9	14.86
cis_mRNA_up10k	61	NA	LTCONS_00031484	NM_018304_dup1	chr17	57187278	57232800	2738	-	chr17	57232860	57284070	6473	+	*		-0.9027	-1	4.12	2.87	5.35	4.34	4.53	2.47
cis_mRNA_dw20k	1323	NA	n375889	NM_130386_dup1	chr18	314887	318033	2411	+	chr18	319355	500729	3134	-	*		-0.8262	-1	0.13	0.05	0.08	0.38	1.43	0.46
cis_mRNA_up10k	7572	NA	LTCONS_00059142	NM_018936_dup1	chr5	140464282	140466666	2385	.	chr5	140474237	140476964	2728	+	*		-0.8046	-1	1	0.42	0.19	27.15	28.18	34.37
cis_mRNA_dw20k	14048	NA	LTCONS_00035107	NM_024762_dup1	chr19	58295131	58304403	9273	+	chr19	58318450	58326281	2352	-	*		0.9984	1	2.02	2.15	2.51	3.7	4.02	5.21
cis_mRNA_up10k	8476	NA	n332610	NM_020924_dup1	chr9	125702254	125702640	387	+	chr9	125680378	125693779	1910	-	*		0.9843	1	0.18	0.14	0.1	5.96	5.55	4.77
cis_mRNA_overlap	0	Lnc-AntiOverlap-mRNA	n339009	NM_022493_dup1	chr16	783055	785525	2162	+	chr16	779767	790997	2093	-	*		0.9936	0.866	0.49	0.49	0.52	12.97	12.58	15.75
cis_mRNA_dw20k	10466	NA	n339009	NM_023933_dup1	chr16	783055	785525	2162	+	chr16	771158	772590	858	+	*		0.7657	0.866	0.49	0.49	0.52	15.83	11.78	17.98
cis_mRNA_dw20k	15576	NA	n339009	NM_024042_dup1	chr16	783055	785525	2162	+	chr16	765173	767480	1092	+	*		-0.91	-0.866	0.49	0.49	0.52	5.96	4.49	2.43
cis_mRNA_overlap	0	Lnc-AntiOverlap-mRNA	n337766	NM_018728_dup1	chr15	52472414	52498071	1972	+	chr15	52484515	52587995	6975	-	*		-0.9449	-1	0.56	0.71	0.59	0.11	0.05	0.08
cis_mRNA_up10k	257	NA	LTCONS_00010341	NM_020338_dup1	chr10	80703083	80828536	2371	-	chr10	80828792	81076285	7555	+	*		0.8613	0.866	0.08	0.08	0	15.01	22.76	7.51
cis_mRNA_dw20k	5110	NA	n381829	NM_018088_dup1	chr12	8332805	8368747	2167	+	chr12	8373856	8380214	2516	-	*		0.9495	1	0.24	0.72	0.45	0	0.07	0.01
cis_mRNA_overlap	0	mRNA	n340265	NM_020840_dup1	chr4	159727246	159789738	2173	+	chr4	159690182	159827969	5801	+	*		-0.9351	-1	1.56	1.89	1.91	4.76	4.17	3.77
cis_mRNA_dw20k	1300	NA	n406650	NM_023074_dup1	chr19	52359056	52391189	938	-	chr19	52392488	52408305	3227	-	*		0.7746	1	0.99	0.54	0.56	8.52	6.36	7.82
cis_mRNA_up10k	51	NA	LTCONS_00021280	NM_020810_dup1	chr14	61447832	61519263	8597	+	chr14	61438159	61447782	5289	-	*		0.9488	1	1.03	1.44	0.85	5.19	5.57	4.65
cis_mRNA_up10k	171	NA	n371241	NM_020924_dup1	chr9	125693949	125702599	4185	+	chr9	125680378	125693779	1910	-	*		0.9843	1	1.99	1.9	1.81	5.96	5.55	4.77
cis_mRNA_dw20k	2716	NA	LTCONS_00036145	NM_020917_dup1	chr19	36803588	36822640	4013	-	chr19	36825355	36870105	7493	-	*		-0.7654	-1	1.58	1.66	1.08	8.69	7.95	8.91
cis_mRNA_dw20k	2287	NA	n410076	NM_023072_dup1	chr19	13945330	13947103	1774	-	chr19	13906274	13943044	4339	+	*		-0.983	-1	0.68	1.37	1.21	1.03	0.8	0.81
cis_mRNA_up10k	4053	NA	n410552	NM_020795_dup1	chr17	7293047	7307450	2049	-	chr17	7311502	7323179	4642	+	*		0.9906	1	1.14	3.16	1.21	10.22	12.3	10.59
cis_mRNA_up10k	930	NA	n339270	NM_018052_dup1	chr16	70835990	70840039	3779	-	chr16	70721342	70835061	3096	-	*		0.7146	0.866	0.01	0.03	0.01	6.98	7.7	7.5
cis_mRNA_dw20k	2016	NA	n340426	NM_030955_dup1	chr5	33523641	33525271	1631	-	chr5	33527286	33892124	4955	-	*		0.9188	1	4.43	3.71	2.89	6.22	4.71	4.41
cis_mRNA_overlap	0	mRNA-AntiCompleteIn-LncIntron	n408108	NM_024070_dup1	chr7	99806559	99869855	4059	-	chr7	99816871	99819111	1569	+	*		0.9529	0.866	10.88	9.77	11.38	0.02	0	0.02
cis_mRNA_up10k	67	NA	LTCONS_00030047	NM_001080519_dup1	chr17	79369012	79373474	565	+	chr17	79373540	79433358	10164	+	*		0.9668	0.866	1.34	2.11	1.34	3.16	3.41	3.07
cis_mRNA_dw20k	6565	NA	LTCONS_00041235	NM_022780_dup1	chr2	87011728	87035519	2159	-	chr2	86947414	87005164	6183	+	*		-0.9109	-1	1.32	0.84	1.64	19.35	20.19	16.41
cis_mRNA_overlap	0	mRNA	n338983	NM_017892_dup1	chr2	153532919	153573887	1037	-	chr2	153508107	153573975	7528	-	*		1	1	9.6	10.32	10.45	12.87	13.38	13.47
cis_mRNA_overlap	0	Lnc-AntiOverlap-mRNA	n406344	NM_024520_dup1	chr2	200793634	200820459	5351	-	chr2	200820040	200828848	1512	+	*		-0.8815	-1	1.76	2.37	2.29	8.21	7.5	7.91
cis_mRNA_up10k	433	NA	LTCONS_00039063	NM_017892_dup1	chr2	153574407	153619183	16236	+	chr2	153508107	153573975	7528	-	*		0.7868	1	0.84	0.89	1.04	12.87	13.38	13.47
cis_mRNA_up10k	38	NA	n368149	NM_020199_dup1	chr5	133304443	133306019	619	+	chr5	133291198	133304406	2281	-	*		0.8099	1	0	0.16	0.05	10.16	12.33	12.07
cis_mRNA_up10k	8735	NA	n326471	NM_018420_dup1	chr1	116508428	116510385	914	+	chr1	116519119	116612675	4720	+	*		0.997	1	0.88	0.61	0.49	4.97	4.59	4.36
tran	NA	NA	LTCONS_00052284	NM_020398_dup1	chr3	165629834	165636022	6189	.	chr20	44169265	44176065	1989	-		-1547.5	-0.9687	-1	0.79	0.98	0.88	0.29	0.21	0.27
cis_mRNA_dw20k	2549	NA	LTCONS_00027159	NM_022493_dup1	chr16	771946	777219	4747	-	chr16	779767	790997	2093	-	*		0.8651	1	0.4	0.19	0.54	12.97	12.58	15.75
cis_mRNA_overlap	0	Lnc-AntiOverlap-mRNA	LTCONS_00027159	NM_023933_dup1	chr16	771946	777219	4747	-	chr16	771158	772590	858	+		-1070.33	0.9982	1	0.4	0.19	0.54	15.83	11.78	17.98
cis_mRNA_overlap	0	Lnc-CompleteIn-mRNAIntron	n341989	NM_021229_dup1	chr12	96113018	96115180	2163	-	chr12	96051583	96184536	3615	-	*		0.8973	0.866	0.03	0.03	0.01	3.78	3.32	2.74
cis_mRNA_dw20k	3163	NA	n346188	NM_018641_dup1	chr7	2477402	2483484	381	+	chr7	2443259	2474240	2093	+	*		-0.7559	-0.866	0	0.1	0.1	1.41	0.78	1.2
cis_mRNA_dw20k	10800	NA	n338087	NM_024813_dup1	chr1	92864531	92867614	3084	+	chr1	92764522	92853732	3112	+	*		-0.8915	-1	1.03	1.31	1.06	6.62	6.49	6.55
cis_mRNA_overlap	0	CompleteIn-LncExon	n410787	NM_017599_dup1	chr12	95611522	95696566	4635	+	chr12	95611522	95696566	4562	+	*		-0.8804	-1	5.17	7.16	5.52	36.7	34.97	35.63
cis_mRNA_up10k	283	NA	n410787	NM_018351_dup1	chr12	95611522	95696566	4635	+	chr12	95470525	95611240	9288	-	*		-0.886	-1	5.17	7.16	5.52	5.4	3.64	4.33
cis_mRNA_overlap	0	Lnc-CompleteIn-mRNAIntron	n338800	NM_020744_dup1	chr2	42815140	42816614	1475	+	chr2	42795671	42936353	1857	+	*		-0.6301	-0.866	0.21	0.15	0.21	3.94	4.4	1.67
cis_mRNA_dw20k	1654	NA	n382286	NM_025268_dup1	chr14	105998192	106000652	2012	+	chr14	105992953	105996539	1519	+	*		0.9977	1	0	0.09	0.2	0.14	1.62	3.93
cis_mRNA_dw20k	4014	NA	n333425	NM_022465_dup1	chr12	56436232	56438028	410	+	chr12	56414689	56432219	5451	+	*		0.9992	1	0	0.19	0.26	2.65	3.28	3.47
cis_mRNA_overlap	0	Lnc-CompleteIn-mRNAExon	n338191	NM_024866_dup1	chr22	50922417	50924864	2447	+	chr22	50920012	50924866	4246	+	*		-0.89	-1	0.01	0	0.12	0.3	0.54	0.08

cis_mRNA_dw20k	7970	NA	n369736	NM_018641_dup1	chr7	2482209	2488305	3042	+	chr7	2443259	2474240	2093	+	*		-0.7769	-1	0.05	0.32	0.31	1.41	0.78	1.2
cis_mRNA_overlap	0	Lnc-AntiOverlap-mRNA	n342585	NM_020846_dup1	chr4	48341837	48344353	2516	-	chr4	48343613	48428215	6011	+	*		0.9998	1	0.98	0.94	0.91	7.37	7.1	6.91
cis_mRNA_up10k	724	NA	n410083	NM_022103_dup1	chr19	56989493	57006805	1427	+	chr19	56950693	56988770	3860	-	*		0.9896	1	1.52	0.78	0	2.58	1.65	0
cis_mRNA_dw20k	11303	NA	n375391	NM_024735_dup1	chr16	87351111	87351640	396	+	chr16	87362942	87417394	3616	-	*		-0.882	-1	0.29	0.53	0.78	11.6	9.9	9.81
cis_mRNA_dw20k	5227	NA	n338260	NM_024100_dup1	chr19	999795	1002756	634	+	chr19	984328	994569	1551	+	*		0.9297	1	4.16	4.01	4.29	11.29	10.61	14.05
cis_mRNA_dw20k	862	NA	LTCONS_00006294	NM_020131_dup1	chr1	156001544	156004231	2518	-	chr1	156005092	156023516	3507	-	*		0.9828	1	0.87	0.91	0.88	19.28	20.43	19.76
cis_mRNA_dw20k	19752	NA	n340130	NM_024747_dup1	chr10	103847546	103849781	2236	-	chr10	103825124	103827795	2672	+	*		0.9657	1	0.18	0.14	0.21	9.58	8.68	11.31
cis_mRNA_up10k	9345	NA	LTCONS_00023844	NM_017851_dup1	chr15	65588362	65588865	504	+	chr15	65550437	65579018	2534	-	*		-0.8398	-1	1.55	2.22	1.45	1.87	1.61	2.35
cis_mRNA_up10k	438	NA	n378276	NM_052841_dup1	chr1	32826899	32827425	362	-	chr1	32827862	32829926	1317	+	*		0.8395	1	0	0.13	0.14	0	0.02	0.05
cis_mRNA_overlap	0	mRNA	LTCONS_00014378	NM_021825_dup1	chr11	82972479	82997786	2226	-	chr11	82972506	82997377	1574	-		-687.39	0.8408	1	13.04	14.28	14.74	30.83	31.59	34.29
cis_mRNA_up10k	9662	NA	n370585	NM_030895_dup1	chr8	144362333	144363898	716	-	chr8	144373559	144382122	4686	+	*		0.9748	1	0.62	0.4	0.47	4.16	3.07	3.18
cis_mRNA_overlap	0	Lnc-CompleteIn-mRNAExon	n387441	NM_020340_dup1	chr6	138661322	138665748	4427	+	chr6	138483053	138665800	14882	+	*		0.99	1	2.37	3.24	1.93	9.08	12.81	8.12
cis_mRNA_dw20k	6039	NA	n407080	NM_018706_dup1	chr10	12171640	12211957	2366	+	chr10	12110934	12165602	5202	+	*		0.9933	1	0.31	0.26	0.42	7.67	6.27	9.64
cis_mRNA_dw20k	9000	NA	n339312	NM_020947_dup1	chr16	84492865	84500967	2998	-	chr16	84509966	84538288	5031	-	*		0.906	1	0.84	0.77	1.01	15.16	14.24	15.6
cis_mRNA_dw20k	1869	NA	n341241	NM_033054_dup1	chr7	44998599	45000392	1794	-	chr7	45002260	45018704	3279	-	*		0.9177	0.866	0	0.02	0.05	0.1	0.1	0.11
cis_mRNA_dw20k	8819	NA	n337840	NM_052841_dup1	chr1	32838744	32840303	1560	-	chr1	32827862	32829926	1317	+	*		0.9294	1	0.82	0.93	0.97	0	0.02	0.05
cis_mRNA_overlap	0	Lnc-AntiOverlap-mRNA	n342892	NM_018099_dup1	chr12	29433351	29470781	2380	-	chr12	29376598	29487006	2095	+	*		0.7637	1	0.13	0.18	0.29	11.97	24.32	24.83
cis_mRNA_overlap	0	Lnc-CompleteIn-mRNAIntron	n341144	NM_022062_dup1	chr11	125287355	125290076	2399	+	chr11	125034559	125303285	3730	+	*		0.8985	1	0.04	0.06	0.01	1.69	3.64	1.22
cis_mRNA_overlap	0	mRNA	n333473	NM_025134_dup1	chr16	53241944	53256701	792	+	chr16	53088945	53361414	11462	+	*		0.9919	1	1.57	1.79	1.37	16.77	17.57	16.31
cis_mRNA_dw20k	5361	NA	n378108	NM_018122_dup1	chr1	173833042	173834044	643	-	chr1	173793797	173827682	3348	+	*		-0.7034	-1	5.06	9.63	4.58	4.13	3.98	4.81
cis_mRNA_dw20k	11539	NA	n339381	NM_020676_dup1	chr3	58291999	58304211	1531	+	chr3	58223259	58280461	2393	+	*		0.9998	1	0.85	0.55	0	5.74	5.14	3.94
cis_mRNA_dw20k	3787	NA	LTCONS_00014945	NM_024771_dup1	chr11	63728244	63730948	2705	.	chr11	63706442	63724458	3348	+	*		0.9959	1	0.86	1.26	0.76	12.81	14.86	12.56
cis_mRNA_up10k	4697	NA	LTCONS_00004249	NM_017940_dup1	chr1	16944678	16971208	3228	-	chr1	16890412	16939982	4339	-	*		0.667	0.866	1.79	1.79	2.09	10.25	8.47	10.74
cis_mRNA_up10k	2092	NA	n409307	NM_021823_dup1	chr15	75287876	75313836	3250	+	chr15	75315927	75343067	2219	+	*		-0.9949	-1	6	2.8	3.81	1.37	1.55	1.51
cis_mRNA_dw20k	6783	NA	n339841	NM_025135_dup1	chr18	34366800	34408805	975	-	chr18	33877702	34360018	4942	+	*		0.9913	1	3.64	4.11	2.77	6.49	6.66	5.92
cis_mRNA_dw20k	17633	NA	n373052	NM_018656_dup1	chr12	69177485	69187742	803	+	chr12	69139936	69159853	2347	+	*		0.9465	1	0.36	0.41	0.44	6.93	7.72	9.27
tran	NA	NA	LTCONS_00029129	NM_024313_dup1	chr17	33895109	33901376	893	+	chr22	38082344	38089485	2840	+		-615.1	-0.9964	-1	15.53	12.73	14.04	1.7	1.94	1.81
tran	NA	NA	LTCONS_00029129	NM_020367_dup1	chr17	33895109	33901376	893	+	chr12	3918027	3982608	4492	-		-732.85	-0.8798	-1	15.53	12.73	14.04	3.04	3.59	3.08
cis_mRNA_dw20k	2713	NA	n405259	NM_018939_dup1	chr5	140535580	140537990	2411	+	chr5	140529839	140532868	3030	+	*		0.9355	1	0.27	0.19	0.22	0.73	0.27	0.28
cis_mRNA_up10k	231	NA	n335503	NM_023012_dup1	chr12	123011790	123012372	478	+	chr12	122989190	123011560	2349	-	*		-0.9482	-1	0.16	0.29	0	14.41	13.88	17.39
cis_mRNA_dw20k	14339	NA	LTCONS_00067907	NM_021930_dup1	chr7	105222462	105223523	1062	.	chr7	105172532	105208124	2976	+	*		-0.9868	-1	0.4	0.45	0.38	8.93	8.32	9.02
cis_mRNA_dw20k	8379	NA	n377963	NM_024686_dup1	chr1	84041459	84326679	3177	-	chr1	84335057	84464833	3648	-	*		-0.7602	-1	0.07	0.02	0.01	10.74	10.93	11.72
cis_mRNA_dw20k	15460	NA	n385350	NM_054028_dup1	chr8	11205154	11223597	1421	+	chr8	11188495	11189695	1201	+	*		-0.8602	-1	0.08	0.12	0.11	0.64	0.24	0.53
cis_mRNA_dw20k	16075	NA	n335664	NM_031309_dup1	chr8	145537786	145538380	595	+	chr8	145554454	145559943	3552	-	*		-0.9808	-1	3.43	0.97	3.6	2.85	3.02	2.79
cis_mRNA_overlap	0	mRNA	n342493	NM_020188_dup1	chr16	81009699	81040500	766	-	chr16	81009699	81040502	730	-	*		-0.883	-1	6.27	4.75	6.51	13.05	15.23	10.05
cis_mRNA_dw20k	17895	NA	n337991	NM_020794_dup1	chr1	70607065	70610043	2979	+	chr1	70225858	70589171	6245	+	*		-0.9965	-1	0.93	1.32	0.97	1.07	0.91	1.04
cis_mRNA_dw20k	414	NA	n337991	NM_017768_dup1	chr1	70607065	70610043	2979	+	chr1	70610456	70671361	2953	-	*		-0.9999	-1	0.93	1.32	0.97	54.81	41.77	53.66
cis_mRNA_dw20k	465	NA	n384489	NM_025212_dup1	chr4	105389566	105392866	3301	-	chr4	105393330	105412467	761	-	*		0.906	1	14.84	17.05	15.75	13.9	16.88	16.29
cis_mRNA_up10k	474	NA	LTCONS_00035034	NM_022103_dup1	chr19	56989243	57006805	1077	+	chr19	56950693	56988770	3860	-	*		0.862	1	4.93	0.8	0	2.58	1.65	0
cis_mRNA_up10k	147	NA	n366364	NM_022149_dup1	chr3	184429982	184433265	672	+	chr3	184428155	184429836	1682	-	*		-0.971	-1	0.93	1.6	2.6	47.16	44.03	42.04
cis_mRNA_overlap	0	Lnc-AntiOverlap-mRNA	LTCONS_00016967	NM_019854_dup1	chr12	3592870	3600428	5998	-	chr12	3600425	3703139	2343	+	*		0.9769	1	1.78	1.44	2.65	9.33	8.32	10.46
cis_mRNA_dw20k	10068	NA	LTCONS_00023120	NM_019589_dup1	chr14	75314080	75316980	2901	.	chr14	75230069	75304013	8137	+	*		0.8819	1	0.68	1.08	0.7	10.78	11.27	11.03
cis_mRNA_overlap	0	mRNA	n407958	NM_020120_dup1	chr2	128848782	128953249	10932	+	chr2	128848754	128953251	10781	+	*		-0.9921	-1	3.11	2.19	2.63	7.15	8.12	7.55
cis_mRNA_up10k	156	NA	LTCONS_00024105	NM_181900_dup1	chr15	81616679	81654504	981	+	chr15	81605007	81616524	1316	-	*		0.9802	1	1.08	2.17	1.22	0	0.94	0.3
cis_mRNA_up10k	4697	NA	LTCONS_00004252	NM_017940_dup1	chr1	16944678	16971217	2811	-	chr1	16890412	16939982	4339	-	*		0.6916	1	1.96	1.9	3.48	10.25	8.47	10.74
cis_mRNA_dw20k	12249	NA	n341086	NM_021825_dup1	chr11	82959131	82960258	1124	-	chr11	82972506	82997377	1574	-	*		0.9502	1	1	1.76	2.51	30.83	31.59	34.29
cis_mRNA_overlap	0	Lnc-CompleteIn-mRNAIntron	n340229	NM_057175_dup1	chr4	140291793	140292401	609	+	chr4	140222676	140311935	5554	+	*		0.9031	1	0.18	0.16	0.21	16.43	15.34	16.71
cis_mRNA_dw20k	8267	NA	n344825	NM_024754_dup1	chr5	71663446	71664218	772	+	chr5	71616200	71655180	2103	+	*		0.8982	1	0.51	0.93	0.87	2.58	3.12	2.82
cis_mRNA_dw20k	6180	NA	n338711	NM_018283_dup1	chr13	48627461	48669198	950	-	chr13	48611703	48621282	2022	+	*		-0.9968	-1	7.82	8.42	5.69	18.55	17.29	26.2
cis_mRNA_up10k	233	NA	n409109	NM_020196_dup1	chr19	7694671	7696510	429	+	chr19	7684411	7694439	2655	-	*		-0.9934	-1	2.36	0.81	1.44	12.53	14.44	13.86
cis_mRNA_dw20k	2480	NA	n409226	NM_017771_dup1	chr3	58413357	58419566	1597	-	chr3	58318617	58410878	2027	+	*		0.9985	1	0.69	0.92	1.27	3.65	4.03	4.74
cis_mRNA_overlap	0	Lnc-CompleteIn-mRNAExon	n341510	NM_017780_dup1	chr8	61712995	61777685	5901	+	chr8	61591324	61780587	11581	+	*		0.995	1	1.62	1.26	1.14	6.18	5.41	5.01
cis_mRNA_overlap	0	CompleteIn-LncExon	n410212	NM_024701_dup1	chr10	5680820	5708558	2808	-	chr10	5680820	5708558	2727	-	*		-0.9958	-1	0.94	1.77	0.61	8.26	7.19	8.88
cis_mRNA_dw20k	5668	NA	LTCONS_00042804	NM_017880_dup1	chr2	70370117	70371350	1234	.	chr2	70377017	70418151	2524	-	*		-0.8393	-1	0.8	0.75	1.2	2.12	2.31	2.01
cis_mRNA_overlap	0	Lnc-CompleteIn-mRNAExon	n408138	NM_018359_dup1	chr4	186320694	186347139	2354	-	chr4	186320694	186347139	2379	-	*		-0.979	-1	0.73	0.51	0.29	10.38	12.72	13.82
cis_mRNA_dw20k	8054	NA	n338886	NM_022492_dup1	chr2	74729744	74732192	2449	+	chr2	74710200	74721691	2929	+	*		0.7107	0.866	0.03	0.03	0	0.47	1.11	0.23
cis_mRNA_up10k	4072	NA	n407433	NM_018339_dup1	chr9	79013515	79014954	1440	+	chr9	79000433	79009444	2707	-	*		0.8302	1	0.46	0.59	0.27	32.3	34.83	32.18
cis_mRNA_dw20k	614	NA	n408191	NM_024086_dup1	chr17	2310275	2318730	576	-	chr17	2319343	2415200	5758	-	*		0.7302	1	0.26	0.17	0.29	3.07	3.04	3.55
cis_mRNA_up10k	2318	NA	n372725	NM_001144936_dup1	chr11	63538430	63580588	623	-	chr11	63527364	63536113	5582	-	*		-0.9671	-1	0.12	0.27	0.16	23.13	15.21	23.1
cis_mRNA_dw20k	6771	NA	n340904	NM_025231_dup1	chr6	28104627	28107454	2828	+	chr6	28092387	28097857	1276	+	*		-0.9988	-1	0.43	0.53	0.45	14.05	12.28	13.78
cis_mRNA_dw20k	125	NA	n407929	NM_024956_dup1	chr15	43477466	43488229	2424	+	chr15	43425722	43477342	2769	+	*		0.7813	1	0.61	0.23	0.6	5.4	4.05	4.52
tran	NA	NA	LTCONS_00038439	NM_020398_dup1	chr2	97935463	97958212	16109	+	chr20	44169265	44176065	1989	-		-1680.2	-0.9862	-1	5.84	7.84	6.64	0.29	0.21	0.27
cis_mRNA_overlap	0	mRNA	LTCONS_00025334	NM_032856_dup1	chr15	85181743	85197533	8477	-	chr15	85185999	85197521	1855	-		-1216.12	-0.9547	-1	1.09	0.64	0.11	10.53	10.96	12.86

cis_mRNA_overlap	0	AntiCompleteIn-mRNAExon	n339744	NM_144703_dup1	chr20	60709736	60710130	395	-	chr20	60697517	60710434	2594	+	*		0.9567	1	0	0.18	0.26	0	0.13	0.3
cis_mRNA_dw20k	12783	NA	n338491	NM_152491_dup1	chr1	205782469	205784368	1900	+	chr1	205797150	205819276	2183	-	*		0.9529	0.866	0.6	0.51	0.31	0.03	0.03	0
cis_mRNA_overlap	0	Lnc-AntiOverlap-mRNA	n406901	NM_152864_dup1	chr20	61885330	61892967	7638	+	chr20	61872136	61885892	1430	-	*		0.9184	1	0.1	0.08	0.09	0.74	0.11	0.19
cis_mRNA_up10k	90	NA	n338630	NM_152325_dup1	chr13	31456978	31506745	2055	-	chr13	31506834	31549153	998	+	*		0.9912	1	0.04	0.06	0	0.14	0.23	0.03
cis_mRNA_overlap	0	mRNA-AntiCompleteIn-LncIntron	n338630	NM_032849_dup1	chr13	31456978	31506745	2055	-	chr13	31480312	31499709	2390	+	*		0.9286	1	0.04	0.06	0	0.01	0.03	0
cis_mRNA_dw20k	5380	NA	n366572	NM_054110_dup1	chr3	16276632	16283253	6340	+	chr3	16216184	16271253	4641	+	*		0.6842	0.866	0.04	0.11	0.04	0.55	0.6	0.39
cis_mRNA_dw20k	5652	NA	n364400	NM_138794_dup1	chr1	219391858	219419234	341	+	chr1	219347192	219386207	1892	+	*		-0.9997	-1	1.56	1.48	1.86	6.64	6.74	6.2
cis_mRNA_dw20k	2165	NA	n381758	NM_144665_dup1	chr11	94898706	94903969	5264	-	chr11	94906133	94964246	1987	-	*		0.9343	1	3.58	4.6	2.46	6.71	10.81	5.71
cis_mRNA_overlap	0	mRNA	LTCONS_00054345	NM_181806_dup1	chr4	57198019	57211157	9096	-	chr4	57204456	57253638	3562	-	*		0.8331	1	10.98	12.72	12.26	4.79	5.59	4.96
cis_mRNA_up10k	9721	NA	n365788	NM_052920_dup1	chr2	23597678	23598578	764	-	chr2	23608298	23931483	5084	+	*		-1	-1	0.24	0.13	0.04	3.44	4.64	5.61
cis_mRNA_overlap	0	Lnc-AntiOverlap-mRNA	n339124	NM_153603_dup1	chr16	23423692	23428001	4310	+	chr16	23399814	23464512	2937	-	*		0.7805	1	0.06	0.08	0.09	2.05	2.07	2.51
cis_mRNA_up10k	177	NA	n379596	NM_153354_dup1	chr5	87564841	87732491	1101	+	chr5	87491023	87564665	2594	-	*		-0.9387	-1	0	0.13	0.22	7.03	6.04	5.96
cis_mRNA_dw20k	10450	NA	n340374	NM_138809_dup1	chr5	10264709	10267258	2546	-	chr5	10277707	10308168	4047	-	*		-0.9815	-1	0.03	0	0.01	0.66	0.99	0.94
tran	NA	NA	LTCONS_00035503	NM_152384_dup1	chr19	9753096	9792200	18833	-	chr2	170336006	170363165	3157	+		-3369.24	-0.9942	-1	2.05	1.7	1.54	2.77	3.26	3.6
cis_mRNA_overlap	0	Lnc-CompleteIn-mRNAIntron	n381754	NM_033395_dup1	chr11	93454680	93455031	352	+	chr11	93394816	93463522	8044	+	*		-0.9502	-1	21.22	25.27	16.72	3.57	3.46	4.05
cis_mRNA_up10k	9263	NA	n372243	NM_147191_dup1	chr10	127473652	127474348	309	-	chr10	127455022	127464390	1919	-	*		-0.9995	-0.866	0.52	0.24	0.23	0	0.03	0.03
cis_mRNA_up10k	2212	NA	n375352	NM_032444_dup1	chr16	3661780	3693096	955	+	chr16	3631182	3659569	7270	-	*		-0.9477	-1	1.36	0.54	0.2	2.34	2.85	3.55
cis_mRNA_dw20k	13147	NA	n344464	NM_001080825_dup1	chr12	122233120	122237862	1139	+	chr12	122150658	122219974	7510	+	*		-0.6343	-0.866	0.03	0.03	0	2.55	2.93	3.01
cis_mRNA_dw20k	9246	NA	n339046	NM_001080545_dup1	chr2	182991664	182993417	1754	+	chr2	182850551	182982419	1014	+	*		0.9303	1	0.45	0.32	0.16	2.78	1.77	1.49
cis_mRNA_dw20k	19094	NA	n410990	NM_001101419_dup1	chr19	47987539	48004854	656	+	chr19	48023947	48059113	4632	-	*		0.8595	0.866	0.45	0	0.22	0.01	0	0.01
cis_mRNA_up10k	139	NA	LTCONS_00018891	NM_207311_dup1	chr12	120425702	120427510	1809	.	chr12	120427648	120532299	3081	+	*		0.9857	1	2.65	1.74	2.68	3.75	3.29	3.87
cis_mRNA_dw20k	15106	NA	n381240	NM_153713_dup1	chr1	145514196	145515899	1011	-	chr1	145477085	145499091	1401	+	*		0.995	1	1.09	1.99	1.73	5.86	6.22	6.15
cis_mRNA_up10k	134	NA	n340152	NM_144661_dup1	chr10	118429614	118432250	778	+	chr10	118423207	118429481	994	-	*		-0.9707	-0.866	0	0.04	0.04	0.96	0.32	0.48
cis_mRNA_overlap	0	mRNA-AntiCompleteIn-LncIntron	LTCONS_00036843	NM_152473_dup1	chr19	53467065	53541136	7498	-	chr19	53517344	53519833	2490	+	*		0.9983	1	0.28	0.69	0.97	1.26	1.45	1.61
tran	429854	NA	LTCONS_00036843	NM_001004301_dup1	chr19	53467065	53541136	7498	-	chr19	53970989	53997546	4188	+		-1848.2	0.9973	1	0.28	0.69	0.97	1.05	1.31	1.54
cis_mRNA_dw20k	5214	NA	n368277	NM_173514_dup1	chr5	54913807	54916463	933	-	chr5	54921676	55008163	2533	-	*		-0.828	-1	0.04	0.03	0.2	8.42	9.49	7.73
cis_mRNA_up10k	5508	NA	n381106	NM_032648_dup1	chr1	32697262	32707311	2713	-	chr1	32712818	32714461	956	+	*		0.9313	0.866	0.06	0.18	0.25	0.04	0.13	0.13
cis_mRNA_overlap	0	mRNA	n408117	NM_182501_dup1	chr2	242026509	242041747	2758	-	chr2	242034981	242041747	1637	-	*		-0.971	-1	3.83	4.05	2.91	10.37	9.58	11.49
cis_mRNA_dw20k	1464	NA	n338554	NM_175876_dup1	chr1	231464488	231467017	1972	-	chr1	231468480	231473578	5099	-	*		0.8612	1	3.03	2.27	2.3	10.23	9.97	10.11
cis_mRNA_up10k	2435	NA	n379667	NM_033120_dup1	chr5	1004860	1006734	1699	+	chr5	1009168	1038925	1798	+	*		-0.9365	-1	0.06	0	0.04	1.11	1.45	1.33
cis_mRNA_dw20k	5340	NA	n203786	NM_001122961_dup1	chr1	109642815	109643234	420	+	chr1	109648573	109656479	731	-	*		0.9841	1	1150.67	1147.36	900.06	0.05	0.04	0
cis_mRNA_overlap	0	mRNA	n338955	NM_032836_dup1	chr19	56104726	56113336	4681	-	chr19	56102737	56110893	2660	-	*		0.8617	1	0.65	0.78	0.82	3.39	3.65	4.28
cis_mRNA_overlap	0	CompleteIn-LncExon	LTCONS_00040389	NM_001008237_dup1	chr2	20096504	20102393	5148	-	chr2	20096514	20101747	822	-		-421.39	-0.9957	-0.866	2.93	3.57	2.93	0.58	0.09	0.53
tran	20512012	NA	LTCONS_00037024	NM_144694_dup1	chr19	58488253	58517254	7337	-	chr19	37959982	37976242	2247	+		-1312.17	0.8517	1	0.24	0.14	0.16	13.04	10.54	12.27
cis_mRNA_dw20k	306	NA	n406195	NM_032856_dup1	chr15	85174691	85185694	3951	+	chr15	85185999	85197521	1855	-	*		-0.858	-1	0.48	0.26	0.15	10.53	10.96	12.86
cis_mRNA_dw20k	18838	NA	LTCONS_00063246	NM_033515_dup1	chr6	129823371	129879403	1360	-	chr6	129898240	130031370	3499	-	*		0.9838	1	0.83	0.55	1.49	12.1	10.76	21.55
cis_mRNA_dw20k	13549	NA	n336906	NM_001101419_dup1	chr19	47995313	48010399	717	-	chr19	48023947	48059113	4632	-	*		-0.866	-0.866	0	0.18	0.09	0.01	0	0.01
cis_mRNA_up10k	7062	NA	n335652	NM_138431_dup1	chr8	145726920	145727491	572	-	chr8	145734552	145736589	1446	+	*		0.936	1	0.39	1	0.69	8.59	9.62	8.76
cis_mRNA_dw20k	14166	NA	n333385	NM_138408_dup1	chr6	111303256	111346793	974	+	chr6	111279763	111289091	950	+	*		0.9759	1	0.06	0.03	0.07	51.02	41.55	51.27
tran	NA	NA	LTCONS_00055968	NM_144721_dup1	chr5	60452705	60453538	834	+	chr4	76439654	76455236	3578	+		-655.64	-0.9608	-1	2.23	1.83	1.61	2.16	2.24	2.36
cis_mRNA_dw20k	3842	NA	LTCONS_00055968	NM_174889_dup1	chr5	60452705	60453538	834	+	chr5	60240956	60448864	719	+	*		0.9899	1	2.23	1.83	1.61	22.13	20.75	19.48
cis_mRNA_dw20k	6592	NA	LTCONS_00042797	NM_145175_dup1	chr2	14786761	14787673	913	.	chr2	14772810	14780170	6355	+	*		0.8772	1	0.19	0.57	0.94	2.83	2.94	6
tran	7062270	NA	LTCONS_00008588	NM_133447_dup1	chr10	81664636	81668229	1185	+	chr10	88730498	88769960	3155	+		-653	0.9169	1	1.04	1.24	1.42	0.41	0.43	0.54
cis_mRNA_overlap	0	Lnc-AntiOverlap-mRNA	n340067	NM_145170_dup1	chr10	75008050	75036742	2842	+	chr10	75013024	75118617	3714	-	*		0.9928	1	0.2	0.38	0.24	0.3	0.72	0.44
cis_mRNA_up10k	278	NA	n379313	NM_152289_dup1	chr19	9732193	9745538	1799	+	chr19	9718002	9731916	4540	-	*		-0.7374	-1	1.23	0.59	1.07	6.78	8.06	7.97
cis_mRNA_overlap	0	mRNA	n342415	NM_152392_dup1	chr2	61404584	61416060	3084	+	chr2	61404555	61414686	3031	+	*		-0.851	-1	4.66	4.09	4.18	5.78	8.01	6.55
cis_mRNA_dw20k	6295	NA	n409717	NM_033318_dup1	chr22	42486937	42521354	1658	+	chr22	42475695	42480643	1585	+	*		0.9789	0.866	0.48	0.31	0.48	6.46	5.39	6.23
cis_mRNA_up10k	115	NA	n376333	NM_144690_dup1	chr19	56905003	56910564	785	+	chr19	56894648	56904889	2297	-	*		0.8764	1	0.06	0	0.36	0.97	0.55	1.23
cis_mRNA_dw20k	5433	NA	n410994	NM_001101419_dup1	chr19	47990891	48018515	1516	-	chr19	48023947	48059113	4632	-	*		0.6529	0.866	1.5	0.98	3.61	0.01	0	0.01
tran	41002691	NA	LTCONS_00008242	NM_133447_dup1	chr10	47708809	47727808	586	+	chr10	88730498	88769960	3155	+		-307.29	0.6841	1	0.95	1.14	1.16	0.41	0.43	0.54
cis_mRNA_overlap	0	CompleteIn-LncExon	n407523	NM_138443_dup1	chr18	43684298	43708299	1165	+	chr18	43684298	43708299	1125	+	*		-0.991	-1	0.13	0.87	1.43	16.37	14.84	12.99
cis_mRNA_up10k	37	NA	LTCONS_00036172	NM_032825_dup1	chr19	37071155	37096185	2753	-	chr19	37096221	37119499	2813	+	*		0.9631	0.866	3.37	1.56	3.37	5.78	5.13	6.03
cis_mRNA_overlap	0	Lnc-AntiOverlap-mRNA	n409145	NM_178463_dup1	chr20	61141438	61148768	2449	-	chr20	61147660	61167971	1220	+	*		0.8677	1	0.44	0.7	1.34	0.03	0.12	0.15
cis_mRNA_dw20k	10144	NA	n387010	NM_145041_dup1	chr17	41381732	41382576	845	+	chr17	41363894	41371589	2795	+	*		-0.9908	-1	0.3	0.46	0.19	0.1	0.05	0.12
cis_mRNA_dw20k	18620	NA	n378578	NM_080657_dup1	chr2	7056982	7058794	757	-	chr2	7017796	7038363	3512	+	*		0.9991	1	0.48	0.2	0	0.38	0.32	0.27
cis_mRNA_overlap	0	Lnc-CompleteIn-mRNAIntron	n332911	NM_152516_dup1	chr2	62230727	62231282	556	+	chr2	62132803	62363642	725	+	*		-0.9585	-1	0.2	0.49	0.28	31.95	30.82	31.35
cis_mRNA_dw20k	13092	NA	n338110	NM_138435_dup1	chr22	40439134	40440534	1401	-	chr22	40390953	40426043	2071	+	*		-0.8998	-0.866	0.27	0.18	0.11	0.02	0.03	0.03
cis_mRNA_overlap	0	Lnc-AntiOverlap-mRNA	LTCONS_00013608	NM_181706_dup1	chr11	31448034	31531188	6316	-	chr11	31391377	31454382	3000	+		-4601.75	0.906	1	0.61	0.59	0.68	6.05	5.11	6.65
cis_mRNA_up10k	4341	NA	n406446	NM_033400_dup1	chr14	24025198	24028787	1239	+	chr14	23990064	24020858	9182	-	*		-0.9391	-1	6.08	5.39	6.57	0.57	0.9	0.54

tran	NA	NA	LTCONS_00052236	NM_144613_dup1	chr3	94224676	94225741	1066	.	chr19	55860652	55866182	1679	-		-504.34	0.9391	1	1.02	1.53	1.41	1	1.64	1.29
tran	NA	NA	LTCONS_00052236	NM_152455_dup1	chr3	94224676	94225741	1066	.	chr15	43650370	43662258	5607	-		-547.12	-0.6997	-1	1.02	1.53	1.41	5.65	5.29	5.64
cis_mRNA_overlap	0	Lnc-CompleteIn-mRNAExon	n408068	NM_201435_dup1	chr12	123259056	123311927	2494	+	chr12	123259056	123311927	3216	+	*		0.9998	1	0	0.09	0.65	0.23	0.25	0.4
cis_mRNA_overlap	0	Lnc-CompleteIn-mRNAIntron	n342630	NM_145265_dup1	chr5	214013	217394	2881	-	chr5	204875	218297	1420	-	*		-0.9907	-1	0.51	0.57	0.43	22.53	21.13	23.69
cis_mRNA_dw20k	1024	NA	n384478	NM_152542_dup1	chr4	89178768	89180508	1741	-	chr4	89181531	89205888	3725	-	*		0.9928	1	15.5	18.14	12.09	20.15	22.58	15.23
cis_mRNA_overlap	0	Lnc-CompleteIn-mRNAIntron	n381958	NM_145648_dup1	chr12	129297749	129299295	1547	-	chr12	129277739	129308541	2789	-	*		0.9892	1	0.56	0	1.58	0.43	0	1.96
cis_mRNA_dw20k	11333	NA	n341847	NM_138572_dup1	chr6	42059976	42061997	2022	-	chr6	42018251	42048644	4323	+	*		0.9177	0.866	0.02	0.03	0.03	3.01	3.86	3.52
cis_mRNA_dw20k	11496	NA	LTCONS_00047082	NM_001002034_dup1	chr22	42486937	42532702	2097	+	chr22	42470255	42475442	2332	+	*		0.9932	1	1.54	2.1	2.16	0.06	0.17	0.2
cis_mRNA_dw20k	98	NA	n381351	NM_144695_dup1	chr1	222906203	222908533	2331	+	chr1	222885906	222906106	1681	+	*		0.9971	1	11.56	13.51	12.82	14.15	15.22	14.77
cis_mRNA_overlap	0	AntiCompleteIn-mRNAIntron	n339583	NM_080826_dup1	chr20	13218068	13220321	1032	-	chr20	13202418	13281297	2592	+	*		-0.7924	-0.866	0.03	0	0.03	0.12	0.17	0.04
cis_mRNA_overlap	0	Lnc-CompleteIn-mRNAIntron	n340910	NM_001082575_dup1	chr17	77264923	77267167	2240	-	chr17	77085427	77303909	2654	-	*		-0.9999	-1	0.09	0.06	0.1	2.39	3.73	1.91
cis_mRNA_dw20k	17376	NA	n345015	NM_152416_dup1	chr8	96088313	96095554	1701	+	chr8	96037221	96070938	1791	+	*		0.9842	1	0.12	0.15	0.08	5.29	5.57	4.53
cis_mRNA_up10k	79	NA	LTCONS_00021260	NM_144581_dup1	chr14	59951151	59972170	4592	+	chr14	59939406	59951073	1381	-	*		-0.9997	-1	3.11	3.81	3.09	1.23	0.73	1.26
cis_mRNA_dw20k	13441	NA	LTCONS_00006736	NM_032360_dup1	chr1	180238555	180243912	4820	-	chr1	180257352	180472022	1616	-	*		-0.9537	-1	1.17	1.73	1.49	38.35	36.34	37.74
cis_mRNA_overlap	0	Lnc-CompleteIn-mRNAExon	n409080	NM_001031738_dup1	chr2	85825670	85829822	1486	-	chr2	85825670	85829822	1554	-	*		-0.9674	-1	0.99	0.2	1.92	1.86	2.08	1.12
cis_mRNA_overlap	0	AntiCompleteIn-mRNAExon	n340678	NM_178126_dup1	chr17	40731535	40732698	1164	+	chr17	40731526	40761445	3809	-	*		0.9929	1	0.03	0.11	0.13	20.97	24.7	25.02
cis_mRNA_up10k	35	NA	LTCONS_00056258	NM_153354_dup1	chr5	87564699	87579435	1267	+	chr5	87491023	87564665	2594	-	*		0.9989	1	1.91	1.7	1.67	7.03	6.04	5.96
cis_mRNA_overlap	0	mRNA	n411666	NM_133450_dup1	chr16	4746511	4784378	2667	-	chr16	4746513	4784163	2615	-	*		0.7837	1	2.1	2.05	3.07	0.92	0.82	0.97
cis_mRNA_up10k	6344	NA	n407465	NM_138770_dup1	chr2	132250386	132279149	3788	+	chr2	132285492	132291239	1457	+	*		0.9482	1	0.38	1.09	0.85	1.72	2.3	1.94
cis_mRNA_dw20k	8	NA	LTCONS_00009126	NM_173791_dup1	chr10	119028886	119042599	7016	+	chr10	119042606	119134937	3837	-	*		0.6934	0.866	0.12	0.13	0.09	9.39	9.43	9.39
cis_mRNA_dw20k	2028	NA	n375785	NM_144611_dup1	chr17	4063018	4063879	808	+	chr17	4046462	4060991	1998	+	*		0.8367	1	0.36	0.83	0	14.65	14.69	12.97
cis_mRNA_up10k	97	NA	n371404	NM_144967_dup1	chrX	130150443	130192120	294	-	chrX	130192216	130223857	3087	+	*		0.9969	1	0	0.27	0.14	0	0.71	0.32
cis_mRNA_overlap	0	mRNA	LTCONS_00000543	NM_032264_dup1	chr1	21766628	21792645	552	+	chr1	21766631	21811393	3766	+		-412.67	0.992	1	0.79	0.98	2.3	0.26	0.32	0.5
cis_mRNA_overlap	0	CompleteIn-LncExon	LTCONS_00006079	NM_053055_dup1	chr1	151841665	151882953	7673	-	chr1	151843342	151882361	5172	-		-2839.69	-0.8701	-1	1.76	1.62	2.09	4.63	6.2	4.12
cis_mRNA_dw20k	229	NA	n341344	NM_052853_dup1	chr7	140395136	140396877	1645	-	chr7	140372953	140394908	2364	+	*		-0.7279	-1	0.04	0.07	0.15	13.42	12.73	12.71
cis_mRNA_dw20k	666	NA	n342908	NM_052907_dup1	chr12	126144254	126146914	2661	+	chr12	125811162	126143589	7578	+	*		0.9919	1	0.12	0.19	0.09	0.26	0.58	0.19
cis_mRNA_dw20k	2213	NA	n376239	NM_177401_dup1	chr19	1261354	1262238	487	+	chr19	1248552	1259142	3793	+	*		-0.9625	-0.866	0.69	1.05	0.55	4.47	4.21	4.47
cis_mRNA_dw20k	12983	NA	n345528	NM_152516_dup1	chr2	62376624	62382117	1332	+	chr2	62132803	62363642	725	+	*		-0.897	-1	0.26	0.62	0.3	31.95	30.82	31.35
cis_mRNA_dw20k	2326	NA	n338647	NM_138799_dup1	chr2	8992820	8994376	1557	-	chr2	8996701	9143876	3804	-	*		0.987	1	14.99	17.48	15.69	18.83	23	19.35
cis_mRNA_dw20k	7509	NA	LTCONS_00017597	NM_032704_dup1	chr12	49674621	49688909	8994	-	chr12	49658865	49667113	1553	+	*		0.7744	0.866	0.32	0.6	1.1	0.78	1.71	1.71
cis_mRNA_dw20k	19140	NA	n338905	NM_138334_dup1	chr19	50988164	50990120	1957	+	chr19	51009259	51013706	756	-	*		0.992	1	0.91	2.05	1.44	0.71	1.91	1.4
cis_mRNA_overlap	0	mRNA	n333535	NM_138481_dup1	chr22	41625501	41634148	1566	-	chr22	41625514	41636935	2533	-	*		1	1	0.07	0.19	0.21	0.31	0.44	0.46
cis_mRNA_up10k	9992	NA	LTCONS_00007593	NM_032125_dup1	chr1	27638130	27638645	516	.	chr1	27648636	27662891	1614	+	*		-0.7891	-1	0.5	1.44	0.13	14.81	14.51	17.05
cis_mRNA_overlap	0	mRNA	n340794	NM_032765_dup1	chr5	180682147	180684315	2168	-	chr5	180683373	180688119	2241	-	*		-0.8655	-1	3.44	4.03	3.28	3.43	3.35	3.6
cis_mRNA_overlap	0	mRNA	n410929	NM_173473_dup1	chr10	73975758	73995618	3008	+	chr10	73975806	73995618	3195	+	*		-0.9778	-1	0.24	0.31	0.5	11.39	10.8	10.1
cis_mRNA_overlap	0	Lnc-AntiOverlap-mRNA	n337727	NM_052929_dup1	chr1	15653176	15670372	3104	-	chr1	15573768	15724622	5090	+	*		-0.866	-0.866	0.03	0.02	0.02	0.02	0.04	0.03
cis_mRNA_up10k	8009	NA	n410120	NM_001089584_dup1	chr5	7859272	7869150	886	-	chr5	7831511	7851264	1026	-	*		-0.9192	-1	0.18	0.09	0.12	0.79	1.69	1.07
cis_mRNA_overlap	0	Lnc-AntiOverlap-mRNA	n340118	NM_032211_dup1	chr10	100011780	100016332	1168	+	chr10	100007443	100028007	3657	-	*		-0.7137	-1	0.12	0.11	0.19	0.13	0.33	0.09
cis_mRNA_dw20k	14266	NA	LTCONS_00040848	NM_033109_dup1	chr2	55834670	55846933	10540	-	chr2	55861198	55921011	4579	-	*		-0.9812	-1	4.44	3.65	3.24	8.93	9.55	10.2
cis_mRNA_overlap	0	CompleteIn-LncExon	LTCONS_00025661	NM_138418_dup1	chr16	691840	699262	3125	+	chr16	691849	698474	910	+		-893.17	-0.9446	-1	0.63	0.75	0.71	6.61	3.43	5.42
cis_mRNA_up10k	855	NA	n384688	NM_032367_dup1	chr5	76383884	76444175	1564	+	chr5	76372514	76383030	1437	-	*		0.9999	1	0.12	0.31	0.13	0.36	0.51	0.37
cis_mRNA_dw20k	409	NA	n407884	NM_207309_dup1	chr9	139979398	139981269	1872	-	chr9	139971953	139978990	3365	+	*		0.9924	1	1	1.06	0.9	0.9	1.05	0.48
cis_mRNA_overlap	0	mRNA	LTCONS_00036065	NM_032816_dup1	chr19	33459798	33462921	2944	-	chr19	33369904	33462869	2605	-	*		0.9792	1	9.24	9.3	11.91	8.81	9.11	10.1
cis_mRNA_dw20k	1207	NA	n342425	NM_001163922_dup1	chr19	51827049	51833589	2296	+	chr19	51834795	51845378	3674	-	*		-0.9945	-1	14.78	17.62	15.06	0.44	0	0.35
cis_mRNA_overlap	0	Lnc-AntiOverlap-mRNA	n409752	NM_001136105_dup1	chr11	111164114	111170539	5499	-	chr11	111169976	111179353	1414	+	*		0.9977	1	0.39	0.56	0.2	0.17	0.34	0.02
cis_mRNA_overlap	0	CompleteIn-LncExon	LTCONS_00027202	NM_080861_dup1	chr16	1826236	1832854	6348	-	chr16	1826713	1832581	1544	-		-1411.97	0.9993	1	0.3	0.28	0.4	18.49	18.07	21.32
cis_mRNA_overlap	0	mRNA	n407259	NM_033082_dup1	chr12	56146247	56211540	1148	-	chr12	56151054	56211540	930	-	*		-0.6415	-1	2.91	3.77	3.76	89.15	73.56	86.45
cis_mRNA_up10k	293	NA	n385677	NM_133465_dup1	chr9	115232003	115248956	402	-	chr9	115249248	115422706	2683	+	*		-0.9918	-1	0.48	0.09	0.77	1.15	1.46	0.79
cis_mRNA_up10k	44	NA	n407721	NM_032825_dup1	chr19	37071155	37096178	2690	-	chr19	37096221	37119499	2813	+	*		-0.977	-1	1.76	2.27	1.31	5.78	5.13	6.03
cis_mRNA_overlap	0	Lnc-AntiOverlap-mRNA	n405703	NM_152346_dup1	chr17	1481377	1489848	3298	+	chr17	1477696	1532130	3001	-	*		0.9782	1	0.08	0.03	0.04	11.77	10.52	11.01
cis_mRNA_overlap	0	Lnc-AntiOverlap-mRNA	n407986	NM_152339_dup1	chr16	89753076	89762772	1725	+	chr16	89762765	89768121	2339	-	*		0.9169	1	1.4	0.2	2.42	6.32	6.09	7.44
cis_mRNA_overlap	0	AntiCompleteIn-mRNAIntron	n341483	NM_080872_dup1	chr8	35529681	35567838	5391	-	chr8	35092975	35652181	7290	+	*		0.9789	0.866	0.02	0.02	0.04	17.76	14.71	28.88
cis_mRNA_dw20k	14046	NA	n377990	NM_080657_dup1	chr2	7052408	7058813	2347	-	chr2	7017796	7038363	3512	+	*		0.9536	1	1.97	1.22	1.06	0.38	0.32	0.27
cis_mRNA_dw20k	2046	NA	n384088	NM_033257_dup1	chr22	20291561	20299716	5868	+	chr22	20301761	20307628	1217	-	*		0.9863	1	0.24	0.16	0.27	22.73	20.75	24.17
cis_mRNA_up10k	72	NA	LTCONS_00041612	NM_153712_dup1	chr2	113236978	113239672	2695	-	chr2	113239743	113290223	5164	+	*		-0.7489	-0.866	0.35	0.43	0.35	58.16	54.1	66.64
cis_mRNA_overlap	0	mRNA	n407220	NM_152835_dup1	chr1	26437656	26452026	4257	+	chr1	26438268	26452034	4148	+	*		-0.9605	-1	3.79	3.57	3.26	3.85	4.14	5.73
cis_mRNA_dw20k	10245	NA	n407037	NM_033259_dup1	chr3	183960117	183966759	1433	-	chr3	183977003	183979251	1360	-	*		-0.8368	-1	0.3	0.25	0	5.04	6.2	6.73

cis_mRNA_dw20k	12874	NA	n338469	NM_152327_dup1	chr14	96967985	96968630	646	-	chr14	96858448	96955112	2648	+	*		1	1	1.35	2.15	1.26	0.48	1.36	0.37
cis_mRNA_up10k	5599	NA	n341941	NM_033276_dup1	chr12	58325307	58329847	524	-	chr12	58335445	58351052	1160	+	*		0.9478	1	5.72	1.45	3.72	1.87	1.35	1.78
cis_mRNA_dw20k	6091	NA	n338223	NM_144618_dup1	chr1	151097097	151098018	917	+	chr1	151043080	151091007	1953	+	*		0.985	1	0.39	0.07	0.31	0.76	0.63	0.75
cis_mRNA_dw20k	16105	NA	LTCONS_00011738	NM_031909_dup1	chr11	47585751	47595112	3769	+	chr11	47611216	47615961	1414	-	*		-0.9259	-1	0.61	0.55	1.03	7.57	8.35	6.73
cis_mRNA_up10k	7935	NA	n341100	NM_152431_dup1	chr11	94292184	94292540	357	+	chr11	94300474	94354587	3199	+	*		0.9563	0.866	0	0.1	0.1	0.17	0.31	0.37
cis_mRNA_up10k	3031	NA	LTCONS_00044939	NM_178463_dup1	chr20	61141438	61144630	2737	-	chr20	61147660	61167971	1220	+	*		0.9894	1	0.14	0.56	0.82	0.03	0.12	0.15
cis_mRNA_dw20k	9203	NA	n345513	NM_001008237_dup1	chr2	20076651	20087312	1889	-	chr2	20096514	20101747	822	-	*		-0.9957	-0.866	0.13	0.17	0.13	0.58	0.09	0.53
cis_mRNA_dw20k	11842	NA	LTCONS_00032016	NM_032932_dup1	chr17	29877073	29877572	500	.	chr17	29718642	29865232	8625	+	*		-0.9409	-1	2.91	2.18	1.4	21.57	21.88	23.4
cis_mRNA_overlap	0	Lnc-CompleteIn-mRNAExon	n335660	NM_001017964_dup1	chr22	21982379	21982768	390	-	chr22	21982378	21984340	1338	-	*		0.9495	1	3.33	1.18	2.52	4.02	2.46	3.91
tran	NA	NA	LTCONS_00072758	NM_172003_dup1	chr9	70195031	70216674	481	-	chr2	114195268	114253781	1827	+		-224.65	0.948	1	0.87	2.04	0.72	26.35	27.93	25.22
cis_mRNA_dw20k	3515	NA	n406993	NM_080662_dup1	chr19	7537723	7538247	525	-	chr19	7541761	7553905	1062	-	*		-0.975	-1	0.21	0.19	0.06	1.29	1.57	2.1
cis_mRNA_up10k	100	NA	n381343	NM_138794_dup1	chr1	219254319	219347093	1588	-	chr1	219347192	219386207	1892	+	*		-0.6431	-0.866	0.04	0	0.04	6.64	6.74	6.2
cis_mRNA_overlap	0	mRNA	LTCONS_00043874	NM_178463_dup1	chr20	61147660	61155660	1626	+	chr20	61147660	61167971	1220	+		-974.53	0.8014	1	0	0.04	0.23	0.03	0.12	0.15
cis_mRNA_overlap	0	Lnc-AntiOverlap-mRNA	n407922	NM_181708_dup1	chr12	50222326	50234937	3088	+	chr12	50229826	50236912	3249	-	*		-0.9691	-1	1.02	0.75	0.98	4.6	4.81	4.68
cis_mRNA_dw20k	5491	NA	n339370	NM_022899_dup1	chr3	53893020	53895603	2584	-	chr3	53901093	53916229	3629	-	*		0.9976	1	2.92	2.96	2.95	21.24	23.25	22.62
cis_mRNA_dw20k	8532	NA	n375381	NM_133458_dup1	chr16	68609570	68610173	374	+	chr16	68573661	68601039	4384	+	*		0.9548	1	0.1	0.66	0.65	16.79	18.86	18.2
cis_mRNA_overlap	0	Lnc-CompleteIn-mRNAExon	n337730	NM_152455_dup1	chr15	43650376	43652236	1860	-	chr15	43650370	43662258	5607	-	*		0.9317	1	0.67	0.49	0.6	5.65	5.29	5.64
cis_mRNA_up10k	6147	NA	n340261	NM_033393_dup1	chr4	153855668	153857989	1124	-	chr4	153864135	153900848	6480	+	*		0.9991	1	0.41	0.11	0.78	1.68	1.29	2.24
cis_mRNA_dw20k	8330	NA	n382231	NM_001039355_dup1	chr14	100745713	100749124	3412	+	chr14	100757453	100772860	2370	-	*		-0.8308	-1	7.14	8.29	8.14	15.69	12.21	14.52
cis_mRNA_overlap	0	mRNA	n408118	NM_182501_dup1	chr2	242026509	242041747	2573	-	chr2	242034981	242041747	1637	-	*		0.9769	1	0.41	0	1.88	10.37	9.58	11.49
cis_mRNA_dw20k	5433	NA	n410995	NM_001101419_dup1	chr19	47990891	48018515	1663	-	chr19	48023947	48059113	4632	-	*		0.7559	0.866	0.77	0.55	1.21	0.01	0	0.01
cis_mRNA_dw20k	14153	NA	n332600	NM_138408_dup1	chr6	111303243	111345548	818	+	chr6	111279763	111289091	950	+	*		0.9957	1	0.07	0	0.08	51.02	41.55	51.27
cis_mRNA_dw20k	11638	NA	LTCONS_00009330	NM_145806_dup1	chr10	135138303	135139037	378	+	chr10	135122423	135126666	1080	+	*		0.9616	1	2.97	2.52	2.11	4.3	4	3.22
cis_mRNA_overlap	0	mRNA	n410660	NM_080678_dup1	chr2	238875587	239008054	2972	+	chr2	238875587	238951423	2206	+	*		0.7888	1	0.93	0.94	1.42	13.31	13.52	13.64
cis_mRNA_up10k	7004	NA	n341113	NM_153705_dup1	chr11	108376162	108377780	1619	-	chr11	108342833	108369159	4311	-	*		0.9449	0.866	1.78	2.14	2.32	1.1	1.41	1.41
cis_mRNA_up10k	9102	NA	n386302	NM_014406_dup1	chr22	17082801	17095998	1053	+	chr22	17071648	17073700	2053	-	*		0.8192	0.866	0.21	0.4	0.07	0.03	0.03	0.01
cis_mRNA_overlap	0	Lnc-AntiOverlap-mRNA	LTCONS_00027090	NM_145039_dup1	chr16	90038988	90052096	1823	+	chr16	90036183	90039240	3058	-	*		0.9058	1	0.62	0.6	0.65	1.56	1.41	1.6
cis_mRNA_overlap	0	mRNA	LTCONS_00003729	NM_198076_dup1	chr1	244998527	245009089	3343	+	chr1	244998639	245008359	2616	+		-1117.6	0.9753	1	4.92	4.77	4.65	38.3	36.55	33.46
cis_mRNA_up10k	7363	NA	n338906	NM_138334_dup1	chr19	51021068	51052107	1914	-	chr19	51009259	51013706	756	-	*		0.9219	1	5.35	10.07	9.86	0.71	1.91	1.4
cis_mRNA_dw20k	2790	NA	n341841	NM_175054_dup1	chr12	14915441	14920865	4728	-	chr12	14923654	14924065	412	-	*		-0.9996	-1	1.34	1.64	0.91	28.48	27.16	30.2
cis_mRNA_dw20k	10742	NA	n410808	NM_198075_dup1	chr11	565657	568457	2303	-	chr11	537522	554916	2774	+	*		0.8767	1	0.26	0.32	0.45	1.08	1.62	1.79
cis_mRNA_up10k	697	NA	n370241	NM_001083537_dup1	chr8	12052320	12060127	1205	+	chr8	12039613	12051624	2438	-	*		-0.8717	-1	2.96	0	5.62	0.4	0.42	0
cis_mRNA_dw20k	17433	NA	LTCONS_00023110	NM_152447_dup1	chr14	42391184	42391931	748	.	chr14	42076764	42373752	3732	+	*		0.9808	1	0.53	0.83	1.12	15.69	18.43	23.83
cis_mRNA_dw20k	6320	NA	n338693	NM_182828_dup1	chr2	20877569	20878997	1428	+	chr2	20866424	20871250	1994	+	*		0.803	0.866	0.02	0.04	0.02	0	0.05	0.03
tran	NA	NA	LTCONS_00037048	NM_152384_dup1	chr19	58916679	58919763	2203	-	chr2	170336006	170363165	3157	+		-1354.49	0.9934	1	1.37	1.73	1.89	2.77	3.26	3.6
cis_mRNA_dw20k	19532	NA	n338086	NM_032856_dup1	chr15	85165252	85166468	1217	-	chr15	85185999	85197521	1855	-	*		0.9954	1	1.73	1.81	2.02	10.53	10.96	12.86
cis_mRNA_dw20k	1308	NA	n364023	NM_145257_dup1	chr1	229455027	229455445	334	+	chr1	229456752	229478688	5121	-	*		0.8184	1	0.12	0.11	0	16.23	14.23	13.12
cis_mRNA_overlap	0	Lnc-AntiOverlap-mRNA	n338343	NM_001017961_dup1	chr1	165880633	166059452	3197	+	chr1	166039256	166135958	1481	-	*		0.9996	1	0.03	0.07	0	3.61	4.36	2.99
cis_mRNA_dw20k	1862	NA	n689	NM_032487_dup1	chr3	169482398	169482848	450	-	chr3	169484709	169487683	2005	-	*		-0.6708	-1	0.42	1.67	1.47	1.17	0.45	1.12
cis_mRNA_dw20k	11590	NA	n341075	NM_033547_dup1	chr11	77576824	77578177	1354	+	chr11	77589766	77705717	3149	-	*		-0.9617	-1	0.2	0.18	0.23	12.51	12.66	11.6
cis_mRNA_dw20k	3316	NA	LTCONS_00073841	NM_182505_dup1	chr9	74591688	74592333	646	.	chr9	74526423	74588373	1317	+	*		0.9802	1	0.39	0.92	0.91	2.24	2.73	2.62
cis_mRNA_dw20k	16925	NA	n409628	NM_144727_dup1	chr7	151106322	151110132	787	+	chr7	151127056	151137099	723	-	*		-0.9936	-1	0.23	0.17	0.72	0.25	0.27	0.18
cis_mRNA_dw20k	2125	NA	n337885	NM_001136273_dup1	chrX	152689210	152691934	2725	+	chrX	152683781	152687086	1251	+	*		0.7857	0.866	0.16	0.21	0.13	0.08	0.08	0
cis_mRNA_dw20k	10245	NA	n375096	NM_138447_dup1	chr16	30597224	30604442	380	+	chr16	30614686	30621682	2722	-	*		0.9285	1	0.2	0.56	0.18	5.69	6.77	4.92
cis_mRNA_dw20k	1520	NA	n325528	NM_001080419_dup1	chr17	73823405	73855474	799	+	chr17	73780920	73821886	3863	+	*		0.7994	0.866	0.04	0.04	0	3.9	3.67	3.52
cis_mRNA_up10k	3591	NA	n338218	NM_021059_dup1	chr1	149816065	149820591	4527	-	chr1	149824181	149824687	507	+	*		0.9843	1	0.99	1.82	1.05	0.08	0.28	0.13
cis_mRNA_dw20k	5901	NA	n407266	NM_032434_dup1	chr2	27851863	27873713	1727	+	chr2	27805893	27845963	3426	+	*		0.9966	1	0.88	0.65	2.2	32.07	31.9	34.62
cis_mRNA_dw20k	13712	NA	n338284	NM_178229_dup1	chr1	156479646	156481486	1840	+	chr1	156495197	156542396	6052	-	*		0.9449	0.866	0.41	0.62	0.48	0.01	0.02	0.01
cis_mRNA_dw20k	8115	NA	n386976	NM_213597_dup1	chr17	8261731	8263859	2129	-	chr17	8271973	8274858	1964	-	*		0.9948	1	9.18	7.07	6.53	7.63	6.65	6.54
cis_mRNA_dw20k	2310	NA	n337711	NM_032368_dup1	chr1	9986451	9987467	1017	-	chr1	9989776	10002826	1407	-	*		0.9803	1	0.21	0	0.56	6.17	5.15	10.48
cis_mRNA_dw20k	10127	NA	n384230	NM_152536_dup1	chr3	14986198	14989931	1876	-	chr3	14860469	14976072	5897	+	*		-0.8722	-1	22.3	17.53	17.02	0.18	0.28	0.41
cis_mRNA_overlap	0	Lnc-AntiOverlap-mRNA	n324472	NM_001010844_dup1	chr6	79576151	79579739	2632	-	chr6	79577189	79608320	1157	+	*		0.9998	1	0.14	0.15	0.07	2.6	2.7	2
cis_mRNA_dw20k	4082	NA	n364661	NM_144709_dup1	chr2	61159963	61163467	2828	+	chr2	61167548	61245365	3810	-	*		0.999	1	0.15	0.17	0.35	0.43	0.48	0.77
cis_mRNA_up10k	7168	NA	n371522	NM_080879_dup1	chrX	102781584	102785819	349	-	chrX	102754681	102774417	1346	-	*		-0.9398	-1	0.39	1.21	0.47	0.49	0.09	0.32
cis_mRNA_dw20k	2020	NA	n338279	NM_052972_dup1	chr19	4523904	4535208	1081	-	chr19	4537227	4540036	1780	-	*		-0.768	-0.866	0.2	0.13	0	0.02	0.07	0.07
cis_mRNA_overlap	0	Lnc-CompleteIn-mRNAIntron	n326309	NM_032360_dup1	chr1	180363055	180364423	628	-	chr1	180257352	180472022	1616	-	*		0.9896	1	0.17	0.11	0.16	38.35	36.34	37.74
cis_mRNA_dw20k	12923	NA	n341882	NM_144973_dup1	chr12	31516415	31522235	868	-	chr12	31535157	31743952	9391	-	*		0.8899	0.866	0.04	0.04	0	7.03	7.77	6.15
cis_mRNA_overlap	0	mRNA	n409487	NM_152792_dup1	chr2	70189395	70314147	2386	-	chr2	70187221	70189397	2177	-	*		0.9317	1	1.5	2.03	1.03	1.67	1.78	1.22
cis_mRNA_overlap	0	Lnc-AntiOverlap-mRNA	n380409	NM_178499_dup1	chr12	119825796	120032045	495	-	chr12	119772517	119978852	2450	+	*		0.9939	1	0	0.28	0.07	0	0.14	0.02
cis_mRNA_dw20k	7254	NA	n368762	NM_001010852_dup1	chr6	123392316	123393064	660	+	chr6	123317582	123385063	2010	+	*		0.9901	1	3.25	3.96	5.01	12.32	13.64	17.08
cis_mRNA_up10k	6352	NA	n338264	NM_145173_dup1	chr19	2727741	2729325	1585	-	chr19	2714565	2721390	3398	-	*		-0.9988	-1	1.86	2.7	2.12	14.56	12.33	13.77
cis_mRNA_dw20k	7192	NA	LTCONS_00025849	NM_033208_dup1	chr16	3331264	3341617	5167	+	chr16	3348808	3355439	3430	-	*		0.9988	1	0.22	0.21	0.29	4.61	3.97	7.63
cis_mRNA_dw20k	1012	NA	n408094	NM_032273_dup1	chr11	85368608	85376182	4618	-	chr11	85358963	85367597	818	+	*		-0.9568	-1	1.23	1.65	0.74	21.54	20.54	25.69
cis_mRNA_overlap	0	Lnc-AntiOverlap-mRNA	n342682	NM_139164_dup1	chr5	110847924	111075423	5523	+	chr5	110834022	110848157	2264	-	*		0.9604	1	0.01	0.02	0	11.81	13.9	5.5

cis_mRNA_dw20k	1933	NA	n338133	NM_052945_dup1	chr22	42318039	42319104	1066	-	chr22	42321036	42322821	934	-	*		-0.9897	-1	0	0.03	0.09	0.08	0.07	0.03
cis_mRNA_up10k	4487	NA	n323814	NM_139282_dup1	chrX	119254333	119263116	2390	-	chrX	119243025	119249847	755	-	*		-0.866	-0.866	0.07	0.05	0.05	0.05	0.09	0.13
tran	NA	NA	LTCONS_00025606	NM_032390_dup1	chr15	90827609	90827981	373	.	chr2	122484095	122494503	1754	-		-480.41	0.9953	1	1.15	0.47	0.37	19.38	16.36	15.49
cis_mRNA_dw20k	4030	NA	n410960	NM_058164_dup1	chr19	9945883	9960365	1173	+	chr19	9964394	10047070	1861	-	*		0.6604	1	10.05	7.32	7.68	51.21	46.82	50.84
cis_mRNA_up10k	643	NA	n406990	NM_053052_dup1	chr1	227916240	227922055	2214	+	chr1	227922697	227968932	2367	+	*		-0.6972	-1	0.45	0.67	0.46	11.04	10.21	10.39
cis_mRNA_dw20k	5120	NA	n375226	NM_144674_dup1	chr16	10715085	10716242	313	-	chr16	10721361	10788802	1609	-	*		-0.7908	-0.866	0.13	0	0.34	0.04	0.16	0.04
cis_mRNA_dw20k	10847	NA	n406246	NM_138374_dup1	chr19	53868968	53889841	6094	+	chr19	53837002	53858122	4311	+	*		0.8642	1	2.08	1.79	2.07	3.38	3.21	3.29
cis_mRNA_up10k	8557	NA	n406246	NM_001040185_dup1	chr19	53868968	53889841	6094	+	chr19	53898397	53915262	4577	+	*		0.739	1	2.08	1.79	2.07	6.91	5.53	5.92
cis_mRNA_up10k	185	NA	n337980	NM_033407_dup1	chr1	63154153	63176365	1228	+	chr1	62920397	63153969	7052	-	*		0.879	1	0.77	0.62	1.24	18.23	16.04	19.25
cis_mRNA_dw20k	16697	NA	n376341	NM_173481_dup1	chr19	781014	789792	489	-	chr19	751146	764318	2851	+	*		-0.9997	-1	0.08	0.07	0	0.06	0.07	0.16
cis_mRNA_up10k	2080	NA	LTCONS_00031999	NM_020877_dup1	chr17	7620567	7620960	394	.	chr17	7623039	7737058	13505	+	*		0.8486	0.866	0	0.09	0.17	0.04	0.04	0.07
cis_mRNA_dw20k	2796	NA	n342961	NM_145235_dup1	chr10	127700956	127703336	2375	-	chr10	127585108	127698161	1296	+	*		0.9399	0.866	7.21	9.66	5.91	0.05	0.29	0.05
cis_mRNA_overlap	0	Lnc-AntiOverlap-mRNA	n341097	NM_033395_dup1	chr11	93459820	93462971	3152	-	chr11	93394816	93463522	8044	+	*		-0.9954	-1	2.42	2.5	1.6	3.57	3.46	4.05
cis_mRNA_overlap	0	Lnc-CompleteIn-mRNAIntron	n332940	NM_032802_dup1	chr15	51034900	51035473	574	-	chr15	50999730	51057910	2010	-	*		0.9015	1	0.38	0	0.34	6.08	5.79	5.93
cis_mRNA_dw20k	3471	NA	n338027	NM_032184_dup1	chr1	85618178	85619886	1709	-	chr1	85623356	85666728	4711	-	*		0.9591	1	0.32	0.22	0.36	1.33	1.09	1.61
cis_mRNA_dw20k	19358	NA	n338027	NM_145172_dup1	chr1	85618178	85619886	1709	-	chr1	85527993	85598821	3007	+	*		-0.8846	-1	0.32	0.22	0.36	0.06	0.08	0.01
cis_mRNA_up10k	634	NA	n339168	NM_145236_dup1	chr2	232258390	232259702	1313	+	chr2	232260335	232265875	3625	+	*		0.9999	1	0.16	0.29	0.07	0.1	0.13	0.08
cis_mRNA_up10k	379	NA	LTCONS_00027229	NM_032271_dup1	chr16	2204144	2205421	1058	-	chr16	2205799	2228130	3667	+	*		-0.906	-1	11.05	7.64	8.52	12.89	14.8	13.57
cis_mRNA_overlap	0	mRNA	n338293	NM_032447_dup1	chr19	8166027	8537075	1702	-	chr19	8130287	8212385	8967	-	*		0.795	1	0.29	0.22	0.31	2.18	2.03	2.81
cis_mRNA_dw20k	12229	NA	n380364	NM_198075_dup1	chr11	567144	568457	816	-	chr11	537522	554916	2774	+	*		0.7754	1	0	0.08	0.56	1.08	1.62	1.79
cis_mRNA_overlap	0	mRNA	n326733	NM_001080484_dup1	chr1	1934512	1936829	1954	-	chr1	1884752	1935276	4710	-	*		0.7206	0.866	0.09	0.04	0.11	0.11	0.11	0.17
cis_mRNA_up10k	92	NA	n410790	NM_032145_dup1	chr6	146136012	146207721	4103	+	chr6	146119271	146135921	4408	-	*		-0.8388	-1	0.43	0.48	0.35	9.23	8.41	9.31
cis_mRNA_overlap	0	Lnc-CompleteIn-mRNAExon	n339042	NM_178123_dup1	chr2	179967007	179969211	2205	-	chr2	179966419	180129350	10448	-	*		-0.8584	-1	0	1.13	0.9	21.19	19.61	20.69
cis_mRNA_overlap	0	AntiCompleteIn-mRNAIntron	n338880	NM_144579_dup1	chr2	73181268	73193366	2279	+	chr2	73169165	73298965	4140	-	*		-0.9192	-1	0.02	0	0.01	1.36	1.98	1.9
cis_mRNA_up10k	15	NA	n410666	NM_144721_dup1	chr4	76404247	76439640	4459	-	chr4	76439654	76455236	3578	+	*		0.9806	1	0.12	0.36	0.54	2.16	2.24	2.36
cis_mRNA_dw20k	8265	NA	n345484	NM_032890_dup1	chr1	223187601	223193977	2196	-	chr1	222988431	223179337	4924	+	*		-0.7811	-1	0.37	0.39	0.33	1.14	0.28	1.18
cis_mRNA_dw20k	11706	NA	n338283	NM_018083_dup1	chr19	7597616	7601370	3755	-	chr19	7581004	7585911	1953	+	*		-0.9402	-1	1.96	2.02	1.67	15.97	15.22	16.76
cis_mRNA_up10k	5616	NA	n407109	NM_181705_dup1	chr5	130494875	130501026	809	-	chr5	130506641	130541119	6213	+	*		0.8634	1	5.51	5.72	3.14	3.92	4.17	3.73
cis_mRNA_overlap	0	mRNA	n410722	NM_001080419_dup1	chr17	73780920	73790279	1328	+	chr17	73780920	73821886	3863	+	*		-0.8975	-1	3.16	3.53	5.03	3.9	3.67	3.52
cis_mRNA_dw20k	11183	NA	n406511	NM_175872_dup1	chr19	35417807	35436076	2664	+	chr19	35447258	35454953	3888	-	*		0.9547	0.866	3.05	2.64	3.23	5.72	5.1	5.72
cis_mRNA_overlap	0	Lnc-AntiOverlap-mRNA	n338624	NM_152834_dup1	chr2	677186	682802	1943	+	chr2	667973	677439	2124	-	*		-0.6487	-0.866	0.02	0	0.02	9	10.82	10.47
cis_mRNA_overlap	0	AntiCompleteIn-mRNAExon	n342335	NM_033116_dup1	chr14	75548828	75549988	1138	+	chr14	75548818	75593778	5543	-	*		0.8958	1	0.03	0.11	0.05	1.82	2.17	2.05
cis_mRNA_dw20k	19702	NA	n378412	NM_001039887_dup1	chr19	36279778	36288787	2167	-	chr19	36249044	36260077	2146	+	*		0.9699	1	0	0.07	0.04	0.93	1.49	1.13
cis_mRNA_overlap	0	Lnc-AntiOverlap-mRNA	n342078	NM_001009994_dup1	chr6	84562078	84563835	1758	-	chr6	84562985	84567234	664	+	*		0.9942	1	0.33	0.41	0.49	2.85	3.35	4.08
cis_mRNA_overlap	0	Lnc-AntiOverlap-mRNA	n339059	NM_032575_dup1	chr16	4385871	4387819	1949	-	chr16	4382225	4389598	3705	+	*		-0.8473	-1	0.34	0.29	0.55	0.73	0.91	0.65
cis_mRNA_dw20k	15202	NA	n370858	NM_198841_dup1	chr9	96192624	96193581	641	-	chr9	96208782	96215874	2765	-	*		-0.6532	-1	0.17	0.05	0.15	3.44	3.99	3.97
cis_mRNA_dw20k	9566	NA	n338476	NM_032037_dup1	chr19	19614692	19615463	375	+	chr19	19625028	19626469	1442	-	*		-0.6073	-1	0.1	0.09	0.28	0.26	0.46	0.24
cis_mRNA_up10k	142	NA	n338174	NM_207327_dup1	chr22	46646334	46649134	2801	+	chr22	46639910	46646193	1492	-	*		0.952	1	0	0.14	0.22	1.88	2.19	2.73
cis_mRNA_overlap	0	CompleteIn-LncExon	n408304	NM_138387_dup1	chr17	42148098	42153712	1637	+	chr17	42148098	42153712	1578	+	*		0.8692	1	0.89	2.24	2.25	12.95	14.42	15.89
cis_mRNA_overlap	0	mRNA	n341713	NM_152573_dup1	chr9	85594505	85597351	2847	-	chr9	85597306	85678043	2770	-	*		0.9905	1	0.72	0.32	0.62	0.75	0.4	0.71
cis_mRNA_overlap	0	Lnc-AntiOverlap-mRNA	n325558	NM_033389_dup1	chr17	28256793	28257715	512	+	chr17	27952965	28257018	9166	-	*		-0.696	-1	0.29	0.07	0.06	9.98	10.67	13.16
cis_mRNA_dw20k	10749	NA	n407984	NM_080751_dup1	chr20	2633178	2639039	2193	+	chr20	2517253	2622430	3169	+	*		0.9995	1	7.9	9.81	6.96	0.03	0.1	0
cis_mRNA_dw20k	12002	NA	n366689	NM_054110_dup1	chr3	16283254	16290513	7115	-	chr3	16216184	16271253	4641	+	*		-0.9572	-1	0.07	0.06	0.08	0.55	0.6	0.39
cis_mRNA_dw20k	11673	NA	n346311	NM_198075_dup1	chr11	566588	568437	1352	-	chr11	537522	554916	2774	+	*		-0.7254	-1	0.63	0.59	0	1.08	1.62	1.79
cis_mRNA_up10k	4124	NA	n406201	NM_138286_dup1	chr19	23945816	24010919	1379	+	chr19	23921997	23941693	6497	-	*		0.9219	1	9.25	5.65	1.18	3.68	3.56	1.99
tran	499903	NA	LTCONS_00036205	NM_144689_dup1	chr19	38120564	38149835	11008	-	chr19	37569382	37620662	2984	+		-1342.59	0.998	1	0.56	0.23	0.28	3.84	3.49	3.52
cis_mRNA_overlap	0	Lnc-AntiOverlap-mRNA	n384341	NM_032025_dup1	chr3	150283627	150321015	1552	-	chr3	150264574	150304179	3894	+	*		0.7465	1	5.29	5.28	3.89	5.43	3.21	2.19
cis_mRNA_dw20k	2623	NA	LTCONS_00060609	NM_001010844_dup1	chr6	79610942	79612988	2047	+	chr6	79577189	79608320	1157	+	*		0.9782	1	1.26	1.57	0.66	2.6	2.7	2
cis_mRNA_dw20k	477	NA	n341743	NM_032342_dup1	chr9	104235457	104237132	1676	-	chr9	104237608	104249475	2089	-	*		0.9881	1	22.32	27.43	20.47	27.14	31.15	26.62
cis_mRNA_up10k	118	NA	n410558	NM_032317_dup1	chr7	73097898	73112542	1426	+	chr7	73095248	73097781	2534	-	*		0.9963	1	0.66	0.17	0.31	1.93	1.6	1.72
cis_mRNA_overlap	0	CompleteIn-LncIntron	n408132	NM_153216_dup1	chr5	92953431	93447404	4427	-	chr5	93076015	93077309	1295	-	*		0.8733	1	0.14	0.23	0	0.03	0.2	0
cis_mRNA_overlap	0	Lnc-CompleteIn-mRNAExon	n341109	NM_032299_dup1	chr11	102921416	102923782	2363	-	chr11	102921413	102962944	12732	-	*		-0.9861	-1	0.18	0.37	0.55	6.95	6.56	5.89
cis_mRNA_up10k	2677	NA	LTCONS_00016144	NM_014903_dup1	chr12	77773588	78222393	650	+	chr12	78225069	78606790	9756	+	*		0.9994	1	0.14	0.27	0	7.7	8.77	6.68
cis_mRNA_dw20k	637	NA	LTCONS_00004023	NM_182752_dup1	chr1	3547331	3549749	1864	-	chr1	3541556	3546695	2428	+	*		0.9925	1	0.6	1.47	0.86	3.56	4.16	3.67
cis_mRNA_dw20k	4878	NA	n340472	NM_138453_dup1	chr5	58152283	58155219	2937	+	chr5	57878939	58147406	1009	+	*		0.9937	1	38.03	39.62	45.53	48.12	49.76	64.61
tran	NA	NA	LTCONS_00067917	NM_032779_dup1	chr7	148787954	148789503	1550	.	chr2	74699959	74710357	4339	-		-1450.48	0.9188	1	1.24	1.72	1.37	0	1.13	0.71
cis_mRNA_dw20k	10374	NA	LTCONS_00067917	NM_001001661_dup1	chr7	148787954	148789503	1550	.	chr7	148799876	148823438	3220	-	*		0.7453	1	1.24	1.72	1.37	2.15	2.32	2.31
cis_mRNA_up10k	999	NA	n372610	NM_153705_dup1	chr11	108370157	108372543	1228	+	chr11	108342833	108369159	4311	-	*		-0.9449	-0.866	0.14	0.08	0.1	1.1	1.41	1.41
cis_mRNA_overlap	0	Lnc-CompleteIn-mRNAExon	n406455	NM_144611_dup1	chr17	4046462	4060991	1756	+	chr17	4046462	4060991	1998	+	*		-0.9955	-1	0.26	0.25	0.33	14.65	14.69	12.97

cis_mRNA_dw20k	5954	NA	LTCONS_00038458	NM_138798_dup1	chr2	99758040	99779773	6597	+	chr2	99785726	99797492	910	-	*	-0.9996	-1	0.83	0.76	1.47	7.8	7.95	5.73
cis_mRNA_overlap	0	CompleteIn-LncExon	LTCONS_00038458	NM_144706_dup1	chr2	99758040	99779773	6597	+	chr2	99758185	99767950	1430	+	-868.23	0.9721	1	0.83	0.76	1.47	1.87	1.52	2.59
cis_mRNA_up10k	83	NA	LTCONS_00010986	NM_001083909_dup1	chr10	134896583	134901327	4745	-	chr10	134901409	134945179	4283	+	*	0.9816	1	1.57	2.41	1.26	1.61	2.37	1.01
cis_mRNA_dw20k	5048	NA	n340655	NM_152349_dup1	chr17	38804774	38806825	1282	+	chr17	38811872	38821416	1735	-	*	-0.9983	-1	2.8	2.97	2.01	8.95	7.37	14.12
cis_mRNA_up10k	5508	NA	n386569	NM_032648_dup1	chr1	32697262	32707311	2323	-	chr1	32712818	32714461	956	+	*	0.9359	0.866	0.19	0.28	0.33	0.04	0.13	0.13
cis_mRNA_up10k	216	NA	n375536	NM_080677_dup1	chr17	56154494	56160565	5871	-	chr17	56160780	56167618	1522	+	*	-0.9543	-0.866	0.07	0.11	0.11	162.15	151.21	154.56
cis_mRNA_dw20k	8422	NA	LTCONS_00007631	NM_144772_dup1	chr1	156572512	156574047	1536	.	chr1	156561558	156564091	1121	+	*	0.9959	1	0.88	0.74	1.07	24.01	23.04	25.86
cis_mRNA_overlap	0	Lnc-CompleteIn-mRNAIntron	n341326	NM_144648_dup1	chr7	133899987	133902516	2530	+	chr7	133812105	133948933	2700	+	*	-0.8278	-0.866	0.12	0.12	0.17	0.58	0.76	0.44
cis_mRNA_dw20k	5724	NA	n372955	NM_032704_dup1	chr12	49672836	49676768	2535	-	chr12	49658865	49667113	1553	+	*	0.891	0.866	0.23	0.34	0.29	0.78	1.71	1.71
cis_mRNA_dw20k	10035	NA	LTCONS_00028390	NM_032039_dup1	chr16	326153	330211	4059	.	chr16	284801	316119	2952	+	*	0.9086	1	0.49	1.07	0.8	9.58	11.77	11.64
cis_mRNA_dw20k	15342	NA	LTCONS_00027056	NM_153025_dup1	chr16	89753016	89762800	4465	+	chr16	89724210	89737675	2190	+	*	-0.8952	-1	0.17	0.12	0.09	1.14	1.17	1.28
cis_mRNA_up10k	4416	NA	n369907	NM_001031617_dup1	chr7	1019650	1020276	417	-	chr7	1004486	1015235	4891	-	*	0.6256	0.866	0	0.08	0.08	3.63	4.06	3.7
cis_mRNA_overlap	0	mRNA	n324698	NM_001145206_dup1	chr22	25584597	25591749	6043	+	chr22	25423941	25593415	10494	+	*	1	1	0.09	0.14	0.09	1.22	1.42	1.22
cis_mRNA_overlap	0	Lnc-CompleteIn-mRNAIntron	n381071	NM_032880_dup1	chr1	18701064	18702174	1111	+	chr1	18434240	18704977	1944	+	*	0.9852	1	0.38	0.81	0.11	20.08	31.1	7.37
cis_mRNA_dw20k	9513	NA	LTCONS_00014546	NM_032424_dup1	chr11	105865549	105869117	3569	-	chr11	105878629	105892954	4087	-	*	-0.7285	-1	1.14	0.88	0.89	25.16	28.82	26.13
cis_mRNA_dw20k	7509	NA	LTCONS_00017598	NM_032704_dup1	chr12	49674621	49688910	9790	-	chr12	49658865	49667113	1553	+	*	-0.968	-0.866	2.8	2.26	2.07	0.78	1.71	1.71
cis_mRNA_dw20k	18048	NA	n364418	NM_001039500_dup1	chr1	20699434	20704252	1177	-	chr1	20617412	20681387	4475	+	*	-0.7559	-0.866	0.03	0.03	0	0.01	0.03	0.04
cis_mRNA_overlap	0	Lnc-CompleteIn-mRNAIntron	n379646	NM_133448_dup1	chr12	130336753	130339489	1986	-	chr12	129556271	130388212	5777	-	*	0.9814	1	0.1	0.06	0.19	2.4	0	5.02
cis_mRNA_dw20k	7401	NA	LTCONS_00047006	NM_152426_dup1	chr22	39436656	39439431	817	+	chr22	39417118	39429256	2503	+	*	0.7232	1	2.07	2.31	2.25	0.65	0.86	0.66
cis_mRNA_up10k	83	NA	n381438	NM_032023_dup1	chr10	45322733	45455137	1799	-	chr10	45455219	45490172	2495	+	*	0.9797	1	0.06	0.07	0.05	8.19	9.41	5.63
cis_mRNA_overlap	0	Lnc-AntiOverlap-mRNA	n342445	NM_032144_dup1	chr2	130724165	130737982	2397	-	chr2	130737235	130740311	3077	+	*	-0.7303	-1	0.3	0.27	0.52	0.8	1.13	0.72
cis_mRNA_up10k	186	NA	n375704	NM_144975_dup1	chr17	33568502	33569901	745	-	chr17	33570086	33594768	4654	+	*	-0.9315	-1	0.58	0.72	0.53	2.33	2.04	2.75
cis_mRNA_up10k	1402	NA	n377531	NM_032775_dup1	chr22	20851571	20853638	519	+	chr22	20795806	20850170	2616	-	*	0.635	1	0.07	0.13	0.44	14.21	15.56	15.58
cis_mRNA_dw20k	453	NA	n368908	NM_138785_dup1	chr6	149912891	149913799	340	-	chr6	149887528	149912439	1122	+	*	0.7628	0.866	0.12	0.21	0.21	4.74	5.1	5.79
cis_mRNA_dw20k	4758	NA	n326647	NM_032648_dup1	chr1	32719218	32726048	2800	+	chr1	32712818	32714461	956	+	*	0.8963	0.866	0.2	0.29	0.25	0.04	0.13	0.13
cis_mRNA_up10k	128	NA	n407659	NM_058190_dup1	chr21	46353199	46359828	1833	-	chr21	46359955	46396888	881	+	*	0.6842	1	1.18	1.1	1.51	4.25	3.31	4.3
cis_mRNA_overlap	0	mRNA	n406594	NM_198077_dup1	chr1	85715637	85725355	3390	-	chr1	85715636	85725355	3264	-	*	-0.9984	-1	10.07	11.41	9.84	4.05	2.49	4.2
cis_mRNA_up10k	3507	NA	n370213	NM_144650_dup1	chr8	67331824	67341212	4366	-	chr8	67344718	67381044	1895	+	*	0.9897	1	0.04	0.06	0.07	0.25	0.41	0.45
cis_mRNA_overlap	0	mRNA	n342491	NM_182523_dup1	chr3	28283075	28361253	3749	+	chr3	28283124	28361264	626	+	*	0.9709	1	0.09	0.1	0.12	2.34	3.07	3.68
cis_mRNA_overlap	0	Lnc-CompleteIn-mRNAExon	n342663	NM_174909_dup1	chr5	82348665	82373191	4505	-	chr5	82348665	82373272	4587	-	*	-0.9982	-1	6.27	3.51	58.39	60.88	67.59	8.93
cis_mRNA_overlap	0	mRNA-AntiCompleteIn-LncIntron	n379409	NM_145046_dup1	chr19	15050246	16748905	1493	+	chr19	16589875	16607003	1285	-	*	-0.7559	-0.866	0.05	0.08	0.06	0.03	0.02	0.02
cis_mRNA_overlap	0	Lnc-AntiOverlap-mRNA	n342019	NM_033121_dup1	chr12	110435224	110437401	1709	-	chr12	110437235	110477237	3910	+	*	-0.9935	-1	0.38	0.4	0.52	14.94	14.24	12.18
cis_mRNA_dw20k	19790	NA	n365530	NM_001017927_dup1	chr2	120038632	120040231	848	-	chr2	120060020	120124258	950	-	*	-0.7313	-0.866	0.11	0.19	0.19	0.59	0.39	0.53
cis_mRNA_dw20k	7427	NA	LTCONS_00028391	NM_001031737_dup1	chr16	756400	765156	8757	.	chr16	772582	776473	1611	-	*	0.9316	1	0.61	0.78	0.68	0.9	1.79	0.95
cis_mRNA_dw20k	4146	NA	n383552	NM_001004301_dup1	chr19	54001691	54007937	1496	+	chr19	53970989	53997546	4188	+	*	0.8852	1	0.33	0.34	0.45	1.05	1.31	1.54
cis_mRNA_dw20k	2864	NA	n339208	NM_033119_dup1	chr16	50671504	50674651	3148	+	chr16	50582241	50668641	2586	+	*	0.999	1	0.26	0.69	0.17	0.41	0.96	0.33
cis_mRNA_overlap	0	Lnc-AntiOverlap-mRNA	n340870	NM_134268_dup1	chr17	74523668	74549660	3372	+	chr17	74523440	74533782	1951	-	*	-0.9006	-1	0.2	0.35	0.17	7.86	7.26	8.66
cis_mRNA_overlap	0	Lnc-CompleteIn-mRNAIntron	n342690	NM_052888_dup1	chr17	30366993	30367713	721	+	chr17	30348155	30380523	3031	+	*	-0.9539	-1	0.25	0.19	0.29	1.83	2.22	1.78
cis_mRNA_overlap	0	other	n407038	NM_138345_dup1	chr3	183960117	183966759	1496	-	chr3	183948317	183960117	4020	+	*	0.6422	0.866	1.64	2.01	0	0.28	0.63	0.28
cis_mRNA_dw20k	16965	NA	n407843	NM_001013838_dup1	chr16	67708436	67753273	1729	-	chr16	67679030	67691472	4479	+	*	-0.6124	-1	0.61	0.57	2.28	1.3	1.37	1.29
cis_mRNA_up10k	7809	NA	n407843	NM_032140_dup1	chr16	67708436	67753273	1729	-	chr16	67696850	67700628	1538	-	*	-0.9688	-1	0.61	0.57	2.28	3.65	3.78	3.31
cis_mRNA_dw20k	2405	NA	n376418	NM_139239_dup1	chr19	36370792	36376739	1245	-	chr19	36379143	36391552	1834	-	*	0.6628	0.866	0.08	0.23	0.27	0.6	0.6	0.95
cis_mRNA_dw20k	2902	NA	n339988	NM_152995_dup1	chr4	47842140	47846356	4215	-	chr4	47849257	47916633	3728	-	*	0.9114	1	3.28	3.08	3.53	5.93	5.91	6.41
cis_mRNA_dw20k	15	NA	LTCONS_00035017	NM_144690_dup1	chr19	56879468	56894634	7052	+	chr19	56894648	56904889	2297	-	*	-0.9366	-1	0.49	0.66	0.1	0.97	0.55	1.23
cis_mRNA_dw20k	17547	NA	n345506	NM_138285_dup1	chr2	184043954	184080015	3597	+	chr2	183989083	184026408	1633	+	*	0.9369	1	0.15	0.31	0.08	8.7	8.83	8.49
cis_mRNA_overlap	0	mRNA	n335546	NM_198920_dup1	chr6	83602163	83732262	990	-	chr6	83602186	83775545	1833	-	*	0.9878	0.866	0.05	0.05	0	3.54	3.5	3.3
cis_mRNA_overlap	0	mRNA-AntiCompleteIn-LncIntron	n387668	NM_001083537_dup1	chr8	11973291	12424354	1914	+	chr8	12039613	12051624	2438	-	*	-0.8227	-1	3.46	1.11	4.98	0.4	0.42	0
cis_mRNA_up10k	112	NA	LTCONS_00036796	NM_032423_dup1	chr19	52892095	52901010	6262	-	chr19	52901121	52921657	3978	+	*	0.9997	1	2.09	1.51	1.02	3.75	3.02	2.45
cis_mRNA_dw20k	9879	NA	n341923	NM_032840_dup1	chr12	53436973	53448222	2861	-	chr12	53458100	53473204	2932	-	*	-0.8465	-1	0.89	1.32	0.72	12.93	12.38	14.92
cis_mRNA_overlap	0	Lnc-AntiOverlap-mRNA	LTCONS_00076281	NM_144967_dup1	chrX	130160054	130202529	6493	-	chrX	130192216	130223857	3087	+	*	0.9993	1	4.34	7.07	5.66	0	0.71	0.32
cis_mRNA_overlap	0	Lnc-AntiOverlap-mRNA	LTCONS_00011445	NM_194285_dup1	chr11	18610455	18631802	1242	+	chr11	18627948	18656020	5734	-	-1571.25	-0.979	-1	0.24	0.18	0.1	2.22	2.39	2.5
cis_mRNA_up10k	1659	NA	n379815	NM_153354_dup1	chr5	87566323	87583474	630	+	chr5	87491023	87564665	2594	-	*	0.9957	1	0.88	0.15	0	7.03	6.04	5.96
cis_mRNA_overlap	0	mRNA	LTCONS_00063415	NM_182503_dup1	chr6	143743466	143754017	6627	-	chr6	143743969	143771841	6253	-	-2452.39	-0.831	-1	0.13	0.07	0.26	0.93	1.15	0.89
cis_mRNA_dw20k	8401	NA	n341067	NM_182969_dup1	chr11	74541627	74543555	1928	-	chr11	74551955	74660232	5001	-	*	-0.8969	-1	0.29	0.11	0.16	0.74	0.96	0.81
cis_mRNA_dw20k	4056	NA	n408251	NM_001017964_dup1	chr22	21921957	21978323	2811	+	chr22	21982378	21984340	1338	-	*	0.9355	1	1.82	1.53	1.7	4.02	2.46	3.91
cis_mRNA_up10k	8035	NA	LTCONS_00037115	NM_001193628_dup1	chr19	57143799	57146493	2695	.	chr19	57154527	57168614	2542	+	*	0.9851	1	1.99	2.31	0.87	2.55	3.09	1.67
cis_mRNA_up10k	87	NA	n410440	NM_033413_dup1	chr17	45900638	45908907	1898	-	chr17	45908993	45915079	1922	+	*	0.9753	1	0.24	0.33	0.41	0	0.08	0.11

cis_mRNA_overlap	0	Lnc-AntiOverlap-mRNA	n324712	NM_152906_dup1	chr22	20006237	20009256	576	-	chr22	20008631	20053447	2271	+	*	0.9996	1	0.13	0.06	0	4.01	3.67	3.35
cis_mRNA_dw20k	10728	NA	n384320	NM_207307_dup1	chr3	129106817	129109437	2621	-	chr3	129120164	129147494	2154	-	*	0.9911	1	1.11	2.16	1.5	0	0.04	0.01
cis_mRNA_up10k	7970	NA	n339187	NM_001130913_dup1	chr16	31716049	31716581	533	+	chr16	31724550	31772886	2774	+	*	-0.9758	-1	0.9	0.89	0.99	6.06	6.43	5.25
cis_mRNA_overlap	0	Lnc-CompleteIn-mRNAExon	n406456	NM_144611_dup1	chr17	4046462	4060991	1306	+	chr17	4046462	4060991	1998	+	*	0.6758	1	18.38	19.41	18.11	14.65	14.69	12.97
cis_mRNA_up10k	62	NA	n375803	NM_138349_dup1	chr17	27894566	27895678	816	+	chr17	27895739	27900175	1453	+	*	0.9799	1	0.09	0.06	0	4.08	3.74	1.7
cis_mRNA_dw20k	4854	NA	n378262	NM_052929_dup1	chr1	15729475	15735929	5178	-	chr1	15573768	15724622	5090	+	*	0.9774	1	0.13	0.29	0.18	0.02	0.04	0.03
cis_mRNA_dw20k	10075	NA	n341112	NM_152434_dup1	chr11	107182858	107186997	4140	-	chr11	107197071	107328572	3280	-	*	0.8648	1	1.08	1.46	1.14	4.68	5.98	5.5
cis_mRNA_overlap	0	Lnc-AntiOverlap-mRNA	n407082	NM_001039374_dup1	chr9	139698379	139703300	2386	-	chr9	139690790	139702193	1716	+	*	0.8336	1	0.09	0.24	0.22	0.16	0.84	0.39
cis_mRNA_dw20k	12283	NA	n341829	NM_058169_dup1	chr12	12632120	12632530	411	+	chr12	12510020	12619838	2085	+	*	-0.8272	-1	0.56	0.34	0.33	3.37	3.64	4.04
cis_mRNA_dw20k	17612	NA	LTCONS_00042498	NM_032726_dup1	chr2	219519520	219524344	2485	-	chr2	219472488	219501909	3237	+	*	0.9569	1	1.05	0.83	0.96	0.11	0.08	0.09
cis_mRNA_up10k	6829	NA	n408048	NM_032773_dup1	chr3	197477261	197511317	3824	+	chr3	197518145	197598456	2258	+	*	0.9659	1	4.55	5.86	5.42	3.61	4.7	4.61
cis_mRNA_overlap	0	Lnc-AntiOverlap-mRNA	n378648	NM_138794_dup1	chr1	219259944	219347303	724	-	chr1	219347192	219386207	1892	+	*	0.7762	1	0.01	0.05	0	6.64	6.74	6.2
cis_mRNA_overlap	0	mRNA	n338895	NM_032827_dup1	chr2	85978467	86015185	1889	+	chr2	85980909	86018506	5863	+	*	-0.866	-0.866	0	0.02	0.02	0.02	0	0.01
cis_mRNA_overlap	0	mRNA-AntiCompleteIn-LncIntron	n342148	NM_032145_dup1	chr6	146056008	146207721	3526	+	chr6	146119271	146135921	4408	-	*	0.994	1	0.04	0	0.05	9.23	8.41	9.31
cis_mRNA_dw20k	393	NA	n345527	NM_144709_dup1	chr2	61157161	61167156	2794	+	chr2	61167548	61245365	3810	-	*	-0.9469	-1	0.79	0.66	0.5	0.43	0.48	0.77
cis_mRNA_dw20k	6834	NA	LTCONS_00073011	NM_198841_dup1	chr9	96195188	96201949	3767	-	chr9	96208782	96215874	2765	-	*	0.8496	1	0.76	1.03	0.88	3.44	3.99	3.97
cis_mRNA_up10k	171	NA	LTCONS_00037100	NM_174905_dup1	chr19	38893273	38893605	333	.	chr19	38893775	38899728	1275	+	*	-0.8953	-0.866	1.31	0.11	0.11	7.2	8.97	8.18
cis_mRNA_up10k	2418	NA	n369394	NM_001010853_dup1	chr6	89830998	89853352	702	+	chr6	89855769	89875284	4708	+	*	0.7517	0.866	0.1	0.05	0.05	3.32	2.74	3.13
cis_mRNA_overlap	0	Lnc-CompleteIn-mRNAIntron	n323826	NM_080879_dup1	chrX	102767660	102768076	361	-	chrX	102754681	102774417	1346	-	*	-0.9715	-1	0	0.3	0.19	0.49	0.09	0.32
cis_mRNA_dw20k	10126	NA	n406444	NM_177401_dup1	chr19	1269267	1274809	3263	+	chr19	1248552	1259142	3793	+	*	-0.9893	-0.866	23.85	32.75	22.19	4.47	4.21	4.47
cis_mRNA_overlap	0	CompleteIn-LncExon	LTCONS_00035501	NM_152289_dup1	chr19	9717424	9792200	13576	-	chr19	9718002	9731916	4540	-	-2599.46	0.9409	1	0.21	0.36	0.3	6.78	8.06	7.97
cis_mRNA_up10k	652	NA	n380563	NM_144665_dup1	chr11	94964897	94966510	1201	+	chr11	94906133	94964246	1987	-	*	-0.6516	-0.866	0.03	0.03	0.05	6.71	10.81	5.71
cis_mRNA_dw20k	5070	NA	n375473	NM_144607_dup1	chr17	7771139	7777031	2846	-	chr17	7761064	7766070	3760	+	*	-0.9734	-1	0.29	0.32	0.16	3.63	2.95	4.65
cis_mRNA_overlap	0	Lnc-AntiOverlap-mRNA	n339908	NM_032160_dup1	chr18	65183783	65566856	2991	+	chr18	65173819	65183967	9281	-	*	0.9985	0.866	0.02	0.05	0.02	4.03	12.86	4.58
cis_mRNA_dw20k	11773	NA	LTCONS_00036197	NM_144694_dup1	chr19	37988014	37999167	2103	-	chr19	37959982	37976242	2247	+	*	0.9976	1	1.8	1.53	1.7	13.04	10.54	12.27
cis_mRNA_dw20k	7306	NA	n410799	NM_001039841_dup1	chr15	30938318	31065209	5750	+	chr15	30918879	30931013	1017	+	*	0.8091	0.866	0.06	0.06	0.05	0.25	0.12	0.03
cis_mRNA_overlap	0	mRNA	n333356	NM_032448_dup1	chr6	170657335	170697497	393	+	chr6	170615844	170714237	3239	+	*	-0.7396	-1	0.2	0.54	0.53	25.91	19.06	23.94
cis_mRNA_dw20k	10127	NA	n384228	NM_152536_dup1	chr3	14986198	14988955	1803	-	chr3	14860469	14976072	5897	+	*	0.9184	1	4.2	5.15	5.39	0.18	0.28	0.41
cis_mRNA_dw20k	3345	NA	n337708	NM_033510_dup1	chr15	40666600	40668192	1593	+	chr15	40650434	40663256	5032	+	*	0.9939	1	3.64	4.03	5	11.69	12.5	16.14
cis_mRNA_overlap	0	Lnc-CompleteIn-mRNAExon	n409153	NM_138389_dup1	chr4	38869354	38947365	3633	+	chr4	38869354	38947365	4138	+	*	-0.9996	-1	0.47	0.26	0.38	0	0.11	0.05
cis_mRNA_dw20k	11200	NA	n377401	NM_145912_dup1	chr22	42760408	42765214	475	-	chr22	42776413	42828401	5605	-	*	-0.9201	-1	1.03	0.99	0.56	0.3	0.48	0.69
cis_mRNA_up10k	132	NA	n338527	NM_152307_dup1	chr14	103991347	103995378	4032	-	chr14	103995509	104003410	3241	+	*	-0.9856	-1	0.9	1.19	0.96	3.17	2.2	3.14
cis_mRNA_dw20k	2469	NA	LTCONS_00052190	NM_052953_dup1	chr3	26754735	26759038	4304	.	chr3	26664300	26752267	1693	+	*	0.9934	1	2.48	1.37	1.19	10.36	5.63	5.55
cis_mRNA_overlap	0	mRNA	LTCONS_00040881	NM_144709_dup1	chr2	61237016	61244554	1332	-	chr2	61167548	61245365	3810	-	*	0.8246	1	0.5	0.92	1.11	0.43	0.48	0.77
cis_mRNA_dw20k	8630	NA	n369093	NM_153369_dup1	chr6	111598892	111617099	663	+	chr6	111580482	111590263	3920	+	*	0.9563	0.866	1.27	1.81	1.27	4.48	4.98	4.63
cis_mRNA_overlap	0	Lnc-CompleteIn-mRNAExon	n407229	NM_178126_dup1	chr17	40731526	40761445	3778	-	chr17	40731526	40761445	3809	-	*	-0.7645	-1	1.81	1.65	1.24	20.97	24.7	25.02
cis_mRNA_up10k	91	NA	n342434	NM_199336_dup1	chr2	97760672	97763200	2528	+	chr2	97749323	97760582	1265	-	*	-0.9668	-1	0.43	0.52	0.39	3.16	1.36	3.26
cis_mRNA_up10k	1197	NA	n382510	NM_138477_dup1	chr15	43030613	43035399	4349	+	chr15	43015760	43029417	4730	-	*	0.9805	0.866	0.04	0.04	0.05	1.89	2	2.42
cis_mRNA_up10k	524	NA	n376338	NM_144690_dup1	chr19	56905412	56910709	1186	+	chr19	56894648	56904889	2297	-	*	-0.9628	-1	0.38	0.52	0.36	0.97	0.55	1.23
cis_mRNA_overlap	0	CompleteIn-LncExon	n410441	NM_032125_dup1	chr1	27648636	27662891	1767	+	chr1	27648636	27662891	1614	+	*	-0.6758	-1	1.02	1.24	0.99	14.81	14.51	17.05
cis_mRNA_up10k	2145	NA	LTCONS_00025869	NM_032444_dup1	chr16	3661713	3708195	5669	+	chr16	3631182	3659569	7270	-	*	0.9619	1	2.94	3.06	3.66	2.34	2.85	3.55
cis_mRNA_overlap	0	Lnc-AntiOverlap-mRNA	LTCONS_00044945	NM_178463_dup1	chr20	61141438	61149374	3699	-	chr20	61147660	61167971	1220	+	*	-0.6934	-0.866	1.11	1.11	0.23	0.03	0.12	0.15
cis_mRNA_dw20k	3833	NA	n339304	NM_152342_dup1	chr16	80631797	80633843	2046	-	chr16	80637675	80838175	2236	-	*	0.968	1	2.33	7.97	4.58	2.41	8.38	6.11
cis_mRNA_dw20k	4056	NA	n408250	NM_001017964_dup1	chr22	21921957	21978323	3016	+	chr22	21982378	21984340	1338	-	*	0.7715	1	0.56	0.09	0.23	4.02	2.46	3.91
cis_mRNA_overlap	0	mRNA	LTCONS_00008456	NM_173473_dup1	chr10	73975686	73996451	3979	+	chr10	73975806	73995618	3195	+	-2146.94	-0.9374	-1	0.49	0.73	0.8	11.39	10.8	10.1
cis_mRNA_dw20k	11295	NA	n339346	NM_153025_dup1	chr16	89748969	89752998	782	-	chr16	89724210	89737675	2190	+	*	0.7948	1	0.09	0.17	0.19	1.14	1.17	1.28
cis_mRNA_dw20k	10532	NA	n339830	NM_203462_dup1	chr4	6694796	6698897	1280	+	chr4	6709428	6711606	1592	-	*	0.6886	0.866	0	0.02	0.02	71.02	72.68	77.83
cis_mRNA_dw20k	15	NA	n339061	NM_144708_dup1	chr2	190611390	190613707	1696	-	chr2	190540711	190611376	4416	+	*	-0.8439	-1	0.15	0.36	0.29	0.41	0.22	0.38
cis_mRNA_overlap	0	Lnc-AntiOverlap-mRNA	n324020	NM_032558_dup1	chr9	97217602	97228899	739	-	chr9	97136833	97223202	3298	+	*	0.7336	0.866	0.44	0.22	0.22	4.05	3.72	3.95
cis_mRNA_dw20k	4825	NA	n345585	NM_001039111_dup1	chr3	32938595	32939273	679	+	chr3	32859510	32933771	3138	+	*	0.92	1	5.07	5.4	6.43	4.81	5.93	6.68
tran	NA	NA	LTCONS_00002060	NM_198449_dup1	chr1	121259966	121313686	5273	+	chr5	49692031	49737234	4309	-	-1555.95	-1	-1	0.61	0.67	0.23	11.51	11.22	13.49
cis_mRNA_dw20k	12062	NA	LTCONS_00070619	NM_032509_dup1	chr8	33370839	33371319	481	.	chr8	33342685	33358778	3574	+	*	-0.9938	-1	1.56	0.85	1.25	7.1	7.7	7.42
cis_mRNA_up10k	6426	NA	n380180	NM_207332_dup1	chr8	687651	1087777	811	+	chr8	614200	681226	1800	-	*	0.9457	1	0.92	0.81	0.66	8.62	7.77	7.43
cis_mRNA_dw20k	7728	NA	n371063	NM_033428_dup1	chr9	7785195	7788763	2224	-	chr9	7796490	7799799	2487	-	*	-0.742	-1	0.24	0.16	0.43	26.15	28.31	26.1
cis_mRNA_dw20k	9018	NA	n342345	NM_052852_dup1	chr19	20318001	20320598	2598	-	chr19	20278083	20308984	1522	+	*	0.8856	0.866	0.2	0.2	0.19	1.64	1.31	0.93
cis_mRNA_up10k	8956	NA	n378209	NM_001024601_dup1	chr1	213029946	213031430	892	-	chr1	213003483	213020991	506	-	*	0.7158	1	0	2.28	0.46	0.29	0.4	0.39
cis_mRNA_up10k	2669	NA	LTCONS_00031998	NM_020877_dup1	chr17	7617762	7620371	2610	.	chr17	7623039	7737058	13505	+	*	-0.61	-0.866	0.08	0.02	0.01	0.04	0.04	0.07
cis_mRNA_dw20k	19801	NA	n385327	NM_207332_dup1	chr8	590408	594400	3993	-	chr8	614200	681226	1800	-	*	0.7206	0.866	1.17	1.17	0.47	8.62	7.77	7.43
cis_mRNA_dw20k	244	NA	n411665	NM_152445_dup1	chr14	74353318	74398991	2602	+	chr14	74399234	74417117	3602	-	*	0.6257	0.866	7.99	5.73	8.43	7.59	7.59	8.93

cis_mRNA_dw20k	2375	NA	n366695	NM_052859_dup1	chr3	53119106	53120125	933	-	chr3	53122499	53164470	5100	-	*	-0.9877	-1	0.32	0.12	0.63	2.61	2.98	2.28
cis_mRNA_overlap	0	Lnc-AntiOverlap-mRNA	n338247	NM_080664_dup1	chr14	31889964	31922089	1213	+	chr14	31915242	31926680	2681	-	*	-0.9219	-0.866	0.06	0.03	0.03	3.88	4.52	4.93
cis_mRNA_dw20k	6364	NA	n364456	NM_001010846_dup1	chr1	154442113	154445591	1538	-	chr1	154451954	154474526	6183	-	*	1	1	0	0.02	0.02	0.01	0.03	0.03
cis_mRNA_dw20k	3881	NA	n370669	NM_145754_dup1	chr8	145703379	145705868	1128	+	chr8	145691738	145699499	3248	+	*	-0.9498	-1	0.1	0.15	0.2	15.31	15.05	14.1
cis_mRNA_overlap	0	Lnc-AntiOverlap-mRNA	n339555	NM_080836_dup1	chr20	2081659	2083525	1867	-	chr20	2082528	2129201	6414	+	*	-0.9939	-1	0.24	0.27	0.15	6.34	6.14	6.71
cis_mRNA_dw20k	13822	NA	n372420	NM_144665_dup1	chr11	94883703	94892312	1926	+	chr11	94906133	94964246	1987	-	*	1	1	0.02	0.1	0	6.71	10.81	5.71
cis_mRNA_up10k	537	NA	n338983	NM_152522_dup1	chr2	153532919	153573887	1037	-	chr2	153574423	153617767	2810	+	*	-0.7346	-1	9.6	10.32	10.45	3.86	3.76	3.27
cis_mRNA_up10k	102	NA	n339279	NM_178868_dup1	chr3	32278182	32280070	1889	-	chr3	32280171	32411813	1170	+	*	-0.9744	-0.866	0.36	0.32	0.19	0.3	0.3	0.4
cis_mRNA_up10k	200	NA	LTCONS_00028555	NM_032530_dup1	chr17	5095377	5138931	1584	+	chr17	5082831	5095178	4877	-	*	0.8612	1	0.78	0.93	0.41	3.39	4.03	3.19
cis_mRNA_overlap	0	CompleteIn-LncExon	LTCONS_00039063	NM_152522_dup1	chr2	153574407	153619183	16236	+	chr2	153574423	153617767	2810	+	-1303.09	-0.9965	-1	0.84	0.89	1.04	3.86	3.76	3.27
cis_mRNA_up10k	6439	NA	n380146	NM_207332_dup1	chr8	687664	704258	965	+	chr8	614200	681226	1800	-	*	0.7206	0.866	0.48	0.48	0.27	8.62	7.77	7.43
cis_mRNA_up10k	48	NA	LTCONS_00027159	NM_032304_dup1	chr16	771946	777219	4747	-	chr16	777266	779715	1352	+	*	-0.9274	-1	0.4	0.19	0.54	11.64	14	11.57
cis_mRNA_dw20k	19393	NA	n385788	NM_145052_dup1	chrX	74544124	74546232	2109	+	chrX	74493894	74524732	2512	+	*	-0.9682	-1	0.83	0.99	0.97	8.66	6.71	7.42
cis_mRNA_dw20k	19801	NA	n344986	NM_207332_dup1	chr8	563151	594400	7219	-	chr8	614200	681226	1800	-	*	0.7593	1	1.52	1.48	0.9	8.62	7.77	7.43
cis_mRNA_up10k	1582	NA	n371573	NM_139282_dup1	chrX	119251428	119253591	334	-	chrX	119243025	119249847	755	-	*	0.866	0.866	0	0.11	0.11	0.05	0.09	0.13
cis_mRNA_dw20k	286	NA	n344998	NM_032336_dup1	chr8	41402850	41406183	1145	+	chr8	41386725	41402565	3841	+	*	-0.9943	-1	0.67	0.76	0.46	0.22	0.19	0.34
cis_mRNA_overlap	0	Lnc-CompleteIn-mRNAIntron	n380530	NM_133448_dup1	chr12	130005848	130007308	739	-	chr12	129556271	130388212	5777	-	*	0.9784	1	0.05	0	0.17	2.4	0	5.02
cis_mRNA_overlap	0	mRNA-AntiCompleteIn-LncIntron	n387021	NM_175882_dup1	chr17	43920723	43972879	839	-	chr17	43922256	43924438	2183	+	*	-0.9822	-1	0.46	0.37	0.95	0.02	0.03	0
cis_mRNA_dw20k	14180	NA	n373225	NM_032704_dup1	chr12	49681292	49683068	1072	-	chr12	49658865	49667113	1553	+	*	0.9231	0.866	0.43	0.73	0.92	0.78	1.71	1.71
cis_mRNA_up10k	4151	NA	n346466	NM_058190_dup1	chr21	46354610	46355805	666	-	chr21	46359955	46396888	881	+	*	0.999	0.866	0.79	0.42	0.79	4.25	3.31	4.3
cis_mRNA_overlap	0	AntiCompleteIn-mRNAIntron	n326310	NM_032360_dup1	chr1	180322309	180325870	1145	+	chr1	180257352	180472022	1616	-	*	0.7339	0.866	0.06	0.03	0.03	38.35	36.34	37.74
cis_mRNA_dw20k	8068	NA	n376424	NM_145056_dup1	chr19	47137991	47142802	3760	-	chr19	47150869	47164395	2834	-	*	-0.9399	-1	0.23	0.44	0.36	19.51	16.99	17.1
cis_mRNA_up10k	53	NA	n376424	NM_033258_dup1	chr19	47137991	47142802	3760	-	chr19	47137333	47137939	213	-	*	-0.9999	-1	0.23	0.44	0.36	0.83	0.37	0.55
cis_mRNA_overlap	0	mRNA	n338260	NM_138690_dup1	chr19	999795	1002756	634	+	chr19	1000437	1009723	3254	+	*	-0.938	-1	4.16	4.01	4.29	0.39	0.7	0.34
cis_mRNA_dw20k	2294	NA	n411518	NM_138435_dup1	chr22	40428336	40432581	4246	+	chr22	40390953	40426043	2071	+	*	0.9707	0.866	0.01	0.05	0.04	0.02	0.03	0.03
cis_mRNA_dw20k	14680	NA	n339152	NM_052902_dup1	chr2	220495852	220496177	326	+	chr2	220462596	220481173	3601	+	*	0.9747	1	1.22	1.55	2.07	2.6	3.43	4.02
cis_mRNA_overlap	0	Lnc-CompleteIn-mRNAIntron	n324429	NM_032131_dup1	chr6	109179550	109196352	1785	+	chr6	109169619	109295352	3413	+	*	-0.9194	-0.866	0.1	0.09	0.09	0.26	0.87	1.27
cis_mRNA_dw20k	1883	NA	n384319	NM_207307_dup1	chr3	129101678	129118282	2282	-	chr3	129120164	129147494	2154	-	*	0.9899	1	0.92	1.58	1.17	0	0.04	0.01
cis_mRNA_dw20k	17517	NA	n340651	NM_152219_dup1	chr17	38497119	38499388	1790	-	chr17	38516904	38520945	4042	-	*	0.9265	1	1.25	1.33	1.3	0.13	0.2	0.15
cis_mRNA_overlap	0	Lnc-AntiOverlap-mRNA	n380242	NM_145056_dup1	chr19	47163621	47180704	496	+	chr19	47150869	47164395	2834	-	*	0.9769	1	0.37	0.14	0.2	19.51	16.99	17.1
cis_mRNA_dw20k	5028	NA	n381331	NM_181644_dup1	chr1	205577073	205578798	1726	-	chr1	205538112	205572046	4088	+	*	0.9253	1	0	0.08	0.13	0.64	0.73	0.97
cis_mRNA_overlap	0	Lnc-AntiOverlap-mRNA	n338401	NM_033343_dup1	chr1	180238800	180243816	2655	-	chr1	180199433	180244188	1879	+	*	-0.723	-0.866	0.25	0.25	0	3.24	1.37	4
cis_mRNA_overlap	0	AntiCompleteIn-mRNAIntron	n386321	NM_001145206_dup1	chr22	25498414	25508659	943	-	chr22	25423941	25593415	10494	+	*	-0.6719	-0.866	0.41	0.14	0.2	1.22	1.42	1.22
cis_mRNA_dw20k	12665	NA	n339520	NM_032343_dup1	chr3	126691908	126695269	3362	+	chr3	126423118	126679244	946	+	*	-0.8813	-1	0.41	0.52	0.48	36.39	31.58	35.44
cis_mRNA_up10k	123	NA	n411587	NM_001143936_dup1	chr15	67807260	67813400	304	-	chr15	67813522	67819641	1297	+	*	0.9928	1	0.09	0.36	0.43	1.67	2.25	2.52
cis_mRNA_overlap	0	Lnc-CompleteIn-mRNAIntron	n385216	NM_173537_dup1	chr7	74233343	74233767	425	-	chr7	74210484	74267841	3551	-	*	-0.9995	-1	0.63	0.49	0.64	3.52	5.93	3.44
cis_mRNA_up10k	6554	NA	n381105	NM_032648_dup1	chr1	32697262	32706265	1410	-	chr1	32712818	32714461	956	+	*	-0.9934	-0.866	0.54	0.46	0.47	0.04	0.13	0.13
cis_mRNA_up10k	4368	NA	n383408	NM_138401_dup1	chr19	17516552	17526545	1804	+	chr19	17530912	17536140	1193	+	*	-0.6133	-0.866	0.06	0.04	0.06	6.16	8.2	7.9
cis_mRNA_up10k	91	NA	n385315	NM_053043_dup1	chr7	155434168	155437113	2946	-	chr7	155437203	155574179	10325	+	*	-0.9949	-1	1.53	2.09	1.45	4.04	3.44	4.05
cis_mRNA_dw20k	13664	NA	n344724	NM_001080825_dup1	chr12	122233637	122241016	2245	-	chr12	122150658	122219974	7510	+	*	0.795	1	0	0.06	0.25	2.55	2.93	3.01
cis_mRNA_up10k	197	NA	n367775	NM_153354_dup1	chr5	87564861	87566247	1039	+	chr5	87491023	87564665	2594	-	*	-0.8976	-1	1.53	1.64	1.75	7.03	6.04	5.96
cis_mRNA_up10k	394	NA	LTCONS_00003880	NM_152486_dup1	chr1	850358	860728	9182	-	chr1	861121	879961	2554	+	*	0.9933	1	0.97	3.9	0.57	2.62	9.05	0.65
cis_mRNA_up10k	42	NA	n377415	NM_032775_dup1	chr22	20850211	20853786	497	+	chr22	20795806	20850170	2616	-	*	-0.9789	-1	0.3	0.13	0.08	14.21	15.56	15.58
cis_mRNA_dw20k	16701	NA	LTCONS_00037011	NM_152475_dup1	chr19	58370253	58400442	407	-	chr19	58417142	58427978	4703	-	*	-0.694	-1	0.91	0.86	0.63	2.17	3.05	3.13
cis_mRNA_up10k	2080	NA	n410486	NM_080603_dup1	chr20	44486220	44507769	2972	+	chr20	44509848	44513905	2787	+	*	-0.8338	-1	0.51	0.36	0.46	2.3	3.09	2.98
cis_mRNA_overlap	0	CompleteIn-LncExon	LTCONS_00025660	NM_138418_dup1	chr16	691816	699262	1868	+	chr16	691849	698474	910	+	-883.34	0.8644	1	2.1	1.77	1.82	6.61	3.43	5.42
cis_mRNA_up10k	9102	NA	n384068	NM_014406_dup1	chr22	17082801	17095998	1573	+	chr22	17071648	17073700	2053	-	*	-0.891	-0.866	0	0.05	0.11	0.03	0.03	0.01
cis_mRNA_dw20k	2523	NA	n409320	NM_144589_dup1	chr10	76970300	76991207	1871	+	chr10	76993729	76995770	975	-	*	-0.9297	-1	1.54	1.44	1.28	12.4	12.42	13.65
cis_mRNA_overlap	0	mRNA	LTCONS_00055124	NM_032783_dup1	chr4	169908741	169931468	15123	-	chr4	169908741	169931468	3502	-	-1517.17	-0.8476	-1	2.04	2.76	2.46	6.17	4.98	6.09
cis_mRNA_dw20k	2105	NA	n375787	NM_144611_dup1	chr17	4063095	4063996	843	+	chr17	4046462	4060991	1998	+	*	0.8031	1	0.46	0.67	0.33	14.65	14.69	12.97
cis_mRNA_up10k	1009	NA	n408333	NM_032319_dup1	chr2	73461364	73480150	1883	+	chr2	73455134	73460356	1083	-	*	-0.8544	-0.866	0.17	0.17	0.62	15.91	14.46	13.12
cis_mRNA_dw20k	1661	NA	n408333	NM_001080410_dup1	chr2	73461364	73480150	1883	+	chr2	73481810	73496758	6928	-	*	0.6405	0.866	0.17	0.17	0.62	4.72	4.81	4.83
cis_mRNA_dw20k	10730	NA	n405483	NM_153713_dup1	chr1	145509820	145516076	6257	-	chr1	145477085	145499091	1401	+	*	-0.9966	-1	0.32	0.21	0.24	5.86	6.22	6.15
cis_mRNA_dw20k	12381	NA	n364026	NM_175876_dup1	chr1	231454150	231456100	1318	+	chr1	231468480	231473578	5099	-	*	-0.9975	-1	0	0.05	0.02	10.23	9.97	10.11
cis_mRNA_dw20k	19501	NA	n384644	NM_139017_dup1	chr5	55232481	55235321	2841	-	chr5	55147334	55212981	2393	+	*	-0.8823	-1	0.73	2.01	0	0.01	0	0.05
cis_mRNA_overlap	0	CompleteIn-LncExon	LTCONS_00040388	NM_001008237_dup1	chr2	20096504	20101826	1883	-	chr2	20096514	20101747	822	-	-355.61	0.7865	1	2.67	2.26	2.38	0.58	0.09	0.53
cis_mRNA_overlap	0	CompleteIn-LncExon	LTCONS_00073804	NM_032937_dup1	chr9	140509689	140513333	1911	-	chr9	140509784	140513308	1452	-	-1172.99	-0.9599	-1	2.22	3.27	2.88	5.11	4.27	4.79
cis_mRNA_up10k	214	NA	n376208	NM_144690_dup1	chr19	56905102	56910539	1608	+	chr19	56894648	56904889	2297	-	*	0.9996	1	0.09	0	0.14	0.97	0.55	1.23

cis_mRNA_dw20k	11666	NA	n387676	NM_145269_dup1	chr8	94752339	94753047	709	+	chr8	94712773	94740674	1122	+	*		-0.6715	-0.866	0	0.09	0.09	16.72	14.51	16.23
cis_mRNA_dw20k	17972	NA	n376230	NM_052852_dup1	chr19	20326955	20331228	1268	-	chr19	20278083	20308984	1522	+	*		0.9807	1	0.55	0.41	0.33	1.64	1.31	0.93
cis_mRNA_overlap	0	Lnc-AntiOverlap-mRNA	n379076	NM_145170_dup1	chr10	75012790	75014102	548	+	chr10	75013024	75118617	3714	-	*		-0.982	-1	0.28	0.26	0.27	0.3	0.72	0.44
cis_mRNA_overlap	0	mRNA	LTCONS_00025874	NM_032575_dup1	chr16	4382102	4383769	774	+	chr16	4382225	4389598	3705	+		-712.24	0.9033	1	1.85	2.01	1.48	0.73	0.91	0.65
cis_mRNA_dw20k	459	NA	n386020	NM_182495_dup1	chr11	114578110	114579041	932	+	chr11	114549200	114577652	1778	+	*		-0.8914	-1	0.08	0.14	0.03	0.1	0.07	0.33
cis_mRNA_overlap	0	Lnc-CompleteIn-mRNAExon	n338275	NM_033064_dup1	chr19	3927008	3928080	1073	+	chr19	3880618	3928080	5036	+	*		0.9876	1	15.69	16.58	18.73	68.81	69.1	70.86
cis_mRNA_overlap	0	Lnc-AntiOverlap-mRNA	n338886	NM_032673_dup1	chr2	74729744	74732192	2449	+	chr2	74732170	74734821	906	-	*		0.9558	0.866	0.03	0.03	0	8.31	8.85	7.06
cis_mRNA_overlap	0	Lnc-AntiOverlap-mRNA	LTCONS_00044944	NM_178463_dup1	chr20	61141438	61149374	3544	-	chr20	61147660	61167971	1220	+	*		0.7397	1	0.97	1.05	2.05	0.03	0.12	0.15
cis_mRNA_overlap	0	Lnc-CompleteIn-mRNAIntron	n381727	NM_207354_dup1	chr11	67064099	67065527	1429	+	chr11	67056762	67069955	2140	+	*		-0.6821	-1	2.1	2.32	2.09	18.34	17.53	21.61
cis_mRNA_dw20k	15263	NA	n363904	NM_144988_dup1	chr1	95392854	95433017	1635	+	chr1	95448279	95538507	1057	-	*		-0.9586	-1	0.05	0.1	0	6.26	6.01	7.04
cis_mRNA_overlap	0	Lnc-AntiOverlap-mRNA	n407252	NM_178012_dup1	chr6	3224535	3234804	1805	+	chr6	3224495	3227968	2019	-	*		-0.735	-1	0.47	1	0.85	1283.63	974.36	1275.17
cis_mRNA_up10k	831	NA	n380406	NM_001146339_dup1	chr19	29777925	30016661	2577	-	chr19	30017491	30055226	1243	+	*		0.9848	1	0.09	0.02	0.06	3.37	1.13	2.76
cis_mRNA_dw20k	1333	NA	n379725	NM_173531_dup1	chr19	21769596	21905511	3669	+	chr19	21906843	21950430	4470	-	*		0.729	0.866	0.48	0.56	0.56	2.2	2.39	2.84
cis_mRNA_dw20k	1943	NA	n342100	NM_198081_dup1	chr6	108018503	108021422	2920	-	chr6	108023364	108145521	4536	-	*		0.9815	1	0.42	0.96	0.16	1.13	1.97	0.31
cis_mRNA_overlap	0	mRNA	n408109	NM_178831_dup1	chr7	99798278	99869855	3550	-	chr7	99798276	99869855	2740	-	*		0.9964	1	1.62	1.99	0	1.39	1.63	0.73
cis_mRNA_dw20k	3758	NA	n374596	NM_001013703_dup1	chr15	40331554	40359902	2827	+	chr15	40226347	40327797	5512	+	*		-0.852	-1	0.08	0	0.15	8.41	8.42	7.63
tran	NA	NA	LTCONS_00064012	NM_178014_dup1	chr6_qbl_hap6	1985012	1985362	351	.	chr6_ssto_hap7	2020464	2025506	2487	+		-235.91	-0.9237	-1	7.22	8.49	7.08	1003.76	950.01	1050.51
cis_mRNA_dw20k	14047	NA	n341948	NM_178169_dup1	chr12	65103238	65104293	1056	+	chr12	65004293	65089192	1337	+	*		-0.9922	-1	0.03	0.26	0.17	0.06	0	0.03
cis_mRNA_overlap	0	Lnc-CompleteIn-mRNAExon	n333313	NM_181845_dup1	chr19	44341247	44351486	480	+	chr19	44331473	44353050	2516	+	*		0.9905	1	0	0.2	0.21	1.3	1.64	1.72
cis_mRNA_overlap	0	mRNA	LTCONS_00025434	NM_207446_dup1	chr15	93160678	93242387	2262	-	chr15	93160678	93199031	2630	-		-1322.41	-1	-1	1.61	2.31	1.53	3.27	2.89	3.31
cis_mRNA_dw20k	1447	NA	n345768	NM_181843_dup1	chr11	67386648	67393963	1778	+	chr11	67395409	67397401	809	-	*		0.9699	1	0.54	1.5	1.63	0.29	0.4	0.46
cis_mRNA_dw20k	14229	NA	LTCONS_00069163	NM_201589_dup1	chr8	144490332	144496002	849	+	chr8	144510230	144512602	2373	-	*		0.811	1	0.4	1.03	0.8	4.37	26.5	5.69
cis_mRNA_up10k	2764	NA	n345175	NM_152641_dup1	chr12	46119808	46120857	1050	-	chr12	46123620	46301819	8466	+	*		-0.861	-1	6.13	6.97	7.48	6.48	6.39	5.84
cis_mRNA_overlap	0	Lnc-AntiOverlap-mRNA	LTCONS_00004773	NM_181809_dup1	chr1	39987243	40025370	1864	-	chr1	39957318	39995541	5626	+	*		-0.7503	-0.866	0.78	0.64	0.35	0.07	0.15	0.15
cis_mRNA_up10k	8796	NA	n382967	NM_152901_dup1	chr16	31237193	31237830	638	+	chr16	31227283	31228398	531	-	*		-0.9835	-1	0.29	0.26	0.2	0	0.06	0.37
cis_mRNA_dw20k	7219	NA	LTCONS_00033692	NM_001012753_dup1	chr19	12098432	12114302	352	+	chr19	12075869	12091214	2639	+	*		0.9993	1	2.22	1.04	0.91	1.22	0.9	0.88
cis_mRNA_dw20k	2438	NA	n380142	NM_001010875_dup1	chr13	45952665	45965014	817	+	chr13	45967451	45992516	3671	-	*		-0.9926	-0.866	0.08	0.08	0	1.66	1.57	2.25
cis_mRNA_overlap	0	mRNA	n339869	NM_001039360_dup1	chr18	45553258	45555546	2111	-	chr18	45553639	45663680	4171	-	*		-0.9912	-1	0.83	0.71	0.28	2.25	2.46	4.74
cis_mRNA_overlap	0	Lnc-AntiOverlap-mRNA	n340152	NM_025015_dup1	chr10	118429614	118432250	778	+	chr10	118430703	118502085	5722	-	*		-0.9995	-0.866	0	0.04	0.04	55.46	51.03	50.87
cis_mRNA_overlap	0	Lnc-CompleteIn-mRNAIntron	n341763	NM_153045_dup1	chr9	117374792	117377101	2309	+	chr9	117373706	117408703	4356	+	*		0.8109	0.866	0.15	0.24	0.24	4.26	4.47	4.77
cis_mRNA_dw20k	12505	NA	LTCONS_00073843	NM_174938_dup1	chr9	85849199	85849796	598	.	chr9	85862300	86153348	2529	-	*		-0.8779	-1	0.49	0.78	0.33	26.03	22.92	38.52
cis_mRNA_dw20k	764	NA	n342436	NM_001136499_dup1	chr19	52561743	52566956	675	-	chr19	52567719	52599018	3876	-	*		-0.9347	-1	0.97	0.78	0.86	4.25	5.03	4.97
cis_mRNA_up10k	7976	NA	LTCONS_00034159	NM_139284_dup1	chr19	35634153	35645207	8574	+	chr19	35615417	35626178	2888	-	*		0.8618	1	2.08	2.54	3.35	2.09	2.51	2.59
cis_mRNA_up10k	280	NA	n384441	NM_174921_dup1	chr4	39640760	39653681	1050	+	chr4	39552535	39640481	1669	-	*		-0.9187	-1	0.87	0.75	0.8	23.81	27.36	24.6
cis_mRNA_overlap	0	mRNA	n324531	NM_153487_dup1	chr6	37619307	37622509	735	-	chr6	37600284	37665766	8907	-	*		0.803	1	0.05	0.22	0.27	2.8	3.1	4.3
cis_mRNA_dw20k	18786	NA	n368936	NM_001029863_dup1	chr6	170125187	170125950	403	-	chr6	170102257	170106402	4146	+	*		-0.9808	-1	0	0.09	0.17	4.76	4.23	3.28
tran	20753680	NA	LTCONS_00037024	NM_152604_dup1	chr19	58488253	58517254	7337	-	chr19	37717366	37734574	1694	+		-872.23	-0.9919	-1	0.24	0.14	0.16	0.99	1.85	1.79
cis_mRNA_dw20k	18008	NA	n370586	NM_173832_dup1	chr8	144362882	144363898	533	-	chr8	144329109	144344875	4786	+	*		-0.9857	-1	0.81	0.97	0.72	1.89	1.57	2.22
cis_mRNA_up10k	523	NA	LTCONS_00055376	NM_178557_dup1	chr4	2060108	2060717	610	.	chr4	2061239	2070816	5871	+	*		0.9702	1	1.14	0.49	1.02	6.02	5.36	5.75
cis_mRNA_dw20k	751	NA	n385517	NM_001007563_dup1	chr9	38406526	38408241	1716	-	chr9	38408991	38424444	1093	-	*		0.9912	1	14.36	17.02	8.45	11.32	13.74	8.09
cis_mRNA_overlap	0	AntiCompleteIn-mRNAIntron	n337866	NM_182703_dup1	chr15	65244342	65248504	1839	-	chr15	65204101	65251042	3113	+	*		0.8816	1	0.22	0.25	0.35	0.24	0.36	0.42
cis_mRNA_dw20k	9481	NA	n407979	NM_198317_dup1	chr1	910579	917473	3040	-	chr1	895967	901099	2564	+	*		-0.948	-1	0.02	0	0.01	1.72	2.15	1.81
cis_mRNA_overlap	0	Lnc-AntiOverlap-mRNA	n409147	NM_173620_dup1	chr17	80347086	80376513	4505	-	chr17	80376252	80400516	2222	+	*		0.9919	1	0.41	0.29	0.83	2.42	2.25	2.75
cis_mRNA_overlap	0	mRNA	n339619	NM_001080412_dup1	chr3	141121660	141161439	649	+	chr3	141043055	141168634	8386	+	*		0.9169	1	0.5	0.25	0.77	0.16	0.15	0.25
cis_mRNA_up10k	278	NA	n410503	NM_170746_dup1	chr11	57479995	57508445	1524	+	chr11	57508722	57510883	1294	+	*		0.8402	1	0.32	0	0.4	27.23	25.63	31.18
cis_mRNA_dw20k	19000	NA	n339186	NM_152901_dup1	chr16	31207452	31208284	833	+	chr16	31227283	31228398	531	-	*		0.7817	1	0.56	0.66	0.69	0	0.06	0.37
cis_mRNA_dw20k	1074	NA	n346469	NM_178566_dup1	chr9	14606167	14609996	529	-	chr9	14611069	14693480	9171	-	*		-0.9496	-1	1.12	0.64	1.13	11.55	13.82	10.4
cis_mRNA_overlap	0	mRNA-AntiCompleteIn-LncIntron	n325551	NM_001007532_dup1	chr17	44068653	44079384	901	-	chr17	44076616	44077060	445	+	*		0.9683	1	0.32	0.36	0.25	0.6	0.62	0.46
cis_mRNA_overlap	0	Lnc-CompleteIn-mRNAExon	n335164	NM_147156_dup1	chr10	52071048	52383612	837	-	chr10	52065345	52383737	3753	-	*		-0.9538	-0.866	0.04	0	0.04	3.44	4.48	3.76
cis_mRNA_dw20k	8335	NA	n338713	NM_198478_dup1	chr19	45596220	45644674	906	+	chr19	45653008	45663408	4687	-	*		-0.9118	-0.866	3.02	1.32	5.44	0.18	0.18	0.16
cis_mRNA_up10k	7199	NA	n366166	NM_198565_dup1	chr3	196358369	196359458	348	-	chr3	196366656	196388875	2465	+	*		-0.7234	-0.866	0.68	0.41	0.3	0.03	0.03	0.07
cis_mRNA_overlap	0	Lnc-CompleteIn-mRNAExon	n338277	NM_133475_dup1	chr19	4186418	4217763	2524	+	chr19	4183351	4224811	4026	+	*		0.656	0.866	1.17	0.03	0.26	0.1	0	0.1
cis_mRNA_dw20k	11367	NA	n385992	NM_173540_dup1	chr10	75547343	75547998	656	+	chr10	75532049	75535977	2056	+	*		0.7119	1	0.61	0.81	1.53	6.2	6.89	6.94
cis_mRNA_dw20k	11895	NA	n342745	NM_001004325_dup1	chr11	1605572	1606513	942	-	chr11	1618407	1619524	1118	-	*		-1	-1	0	0.03	0.02	0.06	0.03	0.04
cis_mRNA_overlap	0	Lnc-CompleteIn-mRNAExon	n342745	NM_001005922_dup1	chr11	1605572	1606513	942	-	chr11	1605572	1606513	942	-	*		1	1	0	0.03	0.02	0	0.03	0.02

cis_mRNA_overlap	0	CompleteIn-LncIntron	n324012	NM_173551_dup1	chr9	101387889	101621992	1544	-	chr9	101494291	101558794	7147	-	*		-0.983	-1	0.09	0.02	0.04	1.32	1.52	1.43
cis_mRNA_overlap	0	AntiCompleteIn-mRNAIntron	n381582	NM_173573_dup1	chr11	559477	560106	535	+	chr11	554850	560779	2098	-	*		0.8539	1	3.53	2.33	3.96	0.22	0.2	0.28
tran	NA	NA	LTCONS_00065177	NM_173812_dup1	chr7	89748714	89761451	10537	+	chr12	63952693	64062354	4057	-		-1607.18	0.951	1	1.2	1.7	2.16	1.05	1.12	1.34
cis_mRNA_up10k	8725	NA	n380381	NM_001145077_dup1	chr11	61263912	61267548	554	+	chr11	61276272	61278490	2219	+	*		-0.9939	-0.866	0	0.06	0.06	0.66	0.44	0.41
cis_mRNA_up10k	9904	NA	n372570	NM_173039_dup1	chr11	77282188	77290777	6065	+	chr11	77300680	77321401	2153	+	*		-0.9245	-0.866	0.03	0.05	0.03	2.77	2.61	2.87
cis_mRNA_overlap	0	mRNA	LTCONS_00013608	NM_144981_dup1	chr11	31448034	31531188	6316	-	chr11	31453949	31531169	795	-		-378.93	0.8214	1	0.61	0.59	0.68	7.94	6.64	8.34
cis_mRNA_up10k	466	NA	n379238	NM_198402_dup1	chr3	123304389	123363415	587	+	chr3	123213363	123303924	1187	-	*		0.7676	1	0.27	0.5	0.44	5.31	7.09	5.5
cis_mRNA_overlap	0	Lnc-AntiOverlap-mRNA	n332932	NM_145030_dup1	chr7	100032899	100033693	697	+	chr7	100032912	100034094	930	-	*		0.8797	1	0.21	0.28	0.32	4	4.2	5.03
cis_mRNA_dw20k	12814	NA	LTCONS_00011029	NM_145306_dup1	chr10	71406168	71406797	630	.	chr10	71390003	71393355	1076	+	*		-0.9965	-1	0.69	1.37	1.6	16.66	14.2	13.69
cis_mRNA_overlap	0	mRNA	LTCONS_00034286	NM_001013659_dup1	chr19	38022121	38034296	5426	+	chr19	37999166	38034239	7094	+		-3003.41	0.9745	1	1.28	1.18	0.74	6.52	6.02	5.25
cis_mRNA_dw20k	8063	NA	n345839	NM_173821_dup1	chr2	242823544	242908807	895	+	chr2	242811886	242815482	1798	+	*		-0.9996	-1	0.44	0.72	0.81	1	0.82	0.77
cis_mRNA_up10k	2233	NA	n381232	NM_001037675_dup1	chr1	144612024	144612727	704	-	chr1	144614959	144828810	2928	+	*		-0.7085	-1	0.26	0.34	0.36	10.32	10.02	6.65
tran	44535204	NA	LTCONS_00052236	NM_001134657_dup1	chr3	94224676	94225741	1066	.	chr3	138760944	138763734	2791	-		-476.73	-0.9744	-0.866	1.02	1.53	1.41	0.06	0.03	0.03
cis_mRNA_up10k	598	NA	n363976	NM_199051_dup1	chr1	190447356	190450667	1460	+	chr1	190066797	190446759	2884	-	*		-0.7468	-1	0.71	0.94	0.67	57.05	50.05	86.49
cis_mRNA_overlap	0	Lnc-CompleteIn-mRNAExon	n339913	NM_152721_dup1	chr18	67510569	67515832	5253	+	chr18	67068284	67516323	8897	+	*		0.9997	1	0.81	0.7	1.39	5.06	4.84	6.49
cis_mRNA_dw20k	5541	NA	n341314	NM_176814_dup1	chr7	126999844	127004813	4970	-	chr7	127010353	127032767	4222	-	*		0.9272	1	0.53	0.72	0.86	3.66	4.78	4.85
cis_mRNA_overlap	0	mRNA	n408036	NM_152744_dup1	chr7	4169317	4308631	3112	+	chr7	3341080	4308632	10397	+	*		0.9555	1	0.24	0.22	0.25	9.38	7.39	9.46
cis_mRNA_dw20k	12321	NA	n338453	NM_001080414_dup1	chr14	91724643	91725347	705	+	chr14	91737667	91884133	7488	-	*		-0.9042	-0.866	0	0.14	0.14	2.47	2.19	2.31
tran	NA	NA	LTCONS_00074776	NM_199286_dup1	chrX	103173181	103227394	4061	+	chr12	7864089	7870155	1040	+		-953.8	0.9446	1	2.57	3.06	1.98	0.03	0.12	0
tran	NA	NA	LTCONS_00066546	NM_173812_dup1	chr7	35118964	35225863	6102	-	chr12	63952693	64062354	4057	-		-1561.04	0.998	1	4.37	4.57	5.48	1.05	1.12	1.34
cis_mRNA_dw20k	2555	NA	n409080	NM_001013649_dup1	chr2	85825670	85829822	1486	-	chr2	85832376	85839179	4265	-	*		-1	-1	0.99	0.2	1.92	1.59	1.71	1.45
cis_mRNA_dw20k	16700	NA	n407391	NM_001024457_dup1	chr2	87257798	87303536	2201	-	chr2	87144738	87241099	6692	+	*		-0.7233	-1	2.58	2.37	3.88	0.02	0.07	0.01
cis_mRNA_dw20k	7589	NA	n340046	NM_178011_dup1	chr10	68868455	68872073	3619	+	chr10	68685792	68860867	5609	+	*		0.9925	0.866	0.08	0.75	0.08	0.96	5.03	1.5
cis_mRNA_overlap	0	Lnc-AntiOverlap-mRNA	n383099	NM_001004306_dup1	chr17	20771746	20807221	2147	+	chr17	20766708	20799453	2806	-	*		0.9723	1	1.98	1.62	0.88	0.43	0.41	0.23
[truncated: 1,383,966 more chars]
